# Combinations of multiple long term conditions and risk of hospital admission or death during winter 2021-22 in England: population based cohort study

**DOI:** 10.1136/bmjmed-2024-001016

**Published:** 2024-11-12

**Authors:** Nazrul Islam, Sharmin Shabnam, Nusrat Khan, Clare Gillies, Francesco Zaccardi, Amitava Banerjee, Vahé Nafilyan, Kamlesh Khunti, Hajira Dambha-Miller

**Affiliations:** 1 Primary Care Research Centre, University of Southampton, Southampton, UK; 2 Diabetes Research Centre, University of Leicester, Leicester, UK; 3 University College London, London, UK; 4 Office for National Statistics, Newport, Newport, UK; 5 Department of Public Health, London School of Hygiene and Tropical Medicine, London, UK

**Keywords:** Public health, Health policy, Epidemiology

## Abstract

**Objective:**

To describe which combinations of long term conditions were associated with a higher risk of hospital admission or death during winter 2021-22 (the third wave of the covid-19 pandemic) in adults in England.

**Design:**

Population based cohort study.

**Setting:**

Linked primary and secondary care data from the General Practice Extraction Service Data for Pandemic Planning and Research (GDPPR) database, Hospital Episode Statistics, and Office for National Statistics death registry, comprising pseudoanonymised routinely collected electronic medical records from the whole population of England registered at a general practice, 1 December 2021 to 31 March 2022.

**Participants:**

48 253 125 individuals, registered in GDPPR in England, aged ≥18 years, and alive on 1 December 2021.

**Main outcomes measures:**

All cause hospital admissions and deaths associated with combinations of multiple long term conditions compared with those with no long term conditions, during the winter season (1 December 2021 to 31 March 2022). Overdispersed Poisson regression models were used to estimate the incidence rate ratios after adjusting for age, sex, ethnic group, and index of multiple deprivation.

**Results:**

Complete data were available for 48 253 125 adults, of whom 15 million (31.2%) had multiple long term conditions. Rates of hospital admissions and deaths among individuals with no long term conditions were 96.3 and 0.8 per 1000 person years, respectively. Compared with those with no long term conditions, the adjusted incidence rate ratio of hospital admissions were 11.0 (95% confidence interval (CI) 9.4 to 12.7) for those with a combination of cancer, chronic kidney disease, cardiovascular disease, and type 2 diabetes mellitus; 9.8 (8.3 to 11.4) for those with cancer, chronic kidney disease, cardiovascular disease, and osteoarthritis; and 9.6 (8.6 to 10.7) for those with cancer, chronic kidney disease, and cardiovascular disease. Compared with those with no long term conditions, the adjusted rate ratio of death was 21.4 (17.5 to 26.0) for those with chronic kidney disease, cardiovascular disease, and dementia; 23.2 (17.5 to 30.3) for those with cancer, chronic kidney disease, cardiovascular disease, and dementia; and 24.3 (19.1 to 30.4) for those with chronic kidney disease, cardiovascular disease, dementia, and osteoarthritis. Cardiovascular disease with dementia appeared in all of the top five combinations of multiple long term conditions for mortality, and this two disease combination was associated with a substantially higher rate of death than many three, four, and five disease combinations.

**Conclusions:**

In this study, rates of hospital admission and death varied by combinations of multiple long term conditions and were substantially higher in those with than in those without any long term conditions. High risk combinations for prioritisation and preventive action by policy makers were highlighted to help manage the challenges imposed by winter pressures on the NHS.

**WHAT IS ALREADY KNOWN ON THIS TOPIC**

- Increasing numbers of multiple long term conditions are associated with a higher rate of hospital admission and a greater risk of mortality
- Multiple long term conditions are also associated with an increased service demand during the winter season, which adds to pressures on the NHS
- Which combinations of multiple long term conditions are associated with the highest risk of winter admissions to hospital or death has not been examined at the population level

**WHAT THIS STUDY ADDS**

- Distinct combinations of long term conditions were identified and the associated risk of hospital admission or death estimated during the winter season based on primary and secondary care data for the whole population of England
- The highest risk of hospital admission was in individuals with the combination of cancer, chronic kidney disease, cardiovascular disease, and diabetes mellitus, and those with cancer, chronic kidney disease, cardiovascular disease, and osteoarthritis
- The highest rate of deaths was in those with the combination of cancer, chronic kidney disease, cardiovascular disease, and dementia, and those with chronic kidney disease, cardiovascular disease, dementia, and osteoarthritis

**HOW THIS STUDY MIGHT AFFECT RESEARCH, PRACTICE, OR POLICY**

- Understanding specific combinations of multiple long term conditions associated with the highest risk of hospital admission and death will allow clinicians and policy makers to prioritise resources for preventive measures

## Introduction

 Every year, the NHS in England faces challenges in delivery of services with the onset of the cold weather and an increase in acute respiratory tract infections in the winter. These challenges are usually referred to as winter pressures, and cover the period from 1 December to 31 March.^1 2^ Data from NHS England over the past decade showed a consistent pattern of high attendance at the accident and emergency department, prolonged wait times, and maximum occupancy of hospital beds during this period.^3^,^5^ Earlier studies suggested that the demand for services and a spike in deaths during this season is, in part, driven by an increased number of people with multiple long term conditions.^5 6^

Multimorbidity, or multiple long term conditions, refers to the presence of two or more long term conditions. In 2015, an estimated 54% of people aged >65 years in England had multiple long term conditions and the number is projected to increase to almost 70% by 2035.^6^ People with multiple long term conditions use more healthcare services, with previous studies suggesting a sixfold higher risk of attendance at accident and emergency departments than people with no long term conditions.^7^,^9^ These individuals are also more likely to be admitted to hospitals and then readmitted after discharge.^8 10^

Previous large scale studies have already established the increased service demand related to multiple long term conditions during the winter season, which adds to pressures on the NHS service.^11 12^ This finding was identified as a critical priority by the National Institute for Health and Care Research, Health Data Research UK, and the Department of Health and Social Care.^13^ So far, an understanding of how multiple long term conditions affect the winter pressures on the NHS has been limited by the lack of granularity in the existing literature. Most studies examined the number of conditions or used higher level groupings of conditions rather than the granularity of individual conditions and their combinations.^8 14 15^

**Figure 1:**
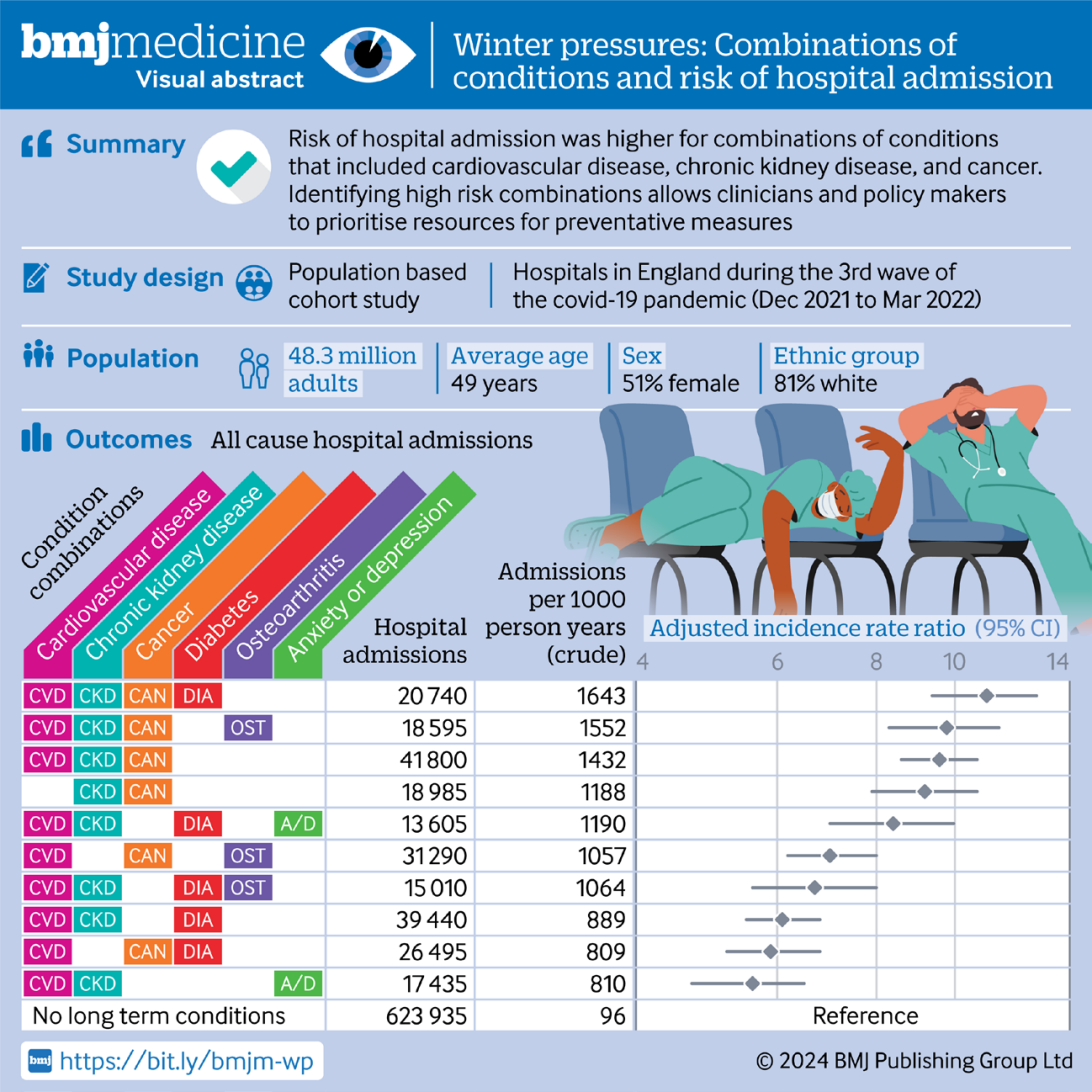
Visual abstract. CI=confidence interval

Given the rising demand for services, the strain on resources, and the challenges imposed on the NHS during the winter season,^16 17^ understanding the effect of combinations of multiple long term conditions in relation to hospital admission or death could highlight at risk groups and inform prioritisation of preventive interventions. In this study, our aim was to describe which combinations of long term conditions were associated with a higher risk of death and admission to hospital during winter 2021-22 (which overlapped with the third wave of the covid-19 pandemic) among adults in England with multiple long term conditions. Figure 1 shows the visual abstract.

## Methods

### Data source

In this retrospective cohort study, we used the General Practice Extraction Service Data for Pandemic Planning and Research (GDPPR), which has pseudoanonymised routinely collected electronic medical records for the whole population of England who are registered at a general practice. The GDPPR dataset was linked to the Hospital Episode Statistics Admitted Patient Care and the Office for National Statistics death registry. These datasets are available in the Secure Data Environment (SDE) service for England, established by NHS England.^18^ The SDE was accessed via the British Heart Foundation Data Science Centre's CVD-COVID-UK/COVID-IMPACT Consortium. The online supplemental material and elsewhere^19 20^ have more details on the premise of the CVD-COVID-UK/COVID-IMPACT Consortium and the datasets used in this study.

### Study design

Our study period was from 1 December 2021, the start of the winter pressure season, to 31 March 2022, the end of the winter season (which also coincided with the third wave of the covid-19 pandemic). We included all individuals registered in GDPPR who were alive and aged ≥18 years at our study start date. Follow-up was censored at the earliest event of death or the study end date (31 March 2022), with individuals followed up from the study start date (1 December 2021). All cause hospital admissions (from Hospital Episode Statistics Admitted Patient Care) and deaths (from the Office for National Statistics deaths registry) were recorded until the end of follow-up.

Our main exposure variable was the combination of long term conditions. Fifty nine long term conditions were selected based on a recent consensus of UK researchers.^21^ These 59 conditions (online supplemental table S1) were defined based on SNOMED (Systematised Nomenclature of Medicine, for primary care data) and ICD-10 (International Classification of Diseases, 10th revision, for secondary care data) codes. Online supplemental table S2 lists the SNOMED and ICD-10 codes used to define the 59 long term conditions.

The amount of missing data was small (<4%) in our cohort, and therefore we included only those individuals with complete sociodemographic variables, including age at the start of the study, sex (men or women), deprivation, and ethnic group (white, South Asian, black, or mixed or other). Data for sex were taken from information in the GDPPR database rather than from patient reported gender. Area based socioeconomic deprivation status was described by the 2019 English index of multiple deprivation, which is the official measure of deprivation in England.^22^ An individual's residential address was mapped with lower layer super output areas, which was then linked to the 2019 English index of multiple deprivation. The index of multiple deprivation ranking was further categorised into five groups, where groups 1 and 5 represented the most and least deprived areas, respectively.

### Statistical analysis

We described the prevalence of multiple long term conditions based on the 59 conditions selected. Subsequent analyses on the association between combinations of long term conditions and hospital admission or death were restricted to 19 long term conditions.

With 59 different long term conditions, the number of combinations (>500 000) was a challenge computationally and with limited clinical use. Therefore, we approached the analysis in two stages. In the first stage, after extensive discussions with clinicians, policy makers, and members of the patient and public involvement contributors, we retained 23 long term conditions that were considered important from clinical and population health perspectives. We further grouped them into 19 long term conditions (online supplemental table S1), and conducted data minimisation with an initial data exploration. We identified more than 52 000 unique combinations (among 19 conditions within our cohort). Several issues emerged: firstly, testing the outcomes in 52 000 combinations of multiple long term conditions would increase the type I error rate exponentially. Secondly, such a large number of comparison groups would mean a smaller number of outcome events in many groups, leading to imprecise effect estimates, especially after adjusting for other covariates. Thirdly, when considering the combinations of multiple long term conditions, rather than individual multiple long term conditions, increasing numbers of conditions in combinations of multiple long term conditions could mask important disease combinations (online supplemental figure S1 has an illustrative example). Lastly, given the limited consultation time in general practice and resources in primary care, handling such complex findings in the current clinical settings is almost impossible. Therefore, after extensive consultation with the practising clinicians, to balance the computational burden with clinical use, and considering the primary aim of this project, we reported 10 combinations of multiple long term conditions (not individual long term conditions) associated with the highest absolute number (volume) of hospital admissions or deaths (separately) during the winter season.

In the second stage of the analysis, after these 10 combinations of multiple long term conditions were identified, we used overdispersed Poisson regression models and estimated the incidence rate ratios with 95% confidence intervals (CI) of hospital admissions or deaths in winter associated with the combinations of multiple long term conditions compared with those with no long term conditions. Within the generalised linear model framework, we used the log of the follow-up time as an offset term (family=quasipoisson in R). We estimated the crude incidence rates of hospital admissions and deaths, and adjusted our regression models for age, sex, ethnic group, and deprivation. Data curation, cleaning, and exploratory analysis were performed with Python (version 3.7) and Spark SQL (version 2.4.5) on Databricks (version 6.4). Statistical analysis was conducted in R (version 4.0.3).

This analysis was performed according to a prespecified analysis plan published on GitHub, along with the phenotyping and analysis code (https://github.com/BHFDSC/CCU059_01).

### Patient and public involvement

Patient and public contributors had a crucial role in shaping our study through two rounds of meetings. We engaged 10 members of the public, all aged ≥18 years with multiple long term conditions, in discussions with the research team. The meetings focused on key concerns, such as the risks associated with living in cold homes during winter, access to adequate healthcare for existing conditions, and the financial effect of a potential increased need for health services. Their valuable insights provided a clearer perspective on these challenges and helped us refine the study's methodology to better deal with the real world concerns of those affected. Results will be disseminated through this publication, infographics, lay summary, electronic (eg, blogs, news) and social media.

## Results

We identified 50 057 280 individuals registered in GDPPR who were aged ≥18 years at the start of the study. Because of minimal missing data on sex, ethnic group, and deprivation (3.7% overall), we excluded those with missing records and conducted a complete case analysis in 48 253 125 individuals (online supplemental figure S2). Median follow-up time was 120 days (interquartile range 120-120 days). During the study period, we recorded 4 710 675 hospital admissions and 176 895 deaths. Overall, 19.7 million individuals (40.5%) had no long term conditions, 13.5 million individuals (27.9%) had one long term condition, and 15.1 million (31.2%) people had multiple long term conditions. Individuals with multiple long term conditions were older (mean age 61.4 (standard deviation (SD) ±17.9 years) compared with those with no long term conditions (39.9±15.5 years) or one long term condition (47.4±16.9 years), with a higher proportion of women (61.3% *v* 40.4% *v* 54.4%) and individuals from the white ethnic group (88.7% *v* 72.5% *v* 84.0%). The distribution of deprivation was similar across the three groups (table 1).

**Table 1:** Baseline characteristics of individuals in the cohort, analysed to identify the combinations of long term conditions associated with the highest risk of hospital admission and death during winter, 2021-22

Characteristics	No of long term conditions		
	0	1	Multiple (≥2)
No of individuals	19 706 155 (40.5)	13 475 240 (27.9)	15 071 730 (31.2)
Mean (SD) age (years)	39.9 (15.5)	47.4 (16.9)	61.4 (17.9)
Women	7 962 365 (40.4)	7 336 705 (54.4)	9 236 450 (61.3)
Ethnic group:			
Black	1 044 605 (5.3)	502 860 (3.7)	426 415 (2.8)
Mixed/other	2 482 290 (12.6)	843 050 (6.3)	562 440 (3.7)
South Asian	1 901 470 (9.6)	806 105 (6.0)	711 790 (4.7)
White	14 277 785 (72.5)	11 323 220 (84.0)	13 371 085 (88.7)
Index of multiple deprivation group:			
1 (most deprived)	3 977 500 (20.2)	2 561 345 (19.0)	3 107 125 (20.6)
2	4 438 705 (22.5)	2 691 690 (20.0)	3 033 610 (20.1)
3	4 022 450 (20.4)	2 749 735 (20.4)	3 072 100 (20.4)
4	3 716 290 (18.9)	2 752 445 (20.4)	3 020 925 (20.0)
5 (least deprived)	3 551 205 (18.0)	2 720 025 (20.2)	2 837 970 (18.8)

Data are number (%) unless indicated otherwise.

Numbers are rounded to the nearest 5, based on the NHS Data Access Environment safe output guidelines. These estimates are based on the 59 chronic conditions selected (online supplemental table S1).

SD, standard deviation.

### Hospital admissions

Table 2 lists the 10 combinations of multiple long term conditions that contributed to the highest rates of hospital admissions during the winter period. Cardiovascular disease appeared in all but one combinations, chronic kidney disease in eight combinations, and cancer in six. We included the rate of hospital admissions for those with one and no long term conditions for comparison (table 2).

**Table 2:** Number and crude incidence rates of admissions to hospital in winter, associated with the combinations of long term conditions

No of long term conditions	Combinations of long term conditions	Follow-up (years)	No of hospital admissions	Incidence rate per 1000 person years (95% CI)
4	Cancer, chronic kidney disease, cardiovascular disease, and type 2 diabetes mellitus	12 626	20 740	1642.6 (1620.3 to 1665.0)
4	Cancer, chronic kidney disease, cardiovascular disease, and osteoarthritis	11 982	18 595	1551.9 (1529.6 to 1574.2)
3	Cancer, chronic kidney disease, and cardiovascular disease	29 181	41 800	1432.4 (1418.7 to 1446.2)
4	Anxiety or depression, chronic kidney disease, cardiovascular disease, and type 2 diabetes mellitus	11 434	13 605	1189.9 (1169.9 to 1209.9)
2	Cancer and chronic kidney disease	15 981	18 985	1188.0 (1171.1 to 1204.9)
4	Chronic kidney disease, cardiovascular disease, type 2 diabetes mellitus, and osteoarthritis	14 102	15 010	1064.4 (1047.4 to 1081.4)
3	Cancer, cardiovascular disease, and osteoarthritis	29 595	31 290	1057.3 (1045.6 to 1069.0)
3	Chronic kidney disease, cardiovascular disease, and type 2 diabetes mellitus	44 354	39 440	889.2 (880.4 to 898.0)
3	Anxiety or depression, chronic kidney disease, and cardiovascular disease	21 530	17 435	809.8 (797.8 to 821.8)
3	Cancer, cardiovascular disease, and type 2 diabetes mellitus	32 768	26 495	808.6 (798.8 to 818.3)
1	One	4 428 164	943 535	213.1 (212.6 to 213.5)
0	None	6 477 947	623 935	96.3 (96.1 to 96.6)

Numbers are rounded to the nearest 5, based on the NHS Data Access Environment safe output guidelines. Combinations of multiple long term conditions are based on the final 19 long term conditions (online supplemental table S1).

CI, confidence interval.

The rate of hospital admissions (per 1000 person years) was higher among individuals with multiple long term conditions than in those with no long term conditions (>1600 *v* 96). The highest crude rates of hospital admissions were found for these combinations: 1643 per 1000 person years for the combination of cancer with chronic kidney disease, cardiovascular disease, and type 2 diabetes mellitus; and 1552 per 1000 person years for the combination of cancer with chronic kidney disease, cardiovascular disease, and osteoarthritis. The rates of hospital admissions increased in a dose-response way in individuals who had cardiovascular disease, osteoarthritis, and type 2 diabetes mellitus in addition to having both cancer and chronic kidney disease (table 2).

**Figure 2:**
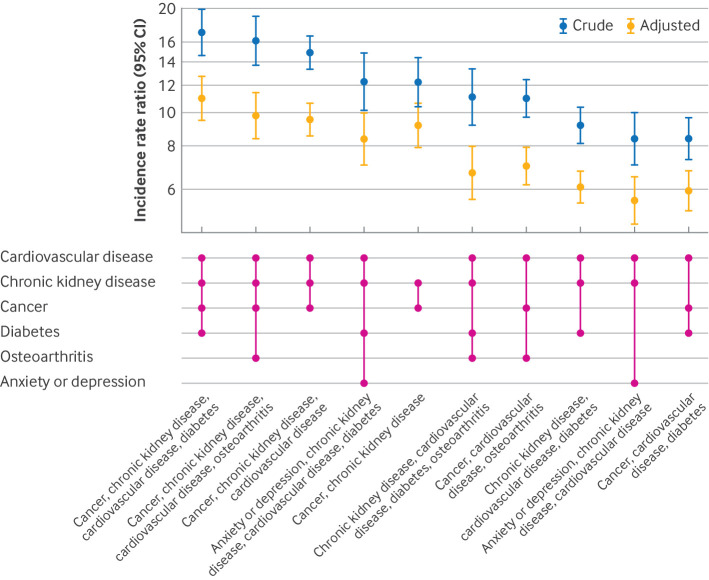
Crude and adjusted incidence rate ratios of admissions to hospital in winter, associated with the combinations of long term conditions compared with no long term conditions. Numbers are rounded to the nearest 5, based on the NHS Data Access Environment safe output guidelines. Combinations of multiple long term conditions are based on the final 19 long term conditions (online supplemental table S1). Estimates are adjusted for age, sex, ethnic group, and deprivation in the overdispersed Poisson regression models, with the log of the follow-up time as an offset term and with those with no long term conditions as the reference. CI=confidence interval

Compared with those with no long term conditions, the adjusted incidence rates of hospital admissions were 11.0 (95% CI 9.4 to 12.7) times higher for those with the combination of cancer, chronic kidney disease, cardiovascular disease, and type 2 diabetes mellitus, 9.8 (8.3 to 11.4) times higher for those with cancer, chronic kidney disease, cardiovascular disease, and osteoarthritis, and 9.6 (8.6 to 10.7) times higher for those with cancer, chronic kidney disease, and cardiovascular disease. The adjusted rate of hospital admissions was 6.1 (95% CI 5.5 to 6.8) times higher in those with chronic kidney disease, cardiovascular disease, and type 2 diabetes mellitus than in those with no long term conditions, which increased to 8.4 (7.0 to 10.0) when anxiety or depression was added to the combinations of multiple long term conditions (figure 2).

### Deaths

Table 3 lists the top 10 combinations of multiple long term conditions that contributed to the highest rates of mortality during the winter period. Cardiovascular disease appeared in all 10 of the combinations, and chronic kidney disease appeared in seven of the top 10 combinations (table 3).

**Table 3:** Number and crude incidence rates of deaths in winter, associated with the combinations of long term conditions

No of long term conditions	Combinations of long term conditions	Follow-up (years)	No of deaths	Incidence rate per 1000 person years (95% CI)
4	Cancer, chronic kidney disease, cardiovascular disease, and dementia	1343	465	346.2 (314.8 to 377.7)
4	Chronic kidney disease, cardiovascular disease, dementia, and osteoarthritis	2101	700	333.2 (308.5 to 357.9)
3	Chronic kidney disease, cardiovascular disease, and dementia	3560	1030	289.3 (271.7 to 307.0)
3	Cardiovascular disease, dementia, and osteoarthritis	2582	480	185.9 (169.3 to 202.5)
2	Cardiovascular disease and dementia	6244	1025	164.2 (154.1 to 174.2)
5	Cancer, chronic kidney disease, cardiovascular disease, type 2 diabetes mellitus, and osteoarthritis	5717	690	120.7 (111.7 to 129.7)
4	Cancer, chronic kidney disease, cardiovascular disease, and osteoarthritis	11 982	1310	109.3 (103.4 to 115.3)
4	Chronic kidney disease, cardiovascular disease, osteoarthritis, and osteoporosis	5242	520	99.2 (90.7 to 107.7)
3	Cancer, chronic obstructive pulmonary disease, and cardiovascular disease	5594	520	93.0 (85.0 to 100.9)
4	Cancer, chronic kidney disease, cardiovascular disease, and type 2 diabetes mellitus	12 626	1170	92.7 (87.4 to 98.0)
1	One	4 428 164	12 970	2.9 (2.9 to 3.0)
0	None	6 477 947	5080	0.8 (0.8 to 0.8)

Numbers are rounded to the nearest 5, based on the NHS Data Access Environment safe output guidelines. Combinations of multiple long term conditions are based on the final 19 long term conditions (online supplemental table S1).

CI, confidence interval.

**Figure 3:**
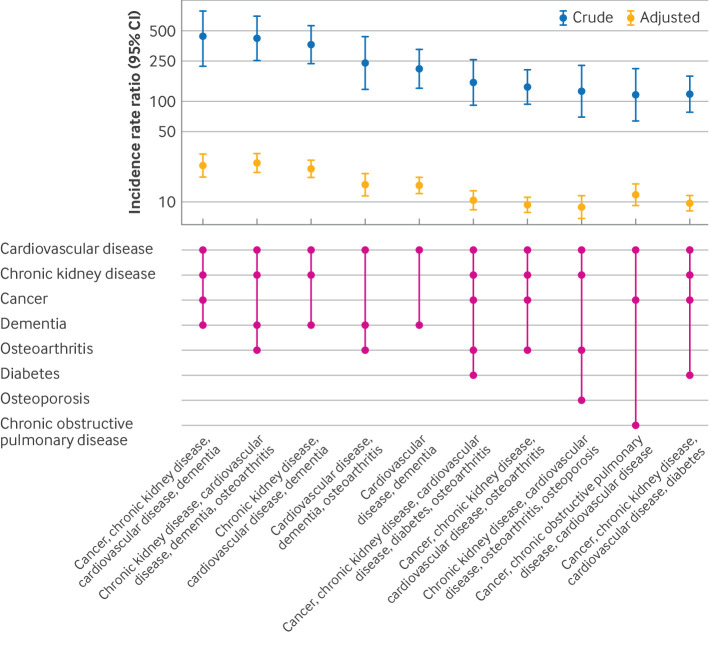
Crude and adjusted incidence rate ratios of deaths in winter, associated with the combinations of long term conditions compared with no long term conditions. Numbers are rounded to the nearest 5, based on the NHS Data Access Environment safe output guidelines. Combinations of multiple long term conditions are based on the final 19 long term conditions (online supplemental table S1). Estimates are adjusted for age, sex, ethnic group, and deprivation in the overdispersed Poisson regression models, with the log of the follow-up time as an offset term and with those with no long term conditions as the reference. CI=confidence interval

The rate of deaths was 1 per 1000 person years in people with no long term conditions; 346 per 1000 person years in people with a combination of cancer, chronic kidney disease, cardiovascular disease, and dementia; and 333 per 1000 person years in those with a combination of chronic kidney disease, cardiovascular disease, dementia, and osteoarthritis (table 3). Cardiovascular disease with dementia seemed to be the key combination that appeared in the top five combinations of multiple long term conditions. This two disease combination was associated with a substantially higher rate of death than many three, four, and five disease combinations. After adjusting for age, sex, ethnic group, and index of multiple deprivation, the rate of death was 14.6 (95% CI 12.0 to 17.8) times higher in people with cardiovascular disease and dementia than in those with no long term conditions. The adjusted rate ratio further increased to 21.4 (95% CI 17.5 to 26.0) in patients who also had chronic kidney disease (chronic kidney disease with cardiovascular disease and dementia), 23.2 (95% CI 17.5 to 30.3) in those who also had cancer and chronic kidney disease (cancer with chronic kidney disease, cardiovascular disease, and dementia), and 24.3 (95% CI 19.1 to 30.4) in those who also had chronic kidney disease and osteoarthritis (chronic kidney disease with cardiovascular disease, dementia, and osteoarthritis; figure 3).

## Discussion

### Principal findings

In this large population based cohort study of more than 48 million adults in England, we examined how the risk of hospital admission and death during the winter was associated with distinct combinations of long term conditions. In a whole population study, we found the highest risk of hospital admission in winter in individuals with the combination of cancer, chronic kidney disease, cardiovascular disease, and diabetes mellitus, and in those with a combination of cancer, chronic kidney disease, cardiovascular disease, and osteoarthritis. The highest rate of deaths, however, was in those with the combination of cancer, chronic kidney disease, cardiovascular disease, and dementia and those with chronic kidney disease, cardiovascular disease, dementia, and osteoarthritis.

### Comparison with other studies

Our study had a higher prevalence of multiple long term conditions than the previously reported 27.2% in the literature,^23^ possibly because of the population (the previous study used a subset of the English population whereas we used the whole population of England) and different definitions of multiple long term conditions. Our results align with earlier studies, however, that have consistently reported a substantial burden of multiple long term conditions in diverse populations, with a prevalence of 37.2% globally and 39.2% across Europe.^24^,^26^ In a recent systematic review in high, middle, and low income countries, a positive association was found between multiple long term conditions and hospital admission, with a 2.5 times higher risk than those with no long term conditions.^27^ Our study also estimated the risks of admission to hospital and death in winter associated with distinct combinations of multiple long term conditions.

### Study implications for research and practice

The cold weather during the winter season is associated with negative health outcomes and puts a major strain on public health services. The NHS in the UK is facing growing challenges in delivering healthcare services of high quality to the population.^28 29^ Research conducted in the UK has shown a pattern of increased hospital admissions during the winter months, specifically for respiratory diseases, which are strongly associated with the adverse effects of cold weather and respiratory infections. Several studies conducted in the UK also showed increased rates of hospital admissions during winter for other conditions, including asthma, falls, specific types of road traffic incidents, atrial fibrillation, heart failure, pulmonary embolism, stroke, and patients requiring intensive care.^30^,^32^ During the winter season, the NHS faces problems with capacity, influenced by several factors other than cold temperatures. These factors include the rising number of patients with chronic health conditions,^33 34^ delays in patient transfers between different healthcare settings, and an increased prevalence of communicable diseases, such as influenza, which tend to peak in winter.^35^,^37^ Hospital systems are under the most strain during the winter period, as hospital admissions reach their highest levels, largely because of an increase in respiratory illnesses associated with cold weather.^35 38^

Our analysis identified specific combinations of chronic conditions that were strongly associated with increased hospital admissions in winter. Cardiovascular disease was present in almost all of the top combinations, indicating the prominent role of cardiovascular disease in the increased use of healthcare services. This finding is consistent with previous research highlighting the substantial effect of cardiovascular disease on hospital admissions in individuals with multiple long term conditions.^39 40^ Furthermore, chronic kidney disease and cancer appeared frequently in the top combinations in our study, emphasising their substantial contributions to hospital admissions. These results corroborate the existing literature that emphasised the importance of comprehensive disease management approaches targeting cardiovascular disease, chronic kidney disease, and cancer to effectively reduce hospital admissions in individuals with multiple long term conditions.^41 42^

Jani et al conducted a study in 500 769 participants from the UK Biobank and identified cardiovascular diseases and cancer as common contributors to all cause mortality in individuals with multiple long term conditions.^40^ These consistent findings across diverse populations emphasise the universal burden of these conditions and highlight the need for comprehensive management strategies dealing with their co-occurrence.^43^,^45^ Potential mechanisms include, but not limited to, a higher burden of respiratory infections in winter, an increased vulnerability to viral infections caused by dysregulated immune responses, coexisting frailty and disability, and unmet social care needs. Also, patients with multiple long term conditions might have a compromised ability to cope with infection related stressors, such as fever or hypoxia.

In our all cause study, the combinations of cardiovascular disease with dementia and chronic kidney disease showed the highest rates of deaths. These findings align with previous research showing the adverse effect of chronic kidney disease and dementia on death.^46^ The substantially raised death rates found in these combinations compared with others emphasise the urgency of targeted interventions and effective management strategies for these conditions.

The high prevalence of multiple long term conditions and the effect on hospital admissions and death rates highlight the complex nature of managing multiple chronic conditions.^47^ The management of multiple long term conditions requires a comprehensive and patient centred approach that considers the interactions between different conditions, potential polypharmacy, and the unique needs of individual patients. Integrated care models that promote collaboration between healthcare professionals and include the active participation of patients are crucial in dealing with the challenges of multiple long term conditions.^48 49^

A person centred approach that focuses on personalised care planning, shared decision making, and coordinated management can help optimise outcomes for individuals with multiple long term conditions.^50^ Collaborative care models, such as the Chronic Care Model and the Guided Care Model, have shown promise in improving patient outcomes, reducing hospital admissions, and enhancing the quality of life for individuals with multiple long term conditions.^51 52^ Interventions targeting cardiovascular disease, chronic kidney disease, and dementia should prioritise the management of cardiovascular risk factors, including hypertension, diabetes, and hyperlipidaemia, while also dealing with cognitive impairment and promoting brain health.^43^,^45^ Cancer care pathways should be tailored to look at the unique needs of individuals with multiple long term conditions, considering potential interactions between cancer treatments and other chronic conditions.^53 54^

Our large scale study used a large sample size (more than 48 million adults in England) to report on multiple long term conditions in England and provided compelling evidence about the substantial burden of multiple long term conditions on hospital admissions and death rates during winter. Future research should focus on longitudinal studies to elucidate the temporal patterns and long term effect of multiple long term conditions on health outcomes. Furthermore, efforts should be directed towards developing integrated care models that look at the complex needs of individuals with multiple long term conditions during winter, particularly those with high risk combinations of chronic conditions.

### Strengths and limitations of this study

A key strength of our study was the large sample size of 48.2 million people covering most of the population in England. Our sample was representative and generalisable, and could quickly be applied to planning for winter pressures over the coming year. The study also had few missing data. We defined the long term conditions based on a combination of primary and secondary care data to maximise the coverage of conditions.

Our study had several limitations, including dependence on the electronic health records for coding of multiple long term conditions: electronic health records are routine clinical records and not necessarily of research standard. Also, grading of conditions was not performed, indicating possible over-representation or under-representation of conditions and their severity. A different set of long term conditions, and different decisions about how to combine and categorise conditions, could have produced different results. Conditions managed through self-care, over-the-counter treatment, private clinics, or screening programmes might not have been captured in these records. Our study focused on selecting long term conditions to inform public health policy. Inclusion of additional long term conditions, especially rare diseases, would increase the overall prevalence of multiple long term conditions.

Our study did not consider the length or severity of illness or frailty, or the sequence of long term conditions. Despite using a combination of primary (GDPPR which represents >90% of the SNOMED codes currently extracted by the General Practice Extraction Service) and secondary care data, underestimation of the burden of multiple long term conditions cannot be ruled out. The amount of missing data in our study was small, but could still introduce some biases, although we believe it would have no major effect on the study findings. Our analyses could not include those admitted to hospitals outside of England, or private hospitals, which are likely to be small. Because we focused on the top 10 combinations of long term conditions with the highest number of admissions to hospital or deaths to inform healthcare policy, rare conditions affecting smaller numbers of people were excluded, although these rare conditions could have had a disproportionately higher risk of these outcomes. This observational study overlapped with the covid-19 pandemic when substantial disruption occurred in health and social care provisions (eg, backlog, waitlists). Therefore, the findings should be interpreted with caution without direct causal association. Nevertheless, rapid availability of data at such a large scale during the pandemic through the consortium allowed us to examine population level trends and the effects of the pandemic. Our analysis did not consider the cause of hospital admissions or deaths, which could be explored in future research.

Finally, our objective was to estimate the healthcare burden of hospital admission and mortality rather than the individual risk of these outcomes. Therefore, the findings should not be interpreted as a guide for individual risk predictions. Future research should investigate whether we can integrate an improved stratification, incorporating more granular data, such as stages of disease severity, or other indicators of patient frailty and functionality, history, length of disease, effectiveness of the drug treatments used to treat the underlying conditions, and any social support received along with clinical care. This approach could help introduce more nuanced, patient centred new models of care in the future.

### Conclusions

In this study, we found that multiple long term conditions were associated with a higher risk of hospital admission and death. This risk varied by the combination of conditions. Current policy and clinical guidance consider the risk of hospital admission and death for multiple long term conditions during the winter season as one homogenous condition. By highlighting specific high risk combinations, our findings will inform planning for winter pressures on the NHS and help policy makers allocate resources where they are needed most.

## Supplementary material

10.1136/bmjmed-2024-001016 online supplemental file 2

## Data Availability

Data may be obtained from a third party and are not publicly available.

## References

[1] Charlton-Perez AJ, Aldridge RW, Grams CM (2019). Winter pressures on the UK health system dominated by the Greenland Blocking weather regime. Weather Clim Extrem.

[2] NHS winter pressures The king’s fund. https://www.kingsfund.org.uk/projects/nhs-winter-pressures.

[3] Barnett K, Mercer SW, Norbury M (2012). Epidemiology of multimorbidity and implications for health care, research, and medical education: a cross-sectional study. Lancet.

[4] Hewitt J, McCormack C, Tay HS (2016). Prevalence of multimorbidity and its association with outcomes in older emergency general surgical patients: an observational study. BMJ Open.

[5] Smith SM, Wallace E, O’Dowd T (2021). Interventions for improving outcomes in patients with multimorbidity in primary care and community settings. Cochrane Database Syst Rev.

[6] Humphreys J, Jameson K, Cooper C (2018). Early-life predictors of future multi-morbidity: results from the Hertfordshire Cohort. Age Ageing.

[7] Whitty CJM, MacEwen C, Goddard A (2020). Rising to the challenge of multimorbidity. BMJ.

[8] Stokes J, Guthrie B, Mercer SW (2021). Multimorbidity combinations, costs of hospital care and potentially preventable emergency admissions in England: A cohort study. PLoS Med.

[9] Hull SA, Homer K, Boomla K (2018). Population and patient factors affecting emergency department attendance in London: retrospective cohort analysis of linked primary and secondary care records. Br J Gen Pract.

[10] Nguyen H, Manolova G, Daskalopoulou C (2019). Prevalence of multimorbidity in community settings: A systematic review and meta-analysis of observational studies. *J Comorb*.

[11] Millwood S, Tomlinson P, Hopwood J (2021). Evaluation of winter pressures on general practice in Manchester: a cross-sectional analysis of nine GP practices. BJGP Open.

[12] Fares A (2013). Winter cardiovascular diseases phenomenon. *North Am J Med Sci*.

[13] Mahase E (2022). NHS England announces “data driven war rooms” to tackle winter pressures. BMJ.

[14] Koné Pefoyo AJ, Bronskill SE, Gruneir A (2015). The increasing burden and complexity of multimorbidity. BMC Public Health.

[15] MacRae C, McMinn M, Mercer SW (2023). The impact of varying the number and selection of conditions on estimated multimorbidity prevalence: A cross-sectional study using a large, primary care population dataset. PLoS Med.

[16] GOV.UK New discharge funding and NHS winter pressures. https://www.gov.uk/government/speeches/oral-statement-on-new-discharge-funding-and-nhs-winter-pressures.

[17] NHS England Winter pressures guidance. https://www.hee.nhs.uk/our-work/winter-pressures-guidance.

[18] NHS Digital Trusted research environment service for england. https://digital.nhs.uk/coronavirus/coronavirus-data-services-updates/trusted-research-environment-service-for-england.

[19] British Heart Foundation (BHF) Data Science Centre CVD-COVID-UK/COVID-IMPACT. https://bhfdatasciencecentre.org/areas/cvd-covid-uk-covid-impact/.

[20] Wood A, Denholm R, Hollings S (2021). Linked electronic health records for research on a nationwide cohort of more than 54 million people in England: data resource. BMJ.

[21] Dambha-Miller H, Farmer A, Nirantharakumar K (2023). Artificial Intelligence for Multiple Long-term conditions (AIM): A consensus statement from the NIHR AIM consortia. NIHR Open Res.

[22] Gov.uk (2019). The English indices of deprivation 2019. https://www.gov.uk/government/statistics/english-indices-of-deprivation-2019.

[23] Cassell A, Edwards D, Harshfield A (2018). The epidemiology of multimorbidity in primary care: a retrospective cohort study. Br J Gen Pract.

[24] Thanakiattiwibun C, Siriussawakul A, Virotjarumart T (2023). Multimorbidity, healthcare utilization, and quality of life for older patients undergoing surgery: A prospective study. Medicine (Baltimore).

[25] Chowdhury SR, Chandra Das D, Sunna TC (2023). Global and regional prevalence of multimorbidity in the adult population in community settings: a systematic review and meta-analysis. eClin Med.

[26] Kudesia P, Salimarouny B, Stanley M (2021). The incidence of multimorbidity and patterns in accumulation of chronic conditions: A systematic review. J Multimorb Comorb.

[27] Rodrigues LP, de Oliveira Rezende AT, Delpino FM (2022). Association between multimorbidity and hospitalization in older adults: systematic review and meta-analysis. Age Ageing.

[28] Ruane S (2019). Integrated care systems in the English NHS: a critical view. Arch Dis Child.

[29] Lacobucci G (2017). NHS in 2017: Keeping pace with society. BMJ.

[30] Patterson S (2018). Do hospital admission rates increase in colder winters? A decadal analysis from an eastern county in England. J Public Health (Oxf).

[31] Levin KA, Crighton EM (2016). Reshaping Care for Older People: Trends in emergency admissions to hospital during a period of simultaneous interventions in Glasgow City, April 2011-March 2015. Maturitas.

[32] McAllister DA, Morling JR, Fischbacher CM (2013). Socioeconomic deprivation increases the effect of winter on admissions to hospital with COPD: retrospective analysis of 10 years of national hospitalisation data. Prim Care Respir J.

[33] Damarell RA, Morgan DD, Tieman JJ (2020). General practitioner strategies for managing patients with multimorbidity: A systematic review and thematic synthesis of qualitative research. BMC Fam Pract.

[34] Smith SM, Wallace E, Clyne B (2021). Interventions for improving outcomes in patients with multimorbidity in primary care and community setting: a systematic review. Syst Rev.

[35] Office for National Statistics Excess winter mortality in England and Wales. https://www.ons.gov.uk/peoplepopulationandcommunity/birthsdeathsandmarriages/deaths/bulletins/excesswintermortalityinenglandandwales/2017to2018provisionaland2016to2017final.

[36] Kabir A, Tran A, Ansari S (2022). Impact of multimorbidity and complex multimorbidity on mortality among older Australians aged 45 years and over: a large population-based record linkage study. BMJ Open.

[37] Willadsen TG, Siersma V, Nicolaisdóttir DR (2018). Multimorbidity and mortality: A 15-year longitudinal registry-based nationwide Danish population study. J Comorb.

[38] Fleming DM, Taylor RJ, Haguinet F (2016). Influenza-attributable burden in United Kingdom primary care. Epidemiol Infect.

[39] Haug N, Deischinger C, Gyimesi M (2020). High-risk multimorbidity patterns on the road to cardiovascular mortality. BMC Med.

[40] Jani BD, Hanlon P, Nicholl BI (2019). Relationship between multimorbidity, demographic factors and mortality: findings from the UK Biobank cohort. BMC Med.

[41] de Boer IH, Khunti K, Sadusky T (2022). Diabetes Management in Chronic Kidney Disease: A Consensus Report by the American Diabetes Association (ADA) and Kidney Disease: Improving Global Outcomes (KDIGO). Diabetes Care.

[42] The Academy of Medical Sciences (2018). Multimorbidity: a priority for global health research. https://acmedsci.ac.uk/file-download/82222577.

[43] Tran VT, Diard E, Ravaud P (2021). Priorities to improve the care for chronic conditions and multimorbidity: a survey of patients and stakeholders nested within the ComPaRe e-cohort. BMJ Qual Saf.

[44] Veronese N, Koyanagi A, Dominguez LJ (2023). Multimorbidity increases the risk of dementia: a 15 year follow-up of the SHARE study. Age Ageing.

[45] Tinetti ME, Fried TR, Boyd CM (2012). Designing Health Care for the Most Common Chronic Condition—Multimorbidity. JAMA.

[46] Sullivan MK, Jani BD, McConnachie A (2021). Hospitalisation events in people with chronic kidney disease as a component of multimorbidity: parallel cohort studies in research and routine care settings. BMC Med.

[47] Bodenheimer T, Wagner EH, Grumbach K (2002). Improving Primary Care for Patients With Chronic Illness. JAMA.

[48] Muth C, Blom JW, Smith SM (2019). Evidence supporting the best clinical management of patients with multimorbidity and polypharmacy: a systematic guideline review and expert consensus. J Intern Med.

[49] Salisbury C, Man MS, Bower P (2018). Management of multimorbidity using a patient-centred care model: a pragmatic cluster-randomised trial of the 3D approach. Lancet.

[50] Michielsen L, Bischoff E, Schermer T (2023). Primary healthcare competencies needed in the management of person-centred integrated care for chronic illness and multimorbidity: Results of a scoping review. BMC Prim Care.

[51] Struckmann V, Leijten FRM, van Ginneken E (2018). Relevant models and elements of integrated care for multi-morbidity: Results of a scoping review. Health Policy.

[52] Boult C, Karm L, Groves C (2008). Improving chronic care: the 'guided care' model. Perm J.

[53] Blane DN, Lewandowska M (2019). Living with cancer and multimorbidity: the role of primary care. Curr Opin Support Palliat Care.

[54] Ahmad T, Gopal D, Dayem Ullah AZM (2022). Multimorbidity in patients living with and beyond cancer: protocol for a scoping review. BMJ Open.

