# Supplementary material for: Combinations of multiple long term conditions and risk of hospital admission or death during winter 2021-22 in England: population based cohort study
Source: BMJ Med. 2024 Nov 12;3(1):e001016. doi: 10.1136/bmjmed-2024-001016 (PMC11580288; doi:10.1136/bmjmed-2024-001016)
Supplement: online supplemental file 2 [file bmjmed-3-1-s001.pdf]

Combinations of multiple long-term conditions and risk of hospitalisation or death during winter 2021-22: population-based cohort study of 48 million people in England

Nazrul Islam, PhD[1], Sharmin Shabnam, PhD[2], Nusrat Khan, PhD[1], Clare Gillies, PhD[2], Francesco Zaccardi, PhD[2], Amitava Banerjee, DPhil[3], Vahé Nafilyan, PhD[4,5], Kamlesh Khunti, FMedSci[2], Hajira Dambha-Miller, PhD[1]

Affiliations:
[1]Primary Care Research Centre, University of Southampton, Southampton, UK
[2]Leicester Real World Evidence Unit, Diabetes Research Centre, University of Leicester, Leicester, UK
[3]Institute of Health Informatics, University College London, London, UK
[4]Office for National Statistics, Newport, UK
[5]Department of Public Health, London School of Hygiene & Tropical Medicine, London, UK

Supplementary Material

Contents

Supplementary Methods: 2

Supplementary Table S1: List of long-term conditions (LTCs)...........................4

Supplementary Table S2: Long-term conditions (LTCs) and a corresponding list of SNOMED and ICD10 codes...6

Supplementary Figure S1: Illustration of combinations of multiple long-term conditions and associated number of outcomes (hospitalisation or death)..243

Supplementary Figure S2: Flowchart of individuals within the cohort analysed to identify the combinations of long-term conditions associated with the highest risk of hospitalization and deaths during winter, 2021-2022.............244

Supplementary Methods

Data sources

GDPPR (primary care)

General Practice Extraction Service (GPES) Data for pandemic planning and research (GDPPR) database was used as a primary care data source [1]. Data validation and quality checks for GDPPR are carried out by the National Health Service (NHS) Digital Data Processing Service platform. The database contains approximately 98% of all general practices in England; covering almost 96% of the English population and excluding those who have explicitly requested an opt-out. GDPPR dataset includes patients' records coded with approximately 34,000 distinct SNOMED-CT concepts, representing over 90% of those currently extracted by GPES [2]. Previous studies using the dataset and details of the dataset can be found elsewhere [2,3].

Hospital Episode Statistics Admitted Patient Care (secondary care)

Hospital Episode Statistics (HES) is an administrative database that contains detailed data from hospitals within the NHS in England [4]. This dataset captures records of all patients, and their interactions on all inpatient admissions (Hospital Episode Statistics Admitted Patient Care – HES APC), outpatient appointments, and accidents and emergency attendances across English hospitals with regular quality checks [5]. HES includes data on patient demographics, diagnoses (using ICD-10 codes), procedures, and outcomes, making it essential for clinical epidemiology and healthcare policy.

Office for National Statistics (ONS) death registry

The Office for National Statistics (ONS) mortality dataset is a comprehensive source of information on deaths registered in In England (and Wales). This dataset provides comprehensive data on dates, and causes (including underlying; coded with ICD-10) of deaths, and are available from April 1997 [6]. The ONS mortality dataset is routinely used for public health monitoring, assessment of life expectancy, and epidemiological studies.

Data Linkage

Data sources described above are linked by the NHS Digital using the Master Person Service which produces a unique non-identifying key for linking multiple data sources [7]. NHS Digital publishes monthly reports for data quality maturity index highlighting a high-level accuracy of the linkage [8].

References

1. General Practice Extraction Service (GPES) Data for pandemic planning and research: a guide for analysts and users of the data [Internet]. NHS England Digital. [cited 2024 Sep 6]. Available from: https://digital.nhs.uk/coronavirus/gpes-data-for-pandemic-planning-and-research/guide-for-analysts-and-users-of-the-data

2. Wood A, Denholm R, Hollings S, Cooper J, Ip S, Walker V, et al. Linked electronic health records for research on a nationwide cohort of more than 54 million people in England: data resource. BMJ. 2021;373:n826.

3. Thygesen JH, Tomlinson C, Hollings S, Mizani MA, Handy A, Akbari A, et al. COVID-19 trajectories among 57 million adults in England: a cohort study using electronic health records. The Lancet Digital Health. 2022;4:e542–57.

4. Hospital Episode Statistics (HES) [Internet]. NHS England Digital. [cited 2024 Sep 6]. Available from: https://digital.nhs.uk/data-and-information/data-tools-and-services/data-services/hospital-episode-statistics

5. The processing cycle and HES data quality [Internet]. NHS England Digital. [cited 2024 Sep 6]. Available from: https://digital.nhs.uk/data-and-information/data-tools-and-services/data-services/hospital-episode-statistics/the-processing-cycle-and-hes-data-quality

6. Mortality statistics in England and Wales QMI - Office for National Statistics [Internet]. [cited 2024 Sep 6]. Available from: https://www.ons.gov.uk/peoplepopulationandcommunity/birthsdeathsandmarriages/deaths/methodologies/mortalitystatisticsinenglandandwalesqmi

7. Master Person Service (MPS) [Internet]. NHS England Digital. [cited 2024 Sep 6]. Available from: https://digital.nhs.uk/services/personal-demographics-service/master-person-service

8. Data quality [Internet]. NHS England Digital. [cited 2024 Sep 6]. Available from: https://digital.nhs.uk/data-and-information/data-tools-and-services/data-services/data-quality

Supplementary Table S1: List of long-term conditions (LTCs).

#	LTCs in the Original 59 Conditions	Inclusion in the final 19 LTCs
1	Addison's Disease	No
2	Anaemia	No
3	Anxiety	Yes
4	Depression	Merged and renamed as: Anxiety or Depression
5	Congenital Heart Disease	No
6	Chromosomal Abnormality	Merged and renamed as: Congenital Disease
7	Bipolar Disorder	Yes
8	Schizophrenia	Merged and renamed as: Bipolar Disorder/ Schizophrenia
9	Chronic Liver Disease	Yes
10	Alcohol-related Liver Disease (ARLD)	Merged and renamed as: Liver Disease
11	Aortic Aneurysm	No
12	Arrhythmia	No
13	Asthma	Yes
14	Autism	No
15	Bronchiectasis	No
16	Cancer	Yes
17	Cerebral Benign Tumors	No
18	Chronic Back Pain	No
19	Chronic Lyme Disease	No
20	Chronic Pancreatitis	No
21	Chronic Kidney Disease (CKD) stage 3-5	Yes
22	Chronic Pain	No
23	Chronic Obstructive Pulmonary Disease (COPD)	Yes
24	Chronic Urinary Tract Infections	No
25	Connective Tissue Disease	No
26	Coronary Heart Disease	Yes
27	Heart Failure	Merged and renamed as: Cardiovascular Diseases
28	Hypertension	
29	Cystic Fibrosis	No
30	Dementia	Yes
31	Type 2 Diabetes mellitus	Yes
32	Drug or Alcohol Misuse	No
33	Eating Disorders	No
34	Endometriosis Adenomyosis	No
35	Epilepsy	Yes
36	Gout	No
37	Hearing Loss	No
38	Hemi/Para/Quadriplegia	No
39	HIV/AIDS	No

**40**	Inflammatory Bowel Disease	**Yes**
**41**	Long COVID	**No**
**42**	Meniere's Disease	**No**
**43**	Multiple Sclerosis	**Yes**
**44**	Musculoskeletal Injury	**No**
**45**	Osteoarthritis	**Yes**
**46**	Osteoporosis	**Yes**
**47**	Parkinson's Disease	**Yes**
**48**	Peptic Ulcer Disease	**No**
**49**	Peripheral Neuropathy	**No**
**50**	Peripheral Vascular Disease	**Yes**
**51**	Post-Traumatic Stress Disorder	**No**
**52**	Sickle Cell Disease	**No**
**53**	Stroke	**Yes**
**54**	Thyroid disease	**Yes**
**55**	Transient Ischemic Attack	**No**
**56**	Tuberculosis	**No**
**57**	Valvular Diseases	**No**
**58**	Venous Thromboembolism	**No**
**59**	Visual Impairment	**No**

Supplementary Table S2: Long-term conditions (LTCs) and a corresponding list of SNOMED and ICD10 codes.

Name	Terminology	Code	Term
Addison's disease	ICD10	E271	Primary adrenocortical insufficiency
Addison's disease	ICD10	E272	Addisonian crisis
Addison's disease	ICD10	E351	Disorders of adrenal glands in diseases classified elsewhere
Addison's disease	ICD10	E896	Postprocedural adrenocortical(-medullary) hypofunction
Addison's disease	ICD10	G735	Myopathy in endocrine diseases
Addison's disease	SNOMED	16685009	Secondary adrenal insufficiency
Addison's disease	SNOMED	237692001	Secondary hypoadrenalism
Addison's disease	SNOMED	237744009	Other corticoadrenal overactivity
Addison's disease	SNOMED	237785004	Adrenal hypofunction
Addison's disease	SNOMED	24867002	Adrenal crisis
Addison's disease	SNOMED	275437005	Disorder of corticoadrenal overactivity
Addison's disease	SNOMED	34253008	Myopathy due to Addison's disease
Addison's disease	SNOMED	36102002	Acute adrenal insufficiency with meningococcal septicemia
Addison's disease	SNOMED	363732003	Addison's disease
Addison's disease	SNOMED	373662000	Primary hypoadrenalism
Addison's disease	SNOMED	386584007	Corticoadrenal insufficiency
Addison's disease	SNOMED	55003009	Adrenal medullary insufficiency
Addison's disease	SNOMED	721290005	Postprocedural adrenocortical hypofunction
Addison's disease	SNOMED	766986002	Addisonian crisis
Anaemia	ICD10	D46	Refractory anaemia
Anaemia	ICD10	D50	Iron deficiency anaemia
Anaemia	ICD10	D51	Vitamin B12 deficiency anaemia
Anaemia	ICD10	D52	Folate deficiency anaemia
Anaemia	ICD10	D53	Other nutritional anaemias
Anaemia	ICD10	D55	Anaemia due to enzyme disorders
Anaemia	ICD10	D56	Thalassaemia
Anaemia	ICD10	D57	Sickle-cell disorders
Anaemia	ICD10	D58	Other hereditary haemolytic anaemias
Anaemia	ICD10	D59	Acquired haemolytic anaemia
Anaemia	ICD10	D60	Acquired pure red cell aplasia [erythroblastopenia]
Anaemia	ICD10	D61	Other aplastic anaemias
Anaemia	ICD10	D62	Acute posthaemorrhagic anaemia
Anaemia	ICD10	D63	Anaemia in chronic diseases classified elsewhere
Anaemia	ICD10	D64	Other anaemias
Anaemia	ICD10	P61	Other perinatal hematological disorders
Anaemia	SNOMED	193213003	Myasthenic syndrome due to pernicious anaemia
Anaemia	SNOMED	234361004	Congenital deficiency of intrinsic factor
Anaemia	SNOMED	234362006	Biermer's congenital pernicious anaemia
Anaemia	SNOMED	84027009	Addison's anaemia
Anaemia	SNOMED	906551000006108	[RFC] Pernicious anaemia
Anxiety Or Depression	ICD10	F32	Depressive episode
Anxiety Or Depression	ICD10	F33	Recurrent depressive disorder
Anxiety Or Depression	ICD10	F40	Phobic anxiety disorders
Anxiety Or Depression	ICD10	F41	Other anxiety disorders
Anxiety Or Depression	SNOMED	102912007	Thanatophobia
Anxiety Or Depression	SNOMED	1086661000000108	Reactive depression, prolonged single episode (disorder)
Anxiety Or Depression	SNOMED	1089631000000109	Recurrent depression with current severe episode without psychotic features (disorder)
Anxiety Or Depression	SNOMED	1089641000000100	Recurrent depression with current moderate episode (disorder)
Anxiety Or Depression	SNOMED	1099001000119103	History of dysthymia (situation)
Anxiety Or Depression	SNOMED	111475002	Neurosis
Anxiety Or Depression	SNOMED	111490003	Panic disorder with agoraphobia, agoraphobic avoidance in partial remission AND severe panic attacks
Anxiety Or Depression	SNOMED	111491004	Panic disorder with agoraphobia, agoraphobic avoidance in full remission AND panic attacks in partial remission
Anxiety Or Depression	SNOMED	11941006	Panic disorder with agoraphobia, agoraphobic avoidance in full remission AND panic attacks in full remission
Anxiety Or Depression	SNOMED	12200008	Impaired insight
Anxiety Or Depression	SNOMED	1380006	Agoraphobia without history of panic disorder with limited symptom attacks
Anxiety Or Depression	SNOMED	1402001	Fear (specify)
Anxiety Or Depression	SNOMED	15639000	Moderate major depression, single episode (disorder)
Anxiety Or Depression	SNOMED	160332003	Family history of anxiety disorder
Anxiety Or Depression	SNOMED	161464003	H/O: psychiatric disorder
Anxiety Or Depression	SNOMED	161470009	H/O: anxiety state
Anxiety Or Depression	SNOMED	16264621000119109	Recurrent mild major depressive disorder co-occurrent with anxiety (disorder)
Anxiety Or Depression	SNOMED	16264901000119109	Recurrent moderate major depressive disorder co-occurrent with anxiety (disorder)
Anxiety Or Depression	SNOMED	16265951000119109	Mild major depressive disorder co-occurrent with anxiety single episode (disorder)

Anxiety Or Depression	SNOMED	16266831000119100	Moderate major depressive disorder co-occurrent with anxiety single episode (disorder)
Anxiety Or Depression	SNOMED	162720009	O/E - afraid
Anxiety Or Depression	SNOMED	1816003	Panic disorder with agoraphobia, severe agoraphobic avoidance AND mild panic attacks
Anxiety Or Depression	SNOMED	183399002	Antiphobic therapy
Anxiety Or Depression	SNOMED	185348006	Encounter for fear
Anxiety Or Depression	SNOMED	18818009	Moderate recurrent major depression (disorder)
Anxiety Or Depression	SNOMED	191601008	Single major depressive episode, mild (disorder)
Anxiety Or Depression	SNOMED	191610000	Recurrent major depressive episodes, mild (disorder)
Anxiety Or Depression	SNOMED	191611001	Recurrent major depressive episodes, moderate (disorder)
Anxiety Or Depression	SNOMED	191616006	Recurrent depression (disorder)
Anxiety Or Depression	SNOMED	191659001	Atypical depressive disorder (disorder)
Anxiety Or Depression	SNOMED	191708009	Chronic anxiety
Anxiety Or Depression	SNOMED	191709001	Recurrent anxiety
Anxiety Or Depression	SNOMED	191722009	Agoraphobia with panic attacks
Anxiety Or Depression	SNOMED	191723004	Agoraphobia without mention of panic attacks
Anxiety Or Depression	SNOMED	191724005	Social phobia, fear of eating in public
Anxiety Or Depression	SNOMED	191725006	Social phobia, fear of public speaking
Anxiety Or Depression	SNOMED	191726007	Social phobia, fear of public washing
Anxiety Or Depression	SNOMED	191728008	Fear of crowds
Anxiety Or Depression	SNOMED	191733007	Fear of pregnancy
Anxiety Or Depression	SNOMED	191744004	Writer's cramp neurosis
Anxiety Or Depression	SNOMED	191746002	Psychasthenic neurosis
Anxiety Or Depression	SNOMED	192049004	Prolonged depressive adjustment reaction (disorder)
Anxiety Or Depression	SNOMED	192080009	Chronic depression (disorder)
Anxiety Or Depression	SNOMED	19527009	Single episode of major depression in full remission (disorder)
Anxiety Or Depression	SNOMED	197480006	Anxiety disorder
Anxiety Or Depression	SNOMED	19766004	Panic disorder with agoraphobia, mild agoraphobic avoidance AND severe panic attacks
Anxiety Or Depression	SNOMED	198288003	Anxiety state
Anxiety Or Depression	SNOMED	19887002	Claustrophobia
Anxiety Or Depression	SNOMED	207363009	Anxiety neurosis
Anxiety Or Depression	SNOMED	21897009	Generalized anxiety disorder
Anxiety Or Depression	SNOMED	22230001	Panic disorder with agoraphobia, agoraphobic avoidance in partial remission AND panic attacks in full remission
Anxiety Or Depression	SNOMED	225624000	Panic attack
Anxiety Or Depression	SNOMED	228560001	AMT - Anxiety management training
Anxiety Or Depression	SNOMED	231499006	Endogenous depression first episode (disorder)
Anxiety Or Depression	SNOMED	231500002	Masked depression (disorder)
Anxiety Or Depression	SNOMED	231501003	Needle phobia
Anxiety Or Depression	SNOMED	231504006	Mixed anxiety and depressive disorder (disorder)
Anxiety Or Depression	SNOMED	231521002	Weight fixation
Anxiety Or Depression	SNOMED	238965007	Venereophobia
Anxiety Or Depression	SNOMED	238966008	Syphilophobia
Anxiety Or Depression	SNOMED	238976006	Bromisodrophobia
Anxiety Or Depression	SNOMED	247803002	Seasonal affective disorder (disorder)
Anxiety Or Depression	SNOMED	24781009	Panic disorder with agoraphobia, mild agoraphobic avoidance AND panic attacks in full remission
Anxiety Or Depression	SNOMED	247845000	Specific fear
Anxiety Or Depression	SNOMED	247853008	Fear of flying
Anxiety Or Depression	SNOMED	251000119105	Severe major depression, single episode (disorder)
Anxiety Or Depression	SNOMED	25501002	Social phobia
Anxiety Or Depression	SNOMED	25922000	Major depressive disorder, single episode with postpartum onset (disorder)
Anxiety Or Depression	SNOMED	268621008	Recurrent major depressive episodes (disorder)
Anxiety Or Depression	SNOMED	268957000	Neurotic condition, insight present
Anxiety Or Depression	SNOMED	268958005	Poor insight into neurotic condition
Anxiety Or Depression	SNOMED	274948002	Endogenous depression - recurrent (disorder)
Anxiety Or Depression	SNOMED	30059008	Panic disorder with agoraphobia, severe agoraphobic avoidance AND moderate panic attacks
Anxiety Or Depression	SNOMED	300706003	Endogenous depression (disorder)
Anxiety Or Depression	SNOMED	310495003	Mild depression (disorder)
Anxiety Or Depression	SNOMED	310496002	Moderate depression (disorder)
Anxiety Or Depression	SNOMED	310497006	Severe depression (disorder)
Anxiety Or Depression	SNOMED	313075001	Phobia counselling
Anxiety Or Depression	SNOMED	313224008	Dysmorphophobia
Anxiety Or Depression	SNOMED	3158007	Panic disorder with agoraphobia, agoraphobic avoidance in partial remission AND panic attacks in partial remission
Anxiety Or Depression	SNOMED	31781004	Panic disorder with agoraphobia, agoraphobic avoidance in partial remission AND mild panic attacks
Anxiety Or Depression	SNOMED	32388005	Panic disorder with agoraphobia, agoraphobic avoidance in partial remission AND moderate panic attacks
Anxiety Or Depression	SNOMED	33693007	Compensation neurosis
Anxiety Or Depression	SNOMED	34116005	Panic disorder with agoraphobia, agoraphobic avoidance in full remission AND severe panic attacks

Anxiety Or Depression	SNOMED	34563004	Cancerophobia
Anxiety Or Depression	SNOMED	35489007	Depressive disorder (disorder)
Anxiety Or Depression	SNOMED	35607004	Panic disorder with agoraphobia
Anxiety Or Depression	SNOMED	357705009	Cotard's syndrome (disorder)
Anxiety Or Depression	SNOMED	36474008	Severe recurrent major depression without psychotic features (disorder)
Anxiety Or Depression	SNOMED	36923009	Major depression, single episode (disorder)
Anxiety Or Depression	SNOMED	370143000	Major depressive disorder (disorder)
Anxiety Or Depression	SNOMED	371631005	Panic disorder (disorder)
Anxiety Or Depression	SNOMED	38328002	Panic disorder with agoraphobia, severe agoraphobic avoidance AND panic attacks in full remission
Anxiety Or Depression	SNOMED	38617005	Dental phobia
Anxiety Or Depression	SNOMED	386808001	Phobia (finding)
Anxiety Or Depression	SNOMED	386810004	Phobic disorder (disorder)
Anxiety Or Depression	SNOMED	395017009	C/O - panic attack (context-dependent category)
Anxiety Or Depression	SNOMED	397701000000102	[X]Severe depressive episode without psychotic symptoms (disorder)
Anxiety Or Depression	SNOMED	403593004	Phobic fear of skin cancer (disorder)
Anxiety Or Depression	SNOMED	40379007	Mild recurrent major depression (disorder)
Anxiety Or Depression	SNOMED	40568001	Recurrent brief depressive disorder (disorder)
Anxiety Or Depression	SNOMED	414371008	H/O: agoraphobia (context-dependent category)
Anxiety Or Depression	SNOMED	417620007	O/E - fearful mood (context-dependent category)
Anxiety Or Depression	SNOMED	417676004	O/E - panic attack (context-dependent category)
Anxiety Or Depression	SNOMED	430421000000104	[X]Mild depressive episode (disorder)
Anxiety Or Depression	SNOMED	442057004	Chronic depressive personality disorder
Anxiety Or Depression	SNOMED	465441000000108	[X]Moderate depressive episode (disorder)
Anxiety Or Depression	SNOMED	4932002	Panic disorder with agoraphobia, moderate agoraphobic avoidance AND mild panic attacks
Anxiety Or Depression	SNOMED	49564006	Panic disorder with agoraphobia, mild agoraphobic avoidance AND moderate panic attacks
Anxiety Or Depression	SNOMED	49971008	Apprehensive mood
Anxiety Or Depression	SNOMED	50983008	Panic disorder with agoraphobia, mild agoraphobic avoidance AND panic attacks in partial remission
Anxiety Or Depression	SNOMED	54307006	Zoophobia
Anxiety Or Depression	SNOMED	54587008	Simple phobia
Anxiety Or Depression	SNOMED	5509004	Panic disorder with agoraphobia AND severe panic attacks
Anxiety Or Depression	SNOMED	58963008	Acrophobia
Anxiety Or Depression	SNOMED	59923000	Panic disorder with agoraphobia AND panic attacks in full remission
Anxiety Or Depression	SNOMED	61212007	Panic disorder with agoraphobia, severe agoraphobic avoidance AND severe panic attacks
Anxiety Or Depression	SNOMED	61569007	Agoraphobia without history of panic disorder
Anxiety Or Depression	SNOMED	62351001	Generalized social phobia
Anxiety Or Depression	SNOMED	63701002	Panic disorder with agoraphobia, mild agoraphobic avoidance AND mild panic attacks
Anxiety Or Depression	SNOMED	63909006	Panic disorder with agoraphobia AND panic attacks in partial remission
Anxiety Or Depression	SNOMED	64060000	Panic disorder with agoraphobia, moderate agoraphobic avoidance AND panic attacks in full remission
Anxiety Or Depression	SNOMED	698957003	Depressive disorder in remission (disorder)
Anxiety Or Depression	SNOMED	70691001	Agoraphobia
Anxiety Or Depression	SNOMED	70747007	Major depression single episode, in partial remission (disorder)
Anxiety Or Depression	SNOMED	709475001	Assessment of fear (procedure)
Anxiety Or Depression	SNOMED	709477009	Assessment of fear about death
Anxiety Or Depression	SNOMED	710199005	Counselling about fear
Anxiety Or Depression	SNOMED	710841007	Assessment of anxiety (procedure)
Anxiety Or Depression	SNOMED	720453001	Moderately severe major depression single episode (disorder)
Anxiety Or Depression	SNOMED	74010007	Panic disorder with agoraphobia, severe agoraphobic avoidance AND panic attacks in partial remission
Anxiety Or Depression	SNOMED	75084000	Severe major depression without psychotic features (disorder)
Anxiety Or Depression	SNOMED	76441001	Severe major depression, single episode, without psychotic features (disorder)
Anxiety Or Depression	SNOMED	764611000000100	Recurrent major depressive episodes, severe (disorder)
Anxiety Or Depression	SNOMED	764691000000109	Recurrent major depressive episodes, in partial remission (disorder)
Anxiety Or Depression	SNOMED	76812003	Panic disorder with agoraphobia, moderate agoraphobic avoidance AND panic attacks in partial remission
Anxiety Or Depression	SNOMED	76868007	Panic disorder with agoraphobia, agoraphobic avoidance in full remission AND mild panic attacks
Anxiety Or Depression	SNOMED	78667006	Dysthymia (disorder)
Anxiety Or Depression	SNOMED	79298009	Mild major depression, single episode (disorder)
Anxiety Or Depression	SNOMED	8185002	Panic disorder with agoraphobia AND moderate panic attacks
Anxiety Or Depression	SNOMED	82415003	Agoraphobia without history of panic disorder without limited symptom attacks
Anxiety Or Depression	SNOMED	82738004	Panic disorder with agoraphobia, moderate agoraphobic avoidance AND moderate panic attacks
Anxiety Or Depression	SNOMED	832007	Moderate major depression (disorder)
Anxiety Or Depression	SNOMED	83458005	Agitated depression (disorder)
Anxiety Or Depression	SNOMED	83631006	Panic disorder with agoraphobia, moderate agoraphobic avoidance AND severe panic attacks

Anxiety Or Depression	SNOMED	87414006	Reactive depression (situational) (disorder)
Anxiety Or Depression	SNOMED	87512008	Mild major depression (disorder)
Anxiety Or Depression	SNOMED	87798009	Panic disorder with agoraphobia, agoraphobic avoidance in full remission AND moderate panic attacks
Anxiety Or Depression	SNOMED	89948007	Panic disorder with agoraphobia AND mild panic attacks
Aortic Aneurysm	ICD10	I71	Dissection of aorta [any part]
Aortic Aneurysm	SNOMED	12232008	Syphilitic aortic aneurysm
Aortic Aneurysm	SNOMED	123679006	Emerg replace aneurysm abdom aorta by anast aorta/aorta NEC
Aortic Aneurysm	SNOMED	14336007	Ruptured aneurysm of abdominal aorta
Aortic Aneurysm	SNOMED	161514008	H/O: aortic aneurysm
Aortic Aneurysm	SNOMED	1728190000000000	Infrarenal abdominal aortic aneurysm
Aortic Aneurysm	SNOMED	1728191000006108	Infrarenal abdominal aortic aneurysm
Aortic Aneurysm	SNOMED	1745991000006104	Aortic aneurysm screen abnormal
Aortic Aneurysm	SNOMED	1746570000000000	Aneurysm of suprarenal aorta
Aortic Aneurysm	SNOMED	1746571000006100	Aneurysm of suprarenal aorta
Aortic Aneurysm	SNOMED	175282008	Replacement of aneurysmal bifurcation of aorta NOS
Aortic Aneurysm	SNOMED	175283003	Emerg repl aneurysm bifurc aorta by anast aorta to fem art
Aortic Aneurysm	SNOMED	175284009	Replace aneurysm bifurc aorta by anast aorta to femoral art
Aortic Aneurysm	SNOMED	175297006	Emergency replacement of aneurysmal segment of aorta
Aortic Aneurysm	SNOMED	175298001	Emerg replace aneurysm asc aorta by anastom aorta to aorta
Aortic Aneurysm	SNOMED	175299009	Emerg replace aneurysm thor aorta by anastom aorta to aorta
Aortic Aneurysm	SNOMED	175300001	Emerg replace aneurysm suprarenal aorta by anast aorta/aorta
Aortic Aneurysm	SNOMED	175301002	Emerg replace aneurysm infrarenal aorta by anast aorta/aorta
Aortic Aneurysm	SNOMED	195258006	Thoracic aortic aneurysm which has ruptured
Aortic Aneurysm	SNOMED	195265003	Thoracoabdominal aortic aneurysm, ruptured
Aortic Aneurysm	SNOMED	195268001	Leaking abdominal aortic aneurysm
Aortic Aneurysm	SNOMED	225041000000107	Endovascular stenting for aorto-uniiliac aneurysm
Aortic Aneurysm	SNOMED	233370007	Other replacement of aneurysmal segment of aorta
Aortic Aneurysm	SNOMED	233374003	Y graft of abdominal Aortic aneurysm (emergency)
Aortic Aneurysm	SNOMED	233376001	Tube graft of Abdominal aortic aneurysm
Aortic Aneurysm	SNOMED	233377005	Tube graft abdominal Aortic aneurysm (emergency)
Aortic Aneurysm	SNOMED	233404000	OS translum ins stent graft for aneurysmal segment of aorta
Aortic Aneurysm	SNOMED	233984007	Thoracoabdominal aortic aneurysm, without mention of rupture
Aortic Aneurysm	SNOMED	233985008	AAA - Abdominal aortic aneurysm
Aortic Aneurysm	SNOMED	265492000	Emerg repl aneurysm bifurc aorta by anast aorta to iliac a
Aortic Aneurysm	SNOMED	265493005	Replace aneurysm bifurc aorta by anast aorta to iliac artery
Aortic Aneurysm	SNOMED	265499009	Transluminal operations on aneurysmal segment of aorta
Aortic Aneurysm	SNOMED	275037003	Y graft abdominal Aortic aneurysm
Aortic Aneurysm	SNOMED	281261000000105	Translum insert stent graft for aneurysmal segment of aorta
Aortic Aneurysm	SNOMED	281281000000101	Endovas insert of stent graft for suprarenal aortic aneurysm
Aortic Aneurysm	SNOMED	307701005	Endovas insert stent for aortic aneurysm of bifurcation NEC
Aortic Aneurysm	SNOMED	308546005	Dissecting aortic aneurysm
Aortic Aneurysm	SNOMED	314184006	Ruptured suprarenal aortic aneurysm
Aortic Aneurysm	SNOMED	314185007	Juxtarenal aortic aneurysm
Aortic Aneurysm	SNOMED	314186008	Inflammatory abdominal aortic aneurysm
Aortic Aneurysm	SNOMED	32907006	Operation on aneurysm of aorta NEC
Aortic Aneurysm	SNOMED	378481000000107	Endovascular insertion stent for suprarenal aortic aneurysm
Aortic Aneurysm	SNOMED	378491000000109	Endovascular stenting of thoracic aortic aneurysm
Aortic Aneurysm	SNOMED	426270006	Aneurysm of suprarenal aorta
Aortic Aneurysm	SNOMED	428809009	Endovas ins stent graft for infrarenal abdom aortic aneurysm
Aortic Aneurysm	SNOMED	429679003	Endov insertion of stent graft for thoracic aortic aneurysm
Aortic Aneurysm	SNOMED	433068007	Aneurysm of thoracic aorta
Aortic Aneurysm	SNOMED	441612006	Endovascul insert stent infrarenal abdominal aortic aneurysm
Aortic Aneurysm	SNOMED	444569004	Infrarenal abdominal aortic aneurysm
Aortic Aneurysm	SNOMED	448122008	Replace aneurysm abdominal aorta by anast aorta to aorta NEC
Aortic Aneurysm	SNOMED	449453007	Emerg bypass suprarenal aorta by anastom aorta to aorta NEC
Aortic Aneurysm	SNOMED	449530008	Emerg bypass infrarenal aorta by anastom aorta to aorta NEC
Aortic Aneurysm	SNOMED	450682002	Other replacement of aneurysmal segment of aorta NOS
Aortic Aneurysm	SNOMED	530151000000109	Abdominal aortic aneurysm without mention of rupture
Aortic Aneurysm	SNOMED	67362008	Aortic aneurysm
Aortic Aneurysm	SNOMED	698462006	Aortic aneurysm screening abnormal
Aortic Aneurysm	SNOMED	73067008	Ruptured aneurysm of aorta
Aortic Aneurysm	SNOMED	74883004	Thoracic aortic aneurysm
Aortic Aneurysm	SNOMED	809101000000106	Abdominal aortic aneurysm screen ultrasound scan abnormal
Aortic Aneurysm	SNOMED	824031000000109	Leaking thoracic aortic aneurysm
Arrhythmia	ICD10	I44	Atrioventricular and left bundle-branch block
Arrhythmia	ICD10	I451	Other and unspecified right bundle-branch block
Arrhythmia	ICD10	I453	Trifascicular block
Arrhythmia	ICD10	I455	Other specified heart block
Arrhythmia	ICD10	I46	Cardiac arrest
Arrhythmia	ICD10	I47	Paroxysmal tachycardia
Arrhythmia	ICD10	I48	Atrial fibrillation and flutter
Arrhythmia	ICD10	I49	Other cardiac arrhythmias
Arrhythmia	ICD10	P291	Neonatal cardiac dysrhythmia
Arrhythmia	SNOMED	1011821000006100	Adverse reaction to Drugs For Supraventricular Arrhythmias

Arrhythmia	SNOMED	1011831000006102	Adverse reaction to Drugs For Ventricular Arrhythmias
Arrhythmia	SNOMED	1025021000000107	High risk of atrial fibrillation
Arrhythmia	SNOMED	102594003	[D]Electrocardiogram (ECG) abnormal
Arrhythmia	SNOMED	1051851000000109	Renewal of dual chamber cardiac pacemaker system
Arrhythmia	SNOMED	1066831000000104	Atrial fibrillation detected
Arrhythmia	SNOMED	1067061000000104	Atrial fibrillation not detected
Arrhythmia	SNOMED	11092001	Sinus tachycardia
Arrhythmia	SNOMED	1110851000000100	QOF (Quality and Outcomes Framework) atrial fibrillation quality indicator-related care invitation
Arrhythmia	SNOMED	111288001	Ventricular flutter
Arrhythmia	SNOMED	11148001	Intracardiac electrophysiologic procedure with ECG
Arrhythmia	SNOMED	111975006	ECG: Q-T interval prolonged
Arrhythmia	SNOMED	118378005	Pacemaker pulse generator
Arrhythmia	SNOMED	119551000000000	Permanent cardiac pacemaker
Arrhythmia	SNOMED	119551000119102	Permanent cardiac pacemaker
Arrhythmia	SNOMED	120041000119109	Atrial fibrillation with rapid ventricular response
Arrhythmia	SNOMED	12026006	Bouveret-Hoffmann syndrome
Arrhythmia	SNOMED	129575004	Pacemaker twiddler's syndrome
Arrhythmia	SNOMED	1322501000000104	Recently performed a direct current defibrillation procedure
Arrhythmia	SNOMED	14106009	Cardiac pacemaker implant
Arrhythmia	SNOMED	142016009	ECG - improved
Arrhythmia	SNOMED	143601000000104	Evaluation of AF (atrial fibrillation) burden
Arrhythmia	SNOMED	149213005	Cardiac massage - open
Arrhythmia	SNOMED	1539441000006104	Stopping Implantable Cardiac Defib. (ICD) discussed with patient
Arrhythmia	SNOMED	1539551000006101	Consent given to stop Implantable Cardiac Defibrillator (ICD)
Arrhythmia	SNOMED	159649E+16	Atrial flutter type 2
Arrhythmia	SNOMED	161513002	H/O ventricular fibrillation
Arrhythmia	SNOMED	161692001	H/O: cardiac pacemaker in situ
Arrhythmia	SNOMED	162710004	O/E - collapse -cardiac arrest
Arrhythmia	SNOMED	164852001	Ambulatory ECG abnormal
Arrhythmia	SNOMED	164861001	ECG: myocardial ischaemia NOS
Arrhythmia	SNOMED	164865005	ECG: myocardial infarction
Arrhythmia	SNOMED	164867002	ECG: old myocardial infarction
Arrhythmia	SNOMED	164868007	ECG: antero-septal infarct
Arrhythmia	SNOMED	164869004	ECG:posterior/inferior infarct
Arrhythmia	SNOMED	164870003	ECG: subendocardial infarct
Arrhythmia	SNOMED	164871004	ECG: lateral infarction
Arrhythmia	SNOMED	164873001	ECG:left ventricle hypertrophy
Arrhythmia	SNOMED	164877000	ECG:right ventricle hypertrop.
Arrhythmia	SNOMED	164881000	ECG: ectopic beats
Arrhythmia	SNOMED	164883002	ECG: extrasystole
Arrhythmia	SNOMED	164884008	ECG: ventricular ectopics
Arrhythmia	SNOMED	164885009	ECG: atrial ectopics
Arrhythmia	SNOMED	164887001	Electrocardiographic supraventricular arrhythmia
Arrhythmia	SNOMED	164888006	Supraventricular arrhythmia absent on electrocardiogram
Arrhythmia	SNOMED	164889003	ECG: atrial fibrillation
Arrhythmia	SNOMED	164890007	ECG: atrial flutter
Arrhythmia	SNOMED	164891006	ECG: paroxysmal atrial tachy.
Arrhythmia	SNOMED	164893009	Electrocardiographic ventricular arrhythmia
Arrhythmia	SNOMED	164895002	ECG: ventricular tachycardia
Arrhythmia	SNOMED	164896001	EKG: ventricular fibrillation
Arrhythmia	SNOMED	164898000	EKG: heart block
Arrhythmia	SNOMED	164900003	ECG: partial sinu-atrial block
Arrhythmia	SNOMED	164901004	ECG: complete sinoatrial block
Arrhythmia	SNOMED	164902006	ECG:partial A-V block-long P-R
Arrhythmia	SNOMED	164903001	Electrocardiogram: partial atrioventricular block - 2:1
Arrhythmia	SNOMED	164904007	ECG: partial A-V block - 3:1
Arrhythmia	SNOMED	164905008	Mobitz type I incomplete atrioventricular block on electrocardiogram
Arrhythmia	SNOMED	164906009	ECG: complete A-V block
Arrhythmia	SNOMED	164907000	ECG: right bundle branch block
Arrhythmia	SNOMED	164909002	ECG: left bundle branch block
Arrhythmia	SNOMED	164912004	ECG: P wave abnormal
Arrhythmia	SNOMED	164913009	ECG: P mitrale
Arrhythmia	SNOMED	164914003	ECG: P pulmonale
Arrhythmia	SNOMED	164917005	ECG: Q wave abnormal
Arrhythmia	SNOMED	164918000	ECG: Q wave pathological
Arrhythmia	SNOMED	164921003	ECG: R wave abnormal
Arrhythmia	SNOMED	164922005	ECG: R wave tall
Arrhythmia	SNOMED	164925007	ECG: S wave abnormal
Arrhythmia	SNOMED	164926008	ECG: S wave deep
Arrhythmia	SNOMED	164928009	ECG S-T interval
Arrhythmia	SNOMED	164930006	ECG: S-T interval abnormal
Arrhythmia	SNOMED	164931005	ECG: S-T elevation
Arrhythmia	SNOMED	164934002	ECG: T wave abnormal
Arrhythmia	SNOMED	164937009	ECG: U wave abnormal

Arrhythmia	SNOMED	164938004	ECG: U wave exaggerated
Arrhythmia	SNOMED	164940009	ECG: F wave NOS
Arrhythmia	SNOMED	164941008	ECG: F wave absent
Arrhythmia	SNOMED	164942001	ECG: F wave present
Arrhythmia	SNOMED	164946003	ECG: P-R interval abnormal
Arrhythmia	SNOMED	164947007	Prolonged P-R interval
Arrhythmia	SNOMED	164951009	ECG: QRS complex abnormal
Arrhythmia	SNOMED	164956004	ECG: Q-T interval abnormal
Arrhythmia	SNOMED	165082004	Negative exercise ECG test
Arrhythmia	SNOMED	165084003	Exercise ECG positive
Arrhythmia	SNOMED	1656520000000000	Arrhythmogenic right ventricular cardiomyopathy
Arrhythmia	SNOMED	1656521000006109	Arrhythmogenic right ventricular cardiomyopathy
Arrhythmia	SNOMED	16797001	Accelerated atrioventricular conduction
Arrhythmia	SNOMED	17338001	Ventricular ectopic beats
Arrhythmia	SNOMED	175135009	Introduction of cardiac pacemaker system via vein
Arrhythmia	SNOMED	175137001	Resiting of lead of intravenous pacemaker system
Arrhythmia	SNOMED	175138006	Maintenance of battery of intravenous cardiac pacemaker syst
Arrhythmia	SNOMED	175140001	Removal of intravenous cardiac pacemaker system
Arrhythmia	SNOMED	175142009	Permanent cardiac pacemaker
Arrhythmia	SNOMED	175143004	Fixed-rate cardiac pacemaker
Arrhythmia	SNOMED	175144005	Implantation of intravenous triggered cardiac pacemaker
Arrhythmia	SNOMED	175145006	Implantation of intravenous atrial overdrive pacemaker
Arrhythmia	SNOMED	175146007	Implant intravenous pacemaker for atrial fibrillation
Arrhythmia	SNOMED	1772641000006109	H/O: diaphragmatic pacemaker in situ
Arrhythmia	SNOMED	17869006	Anomalous atrioventricular excitation
Arrhythmia	SNOMED	180325003	Cardioversion and stimulation
Arrhythmia	SNOMED	180327006	Internal electrode cardioversion
Arrhythmia	SNOMED	1823951000006107	Atrial fibrillation confirmed
Arrhythmia	SNOMED	1856431000006102	Atrial fibrillation follow-up
Arrhythmia	SNOMED	1932021000006101	3D study - problems with atrial fibrillation management
Arrhythmia	SNOMED	195039008	Partial atrioventricular block
Arrhythmia	SNOMED	195042002	Second degree atrioventricular block
Arrhythmia	SNOMED	195046004	Left main stem bundle branch block
Arrhythmia	SNOMED	195060002	Ventricular pre-excitation
Arrhythmia	SNOMED	195069001	PAT - Paroxysmal atrial tachycardia
Arrhythmia	SNOMED	195070000	Atrioventricular paroxysmal tachycardia
Arrhythmia	SNOMED	195071001	Junctional paroxysmal tachycardia
Arrhythmia	SNOMED	195072008	Nodal paroxysmal tachycardia
Arrhythmia	SNOMED	195080001	Atrial fibrillation and flutter
Arrhythmia	SNOMED	195083004	Ventricular fibrillation and flutter
Arrhythmia	SNOMED	195101003	Wandering atrial pacemaker
Arrhythmia	SNOMED	195103000	Bigeminal pulse
Arrhythmia	SNOMED	195105007	Re-entry ventricular arrhythmia
Arrhythmia	SNOMED	1951131000006106	Atrial fibrillation screening
Arrhythmia	SNOMED	1959541000006106	Time cardiopulmonary resuscitation commenced
Arrhythmia	SNOMED	196371000000102	Atrial fibrillation resolved
Arrhythmia	SNOMED	1971321000006108	Single lead ECG abnormal
Arrhythmia	SNOMED	1971331000006106	Single lead ECG normal
Arrhythmia	SNOMED	200061000000108	History of ventricular tachycardia
Arrhythmia	SNOMED	200071000000101	History of supraventricular tachycardia
Arrhythmia	SNOMED	2003711000006104	ECG: corrected QT interval (QTc)
Arrhythmia	SNOMED	2004071000006108	ECG: corrected QT interval (QTc) abnormal
Arrhythmia	SNOMED	2004081000006106	ECG: corrected QT interval (QTc) prolonged
Arrhythmia	SNOMED	2004091000006109	ECG: corrected QT interval (QTc) shortened
Arrhythmia	SNOMED	2006271000006108	Cardiac resynchronisation therapy defibrillator device in situ
Arrhythmia	SNOMED	2010181000006103	12 lead ECG abnormal
Arrhythmia	SNOMED	20143001	Other bilateral bundle branch block
Arrhythmia	SNOMED	20852007	Romano - Ward syndrome
Arrhythmia	SNOMED	213035005	Mechanical complication of cardiac pacemaker
Arrhythmia	SNOMED	213213007	Cardiac arrest as a complication of care
Arrhythmia	SNOMED	230361000000100	Other specified pacemaker testing
Arrhythmia	SNOMED	232334003	Replacement of cardiac pacemaker battery
Arrhythmia	SNOMED	232981000000109	Implantation of single chamber cardiac pacemaker system
Arrhythmia	SNOMED	232991000000106	Implantation of dual chamber cardiac pacemaker system
Arrhythmia	SNOMED	233169004	ICD - Internal cardiac defibrillator procedure
Arrhythmia	SNOMED	233170003	Implantation of automatic cardiac defibrillator
Arrhythmia	SNOMED	233174007	Other cardiac pacemaker system
Arrhythmia	SNOMED	233182007	Permanent cardiac pacemaker procedure
Arrhythmia	SNOMED	233183002	Insertion of permanent epicardial cardiac pacemaker system
Arrhythmia	SNOMED	233184008	Maintenance of cardiac pacemaker system NEC
Arrhythmia	SNOMED	233187001	Removal of implanted cardiac pacemaker system or components
Arrhythmia	SNOMED	233237000	Reinsertion of cardiac pacemaker battery
Arrhythmia	SNOMED	233531000000105	Cardioverter defibrillator introduced through the vein
Arrhythmia	SNOMED	233909000	Right ventricular outflow tract ventricular tachycardia
Arrhythmia	SNOMED	233910005	Lone atrial fibrillation

Arrhythmia	SNOMED	233911009	Non-rheumatic atrial fibrillation
Arrhythmia	SNOMED	233916004	Heart block
Arrhythmia	SNOMED	233917008	Atrioventricular block unspecified
Arrhythmia	SNOMED	233927002	Cardiac arrest with successful resuscitation
Arrhythmia	SNOMED	234172002	Cardiac arrest with electromechanical dissociation
Arrhythmia	SNOMED	234210006	Pacemaker electrode infection
Arrhythmia	SNOMED	234214002	Disorder of pacemaker pulse generator
Arrhythmia	SNOMED	234228008	ICD - Disorder of implantable cardiac defibrillator
Arrhythmia	SNOMED	236721000000106	Implantat intravenous dual chamber cardiac pacemaker system
Arrhythmia	SNOMED	237771000000107	Distant pacemaker test
Arrhythmia	SNOMED	24607002	ECG: Q wave NOS
Arrhythmia	SNOMED	248411000000105	Atrial fibrillation annual review
Arrhythmia	SNOMED	248629002	Pulse missed beats
Arrhythmia	SNOMED	248871000000102	Problem with cardiac pacemaker
Arrhythmia	SNOMED	250980009	Cardioversion
Arrhythmia	SNOMED	251120003	Incomplete left bundle branch block
Arrhythmia	SNOMED	251124007	Incomplete right bundle branch block
Arrhythmia	SNOMED	251135002	Borderline normal ECG
Arrhythmia	SNOMED	251136001	Borderline abnormal ECG
Arrhythmia	SNOMED	251156000	Wide QRS ventricular tachycardia
Arrhythmia	SNOMED	251157009	Narrow QRS ventricular tachycardia
Arrhythmia	SNOMED	251164006	Junctional premature depolarization
Arrhythmia	SNOMED	251175005	Ventricular premature depolarization
Arrhythmia	SNOMED	251269006	Ineffective artificial pacemaker pattern
Arrhythmia	SNOMED	25267002	Insertion of intracardiac pacemaker
Arrhythmia	SNOMED	25569003	Ventricular tachycardia
Arrhythmia	SNOMED	26141007	ECG: S-T depression
Arrhythmia	SNOMED	265482008	Emergency cardiac pacemaker
Arrhythmia	SNOMED	266690007	Closed cardiac massage with ventilation
Arrhythmia	SNOMED	26879000	Atrial cardioversion
Arrhythmia	SNOMED	270492004	First degree atrioventricular block
Arrhythmia	SNOMED	275148007	External electrode cardioversion
Arrhythmia	SNOMED	275531008	H/O: pacemaker in situ
Arrhythmia	SNOMED	276259003	Respiratory arrest preceding cardiac arrest
Arrhythmia	SNOMED	276308001	General maintenance of cardiac pacemaker
Arrhythmia	SNOMED	276513001	Neonatal cardiac dysrhythmia
Arrhythmia	SNOMED	27885002	Complete atrioventricular block
Arrhythmia	SNOMED	28011000033102	Drugs For Supraventricular Arrhythmias
Arrhythmia	SNOMED	28111000033103	Drugs For Ventricular Arrhythmias
Arrhythmia	SNOMED	281170005	Arrhythmogenic right ventricular cardiomyopathy
Arrhythmia	SNOMED	281556002	Insertion of temporary cardiac pacemaker
Arrhythmia	SNOMED	28189009	Mobitz type II atrioventricular block
Arrhythmia	SNOMED	282825002	Intermittent atrial fibrillation
Arrhythmia	SNOMED	28630001	ECG: QRS complex shortened
Arrhythmia	SNOMED	287057009	Atrial premature depolarization
Arrhythmia	SNOMED	287285004	Cardiac pacemaker replaced
Arrhythmia	SNOMED	287699009	Emergency cardiac pacemaker
Arrhythmia	SNOMED	29717002	Extrasystoles
Arrhythmia	SNOMED	300996004	Controlled atrial fibrillation
Arrhythmia	SNOMED	30667004	Right BBB with left anterior fascicular block
Arrhythmia	SNOMED	307280005	Implantation of cardiac pacemaker system NEC
Arrhythmia	SNOMED	308805008	Resiting of lead of cardiac pacemaker system NEC
Arrhythmia	SNOMED	308842001	Defibrillation
Arrhythmia	SNOMED	309405007	Implantation simple one wire intravenous cardiac pacemaker
Arrhythmia	SNOMED	309407004	Implantation of complex 1 wire intravenous cardiac pacemaker
Arrhythmia	SNOMED	309408009	Implantation complex two wire intravenous cardiac pacemaker
Arrhythmia	SNOMED	309471004	Implantation of temporary intravenous cardiac pacemaker
Arrhythmia	SNOMED	309809007	Electromechanical dissociation with successful resuscitation
Arrhythmia	SNOMED	310582005	Implantation of intravenous dual chamber permanent pacemaker
Arrhythmia	SNOMED	312442005	History of atrial fibrillation
Arrhythmia	SNOMED	314208002	Rapid atrial fibrillation
Arrhythmia	SNOMED	314775002	Seen by cardiac pacemaker technician
Arrhythmia	SNOMED	32758004	Right bundle branch block with left bundle branch block
Arrhythmia	SNOMED	33413000	Ectopic beats
Arrhythmia	SNOMED	34450008	Insertion of neural pacemaker into peripheral nerve
Arrhythmia	SNOMED	346011000	Percutaneous transluminal internal cardioversion NEC
Arrhythmia	SNOMED	3515001	Replacement of pacemaker pulse generator
Arrhythmia	SNOMED	35797001	Implantation of pacemaker in brain
Arrhythmia	SNOMED	36083008	Sick sinus syndrome
Arrhythmia	SNOMED	361137007	Heart beats irregular
Arrhythmia	SNOMED	36936009	Major thoracotomy with cardiac massage
Arrhythmia	SNOMED	370359005	ECG equivocal
Arrhythmia	SNOMED	370365005	ECG: left ventricular strain
Arrhythmia	SNOMED	373905003	Jervell and Lange-Nielsen syndrome
Arrhythmia	SNOMED	37760005	Left anterior fascicular block

Arrhythmia	SNOMED	378181000000100	Implantat cardiovert defibrillator us three electrode leads
Arrhythmia	SNOMED	384683008	Replacement of any type of pacemaker device with dual-chamber device
Arrhythmia	SNOMED	38566003	Right bundle branch block, anterior fascicular block AND incomplete left bundle branch block
Arrhythmia	SNOMED	39438000	ECG: U wave NOS
Arrhythmia	SNOMED	395218007	Implantation of internal cardiac defibrillator
Arrhythmia	SNOMED	395224001	Removal of internal cardiac defibrillator
Arrhythmia	SNOMED	39732003	Left axis deviation
Arrhythmia	SNOMED	397829000	Asystole
Arrhythmia	SNOMED	398665005	[D]Asystolic vasovagal syncope
Arrhythmia	SNOMED	400972008	Renewal of intravenous cardiac pacemaker system
Arrhythmia	SNOMED	41011002	ECG: T wave NOS
Arrhythmia	SNOMED	410361000000108	[V]Fitting or adjustment of neuropacemaker device
Arrhythmia	SNOMED	410429000	Cardiac arrest
Arrhythmia	SNOMED	410430005	Cardio-respiratory arrest
Arrhythmia	SNOMED	418818005	Brugada syndrome
Arrhythmia	SNOMED	422970001	Cardiac arrest due to trauma
Arrhythmia	SNOMED	423191000	Cardiac arrest due to cardiac disorder
Arrhythmia	SNOMED	425435003	Implantat cardioverter defibrillator us one electrode lead
Arrhythmia	SNOMED	425934009	Other cardioverter defibrillator
Arrhythmia	SNOMED	426083000	ECG: pacemaker active
Arrhythmia	SNOMED	426177001	ECG: sinus bradycardia
Arrhythmia	SNOMED	426183003	Electrocardiogram: Mobitz type 2 second degree AV block
Arrhythmia	SNOMED	426220008	External ventricular defibrillation
Arrhythmia	SNOMED	426453001	ECG: paced rhythm
Arrhythmia	SNOMED	426525004	Sustained ventricular tachycardia
Arrhythmia	SNOMED	426627000	ECG: bradycardia
Arrhythmia	SNOMED	426749004	Chronic atrial fibrillation
Arrhythmia	SNOMED	426761007	ECG: supraventricular tachycardia
Arrhythmia	SNOMED	426783006	ECG sinus rhythm
Arrhythmia	SNOMED	426995002	ECG: junctional escape rhythm
Arrhythmia	SNOMED	427084000	ECG: sinus tachycardia
Arrhythmia	SNOMED	427393009	ECG: sinus arrhythmia
Arrhythmia	SNOMED	427485008	ECG: asystole
Arrhythmia	SNOMED	427508005	Implant cardioverter defibrillator using two electrode leads
Arrhythmia	SNOMED	427665004	Paroxysmal atrial flutter
Arrhythmia	SNOMED	42807005	Pulsus alternans
Arrhythmia	SNOMED	428076002	History of atrial flutter
Arrhythmia	SNOMED	428625001	Renewal of cardioverter defibrillator
Arrhythmia	SNOMED	429007001	History of cardiac arrest
Arrhythmia	SNOMED	429064006	Implantation of biventricular cardiac pacemaker system
Arrhythmia	SNOMED	429286003	History of combination internal cardiac defibrillator and pacemaker
Arrhythmia	SNOMED	429528001	Implantat intraven single chamber cardiac pacemaker system
Arrhythmia	SNOMED	429542009	Implantat intravenous biventricular cardiac pacemaker system
Arrhythmia	SNOMED	431267001	Advanced cardiopulmonary resuscitation
Arrhythmia	SNOMED	431415002	Management of internal defibrillation
Arrhythmia	SNOMED	432084000	Management of permanent pacemaker
Arrhythmia	SNOMED	433844000	Insertion of cardiac biventricular implantable cardioverter defibrillator (ICD) using fluoroscopic guidance
Arrhythmia	SNOMED	434417009	Insertion of cardiac biventricular permanent pacemaker (PPM) using fluoroscopic guidance
Arrhythmia	SNOMED	440028005	Permanent atrial fibrillation
Arrhythmia	SNOMED	440059007	Persistent atrial fibrillation
Arrhythmia	SNOMED	440142000	Chemical cardioversion
Arrhythmia	SNOMED	441481000000107	[V]Fitting or adjustment of cardiac pacemaker
Arrhythmia	SNOMED	441509002	[V]Cardiac pacemaker in situ
Arrhythmia	SNOMED	443325000	Automatic implantable cardiac defibrillator in situ
Arrhythmia	SNOMED	444179007	Insertion of dual chamber pacemaker pulse generator
Arrhythmia	SNOMED	444566006	Replacement of cardiac pacemaker
Arrhythmia	SNOMED	444658006	Nonsustained ventricular tachycardia
Arrhythmia	SNOMED	445118002	ECG: left anterior fascicular block
Arrhythmia	SNOMED	445211001	Left posterior fascicular block on ECG (electrocardiogram)
Arrhythmia	SNOMED	445263008	ECG: right bundle branch and left anterior fascicular block
Arrhythmia	SNOMED	445309007	ECG: trifascicular block
Arrhythmia	SNOMED	445393004	ECG: right bundle branch and left posterior fascicular block
Arrhythmia	SNOMED	445481009	Bifascicular block on ECG (electrocardiogram)
Arrhythmia	SNOMED	44602002	Persistent sinus bradycardia
Arrhythmia	SNOMED	447365002	Insertion of biventricular implantable cardioverter defibrillator
Arrhythmia	SNOMED	44808001	Cardiac dysrhythmias NOS
Arrhythmia	SNOMED	448150008	Interrogation of cardiac pacemaker
Arrhythmia	SNOMED	448590001	Maintenance of intravenous cardiac pacemaker system NEC
Arrhythmia	SNOMED	449397007	Insertion of permanent cardiac pacemaker pulse generator and electrode
Arrhythmia	SNOMED	449863006	Insertion of pacemaker for control of atrial fibrillation
Arrhythmia	SNOMED	450475007	For cardiopulmonary resuscitation
Arrhythmia	SNOMED	450476008	Not for attempted CPR (cardiopulmonary resuscitation)

Arrhythmia	SNOMED	450811002	Maintenance of battery of cardiac pacemaker system NEC
Arrhythmia	SNOMED	450919004	Atrial standstill
Arrhythmia	SNOMED	46319007	Right BBB with left posterior fascicular block
Arrhythmia	SNOMED	46634008	Resiting of lead of cardioverter defibrillator
Arrhythmia	SNOMED	46935006	Stokes-Adams syndrome
Arrhythmia	SNOMED	47665007	Right axis deviation
Arrhythmia	SNOMED	49044005	Severe sinus bradycardia
Arrhythmia	SNOMED	49436004	Atrial fibrillation
Arrhythmia	SNOMED	49578007	ECG: P-R interval shortened
Arrhythmia	SNOMED	4973001	Left bundle branch hemiblock
Arrhythmia	SNOMED	53359008	Insertion of neural pacemaker in spine
Arrhythmia	SNOMED	5370000	Atrial flutter
Arrhythmia	SNOMED	54016002	Mobitz type I incomplete atrioventricular block
Arrhythmia	SNOMED	54356007	Insertion of carotid pacemaker
Arrhythmia	SNOMED	55455004	Revision of pacemaker electrode leads
Arrhythmia	SNOMED	55475008	Lown-Ganong-Levine syndrome
Arrhythmia	SNOMED	586241000000000	Maintenance of intravenous cardiac pacemaker system NEC
Arrhythmia	SNOMED	586241000000107	Maintenance of intravenous cardiac pacemaker system NEC
Arrhythmia	SNOMED	586261000000108	Cardiac pacemaker system introduced via vein NOS
Arrhythmia	SNOMED	586271000000000	Other cardiac pacemaker system
Arrhythmia	SNOMED	586271000000101	Other cardiac pacemaker system
Arrhythmia	SNOMED	58863009	Initial insertion of cardiac pacemaker dual-chamber device
Arrhythmia	SNOMED	589971000000000	Implantation of cardiac pacemaker system NEC
Arrhythmia	SNOMED	589971000000101	Implantation of cardiac pacemaker system NEC
Arrhythmia	SNOMED	59118001	[X]Other and unspecified right bundle-branch block
Arrhythmia	SNOMED	593361000000108	Cardiac pacemaker removed
Arrhythmia	SNOMED	593381000000104	Other cardiac pacemaker system NOS
Arrhythmia	SNOMED	59632000	Cardiopulmonary resuscitation education
Arrhythmia	SNOMED	59931005	ECG: T wave inverted
Arrhythmia	SNOMED	60423000	Sinoatrial node dysfunction NOS
Arrhythmia	SNOMED	609462000	Termination of pregnancy complicated by defibrination syndrome
Arrhythmia	SNOMED	609506003	Cardiac arrest following abortive pregnancy
Arrhythmia	SNOMED	6180003	Complete left bundle branch block
Arrhythmia	SNOMED	62026008	Left posterior fascicular block
Arrhythmia	SNOMED	621621000000108	Other heart block
Arrhythmia	SNOMED	62881002	Removal of permanent pacemaker
Arrhythmia	SNOMED	630621000000100	Cardiac dysrhythmia NOS
Arrhythmia	SNOMED	63467002	Left bundle branch block
Arrhythmia	SNOMED	63593006	Supraventricular extrasystoles
Arrhythmia	SNOMED	6374002	Other bundle branch block
Arrhythmia	SNOMED	6456007	Supraventricular tachycardia NOS
Arrhythmia	SNOMED	65778007	Sinoatrial block
Arrhythmia	SNOMED	66657009	Paroxysmal ventricular tachycardia
Arrhythmia	SNOMED	66812002	ECG: T wave flattened
Arrhythmia	SNOMED	67198005	Paroxysmal supraventricular tachycardia
Arrhythmia	SNOMED	682581000000000	Cardiac dysrhythmias NOS
Arrhythmia	SNOMED	682581000000105	Cardiac dysrhythmias NOS
Arrhythmia	SNOMED	686041000000108	External cardioversion NEC
Arrhythmia	SNOMED	68971000119104	Biventricular automatic implantable cardioverter defibrillator in situ
Arrhythmia	SNOMED	69031000119105	Presence of biventricular cardiac pacemaker
Arrhythmia	SNOMED	697953001	Maintenance of automatic cardiac defibrillator
Arrhythmia	SNOMED	698247007	Other cardiac dysrhythmias
Arrhythmia	SNOMED	699125007	Insertion of programmable cardiac pacemaker
Arrhythmia	SNOMED	699135001	Implantation of cardiac defibrillator lead
Arrhythmia	SNOMED	699833006	Provision of written information about atrial fibrillation
Arrhythmia	SNOMED	700223001	Automatic implantable cardioverter defibrillator check
Arrhythmia	SNOMED	702896001	Pacemaker clinic
Arrhythmia	SNOMED	703162001	Bradycardic cardiac arrest
Arrhythmia	SNOMED	703396000	Patient with internal cardiac defibrillator pacemaker
Arrhythmia	SNOMED	704708004	Cardiac resynchronisation therapy implantable pacemaker
Arrhythmia	SNOMED	704722004	Dual-chamber implantable pacemaker, rate-responsive
Arrhythmia	SNOMED	706004007	Implantable cardiac pacemaker
Arrhythmia	SNOMED	707832008	Perc transluminal ablation of atrial wall for atrial flutter
Arrhythmia	SNOMED	711166001	Removal of implantable cardiac pacemaker
Arrhythmia	SNOMED	713426002	ECG: Incomplete right bundle branch block
Arrhythmia	SNOMED	71792006	Sinus arrhythmia
Arrhythmia	SNOMED	719008003	At risk of atrial fibrillation
Arrhythmia	SNOMED	71908006	Ventricular fibrillation
Arrhythmia	SNOMED	720448006	Typical atrial flutter
Arrhythmia	SNOMED	736702002	Revision of biventricular permanent pacemaker lead using fluoroscopic guidance
Arrhythmia	SNOMED	74021003	Bifascicular block
Arrhythmia	SNOMED	74390002	Wolff-Parkinson-White syndrome
Arrhythmia	SNOMED	758600000	Referral to atrial fibrillation clinic
Arrhythmia	SNOMED	759671000000100	Direct current cardioversion planned

Arrhythmia	SNOMED	762441000000108	Referral for cardiac pacemaker check
Arrhythmia	SNOMED	77453006	Revision of pacemaker device
Arrhythmia	SNOMED	77867006	ECG: Q-T interval shortened
Arrhythmia	SNOMED	810911000000100	Deactivation of auto implantable cardioverter defibrillator
Arrhythmia	SNOMED	81184002	ECG: R wave
Arrhythmia	SNOMED	81220003	Artificial pacemaker rate check
Arrhythmia	SNOMED	83099006	Closed cardiac massage alone
Arrhythmia	SNOMED	845151000000104	Not aware of DNACPR (do not attempt cardiopulmonary resuscitation) clinical decision
Arrhythmia	SNOMED	86014007	Trifascicular block
Arrhythmia	SNOMED	868391000000107	Unsuccessful direct current cardioversion
Arrhythmia	SNOMED	88553002	Removal of cardioverter defibrillator
Arrhythmia	SNOMED	892501000000109	Evaluation of cardioverter defibrillator
Arrhythmia	SNOMED	89666000	Cardiopulmonary resuscitation
Arrhythmia	SNOMED	897851000000106	Implantation of subcutaneous cardioverter defibrillator
Arrhythmia	SNOMED	897871000000102	Resiting of lead of subcutaneous cardioverter defibrillator
Arrhythmia	SNOMED	897891000000103	Replacement of subcutaneous cardioverter defibrillator
Arrhythmia	SNOMED	897911000000100	Removal of subcutaneous cardioverter defibrillator
Arrhythmia	SNOMED	905431000006109	[RFC] Heart block
Arrhythmia	SNOMED	9061001	Check artificial pacemaker
Arrhythmia	SNOMED	931761000000106	Discussion about DNACPR (do not attempt cardiopulmonary resuscitation) not appropriate at this time
Arrhythmia	SNOMED	95281009	Sudden cardiac death, so described
Arrhythmia	SNOMED	9651007	Long Q-T syndrome
Arrhythmia	SNOMED	974221000006104	ECG - unacceptable
Arrhythmia	SNOMED	975291000000108	Family member informed of CPR (cardiopulmonary resuscitation) clinical decision
Arrhythmia	SNOMED	983221000006109	Asystolic vasovagal syncope
Arrhythmia	SNOMED	991002	ECG: QRS complex prolonged
Asthma	ICD10	J45	Asthma
Asthma	ICD10	J46	Status asthmaticus
Asthma	SNOMED	12428000	Intrinsic asthma without status asthmaticus
Asthma	SNOMED	160377001	FH: Asthma
Asthma	SNOMED	161527007	H/O: asthma
Asthma	SNOMED	162660004	Asthma resolved
Asthma	SNOMED	171231001	Asthma screening
Asthma	SNOMED	195967001	Airway hyperreactivity
Asthma	SNOMED	195977004	Mixed asthma
Asthma	SNOMED	225057002	Brittle asthma
Asthma	SNOMED	233678006	Childhood asthma
Asthma	SNOMED	233679003	Late onset asthma
Asthma	SNOMED	233681001	Extrinsic asthma with asthma attack
Asthma	SNOMED	233683003	Hay fever with asthma
Asthma	SNOMED	233685005	Intrinsic asthma with asthma attack
Asthma	SNOMED	266361008	Intrinsic asthma
Asthma	SNOMED	266364000	Asthma attack
Asthma	SNOMED	266899005	FH: Bronchitis/COAD
Asthma	SNOMED	275125002	FH: Bronchitis
Asthma	SNOMED	275126001	FH: COAD
Asthma	SNOMED	275528007	H/O: bronchitis
Asthma	SNOMED	281239006	Exacerbation of asthma
Asthma	SNOMED	304527002	Acute asthma
Asthma	SNOMED	31387002	Exercise-induced asthma
Asthma	SNOMED	32398004	Bronchitis
Asthma	SNOMED	389145006	Allergic asthma (disorder)
Asthma	SNOMED	394967008	Suspected asthma (context-dependent category)
Asthma	SNOMED	401135008	Health education - asthma (regime/therapy)
Asthma	SNOMED	401193004	Asthma confirmed (context-dependent category)
Asthma	SNOMED	407674008	Aspirin-induced asthma (disorder)
Asthma	SNOMED	424643009	IgE-mediated allergic asthma (disorder)
Asthma	SNOMED	57546000	Asthma with status asthmaticus
Asthma	SNOMED	57607007	Occupational asthma
Asthma	SNOMED	59327009	Intrinsic asthma with status asthmaticus
Asthma	SNOMED	63088003	Extrinsic asthma without status asthmaticus
Asthma	SNOMED	704008007	No family history of asthma
Asthma	SNOMED	708038006	Asthma attack
Asthma	SNOMED	708090002	Acute severe exacerbation of asthma (disorder)
Asthma	SNOMED	708093000	Acute exacerbation of extrinsic asthma
Asthma	SNOMED	708094006	Acute exacerbation of intrinsic asthma
Asthma	SNOMED	708095007	Acute severe exacerbation of extrinsic asthma (disorder)
Asthma	SNOMED	708096008	Acute severe exacerbation of intrinsic asthma (disorder)
Asthma	SNOMED	715191006	Telehealth asthma monitoring (regime/therapy)
Asthma	SNOMED	734346005	Asthma monitoring using asthma symptom diary (regime/therapy)
Asthma	SNOMED	734904007	Life threatening acute exacerbation of asthma (disorder)

Asthma	SNOMED	735587000	Acute severe exacerbation of asthma co-occurrent and due to allergic asthma (disorder)
Asthma	SNOMED	91340006	Extrinsic asthma with status asthmaticus
Autism	ICD10	F840	Childhood autism
Autism	ICD10	F841	Atypical autism
Autism	ICD10	F845	Asperger syndrome
Autism	SNOMED	191689008	Active infantile autism
Autism	SNOMED	191690004	Residual infantile autism
Autism	SNOMED	231536004	[X]Mental retardation with autistic features
Autism	SNOMED	23560001	[X]Asperger's syndrome
Autism	SNOMED	34883005	Autistic thinking
Autism	SNOMED	35919005	[X]Autistic spectrum disorder
Autism	SNOMED	373618009	Autistic spectrum disorder with isolated skills
Autism	SNOMED	408856003	Kanner's syndrome
Autism	SNOMED	408857007	Infantile autism
Autism	SNOMED	43614003	Childhood autism
Autism	SNOMED	702732007	High-functioning autism
Autism	SNOMED	702825006	Autism clinic
Autism	SNOMED	853581000006107	Autism
Bronchiectasis	ICD10	J470	Bronchiectasis
Bronchiectasis	ICD10	J471	Bronchiectasis
Bronchiectasis	ICD10	J472	Bronchiectasis
Bronchiectasis	ICD10	J473	Bronchiectasis
Bronchiectasis	ICD10	J474	Bronchiectasis
Bronchiectasis	ICD10	J475	Bronchiectasis
Bronchiectasis	ICD10	J476	Bronchiectasis
Bronchiectasis	ICD10	J477	Bronchiectasis
Bronchiectasis	ICD10	J478	Bronchiectasis
Bronchiectasis	ICD10	J479	Bronchiectasis
Bronchiectasis	SNOMED	12295008	Bronchiectasis
Bronchiectasis	SNOMED	195984007	Recurrent bronchiectasis
Bronchiectasis	SNOMED	195985008	Postinfectious bronchiectasis
Bronchiectasis	SNOMED	23022004	Tuberculous bronchiectasis
Bronchiectasis	SNOMED	939431000000108	H/O: bronchiectasis
Cancer	ICD10	C00	C00 Malignant neoplasm of lip
Cancer	ICD10	C01	C01 Malignant neoplasm of base of tongue
Cancer	ICD10	C02	C02 Malignant neoplasm of other and unspecified parts of tongue
Cancer	ICD10	C03	C03 Malignant neoplasm of gum
Cancer	ICD10	C04	C04 Malignant neoplasm of floor of mouth
Cancer	ICD10	C05	C05 Malignant neoplasm of palate
Cancer	ICD10	C06	C06 Malignant neoplasm of other and unspecified parts of mouth
Cancer	ICD10	C07	C07 Malignant neoplasm of parotid gland
Cancer	ICD10	C08	C08 Malignant neoplasm of other and unspecified major salivary glands
Cancer	ICD10	C09	C09 Malignant neoplasm of tonsil
Cancer	ICD10	C10	C10 Malignant neoplasm of oropharynx
Cancer	ICD10	C11	C11 Malignant neoplasm of nasopharynx
Cancer	ICD10	C12	C12 Malignant neoplasm of pyriform sinus
Cancer	ICD10	C13	C13 Malignant neoplasm of hypopharynx
Cancer	ICD10	C14	C14 Malignant neoplasm of other and ill-defined sites in the lip, oral cavity and pharynx
Cancer	ICD10	C15	C15 Malignant neoplasm of oesophagus
Cancer	ICD10	C16	C16 Malignant neoplasm of stomach
Cancer	ICD10	C17	C17 Malignant neoplasm of small intestine
Cancer	ICD10	C18	C18 Malignant neoplasm of colon
Cancer	ICD10	C19	C19 Malignant neoplasm of rectosigmoid junction
Cancer	ICD10	C20	C20 Malignant neoplasm of rectum
Cancer	ICD10	C21	C21 Malignant neoplasm of anus and anal canal
Cancer	ICD10	C22	C22 Malignant neoplasm of liver and intrahepatic bile ducts
Cancer	ICD10	C23	C23 Malignant neoplasm of gallbladder
Cancer	ICD10	C24	C24 Malignant neoplasm of other and unspecified parts of biliary tract
Cancer	ICD10	C25	C25 Malignant neoplasm of pancreas
Cancer	ICD10	C26	C26 Malignant neoplasm of other and ill-defined digestive organs
Cancer	ICD10	C30	C30 Malignant neoplasm of nasal cavity and middle ear
Cancer	ICD10	C31	C31 Malignant neoplasm of accessory sinuses
Cancer	ICD10	C32	C32 Malignant neoplasm of larynx
Cancer	ICD10	C33	C33 Malignant neoplasm of trachea
Cancer	ICD10	C34	C34 Malignant neoplasm of bronchus and lung
Cancer	ICD10	C37	C37 Malignant neoplasm of thymus
Cancer	ICD10	C38	C38 Malignant neoplasm of heart, mediastinum and pleura
Cancer	ICD10	C39	C39 Malignant neoplasm of other and ill-defined sites in the respiratory system and intrathoracic organs
Cancer	ICD10	C40	C40 Malignant neoplasm of bone and articular cartilage of limbs
Cancer	ICD10	C41	C41 Malignant neoplasm of bone and articular cartilage of other and unspecified sites
Cancer	ICD10	C42	C42 hematopoietic and reticuloendothelial systems (ICD-O-3 specific)
Cancer	ICD10	C43	C43 Malignant melanoma of skin

Cancer	ICD10	C44	C44 Other malignant neoplasms of skin
Cancer	ICD10	C45	C45 Mesothelioma
Cancer	ICD10	C46	C46 Kaposi's sarcoma
Cancer	ICD10	C47	C47 Malignant neoplasm of peripheral nerves and autonomic nervous system
Cancer	ICD10	C48	C48 Malignant neoplasm of retroperitoneum and peritoneum
Cancer	ICD10	C49	C49 Malignant neoplasm of other connective and soft tissue
Cancer	ICD10	C50	C50 Malignant neoplasm of breast
Cancer	ICD10	C51	C51 Malignant neoplasm of vulva
Cancer	ICD10	C52	C52 Malignant neoplasm of vagina
Cancer	ICD10	C53	C53 Malignant neoplasm of cervix uteri
Cancer	ICD10	C54	C54 Malignant neoplasm of corpus uteri
Cancer	ICD10	C55	C55 Malignant neoplasm of uterus, part unspecified
Cancer	ICD10	C56	C56 Malignant neoplasm of ovary
Cancer	ICD10	C57	C57 Malignant neoplasm of other and unspecified female genital organs
Cancer	ICD10	C58	C58 Malignant neoplasm of placenta
Cancer	ICD10	C60	C60 Malignant neoplasm of penis
Cancer	ICD10	C61	C61 Malignant neoplasm of prostate
Cancer	ICD10	C62	C62 Malignant neoplasm of testis
Cancer	ICD10	C63	C63 Malignant neoplasm of other and unspecified male genital organs
Cancer	ICD10	C64	C64 Malignant neoplasm of kidney, except renal pelvis
Cancer	ICD10	C65	C65 Malignant neoplasm of renal pelvis
Cancer	ICD10	C66	C66 Malignant neoplasm of ureter
Cancer	ICD10	C67	C67 Malignant neoplasm of bladder
Cancer	ICD10	C68	C68 Malignant neoplasm of other and unspecified urinary organs
Cancer	ICD10	C69	C69 Malignant neoplasm of eye and adnexa
Cancer	ICD10	C70	C70 Malignant neoplasm of meninges
Cancer	ICD10	C71	C71 Malignant neoplasm of brain
Cancer	ICD10	C72	C72 Malignant neoplasm of spinal cord, cranial nerves and other parts of central nervous system
Cancer	ICD10	C73	C73 Malignant neoplasm of thyroid gland
Cancer	ICD10	C74	C74 Malignant neoplasm of adrenal gland
Cancer	ICD10	C75	C75 Malignant neoplasm of other endocrine glands and related structures
Cancer	SNOMED	105121000119102	Squamous cell carcinoma of vagina (disorder)
Cancer	SNOMED	107691000119101	Non-seminomatous germ cell neoplasm of testis (disorder)
Cancer	SNOMED	107771000119106	Primary malignant clear cell neoplasm of endometrium (disorder)
Cancer	SNOMED	1078881000119102	Primary adenocarcinoma of lower lobe of left lung (disorder)
Cancer	SNOMED	1078901000119100	Primary adenocarcinoma of upper lobe of left lung (disorder)
Cancer	SNOMED	1078961000119104	Primary adenocarcinoma of upper lobe of right lung (disorder)
Cancer	SNOMED	1080941000119109	Malignant melanoma of left choroid (disorder)
Cancer	SNOMED	1081551000119106	Recurrent primary malignant neoplasm of left female breast (disorder)
Cancer	SNOMED	1081561000119108	Recurrent primary malignant neoplasm of right female breast (disorder)
Cancer	SNOMED	1081781000119105	Primary seminoma of left testis (disorder)
Cancer	SNOMED	1082301000119109	Transitional cell carcinoma of right renal pelvis (disorder)
Cancer	SNOMED	1082311000119107	Transitional cell carcinoma of right ureter (disorder)
Cancer	SNOMED	1090081000000106	Secondary malignant neoplasm of paratracheal lymph nodes (disorder)
Cancer	SNOMED	1090091000000108	Secondary malignant neoplasm of internal mammary lymph nodes (disorder)
Cancer	SNOMED	1090101000000100	Secondary malignant neoplasm of supratrochlear lymph nodes (disorder)
Cancer	SNOMED	1090121000000109	Secondary malignant neoplasm of deep cervical lymph nodes (disorder)
Cancer	SNOMED	1090131000000106	Secondary malignant neoplasm of anterior cervical lymph nodes (disorder)
Cancer	SNOMED	1090141000000102	Secondary malignant neoplasm of superficial cervical lymph nodes (disorder)
Cancer	SNOMED	1090151000000104	Secondary malignant neoplasm of mastoid lymph nodes (disorder)
Cancer	SNOMED	1090161000000101	Secondary malignant neoplasm of diaphragmatic lymph nodes (disorder)
Cancer	SNOMED	1090171000000108	Secondary malignant neoplasm of sacral lymph nodes (disorder)
Cancer	SNOMED	1090181000000105	Secondary malignant neoplasm of inferior epigastric lymph nodes (disorder)
Cancer	SNOMED	1090191000000107	Secondary malignant neoplasm of coeliac lymph nodes (disorder)
Cancer	SNOMED	1090201000000109	Secondary malignant neoplasm of deep inguinal lymph nodes (disorder)
Cancer	SNOMED	1090221000000100	Secondary malignant neoplasm of internal iliac lymph nodes (disorder)
Cancer	SNOMED	1090241000000107	Angioimmunoblastic T-cell lymphoma with dysproteinaemia (disorder)
Cancer	SNOMED	1090251000000105	Malignant neoplasm of nose (disorder)
Cancer	SNOMED	1090261000000108	Malignant neoplasm of descended testis (disorder)
Cancer	SNOMED	1090271000000101	Malignant neoplasm of ventral surface of tongue (disorder)
Cancer	SNOMED	1090281000000104	Malignant neoplasm of genital labia (disorder)
Cancer	SNOMED	1090291000000102	Malignant neoplasm of prepylorus of stomach (disorder)
Cancer	SNOMED	1090301000000103	Malignant neoplasm of flank (disorder)
Cancer	SNOMED	1090821000000101	Malignant neoplasm of bone and articular cartilage (disorder)
Cancer	SNOMED	1090871000000102	Malignant neoplasm of bone and articular cartilage of limb (disorder)
Cancer	SNOMED	1090881000000100	Malignant neoplasm of bronchus or lung (disorder)
Cancer	SNOMED	1090891000000103	Malignant neoplasm of connective and soft tissues of trunk (disorder)
Cancer	SNOMED	1090901000000102	Malignant neoplasm of peripheral nerves of trunk (disorder)
Cancer	SNOMED	1090921000000106	Malignant neoplasm of jaw (disorder)
Cancer	SNOMED	1090931000000108	Malignant neoplasm of multiple endocrine glands (disorder)
Cancer	SNOMED	1090941000000104	Malignant neoplasm of inguinal region (disorder)
Cancer	SNOMED	1090951000000101	Malignant neoplasm of supraclavicular fossa (disorder)
Cancer	SNOMED	1090961000000103	Malignant neoplasm of soft tissues of lower limb (disorder)

Cancer	SNOMED	1090971000000105	Malignant neoplasm of posterior wall of stomach (disorder)
Cancer	SNOMED	1090981000000107	Malignant neoplasm of anterior wall of stomach (disorder)
Cancer	SNOMED	1091861000000100	Follicular lymphoma, cutaneous follicle centre (disorder)
Cancer	SNOMED	1091921000000103	B-cell non-Hodgkin's lymphoma (disorder)
Cancer	SNOMED	109264009	Overlapping malignant neoplasm of skin
Cancer	SNOMED	109267002	Overlapping malignant melanoma of skin
Cancer	SNOMED	1092831000000100	Overlapping malignant neoplasm of mouth (disorder)
Cancer	SNOMED	109347009	Overlapping malignant neoplasm of bone and articular cartilage
Cancer	SNOMED	109348004	Overlapping malignant neoplasm of bone and articular cartilage of limbs (disorder)
Cancer	SNOMED	109358000	Overlapping malignant neoplasm of ill-defined site
Cancer	SNOMED	109367000	Overlapping malignant neoplasm of nasopharynx
Cancer	SNOMED	109368005	Overlapping malignant neoplasm of hypopharynx (disorder)
Cancer	SNOMED	109369002	Overlapping malignant neoplasm of larynx
Cancer	SNOMED	109371002	Overlapping malignant neoplasm of bronchus and lung
Cancer	SNOMED	109378008	Mesothelioma (malignant)(clinical)
Cancer	SNOMED	109383000	Mesothelioma of pericardium
Cancer	SNOMED	109384006	Overlapping malignant neoplasm of heart, mediastinum and pleura
Cancer	SNOMED	109385007	Kaposi's sarcoma (clinical)
Cancer	SNOMED	109386008	Kaposi's sarcoma of skin (disorder)
Cancer	SNOMED	109388009	Kaposi's sarcoma of palate
Cancer	SNOMED	109391009	Kaposi's sarcoma of lymph nodes
Cancer	SNOMED	109392002	Kaposi's sarcoma of multiple organs
Cancer	SNOMED	109823006	Overlapping malignant neoplasm of tongue
Cancer	SNOMED	109824000	Overlapping malignant neoplasm of major salivary gland (disorder)
Cancer	SNOMED	109830000	Overlapping malignant neoplasm of floor of mouth
Cancer	SNOMED	109835005	Overlapping malignant neoplasm of esophagus
Cancer	SNOMED	109836006	Overlapping malignant neoplasm of stomach
Cancer	SNOMED	109837002	Overlapping malignant neoplasm of small intestine
Cancer	SNOMED	109841003	Liver cell carcinoma (clinical)
Cancer	SNOMED	109842005	Intrahepatic bile duct carcinoma (disorder)
Cancer	SNOMED	109843000	Hepatoblastoma (clinical)
Cancer	SNOMED	109844006	Angiosarcoma of liver
Cancer	SNOMED	109847004	Overlapping malignant neoplasm of biliary tract
Cancer	SNOMED	109848009	Overlapping malignant neoplasm of pancreas
Cancer	SNOMED	109853004	Mesothelioma of peritoneum
Cancer	SNOMED	109874003	Overlapping malignant neoplasm of male genital organs
Cancer	SNOMED	109875002	Overlapping malignant neoplasm of penis
Cancer	SNOMED	109876001	Malignant neoplasm of descended testis
Cancer	SNOMED	109878000	Overlapping malignant neoplasm of female genital organs
Cancer	SNOMED	109885001	Overlapping malignant neoplasm of vulva
Cancer	SNOMED	1098961000119105	History of cancer metastatic to lung
Cancer	SNOMED	1098981000119101	Recurrent malignant neoplasm of prostate (disorder)
Cancer	SNOMED	109911004	Overlapping malignant neoplasm of brain and other parts of the central nervous system
Cancer	SNOMED	109912006	Overlapping malignant neoplasm of brain
Cancer	SNOMED	109919002	Overlapping malignant neoplasm of peripheral nerves and autonomic nervous system
Cancer	SNOMED	109948008	Overlapping malignant neoplasm of eye and adnexa (primary)
Cancer	SNOMED	109962001	Diffuse non-Hodgkin's lymphoma
Cancer	SNOMED	109965004	Diffuse non-Hodgkin's lymphoma, lymphoblastic (clinical)
Cancer	SNOMED	109966003	Diffuse non-Hodgkin's lymphoma, immunoblastic (clinical)
Cancer	SNOMED	109969005	Diffuse non-Hodgkin's lymphoma, large cell (clinical)
Cancer	SNOMED	109975001	T-zone lymphoma (clinical)
Cancer	SNOMED	109976000	Lymphoepithelioid lymphoma (clinical)
Cancer	SNOMED	109977009	Peripheral T-cell lymphoma (clinical)
Cancer	SNOMED	109978004	T-cell lymphoma (clinical)
Cancer	SNOMED	109979007	B-cell lymphoma (clinical)
Cancer	SNOMED	109980005	Malignant immunoproliferative disease (clinical)
Cancer	SNOMED	109982002	Alpha heavy chain disease (disorder)
Cancer	SNOMED	109984001	Gamma heavy chain disease (disorder)
Cancer	SNOMED	109988003	True histiocytic lymphoma (clinical)
Cancer	SNOMED	109989006	Multiple myeloma (clinical)
Cancer	SNOMED	109991003	Acute myelofibrosis (clinical)
Cancer	SNOMED	109992005	Vaquez's disease
Cancer	SNOMED	109994006	Essential thrombocythemia (disorder)
Cancer	SNOMED	109996008	Myelodysplastic syndrome: Refractory anemia, without ringed sideroblasts, without excess blasts (disorder)
Cancer	SNOMED	109998009	Refractory anemia with ringed sideroblasts (disorder)
Cancer	SNOMED	110000005	Refractory anemia with excess blasts in transformation (disorder)
Cancer	SNOMED	110002002	Mast cell leukemia (clinical)
Cancer	SNOMED	110004001	Acute promyelocytic leukaemia (clinical)
Cancer	SNOMED	110005000	Acute myelomonocytic leukaemia (clinical)
Cancer	SNOMED	110006004	Prolymphocytic leukemia (clinical)
Cancer	SNOMED	110007008	Adult T-cell leukemia/lymphoma (disorder)

Cancer	SNOMED	110013004	Overlapping malignant neoplasm of tonsil
Cancer	SNOMED	11010461000119101	Small cell carcinoma (disorder)
Cancer	SNOMED	1110871000000109	Quality and Outcomes Framework cancer quality indicator-related care invitation (procedure)
Cancer	SNOMED	116381000119105	Ganglioneuroblastoma (disorder)
Cancer	SNOMED	118599009	Hodgkin's disease (clinical)
Cancer	SNOMED	118600007	Lymphoma (clinical)
Cancer	SNOMED	118601006	Non-Hodgkin's lymphoma (clinical)
Cancer	SNOMED	118602004	Hodgkin's granuloma (clinical)
Cancer	SNOMED	118605002	Hodgkin's paragranuloma (clinical)
Cancer	SNOMED	118606001	Hodgkin's sarcoma (clinical)
Cancer	SNOMED	118607005	Hodgkin's disease, lymphocytic-histiocytic predominance (clinical)
Cancer	SNOMED	118608000	Hodgkin's disease, nodular sclerosis (clinical)
Cancer	SNOMED	118609008	Hodgkin's disease, mixed cellularity (clinical)
Cancer	SNOMED	118610003	Hodgkin's disease, lymphocytic depletion (clinical)
Cancer	SNOMED	118611004	Sézary's disease (clinical)
Cancer	SNOMED	118612006	Malignant histiocytosis (clinical)
Cancer	SNOMED	118613001	Leukemic reticuloendotheliosis (clinical) [obs]
Cancer	SNOMED	118614007	Letterer-Siwe disease (clinical)
Cancer	SNOMED	118615008	Malignant mast cell tumor (clinical)
Cancer	SNOMED	118617000	Burkitt's lymphoma (clinical)
Cancer	SNOMED	118618005	Mycosis fungoides (clinical)
Cancer	SNOMED	12240951000119107	Squamous cell carcinoma of left lung (disorder)
Cancer	SNOMED	12240991000119102	Squamous cell carcinoma of right lung (disorder)
Cancer	SNOMED	123845008	Adenocarcinoma of endometrium (disorder)
Cancer	SNOMED	127225006	Chronic myelomonocytic leukemia
Cancer	SNOMED	127230005	Neoplasm of spleen (disorder)
Cancer	SNOMED	128041000119107	Primary adenocarcinoma of distal third of esophagus (disorder)
Cancer	SNOMED	128462008	Metastatic neoplasm (disease)
Cancer	SNOMED	128875000	Primary cutaneous CD30 antigen positive large T-cell lymphoma (disorder)
Cancer	SNOMED	129000002	Langerhans cell histiocytosis, unifocal (disorder)
Cancer	SNOMED	134421000	Pathological fracture due to metastatic bone disease
Cancer	SNOMED	15635721000119108	Primary malignant neoplasm of both ovaries (disorder)
Cancer	SNOMED	15956341000119105	Adenocarcinoma of left lung (disorder)
Cancer	SNOMED	15956381000119100	Adenocarcinoma of right lung (disorder)
Cancer	SNOMED	161436008	H/O: * leukemia
Cancer	SNOMED	16260631000119101	Secondary malignant neoplasm of lymph node from neoplasm of female breast (disorder)
Cancer	SNOMED	16341002	Parapsoriasis lichenoides (disorder)
Cancer	SNOMED	1691000119104	Metastasis to liver from adenocarcinoma (disorder)
Cancer	SNOMED	169396008	Radiomimetic chemotherapy (procedure)
Cancer	SNOMED	169397004	Radiochemotherapy: local infiltration (procedure)
Cancer	SNOMED	169398009	Radiochemotherapy: to stop spread (procedure)
Cancer	SNOMED	169399001	Radiochemotherapy: oral route (procedure)
Cancer	SNOMED	169400008	Radiochemotherapy via intravenous route (procedure)
Cancer	SNOMED	169401007	Radiochemotherapy: for immunosuppression (procedure)
Cancer	SNOMED	169402000	Radiochemotherapy: local artery (procedure)
Cancer	SNOMED	169403005	Radiochemotherapy: into cavity (procedure)
Cancer	SNOMED	1701000119104	Primary adenocarcinoma of colon (disorder)
Cancer	SNOMED	18121000119104	Primary squamous cell carcinoma of palatine tonsil (disorder)
Cancer	SNOMED	184881000119106	Primary adenocarcinoma of rectosigmoid junction (disorder)
Cancer	SNOMED	187601000	Malignant neoplasm of upper lip, lipstick area
Cancer	SNOMED	187604008	Malignant neoplasm of lower lip, external
Cancer	SNOMED	187606005	Malignant tumour of oral aspect of upper lip
Cancer	SNOMED	187613005	Malignant neoplasm of lower lip, buccal aspect
Cancer	SNOMED	187614004	Malignant tumor of frenum of lower lip
Cancer	SNOMED	187622006	Malignant tumor of labial mucosa
Cancer	SNOMED	187624007	Malignant neoplasm of overlapping lesion of lip
Cancer	SNOMED	187631006	Malignant neoplasm of base of tongue dorsal surface
Cancer	SNOMED	187633009	Malignant neoplasm of dorsal surface of tongue
Cancer	SNOMED	187634003	Malignant tumor of anterior two-thirds of tongue - dorsal surface (disorder)
Cancer	SNOMED	187635002	Malignant neoplasm of midline of tongue (disorder)
Cancer	SNOMED	187637005	Malignant neoplasm of tongue, tip and lateral border
Cancer	SNOMED	187640005	Malignant neoplasm of anterior 2/3 of tongue ventral surface
Cancer	SNOMED	187641009	Malignant tumor of frenum linguae
Cancer	SNOMED	187644001	Malignant tumour of junctional zone of tongue
Cancer	SNOMED	187652003	Malignant tumour of anterior floor of mouth
Cancer	SNOMED	187653008	Malignant neoplasm of lateral portion of floor of mouth
Cancer	SNOMED	187658004	Malignant tumour of vestibule of mouth
Cancer	SNOMED	187659007	Malignant neoplasm of upper buccal sulcus
Cancer	SNOMED	187660002	Malignant tumor of lower buccal sulcus
Cancer	SNOMED	187662005	Malignant tumor of lower labial sulcus (disorder)
Cancer	SNOMED	187666008	Malignant neoplasm of junction of hard and soft palate
Cancer	SNOMED	187675005	Malignant tumour of palatopharyngeal arch
Cancer	SNOMED	187681002	Malignant neoplasm of anterior epiglottis

Cancer	SNOMED	187682009	Malignant neoplasm of epiglottis, free border
Cancer	SNOMED	187683004	Malignant neoplasm of glossoepiglottic fold
Cancer	SNOMED	187685006	Malignant neoplasm of junctional region of epiglottis
Cancer	SNOMED	187688008	Malignant tumour of posterior wall of oropharynx
Cancer	SNOMED	187692001	Malignant tumor of epipharynx
Cancer	SNOMED	187693006	Malignant tumour of posterior wall of nasopharynx
Cancer	SNOMED	187694000	Malignant tumor of adenoid
Cancer	SNOMED	187697007	Malignant tumour of fossa of Rosenmuller
Cancer	SNOMED	187698002	Malignant tumor of opening of auditory tube (disorder)
Cancer	SNOMED	187700006	Malignant tumor of anterior wall of nasopharynx
Cancer	SNOMED	187701005	Malignant neoplasm of floor of nasopharynx (disorder)
Cancer	SNOMED	187702003	Malignant tumor of nasopharyngeal soft palate surface (disorder)
Cancer	SNOMED	187708004	Malignant tumor aryepiglottic fold - hypopharyngeal aspect
Cancer	SNOMED	187709007	Malignant neoplasm of posterior pharynx
Cancer	SNOMED	187716008	Malignant tumour of Waldeyer's ring
Cancer	SNOMED	187722004	Malignant tumour of cervical part of oesophagus
Cancer	SNOMED	187723009	Malignant neoplasm of thoracic esophagus
Cancer	SNOMED	187724003	Malignant tumour of abdominal part of oesophagus
Cancer	SNOMED	187725002	Malignant neoplasm of upper third of esophagus
Cancer	SNOMED	187726001	Malignant tumor of middle third of esophagus
Cancer	SNOMED	187727005	Malignant neoplasm of lower third of oesophagus
Cancer	SNOMED	187732006	Malignant neoplasm of cardia of stomach
Cancer	SNOMED	187733001	Malignant neoplasm of cardiac orifice of stomach
Cancer	SNOMED	187734007	Malignant neoplasm of cardio-esophageal junction of stomach
Cancer	SNOMED	187736009	Malignant tumor of pylorus
Cancer	SNOMED	187738005	Malignant neoplasm of pyloric canal of stomach
Cancer	SNOMED	187740000	Malignant tumor of pyloric antrum
Cancer	SNOMED	187741001	Malignant tumour of fundus of stomach
Cancer	SNOMED	187742008	Malignant tumor of body of stomach
Cancer	SNOMED	187752007	Malignant tumor of Meckel's diverticulum
Cancer	SNOMED	187757001	Malignant neoplasm, overlapping lesion of colon
Cancer	SNOMED	187760008	Malignant neoplasm of rectum, rectosigmoid junction and anus
Cancer	SNOMED	187767006	Malignant neoplasm of liver and intrahepatic bile ducts
Cancer	SNOMED	187769009	Primary carcinoma of liver
Cancer	SNOMED	187773007	Malignant neoplasm of interlobular bile ducts
Cancer	SNOMED	187776004	Malignant neoplasm of intrahepatic canaliculi (disorder)
Cancer	SNOMED	187777008	Malignant neoplasm of intrahepatic gall duct
Cancer	SNOMED	187784000	Malignant neoplasm of hepatic duct
Cancer	SNOMED	187786003	Malignant neoplasm of sphincter of Oddi (disorder)
Cancer	SNOMED	187791002	Malignant tumor of body of pancreas
Cancer	SNOMED	187792009	Malignant tumour of tail of pancreas
Cancer	SNOMED	187793004	Malignant tumor of pancreatic duct
Cancer	SNOMED	187794005	Malignant tumour of Islets of Langerhans
Cancer	SNOMED	187798008	Malignant neoplasm of ectopic pancreatic tissue
Cancer	SNOMED	187801002	Malignant tumour of peritoneum and retroperitoneum
Cancer	SNOMED	187803004	Malignant neoplasm of perinephric tissue
Cancer	SNOMED	187804005	Malignant neoplasm of retrocaecal tissue
Cancer	SNOMED	187807003	Overlapping malignant lesion of retroperitoneum and peritoneum
Cancer	SNOMED	187808008	Malignant neoplasm of specified parts of peritoneum
Cancer	SNOMED	187809000	Malignant neoplasm of mesocolon (disorder)
Cancer	SNOMED	187811009	Malignant neoplasm of mesorectum
Cancer	SNOMED	187814001	Malignant neoplasm of the pouch of Douglas
Cancer	SNOMED	187821001	Angiosarcoma of spleen (disorder)
Cancer	SNOMED	187822008	Fibrosarcoma of spleen
Cancer	SNOMED	187824009	Malignant neoplasm, overlapping lesion of digestive system
Cancer	SNOMED	187828007	Malignant neoplasm of nasal cavities, middle ear and accessory sinuses
Cancer	SNOMED	187829004	Malignant neoplasm of cartilage of nose
Cancer	SNOMED	187830009	Malignant neoplasm of nasal conchae
Cancer	SNOMED	187831008	Malignant tumour of nasal vestibule
Cancer	SNOMED	187833006	Malignant neoplasm of auditory tube, middle ear and mastoid air cells
Cancer	SNOMED	187834000	Malignant tumor of Eustachian tube (disorder)
Cancer	SNOMED	187835004	Malignant tumour of tympanic cavity
Cancer	SNOMED	187836003	Malignant tumor of tympanic antrum
Cancer	SNOMED	187838002	Malignant neoplasm, overlapping lesion of accessory sinuses
Cancer	SNOMED	187841006	Malignant tumor of glottis
Cancer	SNOMED	187842004	Malignant tumour of supraglottis
Cancer	SNOMED	187843009	Malignant neoplasm of arytenoid cartilage
Cancer	SNOMED	187844003	Malignant neoplasm of cricoid cartilage
Cancer	SNOMED	187845002	Malignant neoplasm of cuneiform cartilage (disorder)
Cancer	SNOMED	187846001	Malignant neoplasm of thyroid cartilage
Cancer	SNOMED	187853005	Malignant neoplasm of cartilage of trachea (disorder)
Cancer	SNOMED	187854004	Malignant neoplasm of mucosa of trachea
Cancer	SNOMED	187857006	Malignant neoplasm of carina of bronchus
Cancer	SNOMED	187861000	Malignant neoplasm of upper lobe bronchus
Cancer	SNOMED	187862007	Malignant neoplasm of upper lobe of lung

Cancer	SNOMED	187864008	Malignant neoplasm of middle lobe, bronchus or lung
Cancer	SNOMED	187865009	Malignant neoplasm of middle lobe bronchus
Cancer	SNOMED	187866005	Malignant neoplasm of middle lobe of lung
Cancer	SNOMED	187868006	Malignant neoplasm of lower lobe, bronchus or lung
Cancer	SNOMED	187869003	Malignant neoplasm of lower lobe bronchus
Cancer	SNOMED	187870002	Malignant neoplasm of lower lobe of lung
Cancer	SNOMED	187881004	Malignant neoplasm of thymus, heart and mediastinum
Cancer	SNOMED	187900002	Malignant neoplasm of bones of skull and face
Cancer	SNOMED	187903000	Malignant neoplasm of malar bone
Cancer	SNOMED	187906008	Malignant neoplasm of orbital bone
Cancer	SNOMED	187916000	Malignant neoplasm of cervical vertebra
Cancer	SNOMED	187917009	Malignant neoplasm of thoracic vertebra
Cancer	SNOMED	187918004	Malignant neoplasm of lumbar vertebra
Cancer	SNOMED	187920001	Malignant neoplasm of ribs, sternum and clavicle
Cancer	SNOMED	187925006	Malignant neoplasm of costal cartilage
Cancer	SNOMED	187926007	Malignant neoplasm of costovertebral joint
Cancer	SNOMED	187927003	Malignant neoplasm of xiphoid process
Cancer	SNOMED	187929000	Malignant neoplasm of scapula and long bones of upper arm
Cancer	SNOMED	187932002	Malignant neoplasm of humerus
Cancer	SNOMED	187937008	Malignant neoplasm of carpal bone - scaphoid
Cancer	SNOMED	187938003	Malignant neoplasm of carpal bone - lunate
Cancer	SNOMED	187940008	Malignant neoplasm of carpal bone - pisiform (disorder)
Cancer	SNOMED	187941007	Malignant neoplasm of carpal bone - trapezium (disorder)
Cancer	SNOMED	187944004	Malignant neoplasm of carpal bone - hamate (disorder)
Cancer	SNOMED	187945003	Malignant neoplasm of first metacarpal bone (disorder)
Cancer	SNOMED	187946002	Malignant neoplasm of second metacarpal bone (disorder)
Cancer	SNOMED	187947006	Malignant neoplasm of third metacarpal bone (disorder)
Cancer	SNOMED	187948001	Malignant neoplasm of fourth metacarpal bone (disorder)
Cancer	SNOMED	187949009	Malignant neoplasm of fifth metacarpal bone
Cancer	SNOMED	187950009	Malignant neoplasm of phalanges of hand
Cancer	SNOMED	187952001	Malignant neoplasm of pelvic bones, sacrum and coccyx
Cancer	SNOMED	187956003	Malignant neoplasm of sacral vertebra
Cancer	SNOMED	187957007	Malignant neoplasm of coccygeal vertebra
Cancer	SNOMED	187967002	Malignant neoplasm of calcaneum
Cancer	SNOMED	187968007	Malignant neoplasm of medial cuneiform
Cancer	SNOMED	187973001	Malignant neoplasm of first metatarsal bone
Cancer	SNOMED	187975008	Malignant neoplasm of third metatarsal bone (disorder)
Cancer	SNOMED	187976009	Malignant neoplasm of fourth metatarsal bone
Cancer	SNOMED	187977000	Malignant neoplasm of fifth metatarsal bone (disorder)
Cancer	SNOMED	187978005	Malignant neoplasm of phalanges of foot
Cancer	SNOMED	187987001	Malignant neoplasm of cartilage of ear
Cancer	SNOMED	187988006	Malignant neoplasm of tarsus of eyelid
Cancer	SNOMED	187989003	Malignant neoplasm soft tissues of cervical spine (disorder)
Cancer	SNOMED	187991006	Malignant neoplasm of connective and soft tissue of upper limb and shoulder
Cancer	SNOMED	187992004	Malignant neoplasm of connective and soft tissue of shoulder
Cancer	SNOMED	187993009	Malignant neoplasm of connective and soft tissue, upper arm
Cancer	SNOMED	187994003	Malignant neoplasm of connective and soft tissue of fore-arm
Cancer	SNOMED	187995002	Malignant neoplasm of connective and soft tissue of hand
Cancer	SNOMED	187996001	Malignant neoplasm of connective and soft tissue of finger
Cancer	SNOMED	187997005	Malignant neoplasm of connective and soft tissue of thumb
Cancer	SNOMED	187999008	Malignant neoplasm of connective and soft tissue of hip and lower limb
Cancer	SNOMED	188000002	Malignant neoplasm of connective and soft tissue of hip
Cancer	SNOMED	188001003	Malignant neoplasm of connective and soft tissue of thigh and upper leg
Cancer	SNOMED	188002005	Malignant neoplasm of connective and soft tissue of popliteal space
Cancer	SNOMED	188003000	Malignant neoplasm of connective and soft tissue of lower leg
Cancer	SNOMED	188004006	Malignant neoplasm of connective and soft tissue of foot
Cancer	SNOMED	188005007	Malignant neoplasm of connective and soft tissue of toe
Cancer	SNOMED	188006008	Malignant neoplasm of connective and soft tissue of great toe (disorder)
Cancer	SNOMED	188009001	Malignant neoplasm of connective and soft tissue of thorax
Cancer	SNOMED	188010006	Malignant neoplasm of connective and soft tissue of axilla
Cancer	SNOMED	188013008	Malignant neoplasm of connective and soft tissues of thoracic spine
Cancer	SNOMED	188015001	Malignant neoplasm of connective and soft tissue of abdomen
Cancer	SNOMED	188016000	Malignant neoplasm of connective and soft tissue of abdominal wall
Cancer	SNOMED	188017009	Malignant neoplasm of connective and soft tissues of lumbar spine
Cancer	SNOMED	188019007	Malignant neoplasm of connective and soft tissue of pelvis
Cancer	SNOMED	188020001	Malignant neoplasm of connective and soft tissue of buttock
Cancer	SNOMED	188021002	Malignant neoplasm of connective and soft tissue of inguinal region
Cancer	SNOMED	188022009	Malignant neoplasm of connective and soft tissue of perineum
Cancer	SNOMED	188023004	Malignant neoplasm of connective and soft tissue of sacrum or coccyx (disorder)
Cancer	SNOMED	188029000	Kaposi's sarcoma of soft tissue
Cancer	SNOMED	188030005	Malignant melanoma of lip
Cancer	SNOMED	188032002	Malignant melanoma of ear and external auricular canal
Cancer	SNOMED	188033007	Malignant melanoma of auricle (ear)

Cancer	SNOMED	188034001	Malignant melanoma of external auditory meatus
Cancer	SNOMED	188038003	Malignant melanoma of chin
Cancer	SNOMED	188040008	Malignant melanoma of forehead
Cancer	SNOMED	188042000	Malignant melanoma of temple
Cancer	SNOMED	188044004	Malignant melanoma of scalp and neck
Cancer	SNOMED	188045003	Malignant melanoma of scalp
Cancer	SNOMED	188046002	Malignant melanoma of neck
Cancer	SNOMED	188049009	Malignant melanoma of axilla
Cancer	SNOMED	188050009	Malignant melanoma of breast
Cancer	SNOMED	188051008	Malignant melanoma of buttock
Cancer	SNOMED	188052001	Malignant melanoma of groin
Cancer	SNOMED	188054000	Malignant melanoma of perineum
Cancer	SNOMED	188055004	Malignant melanoma of umbilicus
Cancer	SNOMED	188060000	Malignant melanoma of shoulder
Cancer	SNOMED	188061001	Malignant melanoma of upper arm
Cancer	SNOMED	188062008	Malignant melanoma of fore-arm
Cancer	SNOMED	188063003	Malignant melanoma of hand
Cancer	SNOMED	188064009	Malignant melanoma of finger
Cancer	SNOMED	188065005	Malignant melanoma of thumb
Cancer	SNOMED	188067002	Malignant melanoma of lower limb and hip
Cancer	SNOMED	188068007	Malignant melanoma of hip
Cancer	SNOMED	188069004	Malignant melanoma of thigh
Cancer	SNOMED	188070003	Malignant melanoma of knee
Cancer	SNOMED	188071004	Malignant melanoma of popliteal fossa area
Cancer	SNOMED	188072006	Malignant melanoma of lower leg
Cancer	SNOMED	188073001	Malignant melanoma of ankle
Cancer	SNOMED	188074007	Malignant melanoma of heel
Cancer	SNOMED	188075008	Malignant melanoma of foot
Cancer	SNOMED	188076009	Malignant melanoma of toe
Cancer	SNOMED	188077000	Malignant melanoma of great toe
Cancer	SNOMED	188102008	Malignant neoplasm of scalp AND/OR skin of neck (disorder)
Cancer	SNOMED	188147009	Malignant neoplasm of nipple and areola of female breast
Cancer	SNOMED	188151006	Malignant neoplasm of central part of female breast
Cancer	SNOMED	188152004	Malignant neoplasm of upper-inner quadrant of female breast
Cancer	SNOMED	188153009	Malignant neoplasm of lower-inner quadrant of female breast
Cancer	SNOMED	188154003	Malignant neoplasm of upper-outer quadrant of female breast
Cancer	SNOMED	188155002	Malignant neoplasm of lower-outer quadrant of female breast
Cancer	SNOMED	188156001	Malignant neoplasm of axillary tail of female breast
Cancer	SNOMED	188157005	Malignant neoplasm, overlapping lesion of breast
Cancer	SNOMED	188159008	Malignant neoplasm of ectopic site of female breast
Cancer	SNOMED	188163001	Malignant neoplasm of nipple and areola of male breast
Cancer	SNOMED	188168005	Malignant neoplasm of ectopic site of male breast
Cancer	SNOMED	188176007	Malignant neoplasm of endocervical canal
Cancer	SNOMED	188177003	Malignant neoplasm of endocervical gland
Cancer	SNOMED	188180002	Malignant neoplasm, overlapping lesion of cervix uteri
Cancer	SNOMED	188183000	Malignant neoplasm of cervical stump
Cancer	SNOMED	188184006	Malignant neoplasm of squamocolumnar junction of cervix
Cancer	SNOMED	188188009	Choriocarcinoma
Cancer	SNOMED	188189001	Malignant neoplasm of corpus uteri, excluding isthmus
Cancer	SNOMED	188190005	Malignant neoplasm of cornu of corpus uteri
Cancer	SNOMED	188191009	Malignant neoplasm of fundus of corpus uteri
Cancer	SNOMED	188192002	Malignant neoplasm of endometrium of corpus uteri
Cancer	SNOMED	188193007	Malignant neoplasm of myometrium of corpus uteri
Cancer	SNOMED	188195000	Malignant neoplasm of isthmus of uterine body
Cancer	SNOMED	188198003	Malignant neoplasm of overlapping lesion of corpus uteri
Cancer	SNOMED	188204000	Malignant neoplasm of round ligament (disorder)
Cancer	SNOMED	188208002	Malignant neoplasm of Gartner's duct (disorder)
Cancer	SNOMED	188209005	Malignant neoplasm of vaginal vault
Cancer	SNOMED	188211001	Malignant neoplasm of greater vestibular (Bartholin's) gland
Cancer	SNOMED	188219004	Malignant tumour of undescended testis
Cancer	SNOMED	188220005	Malignant tumor of ectopic testis (disorder)
Cancer	SNOMED	188230001	Malignant tumour of shaft of penis
Cancer	SNOMED	188234005	Malignant neoplasm of seminal vesicle
Cancer	SNOMED	188235006	Malignant tumor of tunica vaginalis
Cancer	SNOMED	188239000	Malignant tumor of trigone of urinary bladder
Cancer	SNOMED	188240003	Malignant tumour of vault of bladder
Cancer	SNOMED	188241004	Malignant neoplasm of lateral wall of urinary bladder
Cancer	SNOMED	188242006	Malignant neoplasm of anterior wall of urinary bladder
Cancer	SNOMED	188243001	Malignant neoplasm of posterior wall of urinary bladder
Cancer	SNOMED	188244007	Malignant tumour of bladder neck
Cancer	SNOMED	188245008	Malignant neoplasm of ureteric orifice
Cancer	SNOMED	188247000	Malignant neoplasm, overlapping lesion of bladder
Cancer	SNOMED	188250002	Malignant neoplasm of kidney parenchyma
Cancer	SNOMED	188252005	Malignant neoplasm of renal calyx
Cancer	SNOMED	188253000	Malignant tumour of pelviureteric junction

Cancer	SNOMED	188256008	Malignant neoplasm of overlapping lesion of urinary organs
Cancer	SNOMED	188261005	Malignant neoplasm of eyeball excluding conjunctiva, cornea, retina and choroid
Cancer	SNOMED	188263008	Malignant neoplasm of ciliary body
Cancer	SNOMED	188264002	Malignant neoplasm of iris
Cancer	SNOMED	188265001	Malignant neoplasm of crystalline lens (disorder)
Cancer	SNOMED	188266000	Malignant neoplasm of sclera (disorder)
Cancer	SNOMED	188268004	Malignant neoplasm of connective tissue of orbit
Cancer	SNOMED	188269007	Malignant neoplasm of extraocular muscle of orbit (disorder)
Cancer	SNOMED	188272000	Malignant neoplasm of lacrimal gland
Cancer	SNOMED	188273005	Malignant neoplasm of lacrimal sac
Cancer	SNOMED	188274004	Malignant neoplasm of nasolacrimal duct
Cancer	SNOMED	188280007	Malignant neoplasm of cerebrum (excluding lobes and ventricles)
Cancer	SNOMED	188281006	Malignant neoplasm of basal ganglia
Cancer	SNOMED	188282004	Malignant neoplasm of cerebral cortex
Cancer	SNOMED	188285002	Malignant neoplasm of globus pallidus
Cancer	SNOMED	188286001	Malignant tumor of hypothalamus
Cancer	SNOMED	188287005	Malignant neoplasm of thalamus
Cancer	SNOMED	188289008	Malignant neoplasm of hippocampus
Cancer	SNOMED	188292007	Malignant tumour of choroid plexus
Cancer	SNOMED	188293002	Malignant neoplasm of floor of cerebral ventricle (disorder)
Cancer	SNOMED	188295009	Malignant neoplasm of cerebral peduncle
Cancer	SNOMED	188296005	Malignant neoplasm of medulla oblongata
Cancer	SNOMED	188297001	Malignant neoplasm of midbrain
Cancer	SNOMED	188298006	Malignant neoplasm of pons
Cancer	SNOMED	188301005	Malignant neoplasm of corpus callosum
Cancer	SNOMED	188302003	Malignant neoplasm of tapetum (disorder)
Cancer	SNOMED	188307009	Malignant tumour of cranial nerve
Cancer	SNOMED	188308004	Malignant neoplasm of olfactory bulb
Cancer	SNOMED	188312005	Malignant neoplasm of cerebral dura mater (disorder)
Cancer	SNOMED	188313000	Malignant neoplasm of cerebral arachnoid mater (disorder)
Cancer	SNOMED	188315007	Malignant neoplasm of cerebral pia mater (disorder)
Cancer	SNOMED	188317004	Malignant neoplasm of spinal dura mater (disorder)
Cancer	SNOMED	188318009	Malignant neoplasm of spinal arachnoid mater (disorder)
Cancer	SNOMED	188319001	Malignant neoplasm of spinal pia mater (disorder)
Cancer	SNOMED	188321006	Malignant neoplasm of peripheral nerves and autonomic nervous system
Cancer	SNOMED	188322004	Malignant neoplasm of peripheral nerves of head, face and neck
Cancer	SNOMED	188323009	Malignant neoplasm of peripheral nerves of upper limb, including shoulder
Cancer	SNOMED	188324003	Malignant neoplasm of peripheral nerves of lower limb, including hip
Cancer	SNOMED	188325002	Malignant neoplasm of peripheral nerve of thorax
Cancer	SNOMED	188326001	Malignant neoplasm of peripheral nerve of abdomen
Cancer	SNOMED	188327005	Malignant neoplasm of peripheral nerve of pelvis
Cancer	SNOMED	188339002	Malignant neoplasm of pituitary gland and craniopharyngeal duct
Cancer	SNOMED	188340000	Malignant tumor of craniopharyngeal duct
Cancer	SNOMED	188353002	Malignant neoplasm of head, neck and face
Cancer	SNOMED	188361007	Malignant neoplasm of thorax
Cancer	SNOMED	188366002	Malignant tumor of abdomen
Cancer	SNOMED	188445006	Secondary malignant neoplasm of retroperitoneum and peritoneum
Cancer	SNOMED	188454009	Secondary malignant neoplasm of skin of head
Cancer	SNOMED	188458007	Secondary malignant neoplasm of skin of shoulder and arm
Cancer	SNOMED	188459004	Secondary malignant neoplasm of skin of hip and leg
Cancer	SNOMED	188462001	Secondary malignant neoplasm of brain and spinal cord
Cancer	SNOMED	188469005	Secondary malignant neoplasm of cervix uteri
Cancer	SNOMED	188478004	Malignant neoplasms of independent (primary) multiple sites
Cancer	SNOMED	188487008	Lymphosarcoma and reticulosarcoma
Cancer	SNOMED	188489006	Reticulosarcoma of lymph nodes of head, face and neck
Cancer	SNOMED	188492005	Reticulosarcoma of lymph nodes of axilla and upper limb (disorder)
Cancer	SNOMED	188493000	Reticulosarcoma of lymph nodes of inguinal region and lower limb (disorder)
Cancer	SNOMED	188498009	Lymphosarcoma
Cancer	SNOMED	188500005	Lymphosarcoma of lymph nodes of head, face and neck
Cancer	SNOMED	188501009	Lymphosarcoma of intrathoracic lymph nodes
Cancer	SNOMED	188502002	Lymphosarcoma of intra-abdominal lymph nodes
Cancer	SNOMED	188503007	Lymphosarcoma of lymph nodes of axilla and upper limb (disorder)
Cancer	SNOMED	188504001	Lymphosarcoma of lymph nodes of inguinal region and lower limb
Cancer	SNOMED	188506004	Lymphosarcoma of spleen
Cancer	SNOMED	188507008	Lymphosarcoma of lymph nodes of multiple sites (disorder)
Cancer	SNOMED	188510001	Burkitt's lymphoma of lymph nodes of head, face and neck
Cancer	SNOMED	188511002	Burkitt's lymphoma of intrathoracic lymph nodes
Cancer	SNOMED	188512009	Burkitt's lymphoma of intra-abdominal lymph nodes
Cancer	SNOMED	188513004	Burkitt's lymphoma of lymph nodes of axilla and upper limb (disorder)
Cancer	SNOMED	188514005	Burkitt's lymphoma of lymph nodes of inguinal region and lower limb
Cancer	SNOMED	188515006	Burkitt's lymphoma of intrapelvic lymph nodes (disorder)
Cancer	SNOMED	188516007	Burkitt's lymphoma of spleen (disorder)
Cancer	SNOMED	188517003	Burkitt's lymphoma of lymph nodes of multiple sites (disorder)
Cancer	SNOMED	188524002	Hodgkin's paragranuloma of intrathoracic lymph nodes (disorder)

Cancer	SNOMED	188531003	Hodgkin's paragranuloma of lymph nodes of multiple sites (disorder)
Cancer	SNOMED	188534006	Hodgkin's granuloma of lymph nodes of head, face and neck
Cancer	SNOMED	188536008	Hodgkin's granuloma of intra-abdominal lymph nodes (disorder)
Cancer	SNOMED	188537004	Hodgkin's granuloma of lymph nodes of axilla and upper limb (disorder)
Cancer	SNOMED	188538009	Hodgkin's granuloma of lymph nodes of inguinal region and lower limb (disorder)
Cancer	SNOMED	188541000	Hodgkin's granuloma of lymph nodes of multiple sites (disorder)
Cancer	SNOMED	188544008	Hodgkin's sarcoma of lymph nodes of head, face and neck (disorder)
Cancer	SNOMED	188547001	Hodgkin's sarcoma of lymph nodes of axilla and upper limb
Cancer	SNOMED	188548006	Hodgkin's sarcoma of lymph nodes of inguinal region and lower limb (disorder)
Cancer	SNOMED	188551004	Hodgkin's sarcoma of lymph nodes of multiple sites (disorder)
Cancer	SNOMED	188554007	Hodgkin's disease, lymphocytic-histiocytic predominance of lymph nodes of head, face and neck
Cancer	SNOMED	188558005	Hodgkin's disease, lymphocytic-histiocytic predominance of lymph nodes of axilla and upper limb (disorder)
Cancer	SNOMED	188559002	Hodgkin's disease, lymphocytic-histiocytic predominance of lymph nodes of inguinal region and lower limb
Cancer	SNOMED	188562004	Hodgkin's disease, lymphocytic-histiocytic predominance of lymph nodes of multiple sites (disorder)
Cancer	SNOMED	188565002	Hodgkin's disease, nodular sclerosis of lymph nodes of head, face and neck
Cancer	SNOMED	188566001	Hodgkin's disease, nodular sclerosis of intrathoracic lymph nodes
Cancer	SNOMED	188567005	Hodgkin's disease, nodular sclerosis of intra-abdominal lymph nodes
Cancer	SNOMED	188568000	Hodgkin's disease, nodular sclerosis of lymph nodes of axilla and upper limb
Cancer	SNOMED	188569008	Hodgkin's disease, nodular sclerosis of lymph nodes of inguinal region and lower limb (disorder)
Cancer	SNOMED	188570009	Hodgkin's disease, nodular sclerosis of intrapelvic lymph nodes (disorder)
Cancer	SNOMED	188572001	Hodgkin's disease, nodular sclerosis of lymph nodes of multiple sites
Cancer	SNOMED	188575004	Hodgkin's disease, mixed cellularity of lymph nodes of head, face and neck
Cancer	SNOMED	188576003	Hodgkin's disease, mixed cellularity of intrathoracic lymph nodes
Cancer	SNOMED	188577007	Hodgkin's disease, mixed cellularity of intra-abdominal lymph nodes (disorder)
Cancer	SNOMED	188578002	Hodgkin's disease, mixed cellularity of lymph nodes of axilla and upper limb (disorder)
Cancer	SNOMED	188579005	Hodgkin's disease, mixed cellularity of lymph nodes of inguinal region and lower limb (disorder)
Cancer	SNOMED	188580008	Hodgkin's disease, mixed cellularity of intrapelvic lymph nodes (disorder)
Cancer	SNOMED	188582000	Hodgkin's disease, mixed cellularity of lymph nodes of multiple sites (disorder)
Cancer	SNOMED	188585003	Hodgkin's disease, lymphocytic depletion of lymph nodes of head, face and neck (disorder)
Cancer	SNOMED	188586002	Hodgkin's disease, lymphocytic depletion of intrathoracic lymph nodes (disorder)
Cancer	SNOMED	188587006	Hodgkin's disease, lymphocytic depletion of intra-abdominal lymph nodes (disorder)
Cancer	SNOMED	188589009	Hodgkin's disease, lymphocytic depletion of lymph nodes of axilla and upper limb
Cancer	SNOMED	188590000	Hodgkin's disease, lymphocytic depletion of lymph nodes of inguinal region and lower limb (disorder)
Cancer	SNOMED	188592008	Hodgkin's disease, lymphocytic depletion of spleen
Cancer	SNOMED	188593003	Hodgkin's disease, lymphocytic depletion of lymph nodes of multiple sites (disorder)
Cancer	SNOMED	188609000	Nodular lymphoma of lymph nodes of head, face and neck
Cancer	SNOMED	188612002	Nodular lymphoma of lymph nodes of axilla and upper limb (disorder)
Cancer	SNOMED	188613007	Nodular lymphoma of lymph nodes of inguinal region and lower limb
Cancer	SNOMED	188627002	Mycosis fungoides of lymph nodes of multiple sites
Cancer	SNOMED	188633006	SÃ©zary's disease of lymph nodes of axilla and upper limb (disorder)
Cancer	SNOMED	188637007	SÃ©zary's disease of lymph nodes of multiple sites (disorder)
Cancer	SNOMED	188640007	Malignant histiocytosis of lymph nodes of head, face and neck
Cancer	SNOMED	188641006	Malignant histiocytosis of lymph nodes of axilla and upper limb (disorder)
Cancer	SNOMED	188642004	Malignant histiocytosis of lymph nodes of inguinal region and lower limb (disorder)
Cancer	SNOMED	188645002	Leukaemic reticuloendotheliosis of lymph nodes of head, face and neck
Cancer	SNOMED	188660004	Malignant mast cell tumours
Cancer	SNOMED	188662007	Mast cell malignancy of lymph nodes of head, face and neck (disorder)
Cancer	SNOMED	188664008	Mast cell malignancy of intra-abdominal lymph nodes (disorder)
Cancer	SNOMED	188665009	Mast cell malignancy of lymph nodes of axilla and upper limb (disorder)
Cancer	SNOMED	188666005	Mast cell malignancy of lymph nodes of inguinal region and lower limb
Cancer	SNOMED	188668006	Mast cell malignancy of spleen (disorder)
Cancer	SNOMED	188669003	Mast cell malignancy of lymph nodes of multiple sites
Cancer	SNOMED	188672005	Follicular non-Hodgkin's mixed small cleaved and large cell lymphoma
Cancer	SNOMED	188674006	Diffuse malignant lymphoma - small non-cleaved cell
Cancer	SNOMED	188675007	Malignant lymphoma - small cleaved cell (disorder)
Cancer	SNOMED	188676008	Malignant lymphoma - mixed small and large cell
Cancer	SNOMED	188679001	Diffuse non-Hodgkin's lymphoma undifferentiated (diffuse)

Cancer	SNOMED	188691005	Malignant immunoproliferative small intestinal disease
Cancer	SNOMED	188718006	Extramedullary plasmacytoma (clinical)
Cancer	SNOMED	188725004	Lymphatic leukaemia
Cancer	SNOMED	188726003	Subacute lymphoid leukemia
Cancer	SNOMED	188728002	Aleukemic lymphoid leukemia (disorder)
Cancer	SNOMED	188729005	Adult T-cell leukaemia
Cancer	SNOMED	188732008	Myeloid leukemia
Cancer	SNOMED	188733003	Chronic eosinophilic leukaemia
Cancer	SNOMED	188734009	Chronic neutrophilic leukemia
Cancer	SNOMED	188736006	Subacute myeloid leukaemia
Cancer	SNOMED	188737002	Chloroma
Cancer	SNOMED	188738007	Granulocytic sarcoma
Cancer	SNOMED	188741003	Aleukemic myeloid leukemia (disorder)
Cancer	SNOMED	188744006	Histiocytic leukemia
Cancer	SNOMED	188745007	Chronic monocytic leukemia
Cancer	SNOMED	188746008	Subacute monocytic leukaemia
Cancer	SNOMED	188754005	Thrombocytic leukemia
Cancer	SNOMED	188768003	Myelomonocytic leukaemia
Cancer	SNOMED	188770007	Subacute myelomonocytic leukaemia
Cancer	SNOMED	189509003	Refractory anemia without sideroblasts, so stated (disorder)
Cancer	SNOMED	190030009	Compound leukemias (disorder)
Cancer	SNOMED	190818004	WaldenstrÃ¶m macroglobulinemia (disorder)
Cancer	SNOMED	190955000	Histiocytosis X syndrome (disorder)
Cancer	SNOMED	230156002	Malignant meningitis (disorder)
Cancer	SNOMED	230257000	Paraneoplastic motor neurone disease
Cancer	SNOMED	230586003	Neuropathy due to multiple myeloma
Cancer	SNOMED	230685009	Myasthenia gravis associated with thymoma (disorder)
Cancer	SNOMED	231829006	Malignant neoplasm of eyelid (disorder)
Cancer	SNOMED	231831002	Squamous cell carcinoma of eyelid (disorder)
Cancer	SNOMED	231833004	Sebaceous adenocarcinoma of eyelid (disorder)
Cancer	SNOMED	231834005	Malignant melanoma of eyelid
Cancer	SNOMED	231835006	Kaposi's sarcoma of eyelid (disorder)
Cancer	SNOMED	232075002	Lymphoma of retina (disorder)
Cancer	SNOMED	232681000000103	Chemotherapy delivery (procedure)
Cancer	SNOMED	233940007	Pulmonary tumor embolism (disorder)
Cancer	SNOMED	235331000000103	Delivery of oral chemotherapy for neoplasm (regime/therapy)
Cancer	SNOMED	235391000000102	Delivery of chemotherapy for neoplasm (procedure)
Cancer	SNOMED	235966007	Cystadenocarcinoma of pancreas (disorder)
Cancer	SNOMED	236005001	Malignant ascites
Cancer	SNOMED	236513009	Lymphoma of kidney (disorder)
Cancer	SNOMED	237252008	Placental site trophoblastic tumor (disorder)
Cancer	SNOMED	237833006	Carcinoid crisis (disorder)
Cancer	SNOMED	23833000	Poisoning caused by antineoplastic AND/OR immunosuppressive drug (disorder)
Cancer	SNOMED	238831000000103	Delivery of exclusively oral chemotherapy for neoplasm (regime/therapy)
Cancer	SNOMED	238841000000107	Delivery of complex chemotherapy for neoplasm including prolonged infusional treatment at first attendance (procedure)
Cancer	SNOMED	238851000000105	Delivery of complex parenteral chemotherapy for neoplasm at first attendance (procedure)
Cancer	SNOMED	238861000000108	Delivery of simple parenteral chemotherapy for neoplasm at first attendance (procedure)
Cancer	SNOMED	239071000000100	Delivery of subsequent element of cycle of chemotherapy for neoplasm (procedure)
Cancer	SNOMED	239297008	Lymphomatoid granulomatosis of the lung (disorder)
Cancer	SNOMED	239898008	Polymyositis with malignant disease
Cancer	SNOMED	239901009	Dermatopolymyositis in neoplastic disease
Cancer	SNOMED	240531002	African Burkitt's lymphoma (disorder)
Cancer	SNOMED	240794006	Splenic schistosomal giant cell lymphoma (disorder)
Cancer	SNOMED	248331000000109	Cancer monitoring telephone invitation (procedure)
Cancer	SNOMED	253000007	Neuroendocrine carcinoma (disorder)
Cancer	SNOMED	253001006	Merkel cell carcinoma (disorder)
Cancer	SNOMED	253003009	Carcinoid bronchial adenoma (disorder)
Cancer	SNOMED	253006001	Somatostatinoma (disorder)
Cancer	SNOMED	253017000	Klatskin's tumor (disorder)
Cancer	SNOMED	253018005	Fibrolamellar hepatocellular carcinoma (disorder)
Cancer	SNOMED	253042009	Myxoid dermatofibrosarcoma protuberans (disorder)
Cancer	SNOMED	253096008	Peripheral neuroectodermal tumor (disorder)
Cancer	SNOMED	254389005	Carcinoma of vermilion border of upper lip (disorder)
Cancer	SNOMED	254412006	Malignant tumor of tip of tongue (disorder)
Cancer	SNOMED	254423005	Carcinoma of lingual tonsil (disorder)
Cancer	SNOMED	254424004	Carcinoma of upper gum (disorder)
Cancer	SNOMED	254425003	Carcinoma of lower gum (disorder)
Cancer	SNOMED	254427006	Carcinoma of anterior part of floor of mouth (disorder)
Cancer	SNOMED	254431000	Carcinoma of lateral part of floor of mouth (disorder)
Cancer	SNOMED	254434008	Carcinoma of hard palate (disorder)

Cancer	SNOMED	254435009	Carcinoma of soft palate (disorder)
Cancer	SNOMED	254436005	Carcinoma of uvula (disorder)
Cancer	SNOMED	254437001	Squamous cell carcinoma of buccal mucosa (disorder)
Cancer	SNOMED	254441002	Carcinoma of upper buccal sulcus (disorder)
Cancer	SNOMED	254445006	Carcinoma of lower buccal sulcus (disorder)
Cancer	SNOMED	254454009	Carcinoma of lower labial sulcus (disorder)
Cancer	SNOMED	254457002	Carcinoma of retromolar area (disorder)
Cancer	SNOMED	254459004	Malignant tumour of anterior pillar of fauces
Cancer	SNOMED	254462001	Carcinoma of parotid gland (disorder)
Cancer	SNOMED	254465004	Carcinoma of submandibular gland (disorder)
Cancer	SNOMED	254466003	Carcinoma of sublingual gland (disorder)
Cancer	SNOMED	254474002	Malignant tumor of nasal skeleton (disorder)
Cancer	SNOMED	254478004	Malignant tumor of inferior turbinate (disorder)
Cancer	SNOMED	254484001	Malignant tumor of posterior margin of nasal septum and choanae
Cancer	SNOMED	254503007	Malignant tumor of inferior surface of soft palate (disorder)
Cancer	SNOMED	254509006	Malignant tumor of anterior commissure (disorder)
Cancer	SNOMED	254517003	Malignant tumor of suprahyoid epiglottis (disorder)
Cancer	SNOMED	254520006	Malignant tumor of infrahyoid epiglottis (disorder)
Cancer	SNOMED	254526000	Malignant tumor of laryngeal ventricle (disorder)
Cancer	SNOMED	254530002	Malignant tumor of parapharyngeal space (disorder)
Cancer	SNOMED	254535007	Carcinoma of cervical part of esophagus (disorder)
Cancer	SNOMED	254539001	Carcinoma of thoracic part of esophagus (disorder)
Cancer	SNOMED	254543002	Carcinoma of abdominal part of esophagus (disorder)
Cancer	SNOMED	254547001	Carcinoma of upper third of esophagus (disorder)
Cancer	SNOMED	254549003	Carcinoma of middle third of esophagus (disorder)
Cancer	SNOMED	254551004	Carcinoma of lower third of esophagus (disorder)
Cancer	SNOMED	254553001	Carcinoma of cardia (disorder)
Cancer	SNOMED	254555008	Carcinoma of fundus of stomach (disorder)
Cancer	SNOMED	254557000	Carcinoma of body of stomach (disorder)
Cancer	SNOMED	254559002	Carcinoma of pyloric antrum (disorder)
Cancer	SNOMED	254561006	Carcinoma of pylorus (disorder)
Cancer	SNOMED	254563009	Carcinoma of lesser curve of stomach (disorder)
Cancer	SNOMED	254567005	Carcinoma of greater curve of stomach (disorder)
Cancer	SNOMED	254570009	Carcinoma of duodenum (disorder)
Cancer	SNOMED	254582000	Carcinoma of rectum
Cancer	SNOMED	254586002	Malignant tumor of anorectal junction (disorder)
Cancer	SNOMED	254601002	Sarcoma of liver (disorder)
Cancer	SNOMED	254609000	Carcinoma of ampulla of Vater (disorder)
Cancer	SNOMED	254611009	Malignant tumor of endocrine pancreas (disorder)
Cancer	SNOMED	254612002	Carcinoma of endocrine pancreas (disorder)
Cancer	SNOMED	254619006	Adenoid cystic carcinoma of trachea (disorder)
Cancer	SNOMED	254620000	Squamous cell carcinoma of trachea (disorder)
Cancer	SNOMED	254622008	Squamous cell carcinoma of bronchus (disorder)
Cancer	SNOMED	254625005	Malignant tumor of lung parenchyma (disorder)
Cancer	SNOMED	254626006	Adenocarcinoma of lung (disorder)
Cancer	SNOMED	254627002	Carcinoid tumor of lung (disorder)
Cancer	SNOMED	254628007	Carcinoma of lung parenchyma (disorder)
Cancer	SNOMED	254629004	Large cell carcinoma of lung (disorder)
Cancer	SNOMED	254631008	Giant cell carcinoma of lung (disorder)
Cancer	SNOMED	254632001	Small cell carcinoma of lung (disorder)
Cancer	SNOMED	254633006	Oat cell carcinoma of lung (disorder)
Cancer	SNOMED	254634000	Squamous cell carcinoma of lung (disorder)
Cancer	SNOMED	254635004	Epithelioid hemangioendothelioma of lung (disorder)
Cancer	SNOMED	254637007	Non-small cell lung cancer (disorder)
Cancer	SNOMED	254638002	Pancoast tumor (disorder)
Cancer	SNOMED	254645002	Malignant mesothelioma of pleura
Cancer	SNOMED	254653005	Spindle cell squamous carcinoma of skin (disorder)
Cancer	SNOMED	254707006	Malignant skin tumor with eccrine differentiation (disorder)
Cancer	SNOMED	254708001	Eccrine porocarcinoma of skin (disorder)
Cancer	SNOMED	254709009	Digital papillary eccrine carcinoma of skin (disorder)
Cancer	SNOMED	254711000	Adenoid cystic eccrine carcinoma of skin (disorder)
Cancer	SNOMED	254712007	Microcystic adnexal carcinoma of skin (disorder)
Cancer	SNOMED	254713002	Mucoepidermoid carcinoma of skin (disorder)
Cancer	SNOMED	254714008	Mucinous eccrine carcinoma of skin (disorder)
Cancer	SNOMED	254726003	Malignant skin tumor with apocrine differentiation (disorder)
Cancer	SNOMED	254730000	Superficial spreading malignant melanoma of skin (disorder)
Cancer	SNOMED	254731001	Nodular malignant melanoma of skin (disorder)
Cancer	SNOMED	254732008	Acral lentiginous malignant melanoma of skin (disorder)
Cancer	SNOMED	254733003	Malignant melanoma arising in intradermal nevus (disorder)
Cancer	SNOMED	254734009	Malignant melanoma arising in congenital nevus (disorder)
Cancer	SNOMED	254748009	Cutaneous fibrosarcoma (disorder)
Cancer	SNOMED	254764001	Peripheral neuroepithelioma (disorder)
Cancer	SNOMED	254771006	Cutaneous leiomyosarcoma (disorder)
Cancer	SNOMED	254792006	Proliferating angioendotheliomatosis
Cancer	SNOMED	254794007	Angiosarcoma of skin (disorder)

Cancer	SNOMED	254797000	Malignant hemangiopericytoma of skin (disorder)
Cancer	SNOMED	254800003	Epithelioid cell sarcoma of skin (disorder)
Cancer	SNOMED	254824006	Malignant tumor of mesothelial tissue (disorder)
Cancer	SNOMED	254828009	Malignant lipomatous tumor (disorder)
Cancer	SNOMED	254829001	Liposarcoma (disorder)
Cancer	SNOMED	254837009	Malignant tumor of breast
Cancer	SNOMED	254838004	Carcinoma of breast (disorder)
Cancer	SNOMED	254839007	Scirrhous carcinoma of breast (disorder)
Cancer	SNOMED	254840009	Inflammatory carcinoma of breast (disorder)
Cancer	SNOMED	254841008	Cancer en cuirasse (disorder)
Cancer	SNOMED	254843006	Familial cancer of breast (disorder)
Cancer	SNOMED	254844000	Malignant phyllodes tumor of breast (disorder)
Cancer	SNOMED	254849005	Malignant epithelial tumor of ovary (disorder)
Cancer	SNOMED	254850005	Serous papillary cystadenocarcinoma ovary (disorder)
Cancer	SNOMED	254851009	Mucinous cystadenocarcinoma of ovary (disorder)
Cancer	SNOMED	254852002	Endometrioid carcinoma ovary (disorder)
Cancer	SNOMED	254855000	Mixed epithelial tumor of ovary (disorder)
Cancer	SNOMED	254856004	Undifferentiated carcinoma of ovary (disorder)
Cancer	SNOMED	254860001	Malignant sex cord tumor of ovary (disorder)
Cancer	SNOMED	254861002	Malignant granulosa cell tumor of ovary (disorder)
Cancer	SNOMED	254863004	Granulosa cell tumor of ovary (disorder)
Cancer	SNOMED	254869000	Malignant germ cell tumor of ovary (disorder)
Cancer	SNOMED	254870004	Choriocarcinoma of ovary (disorder)
Cancer	SNOMED	254871000	Immature teratoma of ovary (disorder)
Cancer	SNOMED	254872007	Embryonal carcinoma of ovary (disorder)
Cancer	SNOMED	254874008	Dysgerminoma of ovary (disorder)
Cancer	SNOMED	254876005	Endodermal sinus tumor of ovary (disorder)
Cancer	SNOMED	254877001	Sarcoma of uterus (disorder)
Cancer	SNOMED	254878006	Endometrial carcinoma (disorder)
Cancer	SNOMED	254886006	Squamous cell carcinoma of cervix (disorder)
Cancer	SNOMED	254887002	Adenocarcinoma of cervix (disorder)
Cancer	SNOMED	254888007	Adenosquamous carcinoma of cervix (disorder)
Cancer	SNOMED	254889004	Carcinoma of cervix stage 0 (disorder)
Cancer	SNOMED	254893005	Carcinoma of vagina (disorder)
Cancer	SNOMED	254895003	Squamous cell carcinoma of vulva (disorder)
Cancer	SNOMED	254896002	Malignant melanoma of vulva (disorder)
Cancer	SNOMED	254897006	Sarcoma of vulva (disorder)
Cancer	SNOMED	254900004	Carcinoma of prostate (disorder)
Cancer	SNOMED	254904008	Carcinoma of glans penis (disorder)
Cancer	SNOMED	254912000	Regressed malignant testicular tumor (disorder)
Cancer	SNOMED	254915003	Clear cell carcinoma of kidney (disorder)
Cancer	SNOMED	254917006	Papillary cystadenocarcinoma of kidney (disorder)
Cancer	SNOMED	254918001	Sarcoma of kidney (disorder)
Cancer	SNOMED	254934003	Malignant tumor of urethral stump (disorder)
Cancer	SNOMED	254938000	Astrocytoma of brain (disorder)
Cancer	SNOMED	254940005	Oligodendroglioma of brain (disorder)
Cancer	SNOMED	254948003	Astrocytoma of spinal cord (disorder)
Cancer	SNOMED	254950006	Oligodendroglioma of spinal cord (disorder)
Cancer	SNOMED	254955001	Pituitary carcinoma (disorder)
Cancer	SNOMED	254969001	Malignant tumor of olfactory tract (disorder)
Cancer	SNOMED	254972008	Malignant tumor of optic nerve and sheath (disorder)
Cancer	SNOMED	254973003	Malignant astrocytoma of optic nerve (disorder)
Cancer	SNOMED	254974009	Malignant tumor of optic nerve sheath (disorder)
Cancer	SNOMED	254975005	Malignant meningioma of optic nerve sheath (disorder)
Cancer	SNOMED	254980001	Malignant tumor of acoustic vestibular nerve
Cancer	SNOMED	254983004	Malignant tumor of spinal nerve and sheath (disorder)
Cancer	SNOMED	254986007	Malignant tumour of peripheral nerve
Cancer	SNOMED	254987003	Adenoid cystic carcinoma of lacrimal gland (disorder)
Cancer	SNOMED	254988008	Adenocarcinoma of lacrimal gland (disorder)
Cancer	SNOMED	254989000	Carcinoma ex pleomorphic adenoma of lacrimal gland (disorder)
Cancer	SNOMED	254990009	Mucoepidermoid tumor of lacrimal gland (disorder)
Cancer	SNOMED	254993006	Liposarcoma of orbit (disorder)
Cancer	SNOMED	254994000	Rhabdomyosarcoma of orbit (disorder)
Cancer	SNOMED	254995004	Malignant hemangiopericytoma of orbit (disorder)
Cancer	SNOMED	254996003	Malignant fibrous histiocytoma of orbit (disorder)
Cancer	SNOMED	255003007	Squamous cell carcinoma of conjunctiva (disorder)
Cancer	SNOMED	255004001	Malignant melanoma of conjunctiva (disorder)
Cancer	SNOMED	255008003	Squamous cell carcinoma of cornea (disorder)
Cancer	SNOMED	255012009	Malignant melanoma of iris (disorder)
Cancer	SNOMED	255015006	Malignant melanoma of ciliary body (disorder)
Cancer	SNOMED	255016007	Adenocarcinoma of pigmented epithelium of ciliary body (disorder)
Cancer	SNOMED	255017003	Adenocarcinoma of non-pigmented epithelium of ciliary body (disorder)
Cancer	SNOMED	255021005	Malignant melanoma of choroid (disorder)
Cancer	SNOMED	255028004	Follicular thyroid carcinoma (disorder)
Cancer	SNOMED	255029007	Papillary thyroid carcinoma (disorder)

Cancer	SNOMED	255030002	Mixed follicular and papillary thyroid carcinoma (disorder)
Cancer	SNOMED	255031003	Anaplastic thyroid carcinoma (disorder)
Cancer	SNOMED	255032005	Medullary thyroid carcinoma (disorder)
Cancer	SNOMED	255035007	Adrenal carcinoma (disorder)
Cancer	SNOMED	255037004	Parathyroid carcinoma (disorder)
Cancer	SNOMED	255044008	Malignant pinealoma (disorder)
Cancer	SNOMED	255052006	Malignant tumor of unknown origin
Cancer	SNOMED	255056009	Malignant tumor of head and neck
Cancer	SNOMED	255066001	Carcinoma of genitourinary organ
Cancer	SNOMED	255067005	Sarcoma of bone and connective tissue
Cancer	SNOMED	255068000	Carcinoma of bone, connective tissue, skin and breast
Cancer	SNOMED	255069008	Carcinoma of lip, oral cavity and pharynx
Cancer	SNOMED	255071008	Squamous cell carcinoma of lip (disorder)
Cancer	SNOMED	255072001	Malignant tumor of salivary gland (disorder)
Cancer	SNOMED	255073006	Malignant tumor of ear, nose and throat
Cancer	SNOMED	255074000	Malignant tumor of nasal cavity and nasopharynx (disorder)
Cancer	SNOMED	255075004	Malignant tumor of lateral nasal wall (disorder)
Cancer	SNOMED	255077007	Malignant tumour of digestive organ
Cancer	SNOMED	255078002	Malignant tumor of esophagus, stomach and duodenum (disorder)
Cancer	SNOMED	255081007	Carcinoma of cecum
Cancer	SNOMED	255083005	Malignant tumor of anus and anal canal
Cancer	SNOMED	255084004	Squamous cell carcinoma of anal margin
Cancer	SNOMED	255086002	Carcinoma common bile duct
Cancer	SNOMED	255087006	Malignant polyp of biliary tract (disorder)
Cancer	SNOMED	255088001	Malignant tumor of exocrine pancreas (disorder)
Cancer	SNOMED	255090000	Malignant neoplasm of carpal bones
Cancer	SNOMED	255091001	Malignant neoplasm of metacarpal bones
Cancer	SNOMED	255093003	Malignant skin tumor with adnexal differentiation (disorder)
Cancer	SNOMED	255096006	Malignant tumor of dermis (disorder)
Cancer	SNOMED	255101006	SÃ©zary disease of skin (disorder)
Cancer	SNOMED	255102004	Angioendotheliomatosis (disorder)
Cancer	SNOMED	255107005	Seminoma of testis
Cancer	SNOMED	255108000	Carcinoma of bladder (disorder)
Cancer	SNOMED	255109008	Transitional cell carcinoma of bladder (disorder)
Cancer	SNOMED	255110003	Adenocarcinoma of bladder (disorder)
Cancer	SNOMED	255111004	Squamous cell carcinoma of bladder (disorder)
Cancer	SNOMED	255114007	Kaposi's sarcoma of conjunctiva (disorder)
Cancer	SNOMED	255118005	Secondary lymphangitic carcinoma (disorder)
Cancer	SNOMED	255119002	Lymphangitis carcinomatosa (disorder)
Cancer	SNOMED	255121007	Carcinomatosis of peritoneal cavity (disorder)
Cancer	SNOMED	255123005	Metastasis to nervous system and eye (disorder)
Cancer	SNOMED	255124004	Metastasis to peripheral nerve (disorder)
Cancer	SNOMED	255127006	Local tumor spread (disorder)
Cancer	SNOMED	255128001	Malignant infiltration of peripheral nerve (disorder)
Cancer	SNOMED	255129009	Malignant infiltration of peripheral nerve plexus (disorder)
Cancer	SNOMED	265760000	Intravenous chemotherapy (procedure)
Cancer	SNOMED	265761001	Intramuscular chemotherapy (procedure)
Cancer	SNOMED	265762008	Subcutaneous chemotherapy (procedure)
Cancer	SNOMED	266719004	Oral chemotherapy (procedure)
Cancer	SNOMED	267540007	Neutropenia caused by irradiation (disorder)
Cancer	SNOMED	268500004	Prophylactic chemotherapy (procedure)
Cancer	SNOMED	269459004	Malignant tumour of lesser curve of stomach
Cancer	SNOMED	269460009	Malignant tumor of greater curve of stomach
Cancer	SNOMED	269463006	Malignant tumor of middle ear and mastoid
Cancer	SNOMED	269464000	Malignant neoplasm of upper lobe, bronchus or lung
Cancer	SNOMED	269467007	Malignant neoplasm of hand bones
Cancer	SNOMED	269469005	Malignant neoplasm of soft tissue
Cancer	SNOMED	269473008	Secondary malignant neoplasm of respiratory and digestive systems
Cancer	SNOMED	269475001	Malignant neoplasm of lymphatic and haemopoietic tissue
Cancer	SNOMED	269476000	Nodular lymphoma
Cancer	SNOMED	269515006	Carcinoma of lip
Cancer	SNOMED	269516007	Tongue carcinoma (disorder)
Cancer	SNOMED	269533000	Carcinoma of colon (disorder)
Cancer	SNOMED	269544008	Carcinoma of the rectosigmoid junction (disorder)
Cancer	SNOMED	269578002	Malignant melanoma of head and neck
Cancer	SNOMED	269579005	Malignant melanoma of trunk
Cancer	SNOMED	269580008	Malignant melanoma of upper limb
Cancer	SNOMED	269581007	Malignant melanoma of lower limb (disorder)
Cancer	SNOMED	269616004	Secondary nodes - axilla/arm (disorder)
Cancer	SNOMED	269617008	Secondary nodes - inguinal/leg (disorder)
Cancer	SNOMED	271323007	Malignant neoplasm of lip, oral cavity and pharynx
Cancer	SNOMED	271467005	Malignant neoplasm of bone, connective tissue, skin and breast
Cancer	SNOMED	271468000	Malignant neoplasm of genitourinary organ
Cancer	SNOMED	271568003	Malig neoplasm of lower lip, oral aspect
Cancer	SNOMED	271943005	Carcinoma of base of tongue (disorder)

Cancer	SNOMED	274084007	Palate carcinoma (disorder)
Cancer	SNOMED	274085008	Tonsil carcinoma (disorder)
Cancer	SNOMED	274087000	Malignant melanoma of eye (disorder)
Cancer	SNOMED	274088005	Secondary malignant neoplasm of unknown site
Cancer	SNOMED	274902006	Combined hepatocellular carcinoma and cholangiocarcinoma (disorder)
Cancer	SNOMED	274905008	Malignant lymphoma - lymphocytic, intermediate differentiation
Cancer	SNOMED	275266006	Metastasis to digestive organs (disorder)
Cancer	SNOMED	275394001	Carcinoma ventral surface of tongue (disorder)
Cancer	SNOMED	275395000	Carcinoma anterior 2/3 tongue ventrum (disorder)
Cancer	SNOMED	275396004	Carcinoma of anterior two-thirds of tongue - dorsal surface (disorder)
Cancer	SNOMED	275399006	Malignant tumour of lipstick area of lip
Cancer	SNOMED	275419009	Primary vulval cancer
Cancer	SNOMED	275490009	Carcinoma of tongue base - dorsal surface (disorder)
Cancer	SNOMED	275524009	Immunoproliferative neoplasm
Cancer	SNOMED	276420005	Malignant tumor of corpus cavernosum (disorder)
Cancer	SNOMED	276738009	Primary signet ring carcinoma of skin (disorder)
Cancer	SNOMED	276750003	Malignant tumor of skin with pilar differentiation (disorder)
Cancer	SNOMED	276751004	Amelanotic malignant melanoma of skin (disorder)
Cancer	SNOMED	276797002	Malignant tumor of fibrous tissue (disorder)
Cancer	SNOMED	276799004	Dermatofibrosarcoma protuberans (disorder)
Cancer	SNOMED	276803003	Adenocarcinoma of esophagus (disorder)
Cancer	SNOMED	276804009	Squamous cell carcinoma of esophagus (disorder)
Cancer	SNOMED	276808007	Carcinoid tumor of stomach (disorder)
Cancer	SNOMED	276809004	Early gastric cancer (disorder)
Cancer	SNOMED	276810009	Late gastric cancer (disorder)
Cancer	SNOMED	276811008	Gastric lymphoma (disorder)
Cancer	SNOMED	276815004	Lymphoma of intestine (disorder)
Cancer	SNOMED	276821000	Malignant melanoma of anus (disorder)
Cancer	SNOMED	276822007	Malignant melanoma of rectum (disorder)
Cancer	SNOMED	276826005	Malignant glioma of brain (disorder)
Cancer	SNOMED	276827001	Malignant glioma of spinal cord (disorder)
Cancer	SNOMED	276828006	Glioblastoma multiforme of brain (disorder)
Cancer	SNOMED	276829003	Glioblastoma multiforme of spinal cord (disorder)
Cancer	SNOMED	276836002	Primary cerebral lymphoma (disorder)
Cancer	SNOMED	276870001	Carcinoma of fallopian tube (disorder)
Cancer	SNOMED	276876007	Carcinoma of Bartholin's gland (disorder)
Cancer	SNOMED	276952000	Squamous cell carcinoma of tongue (disorder)
Cancer	SNOMED	276953005	Squamous cell carcinoma of gum (disorder)
Cancer	SNOMED	276954004	Squamous cell carcinoma of floor of mouth (disorder)
Cancer	SNOMED	276962007	Squamous cell carcinoma of palate (disorder)
Cancer	SNOMED	276975007	Carcinoma of larynx (disorder)
Cancer	SNOMED	277156006	Malignant tumor of external ear (disorder)
Cancer	SNOMED	277461004	Anaplastic astrocytoma of brain (disorder)
Cancer	SNOMED	277466009	Lymphoproliferative disorder (disorder)
Cancer	SNOMED	277473004	B-CLL - B-cell chronic lymphocytic leukemia
Cancer	SNOMED	277474005	B-cell chronic lymphocytic leukaemia variant
Cancer	SNOMED	277505007	Medulloblastoma of cerebellum (disorder)
Cancer	SNOMED	277530005	Malignant melanoma of meninges (disorder)
Cancer	SNOMED	277543005	Malignant white blood cell disorder (disorder)
Cancer	SNOMED	277545003	T-cell chronic lymphocytic leukaemia
Cancer	SNOMED	277549009	Chronic lymphocytic prolymphocytic leukemia syndrome
Cancer	SNOMED	277550009	Richter's syndrome
Cancer	SNOMED	277551008	Splenic lymphoma with villous lymphocytes
Cancer	SNOMED	277567002	T-cell prolymphocytic leukemia
Cancer	SNOMED	277568007	Hairy cell leukaemia variant
Cancer	SNOMED	277569004	Large granular lymphocytic leukemia
Cancer	SNOMED	277570003	Lymphoma with spill
Cancer	SNOMED	277571004	B-cell acute lymphoblastic leukemia
Cancer	SNOMED	277572006	Pre B-cell acute lymphoblastic leukemia (disorder)
Cancer	SNOMED	277573001	Common acute lymphoblastic leukemia (disorder)
Cancer	SNOMED	277574007	Null cell acute lymphoblastic leukemia (disorder)
Cancer	SNOMED	277575008	T-cell acute lymphoblastic leukemia (disorder)
Cancer	SNOMED	277579002	Light chain myeloma (disorder)
Cancer	SNOMED	277580004	Non-secretory myeloma (disorder)
Cancer	SNOMED	277587001	Juvenile chronic myeloid leukemia (disorder)
Cancer	SNOMED	277589003	Atypical chronic myeloid leukemia (disorder)
Cancer	SNOMED	277597005	Myelodysplastic syndrome with isolated del(5q) (disorder)
Cancer	SNOMED	277601005	Acute monoblastic leukemia (disorder)
Cancer	SNOMED	277602003	Acute megakaryoblastic leukemia (disorder)
Cancer	SNOMED	277604002	Acute eosinophilic leukemia (disorder)
Cancer	SNOMED	277605001	Tumor lysis syndrome (disorder)
Cancer	SNOMED	277609007	Hodgkin's disease, lymphocytic predominance - diffuse (disorder)
Cancer	SNOMED	277610002	Hodgkin's disease, nodular sclerosis - lymphocytic predominance (disorder)
Cancer	SNOMED	277612005	Hodgkin's disease, nodular sclerosis - lymphocytic depletion (disorder)
Cancer	SNOMED	277613000	Cutaneous/peripheral T-cell lymphoma

Cancer	SNOMED	277615007	Low grade B-cell lymphoma
Cancer	SNOMED	277616008	Diffuse low grade B-cell lymphoma (disorder)
Cancer	SNOMED	277617004	High grade B-cell lymphoma (disorder)
Cancer	SNOMED	277618009	Follicular low grade B-cell lymphoma (disorder)
Cancer	SNOMED	277619001	B-cell prolymphocytic leukaemia
Cancer	SNOMED	277622004	Mucosa-associated lymphoma
Cancer	SNOMED	277623009	Monocytoid B-cell lymphoma (disorder)
Cancer	SNOMED	277624003	Follicular malignant lymphoma - mixed cell type (disorder)
Cancer	SNOMED	277625002	Follicular non-Hodgkin's small cleaved cell lymphoma
Cancer	SNOMED	277626001	Diffuse high grade B-cell lymphoma (disorder)
Cancer	SNOMED	277627005	Nodular high grade B-cell lymphoma (disorder)
Cancer	SNOMED	277628000	Diffuse malignant lymphoma - large cleaved cell (disorder)
Cancer	SNOMED	277629008	Diffuse malignant lymphoma - large non-cleaved cell (disorder)
Cancer	SNOMED	277637000	Large cell anaplastic lymphoma (disorder)
Cancer	SNOMED	277641001	Malignant lymphoma - histiocytic, nodular
Cancer	SNOMED	277642008	Low grade T-cell lymphoma (disorder)
Cancer	SNOMED	277643003	High grade T-cell lymphoma (disorder)
Cancer	SNOMED	277651000	Peripheral T-cell lymphoma - pleomorphic small cell (disorder)
Cancer	SNOMED	277653002	Peripheral T-cell lymphoma - pleomorphic medium and large cell (disorder)
Cancer	SNOMED	277654008	Enteropathy-associated T-cell lymphoma (disorder)
Cancer	SNOMED	277664004	Malignant lymphoma of testis (disorder)
Cancer	SNOMED	277782009	Malignant peritoneal local recurrence (disorder)
Cancer	SNOMED	278024000	Rhabdomyosarcoma of bladder (disorder)
Cancer	SNOMED	278042005	Malignant teratoma of mediastinum (disorder)
Cancer	SNOMED	278043000	Malignant seminoma of mediastinum (disorder)
Cancer	SNOMED	278044006	Malignant neuroma of mediastinum (disorder)
Cancer	SNOMED	278046008	Sarcoma of bladder (disorder)
Cancer	SNOMED	278050001	Sarcoma of breast (disorder)
Cancer	SNOMED	278051002	Malignant lymphoma of thyroid gland (disorder)
Cancer	SNOMED	278052009	Malignant lymphoma of breast (disorder)
Cancer	SNOMED	278053004	Intraductal carcinoma of breast (disorder)
Cancer	SNOMED	278054005	Lobular carcinoma of breast (disorder)
Cancer	SNOMED	278055006	Malignant Leydig cell tumor of testis (disorder)
Cancer	SNOMED	278057003	Sertoli cell tumor of testis (disorder)
Cancer	SNOMED	278060005	Endometrioid carcinoma of prostate (disorder)
Cancer	SNOMED	278065000	Superior pulmonary sulcus syndrome
Cancer	SNOMED	278433008	Malignant infiltration of soft tissue (disorder)
Cancer	SNOMED	278453007	Acute biphenotypic leukemia (disorder)
Cancer	SNOMED	278491007	Mixed seminoma teratoma of testis (disorder)
Cancer	SNOMED	280959007	Malignant tumor of lacrimal drainage structure (disorder)
Cancer	SNOMED	281560004	Neuroblastoma of brain
Cancer	SNOMED	281562007	Adrenal neuroblastoma
Cancer	SNOMED	281563002	Thoracic neuroblastoma
Cancer	SNOMED	281564008	Pelvic neuroblastoma
Cancer	SNOMED	281565009	Paraspinal neuroblastoma
Cancer	SNOMED	281566005	Abdominothoracic neuroblastoma
Cancer	SNOMED	281702006	Tibial adamantinoma (disorder)
Cancer	SNOMED	285310000	Carcinoma of anal canal (disorder)
Cancer	SNOMED	285312008	Carcinoma of sigmoid colon (disorder)
Cancer	SNOMED	285420006	Immunoglobulin A myeloma (disorder)
Cancer	SNOMED	285421005	Immunoglobulin G myeloma (disorder)
Cancer	SNOMED	285422003	Immunoglobulin D myeloma (disorder)
Cancer	SNOMED	285432005	Carcinoma of cervix
Cancer	SNOMED	285603002	Metastasis to bronchus of unknown primary (disorder)
Cancer	SNOMED	285604008	Metastasis to lung of unknown primary (disorder)
Cancer	SNOMED	285605009	Metastasis to pleura of unknown primary (disorder)
Cancer	SNOMED	285607001	Metastasis to mediastinum of unknown primary (disorder)
Cancer	SNOMED	285609003	Metastasis to small intestine of unknown primary (disorder)
Cancer	SNOMED	285610008	Metastasis to large intestine of unknown primary (disorder)
Cancer	SNOMED	285611007	Metastasis to colon of unknown primary (disorder)
Cancer	SNOMED	285612000	Metastasis to rectum of unknown primary (disorder)
Cancer	SNOMED	285613005	Metastasis to liver of unknown primary (disorder)
Cancer	SNOMED	285614004	Metastasis to pancreas of unknown primary (disorder)
Cancer	SNOMED	285615003	Metastasis to spleen of unknown primary (disorder)
Cancer	SNOMED	285616002	Metastasis to peritoneum of unknown primary (disorder)
Cancer	SNOMED	285617006	Metastasis to retroperitoneum of unknown primary (disorder)
Cancer	SNOMED	285618001	Metastasis to bone of unknown primary (disorder)
Cancer	SNOMED	285619009	Metastasis to vertebral column of unknown primary (disorder)
Cancer	SNOMED	285631006	Metastasis to skin of unknown primary (disorder)
Cancer	SNOMED	285633009	Metastasis to soft tissue of unknown primary (disorder)
Cancer	SNOMED	285634003	Metastasis to breast of unknown primary (disorder)
Cancer	SNOMED	285635002	Metastasis to uterus of unknown primary (disorder)
Cancer	SNOMED	285637005	Metastasis to ovary of unknown primary (disorder)
Cancer	SNOMED	285639008	Metastasis to kidney of unknown primary (disorder)
Cancer	SNOMED	285640005	Metastasis to bladder of unknown primary (disorder)

Cancer	SNOMED	285641009	Metastasis to brain of unknown primary (disorder)
Cancer	SNOMED	285643007	Metastasis to adrenal gland of unknown primary (disorder)
Cancer	SNOMED	285644001	Metastasis to lymph node of unknown primary (disorder)
Cancer	SNOMED	285645000	Disseminated malignancy of unknown primary (disorder)
Cancer	SNOMED	285769009	Acute promyelocytic leukemia - hypogranular variant (disorder)
Cancer	SNOMED	285776004	Intermediate grade B-cell lymphoma (disorder)
Cancer	SNOMED	285839005	Acute myelomonocytic leukemia - eosinophilic variant (disorder)
Cancer	SNOMED	286887005	Carcinoma liver and/or biliary system (disorder)
Cancer	SNOMED	286889008	Carcinoma of upper limb bones/scapula (disorder)
Cancer	SNOMED	286890004	Carcinoma of lower limb bones (disorder)
Cancer	SNOMED	286893002	Carcinoma of breast - upper, inner quadrant (disorder)
Cancer	SNOMED	286894008	Carcinoma of breast - lower, inner quadrant (disorder)
Cancer	SNOMED	286895009	Carcinoma of breast - upper, outer quadrant (disorder)
Cancer	SNOMED	286896005	Carcinoma breast - lower, outer quadrant (disorder)
Cancer	SNOMED	286897001	Carcinoma of breast - axillary tail (disorder)
Cancer	SNOMED	286899003	Carcinoma of genital organ (disorder)
Cancer	SNOMED	286900008	Carcinoma of epididymis/spermatic cord (disorder)
Cancer	SNOMED	286902000	Secondary carcinoma of gastrointestinal tract (disorder)
Cancer	SNOMED	294301000000103	Bowel cancer detected by national screening programme (disorder)
Cancer	SNOMED	300988009	Transitional cell carcinoma of ureter (disorder)
Cancer	SNOMED	301756000	Adenocarcinoma of sigmoid colon (disorder)
Cancer	SNOMED	302816009	Malignant tumour of soft tissue of head, face and neck
Cancer	SNOMED	302817000	Malignant tumor of unknown origin or ill-defined site
Cancer	SNOMED	302835009	Phaeochromocytoma
Cancer	SNOMED	302837001	Lentigo maligna melanoma (disorder)
Cancer	SNOMED	302841002	Malignant lymphoma - small lymphocytic (disorder)
Cancer	SNOMED	302842009	Diffuse malignant lymphoma - centroblastic
Cancer	SNOMED	302845006	Nodular malignant lymphoma, lymphocytic - well differentiated (disorder)
Cancer	SNOMED	302847003	Rhabdomyosarcoma (disorder)
Cancer	SNOMED	302849000	Nephroblastoma (disorder)
Cancer	SNOMED	302851001	Synovial sarcoma (disorder)
Cancer	SNOMED	302855005	Subacute leukemia
Cancer	SNOMED	303012000	Malignant tumor of posterior wall of hypopharynx (disorder)
Cancer	SNOMED	303017006	Malignant lymphoma, convoluted cell type (disorder)
Cancer	SNOMED	303055001	Malignant lymphoma, follicular center cell (disorder)
Cancer	SNOMED	303057009	Malignant lymphoma, follicular center cell, non-cleaved (disorder)
Cancer	SNOMED	303194003	Metastasis to head and neck lymph node
Cancer	SNOMED	303201005	Metastasis to multiple lymph nodes
Cancer	SNOMED	304545002	Adenocarcinoma of ileum (disorder)
Cancer	SNOMED	30664006	Multiple endocrine neoplasia, type 1 (disorder)
Cancer	SNOMED	307216009	Perforated carcinoma of esophagus (disorder)
Cancer	SNOMED	307219002	Retroperitoneal sarcoma (disorder)
Cancer	SNOMED	307226002	Metastatic adenocarcinoma of unknown origin (disorder)
Cancer	SNOMED	307341004	Atypical hairy cell leukemia
Cancer	SNOMED	307502000	Squamous cell carcinoma of mouth (disorder)
Cancer	SNOMED	307576001	Osteosarcoma of bone
Cancer	SNOMED	307593001	Disseminated carcinomatosis
Cancer	SNOMED	307599002	Sebaceous adenocarcinoma (disorder)
Cancer	SNOMED	307601000	Pseudomyxoma peritonei (disorder)
Cancer	SNOMED	307603002	Malignant blue nevus of skin (disorder)
Cancer	SNOMED	307608006	Ewing's sarcoma of bone (disorder)
Cancer	SNOMED	307609003	Adamantinoma of long bone (disorder)
Cancer	SNOMED	307610008	Pilomatrix carcinoma of skin (disorder)
Cancer	SNOMED	307617006	Neutrophilic leukemia (disorder)
Cancer	SNOMED	307623001	Malignant lymphoma - lymphoplasmacytic (disorder)
Cancer	SNOMED	307635002	Hodgkin's disease, nodular sclerosis - cellular phase (disorder)
Cancer	SNOMED	307636001	Malignant lymphoma, mixed lymphocytic-histiocytic, nodular (disorder)
Cancer	SNOMED	307637005	Malignant lymphoma, centroblastic-centrocytic, follicular (disorder)
Cancer	SNOMED	307646004	Malignant lymphoma, lymphocytic, poorly differentiated, nodular (disorder)
Cancer	SNOMED	307647008	Malignant lymphoma, centroblastic type, follicular (disorder)
Cancer	SNOMED	307649006	Microglioma (disorder)
Cancer	SNOMED	307651005	Myelosclerosis with myeloid metaplasia
Cancer	SNOMED	308121000	Follicular non-Hodgkin's lymphoma
Cancer	SNOMED	309245001	Adenocarcinoma of uterus (disorder)
Cancer	SNOMED	31047003	Lymphomatoid papulosis (disorder)
Cancer	SNOMED	310498001	Malignant melanoma of back
Cancer	SNOMED	310504009	Primary malignant neoplasm of unknown site
Cancer	SNOMED	310526005	Malignant tumor of soft tissue of back (disorder)
Cancer	SNOMED	310599006	Malignant neoplasm of canthus (disorder)
Cancer	SNOMED	312104005	Cholangiocarcinoma of biliary tract (disorder)
Cancer	SNOMED	312111009	Carcinoma of ascending colon (disorder)
Cancer	SNOMED	312112002	Carcinoma of transverse colon (disorder)
Cancer	SNOMED	312113007	Carcinoma of descending colon (disorder)
Cancer	SNOMED	312114001	Carcinoma of hepatic flexure (disorder)
Cancer	SNOMED	312115000	Carcinoma of splenic flexure (disorder)

Cancer	SNOMED	312949007	Retinal pigment epithelial adenocarcinoma (disorder)
Cancer	SNOMED	313248004	Malignant melanoma of chest wall
Cancer	SNOMED	313249007	Malignant neoplasm of upper eyelid (disorder)
Cancer	SNOMED	313250007	Malignant neoplasm of lower eyelid (disorder)
Cancer	SNOMED	313353007	Squamous cell carcinoma of bronchus in left lower lobe (disorder)
Cancer	SNOMED	313354001	Squamous cell carcinoma of bronchus in left upper lobe (disorder)
Cancer	SNOMED	313355000	Squamous cell carcinoma of bronchus in right lower lobe (disorder)
Cancer	SNOMED	313356004	Squamous cell carcinoma of bronchus in right middle lobe (disorder)
Cancer	SNOMED	313357008	Squamous cell carcinoma of bronchus in right upper lobe (disorder)
Cancer	SNOMED	313427003	Lambda light chain myeloma
Cancer	SNOMED	313428008	Seminoma of undescended testis
Cancer	SNOMED	313429000	Seminoma of descended testis
Cancer	SNOMED	314191009	Cystadenocarcinoma of ovary (disorder)
Cancer	SNOMED	314418005	Leukemic infiltrate of retina (disorder)
Cancer	SNOMED	314951005	Local recurrence of malignant tumor of tongue (disorder)
Cancer	SNOMED	314952003	Local recurrence of malignant tumor of buccal cavity (disorder)
Cancer	SNOMED	314953008	Local recurrence of malignant tumor of thyroid gland (disorder)
Cancer	SNOMED	314954002	Local recurrence of malignant tumor of lung (disorder)
Cancer	SNOMED	314955001	Local recurrence of malignant tumor of breast (disorder)
Cancer	SNOMED	314960002	Local recurrence of malignant tumor of esophagus (disorder)
Cancer	SNOMED	314961003	Local recurrence of malignant tumor of stomach (disorder)
Cancer	SNOMED	314962005	Local recurrence of malignant tumor of gallbladder (disorder)
Cancer	SNOMED	314963000	Local recurrence of malignant tumor of liver (disorder)
Cancer	SNOMED	314964006	Local recurrence of malignant tumor of pancreas (disorder)
Cancer	SNOMED	314965007	Local recurrence of malignant tumor of colon (disorder)
Cancer	SNOMED	314966008	Local recurrence of malignant tumor of rectum (disorder)
Cancer	SNOMED	314967004	Local recurrence of malignant tumor of kidney (disorder)
Cancer	SNOMED	314968009	Local recurrence of malignant tumor of urinary bladder (disorder)
Cancer	SNOMED	314969001	Local recurrence of malignant tumor of prostate (disorder)
Cancer	SNOMED	314970000	Local recurrence of malignant tumor of cervix (disorder)
Cancer	SNOMED	314973003	Local recurrence of malignant tumor of bone (disorder)
Cancer	SNOMED	314974009	Local recurrence of malignant tumor of soft tissue (disorder)
Cancer	SNOMED	314976006	Local recurrence of malignant melanoma of skin (disorder)
Cancer	SNOMED	314987003	Metastasis from malignant melanoma of skin (disorder)
Cancer	SNOMED	314988008	Metastasis from malignant tumor of skin (disorder)
Cancer	SNOMED	314989000	Metastasis from malignant tumor of soft tissues (disorder)
Cancer	SNOMED	314990009	Metastasis from malignant tumor of bone (disorder)
Cancer	SNOMED	314991008	Metastasis from malignant tumor of adrenal gland (disorder)
Cancer	SNOMED	314992001	Metastasis from malignant tumor of cervix (disorder)
Cancer	SNOMED	314993006	Metastasis from malignant tumor of uterus (disorder)
Cancer	SNOMED	314994000	Metastasis from malignant tumor of prostate (disorder)
Cancer	SNOMED	314995004	Metastasis from malignant tumor of bladder (disorder)
Cancer	SNOMED	314996003	Metastasis from malignant tumor of kidney (disorder)
Cancer	SNOMED	314997007	Metastasis from malignant tumor of rectum (disorder)
Cancer	SNOMED	314998002	Metastasis from malignant tumor of colon (disorder)
Cancer	SNOMED	314999005	Metastasis from malignant tumor of pancreas (disorder)
Cancer	SNOMED	315000005	Metastasis from malignant tumor of liver (disorder)
Cancer	SNOMED	315001009	Metastasis from malignant tumor of gallbladder (disorder)
Cancer	SNOMED	315002002	Metastasis from malignant tumor of stomach (disorder)
Cancer	SNOMED	315003007	Metastasis from malignant tumor of esophagus (disorder)
Cancer	SNOMED	315004001	Metastasis from malignant tumor of breast (disorder)
Cancer	SNOMED	315005000	Metastasis from malignant tumor of bronchus (disorder)
Cancer	SNOMED	315006004	Metastasis from malignant tumor of lung (disorder)
Cancer	SNOMED	315007008	Metastasis from malignant tumor of thyroid (disorder)
Cancer	SNOMED	315008003	Metastasis from malignant tumor of buccal cavity (disorder)
Cancer	SNOMED	315009006	Metastasis from malignant tumor of tongue (disorder)
Cancer	SNOMED	315058005	Hereditary nonpolyposis colon cancer
Cancer	SNOMED	315601005	Ambulatory chemotherapy (procedure)
Cancer	SNOMED	32278006	Myeloma kidney
Cancer	SNOMED	326072005	Carcinoma of head of pancreas (disorder)
Cancer	SNOMED	342511000000104	Siewert type II adenocarcinoma (disorder)
Cancer	SNOMED	342561000000102	Siewert type III adenocarcinoma (disorder)
Cancer	SNOMED	342571000000109	Siewert type I adenocarcinoma (disorder)
Cancer	SNOMED	354351000119105	Primary malignant neoplasm of left kidney (disorder)
Cancer	SNOMED	354361000119107	Primary malignant neoplasm of right kidney (disorder)
Cancer	SNOMED	35868009	Carcinoid syndrome (disorder)
Cancer	SNOMED	359631009	Acute myeloid leukemia, minimal differentiation, FAB M0 (disorder)
Cancer	SNOMED	359785002	Metastatic malignant neoplasm to dome of urinary bladder (disorder)
Cancer	SNOMED	359987004	Krukenberg tumor (disorder)
Cancer	SNOMED	363348004	Malignant tumour of lip
Cancer	SNOMED	363349007	Malignant tumor of stomach
Cancer	SNOMED	363350007	Malignant tumour of caecum
Cancer	SNOMED	363351006	Malignant tumor of rectum
Cancer	SNOMED	363352004	Malignant tumour of anal canal
Cancer	SNOMED	363353009	Malignant tumor of gallbladder

Cancer	SNOMED	363354003	Malignant tumour of cervix
Cancer	SNOMED	363355002	Malignant tumor of adrenal gland
Cancer	SNOMED	363357005	Malignant tumor of ill-defined site (disorder)
Cancer	SNOMED	363358000	Malignant tumor of lung
Cancer	SNOMED	363359008	Malignant tumor of middle ear (disorder)
Cancer	SNOMED	363360003	Malignant tumor of anterior two-thirds of tongue
Cancer	SNOMED	363363001	Malignant tumor of soft tissue of shoulder (disorder)
Cancer	SNOMED	363364007	Malignant tumor of soft tissue of hip (disorder)
Cancer	SNOMED	363365008	Malignant tumor of soft tissue of thorax
Cancer	SNOMED	363366009	Malignant tumour of soft tissue of pelvis
Cancer	SNOMED	363367000	Malignant tumor of vulva
Cancer	SNOMED	363368005	Carcinoma of body of pancreas (disorder)
Cancer	SNOMED	363369002	Carcinoma of tail of pancreas (disorder)
Cancer	SNOMED	363370001	Malignant neoplasm of mesentery
Cancer	SNOMED	363372009	Malignant tumor of vermilion border of upper lip
Cancer	SNOMED	363373004	Malignant tumour of vermilion border of lower lip
Cancer	SNOMED	363374005	Malignant tumor of commissure of lip
Cancer	SNOMED	363375006	Malignant tumour of tongue
Cancer	SNOMED	363376007	Malignant tumor of base of tongue
Cancer	SNOMED	363377003	Malignant tumour of lingual tonsil
Cancer	SNOMED	363378008	Malignant tumor of major salivary gland
Cancer	SNOMED	363379000	Malignant tumour of parotid gland
Cancer	SNOMED	363380002	Malignant tumor of submandibular gland
Cancer	SNOMED	363381003	Malignant tumour of sublingual gland
Cancer	SNOMED	363382005	Malignant tumor of gum
Cancer	SNOMED	363383000	Malignant tumour of upper gingiva
Cancer	SNOMED	363384006	Malignant tumour of lower gingiva
Cancer	SNOMED	363385007	Malignant tumor of floor of mouth
Cancer	SNOMED	363386008	Malignant tumour of buccal mucosa
Cancer	SNOMED	363387004	Malignant tumor of hard palate
Cancer	SNOMED	363388009	Malignant tumour of soft palate
Cancer	SNOMED	363389001	Malignant tumor of uvula
Cancer	SNOMED	363390005	Malignant tumour of palate
Cancer	SNOMED	363391009	Malignant tumor of retromolar area
Cancer	SNOMED	363392002	Malignant tumour of oropharynx
Cancer	SNOMED	363393007	Malignant tumor of tonsil
Cancer	SNOMED	363394001	Malignant tumour of tonsillar fossa
Cancer	SNOMED	363395000	Malignant tumor of vallecula
Cancer	SNOMED	363396004	Malignant tumor of branchial cleft (disorder)
Cancer	SNOMED	363397008	Malignant tumor of roof of nasopharynx
Cancer	SNOMED	363398003	Malignant tumour of lateral wall of nasopharynx
Cancer	SNOMED	363399006	Malignant tumor of hypopharynx
Cancer	SNOMED	363400004	Malignant tumour of postcricoid region
Cancer	SNOMED	363401000	Malignant tumor of pyriform fossa
Cancer	SNOMED	363402007	Malignant tumour of oesophagus
Cancer	SNOMED	363403002	Malignant tumor of duodenum
Cancer	SNOMED	363404008	Malignant tumour of jejunum
Cancer	SNOMED	363405009	Malignant tumor of ileum
Cancer	SNOMED	363406005	Malignant tumour of colon
Cancer	SNOMED	363407001	Malignant tumor of hepatic flexure
Cancer	SNOMED	363408006	Malignant tumour of transverse colon
Cancer	SNOMED	363409003	Malignant tumor of descending colon
Cancer	SNOMED	363410008	Malignant tumour of sigmoid colon
Cancer	SNOMED	363411007	Malignant tumor of appendix
Cancer	SNOMED	363412000	Malignant tumour of ascending colon
Cancer	SNOMED	363413005	Malignant tumor of splenic flexure
Cancer	SNOMED	363414004	Malignant tumour of rectosigmoid junction
Cancer	SNOMED	363415003	Malignant tumor of biliary tract
Cancer	SNOMED	363416002	Malignant tumour of extrahepatic bile duct
Cancer	SNOMED	363417006	Malignant tumor of ampulla of Vater
Cancer	SNOMED	363418001	Malignant tumour of pancreas
Cancer	SNOMED	363419009	Malignant tumor of head of pancreas
Cancer	SNOMED	363420003	Malignant retroperitoneal tumour
Cancer	SNOMED	363421004	Malignant neoplasm of omentum
Cancer	SNOMED	363422006	Malignant tumor of nasal cavity
Cancer	SNOMED	363423001	Malignant tumour of nasal septum
Cancer	SNOMED	363424007	Malignant tumor of mastoid air cells
Cancer	SNOMED	363425008	Malignant tumour of maxillary sinus
Cancer	SNOMED	363426009	Malignant tumor of ethmoid sinus
Cancer	SNOMED	363427000	Malignant tumour of frontal sinus
Cancer	SNOMED	363428005	Malignant tumor of sphenoid sinus
Cancer	SNOMED	363429002	Malignant tumour of larynx
Cancer	SNOMED	363430007	Malignant tumor of subglottis
Cancer	SNOMED	363431006	Malignant tumour of laryngeal cartilage
Cancer	SNOMED	363432004	Malignant tumor of trachea

Cancer	SNOMED	363433009	Malignant tumour of pleura
Cancer	SNOMED	363434003	Malignant tumor of thymus
Cancer	SNOMED	363435002	Malignant tumour of heart
Cancer	SNOMED	363436001	Malignant tumor of endocardium
Cancer	SNOMED	363438000	Malignant tumor of vertebral column
Cancer	SNOMED	363439008	Malignant tumour of soft tissue of head
Cancer	SNOMED	363440005	Malignant tumor of soft tissue of face
Cancer	SNOMED	363441009	Malignant tumour of soft tissue of neck
Cancer	SNOMED	363443007	Malignant tumour of ovary
Cancer	SNOMED	363444001	Malignant tumor of fallopian tube
Cancer	SNOMED	363445000	Malignant tumour of vagina
Cancer	SNOMED	363446004	Malignant neoplasm of labia majora
Cancer	SNOMED	363447008	Malignant neoplasm of labia minora
Cancer	SNOMED	363449006	Malignant tumour of testis
Cancer	SNOMED	363451005	Malignant tumour of glans penis
Cancer	SNOMED	363452003	Malignant tumor of epididymis
Cancer	SNOMED	363453008	Malignant tumour of spermatic cord
Cancer	SNOMED	363454002	Malignant tumor of scrotum
Cancer	SNOMED	363456000	Malignant tumor of urachus
Cancer	SNOMED	363457009	Malignant tumour of renal pelvis
Cancer	SNOMED	363458004	Malignant tumor of ureter
Cancer	SNOMED	363459007	Malignant tumour of urethra
Cancer	SNOMED	363460002	Malignant tumor of paraurethral gland
Cancer	SNOMED	363461003	Malignant tumour of eye
Cancer	SNOMED	363462005	Malignant tumor of orbit
Cancer	SNOMED	363463000	Malignant tumour of conjunctiva
Cancer	SNOMED	363464006	Malignant tumor of cornea
Cancer	SNOMED	363465007	Malignant tumour of retina
Cancer	SNOMED	363466008	Malignant tumor of choroid
Cancer	SNOMED	363467004	Malignant neoplasm of frontal lobe
Cancer	SNOMED	363468009	Malignant neoplasm of temporal lobe
Cancer	SNOMED	363469001	Malignant neoplasm of parietal lobe
Cancer	SNOMED	363470000	Malignant neoplasm of occipital lobe
Cancer	SNOMED	363471001	Malignant neoplasm of cerebral ventricles
Cancer	SNOMED	363473003	Malignant neoplasm of brainstem
Cancer	SNOMED	363474009	Malignant neoplasm of cerebral meninges
Cancer	SNOMED	363475005	Malignant tumour of spinal cord
Cancer	SNOMED	363476006	Malignant neoplasm of spinal meninges
Cancer	SNOMED	363477002	Malignant neoplasm of cauda equina
Cancer	SNOMED	363478007	Malignant tumor of thyroid gland
Cancer	SNOMED	363481002	Malignant tumour of parathyroid gland
Cancer	SNOMED	363482009	Malignant tumor of pituitary gland
Cancer	SNOMED	363483004	Malignant tumour of pineal gland
Cancer	SNOMED	363484005	Malignant tumor of pelvis
Cancer	SNOMED	363485006	Malignant tumor of minor salivary gland (disorder)
Cancer	SNOMED	363486007	Malignant tumor of vocal cord (disorder)
Cancer	SNOMED	363487003	Malignant tumor of aryepiglottic fold - laryngeal aspect (disorder)
Cancer	SNOMED	363488008	Malignant tumor of false cord (disorder)
Cancer	SNOMED	363489000	Malignant tumour of neck
Cancer	SNOMED	363490009	Malignant tumor of anus
Cancer	SNOMED	363491008	Malignant tumour of cloacogenic zone
Cancer	SNOMED	363492001	Malignant tumor of peritoneum
Cancer	SNOMED	363493006	Malignant tumor of bronchus (disorder)
Cancer	SNOMED	363494000	Malignant tumor of mediastinum
Cancer	SNOMED	363495004	Malignant tumor of muscle (disorder)
Cancer	SNOMED	363496003	Malignant tumor of soft tissue of abdomen (disorder)
Cancer	SNOMED	363497007	Malignant tumour of meninges
Cancer	SNOMED	363498002	Malignant tumor of optic nerve
Cancer	SNOMED	363499005	Malignant tumour of spleen
Cancer	SNOMED	363500001	Multiple malignancy (disorder)
Cancer	SNOMED	363501002	Malignant tumor of face
Cancer	SNOMED	363502009	Malignant tumour of axilla
Cancer	SNOMED	363503004	Malignant tumor of upper limb
Cancer	SNOMED	363504005	Malignant tumour of lower limb
Cancer	SNOMED	363505006	Malignant tumor of oral cavity
Cancer	SNOMED	363506007	Malignant tumour of nasal sinuses
Cancer	SNOMED	363507003	Malignant tumor of pharynx
Cancer	SNOMED	363508008	Malignant tumour of intestine
Cancer	SNOMED	363509000	Malignant tumor of small intestine
Cancer	SNOMED	363510005	Malignant tumor of large intestine (disorder)
Cancer	SNOMED	363514001	Malignant tumour of female genital organ
Cancer	SNOMED	363515000	Malignant tumor of male genital organ
Cancer	SNOMED	363516004	Malignant tumour of penis
Cancer	SNOMED	363517008	Malignant tumor of urinary tract proper (disorder)
Cancer	SNOMED	363518003	Malignant tumour of kidney

Cancer	SNOMED	367336001	Chemotherapy (procedure)
Cancer	SNOMED	370967009	Retinoblastoma (disorder)
Cancer	SNOMED	371964008	Malignant neoplasm of adrenal cortex (disorder)
Cancer	SNOMED	371965009	Malignant neoplasm of adrenal medulla (disorder)
Cancer	SNOMED	371967001	Primary malignant neoplasm of ampulla of Vater (disorder)
Cancer	SNOMED	371972005	Malignant neoplasm of body of uterus (disorder)
Cancer	SNOMED	371973000	Malignant neoplasm of uterus (disorder)
Cancer	SNOMED	371974006	Malignant neoplasm of border of tongue (disorder)
Cancer	SNOMED	371977004	Primary malignant neoplasm of cecum (disorder)
Cancer	SNOMED	371979001	Malignant neoplasm of clitoris (disorder)
Cancer	SNOMED	371982006	Malignant neoplasm of endocrine gland (disorder)
Cancer	SNOMED	371984007	Primary malignant neoplasm of esophagus (disorder)
Cancer	SNOMED	372001002	Primary malignant neoplasm of oral cavity (disorder)
Cancer	SNOMED	372003004	Primary malignant neoplasm of pancreas (disorder)
Cancer	SNOMED	372004005	Primary malignant neoplasm of parotid gland (disorder)
Cancer	SNOMED	372005006	Primary malignant neoplasm of penis (disorder)
Cancer	SNOMED	372011009	Malignant tumor of soft tissue of upper limb (disorder)
Cancer	SNOMED	372014001	Primary malignant neoplasm of stomach (disorder)
Cancer	SNOMED	372016004	Primary malignant neoplasm of the peritoneum (disorder)
Cancer	SNOMED	372020000	Primary malignant neoplasm of tonsil (disorder)
Cancer	SNOMED	372023003	Primary malignant neoplasm of upper third of esophagus (disorder)
Cancer	SNOMED	372030009	Primary malignant neoplasm of vocal cord (disorder)
Cancer	SNOMED	372062007	Malignant neoplasm of central nervous system (disorder)
Cancer	SNOMED	372063002	Malignant neoplasm of nervous system (disorder)
Cancer	SNOMED	372064008	Malignant neoplasm of female breast (disorder)
Cancer	SNOMED	372065009	Malignant neoplasm of main bronchus (disorder)
Cancer	SNOMED	372087000	Primary malignant neoplasm (disorder)
Cancer	SNOMED	372094002	Malignant neoplasm of axillary tail of breast (disorder)
Cancer	SNOMED	372095001	Malignant neoplasm of male breast (disorder)
Cancer	SNOMED	372096000	Carcinoma of male breast (disorder)
Cancer	SNOMED	372097009	Malignant neoplasm of endocervix (disorder)
Cancer	SNOMED	372099007	Malignant neoplasm of exocervix (disorder)
Cancer	SNOMED	372100004	Carcinoma of exocervix (disorder)
Cancer	SNOMED	372101000	Carcinoma of extrahepatic bile duct (disorder)
Cancer	SNOMED	372106005	Carcinoma of penis (disorder)
Cancer	SNOMED	372108006	Malignant neoplasm of bone of lower limb (disorder)
Cancer	SNOMED	372111007	Carcinoma of lower lobe, bronchus or lung (disorder)
Cancer	SNOMED	372113005	Carcinoma of middle lobe, bronchus or lung (disorder)
Cancer	SNOMED	372119009	Primary malignant neoplasm of head of pancreas (disorder)
Cancer	SNOMED	372131006	Malignant neoplasm of upper limb bones and scapula (disorder)
Cancer	SNOMED	372136001	Carcinoma of upper lobe, bronchus or lung (disorder)
Cancer	SNOMED	372137005	Primary malignant neoplasm of breast (disorder)
Cancer	SNOMED	372138000	Carcinoma of esophagus (disorder)
Cancer	SNOMED	372139008	Primary malignant neoplasm of gallbladder (disorder)
Cancer	SNOMED	372140005	Carcinoma of gallbladder (disorder)
Cancer	SNOMED	372141009	Carcinoma of vocal cord (disorder)
Cancer	SNOMED	372142002	Carcinoma of pancreas (disorder)
Cancer	SNOMED	372143007	Carcinoma of stomach (disorder)
Cancer	SNOMED	372244006	Malignant melanoma (disorder)
Cancer	SNOMED	373080008	Malignant neoplasm of breast lower inner quadrant (disorder)
Cancer	SNOMED	373082000	Malignant neoplasm of breast upper inner quadrant (disorder)
Cancer	SNOMED	373083005	Malignant neoplasm of breast upper outer quadrant (disorder)
Cancer	SNOMED	373168002	Reticulosarcoma (disorder)
Cancer	SNOMED	38216008	Infusion chemotherapy for malignant neoplasm
Cancer	SNOMED	393563007	Glioblastoma multiforme (disorder)
Cancer	SNOMED	394894008	Pre-operative chemotherapy (procedure)
Cancer	SNOMED	394895009	Postoperative chemotherapy (procedure)
Cancer	SNOMED	396181000000106	[X]Melanoma and other malignant neoplasms of skin (disorder)
Cancer	SNOMED	397009000	Mast cell malignancy (disorder)
Cancer	SNOMED	39795003	Hand-SchÃ¼ller-Christian disease (disorder)
Cancer	SNOMED	398271008	Predominantly T-cell defect (finding)
Cancer	SNOMED	398623004	Refractory anemia with excess blasts (disorder)
Cancer	SNOMED	398670003	Pigmented dermatofibrosarcoma protuberans of skin (disorder)
Cancer	SNOMED	399042005	Chemotherapy cycle (procedure)
Cancer	SNOMED	399068003	Malignant tumor of prostate (disorder)
Cancer	SNOMED	399326009	Malignant tumor of urinary bladder (disorder)
Cancer	SNOMED	399490008	Adenocarcinoma of prostate (disorder)
Cancer	SNOMED	399660006	Ring melanoma of ciliary body (disorder)
Cancer	SNOMED	400001003	Primary cutaneous lymphoma (disorder)
Cancer	SNOMED	400122007	Primary cutaneous T-cell lymphoma (disorder)
Cancer	SNOMED	402537005	Metastatic basal cell carcinoma (disorder)
Cancer	SNOMED	402558004	Malignant melanoma (vertical growth phase) (disorder)
Cancer	SNOMED	402561003	Malignant melanoma of soft tissues (disorder)
Cancer	SNOMED	402815007	Squamous cell carcinoma (disorder)
Cancer	SNOMED	403900000	Spindle cell squamous cell carcinoma (disorder)

Cancer	SNOMED	403906006	Metastatic squamous cell carcinoma (disorder)
Cancer	SNOMED	403923002	Spindle cell malignant melanoma (disorder)
Cancer	SNOMED	403924008	Desmoplastic malignant melanoma (disorder)
Cancer	SNOMED	403927001	Malignant melanoma of nail apparatus (disorder)
Cancer	SNOMED	403977003	Angiosarcoma (disorder)
Cancer	SNOMED	404037002	Malignant peripheral nerve sheath tumor (disorder)
Cancer	SNOMED	404051002	Embryonal rhabdomyosarcoma (disorder)
Cancer	SNOMED	404071006	Pleomorphic liposarcoma (disorder)
Cancer	SNOMED	404133000	Subcutaneous panniculitic cutaneous T-cell lymphoma (disorder)
Cancer	SNOMED	404149003	Lymphoplasmacytic B-cell lymphoma, nodal/systemic with skin involvement (disorder)
Cancer	SNOMED	405546008	Malignant pericardial effusion (disorder)
Cancer	SNOMED	405843009	Widespread metastatic malignant neoplastic disease (disorder)
Cancer	SNOMED	405945003	Malignant neoplasm of metatarsal bone of foot (disorder)
Cancer	SNOMED	408642003	Transitional cell carcinoma of kidney (disorder)
Cancer	SNOMED	408643008	Infiltrating duct carcinoma of breast (disorder)
Cancer	SNOMED	408645001	Adenocarcinoma of large intestine (disorder)
Cancer	SNOMED	408646000	Adenocarcinoma of liver (disorder)
Cancer	SNOMED	408647009	Adenocarcinoma of stomach (disorder)
Cancer	SNOMED	409231000000104	[X]Malignant neoplasm of mesothelial and soft tissue (disorder)
Cancer	SNOMED	413441006	Acute monocytic leukemia, FAB M5b (disorder)
Cancer	SNOMED	413445002	Adenocarcinoma of appendix (disorder)
Cancer	SNOMED	413446001	Adenocarcinoma of cecum (disorder)
Cancer	SNOMED	413537009	Angioimmunoblastic T-cell lymphoma (disorder)
Cancer	SNOMED	413587002	Smoldering myeloma (disorder)
Cancer	SNOMED	413738001	Cancer monitoring first letter (procedure)
Cancer	SNOMED	413739009	Cancer monitoring invitation (procedure)
Cancer	SNOMED	413740006	Cancer monitoring second letter (procedure)
Cancer	SNOMED	413741005	Cancer monitoring third letter (procedure)
Cancer	SNOMED	413842007	Chronic myeloid leukemia in lymphoid blast crisis (disorder)
Cancer	SNOMED	413843002	Chronic myeloid leukemia in myeloid blast crisis (disorder)
Cancer	SNOMED	413847001	Chronic phase chronic myeloid leukemia (disorder)
Cancer	SNOMED	414166008	Extranodal natural killer/T-cell lymphoma, nasal type (disorder)
Cancer	SNOMED	414553000	Kappa light chain myeloma (disorder)
Cancer	SNOMED	414785000	Multiple solitary plasmacytomas (disorder)
Cancer	SNOMED	415082004	Personal history of primary malignant neoplasm of lung (context-dependent category)
Cancer	SNOMED	415110002	Plasma cell myeloma/plasmacytoma (disorder)
Cancer	SNOMED	415112005	Plasmacytoma (disorder)
Cancer	SNOMED	416402001	Gestational trophoblastic disease (disorder)
Cancer	SNOMED	416669000	Invasive hydatidiform mole (disorder)
Cancer	SNOMED	416712009	Clear cell (mesonephric) neoplasm of ovary (disorder)
Cancer	SNOMED	416769008	Malignant teratoma of testis (disorder)
Cancer	SNOMED	416842003	Malignant sacral teratoma (disorder)
Cancer	SNOMED	416901002	Malignant medulloepithelioma of ciliary body (disorder)
Cancer	SNOMED	417036008	Liquid based cervical cytology screening (procedure)
Cancer	SNOMED	417084004	Cancer monitoring verbal invitation (procedure)
Cancer	SNOMED	417417007	Malignant teratoma of undescended testis (disorder)
Cancer	SNOMED	417554000	Malignant teratoma of descended testis (disorder)
Cancer	SNOMED	417570003	Gestational choriocarcinoma (disorder)
Cancer	SNOMED	419052002	Malignant tumor of urinary system (disorder)
Cancer	SNOMED	420788006	Intraocular non-Hodgkin malignant lymphoma (disorder)
Cancer	SNOMED	421249001	Malignant tumor of vermilion border of lip (disorder)
Cancer	SNOMED	422599000	Squamous cell carcinoma of back (disorder)
Cancer	SNOMED	422691006	Squamous cell carcinoma of nasopharynx (disorder)
Cancer	SNOMED	423158009	Hurthle cell carcinoma of thyroid (disorder)
Cancer	SNOMED	423464009	Squamous cell carcinoma of oropharynx (disorder)
Cancer	SNOMED	423746001	Adenocarcinoma of pelvis (disorder)
Cancer	SNOMED	423973006	Carcinoma of uterine cervix, invasive (disorder)
Cancer	SNOMED	424151006	Anaplastic glioma of brain (disorder)
Cancer	SNOMED	424413001	Sarcoma (disorder)
Cancer	SNOMED	424952003	Sarcoma of soft tissue (disorder)
Cancer	SNOMED	425066001	Carcinoma of urinary bladder, invasive (disorder)
Cancer	SNOMED	425111000000106	[X]Additional neoplasm classification terms (disorder)
Cancer	SNOMED	425178004	Adenocarcinoma of rectosigmoid junction (disorder)
Cancer	SNOMED	425231005	Carcinoma of urinary bladder, superficial (disorder)
Cancer	SNOMED	426071002	Hodgkin's disease in remission (disorder)
Cancer	SNOMED	426202004	Immune reconstitution syndrome (disorder)
Cancer	SNOMED	426336007	Solitary osseous myeloma (disorder)
Cancer	SNOMED	426964009	Non-small cell lung cancer, positive for epidermal growth factor receptor expression (disorder)
Cancer	SNOMED	427141003	Malignant lymphoma in remission (disorder)
Cancer	SNOMED	427492003	Hormone refractory prostate cancer (disorder)
Cancer	SNOMED	427685000	Human epidermal growth factor 2 positive carcinoma of breast (disorder)
Cancer	SNOMED	428061005	Malignant neoplasm of brain (disorder)

Cancer	SNOMED	428100006	Malignant neoplasm of thoracic cavity structure (disorder)
Cancer	SNOMED	428281000	Malignant neoplasm of bone (disorder)
Cancer	SNOMED	428322007	Malignant neoplasm of uterine adnexa (disorder)
Cancer	SNOMED	428905002	Malignant neoplasm of gastrointestinal tract (disorder)
Cancer	SNOMED	429014004	History of malignant lymphoma (situation)
Cancer	SNOMED	429033009	Malignant neoplasm of cerebrum (disorder)
Cancer	SNOMED	430556008	Malignant neoplasm of genital structure (disorder)
Cancer	SNOMED	430621000	Malignant neoplasm of lower respiratory tract (disorder)
Cancer	SNOMED	431396003	Human epidermal growth factor 2 negative carcinoma of breast (disorder)
Cancer	SNOMED	440422002	Asymptomatic multiple myeloma (disorder)
Cancer	SNOMED	441313008	Indolent multiple myeloma (disorder)
Cancer	SNOMED	443144000	Metastatic sarcoma (disorder)
Cancer	SNOMED	443487006	Mantle cell lymphoma (disorder)
Cancer	SNOMED	443488001	Malignant neoplasm of anorectum (disorder)
Cancer	SNOMED	443493003	Metastatic malignant melanoma (disorder)
Cancer	SNOMED	443520009	Chondrosarcoma (disorder)
Cancer	SNOMED	443675005	Seminoma (disorder)
Cancer	SNOMED	443679004	Malignant neoplasm of skeletal system (disorder)
Cancer	SNOMED	443719001	Leiomyosarcoma (disorder)
Cancer	SNOMED	443961001	Malignant adenomatous neoplasm (disorder)
Cancer	SNOMED	444604002	Carcinoma of breast with ductal and lobular features (disorder)
Cancer	SNOMED	444910004	Primary mediastinal (thymic) large B-cell lymphoma (disorder)
Cancer	SNOMED	445105005	Blastic plasmacytoid dendritic cell neoplasm (disorder)
Cancer	SNOMED	445227008	Juvenile myelomonocytic leukemia (disorder)
Cancer	SNOMED	445238008	Malignant carcinoid tumor (disorder)
Cancer	SNOMED	445269007	Extranodal marginal zone B-cell lymphoma of mucosa-associated lymphoid tissue (disorder)
Cancer	SNOMED	445406001	Hepatosplenic T-cell lymphoma (disorder)
Cancer	SNOMED	445448008	Acute myeloid leukemia with myelodysplasia-related changes (disorder)
Cancer	SNOMED	445738007	Myelodysplastic/myeloproliferative disease (disorder)
Cancer	SNOMED	446022000	Malignant epithelial neoplasm of uterus (disorder)
Cancer	SNOMED	446643000	Sarcoma of dendritic cells (accessory cells) (disorder)
Cancer	SNOMED	447706001	Leiomyosarcoma of scalp (disorder)
Cancer	SNOMED	447882007	Malignant epithelial neoplasm of vulva (disorder)
Cancer	SNOMED	447883002	Malignant neoplasm of carotid body (disorder)
Cancer	SNOMED	447949005	Malignant epithelial neoplasm of cheek (disorder)
Cancer	SNOMED	448212009	Anaplastic lymphoma kinase negative anaplastic large cell lymphoma (disorder)
Cancer	SNOMED	448216007	Malignant epithelial neoplasm of thyroid (disorder)
Cancer	SNOMED	448233000	Malignant neoplasm of urinary organ (disorder)
Cancer	SNOMED	448668007	Malignant neoplasm of mandible (disorder)
Cancer	SNOMED	448670003	Malignant neoplasm of posterior mediastinum (disorder)
Cancer	SNOMED	448674007	Malignant neoplasm of parametrium (disorder)
Cancer	SNOMED	448868009	Malignant neoplasm of lateral wall of oropharynx (disorder)
Cancer	SNOMED	448882009	Malignant neoplasm of intraabdominal organ (disorder)
Cancer	SNOMED	448930008	Squamous cell carcinoma of nose (disorder)
Cancer	SNOMED	448931007	Squamous cell carcinoma of shoulder (disorder)
Cancer	SNOMED	448952004	Infiltrating duct carcinoma of female breast (disorder)
Cancer	SNOMED	448992002	Malignant epithelial neoplasm of appendix (disorder)
Cancer	SNOMED	448993007	Malignant epithelial neoplasm of lung (disorder)
Cancer	SNOMED	448994001	Malignant epithelial neoplasm of upper rectum (disorder)
Cancer	SNOMED	449066004	Malignant neoplasm of upper respiratory tract (disorder)
Cancer	SNOMED	449067008	Malignant neoplasm of parietal pleura (disorder)
Cancer	SNOMED	449077005	Malignant epithelial neoplasm of maxilla (disorder)
Cancer	SNOMED	449096009	Malignant neoplasm of respiratory system (disorder)
Cancer	SNOMED	449101009	Sarcoma of sternum (disorder)
Cancer	SNOMED	449108003	Philadelphia chromosome positive chronic myelogenous leukemia (disorder)
Cancer	SNOMED	449153001	Adenocarcinoma of lower esophagus (disorder)
Cancer	SNOMED	449220000	Diffuse follicle center lymphoma (disorder)
Cancer	SNOMED	449224009	Malignant neoplasm of anterior mediastinum (disorder)
Cancer	SNOMED	449248000	Nasopharyngeal carcinoma (disorder)
Cancer	SNOMED	449308006	Malignant neoplasm of visceral pleura (disorder)
Cancer	SNOMED	449377002	Malignant neoplasm of pelvic peritoneum (disorder)
Cancer	SNOMED	449386007	Philadelphia chromosome negative chronic myelogenous leukemia (disorder)
Cancer	SNOMED	449420002	Malignant neoplasm of cerebellum (disorder)
Cancer	SNOMED	449472007	Malignant epithelial neoplasm of alveolus dentalis (disorder)
Cancer	SNOMED	449578008	Malignant neoplasm of alveolus of mandible (disorder)
Cancer	SNOMED	449627008	Malignant neoplasm of long bone of lower limb (disorder)
Cancer	SNOMED	449635006	Primary malignant neoplasm of lower leg (disorder)
Cancer	SNOMED	45221000119105	Primary invasive malignant neoplasm of female breast (disorder)
Cancer	SNOMED	462401000000104	[X]Malignant neoplasms of lymphoid, haematopoietic and related tissue (disorder)
Cancer	SNOMED	462461000000100	[X]Malignant neoplasm of female genital organs (disorder)
Cancer	SNOMED	53523003	Poisoning caused by cactinomycin (disorder)
Cancer	SNOMED	58961005	Lethal midline granuloma (disorder)

Cancer	SNOMED	62497000	Stewart-Treves syndrome (disorder)
Cancer	SNOMED	65399007	Langerhans cell histiocytosis (disorder)
Cancer	SNOMED	67821000119109	Primary small cell malignant neoplasm of lung, TNM stage 2 (disorder)
Cancer	SNOMED	681601000119101	Primary adenocarcinoma of ascending colon (disorder)
Cancer	SNOMED	683991000119103	Extensive stage primary small cell carcinoma of lung (disorder)
Cancer	SNOMED	684911000119105	Primary glioblastoma multiforme of frontal lobe (disorder)
Cancer	SNOMED	68979007	Heavy chain disease (disorder)
Cancer	SNOMED	698040004	Malignant melanoma of nasal cavity (disorder)
Cancer	SNOMED	699005005	History of malignant hematologic neoplasm
Cancer	SNOMED	699357004	Low grade endometrial stromal sarcoma (disorder)
Cancer	SNOMED	699818003	T-cell large granular lymphocytic leukemia (disorder)
Cancer	SNOMED	700423003	Adenocarcinoma of pancreas (disorder)
Cancer	SNOMED	702369008	Carcinosarcoma of uterus (disorder)
Cancer	SNOMED	702391001	Renal cell carcinoma
Cancer	SNOMED	702392008	Metastatic renal cell carcinoma (disorder)
Cancer	SNOMED	703423002	Combined chemotherapy and radiation therapy (procedure)
Cancer	SNOMED	706970001	Triple negative malignant neoplasm of breast (disorder)
Cancer	SNOMED	707357005	Primary squamous cell carcinoma of laryngeal cartilage (disorder)
Cancer	SNOMED	707358000	Primary squamous cell carcinoma of larynx (disorder)
Cancer	SNOMED	707422006	Primary spindle cell squamous cell carcinoma of larynx (disorder)
Cancer	SNOMED	707452003	Primary mucinous adenocarcinoma of lung (disorder)
Cancer	SNOMED	707456000	Primary undifferentiated carcinoma of lung (disorder)
Cancer	SNOMED	707492001	Primary squamous cell carcinoma of hypopharynx (disorder)
Cancer	SNOMED	707538002	Primary squamous cell carcinoma of vallecula (disorder)
Cancer	SNOMED	707582006	Primary spindle cell squamous cell carcinoma of oropharynx (disorder)
Cancer	SNOMED	707664003	Primary squamous cell carcinoma of glottis (disorder)
Cancer	SNOMED	708921005	Carcinoma of central portion of breast (disorder)
Cancer	SNOMED	709830006	Malignant carcinoid tumor of stomach (disorder)
Cancer	SNOMED	71111000119109	Recurrent primary malignant neoplasm of vulva (disorder)
Cancer	SNOMED	711414003	Primary clear cell adenocarcinoma of lung (disorder)
Cancer	SNOMED	712525007	Malignant neoplasm of short bone of lower limb
Cancer	SNOMED	712750007	Malignant neoplasm of chest wall
Cancer	SNOMED	712849003	Primary malignant neoplasm of prostate metastatic to bone (disorder)
Cancer	SNOMED	713609000	Invasive carcinoma of breast (disorder)
Cancer	SNOMED	716649003	Extraovarian primary peritoneal carcinoma (disorder)
Cancer	SNOMED	716659002	Squamous cell carcinoma of head and neck (disorder)
Cancer	SNOMED	716872004	Antineoplastic chemotherapy regimen (regime/therapy)
Cancer	SNOMED	717735006	Renal cell carcinoma of kidney except renal pelvis (disorder)
Cancer	SNOMED	719052006	Recurrent squamous cell carcinoma (disorder)
Cancer	SNOMED	71985006	Carcinomatous myopathic syndrome (disorder)
Cancer	SNOMED	720006006	Cancer care review (procedure)
Cancer	SNOMED	721304007	Refractory thrombocytopenia (disorder)
Cancer	SNOMED	721567004	Primary malignant neoplasm of placenta (disorder)
Cancer	SNOMED	721575005	Primary angiosarcoma of heart (disorder)
Cancer	SNOMED	721618006	Primary squamous cell carcinoma of upper third of esophagus (disorder)
Cancer	SNOMED	721619003	Primary squamous cell carcinoma of middle third of esophagus (disorder)
Cancer	SNOMED	721628002	Primary adenocarcinoma of esophagogastric junction (disorder)
Cancer	SNOMED	721629005	Linitis plastica of stomach (disorder)
Cancer	SNOMED	721636006	Primary malignant neuroendocrine neoplasm of body of stomach (disorder)
Cancer	SNOMED	721638007	Primary neuroendocrine carcinoma of stomach (disorder)
Cancer	SNOMED	721696009	Primary adenocarcinoma of transverse colon (disorder)
Cancer	SNOMED	721716004	Primary cholangiocarcinoma of intrahepatic biliary tract (disorder)
Cancer	SNOMED	722103009	Hormone sensitive prostate cancer (disorder)
Cancer	SNOMED	722482005	Doxorubicin, bleomycin, vinblastine and dacarbazine chemotherapy regimen (regime/therapy)
Cancer	SNOMED	722524005	Primary invasive pleomorphic lobular carcinoma of breast (disorder)
Cancer	SNOMED	722528008	Primary malignant neuroendocrine neoplasm of lung (disorder)
Cancer	SNOMED	722542000	Primary squamous cell carcinoma of anal canal (disorder)
Cancer	SNOMED	722672002	Primary squamous cell carcinoma of base of tongue (disorder)
Cancer	SNOMED	722682001	Primary small cell carcinoma of endometrium (disorder)
Cancer	SNOMED	722685004	Primary high grade serous adenocarcinoma of ovary (disorder)
Cancer	SNOMED	723265000	Primary squamous cell carcinoma of anus (disorder)
Cancer	SNOMED	723301009	Squamous non-small cell lung cancer (disorder)
Cancer	SNOMED	724059003	Malignant neoplasm of lower lobe of left lung (disorder)
Cancer	SNOMED	724060008	Malignant neoplasm of right upper lobe of lung (disorder)
Cancer	SNOMED	724649000	Langerhans cell sarcoma (disorder)
Cancer	SNOMED	726654006	Malignant carcinoid tumor of colon (disorder)
Cancer	SNOMED	733343005	Primary squamous cell carcinoma of oral cavity (disorder)
Cancer	SNOMED	733361001	Primary mucinous adenocarcinoma of ovary (disorder)
Cancer	SNOMED	733608000	Papillary renal cell carcinoma (disorder)
Cancer	SNOMED	735735001	Primary malignant neuroendocrine neoplasm of pancreas (disorder)
Cancer	SNOMED	736322001	Pediatric follicular lymphoma (disorder)
Cancer	SNOMED	737310005	Primary squamous cell carcinoma of submandibular gland (disorder)
Cancer	SNOMED	738770003	Anaplastic lymphoma kinase positive anaplastic large cell lymphoma (disorder)

Cancer	SNOMED	762458004	Primary endometrioid carcinoma of endometrium of body of uterus (disorder)
Cancer	SNOMED	762690000	Classical Hodgkin lymphoma (disorder)
Cancer	SNOMED	763477007	Primary lymphoma of conjunctiva (disorder)
Cancer	SNOMED	763666008	Splenic marginal zone B-cell lymphoma (disorder)
Cancer	SNOMED	764694005	MiT family translocation renal cell carcinoma (disorder)
Cancer	SNOMED	766979005	Squamous cell carcinoma of rectum (disorder)
Cancer	SNOMED	79268002	Polyneuropathy, organomegaly, endocrinopathy, monoclonal gammopathy, and skin changes syndrome (disorder)
Cancer	SNOMED	80914001	Anaplasia of cervix (disorder)
Cancer	SNOMED	813671000000107	Secondary malignant neoplasm of liver and intrahepatic bile duct (disorder)
Cancer	SNOMED	815361000000107	Acute myeloid leukaemia with 11q23 abnormality (disorder)
Cancer	SNOMED	820601000000103	Refractory anaemia with multilineage dysplasia (disorder)
Cancer	SNOMED	829591000000101	Neoadjuvant chemotherapy (procedure)
Cancer	SNOMED	83270006	Neoplastic pleural effusion (disorder)
Cancer	SNOMED	843671000000104	Electrochemotherapy (procedure)
Cancer	SNOMED	847481000000109	Follicular lymphoma grade 1 (disorder)
Cancer	SNOMED	847631000000107	Follicular lymphoma grade 2 (disorder)
Cancer	SNOMED	847651000000100	Follicular lymphoma grade 3 (disorder)
Cancer	SNOMED	847691000000108	Follicular lymphoma grade 3a (disorder)
Cancer	SNOMED	847701000000108	Follicular lymphoma grade 3b (disorder)
Cancer	SNOMED	847741000000106	Diffuse large B-cell lymphoma (disorder)
Cancer	SNOMED	863741000000108	Clinical stage A chronic lymphocytic leukaemia (disorder)
Cancer	SNOMED	863761000000109	Clinical stage B chronic lymphocytic leukaemia (disorder)
Cancer	SNOMED	863781000000100	Clinical stage C chronic lymphocytic leukaemia (disorder)
Cancer	SNOMED	87776000	Intra-arterial infusion of antineoplastic agent (procedure)
Cancer	SNOMED	884601000000103	Bowel scope (flexible sigmoidoscopy) screen: cancer detected (finding)
Cancer	SNOMED	91151000119103	Primary squamous cell carcinoma of chest wall (disorder)
Cancer	SNOMED	91281000119103	Secondary adenocarcinoma of bone (disorder)
Cancer	SNOMED	91855006	Acute leukemia
Cancer	SNOMED	91857003	Acute lymphoid leukemia
Cancer	SNOMED	91861009	Acute myeloid leukemia
Cancer	SNOMED	92754004	Carcinoma in situ of spleen (disorder)
Cancer	SNOMED	92767001	Carcinoma in situ of thyroid gland (disorder)
Cancer	SNOMED	92812005	Chronic leukemia
Cancer	SNOMED	92814006	Chronic lymphoid leukemia
Cancer	SNOMED	92818009	Chronic myeloid leukemia
Cancer	SNOMED	93133006	Letterer-Siwe disease of intra-abdominal lymph nodes (disorder)
Cancer	SNOMED	93135004	Letterer-Siwe disease of intrathoracic lymph nodes
Cancer	SNOMED	93137007	Letterer-Siwe disease of lymph nodes of head, face AND/OR neck (disorder)
Cancer	SNOMED	93138002	Letterer-Siwe disease of lymph nodes of inguinal region AND/OR lower limb (disorder)
Cancer	SNOMED	93139005	Letterer-Siwe disease of lymph nodes of multiple sites
Cancer	SNOMED	93140007	Letterer-Siwe disease of spleen (disorder)
Cancer	SNOMED	93143009	Leukaemia
Cancer	SNOMED	93146001	Leukemic reticuloendotheliosis of intrathoracic lymph nodes
Cancer	SNOMED	93182006	Malignant histiocytosis of intra-abdominal lymph nodes
Cancer	SNOMED	93183001	Malignant histiocytosis of intrapelvic lymph nodes (disorder)
Cancer	SNOMED	93184007	Malignant histiocytosis of intrathoracic lymph nodes (disorder)
Cancer	SNOMED	93188005	Malignant histiocytosis of lymph nodes of multiple sites (disorder)
Cancer	SNOMED	93189002	Malignant histiocytosis of spleen (disorder)
Cancer	SNOMED	93191005	Malignant lymphoma of intra-abdominal lymph nodes
Cancer	SNOMED	93192003	Malignant lymphoma of intrapelvic lymph nodes
Cancer	SNOMED	93193008	Malignant lymphoma of intrathoracic lymph nodes
Cancer	SNOMED	93194002	Malignant lymphoma of lymph nodes of axilla AND/OR upper limb
Cancer	SNOMED	93195001	Malignant lymphoma of lymph nodes of head, face AND/OR neck
Cancer	SNOMED	93196000	Malignant lymphoma of lymph nodes of inguinal region AND/OR lower limb
Cancer	SNOMED	93197009	Malignant lymphoma of lymph nodes of multiple sites
Cancer	SNOMED	93198004	Malignant lymphoma of spleen
Cancer	SNOMED	93209006	Malignant melanoma of perianal skin (disorder)
Cancer	SNOMED	93210001	Malignant melanoma of skin of abdomen (disorder)
Cancer	SNOMED	93217003	Malignant melanoma of skin of cheek
Cancer	SNOMED	93223008	Malignant melanoma of skin of eyebrow
Cancer	SNOMED	93225001	Malignant melanoma of skin of face
Cancer	SNOMED	93230002	Malignant melanoma of skin of groin (disorder)
Cancer	SNOMED	93451002	Erythroleukaemia
Cancer	SNOMED	93493001	Hodgkin's disease, lymphocytic-histiocytic predominance of intra-abdominal lymph nodes
Cancer	SNOMED	93494007	Hodgkin's disease, lymphocytic-histiocytic predominance of intrapelvic lymph nodes
Cancer	SNOMED	93495008	Hodgkin's disease, lymphocytic-histiocytic predominance of intrathoracic lymph nodes
Cancer	SNOMED	93518009	Hodgkin's disease, nodular sclerosis of spleen (disorder)
Cancer	SNOMED	93520007	Hodgkin's disease of intra-abdominal lymph nodes (disorder)
Cancer	SNOMED	93521006	Hodgkin's disease of intrapelvic lymph nodes (disorder)

Cancer	SNOMED	93522004	Hodgkin's disease of intrathoracic lymph nodes
Cancer	SNOMED	93523009	Hodgkin's disease of lymph nodes of axilla AND/OR upper limb
Cancer	SNOMED	93524003	Hodgkin's disease of lymph nodes of head, face AND/OR neck
Cancer	SNOMED	93525002	Hodgkin's disease of lymph nodes of inguinal region AND/OR lower limb
Cancer	SNOMED	93526001	Hodgkin's disease of lymph nodes of multiple sites
Cancer	SNOMED	93527005	Hodgkin's disease of spleen
Cancer	SNOMED	93531004	Hodgkin's granuloma of intrathoracic lymph nodes (disorder)
Cancer	SNOMED	93536009	Hodgkin's granuloma of spleen (disorder)
Cancer	SNOMED	93541001	Hodgkin's paragranuloma of lymph nodes of axilla AND/OR upper limb (disorder)
Cancer	SNOMED	93542008	Hodgkin's paragranuloma of lymph nodes of head, face AND/OR neck
Cancer	SNOMED	93545005	Hodgkin's paragranuloma of spleen (disorder)
Cancer	SNOMED	93547002	Hodgkin's sarcoma of intra-abdominal lymph nodes (disorder)
Cancer	SNOMED	93549004	Hodgkin's sarcoma of intrathoracic lymph nodes (disorder)
Cancer	SNOMED	93643005	Malignant melanoma of skin of nose
Cancer	SNOMED	93647006	Malignant melanoma of skin of shoulder (disorder)
Cancer	SNOMED	93651008	Malignant melanoma of skin of trunk
Cancer	SNOMED	93655004	Malignant melanoma of skin
Cancer	SNOMED	93661001	Primary malignant neoplasm of acromion (disorder)
Cancer	SNOMED	93665005	Primary malignant neoplasm of adrenal gland (disorder)
Cancer	SNOMED	93669004	Primary malignant neoplasm of anal canal (disorder)
Cancer	SNOMED	93676009	Primary malignant neoplasm of anus (disorder)
Cancer	SNOMED	93677000	Malignant neoplasm of aortic body
Cancer	SNOMED	93679002	Primary malignant neoplasm of appendix (disorder)
Cancer	SNOMED	93680004	Malignant neoplasm of areola of female breast
Cancer	SNOMED	93681000	Malignant neoplasm of areola of male breast
Cancer	SNOMED	93683002	Primary malignant neoplasm of ascending colon (disorder)
Cancer	SNOMED	93687001	Primary malignant neoplasm of base of tongue (disorder)
Cancer	SNOMED	93689003	Primary malignant neoplasm of bladder (disorder)
Cancer	SNOMED	93725000	Malignant neoplasm of bone
Cancer	SNOMED	93727008	Primary malignant neoplasm of brain (disorder)
Cancer	SNOMED	93728003	Malignant neoplasm of broad ligament
Cancer	SNOMED	93734005	Primary malignant neoplasm of bronchus (disorder)
Cancer	SNOMED	93745008	Primary malignant neoplasm of central portion of female breast (disorder)
Cancer	SNOMED	93746009	Malignant neoplasm of cerebellum
Cancer	SNOMED	93753000	Primary malignant neoplasm of cheek (disorder)
Cancer	SNOMED	93757004	Malignant neoplasm of clavicle
Cancer	SNOMED	93759001	Malignant neoplasm of coccygeal body
Cancer	SNOMED	93761005	Primary malignant neoplasm of colon (disorder)
Cancer	SNOMED	93763008	Malignant neoplasm of common bile duct
Cancer	SNOMED	93769007	Primary malignant neoplasm of cuboid (disorder)
Cancer	SNOMED	93770008	Malignant neoplasm of cystic duct
Cancer	SNOMED	93771007	Primary malignant neoplasm of descending colon (disorder)
Cancer	SNOMED	93772000	Malignant neoplasm of diaphragm
Cancer	SNOMED	93775003	Primary malignant neoplasm of duodenum (disorder)
Cancer	SNOMED	93779009	Malignant neoplasm of endocervix
Cancer	SNOMED	93781006	Primary malignant neoplasm of endometrium (disorder)
Cancer	SNOMED	93782004	Primary malignant neoplasm of epicardium (disorder)
Cancer	SNOMED	93784003	Primary malignant neoplasm of epiglottis (disorder)
Cancer	SNOMED	93786001	Malignant neoplasm of ethmoid bone
Cancer	SNOMED	93789008	Malignant neoplasm of exocervix
Cancer	SNOMED	93796005	Primary malignant neoplasm of female breast (disorder)
Cancer	SNOMED	93798006	Malignant neoplasm of femur
Cancer	SNOMED	93799003	Malignant neoplasm of fibula
Cancer	SNOMED	93806005	Malignant neoplasm of frontal bone
Cancer	SNOMED	93814004	Malignant neoplasm of glomus jugulare
Cancer	SNOMED	93817006	Malignant neoplasm of great vessels
Cancer	SNOMED	93827000	Malignant neoplasm of hilus of lung
Cancer	SNOMED	93833009	Malignant neoplasm of ilium
Cancer	SNOMED	93841009	Malignant neoplasm of intrathoracic organs
Cancer	SNOMED	93842002	Malignant neoplasm of ischium
Cancer	SNOMED	93849006	Primary malignant neoplasm of kidney (disorder)
Cancer	SNOMED	93870000	Malignant neoplasm of liver
Cancer	SNOMED	93880001	Malignant neoplasm of lung
Cancer	SNOMED	93884005	Malignant neoplasm of male breast
Cancer	SNOMED	93888008	Malignant neoplasm of maxilla
Cancer	SNOMED	93889000	Primary malignant neoplasm of maxillary sinus (disorder)
Cancer	SNOMED	93916003	Malignant neoplasm of nasal bone
Cancer	SNOMED	93924008	Malignant neoplasm of nipple of female breast
Cancer	SNOMED	93925009	Malignant neoplasm of nipple of male breast
Cancer	SNOMED	93927001	Malignant neoplasm of occipital bone
Cancer	SNOMED	93933005	Primary malignant neoplasm of oropharynx (disorder)
Cancer	SNOMED	93934004	Primary malignant neoplasm of ovary (disorder)
Cancer	SNOMED	93941005	Malignant neoplasm of paraganglion
Cancer	SNOMED	93945001	Malignant neoplasm of parietal bone

Cancer	SNOMED	93947009	Malignant neoplasm of parietal peritoneum
Cancer	SNOMED	93950007	Primary malignant neoplasm of patella (disorder)
Cancer	SNOMED	93952004	Malignant neoplasm of pelvic peritoneum
Cancer	SNOMED	93955002	Primary malignant neoplasm of periadrenal tissue (disorder)
Cancer	SNOMED	93956001	Primary malignant neoplasm of perianal skin (disorder)
Cancer	SNOMED	93957005	Malignant neoplasm of pericardium
Cancer	SNOMED	93961004	Primary malignant neoplasm of pharynx (disorder)
Cancer	SNOMED	93973004	Malignant neoplasm of presacral region
Cancer	SNOMED	93974005	Primary malignant neoplasm of prostate (disorder)
Cancer	SNOMED	93975006	Malignant neoplasm of pubis
Cancer	SNOMED	93979000	Malignant neoplasm of radius
Cancer	SNOMED	93980002	Primary malignant neoplasm of rectosigmoid junction (disorder)
Cancer	SNOMED	93984006	Primary malignant neoplasm of rectum (disorder)
Cancer	SNOMED	93985007	Primary malignant neoplasm of renal pelvis (disorder)
Cancer	SNOMED	93986008	Malignant neoplasm of respiratory tract
Cancer	SNOMED	93990005	Malignant neoplasm of rib
Cancer	SNOMED	93995000	Primary malignant neoplasm of sacrococcygeal region (disorder)
Cancer	SNOMED	93997008	Malignant neoplasm of scapula
Cancer	SNOMED	94000008	Primary malignant neoplasm of sebaceous gland (disorder)
Cancer	SNOMED	94006002	Primary malignant neoplasm of sigmoid colon (disorder)
Cancer	SNOMED	94066004	Malignant neoplasm of sphenoid bone
Cancer	SNOMED	94073009	Malignant neoplasm of sternum
Cancer	SNOMED	94081005	Primary malignant neoplasm of sweat gland (disorder)
Cancer	SNOMED	94083008	Malignant neoplasm of talus
Cancer	SNOMED	94085001	Malignant neoplasm of temporal bone
Cancer	SNOMED	94087009	Primary malignant neoplasm of testis (disorder)
Cancer	SNOMED	94096009	Primary malignant neoplasm of thymus (disorder)
Cancer	SNOMED	94098005	Primary malignant neoplasm of thyroid gland (disorder)
Cancer	SNOMED	94099002	Malignant neoplasm of tibia
Cancer	SNOMED	94101009	Primary malignant neoplasm of tongue (disorder)
Cancer	SNOMED	94102002	Primary malignant neoplasm of tonsillar fossa (disorder)
Cancer	SNOMED	94105000	Primary malignant neoplasm of transverse colon (disorder)
Cancer	SNOMED	94112009	Malignant neoplasm of ulna
Cancer	SNOMED	94125001	Primary malignant neoplasm of urinary system (disorder)
Cancer	SNOMED	94134006	Malignant neoplasm of ventral surface of tongue
Cancer	SNOMED	94142007	Malignant neoplasm of vomer
Cancer	SNOMED	94143002	Primary malignant neoplasm of vulva (disorder)
Cancer	SNOMED	94145009	Malignant neoplasm of zygomatic bone
Cancer	SNOMED	94161006	Secondary malignant neoplasm of adrenal gland
Cancer	SNOMED	94180008	Secondary malignant neoplasm of axilla (disorder)
Cancer	SNOMED	94181007	Secondary malignant neoplasm of axillary lymph nodes
Cancer	SNOMED	94186002	Secondary malignant neoplasm of bladder
Cancer	SNOMED	94217008	Secondary malignant neoplasm of bone marrow (disorder)
Cancer	SNOMED	94220000	Secondary malignant neoplasm of bone of skull (disorder)
Cancer	SNOMED	94222008	Secondary malignant neoplasm of bone
Cancer	SNOMED	94225005	Secondary malignant neoplasm of brain
Cancer	SNOMED	94227002	Secondary malignant neoplasm of bronchopulmonary lymph nodes
Cancer	SNOMED	94230009	Secondary malignant neoplasm of bronchus of right lower lobe (disorder)
Cancer	SNOMED	94233006	Secondary malignant neoplasm of bronchus (disorder)
Cancer	SNOMED	94235004	Secondary malignant neoplasm of cecum (disorder)
Cancer	SNOMED	94243009	Secondary malignant neoplasm of central nervous system (disorder)
Cancer	SNOMED	94246001	Secondary malignant neoplasm of cerebral meninges (disorder)
Cancer	SNOMED	94248000	Secondary malignant neoplasm of cerebrum
Cancer	SNOMED	94253005	Secondary malignant neoplasm of chest wall (disorder)
Cancer	SNOMED	94254004	Secondary malignant neoplasm of choroid (disorder)
Cancer	SNOMED	94260004	Secondary malignant neoplasm of colon
Cancer	SNOMED	94264008	Secondary malignant neoplasm of soft tissues (disorder)
Cancer	SNOMED	94274006	Secondary malignant neoplasm of thoracic vertebral column (disorder)
Cancer	SNOMED	94275007	Secondary malignant neoplasm of duodenum
Cancer	SNOMED	94280003	Secondary malignant neoplasm of endocrine gland (disorder)
Cancer	SNOMED	94281004	Secondary malignant neoplasm of endometrium (disorder)
Cancer	SNOMED	94286009	Secondary malignant neoplasm of esophagus (disorder)
Cancer	SNOMED	94291000119103	Mucinous adenocarcinoma of gastrointestinal tract (disorder)
Cancer	SNOMED	94292003	Secondary malignant neoplasm of eye (disorder)
Cancer	SNOMED	94297009	Secondary malignant neoplasm of female breast
Cancer	SNOMED	94298004	Secondary malignant neoplasm of female genital organ (disorder)
Cancer	SNOMED	94300004	Secondary malignant neoplasm of femur (disorder)
Cancer	SNOMED	94312000	Secondary malignant neoplasm of gallbladder (disorder)
Cancer	SNOMED	94313005	Secondary malignant neoplasm of gastrointestinal tract
Cancer	SNOMED	94326009	Secondary malignant neoplasm of head (disorder)
Cancer	SNOMED	94327000	Secondary malignant neoplasm of heart (disorder)
Cancer	SNOMED	94335002	Secondary malignant neoplasm of ileum
Cancer	SNOMED	94336001	Secondary malignant neoplasm of iliac lymph nodes
Cancer	SNOMED	94338000	Secondary malignant neoplasm of infraclavicular lymph nodes
Cancer	SNOMED	94344001	Secondary malignant neoplasm of intercostal lymph nodes (disorder)

Cancer	SNOMED	94347008	Secondary malignant neoplasm of intra-abdominal lymph nodes
Cancer	SNOMED	94350006	Secondary malignant neoplasm of intrapelvic lymph nodes
Cancer	SNOMED	94351005	Secondary malignant neoplasm of intrathoracic lymph nodes
Cancer	SNOMED	94357009	Secondary malignant neoplasm of jejunum (disorder)
Cancer	SNOMED	94360002	Secondary malignant neoplasm of kidney
Cancer	SNOMED	94361000000105	Breast cancer detected by national screening programme (disorder)
Cancer	SNOMED	94365007	Secondary malignant neoplasm of large intestine
Cancer	SNOMED	94376006	Secondary malignant neoplasm of left upper lobe of lung (disorder)
Cancer	SNOMED	94381002	Secondary malignant neoplasm of liver
Cancer	SNOMED	94389000	Secondary malignant neoplasm of lumbar vertebral column (disorder)
Cancer	SNOMED	94391008	Secondary malignant neoplasm of lung
Cancer	SNOMED	94392001	Secondary malignant neoplasm of lymph node
Cancer	SNOMED	94393006	Secondary malignant neoplasm of lymph nodes of face
Cancer	SNOMED	94395004	Secondary malignant neoplasm of lymph nodes of lower limb
Cancer	SNOMED	94396003	Secondary malignant neoplasm of lymph nodes of multiple sites
Cancer	SNOMED	94397007	Secondary malignant neoplasm of lymph nodes of neck (disorder)
Cancer	SNOMED	94398002	Secondary malignant neoplasm of lymph nodes of upper limb
Cancer	SNOMED	94402006	Secondary malignant neoplasm of male genital organ (disorder)
Cancer	SNOMED	94408005	Secondary malignant neoplasm of mediastinal lymph nodes
Cancer	SNOMED	94409002	Secondary malignant neoplasm of mediastinum
Cancer	SNOMED	94410007	Secondary malignant neoplasm of mesenteric lymph nodes
Cancer	SNOMED	94416001	Secondary malignant neoplasm of mouth (disorder)
Cancer	SNOMED	94418000	Secondary malignant neoplasm of muscle of abdomen (disorder)
Cancer	SNOMED	94432003	Secondary malignant neoplasm of muscle (disorder)
Cancer	SNOMED	94442001	Secondary malignant neoplasm of nervous system
Cancer	SNOMED	94446003	Secondary malignant neoplasm of obturator lymph nodes (disorder)
Cancer	SNOMED	94449005	Secondary malignant neoplasm of occipital lymph nodes
Cancer	SNOMED	94453007	Secondary malignant neoplasm of orbit proper (disorder)
Cancer	SNOMED	94454001	Secondary malignant neoplasm of oropharynx (disorder)
Cancer	SNOMED	94455000	Secondary malignant neoplasm of ovary
Cancer	SNOMED	94459006	Secondary malignant neoplasm of pancreas (disorder)
Cancer	SNOMED	94460001	Secondary malignant neoplasm of pancreatic duct (disorder)
Cancer	SNOMED	94475009	Secondary malignant neoplasm of parotid lymph nodes
Cancer	SNOMED	94477001	Secondary malignant neoplasm of pectoral axillary lymph nodes
Cancer	SNOMED	94478006	Secondary malignant neoplasm of pelvic bone (disorder)
Cancer	SNOMED	94480000	Secondary malignant neoplasm of pelvis (disorder)
Cancer	SNOMED	94481001	Secondary malignant neoplasm of penis
Cancer	SNOMED	94491007	Secondary malignant neoplasm of pituitary gland (disorder)
Cancer	SNOMED	94493005	Secondary malignant neoplasm of pleura
Cancer	SNOMED	94494004	Secondary malignant neoplasm of popliteal lymph nodes (disorder)
Cancer	SNOMED	94503003	Secondary malignant neoplasm of prostate
Cancer	SNOMED	94513006	Secondary malignant neoplasm of rectum
Cancer	SNOMED	94514000	Secondary malignant neoplasm of renal pelvis (disorder)
Cancer	SNOMED	94515004	Secondary malignant neoplasm of respiratory tract
Cancer	SNOMED	94521000	Secondary malignant neoplasm of rib (disorder)
Cancer	SNOMED	94527001	Secondary malignant neoplasm of sacrum (disorder)
Cancer	SNOMED	94531007	Secondary malignant neoplasm of scrotum (disorder)
Cancer	SNOMED	94537006	Secondary malignant neoplasm of shoulder (disorder)
Cancer	SNOMED	94538001	Secondary malignant neoplasm of sigmoid colon (disorder)
Cancer	SNOMED	94544002	Secondary malignant neoplasm of skin of breast
Cancer	SNOMED	94554003	Secondary malignant neoplasm of skin of face
Cancer	SNOMED	94566009	Secondary malignant neoplasm of skin of neck
Cancer	SNOMED	94575006	Secondary malignant neoplasm of skin of trunk
Cancer	SNOMED	94579000	Secondary malignant neoplasm of skin
Cancer	SNOMED	94580002	Secondary malignant neoplasm of small intestine
Cancer	SNOMED	94582005	Secondary malignant neoplasm of soft tissues of abdomen (disorder)
Cancer	SNOMED	94597008	Secondary malignant neoplasm of spermatic cord (disorder)
Cancer	SNOMED	94600009	Secondary malignant neoplasm of spinal cord
Cancer	SNOMED	94602001	Secondary malignant neoplasm of vertebral column
Cancer	SNOMED	94603006	Secondary malignant neoplasm of spleen (disorder)
Cancer	SNOMED	94604000	Secondary malignant neoplasm of splenic flexure of colon (disorder)
Cancer	SNOMED	94606003	Secondary malignant neoplasm of stomach (disorder)
Cancer	SNOMED	94609005	Secondary malignant neoplasm of submandibular lymph nodes
Cancer	SNOMED	94611001	Secondary malignant neoplasm of submental lymph nodes
Cancer	SNOMED	94612008	Secondary malignant neoplasm of superficial inguinal lymph nodes
Cancer	SNOMED	94623007	Secondary malignant neoplasm of testis
Cancer	SNOMED	94626004	Secondary malignant neoplasm of omentum (disorder)
Cancer	SNOMED	94627008	Secondary malignant neoplasm of the peritoneum
Cancer	SNOMED	94628003	Secondary malignant neoplasm of the retroperitoneum
Cancer	SNOMED	94632009	Secondary malignant neoplasm of thymus (disorder)
Cancer	SNOMED	94634005	Secondary malignant neoplasm of thyroid gland (disorder)
Cancer	SNOMED	94638008	Secondary malignant neoplasm of tongue
Cancer	SNOMED	94641004	Secondary malignant neoplasm of trachea (disorder)
Cancer	SNOMED	94642006	Secondary malignant neoplasm of tracheobronchial lymph nodes
Cancer	SNOMED	94659001	Secondary malignant neoplasm of ureter

Cancer	SNOMED	94661005	Secondary malignant neoplasm of urethra
Cancer	SNOMED	94663008	Secondary malignant neoplasm of urinary system
Cancer	SNOMED	94665001	Secondary malignant neoplasm of uterus
Cancer	SNOMED	94668004	Secondary malignant neoplasm of vagina
Cancer	SNOMED	94681006	Secondary malignant neoplasm of vulva
Cancer	SNOMED	94709001	Mycosis fungoides of intrathoracic lymph nodes (disorder)
Cancer	SNOMED	94710006	Mycosis fungoides of lymph nodes of axilla AND/OR upper limb
Cancer	SNOMED	94711005	Mycosis fungoides of lymph nodes of head, face AND/OR neck (disorder)
Cancer	SNOMED	94712003	Mycosis fungoides of lymph nodes of inguinal region AND/OR lower limb
Cancer	SNOMED	94719007	Myeloid sarcoma
Cancer	SNOMED	95111006	Neoplasm of uncertain behavior of spleen (disorder)
Cancer	SNOMED	95186006	Nodular lymphoma of intra-abdominal lymph nodes
Cancer	SNOMED	95188007	Nodular lymphoma of intrathoracic lymph nodes (disorder)
Cancer	SNOMED	95192000	Nodular lymphoma of lymph nodes of multiple sites
Cancer	SNOMED	95193005	Nodular lymphoma of spleen (disorder)
Cancer	SNOMED	95210003	Plasma cell leukemia
Cancer	SNOMED	95214007	Primary malignant neoplasm of liver
Cancer	SNOMED	95224004	Reticulosarcoma of intra-abdominal lymph nodes
Cancer	SNOMED	95231000	Reticulosarcoma of spleen
Cancer	SNOMED	95263006	SÃ©zary's disease of spleen (disorder)
Cancer	SNOMED	9541000119105	Primary adenocarcinoma of gallbladder (disorder)
Cancer	SNOMED	956331000000107	Malignant melanoma stage IA (finding)
Cancer	SNOMED	956351000000100	Malignant melanoma stage IB (finding)
Cancer	SNOMED	956371000000109	Malignant melanoma stage IIA (finding)
Cancer	SNOMED	956391000000108	Malignant melanoma stage IIB (finding)
Cancer	SNOMED	956411000000108	Malignant melanoma stage IIC (finding)
Cancer	SNOMED	956431000000100	Malignant melanoma stage IIIA (finding)
Cancer	SNOMED	956451000000107	Malignant melanoma stage IIIB (finding)
Cancer	SNOMED	956471000000103	Malignant melanoma stage IIIC (finding)
Cancer	SNOMED	956511000000107	Malignant melanoma stage IV M1a (finding)
Cancer	SNOMED	956531000000104	Malignant melanoma stage IV M1b (finding)
Cancer	SNOMED	956551000000106	Malignant melanoma stage IV M1c (finding)
Cancer	SNOMED	9893005	Immunodeficiency with thymoma (disorder)
Cerebral Benign Tumors	ICD10	D32	Benign neoplasm:cerebral meninges
Cerebral Benign Tumors	ICD10	D33	Benign neoplasm:Brain - supratentorial, intratentorial
Cerebral Benign Tumors	SNOMED	275269004	Benign cerebral tumor
Chronic Back Pain	SNOMED	183868005	Referral to back pain clinic
Chronic Back Pain	SNOMED	906561000006105	[RFC] Chronic back and neck problems
Chronic Kidney Disease	ICD10	N00	Acute nephritic syndrome
Chronic Kidney Disease	ICD10	N01	Rapidly progressive nephritic syndrome
Chronic Kidney Disease	ICD10	N03	Chronic nephritic syndrome
Chronic Kidney Disease	ICD10	N052	Unspecified nephritic syndrome ; Diffuse membranous glomerulonephritis
Chronic Kidney Disease	ICD10	N053	Unspecified nephritic syndrome ; Diffuse mesangial proliferative glomerulonephritis
Chronic Kidney Disease	ICD10	N054	Unspecified nephritic syndrome ; Diffuse endocapillary proliferative glomerulonephritis
Chronic Kidney Disease	ICD10	N055	Unspecified nephritic syndrome ; Diffuse mesangiocapillary glomerulonephritis
Chronic Kidney Disease	ICD10	N056	Unspecified nephritic syndrome ; Dense deposit disease
Chronic Kidney Disease	ICD10	N072	Hereditary nephropathy, not elsewhere classified ; Diffuse membranous glomerulonephritis
Chronic Kidney Disease	ICD10	N073	Hereditary nephropathy, not elsewhere classified ; Diffuse mesangial proliferative glomerulonephritis
Chronic Kidney Disease	ICD10	N074	Hereditary nephropathy, not elsewhere classified ; Diffuse endocapillary proliferative glomerulonephritis
Chronic Kidney Disease	ICD10	N100	Acute tubulo-interstitial nephritis
Chronic Kidney Disease	ICD10	N170	Acute renal failure
Chronic Kidney Disease	ICD10	N181	Chronic kidney disease, stage 1
Chronic Kidney Disease	ICD10	N182	Chronic kidney disease, stage 2
Chronic Kidney Disease	ICD10	N183	Chronic kidney disease, stage 3
Chronic Kidney Disease	ICD10	N184	Chronic kidney disease, stage 4
Chronic Kidney Disease	ICD10	N189	Chronic kidney disease, unspecified
Chronic Kidney Disease	ICD10	N190	Unspecified kidney failure
Chronic Kidney Disease	ICD10	N250	Disorders resulting from impaired renal tubular function
Chronic Kidney Disease	ICD10	Z49	Care involving dialysis
Chronic Kidney Disease	SNOMED	324121000000109	Chronic kidney disease stage 1 with proteinuria (disorder)
Chronic Kidney Disease	SNOMED	324151000000104	Chronic kidney disease stage 1 without proteinuria (disorder)
Chronic Kidney Disease	SNOMED	324181000000105	Chronic kidney disease stage 2 with proteinuria (disorder)
Chronic Kidney Disease	SNOMED	324211000000106	Chronic kidney disease stage 2 without proteinuria (disorder)
Chronic Kidney Disease	SNOMED	324251000000105	Chronic kidney disease stage 3 with proteinuria (disorder)
Chronic Kidney Disease	SNOMED	324281000000104	Chronic kidney disease stage 3 without proteinuria (disorder)
Chronic Kidney Disease	SNOMED	324311000000101	Chronic kidney disease stage 3A with proteinuria (disorder)
Chronic Kidney Disease	SNOMED	324341000000100	Chronic kidney disease stage 3A without proteinuria (disorder)
Chronic Kidney Disease	SNOMED	324371000000106	Chronic kidney disease stage 3B with proteinuria (disorder)
Chronic Kidney Disease	SNOMED	324411000000105	Chronic kidney disease stage 3B without proteinuria (disorder)

Chronic Kidney Disease	SNOMED	324441000000106	Chronic kidney disease stage 4 with proteinuria (disorder)
Chronic Kidney Disease	SNOMED	324471000000100	Chronic kidney disease stage 4 without proteinuria (disorder)
Chronic Kidney Disease	SNOMED	324501000000107	Chronic kidney disease stage 5 with proteinuria (disorder)
Chronic Kidney Disease	SNOMED	324541000000105	Chronic kidney disease stage 5 without proteinuria (disorder)
Chronic Kidney Disease	SNOMED	431855005	Chronic kidney disease stage 1 (disorder)
Chronic Kidney Disease	SNOMED	431856006	Chronic kidney disease stage 2 (disorder)
Chronic Kidney Disease	SNOMED	431857002	Chronic kidney disease stage 4 (disorder)
Chronic Kidney Disease	SNOMED	433144002	Chronic kidney disease stage 3 (disorder)
Chronic Kidney Disease	SNOMED	700378005	Chronic kidney disease stage 3A (disorder)
Chronic Kidney Disease	SNOMED	700379002	Chronic kidney disease stage 3B (disorder)
Chronic Kidney Disease	SNOMED	949401000000103	Chronic kidney disease with glomerular filtration rate category G1 and albuminuria category A1 (disorder)
Chronic Kidney Disease	SNOMED	949421000000107	Chronic kidney disease with glomerular filtration rate category G1 and albuminuria category A2 (disorder)
Chronic Kidney Disease	SNOMED	949481000000108	Chronic kidney disease with glomerular filtration rate category G1 and albuminuria category A3 (disorder)
Chronic Kidney Disease	SNOMED	949521000000108	Chronic kidney disease with glomerular filtration rate category G2 and albuminuria category A1 (disorder)
Chronic Kidney Disease	SNOMED	949561000000100	Chronic kidney disease with glomerular filtration rate category G2 and albuminuria category A2 (disorder)
Chronic Kidney Disease	SNOMED	949621000000109	Chronic kidney disease with glomerular filtration rate category G2 and albuminuria category A3 (disorder)
Chronic Kidney Disease	SNOMED	949881000000106	Chronic kidney disease with glomerular filtration rate category G3a and albuminuria category A1 (disorder)
Chronic Kidney Disease	SNOMED	949901000000109	Chronic kidney disease with glomerular filtration rate category G3a and albuminuria category A2 (disorder)
Chronic Kidney Disease	SNOMED	949921000000100	Chronic kidney disease with glomerular filtration rate category G3a and albuminuria category A3 (disorder)
Chronic Kidney Disease	SNOMED	950061000000103	Chronic kidney disease with glomerular filtration rate category G3b and albuminuria category A1 (disorder)
Chronic Kidney Disease	SNOMED	950081000000107	Chronic kidney disease with glomerular filtration rate category G3b and albuminuria category A2 (disorder)
Chronic Kidney Disease	SNOMED	950101000000101	Chronic kidney disease with glomerular filtration rate category G3b and albuminuria category A3 (disorder)
Chronic Kidney Disease	SNOMED	950181000000106	Chronic kidney disease with glomerular filtration rate category G4 and albuminuria category A1 (disorder)
Chronic Kidney Disease	SNOMED	950211000000107	Chronic kidney disease with glomerular filtration rate category G4 and albuminuria category A2 (disorder)
Chronic Kidney Disease	SNOMED	950231000000104	Chronic kidney disease with glomerular filtration rate category G4 and albuminuria category A3 (disorder)
Chronic Kidney Disease	SNOMED	950251000000106	Chronic kidney disease with glomerular filtration rate category G5 and albuminuria category A1 (disorder)
Chronic Kidney Disease	SNOMED	950291000000103	Chronic kidney disease with glomerular filtration rate category G5 and albuminuria category A2 (disorder)
Chronic Kidney Disease	SNOMED	950311000000102	Chronic kidney disease with glomerular filtration rate category G5 and albuminuria category A3 (disorder)
Chronic Lyme Disease	ICD10	A692	Lyme disease, unspecified
Chronic Lyme Disease	SNOMED	230150008	Meningitis in Lyme disease
Chronic Lyme Disease	SNOMED	23502006	Lyme disease
Chronic Lyme Disease	SNOMED	310567000	Lyme enzyme-linked immunosorbent assay positive
Chronic Lyme Disease	SNOMED	310568005	Lyme immunoblot positive
Chronic Lyme Disease	SNOMED	33937009	Lyme arthritis
Chronic Lyme Disease	SNOMED	715507005	Lyme neuroborreliosis
Chronic Lyme Disease	SNOMED	77863005	Lyme carditis
Chronic Obstructive Pulmonary Disease	ICD10	J40	Bronchitis not specified as acute or chronic
Chronic Obstructive Pulmonary Disease	ICD10	J41	Simple and mucopurulent chronic bronchitis
Chronic Obstructive Pulmonary Disease	ICD10	J42	Unspecified chronic bronchitis
Chronic Obstructive Pulmonary Disease	ICD10	J43	Emphysema
Chronic Obstructive Pulmonary Disease	ICD10	J44	Other chronic obstructive pulmonary disease
Chronic Obstructive Pulmonary Disease	SNOMED	10692761000119107	Asthma-chronic obstructive pulmonary disease overlap syndrome (disorder)
Chronic Obstructive Pulmonary Disease	SNOMED	135836000	End stage chronic obstructive airways disease (disorder)
Chronic Obstructive Pulmonary Disease	SNOMED	13645005	Chronic obstructive lung disease (disorder)
Chronic Obstructive Pulmonary Disease	SNOMED	16003001	Giant bullous emphysema (disorder)
Chronic Obstructive Pulmonary Disease	SNOMED	185086009	Chronic obstructive bronchitis (disorder)
Chronic Obstructive Pulmonary Disease	SNOMED	195949008	Chronic asthmatic bronchitis (disorder)
Chronic Obstructive Pulmonary Disease	SNOMED	195953005	Mixed simple and mucopurulent chronic bronchitis (disorder)
Chronic Obstructive Pulmonary Disease	SNOMED	195957006	Chronic bullous emphysema (disorder)
Chronic Obstructive Pulmonary Disease	SNOMED	195958001	Segmental bullous emphysema (disorder)
Chronic Obstructive Pulmonary Disease	SNOMED	195959009	Zonal bullous emphysema (disorder)
Chronic Obstructive Pulmonary Disease	SNOMED	195963002	Acute vesicular emphysema (disorder)
Chronic Obstructive Pulmonary Disease	SNOMED	196026004	Chronic emphysema caused by chemical fumes (disorder)
Chronic Obstructive Pulmonary Disease	SNOMED	196027008	Toxic bronchiolitis obliterans (disorder)
Chronic Obstructive Pulmonary Disease	SNOMED	233672007	Byssinosis grade 3 (disorder)
Chronic Obstructive Pulmonary Disease	SNOMED	233673002	Drug-induced bronchiolitis obliterans (disorder)

Chronic Obstructive Pulmonary Disease	SNOMED	233674008	Pulmonary emphysema in alpha-1 primary immunodeficiency deficiency (disorder)
Chronic Obstructive Pulmonary Disease	SNOMED	233675009	Toxic emphysema (disorder)
Chronic Obstructive Pulmonary Disease	SNOMED	233677001	Scar emphysema (disorder)
Chronic Obstructive Pulmonary Disease	SNOMED	266355005	Bullous emphysema with collapse (disorder)
Chronic Obstructive Pulmonary Disease	SNOMED	266356006	Atrophic (senile) emphysema (disorder)
Chronic Obstructive Pulmonary Disease	SNOMED	293991000000106	Very severe chronic obstructive pulmonary disease (disorder)
Chronic Obstructive Pulmonary Disease	SNOMED	313296004	Mild chronic obstructive pulmonary disease (disorder)
Chronic Obstructive Pulmonary Disease	SNOMED	313297008	Moderate chronic obstructive pulmonary disease (disorder)
Chronic Obstructive Pulmonary Disease	SNOMED	313299006	Severe chronic obstructive pulmonary disease (disorder)
Chronic Obstructive Pulmonary Disease	SNOMED	32544004	Chronic obliterative bronchiolitis caused by inhalation of chemical fumes AND/OR vapors (disorder)
Chronic Obstructive Pulmonary Disease	SNOMED	360470001	Chronic mucus hypersecretion (disorder)
Chronic Obstructive Pulmonary Disease	SNOMED	40100001	Obliterative bronchiolitis (disorder)
Chronic Obstructive Pulmonary Disease	SNOMED	45145000	Unilateral emphysema (situation)
Chronic Obstructive Pulmonary Disease	SNOMED	49691004	Occupational bronchitis (disorder)
Chronic Obstructive Pulmonary Disease	SNOMED	4981000	Panacinar emphysema (disorder)
Chronic Obstructive Pulmonary Disease	SNOMED	52571006	Chronic tracheobronchitis (disorder)
Chronic Obstructive Pulmonary Disease	SNOMED	61937009	Simple chronic bronchitis (disorder)
Chronic Obstructive Pulmonary Disease	SNOMED	63480004	Chronic bronchitis (disorder)
Chronic Obstructive Pulmonary Disease	SNOMED	66987001	Congenital lobar emphysema (disorder)
Chronic Obstructive Pulmonary Disease	SNOMED	68328006	Centriacinar emphysema (disorder)
Chronic Obstructive Pulmonary Disease	SNOMED	74417001	Mucopurulent chronic bronchitis (disorder)
Chronic Obstructive Pulmonary Disease	SNOMED	77690003	Interstitial emphysema of lung (disorder)
Chronic Obstructive Pulmonary Disease	SNOMED	84409004	Fetid chronic bronchitis (disorder)
Chronic Obstructive Pulmonary Disease	SNOMED	866901000000103	Eosinophilic bronchitis (disorder)
Chronic Obstructive Pulmonary Disease	SNOMED	87433001	Pulmonary emphysema (disorder)
Chronic Obstructive Pulmonary Disease	SNOMED	89549007	Catarrhal bronchitis (disorder)
Chronic Pain	ICD10	R521	Chronic intractable pain
Chronic Pain	ICD10	R522	Other chronic pain
Chronic Pain	SNOMED	128200000	Complex regional pain syndrome
Chronic Pain	SNOMED	1727540000000000	Complex regional pain syndrome NOS
Chronic Pain	SNOMED	274665008	[D]Chronic intractable pain
Chronic Pain	SNOMED	278860009	CLBP - Chronic low back pain
Chronic Pain	SNOMED	408751001	Complex regional pain syndrome type II
Chronic Pain	SNOMED	712537009	Complex regional pain syndrome of upper limb
Chronic Pain	SNOMED	734947007	Complex regional pain syndrome type 1
Chronic Pain	SNOMED	734987002	Algodystrophy of foot
Chronic Pain	SNOMED	734988007	Algodystrophy of knee
Chronic Pain	SNOMED	734989004	Algodystrophy of hand
Chronic Pain	SNOMED	82423001	[X]Other chronic pain
Chronic Pancreatitis	ICD10	K861	Other chronic pancreatitis
Chronic Pancreatitis	SNOMED	235494005	Chronic pancreatitis
Chronic Pancreatitis	SNOMED	235951009	Gallstone chronic pancreatitis
Chronic Pancreatitis	SNOMED	247431000000103	History of chronic pancreatitis
Chronic Pancreatitis	SNOMED	25942009	Fibrosis of pancreas
Chronic Pancreatitis	SNOMED	88281007	Atrophy of pancreas
Chronic Urinary Tract Infections	ICD10	N39	Urinary tract infection, site not specified
Chronic Urinary Tract Infections	SNOMED	13285005	Cystitis cystica
Chronic Urinary Tract Infections	SNOMED	161549001	H/O: recurrent cystitis
Chronic Urinary Tract Infections	SNOMED	197760006	Chronic pyelonephritis without medullary necrosis
Chronic Urinary Tract Infections	SNOMED	197761005	Chronic pyelonephritis with medullary necrosis
Chronic Urinary Tract Infections	SNOMED	197762003	Chronic pyelitis
Chronic Urinary Tract Infections	SNOMED	197763008	Chronic pyonephrosis
Chronic Urinary Tract Infections	SNOMED	197764002	Nonobstructive reflux-associated chronic pyelonephritis
Chronic Urinary Tract Infections	SNOMED	197853008	Recurrent cystitis
Chronic Urinary Tract Infections	SNOMED	197927001	Recurrent urinary tract infection
Chronic Urinary Tract Infections	SNOMED	197928006	Chronic urinary tract infection
Chronic Urinary Tract Infections	SNOMED	236374007	Chronic infective interstitial nephritis
Chronic Urinary Tract Infections	SNOMED	236379002	Chronic obstructive pyelonephritis
Chronic Urinary Tract Infections	SNOMED	38898003	Xanthogranulomatous pyelonephritis
Chronic Urinary Tract Infections	SNOMED	441547007	History of chronic urinary tract infection
Chronic Urinary Tract Infections	SNOMED	473116008	History of recurrent urinary tract infection
Chronic Urinary Tract Infections	SNOMED	63302006	Chronic pyelonephritis
Chronic Urinary Tract Infections	SNOMED	88813005	Chronic gonococcal cystitis
Congenital Disease	ICD10	Q000	Hemicephaly (disorder)
Congenital Disease	ICD10	Q001	Derencephalus (disorder)
Congenital Disease	ICD10	Q002	Iniencephaly (disorder)
Congenital Disease	ICD10	Q010	Frontal encephalocele (disorder)
Congenital Disease	ICD10	Q011	Nasal encephalocele (disorder)
Congenital Disease	ICD10	Q012	Occipital encephalocele (disorder)
Congenital Disease	ICD10	Q018	Encephalocele of orbit (disorder)
Congenital Disease	ICD10	Q019	Congenital cerebral meningocele (disorder)
Congenital Disease	ICD10	Q02	Hydromicrocephaly (disorder)
Congenital Disease	ICD10	Q030	Aqueduct of Sylvius anomaly (disorder)

Congenital Disease	ICD10	Q031	Dandy-Walker syndrome (disorder)
Congenital Disease	ICD10	Q038	Communicating hydrocephalus co-occurrent and due to congenital agenesis of arachnoid villi (disorder)
Congenital Disease	ICD10	Q039	Congenital hydrocephalus (disorder)
Congenital Disease	ICD10	Q040	Agenesis of corpus callosum (disorder)
Congenital Disease	ICD10	Q041	Isolated arhinencephaly (disorder)
Congenital Disease	ICD10	Q042	Holoprosencephaly sequence (disorder)
Congenital Disease	ICD10	Q043	Microgyria (disorder)
Congenital Disease	ICD10	Q044	Septo-optic dysplasia sequence (disorder)
Congenital Disease	ICD10	Q045	Macroencephaly (disorder)
Congenital Disease	ICD10	Q046	Congenital cerebral cyst (disorder)
Congenital Disease	ICD10	Q048	Macrogyria (disorder)
Congenital Disease	ICD10	Q049	Congenital anomaly of brain (disorder)
Congenital Disease	ICD10	Q050	Spina bifida of cervical region (disorder)
Congenital Disease	ICD10	Q051	Thoracic spina bifida with hydrocephalus (disorder)
Congenital Disease	ICD10	Q052	Lumbar spina bifida with hydrocephalus (disorder)
Congenital Disease	ICD10	Q053	Sacral spina bifida with hydrocephalus - open (disorder)
Congenital Disease	ICD10	Q054	Spina bifida with hydrocephalus (disorder)
Congenital Disease	ICD10	Q055	Spina bifida without hydrocephalus (disorder)
Congenital Disease	ICD10	Q056	Spina bifida of dorsal region (disorder)
Congenital Disease	ICD10	Q057	Spina bifida of lumbar region (disorder)
Congenital Disease	ICD10	Q058	Congenital sacral meningocele (disorder)
Congenital Disease	ICD10	Q059	Spina bifida aperta (disorder)
Congenital Disease	ICD10	Q060	Amyelia (disorder)
Congenital Disease	ICD10	Q061	Spinal cord dysplasia (disorder)
Congenital Disease	ICD10	Q062	Faun tail syndrome (disorder)
Congenital Disease	ICD10	Q063	Congenital anomaly of cauda equina (disorder)
Congenital Disease	ICD10	Q064	Hydromyelia (disorder)
Congenital Disease	ICD10	Q068	Occult spinal dysraphism sequence (disorder)
Congenital Disease	ICD10	Q069	Congenital anomaly of spinal meninges (disorder)
Congenital Disease	ICD10	Q070	Closed spina bifida with Arnold-Chiari malformation (disorder)
Congenital Disease	ICD10	Q078	Jaw-winking syndrome (disorder)
Congenital Disease	ICD10	Q079	Congenital anomaly of the peripheral nervous system (disorder)
Congenital Disease	ICD10	Q100	Congenital ptosis (disorder)
Congenital Disease	ICD10	Q101	Congenital ectropion (disorder)
Congenital Disease	ICD10	Q102	Congenital entropion (disorder)
Congenital Disease	ICD10	Q103	Ablepharon (disorder)
Congenital Disease	ICD10	Q104	Congenitally small punctum lacrimale (disorder)
Congenital Disease	ICD10	Q105	Congenital obstruction of lacrimal canaliculus (disorder)
Congenital Disease	ICD10	Q106	Congenital anomaly of lacrimal system (disorder)
Congenital Disease	ICD10	Q107	Congenital anomaly of orbit proper (disorder)
Congenital Disease	ICD10	Q110	Congenital cystic eyeball (disorder)
Congenital Disease	ICD10	Q111	Anophthalmos (disorder)
Congenital Disease	ICD10	Q112	Dysplasia of eye (disorder)
Congenital Disease	ICD10	Q113	Macrophthalmos (disorder)
Congenital Disease	ICD10	Q120	Embryonal nuclear cataract (disorder)
Congenital Disease	ICD10	Q121	Congenital ectopic lens (disorder)
Congenital Disease	ICD10	Q122	Persistent tunica vasculosa lentis (disorder)
Congenital Disease	ICD10	Q123	Congenital aphakia (disorder)
Congenital Disease	ICD10	Q124	Spherophakia (disorder)
Congenital Disease	ICD10	Q128	Congenital anomaly of lens shape (disorder)
Congenital Disease	ICD10	Q129	Congenital anomaly of lens (disorder)
Congenital Disease	ICD10	Q130	Congenital coloboma of iris (disorder)
Congenital Disease	ICD10	Q131	Congenital aniridia (disorder)
Congenital Disease	ICD10	Q132	Atresia of pupil (disorder)
Congenital Disease	ICD10	Q133	Congenital corneal opacity interfering with vision (disorder)
Congenital Disease	ICD10	Q134	Microcornea (disorder)
Congenital Disease	ICD10	Q135	Blue sclera (disorder)
Congenital Disease	ICD10	Q138	Rieger syndrome (disorder)
Congenital Disease	ICD10	Q139	Congenital anomaly of anterior chamber of eye (disorder)
Congenital Disease	ICD10	Q140	Vestigial remnants of canal of Cloquet (disorder)
Congenital Disease	ICD10	Q141	Congenital retinal aneurysm (disorder)
Congenital Disease	ICD10	Q142	Congenital coloboma of optic disc (disorder)
Congenital Disease	ICD10	Q143	Congenital anomaly of choroid (disorder)
Congenital Disease	ICD10	Q148	Congenital chorioretinal degeneration (disorder)
Congenital Disease	ICD10	Q149	Congenital anomaly of posterior segment of eye (disorder)
Congenital Disease	ICD10	Q150	Glaucoma of childhood (disorder)
Congenital Disease	ICD10	Q158	Microphthalmos associated with other anomalies of eye AND/OR adnexa (disorder)
Congenital Disease	ICD10	Q159	Congenital anomaly of eye (disorder)
Congenital Disease	ICD10	Q160	Congenital absence of auricle with stenosis of auditory canal (disorder)
Congenital Disease	ICD10	Q161	Congenital stricture of osseous meatus of middle ear (disorder)
Congenital Disease	ICD10	Q162	Congenital absence of eustachian tube (disorder)
Congenital Disease	ICD10	Q163	Congenital fusion of ossicles of ear (disorder)
Congenital Disease	ICD10	Q164	Congenital anomaly of middle ear (disorder)

Congenital Disease	ICD10	Q165	Congenital absence of membranous labyrinth (disorder)
Congenital Disease	ICD10	Q169	Congenital absence of ear (disorder)
Congenital Disease	ICD10	Q170	Polyotia (disorder)
Congenital Disease	ICD10	Q171	Macrotia (disorder)
Congenital Disease	ICD10	Q172	Microtia (disorder)
Congenital Disease	ICD10	Q173	Congenital abnormal shape of pinna (disorder)
Congenital Disease	ICD10	Q174	Congenital malposition of pinna (disorder)
Congenital Disease	ICD10	Q175	Bat ear (disorder)
Congenital Disease	ICD10	Q178	Congenital absence of ear lobe (disorder)
Congenital Disease	ICD10	Q179	Congenital malformation of ear (disorder)
Congenital Disease	ICD10	Q180	Branchial cleft cyst (disorder)
Congenital Disease	ICD10	Q181	Preauricular dimple (disorder)
Congenital Disease	ICD10	Q182	Cervical auricle (disorder)
Congenital Disease	ICD10	Q183	Neck webbing (disorder)
Congenital Disease	ICD10	Q184	Congenital macrostomia (disorder)
Congenital Disease	ICD10	Q185	Microstomia (disorder)
Congenital Disease	ICD10	Q186	Congenital macrocheilia (disorder)
Congenital Disease	ICD10	Q187	Congenital microcheilia (disorder)
Congenital Disease	ICD10	Q188	Opocephalus (disorder)
Congenital Disease	ICD10	Q189	Congenital anomaly of neck (disorder)
Congenital Disease	ICD10	Q200	Common arterial trunk and separate origin of pulmonary arteries (disorder)
Congenital Disease	ICD10	Q201	Double outlet right ventricle (disorder)
Congenital Disease	ICD10	Q202	Double outlet left ventricle (disorder)
Congenital Disease	ICD10	Q203	Complete transposition of great vessels (disorder)
Congenital Disease	ICD10	Q204	Common ventricle (disorder)
Congenital Disease	ICD10	Q205	Corrected transposition of great vessels (disorder)
Congenital Disease	ICD10	Q206	Isomerism of atrial appendages (disorder)
Congenital Disease	ICD10	Q208	Left ventricular-right atrial communication (disorder)
Congenital Disease	ICD10	Q209	Left atrial abnormality (disorder)
Congenital Disease	ICD10	Q210	Ventricular septal defect (disorder)
Congenital Disease	ICD10	Q211	Ebstein's anomaly with atrial septal defect (disorder)
Congenital Disease	ICD10	Q212	Endocardial cushion defect (disorder)
Congenital Disease	ICD10	Q213	Tetralogy of Fallot (disorder)
Congenital Disease	ICD10	Q214	Aortopulmonary window (disorder)
Congenital Disease	ICD10	Q218	Pentalogy of Fallot (disorder)
Congenital Disease	ICD10	Q219	Congenital septal defect of heart (disorder)
Congenital Disease	ICD10	Q240	Dextrocardia (disorder)
Congenital Disease	ICD10	Q241	Situs inversus with levocardia (disorder)
Congenital Disease	ICD10	Q242	Cor triatriatum (disorder)
Congenital Disease	ICD10	Q245	Congenital absence of coronary artery (disorder)
Congenital Disease	ICD10	Q246	Congenital heart block (disorder)
Congenital Disease	ICD10	Q248	Congenital valvular insufficiency (disorder)
Congenital Disease	ICD10	Q249	Cyanotic congenital heart disease (disorder)
Congenital Disease	ICD10	Q250	Patent ductus arteriosus (disorder)
Congenital Disease	ICD10	Q251	Coarctation of aorta (disorder)
Congenital Disease	ICD10	Q252	Congenital atresia of aorta (disorder)
Congenital Disease	ICD10	Q253	Congenital supravalvular aortic stenosis (disorder)
Congenital Disease	ICD10	Q254	Double aortic arch (disorder)
Congenital Disease	ICD10	Q255	Congenital atresia of pulmonary artery (disorder)
Congenital Disease	ICD10	Q256	Coarctation of pulmonary artery (disorder)
Congenital Disease	ICD10	Q257	Congenital absence of left pulmonary artery (disorder)
Congenital Disease	ICD10	Q258	Origin of innominate artery from left side of aortic arch (disorder)
Congenital Disease	ICD10	Q260	Congenital stenosis of superior vena cava (disorder)
Congenital Disease	ICD10	Q261	Persistent left superior vena cava (disorder)
Congenital Disease	ICD10	Q262	Anomalous pulmonary venous drainage to abdominal portion of inferior vena cava (disorder)
Congenital Disease	ICD10	Q263	Partial anomalous pulmonary venous connection (disorder)
Congenital Disease	ICD10	Q264	Anomalous pulmonary venous drainage to coronary sinus (disorder)
Congenital Disease	ICD10	Q265	Congenital portal-systemic shunt (disorder)
Congenital Disease	ICD10	Q266	Portal vein-hepatic artery fistula (disorder)
Congenital Disease	ICD10	Q268	Congenital absence of vena cava (disorder)
Congenital Disease	ICD10	Q269	Congenital anomaly of vena cava (disorder)
Congenital Disease	ICD10	Q270	Congenital hypoplasia of umbilical artery (disorder)
Congenital Disease	ICD10	Q271	Congenital renal artery stenosis (disorder)
Congenital Disease	ICD10	Q272	Multiple renal arteries (disorder)
Congenital Disease	ICD10	Q273	Cerebral-retinal arteriovenous aneurysm (disorder)
Congenital Disease	ICD10	Q274	Congenital phlebectasia (disorder)
Congenital Disease	ICD10	Q278	Congenital vascular anomaly of eye (disorder)
Congenital Disease	ICD10	Q279	Anomalous pulmonary venous drainage to hepatic veins (disorder)
Congenital Disease	ICD10	Q280	Arteriovenous malformation of precerebral vessels (disorder)
Congenital Disease	ICD10	Q281	Congenital absence of carotid artery (disorder)
Congenital Disease	ICD10	Q282	Congenital aneurysm of anterior communicating artery (disorder)
Congenital Disease	ICD10	Q283	Congenital anomaly of cerebral artery (disorder)
Congenital Disease	ICD10	Q288	Idiopathic arterial calcification of infancy (disorder)

Congenital Disease	ICD10	Q289	Congenital cardiovascular disorder in mother complicating pregnancy, childbirth AND/OR puerperium (disorder)
Congenital Disease	ICD10	Q300	Congenital stenosis of nares (disorder)
Congenital Disease	ICD10	Q301	Congenital absence of nasal turbinate (disorder)
Congenital Disease	ICD10	Q302	Congenital notching of tip of nose (disorder)
Congenital Disease	ICD10	Q303	Congenital perforation of nasal septum (disorder)
Congenital Disease	ICD10	Q308	Accessory nose (disorder)
Congenital Disease	ICD10	Q309	Congenital deformity of nose (disorder)
Congenital Disease	ICD10	Q310	Congenital web of larynx (disorder)
Congenital Disease	ICD10	Q311	Congenital subglottic stenosis (disorder)
Congenital Disease	ICD10	Q312	Laryngeal hypoplasia (disorder)
Congenital Disease	ICD10	Q313	Congenital laryngocele (disorder)
Congenital Disease	ICD10	Q315	Congenital laryngomalacia (disorder)
Congenital Disease	ICD10	Q318	Congenital atresia of epiglottis (disorder)
Congenital Disease	ICD10	Q319	Congenital anomaly of larynx (disorder)
Congenital Disease	ICD10	Q320	Congenital tracheomalacia (disorder)
Congenital Disease	ICD10	Q321	Congenital absence of trachea (disorder)
Congenital Disease	ICD10	Q322	Congenital bronchomalacia (disorder)
Congenital Disease	ICD10	Q323	Congenital bronchial stenosis (disorder)
Congenital Disease	ICD10	Q324	Congenital absence of bronchus (disorder)
Congenital Disease	ICD10	Q330	Congenital honeycomb lung (disorder)
Congenital Disease	ICD10	Q331	Accessory lobe of lung (disorder)
Congenital Disease	ICD10	Q332	Extrapulmonary subpleural pulmonary sequestration (disorder)
Congenital Disease	ICD10	Q333	Congenital absence of lobe of lung (disorder)
Congenital Disease	ICD10	Q334	Congenital bronchiectasis (disorder)
Congenital Disease	ICD10	Q335	Ectopic bone and cartilage in lung (disorder)
Congenital Disease	ICD10	Q336	Congenital hypoplasia of lung (disorder)
Congenital Disease	ICD10	Q338	Bilobed right lung (disorder)
Congenital Disease	ICD10	Q339	Congenital anomaly of lung (disorder)
Congenital Disease	ICD10	Q340	Congenital anomaly of pleural folds (disorder)
Congenital Disease	ICD10	Q341	Congenital cyst of mediastinum (disorder)
Congenital Disease	ICD10	Q348	Congenital atresia of nasopharynx (disorder)
Congenital Disease	ICD10	Q349	Congenital anomaly of upper respiratory system (disorder)
Congenital Disease	ICD10	Q351	Submucous cleft of hard palate (disorder)
Congenital Disease	ICD10	Q353	Cleft of soft palate (disorder)
Congenital Disease	ICD10	Q355	Complete bilateral cleft palate (disorder)
Congenital Disease	ICD10	Q357	Cleft uvula (disorder)
Congenital Disease	ICD10	Q359	Incomplete bilateral cleft palate (disorder)
Congenital Disease	ICD10	Q360	Incomplete bilateral cleft lip (disorder)
Congenital Disease	ICD10	Q361	Central cleft lip (disorder)
Congenital Disease	ICD10	Q369	Cheilognathoschisis (disorder)
Congenital Disease	ICD10	Q370	Cleft hard palate with cleft lip, bilateral (disorder)
Congenital Disease	ICD10	Q371	Cleft hard palate with left cleft lip (disorder)
Congenital Disease	ICD10	Q372	Cleft soft palate with bilateral cleft lip and bilateral cleft of alveolar process of maxilla (disorder)
Congenital Disease	ICD10	Q373	Cleft soft palate with left cleft lip (disorder)
Congenital Disease	ICD10	Q374	Cleft hard and soft palate with bilateral cleft lip and bilateral cleft of alveolar process of maxilla (disorder)
Congenital Disease	ICD10	Q375	Bilateral complete cleft palate with cleft lip (disorder)
Congenital Disease	ICD10	Q378	Cleft palate with bilateral cleft lip and bilateral cleft of alveolar process of maxilla (disorder)
Congenital Disease	ICD10	Q379	Cleft palate with cleft lip (disorder)
Congenital Disease	ICD10	Q380	Congenital fistula of lip (disorder)
Congenital Disease	ICD10	Q381	Tongue tie (disorder)
Congenital Disease	ICD10	Q382	Congenital macroglossia (disorder)
Congenital Disease	ICD10	Q383	Congenital adhesions of tongue (disorder)
Congenital Disease	ICD10	Q384	Congenital absence of salivary gland (disorder)
Congenital Disease	ICD10	Q385	Congenital absence of uvula (disorder)
Congenital Disease	ICD10	Q386	Synchilia (disorder)
Congenital Disease	ICD10	Q387	Pharyngeal diverticulitis (disorder)
Congenital Disease	ICD10	Q388	Congenital anomaly of pharynx (disorder)
Congenital Disease	ICD10	Q390	Congenital atresia of esophagus (disorder)
Congenital Disease	ICD10	Q391	Esophageal atresia with tracheoesophageal fistula (disorder)
Congenital Disease	ICD10	Q392	H-type congenital tracheoesophageal fistula (disorder)
Congenital Disease	ICD10	Q393	Vascular compression of esophagus by aberrant artery (disorder)
Congenital Disease	ICD10	Q394	Upper esophageal web (disorder)
Congenital Disease	ICD10	Q395	Congenital dilatation of esophagus (disorder)
Congenital Disease	ICD10	Q396	Congenital esophageal pouch (disorder)
Congenital Disease	ICD10	Q398	Congenital absence of esophagus (disorder)
Congenital Disease	ICD10	Q399	Congenital anomaly of esophagus (disorder)
Congenital Disease	ICD10	Q400	Congenital hypertrophic pyloric stenosis (disorder)
Congenital Disease	ICD10	Q401	Congenital hiatus hernia (disorder)
Congenital Disease	ICD10	Q402	Congenital duplication of stomach (disorder)
Congenital Disease	ICD10	Q403	Congenital anomaly of stomach (disorder)
Congenital Disease	ICD10	Q409	Congenital malformation of upper alimentary tract (disorder)

Congenital Disease	ICD10	Q410	Congenital duodenal stenosis (disorder)
Congenital Disease	ICD10	Q411	Congenital absence of jejunum (disorder)
Congenital Disease	ICD10	Q412	Congenital atresia of ileum (disorder)
Congenital Disease	ICD10	Q419	Partial congenital duodenal obstruction (disorder)
Congenital Disease	ICD10	Q420	Congenital absence of rectum with fistula (disorder)
Congenital Disease	ICD10	Q421	Anorectal agenesis (disorder)
Congenital Disease	ICD10	Q422	Anorectal agenesis (disorder)
Congenital Disease	ICD10	Q423	Congenital occlusion of anus (disorder)
Congenital Disease	ICD10	Q428	Congenital absence of appendix (disorder)
Congenital Disease	ICD10	Q429	Congenital atresia of colon (disorder)
Congenital Disease	ICD10	Q430	Meckel's diverticulum (disorder)
Congenital Disease	ICD10	Q431	Congenital neurogenic ileus syndrome (disorder)
Congenital Disease	ICD10	Q432	Congenital dilatation of colon (disorder)
Congenital Disease	ICD10	Q433	Malrotation of colon (disorder)
Congenital Disease	ICD10	Q434	Congenital duplication of intestine (disorder)
Congenital Disease	ICD10	Q435	Ectopic anus (disorder)
Congenital Disease	ICD10	Q436	Congenital rectocloacal fistula (disorder)
Congenital Disease	ICD10	Q437	Persistent cloaca (disorder)
Congenital Disease	ICD10	Q438	Transposition of appendix (disorder)
Congenital Disease	ICD10	Q439	Congenital anomaly of large intestine (disorder)
Congenital Disease	ICD10	Q440	Congenital absence of gallbladder (disorder)
Congenital Disease	ICD10	Q441	Congenital septation of gallbladder (disorder)
Congenital Disease	ICD10	Q442	Congenital hypoplasia of bile duct (disorder)
Congenital Disease	ICD10	Q443	Congenital stricture of bile duct (disorder)
Congenital Disease	ICD10	Q444	Congenital choledochal cyst (disorder)
Congenital Disease	ICD10	Q445	Congenital absence of bile duct (disorder)
Congenital Disease	ICD10	Q446	Congenital cystic disease of liver (disorder)
Congenital Disease	ICD10	Q447	Congenital hepatomegaly (disorder)
Congenital Disease	ICD10	Q450	Congenital hypoplasia of pancreas (disorder)
Congenital Disease	ICD10	Q451	Annular pancreas (disorder)
Congenital Disease	ICD10	Q452	Congenital cyst of pancreas (disorder)
Congenital Disease	ICD10	Q453	Ectopic pancreas in duodenum (disorder)
Congenital Disease	ICD10	Q458	Cyst of omentum (disorder)
Congenital Disease	ICD10	Q459	Congenital anomaly of lower alimentary tract (disorder)
Congenital Disease	ICD10	Q500	Congenital absence of ovary (disorder)
Congenital Disease	ICD10	Q501	Embryonic cyst of ovary (disorder)
Congenital Disease	ICD10	Q502	Congenital torsion of ovary (disorder)
Congenital Disease	ICD10	Q503	Accessory ovary (disorder)
Congenital Disease	ICD10	Q504	Embryonic cyst of fimbria of fallopian tube (disorder)
Congenital Disease	ICD10	Q505	Embryonic cyst of Gartner's duct (disorder)
Congenital Disease	ICD10	Q506	Accessory broad ligament (disorder)
Congenital Disease	ICD10	Q510	Congenital absence of uterus (disorder)
Congenital Disease	ICD10	Q511	Uterine cervix double (finding)
Congenital Disease	ICD10	Q512	Uterus subseptus (disorder)
Congenital Disease	ICD10	Q513	Uterus cordiformis (disorder)
Congenital Disease	ICD10	Q514	Uterus unicornis (disorder)
Congenital Disease	ICD10	Q515	Congenital absence of cervix (disorder)
Congenital Disease	ICD10	Q516	Embryonic cyst of cervix (disorder)
Congenital Disease	ICD10	Q517	Congenital fistulae between uterus and digestive and urinary tracts (disorder)
Congenital Disease	ICD10	Q518	Congenital stenosis of cervical canal (disorder)
Congenital Disease	ICD10	Q519	Congenital uterine anomaly (disorder)
Congenital Disease	ICD10	Q520	Rokitansky sequence (disorder)
Congenital Disease	ICD10	Q521	Congenital duplication of vagina (disorder)
Congenital Disease	ICD10	Q522	Congenital rectovestibular fistula (disorder)
Congenital Disease	ICD10	Q523	Imperforate hymen (disorder)
Congenital Disease	ICD10	Q524	Congenital stenosis of vagina (disorder)
Congenital Disease	ICD10	Q525	Congenital fusion of labia (disorder)
Congenital Disease	ICD10	Q526	Congenital absence of clitoris (disorder)
Congenital Disease	ICD10	Q527	Congenital cyst of vulva (disorder)
Congenital Disease	ICD10	Q528	Congenital aplasia of round ligament (disorder)
Congenital Disease	ICD10	Q529	Congenital anomaly of female genital system (disorder)
Congenital Disease	ICD10	Q530	Ectopic testis (disorder)
Congenital Disease	ICD10	Q531	Undescended left testicle (disorder)
Congenital Disease	ICD10	Q532	Undescended testes - bilateral (disorder)
Congenital Disease	ICD10	Q539	Congenital malposition of testis (disorder)
Congenital Disease	ICD10	Q540	Spina bifida and hypospadias syndrome (disorder)
Congenital Disease	ICD10	Q541	Penile hypospadias (disorder)
Congenital Disease	ICD10	Q542	Penoscrotal hypospadias (disorder)
Congenital Disease	ICD10	Q543	Perineal hypospadias (disorder)
Congenital Disease	ICD10	Q544	Congenital chordee (disorder)
Congenital Disease	ICD10	Q548	Male subcoronal hypospadias (disorder)
Congenital Disease	ICD10	Q549	Paraspadias (disorder)
Congenital Disease	ICD10	Q550	Leydig cell agenesis (disorder)
Congenital Disease	ICD10	Q551	Congenital fusion of testis (disorder)

Congenital Disease	ICD10	Q552	Polyorchism (disorder)
Congenital Disease	ICD10	Q553	Congenital atresia of vas deferens (disorder)
Congenital Disease	ICD10	Q554	Congenital absence of vas deferens (disorder)
Congenital Disease	ICD10	Q555	Congenital absence of penis (disorder)
Congenital Disease	ICD10	Q556	Congenital hypoplasia of penis (disorder)
Congenital Disease	ICD10	Q558	Patent processus vaginalis (disorder)
Congenital Disease	ICD10	Q559	Congenital anomaly of male genital system (disorder)
Congenital Disease	ICD10	Q560	Palmoplantar keratoderma, 46,XX sex reversal, predisposition to squamous cell carcinoma syndrome (disorder)
Congenital Disease	ICD10	Q561	3-Oxo-5 alpha-steroid delta 4-dehydrogenase deficiency (disorder)
Congenital Disease	ICD10	Q562	Female pseudohermaphroditism (disorder)
Congenital Disease	ICD10	Q563	Pseudohermaphroditism (disorder)
Congenital Disease	ICD10	Q564	Ambiguous genitalia (disorder)
Congenital Disease	ICD10	Q600	Double uterus, hemivagina, renal agenesis syndrome (disorder)
Congenital Disease	ICD10	Q602	Congenital absence of renal papilla (disorder)
Congenital Disease	ICD10	Q603	Agenesis of left kidney co-occurrent with hypoplasia of right kidney (disorder)
Congenital Disease	ICD10	Q604	Bilateral renal hypoplasia (disorder)
Congenital Disease	ICD10	Q605	Oligomeganephronic hypoplasia of kidney (disorder)
Congenital Disease	ICD10	Q606	Potter's facies (disorder)
Congenital Disease	ICD10	Q610	Simple renal cyst (disorder)
Congenital Disease	ICD10	Q611	Polycystic kidney disease, infantile type (disorder)
Congenital Disease	ICD10	Q612	Adult type polycystic kidney disease type 1 (disorder)
Congenital Disease	ICD10	Q613	Neonatal diabetes, congenital hypothyroidism, congenital glaucoma, hepatic fibrosis, polycystic kidney syndrome (disorder)
Congenital Disease	ICD10	Q614	Renal dysplasia (disorder)
Congenital Disease	ICD10	Q615	Medullary cystic disease (disorder)
Congenital Disease	ICD10	Q618	Microcystic renal disease (disorder)
Congenital Disease	ICD10	Q619	Meckel-Gruber syndrome (disorder)
Congenital Disease	ICD10	Q620	Congenital hydronephrosis (disorder)
Congenital Disease	ICD10	Q621	Congenital stricture of ureteropelvic junction (disorder)
Congenital Disease	ICD10	Q622	Megacystis-megaureter syndrome (disorder)
Congenital Disease	ICD10	Q623	Congenital hydroureter (disorder)
Congenital Disease	ICD10	Q624	Congenital absence of ureter (disorder)
Congenital Disease	ICD10	Q625	Double renal pelvis (disorder)
Congenital Disease	ICD10	Q626	Congenital deviation of ureter (disorder)
Congenital Disease	ICD10	Q627	Congenital vesicoureterorenal reflux, bilateral (disorder)
Congenital Disease	ICD10	Q628	Congenital anomaly of ureter (disorder)
Congenital Disease	ICD10	Q630	Accessory kidney (disorder)
Congenital Disease	ICD10	Q631	Congenital lobulation of kidney (disorder)
Congenital Disease	ICD10	Q632	Ectopic kidney (disorder)
Congenital Disease	ICD10	Q633	Congenital hypertrophy of ureteric valve (disorder)
Congenital Disease	ICD10	Q638	Congenital calculus of kidney (disorder)
Congenital Disease	ICD10	Q639	Congenital anomaly of renal pelvis (disorder)
Congenital Disease	ICD10	Q640	Female epispadias (disorder)
Congenital Disease	ICD10	Q641	Exstrophy of cloaca sequence (disorder)
Congenital Disease	ICD10	Q643	Congenital stricture of urethra (disorder)
Congenital Disease	ICD10	Q644	Allantoic cyst (disorder)
Congenital Disease	ICD10	Q645	Congenital absence of urethra (disorder)
Congenital Disease	ICD10	Q646	Congenital diverticulum of bladder (disorder)
Congenital Disease	ICD10	Q647	Double urinary meatus (disorder)
Congenital Disease	ICD10	Q648	Congenital gastrointestinal-urinary tract fistula (disorder)
Congenital Disease	ICD10	Q649	Congenital anomaly of the urinary tract proper (disorder)
Congenital Disease	ICD10	Q650	Congenital dislocation of left hip (disorder)
Congenital Disease	ICD10	Q651	Bilateral congenital dislocation of hip (disorder)
Congenital Disease	ICD10	Q652	Congenital dislocation of hip (disorder)
Congenital Disease	ICD10	Q653	Congenital dislocation of left hip co-occurrent with congenital subluxation of right hip (disorder)
Congenital Disease	ICD10	Q654	Congenital subluxation of hip, bilateral (disorder)
Congenital Disease	ICD10	Q655	Congenital subluxation of hip (disorder)
Congenital Disease	ICD10	Q656	Dislocatable hip (disorder)
Congenital Disease	ICD10	Q658	Congenital anteversion of femur (finding)
Congenital Disease	ICD10	Q659	Congenital deformity of hip joint (disorder)
Congenital Disease	ICD10	Q660	Equinovarus deformity of foot (finding)
Congenital Disease	ICD10	Q661	Talipes calcaneovarus (disorder)
Congenital Disease	ICD10	Q662	Metatarsus adductus (disorder)
Congenital Disease	ICD10	Q663	Congenital varus deformity of foot (disorder)
Congenital Disease	ICD10	Q664	Congenital talipes calcaneovalgus (disorder)
Congenital Disease	ICD10	Q665	Congenital pes planus (disorder)
Congenital Disease	ICD10	Q666	Congenital valgus deformity of foot (disorder)
Congenital Disease	ICD10	Q667	Talipes cavus (disorder)
Congenital Disease	ICD10	Q668	Talipes equinus (disorder)
Congenital Disease	ICD10	Q669	Bean-shaped foot (finding)
Congenital Disease	ICD10	Q670	Facial asymmetry (disorder)
Congenital Disease	ICD10	Q672	Dolichocephalic dwarfism (disorder)

Congenital Disease	ICD10	Q673	Plagiocephaly (disorder)
Congenital Disease	ICD10	Q674	Congenital bent nose (disorder)
Congenital Disease	ICD10	Q675	Congenital postural scoliosis (disorder)
Congenital Disease	ICD10	Q676	Congenital pectus excavatum (disorder)
Congenital Disease	ICD10	Q677	Pectus carinatum (disorder)
Congenital Disease	ICD10	Q678	Flat chest (disorder)
Congenital Disease	ICD10	Q680	Congenital anomaly of sternocleidomastoid muscle (disorder)
Congenital Disease	ICD10	Q681	Congenital spade-like hand (disorder)
Congenital Disease	ICD10	Q682	Congenital dislocation of knee with genu recurvatum (disorder)
Congenital Disease	ICD10	Q683	Congenital bowing of femur (disorder)
Congenital Disease	ICD10	Q684	Congenital bowing of tibia and/or fibula (disorder)
Congenital Disease	ICD10	Q685	Congenital leg bone bowing (disorder)
Congenital Disease	ICD10	Q688	Congenital articular rigidity with myopathy (disorder)
Congenital Disease	ICD10	Q690	Congenital hypoplasia of finger (disorder)
Congenital Disease	ICD10	Q691	Bifid thumb (disorder)
Congenital Disease	ICD10	Q692	Polydactyly of toes (disorder)
Congenital Disease	ICD10	Q699	Hexadactyly (disorder)
Congenital Disease	ICD10	Q700	Syndactyly of fingers with fusion of bones (disorder)
Congenital Disease	ICD10	Q701	Syndactyly of fingers (disorder)
Congenital Disease	ICD10	Q702	Syndactyly of toes with fusion of bones (disorder)
Congenital Disease	ICD10	Q703	Simple syndactyly of toes (disorder)
Congenital Disease	ICD10	Q704	Polysyndactyly (disorder)
Congenital Disease	ICD10	Q709	Syndactyly of toes (disorder)
Congenital Disease	ICD10	Q710	Longitudinal deficiency of upper limb (disorder)
Congenital Disease	ICD10	Q711	Complete phocomelia of upper limb (disorder)
Congenital Disease	ICD10	Q712	Congenital absence of forearm and hand (disorder)
Congenital Disease	ICD10	Q713	Partial aphalangia of upper limb (disorder)
Congenital Disease	ICD10	Q714	Talipomanus (disorder)
Congenital Disease	ICD10	Q715	Longitudinal deficiency of ulna (disorder)
Congenital Disease	ICD10	Q716	Congenital cleft hand (disorder)
Congenital Disease	ICD10	Q718	Longitudinal absence of radius AND ulna (disorder)
Congenital Disease	ICD10	Q719	Ectromelia of upper limb (disorder)
Congenital Disease	ICD10	Q720	Transverse deficiency of lower limb (disorder)
Congenital Disease	ICD10	Q721	Complete phocomelia of lower limb (disorder)
Congenital Disease	ICD10	Q722	Congenital absence of lower leg and foot (disorder)
Congenital Disease	ICD10	Q723	Congenital absence of all toes (disorder)
Congenital Disease	ICD10	Q724	Congenital absence of femur (disorder)
Congenital Disease	ICD10	Q725	Longitudinal deficiency of tibia (disorder)
Congenital Disease	ICD10	Q726	Longitudinal deficiency of fibula (disorder)
Congenital Disease	ICD10	Q727	Split foot (disorder)
Congenital Disease	ICD10	Q728	Longitudinal deficiency of tarsal bone (disorder)
Congenital Disease	ICD10	Q729	Ectromelia of lower limb (disorder)
Congenital Disease	ICD10	Q730	Partial congenital absence of limb (disorder)
Congenital Disease	ICD10	Q731	Phocomelia (disorder)
Congenital Disease	ICD10	Q738	Brachymegalodactyly (disorder)
Congenital Disease	ICD10	Q740	Madelung's deformity (disorder)
Congenital Disease	ICD10	Q741	Congenital absence of patella (disorder)
Congenital Disease	ICD10	Q742	Macrodactyly of toe (disorder)
Congenital Disease	ICD10	Q743	Amyoplasia congenita disruptive sequence (disorder)
Congenital Disease	ICD10	Q748	Anisomelia (disorder)
Congenital Disease	ICD10	Q749	Congenital anomaly of limb (disorder)
Congenital Disease	ICD10	Q750	Brachycephaly (disorder)
Congenital Disease	ICD10	Q751	Crouzon syndrome (disorder)
Congenital Disease	ICD10	Q752	Hypertelorism (disorder)
Congenital Disease	ICD10	Q753	Familial megalencephaly (disorder)
Congenital Disease	ICD10	Q754	Miller syndrome (disorder)
Congenital Disease	ICD10	Q755	Oculomandibular dysostosis (disorder)
Congenital Disease	ICD10	Q758	Congenital absence of skull bone (disorder)
Congenital Disease	ICD10	Q759	Overriding skull bones (disorder)
Congenital Disease	ICD10	Q760	Spina bifida occulta (disorder)
Congenital Disease	ICD10	Q761	Klippel-Feil sequence (disorder)
Congenital Disease	ICD10	Q762	Congenital spondylolisthesis (disorder)
Congenital Disease	ICD10	Q763	Bertolotti's syndrome (disorder)
Congenital Disease	ICD10	Q764	Platyspondylia (disorder)
Congenital Disease	ICD10	Q765	Cervical rib (disorder)
Congenital Disease	ICD10	Q766	Rib syndrome (finding)
Congenital Disease	ICD10	Q767	Congenital absence of sternum (disorder)
Congenital Disease	ICD10	Q768	Jarcho-Levin syndrome (disorder)
Congenital Disease	ICD10	Q769	Congenital anomaly of thoracic cage (disorder)
Congenital Disease	ICD10	Q770	Achondrogenesis (disorder)
Congenital Disease	ICD10	Q771	Thanatophoric dysplasia (disorder)
Congenital Disease	ICD10	Q772	Short rib-polydactyly syndrome, Majewski type (disorder)
Congenital Disease	ICD10	Q773	Rhizomelic chondrodysplasia punctata syndrome (disorder)
Congenital Disease	ICD10	Q774	Achondroplasia (disorder)
Congenital Disease	ICD10	Q775	Diastrophic dysplasia (disorder)

Congenital Disease	ICD10	Q776	Chondroectodermal dysplasia (disorder)
Congenital Disease	ICD10	Q777	Spondyloepiphyseal dysplasia tarda (disorder)
Congenital Disease	ICD10	Q778	Leri-Weill dyschondrosteosis (disorder)
Congenital Disease	ICD10	Q779	Fibrochondrogenesis (disorder)
Congenital Disease	ICD10	Q780	Osteogenesis imperfecta, dominant perinatal lethal (disorder)
Congenital Disease	ICD10	Q781	Polyostotic fibrous dysplasia of bone (disorder)
Congenital Disease	ICD10	Q782	Osteopetrosis (disorder)
Congenital Disease	ICD10	Q783	Diaphyseal dysplasia (disorder)
Congenital Disease	ICD10	Q784	Maffucci syndrome (disorder)
Congenital Disease	ICD10	Q785	Metaphyseal chondrodysplasia, McKusick type (disorder)
Congenital Disease	ICD10	Q786	Metachondromatosis (disorder)
Congenital Disease	ICD10	Q788	Lenz-Majewski hyperostosis syndrome (disorder)
Congenital Disease	ICD10	Q789	Hypoplastic chondrodystrophy (disorder)
Congenital Disease	ICD10	Q790	Congenital diaphragmatic hernia (disorder)
Congenital Disease	ICD10	Q791	Congenital eventration of right crus of diaphragm (disorder)
Congenital Disease	ICD10	Q792	Omphalocele with obstruction (disorder)
Congenital Disease	ICD10	Q793	Gastroschisis (disorder)
Congenital Disease	ICD10	Q794	Prune belly syndrome (disorder)
Congenital Disease	ICD10	Q795	Hologastroschisis (disorder)
Congenital Disease	ICD10	Q796	Ehlers-Danlos syndrome, procollagen proteinase resistant (disorder)
Congenital Disease	ICD10	Q798	Hyperphosphatasemia with bone disease (disorder)
Congenital Disease	ICD10	Q799	Congenital anomaly of skeletal bone (disorder)
Congenital Disease	ICD10	Q800	Ichthyosis vulgaris (disorder)
Congenital Disease	ICD10	Q801	X-linked ichthyosis with steryl-sulfatase deficiency (disorder)
Congenital Disease	ICD10	Q802	Lamellar ichthyosis AND trichorrhexis invaginata syndrome (disorder)
Congenital Disease	ICD10	Q803	Congenital ichthyosiform erythroderma (disorder)
Congenital Disease	ICD10	Q804	Harlequin ichthyosis (disorder)
Congenital Disease	ICD10	Q808	Ichthyosis linearis circumflexa (disorder)
Congenital Disease	ICD10	Q809	Congenital ichthyosis of skin (disorder)
Congenital Disease	ICD10	Q810	Epidermolysis bullosa simplex (disorder)
Congenital Disease	ICD10	Q812	Dominant dystrophic epidermolysis bullosa with absence of skin (disorder)
Congenital Disease	ICD10	Q818	Congenital junctional epidermolysis bullosa-pyloric atresia syndrome (disorder)
Congenital Disease	ICD10	Q819	Epidermolysis bullosa (disorder)
Congenital Disease	ICD10	Q820	Cholestasis-edema syndrome, Norwegian type (disorder)
Congenital Disease	ICD10	Q821	Xeroderma pigmentosum, group B (disorder)
Congenital Disease	ICD10	Q822	Reactive mastocytosis (disorder)
Congenital Disease	ICD10	Q823	Incontinentia pigmenti achromians syndrome (disorder)
Congenital Disease	ICD10	Q824	Xeroderma, talipes and enamel defect syndrome (disorder)
Congenital Disease	ICD10	Q825	Cutis marmorata (finding)
Congenital Disease	ICD10	Q828	Congenital deficiency of pigment of skin (disorder)
Congenital Disease	ICD10	Q829	Congenital anomaly of skin (disorder)
Congenital Disease	ICD10	Q830	Congenital absence of breast with absent nipple (disorder)
Congenital Disease	ICD10	Q831	Accessory breast (disorder)
Congenital Disease	ICD10	Q832	Congenital absence of nipple (disorder)
Congenital Disease	ICD10	Q833	Accessory nipple (disorder)
Congenital Disease	ICD10	Q838	Ectopic breast tissue (disorder)
Congenital Disease	ICD10	Q839	Congenital anomaly of breast (disorder)
Congenital Disease	ICD10	Q840	Congenital alopecia (disorder)
Congenital Disease	ICD10	Q841	Pili torti (disorder)
Congenital Disease	ICD10	Q842	Congenital hypertrichosis (disorder)
Congenital Disease	ICD10	Q843	Anonychia (disorder)
Congenital Disease	ICD10	Q844	Knuckle pads, leukonychia, sensorineural deafness, palmoplantar hyperkeratosis syndrome (disorder)
Congenital Disease	ICD10	Q845	Pachyonychia congenita syndrome (disorder)
Congenital Disease	ICD10	Q846	Congenital koilonychia (disorder)
Congenital Disease	ICD10	Q848	Aplasia cutis congenita (disorder)
Congenital Disease	ICD10	Q849	Congenital anomaly of integument (disorder)
Congenital Disease	ICD10	Q850	Neurofibromatosis syndrome (disorder)
Congenital Disease	ICD10	Q851	Tuberous sclerosis syndrome (disorder)
Congenital Disease	ICD10	Q858	Sturge-Weber syndrome (disorder)
Congenital Disease	ICD10	Q859	Neurocutaneous syndrome (disorder)
Congenital Disease	ICD10	Q860	Fetal alcohol syndrome (disorder)
Congenital Disease	ICD10	Q861	Fetal hydantoin syndrome (disorder)
Congenital Disease	ICD10	Q868	Fetal valproate syndrome (disorder)
Congenital Disease	ICD10	Q870	Melnick-Fraser syndrome (disorder)
Congenital Disease	ICD10	Q871	Dubowitz's syndrome (disorder)
Congenital Disease	ICD10	Q872	Vertebral abnormalities, anal atresia, cardiac abnormalities, tracheo-esophageal fistula, limb defects syndrome (disorder)
Congenital Disease	ICD10	Q873	Proteus syndrome (disorder)
Congenital Disease	ICD10	Q874	Marfan's syndrome (disorder)
Congenital Disease	ICD10	Q875	Caudal regression syndrome (disorder)
Congenital Disease	ICD10	Q878	Senter syndrome (disorder)
Congenital Disease	ICD10	Q890	Accessory spleen (disorder)
Congenital Disease	ICD10	Q891	Accessory adrenal cortex (disorder)

Congenital Disease	ICD10	Q892	X-linked absence of thyroxine-binding globulin (disorder)
Congenital Disease	ICD10	Q893	Situs ambiguus (disorder)
Congenital Disease	ICD10	Q894	Thoracodidymus (disorder)
Congenital Disease	ICD10	Q897	Multiple malformation syndrome with senile-like appearance (disorder)
Congenital Disease	ICD10	Q898	Monocephalus tripus dibrachius (disorder)
Congenital Disease	ICD10	Q899	Hemoglobin E disease (disorder)
Congenital Disease	ICD10	Q900	Trisomy 21- meiotic nondisjunction (disorder)
Congenital Disease	ICD10	Q901	Trisomy 21- mitotic nondisjunction mosaicism (disorder)
Congenital Disease	ICD10	Q902	Translocation Down syndrome (disorder)
Congenital Disease	ICD10	Q909	Complete trisomy 21 syndrome (disorder)
Congenital Disease	ICD10	Q910	Trisomy 18 - meiotic nondisjunction (disorder)
Congenital Disease	ICD10	Q911	Trisomy 18 - mitotic nondisjunction mosaicism (disorder)
Congenital Disease	ICD10	Q913	18p partial trisomy syndrome (disorder)
Congenital Disease	ICD10	Q914	Trisomy 13, meiotic nondisjunction (disorder)
Congenital Disease	ICD10	Q915	Trisomy 13 - mitotic nondisjunction mosaicism (disorder)
Congenital Disease	ICD10	Q917	13p partial trisomy syndrome (disorder)
Congenital Disease	ICD10	Q920	Whole chromosome trisomy meiotic nondisjunction (disorder)
Congenital Disease	ICD10	Q921	Whole chromosome trisomy, mosaicism (disorder)
Congenital Disease	ICD10	Q922	Major partial trisomy (disorder)
Congenital Disease	ICD10	Q923	Minor partial trisomy (disorder)
Congenital Disease	ICD10	Q924	Duplication seen only at prometaphase (disorder)
Congenital Disease	ICD10	Q925	Duplication with other complex rearrangement (disorder)
Congenital Disease	ICD10	Q926	Familial extra unidentified structurally abnormal chromosome (disorder)
Congenital Disease	ICD10	Q927	Triploidy, diploidy, mixoploidy syndrome (disorder)
Congenital Disease	ICD10	Q928	2p partial trisomy syndrome (disorder)
Congenital Disease	ICD10	Q929	Whole chromosome trisomy syndrome (disorder)
Congenital Disease	ICD10	Q930	Complete monosomy 21 (disorder)
Congenital Disease	ICD10	Q931	Monosomy 21, mosaicism (disorder)
Congenital Disease	ICD10	Q932	Ring chromosome 22 syndrome (disorder)
Congenital Disease	ICD10	Q933	4p partial monosomy syndrome (disorder)
Congenital Disease	ICD10	Q934	Cri du chat (finding)
Congenital Disease	ICD10	Q935	11p partial monosomy syndrome (disorder)
Congenital Disease	ICD10	Q936	Deletion seen only at prometaphase (disorder)
Congenital Disease	ICD10	Q937	Deletion with complex rearrangement (disorder)
Congenital Disease	ICD10	Q938	18q partial monosomy syndrome (disorder)
Congenital Disease	ICD10	Q939	Monosomy and deletion from autosome (disorder)
Congenital Disease	ICD10	Q950	Balanced translocation and insertion in normal individual (disorder)
Congenital Disease	ICD10	Q951	Chromosome inversion in normal individual (disorder)
Congenital Disease	ICD10	Q952	Balanced autosomal rearrangement in abnormal individual (disorder)
Congenital Disease	ICD10	Q953	Balanced sex/autosomal rearrangement in abnormal individual (disorder)
Congenital Disease	ICD10	Q954	Individual with marker heterochromatin (disorder)
Congenital Disease	ICD10	Q955	Individual with autosomal fragile site (disorder)
Congenital Disease	ICD10	Q959	Balanced autosomal translocation (disorder)
Congenital Disease	ICD10	Q961	Karyotype 46, X iso (Xq) (disorder)
Congenital Disease	ICD10	Q962	Karyotype 46, X with abnormal sex chromosome except iso (Xq) (disorder)
Congenital Disease	ICD10	Q963	Mixed gonadal dysgenesis (disorder)
Congenital Disease	ICD10	Q964	Mosaicism 45, X or other cell line with abnormal sex chromosome (disorder)
Congenital Disease	ICD10	Q968	Turner's phenotype, karyotype normal (disorder)
Congenital Disease	ICD10	Q969	Turner syndrome (disorder)
Congenital Disease	ICD10	Q970	Trisomy X syndrome (disorder)
Congenital Disease	ICD10	Q971	Four X syndrome (disorder)
Congenital Disease	ICD10	Q972	Mosaicism - lines with various numbers of X chromosomes (disorder)
Congenital Disease	ICD10	Q973	XY females (disorder)
Congenital Disease	ICD10	Q979	Anomaly of chromosome X (disorder)
Congenital Disease	ICD10	Q980	Mosaic XY/XXY (disorder)
Congenital Disease	ICD10	Q981	XXXXY syndrome (disorder)
Congenital Disease	ICD10	Q982	Klinefelter syndrome, male with 46,XX karyotype (disorder)
Congenital Disease	ICD10	Q983	XX males (disorder)
Congenital Disease	ICD10	Q984	Klinefelter syndrome (disorder)
Congenital Disease	ICD10	Q985	Double Y syndrome (disorder)
Congenital Disease	ICD10	Q986	Male with structurally abnormal sex chromosome (disorder)
Congenital Disease	ICD10	Q987	Mosaic XO/XX (disorder)
Congenital Disease	ICD10	Q988	XXYY syndrome (disorder)
Congenital Disease	ICD10	Q989	Anomaly of chromosome Y (disorder)
Congenital Disease	ICD10	Q990	Chimera 46, XX; 46, XY (disorder)
Congenital Disease	ICD10	Q991	Pure gonadal dysgenesis 46,XX (disorder)
Congenital Disease	ICD10	Q992	Fragile X syndrome (disorder)
Congenital Disease	ICD10	Q998	Isologous chimera (disorder)
Congenital Disease	ICD10	Q999	Autosomal hereditary disorder (disorder)
Congenital Disease	SNOMED	10007009	Coffin-Siris syndrome (disorder)
Congenital Disease	SNOMED	10033001	Ehlers-Danlos syndrome, non hydroxylysine deficient ocular type (disorder)
Congenital Disease	SNOMED	1003322006	Distal deletion of short arm of chromosome 3 (disorder)
Congenital Disease	SNOMED	1003324007	Congenital synostosis of bilateral tibias and fibulas (disorder)
Congenital Disease	SNOMED	1003330007	Simple syndactyly of toes of first web space of bilateral feet (disorder)
Congenital Disease	SNOMED	1003337005	Congenital split of bilateral feet (disorder)

Congenital Disease	SNOMED	1003339008	Congenital split of bilateral hands (disorder)
Congenital Disease	SNOMED	1003358004	Distal deletion of short arm of chromosome 8 (disorder)
Congenital Disease	SNOMED	1003364006	Distal deletion of chromosome 13 (disorder)
Congenital Disease	SNOMED	1003369001	Microphthalmos due to Delleman syndrome (disorder)
Congenital Disease	SNOMED	1003370000	Microphthalmos due to Fryns syndrome (disorder)
Congenital Disease	SNOMED	1003371001	Micromelic dwarfism Fryn type (disorder)
Congenital Disease	SNOMED	1003372008	Microphthalmos due to branchio-oculo-facial syndrome (disorder)
Congenital Disease	SNOMED	1003373003	Microcephaly with simplified gyral pattern (disorder)
Congenital Disease	SNOMED	1003374009	Microlissencephaly (disorder)
Congenital Disease	SNOMED	1003376006	Medial duplication of long arm of chromosome 9 (disorder)
Congenital Disease	SNOMED	1003377002	Medial duplication of short arm of chromosome 1 (disorder)
Congenital Disease	SNOMED	1003378007	Optic nerve hypoplasia due to endocrine deficiency (disorder)
Congenital Disease	SNOMED	1003379004	Osteogenesis imperfecta type 5 (disorder)
Congenital Disease	SNOMED	1003380001	6q16 microdeletion syndrome (disorder)
Congenital Disease	SNOMED	1003384005	Multiple congenital defects of vertebral segmentation (disorder)
Congenital Disease	SNOMED	1003389000	Mosaic 1q duplication (disorder)
Congenital Disease	SNOMED	1003391008	Medial deletion of long arm of chromosome 4 (disorder)
Congenital Disease	SNOMED	1003392001	Medial deletion of long arm of chromosome 5 (disorder)
Congenital Disease	SNOMED	1003393006	Medial deletion of chromosome 14 (disorder)
Congenital Disease	SNOMED	1003394000	Medial deletion of long arm of chromosome 2 (disorder)
Congenital Disease	SNOMED	1003395004	Maternal uniparental disomy of chromosome 7 (disorder)
Congenital Disease	SNOMED	1003396003	Medial deletion of chromosome 13 (disorder)
Congenital Disease	SNOMED	1003397007	Congenital atresia of intestine at multiple levels (disorder)
Congenital Disease	SNOMED	1003398002	Congenital anorectal fistula due to intermediate anorectal malformation (disorder)
Congenital Disease	SNOMED	1003399005	Congenital anomaly of first branchial cleft (disorder)
Congenital Disease	SNOMED	1003400003	Congenital gastric heterotopia of duodenum (disorder)
Congenital Disease	SNOMED	1003402006	Maternal uniparental disomy of chromosome 15 (disorder)
Congenital Disease	SNOMED	1003403001	Maternal uniparental disomy of chromosome 11 (disorder)
Congenital Disease	SNOMED	1003405008	Maternal uniparental disomy of chromosome 14 (disorder)
Congenital Disease	SNOMED	1003407000	Marfan syndrome type 1 (disorder)
Congenital Disease	SNOMED	1003410007	Medial duplication of long arm of chromosome 5 (disorder)
Congenital Disease	SNOMED	1003411006	Medial duplication of long arm of chromosome 7 (disorder)
Congenital Disease	SNOMED	1003412004	Medial duplication of long arm of chromosome 2 (disorder)
Congenital Disease	SNOMED	1003413009	Medial duplication of long arm of chromosome 4 (disorder)
Congenital Disease	SNOMED	1003414003	Medial duplication of chromosome 14 (disorder)
Congenital Disease	SNOMED	1003415002	Medial duplication of long arm of chromosome 1 (disorder)
Congenital Disease	SNOMED	1003416001	Medial duplication of chromosome 13 (disorder)
Congenital Disease	SNOMED	1003417005	Medial deletion of short arm of chromosome 1 (disorder)
Congenital Disease	SNOMED	1003418000	Medial deletion of long arm of chromosome 1 (disorder)
Congenital Disease	SNOMED	1003419008	Medial deletion of long arm of chromosome 7 (disorder)
Congenital Disease	SNOMED	1003420002	Medial deletion of long arm of chromosome 9 (disorder)
Congenital Disease	SNOMED	1003421003	Intra-abdominal omphalomesenteric duct cyst (disorder)
Congenital Disease	SNOMED	1003422005	Hypoplasia of optic nerve due to central nervous system malformation (disorder)
Congenital Disease	SNOMED	1003424006	Hutterite type cataract (disorder)
Congenital Disease	SNOMED	1003427004	Genochondromatosis type 1 (disorder)
Congenital Disease	SNOMED	1003428009	Glaucoma due to congenital anomaly of eye (disorder)
Congenital Disease	SNOMED	1003429001	Focal cortical dysplasia type IIa (disorder)
Congenital Disease	SNOMED	1003430006	Focal cortical dysplasia type IIb (disorder)
Congenital Disease	SNOMED	1003431005	Mandibuloacral dysostosis co-occurrent with type A lipodystrophy (disorder)
Congenital Disease	SNOMED	1003432003	Mandibuloacral dysostosis co-occurrent with type B lipodystrophy (disorder)
Congenital Disease	SNOMED	1003433008	Malrotation of small intestine (disorder)
Congenital Disease	SNOMED	1003434002	Lipoma due to neurospinal dysraphism (disorder)
Congenital Disease	SNOMED	1003435001	Linear basal cell nevus (disorder)
Congenital Disease	SNOMED	1003437009	Leydig cell hypoplasia due to complete luteinizing hormone receptor inactivation (disorder)
Congenital Disease	SNOMED	1003438004	Leydig cell hypoplasia due to partial luteinizing hormone receptor inactivation (disorder)
Congenital Disease	SNOMED	1003439007	Intra-abdominal vitelline remnant (disorder)
Congenital Disease	SNOMED	1003440009	Laryngeal cleft type 0 (disorder)
Congenital Disease	SNOMED	1003442001	Synostosis of bilateral humerus radius and ulna (disorder)
Congenital Disease	SNOMED	1003443006	Congenital stenosis of male external urethral orifice (disorder)
Congenital Disease	SNOMED	1003444000	Type 3 lissencephaly (disorder)
Congenital Disease	SNOMED	1003445004	Lumbosacral spina bifida aperta with hydrocephalus (disorder)
Congenital Disease	SNOMED	1003446003	Synostosis of bilateral humerus and ulna (disorder)
Congenital Disease	SNOMED	1003448002	Lumbosacral spina bifida aperta (disorder)
Congenital Disease	SNOMED	1003449005	Paternal 14q32.2 microdeletion (disorder)
Congenital Disease	SNOMED	1003461002	Focal cortical dysplasia type II (disorder)
Congenital Disease	SNOMED	1003462009	Focal cortical dysplasia type Ib (disorder)
Congenital Disease	SNOMED	1003463004	Focal cortical dysplasia type I (disorder)
Congenital Disease	SNOMED	1003464005	Focal cortical dysplasia type Ia (disorder)
Congenital Disease	SNOMED	1003465006	Familial spinal neurofibromatosis (disorder)
Congenital Disease	SNOMED	1003505005	Agenesis of rib (disorder)
Congenital Disease	SNOMED	1003507002	Agenesis of lobe of lung (disorder)

Congenital Disease	SNOMED	1003509004	Agenesis of foot (disorder)
Congenital Disease	SNOMED	1003510009	Agenesis of hand (disorder)
Congenital Disease	SNOMED	1003512001	Agenesis of ovary (disorder)
Congenital Disease	SNOMED	1003513006	Agenesis of femur (disorder)
Congenital Disease	SNOMED	1003514000	Agenesis of liver (disorder)
Congenital Disease	SNOMED	1003515004	Agenesis of tibia (disorder)
Congenital Disease	SNOMED	1003516003	Agenesis of ilium (disorder)
Congenital Disease	SNOMED	1003518002	Agenesis of pubis (disorder)
Congenital Disease	SNOMED	1003519005	Agenesis of talus (disorder)
Congenital Disease	SNOMED	1003520004	Agenesis of vomer (disorder)
Congenital Disease	SNOMED	1003546000	Agenesis of fibula (disorder)
Congenital Disease	SNOMED	1003547009	Agenesis of ureter (disorder)
Congenital Disease	SNOMED	1003548004	Agenesis of testis (disorder)
Congenital Disease	SNOMED	1003549007	Agenesis of radius (disorder)
Congenital Disease	SNOMED	1003550007	Agenesis of thymus (disorder)
Congenital Disease	SNOMED	1003551006	Agenesis of spleen (disorder)
Congenital Disease	SNOMED	1003552004	Agenesis of bladder (disorder)
Congenital Disease	SNOMED	1003553009	Agenesis of cilia of eyelid (disorder)
Congenital Disease	SNOMED	1003554003	Agenesis of humerus (disorder)
Congenital Disease	SNOMED	1003555002	Agenesis of trachea (disorder)
Congenital Disease	SNOMED	1003556001	Agenesis of stomach (disorder)
Congenital Disease	SNOMED	1003557005	Agenesis of ischium (disorder)
Congenital Disease	SNOMED	1003559008	Agenesis of scapula (disorder)
Congenital Disease	SNOMED	1003563001	Agenesis of vertebra (disorder)
Congenital Disease	SNOMED	1003564007	Agenesis of clitoris (disorder)
Congenital Disease	SNOMED	1003567000	Agenesis of bile duct (disorder)
Congenital Disease	SNOMED	1003568005	Agenesis of diaphragm (disorder)
Congenital Disease	SNOMED	1003569002	Agenesis of esophagus (disorder)
Congenital Disease	SNOMED	1003571002	Agenesis of calcaneus (disorder)
Congenital Disease	SNOMED	1003573004	Agenesis of sternebra (disorder)
Congenital Disease	SNOMED	1003574005	Agenesis of left hand (disorder)
Congenital Disease	SNOMED	1003575006	Agenesis of epididymis (disorder)
Congenital Disease	SNOMED	1003576007	Agenesis of hyoid bone (disorder)
Congenital Disease	SNOMED	1003577003	Agenesis of nasal bone (disorder)
Congenital Disease	SNOMED	1003579000	Agenesis of right hand (disorder)
Congenital Disease	SNOMED	1003581003	Agenesis of carpal bone (disorder)
Congenital Disease	SNOMED	1003582005	Agenesis of tarsal bone (disorder)
Congenital Disease	SNOMED	1003583000	Agenesis of bilateral testes (disorder)
Congenital Disease	SNOMED	1003620005	Congenital clinodactyly of finger (disorder)
Congenital Disease	SNOMED	1003621009	Congenital clinodactyly of little finger (disorder)
Congenital Disease	SNOMED	1003644007	Agenesis of bilateral feet (disorder)
Congenital Disease	SNOMED	1003657004	Congenital glaucoma of left eye (disorder)
Congenital Disease	SNOMED	1003704006	Agenesis of metacarpal bone (disorder)
Congenital Disease	SNOMED	1003841002	Remnant of vitelline vein (disorder)
Congenital Disease	SNOMED	1003842009	Renal tubular dysgenesis caused by drug (disorder)
Congenital Disease	SNOMED	1003858007	Rhizomelic chondrodysplasia punctata type 3 (disorder)
Congenital Disease	SNOMED	1003860009	Rhizomelic chondrodysplasia punctata type 2 (disorder)
Congenital Disease	SNOMED	1003861008	Renal tubular dysgenesis due to twin to twin transfusion (disorder)
Congenital Disease	SNOMED	1003862001	Rhizomelic chondrodysplasia punctata type 1 (disorder)
Congenital Disease	SNOMED	1003864000	Proximal duplication of long arm of chromosome 5 (disorder)
Congenital Disease	SNOMED	1003865004	Proximal duplication of long arm of chromosome 6 (disorder)
Congenital Disease	SNOMED	1003866003	Proximal duplication of long arm of chromosome 3 (disorder)
Congenital Disease	SNOMED	1003867007	Proximal duplication of long arm of chromosome 4 (disorder)
Congenital Disease	SNOMED	1003868002	Proximal duplication of long arm of chromosome 18 (disorder)
Congenital Disease	SNOMED	1003869005	Proximal duplication of long arm of chromosome 2 (disorder)
Congenital Disease	SNOMED	1003870006	Uniparental disomy of paternal origin of chromosome 4 (disorder)
Congenital Disease	SNOMED	1003871005	Uniparental disomy of paternal origin of chromosome 15 (disorder)
Congenital Disease	SNOMED	1003872003	Uniparental disomy of paternal origin of chromosome 14 (disorder)
Congenital Disease	SNOMED	1003873008	Uniparental disomy of paternal origin of chromosome 11 (disorder)
Congenital Disease	SNOMED	1003875001	Proximal duplication of long arm of chromosome 17 (disorder)
Congenital Disease	SNOMED	1003876000	Proximal duplication of long arm of chromosome 12 (disorder)
Congenital Disease	SNOMED	1003877009	Pfeiffer syndrome type 1 (disorder)
Congenital Disease	SNOMED	1003878004	Proximal duplication of long arm of chromosome 16 (disorder)
Congenital Disease	SNOMED	1003879007	Megalourethra of spongiose portion of urethra (disorder)
Congenital Disease	SNOMED	1003880005	Proximal duplication of long arm of chromosome 10 (disorder)
Congenital Disease	SNOMED	1003882002	Proximal duplication of long arm of chromosome 11 (disorder)
Congenital Disease	SNOMED	1003883007	Proximal duplication of short arm of chromosome 9 (disorder)
Congenital Disease	SNOMED	1003884001	Pulverulent cataract (disorder)
Congenital Disease	SNOMED	1003885000	Proximal duplication of short arm of chromosome 7 (disorder)
Congenital Disease	SNOMED	1003886004	Proximal duplication of short arm of chromosome 8 (disorder)
Congenital Disease	SNOMED	1003887008	Proximal duplication of short arm of chromosome 3 (disorder)
Congenital Disease	SNOMED	1003888003	Proximal duplication of short arm of chromosome 6 (disorder)
Congenital Disease	SNOMED	1003889006	Proximal duplication of short arm of chromosome 2 (disorder)
Congenital Disease	SNOMED	1003890002	Proximal duplication of long arm of chromosome 9 (disorder)
Congenital Disease	SNOMED	1003891003	Proximal duplication of short arm of chromosome 1 (disorder)

Congenital Disease	SNOMED	1003892005	Proximal duplication of long arm of chromosome 7 (disorder)
Congenital Disease	SNOMED	1003893000	Proximal duplication of long arm of chromosome 8 (disorder)
Congenital Disease	SNOMED	1003894006	Proximal deletion of long arm of chromosome 8 (disorder)
Congenital Disease	SNOMED	1003895007	Proximal deletion of long arm of chromosome 9 (disorder)
Congenital Disease	SNOMED	1003896008	Proximal deletion of long arm of chromosome 6 (disorder)
Congenital Disease	SNOMED	1003897004	Proximal deletion of long arm of chromosome 7 (disorder)
Congenital Disease	SNOMED	1003898009	Proximal deletion of long arm of chromosome 5 (disorder)
Congenital Disease	SNOMED	1003899001	Proximal deletion of long arm of chromosome 3 (disorder)
Congenital Disease	SNOMED	1003900006	Proximal deletion of long arm of chromosome 4 (disorder)
Congenital Disease	SNOMED	1003901005	Proximal deletion of long arm of chromosome 18 (disorder)
Congenital Disease	SNOMED	1003902003	Proximal deletion of long arm of chromosome 2 (disorder)
Congenital Disease	SNOMED	1003903008	Proximal deletion of long arm of chromosome 16 (disorder)
Congenital Disease	SNOMED	1003904002	Proximal deletion of long arm of chromosome 17 (disorder)
Congenital Disease	SNOMED	1003905001	Proximal duplication of chromosome 21 (disorder)
Congenital Disease	SNOMED	1003906000	Proximal duplication of long arm of chromosome 1 (disorder)
Congenital Disease	SNOMED	1003907009	Proximal duplication of chromosome 14 (disorder)
Congenital Disease	SNOMED	1003908004	Proximal duplication of chromosome 15 (disorder)
Congenital Disease	SNOMED	1003909007	Proximal duplication of chromosome 13 (disorder)
Congenital Disease	SNOMED	1003910002	Proximal deletion of short arm of chromosome 8 (disorder)
Congenital Disease	SNOMED	1003911003	Proximal deletion of short arm of chromosome 9 (disorder)
Congenital Disease	SNOMED	1003912005	Proximal deletion of short arm of chromosome 6 (disorder)
Congenital Disease	SNOMED	1003913000	Proximal deletion of short arm of chromosome 7 (disorder)
Congenital Disease	SNOMED	1003914006	Proximal deletion of short arm of chromosome 1 (disorder)
Congenital Disease	SNOMED	1003915007	Proximal deletion of short arm of chromosome 3 (disorder)
Congenital Disease	SNOMED	1003916008	Pfeiffer syndrome type 2 (disorder)
Congenital Disease	SNOMED	1003917004	Secondary congenital hyperplasia of lung (disorder)
Congenital Disease	SNOMED	1003918009	Pfeiffer syndrome type 3 (disorder)
Congenital Disease	SNOMED	1003920007	Sabinas brittle hair syndrome (disorder)
Congenital Disease	SNOMED	1003921006	Sclerocornea of bilateral corneas (disorder)
Congenital Disease	SNOMED	1003922004	Rothmund Thomson syndrome type 1 (disorder)
Congenital Disease	SNOMED	1003923009	Rothmund Thomson syndrome type 2 (disorder)
Congenital Disease	SNOMED	1003929008	Proximal deletion of long arm of chromosome 11 (disorder)
Congenital Disease	SNOMED	1003930003	Proximal deletion of long arm of chromosome 12 (disorder)
Congenital Disease	SNOMED	1003931004	Proximal deletion of long arm of chromosome 10 (disorder)
Congenital Disease	SNOMED	1003932006	Proximal deletion of chromosome 21 (disorder)
Congenital Disease	SNOMED	1003933001	Proximal deletion of long arm of chromosome 1 (disorder)
Congenital Disease	SNOMED	1003934007	Proximal deletion of chromosome 14 (disorder)
Congenital Disease	SNOMED	1003935008	Proximal deletion of chromosome 15 (disorder)
Congenital Disease	SNOMED	1003936009	Primary congenital hyperplasia of lung (disorder)
Congenital Disease	SNOMED	1003937000	Proximal deletion of chromosome 13 (disorder)
Congenital Disease	SNOMED	1003939002	Polydactyly of bilateral index fingers (disorder)
Congenital Disease	SNOMED	1010212005	Congenital dysplasia of left hip (disorder)
Congenital Disease	SNOMED	1010213000	Congenital dysplasia of right hip (disorder)
Congenital Disease	SNOMED	1010215007	Congenital subluxation of left hip joint (disorder)
Congenital Disease	SNOMED	1010217004	Congenital subluxation of right hip joint (disorder)
Congenital Disease	SNOMED	1010276004	Ring chromosome (disorder)
Congenital Disease	SNOMED	1010464002	Agenesis of right hemisphere of cerebellum (disorder)
Congenital Disease	SNOMED	1010465001	Agenesis of left hemisphere of cerebellum (disorder)
Congenital Disease	SNOMED	1010568002	Undescended left testicle (disorder)
Congenital Disease	SNOMED	1010569005	Undescended right testicle (disorder)
Congenital Disease	SNOMED	1010604007	Ventriculomegaly due to developmental anomaly (disorder)
Congenital Disease	SNOMED	1010609002	Mesomelic dysplasia of upper limb (disorder)
Congenital Disease	SNOMED	1010611006	Thoracoomphalopagus (disorder)
Congenital Disease	SNOMED	1010613009	Tetrasomy 15q (disorder)
Congenital Disease	SNOMED	1010614003	Tetrasomy 5p mosaicism (disorder)
Congenital Disease	SNOMED	1010618000	Syndactyly of fingers of bilateral hands (disorder)
Congenital Disease	SNOMED	1010628009	X-linked congenital generalized hypertrichosis (disorder)
Congenital Disease	SNOMED	1010630006	X-linked complicated corpus callosum dysgenesis (disorder)
Congenital Disease	SNOMED	1010639007	Anomaly of umbilical vein group I (disorder)
Congenital Disease	SNOMED	1010641008	Anomaly of umbilical vein group II (disorder)
Congenital Disease	SNOMED	1010643006	Thoracolumbosacral spina bifida aperta (disorder)
Congenital Disease	SNOMED	1010644000	Spina bifida aperta of upper thoracic spine (disorder)
Congenital Disease	SNOMED	1010645004	Single umbilical cord artery type III (disorder)
Congenital Disease	SNOMED	1010646003	Single umbilical cord artery type IV (disorder)
Congenital Disease	SNOMED	1010652002	Single umbilical cord artery type II (disorder)
Congenital Disease	SNOMED	1010653007	Segmental neurofibromatosis type 1 (disorder)
Congenital Disease	SNOMED	1010655000	Single umbilical cord artery type I (disorder)
Congenital Disease	SNOMED	1010657008	Secondary hypoplasia of bilateral lungs (disorder)
Congenital Disease	SNOMED	1010663004	Subcortical nodular heterotopia (disorder)
Congenital Disease	SNOMED	1010664005	Stickler syndrome type 2 (disorder)
Congenital Disease	SNOMED	1010665006	Helical ulceration of umbilical cord (disorder)
Congenital Disease	SNOMED	1010666007	Stickler syndrome type 4 (disorder)
Congenital Disease	SNOMED	1010667003	Sternopagus (disorder)
Congenital Disease	SNOMED	1010668008	Stickler syndrome type 1 (disorder)
Congenital Disease	SNOMED	1010678006	Anomaly of umbilical vein group III (disorder)

Congenital Disease	SNOMED	1010680000	Anomaly of umbilical vein group IV (disorder)
Congenital Disease	SNOMED	1010685005	Oculo-auriculo-vertebral spectrum (disorder)
Congenital Disease	SNOMED	1010709006	Longitudinal deficiency of bilateral fibulae (disorder)
Congenital Disease	SNOMED	10155006	Bilateral congenital dislocation of hip (disorder)
Congenital Disease	SNOMED	10177005	Triploidy, diploidy, mixoploidy syndrome (disorder)
Congenital Disease	SNOMED	10283004	Congenital stenosis of cervical canal (disorder)
Congenital Disease	SNOMED	10294000	Reactive mastocytosis (disorder)
Congenital Disease	SNOMED	103019006	Bulging fontanelle (finding)
Congenital Disease	SNOMED	10331000132107	Tortuous colon (disorder)
Congenital Disease	SNOMED	10362008	Accessory spleen (disorder)
Congenital Disease	SNOMED	10375008	Extrapulmonary subpleural pulmonary sequestration (disorder)
Congenital Disease	SNOMED	10451007	Double aortic arch (disorder)
Congenital Disease	SNOMED	1052326006	Rachischisis partialis (disorder)
Congenital Disease	SNOMED	10567003	Four X syndrome (disorder)
Congenital Disease	SNOMED	10572007	13q partial trisomy syndrome (disorder)
Congenital Disease	SNOMED	105985007	Osteochondrodysplasia syndrome (disorder)
Congenital Disease	SNOMED	105986008	Congenital skeletal dysplasia (disorder)
Congenital Disease	SNOMED	105989001	Malformation sequence (disorder)
Congenital Disease	SNOMED	10624871000119109	Congenital skin contracture (disorder)
Congenital Disease	SNOMED	10631000	Trilobed left lung (disorder)
Congenital Disease	SNOMED	1073003	Xeroderma pigmentosum, group B (disorder)
Congenital Disease	SNOMED	10736081000119101	Congenital atresia of vagina (disorder)
Congenital Disease	SNOMED	10736711000119105	Congenital hypoplasia of uterine cervix (disorder)
Congenital Disease	SNOMED	10738211000119102	Congenital fistula between uterus and urinary tract (disorder)
Congenital Disease	SNOMED	1078151000119103	Congenital hallux valgus of left great toe (disorder)
Congenital Disease	SNOMED	1078161000119101	Congenital hallux valgus of right great toe (disorder)
Congenital Disease	SNOMED	1078191000119108	Congenital shortening of right Achilles tendon (disorder)
Congenital Disease	SNOMED	1078201000119106	Congenital shortening of left Achilles tendon (disorder)
Congenital Disease	SNOMED	1078251000119105	Congenital absence of right forearm and hand (disorder)
Congenital Disease	SNOMED	1078261000119107	Congenital absence of right hand (disorder)
Congenital Disease	SNOMED	1078361000119103	Congenital genu varum of left knee (disorder)
Congenital Disease	SNOMED	1078371000119109	Congenital genu varum of right knee (disorder)
Congenital Disease	SNOMED	10805641000119102	Congenital cardiovascular anomaly in mother complicating childbirth (disorder)
Congenital Disease	SNOMED	10818008	Congenital malposition of heart (disorder)
Congenital Disease	SNOMED	10835621000119104	Congenital anomaly of vagina in mother complicating pregnancy (disorder)
Congenital Disease	SNOMED	10835661000119109	Doubling of uterus with doubling of cervix and vagina with obstruction (disorder)
Congenital Disease	SNOMED	10835701000119102	Congenital anomaly of vulva in mother complicating pregnancy (disorder)
Congenital Disease	SNOMED	1085291000119101	Cleft hard and soft palate with cleft lip (disorder)
Congenital Disease	SNOMED	1085301000119100	Cleft of hard palate and cleft lip (disorder)
Congenital Disease	SNOMED	1085331000119107	Cleft palate and bilateral cleft lip (disorder)
Congenital Disease	SNOMED	1085341000119103	Cleft of soft palate and bilateral cleft lip (disorder)
Congenital Disease	SNOMED	1085351000119101	Cleft of soft palate and cleft lip (disorder)
Congenital Disease	SNOMED	1085651000119109	Congenital anal fissure (disorder)
Congenital Disease	SNOMED	1085671000119100	Congenital malformation of larynx and trachea (disorder)
Congenital Disease	SNOMED	1085681000119102	Congenital rectal fissure (disorder)
Congenital Disease	SNOMED	1085711000119101	Congenital split ear lobe (disorder)
Congenital Disease	SNOMED	108761000119101	Congenital internal tibial torsion (finding)
Congenital Disease	SNOMED	1088611000119103	Congenital split left ear lobe (disorder)
Congenital Disease	SNOMED	1089891000119102	Misshapen ear (disorder)
Congenital Disease	SNOMED	1091221000119100	Congenital split right ear lobe (disorder)
Congenital Disease	SNOMED	10930001	Congenital atresia of pulmonary artery (disorder)
Congenital Disease	SNOMED	109393007	Otomandibular dysostosis (disorder)
Congenital Disease	SNOMED	109394001	Intermandibular dysostosis (disorder)
Congenital Disease	SNOMED	109395000	Temporo-auro-mandibular dysostosis (disorder)
Congenital Disease	SNOMED	109397008	Temporo-aural dysostosis (disorder)
Congenital Disease	SNOMED	109398003	Maxillary dysostosis (disorder)
Congenital Disease	SNOMED	109399006	Maxillo-zygomatic dysostosis (disorder)
Congenital Disease	SNOMED	109400004	Naso-maxillary dysostosis (disorder)
Congenital Disease	SNOMED	109402007	Anterior perimaxillary faciosynostosis (disorder)
Congenital Disease	SNOMED	109403002	Complete perimaxillary faciosynostosis (disorder)
Congenital Disease	SNOMED	109404008	Posterior perimaxillary faciosynostosis (disorder)
Congenital Disease	SNOMED	109406005	Vomero-premaxillary faciosynostosis (disorder)
Congenital Disease	SNOMED	109407001	Internasal dysostosis (disorder)
Congenital Disease	SNOMED	109408006	Frontal dysostosis (disorder)
Congenital Disease	SNOMED	109409003	Interfrontal craniofaciosynostosis (disorder)
Congenital Disease	SNOMED	109410008	Fronto-frontal dysostosis (disorder)
Congenital Disease	SNOMED	109411007	Fronto-naso-ethmoidal dysostosis (disorder)
Congenital Disease	SNOMED	109413005	Fronto-malar faciosynostosis (disorder)
Congenital Disease	SNOMED	109414004	Sphenoidal dysostosis (disorder)
Congenital Disease	SNOMED	109415003	Spheno-frontal dysostosis (disorder)
Congenital Disease	SNOMED	109416002	Spheno-fronto-parietal craniofaciosynostosis (disorder)
Congenital Disease	SNOMED	109417006	Parieto-occipital craniosynostosis (disorder)
Congenital Disease	SNOMED	109418001	Interparietal craniosynostosis (disorder)

Congenital Disease	SNOMED	109419009	Mandibuloacral dysostosis (disorder)
Congenital Disease	SNOMED	109420003	Dysostosis (disorder)
Congenital Disease	SNOMED	109422006	Accessory cuboid bone (disorder)
Congenital Disease	SNOMED	109423001	Accessory navicular bone of foot (disorder)
Congenital Disease	SNOMED	109425008	Single right ventricle (disorder)
Congenital Disease	SNOMED	109426009	Single left ventricle (disorder)
Congenital Disease	SNOMED	109427000	Supracristal ventricular septal defect (disorder)
Congenital Disease	SNOMED	109428005	Perimembranous ventricular septal defect (disorder)
Congenital Disease	SNOMED	109429002	Single muscular ventricular septum defect (disorder)
Congenital Disease	SNOMED	109430007	Multiple muscular ventricular septum defect (disorder)
Congenital Disease	SNOMED	109431006	Ebstein-like malformation of mitral valve (disorder)
Congenital Disease	SNOMED	109432004	Anomalous cardiac muscle bands (disorder)
Congenital Disease	SNOMED	109434003	Congenital anomaly of oral mucosa (disorder)
Congenital Disease	SNOMED	109478007	Kohlschutter's syndrome (disorder)
Congenital Disease	SNOMED	109513007	Congenital mandibular asymmetry (disorder)
Congenital Disease	SNOMED	109514001	Congenital maxillary asymmetry (disorder)
Congenital Disease	SNOMED	109546001	Cleft of primary palate (disorder)
Congenital Disease	SNOMED	109548000	Bilateral cleft of primary palate (disorder)
Congenital Disease	SNOMED	109549008	Congenital anomaly of lip (disorder)
Congenital Disease	SNOMED	109550008	Congenital commissural pits (disorder)
Congenital Disease	SNOMED	109551007	Aberrant insertion of labial frenulum (disorder)
Congenital Disease	SNOMED	109556002	Aberrant insertion of frenum of tongue (disorder)
Congenital Disease	SNOMED	109557006	Congenital anomaly of uvula (disorder)
Congenital Disease	SNOMED	109559009	Riedel's lobe of liver (disorder)
Congenital Disease	SNOMED	109560004	Cystic testicular dysplasia (disorder)
Congenital Disease	SNOMED	109561000	Cerebrofacial dysplasia (disorder)
Congenital Disease	SNOMED	109725007	Maxillary asymmetry due to hemifacial atrophy (disorder)
Congenital Disease	SNOMED	109726008	Maxillary asymmetry due to hemifacial hypertrophy (disorder)
Congenital Disease	SNOMED	10989008	Talipes equinus (disorder)
Congenital Disease	SNOMED	109905002	Acquired myelocele (disorder)
Congenital Disease	SNOMED	110992006	Bifid nail (disorder)
Congenital Disease	SNOMED	11102005	Congenital fistula of lip (disorder)
Congenital Disease	SNOMED	111030006	Howel-Evans' syndrome (disorder)
Congenital Disease	SNOMED	111246005	Arthrogryposis (disorder)
Congenital Disease	SNOMED	111303009	Sjögren-Larsson syndrome (disorder)
Congenital Disease	SNOMED	111304003	Kozlowski spondylometaphyseal dysplasia (disorder)
Congenital Disease	SNOMED	111309008	8q partial trisomy syndrome (disorder)
Congenital Disease	SNOMED	11131000119108	Anomaly of eye (disorder)
Congenital Disease	SNOMED	111310003	Ring chromosome 11 syndrome (disorder)
Congenital Disease	SNOMED	111311004	20p partial trisomy syndrome (disorder)
Congenital Disease	SNOMED	111312006	Anomaly of chromosome X (disorder)
Congenital Disease	SNOMED	111315008	Longitudinal deficiency of tibia AND/OR fibula (disorder)
Congenital Disease	SNOMED	111317000	Congenital absence of nose (disorder)
Congenital Disease	SNOMED	111318005	Congenital cystic adenomatoid malformation of lung (disorder)
Congenital Disease	SNOMED	111319002	Truncus arteriosus, Edwards' type IV (disorder)
Congenital Disease	SNOMED	111321007	Right aortic arch (disorder)
Congenital Disease	SNOMED	111322000	Congenital anomaly of pulmonary veins (disorder)
Congenital Disease	SNOMED	111323005	Total anomalous pulmonary venous return (disorder)
Congenital Disease	SNOMED	111324004	Congenital absence of breast (disorder)
Congenital Disease	SNOMED	111327006	Congenital anomaly of salivary gland (disorder)
Congenital Disease	SNOMED	111328001	Congenital diverticulum of pharynx (disorder)
Congenital Disease	SNOMED	111329009	Congenital prolapsed rectum (disorder)
Congenital Disease	SNOMED	111330004	Congenital rectovaginal fistula (disorder)
Congenital Disease	SNOMED	111331000	Congenital dilatation of lobar intrahepatic bile duct (disorder)
Congenital Disease	SNOMED	111332007	Male pseudohermaphroditism (disorder)
Congenital Disease	SNOMED	111334008	Embryonic cyst of epoophoron (disorder)
Congenital Disease	SNOMED	111335009	Congenital absence of vulva (disorder)
Congenital Disease	SNOMED	111336005	Congenital lateral curvature of penis (disorder)
Congenital Disease	SNOMED	111338006	Agenesis of nerve (disorder)
Congenital Disease	SNOMED	111339003	Congenital anomaly of ear with impairment of hearing (disorder)
Congenital Disease	SNOMED	111341002	Cephalodiprosopus (disorder)
Congenital Disease	SNOMED	111388003	Cutis laxa, autosomal dominant (disorder)
Congenital Disease	SNOMED	111389006	Dominant dystrophic epidermolysis bullosa (disorder)
Congenital Disease	SNOMED	11144004	Congenital stricture of osseous meatus of middle ear (disorder)
Congenital Disease	SNOMED	111504002	Walker-Warburg congenital muscular dystrophy (disorder)
Congenital Disease	SNOMED	111505001	Muscle-eye-brain disease, congenital muscular dystrophy (disorder)
Congenital Disease	SNOMED	111631000119106	Congenital dilatation of renal pelvis (disorder)
Congenital Disease	SNOMED	11164009	Autosomal dominant hereditary disorder (disorder)
Congenital Disease	SNOMED	111641000119102	Congenital choroid plexus cyst (disorder)
Congenital Disease	SNOMED	1119290007	Systematized linear porokeratosis (disorder)
Congenital Disease	SNOMED	1119299008	Mitral regurgitation due to congenital abnormality of mitral subvalvular apparatus (disorder)
Congenital Disease	SNOMED	1119390005	Cleft of right hard palate (disorder)
Congenital Disease	SNOMED	1119391009	Cleft of left hard palate (disorder)
Congenital Disease	SNOMED	1119392002	Complete cleft of right hard and soft palate (disorder)

Congenital Disease	SNOMED	1119393007	Complete cleft of left hard and soft palate (disorder)
Congenital Disease	SNOMED	11194003	Congenital anomaly of anus (disorder)
Congenital Disease	SNOMED	11197005	Hydromyelia (disorder)
Congenital Disease	SNOMED	11211000119108	Congenital anomaly of pupil (disorder)
Congenital Disease	SNOMED	11223009	Congenital anomaly of pharynx (disorder)
Congenital Disease	SNOMED	11266002	Upper esophageal web (disorder)
Congenital Disease	SNOMED	112751000119103	Congenital superior sulcus anomaly of orbit (disorder)
Congenital Disease	SNOMED	112871000119104	Congenital contracture of toe joint (disorder)
Congenital Disease	SNOMED	1131009	Congenital valvular insufficiency (disorder)
Congenital Disease	SNOMED	1137561000	Congenital megacalycosis (disorder)
Congenital Disease	SNOMED	1141000119101	Congenital labial adhesion (disorder)
Congenital Disease	SNOMED	1142089004	Congenital extramedullary dermal hematopoiesis (disorder)
Congenital Disease	SNOMED	11433004	Congenital coronary artery fistula (disorder)
Congenital Disease	SNOMED	1144273005	Fetal sirenomelia (disorder)
Congenital Disease	SNOMED	1144892000	Congenital paucity of intrahepatic bile ducts (disorder)
Congenital Disease	SNOMED	1144984005	Azoospermia due to absent testes (disorder)
Congenital Disease	SNOMED	1145402008	Congenital macrocephaly (disorder)
Congenital Disease	SNOMED	1145403003	Macrocephaly (finding)
Congenital Disease	SNOMED	1145543005	Complete left cleft lip and incomplete right cleft lip (disorder)
Congenital Disease	SNOMED	1145544004	Complete right cleft lip and incomplete left cleft lip (disorder)
Congenital Disease	SNOMED	1148541006	Bilateral incomplete cleft palate and bilateral incomplete cleft lip (disorder)
Congenital Disease	SNOMED	1148757008	Microcephaly (finding)
Congenital Disease	SNOMED	1148758003	Congenital microcephaly (disorder)
Congenital Disease	SNOMED	1148887009	Disorder of glomerulus due to Alport syndrome (disorder)
Congenital Disease	SNOMED	1148891004	Bullous diffuse cutaneous mastocytosis (disorder)
Congenital Disease	SNOMED	1148933002	Congenital hydronephrosis due to ureteral orifice obstruction (disorder)
Congenital Disease	SNOMED	1148937001	Congenital hydronephrosis due to bladder obstruction (disorder)
Congenital Disease	SNOMED	1148939003	Congenital hydronephrosis due to ureteral obstruction (disorder)
Congenital Disease	SNOMED	1149087003	Congenital microencephaly (disorder)
Congenital Disease	SNOMED	1149232006	Infection of urachal sinus (disorder)
Congenital Disease	SNOMED	1149233001	Infection of urachal remnant (disorder)
Congenital Disease	SNOMED	1150009	Congenital microcheilia (disorder)
Congenital Disease	SNOMED	1153392009	Short stature disorder due to osteosclerosis (disorder)
Congenital Disease	SNOMED	1153430004	Partial deletion of long arm of chromosome 14 (disorder)
Congenital Disease	SNOMED	11535009	Leonine facies (finding)
Congenital Disease	SNOMED	1153583005	Deletion of part of short arm of chromosome 5 (disorder)
Congenital Disease	SNOMED	11552008	Complete congenital duodenal obstruction (disorder)
Congenital Disease	SNOMED	1155640009	Supernumerary bone of foot (disorder)
Congenital Disease	SNOMED	1155723008	Congenital anomaly of blood vessel of spine (disorder)
Congenital Disease	SNOMED	1155727009	Congenital dilatation of common bile duct (disorder)
Congenital Disease	SNOMED	1155732005	Cystic dysplasia of kidney (disorder)
Congenital Disease	SNOMED	1156184000	Metatarsus primus varus of left foot (disorder)
Congenital Disease	SNOMED	1156185004	Metatarsus primus varus of right foot (disorder)
Congenital Disease	SNOMED	1156186003	Metatarsus primus varus of bilateral feet (disorder)
Congenital Disease	SNOMED	1156217009	Congenital obstruction of ureter (disorder)
Congenital Disease	SNOMED	1156306006	Congenital lumbosacral spondylolisthesis (disorder)
Congenital Disease	SNOMED	1156475005	Congenital talipes equinovarus (disorder)
Congenital Disease	SNOMED	1156584007	X-linked intellectual disability hypotonic face syndrome (disorder)
Congenital Disease	SNOMED	1156827007	Inverse Marcus-Gunn phenomenon (disorder)
Congenital Disease	SNOMED	1156849001	Autosomal recessive epidermolysis bullosa simplex (disorder)
Congenital Disease	SNOMED	1156870007	Absence of liver (finding)
Congenital Disease	SNOMED	1156992005	Congenital interstitial cell of Cajal hyperplasia with neuronal intestinal dysplasia (disorder)
Congenital Disease	SNOMED	11614003	Congenital stenosis of pulmonary veins (disorder)
Congenital Disease	SNOMED	1162440009	Deletion of part of short arm of chromosome 12 (disorder)
Congenital Disease	SNOMED	1162460001	Partial deletion of long arm of chromosome 15 (disorder)
Congenital Disease	SNOMED	1162462009	Angelman syndrome due to maternal monosomy 15q11q13 (disorder)
Congenital Disease	SNOMED	1162463004	Partial duplication of long arm of chromosome 15 (disorder)
Congenital Disease	SNOMED	1162478006	Deletion of part of long arm of chromosome 16 (disorder)
Congenital Disease	SNOMED	1162487002	Duplication of part of short arm of chromosome 16 (disorder)
Congenital Disease	SNOMED	1162488007	Duplication of part of long arm of chromosome 16 (disorder)
Congenital Disease	SNOMED	1162587004	Fetal intrauterine perforation of intestine due to stenosis of intestine (disorder)
Congenital Disease	SNOMED	1162589001	Neonatal perforation of intestine due to congenital stenosis of intestine (disorder)
Congenital Disease	SNOMED	1162716000	Camptodactyly of finger (disorder)
Congenital Disease	SNOMED	1162826002	Split spinal cord malformation type I (disorder)
Congenital Disease	SNOMED	1162832007	Mayer Rokitansky Küster Hauser syndrome type 1 (disorder)
Congenital Disease	SNOMED	1162837001	Proximal interphalangeal joint symphalangism Cushing type (disorder)
Congenital Disease	SNOMED	1162839003	XK aprosencephaly syndrome (disorder)
Congenital Disease	SNOMED	1162864000	Familial porencephaly (disorder)
Congenital Disease	SNOMED	1162883007	Congenital instability of bilateral hip joints (disorder)
Congenital Disease	SNOMED	1162915007	Congenital accessory tissue of tricuspid valve (disorder)
Congenital Disease	SNOMED	1163259003	Non syndromic dextrocardia (disorder)
Congenital Disease	SNOMED	1163260008	Non syndromic camptodactyly of fingers (disorder)

Congenital Disease	SNOMED	1163456008	Gingival enlargement due to Cowden syndrome (disorder)
Congenital Disease	SNOMED	1163556006	Agenesis of cervical vertebra (disorder)
Congenital Disease	SNOMED	1163579007	Congenital deformity of bone of forearm (disorder)
Congenital Disease	SNOMED	11646003	Congenital inversion of nipple (disorder)
Congenital Disease	SNOMED	1167375003	Microcephaly, corpus callosum and cerebellar vermis hypoplasia, facial dysmorphism, intellectual disability syndrome (disorder)
Congenital Disease	SNOMED	1169355000	Brain malformations, musculoskeletal abnormalities, facial dysmorphism, intellectual disability syndrome (disorder)
Congenital Disease	SNOMED	1169356004	Early-onset progressive encephalopathy, hearing loss, pons hypoplasia, brain atrophy syndrome (disorder)
Congenital Disease	SNOMED	1169358003	Multinucleated neurons, anhydramnios, renal dysplasia, cerebellar hypoplasia, hydranencephaly syndrome (disorder)
Congenital Disease	SNOMED	1169359006	Tall stature, intellectual disability, renal anomalies syndrome (disorder)
Congenital Disease	SNOMED	1169362009	Overgrowth syndrome with 2q37 translocation (disorder)
Congenital Disease	SNOMED	1169363004	Overgrowth, metaphyseal undermodeling, spondylar dysplasia syndrome (disorder)
Congenital Disease	SNOMED	1169365006	Aquagenic palmoplantar keratoderma (disorder)
Congenital Disease	SNOMED	11701009	Hemicephaly (disorder)
Congenital Disease	SNOMED	1172358003	Gingival enlargement due to Sturge-Weber syndrome (disorder)
Congenital Disease	SNOMED	1172589000	Congenital omphalocele, diaphragmatic hernia, cardiovascular anomalies, radial ray defect syndrome (disorder)
Congenital Disease	SNOMED	1172594000	Congenital labioscrotal agenesis, cerebellar malformation, corneal dystrophy, facial dysmorphism syndrome (disorder)
Congenital Disease	SNOMED	1172605003	Retinitis pigmentosa, hearing loss, premature aging, short stature, facial dysmorphism syndrome (disorder)
Congenital Disease	SNOMED	1172624000	Arginine-glutamic acid dipeptide repeats-related neurodevelopmental syndrome (disorder)
Congenital Disease	SNOMED	1172626003	Telomere maintenance 2-related intellectual disability, neurodevelopmental disorder (disorder)
Congenital Disease	SNOMED	1172628002	TBC1 domain containing kinase-related intellectual disability syndrome (disorder)
Congenital Disease	SNOMED	1172629005	Severe growth deficiency, strabismus, extensive dermal melanocytosis, intellectual disability syndrome (disorder)
Congenital Disease	SNOMED	1172632008	SIX homeobox 2-related frontonasal dysplasia (disorder)
Congenital Disease	SNOMED	1172633003	Camptodactyly syndrome Guadalajara type 3 (disorder)
Congenital Disease	SNOMED	1172635005	Split-foot malformation, mesoaxial polydactyly syndrome (disorder)
Congenital Disease	SNOMED	1172636006	Familial progressive retinal dystrophy, iris coloboma, congenital cataract syndrome (disorder)
Congenital Disease	SNOMED	1172685001	Macrothrombocytopenia, lymphedema, developmental delay, facial dysmorphism, camptodactyly syndrome (disorder)
Congenital Disease	SNOMED	1172692006	X-linked keloid scarring, reduced joint mobility, increased optic cup-to-disc ratio syndrome (disorder)
Congenital Disease	SNOMED	1172697000	X-linked female restricted facial dysmorphism, short stature, choanal atresia, intellectual disability (disorder)
Congenital Disease	SNOMED	1172705006	Lethal hydranencephaly, diaphragmatic hernia syndrome (disorder)
Congenital Disease	SNOMED	1172889005	Palatal anomalies, widely spaced teeth, facial dysmorphism, developmental delay syndrome (disorder)
Congenital Disease	SNOMED	1172898008	Kosaki overgrowth syndrome (disorder)
Congenital Disease	SNOMED	1172899000	Peripheral myelin protein 22-retinoic acid induced 1 contiguous gene duplication syndrome (disorder)
Congenital Disease	SNOMED	1172900005	Progressive microcephaly, seizures, cortical blindness, developmental delay syndrome (disorder)
Congenital Disease	SNOMED	11731003	Neck webbing (disorder)
Congenital Disease	SNOMED	1177166006	Frontonasal dysplasia, bifid nose, upper limb anomalies syndrome (disorder)
Congenital Disease	SNOMED	1177167002	Intellectual disability, seizures, abnormal gait, facial dysmorphism syndrome (disorder)
Congenital Disease	SNOMED	1177175008	Skeletal dysplasia, T-cell immunodeficiency, developmental delay syndrome (disorder)
Congenital Disease	SNOMED	1177176009	Intermediate epidermolysis bullosa simplex with cardiomyopathy (disorder)
Congenital Disease	SNOMED	1177178005	Intrauterine growth restriction, congenital multiple café au lait macules, increased sister chromatid exchange syndrome (disorder)
Congenital Disease	SNOMED	1177179002	Oral-facial-digital syndrome with short stature and brachymesophalangia (disorder)
Congenital Disease	SNOMED	1179283004	Metopic ridging, ptosis, facial dysmorphism syndrome (disorder)
Congenital Disease	SNOMED	1179293006	Erythrokeratodermia cardiomyopathy syndrome (disorder)
Congenital Disease	SNOMED	1179296003	Colobomatous macrophthalmia with microcornea syndrome (disorder)
Congenital Disease	SNOMED	1179298002	Familial patent arterial duct (disorder)
Congenital Disease	SNOMED	1179299005	NIMA related kinase 9 lethal skeletal dysplasia (disorder)
Congenital Disease	SNOMED	1179301003	Dual specificity tyrosine phosphorylation regulated kinase 1A-related intellectual disability syndrome (disorder)
Congenital Disease	SNOMED	1179404005	Congenital hypothyroidism due to congenital anomaly of thyroid gland (disorder)
Congenital Disease	SNOMED	1179408008	Chromodomain helicase dna-binding protein 3- related developmental delay, speech delay, intellectual disability, abnormalities of vision, facial dysmorphism syndrome (disorder)

Congenital Disease	SNOMED	1179719005	Diffuse pulmonary lymphangiomatosis (disorder)
Congenital Disease	SNOMED	118642009	Congenital anomaly of the urinary tract proper (disorder)
Congenital Disease	SNOMED	1186709006	Filamin A-related X-linked myxomatous valvular dysplasia (disorder)
Congenital Disease	SNOMED	1186725001	Warts, immunodeficiency, lymphedema, anogenital dysplasia syndrome (disorder)
Congenital Disease	SNOMED	1186729007	Intellectual disability, cardiac anomalies, short stature, joint laxity syndrome (disorder)
Congenital Disease	SNOMED	1186730002	Gabriele-de Vries syndrome (disorder)
Congenital Disease	SNOMED	1186846000	Male infertility due to undescended testicle (disorder)
Congenital Disease	SNOMED	1186970002	Periodontitis due to Ehlers-Danlos syndrome type 4 (disorder)
Congenital Disease	SNOMED	1187039001	Cleft lip and palate, craniofacial dysmorphism, congenital heart defect, hearing loss syndrome (disorder)
Congenital Disease	SNOMED	1187041000	Stromal antigen 1-related intellectual disability, facial dysmorphism, gastroesophageal reflux syndrome (disorder)
Congenital Disease	SNOMED	1187042007	Early-onset seizures, distal limb anomalies, facial dysmorphism, global developmental delay syndrome (disorder)
Congenital Disease	SNOMED	1187114007	Micrognathia, recurrent infections, behavioral abnormalities, mild intellectual disability syndrome (disorder)
Congenital Disease	SNOMED	1187115008	Autosomal dominant preaxial polydactyly, upper back hypertrichosis syndrome (disorder)
Congenital Disease	SNOMED	1187120008	Stromme syndrome (disorder)
Congenital Disease	SNOMED	1187122000	Witteveen Kolk syndrome (disorder)
Congenital Disease	SNOMED	1187130004	Agenesis of scrotum (disorder)
Congenital Disease	SNOMED	1187132007	Sugarman brachydactyly (disorder)
Congenital Disease	SNOMED	1187195007	Microcephalic cortical malformations, short stature due to rotatin deficiency (disorder)
Congenital Disease	SNOMED	1187215002	Tubulinopathy-associated dysgyria (disorder)
Congenital Disease	SNOMED	1187247007	WW domain containing adaptor with coiled-coil-related facial dysmorphism, developmental delay, behavioral abnormalities syndrome (disorder)
Congenital Disease	SNOMED	1187277001	Short stature, brachydactyly, obesity, global developmental delay syndrome (disorder)
Congenital Disease	SNOMED	1187292000	Congenital lordosis deformity of spine due to congenital malformation of skeletal bone (disorder)
Congenital Disease	SNOMED	1187303004	Progressive spondyloepimetaphyseal dysplasia, short stature, short fourth metatarsals, intellectual disability syndrome (disorder)
Congenital Disease	SNOMED	1187304005	Macrocephaly, intellectual disability, neurodevelopmental disorder, small thorax syndrome (disorder)
Congenital Disease	SNOMED	1187358001	Congenital lordosis deformity of spine (disorder)
Congenital Disease	SNOMED	1187359009	Congenital lordosis and scoliosis deformity of spine (disorder)
Congenital Disease	SNOMED	1187362007	Lordosis deformity of spine due to congenital skeletal dysplasia (disorder)
Congenital Disease	SNOMED	11873751000119106	Congenital pes cavus of bilateral feet (disorder)
Congenital Disease	SNOMED	1187460003	Unilateral multicystic renal dysplasia (disorder)
Congenital Disease	SNOMED	1187522009	Peroxisome biogenesis disorder due to PEX26 mutation (disorder)
Congenital Disease	SNOMED	1187523004	Peroxisome biogenesis disorder due to PEX3 mutation (disorder)
Congenital Disease	SNOMED	1187524005	Peroxisome biogenesis disorder due to PEX19 mutation (disorder)
Congenital Disease	SNOMED	1187525006	Peroxisome biogenesis disorder due to PEX2 mutation (disorder)
Congenital Disease	SNOMED	1187526007	Peroxisome biogenesis disorder due to PEX16 mutation (disorder)
Congenital Disease	SNOMED	1187527003	Peroxisome biogenesis disorder due to PEX13 mutation (disorder)
Congenital Disease	SNOMED	1187528008	Peroxisome biogenesis disorder due to PEX14 mutation (disorder)
Congenital Disease	SNOMED	1187529000	Peroxisome biogenesis disorder due to PEX10 mutation (disorder)
Congenital Disease	SNOMED	1187530005	Peroxisome biogenesis disorder due to PEX12 mutation (disorder)
Congenital Disease	SNOMED	1187532002	Peroxisome biogenesis disorder due to PEX1 mutation (disorder)
Congenital Disease	SNOMED	1187548001	Peroxisome biogenesis disorder due to PEX5 mutation (disorder)
Congenital Disease	SNOMED	1187550009	Peroxisome biogenesis disorder due to PEX6 mutation (disorder)
Congenital Disease	SNOMED	1187642008	Macrocephaly, intellectual disability, left ventricular non compaction syndrome (disorder)
Congenital Disease	SNOMED	1187644009	Basel Vanagaite Smirin Yosef syndrome (disorder)
Congenital Disease	SNOMED	1197018005	Osteogenesis imperfecta type IIC (disorder)
Congenital Disease	SNOMED	1197057002	Hallermann Streiff like syndrome (disorder)
Congenital Disease	SNOMED	1197059004	Congenital ichthyosis, microcephalus, tetraplegia syndrome (disorder)
Congenital Disease	SNOMED	1197148005	Sanjad Sakati syndrome (disorder)
Congenital Disease	SNOMED	1197212001	Posterior meningocele (disorder)
Congenital Disease	SNOMED	1197215004	Microform holoprosencephaly (disorder)
Congenital Disease	SNOMED	1197357008	Colobomatous optic disc, macular atrophy, chorioretinopathy syndrome (disorder)
Congenital Disease	SNOMED	1197358003	Autosomal recessive dysgenesis of anterior segment of eye (disorder)
Congenital Disease	SNOMED	1197365006	Familial cavitary optic disc anomaly (disorder)
Congenital Disease	SNOMED	1197428008	Combined immunodeficiency, enteropathy spectrum (disorder)
Congenital Disease	SNOMED	1197588008	X-linked microcephaly, growth retardation, prognathism, cryptorchidism syndrome (disorder)
Congenital Disease	SNOMED	1197589000	Bilateral hip and radial head dislocations, short stature, scoliosis, carpal coalition, pes cavus, facial dysmorphism syndrome (disorder)
Congenital Disease	SNOMED	1197591008	Severe intellectual disability, hypotonia, strabismus, coarse face, planovalgus syndrome (disorder)

Congenital Disease	SNOMED	1197592001	Intrauterine growth restriction, short stature, early adult-onset diabetes syndrome (disorder)
Congenital Disease	SNOMED	1197593006	Intellectual disability, expressive aphasia, facial dysmorphism syndrome (disorder)
Congenital Disease	SNOMED	1197754004	Congenital brachyesophagus, intrathoracic stomach, vertebral anomalies syndrome (disorder)
Congenital Disease	SNOMED	11991000119105	Urinary tract obstruction due to duplicated collecting system (disorder)
Congenital Disease	SNOMED	12010005	Osteodystrophy (disorder)
Congenital Disease	SNOMED	12011000119105	Congenital pes valgo planus (disorder)
Congenital Disease	SNOMED	12017008	Congenital absence of ovary (disorder)
Congenital Disease	SNOMED	1201776003	Congenital complete absence of right lower limb (disorder)
Congenital Disease	SNOMED	1201777007	Congenital complete absence of left lower limb (disorder)
Congenital Disease	SNOMED	1204130006	Central basal perimembranous ventricular septal defect (disorder)
Congenital Disease	SNOMED	1204193005	Aneurysm of aorta due to congenital heart disease (disorder)
Congenital Disease	SNOMED	1204346009	Congenital cystic dilatation of common bile duct (disorder)
Congenital Disease	SNOMED	1204421005	Lymphedema, posterior choanal atresia syndrome (disorder)
Congenital Disease	SNOMED	12070002	Congenital stenosis of larynx (disorder)
Congenital Disease	SNOMED	12075007	Congenital hypoplasia of ascending aorta (disorder)
Congenital Disease	SNOMED	1208338004	Dysraphism, cleft lip and palate, limb reduction defect syndrome (disorder)
Congenital Disease	SNOMED	1208341008	Severe oculo-renal-cerebellar syndrome (disorder)
Congenital Disease	SNOMED	1208342001	Eye defects, arachnodactyly, cardiopathy syndrome (disorder)
Congenital Disease	SNOMED	1208344000	Fryns Smeets Thiry syndrome (disorder)
Congenital Disease	SNOMED	1208346003	Congenital hydrocephalus, low insertion of umbilicus syndrome (disorder)
Congenital Disease	SNOMED	1208348002	Microcephalic osteodysplastic primordial dwarfism type II (disorder)
Congenital Disease	SNOMED	1208480004	Epibulbar lipodermoid, preauricular appendage, polythelia syndrome (disorder)
Congenital Disease	SNOMED	1208482007	Distal arthrogryposis type 10 (disorder)
Congenital Disease	SNOMED	1208488006	Special AT-rich sequence-binding protein 2-associated syndrome (disorder)
Congenital Disease	SNOMED	1208614008	Autosomal dominant deafness with onychodystrophy syndrome (disorder)
Congenital Disease	SNOMED	1208720000	Agenesis of corpus callosum, macrocephaly, hypertelorism syndrome (disorder)
Congenital Disease	SNOMED	1208727002	Severe intellectual disability, agenesis of corpus callosum, facial dysmorphism, cerebellar ataxia syndrome (disorder)
Congenital Disease	SNOMED	1208746001	Intellectual disability, muscle weakness, short stature, facial dysmorphism syndrome (disorder)
Congenital Disease	SNOMED	1208845005	Secondary hypertension due to congenital heart disorder (disorder)
Congenital Disease	SNOMED	1208935007	Polymicrogyria due to tubulin beta 2B class IIb mutation (disorder)
Congenital Disease	SNOMED	1208936008	Congenital ichthyosis, intellectual disability, spastic quadriplegia syndrome (disorder)
Congenital Disease	SNOMED	1208985003	Linear hypopigmentation and craniofacial asymmetry with acral, ocular and brain anomalies (disorder)
Congenital Disease	SNOMED	1208987006	Pleckstrin homology domain interacting protein-related behavioral problems, intellectual disability, obesity, dysmorphic features syndrome (disorder)
Congenital Disease	SNOMED	1208998007	Tumor necrosis factor receptor associated factor 7-associated heart defect, digital anomalies, facial dysmorphism, motor and speech delay syndrome (disorder)
Congenital Disease	SNOMED	12104008	Congenital rectocloacal fistula (disorder)
Congenital Disease	SNOMED	12121000119102	Congenital trigger finger and trigger thumb (disorder)
Congenital Disease	SNOMED	1216940001	Joint contractures, developmental delay, Pierre Robin syndrome (disorder)
Congenital Disease	SNOMED	1216942009	Cerebral ventriculomegaly, cystic kidney disease (disorder)
Congenital Disease	SNOMED	1216943004	Mandibulofacial dysostosis with alopecia (disorder)
Congenital Disease	SNOMED	1217207008	Congenital oculomotor nerve palsy (disorder)
Congenital Disease	SNOMED	1217225001	Klippel-Feil anomaly, myopathy, facial dysmorphism syndrome (disorder)
Congenital Disease	SNOMED	1217228004	X-linked intellectual disability, cerebellar hypoplasia, spondyloepiphyseal dysplasia syndrome (disorder)
Congenital Disease	SNOMED	1217229007	Craniofacial dysplasia, short stature, ectodermal anomalies, intellectual disability syndrome (disorder)
Congenital Disease	SNOMED	1217372003	Severe myopia, generalized joint laxity, short stature syndrome (disorder)
Congenital Disease	SNOMED	1217373008	Diaphragmatic hernia, short bowel, asplenia syndrome (disorder)
Congenital Disease	SNOMED	1217382002	Intellectual disability, autism, speech apraxia, craniofacial dysmorphism syndrome (disorder)
Congenital Disease	SNOMED	1217623004	Congenital membrane of lacrimal punctum (disorder)
Congenital Disease	SNOMED	1217624005	Congenital distortion of orbit (disorder)
Congenital Disease	SNOMED	1217625006	Congenital expansion of orbit (disorder)
Congenital Disease	SNOMED	1217626007	Congenital complete absence of nasolacrimal drainage system (disorder)
Congenital Disease	SNOMED	1217627003	Congenital contraction of orbit (disorder)
Congenital Disease	SNOMED	1217628008	Congenital absence of eye with orbital implant (disorder)
Congenital Disease	SNOMED	1217629000	Congenital combined bony and soft tissue deformity of orbit (disorder)
Congenital Disease	SNOMED	1217664009	Congenital colobomatous cyst of orbit (disorder)
Congenital Disease	SNOMED	1217688006	Congenital corneal leukoma (disorder)
Congenital Disease	SNOMED	121801000119105	Fetal gastrointestinal abnormality (disorder)
Congenital Disease	SNOMED	12195004	Coronary cataract (disorder)
Congenital Disease	SNOMED	1220568003	Glutamine rich 1-related intellectual disability, chondrodysplasia syndrome (disorder)

Congenital Disease	SNOMED	1220575002	Fetal encasement syndrome (disorder)
Congenital Disease	SNOMED	1220594007	Pierpont syndrome (disorder)
Congenital Disease	SNOMED	1220596009	Microcephalic primordial dwarfism, insulin resistance syndrome (disorder)
Congenital Disease	SNOMED	1220597000	Retinitis pigmentosa, juvenile cataract, short stature, intellectual disability syndrome (disorder)
Congenital Disease	SNOMED	1220599002	Pseudoxanthoma elasticum-like skin manifestations with retinitis pigmentosa (disorder)
Congenital Disease	SNOMED	1222625002	Hypercyanotic spell due to congenital heart disease (finding)
Congenital Disease	SNOMED	1222645005	Keratin 1-related diffuse nonepidermolytic palmoplantar keratoderma (disorder)
Congenital Disease	SNOMED	1222657001	Prune exopolyphosphatase 1-related neurological syndrome (disorder)
Congenital Disease	SNOMED	1222660008	Pancreatic agenesis, holoprosencephaly syndrome (disorder)
Congenital Disease	SNOMED	1222708006	Transmembrane protein 94-associated congenital heart defect, facial dysmorphism, developmental delay syndrome (disorder)
Congenital Disease	SNOMED	1222710008	Neurodevelopmental disorder, craniofacial dysmorphism, cardiac defect, skeletal anomalies syndrome (disorder)
Congenital Disease	SNOMED	12235641000119107	Congenital ptosis of bilateral upper eyelids (disorder)
Congenital Disease	SNOMED	12235681000119102	Congenital ptosis of left upper eyelid (disorder)
Congenital Disease	SNOMED	12235721000119108	Congenital ptosis of right upper eyelid (disorder)
Congenital Disease	SNOMED	12235861000119107	Congenital pes cavus of left foot (disorder)
Congenital Disease	SNOMED	12235901000119101	Congenital pes cavus of right foot (disorder)
Congenital Disease	SNOMED	12235941000119104	Congenital deformity of left foot (disorder)
Congenital Disease	SNOMED	12235981000119109	Congenital deformity of right foot (disorder)
Congenital Disease	SNOMED	12236021000119104	Congenital deformity of left upper limb (disorder)
Congenital Disease	SNOMED	12236061000119109	Congenital deformity of right upper limb (disorder)
Congenital Disease	SNOMED	12252009	Congenital absence of sternum (disorder)
Congenital Disease	SNOMED	12275031000119106	Congenital cerebral ventriculomegaly (disorder)
Congenital Disease	SNOMED	1228844002	1p35.2 microdeletion syndrome (disorder)
Congenital Disease	SNOMED	1228858000	Complex lethal osteochondrodysplasia (disorder)
Congenital Disease	SNOMED	1228860003	Spondyloepiphyseal dysplasia Stanescu type (disorder)
Congenital Disease	SNOMED	1228886008	9q33.3q34.11 microdeletion syndrome (disorder)
Congenital Disease	SNOMED	1228890005	16p13.2 microdeletion syndrome (disorder)
Congenital Disease	SNOMED	1229872004	Xq25 microduplication syndrome (disorder)
Congenital Disease	SNOMED	1229873009	17q24.2 microdeletion syndrome (disorder)
Congenital Disease	SNOMED	1229875002	9q21.13 microdeletion syndrome (disorder)
Congenital Disease	SNOMED	1229876001	Lethal brain and heart developmental defects syndrome (disorder)
Congenital Disease	SNOMED	1229882003	11q22.2q22.3 microdeletion syndrome (disorder)
Congenital Disease	SNOMED	1229883008	19p13.3 microduplication syndrome (disorder)
Congenital Disease	SNOMED	1229891004	20q11.2 microdeletion syndrome (disorder)
Congenital Disease	SNOMED	1229895008	8q24.3 microdeletion syndrome (disorder)
Congenital Disease	SNOMED	1229943004	SIM bHLH transcription factor 1-related Prader-Willi-like syndrome (disorder)
Congenital Disease	SNOMED	1229946007	MAGE family member L2-related Prader-Willi-like syndrome (disorder)
Congenital Disease	SNOMED	1229998009	Combined hamartoma of retina and retinal pigment epithelium (disorder)
Congenital Disease	SNOMED	1229999001	Autosomal dominant myopia, midfacial retrusion, sensorineural hearing loss, rhizomelic dysplasia syndrome (disorder)
Congenital Disease	SNOMED	1230005002	Pigmentation defects, palmoplantar keratoderma, skin carcinoma syndrome (disorder)
Congenital Disease	SNOMED	1230016009	Familial congenital nasolacrimal duct obstruction (disorder)
Congenital Disease	SNOMED	1230020008	Congenital peripapillary staphyloma (disorder)
Congenital Disease	SNOMED	1230021007	Frontorhiny (disorder)
Congenital Disease	SNOMED	1230024004	Congenital deformity of lower limb (disorder)
Congenital Disease	SNOMED	1230025003	Complete septate uterus (disorder)
Congenital Disease	SNOMED	1230026002	Lethal acantholytic erosive disorder (disorder)
Congenital Disease	SNOMED	1230062001	Congenital atresia of inferior vena cava without azygos continuation (disorder)
Congenital Disease	SNOMED	1230066003	Isolated microspherophakia (disorder)
Congenital Disease	SNOMED	1230098009	Femur fibula ulna complex (disorder)
Congenital Disease	SNOMED	1230239009	Congenital laryngotracheoesophageal cleft (disorder)
Congenital Disease	SNOMED	1230270001	Exstrophy epispadias complex (disorder)
Congenital Disease	SNOMED	1230271002	Congenital deformity of upper limb (disorder)
Congenital Disease	SNOMED	1230295000	B-cell immunodeficiency, limb anomaly, urogenital malformation syndrome (disorder)
Congenital Disease	SNOMED	1230344000	Microphthalmia, microtia, fetal akinesia syndrome (disorder)
Congenital Disease	SNOMED	1230354001	Congenital deformity of hand (disorder)
Congenital Disease	SNOMED	1230355000	Congenital anomaly of bilateral upper limbs (disorder)
Congenital Disease	SNOMED	1230356004	Congenital anomaly of right upper limb (disorder)
Congenital Disease	SNOMED	1230357008	Congenital anomaly of left upper limb (disorder)
Congenital Disease	SNOMED	1230376005	Contactin associated protein 2-related developmental and epileptic encephalopathy (disorder)
Congenital Disease	SNOMED	1230384009	Congenital deformity of shoulder (disorder)
Congenital Disease	SNOMED	1230385005	Congenital deformity of upper arm (disorder)
Congenital Disease	SNOMED	1231148002	Linear verrucous nevus syndrome (disorder)
Congenital Disease	SNOMED	1231169000	Congenital anomaly of second branchial cleft (disorder)
Congenital Disease	SNOMED	1231170004	Congenital anomaly of third branchial cleft (disorder)
Congenital Disease	SNOMED	1231175009	Congenital anomaly of fourth branchial cleft (disorder)

Congenital Disease	SNOMED	1231176005	Congenital fistula of commissure of lips (disorder)
Congenital Disease	SNOMED	1231181001	Non-syndromic metopic craniosynostosis (disorder)
Congenital Disease	SNOMED	1231182008	Isolated osteopoikilosis (disorder)
Congenital Disease	SNOMED	1231206001	Meningoencephalocele of orbit (disorder)
Congenital Disease	SNOMED	1231209008	Congenital meningocele of orbit (disorder)
Congenital Disease	SNOMED	1231284001	Autosomal dominant generalized dystrophic epidermolysis bullosa (disorder)
Congenital Disease	SNOMED	1231288003	Congenital anomaly of left lower limb (disorder)
Congenital Disease	SNOMED	1231289006	Congenital anomaly of right lower limb (disorder)
Congenital Disease	SNOMED	1231455008	Incomplete cleft lip (disorder)
Congenital Disease	SNOMED	1231525004	Complete cleft lip (disorder)
Congenital Disease	SNOMED	12316007	Persistent fetal uterus (disorder)
Congenital Disease	SNOMED	1231626009	Syndromic nanophthalmos due to Kenny-Caffey syndrome (disorder)
Congenital Disease	SNOMED	1231686007	Congenital pseudopapilledema (disorder)
Congenital Disease	SNOMED	1231746006	Isolated agenesis of cerebellar vermis (disorder)
Congenital Disease	SNOMED	1232006	Congenital articular rigidity with myopathy (disorder)
Congenital Disease	SNOMED	12322003	Congenital eventration of left crus of diaphragm (disorder)
Congenital Disease	SNOMED	123276005	Congenital anomaly of umbilical artery (disorder)
Congenital Disease	SNOMED	1234830005	14q32 duplication syndrome (disorder)
Congenital Disease	SNOMED	1234906009	46,XX ovotesticular disorder of sex development (disorder)
Congenital Disease	SNOMED	1234907000	Ovotesticular disorder of sex development (disorder)
Congenital Disease	SNOMED	1234908005	Congenital azygos continuation of inferior vena cava (disorder)
Congenital Disease	SNOMED	1234910007	Congenital straddling and overriding tricuspid valve (disorder)
Congenital Disease	SNOMED	1234911006	Congenital cochleovestibular malformation (disorder)
Congenital Disease	SNOMED	1235003006	Congenital vascular malformation of orbital region (disorder)
Congenital Disease	SNOMED	123557007	Congenital anomaly of scapula (disorder)
Congenital Disease	SNOMED	123558002	Congenital anomaly of humerus (disorder)
Congenital Disease	SNOMED	123559005	Congenital anomaly of radius (disorder)
Congenital Disease	SNOMED	123560000	Congenital anomaly of ulna (disorder)
Congenital Disease	SNOMED	123561001	Congenital anomaly of femur (disorder)
Congenital Disease	SNOMED	123562008	Congenital anomaly of fibula (disorder)
Congenital Disease	SNOMED	123563003	Congenital anomaly of tibia (disorder)
Congenital Disease	SNOMED	123566006	Congenital anomaly of calcaneus (disorder)
Congenital Disease	SNOMED	123568007	Congenital anomaly of tarsal bone (disorder)
Congenital Disease	SNOMED	123569004	Congenital anomaly of metatarsal bone (disorder)
Congenital Disease	SNOMED	123570003	Congenital anomaly of carpal bone (disorder)
Congenital Disease	SNOMED	123571004	Congenital anomaly of metacarpal bone (disorder)
Congenital Disease	SNOMED	123572006	Congenital anomaly of rib (disorder)
Congenital Disease	SNOMED	123644009	Gynandromorphism syndrome (disorder)
Congenital Disease	SNOMED	123645005	Group chromosomal alteration (disorder)
Congenital Disease	SNOMED	123646006	Chromosomal alterations of group A (disorder)
Congenital Disease	SNOMED	123647002	Chromosomal alterations of group B (disorder)
Congenital Disease	SNOMED	123648007	Chromosomal alterations of group C and X (disorder)
Congenital Disease	SNOMED	123649004	Chromosomal alterations of group D (disorder)
Congenital Disease	SNOMED	123650004	Chromosomal alterations of group E (disorder)
Congenital Disease	SNOMED	123651000	Chromosomal alterations of group F (disorder)
Congenital Disease	SNOMED	123652007	Chromosomal alterations of group G and Y (disorder)
Congenital Disease	SNOMED	123654008	Congenital anomaly of lower respiratory system (disorder)
Congenital Disease	SNOMED	123655009	Accessory structure of lower respiratory tract (disorder)
Congenital Disease	SNOMED	123656005	Congenital atresia of cardiac valve (disorder)
Congenital Disease	SNOMED	123657001	Congenital stenosis of cardiac valve (disorder)
Congenital Disease	SNOMED	123658006	Congenital cleft of cardiac valve (disorder)
Congenital Disease	SNOMED	123659003	Congenital malrotation of heart (disorder)
Congenital Disease	SNOMED	123660008	Dextrorotation of heart (disorder)
Congenital Disease	SNOMED	123661007	Levoatrial cardinal vein (disorder)
Congenital Disease	SNOMED	123662000	Persistent dorsal mesentery (disorder)
Congenital Disease	SNOMED	123665003	Uterus bicornis unicollis with septate vagina (disorder)
Congenital Disease	SNOMED	123666002	Rudimentary uterus in male (disorder)
Congenital Disease	SNOMED	123669009	Pseudolipomatosis hypertrophy of pancreas (disorder)
Congenital Disease	SNOMED	1236806006	Persistent congenital anteversion of femur (disorder)
Congenital Disease	SNOMED	1236843008	Phosphodiesterase 4D haploinsufficiency syndrome (disorder)
Congenital Disease	SNOMED	1236845001	DNA replication fork stabilization factor DONSON-related microcephaly, short stature, limb abnormalities spectrum (disorder)
Congenital Disease	SNOMED	1237074000	Congenital atrioventricular septal defect (disorder)
Congenital Disease	SNOMED	123714004	Spontaneous closure of ventricular septal defect (finding)
Congenital Disease	SNOMED	1237179007	FG syndrome type 1 (disorder)
Congenital Disease	SNOMED	1237225007	Dermatosparaxis Ehlers-Danlos syndrome (disorder)
Congenital Disease	SNOMED	1237226008	Embryopathy caused by isotretinoin (disorder)
Congenital Disease	SNOMED	1237228009	Night blindness, skeletal anomalies, dysmorphism syndrome (disorder)
Congenital Disease	SNOMED	1237303004	Congenital abnormal retraction of nipple (disorder)
Congenital Disease	SNOMED	1237337007	Extensive peripapillary myelinated nerve fibers of retina (disorder)
Congenital Disease	SNOMED	1237342004	Lethal fetal cerebrorenogenitourinary agenesis or hypoplasia syndrome (disorder)
Congenital Disease	SNOMED	1237344003	Symptomatic form of fragile X syndrome in female carrier (disorder)
Congenital Disease	SNOMED	1237345002	46,XX ovarian dysgenesis, short stature syndrome (disorder)
Congenital Disease	SNOMED	1237346001	Caroli syndrome (disorder)

Congenital Disease	SNOMED	1237365009	Aprosencephaly/atelencephaly spectrum (disorder)
Congenital Disease	SNOMED	1237366005	Aprosencephaly cerebellar dysgenesis (disorder)
Congenital Disease	SNOMED	1237370002	Myelinated nerve fiber layer of retina (disorder)
Congenital Disease	SNOMED	1237412001	Regressive spondylometaphyseal dysplasia (disorder)
Congenital Disease	SNOMED	1237418002	Spastic tetraplegia, thin corpus callosum, progressive postnatal microcephaly syndrome (disorder)
Congenital Disease	SNOMED	1237420004	X-linked intellectual disability, global development delay, facial dysmorphism, sacral caudal remnant syndrome (disorder)
Congenital Disease	SNOMED	1237421000	Pyrroline-5-carboxylate reductase 2 related microcephaly, progressive leukoencephalopathy (disorder)
Congenital Disease	SNOMED	1237462006	nudE neurodevelopment protein 1-related microhydranencephaly (disorder)
Congenital Disease	SNOMED	1237470001	Lethal fetal brain malformation, duodenal atresia, bilateral renal hypoplasia syndrome (disorder)
Congenital Disease	SNOMED	1237475006	Cerebellar-facial-dental syndrome (disorder)
Congenital Disease	SNOMED	1237509001	Peeling skin, leukonychia, acral punctate keratoses, cheilitis, knuckle pads syndrome (disorder)
Congenital Disease	SNOMED	1237513008	Osteosclerotic metaphyseal dysplasia (disorder)
Congenital Disease	SNOMED	1237577000	Symptomatic form of Coffin-Lowry syndrome in female carrier (disorder)
Congenital Disease	SNOMED	1237618009	Short stature, optic nerve atrophy, Pelger-Huët anomaly syndrome (disorder)
Congenital Disease	SNOMED	1239002	Congenital anteversion of femur (finding)
Congenital Disease	SNOMED	1240462005	Generalized inflammatory peeling skin syndrome (disorder)
Congenital Disease	SNOMED	1240463000	Generalized non-inflammatory peeling skin syndrome (disorder)
Congenital Disease	SNOMED	1251449006	Ubiquitin specific peptidase 18 deficiency (disorder)
Congenital Disease	SNOMED	1251450006	16p12.1p12.3 triplication syndrome (disorder)
Congenital Disease	SNOMED	1251451005	Myosin binding protein C1-related autosomal recessive non-lethal arthrogryposis multiplex congenita syndrome (disorder)
Congenital Disease	SNOMED	1251452003	4q25 proximal deletion syndrome (disorder)
Congenital Disease	SNOMED	1251453008	Lamb Shaffer syndrome (disorder)
Congenital Disease	SNOMED	1251488008	Spondylodysplastic Ehlers-Danlos syndrome (disorder)
Congenital Disease	SNOMED	1251499005	Beta-1,3-galactosyltransferase 6-related spondylodysplastic Ehlers-Danlos syndrome (disorder)
Congenital Disease	SNOMED	1254650002	Microcephaly, corpus callosum hypoplasia, intellectual disability, facial dysmorphism syndrome (disorder)
Congenital Disease	SNOMED	1254651003	Microcephaly, intellectual disability, sensorineural hearing loss, epilepsy, abnormal muscle tone syndrome (disorder)
Congenital Disease	SNOMED	1254652005	Intellectual disability, macrocephaly, hypotonia, behavioral abnormalities syndrome (disorder)
Congenital Disease	SNOMED	1254751008	Congenital prepapillary vascular loop (disorder)
Congenital Disease	SNOMED	1254877009	Nail dystrophy due to epidermolysis bullosa (disorder)
Congenital Disease	SNOMED	1254890002	Localized junctional epidermolysis bullosa non-Herlitz type (disorder)
Congenital Disease	SNOMED	1254893000	Congenital isolated onychodysplasia (disorder)
Congenital Disease	SNOMED	125491000119103	Fetus with Turner syndrome (disorder)
Congenital Disease	SNOMED	1254936009	Pseudoxanthoma elasticum due to hemoglobinopathy (disorder)
Congenital Disease	SNOMED	125501000119105	Fetus with complete trisomy 21 syndrome (disorder)
Congenital Disease	SNOMED	125511000119108	Fetus with complete trisomy 18 syndrome (disorder)
Congenital Disease	SNOMED	1255116001	Myopathic Ehlers-Danlos syndrome (disorder)
Congenital Disease	SNOMED	1255121003	Classical-like Ehlers-Danlos syndrome type 2 (disorder)
Congenital Disease	SNOMED	1255143006	Ichthyosis hystrix gravior (disorder)
Congenital Disease	SNOMED	125521000119101	Fetus with complete trisomy 13 syndrome (disorder)
Congenital Disease	SNOMED	1255267007	Mirror-image polydactyly (disorder)
Congenital Disease	SNOMED	1255268002	Oculocerebrodental syndrome (disorder)
Congenital Disease	SNOMED	1255319004	Autosomal dominant intellectual disability, craniofacial anomalies, cardiac defects syndrome (disorder)
Congenital Disease	SNOMED	1255322002	Congenital contracture of limbs and face, hypotonia, developmental delay syndrome (disorder)
Congenital Disease	SNOMED	1255335006	X-linked intellectual disability, short stature, overweight syndrome (disorder)
Congenital Disease	SNOMED	1256046005	Sporadic camptodactyly (disorder)
Congenital Disease	SNOMED	1258935001	Congenital complete absence of left upper limb (disorder)
Congenital Disease	SNOMED	1258936000	Congenital complete absence of right upper limb (disorder)
Congenital Disease	SNOMED	1258972007	Baraitser Winter cerebrofrontofacial syndrome (disorder)
Congenital Disease	SNOMED	1259114008	Cerebrospinal fluid otorrhea due to congenital deformity of labyrinth (disorder)
Congenital Disease	SNOMED	1259117001	Cerebrospinal fluid otorrhea due to encephalocele (disorder)
Congenital Disease	SNOMED	1259119003	Antenatal multi-minicore disease with arthrogryposis multiplex congenita (disorder)
Congenital Disease	SNOMED	1259473000	Dementia due to fragile X syndrome (disorder)
Congenital Disease	SNOMED	125963005	Patent ductus arteriosus with left-to-right shunt (disorder)
Congenital Disease	SNOMED	125964004	Patent ductus arteriosus with right-to-left shunt (disorder)
Congenital Disease	SNOMED	1259870009	Pain due to retraction of testis (disorder)
Congenital Disease	SNOMED	1260095004	Menke Hennekam syndrome (disorder)
Congenital Disease	SNOMED	1260142000	Congenital vertebral, cardiac, renal anomalies syndrome (disorder)
Congenital Disease	SNOMED	1260143005	Megalencephaly, severe kyphoscoliosis, overgrowth syndrome (disorder)
Congenital Disease	SNOMED	1260203008	Epiphyseal, vertebral, ear dysplasia, nose plus associated findings syndrome (disorder)
Congenital Disease	SNOMED	1260377005	Epilepsy due to congenital anomaly of brain (disorder)

Congenital Disease	SNOMED	1260388004	Hemichorea due to cerebral arteriovenous malformation (disorder)
Congenital Disease	SNOMED	1260450002	Infantile multisystem neurologic, endocrine, pancreatic disease (disorder)
Congenital Disease	SNOMED	1260463008	Isolated focal non-epidermolytic palmoplantar keratoderma (disorder)
Congenital Disease	SNOMED	1263449003	Isolated encephalocele (disorder)
Congenital Disease	SNOMED	1263461006	Isolated congenital distichiasis (disorder)
Congenital Disease	SNOMED	12674005	Multiple malformation syndrome with senile-like appearance (disorder)
Congenital Disease	SNOMED	126762003	Talocalcaneal coalition (disorder)
Congenital Disease	SNOMED	126763008	Congenital anomaly of perineum (disorder)
Congenital Disease	SNOMED	126764002	Congenital anomaly of intestinal tract (disorder)
Congenital Disease	SNOMED	127047000	Sickle cell-hemoglobin Lepore disease (disorder)
Congenital Disease	SNOMED	127063008	Erythrocytosis due to cyanotic congenital heart disease (disorder)
Congenital Disease	SNOMED	1271009	Knuckle pads, leukonychia, sensorineural deafness, palmoplantar hyperkeratosis syndrome (disorder)
Congenital Disease	SNOMED	12721007	Trifid tongue (disorder)
Congenital Disease	SNOMED	127328006	Congenital absence of skeletal bone (disorder)
Congenital Disease	SNOMED	127329003	Congenital anomaly of visual system (disorder)
Congenital Disease	SNOMED	127551000119100	Congenital hypoplasia of brain (disorder)
Congenital Disease	SNOMED	12770006	Cyanotic congenital heart disease (disorder)
Congenital Disease	SNOMED	127701000119109	Congenital positive ulnar variant of wrist (disorder)
Congenital Disease	SNOMED	1280009	Isologous chimera (disorder)
Congenital Disease	SNOMED	128061008	Longitudinal deficiency of foot (disorder)
Congenital Disease	SNOMED	128062001	Congenital portal-systemic shunt (disorder)
Congenital Disease	SNOMED	128063006	Congenital extrahepatic portal-systemic shunt (disorder)
Congenital Disease	SNOMED	128064000	Congenital absence of portal vein (disorder)
Congenital Disease	SNOMED	128065004	Congenital partial portal-systemic shunt (disorder)
Congenital Disease	SNOMED	128066003	Congenital splenorenal shunt (disorder)
Congenital Disease	SNOMED	128067007	Congenital intrahepatic portal-systemic shunt (disorder)
Congenital Disease	SNOMED	128096008	Hereditary platelet function disorder (disorder)
Congenital Disease	SNOMED	128124001	Congenital anomaly of central nervous system (disorder)
Congenital Disease	SNOMED	128219005	Dysostosis of bone of skull (disorder)
Congenital Disease	SNOMED	128274005	Congenital anomaly of nose (disorder)
Congenital Disease	SNOMED	128275006	Congenital anomaly of nasal sinuses (disorder)
Congenital Disease	SNOMED	128327004	Congenital anomaly of ocular adnexa (disorder)
Congenital Disease	SNOMED	128332003	Congenital anomaly of digestive organ (disorder)
Congenital Disease	SNOMED	128334002	Congenital anomaly of mouth (disorder)
Congenital Disease	SNOMED	128335001	Congenital anomaly of duodenum (disorder)
Congenital Disease	SNOMED	128336000	Congenital anomaly of palate (disorder)
Congenital Disease	SNOMED	128346003	Congenital anomaly of peritoneum (disorder)
Congenital Disease	SNOMED	128347007	Congenital anomaly of gastrointestinal tract (disorder)
Congenital Disease	SNOMED	128352002	Congenital anomaly of cornea (disorder)
Congenital Disease	SNOMED	128353007	Congenital anomaly of lens (disorder)
Congenital Disease	SNOMED	128533009	Micropapilla (disorder)
Congenital Disease	SNOMED	128534003	Congenital anomaly of posterior segment of eye (disorder)
Congenital Disease	SNOMED	128544001	Congenital anomaly of talus (disorder)
Congenital Disease	SNOMED	128555001	Congenital coronary artery fistula to left atrium (disorder)
Congenital Disease	SNOMED	128556000	Congenital coronary artery fistula to left ventricle (disorder)
Congenital Disease	SNOMED	128557009	Congenital coronary artery fistula to right atrium (disorder)
Congenital Disease	SNOMED	128558004	Congenital coronary artery fistula to right ventricle (disorder)
Congenital Disease	SNOMED	128563000	Juxtaposed atrial appendage (disorder)
Congenital Disease	SNOMED	128566008	Congenital pulmonary vein confluence (disorder)
Congenital Disease	SNOMED	128567004	Congenital pulmonary venous atrium (disorder)
Congenital Disease	SNOMED	128568009	Congenital systemic venous atrium (disorder)
Congenital Disease	SNOMED	128584005	Congenital pulmonary artery conduit (disorder)
Congenital Disease	SNOMED	12897005	Congenital stricture of ureteropelvic junction (disorder)
Congenital Disease	SNOMED	128985003	Congenital anomaly of iris (disorder)
Congenital Disease	SNOMED	129582000	Congenital coronary artery fistula to pulmonary artery (disorder)
Congenital Disease	SNOMED	129601002	Congenital neurogenic ileus syndrome (disorder)
Congenital Disease	SNOMED	13015741000119105	Congenital ocular melanocytosis of left eye (disorder)
Congenital Disease	SNOMED	13015781000119100	Congenital ocular melanocytosis of right eye (disorder)
Congenital Disease	SNOMED	13015821000119105	Congenital ocular melanocytosis of bilateral eyes (disorder)
Congenital Disease	SNOMED	13059002	Congenital ichthyosis of skin (disorder)
Congenital Disease	SNOMED	13206009	Trachea displaced (disorder)
Congenital Disease	SNOMED	13236000	Congenital spondylolisthesis (disorder)
Congenital Disease	SNOMED	13280000	Femoral hypoplasia - unusual facies syndrome (disorder)
Congenital Disease	SNOMED	13282008	Congenital corneal opacity interfering with vision (disorder)
Congenital Disease	SNOMED	13401001	Ablepharon (disorder)
Congenital Disease	SNOMED	134219008	Os trigonum (disorder)
Congenital Disease	SNOMED	13449007	Melnick-Needles syndrome (disorder)
Congenital Disease	SNOMED	13453009	Anomalous muscle bands of left ventricle (disorder)
Congenital Disease	SNOMED	13491000119101	Vesicoureteric reflux due to duplicated collecting system (disorder)
Congenital Disease	SNOMED	13499006	Congenital anomaly of lacrimal system (disorder)
Congenital Disease	SNOMED	13530005	Congenital lobulation of kidney (disorder)
Congenital Disease	SNOMED	13555004	Ring chromosome 22 syndrome (disorder)
Congenital Disease	SNOMED	13568007	Congenital duplication of stomach (disorder)
Congenital Disease	SNOMED	13624003	Congenital cleft hand (disorder)

Congenital Disease	SNOMED	13630003	Congenital absence of lobe of liver (disorder)
Congenital Disease	SNOMED	13649004	Brachycephaly (disorder)
Congenital Disease	SNOMED	13671009	Congenital stricture of urethra (disorder)
Congenital Disease	SNOMED	13674001	Anomaly of chromosome pair 3 (disorder)
Congenital Disease	SNOMED	13689005	Congenital anomaly of aortic valve (disorder)
Congenital Disease	SNOMED	1372004	Uterus unicornis (disorder)
Congenital Disease	SNOMED	13806003	Congenital anomaly of urethra (disorder)
Congenital Disease	SNOMED	13851000119109	Congenital facial asymmetry (disorder)
Congenital Disease	SNOMED	13867009	Preductal coarctation of aorta (disorder)
Congenital Disease	SNOMED	1393001	Lenz-Majewski hyperostosis syndrome (disorder)
Congenital Disease	SNOMED	14061004	Derencephalus (disorder)
Congenital Disease	SNOMED	14091009	12p partial trisomy syndrome (disorder)
Congenital Disease	SNOMED	14178006	Double renal pelvis (disorder)
Congenital Disease	SNOMED	1418007	Hypoplastic chondrodystrophy (disorder)
Congenital Disease	SNOMED	142191000119104	Congenital transverse septate vagina (disorder)
Congenital Disease	SNOMED	142201000119101	Congenital longitudinal septate vagina (disorder)
Congenital Disease	SNOMED	14365001	Congenital prolapse of bladder (disorder)
Congenital Disease	SNOMED	14430002	Congenital stenosis of small intestine (disorder)
Congenital Disease	SNOMED	14447001	Dandy-Walker syndrome (disorder)
Congenital Disease	SNOMED	14482000	Anomalous origin of right subclavian artery (disorder)
Congenital Disease	SNOMED	14532008	Congenital anomaly of trachea (disorder)
Congenital Disease	SNOMED	14552009	Vestigial remnants of canal of Cloquet (disorder)
Congenital Disease	SNOMED	14582003	Microstomia (disorder)
Congenital Disease	SNOMED	14689000	Uterus cordiformis (disorder)
Congenital Disease	SNOMED	1479009	20q partial trisomy (disorder)
Congenital Disease	SNOMED	14821001	Situs ambiguus (disorder)
Congenital Disease	SNOMED	14870002	Achondrogenesis, type IB (disorder)
Congenital Disease	SNOMED	14886009	Abdominal heart (disorder)
Congenital Disease	SNOMED	14911005	Subphrenic interposition syndrome (disorder)
Congenital Disease	SNOMED	1492007	Congenital anomaly of large intestine (disorder)
Congenital Disease	SNOMED	14921002	Aarskog syndrome (disorder)
Congenital Disease	SNOMED	15069006	Russell-Silver syndrome (disorder)
Congenital Disease	SNOMED	1512006	Congenital stricture of bile duct (disorder)
Congenital Disease	SNOMED	15135007	Congenital transposition of stomach (disorder)
Congenital Disease	SNOMED	15182000	Coffin-Lowry syndrome (disorder)
Congenital Disease	SNOMED	1519002	Congenital phlebectasia (disorder)
Congenital Disease	SNOMED	15191001	Origin of innominate artery from left side of aortic arch (disorder)
Congenital Disease	SNOMED	15228007	Atrophia bulborum hereditaria (disorder)
Congenital Disease	SNOMED	15253005	Facial asymmetry (disorder)
Congenital Disease	SNOMED	15419008	Congenital pyloric membrane (disorder)
Congenital Disease	SNOMED	1542009	Omphalocele with obstruction (disorder)
Congenital Disease	SNOMED	15453007	Congenital dislocation of knee with genu recurvatum (disorder)
Congenital Disease	SNOMED	15459006	Endocardial cushion defect (disorder)
Congenital Disease	SNOMED	15523002	Benign focal epilepsy of childhood (disorder)
Congenital Disease	SNOMED	15545001	Congenital duplication of uterus (disorder)
Congenital Disease	SNOMED	15552004	Osteogenesis imperfecta, recessive perinatal lethal, with microcephaly AND cataracts (disorder)
Congenital Disease	SNOMED	15557005	First arch syndrome (disorder)
Congenital Disease	SNOMED	15582005	Congenital absence of lobe of lung (disorder)
Congenital Disease	SNOMED	15630081000119103	Congenital anteversion of right femur (finding)
Congenital Disease	SNOMED	15630121000119101	Congenital anteversion of left femur (finding)
Congenital Disease	SNOMED	15630161000119106	Congenital anteversion of bilateral femurs (finding)
Congenital Disease	SNOMED	15637111000119107	Congenital hammer toe of lesser toe of bilateral feet (disorder)
Congenital Disease	SNOMED	15665321000119101	Congenital chorioretinal coloboma of right eye (disorder)
Congenital Disease	SNOMED	15665361000119106	Congenital chorioretinal coloboma of left eye (disorder)
Congenital Disease	SNOMED	15665401000119102	Congenital chorioretinal coloboma of bilateral eyes (disorder)
Congenital Disease	SNOMED	15665641000119103	Anophthalmos of bilateral eyes (disorder)
Congenital Disease	SNOMED	15665801000119104	Congenital capsular cataract of bilateral eyes (disorder)
Congenital Disease	SNOMED	15665841000119102	Congenital capsular cataract of right eye (disorder)
Congenital Disease	SNOMED	15665881000119107	Congenital capsular cataract of left eye (disorder)
Congenital Disease	SNOMED	15665921000119100	Tarsal coalitions of bilateral feet (disorder)
Congenital Disease	SNOMED	15666081000119105	Accessory bilateral ribs (disorder)
Congenital Disease	SNOMED	15666481000119100	Accessory left tarsal navicular bone (disorder)
Congenital Disease	SNOMED	15666521000119100	Accessory right tarsal navicular bone (disorder)
Congenital Disease	SNOMED	15666561000119105	Accessory bilateral tarsal navicular bones (disorder)
Congenital Disease	SNOMED	15666641000119107	Accessory tarsal bone of right foot (disorder)
Congenital Disease	SNOMED	15666681000119102	Accessory tarsal bone of left foot (disorder)
Congenital Disease	SNOMED	15666721000119108	Accessory carpal bone of left wrist (disorder)
Congenital Disease	SNOMED	15666761000119103	Accessory carpal bone of bilateral wrists (disorder)
Congenital Disease	SNOMED	15666801000119106	Accessory carpal bone of right wrist (disorder)
Congenital Disease	SNOMED	15667041000119103	Radioulnar synostosis of bilateral upper limbs (disorder)
Congenital Disease	SNOMED	15667401000119106	Right metatarsus adductus (disorder)
Congenital Disease	SNOMED	15667441000119108	Bilateral metatarsus adductus (disorder)
Congenital Disease	SNOMED	15667481000119103	Left metatarsus adductus (disorder)
Congenital Disease	SNOMED	15667841000119106	Congenital deformity of bilateral hip joints (disorder)

Congenital Disease	SNOMED	15667881000119101	Congenital deformity of right hip joint (disorder)
Congenital Disease	SNOMED	15667921000119108	Congenital deformity of left hip joint (disorder)
Congenital Disease	SNOMED	15668321000119108	Congenital pes valgo planus of bilateral feet (disorder)
Congenital Disease	SNOMED	15668521000119104	Talipes calcaneovalgus of bilateral feet (disorder)
Congenital Disease	SNOMED	15668561000119109	Talipes valgus of left foot (disorder)
Congenital Disease	SNOMED	15668601000119109	Talipes valgus of right foot (disorder)
Congenital Disease	SNOMED	15668641000119106	Talipes valgus of bilateral feet (disorder)
Congenital Disease	SNOMED	15668721000119107	Congenital deformity of bilateral feet (disorder)
Congenital Disease	SNOMED	15668761000119102	Congenital deformity of bilateral hands (disorder)
Congenital Disease	SNOMED	15668801000119105	Congenital deformity of right hand (disorder)
Congenital Disease	SNOMED	15668921000119109	Congenital deformity of left hand (disorder)
Congenital Disease	SNOMED	15669241000119104	Congenital absence of left lower limb (disorder)
Congenital Disease	SNOMED	15669321000119104	Congenital absence of right lower limb (disorder)
Congenital Disease	SNOMED	15669601000119103	Brachydactyly of finger of right hand (disorder)
Congenital Disease	SNOMED	15669641000119101	Brachydactyly of finger of left hand (disorder)
Congenital Disease	SNOMED	15669681000119106	Brachydactyly of finger of bilateral hands (disorder)
Congenital Disease	SNOMED	15669721000119100	Talipes of bilateral feet (disorder)
Congenital Disease	SNOMED	15669761000119105	Talipes of right foot (disorder)
Congenital Disease	SNOMED	15669801000119102	Talipes of left foot (disorder)
Congenital Disease	SNOMED	15669881000119105	Congenital retroversion of bilateral femurs (finding)
Congenital Disease	SNOMED	15670201000119103	Congenital elevation of bilateral scapulae (disorder)
Congenital Disease	SNOMED	15671007	Encephalocele of orbit (disorder)
Congenital Disease	SNOMED	15671281000119104	Brachydactyly of toes of left foot (disorder)
Congenital Disease	SNOMED	15671321000119109	Brachydactyly of toes of bilateral feet (disorder)
Congenital Disease	SNOMED	15671361000119104	Brachydactyly of toes of right foot (disorder)
Congenital Disease	SNOMED	15672201000119104	Macrodactyly of finger of right hand (disorder)
Congenital Disease	SNOMED	15672241000119102	Macrodactyly of finger of left hand (disorder)
Congenital Disease	SNOMED	15673041000119101	Congenital glaucoma of right eye (disorder)
Congenital Disease	SNOMED	156936000	Agenesis of lung (disorder)
Congenital Disease	SNOMED	15714081000119105	Acquired bilateral hallux valgus due to metatarsus primus varus (disorder)
Congenital Disease	SNOMED	15714121000119107	Acquired left hallux valgus due to metatarsus primus varus (disorder)
Congenital Disease	SNOMED	15714161000119102	Acquired right hallux valgus due to metatarsus primus varus (disorder)
Congenital Disease	SNOMED	15715321000119108	Congenital bilateral internal tibial torsion (finding)
Congenital Disease	SNOMED	15718481000119105	Os acromiale of right scapula (disorder)
Congenital Disease	SNOMED	15718521000119105	Os acromiale of left scapula (disorder)
Congenital Disease	SNOMED	15788006	Congenital cleft thyroid cartilage (disorder)
Congenital Disease	SNOMED	15841002	21q partial monosomy syndrome (disorder)
Congenital Disease	SNOMED	15843004	Congenital absence of vertebra (disorder)
Congenital Disease	SNOMED	15863971000119106	Cyst of bilateral preauricular regions (disorder)
Congenital Disease	SNOMED	15864011000119101	Cyst of left preauricular region (disorder)
Congenital Disease	SNOMED	15864051000119100	Cyst of right preauricular region (disorder)
Congenital Disease	SNOMED	15864091000119105	Congenital malformation of bilateral ears (disorder)
Congenital Disease	SNOMED	15864131000119107	Congenital anomaly of left ear (disorder)
Congenital Disease	SNOMED	15864171000119105	Congenital anomaly of right ear (disorder)
Congenital Disease	SNOMED	15864211000119107	Congenital atresia of bilateral external ears (disorder)
Congenital Disease	SNOMED	15864251000119108	Congenital atresia of left external ear (disorder)
Congenital Disease	SNOMED	15864291000119103	Congenital atresia of right external ear (disorder)
Congenital Disease	SNOMED	15864481000119102	Congenital deformity of toe of bilateral feet (disorder)
Congenital Disease	SNOMED	15864521000119102	Congenital deformity of toe of left foot (disorder)
Congenital Disease	SNOMED	15864561000119107	Congenital deformity of toe of right foot (disorder)
Congenital Disease	SNOMED	15864601000119107	Congenital overriding toes of bilateral feet (disorder)
Congenital Disease	SNOMED	15864641000119109	Congenital overriding toes of left foot (disorder)
Congenital Disease	SNOMED	15864681000119104	Congenital overriding toes of right foot (disorder)
Congenital Disease	SNOMED	15913071000119102	Abnormal gluteal crease (finding)
Congenital Disease	SNOMED	15916651000119107	Congenital pes planus of bilateral feet (disorder)
Congenital Disease	SNOMED	15931821000119104	Congenital torsion of left ovary (disorder)
Congenital Disease	SNOMED	15931861000119109	Congenital torsion of right ovary (disorder)
Congenital Disease	SNOMED	15934341000119103	Congenital bilateral short Achilles tendons (disorder)
Congenital Disease	SNOMED	15935701000119103	Congenital deformity of left clavicle (disorder)
Congenital Disease	SNOMED	15935781000119106	Congenital deformity of right clavicle (disorder)
Congenital Disease	SNOMED	15937301000119106	Congenital distichiasis of left eyelid (disorder)
Congenital Disease	SNOMED	15937341000119108	Congenital distichiasis of right eyelid (disorder)
Congenital Disease	SNOMED	15959861000119107	Embryonic cyst of right Gartner's duct (disorder)
Congenital Disease	SNOMED	15959901000119101	Embryonic cyst of left Gartner's duct (disorder)
Congenital Disease	SNOMED	15960021000119107	Dilatation of aortic [REDACTED] due to Marfan's syndrome (disorder)
Congenital Disease	SNOMED	15964981000119104	Congenital anomaly of cardiac chamber (disorder)
Congenital Disease	SNOMED	15969009	Desmiognathus (disorder)
Congenital Disease	SNOMED	15973301000119100	Congenital deformity of bilateral lower limbs (disorder)
Congenital Disease	SNOMED	15973381000119108	Congenital malformation of right calcaneus (disorder)
Congenital Disease	SNOMED	15973421000119104	Congenital malformation of left calcaneus (disorder)
Congenital Disease	SNOMED	15973541000119106	Congenital deformity of right lower limb (disorder)
Congenital Disease	SNOMED	15973621000119101	Congenital deformity of left lower limb (disorder)
Congenital Disease	SNOMED	15980791000119108	Congenital anomaly of left eyelid (disorder)
Congenital Disease	SNOMED	15980831000119102	Congenital anomaly of right eyelid (disorder)
Congenital Disease	SNOMED	15980871000119104	Congenital blepharophimosis of bilateral eyelids (disorder)

Congenital Disease	SNOMED	15983231000119107	Congenital malformation of bilateral external ears (disorder)
Congenital Disease	SNOMED	15983431000119106	Accessory auricle of right ear (disorder)
Congenital Disease	SNOMED	15983471000119109	Accessory auricle of left ear (disorder)
Congenital Disease	SNOMED	15984431000119109	Impairment of hearing of right ear co-occurrent and due to congenital ear malformation (disorder)
Congenital Disease	SNOMED	15984471000119107	Impairment of hearing of left ear co-occurrent and due to congenital ear malformation (disorder)
Congenital Disease	SNOMED	15984511000119103	Impairment of hearing of bilateral ears co-occurrent and due to congenital ear malformation (disorder)
Congenital Disease	SNOMED	15985951000119109	Congenital malformation of blood vessel of bilateral orbits proper (disorder)
Congenital Disease	SNOMED	15985991000119104	Congenital malformation of blood vessel of right orbit proper (disorder)
Congenital Disease	SNOMED	15986031000119105	Congenital malformation of blood vessel of left orbit proper (disorder)
Congenital Disease	SNOMED	15986071000119108	Congenital anomaly of right pupil (disorder)
Congenital Disease	SNOMED	15986111000119101	Congenital coloboma of bilateral optic discs (disorder)
Congenital Disease	SNOMED	15986151000119100	Congenital coloboma of right optic disc (disorder)
Congenital Disease	SNOMED	15986191000119105	Congenital coloboma of left optic disc (disorder)
Congenital Disease	SNOMED	15986231000119101	Congenital anomaly of right optic disc (disorder)
Congenital Disease	SNOMED	15986271000119103	Congenital anomaly of bilateral optic discs (disorder)
Congenital Disease	SNOMED	15986311000119103	Congenital anomaly of left optic disc (disorder)
Congenital Disease	SNOMED	15986471000119104	Congenital anomaly of retina of right eye (disorder)
Congenital Disease	SNOMED	15986511000119108	Congenital hypertrophy of retinal pigment epithelium of right eye (disorder)
Congenital Disease	SNOMED	15986551000119109	Congenital hypertrophy of retinal pigment epithelium of bilateral eyes (disorder)
Congenital Disease	SNOMED	15986591000119104	Congenital hypertrophy of retinal pigment epithelium of left eye (disorder)
Congenital Disease	SNOMED	15986751000119101	Congenital anomaly of retina of left eye (disorder)
Congenital Disease	SNOMED	15986791000119106	Congenital anomaly of bilateral retinas (disorder)
Congenital Disease	SNOMED	15986831000119100	Persistent hyperplastic primary vitreous of left eye (disorder)
Congenital Disease	SNOMED	15986871000119102	Persistent hyperplastic primary vitreous of right eye (disorder)
Congenital Disease	SNOMED	15986951000119103	Congenital aniridia of right eye (disorder)
Congenital Disease	SNOMED	15986991000119108	Congenital aniridia of left eye (disorder)
Congenital Disease	SNOMED	15987031000119108	Congenital aniridia of bilateral eyes (disorder)
Congenital Disease	SNOMED	15987071000119106	Congenital anomaly of left pupil (disorder)
Congenital Disease	SNOMED	15987111000119104	Congenital anomaly of bilateral pupils (disorder)
Congenital Disease	SNOMED	15987151000119103	Microphthalmos of bilateral eyes (disorder)
Congenital Disease	SNOMED	15987191000119108	Microphthalmos of right eye (disorder)
Congenital Disease	SNOMED	15987231000119104	Microphthalmos of left eye (disorder)
Congenital Disease	SNOMED	15987391000119105	Congenital opacity of cornea of left eye (disorder)
Congenital Disease	SNOMED	15987431000119100	Congenital opacity of cornea of right eye (disorder)
Congenital Disease	SNOMED	15987551000119107	Congenital anomaly of bilateral corneas (disorder)
Congenital Disease	SNOMED	15987871000119100	Congenital coloboma of right lens (disorder)
Congenital Disease	SNOMED	15987911000119102	Congenital coloboma of left lens (disorder)
Congenital Disease	SNOMED	15988031000119109	Congenital coloboma of left eyelid (disorder)
Congenital Disease	SNOMED	15988071000119107	Congenital coloboma of right eyelid (disorder)
Congenital Disease	SNOMED	15988111000119100	Congenital anomaly of bilateral sclerae (disorder)
Congenital Disease	SNOMED	15988151000119104	Congenital anomaly of left sclera (disorder)
Congenital Disease	SNOMED	15988191000119109	Congenital anomaly of right sclera (disorder)
Congenital Disease	SNOMED	15988231000119100	Congenital abnormality of left lacrimal drainage system (disorder)
Congenital Disease	SNOMED	15988271000119102	Congenital abnormality of right lacrimal drainage system (disorder)
Congenital Disease	SNOMED	15988311000119102	Congenital abnormality of bilateral lacrimal drainage systems (disorder)
Congenital Disease	SNOMED	15993551000119100	Disorder of eye co-occurrent and due to Marfan syndrome (disorder)
Congenital Disease	SNOMED	16001191000119109	Buphthalmos of left eye (finding)
Congenital Disease	SNOMED	16001231000119100	Buphthalmos of right eye (finding)
Congenital Disease	SNOMED	16004071000119105	Congenital trigger thumb of bilateral hands (disorder)
Congenital Disease	SNOMED	16004111000119103	Congenital trigger thumb of right hand (disorder)
Congenital Disease	SNOMED	16004151000119102	Congenital trigger thumb of left hand (disorder)
Congenital Disease	SNOMED	16007311000119107	Congenital abnormal fusion of right carpal bones (disorder)
Congenital Disease	SNOMED	16007351000119108	Congenital abnormal fusion of left carpal bones (disorder)
Congenital Disease	SNOMED	16007391000119103	Congenital abnormal fusion of bilateral carpal bones (disorder)
Congenital Disease	SNOMED	16008831000119109	Congenital dysplasia of left upper limb (disorder)
Congenital Disease	SNOMED	16009071000119106	Congenital dysplasia of right upper limb (disorder)
Congenital Disease	SNOMED	16009151000119103	Congenital dysplasia of bilateral upper limbs (disorder)
Congenital Disease	SNOMED	16009191000119108	Congenital deformity of bilateral upper limbs (disorder)
Congenital Disease	SNOMED	16018591000119107	Congenital trigger finger of right hand (disorder)
Congenital Disease	SNOMED	16018671000119105	Congenital trigger finger of left hand (disorder)
Congenital Disease	SNOMED	16018711000119109	Congenital deformity of right finger (disorder)
Congenital Disease	SNOMED	16018751000119105	Congenital deformity of left finger (disorder)
Congenital Disease	SNOMED	16018791000119100	Congenital deformity of bilateral fingers (disorder)
Congenital Disease	SNOMED	16026008	Congenital cerebellar hypoplasia (disorder)
Congenital Disease	SNOMED	16026031000119109	Congenital arteriovenous malformation of right lower limb (disorder)
Congenital Disease	SNOMED	16026151000119104	Congenital arteriovenous malformation of left lower limb (disorder)
Congenital Disease	SNOMED	16054391000119102	Congenital hypoplasia of right optic nerve (disorder)
Congenital Disease	SNOMED	16054431000119107	Congenital hypoplasia of left optic nerve (disorder)
Congenital Disease	SNOMED	16054471000119105	Congenital hypoplasia of bilateral optic nerves (disorder)
Congenital Disease	SNOMED	16055631000119106	Disorder of cardiovascular system co-occurrent and due to Marfan syndrome (disorder)

Congenital Disease	SNOMED	16064571000119103	Congenital hamartoma of bilateral irises (disorder)
Congenital Disease	SNOMED	16090091000119100	Congenital absence of left hand (disorder)
Congenital Disease	SNOMED	16095003	Heterodymus (disorder)
Congenital Disease	SNOMED	16129004	10q partial monosomy (disorder)
Congenital Disease	SNOMED	16297002	Congenital hydronephrosis (disorder)
Congenital Disease	SNOMED	16376000	Congenital duodenal stenosis (disorder)
Congenital Disease	SNOMED	16469741000119106	Microperforate hymen (disorder)
Congenital Disease	SNOMED	16507009	Ectopic kidney (disorder)
Congenital Disease	SNOMED	16512005	Congenital absence of membranous labyrinth (disorder)
Congenital Disease	SNOMED	165185004	Dermatoglyphs - skin lines (disorder)
Congenital Disease	SNOMED	16520041000119104	Congenital fenestration of basilar artery (disorder)
Congenital Disease	SNOMED	16528221000119104	Congenital duplication of anterior communicating artery (disorder)
Congenital Disease	SNOMED	16567006	Mesocardia (disorder)
Congenital Disease	SNOMED	16569009	Anomaly of chromosome pair 15 (disorder)
Congenital Disease	SNOMED	16585004	Congenital spade-like hand (disorder)
Congenital Disease	SNOMED	16603000	Talipomanus (disorder)
Congenital Disease	SNOMED	16619007	Congenital occlusion of ureter (disorder)
Congenital Disease	SNOMED	1667003	Early fontanel closure (finding)
Congenital Disease	SNOMED	16696981000119105	Congenital anomaly of bilateral renal arteries (disorder)
Congenital Disease	SNOMED	16697061000119100	Congenital anomaly of left renal artery (disorder)
Congenital Disease	SNOMED	16697181000119105	Congenital anomaly of right renal artery (disorder)
Congenital Disease	SNOMED	16856000	Longitudinal deficiency of humerus (disorder)
Congenital Disease	SNOMED	16888881000119105	Congenital stenosis of right external auditory canal (disorder)
Congenital Disease	SNOMED	16889021000119105	Congenital stenosis of left external auditory canal (disorder)
Congenital Disease	SNOMED	16889061000119100	Congenital stenosis of bilateral external auditory canals (disorder)
Congenital Disease	SNOMED	16904009	Incomplete congenital absence of thigh AND leg (disorder)
Congenital Disease	SNOMED	1694004	Accessory lobe of lung (disorder)
Congenital Disease	SNOMED	16972009	Congenital aneurysm of aorta (disorder)
Congenital Disease	SNOMED	17024001	Aortopulmonary window (disorder)
Congenital Disease	SNOMED	17025000	Ehlers-Danlos syndrome, type 4 (disorder)
Congenital Disease	SNOMED	171131006	Meningocele (disorder)
Congenital Disease	SNOMED	17122004	4p partial monosomy syndrome (disorder)
Congenital Disease	SNOMED	17128000	Congenital eventration of diaphragm (disorder)
Congenital Disease	SNOMED	17142008	Congenital absence of uterus (disorder)
Congenital Disease	SNOMED	17144009	Fibrochondrogenesis (disorder)
Congenital Disease	SNOMED	17170005	Pili torti (disorder)
Congenital Disease	SNOMED	17190001	Congenital diaphragmatic hernia (disorder)
Congenital Disease	SNOMED	172069000	Congenital meningocele (disorder)
Congenital Disease	SNOMED	17231009	Fetal valproate syndrome (disorder)
Congenital Disease	SNOMED	17234001	Allantoic cyst (disorder)
Congenital Disease	SNOMED	17268007	Congenital clinodactyly (disorder)
Congenital Disease	SNOMED	17337006	Double artery (disorder)
Congenital Disease	SNOMED	17394001	Ebstein's anomaly with atrial septal defect (disorder)
Congenital Disease	SNOMED	17422006	Ectopic thymic tissue (disorder)
Congenital Disease	SNOMED	17471001	Polyorchism (disorder)
Congenital Disease	SNOMED	17480001	Atresia of pupil (disorder)
Congenital Disease	SNOMED	17484005	Sacralization of lumbar vertebra (disorder)
Congenital Disease	SNOMED	17527002	Overriding skull bones (disorder)
Congenital Disease	SNOMED	17568006	Sclerosteosis (disorder)
Congenital Disease	SNOMED	17601009	Congenital subluxation of carpus (disorder)
Congenital Disease	SNOMED	17608003	Child syndrome (disorder)
Congenital Disease	SNOMED	1769008	Thoracodidymus (disorder)
Congenital Disease	SNOMED	17718000	Ostium primum defect (disorder)
Congenital Disease	SNOMED	177504007	Acheiropodia (disorder)
Congenital Disease	SNOMED	17760001	Anomaly of chromosome pair 13 (disorder)
Congenital Disease	SNOMED	17761000119109	High lumbar myelomeningocele (disorder)
Congenital Disease	SNOMED	17771000119103	Low lumbar myelomeningocele (disorder)
Congenital Disease	SNOMED	1779005	Mohr syndrome (disorder)
Congenital Disease	SNOMED	17808001	Azygos lobe of lung (disorder)
Congenital Disease	SNOMED	17818006	Leri-Weill dyschondrosteosis (disorder)
Congenital Disease	SNOMED	18077009	Trichorhinophalangeal syndrome (disorder)
Congenital Disease	SNOMED	18132009	Congenital deformity of sacroiliac joint (disorder)
Congenital Disease	SNOMED	18166000	Accessory breast (disorder)
Congenital Disease	SNOMED	18241005	Early urethral obstruction sequence (disorder)
Congenital Disease	SNOMED	18269002	Congenital duodenal obstruction (disorder)
Congenital Disease	SNOMED	18389004	Microcolon (disorder)
Congenital Disease	SNOMED	18417009	Oligomeganephronic hypoplasia of kidney (disorder)
Congenital Disease	SNOMED	1856001	Accessory nose (disorder)
Congenital Disease	SNOMED	18620009	Congenital sequestration of lung (disorder)
Congenital Disease	SNOMED	18735004	Congenital omphalocele (disorder)
Congenital Disease	SNOMED	18749008	Congenital koilonychia (disorder)
Congenital Disease	SNOMED	18792003	H-type congenital tracheoesophageal fistula (disorder)
Congenital Disease	SNOMED	18820007	Preauricular cyst (disorder)
Congenital Disease	SNOMED	18821006	Dysplasia of eye (disorder)
Congenital Disease	SNOMED	18899000	Schinzel-Giedion syndrome (disorder)

Congenital Disease	SNOMED	18910001	Cleft uvula (disorder)
Congenital Disease	SNOMED	1896004	Ectopic breast tissue (disorder)
Congenital Disease	SNOMED	1899006	Autosomal hereditary disorder (disorder)
Congenital Disease	SNOMED	19042000	Congenital prolapse of urethra (disorder)
Congenital Disease	SNOMED	19092004	Holt-Oram syndrome (disorder)
Congenital Disease	SNOMED	191202009	Hemoglobin Zurich disease (disorder)
Congenital Disease	SNOMED	19133005	Neurofibromatosis syndrome (disorder)
Congenital Disease	SNOMED	19179000	Ischiopagus (disorder)
Congenital Disease	SNOMED	19216006	Esophageal web (disorder)
Congenital Disease	SNOMED	1922008	Congenital absence of urethra (disorder)
Congenital Disease	SNOMED	19249002	Premature closure of foramen ovale (disorder)
Congenital Disease	SNOMED	1926006	Osteopetrosis (disorder)
Congenital Disease	SNOMED	19276002	Congenital cerebral cyst (disorder)
Congenital Disease	SNOMED	192814005	Cerebral degeneration due to congenital hydrocephalus (disorder)
Congenital Disease	SNOMED	19346006	Marfan's syndrome (disorder)
Congenital Disease	SNOMED	193553001	Glaucoma due to iris anomaly (disorder)
Congenital Disease	SNOMED	19387007	Ectopic pancreas (disorder)
Congenital Disease	SNOMED	193994000	Congenital nasolacrimal duct obstruction (disorder)
Congenital Disease	SNOMED	194023005	Orbital deformity associated with craniofacial deformity (disorder)
Congenital Disease	SNOMED	19416009	Congenital anomaly of eye (disorder)
Congenital Disease	SNOMED	19419002	8p partial monosomy syndrome (disorder)
Congenital Disease	SNOMED	19441002	Occipital dysplasia (disorder)
Congenital Disease	SNOMED	1953005	Congenital deficiency of pigment of skin (disorder)
Congenital Disease	SNOMED	19550003	22q partial monosomy (disorder)
Congenital Disease	SNOMED	1955003	Preauricular dimple (disorder)
Congenital Disease	SNOMED	1967001	Longitudinal absence of radius AND ulna (disorder)
Congenital Disease	SNOMED	196856007	Omphalocele with gangrene (disorder)
Congenital Disease	SNOMED	196864001	Omphalocele - irreducible (disorder)
Congenital Disease	SNOMED	197140007	Secondary congenital megacolon (disorder)
Congenital Disease	SNOMED	19833008	Nodular calcific aortic valve stenosis (disorder)
Congenital Disease	SNOMED	19886006	Sturge-Weber syndrome (disorder)
Congenital Disease	SNOMED	199527003	Fetus with chromosomal abnormality with antenatal problem (disorder)
Congenital Disease	SNOMED	199879009	Congenital anomaly of skin (disorder)
Congenital Disease	SNOMED	201055009	Symmetrical keratoderma (disorder)
Congenital Disease	SNOMED	201161009	Hair nevus (disorder)
Congenital Disease	SNOMED	20136007	Accessory carpal bones (disorder)
Congenital Disease	SNOMED	201698009	Hexadactyly (disorder)
Congenital Disease	SNOMED	20348002	14q partial distal trisomy syndrome (disorder)
Congenital Disease	SNOMED	203663000	Scoliosis in neurofibromatosis (disorder)
Congenital Disease	SNOMED	20392000	Congenital entropion (disorder)
Congenital Disease	SNOMED	203923004	Acrania (disorder)
Congenital Disease	SNOMED	203927003	Iniencephaly - closed (disorder)
Congenital Disease	SNOMED	203928008	Iniencephaly - open (disorder)
Congenital Disease	SNOMED	203934001	Cervical spina bifida with hydrocephalus (disorder)
Congenital Disease	SNOMED	203935000	Thoracic spina bifida with hydrocephalus (disorder)
Congenital Disease	SNOMED	203936004	Lumbar spina bifida with hydrocephalus (disorder)
Congenital Disease	SNOMED	203941007	Cervical spina bifida with hydrocephalus - open (disorder)
Congenital Disease	SNOMED	203942000	Thoracic spina bifida with hydrocephalus - open (disorder)
Congenital Disease	SNOMED	203943005	Lumbar spina bifida with hydrocephalus - open (disorder)
Congenital Disease	SNOMED	203944004	Sacral spina bifida with hydrocephalus - open (disorder)
Congenital Disease	SNOMED	203946002	Spina bifida with hydrocephalus - closed (disorder)
Congenital Disease	SNOMED	203948001	Cervical spina bifida with hydrocephalus - closed (disorder)
Congenital Disease	SNOMED	203949009	Thoracic spina bifida with hydrocephalus - closed (disorder)
Congenital Disease	SNOMED	203950009	Lumbar spina bifida with hydrocephalus - closed (disorder)
Congenital Disease	SNOMED	203951008	Sacral spina bifida with hydrocephalus - closed (disorder)
Congenital Disease	SNOMED	203954000	Spina bifida with hydrocephalus of late onset (disorder)
Congenital Disease	SNOMED	203955004	Spina bifida with stenosis of aqueduct of Sylvius (disorder)
Congenital Disease	SNOMED	203957007	Dandy-Walker syndrome with spina bifida (disorder)
Congenital Disease	SNOMED	203980004	Cervical spinal meningocele (disorder)
Congenital Disease	SNOMED	203981000	Thoracic spinal meningocele (disorder)
Congenital Disease	SNOMED	203982007	Lumbar spinal meningocele (disorder)
Congenital Disease	SNOMED	203985009	Cervical meningomyelocele (disorder)
Congenital Disease	SNOMED	203986005	Thoracic meningomyelocele (disorder)
Congenital Disease	SNOMED	203987001	Lumbar meningomyelocele (disorder)
Congenital Disease	SNOMED	203990007	Cervical myelocele (disorder)
Congenital Disease	SNOMED	203991006	Thoracic myelocele (disorder)
Congenital Disease	SNOMED	203992004	Lumbar myelocele (disorder)
Congenital Disease	SNOMED	203994003	Myelocystocele (disorder)
Congenital Disease	SNOMED	203996001	Cervical myelocystocele (disorder)
Congenital Disease	SNOMED	203997005	Thoracic myelocystocele (disorder)
Congenital Disease	SNOMED	203998000	Lumbar myelocystocele (disorder)
Congenital Disease	SNOMED	204003007	Cervical spina bifida without hydrocephalus - open (disorder)
Congenital Disease	SNOMED	204004001	Thoracic spina bifida without hydrocephalus - open (disorder)
Congenital Disease	SNOMED	204005000	Lumbar spina bifida without hydrocephalus - open (disorder)
Congenital Disease	SNOMED	204006004	Sacral spina bifida without hydrocephalus - open (disorder)

Congenital Disease	SNOMED	204008003	Spina bifida without hydrocephalus - closed (disorder)
Congenital Disease	SNOMED	204010001	Cervical spina bifida without hydrocephalus - closed (disorder)
Congenital Disease	SNOMED	204011002	Thoracic spina bifida without hydrocephalus - closed (disorder)
Congenital Disease	SNOMED	204012009	Lumbar spina bifida without hydrocephalus - closed (disorder)
Congenital Disease	SNOMED	204013004	Sacral spina bifida without hydrocephalus - closed (disorder)
Congenital Disease	SNOMED	204021005	Encephalomyelocele (disorder)
Congenital Disease	SNOMED	204036008	Lissencephaly (disorder)
Congenital Disease	SNOMED	204040004	Agenesis of cerebrum (disorder)
Congenital Disease	SNOMED	204042007	Congenital malformation of corpus callosum (disorder)
Congenital Disease	SNOMED	204043002	Hypoplasia of corpus callosum (disorder)
Congenital Disease	SNOMED	204044008	Aplasia of corpus callosum (disorder)
Congenital Disease	SNOMED	204046005	Anomalies of hypothalamus (disorder)
Congenital Disease	SNOMED	204047001	Anomalies of cerebellum (disorder)
Congenital Disease	SNOMED	204049003	Aplasia of cerebellum (disorder)
Congenital Disease	SNOMED	204052006	Cebocephaly (disorder)
Congenital Disease	SNOMED	204061006	Foramen of Magendie atresia (disorder)
Congenital Disease	SNOMED	204062004	Foramen of Luschka atresia (disorder)
Congenital Disease	SNOMED	204067005	Single congenital cerebral cyst (disorder)
Congenital Disease	SNOMED	204068000	Multiple congenital cerebral cysts (disorder)
Congenital Disease	SNOMED	204072001	Congenital adhesions of cerebral meninges (disorder)
Congenital Disease	SNOMED	204074000	Multiple brain anomalies (disorder)
Congenital Disease	SNOMED	204081007	Spinal cord hypoplasia (disorder)
Congenital Disease	SNOMED	204086002	Brachial plexus displacement (disorder)
Congenital Disease	SNOMED	204102004	Cryptophthalmos syndrome (disorder)
Congenital Disease	SNOMED	204108000	Simple microphthalmos (disorder)
Congenital Disease	SNOMED	204113001	Congenital glaucoma (disorder)
Congenital Disease	SNOMED	204118005	Congenital keratoglobus (disorder)
Congenital Disease	SNOMED	204127006	Cortical and zonular cataract (disorder)
Congenital Disease	SNOMED	204128001	Congenital lamellar cataract (disorder)
Congenital Disease	SNOMED	204134008	Coloboma of lens (disorder)
Congenital Disease	SNOMED	204138006	Congenital blue dot cataract (disorder)
Congenital Disease	SNOMED	204139003	Congenital membranous cataract (disorder)
Congenital Disease	SNOMED	204142009	Anterior chamber anomalies (disorder)
Congenital Disease	SNOMED	204143004	Corneal size and shape anomalies (disorder)
Congenital Disease	SNOMED	204145006	Cornea plana (disorder)
Congenital Disease	SNOMED	204148008	Congenital corneal opacity with visual deficit (disorder)
Congenital Disease	SNOMED	204149000	Congenital corneal opacity without visual deficit (disorder)
Congenital Disease	SNOMED	204152008	Axenfeld anomaly (disorder)
Congenital Disease	SNOMED	204153003	Irido-corneo-trabecular dysgenesis (disorder)
Congenital Disease	SNOMED	204154009	Irido-trabecular dysgenesis (disorder)
Congenital Disease	SNOMED	204159004	Polycoria (disorder)
Congenital Disease	SNOMED	204164000	Blue sclera (disorder)
Congenital Disease	SNOMED	204166003	Multiple anterior segment anomalies (disorder)
Congenital Disease	SNOMED	204171005	Congenital vitreous opacity (disorder)
Congenital Disease	SNOMED	204173008	Coloboma of retina (disorder)
Congenital Disease	SNOMED	204175001	Congenital fold and cyst of posterior segment of eye (disorder)
Congenital Disease	SNOMED	204181009	Congenital retinal fold (disorder)
Congenital Disease	SNOMED	204189006	Congenital stricture of retinal artery (disorder)
Congenital Disease	SNOMED	204192005	Coloboma of choroid (disorder)
Congenital Disease	SNOMED	204203001	Hypoplasia of eyelid (disorder)
Congenital Disease	SNOMED	204208005	Agenesis of punctum lacrimale (disorder)
Congenital Disease	SNOMED	204216001	Multiple supernumerary eye muscles (disorder)
Congenital Disease	SNOMED	204217005	Hypoplasia of eye muscle (disorder)
Congenital Disease	SNOMED	204223000	Ear, face and neck congenital anomalies (disorder)
Congenital Disease	SNOMED	204224006	Ear anomalies with hearing impairment (disorder)
Congenital Disease	SNOMED	204245004	Accessory tragus (disorder)
Congenital Disease	SNOMED	204250005	Congenital abnormality of Eustachian tube (disorder)
Congenital Disease	SNOMED	204252002	Congenital stenosis of eustachian tube (disorder)
Congenital Disease	SNOMED	204256004	Congenital pointed ear (disorder)
Congenital Disease	SNOMED	204257008	Congenital prominent auricle (disorder)
Congenital Disease	SNOMED	204258003	Congenital ridge ear (disorder)
Congenital Disease	SNOMED	204266007	Branchial cleft external sinus (disorder)
Congenital Disease	SNOMED	204267003	Branchial cleft internal sinus (disorder)
Congenital Disease	SNOMED	204268008	Fistula of branchial cleft (disorder)
Congenital Disease	SNOMED	204272007	Preauricular fistula (disorder)
Congenital Disease	SNOMED	204285005	Congenital absence of chin (disorder)
Congenital Disease	SNOMED	204296002	Discordant ventriculoarterial connection (disorder)
Congenital Disease	SNOMED	204299009	Dextrotransposition of aorta (disorder)
Congenital Disease	SNOMED	204300001	Incomplete great vessel transposition (disorder)
Congenital Disease	SNOMED	204306007	Pentalogy of Fallot (disorder)
Congenital Disease	SNOMED	204312002	Ventricular septal defect between left ventricle and right atrium (disorder)
Congenital Disease	SNOMED	204315000	Atrial septal defect within oval fossa (disorder)
Congenital Disease	SNOMED	204317008	Patent foramen ovale (disorder)
Congenital Disease	SNOMED	204318003	Persistent ostium secundum (disorder)
Congenital Disease	SNOMED	204319006	Lutembacher's syndrome (disorder)

Congenital Disease	SNOMED	204330009	Common atrioventricular-type ventricular septal defect (disorder)
Congenital Disease	SNOMED	204345002	Congenital fusion of pulmonic cusps (disorder)
Congenital Disease	SNOMED	204351007	Fallot's trilogy (disorder)
Congenital Disease	SNOMED	204354004	Congenital tricuspid atresia and stenosis (disorder)
Congenital Disease	SNOMED	204378009	Congenital coronary aneurysm (disorder)
Congenital Disease	SNOMED	204379001	Congenital stricture of coronary artery (disorder)
Congenital Disease	SNOMED	204383001	Congenital complete atrioventricular heart block (disorder)
Congenital Disease	SNOMED	204384007	Congenital incomplete atrioventricular heart block (disorder)
Congenital Disease	SNOMED	204391005	Hypoplasia of cardiac vein (disorder)
Congenital Disease	SNOMED	204394002	Congenital anomaly of myocardium (disorder)
Congenital Disease	SNOMED	204395001	Congenital aneurysm of heart (disorder)
Congenital Disease	SNOMED	204397009	Cor triloculare (disorder)
Congenital Disease	SNOMED	204398004	Congenital epicardial cyst (disorder)
Congenital Disease	SNOMED	204399007	Hemicardia (disorder)
Congenital Disease	SNOMED	204407002	Anomalous bands of heart (disorder)
Congenital Disease	SNOMED	204409004	Anomalous ventricular bands (disorder)
Congenital Disease	SNOMED	204423002	Anomalous origin of the aortic arch (disorder)
Congenital Disease	SNOMED	204427001	Persistent aortic arch convolutions (disorder)
Congenital Disease	SNOMED	204431007	Atresia and stenosis of aorta (disorder)
Congenital Disease	SNOMED	204433005	Aplasia of aorta (disorder)
Congenital Disease	SNOMED	204448004	Atresia of pulmonary artery with septal defect (disorder)
Congenital Disease	SNOMED	204451006	Anomalies of great veins (disorder)
Congenital Disease	SNOMED	204456001	Subdiaphragmatic total anomalous pulmonary venous return (disorder)
Congenital Disease	SNOMED	204457005	Supradiaphragmatic total anomalous pulmonary venous return (disorder)
Congenital Disease	SNOMED	204460003	Anomalous portal vein termination (disorder)
Congenital Disease	SNOMED	204461004	Portal vein-hepatic artery fistula (disorder)
Congenital Disease	SNOMED	204463001	Absence of inferior vena cava (disorder)
Congenital Disease	SNOMED	204464007	Absence of superior vena cava (disorder)
Congenital Disease	SNOMED	204467000	Pulmonary vein atresia (disorder)
Congenital Disease	SNOMED	204470001	Single umbilical artery (disorder)
Congenital Disease	SNOMED	204482005	Congenital venous varix (disorder)
Congenital Disease	SNOMED	204490005	Hypoplasia of spinal vessel (disorder)
Congenital Disease	SNOMED	204493007	Arteriovenous malformation of precerebral vessels (disorder)
Congenital Disease	SNOMED	204501003	Congenital stricture of cerebral artery (disorder)
Congenital Disease	SNOMED	204508009	Choanal atresia (disorder)
Congenital Disease	SNOMED	204511005	Atresia of the anterior nares (disorder)
Congenital Disease	SNOMED	204513008	Congenital stenosis of the anterior nares (disorder)
Congenital Disease	SNOMED	204519007	Underdevelopment of nose (disorder)
Congenital Disease	SNOMED	204521002	Congenital cleft nose (disorder)
Congenital Disease	SNOMED	204524005	Congenital perforation of the nasal sinus wall (disorder)
Congenital Disease	SNOMED	204526007	Congenital fissure of nose (disorder)
Congenital Disease	SNOMED	204529000	Congenital glottic web of larynx (disorder)
Congenital Disease	SNOMED	204530005	Congenital subglottic web of larynx (disorder)
Congenital Disease	SNOMED	204533007	Agenesis of larynx, trachea and bronchus (disorder)
Congenital Disease	SNOMED	204534001	Agenesis of bronchus (disorder)
Congenital Disease	SNOMED	204535000	Agenesis of larynx (disorder)
Congenital Disease	SNOMED	204537008	Anomaly of laryngeal and/or tracheal cartilage (disorder)
Congenital Disease	SNOMED	204539006	Anomaly of epiglottis (disorder)
Congenital Disease	SNOMED	204544004	Atresia of larynx and trachea (disorder)
Congenital Disease	SNOMED	204550009	Congenital stenosis of larynx, trachea and bronchus (disorder)
Congenital Disease	SNOMED	204551008	Congenital bronchial stenosis (disorder)
Congenital Disease	SNOMED	204552001	Congenital subglottic stenosis (disorder)
Congenital Disease	SNOMED	204553006	Congenital supraglottic stenosis (disorder)
Congenital Disease	SNOMED	204557007	Congenital fissure of epiglottis (disorder)
Congenital Disease	SNOMED	204558002	Congenital cleft of posterior cricoid cartilage (disorder)
Congenital Disease	SNOMED	204575008	Congenital absence of lung fissures (disorder)
Congenital Disease	SNOMED	204578005	Fusion of lobes of lung (disorder)
Congenital Disease	SNOMED	204589003	Abnormal pericardiopleural communication (disorder)
Congenital Disease	SNOMED	204608004	Central cleft lip (disorder)
Congenital Disease	SNOMED	204614006	Bilateral complete cleft palate with cleft lip (disorder)
Congenital Disease	SNOMED	204616008	Central complete cleft palate with cleft lip (disorder)
Congenital Disease	SNOMED	204617004	Central incomplete cleft palate with cleft lip (disorder)
Congenital Disease	SNOMED	204620007	Cleft hard palate with cleft lip, bilateral (disorder)
Congenital Disease	SNOMED	204630003	Cleft tongue (disorder)
Congenital Disease	SNOMED	204631004	Congenital plicated tongue (disorder)
Congenital Disease	SNOMED	204635008	Congenital salivary gland fistula (disorder)
Congenital Disease	SNOMED	204642008	Congenital pharyngeal polyp (disorder)
Congenital Disease	SNOMED	204644009	Congenital ranula (disorder)
Congenital Disease	SNOMED	204647002	Congenital ectropion of lip (disorder)
Congenital Disease	SNOMED	204652007	Congenital esophageal fistula (disorder)
Congenital Disease	SNOMED	204658006	Congenital absence of esophagus with tracheoesophageal fistula (disorder)
Congenital Disease	SNOMED	204659003	Esophageal atresia with tracheoesophageal fistula (disorder)
Congenital Disease	SNOMED	204667006	Congenital esophageal pouch (disorder)
Congenital Disease	SNOMED	204670005	Congenital pyloric spasm (disorder)
Congenital Disease	SNOMED	204671009	Congenital pyloric stenosis (disorder)

Congenital Disease	SNOMED	204676004	Ectopic gastric mucosa (disorder)
Congenital Disease	SNOMED	204687007	Displaced Meckel's diverticulum (disorder)
Congenital Disease	SNOMED	204688002	Hypertrophic Meckel's diverticulum (disorder)
Congenital Disease	SNOMED	204691002	Small intestine atresia and stenosis (disorder)
Congenital Disease	SNOMED	204695006	Congenital absence of duodenum (disorder)
Congenital Disease	SNOMED	204696007	Congenital absence of jejunum (disorder)
Congenital Disease	SNOMED	204697003	Congenital absence of ileum (disorder)
Congenital Disease	SNOMED	204699000	Congenital jejunal stenosis (disorder)
Congenital Disease	SNOMED	204700004	Congenital ileal stenosis (disorder)
Congenital Disease	SNOMED	204702007	Imperforate jejunum (disorder)
Congenital Disease	SNOMED	204708006	Congenital absence of anus with fistula (disorder)
Congenital Disease	SNOMED	204709003	Congenital absence of rectum with fistula (disorder)
Congenital Disease	SNOMED	204711007	Atresia of large intestine (disorder)
Congenital Disease	SNOMED	204712000	Anal atresia (disorder)
Congenital Disease	SNOMED	204715003	Congenital atresia of appendix (disorder)
Congenital Disease	SNOMED	204716002	Atresia of anus with fistula (disorder)
Congenital Disease	SNOMED	204717006	Atresia of rectum with fistula (disorder)
Congenital Disease	SNOMED	204721004	Congenital occlusion of anus with fistula (disorder)
Congenital Disease	SNOMED	204723001	Congenital stricture of anus (disorder)
Congenital Disease	SNOMED	204724007	Congenital stricture of anus with fistula (disorder)
Congenital Disease	SNOMED	204728005	Congenital stricture of rectum with fistula (disorder)
Congenital Disease	SNOMED	204736001	Imperforate large intestine (disorder)
Congenital Disease	SNOMED	204739008	Hirschsprung's disease (disorder)
Congenital Disease	SNOMED	204740005	Long segment Hirschsprung's disease (disorder)
Congenital Disease	SNOMED	204741009	Short segment Hirschsprung's disease (disorder)
Congenital Disease	SNOMED	204745000	Total intestinal aganglionosis (disorder)
Congenital Disease	SNOMED	204748003	Congenital intestinal adhesions (disorder)
Congenital Disease	SNOMED	204750006	Malrotation of colon and cecum (disorder)
Congenital Disease	SNOMED	204762005	Transposition of cecum (disorder)
Congenital Disease	SNOMED	204766008	Enterogenous cyst (disorder)
Congenital Disease	SNOMED	204769001	Congenital fecal fistula (disorder)
Congenital Disease	SNOMED	204787003	Congenital absence of liver and/or gallbladder (disorder)
Congenital Disease	SNOMED	204788008	Congenital atrophy of left lobe of liver (disorder)
Congenital Disease	SNOMED	204795004	Congenital floating liver (disorder)
Congenital Disease	SNOMED	204798002	Congenital diverticulum of bile duct (disorder)
Congenital Disease	SNOMED	204799005	Liver hyperplasia (disorder)
Congenital Disease	SNOMED	204805004	Anomalies of pancreas (disorder)
Congenital Disease	SNOMED	204806003	Agenesis of pancreas (disorder)
Congenital Disease	SNOMED	204808002	Congenital cyst of pancreas (disorder)
Congenital Disease	SNOMED	204826004	Fallopian tube and broad ligament anomalies (disorder)
Congenital Disease	SNOMED	204844007	Agenesis of uterus (disorder)
Congenital Disease	SNOMED	204847000	Congenital fistulae between uterus and digestive and urinary tracts (disorder)
Congenital Disease	SNOMED	204848005	Congenital uterointestinal fistula (disorder)
Congenital Disease	SNOMED	204849002	Congenital uterovesical fistula (disorder)
Congenital Disease	SNOMED	204867009	Agenesis of vulva (disorder)
Congenital Disease	SNOMED	204878001	Undescended testicle (disorder)
Congenital Disease	SNOMED	204888000	Penile hypospadias (disorder)
Congenital Disease	SNOMED	204889008	Penoscrotal hypospadias (disorder)
Congenital Disease	SNOMED	204890004	Perineal hypospadias (disorder)
Congenital Disease	SNOMED	204905003	Congenital aplasia of round ligament (disorder)
Congenital Disease	SNOMED	204907006	Congenital aplasia of scrotum (disorder)
Congenital Disease	SNOMED	204908001	Aplasia of penis (disorder)
Congenital Disease	SNOMED	20491006	Calcaneonavicular bar (disorder)
Congenital Disease	SNOMED	204912007	Hypoplasia of scrotum (disorder)
Congenital Disease	SNOMED	204920009	Cyst of hydatid of Morgagni (disorder)
Congenital Disease	SNOMED	204922001	Hydatid cyst of Morgagni - male (disorder)
Congenital Disease	SNOMED	204923006	Hydatid cyst of Morgagni - female (disorder)
Congenital Disease	SNOMED	204924000	Wolffian duct cyst - male (disorder)
Congenital Disease	SNOMED	204925004	Wolffian duct cyst - female (disorder)
Congenital Disease	SNOMED	204933003	Hooded penis (disorder)
Congenital Disease	SNOMED	204938007	Renal agenesis and dysgenesis (disorder)
Congenital Disease	SNOMED	204941003	Congenital renal atrophy (disorder)
Congenital Disease	SNOMED	204942005	Renal agenesis (disorder)
Congenital Disease	SNOMED	204949001	Renal dysplasia (disorder)
Congenital Disease	SNOMED	204950001	Bilateral renal dysplasia (disorder)
Congenital Disease	SNOMED	204957003	Medullary cystic disease (disorder)
Congenital Disease	SNOMED	204958008	Nephronophthisis (disorder)
Congenital Disease	SNOMED	204967008	Renal pelvis and ureter obstructive defects (disorder)
Congenital Disease	SNOMED	204973009	Simple ureterocele (disorder)
Congenital Disease	SNOMED	204974003	Impervious ureter (disorder)
Congenital Disease	SNOMED	204984002	Fusion of kidneys (disorder)
Congenital Disease	SNOMED	204985001	Hyperplasia of kidney (disorder)
Congenital Disease	SNOMED	204991004	Deviation of ureter (disorder)
Congenital Disease	SNOMED	204998005	Urethra and bladder neck atresia and stenosis (disorder)

Congenital Disease	SNOMED	204999002	Atresia of bladder neck (disorder)
Congenital Disease	SNOMED	205003000	Atresia of anterior urethra (disorder)
Congenital Disease	SNOMED	205016000	Malformation of urachus (disorder)
Congenital Disease	SNOMED	205022009	Congenital bladder hernia (disorder)
Congenital Disease	SNOMED	205023004	Congenital prolapse of bladder mucosa (disorder)
Congenital Disease	SNOMED	205024005	Congenital hourglass bladder (disorder)
Congenital Disease	SNOMED	205026007	Female epispadias (disorder)
Congenital Disease	SNOMED	205027003	Female hypospadias (disorder)
Congenital Disease	SNOMED	205043005	Congenital deformity of spine (disorder)
Congenital Disease	SNOMED	205045003	Congenital scoliosis due to bony malformation (disorder)
Congenital Disease	SNOMED	205052001	Congenital subluxation of hip, bilateral (disorder)
Congenital Disease	SNOMED	205057007	Bilateral dysplastic hip (disorder)
Congenital Disease	SNOMED	205063003	Congenital genu recurvatum (disorder)
Congenital Disease	SNOMED	205064009	Congenital dislocation of knee grade I (disorder)
Congenital Disease	SNOMED	205065005	Congenital dislocation of knee grade II (disorder)
Congenital Disease	SNOMED	205066006	Congenital dislocation of knee grade III (disorder)
Congenital Disease	SNOMED	205067002	Congenital dislocation of patella (disorder)
Congenital Disease	SNOMED	205068007	Congenital bowing of fibula (disorder)
Congenital Disease	SNOMED	205073001	Congenital complex varus foot deformity (disorder)
Congenital Disease	SNOMED	205082007	Congenital vertical talus (disorder)
Congenital Disease	SNOMED	205083002	Congenital talipes calcaneovalgus (disorder)
Congenital Disease	SNOMED	205091006	Congenital pes cavus (disorder)
Congenital Disease	SNOMED	205092004	Congenital claw foot (disorder)
Congenital Disease	SNOMED	205093009	Congenital short Achilles tendon (disorder)
Congenital Disease	SNOMED	205097005	Congenital talipes equinus (disorder)
Congenital Disease	SNOMED	205101001	Congenital pectus carinatum (disorder)
Congenital Disease	SNOMED	205108007	Congenital flexion contracture of hip (disorder)
Congenital Disease	SNOMED	205109004	Congenital abduction contracture of hip (disorder)
Congenital Disease	SNOMED	205110009	Congenital flexion contracture of knee (disorder)
Congenital Disease	SNOMED	205111008	Congenital short quadriceps (disorder)
Congenital Disease	SNOMED	205122007	Radial polydactyly Wassel 1 (disorder)
Congenital Disease	SNOMED	205123002	Radial polydactyly Wassel 2 (disorder)
Congenital Disease	SNOMED	205124008	Radial polydactyly Wassel 3 (disorder)
Congenital Disease	SNOMED	205125009	Radial polydactyly Wassel 4 (disorder)
Congenital Disease	SNOMED	205126005	Radial polydactyly Wassel 5 (disorder)
Congenital Disease	SNOMED	205127001	Radial polydactyly Wassel 6 (disorder)
Congenital Disease	SNOMED	205129003	Radial polydactyly Wassel 7 (disorder)
Congenital Disease	SNOMED	205130008	Central polydactyly of fingers (disorder)
Congenital Disease	SNOMED	205131007	Ulnar polydactyly of fingers (disorder)
Congenital Disease	SNOMED	205132000	Preaxial polydactyly of toe (disorder)
Congenital Disease	SNOMED	205133005	Postaxial polydactyly of toe (disorder)
Congenital Disease	SNOMED	205135003	Radial polydactyly (disorder)
Congenital Disease	SNOMED	205139009	Simple syndactyly of fingers - first web (disorder)
Congenital Disease	SNOMED	205140006	Simple syndactyly of fingers - second to fourth web (disorder)
Congenital Disease	SNOMED	205142003	Osseous syndactyly of fingers - first web (disorder)
Congenital Disease	SNOMED	205143008	Osseous syndactyly of fingers - second to fourth web (disorder)
Congenital Disease	SNOMED	205144002	Simple syndactyly of toes (disorder)
Congenital Disease	SNOMED	205145001	Simple syndactyly of toes, first web space (disorder)
Congenital Disease	SNOMED	205146000	Simple syndactyly lesser toes (disorder)
Congenital Disease	SNOMED	205148004	Osseous syndactyly of toes first web space (disorder)
Congenital Disease	SNOMED	205149007	Osseous syndactyly lesser toes (disorder)
Congenital Disease	SNOMED	205161004	Congenital amputation of upper limb (disorder)
Congenital Disease	SNOMED	205163001	Transverse deficiency of arm, upper arm level - short (disorder)
Congenital Disease	SNOMED	205164007	Transverse deficiency of arm, upper arm level - long (disorder)
Congenital Disease	SNOMED	205170001	Hypoplasia of radius (disorder)
Congenital Disease	SNOMED	205171002	Partial radial absence (disorder)
Congenital Disease	SNOMED	205174005	Hypoplastic thumb-Blauth 1 (disorder)
Congenital Disease	SNOMED	205175006	Hypoplastic thumb-Blauth 2 (disorder)
Congenital Disease	SNOMED	205176007	Hypoplastic thumb-Blauth 3 (disorder)
Congenital Disease	SNOMED	205177003	Hypoplastic thumb-Blauth 4 (disorder)
Congenital Disease	SNOMED	205178008	Hypoplastic thumb-Blauth 5 (disorder)
Congenital Disease	SNOMED	205179000	Agenesis of ulna (disorder)
Congenital Disease	SNOMED	205180002	Partial defect of ulna (disorder)
Congenital Disease	SNOMED	205183000	Transverse arrest carpal level (disorder)
Congenital Disease	SNOMED	205184006	Transverse arrest metacarpal first ray (disorder)
Congenital Disease	SNOMED	205187004	Transverse arrest phalangeal level first ray (disorder)
Congenital Disease	SNOMED	205188009	Transverse arrest phalangeal level second ray (disorder)
Congenital Disease	SNOMED	205189001	Transverse arrest phalangeal level third ray (disorder)
Congenital Disease	SNOMED	205190005	Transverse arrest phalangeal level fourth ray (disorder)
Congenital Disease	SNOMED	205191009	Transverse arrest phalangeal level fifth ray (disorder)
Congenital Disease	SNOMED	205203006	Transverse deficiency lower limb - hip level (disorder)
Congenital Disease	SNOMED	205204000	Transverse deficiency lower limb - metatarsal level (disorder)
Congenital Disease	SNOMED	205206003	Transverse deficiency lower limb - through femur (disorder)
Congenital Disease	SNOMED	205207007	Transverse deficiency lower limb - through tibia/fibula (disorder)
Congenital Disease	SNOMED	205211001	Proximal femoral focal deficiency (disorder)

Congenital Disease	SNOMED	205212008	Congenital tibial deficiency type I (disorder)
Congenital Disease	SNOMED	205213003	Congenital tibial deficiency type II (disorder)
Congenital Disease	SNOMED	205215005	Congenital tibial deficiency type III (disorder)
Congenital Disease	SNOMED	205216006	Congenital fibular deficiency type I (disorder)
Congenital Disease	SNOMED	205217002	Congenital fibular deficiency type II (disorder)
Congenital Disease	SNOMED	205218007	Congenital fibular deficiency type III (disorder)
Congenital Disease	SNOMED	205221009	Agenesis of multiple tarsal bones (disorder)
Congenital Disease	SNOMED	205222002	Agenesis of first metatarsal (disorder)
Congenital Disease	SNOMED	205223007	Agenesis of fifth metatarsal (disorder)
Congenital Disease	SNOMED	205225000	Agenesis of fourth and fifth metatarsals (disorder)
Congenital Disease	SNOMED	205227008	Congenital absence of great toe (disorder)
Congenital Disease	SNOMED	205228003	Congenital absence of fifth toe (disorder)
Congenital Disease	SNOMED	205230001	Congenital absence of fourth and fifth toes (disorder)
Congenital Disease	SNOMED	205253000	Proximal radioulnar synostosis (disorder)
Congenital Disease	SNOMED	205254006	Radioulnar synostosis and dislocation of radial head (disorder)
Congenital Disease	SNOMED	205255007	Distal radioulnar synostosis (disorder)
Congenital Disease	SNOMED	205258009	Acrocephalosyndactyly type I (disorder)
Congenital Disease	SNOMED	205260006	Acrocephalopolysyndactyly (disorder)
Congenital Disease	SNOMED	205261005	Macrodactyly of fingers - simple (disorder)
Congenital Disease	SNOMED	205262003	Macrodactyly of fingers- fatty nerve tumor (disorder)
Congenital Disease	SNOMED	205264002	Cleft hand - first cleft (disorder)
Congenital Disease	SNOMED	205265001	Cleft hand - central (disorder)
Congenital Disease	SNOMED	205266000	Cleft hand with syndactyly (disorder)
Congenital Disease	SNOMED	205267009	Cleft hand with polydactyly (disorder)
Congenital Disease	SNOMED	205269007	Windblown hand (disorder)
Congenital Disease	SNOMED	205270008	Aberrant forearm flexor muscle (disorder)
Congenital Disease	SNOMED	205271007	Aberrant forearm extensor muscle (disorder)
Congenital Disease	SNOMED	205273005	Thumb in palm deformity (disorder)
Congenital Disease	SNOMED	205274004	Congenital trigger thumb (disorder)
Congenital Disease	SNOMED	205276002	Lunate-triquetrum synostosis (disorder)
Congenital Disease	SNOMED	205277006	Capitate-hamate synostosis (disorder)
Congenital Disease	SNOMED	205278001	Scaphoid-lunate synostosis (disorder)
Congenital Disease	SNOMED	205280007	Proximal interphalangeal joint symphalangism (disorder)
Congenital Disease	SNOMED	205281006	Distal interphalangeal joint symphalangism (disorder)
Congenital Disease	SNOMED	205284003	Duplication of whole limb (disorder)
Congenital Disease	SNOMED	205285002	Duplication of humerus (disorder)
Congenital Disease	SNOMED	205286001	Duplication of radius (disorder)
Congenital Disease	SNOMED	205288000	Duplication of whole hand (disorder)
Congenital Disease	SNOMED	205291000	Overgrowth of whole upper limb (disorder)
Congenital Disease	SNOMED	205292007	Overgrowth of partial upper limb (disorder)
Congenital Disease	SNOMED	205297001	Undergrowth of whole hand (disorder)
Congenital Disease	SNOMED	205299003	Constriction ring syndrome of upper limb (disorder)
Congenital Disease	SNOMED	205304002	Constriction ring of upper limb with acrosyndactyly and amputation (disorder)
Congenital Disease	SNOMED	205306000	Congenital complete absence of upper limb (disorder)
Congenital Disease	SNOMED	205308004	Triphalangeal thumb (disorder)
Congenital Disease	SNOMED	205311003	Brachydactyly-all 3 phalanges (disorder)
Congenital Disease	SNOMED	205312005	Brachydactyly-missing phalanx (disorder)
Congenital Disease	SNOMED	205313000	Camptodactyly-little finger (disorder)
Congenital Disease	SNOMED	205315007	Clinodactyly with delta phalanx (disorder)
Congenital Disease	SNOMED	205316008	Clinodactyly, with no delta phalanx (disorder)
Congenital Disease	SNOMED	205317004	Brachymesophalangia (disorder)
Congenital Disease	SNOMED	205322004	Radioulnar dysostosis (disorder)
Congenital Disease	SNOMED	205328000	Congenital humeral varus (disorder)
Congenital Disease	SNOMED	205329008	Humeroradial synostosis (disorder)
Congenital Disease	SNOMED	205330003	Humeroulnar synostosis (disorder)
Congenital Disease	SNOMED	205342008	Macrodactyly of toes - simple (disorder)
Congenital Disease	SNOMED	205343003	Macrodactyly of toes - fatty nerve tumor (disorder)
Congenital Disease	SNOMED	205346006	Brachydactyly of toes (disorder)
Congenital Disease	SNOMED	205347002	Congenital crossed toes (disorder)
Congenital Disease	SNOMED	205348007	Congenital curly toes (disorder)
Congenital Disease	SNOMED	205349004	Brachyphalangia of little toe (disorder)
Congenital Disease	SNOMED	205351000	Perodactylia of great toe (disorder)
Congenital Disease	SNOMED	205352007	Perodactylia of lesser toe (disorder)
Congenital Disease	SNOMED	205353002	Perodactylia of multiple toes (disorder)
Congenital Disease	SNOMED	205354008	Triphalangeal great toe (disorder)
Congenital Disease	SNOMED	205358006	Split foot (disorder)
Congenital Disease	SNOMED	205361007	Naviculocuneiform bar (disorder)
Congenital Disease	SNOMED	205362000	Complex tarsal coalition (disorder)
Congenital Disease	SNOMED	205364004	Failure of soft tissue differentiation of lower limb (disorder)
Congenital Disease	SNOMED	205365003	Aberrant muscle of the lower limb (disorder)
Congenital Disease	SNOMED	205366002	Failure of differentiation of bones of lower limb (disorder)
Congenital Disease	SNOMED	205367006	Congenital synostosis of lower limb bones (disorder)
Congenital Disease	SNOMED	205368001	Duplication of lower limb bone (disorder)
Congenital Disease	SNOMED	205369009	Congenital overgrowth of lower limb (disorder)

Congenital Disease	SNOMED	205370005	Congenital overgrowth of proximal lower limb (disorder)
Congenital Disease	SNOMED	205371009	Congenital overgrowth of distal lower limb (disorder)
Congenital Disease	SNOMED	205372002	Congenital overgrowth of foot (disorder)
Congenital Disease	SNOMED	205373007	Congenital overgrowth of whole lower limb (disorder)
Congenital Disease	SNOMED	205382001	Constriction ring syndrome of lower limb (disorder)
Congenital Disease	SNOMED	205385004	Constriction ring of lower limb with lymphedema (disorder)
Congenital Disease	SNOMED	205386003	Intrauterine amputation of lower limb (disorder)
Congenital Disease	SNOMED	205387007	Constriction ring syndrome of lower limb with amputation (disorder)
Congenital Disease	SNOMED	205395006	Congenital angulation of tibia (disorder)
Congenital Disease	SNOMED	205397003	Congenital varus ankle (disorder)
Congenital Disease	SNOMED	205398008	Congenital valgus ankle (disorder)
Congenital Disease	SNOMED	205399000	Congenital ball and socket ankle (disorder)
Congenital Disease	SNOMED	205418005	Goldenhar syndrome (disorder)
Congenital Disease	SNOMED	205425003	Sacral agenesis (disorder)
Congenital Disease	SNOMED	205427006	Cervical hemivertebra- balanced (disorder)
Congenital Disease	SNOMED	205428001	Cervical hemivertebra - unbalanced (disorder)
Congenital Disease	SNOMED	205429009	Thoracic hemivertebra- balanced (disorder)
Congenital Disease	SNOMED	205430004	Thoracic hemivertebra - unbalanced (disorder)
Congenital Disease	SNOMED	205431000	Lumbar hemivertebra - balanced (disorder)
Congenital Disease	SNOMED	205432007	Lumbar hemivertebra - unbalanced (disorder)
Congenital Disease	SNOMED	205435009	Congenital complete fusion of spine (disorder)
Congenital Disease	SNOMED	205436005	Congenital partial fusion of spine - balanced (disorder)
Congenital Disease	SNOMED	205437001	Congenital partial fusion of spine - unbalanced (disorder)
Congenital Disease	SNOMED	205438006	Congenital partial fusion of spine with hemivertebra - balanced (disorder)
Congenital Disease	SNOMED	205439003	Congenital partial fusion of spine with hemivertebra - unbalanced (disorder)
Congenital Disease	SNOMED	205455005	Defect of vertebral segmentation (disorder)
Congenital Disease	SNOMED	205456006	Hypoplasia of spine (disorder)
Congenital Disease	SNOMED	205460009	Accessory rib (disorder)
Congenital Disease	SNOMED	205461008	Mis-shapen sternum (disorder)
Congenital Disease	SNOMED	205465004	Chondrodysplasia (disorder)
Congenital Disease	SNOMED	205468002	Hypochondroplasia (disorder)
Congenital Disease	SNOMED	205473008	Mesomelic dysplasia (disorder)
Congenital Disease	SNOMED	205480005	Dysplasia epiphysealis hemimelica (disorder)
Congenital Disease	SNOMED	205481009	Metachondromatosis (disorder)
Congenital Disease	SNOMED	205482002	Lethal retarded ossification syndromes (disorder)
Congenital Disease	SNOMED	205483007	Hypochondrogenesis (disorder)
Congenital Disease	SNOMED	205484001	Short rib polydactyly syndrome (disorder)
Congenital Disease	SNOMED	205496008	Osteogenesis imperfecta type II (disorder)
Congenital Disease	SNOMED	205497004	Osteogenesis imperfecta type IV (disorder)
Congenital Disease	SNOMED	205503007	Osteopetrosis - delayed type (disorder)
Congenital Disease	SNOMED	205506004	Craniodiaphyseal dysplasia (disorder)
Congenital Disease	SNOMED	205530002	Hypoplasia of muscle (disorder)
Congenital Disease	SNOMED	205532005	Aplasia of muscle (disorder)
Congenital Disease	SNOMED	205545009	Congenital elephantiasis (disorder)
Congenital Disease	SNOMED	205548006	Harlequin ichthyosis (disorder)
Congenital Disease	SNOMED	205550003	Lamellar ichthyosis (disorder)
Congenital Disease	SNOMED	205557000	Abnormal palmar creases (disorder)
Congenital Disease	SNOMED	205562004	Angiomatosis (disorder)
Congenital Disease	SNOMED	205564003	Congenital pigmentary skin anomalies (disorder)
Congenital Disease	SNOMED	205570009	Brugsch's syndrome (disorder)
Congenital Disease	SNOMED	205573006	Focal dermal hypoplasia (disorder)
Congenital Disease	SNOMED	205583005	Hereditary benign acanthosis nigricans (disorder)
Congenital Disease	SNOMED	205592008	Congenital localized alopecia (disorder)
Congenital Disease	SNOMED	205597002	Taenzer's hair (disorder)
Congenital Disease	SNOMED	205615000	Trisomy 21- meiotic nondisjunction (disorder)
Congenital Disease	SNOMED	205616004	Trisomy 21- mitotic nondisjunction mosaicism (disorder)
Congenital Disease	SNOMED	205619006	Trisomy 13, meiotic nondisjunction (disorder)
Congenital Disease	SNOMED	205620000	Trisomy 13 - mitotic nondisjunction mosaicism (disorder)
Congenital Disease	SNOMED	205623003	Trisomy 18 - meiotic nondisjunction (disorder)
Congenital Disease	SNOMED	205624009	Trisomy 18 - mitotic nondisjunction mosaicism (disorder)
Congenital Disease	SNOMED	205627002	Monosomy and deletion from autosome (disorder)
Congenital Disease	SNOMED	205630009	Deletion of long arm of chromosome 13 (disorder)
Congenital Disease	SNOMED	205634000	Deletion seen only at prometaphase (disorder)
Congenital Disease	SNOMED	205636003	Whole chromosome monosomy - meiotic nondisjunction (disorder)
Congenital Disease	SNOMED	205638002	Monosomy 21, mosaicism (disorder)
Congenital Disease	SNOMED	205644003	Balanced autosomal translocation (disorder)
Congenital Disease	SNOMED	205646001	Whole chromosome trisomy syndrome (disorder)
Congenital Disease	SNOMED	205647005	Trisomy 6 (disorder)
Congenital Disease	SNOMED	205648000	Trisomy 7 (disorder)
Congenital Disease	SNOMED	205649008	Trisomy 8 (disorder)
Congenital Disease	SNOMED	205650008	Trisomy 9 (disorder)
Congenital Disease	SNOMED	205651007	Trisomy 10 (disorder)
Congenital Disease	SNOMED	205652000	Trisomy 11 (disorder)
Congenital Disease	SNOMED	205653005	Trisomy 12 (disorder)
Congenital Disease	SNOMED	205655003	Trisomy 22 (disorder)

Congenital Disease	SNOMED	205657006	Whole chromosome trisomy, mosaicism (disorder)
Congenital Disease	SNOMED	205660004	Partial trisomy syndromes (disorder)
Congenital Disease	SNOMED	205661000	Major partial trisomy (disorder)
Congenital Disease	SNOMED	205662007	Minor partial trisomy (disorder)
Congenital Disease	SNOMED	205665009	Duplication seen only at prometaphase (disorder)
Congenital Disease	SNOMED	205666005	Duplication with other complex rearrangement (disorder)
Congenital Disease	SNOMED	205672005	Chromosome inversion in normal individual (disorder)
Congenital Disease	SNOMED	205673000	Balanced autosomal rearrangement in abnormal individual (disorder)
Congenital Disease	SNOMED	205674006	Balanced sex/autosomal rearrangement in abnormal individual (disorder)
Congenital Disease	SNOMED	205675007	Individual with marker heterochromatin (disorder)
Congenital Disease	SNOMED	205676008	Individual with autosomal fragile site (disorder)
Congenital Disease	SNOMED	205681004	Gonadal dysgenesis (disorder)
Congenital Disease	SNOMED	205683001	Ovarian dysgenesis (disorder)
Congenital Disease	SNOMED	205684007	Turner's phenotype, karyotype normal (disorder)
Congenital Disease	SNOMED	205686009	Karyotype 46, X iso (Xq) (disorder)
Congenital Disease	SNOMED	205687000	Karyotype 46, X with abnormal sex chromosome except iso (Xq) (disorder)
Congenital Disease	SNOMED	205693008	XY, female phenotype (disorder)
Congenital Disease	SNOMED	205698004	Klinefelter syndrome, male with 46,XX karyotype (disorder)
Congenital Disease	SNOMED	205699007	Klinefelter's syndrome, XXYY (disorder)
Congenital Disease	SNOMED	205700008	Klinefelter's syndrome, XY/XXY mosaic (disorder)
Congenital Disease	SNOMED	205706002	Sex chromosome mosaicism (disorder)
Congenital Disease	SNOMED	205707006	Mosaic XO/XY (disorder)
Congenital Disease	SNOMED	205708001	Mosaic XO/XX (disorder)
Congenital Disease	SNOMED	205709009	Mosaic XY/XXY (disorder)
Congenital Disease	SNOMED	205710004	Mosaic including XXXXY (disorder)
Congenital Disease	SNOMED	205718006	Chimera 46, XX; 46, XY (disorder)
Congenital Disease	SNOMED	205720009	Fragile X chromosome (disorder)
Congenital Disease	SNOMED	205728002	Duplication of chromosome (disorder)
Congenital Disease	SNOMED	205735005	Hypoplasia of spleen (disorder)
Congenital Disease	SNOMED	205736006	Mis-shapen spleen (disorder)
Congenital Disease	SNOMED	205744006	Congenital cyst of adrenal gland (disorder)
Congenital Disease	SNOMED	205749001	Congenital absence of pituitary gland (disorder)
Congenital Disease	SNOMED	205750001	Accessory pituitary gland (disorder)
Congenital Disease	SNOMED	205757003	Aberrant parathyroid gland (disorder)
Congenital Disease	SNOMED	205768003	Complete situs inversus with dextrocardia (disorder)
Congenital Disease	SNOMED	205769006	Situs inversus with levocardia (disorder)
Congenital Disease	SNOMED	205771006	Craniopagus frontalis (disorder)
Congenital Disease	SNOMED	205774003	Craniopagus parasiticus (disorder)
Congenital Disease	SNOMED	205798005	Cyclopia (disorder)
Congenital Disease	SNOMED	205800003	Gorlin-Chaudhry-Moss syndrome (disorder)
Congenital Disease	SNOMED	205802006	Oculo-palato-digital syndrome (disorder)
Congenital Disease	SNOMED	205808005	Congenital malformation syndromes associated with short stature (disorder)
Congenital Disease	SNOMED	205817005	Aglossia-adactyly syndrome (disorder)
Congenital Disease	SNOMED	205819008	Multiple pterygium syndrome (disorder)
Congenital Disease	SNOMED	205821003	Congenital contractural arachnodactyly (disorder)
Congenital Disease	SNOMED	205824006	Noonan's syndrome (disorder)
Congenital Disease	SNOMED	205828009	Biemond's syndrome (disorder)
Congenital Disease	SNOMED	205834002	Acardia (disorder)
Congenital Disease	SNOMED	205835001	Acephalobrachius (disorder)
Congenital Disease	SNOMED	205836000	Acephalogaster (disorder)
Congenital Disease	SNOMED	205838004	Congenital hemihypertrophy (disorder)
Congenital Disease	SNOMED	206469004	Perinatal jaundice due to congenital obstruction of bile duct (disorder)
Congenital Disease	SNOMED	20766005	Ehlers-Danlos syndrome, type 2 (disorder)
Congenital Disease	SNOMED	20815007	Exstrophy of cloaca sequence (disorder)
Congenital Disease	SNOMED	20834007	Congenital cubitus varus (disorder)
Congenital Disease	SNOMED	2091005	Pharyngeal diverticulitis (disorder)
Congenital Disease	SNOMED	20915006	Gastric dysplasia (disorder)
Congenital Disease	SNOMED	20919000	Congenital liver grooves (disorder)
Congenital Disease	SNOMED	20944008	Congenital postural scoliosis (disorder)
Congenital Disease	SNOMED	20948006	Congenital anomaly of finger (disorder)
Congenital Disease	SNOMED	21049007	Trichostasis spinulosa (disorder)
Congenital Disease	SNOMED	2107001	Anisomelia (disorder)
Congenital Disease	SNOMED	21086008	Cockayne syndrome (disorder)
Congenital Disease	SNOMED	2111000119106	Congenital glenoid dysplasia (disorder)
Congenital Disease	SNOMED	21111006	Complete trisomy 13 syndrome (disorder)
Congenital Disease	SNOMED	21234008	Congenital stenosis of aorta (disorder)
Congenital Disease	SNOMED	21279007	Lingual thyroid (disorder)
Congenital Disease	SNOMED	21321009	Ambiguous genitalia (disorder)
Congenital Disease	SNOMED	21350002	Colloid cyst of third ventricle (disorder)
Congenital Disease	SNOMED	21379009	Ruptured sinus of Valsalva (disorder)
Congenital Disease	SNOMED	21530000	Cephalothoracopagus (disorder)
Congenital Disease	SNOMED	21565000	Accessory broad ligament (disorder)
Congenital Disease	SNOMED	21590003	Congenital zonular cataract (disorder)
Congenital Disease	SNOMED	21634003	Borjeson-Forssman-Lehmann syndrome (disorder)
Congenital Disease	SNOMED	21779006	Retractile testis (disorder)

Congenital Disease	SNOMED	218358001	Incontinentia pigmenti achromians syndrome (disorder)
Congenital Disease	SNOMED	21850008	Plagiocephaly (disorder)
Congenital Disease	SNOMED	218728005	Interrupted aortic arch (disorder)
Congenital Disease	SNOMED	21893008	Manus valga (disorder)
Congenital Disease	SNOMED	21905007	Congenital anomaly of middle ear (disorder)
Congenital Disease	SNOMED	21926007	Pili annulati (disorder)
Congenital Disease	SNOMED	21981000	Single coronary artery (disorder)
Congenital Disease	SNOMED	21998000	Robert's pelvis (disorder)
Congenital Disease	SNOMED	22006008	Hypertelorism (disorder)
Congenital Disease	SNOMED	22053006	Klinefelter syndrome (disorder)
Congenital Disease	SNOMED	22099008	Congenital valgus deformity of foot (disorder)
Congenital Disease	SNOMED	2213002	Congenital anomaly of vena cava (disorder)
Congenital Disease	SNOMED	22133005	Congenital anomaly of the peripheral nervous system (disorder)
Congenital Disease	SNOMED	22138001	Congenital absence of ear lobe (disorder)
Congenital Disease	SNOMED	22199006	Nail-patella syndrome (disorder)
Congenital Disease	SNOMED	2229005	Chimera (disorder)
Congenital Disease	SNOMED	22301000119102	Congenital positional plagiocephaly (disorder)
Congenital Disease	SNOMED	22395006	Esophageal body web (disorder)
Congenital Disease	SNOMED	2241003	X-linked absence of thyroxine-binding globulin (disorder)
Congenital Disease	SNOMED	22421007	Congenital absence of bladder (disorder)
Congenital Disease	SNOMED	22471005	Hemispheric cerebellar agenesis (disorder)
Congenital Disease	SNOMED	22497004	9q partial trisomy syndrome (disorder)
Congenital Disease	SNOMED	22504001	Uterus bilocularis (disorder)
Congenital Disease	SNOMED	22567005	Pseudoachondroplastic spondyloepiphyseal dysplasia syndrome (disorder)
Congenital Disease	SNOMED	22589009	Congenital absence of salivary gland (disorder)
Congenital Disease	SNOMED	227035005	21q partial distal trisomy (disorder)
Congenital Disease	SNOMED	22750001	Anomalous pulmonary venous drainage to abdominal portion of inferior vena cava (disorder)
Congenital Disease	SNOMED	22764001	Metatropic dysplasia (disorder)
Congenital Disease	SNOMED	228050004	21q partial trisomy (disorder)
Congenital Disease	SNOMED	22841008	Phocomelia (disorder)
Congenital Disease	SNOMED	22845004	Congenital septation of gallbladder (disorder)
Congenital Disease	SNOMED	22868008	Longitudinal deficiency of phalanges of hand (disorder)
Congenital Disease	SNOMED	229845003	Congenital forefoot varus (disorder)
Congenital Disease	SNOMED	229846002	Congenital rearfoot varus (disorder)
Congenital Disease	SNOMED	229847006	Plantarflexed cuboid (disorder)
Congenital Disease	SNOMED	23024003	Macrogyria (disorder)
Congenital Disease	SNOMED	230263009	Autosomal dominant spastic paraplegia type 17 (disorder)
Congenital Disease	SNOMED	230306001	Benign hereditary chorea (disorder)
Congenital Disease	SNOMED	230382002	Benign frontal epilepsy of childhood (disorder)
Congenital Disease	SNOMED	230384001	Benign atypical partial epilepsy in childhood (disorder)
Congenital Disease	SNOMED	230504002	Tilted optic disc (disorder)
Congenital Disease	SNOMED	230506000	Myelinated nerve fibers of optic disc (disorder)
Congenital Disease	SNOMED	230529008	Congenital failure of eye elevation (disorder)
Congenital Disease	SNOMED	230530003	Congenital nuclear ophthalmoplegia (disorder)
Congenital Disease	SNOMED	230541001	Congenital disorder of facial nerve (disorder)
Congenital Disease	SNOMED	230561007	Congenital neuropathy with arthrogryposis multiplex congenita (disorder)
Congenital Disease	SNOMED	23061007	Congenital anomaly of cricoid cartilage (disorder)
Congenital Disease	SNOMED	23063005	Congenital atresia of mitral valve (disorder)
Congenital Disease	SNOMED	230779009	Congenital spastic foot (disorder)
Congenital Disease	SNOMED	230785002	Congenital dysarthria (disorder)
Congenital Disease	SNOMED	230786001	Congenital dysphasia (disorder)
Congenital Disease	SNOMED	230787005	Congenital expressive dysphasia (disorder)
Congenital Disease	SNOMED	230788000	Congenital receptive dysphasia (disorder)
Congenital Disease	SNOMED	230791000	Hypothalamic neuronal hamartoma (disorder)
Congenital Disease	SNOMED	230794008	Neuronal choristoma (disorder)
Congenital Disease	SNOMED	231250005	Congenital talipes calcaneus (disorder)
Congenital Disease	SNOMED	23150001	Proteus syndrome (disorder)
Congenital Disease	SNOMED	232059000	Laurence-Moon syndrome (disorder)
Congenital Disease	SNOMED	232074003	Congenital hypertrophy of retinal pigment epithelium (disorder)
Congenital Disease	SNOMED	232083008	Glaucoma and corneal anomaly (disorder)
Congenital Disease	SNOMED	232217008	Congenital deformity of pinna (disorder)
Congenital Disease	SNOMED	232298004	Structural anomaly of the cochlea and vestibular labyrinth (disorder)
Congenital Disease	SNOMED	232299007	Aplasia of the cochlea and vestibular labyrinth (disorder)
Congenital Disease	SNOMED	232300004	Congenital deformity of labyrinth (disorder)
Congenital Disease	SNOMED	232301000	Incomplete formation of the bony cochlea (disorder)
Congenital Disease	SNOMED	232302007	Mondini defect (disorder)
Congenital Disease	SNOMED	232335002	Earpit syndrome (disorder)
Congenital Disease	SNOMED	232373003	Choanal atresia with radial ray hypoplasia (disorder)
Congenital Disease	SNOMED	232381002	Agenesis of nasal cartilages (disorder)
Congenital Disease	SNOMED	232412004	Bifid epiglottis (disorder)
Congenital Disease	SNOMED	232461002	Congenital cleft larynx (disorder)
Congenital Disease	SNOMED	23359005	Multiple malformation syndrome with facial-limb defects as major feature (disorder)
Congenital Disease	SNOMED	233627004	Congenital cystic bronchiectasis (disorder)

Congenital Disease	SNOMED	233663004	Primary ciliary dyskinesia due to transposition of ciliary microtubules (disorder)
Congenital Disease	SNOMED	233664005	Immotile cilia syndrome due to defective radial spokes (disorder)
Congenital Disease	SNOMED	233665006	Immotile cilia syndrome due to excessively long cilia (disorder)
Congenital Disease	SNOMED	233666007	Young's syndrome (disorder)
Congenital Disease	SNOMED	233667003	Rutland ciliary disorientation syndrome (disorder)
Congenital Disease	SNOMED	233717003	Diffuse pulmonary neurofibromatosis (disorder)
Congenital Disease	SNOMED	233718008	Pulmonary tuberous sclerosis (disorder)
Congenital Disease	SNOMED	233778002	Tracheal fistula (disorder)
Congenital Disease	SNOMED	233779005	Congenital tracheal fistula (disorder)
Congenital Disease	SNOMED	233862006	Calcific aortic stenosis - bicuspid valve (disorder)
Congenital Disease	SNOMED	233965009	Stenosis of thoracic aorta (disorder)
Congenital Disease	SNOMED	233966005	Stenosis of abdominal aorta (disorder)
Congenital Disease	SNOMED	233990006	Ductus arteriosus aneurysm (disorder)
Congenital Disease	SNOMED	23402009	Congenital stricture of ureterovesical orifice (disorder)
Congenital Disease	SNOMED	234035006	Marfan's syndrome affecting skin (disorder)
Congenital Disease	SNOMED	23407003	Congenital pes planus (disorder)
Congenital Disease	SNOMED	234095009	Lymphatic malformation (disorder)
Congenital Disease	SNOMED	234118009	Capillary malformation (disorder)
Congenital Disease	SNOMED	234119001	Arterial malformation (disorder)
Congenital Disease	SNOMED	234121006	Hypoplasia of artery (disorder)
Congenital Disease	SNOMED	234122004	Persistence of primitive artery (disorder)
Congenital Disease	SNOMED	234123009	Persistent cerebral embryonic artery (disorder)
Congenital Disease	SNOMED	234124003	Venous malformation (disorder)
Congenital Disease	SNOMED	234125002	Venous valvular anomaly (disorder)
Congenital Disease	SNOMED	234126001	Deep vein aplasia (disorder)
Congenital Disease	SNOMED	234127005	Deep vein hypoplasia (disorder)
Congenital Disease	SNOMED	234129008	True congenital varicose veins (disorder)
Congenital Disease	SNOMED	234130003	Spongy venous malformation (disorder)
Congenital Disease	SNOMED	234131004	Splenoportal vascular anomaly (disorder)
Congenital Disease	SNOMED	234132006	Congenital abnormality of great veins and coronary sinus (disorder)
Congenital Disease	SNOMED	234133001	Capillary-venous malformation (disorder)
Congenital Disease	SNOMED	234134007	Venous-lymphatic malformation (disorder)
Congenital Disease	SNOMED	234136009	Capillary-venous-lymphatic malformation (disorder)
Congenital Disease	SNOMED	234138005	Bannayan syndrome (disorder)
Congenital Disease	SNOMED	234139002	Vascular neurocutaneous syndrome (disorder)
Congenital Disease	SNOMED	234141001	Congenital arteriovenous malformation (disorder)
Congenital Disease	SNOMED	234142008	Cerebral arteriovenous malformation (disorder)
Congenital Disease	SNOMED	234143003	Parkes Weber syndrome (disorder)
Congenital Disease	SNOMED	234146006	Hennekam lymphangiectasia-lymphedema syndrome (disorder)
Congenital Disease	SNOMED	234148007	Congenital arteriovenous fistula (disorder)
Congenital Disease	SNOMED	234149004	Congenital arteriovenous fistula of brain (disorder)
Congenital Disease	SNOMED	234150004	Congenital arteriovenous fistula occlusion (disorder)
Congenital Disease	SNOMED	234151000	Congenital arteriovenous fistula stenosis (disorder)
Congenital Disease	SNOMED	234152007	Congenital arteriovenous fistula thrombosis (disorder)
Congenital Disease	SNOMED	234153002	Congenital arteriovenous fistula infection (disorder)
Congenital Disease	SNOMED	234154008	Congenital arteriovenous fistula aneurysm (disorder)
Congenital Disease	SNOMED	234155009	Congenital arteriovenous fistula hemorrhage (disorder)
Congenital Disease	SNOMED	234156005	Congenital arteriovenous fistula rupture (disorder)
Congenital Disease	SNOMED	234157001	Arteriovenous-lymphatic malformation (disorder)
Congenital Disease	SNOMED	234158006	Specific mixed vascular syndrome (disorder)
Congenital Disease	SNOMED	234159003	Multiple dysplasia syndrome (disorder)
Congenital Disease	SNOMED	234160008	Weber's true diffuse phlebarteriectasis (disorder)
Congenital Disease	SNOMED	234633000	Centromeric instability of chromosomes 1,9 and 16 and immunodeficiency (disorder)
Congenital Disease	SNOMED	234635007	Chromosome 22 abnormalities with hypogammaglobulinemia (disorder)
Congenital Disease	SNOMED	234636008	Monosomy 22 and absence of immunoglobulin A (disorder)
Congenital Disease	SNOMED	234638009	Microcephaly, normal intelligence and immunodeficiency (disorder)
Congenital Disease	SNOMED	234639001	Triple X syndrome, epilepsy, and hypogammaglobulinemia (disorder)
Congenital Disease	SNOMED	23512004	Atresia of salivary duct (disorder)
Congenital Disease	SNOMED	235143007	Midline sinus of the upper lip (disorder)
Congenital Disease	SNOMED	23544000	Congenital stenosis of vagina (disorder)
Congenital Disease	SNOMED	2355008	Rud's syndrome (disorder)
Congenital Disease	SNOMED	235622007	Esophageal web / ring (disorder)
Congenital Disease	SNOMED	23568008	Metatarsus adductus (disorder)
Congenital Disease	SNOMED	235685007	Hamartoma of stomach (disorder)
Congenital Disease	SNOMED	235699006	Megaduodenum (disorder)
Congenital Disease	SNOMED	235729009	Congenital microvillous atrophy (disorder)
Congenital Disease	SNOMED	235730004	Familial absence of villi (disorder)
Congenital Disease	SNOMED	235812003	Malrotation of the intestine type IA (disorder)
Congenital Disease	SNOMED	235813008	Malrotation of the intestine type IIA (disorder)
Congenital Disease	SNOMED	235814002	Malrotation of the intestine type IIB (disorder)
Congenital Disease	SNOMED	235815001	Malrotation of the intestine type IIC (disorder)
Congenital Disease	SNOMED	235816000	Malrotation of the intestine type IIIA (disorder)
Congenital Disease	SNOMED	235817009	Malrotation of the intestine type IIIB (disorder)

Congenital Disease	SNOMED	235913009	Congenital disorder of gallbladder and biliary tract (disorder)
Congenital Disease	SNOMED	235961002	Polycystic disease of pancreas (disorder)
Congenital Disease	SNOMED	235977001	Congenital malformation of pancreas (disorder)
Congenital Disease	SNOMED	236026001	Patent processus vaginalis (disorder)
Congenital Disease	SNOMED	236033001	Congenital umbilical defect (disorder)
Congenital Disease	SNOMED	23610003	Anonychia (disorder)
Congenital Disease	SNOMED	236419006	Progressive hereditary glomerulonephritis without deafness (disorder)
Congenital Disease	SNOMED	236443009	Medullary sponge kidney (disorder)
Congenital Disease	SNOMED	236482006	Inherited renal tubule insufficiency with cholestatic jaundice (disorder)
Congenital Disease	SNOMED	236492003	Congenital renal artery aneurysm (disorder)
Congenital Disease	SNOMED	236529001	Prune belly syndrome with pulmonic stenosis, intellectual disability and deafness (disorder)
Congenital Disease	SNOMED	236530006	Pulmonic stenosis and congenital nephrosis (disorder)
Congenital Disease	SNOMED	236531005	Renal dysplasia and retinal aplasia (disorder)
Congenital Disease	SNOMED	236533008	Ochoa syndrome (disorder)
Congenital Disease	SNOMED	236705005	Derodidymis (disorder)
Congenital Disease	SNOMED	236759008	Congenital familial idiopathic priapism (disorder)
Congenital Disease	SNOMED	236779000	Congenital abnormality of scrotum (disorder)
Congenital Disease	SNOMED	236780002	Bifid scrotum (disorder)
Congenital Disease	SNOMED	23678004	Congenital pyloric antral membrane (disorder)
Congenital Disease	SNOMED	236793007	Azoospermia with absent vasa in association with cystic fibrosis trait (disorder)
Congenital Disease	SNOMED	236796004	Hypogonadism with prune belly syndrome (disorder)
Congenital Disease	SNOMED	236799006	Congenitally impaired spermatogenesis (disorder)
Congenital Disease	SNOMED	236807008	Congenital obstructive azoospermia (disorder)
Congenital Disease	SNOMED	23686004	Ring chromosome 20 syndrome (disorder)
Congenital Disease	SNOMED	237227006	Congenital heart disease in pregnancy (disorder)
Congenital Disease	SNOMED	237513002	Congenital anomaly of bone and joint (disorder)
Congenital Disease	SNOMED	237606005	Hereditary benign acanthosis nigricans with insulin resistance (disorder)
Congenital Disease	SNOMED	237608006	Lipodystrophy, partial, with Rieger anomaly, short stature, and insulinopenic diabetes mellitus (disorder)
Congenital Disease	SNOMED	237610008	Acrorenal field defect, ectodermal dysplasia, and lipoatrophic diabetes (disorder)
Congenital Disease	SNOMED	237614004	Bird-headed dwarfism with progressive ataxia, insulin-resistant diabetes, goiter, and primary gonadal insufficiency (disorder)
Congenital Disease	SNOMED	237714006	Hamartoma of hypothalamus (disorder)
Congenital Disease	SNOMED	237720007	Hamartoma of pituitary and hypothalamus (disorder)
Congenital Disease	SNOMED	237764004	Congenital adrenal hypoplasia, X-linked (disorder)
Congenital Disease	SNOMED	237804005	Absent testes (finding)
Congenital Disease	SNOMED	237918004	Waardenburg syndrome type 3 (disorder)
Congenital Disease	SNOMED	238064009	Zellweger's-like syndrome (disorder)
Congenital Disease	SNOMED	23817003	Levy-Hollister syndrome (disorder)
Congenital Disease	SNOMED	238625005	Follicular keratosis (disorder)
Congenital Disease	SNOMED	238626006	Keratosis pilaris decalvans (disorder)
Congenital Disease	SNOMED	238627002	Follicular ichthyosis (disorder)
Congenital Disease	SNOMED	238631008	Linear porokeratosis (disorder)
Congenital Disease	SNOMED	238632001	Giant porokeratosis (disorder)
Congenital Disease	SNOMED	238633006	Disseminated superficial porokeratosis (disorder)
Congenital Disease	SNOMED	238634000	Benign acanthosis nigricans (disorder)
Congenital Disease	SNOMED	238715009	Developmental abnormality of nail (disorder)
Congenital Disease	SNOMED	23876003	Congenital dislocation of shoulder (disorder)
Congenital Disease	SNOMED	23880008	Congenital anomaly of peripheral nerve (disorder)
Congenital Disease	SNOMED	238826008	de Barsy syndrome (disorder)
Congenital Disease	SNOMED	238836000	Kindler's syndrome (disorder)
Congenital Disease	SNOMED	238851009	Extensive congenital erosions, vesicles and reticulate scarring (disorder)
Congenital Disease	SNOMED	238855000	Hereditary camptodactyly (disorder)
Congenital Disease	SNOMED	238871000	Metageria (disorder)
Congenital Disease	SNOMED	238872007	Acrogeria (disorder)
Congenital Disease	SNOMED	238875009	Wrinkly skin syndrome (disorder)
Congenital Disease	SNOMED	239001006	Genodermatosis (disorder)
Congenital Disease	SNOMED	239006001	Ectodermal dysplasia with hair-tooth-nail-sweating defect (disorder)
Congenital Disease	SNOMED	239007005	Hypohidrotic X-linked ectodermal dysplasia (disorder)
Congenital Disease	SNOMED	239009008	Roselli-Gulienetti ectodermal dysplasia (disorder)
Congenital Disease	SNOMED	239010003	Alopecia, onychodysplasia, hypohidrosis, deafness ectodermal dysplasia (disorder)
Congenital Disease	SNOMED	239011004	Basan syndrome (disorder)
Congenital Disease	SNOMED	239012006	Greither type of ectodermal dysplasia (disorder)
Congenital Disease	SNOMED	239013001	Anonychia with bizarre flexural pigmentation (disorder)
Congenital Disease	SNOMED	239014007	Tricho-onychodental dysplasia (disorder)
Congenital Disease	SNOMED	239015008	Ectodermal dysplasia with hair-tooth-nail defects (disorder)
Congenital Disease	SNOMED	239018005	Schoepf-Schulz-Passage syndrome (disorder)
Congenital Disease	SNOMED	239019002	Odonto-onychial dysplasia with alopecia (disorder)
Congenital Disease	SNOMED	239020008	Fried's tooth and nail syndrome (disorder)
Congenital Disease	SNOMED	239021007	Hypodontia and nail dysgenesis (disorder)
Congenital Disease	SNOMED	239022000	Dermodental dysplasia (disorder)

Congenital Disease	SNOMED	239023005	Salamon's syndrome (disorder)
Congenital Disease	SNOMED	239024004	Ectodermal dysplasia, syndactyly and pili torti (disorder)
Congenital Disease	SNOMED	239025003	Dwarfism, alopecia, pseudoanodontia, cutis laxa (disorder)
Congenital Disease	SNOMED	239027006	Ectodermal dysplasia with hair-tooth defects (disorder)
Congenital Disease	SNOMED	239028001	Odontotrichomelic syndrome (disorder)
Congenital Disease	SNOMED	239030004	Orofacial-digital syndrome III (disorder)
Congenital Disease	SNOMED	239031000	Orofacial-digital syndrome IV (disorder)
Congenital Disease	SNOMED	239032007	Berlin syndrome (disorder)
Congenital Disease	SNOMED	239035009	Ectodermal dysplasia with hair-nail defect (disorder)
Congenital Disease	SNOMED	239036005	Tricho-oculodermovertebral syndrome (disorder)
Congenital Disease	SNOMED	239037001	Curly hair, ankyloblepharon, nail dysplasia syndrome (disorder)
Congenital Disease	SNOMED	239038006	Kirman syndrome (disorder)
Congenital Disease	SNOMED	239040001	Ectodermal dysplasia with tooth-nail defects (disorder)
Congenital Disease	SNOMED	239041002	Dento-oculocutaneous syndrome (disorder)
Congenital Disease	SNOMED	239042009	Ectodermal dysplasia with tooth-sweating defect (disorder)
Congenital Disease	SNOMED	239043004	Sandman-Andra syndrome (disorder)
Congenital Disease	SNOMED	239046007	Ectodermal dysplasia with nail defect (disorder)
Congenital Disease	SNOMED	239048008	Ectodermal dysplasia with sweating defect (disorder)
Congenital Disease	SNOMED	239049000	Hypohidrosis with neurolabyrinthitis (disorder)
Congenital Disease	SNOMED	239050000	Alopecia, nail dystrophy, ophthalmic complications, thyroid dysfunction, hypohidrosis, ephelides, enteropathy and respiratory tract infections (disorder)
Congenital Disease	SNOMED	239053003	Multiple benign annular creases of extremities (disorder)
Congenital Disease	SNOMED	239055005	Hereditary clubbing (disorder)
Congenital Disease	SNOMED	239056006	Flynn-Aird syndrome (disorder)
Congenital Disease	SNOMED	239057002	Cutaneous syndrome with ichthyosis (disorder)
Congenital Disease	SNOMED	239059004	Keratitis ichthyosis and deafness syndrome (disorder)
Congenital Disease	SNOMED	239060009	Atypical ichthyosis vulgaris with hypogonadism (disorder)
Congenital Disease	SNOMED	239062001	Erythrokeratoderma en cocardes (disorder)
Congenital Disease	SNOMED	239063006	Erythrokeratoderma progressiva of Gottron (disorder)
Congenital Disease	SNOMED	239064000	Keratolytic winter erythema (disorder)
Congenital Disease	SNOMED	239065004	Keratolysis exfoliativa (disorder)
Congenital Disease	SNOMED	239066003	Hereditary palmoplantar keratoderma (disorder)
Congenital Disease	SNOMED	239067007	Palmoplantar keratoderma transgrediens (disorder)
Congenital Disease	SNOMED	239069005	Acroerythrokeratoderma (disorder)
Congenital Disease	SNOMED	239070006	Progressive palmoplantar keratoderma of Greither (disorder)
Congenital Disease	SNOMED	239071005	Epidermolytic palmoplantar keratoderma of Vorner (disorder)
Congenital Disease	SNOMED	239072003	Congenital palmoplantar and perioral keratoderma of Olmsted (disorder)
Congenital Disease	SNOMED	239073008	Circumscribed palmoplantar keratoderma (disorder)
Congenital Disease	SNOMED	239075001	Palmoplantar keratoderma with leukoplakia (disorder)
Congenital Disease	SNOMED	239076000	Palmoplantar hyperkeratosis sclerodactyly syndrome (disorder)
Congenital Disease	SNOMED	239078004	Papuloverrucous palmoplantar keratoderma of Jakac-Wolf (disorder)
Congenital Disease	SNOMED	239079007	Inherited cutaneous hyperpigmentation (disorder)
Congenital Disease	SNOMED	239084001	Naegeli-Franceschetti-Jadassohn syndrome (disorder)
Congenital Disease	SNOMED	239087008	Cantu's syndrome (disorder)
Congenital Disease	SNOMED	239088003	Dermatopathia pigmentosa reticularis (disorder)
Congenital Disease	SNOMED	2391001	Achondrogenesis (disorder)
Congenital Disease	SNOMED	239112008	Epidermal nevus syndrome (disorder)
Congenital Disease	SNOMED	239115005	Eccrine angiomatous hamartoma (disorder)
Congenital Disease	SNOMED	239118007	Porokeratotic eccrine ostial and dermal duct nevus (disorder)
Congenital Disease	SNOMED	239121009	Syringocystadenoma papilliferum (disorder)
Congenital Disease	SNOMED	239123007	Straight hair nevus (disorder)
Congenital Disease	SNOMED	239126004	Hairy malformation of palms and soles (disorder)
Congenital Disease	SNOMED	239128003	Moniliform hamartoma (disorder)
Congenital Disease	SNOMED	239140003	Nevus elasticus (disorder)
Congenital Disease	SNOMED	23914009	Ectopic splenic tissue (disorder)
Congenital Disease	SNOMED	239142006	Michelin-tire baby (disorder)
Congenital Disease	SNOMED	239144007	Congenital erector pili hamartoma (disorder)
Congenital Disease	SNOMED	239145008	Diffuse smooth muscle hamartoma (disorder)
Congenital Disease	SNOMED	239146009	Rhabdomyomatous mesenchymal hamartoma (disorder)
Congenital Disease	SNOMED	239152005	Congenital absence of skin on scalp (disorder)
Congenital Disease	SNOMED	239153000	Congenital absence of skin on scalp with epidermal nevi (disorder)
Congenital Disease	SNOMED	23931000119104	Hydrocephalus due to Arnold Chiari malformation type 2 (disorder)
Congenital Disease	SNOMED	23939000	Imperfect fusion of skull (disorder)
Congenital Disease	SNOMED	23941000119108	Arnold Chiari type 2 without hydrocephalus (disorder)
Congenital Disease	SNOMED	23971000119101	Congenital lumbar spondylolisthesis (disorder)
Congenital Disease	SNOMED	239826001	Chronic infantile neurological, cutaneous and articular syndrome (disorder)
Congenital Disease	SNOMED	240061000	Congenital muscular dystrophy with arthrogryposis multiplex congenita (disorder)
Congenital Disease	SNOMED	24194000	Complete bilateral cleft palate (disorder)
Congenital Disease	SNOMED	24210004	Congenital chorioretinal degeneration (disorder)
Congenital Disease	SNOMED	24269006	Distal arthrogryposis syndrome (disorder)
Congenital Disease	SNOMED	24291004	Congenital dilatation of colon (disorder)
Congenital Disease	SNOMED	24297000	Xiphopagus (disorder)
Congenital Disease	SNOMED	24358005	Accessory thymic tissue (disorder)

Congenital Disease	SNOMED	2438005	Iniencephaly (disorder)
Congenital Disease	SNOMED	24533004	Thoracodelphus (disorder)
Congenital Disease	SNOMED	24559001	Mutilating keratoderma (disorder)
Congenital Disease	SNOMED	24606006	Accessory eyelid (disorder)
Congenital Disease	SNOMED	24614000	Laterality sequence (disorder)
Congenital Disease	SNOMED	24629003	Metaphyseal chondrodysplasia, Jansen type (disorder)
Congenital Disease	SNOMED	24679000	Thoracopagus parasiticus (disorder)
Congenital Disease	SNOMED	246836006	Absence of meibomian glands (finding)
Congenital Disease	SNOMED	247127002	Retinal arteriovenous shunt (disorder)
Congenital Disease	SNOMED	247204001	Morning glory disc (disorder)
Congenital Disease	SNOMED	247249003	Absence of ear canal (finding)
Congenital Disease	SNOMED	247476001	Raised birthmark (disorder)
Congenital Disease	SNOMED	247477005	Flat birthmark (disorder)
Congenital Disease	SNOMED	24750000	Townes syndrome (disorder)
Congenital Disease	SNOMED	24752008	Infantile cortical hyperostosis (disorder)
Congenital Disease	SNOMED	247524003	Lanugo (finding)
Congenital Disease	SNOMED	247526001	Partial failure of hair growth (finding)
Congenital Disease	SNOMED	24786004	7p partial monosomy (disorder)
Congenital Disease	SNOMED	24787008	Congenital absence of broad ligament (disorder)
Congenital Disease	SNOMED	24814002	Potter's facies (disorder)
Congenital Disease	SNOMED	248199009	Down's facies (finding)
Congenital Disease	SNOMED	248200007	Dysmorphic facies (finding)
Congenital Disease	SNOMED	248206001	Marfanoid facies (finding)
Congenital Disease	SNOMED	248372000	Asymmetrical skull (finding)
Congenital Disease	SNOMED	248391000	Apparent excessive orbital separation (finding)
Congenital Disease	SNOMED	248409006	Single transverse palmar crease (finding)
Congenital Disease	SNOMED	248814001	Tubular breast (finding)
Congenital Disease	SNOMED	248845002	Septate hymen (disorder)
Congenital Disease	SNOMED	248846001	Hymenal orifice dilated (finding)
Congenital Disease	SNOMED	248847005	Annular hymen (finding)
Congenital Disease	SNOMED	248848000	Horseshoe shape hymen (finding)
Congenital Disease	SNOMED	248868006	Clitoris resembles penis (finding)
Congenital Disease	SNOMED	248871003	Vagina absent (finding)
Congenital Disease	SNOMED	248877004	Vaginal septum (finding)
Congenital Disease	SNOMED	248912003	Uterine cervix double (finding)
Congenital Disease	SNOMED	248919007	Elongated cervix (finding)
Congenital Disease	SNOMED	249233008	Absent scrotum (finding)
Congenital Disease	SNOMED	249239007	Absence of testicle in scrotum (finding)
Congenital Disease	SNOMED	249257008	Hooded foreskin (finding)
Congenital Disease	SNOMED	249326006	Bifid nasal tip (finding)
Congenital Disease	SNOMED	249341006	Cleft lip nasal deformity (finding)
Congenital Disease	SNOMED	249372005	Absent maxilla (finding)
Congenital Disease	SNOMED	249375007	Defect in wall of frontal sinus (finding)
Congenital Disease	SNOMED	249388005	Short frenulum of tongue (finding)
Congenital Disease	SNOMED	249389002	Split frenulum of tongue (finding)
Congenital Disease	SNOMED	249435003	Absence of larynx (finding)
Congenital Disease	SNOMED	249439009	Vocal cord absent (finding)
Congenital Disease	SNOMED	249442003	Vocal cords webbed (finding)
Congenital Disease	SNOMED	249491000119100	Structural developmental anomalies of neurenteric canal (disorder)
Congenital Disease	SNOMED	2495006	Congenital cerebral arteriovenous aneurysm (disorder)
Congenital Disease	SNOMED	24963004	Congenital anomaly of sternocleidomastoid muscle (disorder)
Congenital Disease	SNOMED	249654008	Funneled anus (finding)
Congenital Disease	SNOMED	249672002	Asymmetrical thorax (finding)
Congenital Disease	SNOMED	249673007	Shield-shaped chest (finding)
Congenital Disease	SNOMED	249678003	Absence of clavicle (finding)
Congenital Disease	SNOMED	249692009	Absence of sternum (finding)
Congenital Disease	SNOMED	249696007	Short rib (finding)
Congenital Disease	SNOMED	249703002	Wavy constrictions of ribs (finding)
Congenital Disease	SNOMED	249712000	Lordosis absent (finding)
Congenital Disease	SNOMED	249713005	Lordosis reversed (finding)
Congenital Disease	SNOMED	249714004	Lacks lumbar extension (finding)
Congenital Disease	SNOMED	249726009	Absence of scapula (finding)
Congenital Disease	SNOMED	249732004	Pelvis tilted (finding)
Congenital Disease	SNOMED	249765007	Short finger (finding)
Congenital Disease	SNOMED	249769001	Webbed fingers (finding)
Congenital Disease	SNOMED	249773003	Broad thumbs (finding)
Congenital Disease	SNOMED	249779004	Hypertrophy of leg (finding)
Congenital Disease	SNOMED	249808002	Equinovarus deformity of foot (finding)
Congenital Disease	SNOMED	249810000	Forefoot adductus (finding)
Congenital Disease	SNOMED	249814009	Bean-shaped foot (finding)
Congenital Disease	SNOMED	249883009	Drooping of soft palate (finding)
Congenital Disease	SNOMED	249884003	Deviation of uvula (finding)
Congenital Disease	SNOMED	249885002	Poor elevation soft palate (finding)
Congenital Disease	SNOMED	25065001	Hemoglobin E disease (disorder)
Congenital Disease	SNOMED	250941001	Right ventricular fibromuscular infundibular stenosis (disorder)

Congenital Disease	SNOMED	250942008	Right ventricular muscular infundibular stenosis (disorder)
Congenital Disease	SNOMED	250982001	Commissural fusion of aortic cusp (disorder)
Congenital Disease	SNOMED	250983006	Bicuspid doming of aortic cusp (disorder)
Congenital Disease	SNOMED	250994005	Slit-like mitral valve orifice (finding)
Congenital Disease	SNOMED	250995006	Irregular mitral valve orifice (finding)
Congenital Disease	SNOMED	250997003	Multiple mitral valve orifice (finding)
Congenital Disease	SNOMED	251038002	Aortic [REDACTED] congenital abnormality (disorder)
Congenital Disease	SNOMED	251046001	Multiple peripheral pulmonary artery stenoses (disorder)
Congenital Disease	SNOMED	25148007	Congenital absence of uvula (disorder)
Congenital Disease	SNOMED	251729009	Congenital malformation of angle of anterior chamber of eye (disorder)
Congenital Disease	SNOMED	251730004	Goniodysgenesis (disorder)
Congenital Disease	SNOMED	25201000119104	Transitional lumbosacral vertebra (disorder)
Congenital Disease	SNOMED	252246005	Pseudoxanthoma elasticum (disorder)
Congenital Disease	SNOMED	253098009	Neural tube defect (disorder)
Congenital Disease	SNOMED	253101008	Congenital cerebral hernia (disorder)
Congenital Disease	SNOMED	253103006	Frontal encephalocele (disorder)
Congenital Disease	SNOMED	253104000	Frontoethmoidal encephalocele (disorder)
Congenital Disease	SNOMED	253105004	Absence of roof of orbit (disorder)
Congenital Disease	SNOMED	253106003	Nasofrontal encephalocele (disorder)
Congenital Disease	SNOMED	253107007	Nasopharyngeal encephalocele (disorder)
Congenital Disease	SNOMED	253108002	Temporal encephalocele (disorder)
Congenital Disease	SNOMED	253109005	Parietal encephalocele (disorder)
Congenital Disease	SNOMED	253111001	Thoracolumbar spina bifida without hydrocephalus - closed (disorder)
Congenital Disease	SNOMED	253113003	Rachischisis with hydrocephalus (disorder)
Congenital Disease	SNOMED	253114009	Myelocele with hydrocephalus (disorder)
Congenital Disease	SNOMED	253117002	Closed spina bifida with Arnold-Chiari malformation (disorder)
Congenital Disease	SNOMED	253118007	Thoracolumbar spina bifida with hydrocephalus - closed (disorder)
Congenital Disease	SNOMED	253119004	Hemimyelocele (disorder)
Congenital Disease	SNOMED	253120005	Lipomeningocele (disorder)
Congenital Disease	SNOMED	253124001	Myelodysplasia of spinal cord (disorder)
Congenital Disease	SNOMED	253125000	Spinal hamartoma (disorder)
Congenital Disease	SNOMED	253128003	Abnormality of neurogenesis (disorder)
Congenital Disease	SNOMED	253130001	Secondary microcephaly (disorder)
Congenital Disease	SNOMED	253133004	Hydrocephalus with anomaly of aqueduct of Sylvius (disorder)
Congenital Disease	SNOMED	253135006	Defect of telencephalic division (disorder)
Congenital Disease	SNOMED	253136007	Lobar holoprosencephaly (disorder)
Congenital Disease	SNOMED	253137003	Alobar holoprosencephaly (disorder)
Congenital Disease	SNOMED	253138008	Semi-lobar holoprosencephaly (disorder)
Congenital Disease	SNOMED	253139000	Agenesis of corpus callosum with lipoma (disorder)
Congenital Disease	SNOMED	253140003	Partial agenesis of corpus callosum (disorder)
Congenital Disease	SNOMED	253142006	Atrophy of corpus callosum (disorder)
Congenital Disease	SNOMED	253143001	Absence of septum pellucidum (disorder)
Congenital Disease	SNOMED	253146009	Disorder of neuronal migration and differentiation (disorder)
Congenital Disease	SNOMED	253147000	Type 1 lissencephaly (disorder)
Congenital Disease	SNOMED	253148005	Miller Dieker syndrome (disorder)
Congenital Disease	SNOMED	253149002	Type 2 lissencephaly (disorder)
Congenital Disease	SNOMED	253150002	Neuronal heterotopia (disorder)
Congenital Disease	SNOMED	253151003	Nodular heterotopia (disorder)
Congenital Disease	SNOMED	253152005	Laminar heterotopia (disorder)
Congenital Disease	SNOMED	253153000	Cortical dysplasia (disorder)
Congenital Disease	SNOMED	253154006	Localized cortical dysplasia (disorder)
Congenital Disease	SNOMED	253156008	Cortical dysplasia with hemimegalencephaly (disorder)
Congenital Disease	SNOMED	253158009	Hydranencephaly with proliferative vasculopathy (disorder)
Congenital Disease	SNOMED	253159001	Schizencephaly (disorder)
Congenital Disease	SNOMED	253160006	Colpocephaly (disorder)
Congenital Disease	SNOMED	253166000	Lateral meningocele (disorder)
Congenital Disease	SNOMED	253167009	Microdysgenesis (disorder)
Congenital Disease	SNOMED	253168004	Familial megalencephaly (disorder)
Congenital Disease	SNOMED	253169007	Sporadic megalencephaly (disorder)
Congenital Disease	SNOMED	253170008	Hemimegalencephaly (disorder)
Congenital Disease	SNOMED	253171007	Dysgenesis of the cerebellum (disorder)
Congenital Disease	SNOMED	253172000	Agenesis of cerebellum (disorder)
Congenital Disease	SNOMED	253174004	Aplasia of the vermis (disorder)
Congenital Disease	SNOMED	253175003	Familial aplasia of the vermis (disorder)
Congenital Disease	SNOMED	253176002	Gillespie syndrome (disorder)
Congenital Disease	SNOMED	253177006	Absence of the vermis (disorder)
Congenital Disease	SNOMED	253178001	Granular cell hypoplasia (disorder)
Congenital Disease	SNOMED	253179009	Cerebellar cortical dysplasia (disorder)
Congenital Disease	SNOMED	253180007	Dysgenesis of the brainstem (disorder)
Congenital Disease	SNOMED	253181006	Olive dysplasia (disorder)
Congenital Disease	SNOMED	253182004	Dentate dysplasia (disorder)
Congenital Disease	SNOMED	253183009	Olivary heterotopia (disorder)
Congenital Disease	SNOMED	253184003	Chiari malformation (disorder)
Congenital Disease	SNOMED	253185002	Chiari malformation type I (disorder)
Congenital Disease	SNOMED	253186001	Chiari malformation type III (disorder)

Congenital Disease	SNOMED	253187005	Chiari malformation type IV (disorder)
Congenital Disease	SNOMED	253188000	Abnormality of canalization and retrogressive differentiation (disorder)
Congenital Disease	SNOMED	253189008	Sacral dysgenesis (disorder)
Congenital Disease	SNOMED	253190004	Lumbosacral agenesis (disorder)
Congenital Disease	SNOMED	253192007	Fibrolipoma of filum terminale (disorder)
Congenital Disease	SNOMED	253193002	Vascular malformation of the nervous system (disorder)
Congenital Disease	SNOMED	253195009	Persistent embryonic trigeminal artery (disorder)
Congenital Disease	SNOMED	253196005	Persistent embryonic otic artery (disorder)
Congenital Disease	SNOMED	253197001	Persistent embryonic hypoglossal artery (disorder)
Congenital Disease	SNOMED	253198006	Persistent embryonic proatlantal intersegmental artery (disorder)
Congenital Disease	SNOMED	253199003	Congenital malformation of the meninges (disorder)
Congenital Disease	SNOMED	253203003	Hypoplasia of brain gyri (disorder)
Congenital Disease	SNOMED	253206006	Congenital malformation of the eyebrow (disorder)
Congenital Disease	SNOMED	253207002	Synophrys (disorder)
Congenital Disease	SNOMED	253208007	Absent eyebrow (disorder)
Congenital Disease	SNOMED	253209004	Double eyebrow (disorder)
Congenital Disease	SNOMED	253212001	Epiblepharon (disorder)
Congenital Disease	SNOMED	253213006	Congenital ankyloblepharon (disorder)
Congenital Disease	SNOMED	253214000	Congenital distichiasis (disorder)
Congenital Disease	SNOMED	253215004	Alacrima (disorder)
Congenital Disease	SNOMED	253217007	Imperforate lacrimal punctum (disorder)
Congenital Disease	SNOMED	253218002	Supernumerary lacrimal punctum (disorder)
Congenital Disease	SNOMED	253219005	Agenesis of nasolacrimal duct (disorder)
Congenital Disease	SNOMED	253220004	Congenital lacrimal fistula (disorder)
Congenital Disease	SNOMED	253221000	Lenticonus (disorder)
Congenital Disease	SNOMED	253223002	Congenital polar cataract (disorder)
Congenital Disease	SNOMED	253224008	Congenital anterior polar cataract (disorder)
Congenital Disease	SNOMED	253225009	Congenital posterior polar cataract (disorder)
Congenital Disease	SNOMED	253226005	Congenital sutural cataract (disorder)
Congenital Disease	SNOMED	253227001	Rubella cataract (disorder)
Congenital Disease	SNOMED	253228006	Embryotoxon (disorder)
Congenital Disease	SNOMED	253231007	Aniridia type 1 (disorder)
Congenital Disease	SNOMED	253232000	Aniridia type 2 (disorder)
Congenital Disease	SNOMED	253233005	Pseudo-polycoria (disorder)
Congenital Disease	SNOMED	253234004	Congenital heterochromia iridis (disorder)
Congenital Disease	SNOMED	253235003	Congenital cyst of iris (disorder)
Congenital Disease	SNOMED	253236002	Congenital malformation of vitreous humor (disorder)
Congenital Disease	SNOMED	253238001	Partial hypoplasia of optic disc (disorder)
Congenital Disease	SNOMED	253239009	Sectorial hypoplasia of optic disc (disorder)
Congenital Disease	SNOMED	253240006	Trabecular dysgenesis (disorder)
Congenital Disease	SNOMED	253241005	Orbital dystopia (disorder)
Congenital Disease	SNOMED	253242003	Horizontal orbital dystopia (disorder)
Congenital Disease	SNOMED	253243008	Vertical orbital dystopia (disorder)
Congenital Disease	SNOMED	253244002	Rotational orbital dystopia (disorder)
Congenital Disease	SNOMED	253247009	Congenital stricture of osseous meatus (disorder)
Congenital Disease	SNOMED	253251006	Posteriorly rotated ear (disorder)
Congenital Disease	SNOMED	253252004	Vulcan ear (disorder)
Congenital Disease	SNOMED	253253009	Cat ear (disorder)
Congenital Disease	SNOMED	253254003	Aztec ear (disorder)
Congenital Disease	SNOMED	253255002	Simple ear (disorder)
Congenital Disease	SNOMED	253259008	Sinus of branchial cleft (disorder)
Congenital Disease	SNOMED	253264007	Congenital heart disease, septal and bulbar anomalies (disorder)
Congenital Disease	SNOMED	253267000	Congenital abnormality of relationship of cardiac component (disorder)
Congenital Disease	SNOMED	253268005	Abnormal relationship of right ventricle to left ventricle (disorder)
Congenital Disease	SNOMED	253269002	Criss-cross heart (disorder)
Congenital Disease	SNOMED	253270001	Abnormal relationship of aortic orifice to pulmonary orifice (disorder)
Congenital Disease	SNOMED	253271002	Mirror-imaged heart (disorder)
Congenital Disease	SNOMED	253272009	Congenital abnormality of cardiac connection (disorder)
Congenital Disease	SNOMED	253273004	Cardiac septal defects (disorder)
Congenital Disease	SNOMED	253274005	Abnormal atrioventricular connection (disorder)
Congenital Disease	SNOMED	253275006	Abnormal atrioventricular connection - biventricular (disorder)
Congenital Disease	SNOMED	253276007	Cor triloculare biventriculare (disorder)
Congenital Disease	SNOMED	253277003	Discordant atrioventricular connection (disorder)
Congenital Disease	SNOMED	253278008	Ambiguous atrioventricular connection (disorder)
Congenital Disease	SNOMED	253279000	Absent atrioventricular connection with straddling valve (disorder)
Congenital Disease	SNOMED	253280002	Abnormal atrioventricular connection - univentricular (disorder)
Congenital Disease	SNOMED	253281003	Double inlet ventricle (disorder)
Congenital Disease	SNOMED	253282005	Double inlet right ventricle (disorder)
Congenital Disease	SNOMED	253283000	Double inlet left ventricle (disorder)
Congenital Disease	SNOMED	253284006	Double inlet to ventricle of indeterminate morphology (disorder)
Congenital Disease	SNOMED	253285007	Absent right sided atrioventricular connection (disorder)
Congenital Disease	SNOMED	253286008	Left sided atrium connecting to left ventricle (disorder)
Congenital Disease	SNOMED	253287004	Left sided atrium connecting to right ventricle (disorder)
Congenital Disease	SNOMED	253288009	Left sided atrium connecting to both ventricles (disorder)

Congenital Disease	SNOMED	253289001	Left sided atrium connecting to ventricle of indeterminate morphology (disorder)
Congenital Disease	SNOMED	253290005	Absent left sided atrioventricular connection (disorder)
Congenital Disease	SNOMED	253291009	Right sided atrium connecting to right ventricle (disorder)
Congenital Disease	SNOMED	253293007	Right sided atrium connecting to both ventricles (disorder)
Congenital Disease	SNOMED	253294001	Right sided atrium connecting to ventricle of indeterminate morphology (disorder)
Congenital Disease	SNOMED	253295000	Abnormal ventriculoarterial connection (disorder)
Congenital Disease	SNOMED	253297008	Transposition of aorta (disorder)
Congenital Disease	SNOMED	253298003	Double outlet right ventricle with subaortic ventricular septal defect (disorder)
Congenital Disease	SNOMED	253299006	Double outlet right ventricle with noncommitted ventricular septal defect (disorder)
Congenital Disease	SNOMED	253300003	Double outlet right ventricle with doubly committed ventricular septal defect (disorder)
Congenital Disease	SNOMED	253301004	Double outlet from ventricle of indeterminate morphology (disorder)
Congenital Disease	SNOMED	253302006	Single outlet ventriculoarterial connection (disorder)
Congenital Disease	SNOMED	253303001	Solitary aortic trunk with pulmonary atresia (disorder)
Congenital Disease	SNOMED	253304007	Solitary pulmonary trunk with aortic atresia (disorder)
Congenital Disease	SNOMED	253305008	Solitary arterial trunk (disorder)
Congenital Disease	SNOMED	253306009	Abnormality of right superior vena cava (disorder)
Congenital Disease	SNOMED	253307000	Atretic right superior vena cava (disorder)
Congenital Disease	SNOMED	253308005	Absent right superior vena cava (disorder)
Congenital Disease	SNOMED	253310007	Anomalous insertion of right superior vena cava to left atrium (disorder)
Congenital Disease	SNOMED	253311006	Bilateral superior vena cava (disorder)
Congenital Disease	SNOMED	253312004	Absent bridging vein (disorder)
Congenital Disease	SNOMED	253313009	Inferior vena cava interruption with left sided hemiazygos continuation (disorder)
Congenital Disease	SNOMED	253314003	Inferior vena cava interruption with right sided azygos continuation (disorder)
Congenital Disease	SNOMED	253315002	Inferior vena cava interruption with bilateral azygos continuation (disorder)
Congenital Disease	SNOMED	253316001	Abnormal inferior vena caval connection (disorder)
Congenital Disease	SNOMED	253317005	Inferior vena cava connecting to morphological left atrium (disorder)
Congenital Disease	SNOMED	253318000	Inferior vena cava connecting to coronary sinus (disorder)
Congenital Disease	SNOMED	253319008	Inferior vena cava to left of spine (disorder)
Congenital Disease	SNOMED	253320002	Inferior cava to left of spine with right descending aorta (disorder)
Congenital Disease	SNOMED	253321003	Anomalous termination of right pulmonary vein (disorder)
Congenital Disease	SNOMED	253322005	Obstructed pulmonary venous connection (disorder)
Congenital Disease	SNOMED	253323000	Coronary sinus abnormality (disorder)
Congenital Disease	SNOMED	253324006	Coronary sinus defect in left atrium (disorder)
Congenital Disease	SNOMED	253326008	Coronary sinus orifice atresia (disorder)
Congenital Disease	SNOMED	253327004	Congenital coronary sinus stenosis (disorder)
Congenital Disease	SNOMED	253328009	Coronary sinus to left ventricle fistula (disorder)
Congenital Disease	SNOMED	253329001	Ductus venosus abnormality (disorder)
Congenital Disease	SNOMED	253330006	Patent ductus venosus (disorder)
Congenital Disease	SNOMED	253331005	Closed ductus venosus (disorder)
Congenital Disease	SNOMED	253333008	Abnormal connection of hepatic vein to atrium (disorder)
Congenital Disease	SNOMED	253335001	Isomerism of atrial appendages (disorder)
Congenital Disease	SNOMED	253336000	Isomerism of right atrial appendage (disorder)
Congenital Disease	SNOMED	253337009	Isomerism of left atrial appendage (disorder)
Congenital Disease	SNOMED	253338004	Mirror imaged atria (disorder)
Congenital Disease	SNOMED	253339007	Right atrial abnormality (disorder)
Congenital Disease	SNOMED	253340009	Prominent valve of inferior vena cava (disorder)
Congenital Disease	SNOMED	253341008	Obstructive Eustachian valve (disorder)
Congenital Disease	SNOMED	253342001	Prolapse of Eustachian valve (disorder)
Congenital Disease	SNOMED	253343006	Anomalous valve of coronary sinus (disorder)
Congenital Disease	SNOMED	253344000	Abnormality of right atrial appendage (disorder)
Congenital Disease	SNOMED	253345004	Right atrial appendage absent (disorder)
Congenital Disease	SNOMED	253346003	Right atrial appendage - left - juxtaposition (disorder)
Congenital Disease	SNOMED	253347007	Right atrial appendage aneurysm (disorder)
Congenital Disease	SNOMED	253349005	Right atrial hypoplasia (disorder)
Congenital Disease	SNOMED	253351009	Giant right atrium (disorder)
Congenital Disease	SNOMED	253352002	Left atrial abnormality (disorder)
Congenital Disease	SNOMED	253353007	Divided left atrium (disorder)
Congenital Disease	SNOMED	253354001	Supramitral left atrial ring (disorder)
Congenital Disease	SNOMED	253355000	Abnormality of left atrial appendage (disorder)
Congenital Disease	SNOMED	253356004	Left atrial appendage absent (disorder)
Congenital Disease	SNOMED	253357008	Left atrial appendage - right - juxtaposition (disorder)
Congenital Disease	SNOMED	253360001	Left atrial hypoplasia (disorder)
Congenital Disease	SNOMED	25336002	Uterus parvicollis (disorder)
Congenital Disease	SNOMED	253362009	Giant left atrium (disorder)
Congenital Disease	SNOMED	253363004	Abnormality of atrial septum (disorder)
Congenital Disease	SNOMED	253364005	Foramen ovale valvar aneurysm (disorder)
Congenital Disease	SNOMED	253366007	Interauricular septal defect (disorder)
Congenital Disease	SNOMED	253369000	Sinus venosus defect with overriding superior vena cava (disorder)

Congenital Disease	SNOMED	253370004	Sinus venosus defect with overriding inferior vena cava (disorder)
Congenital Disease	SNOMED	253371000	Atrial septal defect through coronary sinus orifice (disorder)
Congenital Disease	SNOMED	253373002	Atrioventricular septal defect - isolated atrial component (disorder)
Congenital Disease	SNOMED	253374008	Congenital abnormality of atrioventricular valves in atrioventricular septal defect (disorder)
Congenital Disease	SNOMED	253375009	Tricuspid leaflet dysplasia (disorder)
Congenital Disease	SNOMED	253376005	Tricuspid annulus hypoplasia (disorder)
Congenital Disease	SNOMED	253377001	Dilatation of tricuspid annulus (disorder)
Congenital Disease	SNOMED	253378006	Overriding tricuspid valve (disorder)
Congenital Disease	SNOMED	253379003	Straddling tricuspid valve (disorder)
Congenital Disease	SNOMED	253380000	Tricuspid leaflet abnormality (disorder)
Congenital Disease	SNOMED	253381001	Absent tricuspid leaflet (disorder)
Congenital Disease	SNOMED	253382008	Double orifice of tricuspid valve (disorder)
Congenital Disease	SNOMED	253384009	Accessory tissue on tricuspid leaflet (disorder)
Congenital Disease	SNOMED	253385005	Abnormality of tricuspid chordae tendinae (disorder)
Congenital Disease	SNOMED	253386006	Arcade abnormality of tricuspid chordae tendinae (disorder)
Congenital Disease	SNOMED	253387002	Tricuspid chordae tendinae too short (disorder)
Congenital Disease	SNOMED	253388007	Tricuspid chordae tendinae too long (disorder)
Congenital Disease	SNOMED	253389004	Tricuspid chordae tendinae to outlet septum (disorder)
Congenital Disease	SNOMED	253390008	Tricuspid papillary muscle abnormality (disorder)
Congenital Disease	SNOMED	253391007	Parachute malformation of tricuspid valve (disorder)
Congenital Disease	SNOMED	253392000	Absent tricuspid papillary muscle (disorder)
Congenital Disease	SNOMED	253393005	Fused tricuspid papillary muscle (disorder)
Congenital Disease	SNOMED	253394004	Hypoplastic tricuspid papillary muscle (disorder)
Congenital Disease	SNOMED	253395003	Mitral valve dysplasia (disorder)
Congenital Disease	SNOMED	253396002	Mitral leaflet dysplasia (disorder)
Congenital Disease	SNOMED	253397006	Overriding mitral valve (disorder)
Congenital Disease	SNOMED	253399009	Straddling mitral valve (disorder)
Congenital Disease	SNOMED	253400002	Mitral leaflet abnormality (disorder)
Congenital Disease	SNOMED	2534005	Congenital absence of vena cava (disorder)
Congenital Disease	SNOMED	253401003	Absent mitral leaflets (disorder)
Congenital Disease	SNOMED	253402005	Double orifice of mitral valve (disorder)
Congenital Disease	SNOMED	253403000	Ebstein-like downward displacement of mitral valve (disorder)
Congenital Disease	SNOMED	253404006	Anterior leaflet of mitral valve attached to septum (disorder)
Congenital Disease	SNOMED	253405007	Accessory tissue on mitral leaflet (disorder)
Congenital Disease	SNOMED	253406008	Abnormality of mitral chordae tendinae (disorder)
Congenital Disease	SNOMED	253407004	Arcade abnormality of mitral chordae tendinae (disorder)
Congenital Disease	SNOMED	253408009	Mitral chordae tendinae too short (disorder)
Congenital Disease	SNOMED	253409001	Mitral chordae tendinae too long (disorder)
Congenital Disease	SNOMED	253410006	Mitral papillary muscle abnormality (disorder)
Congenital Disease	SNOMED	253411005	Absent mitral papillary muscle (disorder)
Congenital Disease	SNOMED	253412003	Fused mitral papillary muscles (disorder)
Congenital Disease	SNOMED	253413008	Hypoplastic mitral papillary muscle (disorder)
Congenital Disease	SNOMED	253414002	Atrioventricular septal defect and common atrioventricular junction (disorder)
Congenital Disease	SNOMED	253415001	Atrioventricular septal defect - isolated ventricular component (disorder)
Congenital Disease	SNOMED	253416000	Atrioventricular septal defect: atrial and ventricular components (disorder)
Congenital Disease	SNOMED	253417009	Atrioventricular septal defect - ventricular component (disorder)
Congenital Disease	SNOMED	253418004	Atrioventricular septal defect - ventricular component under superior bridging leaflet (disorder)
Congenital Disease	SNOMED	253419007	Atrioventricular septal defect with ventricular component under superior bridging leaflet with chords at crest ventricular septum (disorder)
Congenital Disease	SNOMED	253420001	Atrioventricular septal defect with ventricular component under superior bridging leaflet with chords to papillary muscle on right ventricular side septum (disorder)
Congenital Disease	SNOMED	253421002	Atrioventricular septal defect with ventricular component under free floating superior bridging leaflet and chords to papillary muscle at right ventricular free wall (disorder)
Congenital Disease	SNOMED	253422009	Atrioventricular septal defect - ventricular component under inferior bridging leaflet (disorder)
Congenital Disease	SNOMED	253423004	Malaligned atrial septum (disorder)
Congenital Disease	SNOMED	253425006	Double outlet right atrium (disorder)
Congenital Disease	SNOMED	253426007	Double outlet left atrium (disorder)
Congenital Disease	SNOMED	253427003	Abnormality of atrioventricular (non-mitral, non-tricuspid) valve (disorder)
Congenital Disease	SNOMED	253428008	Abnormality of common atrioventricular valve in atrioventricular septal defect (disorder)
Congenital Disease	SNOMED	253429000	Atresia of common atrioventricular valve (disorder)
Congenital Disease	SNOMED	253430005	Imperforate common atrioventricular valve (disorder)
Congenital Disease	SNOMED	253431009	Hypoplasia of common atrioventricular valve (disorder)
Congenital Disease	SNOMED	253432002	Dysplasia of common atrioventricular valve (disorder)
Congenital Disease	SNOMED	253433007	Regurgitation of common atrioventricular valve (disorder)
Congenital Disease	SNOMED	253434001	Common atrioventricular valve limited to one ventricle (disorder)
Congenital Disease	SNOMED	253435000	Dilatation of common atrioventricular valve annulus (disorder)
Congenital Disease	SNOMED	253436004	Common atrioventricular valve stenosis (disorder)
Congenital Disease	SNOMED	253437008	Common atrioventricular valve leaflet abnormality (disorder)

Congenital Disease	SNOMED	253438003	Common atrioventricular valve prolapse (disorder)
Congenital Disease	SNOMED	253439006	True cleft of common atrioventricular valve leaflet (disorder)
Congenital Disease	SNOMED	253440008	Accessory tissue on common atrioventricular valve leaflet (disorder)
Congenital Disease	SNOMED	253441007	Double orifice of common atrioventricular valve (disorder)
Congenital Disease	SNOMED	253442000	Triple orifice of left ventricular component of common atrioventricular valve (disorder)
Congenital Disease	SNOMED	253443005	Ebstein's anomaly of common atrioventricular valve (disorder)
Congenital Disease	SNOMED	253444004	Abnormality of common atrioventricular valve chordae tendinae (disorder)
Congenital Disease	SNOMED	253445003	Common atrioventricular valve chordae too short (disorder)
Congenital Disease	SNOMED	253446002	Common atrioventricular valve chordae too long (disorder)
Congenital Disease	SNOMED	253447006	Common atrioventricular valve chordae to outlet septum (disorder)
Congenital Disease	SNOMED	253448001	Arcade abnormality of common atrioventricular valve chordae (disorder)
Congenital Disease	SNOMED	253449009	Abnormality of common atrioventricular valve papillary muscle (disorder)
Congenital Disease	SNOMED	253450009	Parachute malformation of common atrioventricular valve (disorder)
Congenital Disease	SNOMED	253451008	Absent common atrioventricular valve papillary muscle (disorder)
Congenital Disease	SNOMED	253452001	Fused common atrioventricular valve papillary muscle (disorder)
Congenital Disease	SNOMED	253453006	Hypoplastic common atrioventricular valve papillary muscle (disorder)
Congenital Disease	SNOMED	253454000	Abnormality of right atrioventricular valve in double inlet ventricle (disorder)
Congenital Disease	SNOMED	253455004	Right atrioventricular valve atresia (disorder)
Congenital Disease	SNOMED	253456003	Imperforate right atrioventricular valve (disorder)
Congenital Disease	SNOMED	253457007	Right atrioventricular valve dysplasia (disorder)
Congenital Disease	SNOMED	253458002	Right atrioventricular valve hypoplasia (disorder)
Congenital Disease	SNOMED	253460000	Overriding right atrioventricular valve (disorder)
Congenital Disease	SNOMED	253463003	Straddling right atrioventricular valve (disorder)
Congenital Disease	SNOMED	253464009	Right atrioventricular valve leaflet abnormality (disorder)
Congenital Disease	SNOMED	253465005	Absent right atrioventricular valve leaflets (disorder)
Congenital Disease	SNOMED	253467002	Double orifice of right atrioventricular valve (disorder)
Congenital Disease	SNOMED	253468007	Ebstein's anomaly of right atrioventricular valve (disorder)
Congenital Disease	SNOMED	253470003	True cleft of right atrioventricular valve leaflet (disorder)
Congenital Disease	SNOMED	253471004	Accessory tissue on right atrioventricular valve leaflet (disorder)
Congenital Disease	SNOMED	253472006	Right atrioventricular valve leaflet dysplasia (disorder)
Congenital Disease	SNOMED	253473001	Abnormality of right atrioventricular valve chordae tendinae (disorder)
Congenital Disease	SNOMED	253474007	Right atrioventricular valve chordae too short (disorder)
Congenital Disease	SNOMED	253475008	Right atrioventricular valve chordae too long (disorder)
Congenital Disease	SNOMED	253476009	Right atrioventricular valve chordae to outlet septum (disorder)
Congenital Disease	SNOMED	253477000	Arcade abnormality of right atrioventricular valve chordae (disorder)
Congenital Disease	SNOMED	253478005	Congenital abnormality of right atrioventricular valve papillary muscle (disorder)
Congenital Disease	SNOMED	253479002	Congenital parachute malformation of right atrioventricular valve (disorder)
Congenital Disease	SNOMED	253480004	Congenital absence of right atrioventricular valve papillary muscle (disorder)
Congenital Disease	SNOMED	253481000	Congenital fusion of right atrioventricular valve papillary muscles (disorder)
Congenital Disease	SNOMED	253482007	Congenital hypoplastic right atrioventricular valve papillary muscle (disorder)
Congenital Disease	SNOMED	253483002	Abnormality of left atrioventricular valve in double inlet ventricle (disorder)
Congenital Disease	SNOMED	253484008	Left atrioventricular valve atresia (disorder)
Congenital Disease	SNOMED	253485009	Imperforate left atrioventricular valve (disorder)
Congenital Disease	SNOMED	253486005	Left atrioventricular valve dysplasia (disorder)
Congenital Disease	SNOMED	253487001	Left atrioventricular valve hypoplasia (disorder)
Congenital Disease	SNOMED	253489003	Overriding left atrioventricular valve (disorder)
Congenital Disease	SNOMED	253490007	Dilatation of left atrioventricular valve annulus (disorder)
Congenital Disease	SNOMED	253492004	Straddling left atrioventricular valve (disorder)
Congenital Disease	SNOMED	253493009	Left atrioventricular valve leaflet abnormality (disorder)
Congenital Disease	SNOMED	253494003	Absent left atrioventricular valve leaflets (disorder)
Congenital Disease	SNOMED	253495002	Double orifice of left atrioventricular valve (disorder)
Congenital Disease	SNOMED	253496001	Ebstein's anomaly of left atrioventricular valve (disorder)
Congenital Disease	SNOMED	253498000	True cleft of left atrioventricular valve leaflet (disorder)
Congenital Disease	SNOMED	253499008	Accessory tissue on left atrioventricular valve leaflet (disorder)
Congenital Disease	SNOMED	253500004	Left atrioventricular valve leaflet dysplasia (disorder)
Congenital Disease	SNOMED	253501000	Abnormality of left atrioventricular valve chordae tendinae (disorder)
Congenital Disease	SNOMED	253502007	Left atrioventricular valve chordae too short (disorder)
Congenital Disease	SNOMED	253503002	Left atrioventricular valve chordae too long (disorder)
Congenital Disease	SNOMED	253504008	Left atrioventricular valve chordae to outlet septum (disorder)
Congenital Disease	SNOMED	253505009	Arcade abnormality of left atrioventricular valve chordae (disorder)
Congenital Disease	SNOMED	253506005	Congenital abnormality of left atrioventricular valve papillary muscle (disorder)
Congenital Disease	SNOMED	253507001	Congenital parachute malformation of left atrioventricular valve (disorder)
Congenital Disease	SNOMED	253508006	Congenital absence of left atrioventricular valve papillary muscle (disorder)
Congenital Disease	SNOMED	253509003	Congenital fusion of left atrioventricular valve papillary muscles (disorder)
Congenital Disease	SNOMED	253510008	Congenital hypoplastic left atrioventricular valve papillary muscle (disorder)
Congenital Disease	SNOMED	253511007	Congenital abnormality of ventricles and ventricular septum (disorder)
Congenital Disease	SNOMED	253512000	Tetralogy of Fallot with pulmonary stenosis (disorder)
Congenital Disease	SNOMED	253513005	Tetralogy of Fallot with pulmonary atresia (disorder)
Congenital Disease	SNOMED	253514004	Dextraposition of aorta in Fallot's tetralogy (disorder)
Congenital Disease	SNOMED	253515003	Ventricular septal defect in Fallot's tetralogy (disorder)
Congenital Disease	SNOMED	253516002	Right ventricular abnormality (disorder)
Congenital Disease	SNOMED	253518001	Diffuse hypoplasia of right ventricle (disorder)

Congenital Disease	SNOMED	253519009	Hypoplasia of right ventricular inflow tract (disorder)
Congenital Disease	SNOMED	253520003	Hypoplasia of right ventricular outflow tract (disorder)
Congenital Disease	SNOMED	253521004	Hypoplasia of right ventricular outflow tract and trabecular area (disorder)
Congenital Disease	SNOMED	253524007	Two chambered right ventricle (disorder)
Congenital Disease	SNOMED	253525008	Congenital right ventricular diverticulum (disorder)
Congenital Disease	SNOMED	253527000	Congenital right ventricular aneurysm (disorder)
Congenital Disease	SNOMED	253528005	Arrhythmogenic right ventricular dysplasia (disorder)
Congenital Disease	SNOMED	253529002	Right ventricular outflow tract abnormality (disorder)
Congenital Disease	SNOMED	253530007	Right ventricular outflow tract obstruction (disorder)
Congenital Disease	SNOMED	253531006	Right ventricular outflow obstruction - localized (disorder)
Congenital Disease	SNOMED	253533009	Right ventricular outflow tract atresia (disorder)
Congenital Disease	SNOMED	253534003	Right ventricular outflow tract absent (disorder)
Congenital Disease	SNOMED	253535002	Left ventricular abnormality (disorder)
Congenital Disease	SNOMED	253536001	Left ventricular hypoplasia (disorder)
Congenital Disease	SNOMED	253537005	Diffuse hypoplasia of left ventricle (disorder)
Congenital Disease	SNOMED	253538000	Hypoplasia of left ventricular inflow tract (disorder)
Congenital Disease	SNOMED	253539008	Hypoplasia of left ventricular outflow tract (disorder)
Congenital Disease	SNOMED	253540005	Hypoplasia of left ventricular outflow tract and trabecular area (disorder)
Congenital Disease	SNOMED	253542002	Abnormal left ventricular muscle band (disorder)
Congenital Disease	SNOMED	253544001	Congenital left ventricular aneurysm (disorder)
Congenital Disease	SNOMED	253545000	Left ventricular outflow tract abnormality (disorder)
Congenital Disease	SNOMED	253546004	Left ventricular outflow tract obstruction (disorder)
Congenital Disease	SNOMED	253547008	Left ventricular outflow tract atresia (disorder)
Congenital Disease	SNOMED	253548003	Indeterminate ventricular outflow tract obstruction (disorder)
Congenital Disease	SNOMED	253549006	Ventricular septal abnormality (disorder)
Congenital Disease	SNOMED	253550006	Multiple ventricular septal defects (disorder)
Congenital Disease	SNOMED	253551005	Restrictive ventricular septal defect (disorder)
Congenital Disease	SNOMED	253552003	Perimembranous ventricular septal defect with extension to right ventricular inlet (disorder)
Congenital Disease	SNOMED	253553008	Perimembranous ventricular septal defect with extension to right ventricular trabecular component (disorder)
Congenital Disease	SNOMED	253554002	Perimembranous ventricular septal defect with extension to right ventricular outlet (disorder)
Congenital Disease	SNOMED	253555001	Perimembranous ventricular septal defect with extension to all right ventricular components (disorder)
Congenital Disease	SNOMED	253556000	Ventricular septal defect with malaligned outlet septum to right (disorder)
Congenital Disease	SNOMED	253559007	Ventricular septal defect with malaligned outlet septum to left (disorder)
Congenital Disease	SNOMED	253562005	Ventricular septal defect with absent outlet septum and overriding truncal valve (disorder)
Congenital Disease	SNOMED	253563000	Muscular ventricular septal defect in inlet septum (disorder)
Congenital Disease	SNOMED	253564006	Muscular ventricular septal defect in central trabecular septum (disorder)
Congenital Disease	SNOMED	253565007	Muscular ventricular septal defect in apical trabecular septum (disorder)
Congenital Disease	SNOMED	253566008	Muscular ventricular septal defect in marginal septum (disorder)
Congenital Disease	SNOMED	253567004	Muscular ventricular septal defect in outlet septum (disorder)
Congenital Disease	SNOMED	253568009	Doubly committed subarterial ventricular septal defect (disorder)
Congenital Disease	SNOMED	253569001	Doubly committed subarterial ventricular septal defect with membranous septum extension (disorder)
Congenital Disease	SNOMED	253570000	Doubly committed subarterial ventricular septal defect with muscular posterior inferior rim (disorder)
Congenital Disease	SNOMED	253571001	Giant ventricular septal defect (disorder)
Congenital Disease	SNOMED	253573003	Subpulmonary infundibulum (disorder)
Congenital Disease	SNOMED	253574009	Subaortic infundibulum (disorder)
Congenital Disease	SNOMED	253575005	Bilateral muscular infundibula (disorder)
Congenital Disease	SNOMED	253576006	Bilateral deficient infundibula (disorder)
Congenital Disease	SNOMED	253578007	Congenital abnormality of arterial valves (disorder)
Congenital Disease	SNOMED	253579004	Truncal valve abnormality (disorder)
Congenital Disease	SNOMED	253582009	Truncal valve regurgitation (disorder)
Congenital Disease	SNOMED	253584005	Accessory tissue on truncal valve cusp (disorder)
Congenital Disease	SNOMED	253585006	Pulmonary valve cusp hypoplasia (disorder)
Congenital Disease	SNOMED	253586007	Pulmonary valve ring hypoplasia (disorder)
Congenital Disease	SNOMED	253587003	Commissural fusion of pulmonary valve (disorder)
Congenital Disease	SNOMED	253588008	Pulmonary valve dysplasia (disorder)
Congenital Disease	SNOMED	253590009	Pulmonary atresia with intact ventricular septum (disorder)
Congenital Disease	SNOMED	253591008	Pulmonary atresia with ventricular septal defect (disorder)
Congenital Disease	SNOMED	253592001	Pulmonary valve atresia without ventricular outflow tract (disorder)
Congenital Disease	SNOMED	253593006	Imperforate pulmonary valve (disorder)
Congenital Disease	SNOMED	253594000	Muscular pulmonary atresia (disorder)
Congenital Disease	SNOMED	253596003	Absent pulmonary valve syndrome (disorder)
Congenital Disease	SNOMED	253597007	Accessory tissue on pulmonary valve cusp (disorder)
Congenital Disease	SNOMED	253598002	Unicuspid pulmonary valve (disorder)
Congenital Disease	SNOMED	253599005	Bicuspid pulmonary valve (disorder)
Congenital Disease	SNOMED	253600008	Quadricuspid pulmonary valve (disorder)
Congenital Disease	SNOMED	253602000	Commissural fusion of aortic valve (disorder)
Congenital Disease	SNOMED	253603005	Eccentric opening of aortic valve (disorder)
Congenital Disease	SNOMED	253604004	Aortic valve dysplasia (disorder)

Congenital Disease	SNOMED	253606002	Aortic valve cusp abnormality (disorder)
Congenital Disease	SNOMED	253607006	Hypoplasia of aortic valve cusp (disorder)
Congenital Disease	SNOMED	253608001	Accessory tissue on aortic valve cusp (disorder)
Congenital Disease	SNOMED	253609009	Abnormal number of aortic valve cusps (disorder)
Congenital Disease	SNOMED	253610004	Unicuspid aortic valve (disorder)
Congenital Disease	SNOMED	253611000	Quadricuspid aortic valve (disorder)
Congenital Disease	SNOMED	253612007	Aortic valve cusp prolapse (disorder)
Congenital Disease	SNOMED	253614008	Tubular hypoplasia of aorta (disorder)
Congenital Disease	SNOMED	253615009	Anomalies of the aorta excluding coarction (disorder)
Congenital Disease	SNOMED	253620009	Pulmonary trunk abnormality (disorder)
Congenital Disease	SNOMED	253621008	Pulmonary trunk stenosis (disorder)
Congenital Disease	SNOMED	253622001	Pulmonary trunk hypoplasia (disorder)
Congenital Disease	SNOMED	253623006	Pulmonary trunk atresia (disorder)
Congenital Disease	SNOMED	253624000	Pulmonary atresia with confluent pulmonary arteries (disorder)
Congenital Disease	SNOMED	253625004	Pulmonary atresia with absent pulmonary artery (disorder)
Congenital Disease	SNOMED	253627007	Pulmonary trunk absent with confluent pulmonary arteries (disorder)
Congenital Disease	SNOMED	253628002	Pulmonary trunk absent with non-confluent pulmonary arteries (disorder)
Congenital Disease	SNOMED	253629005	Pulmonary trunk absent with absent pulmonary artery (disorder)
Congenital Disease	SNOMED	253631001	Peripheral pulmonary artery stenosis (disorder)
Congenital Disease	SNOMED	253632008	Abnormal origin of right pulmonary artery (disorder)
Congenital Disease	SNOMED	253633003	Anomalous origin of right pulmonary artery from ductus arteriosus (disorder)
Congenital Disease	SNOMED	253634009	Anomalous origin of right pulmonary artery from ascending aorta (disorder)
Congenital Disease	SNOMED	253635005	Abnormal origin of left pulmonary artery (disorder)
Congenital Disease	SNOMED	253636006	Anomalous origin of left pulmonary artery from ductus arteriosus (disorder)
Congenital Disease	SNOMED	253637002	Anomalous origin of left pulmonary artery from ascending aorta (disorder)
Congenital Disease	SNOMED	253638007	Anomalous origin of left pulmonary artery from right pulmonary artery (disorder)
Congenital Disease	SNOMED	253640002	Ascending aorta abnormality (disorder)
Congenital Disease	SNOMED	253641003	Localized supravalvar aortic stenosis (disorder)
Congenital Disease	SNOMED	253642005	Diffuse supravalvar aortic stenosis (disorder)
Congenital Disease	SNOMED	253643000	Ascending aortic atresia (disorder)
Congenital Disease	SNOMED	253644006	Ascending aorta absent (disorder)
Congenital Disease	SNOMED	253646008	Congenital aneurysm of ascending aorta (disorder)
Congenital Disease	SNOMED	253647004	Sinus of Valsalva abnormality (disorder)
Congenital Disease	SNOMED	253648009	Sinus of Valsalva aneurysm with rupture (disorder)
Congenital Disease	SNOMED	253649001	Aortic tunnel (disorder)
Congenital Disease	SNOMED	253650001	Aorta to right ventricle tunnel (disorder)
Congenital Disease	SNOMED	253651002	Aortic arch and descending aorta abnormality (disorder)
Congenital Disease	SNOMED	253652009	Right descending aorta (disorder)
Congenital Disease	SNOMED	253653004	Left aortic arch and right descending aorta (disorder)
Congenital Disease	SNOMED	253654005	Right aortic arch and right descending aorta (disorder)
Congenital Disease	SNOMED	253655006	Right aortic arch and left descending aorta (disorder)
Congenital Disease	SNOMED	253656007	Aortic arch centrally descending (disorder)
Congenital Disease	SNOMED	253657003	Cervical aortic arch (disorder)
Congenital Disease	SNOMED	253658008	Persisting fifth aortic arch (disorder)
Congenital Disease	SNOMED	253660005	Double aortic arch with both patent (disorder)
Congenital Disease	SNOMED	253663007	Vascular ring with left aortic arch (disorder)
Congenital Disease	SNOMED	253664001	Vascular ring with right aortic arch (disorder)
Congenital Disease	SNOMED	253667008	Aberrant retroesophageal brachiocephalic artery (disorder)
Congenital Disease	SNOMED	253668003	Isolation of subclavian artery (disorder)
Congenital Disease	SNOMED	253669006	Ductus arteriosus from subclavian artery (disorder)
Congenital Disease	SNOMED	253670007	Ductus arteriosus from retroesophageal aortic diverticulum (disorder)
Congenital Disease	SNOMED	253673009	Preductal interruption of aorta (disorder)
Congenital Disease	SNOMED	253674003	Preductal hypoplasia of aorta (disorder)
Congenital Disease	SNOMED	253675002	Juxtaductal aortic coarctation (disorder)
Congenital Disease	SNOMED	253677005	Postductal interruption of aorta (disorder)
Congenital Disease	SNOMED	253678000	Thoracic aortic coarctation (disorder)
Congenital Disease	SNOMED	253679008	Abdominal aortic coarctation (disorder)
Congenital Disease	SNOMED	253680006	Postductal hypoplasia of aorta (disorder)
Congenital Disease	SNOMED	253681005	Interrupted aortic arch distal to left subclavian artery (disorder)
Congenital Disease	SNOMED	253682003	Interrupted aortic arch between left subclavian and left common carotid artery (disorder)
Congenital Disease	SNOMED	253683008	Interrupted aortic arch between left common carotid and brachiocephalic artery (disorder)
Congenital Disease	SNOMED	253687009	Abnormal origin of ductus arteriosus (disorder)
Congenital Disease	SNOMED	253688004	Collaterals to pulmonary arteries (disorder)
Congenital Disease	SNOMED	253689007	Major aortopulmonary collateral artery (disorder)
Congenital Disease	SNOMED	253690003	Systemic to pulmonary collateral artery (disorder)
Congenital Disease	SNOMED	253691004	Stenosis of systemic to pulmonary artery collateral artery (disorder)
Congenital Disease	SNOMED	253693001	Congenital abnormalities of thoracic aortic branches (disorder)
Congenital Disease	SNOMED	253695008	Abnormality of aortic arch branch (disorder)
Congenital Disease	SNOMED	253696009	Distal origin of brachiocephalic trunk (disorder)
Congenital Disease	SNOMED	253697000	Isolation of branch of aortic arch (disorder)
Congenital Disease	SNOMED	253698005	Isolation of brachiocephalic trunk (disorder)
Congenital Disease	SNOMED	253699002	Isolation of common carotid artery (disorder)

Congenital Disease	SNOMED	253700001	Variant coronary origin from aortic sinus (disorder)
Congenital Disease	SNOMED	253703004	Anomalous origin of coronary artery from non-facing sinus (disorder)
Congenital Disease	SNOMED	253704005	Anomalous origin of left anterior descending from right coronary artery (disorder)
Congenital Disease	SNOMED	253706007	Anomalous origin of coronary artery from pulmonary arterial tree (disorder)
Congenital Disease	SNOMED	253707003	Anomalous origin of coronary artery from right pulmonary artery (disorder)
Congenital Disease	SNOMED	253708008	Anomalous origin of coronary artery from left pulmonary artery (disorder)
Congenital Disease	SNOMED	253710005	Coronary orifice abnormally high (disorder)
Congenital Disease	SNOMED	253711009	Coronary orifice asymmetrical (disorder)
Congenital Disease	SNOMED	253712002	Dual coronary orifice (disorder)
Congenital Disease	SNOMED	253714001	Abnormal coronary artery course (disorder)
Congenital Disease	SNOMED	253715000	Intramural coronary artery course (disorder)
Congenital Disease	SNOMED	253716004	Aberrant course of left anterior descending coronary artery from right coronary artery crossing right ventricular outflow tract (disorder)
Congenital Disease	SNOMED	253717008	Coronary artery runs between aorta and pulmonary trunk (disorder)
Congenital Disease	SNOMED	253718003	Circumflex runs posterior to pulmonary trunk (disorder)
Congenital Disease	SNOMED	253719006	Circumflex runs posterior to aorta (disorder)
Congenital Disease	SNOMED	253720000	Congenital coronary arteriovenous fistula (disorder)
Congenital Disease	SNOMED	253726006	Right ventricle-dependent coronary circulation (disorder)
Congenital Disease	SNOMED	253727002	Variant dominance of coronary circulation (disorder)
Congenital Disease	SNOMED	253728007	Right dominant coronary system (disorder)
Congenital Disease	SNOMED	253729004	Left dominant coronary system (disorder)
Congenital Disease	SNOMED	253730009	Balanced coronary system (disorder)
Congenital Disease	SNOMED	253731008	Partial agenesis of pericardium (disorder)
Congenital Disease	SNOMED	253732001	Totally absent pericardium (disorder)
Congenital Disease	SNOMED	253734000	Pericardial diverticulum (disorder)
Congenital Disease	SNOMED	253736003	Laryngeal hypoplasia (disorder)
Congenital Disease	SNOMED	253737007	Congenital laryngomalacia (disorder)
Congenital Disease	SNOMED	253740007	Congenital malformation of trachea and bronchus (disorder)
Congenital Disease	SNOMED	253741006	Congenital tracheoesophageal cleft (disorder)
Congenital Disease	SNOMED	253742004	Primary congenital bronchomalacia (disorder)
Congenital Disease	SNOMED	253743009	Secondary congenital bronchomalacia (disorder)
Congenital Disease	SNOMED	253745002	Dysplasia of lung (disorder)
Congenital Disease	SNOMED	253746001	Ectopic bone and cartilage in lung (disorder)
Congenital Disease	SNOMED	253747005	Bronchopulmonary isomerism (disorder)
Congenital Disease	SNOMED	253748000	Anomaly of pleura (disorder)
Congenital Disease	SNOMED	253750008	Congenital malformation of tongue, mouth and pharynx (disorder)
Congenital Disease	SNOMED	253751007	Flat palate (disorder)
Congenital Disease	SNOMED	253752000	Lobulated tongue (disorder)
Congenital Disease	SNOMED	253755003	Congenital malformation of salivary glands and ducts (disorder)
Congenital Disease	SNOMED	253756002	Displacement of Wharton's duct (disorder)
Congenital Disease	SNOMED	253757006	Accessory salivary duct (disorder)
Congenital Disease	SNOMED	253758001	Congenital palatoesophageal incoordination (disorder)
Congenital Disease	SNOMED	253760004	Congenital esophageal ring (disorder)
Congenital Disease	SNOMED	253764008	Congenital absence, atresia and stenosis of small intestine (disorder)
Congenital Disease	SNOMED	253768006	Congenital dysmotility of small intestine (disorder)
Congenital Disease	SNOMED	253769003	Congenital stenosis of colon (disorder)
Congenital Disease	SNOMED	253770002	Congenital stenosis of appendix (disorder)
Congenital Disease	SNOMED	253771003	High anorectal malformation (disorder)
Congenital Disease	SNOMED	253772005	Low anorectal malformation (disorder)
Congenital Disease	SNOMED	253773000	Congenital fistula of rectum (disorder)
Congenital Disease	SNOMED	253774006	Congenital fistula of anus (disorder)
Congenital Disease	SNOMED	253778009	Congenital functional disorders of the colon (disorder)
Congenital Disease	SNOMED	253780003	Total colonic aganglionosis (disorder)
Congenital Disease	SNOMED	253781004	Megacystis, microcolon, hypoperistalsis syndrome (disorder)
Congenital Disease	SNOMED	253782006	Small left colon syndrome (disorder)
Congenital Disease	SNOMED	253783001	Neuronal intestinal dysplasia (disorder)
Congenital Disease	SNOMED	253784007	Hyperganglionosis (disorder)
Congenital Disease	SNOMED	253785008	Generalized congenital intestinal dysmotility (disorder)
Congenital Disease	SNOMED	253786009	Congenital volvulus (disorder)
Congenital Disease	SNOMED	253789002	Familial intestinal malrotation (disorder)
Congenital Disease	SNOMED	253791005	Congenital blind loop syndrome (disorder)
Congenital Disease	SNOMED	253792003	Congenital diverticulosis (disorder)
Congenital Disease	SNOMED	253793008	Congenital redundant rectal mucosa (disorder)
Congenital Disease	SNOMED	253794002	Congenital redundant colon (disorder)
Congenital Disease	SNOMED	253795001	Enteric duplication (disorder)
Congenital Disease	SNOMED	253796000	Jejunum duplex (disorder)
Congenital Disease	SNOMED	253797009	Ileum duplex (disorder)
Congenital Disease	SNOMED	253799007	Partial duplication of appendix (disorder)
Congenital Disease	SNOMED	253800006	Complete duplication of appendix (disorder)
Congenital Disease	SNOMED	253801005	Triplication of appendix (disorder)
Congenital Disease	SNOMED	253804002	Biliary anomalies (disorder)
Congenital Disease	SNOMED	253806000	Aplasia of gallbladder (disorder)
Congenital Disease	SNOMED	253808004	Congenital kink of cystic duct (disorder)
Congenital Disease	SNOMED	253810002	Liver hamartoma (disorder)

Congenital Disease	SNOMED	253811003	Abnormal liver lobulation (disorder)
Congenital Disease	SNOMED	253812005	Trilobular liver (disorder)
Congenital Disease	SNOMED	253813000	Ectopic liver (disorder)
Congenital Disease	SNOMED	253814006	Focal nodular hypoplasia of liver (disorder)
Congenital Disease	SNOMED	253815007	Pancreatic duct anomaly (disorder)
Congenital Disease	SNOMED	253817004	Congenital absent hemidiaphragm - bilateral (disorder)
Congenital Disease	SNOMED	253818009	Congenital malformation of anterior abdominal wall (disorder)
Congenital Disease	SNOMED	253822004	Congenital torsion of ovary (disorder)
Congenital Disease	SNOMED	253823009	Embryonic cyst of ovary (disorder)
Congenital Disease	SNOMED	253825002	Congenital hydrosalpinx (disorder)
Congenital Disease	SNOMED	253826001	Embryonic cyst of broad ligament (disorder)
Congenital Disease	SNOMED	253827005	Congenital malformation of uterus and cervix (disorder)
Congenital Disease	SNOMED	253828000	Mullerian aplasia (disorder)
Congenital Disease	SNOMED	253829008	Solid rudimentary uterus (disorder)
Congenital Disease	SNOMED	253832006	Hypoplasia of uterus and cervix (disorder)
Congenital Disease	SNOMED	253833001	Embryonic cyst of cervix (disorder)
Congenital Disease	SNOMED	253834007	Agenesis of lower vagina (disorder)
Congenital Disease	SNOMED	253835008	Atresia of lower vagina (disorder)
Congenital Disease	SNOMED	253836009	Hypoplasia of vagina (disorder)
Congenital Disease	SNOMED	253837000	Congenital urethrovaginal fistula (disorder)
Congenital Disease	SNOMED	253838005	Congenital absence of labium minor (disorder)
Congenital Disease	SNOMED	253839002	Congenital absence of labium major (disorder)
Congenital Disease	SNOMED	253842008	Congenital malformation of clitoris (disorder)
Congenital Disease	SNOMED	253843003	Hooded clitoris (disorder)
Congenital Disease	SNOMED	253844009	Duplication of clitoris (disorder)
Congenital Disease	SNOMED	253849004	Congenital buried penis (disorder)
Congenital Disease	SNOMED	253850004	Rotated penis (disorder)
Congenital Disease	SNOMED	253851000	Diphallus (disorder)
Congenital Disease	SNOMED	253852007	Webbed penis (disorder)
Congenital Disease	SNOMED	253855009	Short preputial frenulum (disorder)
Congenital Disease	SNOMED	253856005	Congenital parameatal cyst (disorder)
Congenital Disease	SNOMED	253857001	False hermaphrodite (disorder)
Congenital Disease	SNOMED	253859003	Congenital malformation of the urinary system (disorder)
Congenital Disease	SNOMED	253862000	Trifid kidney (disorder)
Congenital Disease	SNOMED	253864004	Familial hypoplastic, glomerulocystic kidney (disorder)
Congenital Disease	SNOMED	253865003	Crossed ectopia of kidney, without fusion (disorder)
Congenital Disease	SNOMED	253866002	Crossed ectopia of kidney with fusion anomaly (disorder)
Congenital Disease	SNOMED	253867006	Thoracic kidney (disorder)
Congenital Disease	SNOMED	253869009	Cake kidney (disorder)
Congenital Disease	SNOMED	253875000	Congenital calyceal diverticulum (disorder)
Congenital Disease	SNOMED	253876004	Fibrocystic renal degeneration (disorder)
Congenital Disease	SNOMED	253878003	Adult type polycystic kidney disease type 1 (disorder)
Congenital Disease	SNOMED	253879006	Adult type polycystic kidney disease type 2 (disorder)
Congenital Disease	SNOMED	253880009	Autosomal dominant polycystic kidney disease in childhood (disorder)
Congenital Disease	SNOMED	253881008	Cortical cystic disease (disorder)
Congenital Disease	SNOMED	253882001	Nephronophthisis - medullary cystic disease (disorder)
Congenital Disease	SNOMED	253884000	Medullary sponge kidney with nephrocalcinosis (disorder)
Congenital Disease	SNOMED	253885004	Medullary sponge kidney without nephrocalcinosis (disorder)
Congenital Disease	SNOMED	253886003	Congenital obstructive defect of renal pelvis (disorder)
Congenital Disease	SNOMED	253888002	Triplex ureter (disorder)
Congenital Disease	SNOMED	253890001	Crossed ectopic ureter (disorder)
Congenital Disease	SNOMED	253891002	Transcaval ureter (disorder)
Congenital Disease	SNOMED	253892009	Congenital diverticulum of ureter (disorder)
Congenital Disease	SNOMED	253893004	Congenital polyp of ureter (disorder)
Congenital Disease	SNOMED	253894005	Ectopic ureterocele (disorder)
Congenital Disease	SNOMED	253897003	Congenital vesicoureterorenal reflux, bilateral (disorder)
Congenital Disease	SNOMED	253899000	Urachal diverticulum (disorder)
Congenital Disease	SNOMED	253902002	Atresia of urethra (disorder)
Congenital Disease	SNOMED	253903007	Congenital short urethra (disorder)
Congenital Disease	SNOMED	253904001	Megacystis-megaureter syndrome (disorder)
Congenital Disease	SNOMED	253905000	Megalourethra (disorder)
Congenital Disease	SNOMED	253906004	Congenital urethral syringocele (disorder)
Congenital Disease	SNOMED	253907008	Congenital anterior urethral valve (disorder)
Congenital Disease	SNOMED	253909006	Congenital gastrointestinal-urinary tract fistula (disorder)
Congenital Disease	SNOMED	253916007	Aberrant muscle of the upper limb (disorder)
Congenital Disease	SNOMED	253917003	Failure of differentiation of bones of forearm (disorder)
Congenital Disease	SNOMED	253918008	Duplication of upper limb (disorder)
Congenital Disease	SNOMED	253919000	Duplication of whole upper limb (disorder)
Congenital Disease	SNOMED	253920006	Overgrowth of upper limb (disorder)
Congenital Disease	SNOMED	253924002	Acrosyndactyly of thumb (disorder)
Congenital Disease	SNOMED	253925001	Acrosyndactyly of the fingers (disorder)
Congenital Disease	SNOMED	253926000	Phocomelia of upper limb (disorder)
Congenital Disease	SNOMED	253929007	Hypoplasia of upper limb (disorder)
Congenital Disease	SNOMED	253932005	Brachysyndactyly of thumb (disorder)
Congenital Disease	SNOMED	253933000	Macrodactyly of thumb (disorder)

Congenital Disease	SNOMED	253934006	Hitch-hiker thumb (finding)
Congenital Disease	SNOMED	253936008	Hypoplasia of thumb (disorder)
Congenital Disease	SNOMED	253937004	Congenital abnormality of lower limb and pelvic girdle (disorder)
Congenital Disease	SNOMED	253939001	Duplication of whole lower limb (disorder)
Congenital Disease	SNOMED	253940004	Duplication of femur (disorder)
Congenital Disease	SNOMED	253941000	Duplication of tibia (disorder)
Congenital Disease	SNOMED	253942007	Duplication of fibula (disorder)
Congenital Disease	SNOMED	253943002	Duplication of tarsal bone (disorder)
Congenital Disease	SNOMED	253944008	Duplication of the whole foot (disorder)
Congenital Disease	SNOMED	253945009	Congenital overgrowth of partial lower limb (disorder)
Congenital Disease	SNOMED	253946005	Congenital undergrowth of partial lower limb (disorder)
Congenital Disease	SNOMED	253947001	Congenital leg bone bowing (disorder)
Congenital Disease	SNOMED	253953001	Dislocatable hip (disorder)
Congenital Disease	SNOMED	253954007	Subluxatable hip (disorder)
Congenital Disease	SNOMED	253955008	Congenital deformity of foot and ankle (disorder)
Congenital Disease	SNOMED	253958005	Congenital metatarsus valgus (disorder)
Congenital Disease	SNOMED	253959002	Hypoplasia of lower limb (disorder)
Congenital Disease	SNOMED	253961006	Transverse deficiency of toe (disorder)
Congenital Disease	SNOMED	253963009	Phocomelia of the lower limb (disorder)
Congenital Disease	SNOMED	253965002	Agenesis of multiple metatarsal bones (disorder)
Congenital Disease	SNOMED	253966001	Congenital abnormality of foot and toes (disorder)
Congenital Disease	SNOMED	253967005	Mesoaxial polydactyly of toe (disorder)
Congenital Disease	SNOMED	253968000	Simple syndactyly of toes second to fourth web (disorder)
Congenital Disease	SNOMED	25397008	Aqueduct of Sylvius anomaly (disorder)
Congenital Disease	SNOMED	253971008	Bifid digit (disorder)
Congenital Disease	SNOMED	253972001	Syndactyly of thumb (disorder)
Congenital Disease	SNOMED	253975004	Symphalangism (disorder)
Congenital Disease	SNOMED	253977007	Congenital sternomastoid tumor (disorder)
Congenital Disease	SNOMED	253978002	Dysmorphic features (finding)
Congenital Disease	SNOMED	253979005	Skull congenital deformities (disorder)
Congenital Disease	SNOMED	253980008	Defect of skull ossification (disorder)
Congenital Disease	SNOMED	253989009	Bilateral incomplete cleft lip and bilateral incomplete cleft of alveolar process of maxilla (disorder)
Congenital Disease	SNOMED	253993003	Cleft hard palate, central (disorder)
Congenital Disease	SNOMED	253994009	Cleft hard palate, bilateral (disorder)
Congenital Disease	SNOMED	253995005	Incomplete cleft hard and soft palate (disorder)
Congenital Disease	SNOMED	253996006	Complete cleft hard and soft palate (disorder)
Congenital Disease	SNOMED	253997002	Cleft of soft palate (disorder)
Congenital Disease	SNOMED	254000002	Cleft soft palate, bilateral (disorder)
Congenital Disease	SNOMED	254001003	Complete cleft of soft palate (disorder)
Congenital Disease	SNOMED	254002005	Incomplete cleft of soft palate (disorder)
Congenital Disease	SNOMED	254003000	Occult submucous cleft palate (disorder)
Congenital Disease	SNOMED	254004006	Midline facial cleft - Tessier cleft 0 (disorder)
Congenital Disease	SNOMED	254005007	Midline facial cleft - Tessier cleft 14 (disorder)
Congenital Disease	SNOMED	254006008	Midline facial cleft - Tessier cleft 30 (disorder)
Congenital Disease	SNOMED	254007004	Paramedian facial cleft - Tessier cleft 1 (disorder)
Congenital Disease	SNOMED	254008009	Paramedian facial cleft - Tessier cleft 2 (disorder)
Congenital Disease	SNOMED	254009001	Paramedian facial cleft - Tessier cleft 3 (disorder)
Congenital Disease	SNOMED	254010006	Supraorbital facial cleft - Tessier cleft 8 (disorder)
Congenital Disease	SNOMED	254011005	Supraorbital facial cleft - Tessier cleft 9 (disorder)
Congenital Disease	SNOMED	254012003	Supraorbital facial cleft - Tessier cleft 10 (disorder)
Congenital Disease	SNOMED	254013008	Supraorbital facial cleft - Tessier cleft 11 (disorder)
Congenital Disease	SNOMED	254014002	Supraorbital facial cleft - Tessier cleft 12 (disorder)
Congenital Disease	SNOMED	254015001	Supraorbital facial cleft - Tessier cleft 13 (disorder)
Congenital Disease	SNOMED	254016000	Infraorbital facial cleft - Tessier cleft 4 (disorder)
Congenital Disease	SNOMED	254017009	Infraorbital facial cleft - Tessier cleft 5 (disorder)
Congenital Disease	SNOMED	254018004	Infraorbital facial cleft - Tessier cleft 6 (disorder)
Congenital Disease	SNOMED	254019007	Infraorbital facial cleft - Tessier cleft 7 (disorder)
Congenital Disease	SNOMED	254020001	Unicoronal craniosynostosis (disorder)
Congenital Disease	SNOMED	254021002	Bicoronal craniosynostosis (disorder)
Congenital Disease	SNOMED	254022009	Cloverleaf skull syndrome (disorder)
Congenital Disease	SNOMED	254023004	Congenital abnormality of skull shape (disorder)
Congenital Disease	SNOMED	254024005	Postural plagiocephaly (disorder)
Congenital Disease	SNOMED	254025006	Hemifacial microsomia (disorder)
Congenital Disease	SNOMED	254026007	Craniofacial microsomia (disorder)
Congenital Disease	SNOMED	254027003	Bilateral craniofacial microsomia (disorder)
Congenital Disease	SNOMED	254033007	Congenital deformity of lumbosacral region (disorder)
Congenital Disease	SNOMED	254034001	Congenital deformity of lumbosacral joint (disorder)
Congenital Disease	SNOMED	254038003	Congenital absence of spine (disorder)
Congenital Disease	SNOMED	254039006	Congenital lumbosacral fusion (disorder)
Congenital Disease	SNOMED	254040008	Congenital sacrococcygeal anomaly (disorder)
Congenital Disease	SNOMED	254041007	Congenital malformation of sternum (disorder)
Congenital Disease	SNOMED	254043005	Defects of the tubular (and flat) bones and/or axial skeleton (disorder)
Congenital Disease	SNOMED	254044004	Multiple congenital exostosis (disorder)
Congenital Disease	SNOMED	254045003	Spondylodysplasia (disorder)

Congenital Disease	SNOMED	254046002	Spondylodysplasia, San Diego type (disorder)
Congenital Disease	SNOMED	254047006	Spondylodysplasia, Torrance type (disorder)
Congenital Disease	SNOMED	254048001	Spondylodysplasia, Luton type (disorder)
Congenital Disease	SNOMED	254049009	Schneckenbecken dysplasia (disorder)
Congenital Disease	SNOMED	254050009	Short rib dysplasia (disorder)
Congenital Disease	SNOMED	254051008	Type III short rib polydactyly syndrome (disorder)
Congenital Disease	SNOMED	254052001	Type IV short rib polydactyly syndrome (disorder)
Congenital Disease	SNOMED	254054000	Boomerang dysplasia (disorder)
Congenital Disease	SNOMED	254055004	Atelosteogenesis type 2 (disorder)
Congenital Disease	SNOMED	254058002	Pseudodiastrophic dysplasia (disorder)
Congenital Disease	SNOMED	254059005	Kniest-Stickler dysplasia (disorder)
Congenital Disease	SNOMED	254060000	Otospondylomegaepiphyseal dysplasia (disorder)
Congenital Disease	SNOMED	254061001	Achondrogenesis, type II (disorder)
Congenital Disease	SNOMED	254062008	Spondyloepimetaphyseal disorder (disorder)
Congenital Disease	SNOMED	254064009	Namaqualand hip dysplasia (disorder)
Congenital Disease	SNOMED	254065005	Progressive pseudorheumatoid dysplasia (disorder)
Congenital Disease	SNOMED	254066006	Wolcott-Rallison dysplasia (disorder)
Congenital Disease	SNOMED	254067002	Immuno-osseous dysplasia (disorder)
Congenital Disease	SNOMED	254068007	Opsismodysplasia (disorder)
Congenital Disease	SNOMED	254078005	Spondylometaphyseal dysplasia - Sutcliffe type (disorder)
Congenital Disease	SNOMED	254079002	Spondyloenchondrodysplasia (disorder)
Congenital Disease	SNOMED	254080004	Epiphyseal dysplasia (disorder)
Congenital Disease	SNOMED	254082007	Chondrodysplasia punctata, X-linked recessive type (disorder)
Congenital Disease	SNOMED	254083002	Chondrodysplasia punctata, tibia-metacarpal type (disorder)
Congenital Disease	SNOMED	254084008	Metaphyseal chondrodysplasia, Spahr type (disorder)
Congenital Disease	SNOMED	254085009	Metaphyseal anadysplasia (disorder)
Congenital Disease	SNOMED	254088006	Brachyolmia (disorder)
Congenital Disease	SNOMED	254090007	Acromicric dysplasia (disorder)
Congenital Disease	SNOMED	254091006	Trichorhinophalangeal dysplasia type I (disorder)
Congenital Disease	SNOMED	254092004	Saldino-Mainzer dysplasia (disorder)
Congenital Disease	SNOMED	254093009	Cranioectodermal dysplasia (disorder)
Congenital Disease	SNOMED	254094003	Dysplasias with significant membranous bone involvement (disorder)
Congenital Disease	SNOMED	254096001	Kyphomelic dysplasia (disorder)
Congenital Disease	SNOMED	254097005	Stuve-Wiedemann dysplasia (disorder)
Congenital Disease	SNOMED	254098000	Multiple dislocations with dysplasia (disorder)
Congenital Disease	SNOMED	254099008	Desbuquois syndrome (disorder)
Congenital Disease	SNOMED	254100000	Spondyloepimetaphyseal dysplasia with joint laxity (disorder)
Congenital Disease	SNOMED	254101001	Osteodysplastic primordial dwarfism (disorder)
Congenital Disease	SNOMED	254102008	Osteodysplastic primordial dwarfism, type 1 (disorder)
Congenital Disease	SNOMED	254104009	Dysplasia with decreased bone density (disorder)
Congenital Disease	SNOMED	254110009	Osteogenesis imperfecta type IIA (disorder)
Congenital Disease	SNOMED	254111008	Osteogenesis imperfecta type IIB (disorder)
Congenital Disease	SNOMED	254112001	Osteoporosis with pseudoglioma (disorder)
Congenital Disease	SNOMED	254113006	Bruck syndrome (disorder)
Congenital Disease	SNOMED	254114000	Singleton-Merten syndrome (disorder)
Congenital Disease	SNOMED	254116003	Geroderma osteodysplastica (disorder)
Congenital Disease	SNOMED	254117007	Dysplasia with defective mineralization (disorder)
Congenital Disease	SNOMED	254120004	Dysplasia with increased bone density (disorder)
Congenital Disease	SNOMED	254121000	Osteopetrosis - intermediate type (disorder)
Congenital Disease	SNOMED	254122007	Osteopetrosis with renal tubular acidosis (disorder)
Congenital Disease	SNOMED	254123002	Dysosteosclerosis (disorder)
Congenital Disease	SNOMED	254124008	Osteosclerosis - Stanescu type (disorder)
Congenital Disease	SNOMED	254125009	Axial osteosclerosis (disorder)
Congenital Disease	SNOMED	254129003	Osteopathia striata with cranial sclerosis (disorder)
Congenital Disease	SNOMED	254131007	Worth disease (disorder)
Congenital Disease	SNOMED	254132000	Endosteal hyperostoses with cerebellar hypoplasia (disorder)
Congenital Disease	SNOMED	254134004	Craniometaphyseal dysplasia - severe type (disorder)
Congenital Disease	SNOMED	254135003	Craniometaphyseal dysplasia - mild type (disorder)
Congenital Disease	SNOMED	254137006	Oculodento-osseous dysplasia (disorder)
Congenital Disease	SNOMED	254138001	Oculodento-osseous dysplasia - severe type (disorder)
Congenital Disease	SNOMED	254139009	Oculodento-osseous dysplasia - mild type (disorder)
Congenital Disease	SNOMED	254140006	Disorganized development of cartilaginous and fibrous components of the skeleton (disorder)
Congenital Disease	SNOMED	254142003	Pseudochondroplasia (disorder)
Congenital Disease	SNOMED	254147009	Idiopathic osteolyses (disorder)
Congenital Disease	SNOMED	254148004	Hereditary acroosteolysis (disorder)
Congenital Disease	SNOMED	254149007	Carpal-tarsal osteolysis with nephropathy (disorder)
Congenital Disease	SNOMED	254150007	Francois syndrome (disorder)
Congenital Disease	SNOMED	254151006	Winchester syndrome (disorder)
Congenital Disease	SNOMED	254153009	Familial expansile osteolysis (disorder)
Congenital Disease	SNOMED	254154003	Congenital ectodermal defect (disorder)
Congenital Disease	SNOMED	254156001	Congenital ichthyosiform erythroderma (disorder)
Congenital Disease	SNOMED	254157005	Ichthyosis vulgaris (disorder)
Congenital Disease	SNOMED	254158000	Autosomal dominant ichthyosis vulgaris (disorder)
Congenital Disease	SNOMED	254159008	Severe ichthyoses (disorder)

Congenital Disease	SNOMED	254161004	Erythrodermic lamellar ichthyosis (disorder)
Congenital Disease	SNOMED	254163001	Non-erythrodermic lamellar ichthyosis (disorder)
Congenital Disease	SNOMED	254164007	Autosomal dominant lamellar ichthyosis (disorder)
Congenital Disease	SNOMED	254167000	Bullous ichthyosiform erythroderma (disorder)
Congenital Disease	SNOMED	254168005	Localized bullous ichthyosiform erythroderma (disorder)
Congenital Disease	SNOMED	254169002	Ichthyosis bullosa of Siemens (disorder)
Congenital Disease	SNOMED	254170001	Ichthyosis hystrix of Curth-Macklin (disorder)
Congenital Disease	SNOMED	254171002	Ichthyosis hystrix Bäfverstedt type (disorder)
Congenital Disease	SNOMED	254176007	Progressive recessive dystrophic epidermolysis bullosa (disorder)
Congenital Disease	SNOMED	254177003	Epidermolysis bullosa simplex with hypodontia (disorder)
Congenital Disease	SNOMED	254179000	Epidermolysis bullosa simplex herpetiformis (disorder)
Congenital Disease	SNOMED	254180002	Epidermolysis bullosa simplex with mottled pigmentation (disorder)
Congenital Disease	SNOMED	254181003	Epidermolysis simplex superficialis (disorder)
Congenital Disease	SNOMED	254183000	Lethal autosomal recessive epidermolysis bullosa simplex (disorder)
Congenital Disease	SNOMED	254185007	Dystrophic epidermolysis bullosa (disorder)
Congenital Disease	SNOMED	254186008	Localized dystrophic epidermolysis bullosa (disorder)
Congenital Disease	SNOMED	254187004	Localized recessive dystrophic epidermolysis bullosa (disorder)
Congenital Disease	SNOMED	254188009	Generalized dystrophic epidermolysis bullosa (disorder)
Congenital Disease	SNOMED	254189001	Generalized recessive dystrophic epidermolysis bullosa mitis (disorder)
Congenital Disease	SNOMED	254191009	Localized junctional epidermolysis bullosa (disorder)
Congenital Disease	SNOMED	254192002	Inverse junctional epidermolysis bullosa (disorder)
Congenital Disease	SNOMED	254193007	Progressive junctional epidermolysis bullosa (neurotrophic) (disorder)
Congenital Disease	SNOMED	254194001	Generalized junctional epidermolysis bullosa (disorder)
Congenital Disease	SNOMED	254196004	Junctional epidermolysis bullosa mitis (disorder)
Congenital Disease	SNOMED	254197008	Cicatricial junctional epidermolysis bullosa (disorder)
Congenital Disease	SNOMED	254199006	Hereditary lymphedema (disorder)
Congenital Disease	SNOMED	254206003	Congenital vascular nevus (disorder)
Congenital Disease	SNOMED	254207007	Reticulate vascular nevus (disorder)
Congenital Disease	SNOMED	254208002	Nevus sanguineous (disorder)
Congenital Disease	SNOMED	254211001	Salmon patch nevus (disorder)
Congenital Disease	SNOMED	254214009	Inherited disorder of keratinization (disorder)
Congenital Disease	SNOMED	254215005	Erythrokeratoderma (disorder)
Congenital Disease	SNOMED	254216006	Hereditary erythrokeratolysis (disorder)
Congenital Disease	SNOMED	254218007	Hereditary follicular keratoses (disorder)
Congenital Disease	SNOMED	254220005	Inherited cutis laxa (disorder)
Congenital Disease	SNOMED	254221009	Neonatal cutis laxa with marfanoid phenotype (disorder)
Congenital Disease	SNOMED	254222002	Cutis laxa, recessive, type I (disorder)
Congenital Disease	SNOMED	254223007	Cutis laxa, recessive, type II (disorder)
Congenital Disease	SNOMED	254225000	Congenital alopecia with keratin cysts (disorder)
Congenital Disease	SNOMED	254226004	Hypotrichosis with keratosis pilaris (disorder)
Congenital Disease	SNOMED	254227008	Hypotrichosis with keratosis pilaris and lentiginosis (disorder)
Congenital Disease	SNOMED	254230001	Uncombable hair syndrome (disorder)
Congenital Disease	SNOMED	254231002	Congenital wooly hair (disorder)
Congenital Disease	SNOMED	254233004	Congenital ringed hair (disorder)
Congenital Disease	SNOMED	254234005	Marie Unna syndrome (disorder)
Congenital Disease	SNOMED	254236007	Congenital enlarged nails (disorder)
Congenital Disease	SNOMED	254237003	Aplasia of skin (disorder)
Congenital Disease	SNOMED	254239000	Congenital retraction of nipple (finding)
Congenital Disease	SNOMED	254241004	Segmental neurofibromatosis (disorder)
Congenital Disease	SNOMED	254242006	Diffuse neurofibroma (disorder)
Congenital Disease	SNOMED	254243001	Ash leaf spot, tuberous sclerosis (disorder)
Congenital Disease	SNOMED	254246009	Congenital malformation caused by cytotoxic agents (disorder)
Congenital Disease	SNOMED	254249002	Fetal carbamazepine syndrome (disorder)
Congenital Disease	SNOMED	254250002	Fetal cocaine syndrome (disorder)
Congenital Disease	SNOMED	254254006	Fetal toluene syndrome (disorder)
Congenital Disease	SNOMED	254255007	Congenital malformation of anterior pituitary (disorder)
Congenital Disease	SNOMED	254256008	Congenital malformation of posterior pituitary (disorder)
Congenital Disease	SNOMED	254259001	Absence of sex chromosome (disorder)
Congenital Disease	SNOMED	254261005	Pseudotrisomy 18 (disorder)
Congenital Disease	SNOMED	254262003	Unbalanced translocation and insertion (disorder)
Congenital Disease	SNOMED	254264002	Partial trisomy 21 in Down's syndrome (disorder)
Congenital Disease	SNOMED	254266000	Partial trisomy 18 in Edward's syndrome (disorder)
Congenital Disease	SNOMED	254268004	Partial trisomy 13 in Patau's syndrome (disorder)
Congenital Disease	SNOMED	254269007	Whole chromosome trisomy meiotic nondisjunction (disorder)
Congenital Disease	SNOMED	254270008	Whole chromosome trisomy - mitotic nondisjunction mosaicism (disorder)
Congenital Disease	SNOMED	254272000	Triploidy and polyploidy (disorder)
Congenital Disease	SNOMED	254273005	Autosomal deletion - mosaicism (disorder)
Congenital Disease	SNOMED	254274004	Deletion of part of autosome (disorder)
Congenital Disease	SNOMED	254275003	Balanced rearrangement and structural marker (disorder)
Congenital Disease	SNOMED	254276002	Balanced translocation and insertion in normal individual (disorder)
Congenital Disease	SNOMED	254277006	Sex chromosome abnormality - female phenotype (disorder)
Congenital Disease	SNOMED	254280007	Turner's phenotype, partial X deletion karyotype (disorder)
Congenital Disease	SNOMED	254281006	Turner's phenotype - ring chromosome karyotype (disorder)
Congenital Disease	SNOMED	254282004	Female with more than three X chromosomes (disorder)
Congenital Disease	SNOMED	254283009	Mosaicism - lines with various numbers of X chromosomes (disorder)

Congenital Disease	SNOMED	254284003	Sex chromosome abnormality - male phenotype (disorder)
Congenital Disease	SNOMED	254285002	Male with structurally abnormal sex chromosome (disorder)
Congenital Disease	SNOMED	254286001	Male with sex chromosome mosaicism (disorder)
Congenital Disease	SNOMED	254287005	FRAXA (disorder)
Congenital Disease	SNOMED	254288000	FRAXE (disorder)
Congenital Disease	SNOMED	254594009	Hamartoma of intestine (disorder)
Congenital Disease	SNOMED	254705003	Basal cell nevus with comedones (disorder)
Congenital Disease	SNOMED	254760005	Neuromuscular hamartoma (disorder)
Congenital Disease	SNOMED	254774003	Cobb's syndrome (disorder)
Congenital Disease	SNOMED	254775002	Bregeat's syndrome (disorder)
Congenital Disease	SNOMED	254778000	Congenital livedo reticularis (disorder)
Congenital Disease	SNOMED	254780006	Arteriovenous malformation of skin (disorder)
Congenital Disease	SNOMED	254782003	Multiple progressive hemangiomata (disorder)
Congenital Disease	SNOMED	254784002	Blue rubber bleb nevus (disorder)
Congenital Disease	SNOMED	254793001	Cavernous lymphangioma of skin (disorder)
Congenital Disease	SNOMED	255190002	Ulcero - mutilating hemangiomatosis (disorder)
Congenital Disease	SNOMED	255581000119100	Simple craniosynostosis (disorder)
Congenital Disease	SNOMED	25559009	Congenital absence of left pulmonary artery (disorder)
Congenital Disease	SNOMED	2560006	Syndactyly of fingers with fusion of bones (disorder)
Congenital Disease	SNOMED	25606004	Ehlers-Danlos syndrome, hydroxylysine-deficient (disorder)
Congenital Disease	SNOMED	25617003	Congenital duodenal obstruction due to malrotation of intestine (disorder)
Congenital Disease	SNOMED	25642007	Congenital fissure of sternum (disorder)
Congenital Disease	SNOMED	25784009	Xeroderma pigmentosum, group C (disorder)
Congenital Disease	SNOMED	25896009	Congenital atresia of ileum (disorder)
Congenital Disease	SNOMED	2593002	Dubowitz's syndrome (disorder)
Congenital Disease	SNOMED	25971000119100	Vascular birthmark (disorder)
Congenital Disease	SNOMED	25972003	Congenital absence of rectum (disorder)
Congenital Disease	SNOMED	26061003	Congenital absence of external auditory canal (disorder)
Congenital Disease	SNOMED	26098002	Microcornea (disorder)
Congenital Disease	SNOMED	261407007	Band of Ladd (disorder)
Congenital Disease	SNOMED	26146002	Complete transposition of great vessels (disorder)
Congenital Disease	SNOMED	261482004	Spinal arteriovenous malformation (disorder)
Congenital Disease	SNOMED	26155004	Disorder: ectopic bone tissue, congenital (disorder)
Congenital Disease	SNOMED	26179002	Congenital atresia of esophagus (disorder)
Congenital Disease	SNOMED	26201005	Aortic left ventricular tunnel (disorder)
Congenital Disease	SNOMED	262263007	Vitellointestinal band (disorder)
Congenital Disease	SNOMED	2625009	Senter syndrome (disorder)
Congenital Disease	SNOMED	26287007	Uterus incudiformis (disorder)
Congenital Disease	SNOMED	26315009	Congenital obstruction of small intestine (disorder)
Congenital Disease	SNOMED	263944006	Anterolateral muscle band (disorder)
Congenital Disease	SNOMED	263961009	Bilateral isomeric atria (disorder)
Congenital Disease	SNOMED	263993001	Epoophoron (disorder)
Congenital Disease	SNOMED	26408002	Congenital tracheocele (disorder)
Congenital Disease	SNOMED	264086008	Malaligned outlet septum (disorder)
Congenital Disease	SNOMED	264162009	Posteromedial muscle band (disorder)
Congenital Disease	SNOMED	264195003	Simonart's band (disorder)
Congenital Disease	SNOMED	264258007	Persistent vertical vein (disorder)
Congenital Disease	SNOMED	26445008	Cat eye syndrome (disorder)
Congenital Disease	SNOMED	264467005	False tendon - heart (disorder)
Congenital Disease	SNOMED	264480008	Persistent Gartner's duct (disorder)
Congenital Disease	SNOMED	264491001	Mullerian remnant (disorder)
Congenital Disease	SNOMED	264571006	Septoparietal trabeculations (disorder)
Congenital Disease	SNOMED	26480007	11p partial trisomy syndrome (disorder)
Congenital Disease	SNOMED	264917009	Upper moiety ureter of duplex kidney (disorder)
Congenital Disease	SNOMED	264918004	Lower moiety ureter of duplex kidney (disorder)
Congenital Disease	SNOMED	26568002	Faun tail syndrome (disorder)
Congenital Disease	SNOMED	265798000	Congenital complete absence of lower limb (disorder)
Congenital Disease	SNOMED	26590002	Congenital ectropion (disorder)
Congenital Disease	SNOMED	26595007	Congenital absence of part of brain (disorder)
Congenital Disease	SNOMED	266673001	Ectopic neuronal tissue (disorder)
Congenital Disease	SNOMED	26718008	Robinson nail dystrophy-deafness syndrome (disorder)
Congenital Disease	SNOMED	26730002	Persistent thyroglossal duct (disorder)
Congenital Disease	SNOMED	267372009	Congenital non bullous ichthyosiform erythroderma (disorder)
Congenital Disease	SNOMED	26780008	Coarctation of pulmonary artery (disorder)
Congenital Disease	SNOMED	268143001	Spina bifida with hydrocephalus - open (disorder)
Congenital Disease	SNOMED	268146009	Spina bifida without hydrocephalus - open (disorder)
Congenital Disease	SNOMED	268158009	Megalocornea (disorder)
Congenital Disease	SNOMED	268160006	Congenital corneal opacity (disorder)
Congenital Disease	SNOMED	268163008	Congenital ptosis (disorder)
Congenital Disease	SNOMED	268166000	Ear auricle and external auditory canal absent (disorder)
Congenital Disease	SNOMED	268172000	Congenital malposition of ear (disorder)
Congenital Disease	SNOMED	268173005	Branchial cleft sinus and fistula (disorder)
Congenital Disease	SNOMED	268174004	Bulbus cordis and cardiac septal closure anomalies (disorder)
Congenital Disease	SNOMED	268180007	Right hypoplastic heart syndrome (disorder)
Congenital Disease	SNOMED	268184003	Hypoplasia of aorta (disorder)

Congenital Disease	SNOMED	268187005	Congenital pulmonary artery aneurysm (disorder)
Congenital Disease	SNOMED	268190004	Congenital peripheral aneurysm (disorder)
Congenital Disease	SNOMED	268195009	Ectopic tissue in lung (disorder)
Congenital Disease	SNOMED	268197001	Central incomplete cleft palate (disorder)
Congenital Disease	SNOMED	268201001	Esophageal atresia, stenosis and fistula (disorder)
Congenital Disease	SNOMED	268205005	Atresia and stenosis of large intestine, rectum and anal canal (disorder)
Congenital Disease	SNOMED	268209004	Idiopathic congenital megacolon (disorder)
Congenital Disease	SNOMED	268213006	Congenital abnormality of liver and/or biliary tract (disorder)
Congenital Disease	SNOMED	268219005	Embryonic cyst of fallopian tube and broad ligament (disorder)
Congenital Disease	SNOMED	268223002	Congenital fusion of labia (disorder)
Congenital Disease	SNOMED	268228006	Undescended testes - bilateral (disorder)
Congenital Disease	SNOMED	268232000	Bilateral renal hypoplasia (disorder)
Congenital Disease	SNOMED	268234004	Fibrocystic kidney disease (disorder)
Congenital Disease	SNOMED	268236002	Congenital bladder neck stenosis (disorder)
Congenital Disease	SNOMED	268239009	Congenital abnormality of skull and face bones (disorder)
Congenital Disease	SNOMED	268240006	Congenital torticollis (disorder)
Congenital Disease	SNOMED	268243008	Genu recurvatum and long leg bone bowing (disorder)
Congenital Disease	SNOMED	268245001	Harlequin fetus (disorder)
Congenital Disease	SNOMED	26825009	Cutis marmorata (finding)
Congenital Disease	SNOMED	268251006	Simple syndactyly of fingers (disorder)
Congenital Disease	SNOMED	268262006	Acrocephalosyndactyly (disorder)
Congenital Disease	SNOMED	268264007	Constriction ring of upper limb with lymphedema (disorder)
Congenital Disease	SNOMED	268265008	Congenital anomalies of elbow and upper arm (disorder)
Congenital Disease	SNOMED	268267000	Oculomandibular dysostosis (disorder)
Congenital Disease	SNOMED	268274005	Enchondromatosis (disorder)
Congenital Disease	SNOMED	268276007	Congenital exostosis (disorder)
Congenital Disease	SNOMED	268282005	Ichthyosiform erythroderma (disorder)
Congenital Disease	SNOMED	268284006	Vascular hamartomas (disorder)
Congenital Disease	SNOMED	268288009	Congenital generalized alopecia (disorder)
Congenital Disease	SNOMED	268290005	Hypoplasia of nipple (disorder)
Congenital Disease	SNOMED	268302006	Aberrant thyroid gland (disorder)
Congenital Disease	SNOMED	26865008	Congenital absence of superior vena cava (disorder)
Congenital Disease	SNOMED	26885007	Cervical auricle (disorder)
Congenital Disease	SNOMED	2689001	Dominant dystrophic epidermolysis bullosa with absence of skin (disorder)
Congenital Disease	SNOMED	27025001	Autosomal recessive hypohidrotic ectodermal dysplasia syndrome (disorder)
Congenital Disease	SNOMED	270510008	Anomalous coronary artery communication (disorder)
Congenital Disease	SNOMED	270513005	Central complete cleft palate (disorder)
Congenital Disease	SNOMED	270516002	Congenital macroglossia (disorder)
Congenital Disease	SNOMED	270517006	Triple kidney with triple pelvis (disorder)
Congenital Disease	SNOMED	270519009	Localized congenital skull defect (disorder)
Congenital Disease	SNOMED	270520003	Whole chromosome monosomy - mitotic nondisjunction mosaicism (disorder)
Congenital Disease	SNOMED	270521004	Trisomy and partial trisomy of autosome (disorder)
Congenital Disease	SNOMED	270889005	Deletion of long arm of chromosome 18 (disorder)
Congenital Disease	SNOMED	270890001	Deletion of short arm of chromosome 18 (disorder)
Congenital Disease	SNOMED	270910001	Medullary cystic disease, adult type (disorder)
Congenital Disease	SNOMED	270963009	Congenital absence of thumb (disorder)
Congenital Disease	SNOMED	271015004	Congenital instability of hip joint (disorder)
Congenital Disease	SNOMED	271018002	Congenital absence of tendon (disorder)
Congenital Disease	SNOMED	271020004	Congenital absence of breast with absent nipple (disorder)
Congenital Disease	SNOMED	271387005	Congenital enlarged kidney (disorder)
Congenital Disease	SNOMED	271431003	Congenital stenosis of nasolacrimal duct (disorder)
Congenital Disease	SNOMED	271432005	Congenital renal artery stenosis (disorder)
Congenital Disease	SNOMED	271542005	Aberrant intrinsic muscles of hand (disorder)
Congenital Disease	SNOMED	271573009	Congenital abnormality of thoracic aorta and pulmonary arteries (disorder)
Congenital Disease	SNOMED	271574003	Congenital partial fusion of spine (disorder)
Congenital Disease	SNOMED	271630007	Trachea displaced to left (disorder)
Congenital Disease	SNOMED	27173008	Tarsal coalitions (disorder)
Congenital Disease	SNOMED	27183007	Anomaly of chromosome pair 14 (disorder)
Congenital Disease	SNOMED	271961001	Congenital malformation of ovaries and fallopian tubes (disorder)
Congenital Disease	SNOMED	27262009	Congenital absence of pectoral muscle (disorder)
Congenital Disease	SNOMED	27272007	Byzanthine arch palate (disorder)
Congenital Disease	SNOMED	27409004	Congenital macrocheilia (disorder)
Congenital Disease	SNOMED	274151005	Congenital absence of testis (disorder)
Congenital Disease	SNOMED	2749000	Congenital deformity of hip joint (disorder)
Congenital Disease	SNOMED	274908005	Deletion with complex rearrangement (disorder)
Congenital Disease	SNOMED	274947007	Divided right atrium (disorder)
Congenital Disease	SNOMED	275259005	Congenital malformation of ear (disorder)
Congenital Disease	SNOMED	275260000	Congenital malformation of the respiratory system (disorder)
Congenital Disease	SNOMED	275262008	Congenital malformation of upper alimentary tract (disorder)
Congenital Disease	SNOMED	275263003	Klinefelter's syndrome XXXY (disorder)
Congenital Disease	SNOMED	275264009	Klinefelter's syndrome XXXXY (disorder)
Congenital Disease	SNOMED	275346000	Absence of tibia (finding)
Congenital Disease	SNOMED	275347009	Absence of fibula (finding)
Congenital Disease	SNOMED	275348004	Adactyly (disorder)

Congenital Disease	SNOMED	275349007	Absence of skull bone (finding)
Congenital Disease	SNOMED	275353009	Supination deformity of foot (finding)
Congenital Disease	SNOMED	275407001	Double kidney with double pelvis (disorder)
Congenital Disease	SNOMED	275416002	Congenital bilateral aplasia of vas deferens (disorder)
Congenital Disease	SNOMED	275445000	Enlarged nails (finding)
Congenital Disease	SNOMED	275478007	Prominent ear (disorder)
Congenital Disease	SNOMED	275507003	Absence of lung (finding)
Congenital Disease	SNOMED	275519006	Peripheral arteriovenous malformation (disorder)
Congenital Disease	SNOMED	275521001	Blue baby (disorder)
Congenital Disease	SNOMED	27637000	Dextrocardia (disorder)
Congenital Disease	SNOMED	276518005	Transient tricuspid regurgitation of newborn (disorder)
Congenital Disease	SNOMED	276654001	Congenital malformation (disorder)
Congenital Disease	SNOMED	276655000	Congenital deformity (disorder)
Congenital Disease	SNOMED	276656004	Fetal postural deformity (disorder)
Congenital Disease	SNOMED	276697006	Harlequin change (disorder)
Congenital Disease	SNOMED	276699009	Third fontanelle (disorder)
Congenital Disease	SNOMED	276720006	Dysmorphism (disorder)
Congenital Disease	SNOMED	276723008	Intrahepatic biliary hypoplasia (disorder)
Congenital Disease	SNOMED	27680009	Congenital hyperplasia of sebaceous glands of lip (disorder)
Congenital Disease	SNOMED	277229001	Nasal turbinate absent (finding)
Congenital Disease	SNOMED	27729002	Pyloric atresia (disorder)
Congenital Disease	SNOMED	277301002	Ruptured spinal arteriovenous malformation (disorder)
Congenital Disease	SNOMED	277369003	Hamartoma of brain (disorder)
Congenital Disease	SNOMED	27742002	Vertebral anomalies/dysgenesis, anal atresia, tracheo-esophageal fistula, esophageal atresia, renal anomalies, radial dysplasia association (disorder)
Congenital Disease	SNOMED	277485007	Secondary pulmonary hypoplasia (disorder)
Congenital Disease	SNOMED	277494001	Vascular loops of inner ear (disorder)
Congenital Disease	SNOMED	277495000	Vascular malformation of inner ear (disorder)
Congenital Disease	SNOMED	277656005	Primary pulmonary hypoplasia (disorder)
Congenital Disease	SNOMED	27774009	Congenital deformity of ankle joint (disorder)
Congenital Disease	SNOMED	277807007	Curry-Hall syndrome (disorder)
Congenital Disease	SNOMED	277810000	Trichodental syndrome (disorder)
Congenital Disease	SNOMED	277812008	Juvenile elastoma (disorder)
Congenital Disease	SNOMED	277921008	Atelencephaly (disorder)
Congenital Disease	SNOMED	277922001	Aprosencephaly (disorder)
Congenital Disease	SNOMED	277949001	Combined malformation of central nervous system and skeletal muscle (disorder)
Congenital Disease	SNOMED	277950001	Muscle eye brain disease (disorder)
Congenital Disease	SNOMED	278088004	Flat sacral curve (finding)
Congenital Disease	SNOMED	27837003	Pyle metaphyseal dysplasia (disorder)
Congenital Disease	SNOMED	278523002	Hereditary striate leuconychia (disorder)
Congenital Disease	SNOMED	278530008	Atresia of nasolacrimal duct (disorder)
Congenital Disease	SNOMED	278531007	Congenital hydrocalicosis (disorder)
Congenital Disease	SNOMED	278532000	Transverse deficiency lower limb - knee level (disorder)
Congenital Disease	SNOMED	278708009	Spondylodysplastic group (disorder)
Congenital Disease	SNOMED	278713008	Spondyloepiphyseal dysplasia congenita group (disorder)
Congenital Disease	SNOMED	278715001	Chondrodysplasia punctata (stippled epiphyses) group (disorder)
Congenital Disease	SNOMED	278832007	Bent bone dysplasia group (disorder)
Congenital Disease	SNOMED	278833002	Craniometadiaphyseal dysplasia (disorder)
Congenital Disease	SNOMED	278834008	Idiopathic multicentric osteolysis (disorder)
Congenital Disease	SNOMED	278928000	Transient mitral regurgitation of newborn (disorder)
Congenital Disease	SNOMED	279014003	Congenital abnormality of nipple (disorder)
Congenital Disease	SNOMED	279081001	Dysostosis multiplex group (disorder)
Congenital Disease	SNOMED	279082008	Acromesomelic dysplasia group (disorder)
Congenital Disease	SNOMED	279309008	Osteogenesis imperfecta, type IV B (disorder)
Congenital Disease	SNOMED	27986000	Congenital pulmonary arteriovenous aneurysm (disorder)
Congenital Disease	SNOMED	279919005	Tubule of epoophoron (disorder)
Congenital Disease	SNOMED	279920004	Duct of epoophoron (disorder)
Congenital Disease	SNOMED	279921000	Vesicular appendix of ovary (disorder)
Congenital Disease	SNOMED	279922007	Paroophoron (disorder)
Congenital Disease	SNOMED	279923002	Tubule of paroophoron (disorder)
Congenital Disease	SNOMED	280143008	Venous remnant (disorder)
Congenital Disease	SNOMED	280144002	Persistent descending vein (disorder)
Congenital Disease	SNOMED	280159008	Osteogenesis imperfecta, type IV A (disorder)
Congenital Disease	SNOMED	28016005	Jackson's membrane (disorder)
Congenital Disease	SNOMED	28041003	Congenital lip pits (disorder)
Congenital Disease	SNOMED	28065000	Intralobar bronchopulmonary sequestration (disorder)
Congenital Disease	SNOMED	280831005	Arterial embryological remnant (disorder)
Congenital Disease	SNOMED	281095009	Congenital stricture of common bile duct (disorder)
Congenital Disease	SNOMED	281109007	Vestigial gastrointestinal remnant (disorder)
Congenital Disease	SNOMED	281372009	Lumbarized first sacral vertebra (disorder)
Congenital Disease	SNOMED	281373004	Sacralization of fifth lumbar vertebra (disorder)
Congenital Disease	SNOMED	281585008	Crossed ectopia of testis (disorder)
Congenital Disease	SNOMED	281587000	Pentalogy of Cantrell (disorder)
Congenital Disease	SNOMED	2818004	Congenital vascular anomaly of eye (disorder)

Congenital Disease	SNOMED	282036005	Congenital arterial aneurysm (disorder)
Congenital Disease	SNOMED	282038006	Congenital abnormality of external ear (disorder)
Congenital Disease	SNOMED	282040001	Congenital abnormality of nose and nasopharynx (disorder)
Congenital Disease	SNOMED	28204005	Inherited arthrogryposis (disorder)
Congenital Disease	SNOMED	282041002	Congenital abnormality of oral cavity (disorder)
Congenital Disease	SNOMED	282042009	Congenital abnormality of salivary duct (disorder)
Congenital Disease	SNOMED	2828008	Congenital stenosis of nares (disorder)
Congenital Disease	SNOMED	2829000	Uhl's disease (disorder)
Congenital Disease	SNOMED	285251000119101	Dextrotransposition of the great arteries (disorder)
Congenital Disease	SNOMED	28550007	Congenital capsular cataract (disorder)
Congenital Disease	SNOMED	28557005	Geleophysic dysplasia (disorder)
Congenital Disease	SNOMED	28574005	Congenital anomaly of coronary artery (disorder)
Congenital Disease	SNOMED	286071000119109	Congenital peripheral pulmonary artery stenosis (disorder)
Congenital Disease	SNOMED	286331000119109	Total anomalous pulmonary venous connection to coronary sinus (disorder)
Congenital Disease	SNOMED	286341000119100	Total anomalous pulmonary venous connection to hepatic vein (disorder)
Congenital Disease	SNOMED	286351000119103	Total anomalous pulmonary venous connection to right atrium (disorder)
Congenital Disease	SNOMED	286361000119101	Total anomalous pulmonary venous connection to superior vena cava (disorder)
Congenital Disease	SNOMED	28681006	Metaphyseal chondrodysplasia (disorder)
Congenital Disease	SNOMED	28682004	Congenital duplication of colon (disorder)
Congenital Disease	SNOMED	287080001	Congenital anomaly of nervous system of head/neck (disorder)
Congenital Disease	SNOMED	28724005	Cholestasis-edema syndrome, Norwegian type (disorder)
Congenital Disease	SNOMED	28740008	Trigonocephaly (disorder)
Congenital Disease	SNOMED	28770003	Polycystic kidney disease, infantile type (disorder)
Congenital Disease	SNOMED	288248009	Congenital bowing of tibia, fibula and femur (disorder)
Congenital Disease	SNOMED	28828001	Gastric atresia (disorder)
Congenital Disease	SNOMED	2884008	Weill-Marchesani syndrome (disorder)
Congenital Disease	SNOMED	28861008	Crouzon syndrome (disorder)
Congenital Disease	SNOMED	2893009	Anomaly of chromosome pair 10 (disorder)
Congenital Disease	SNOMED	289467001	Vulva absent (finding)
Congenital Disease	SNOMED	289502006	Clitoris absent (finding)
Congenital Disease	SNOMED	289634008	Uterus irregular in shape (finding)
Congenital Disease	SNOMED	289661008	Uterus asymmetrical (finding)
Congenital Disease	SNOMED	289758000	Cervix irregular in shape (finding)
Congenital Disease	SNOMED	289837004	Small ovary (finding)
Congenital Disease	SNOMED	290006	Melnick-Fraser syndrome (disorder)
Congenital Disease	SNOMED	29052002	Bilobed right lung (disorder)
Congenital Disease	SNOMED	29057008	Venous anomaly of umbilical cord (disorder)
Congenital Disease	SNOMED	29076005	Meckel-Gruber syndrome (disorder)
Congenital Disease	SNOMED	29110005	Congenital absence of small intestine (disorder)
Congenital Disease	SNOMED	29145002	Schwartz-Jampel syndrome (disorder)
Congenital Disease	SNOMED	29155003	Ectromelia of upper limb (disorder)
Congenital Disease	SNOMED	29248006	Metaphyseal chondrodysplasia, Schmid type (disorder)
Congenital Disease	SNOMED	29257000	13q partial monosomy syndrome (disorder)
Congenital Disease	SNOMED	29271008	Camptodactyly (disorder)
Congenital Disease	SNOMED	29307005	Craniolacunia (disorder)
Congenital Disease	SNOMED	29328001	Notomelus (disorder)
Congenital Disease	SNOMED	29345006	Congenital atresia of ejaculatory duct (disorder)
Congenital Disease	SNOMED	29352008	Thanatophoric dysplasia (disorder)
Congenital Disease	SNOMED	29375001	Abnormal number of cusps (disorder)
Congenital Disease	SNOMED	29379007	8q partial monosomy syndrome (disorder)
Congenital Disease	SNOMED	294705005	Dominant epidermolysis bullosa simplex, Weber-Cockayne type (disorder)
Congenital Disease	SNOMED	29581008	Longitudinal deficiency of tarsal bone (disorder)
Congenital Disease	SNOMED	29590001	Congenital total cataract (disorder)
Congenital Disease	SNOMED	29632002	Congenital atresia of pharynx (disorder)
Congenital Disease	SNOMED	29642000	Congenital adhesions of peritoneum (disorder)
Congenital Disease	SNOMED	2965006	Congenital alopecia (disorder)
Congenital Disease	SNOMED	29715005	Darwin's tubercle (disorder)
Congenital Disease	SNOMED	297159008	Laryngeal web (disorder)
Congenital Disease	SNOMED	297163001	Congenital urethral valve (disorder)
Congenital Disease	SNOMED	297165008	Duodenal web (disorder)
Congenital Disease	SNOMED	297195000	Macrodactyly of hand (disorder)
Congenital Disease	SNOMED	297218007	Congenital abnormality of ductus arteriosus (disorder)
Congenital Disease	SNOMED	297222002	Congenital abnormality of vein (disorder)
Congenital Disease	SNOMED	297267009	Retrosternal thyroid gland (disorder)
Congenital Disease	SNOMED	298145003	Joint absent (finding)
Congenital Disease	SNOMED	298727009	Deformity of sternum (disorder)
Congenital Disease	SNOMED	298764003	Thin clavicle (finding)
Congenital Disease	SNOMED	29934004	Anomalous pulmonary venous drainage to coronary sinus (disorder)
Congenital Disease	SNOMED	29938001	Extralobar bronchopulmonary sequestration (disorder)
Congenital Disease	SNOMED	29956001	Myelatelia (disorder)
Congenital Disease	SNOMED	299661006	Asymmetry of pelvis (finding)
Congenital Disease	SNOMED	29980002	Congenital malrotation of intestine (disorder)
Congenital Disease	SNOMED	300069000	Fusion of vulva (disorder)
Congenital Disease	SNOMED	300176008	Incus abnormal (finding)

Congenital Disease	SNOMED	300181004	Stapes abnormal (finding)
Congenital Disease	SNOMED	300183001	Ossicles abnormal (finding)
Congenital Disease	SNOMED	30023002	Hydranencephaly (disorder)
Congenital Disease	SNOMED	300245009	Tongue asymmetrical (finding)
Congenital Disease	SNOMED	30028006	Spondyloschisis (disorder)
Congenital Disease	SNOMED	300309008	Appendix absent (finding)
Congenital Disease	SNOMED	300315008	Anus absent (finding)
Congenital Disease	SNOMED	3004001	Congenital dilatation of esophagus (disorder)
Congenital Disease	SNOMED	300451002	Ureter absent (finding)
Congenital Disease	SNOMED	300495008	Epididymis absent (finding)
Congenital Disease	SNOMED	300506000	Vas deferens absent (finding)
Congenital Disease	SNOMED	300564004	Spleen absent (finding)
Congenital Disease	SNOMED	301130004	Atrial septal defect murmur (finding)
Congenital Disease	SNOMED	301318003	Head abnormal shape (finding)
Congenital Disease	SNOMED	302174000	Congenital abnormality of iris and ciliary body (disorder)
Congenital Disease	SNOMED	302295001	Cavovarus deformity of foot (disorder)
Congenital Disease	SNOMED	302297009	Congenital deformity of foot (disorder)
Congenital Disease	SNOMED	302298004	Congenital rearfoot valgus (disorder)
Congenital Disease	SNOMED	302299007	Congenital forefoot valgus (disorder)
Congenital Disease	SNOMED	30248008	Pygoamorphus (disorder)
Congenital Disease	SNOMED	30275001	Accessory kidney (disorder)
Congenital Disease	SNOMED	30278004	Kundrat's syndrome (disorder)
Congenital Disease	SNOMED	30288003	Ventricular septal defect (disorder)
Congenital Disease	SNOMED	302882002	Hydrocephalus associated with congenital aqueduct stenosis (disorder)
Congenital Disease	SNOMED	302943003	Abnormal number of pulmonary valve cusps (disorder)
Congenital Disease	SNOMED	302948007	Accessory salivary gland or duct (disorder)
Congenital Disease	SNOMED	302949004	Congenital bronchoesophageal fistula without atresia (disorder)
Congenital Disease	SNOMED	302951000	Congenital macrocolon, not aganglionic (disorder)
Congenital Disease	SNOMED	302952007	Congenital fistula of rectum and anus (disorder)
Congenital Disease	SNOMED	302953002	Agenesis of gallbladder (disorder)
Congenital Disease	SNOMED	302954008	Embryonic cyst of fallopian tube (disorder)
Congenital Disease	SNOMED	302955009	Congenital extension contracture of the knee (disorder)
Congenital Disease	SNOMED	302956005	Transverse deficiency of hand (disorder)
Congenital Disease	SNOMED	302957001	Transverse arrest metacarpal second to fifth rays (disorder)
Congenital Disease	SNOMED	302958006	Congenital absence of multiple toes (disorder)
Congenital Disease	SNOMED	302959003	Duplication of lower limb (disorder)
Congenital Disease	SNOMED	302960008	Mosaicism 45, X; 46, XX (disorder)
Congenital Disease	SNOMED	302961007	Hereditary splenic hypoplasia (disorder)
Congenital Disease	SNOMED	303070000	Pulmonary arteriovenous malformation (disorder)
Congenital Disease	SNOMED	303085006	Paratubal cyst arising in mesonephric duct (disorder)
Congenital Disease	SNOMED	303138003	Thyroglossal duct anomaly (disorder)
Congenital Disease	SNOMED	30361005	1q partial monosomy (disorder)
Congenital Disease	SNOMED	304068004	Bilateral cleft lip (disorder)
Congenital Disease	SNOMED	30449003	Talipes calcaneovarus (disorder)
Congenital Disease	SNOMED	30468000	Dolichocolon (disorder)
Congenital Disease	SNOMED	304931000119109	Congenital absence of left forearm and hand (disorder)
Congenital Disease	SNOMED	304961000119101	Congenital absence of left lower leg and foot (disorder)
Congenital Disease	SNOMED	304971000119107	Congenital absence of right lower leg and foot (disorder)
Congenital Disease	SNOMED	305121000119107	Congenital kyphosis of cervicothoracic spine (disorder)
Congenital Disease	SNOMED	30526003	Omocephalus (disorder)
Congenital Disease	SNOMED	30592006	Brachymetatarsia (disorder)
Congenital Disease	SNOMED	30620003	Spina bifida of dorsal region (disorder)
Congenital Disease	SNOMED	30652003	Hypermobile Ehlers-Danlos syndrome (disorder)
Congenital Disease	SNOMED	306915005	Abnormally shaped pinna (finding)
Congenital Disease	SNOMED	306949002	Laryngeal cleft type I (disorder)
Congenital Disease	SNOMED	306950002	Laryngeal cleft type II (disorder)
Congenital Disease	SNOMED	306951003	Laryngeal cleft type III (disorder)
Congenital Disease	SNOMED	306953000	Laryngeal cleft type IV (disorder)
Congenital Disease	SNOMED	3073006	Ruvalcaba syndrome (disorder)
Congenital Disease	SNOMED	307359001	Congenital agenesis of brainstem nuclei (disorder)
Congenital Disease	SNOMED	307670000	Congenital nystagmus with sensory abnormality (disorder)
Congenital Disease	SNOMED	308131007	Dysplasia of vocal cord (disorder)
Congenital Disease	SNOMED	308132000	Dysplasia of larynx (disorder)
Congenital Disease	SNOMED	308561000119101	Syndactyly of toes of bilateral feet (disorder)
Congenital Disease	SNOMED	308855000	Moderate stomach dysplasia (disorder)
Congenital Disease	SNOMED	308856004	Severe stomach dysplasia (disorder)
Congenital Disease	SNOMED	308861002	Biliary tract dysplasia (disorder)
Congenital Disease	SNOMED	308862009	Mild biliary tract dysplasia (disorder)
Congenital Disease	SNOMED	308863004	Moderate biliary tract dysplasia (disorder)
Congenital Disease	SNOMED	308864005	Severe biliary tract dysplasia (disorder)
Congenital Disease	SNOMED	308865006	Pancreatic duct dysplasia (disorder)
Congenital Disease	SNOMED	308873002	Severe dysplasia of colon (disorder)
Congenital Disease	SNOMED	30915001	Holoprosencephaly sequence (disorder)
Congenital Disease	SNOMED	309244002	Absent patella (finding)
Congenital Disease	SNOMED	309776008	Costello syndrome (disorder)

Congenital Disease	SNOMED	31076000	Congenital ischemic atrophy of central nervous system structure (disorder)
Congenital Disease	SNOMED	310798000	Brachydactyly of hand (disorder)
Congenital Disease	SNOMED	310800007	Brachyphalangia of toe (disorder)
Congenital Disease	SNOMED	31080005	Pericarditis secondary to Mulibrey nanism (disorder)
Congenital Disease	SNOMED	311808009	Aberrant retroesophageal subclavian artery causing dysphagia lusoria (disorder)
Congenital Disease	SNOMED	312005008	Congenital penoscrotal transposition (disorder)
Congenital Disease	SNOMED	312214005	Floating-Harbor syndrome (disorder)
Congenital Disease	SNOMED	312514006	Netherton's syndrome (disorder)
Congenital Disease	SNOMED	312601003	Thoracic aorta abnormality (disorder)
Congenital Disease	SNOMED	31290005	Congenital hydroureter (disorder)
Congenital Disease	SNOMED	31291009	Ectodermal dysplasia-ocular malformation syndrome (disorder)
Congenital Disease	SNOMED	31325007	Ring chromosome 21 syndrome (disorder)
Congenital Disease	SNOMED	313339007	Multiple epiphyseal dysplasia tarda type IIIa (disorder)
Congenital Disease	SNOMED	31339007	Congenital cyst of vulva (disorder)
Congenital Disease	SNOMED	313426007	Kabuki make-up syndrome (disorder)
Congenital Disease	SNOMED	31401003	Bicornuate uterus (disorder)
Congenital Disease	SNOMED	314270008	Persistent hyperplastic primary vitreous (disorder)
Congenital Disease	SNOMED	31429000	Cerebral cortical dysgenesis (disorder)
Congenital Disease	SNOMED	314508003	Congenital myogenic ptosis (disorder)
Congenital Disease	SNOMED	315027009	Congenital conduction defect (disorder)
Congenital Disease	SNOMED	315271000119104	Osteochondrodysplasia co-occurrent with defects of growth of tubular bones and spine (disorder)
Congenital Disease	SNOMED	315297006	Congenital dysgenetic ptosis (disorder)
Congenital Disease	SNOMED	31570000	Congenital atresia of artery (disorder)
Congenital Disease	SNOMED	31686000	Congenital anomaly of lower alimentary tract (disorder)
Congenital Disease	SNOMED	31915006	Congenital deviation of ureter (disorder)
Congenital Disease	SNOMED	32003007	Congenital anomaly of face bones (disorder)
Congenital Disease	SNOMED	32107005	Anomaly of chromosome pair 17 (disorder)
Congenital Disease	SNOMED	32113001	Syndactyly of toes (disorder)
Congenital Disease	SNOMED	32194006	Anomalous pulmonary venous drainage to hepatic veins (disorder)
Congenital Disease	SNOMED	32219008	Craniorachischisis (disorder)
Congenital Disease	SNOMED	32232003	Spina bifida of cervical region (disorder)
Congenital Disease	SNOMED	32299009	Anomaly of chromosome pair 2 (disorder)
Congenital Disease	SNOMED	32339005	Longitudinal deficiency of metacarpal bone (disorder)
Congenital Disease	SNOMED	323751000119109	Simple syndactyly of toes of bilateral feet (disorder)
Congenital Disease	SNOMED	323761000119106	Simple syndactyly of toes of left foot (disorder)
Congenital Disease	SNOMED	323771000119100	Simple syndactyly of toes of right foot (disorder)
Congenital Disease	SNOMED	32454003	Congenital anomaly of the thyroid gland (disorder)
Congenital Disease	SNOMED	32570681000036106	Indeterminate sex (finding)
Congenital Disease	SNOMED	32614006	Microglossia (disorder)
Congenital Disease	SNOMED	32659003	Congenital hypoplasia of kidney (disorder)
Congenital Disease	SNOMED	3274008	Flat chest (disorder)
Congenital Disease	SNOMED	328011000119101	Congenital imperforate cervix (disorder)
Congenital Disease	SNOMED	32809005	Congenital anomaly of sclera (disorder)
Congenital Disease	SNOMED	328641000119109	Genetic disorder of surfactant dysfunction (disorder)
Congenital Disease	SNOMED	32985001	Greig cephalopolysyndactyly syndrome (disorder)
Congenital Disease	SNOMED	330041000119103	Congenital porencephalic cyst (disorder)
Congenital Disease	SNOMED	331011000119109	Vascular anomaly of right upper eyelid (disorder)
Congenital Disease	SNOMED	331021000119102	Vascular anomaly of right lower eyelid (disorder)
Congenital Disease	SNOMED	331031000119104	Vascular anomaly of right eyelid (disorder)
Congenital Disease	SNOMED	331041000119108	Vascular anomaly of left upper eyelid (disorder)
Congenital Disease	SNOMED	331051000119105	Vascular anomaly of left lower eyelid (disorder)
Congenital Disease	SNOMED	331061000119107	Vascular anomaly of left eyelid (disorder)
Congenital Disease	SNOMED	33225004	Anorectal anomaly (disorder)
Congenital Disease	SNOMED	33229005	Microphthalmos associated with other anomalies of eye AND/OR adnexa (disorder)
Congenital Disease	SNOMED	33257003	Congenital duplication of digestive organs (disorder)
Congenital Disease	SNOMED	33313004	Radioulnar synostosis (disorder)
Congenital Disease	SNOMED	33322003	Congenital deformity of forehead (disorder)
Congenital Disease	SNOMED	33410002	Marshall syndrome (disorder)
Congenital Disease	SNOMED	33494005	Talipes calcaneus (disorder)
Congenital Disease	SNOMED	33521009	Congenital anomaly of lens shape (disorder)
Congenital Disease	SNOMED	33534005	Congenital bowing of femur (disorder)
Congenital Disease	SNOMED	335831000119107	Congenital posterior subcapsular polar cataract of right eye (disorder)
Congenital Disease	SNOMED	335841000119103	Congenital nuclear cataract of right eye (disorder)
Congenital Disease	SNOMED	335851000119101	Congenital combined form cataract of right eye (disorder)
Congenital Disease	SNOMED	335861000119104	Congenital cataract of right eye (disorder)
Congenital Disease	SNOMED	335871000119105	Congenital anterior subcapsular polar cataract of right eye (disorder)
Congenital Disease	SNOMED	33700007	Ruptured sinus of Valsalva into right atrium (disorder)
Congenital Disease	SNOMED	33706001	7q partial trisomy (disorder)
Congenital Disease	SNOMED	337471007	Cleft upper lip, upper jaw AND palate (disorder)
Congenital Disease	SNOMED	33754009	Congenital coxa valga (disorder)
Congenital Disease	SNOMED	338486003	Cheilognathouranoschisis (disorder)
Congenital Disease	SNOMED	33979003	Nievergelt's syndrome (disorder)

Congenital Disease	SNOMED	33990008	Ectopic parotid gland tissue (disorder)
Congenital Disease	SNOMED	34048007	Syndactyly of fingers (disorder)
Congenital Disease	SNOMED	34111000	Congenital anomaly of hand (disorder)
Congenital Disease	SNOMED	341441000119102	Congenital posterior subcapsular polar cataract of left eye (disorder)
Congenital Disease	SNOMED	341451000119100	Congenital nuclear cataract of left eye (disorder)
Congenital Disease	SNOMED	341461000119103	Congenital combined form cataract of left eye (disorder)
Congenital Disease	SNOMED	341471000119109	Congenital cataract of left eye (disorder)
Congenital Disease	SNOMED	341481000119107	Congenital anterior subcapsular polar cataract of left eye (disorder)
Congenital Disease	SNOMED	34168003	Diaphragmatic eventration (disorder)
Congenital Disease	SNOMED	342821000119103	Congenital posterior subcapsular polar cataract (disorder)
Congenital Disease	SNOMED	342831000119100	Congenital combined form cataract (disorder)
Congenital Disease	SNOMED	342841000119109	Congenital choroidal fold (disorder)
Congenital Disease	SNOMED	342911000119104	Congenital anterior subcapsular polar cataract (disorder)
Congenital Disease	SNOMED	34424009	Congenital duplication of vagina (disorder)
Congenital Disease	SNOMED	34488005	Dimelia (disorder)
Congenital Disease	SNOMED	34612006	Pelvis justo major (disorder)
Congenital Disease	SNOMED	34638006	Lamellar ichthyosis AND trichorrhexis invaginata syndrome (disorder)
Congenital Disease	SNOMED	34643004	Diaphyseal dysplasia (disorder)
Congenital Disease	SNOMED	346691000119104	Congenital posterior subcapsular polar cataract of bilateral eyes (disorder)
Congenital Disease	SNOMED	346701000119104	Congenital nuclear cataract of bilateral eyes (disorder)
Congenital Disease	SNOMED	346711000119101	Congenital combined form cataract of bilateral eyes (disorder)
Congenital Disease	SNOMED	346721000119108	Congenital cataract of bilateral eyes (disorder)
Congenital Disease	SNOMED	346731000119106	Congenital anterior subcapsular polar cataract of bilateral eyes (disorder)
Congenital Disease	SNOMED	34739009	Urticaria pigmentosa, adult form (disorder)
Congenital Disease	SNOMED	34748004	Adams-Oliver syndrome (disorder)
Congenital Disease	SNOMED	34774005	Congenital absence of bronchus (disorder)
Congenital Disease	SNOMED	348151000119103	Congenital pit of optic disc of right eye (disorder)
Congenital Disease	SNOMED	34821005	Congenital stenosis of choanae (disorder)
Congenital Disease	SNOMED	348601000119109	Congenital pit of optic disc of left eye (disorder)
Congenital Disease	SNOMED	34911001	Congenital hypoplasia of penis (disorder)
Congenital Disease	SNOMED	349281000119109	Congenital zonular cataract of bilateral eyes (disorder)
Congenital Disease	SNOMED	35031005	Hanhart's syndrome (disorder)
Congenital Disease	SNOMED	35045004	Microtia (disorder)
Congenital Disease	SNOMED	35082008	Cervical thymic remnant (disorder)
Congenital Disease	SNOMED	35111000119109	Cystic malformation of posterior fossa (disorder)
Congenital Disease	SNOMED	35111009	Trisomy X syndrome (disorder)
Congenital Disease	SNOMED	35162007	Ruptured sinus of Valsalva into right ventricle (disorder)
Congenital Disease	SNOMED	35266001	Congenital duplication of appendix (disorder)
Congenital Disease	SNOMED	35272001	Microphakia (disorder)
Congenital Disease	SNOMED	35387008	Congenital aphakia (disorder)
Congenital Disease	SNOMED	35484002	Aplasia cutis congenita (disorder)
Congenital Disease	SNOMED	35520007	Nager syndrome (disorder)
Congenital Disease	SNOMED	35547002	Polyotia (disorder)
Congenital Disease	SNOMED	35555009	Accessory trachea (disorder)
Congenital Disease	SNOMED	35577008	Holoacardius acephalus (disorder)
Congenital Disease	SNOMED	35595006	Deradelphus (disorder)
Congenital Disease	SNOMED	359531004	Amegakaryocytic thrombocytopenia with congenital malformation (disorder)
Congenital Disease	SNOMED	35962006	Nevus comedonicus (disorder)
Congenital Disease	SNOMED	35964007	Congenital anomaly of nail (disorder)
Congenital Disease	SNOMED	359824007	Incomplete anencephaly (disorder)
Congenital Disease	SNOMED	36010004	Congenital cerebral meningocele (disorder)
Congenital Disease	SNOMED	36025004	Fibrous skin tumor of tuberous sclerosis (disorder)
Congenital Disease	SNOMED	360422007	Doubling of uterus with doubling of cervix and vagina (disorder)
Congenital Disease	SNOMED	360424008	Dysplastic ovary (disorder)
Congenital Disease	SNOMED	360426005	Congenital ovarian dysplasia (disorder)
Congenital Disease	SNOMED	360429003	Congenital epiblepharon-inferior oblique syndrome (disorder)
Congenital Disease	SNOMED	360434004	Aganglionosis of Auerbach's plexus (disorder)
Congenital Disease	SNOMED	360441005	Aganglionosis of colon (disorder)
Congenital Disease	SNOMED	360473004	Congenital muscular subaortic stenosis (disorder)
Congenital Disease	SNOMED	360481003	Common atrioventricular canal (disorder)
Congenital Disease	SNOMED	360491009	Congenital atresia of jejunum (disorder)
Congenital Disease	SNOMED	360494001	Malrotation of the intestine type IIID (disorder)
Congenital Disease	SNOMED	360507004	Chondrodysplasia punctata congenita (disorder)
Congenital Disease	SNOMED	360526007	Congenital absence of upper limb (disorder)
Congenital Disease	SNOMED	360527003	Diplomyelia (disorder)
Congenital Disease	SNOMED	360530005	Myeloschisis (disorder)
Congenital Disease	SNOMED	36079008	Double cardiac valve orifice (disorder)
Congenital Disease	SNOMED	36110001	Congenital anomaly of pulmonary artery (disorder)
Congenital Disease	SNOMED	36114005	Sex phenotype-karyotype dissociation syndrome (disorder)
Congenital Disease	SNOMED	361146001	Congenital small renal papilla (disorder)
Congenital Disease	SNOMED	361147005	Congenital hypoplasia of renal papilla (disorder)
Congenital Disease	SNOMED	361213004	Congenital absence of pelvis and lower limb (disorder)
Congenital Disease	SNOMED	361263009	Paravaginal cyst arising in mesonephric duct (disorder)
Congenital Disease	SNOMED	361264003	Congenital arteriovenous fistula of kidney (disorder)
Congenital Disease	SNOMED	361265002	Congenital stenosis of external auditory canal (disorder)

Congenital Disease	SNOMED	36133000	Abnormal position of cardiac valve (disorder)
Congenital Disease	SNOMED	36172001	Congenital subluxation of hip (disorder)
Congenital Disease	SNOMED	36193003	Thalidomide embryopathy syndrome (disorder)
Congenital Disease	SNOMED	362984008	Anomaly of chromosome pair (disorder)
Congenital Disease	SNOMED	362998000	Branchial cleft anomaly (disorder)
Congenital Disease	SNOMED	363024001	Congenital anomaly of abdomen (disorder)
Congenital Disease	SNOMED	363025000	Congenital anomaly of back (disorder)
Congenital Disease	SNOMED	363026004	Congenital anomaly of body cavity (disorder)
Congenital Disease	SNOMED	363027008	Congenital anomaly of body wall (disorder)
Congenital Disease	SNOMED	363028003	Congenital anomaly of cardiovascular structure of trunk (disorder)
Congenital Disease	SNOMED	363029006	Congenital anomaly of epidermal appendages (disorder)
Congenital Disease	SNOMED	363030001	Congenital anomaly of lower trunk (disorder)
Congenital Disease	SNOMED	363031002	Congenital anomaly of lymphatic structure of trunk (disorder)
Congenital Disease	SNOMED	363032009	Congenital anomaly of musculoskeletal structure of trunk (disorder)
Congenital Disease	SNOMED	363034005	Congenital anomaly of neural structure of trunk (disorder)
Congenital Disease	SNOMED	363035006	Congenital anomaly of thorax (disorder)
Congenital Disease	SNOMED	363036007	Congenital anomaly of tympanic anulus (disorder)
Congenital Disease	SNOMED	363037003	Congenital anomaly of upper trunk (disorder)
Congenital Disease	SNOMED	363039000	Congenital connective tissue disorder (disorder)
Congenital Disease	SNOMED	36313005	Dolichopellic pelvis (disorder)
Congenital Disease	SNOMED	363627009	Parathyromatosis (disorder)
Congenital Disease	SNOMED	36369001	1p partial monosomy (disorder)
Congenital Disease	SNOMED	36376006	Congenital absence of esophagus (disorder)
Congenital Disease	SNOMED	36422005	Transposition of pulmonary veins (disorder)
Congenital Disease	SNOMED	36454001	Xeroderma pigmentosum, group G (disorder)
Congenital Disease	SNOMED	3650004	Congenital absence of liver (disorder)
Congenital Disease	SNOMED	36517007	Polyostotic fibrous dysplasia of bone (disorder)
Congenital Disease	SNOMED	36574005	Transverse deficiency of lower limb (disorder)
Congenital Disease	SNOMED	36601008	Craniometaphyseal dysplasia (disorder)
Congenital Disease	SNOMED	36619004	Congenital duplication of cystic duct (disorder)
Congenital Disease	SNOMED	36631002	Hepatomphalocele (disorder)
Congenital Disease	SNOMED	36659001	Congenital notching of tip of nose (disorder)
Congenital Disease	SNOMED	367101000119105	Congenital malformation of bladder and urethra (disorder)
Congenital Disease	SNOMED	367121000119101	Congenital prolapse of urinary meatus (disorder)
Congenital Disease	SNOMED	367462009	Facio-auriculo-vertebral spectrum (disorder)
Congenital Disease	SNOMED	367468008	Congenital atresia of nares (disorder)
Congenital Disease	SNOMED	367489004	Infantile malignant osteopetrosis (disorder)
Congenital Disease	SNOMED	367506006	Polydactyly (disorder)
Congenital Disease	SNOMED	367520004	Incontinentia pigmenti syndrome (disorder)
Congenital Disease	SNOMED	36752001	Congenital splenomegaly (disorder)
Congenital Disease	SNOMED	36755004	Talipes cavus (disorder)
Congenital Disease	SNOMED	36775008	Displaced ureteric orifice (disorder)
Congenital Disease	SNOMED	3680009	Monocephalus tripus dibrachius (disorder)
Congenital Disease	SNOMED	369071000119105	Congenital renal cyst (disorder)
Congenital Disease	SNOMED	3699000	Transverse deficiency of upper limb (disorder)
Congenital Disease	SNOMED	370473000	Congenital arthrogryposis caused by teratogen (disorder)
Congenital Disease	SNOMED	370480003	Hepatoportal microvascular dysplasia (disorder)
Congenital Disease	SNOMED	370481004	Transitional vertebra (disorder)
Congenital Disease	SNOMED	370483001	Mittendorf's dot (disorder)
Congenital Disease	SNOMED	3705009	Congenital anomaly of anterior chamber of eye (disorder)
Congenital Disease	SNOMED	37054000	Congenital atresia of colon (disorder)
Congenital Disease	SNOMED	370551009	Dynamic right ventricular outflow tract obstruction (disorder)
Congenital Disease	SNOMED	370552002	Dynamic aortic outflow tract obstruction (disorder)
Congenital Disease	SNOMED	370966000	Congenital anomaly of endocrine ovary (disorder)
Congenital Disease	SNOMED	371015003	Congenital absence of both testes (disorder)
Congenital Disease	SNOMED	37104009	Congenital enlargement of coronary sinus (disorder)
Congenital Disease	SNOMED	371045000	Translocation Down syndrome (disorder)
Congenital Disease	SNOMED	371080001	Congenital leg length discrepancy (disorder)
Congenital Disease	SNOMED	371110006	Immature eyes (disorder)
Congenital Disease	SNOMED	371118004	Congenital anomaly of endocrine gonad (disorder)
Congenital Disease	SNOMED	371122009	Congenital anomaly of endocrine testis (disorder)
Congenital Disease	SNOMED	371189003	Bilateral acheiria (disorder)
Congenital Disease	SNOMED	371191006	Bilateral congenital absence of feet (disorder)
Congenital Disease	SNOMED	371197005	Congenital absence of foot (disorder)
Congenital Disease	SNOMED	371199008	Congenital absence of hand (disorder)
Congenital Disease	SNOMED	371389007	Trochlear notch incongruity (disorder)
Congenital Disease	SNOMED	371629001	Congenital dislocation of elbow (disorder)
Congenital Disease	SNOMED	37221009	Congenital absence of all toes (disorder)
Congenital Disease	SNOMED	37281006	Cyclops hypognathus (disorder)
Congenital Disease	SNOMED	373092008	Coronary artery fistula to left atrium (disorder)
Congenital Disease	SNOMED	373093003	Coronary artery fistula (disorder)
Congenital Disease	SNOMED	373131000	Non-restrictive ventricular septal defect (disorder)
Congenital Disease	SNOMED	373133002	Enlarged aortic [REDACTED] (finding)
Congenital Disease	SNOMED	3733009	Congenital eventration of right crus of diaphragm (disorder)
Congenital Disease	SNOMED	373413006	Syndactyly (disorder)

Congenital Disease	SNOMED	373427001	Congenital bony fusion of phalanges (disorder)
Congenital Disease	SNOMED	373583002	Congenital obstructive megaureter (disorder)
Congenital Disease	SNOMED	373584008	Congenital obstruction of ureteropelvic junction (disorder)
Congenital Disease	SNOMED	373585009	Congenital obstruction of ureteral orifice (disorder)
Congenital Disease	SNOMED	373586005	Congenital urinary meatus obstruction (disorder)
Congenital Disease	SNOMED	373587001	Chiari malformation type II (disorder)
Congenital Disease	SNOMED	373637000	Congenital vesicoureterorenal reflux (disorder)
Congenital Disease	SNOMED	373643003	Cleft lip and cleft of alveolar process of maxilla (disorder)
Congenital Disease	SNOMED	373650004	Hypoplasia of optic disc (disorder)
Congenital Disease	SNOMED	373661007	Optic disc structural anomaly (disorder)
Congenital Disease	SNOMED	37367006	Anomaly of chromosome pair 7 (disorder)
Congenital Disease	SNOMED	37373007	Meckel's diverticulum (disorder)
Congenital Disease	SNOMED	37404003	Failure of rotation of colon (disorder)
Congenital Disease	SNOMED	37506004	4q partial monosomy syndrome (disorder)
Congenital Disease	SNOMED	37528004	Malrotation of cecum (disorder)
Congenital Disease	SNOMED	37535007	Anomaly of chromosome pair 12 (disorder)
Congenital Disease	SNOMED	37639005	Anomalous pulmonary venous drainage to right atrium (disorder)
Congenital Disease	SNOMED	37687000	Congenital absence of cervix (disorder)
Congenital Disease	SNOMED	37767008	Congenital deformity of wall of nasal sinus (disorder)
Congenital Disease	SNOMED	37849005	Congenital uterine anomaly (disorder)
Congenital Disease	SNOMED	37939008	Congenital anomaly of the bladder (disorder)
Congenital Disease	SNOMED	37975005	Accessory ovary (disorder)
Congenital Disease	SNOMED	38096003	Functional asplenia (disorder)
Congenital Disease	SNOMED	38164009	Congenital anomaly of integument (disorder)
Congenital Disease	SNOMED	38215007	Oculodentodigital syndrome (disorder)
Congenital Disease	SNOMED	38230003	Ruptured sinus of Valsalva into left ventricle (disorder)
Congenital Disease	SNOMED	38296007	Congenital anomaly of ovary (disorder)
Congenital Disease	SNOMED	38353004	Congenital porencephaly (disorder)
Congenital Disease	SNOMED	38371006	Poland anomaly (disorder)
Congenital Disease	SNOMED	38385001	Persistent left posterior cardinal vein (disorder)
Congenital Disease	SNOMED	38437003	Uterus arcuatus (disorder)
Congenital Disease	SNOMED	38439000	Cranial duplication (disorder)
Congenital Disease	SNOMED	3845008	Congenital duplication of intestine (disorder)
Congenital Disease	SNOMED	38494008	Langer mesomelic dysplasia syndrome (disorder)
Congenital Disease	SNOMED	385482004	Osteogenesis imperfecta type I (disorder)
Congenital Disease	SNOMED	385483009	Osteogenesis imperfecta type III (disorder)
Congenital Disease	SNOMED	38632003	Pharyngeal pituitary tissue (disorder)
Congenital Disease	SNOMED	38774000	Pectus carinatum (disorder)
Congenital Disease	SNOMED	38776003	Congenital absence of skeletal muscle (disorder)
Congenital Disease	SNOMED	38804009	Turner syndrome (disorder)
Congenital Disease	SNOMED	38824008	Congenital anomaly of the thymus (disorder)
Congenital Disease	SNOMED	38827001	Congenital fusion of spine (disorder)
Congenital Disease	SNOMED	38847009	XXXXY syndrome (disorder)
Congenital Disease	SNOMED	38856001	Congenital anomaly of appendix (disorder)
Congenital Disease	SNOMED	38859008	Syndactyly of toes with fusion of bones (disorder)
Congenital Disease	SNOMED	388981000	Congenital dystrophia brevicollis (disorder)
Congenital Disease	SNOMED	389157002	Thanatophoric dysplasia, type 1 (disorder)
Congenital Disease	SNOMED	389158007	Thanatophoric dysplasia, type 2 (disorder)
Congenital Disease	SNOMED	389159004	Mild spondyloepiphyseal dysplasia with premature onset arthrosis (disorder)
Congenital Disease	SNOMED	389160009	Spondyloepiphyseal dysplasia with joint laxity (disorder)
Congenital Disease	SNOMED	389161008	Sponastrime dysplasia (disorder)
Congenital Disease	SNOMED	389162001	Acroscyphodysplasia (disorder)
Congenital Disease	SNOMED	389163006	Metaphyseal chondrodysplasia, Sedaghatian type (disorder)
Congenital Disease	SNOMED	389165004	Brachyolmia - Maroteaux type (disorder)
Congenital Disease	SNOMED	389166003	Trichorhinophalangeal dysplasia type III (disorder)
Congenital Disease	SNOMED	389167007	Acromesomelic dysplasia Hunter-Thompson type (disorder)
Congenital Disease	SNOMED	389168002	Brachydactyly syndrome type B (disorder)
Congenital Disease	SNOMED	389169005	Brachydactyly syndrome type C (disorder)
Congenital Disease	SNOMED	389170006	Precocious osteodysplasty (disorder)
Congenital Disease	SNOMED	389171005	Yunis-Varon dysplasia (disorder)
Congenital Disease	SNOMED	38919006	Congenital absence of auricle with atresia of auditory canal (disorder)
Congenital Disease	SNOMED	389191003	Osteoplastic dysplasia (disorder)
Congenital Disease	SNOMED	389193000	Osteodysplastic dysplasia, type I (disorder)
Congenital Disease	SNOMED	389195007	Osteodysplastic dysplasia, type II (disorder)
Congenital Disease	SNOMED	389199001	Cole-Carpenter dysplasia (disorder)
Congenital Disease	SNOMED	389203001	White sponge nevus of mucosa (disorder)
Congenital Disease	SNOMED	389207000	Mixed sclerosing bone dysplasia (disorder)
Congenital Disease	SNOMED	389214003	Ghosal hematodiaphyseal dysplasia (disorder)
Congenital Disease	SNOMED	389236000	Neonatal osteosclerotic dysplasia (disorder)
Congenital Disease	SNOMED	389237009	Blomstrand dysplasia (disorder)
Congenital Disease	SNOMED	389239007	Raine dysplasia (disorder)
Congenital Disease	SNOMED	389260001	Lethal chondrodysplasia with fragmented bone (disorder)
Congenital Disease	SNOMED	389261002	Greenberg dysplasia (disorder)
Congenital Disease	SNOMED	389262009	Dappled diaphyseal dysplasia (disorder)
Congenital Disease	SNOMED	389263004	Astley-Kendall dysplasia (disorder)

Congenital Disease	SNOMED	389264005	Genochondromatosis (disorder)
Congenital Disease	SNOMED	389268008	Spondyloenchondromatosis (disorder)
Congenital Disease	SNOMED	389271000	Spondyloenchondromatosis with basal ganglia calcification (disorder)
Congenital Disease	SNOMED	389275009	Giacci familial neurogenic acroosteolysis (disorder)
Congenital Disease	SNOMED	389276005	Congenital dysplasia of patella (disorder)
Congenital Disease	SNOMED	389277001	Scypho-patellar dysplasia (disorder)
Congenital Disease	SNOMED	38993008	Tricho-dento-osseous syndrome (disorder)
Congenital Disease	SNOMED	38998004	Brachyphalangia (disorder)
Congenital Disease	SNOMED	39150004	Congenital anomaly of spinal meninges (disorder)
Congenital Disease	SNOMED	391982004	Congenital pectus excavatum (disorder)
Congenital Disease	SNOMED	391987005	Pectus excavatum (disorder)
Congenital Disease	SNOMED	392437005	Posterior embryotoxon (disorder)
Congenital Disease	SNOMED	392461003	Congenital ectopic pupil (disorder)
Congenital Disease	SNOMED	39302008	Fundus coloboma (disorder)
Congenital Disease	SNOMED	39401000	Dolichocephalic dwarfism (disorder)
Congenital Disease	SNOMED	39427000	Pachyonychia congenita syndrome (disorder)
Congenital Disease	SNOMED	39462005	Thyroglossal duct cyst (disorder)
Congenital Disease	SNOMED	39476006	Congenital stricture of rectum (disorder)
Congenital Disease	SNOMED	394910004	Congenital malformation of thumb (disorder)
Congenital Disease	SNOMED	39513007	Congenital atresia of vas deferens (disorder)
Congenital Disease	SNOMED	39540007	Congenital depression in skull (disorder)
Congenital Disease	SNOMED	39564008	Monocephalus (disorder)
Congenital Disease	SNOMED	395693008	Congenital positional talipes (disorder)
Congenital Disease	SNOMED	39574006	Congenital hypoplasia of inner granular layer of cerebellum (disorder)
Congenital Disease	SNOMED	39589002	Hypoplasia of right heart (disorder)
Congenital Disease	SNOMED	3961000119101	Relative macrocephaly (finding)
Congenital Disease	SNOMED	396461001	Hepatic duct dysplasia (disorder)
Congenital Disease	SNOMED	397007003	Mast cell disorder (disorder)
Congenital Disease	SNOMED	397012002	Cutaneous mastocytosis (disorder)
Congenital Disease	SNOMED	397014001	Diffuse erythrodermic mastocytosis (disorder)
Congenital Disease	SNOMED	39719008	Transposition of intestine (disorder)
Congenital Disease	SNOMED	397433001	Congenital tracheal collapse (disorder)
Congenital Disease	SNOMED	397830005	Pericardial anomaly (disorder)
Congenital Disease	SNOMED	397868007	Choledochocele (disorder)
Congenital Disease	SNOMED	39788007	Ectrodactyly-ectodermal dysplasia-clefting syndrome (disorder)
Congenital Disease	SNOMED	397894003	Congenital pericardial defect (disorder)
Congenital Disease	SNOMED	397932003	Talipes equinovarus (disorder)
Congenital Disease	SNOMED	398071000	Epidermolysis bullosa simplex, Ogna type (disorder)
Congenital Disease	SNOMED	398078006	Pericardial defect (disorder)
Congenital Disease	SNOMED	398114001	Ehlers-Danlos syndrome (disorder)
Congenital Disease	SNOMED	398147005	Abnormal communication between pericardial sac and pleura (disorder)
Congenital Disease	SNOMED	398170002	Autosomal dominant epidermolysis bullosa simplex (disorder)
Congenital Disease	SNOMED	398197009	Congenital choledochal cyst (disorder)
Congenital Disease	SNOMED	398206004	Congenital deformity of face (disorder)
Congenital Disease	SNOMED	398302004	Congenital anomaly of face (disorder)
Congenital Disease	SNOMED	398309008	Talipes (disorder)
Congenital Disease	SNOMED	398316009	Patent urachus (disorder)
Congenital Disease	SNOMED	398320008	Fistula of urachus (disorder)
Congenital Disease	SNOMED	3987009	Congenital absence of trachea (disorder)
Congenital Disease	SNOMED	398719004	Chondrodysplasia punctata, Conradi-Hünermann type (disorder)
Congenital Disease	SNOMED	398958000	Chondrodysplasia punctata, X-linked dominant type (disorder)
Congenital Disease	SNOMED	399046008	L - transposition of the great vessels (disorder)
Congenital Disease	SNOMED	39905002	Scimitar syndrome (disorder)
Congenital Disease	SNOMED	399105000	Redundant soft palate (finding)
Congenital Disease	SNOMED	399216004	D - transposition of the great vessels (disorder)
Congenital Disease	SNOMED	399228007	Tetralogy of Fallot with absent pulmonary valve (disorder)
Congenital Disease	SNOMED	399340005	Hereditary nephritis (disorder)
Congenital Disease	SNOMED	399691000	Dysplasia of extrahepatic bile ducts (disorder)
Congenital Disease	SNOMED	39987008	Congenital absence of right pulmonary artery (disorder)
Congenital Disease	SNOMED	399889006	Hereditary lymphedema type I (disorder)
Congenital Disease	SNOMED	399906000	Hamartoma of skin appendage (disorder)
Congenital Disease	SNOMED	399921006	Hamartoma of skin (disorder)
Congenital Disease	SNOMED	399940000	Hamartoma of muscle (disorder)
Congenital Disease	SNOMED	399947002	Progeroid short stature with pigmented nevi (disorder)
Congenital Disease	SNOMED	399955009	Diffuse palmoplantar keratoderma of Thost-Unna (disorder)
Congenital Disease	SNOMED	399960008	Congenital hamartoma (disorder)
Congenital Disease	SNOMED	399971009	Junctional epidermolysis bullosa (disorder)
Congenital Disease	SNOMED	399974001	Ectodermal dysplasia with tooth-nail-sweating defect (disorder)
Congenital Disease	SNOMED	399982001	Congenital vascular disorder (disorder)
Congenital Disease	SNOMED	399995006	Inflammatory linear verrucous epidermal nevus (disorder)
Congenital Disease	SNOMED	400006008	Hamartoma (disorder)
Congenital Disease	SNOMED	400014002	Hereditary benign intraepithelial dyskeratosis (disorder)
Congenital Disease	SNOMED	400017009	Mixed vascular malformation (disorder)
Congenital Disease	SNOMED	400018004	Acrokeratosis verruciformis of Darier disease (disorder)
Congenital Disease	SNOMED	400035000	Hamartoma of apocrine sweat gland apparatus (disorder)

Congenital Disease	SNOMED	400036004	Hypoplastic enamel-onycholysis-hypohidrosis syndrome (disorder)
Congenital Disease	SNOMED	400038003	Congenital malformation syndrome (disorder)
Congenital Disease	SNOMED	400040008	Hereditary lymphedema type II (disorder)
Congenital Disease	SNOMED	400042000	Cutaneous vascular malformation (disorder)
Congenital Disease	SNOMED	400043005	Keratosis rubra pilaris (disorder)
Congenital Disease	SNOMED	400059005	Keratosis pilaris atrophicans (disorder)
Congenital Disease	SNOMED	400080004	Porokeratosis (disorder)
Congenital Disease	SNOMED	400083002	Congenital hamartoma of skin (disorder)
Congenital Disease	SNOMED	400085009	Acrokeratosis verruciformis of Hopf (disorder)
Congenital Disease	SNOMED	400111008	Lipomatous hamartoma (disorder)
Congenital Disease	SNOMED	400120004	Hamartoma of pilosebaceous apparatus (disorder)
Congenital Disease	SNOMED	400123002	Hereditary diffuse palmoplantar keratoderma (disorder)
Congenital Disease	SNOMED	400126005	Ulerythema ophryogenes (disorder)
Congenital Disease	SNOMED	400128006	Lethal tight skin contracture syndrome (disorder)
Congenital Disease	SNOMED	400140006	Junctional epidermolysis bullosa gravis of Herlitz (disorder)
Congenital Disease	SNOMED	400146000	Splenic hamartoma (disorder)
Congenital Disease	SNOMED	400158000	Primary lymphedema tardum (disorder)
Congenital Disease	SNOMED	400159008	Congenital vascular malformation (disorder)
Congenital Disease	SNOMED	400206003	Hamartoma of integument (disorder)
Congenital Disease	SNOMED	40028009	Congenital hyperplasia of intrahepatic bile duct (disorder)
Congenital Disease	SNOMED	40052002	Congenital anomaly of neck (disorder)
Congenital Disease	SNOMED	400951005	Cryptophthalmos (disorder)
Congenital Disease	SNOMED	400952003	Ankyloblepharon filiforme adnatum (disorder)
Congenital Disease	SNOMED	400953008	Congenital tarsal kink (disorder)
Congenital Disease	SNOMED	400954002	Euryblepharon (disorder)
Congenital Disease	SNOMED	400955001	Epicanthus tarsalis (finding)
Congenital Disease	SNOMED	400956000	Epicanthus inversus (finding)
Congenital Disease	SNOMED	400957009	Epicanthus palpebralis (finding)
Congenital Disease	SNOMED	400962005	Congenital miosis (disorder)
Congenital Disease	SNOMED	400965007	Congenital iris ectropion (disorder)
Congenital Disease	SNOMED	401046009	Nicolaides-Baraitser syndrome (disorder)
Congenital Disease	SNOMED	401138005	Pena-Shokeir syndrome type I (disorder)
Congenital Disease	SNOMED	40130009	Spina bifida without hydrocephalus (disorder)
Congenital Disease	SNOMED	401315004	Smith-Magenis syndrome (disorder)
Congenital Disease	SNOMED	40158001	Papillon-Lefèvre syndrome (disorder)
Congenital Disease	SNOMED	40159009	Congenital macrostomia (disorder)
Congenital Disease	SNOMED	40193008	Single vessel of umbilical cord (disorder)
Congenital Disease	SNOMED	40210001	Floating liver (disorder)
Congenital Disease	SNOMED	402600008	Xeroderma in genetic syndrome (disorder)
Congenital Disease	SNOMED	402681005	Disorder due to abnormality of dermal elastin (disorder)
Congenital Disease	SNOMED	40272001	Congenital absence of coronary sinus (disorder)
Congenital Disease	SNOMED	402767001	Ectodermal syndrome with hair-tooth-sweating defects (disorder)
Congenital Disease	SNOMED	402768006	Ectodermal syndrome with hair-sweating defects (disorder)
Congenital Disease	SNOMED	402770002	Autosomal dominant ichthyosis (disorder)
Congenital Disease	SNOMED	402772005	Autosomal recessive ichthyosis (disorder)
Congenital Disease	SNOMED	402774006	Genetic defect of hair shaft (disorder)
Congenital Disease	SNOMED	402782006	Inherited pseudoxanthoma elasticum (disorder)
Congenital Disease	SNOMED	402808004	Congenital vascular malformation due to inherited syndrome (disorder)
Congenital Disease	SNOMED	402809007	Congenital malformation of lymphatic vessel of skin (disorder)
Congenital Disease	SNOMED	402810002	Developmental malformation of branchial arch (disorder)
Congenital Disease	SNOMED	40291001	Mietens syndrome (disorder)
Congenital Disease	SNOMED	40315008	Annular pancreas (disorder)
Congenital Disease	SNOMED	40320008	Double auditory canal (disorder)
Congenital Disease	SNOMED	403280008	Polyonychia (disorder)
Congenital Disease	SNOMED	403281007	Congenital onychodysplasia of index fingers (disorder)
Congenital Disease	SNOMED	403282000	Congenital malalignment of multiple toenails (disorder)
Congenital Disease	SNOMED	403283005	Congenital pterygium of nail (disorder)
Congenital Disease	SNOMED	403368002	Fordyce spots of lips (disorder)
Congenital Disease	SNOMED	403369005	Fordyce spots of buccal mucosa (disorder)
Congenital Disease	SNOMED	403398009	Cutis laxa following urticaria-angioedema (disorder)
Congenital Disease	SNOMED	403399001	Cutis laxa following hypersensitivity reaction (disorder)
Congenital Disease	SNOMED	403400008	Cutis laxa with complement deficiency (disorder)
Congenital Disease	SNOMED	403438007	Congenital lower lip pits (disorder)
Congenital Disease	SNOMED	403442005	Hereditary mucoepithelial dysplasia (disorder)
Congenital Disease	SNOMED	403460005	Patent vitelline duct (disorder)
Congenital Disease	SNOMED	403461009	Vitelline duct polyp (disorder)
Congenital Disease	SNOMED	403532006	Hypertrichosis with congenital macrogingivae (disorder)
Congenital Disease	SNOMED	403533001	Vertical alopecia (disorder)
Congenital Disease	SNOMED	403534007	Sutural alopecia (disorder)
Congenital Disease	SNOMED	403535008	Triangular alopecia (disorder)
Congenital Disease	SNOMED	40354009	De Lange syndrome (disorder)
Congenital Disease	SNOMED	403542008	Fibrolipomatous hamartoma of nerve (disorder)
Congenital Disease	SNOMED	403543003	Fat-storing hamartoma of dermal dendrocytes (disorder)
Congenital Disease	SNOMED	403544009	Port-wine stain with associated anomalies (disorder)
Congenital Disease	SNOMED	403545005	Port-wine stain with oculocutaneous melanosis (disorder)

Congenital Disease	SNOMED	403548007	Aplasia cutis congenita due to underlying malformation (Type 4) (disorder)
Congenital Disease	SNOMED	403549004	Aplasia cutis congenita associated with fetus papyraceus (Type 5) (disorder)
Congenital Disease	SNOMED	403550004	Aplasia cutis congenita in association with epidermolysis bullosa (Type 6) (disorder)
Congenital Disease	SNOMED	403551000	Aplasia cutis congenita due to teratogenic drug (Type 7) (disorder)
Congenital Disease	SNOMED	403552007	Aplasia cutis congenita following intra-uterine infection (Type 8) (disorder)
Congenital Disease	SNOMED	403553002	Aplasia cutis congenita secondary to malformation syndrome (Type 9) (disorder)
Congenital Disease	SNOMED	403554008	Oculocerebrocutaneous syndrome (disorder)
Congenital Disease	SNOMED	403556005	Constricting band of extremity (disorder)
Congenital Disease	SNOMED	403557001	Midline cervical cleft (disorder)
Congenital Disease	SNOMED	403559003	Cutaneous lesion resulting from spinal dysraphism (disorder)
Congenital Disease	SNOMED	403560008	Port-wine stain associated with spinal dysraphism (disorder)
Congenital Disease	SNOMED	403561007	Cutaneous lesion resulting from spina bifida (disorder)
Congenital Disease	SNOMED	403562000	Rudimentary digit (disorder)
Congenital Disease	SNOMED	403605007	Congenital indifference to pain (finding)
Congenital Disease	SNOMED	403756008	Aplasia cutis in Trisomy 13 syndrome (disorder)
Congenital Disease	SNOMED	403757004	Aplasia cutis in Chromosome 4 short-arm deletion syndrome (Wolf-Hirschhorn) (disorder)
Congenital Disease	SNOMED	403759001	Autosomal chromosomal disorder (disorder)
Congenital Disease	SNOMED	403760006	XXYY syndrome (disorder)
Congenital Disease	SNOMED	403761005	Poly Y syndrome (disorder)
Congenital Disease	SNOMED	403762003	Odonto-onycho-dermal dysplasia (disorder)
Congenital Disease	SNOMED	403763008	Aplasia cutis in Johanson-Blizzard syndrome (disorder)
Congenital Disease	SNOMED	403764002	Odontomicronychial ectodermal dysplasia (disorder)
Congenital Disease	SNOMED	403765001	Port-wine stain in Rubinstein-Taybi syndrome (disorder)
Congenital Disease	SNOMED	403766000	Acromegaloid phenotype with cutis verticis gyrata and corneal leukoma (disorder)
Congenital Disease	SNOMED	403767009	Acrocephalopolysyndactyly type II (disorder)
Congenital Disease	SNOMED	403768004	Acrocephalopolysyndactyly type III (disorder)
Congenital Disease	SNOMED	403769007	Cardio-acral-facial syndrome (disorder)
Congenital Disease	SNOMED	403770008	Cardio-facio-cutaneous syndrome (disorder)
Congenital Disease	SNOMED	403772000	Cleft palate lateral synechia syndrome (disorder)
Congenital Disease	SNOMED	403773005	Facial milia, lobate tongue, lingual and labial frenula syndrome (disorder)
Congenital Disease	SNOMED	403774004	Hereditary sclerosing poikiloderma of Weary (disorder)
Congenital Disease	SNOMED	403775003	Hereditary neurocutaneous angiomata (disorder)
Congenital Disease	SNOMED	403777006	Lamellar ichthyosis (limited type) (disorder)
Congenital Disease	SNOMED	403779009	Ichthyosis, cerebellar degeneration and hepatosplenomegaly (disorder)
Congenital Disease	SNOMED	403780007	Autosomal recessive keratitis-ichthyosis-deafness syndrome (disorder)
Congenital Disease	SNOMED	403781006	Photosensitivity with ichthyosis, brittle hair, impaired intelligence, decreased fertility and short stature syndrome (disorder)
Congenital Disease	SNOMED	403782004	Ichthyosis follicularis with alopecia and photophobia (disorder)
Congenital Disease	SNOMED	403783009	Flexural Darier's disease (disorder)
Congenital Disease	SNOMED	403784003	Hypertrophic Darier's disease (disorder)
Congenital Disease	SNOMED	403785002	Linear/nevoid/zosteriform Darier's disease (disorder)
Congenital Disease	SNOMED	403786001	Acral Darier's disease (disorder)
Congenital Disease	SNOMED	403787005	Palmar pitting due to Darier disease (disorder)
Congenital Disease	SNOMED	403788000	Nail dystrophy due to Darier's disease (disorder)
Congenital Disease	SNOMED	403789008	Nail dystrophy due to benign familial pemphigus (disorder)
Congenital Disease	SNOMED	403790004	Palmoplantar hyperkeratosis-hyperpigmentation syndrome of Cantu (disorder)
Congenital Disease	SNOMED	403792007	Keratoderma due to Dowling-Meara type epidermolysis bullosa simplex (disorder)
Congenital Disease	SNOMED	403794008	Autosomal dominant familial wooly hair (disorder)
Congenital Disease	SNOMED	403795009	Autosomal recessive familial wooly hair (disorder)
Congenital Disease	SNOMED	403796005	Brittle hair-impaired intellect-decreased fertility-short stature syndrome (disorder)
Congenital Disease	SNOMED	403798006	Atrichia congenita (disorder)
Congenital Disease	SNOMED	403799003	Congenital hypertrichosis lanuginosa (disorder)
Congenital Disease	SNOMED	403801000	Pachyonychia congenita type II of Jackson-Lawler (disorder)
Congenital Disease	SNOMED	403802007	Pachyonychia congenita type III of Schafer-Brunauer (disorder)
Congenital Disease	SNOMED	403807001	Phylloid hypomelanosis (disorder)
Congenital Disease	SNOMED	403808006	Generalized recessive non-mutilating dystrophic epidermolysis bullosa (disorder)
Congenital Disease	SNOMED	403809003	Dystrophic epidermolysis bullosa inverse type (disorder)
Congenital Disease	SNOMED	403810008	Epidermolysis bullosa pruriginosa (disorder)
Congenital Disease	SNOMED	403811007	Autosomal dominant pseudoxanthoma elasticum (disorder)
Congenital Disease	SNOMED	403812000	Autosomal recessive pseudoxanthoma elasticum (disorder)
Congenital Disease	SNOMED	403813005	Localized congenital cutis laxa (disorder)
Congenital Disease	SNOMED	403814004	Cutis laxa secondary to inherited disorder of connective tissue (disorder)
Congenital Disease	SNOMED	403815003	Axillary freckling due to neurofibromatosis (disorder)
Congenital Disease	SNOMED	403816002	Multiple café-au-lait macules due to neurofibromatosis (disorder)
Congenital Disease	SNOMED	403817006	Multiple neurofibromas in neurofibromatosis (disorder)
Congenital Disease	SNOMED	403819009	Elephantiasis neurofibromatosa (disorder)
Congenital Disease	SNOMED	403820003	Café-au-lait macules with pulmonary stenosis (disorder)

Congenital Disease	SNOMED	403821004	Café-au-lait macules with temporal dysrhythmia (disorder)
Congenital Disease	SNOMED	403823001	Periungual fibroma in tuberous sclerosis (disorder)
Congenital Disease	SNOMED	403824007	Torré-Muir syndrome (disorder)
Congenital Disease	SNOMED	403855001	Congenital anomaly of digit (disorder)
Congenital Disease	SNOMED	403856000	Port-wine stain in proteus syndrome (disorder)
Congenital Disease	SNOMED	40389006	12q partial trisomy syndrome (disorder)
Congenital Disease	SNOMED	4041005	Congenital anomaly of macula (disorder)
Congenital Disease	SNOMED	404170009	Bullous urticaria pigmentosa (disorder)
Congenital Disease	SNOMED	404173006	Familial mastocytosis (disorder)
Congenital Disease	SNOMED	404625000	Eyelid malposition (disorder)
Congenital Disease	SNOMED	404691000	Tadpole pupil (finding)
Congenital Disease	SNOMED	404692007	Midbrain corectopia (disorder)
Congenital Disease	SNOMED	405752007	Congenital atrial septal defect (disorder)
Congenital Disease	SNOMED	405769009	Klinefelter's syndrome, XXY (disorder)
Congenital Disease	SNOMED	405772002	Congenital kyphoscoliosis (disorder)
Congenital Disease	SNOMED	4061000119104	Myelomeningocele without hydrocephalus (disorder)
Congenital Disease	SNOMED	40627003	Congenital absence of vein (disorder)
Congenital Disease	SNOMED	406428006	Hypertrophy of septomarginal trabeculation (disorder)
Congenital Disease	SNOMED	406476007	Epispadias (disorder)
Congenital Disease	SNOMED	406477003	Male epispadias (disorder)
Congenital Disease	SNOMED	407000	Congenital hepatomegaly (disorder)
Congenital Disease	SNOMED	407552008	Broad epicanthus (finding)
Congenital Disease	SNOMED	408537003	Barber-Say syndrome (disorder)
Congenital Disease	SNOMED	40888008	Congenital anomaly of the hematopoietic system (disorder)
Congenital Disease	SNOMED	40929003	Nevus anemicus (disorder)
Congenital Disease	SNOMED	409709004	Chromosomal disorder (disorder)
Congenital Disease	SNOMED	410065004	Congenital anomaly of aortic arch AND/OR descending aorta (disorder)
Congenital Disease	SNOMED	41040004	Complete trisomy 21 syndrome (disorder)
Congenital Disease	SNOMED	41049003	Holoacardius amorphus (disorder)
Congenital Disease	SNOMED	41069008	Langer-Giedion syndrome (disorder)
Congenital Disease	SNOMED	41132007	Dicephalus tripus tribrachius (disorder)
Congenital Disease	SNOMED	412787009	Intellectual disability, congenital heart disease, blepharophimosis, blepharoptosis and hypoplastic teeth (disorder)
Congenital Disease	SNOMED	41279003	Congenital esophagobronchial fistula (disorder)
Congenital Disease	SNOMED	41283003	Cerebro-oculo-facio-skeletal syndrome (disorder)
Congenital Disease	SNOMED	413221004	Ectopic gastric mucosa - multiple sites (disorder)
Congenital Disease	SNOMED	413237005	Abnormal biliary-pancreatic junction (finding)
Congenital Disease	SNOMED	413255001	Pancreas divisum - complete (disorder)
Congenital Disease	SNOMED	413256000	Pancreas divisum - incomplete (disorder)
Congenital Disease	SNOMED	41337007	Conjoined twins (disorder)
Congenital Disease	SNOMED	4135001	11p partial monosomy syndrome (disorder)
Congenital Disease	SNOMED	4136000	Macrodactyly of toe (disorder)
Congenital Disease	SNOMED	41371000119100	Shone complex (disorder)
Congenital Disease	SNOMED	413728006	Buphthalmos (finding)
Congenital Disease	SNOMED	413808003	Cerebral ventriculomegaly (disorder)
Congenital Disease	SNOMED	413905004	Congenital absence of heart structure (disorder)
Congenital Disease	SNOMED	413936007	Currarino triad (disorder)
Congenital Disease	SNOMED	414133009	Diverticulum of esophagus (disorder)
Congenital Disease	SNOMED	414393003	Hereditary disorder of cellular element of blood (disorder)
Congenital Disease	SNOMED	41443008	Multiple malformation syndrome with limb defect as major feature (disorder)
Congenital Disease	SNOMED	414494005	Infertility due to testicular hypoplasia (disorder)
Congenital Disease	SNOMED	41452004	Uterus acollis (disorder)
Congenital Disease	SNOMED	414667000	Meningomyelocele (disorder)
Congenital Disease	SNOMED	41483000	Multiple malformation syndrome, small stature, without skeletal dysplasia (disorder)
Congenital Disease	SNOMED	41495000	Disseminated superficial actinic porokeratosis (disorder)
Congenital Disease	SNOMED	41505007	Débré-Sémélaigne's syndrome (disorder)
Congenital Disease	SNOMED	41514002	Congenital supravalvular mitral stenosis (disorder)
Congenital Disease	SNOMED	41517009	Congenital hypertrophy of sphenoid bone (disorder)
Congenital Disease	SNOMED	415176004	Primary congenital glaucoma (disorder)
Congenital Disease	SNOMED	4160001	Congenital anomaly of upper respiratory system (disorder)
Congenital Disease	SNOMED	416010008	Hypospadias (disorder)
Congenital Disease	SNOMED	41620007	Congenital anomaly of lacrimal gland (disorder)
Congenital Disease	SNOMED	416377005	Port-wine stain of skin (disorder)
Congenital Disease	SNOMED	41656005	Leri's pleonosteosis syndrome (disorder)
Congenital Disease	SNOMED	416671000	Microspherophakia (disorder)
Congenital Disease	SNOMED	416792008	Vein of Galen malformation (disorder)
Congenital Disease	SNOMED	416834004	Epicapsular star (disorder)
Congenital Disease	SNOMED	4170004	Ehlers-Danlos syndrome, procollagen proteinase resistant (disorder)
Congenital Disease	SNOMED	417078002	Conjunctivitis associated with epidermolysis bullosa (disorder)
Congenital Disease	SNOMED	417125003	Hamartoma of retina (disorder)
Congenital Disease	SNOMED	417161008	Supernumerary canaliculus (disorder)
Congenital Disease	SNOMED	41729002	Horseshoe kidney (disorder)
Congenital Disease	SNOMED	417426005	Four vessels in umbilical cord (disorder)

Congenital Disease	SNOMED	417604002	Axenfeld-Rieger syndrome (disorder)
Congenital Disease	SNOMED	417658006	Holoanencephaly (disorder)
Congenital Disease	SNOMED	417663005	Vertebral facet asymmetry (finding)
Congenital Disease	SNOMED	417893002	Deformity (finding)
Congenital Disease	SNOMED	418087009	Congenital absence of lacrimal drainage structure (disorder)
Congenital Disease	SNOMED	4184009	Congenital anomaly of endocrine gland (disorder)
Congenital Disease	SNOMED	418653000	Secondary lens coloboma (disorder)
Congenital Disease	SNOMED	41878001	Symbrachydactyly (disorder)
Congenital Disease	SNOMED	41893002	Left ventricular-right atrial communication (disorder)
Congenital Disease	SNOMED	41895009	Longitudinal deficiency of carpal bone (disorder)
Congenital Disease	SNOMED	419237004	Ectopia lentis et pupillae (disorder)
Congenital Disease	SNOMED	419281007	Lentiglobus (disorder)
Congenital Disease	SNOMED	419496009	Congenital corneal keloid (disorder)
Congenital Disease	SNOMED	4195003	Congenital duplication of anus (disorder)
Congenital Disease	SNOMED	419544009	Primary lens coloboma (disorder)
Congenital Disease	SNOMED	41962002	Oligohydramnios sequence (disorder)
Congenital Disease	SNOMED	4199009	18p partial trisomy syndrome (disorder)
Congenital Disease	SNOMED	420049001	Congenital abnormality of lacrimal drainage system (disorder)
Congenital Disease	SNOMED	420515004	Retinal venous tortuosity (finding)
Congenital Disease	SNOMED	42162004	Congenital varus deformity of foot (disorder)
Congenital Disease	SNOMED	42190000	Congenital absence of abdominal muscle (disorder)
Congenital Disease	SNOMED	421910007	Teardrop pupil (finding)
Congenital Disease	SNOMED	422311004	Simple ectopia lentis (disorder)
Congenital Disease	SNOMED	422437002	X-linked intellectual disability with marfanoid habitus (disorder)
Congenital Disease	SNOMED	422474003	Partial absence of septum pellucidum (disorder)
Congenital Disease	SNOMED	422731002	Dysgenesis of lacrimal punctum (disorder)
Congenital Disease	SNOMED	42283001	Cochleate uterus (disorder)
Congenital Disease	SNOMED	423095007	Congenital plagiocephaly with pelvic obliquity (disorder)
Congenital Disease	SNOMED	423197001	Arteriovenous abnormality of orbit (disorder)
Congenital Disease	SNOMED	42324007	Congenital duplication of cecum (disorder)
Congenital Disease	SNOMED	423270001	Ectopic lacrimal papilla (disorder)
Congenital Disease	SNOMED	423368008	Congenital pelvic obliquity (disorder)
Congenital Disease	SNOMED	423462008	Dural carotid cavernous fistula (disorder)
Congenital Disease	SNOMED	42376006	Occipital encephalocele (disorder)
Congenital Disease	SNOMED	42402006	Kartagener syndrome (disorder)
Congenital Disease	SNOMED	424045003	Myocardial bridge of coronary artery (disorder)
Congenital Disease	SNOMED	424087001	Congenital ectopia of lacrimal punctum (disorder)
Congenital Disease	SNOMED	4242009	18q partial monosomy syndrome (disorder)
Congenital Disease	SNOMED	424286001	Fibrous dysplasia of orbit (disorder)
Congenital Disease	SNOMED	42432003	Oto-palato-digital syndrome, type II (disorder)
Congenital Disease	SNOMED	424728002	Prepapillary vascular loop (disorder)
Congenital Disease	SNOMED	424889004	Sinistrocardia (disorder)
Congenital Disease	SNOMED	42530008	Xeroderma pigmentosum, group F (disorder)
Congenital Disease	SNOMED	425687007	Spina bifida aperta of cervical spine (disorder)
Congenital Disease	SNOMED	425871007	Congenital spondylolisthesis of cervical vertebra (disorder)
Congenital Disease	SNOMED	42618007	Congenital atresia of fallopian tube (disorder)
Congenital Disease	SNOMED	42666000	Congenital anomaly of tracheal cartilage (disorder)
Congenital Disease	SNOMED	427103005	Congenital contracture of gastrocnemius muscle (disorder)
Congenital Disease	SNOMED	42712003	Cri du chat (finding)
Congenital Disease	SNOMED	427199002	Congenital arteriovenous malformation of small intestine (disorder)
Congenital Disease	SNOMED	427216002	Spina bifida aperta of thoracic spine (disorder)
Congenital Disease	SNOMED	42725006	Achondrogenesis, type IA (disorder)
Congenital Disease	SNOMED	427791009	Congenital velopharyngeal incompetence (disorder)
Congenital Disease	SNOMED	42780004	Congenital lobulation of spleen (disorder)
Congenital Disease	SNOMED	427944007	Congenital asymmetry of forehead (disorder)
Congenital Disease	SNOMED	42808000	Longitudinal deficiency of tibia (disorder)
Congenital Disease	SNOMED	428113000	Autosomal aneuploidy (disorder)
Congenital Disease	SNOMED	428190001	Neurogenic bladder due to spina bifida (disorder)
Congenital Disease	SNOMED	428522008	Congenital anomaly of male urogenital tract (disorder)
Congenital Disease	SNOMED	428543009	Congenital vascular malformation of lip (disorder)
Congenital Disease	SNOMED	42866003	Congenital coronary artery sclerosis (disorder)
Congenital Disease	SNOMED	428680008	Congenital female urogenital anomaly (disorder)
Congenital Disease	SNOMED	428850001	Li-Fraumeni syndrome (disorder)
Congenital Disease	SNOMED	428862002	Congenital flat back deformity (disorder)
Congenital Disease	SNOMED	429442006	Autosomal duplication (disorder)
Congenital Disease	SNOMED	429448005	Congenital anomaly of anterior segment of eye (disorder)
Congenital Disease	SNOMED	429449002	Congenital hypoplasia of fovea centralis (disorder)
Congenital Disease	SNOMED	429466000	Spina bifida aperta of lumbar spine (disorder)
Congenital Disease	SNOMED	429631000124104	Glanular hypospadias (disorder)
Congenital Disease	SNOMED	429641000124109	Coronal hypospadias (disorder)
Congenital Disease	SNOMED	429651000124106	Penile shaft hypospadias (disorder)
Congenital Disease	SNOMED	429661000124108	Penile mid-shaft hypospadias (disorder)
Congenital Disease	SNOMED	429753001	Congenital nonprogressive myopathy with Moebius and Robin sequences (disorder)
Congenital Disease	SNOMED	429967001	Cryptotia (disorder)

Congenital Disease	SNOMED	430031008	Fetal hydronephrosis (disorder)
Congenital Disease	SNOMED	430166008	Congenital anomaly of peripheral blood vessel (disorder)
Congenital Disease	SNOMED	43036001	Ectromelia (disorder)
Congenital Disease	SNOMED	43039008	Mesatipellic pelvis (disorder)
Congenital Disease	SNOMED	43063000	Congenital atresia of osseous meatus of middle ear (disorder)
Congenital Disease	SNOMED	430686008	Spinal dysgenesis (disorder)
Congenital Disease	SNOMED	430904007	Basilar skull invagination (disorder)
Congenital Disease	SNOMED	431040002	Velopharyngeal incompetence due to cleft palate (disorder)
Congenital Disease	SNOMED	431265009	Fetal microcephaly (disorder)
Congenital Disease	SNOMED	4313003	Acardiacus anceps (disorder)
Congenital Disease	SNOMED	431395004	Vertebral abnormalities, anal atresia, cardiac abnormalities, tracheo-esophageal fistula, renal anomalies, limb defects syndrome (disorder)
Congenital Disease	SNOMED	43176009	Congenital hypoplasia of tricuspid valve (disorder)
Congenital Disease	SNOMED	431768007	Congenital malformation of sphenoid wing (disorder)
Congenital Disease	SNOMED	431770003	Fetal macrocephaly (disorder)
Congenital Disease	SNOMED	432293006	Congenital anomaly of blood vessel of upper limb (disorder)
Congenital Disease	SNOMED	432461000	Congenital anomaly of renal blood vessel (disorder)
Congenital Disease	SNOMED	43248007	Penta X syndrome (disorder)
Congenital Disease	SNOMED	4325000	11q partial monosomy syndrome (disorder)
Congenital Disease	SNOMED	432734004	Congenital asymmetry of breasts (finding)
Congenital Disease	SNOMED	432993002	Congenital absence of quadriceps muscle (disorder)
Congenital Disease	SNOMED	43353004	Congenital anomaly of inner ear (disorder)
Congenital Disease	SNOMED	43383008	Congenital diverticulum of bronchus (disorder)
Congenital Disease	SNOMED	43387009	Fetus in fetu (disorder)
Congenital Disease	SNOMED	43420005	9q partial monosomy syndrome (disorder)
Congenital Disease	SNOMED	43427008	Ectopic glial tissue (disorder)
Congenital Disease	SNOMED	43437003	Submucous cleft of hard palate (disorder)
Congenital Disease	SNOMED	43476002	Brachydactyly (disorder)
Congenital Disease	SNOMED	43477006	Xeroderma pigmentosum, group A (disorder)
Congenital Disease	SNOMED	43557002	Congenital stricture of external auditory canal (disorder)
Congenital Disease	SNOMED	43814000	Atelosteogenesis (disorder)
Congenital Disease	SNOMED	438504004	Lenz microphthalmia syndrome (disorder)
Congenital Disease	SNOMED	438583008	Congenital bilateral perisylvian syndrome (disorder)
Congenital Disease	SNOMED	43876007	Situs inversus viscerum (disorder)
Congenital Disease	SNOMED	43910005	Congenital hypoplasia of aorta (disorder)
Congenital Disease	SNOMED	439143004	Simpson-Golabi-Behmel syndrome (disorder)
Congenital Disease	SNOMED	439258003	Deformity of toe due to amniotic band (disorder)
Congenital Disease	SNOMED	43929004	Smith-Lemli-Opitz syndrome (disorder)
Congenital Disease	SNOMED	4397001	Partial congenital duodenal obstruction (disorder)
Congenital Disease	SNOMED	440350001	Fibroblast growth factor receptor 3-related craniosynostosis (disorder)
Congenital Disease	SNOMED	440391004	Congenital deformity of scapula (disorder)
Congenital Disease	SNOMED	4406004	Congenital anomaly of male genital system (disorder)
Congenital Disease	SNOMED	440624006	Deformity of digit of hand due to amniotic band (disorder)
Congenital Disease	SNOMED	441686004	Delta phalanx of finger (disorder)
Congenital Disease	SNOMED	441944007	Oto-onycho-peroneal syndrome (disorder)
Congenital Disease	SNOMED	44215001	Radiation chimera (disorder)
Congenital Disease	SNOMED	442300000	Rhombencephalosynapsis (disorder)
Congenital Disease	SNOMED	44295002	Congenital coloboma of optic disc (disorder)
Congenital Disease	SNOMED	44315004	Congenital absence of auricle with stenosis of auditory canal (disorder)
Congenital Disease	SNOMED	443379009	Functional single ventricle (disorder)
Congenital Disease	SNOMED	4434006	Bloom syndrome (disorder)
Congenital Disease	SNOMED	44444001	Longitudinal deficiency of ulna (disorder)
Congenital Disease	SNOMED	444550009	Developmental anomaly of odontoid process of axis (disorder)
Congenital Disease	SNOMED	444558002	Infantile nephronophthisis (disorder)
Congenital Disease	SNOMED	444655009	Extra unidentified structurally abnormal chromosome (disorder)
Congenital Disease	SNOMED	444667006	Congenital deformity of pharynx (disorder)
Congenital Disease	SNOMED	444668001	Congenital scleral show (finding)
Congenital Disease	SNOMED	444749006	Adolescent nephronophthisis (disorder)
Congenital Disease	SNOMED	444830001	Juvenile nephronophthisis (disorder)
Congenital Disease	SNOMED	444844007	Congenital aplasia of odontoid process (disorder)
Congenital Disease	SNOMED	444851003	Bifid apex of heart (disorder)
Congenital Disease	SNOMED	444858009	Unbalanced translocation of chromosome (disorder)
Congenital Disease	SNOMED	444860006	Meningomyelocele of lumbosacral spine (disorder)
Congenital Disease	SNOMED	444879009	Stenosis of foramen magnum (disorder)
Congenital Disease	SNOMED	444921008	Subglottic web (disorder)
Congenital Disease	SNOMED	444961009	Atrioventricular septal defect with common atrioventricular orifice (disorder)
Congenital Disease	SNOMED	444964001	Lungs in mirror image arrangement (disorder)
Congenital Disease	SNOMED	444978000	Meningocele of vertex (disorder)
Congenital Disease	SNOMED	445001003	Left ventricular outflow tract obstruction due to malaligned outlet septum (disorder)
Congenital Disease	SNOMED	445002005	Atrioventricular septal defect with separate atrioventricular orifices (disorder)
Congenital Disease	SNOMED	445003000	Left ventricular outflow tract obstruction due to subpulmonary fibromuscular shelf (disorder)
Congenital Disease	SNOMED	445027003	Left superior caval vein persisting to coronary sinus (disorder)

Congenital Disease	SNOMED	445106006	Congenital dilation of left pulmonary artery (disorder)
Congenital Disease	SNOMED	445109004	Isolation of left common carotid artery (disorder)
Congenital Disease	SNOMED	445116003	Encephalocele of vertex (disorder)
Congenital Disease	SNOMED	44513007	Congenital anomaly of the kidney (disorder)
Congenital Disease	SNOMED	445131007	Left ventricular outflow tract obstruction due to fibromuscular tunnel (disorder)
Congenital Disease	SNOMED	445135003	Angiomatosis of meninges (disorder)
Congenital Disease	SNOMED	445162006	Bleeding Meckel's diverticulitis (disorder)
Congenital Disease	SNOMED	445163001	Bleeding Meckel's diverticulum (disorder)
Congenital Disease	SNOMED	445167000	Congenital dilation of right pulmonary artery (disorder)
Congenital Disease	SNOMED	445175006	Symbrachydactyly of toe (disorder)
Congenital Disease	SNOMED	445176007	Congenital dilation of ascending aorta (disorder)
Congenital Disease	SNOMED	44518003	Celosomus (disorder)
Congenital Disease	SNOMED	445208002	Congenital hypoplasia of right pulmonary artery (disorder)
Congenital Disease	SNOMED	445209005	Congenital hypoplasia of left pulmonary artery (disorder)
Congenital Disease	SNOMED	445235006	Left ventricular outflow tract obstruction due to anterolateral muscle band (disorder)
Congenital Disease	SNOMED	445251003	Os acromiale (disorder)
Congenital Disease	SNOMED	445257004	Nance-Horan syndrome (disorder)
Congenital Disease	SNOMED	445264002	Atrioventricular septal defect with ventricular imbalance consisting of dominant left ventricle and hypoplastic right ventricle (disorder)
Congenital Disease	SNOMED	445268004	Apex of heart anterior to cardiac base (disorder)
Congenital Disease	SNOMED	445270008	Aortic orifice left side by side with respect to pulmonary orifice (disorder)
Congenital Disease	SNOMED	445284003	Aortic sinus of Valsalva aneurysm from noncoronary sinus (disorder)
Congenital Disease	SNOMED	445285002	Aortic sinus of Valsalva aneurysm from left coronary sinus (disorder)
Congenital Disease	SNOMED	445286001	Aortic sinus of Valsalva aneurysm from right coronary sinus (disorder)
Congenital Disease	SNOMED	445294008	Common atrioventricular orifice in double inlet ventricle (disorder)
Congenital Disease	SNOMED	445296005	Infracardiac location of anomalous pulmonary venous connections to portal system (disorder)
Congenital Disease	SNOMED	445298006	Aortic orifice anterior with respect to pulmonary orifice (disorder)
Congenital Disease	SNOMED	445299003	Atrioventricular septal defect with ventricular imbalance consisting of dominant right ventricle and hypoplastic left ventricle (disorder)
Congenital Disease	SNOMED	445307009	Spina bifida of sacral region (disorder)
Congenital Disease	SNOMED	445308004	Split spinal cord malformation (disorder)
Congenital Disease	SNOMED	445310002	Left ventricular outflow tract obstruction due to diaphragm (disorder)
Congenital Disease	SNOMED	445330003	Right atrioventricular valve leaflets absent in double inlet ventricle (unguarded orifice) (disorder)
Congenital Disease	SNOMED	445334007	Atrioventricular septal defect with additional muscular ventricular septal defect (disorder)
Congenital Disease	SNOMED	445338005	Testicular dysgenesis syndrome (disorder)
Congenital Disease	SNOMED	445349004	Isolation of right common carotid artery (disorder)
Congenital Disease	SNOMED	445350004	Isolation of left subclavian artery (disorder)
Congenital Disease	SNOMED	445351000	Isolation of right subclavian artery (disorder)
Congenital Disease	SNOMED	445371009	Infracardiac location of anomalous pulmonary venous connections to inferior caval vein (disorder)
Congenital Disease	SNOMED	445373007	Aortic orifice posterior with respect to pulmonary orifice (disorder)
Congenital Disease	SNOMED	445390001	Dysplasia of testis (disorder)
Congenital Disease	SNOMED	445431000	Frasier syndrome (disorder)
Congenital Disease	SNOMED	445435009	Apex of heart posterior to cardiac base (disorder)
Congenital Disease	SNOMED	445436005	Left superior caval vein persisting to left sided atrium (disorder)
Congenital Disease	SNOMED	445453003	Left ventricular outflow tract obstruction due to aneurysm of membranous septum (disorder)
Congenital Disease	SNOMED	445454009	Left ventricular outflow tract obstruction due to atrioventricular valve (disorder)
Congenital Disease	SNOMED	445468002	Occipital meningocele (disorder)
Congenital Disease	SNOMED	445486004	Congenital malformation of blood vessel of orbit proper (disorder)
Congenital Disease	SNOMED	445539001	Right ventricular diverticulum (disorder)
Congenital Disease	SNOMED	445540004	Left ventricular diverticulum (disorder)
Congenital Disease	SNOMED	445543002	Intracardiac location of anomalous pulmonary venous connections to bilateral isomeric atriums (disorder)
Congenital Disease	SNOMED	445554007	Congenital asymmetry of tonsils (disorder)
Congenital Disease	SNOMED	445580008	Familial extra unidentified structurally abnormal chromosome (disorder)
Congenital Disease	SNOMED	445607003	Aortic orifice posterior left with respect to pulmonary orifice (disorder)
Congenital Disease	SNOMED	445636003	Aortic orifice anterior right with respect to pulmonary orifice (disorder)
Congenital Disease	SNOMED	445650008	Aortic orifice anterior left with respect to pulmonary orifice (disorder)
Congenital Disease	SNOMED	445898001	Abnormal atrial arrangement (disorder)
Congenital Disease	SNOMED	44593008	Orbital separation diminished (disorder)
Congenital Disease	SNOMED	44600005	Xeroderma pigmentosum (disorder)
Congenital Disease	SNOMED	446112008	Cyst of pinna (disorder)
Congenital Disease	SNOMED	44621005	Congenital anomaly of organ of Corti (disorder)
Congenital Disease	SNOMED	44622003	Congenital hypoplasia of umbilical artery (disorder)
Congenital Disease	SNOMED	446263001	Loeys-Dietz syndrome (disorder)
Congenital Disease	SNOMED	446326008	Premature restriction of foramen ovale (disorder)
Congenital Disease	SNOMED	446432002	Pulmonary venous hypoplasia (disorder)
Congenital Disease	SNOMED	446449009	Renal coloboma syndrome (disorder)

Congenital Disease	SNOMED	44647001	Persistent hyaloid artery (disorder)
Congenital Disease	SNOMED	4465002	Spherophakia (disorder)
Congenital Disease	SNOMED	446628002	Tricuspid leaflet gelatinous (disorder)
Congenital Disease	SNOMED	446630000	Tricuspid leaflet noncoapting (disorder)
Congenital Disease	SNOMED	446656007	Mirror image bronchial anatomy (disorder)
Congenital Disease	SNOMED	446657003	Criss-cross heart with rightward rotation (disorder)
Congenital Disease	SNOMED	446659000	Ventricular imbalance with dominant left ventricle and hypoplastic right ventricle (disorder)
Congenital Disease	SNOMED	446667008	Two atrioventricular valves in double inlet ventricle (disorder)
Congenital Disease	SNOMED	446670007	Aortic orifice right side by side with respect to pulmonary orifice (disorder)
Congenital Disease	SNOMED	446699002	Tricuspid leaflet deficiency (disorder)
Congenital Disease	SNOMED	446781004	Tricuspid leaflet flail (disorder)
Congenital Disease	SNOMED	446786009	Tricuspid leaflet fenestration (disorder)
Congenital Disease	SNOMED	446890001	Obstructed pulmonary venous connection at coronary sinus orifice (disorder)
Congenital Disease	SNOMED	446909006	Midline apex of heart (disorder)
Congenital Disease	SNOMED	446916007	Functionally univentricular heart (disorder)
Congenital Disease	SNOMED	44697002	Melorheostosis (disorder)
Congenital Disease	SNOMED	446989009	Nephronophthisis type 4 (disorder)
Congenital Disease	SNOMED	446991001	Nephronophthisis type 5 (disorder)
Congenital Disease	SNOMED	447085008	Functionally congenital single lung (disorder)
Congenital Disease	SNOMED	44710007	Anomaly of chromosome pair 6 (disorder)
Congenital Disease	SNOMED	447269006	Persistent common pulmonary vein (disorder)
Congenital Disease	SNOMED	447270007	Spontaneous closure of foramen ovale (finding)
Congenital Disease	SNOMED	447275002	Alveolar capillary dysplasia with pulmonary venous misalignment (disorder)
Congenital Disease	SNOMED	447283008	Solitary ventricle of indeterminate morphology (disorder)
Congenital Disease	SNOMED	447284002	Double outlet right ventricle with intact ventricular septum (disorder)
Congenital Disease	SNOMED	447285001	Ventricular imbalance (disorder)
Congenital Disease	SNOMED	447286000	Ventricular imbalance with dominant right ventricle and hypoplastic left ventricle (disorder)
Congenital Disease	SNOMED	447289007	Criss-cross heart with leftward rotation (disorder)
Congenital Disease	SNOMED	447335007	Nephronophthisis type 6 (disorder)
Congenital Disease	SNOMED	447660003	Retroaortic brachiocephalic vein (disorder)
Congenital Disease	SNOMED	447661004	Diverticulum of coronary sinus (disorder)
Congenital Disease	SNOMED	447663001	Pulmonary venous confluence remote from left atrium (disorder)
Congenital Disease	SNOMED	447664007	Partial anomalous pulmonary venous connection of part of left lung (disorder)
Congenital Disease	SNOMED	447665008	Interatrial communication through coronary sinus orifice (disorder)
Congenital Disease	SNOMED	447666009	Divided left atrium with all pulmonary veins to proximal chamber and then to left atrium (disorder)
Congenital Disease	SNOMED	447667000	Divided left atrium with all pulmonary veins to proximal chamber without communication to left atrium (disorder)
Congenital Disease	SNOMED	447668005	Discontinuous pulmonary arteries (disorder)
Congenital Disease	SNOMED	447669002	Hypoplasia of left heart without intrinsic valve stenosis and without atresia (disorder)
Congenital Disease	SNOMED	447670001	Ventricular septal defect with anterior malaligned outlet septum with overriding pulmonary valve (disorder)
Congenital Disease	SNOMED	447671002	Ventricular septal defect with posterior malaligned outlet septum with overriding aortic valve (disorder)
Congenital Disease	SNOMED	447672009	Ventricular septal defect with posterior malaligned outlet septum with overriding pulmonary valve (disorder)
Congenital Disease	SNOMED	447673004	Right ventricle to left of left ventricle (disorder)
Congenital Disease	SNOMED	447674005	Right bronchial isomerism (disorder)
Congenital Disease	SNOMED	447675006	Spontaneous closure of ventricular septal defect due to fibromuscular reaction (finding)
Congenital Disease	SNOMED	447681003	Continuity between mitral valve and pulmonary valve (disorder)
Congenital Disease	SNOMED	447682005	Discontinuity between mitral valve and aortic valve (disorder)
Congenital Disease	SNOMED	447683000	Discontinuity between mitral valve and pulmonary valve (disorder)
Congenital Disease	SNOMED	447689001	Prolapse of Eustachian valve through atrial septum (disorder)
Congenital Disease	SNOMED	447690005	Prolapse of Eustachian valve through tricuspid valve (disorder)
Congenital Disease	SNOMED	447691009	Pulmonary venous confluence in direct proximity to left atrium (disorder)
Congenital Disease	SNOMED	447695000	Tricuspid truncal valve (disorder)
Congenital Disease	SNOMED	447696004	Unicommissural unicuspid aortic valve (disorder)
Congenital Disease	SNOMED	447697008	Unicommissural unicuspid pulmonary valve (disorder)
Congenital Disease	SNOMED	447698003	Ventricular septal defect with anterior malaligned outlet septum with overriding aortic valve (disorder)
Congenital Disease	SNOMED	447700007	Distal aortopulmonary window with minimal superior rim (disorder)
Congenital Disease	SNOMED	447701006	Intermediate aortopulmonary window with adequate superior and inferior rim (disorder)
Congenital Disease	SNOMED	447702004	Confluent aortopulmonary window with minimal superior and inferior rim (disorder)
Congenital Disease	SNOMED	447703009	Double aortic arch with left arch dominant and coarctation of right arch (disorder)
Congenital Disease	SNOMED	447772003	Persisting fifth aortic arch with double barrell arch (disorder)
Congenital Disease	SNOMED	447773008	Proximal aortopulmonary window with minimal inferior rim (disorder)
Congenital Disease	SNOMED	447774002	Congenital midvalvar ring of mitral leaflet (disorder)

Congenital Disease	SNOMED	447777009	Spontaneous closure of ventricular component of atrioventricular septal defect (finding)
Congenital Disease	SNOMED	447778004	Spontaneous reduction in size of ventricular septal defect (finding)
Congenital Disease	SNOMED	447779007	Trifoliate left atrioventricular valve (disorder)
Congenital Disease	SNOMED	447780005	Restrictive interatrial communication with obligatory shunt (disorder)
Congenital Disease	SNOMED	447810006	Congenital stenosis of trachea due to complete rings (disorder)
Congenital Disease	SNOMED	447811005	Congenital stenosis of trachea due to tracheal web (disorder)
Congenital Disease	SNOMED	447812003	Left superior vena cava persisting to right atrium and left atrium (disorder)
Congenital Disease	SNOMED	447813008	Pulmonary venous confluence in horizontal orientation (disorder)
Congenital Disease	SNOMED	447814002	Pulmonary venous confluence in vertical orientation (disorder)
Congenital Disease	SNOMED	447817009	Obstruction of aortic arch (disorder)
Congenital Disease	SNOMED	447821002	Congenital posterolateral diaphragmatic hernia (disorder)
Congenital Disease	SNOMED	447822009	Congenital prolapse of aortic valve (disorder)
Congenital Disease	SNOMED	447823004	Congenital abnormality of hepatic vein (disorder)
Congenital Disease	SNOMED	447824005	Congenital abnormality of left atrium (disorder)
Congenital Disease	SNOMED	447825006	Congenital abnormality of middle cardiac vein (disorder)
Congenital Disease	SNOMED	447827003	Partial anomalous pulmonary venous connection of entire right lung (disorder)
Congenital Disease	SNOMED	447829000	Congenital abnormality of tricuspid chordae tendinae (disorder)
Congenital Disease	SNOMED	447830005	Congenital abnormality of tricuspid leaflet (disorder)
Congenital Disease	SNOMED	447832002	Total anomalous pulmonary venous connection of supracardiac type (disorder)
Congenital Disease	SNOMED	447838003	Pulmonary atresia and ventricular septal defect with aorta from left ventricle (disorder)
Congenital Disease	SNOMED	447839006	Pulmonary atresia and ventricular septal defect with aorta from right ventricle (disorder)
Congenital Disease	SNOMED	447840008	Stenosis of right pulmonary artery (disorder)
Congenital Disease	SNOMED	447845003	Right ventricle posterior to left ventricle (disorder)
Congenital Disease	SNOMED	447846002	Obstruction of ascending aorta (disorder)
Congenital Disease	SNOMED	447847006	Prolapse of right coronary aortic valve cusp (disorder)
Congenital Disease	SNOMED	447849009	Double aortic arch with right arch dominant and atresia of left arch (disorder)
Congenital Disease	SNOMED	447850009	Double aortic arch with right arch dominant and coarctation of left arch (disorder)
Congenital Disease	SNOMED	447851008	Congenital tracheoesophageal fistula with esophageal stenosis (disorder)
Congenital Disease	SNOMED	447852001	Ventricular septal defect of inlet of right aspect of ventricular septum (disorder)
Congenital Disease	SNOMED	447860000	Partial anomalous pulmonary venous connection of part of right lung (disorder)
Congenital Disease	SNOMED	447861001	Partial anomalous pulmonary venous connection with anomalous veins connecting first to pulmonary venous confluence (disorder)
Congenital Disease	SNOMED	447874007	Congenital abnormality of atrial septum (disorder)
Congenital Disease	SNOMED	447875008	Congenital mass of mitral leaflet (disorder)
Congenital Disease	SNOMED	447876009	Congenital aneurysm of subaortic left ventricle (disorder)
Congenital Disease	SNOMED	447901004	Aortopulmonary window with tubular connection (disorder)
Congenital Disease	SNOMED	447902006	Atresia of left superior vena cava (disorder)
Congenital Disease	SNOMED	447903001	Coarctation of right pulmonary artery (disorder)
Congenital Disease	SNOMED	447913009	Completely unroofed coronary sinus defect in left atrium (disorder)
Congenital Disease	SNOMED	447914003	Total anomalous pulmonary venous connection of intracardiac type (disorder)
Congenital Disease	SNOMED	447915002	Ebstein's anomaly of left sided tricuspid valve with discordant atrioventricular connections (disorder)
Congenital Disease	SNOMED	447917005	Liver in central position (disorder)
Congenital Disease	SNOMED	447919008	Univentricular atrioventricular connection with absent right sided atrioventricular connection (disorder)
Congenital Disease	SNOMED	447923000	Thin cardiac ventricular septum (disorder)
Congenital Disease	SNOMED	447925007	Hypoperfusion of left pulmonary artery due to preferential flow to right pulmonary artery (disorder)
Congenital Disease	SNOMED	447926008	Hypoperfusion of right pulmonary artery due to preferential flow to left pulmonary artery (disorder)
Congenital Disease	SNOMED	447928009	Double aortic arch with balanced arches (disorder)
Congenital Disease	SNOMED	447929001	Double aortic arch with left arch dominant (disorder)
Congenital Disease	SNOMED	447930006	Double aortic arch with right arch dominant (disorder)
Congenital Disease	SNOMED	447932003	Double outlet ventriculoarterial connections (disorder)
Congenital Disease	SNOMED	447933008	Nonfenestrated interatrial communication within oval fossa (disorder)
Congenital Disease	SNOMED	447937009	Congenital abnormality of aortic valve cusp (disorder)
Congenital Disease	SNOMED	447938004	Congenital abnormality of cardiac vein (disorder)
Congenital Disease	SNOMED	447939007	Partial anomalous pulmonary venous connection of entire left lung (disorder)
Congenital Disease	SNOMED	447941008	Residual ventricular septal defect (disorder)
Congenital Disease	SNOMED	447942001	Spontaneous closure of ventricular septal defect due to tissue of membranous septum (finding)
Congenital Disease	SNOMED	447943006	Tripartite right ventricle (disorder)
Congenital Disease	SNOMED	44796002	Ectopic ureter (disorder)

Congenital Disease	SNOMED	447962009	Divided left atrium with restrictive outlet of proximal chamber to left atrium (disorder)
Congenital Disease	SNOMED	447968008	Descending aorta anterior and same side as azygos vein with azygos continuity of inferior vena cava (disorder)
Congenital Disease	SNOMED	447970004	Double aortic arch with left arch dominant and atresia of right arch (disorder)
Congenital Disease	SNOMED	447988007	Common arterial trunk with pulmonary arteries arising from trunk and unobstructed aortic arch (disorder)
Congenital Disease	SNOMED	447997006	Vascular ring with retrotracheal right pulmonary artery from ascending aorta (disorder)
Congenital Disease	SNOMED	447998001	Single ventricular outlet above right ventricle (disorder)
Congenital Disease	SNOMED	447999009	Single ventricular outlet above left ventricle (disorder)
Congenital Disease	SNOMED	448000003	Right superior vena cava connecting to left atrium and right atrium (disorder)
Congenital Disease	SNOMED	448001004	Right ventricular outflow tract obstruction due to aneurysm of membranous septum (disorder)
Congenital Disease	SNOMED	448004007	Acommissural unicuspid pulmonary valve (disorder)
Congenital Disease	SNOMED	448005008	Atresia of systemic vein (disorder)
Congenital Disease	SNOMED	448007000	Atrioventricular septal defect associated with tetralogy of Fallot (disorder)
Congenital Disease	SNOMED	448016001	Congenital aneurysm of systemic artery (disorder)
Congenital Disease	SNOMED	448026008	Left atrioventricular valve bifoliate with fused left sided superior and inferior bridging leaflet (disorder)
Congenital Disease	SNOMED	448027004	Supravalvar pulmonary trunk stenosis (disorder)
Congenital Disease	SNOMED	448028009	Congenital left ventricular submitral valve aneurysm (disorder)
Congenital Disease	SNOMED	448045004	Fragile X associated tremor ataxia syndrome (disorder)
Congenital Disease	SNOMED	448059006	Pulmonary trunk absent with absent left pulmonary artery (disorder)
Congenital Disease	SNOMED	448060001	Pulmonary trunk absent with absent right pulmonary artery (disorder)
Congenital Disease	SNOMED	448061002	Shelf-like supravalvar aortic stenosis (disorder)
Congenital Disease	SNOMED	448062009	Ventricular septal defect with malaligned outlet septum (disorder)
Congenital Disease	SNOMED	448063004	Congenital abnormality of posterior cardiac vein of left ventricle (disorder)
Congenital Disease	SNOMED	448064005	Congenital abnormality of right atrium (disorder)
Congenital Disease	SNOMED	448065006	Congenital abnormality of systemic vein (disorder)
Congenital Disease	SNOMED	448066007	Divided left atrium with nonrestrictive outlet of proximal chamber to left atrium (disorder)
Congenital Disease	SNOMED	448067003	Recoarctation of aorta (disorder)
Congenital Disease	SNOMED	448072007	Single inlet ventricle with absent atrioventricular connection (disorder)
Congenital Disease	SNOMED	448073002	Congenital abnormality of tricuspid papillary muscle (disorder)
Congenital Disease	SNOMED	448074008	Ebstein's anomaly with functional tricuspid stenosis (disorder)
Congenital Disease	SNOMED	448075009	Uniatrial biventricular connection with absent right sided atrioventricular connection with straddling valve (disorder)
Congenital Disease	SNOMED	448078006	Vascular ring with right aortic arch and right arterial ligament with absent left pulmonary artery (disorder)
Congenital Disease	SNOMED	448079003	Vascular ring with right aortic arch and right patent arterial duct with absent left pulmonary artery (disorder)
Congenital Disease	SNOMED	448080000	Single ventricular outlet above ventricle of indeterminate morphology (disorder)
Congenital Disease	SNOMED	448081001	Hepatic vein to coronary sinus (disorder)
Congenital Disease	SNOMED	448082008	Right ventricular outflow tract obstruction due to prolapse of Eustachian valve (disorder)
Congenital Disease	SNOMED	448083003	Right ventricular outflow tract obstruction due to prolapsed arterial valve (disorder)
Congenital Disease	SNOMED	448084009	Absent pulmonary trunk (disorder)
Congenital Disease	SNOMED	448085005	Acommissural unicuspid aortic valve (disorder)
Congenital Disease	SNOMED	448086006	Atresia of pulmonary trunk with absent left pulmonary artery (disorder)
Congenital Disease	SNOMED	448087002	Atresia of pulmonary trunk with absent right pulmonary artery (disorder)
Congenital Disease	SNOMED	448092000	Vascular ring due to aberrant subclavian artery and bilateral arterial ducts (disorder)
Congenital Disease	SNOMED	448095003	Aortic left ventricular tunnel with right coronary artery from tunnel (disorder)
Congenital Disease	SNOMED	448096002	Aberrant origin of left subclavian artery (disorder)
Congenital Disease	SNOMED	448097006	Abnormal course of aortic arch (disorder)
Congenital Disease	SNOMED	448098001	Aneurysm of aortic sinus of Valsalva with protrusion into pulmonary artery (disorder)
Congenital Disease	SNOMED	448099009	Aneurysm of aortic sinus of Valsalva with protrusion into right atrium (disorder)
Congenital Disease	SNOMED	448100001	Aneurysm of aortic sinus of Valsalva with protrusion into right ventricle (disorder)
Congenital Disease	SNOMED	448102009	Quadricuspid truncal valve (disorder)
Congenital Disease	SNOMED	448103004	Right ventricle inferior to left ventricle (disorder)
Congenital Disease	SNOMED	448104005	Localized supravalvar aortic stenosis at sinutubular junction (disorder)
Congenital Disease	SNOMED	448105006	Anomalous origin of pulmonary artery from patent arterial duct (disorder)
Congenital Disease	SNOMED	448113007	Right superior vena cava connecting to coronary sinus (disorder)
Congenital Disease	SNOMED	448115000	Aneurysm of aortic sinus of Valsalva with protrusion into left atrium (disorder)
Congenital Disease	SNOMED	448116004	Aneurysm of aortic sinus of Valsalva with protrusion into left ventricle (disorder)

Congenital Disease	SNOMED	448117008	Aneurysm of aortic sinus of Valsalva with protrusion into pericardial cavity (disorder)
Congenital Disease	SNOMED	448118003	Atrioventricular septal defect with atrial and ventricular components and separate atrioventricular valves (disorder)
Congenital Disease	SNOMED	448119006	Atrioventricular septal defect with atrioventricular valve regurgitation (disorder)
Congenital Disease	SNOMED	448120000	Common arterial trunk with crossed over pulmonary arteries (disorder)
Congenital Disease	SNOMED	448153005	Vascular ring with left aortic arch and right arterial duct arising from aberrant retroesophageal right subclavian artery (disorder)
Congenital Disease	SNOMED	448154004	Vascular ring with left aortic arch and right arterial duct arising from retroesophageal aortic diverticulum (disorder)
Congenital Disease	SNOMED	448155003	Pulmonary atresia with ventricular septal defect of Fallot type (disorder)
Congenital Disease	SNOMED	448159009	Abnormality of tricuspid subvalvar apparatus (disorder)
Congenital Disease	SNOMED	448160004	Aortic orifice to posterior right of pulmonary orifice (finding)
Congenital Disease	SNOMED	448161000	Aortic valve overriding ventricular septum (disorder)
Congenital Disease	SNOMED	448162007	Bipartite right ventricle (disorder)
Congenital Disease	SNOMED	448164008	Cecum in left sided position (disorder)
Congenital Disease	SNOMED	448181004	Anomalous coronary venous return (disorder)
Congenital Disease	SNOMED	448182006	Atrioventricular septal defect with ventricular component and interchordal shunting under inferior bridging leaflet (disorder)
Congenital Disease	SNOMED	448183001	Atrioventricular septal defect with ventricular component and interchordal shunting under superior bridging leaflet (disorder)
Congenital Disease	SNOMED	448184007	Atrioventricular septal defect with ventricular component and shunting under connecting tongue with separate orifices (disorder)
Congenital Disease	SNOMED	448201009	Spontaneous closure of ventricular septal defect due to accessory tissue of atrioventricular valve (finding)
Congenital Disease	SNOMED	448202002	Left bronchial isomerism (disorder)
Congenital Disease	SNOMED	448208003	Nonrestrictive interatrial communication (disorder)
Congenital Disease	SNOMED	448227009	X-linked periventricular heterotopia (disorder)
Congenital Disease	SNOMED	448277007	Midline posterior apex of heart (disorder)
Congenital Disease	SNOMED	448278002	Coronary sinus drainage cephalad to left superior vena cava (disorder)
Congenital Disease	SNOMED	448279005	Right ventricle anterior to left ventricle (finding)
Congenital Disease	SNOMED	448280008	Malalignment of aortic sinus in relation to pulmonary sinus (disorder)
Congenital Disease	SNOMED	448281007	Abnormality of hepatic vein (disorder)
Congenital Disease	SNOMED	448303009	Vascular ring with left aortic arch to right descending aorta and right arterial ligament (disorder)
Congenital Disease	SNOMED	448304003	Vascular ring with left aortic arch to right descending aorta and right patent arterial duct (disorder)
Congenital Disease	SNOMED	448305002	Vascular ring with malrotation and dextroversion of heart and hypoplasia of right lung and left arterial duct (disorder)
Congenital Disease	SNOMED	448308000	Right ventricular outflow tract obstruction due to common atrioventricular valve (disorder)
Congenital Disease	SNOMED	448309008	Right ventricular outflow tract obstruction due to fibromuscular shelf (disorder)
Congenital Disease	SNOMED	448310003	Right ventricular outflow tract obstruction due to malaligned outlet septum (disorder)
Congenital Disease	SNOMED	448320008	Divided left atrium with some pulmonary veins to proximal chamber (disorder)
Congenital Disease	SNOMED	448326002	Right inferior vena cava connecting to left atrium and right atrium (disorder)
Congenital Disease	SNOMED	448328001	Aneurysm of aortic sinus of Valsalva without rupture (disorder)
Congenital Disease	SNOMED	448331000	Atrioventricular septal defect with restrictive ventricular component (disorder)
Congenital Disease	SNOMED	448332007	Left superior vena cava persisting to right sided atrium (disorder)
Congenital Disease	SNOMED	448356006	Coronary sinus drainage cephalad to right superior vena cava (disorder)
Congenital Disease	SNOMED	448357002	Midline anterior apex of heart (disorder)
Congenital Disease	SNOMED	448359004	Right ventricle superior to left ventricle (finding)
Congenital Disease	SNOMED	448411000	Spontaneous closure of ventricular septal defect due to prolapse of cusp of aortic valve (finding)
Congenital Disease	SNOMED	448412007	Tracheal origin of right upper lobe bronchus (disorder)
Congenital Disease	SNOMED	448413002	Pulmonary valve overriding ventricular septum (disorder)
Congenital Disease	SNOMED	448414008	Restrictive interatrial communication (disorder)
Congenital Disease	SNOMED	448415009	Subaortic stenosis due to common atrioventricular valve (disorder)
Congenital Disease	SNOMED	448470007	Confluent pulmonary arteries (finding)
Congenital Disease	SNOMED	448471006	Congenital abnormality of ascending aorta (disorder)
Congenital Disease	SNOMED	448472004	Congenital abnormality of pulmonary trunk (disorder)
Congenital Disease	SNOMED	448475002	Absent pulmonary valve syndrome with ventricular septal defect of non Fallot type (disorder)
Congenital Disease	SNOMED	448476001	Subpulmonary stenosis (disorder)
Congenital Disease	SNOMED	448477005	Superior to inferior ventricular relationship (disorder)
Congenital Disease	SNOMED	448478000	Systemic to pulmonary collateral artery from coronary artery (disorder)
Congenital Disease	SNOMED	448479008	Ductus arteriosus from brachiocephalic artery (disorder)
Congenital Disease	SNOMED	448480006	Ductus arteriosus from left subclavian artery (disorder)
Congenital Disease	SNOMED	448486000	Anomalous pulmonary to systemic collateral vein (disorder)
Congenital Disease	SNOMED	448487009	Anomalous pulmonary venous connection of mixed type (disorder)
Congenital Disease	SNOMED	448493001	Stomach in central position (disorder)

Congenital Disease	SNOMED	448495008	Stomach in right sided position (disorder)
Congenital Disease	SNOMED	448497000	Congenital deformity of mitral valve annulus (disorder)
Congenital Disease	SNOMED	448499002	Infracardiac location of anomalous pulmonary venous connection (disorder)
Congenital Disease	SNOMED	448500006	Intracardiac location of anomalous pulmonary venous connection (disorder)
Congenital Disease	SNOMED	448501005	Interrupted left inferior vena cava (disorder)
Congenital Disease	SNOMED	448510002	Stenosis of left pulmonary artery (disorder)
Congenital Disease	SNOMED	448516008	Double outlet right ventricle with doubly committed ventricular septal defect and pulmonary stenosis (disorder)
Congenital Disease	SNOMED	448517004	Vascular ring with left aortic arch and retroesophageal right descending aorta and right arterial duct arising from aortic diverticulum and aberrant right subclavian artery (disorder)
Congenital Disease	SNOMED	448574001	Unipartite right ventricle (disorder)
Congenital Disease	SNOMED	448575000	Fenestrated interatrial communication within oval fossa (disorder)
Congenital Disease	SNOMED	448576004	Fenestration of aortic valve cusp (disorder)
Congenital Disease	SNOMED	448577008	Muscular subvalvar atresia of aorta (disorder)
Congenital Disease	SNOMED	448595006	Scimitar syndrome with additional anomalous pulmonary venous connection (disorder)
Congenital Disease	SNOMED	448599000	Total anomalous pulmonary venous connection of infracardiac type (disorder)
Congenital Disease	SNOMED	448602005	Spleen in right sided position (disorder)
Congenital Disease	SNOMED	448604006	Congenital abnormality of pulmonary valve cusp (disorder)
Congenital Disease	SNOMED	448611005	Vascular ring with left aortic arch and retrotracheal right patent arterial duct (disorder)
Congenital Disease	SNOMED	448612003	Single ventricular outlet above both ventricles (disorder)
Congenital Disease	SNOMED	448614002	Inferior vena cava anterior and same side as descending aorta (disorder)
Congenital Disease	SNOMED	448618004	Abnormality of mitral valve annulus (disorder)
Congenital Disease	SNOMED	448619007	Anterior deviation of infundibular septum of Fallot type (disorder)
Congenital Disease	SNOMED	448620001	Azygos continuation of inferior vena cava to right superior vena cava (disorder)
Congenital Disease	SNOMED	448624005	Uniatrial biventricular connection with absent left sided atrioventricular connection with straddling valve (disorder)
Congenital Disease	SNOMED	448625006	Univentricular atrioventricular connection with absent left sided atrioventricular connection (disorder)
Congenital Disease	SNOMED	448626007	Gelatinous atrioventricular valve leaflet in atrioventricular septal defect (disorder)
Congenital Disease	SNOMED	448627003	Vascular ring with left aortic arch and right arterial duct arising from aberrant retroesophageal brachiocephalic artery (disorder)
Congenital Disease	SNOMED	448628008	Vascular ring with left aortic arch and right arterial duct arising from retroesophageal aortic diverticulum and aberrant right subclavian artery (disorder)
Congenital Disease	SNOMED	448629000	Vascular ring with left aortic arch and right arterial ligament (disorder)
Congenital Disease	SNOMED	448630005	Vascular ring with left aortic arch and right patent arterial duct (disorder)
Congenital Disease	SNOMED	448631009	Right inferior vena cava connecting to left sided atrium (disorder)
Congenital Disease	SNOMED	448632002	Left inferior vena cava connecting to left atrium and right atrium (disorder)
Congenital Disease	SNOMED	448633007	Right ventricular outflow tract obstruction due to atrioventricular valve (disorder)
Congenital Disease	SNOMED	448634001	Absent aortic valve cusp (disorder)
Congenital Disease	SNOMED	448635000	Absent ductus venosus (disorder)
Congenital Disease	SNOMED	448637008	Coarctation of left pulmonary artery (disorder)
Congenital Disease	SNOMED	448643005	Abnormality of pulmonary valve (disorder)
Congenital Disease	SNOMED	448644004	Abnormality of right inferior vena cava (disorder)
Congenital Disease	SNOMED	448645003	Aortic arch hypoplasia between subclavian and common carotid arteries (disorder)
Congenital Disease	SNOMED	448646002	Aortic arch hypoplasia distal to subclavian artery (disorder)
Congenital Disease	SNOMED	448647006	Bilateral bilobed lungs due to isomerism of left lung (disorder)
Congenital Disease	SNOMED	448648001	Bilateral trilobed lungs due to isomerism of right lung (disorder)
Congenital Disease	SNOMED	448680004	Concordant ventriculoarterial connections (finding)
Congenital Disease	SNOMED	448681000	Indeterminate atrial arrangement (disorder)
Congenital Disease	SNOMED	448682007	Thickened atrial septum (disorder)
Congenital Disease	SNOMED	448683002	Liver in left sided position (disorder)
Congenital Disease	SNOMED	448684008	Prolapse of left coronary aortic valve cusp (disorder)
Congenital Disease	SNOMED	448721009	Abnormal course of aortic arch and descending aorta (disorder)
Congenital Disease	SNOMED	448725000	Continuity between aortic valve and mitral valve (finding)
Congenital Disease	SNOMED	448726004	Parallel course of aorta and pulmonary artery (disorder)
Congenital Disease	SNOMED	448727008	Total anomalous pulmonary venous connections of mixed type (disorder)
Congenital Disease	SNOMED	448728003	Supracardiac location of anomalous pulmonary venous connection (disorder)
Congenital Disease	SNOMED	448729006	Common atrioventricular valve in functionally univentricular heart (disorder)
Congenital Disease	SNOMED	448742006	Abnormality of aortic arch (disorder)
Congenital Disease	SNOMED	448743001	Abnormality of aortic valve (disorder)
Congenital Disease	SNOMED	448747000	Common arterial trunk with pulmonary origin from truncal valve sinus (disorder)
Congenital Disease	SNOMED	448777006	Noncoapting atrioventricular valve leaflet in atrioventricular septal defect (disorder)
Congenital Disease	SNOMED	448778001	Obstruction of right ventricular outflow tract due to aortico-left ventricular tunnel (disorder)

Congenital Disease	SNOMED	448780007	Ventricular septal defect with absent outlet septum and overriding truncal valve with extension of membranous septum (disorder)
Congenital Disease	SNOMED	448782004	Interrupted right inferior vena cava (disorder)
Congenital Disease	SNOMED	448786001	Posterior deviation of infundibular septum of obstructive aortic arch type (disorder)
Congenital Disease	SNOMED	448790004	Anterior-posterior orientation of bicuspid aortic valve (disorder)
Congenital Disease	SNOMED	448793002	Bicuspid truncal valve (disorder)
Congenital Disease	SNOMED	448794008	Double outlet right ventricle with subpulmonary ventricular septal defect (disorder)
Congenital Disease	SNOMED	448809003	Common arterial trunk with obstruction of aortic arch (disorder)
Congenital Disease	SNOMED	448814004	Spontaneous closure of atrial component of atrioventricular septal defect (finding)
Congenital Disease	SNOMED	448819009	Right-left orientation of bicuspid aortic valve (disorder)
Congenital Disease	SNOMED	448821004	Abnormality of truncal valve cusp (disorder)
Congenital Disease	SNOMED	448822006	Deficiency of atrioventricular valve leaflet in atrioventricular septal defect (disorder)
Congenital Disease	SNOMED	448823001	Left ventricular outflow tract obstruction due to prolapse of Eustachian valve (disorder)
Congenital Disease	SNOMED	448827000	Ventricular septal defect with absent outlet septum and overriding truncal valve with inferior muscular rim (disorder)
Congenital Disease	SNOMED	448828005	Deficiency of mitral leaflet (disorder)
Congenital Disease	SNOMED	448836001	Thickened atrioventricular valve leaflet in atrioventricular septal defect (disorder)
Congenital Disease	SNOMED	448842002	Aortico-left ventricular tunnel of simple type (disorder)
Congenital Disease	SNOMED	448843007	Aortico-left ventricular tunnel with aneurysm of intracardiac septal wall and aneurysm of extracardiac aortic wall (disorder)
Congenital Disease	SNOMED	448844001	Multiple mitral papillary muscles with hammock valve (disorder)
Congenital Disease	SNOMED	448871001	Subaortic stenosis due to restrictive ventricular septal defect in functionally univentricular heart (disorder)
Congenital Disease	SNOMED	448872008	Systemic venovenous collateral vein (disorder)
Congenital Disease	SNOMED	448876006	Subarterial ventricular septal defect (disorder)
Congenital Disease	SNOMED	448887003	Common arterial trunk with isolated pulmonary artery (disorder)
Congenital Disease	SNOMED	448898002	Outflow tract abnormality in solitary indeterminate ventricle (disorder)
Congenital Disease	SNOMED	448915004	Cleft of hard palate (disorder)
Congenital Disease	SNOMED	448919005	Atrioventricular septal defect with ventricular component under superior bridging leaflet without chordal attachment to ventricular septal crest (disorder)
Congenital Disease	SNOMED	448920004	Congenital abnormality of mitral subvalvular apparatus (disorder)
Congenital Disease	SNOMED	448923002	Hypoplasia of infundibular septum (disorder)
Congenital Disease	SNOMED	448946000	Anterior-posterior orientation of bicuspid pulmonary valve (disorder)
Congenital Disease	SNOMED	448947009	Congenital abnormality of left atrioventricular valve in double inlet ventricle (disorder)
Congenital Disease	SNOMED	448965008	Inferior vena cava connecting to right atrium and left atrium (disorder)
Congenital Disease	SNOMED	448966009	Hypoplasia of trabecular portion of right ventricle (disorder)
Congenital Disease	SNOMED	448968005	Persisting fifth aortic arch with atresia of fourth arch (disorder)
Congenital Disease	SNOMED	448975006	Aortico-left ventricular tunnel with extracardiac aneurysm of aortic wall (disorder)
Congenital Disease	SNOMED	449005003	Absence of mitral chordae tendinae (disorder)
Congenital Disease	SNOMED	449008001	Flail atrioventricular valve leaflet in atrioventricular septal defect (disorder)
Congenital Disease	SNOMED	449009009	Left inferior vena cava connecting to left sided atrium (disorder)
Congenital Disease	SNOMED	449010004	Left inferior vena cava connecting to right sided atrium (disorder)
Congenital Disease	SNOMED	449011000	Fenestration of atrioventricular valve leaflet in atrioventricular septal defect (disorder)
Congenital Disease	SNOMED	449014008	Hepatic vein to left sided atrium (disorder)
Congenital Disease	SNOMED	449015009	Hepatic vein to right sided atrium (disorder)
Congenital Disease	SNOMED	449016005	Hepatic vein to left atrium and right atrium (disorder)
Congenital Disease	SNOMED	449025004	Vascular ring with left aortic arch and right arterial duct ligament arising from retroesophageal aortic diverticulum with aberrant right subclavian artery (disorder)
Congenital Disease	SNOMED	449027007	Distal origin of brachiocephalic artery with tracheal compression (disorder)
Congenital Disease	SNOMED	449031001	Congenital abnormality of right atrioventricular valve leaflet in double inlet ventricle (disorder)
Congenital Disease	SNOMED	449032008	Congenital abnormality of right atrioventricular valve papillary muscle in double inlet ventricle (disorder)
Congenital Disease	SNOMED	449037002	Truncal valve overriding ventricular septum (disorder)
Congenital Disease	SNOMED	449040002	Hypoplasia of right atrioventricular valve annulus in double inlet ventricle (disorder)
Congenital Disease	SNOMED	449085001	Pulmonary artery connecting to coronary artery via collateral artery (disorder)
Congenital Disease	SNOMED	449086000	Ebstein's anomaly of left atrioventricular valve in functionally univentricular heart (disorder)
Congenital Disease	SNOMED	449087009	Ebstein's anomaly of right atrioventricular valve in functionally univentricular heart (disorder)
Congenital Disease	SNOMED	449098005	Congenital abnormality of left atrioventricular valve chordae tendinae in double inlet ventricle (disorder)

Congenital Disease	SNOMED	449099002	Left atrioventricular valve stenosis in double inlet ventricle (disorder)
Congenital Disease	SNOMED	449110001	Congenital billowing of mitral valve leaflet (disorder)
Congenital Disease	SNOMED	449111002	Double orifice of right atrioventricular valve in double inlet ventricle (disorder)
Congenital Disease	SNOMED	449112009	Prolapse of noncoronary aortic valve cusp (disorder)
Congenital Disease	SNOMED	449116007	Tricuspid but functionally bicuspid aortic valve (disorder)
Congenital Disease	SNOMED	449118008	Left ventricular outflow tract obstruction due to prolapsed arterial valve (disorder)
Congenital Disease	SNOMED	449119000	Obstruction of aortic outflow (disorder)
Congenital Disease	SNOMED	449123008	Eccentric opening of tricuspid aortic valve (disorder)
Congenital Disease	SNOMED	449124002	Eccentric opening of tricuspid pulmonary valve (disorder)
Congenital Disease	SNOMED	449125001	Congenital stenosis of pulmonary artery (disorder)
Congenital Disease	SNOMED	449133000	Absence of pulmonary valve cusp (disorder)
Congenital Disease	SNOMED	449135007	Congenital stenosis of mitral subvalvular apparatus (disorder)
Congenital Disease	SNOMED	449136008	Fenestration of mitral leaflet (disorder)
Congenital Disease	SNOMED	449138009	Right ventricular outflow tract obstruction due to septal hypertrophy (disorder)
Congenital Disease	SNOMED	449139001	Commissural fusion of truncal valve (disorder)
Congenital Disease	SNOMED	449140004	Right-left orientation of bicuspid pulmonary valve (disorder)
Congenital Disease	SNOMED	449149003	Deficiency of aortic valve cusp (disorder)
Congenital Disease	SNOMED	449158005	Ebstein's anomaly of tricuspid valve with atrialization of right ventricular chamber (disorder)
Congenital Disease	SNOMED	449178002	Doubly committed ventricular septal defect in double outlet ventriculoarterial connection (disorder)
Congenital Disease	SNOMED	449188001	Left superior vena cava persisting to coronary sinus and then to right sided atrium (disorder)
Congenital Disease	SNOMED	449189009	Mass associated with atrioventricular valve leaflet in atrioventricular septal defect (disorder)
Congenital Disease	SNOMED	449190000	Muscular ventricular septal defect in trabecular septum (disorder)
Congenital Disease	SNOMED	449228007	Hypoplasia of left atrioventricular valve annulus in double inlet ventricle (disorder)
Congenital Disease	SNOMED	449232001	Aortic arch hypoplasia between carotid arteries (disorder)
Congenital Disease	SNOMED	449270002	Hypoplasia of mitral valve annulus (disorder)
Congenital Disease	SNOMED	449271003	Residual coarctation of aorta (disorder)
Congenital Disease	SNOMED	449315003	Congenital abnormality of right atrioventricular valve chordae tendinae in double inlet ventricle (disorder)
Congenital Disease	SNOMED	449316002	Congenital abnormality of right atrioventricular valve in double inlet ventricle (disorder)
Congenital Disease	SNOMED	449337005	Residual ductus arteriosus patency (disorder)
Congenital Disease	SNOMED	449350006	Pulmonary artery with absent proximal arterial connection (disorder)
Congenital Disease	SNOMED	449351005	Residual interatrial communication (disorder)
Congenital Disease	SNOMED	449352003	Right ventricular outflow tract obstruction due to abnormal cardiac muscle bands (disorder)
Congenital Disease	SNOMED	449353008	Hypoplasia of trabecular portion of left ventricle (disorder)
Congenital Disease	SNOMED	449354002	Hypoplasia of pulmonary cusps of absent pulmonary valve type (disorder)
Congenital Disease	SNOMED	449379004	Atrioventricular septal defect with ventricular component under inferior bridging leaflet with chords to crest ventricular septum (disorder)
Congenital Disease	SNOMED	449382009	Congenital abnormality of atrioventricular valve leaflet in atrioventricular septal defect (disorder)
Congenital Disease	SNOMED	449383004	Congenital abnormality of atrioventricular valve papillary muscle in atrioventricular septal defect (disorder)
Congenital Disease	SNOMED	449385006	Confluent muscular ventricular septal defect (disorder)
Congenital Disease	SNOMED	449388008	Abnormality of atrioventricular valve leaflet in atrioventricular septal defect (disorder)
Congenital Disease	SNOMED	449398002	Aortico-left ventricular tunnel with intracardiac aneurysm of septal portion (disorder)
Congenital Disease	SNOMED	449425007	Intracardiac location of anomalous pulmonary venous connection to coronary sinus (disorder)
Congenital Disease	SNOMED	449426008	Left sided azygos continuation of inferior vena cava to left superior vena cava (disorder)
Congenital Disease	SNOMED	449427004	Double aortic arch with right arch dominant and left arch patent (disorder)
Congenital Disease	SNOMED	449428009	Divided left atrium with all pulmonary veins to proximal chamber and then to left atrium with additional pulmonary venous chamber communication (disorder)
Congenital Disease	SNOMED	449429001	Divided left atrium with some pulmonary veins to proximal chamber draining to right atrium (disorder)
Congenital Disease	SNOMED	449430006	Double aortic arch with left arch dominant and right arch patent (disorder)
Congenital Disease	SNOMED	449433008	Diffuse stenosis of left pulmonary artery (disorder)
Congenital Disease	SNOMED	449434002	Supracardiac location of anomalous pulmonary venous connection to left superior vena cava (disorder)
Congenital Disease	SNOMED	449435001	Infracardiac location of anomalous pulmonary venous connection with two descending veins (disorder)
Congenital Disease	SNOMED	449436000	Divided left atrium with some pulmonary veins to proximal chamber draining to left atrium (disorder)

Congenital Disease	SNOMED	449439007	Absent ductus venosus with direct connection of umbilical vein to right atrium (disorder)
Congenital Disease	SNOMED	449440009	Divided left atrium with all pulmonary veins to proximal chamber without communication to left atrium with extracardiac pulmonary venous chamber communication (disorder)
Congenital Disease	SNOMED	449441008	Divided left atrium with all pulmonary veins to proximal chamber without communication to left atrium with pulmonary venous chamber communication to right atrium (disorder)
Congenital Disease	SNOMED	449442001	Congenital abnormality of great cardiac vein (disorder)
Congenital Disease	SNOMED	449443006	Supracardiac location of anomalous pulmonary venous connection to left sided vertical vein (disorder)
Congenital Disease	SNOMED	449444000	Infracardiac location of anomalous pulmonary venous connection to hepatic vein (disorder)
Congenital Disease	SNOMED	449445004	Anatomically corrected malposition with concordant ventriculoarterial connections and parallel great arteries (disorder)
Congenital Disease	SNOMED	449451009	Supracardiac location of anomalous pulmonary venous connection to hemiazygos vein (disorder)
Congenital Disease	SNOMED	449452002	Intracardiac location of anomalous pulmonary venous connection to right atrium (disorder)
Congenital Disease	SNOMED	449455000	Abnormal attachment of tricuspid chordae tendinae (disorder)
Congenital Disease	SNOMED	449456004	Atrioventricular septal defect with atrioventricular valve regurgitation through left septal commissure (disorder)
Congenital Disease	SNOMED	449457008	Atrioventricular septal defect with atrioventricular valve regurgitation through left superior bridging leaflet lateral mural commissure (disorder)
Congenital Disease	SNOMED	449458003	Right superior vena cava connecting to coronary sinus and then to left sided atrium (disorder)
Congenital Disease	SNOMED	449459006	Atrioventricular septal defect with atrioventricular valve regurgitation through left inferior bridging leaflet lateral mural commissure (disorder)
Congenital Disease	SNOMED	449467003	Diffuse stenosis of right pulmonary artery (disorder)
Congenital Disease	SNOMED	449479003	Malattachment of atrial septum (disorder)
Congenital Disease	SNOMED	449486006	Absent ductus venosus with direct connection of umbilical vein to renal vein (disorder)
Congenital Disease	SNOMED	449490008	Atrioventricular septal defect with atrioventricular valve regurgitation through right septal commissure (disorder)
Congenital Disease	SNOMED	449492000	Divided left atrium with all pulmonary veins to proximal chamber and then to left atrium with additional pulmonary venous chamber communication to right atrium (disorder)
Congenital Disease	SNOMED	449493005	Supracardiac location of anomalous pulmonary venous connection to right sided vertical vein (disorder)
Congenital Disease	SNOMED	449494004	Supracardiac location of anomalous pulmonary venous connection to right superior vena cava (disorder)
Congenital Disease	SNOMED	449495003	Infracardiac location of anomalous pulmonary venous connection to patent ductus venosus (disorder)
Congenital Disease	SNOMED	4495005	Congenital hypertrophy of ureteric valve (disorder)
Congenital Disease	SNOMED	449503003	Spontaneous reduction in size of ventricular septal defect due to prolapse of cusp of aortic valve (finding)
Congenital Disease	SNOMED	449513006	Anomalous pulmonary venous connection of mixed type with one pulmonary venous confluence (disorder)
Congenital Disease	SNOMED	449514000	Intracardiac location of anomalous pulmonary venous connection to midline with isomeric atria (disorder)
Congenital Disease	SNOMED	449516003	Separate hepatic vein and inferior vena cava connections to heart (disorder)
Congenital Disease	SNOMED	449520004	Right ventricular outflow tract obstruction due to septoparietal trabeculation (disorder)
Congenital Disease	SNOMED	449521000	Anomalous pulmonary venous connection of mixed type with two pulmonary venous confluences (disorder)
Congenital Disease	SNOMED	449523002	Right superior vena cava persisting to coronary sinus and then to right sided atrium (disorder)
Congenital Disease	SNOMED	449524008	Spontaneous reduction in size of ventricular septal defect due to accessory tissue of atrioventricular valve (finding)
Congenital Disease	SNOMED	449525009	Spontaneous reduction in size of ventricular septal defect due to fibromuscular reaction (finding)
Congenital Disease	SNOMED	449526005	Spontaneous reduction in size of ventricular septal defect due to tissue of membranous septum (finding)
Congenital Disease	SNOMED	449527001	Absent ductus venosus with direct connection of umbilical vein to inferior vena cava (disorder)
Congenital Disease	SNOMED	449529003	Abnormality of pulmonary valve cusp (disorder)
Congenital Disease	SNOMED	449532000	Congenital abnormality of anterior cardiac vein (disorder)
Congenital Disease	SNOMED	449533005	Supracardiac location of anomalous pulmonary venous connection to azygos vein (disorder)
Congenital Disease	SNOMED	449534004	Obstructed interchordal space of tricuspid chordae tendinae (disorder)
Congenital Disease	SNOMED	449536002	Double aortic arch with right arch dominant and atresia of left arch and left ligament to diverticulum (disorder)
Congenital Disease	SNOMED	449537006	Atrioventricular septal defect with atrioventricular valve regurgitation through right anterosuperior inferior mural commissure (disorder)

Congenital Disease	SNOMED	449538001	Atrioventricular septal defect with atrioventricular valve regurgitation through right anterosuperior superior bridging leaflet commissure (disorder)
Congenital Disease	SNOMED	449539009	Atrioventricular septal defect with atrioventricular valve regurgitation through right inferior bridging leaflet inferior mural commissure (disorder)
Congenital Disease	SNOMED	449542003	Outflow tract obstruction in solitary indeterminate ventricle (disorder)
Congenital Disease	SNOMED	449544002	Deficiency of truncal valve cusp (disorder)
Congenital Disease	SNOMED	449547009	Right pulmonary artery with absent proximal arterial connection (disorder)
Congenital Disease	SNOMED	449551006	Single stenosis of left pulmonary artery (disorder)
Congenital Disease	SNOMED	449555002	Aortic valve commissural abnormality (disorder)
Congenital Disease	SNOMED	449559008	Multiple stenoses of left pulmonary artery (disorder)
Congenital Disease	SNOMED	449560003	Multiple stenoses of right pulmonary artery (disorder)
Congenital Disease	SNOMED	449563001	Absence of primary mitral chordae tendinae (disorder)
Congenital Disease	SNOMED	449564007	Fenestration of truncal valve cusp (disorder)
Congenital Disease	SNOMED	449576007	Left pulmonary artery with absent proximal arterial connection (disorder)
Congenital Disease	SNOMED	449587004	Divided left atrium with all pulmonary veins to proximal chamber and then to left atrium with additional pulmonary venous chamber extracardiac communication (disorder)
Congenital Disease	SNOMED	449589001	Single stenosis of right pulmonary artery (disorder)
Congenital Disease	SNOMED	449593007	Divided left atrium with some pulmonary veins to proximal chamber draining to left atrium and others connecting directly to left atrium (disorder)
Congenital Disease	SNOMED	449594001	Divided left atrium with some pulmonary veins to proximal chamber draining to left atrium and others connecting anomalously (disorder)
Congenital Disease	SNOMED	449595000	Divided left atrium with some pulmonary veins to proximal chamber draining to right atrium and others connecting anomalously (disorder)
Congenital Disease	SNOMED	449596004	Divided left atrium with some pulmonary veins to proximal chamber draining to right atrium and others connecting directly to left atrium (disorder)
Congenital Disease	SNOMED	449599006	Malattachment of atrial septum with posterior aspect of septum primum to left (disorder)
Congenital Disease	SNOMED	449600009	Malattachment of atrial septum with superior aspect of septum primum to left and posterior (disorder)
Congenital Disease	SNOMED	449601008	Fenestration of pulmonary valve cusp (disorder)
Congenital Disease	SNOMED	449602001	Subaortic outflow tract obstruction in solitary indeterminate ventricle (disorder)
Congenital Disease	SNOMED	449604000	Subpulmonary outflow tract obstruction in solitary indeterminate ventricle (disorder)
Congenital Disease	SNOMED	449605004	Deficiency of pulmonary valve cusp (disorder)
Congenital Disease	SNOMED	44964000	Congenital fusion of testis (disorder)
Congenital Disease	SNOMED	449682004	Congenital absence of part of upper limb (disorder)
Congenital Disease	SNOMED	449683009	Congenital absence of part of upper arm (disorder)
Congenital Disease	SNOMED	449692007	Congenital shortening of upper arm (disorder)
Congenital Disease	SNOMED	449777000	Transient infantile osteopetrosis (disorder)
Congenital Disease	SNOMED	449790007	Orofacial cleft (disorder)
Congenital Disease	SNOMED	449817000	Peters plus syndrome (disorder)
Congenital Disease	SNOMED	449819002	3p partial monosomy syndrome (disorder)
Congenital Disease	SNOMED	449821007	Branchiooculofacial syndrome (disorder)
Congenital Disease	SNOMED	449824004	Marden Walker syndrome (disorder)
Congenital Disease	SNOMED	449866003	Vitreoretinal dysplasia (disorder)
Congenital Disease	SNOMED	449870006	Congenital anomaly of blood vessel of limb (disorder)
Congenital Disease	SNOMED	449871005	Congenital anomaly of blood vessel of lower limb (disorder)
Congenital Disease	SNOMED	449904002	Intramedullary glomus arteriovenous malformation of spinal cord (disorder)
Congenital Disease	SNOMED	449905001	Intramedullary and extramedullary arteriovenous malformation of spinal cord (disorder)
Congenital Disease	SNOMED	450300002	Anomalous origin of right coronary artery from pulmonary artery (disorder)
Congenital Disease	SNOMED	450301003	Anomalous origin of left coronary artery from pulmonary artery (disorder)
Congenital Disease	SNOMED	450302005	Anomalous origin of left anterior descending coronary artery from pulmonary artery (disorder)
Congenital Disease	SNOMED	450304006	Coarctation of suprarenal abdominal aorta (disorder)
Congenital Disease	SNOMED	450305007	Coarctation of infrarenal abdominal aorta (disorder)
Congenital Disease	SNOMED	450306008	Major systemic to pulmonary collateral artery supplying both lungs (disorder)
Congenital Disease	SNOMED	450307004	Major systemic to pulmonary collateral artery supplying entire right lung (disorder)
Congenital Disease	SNOMED	450308009	Major systemic to pulmonary collateral artery supplying entire left lung (disorder)
Congenital Disease	SNOMED	450309001	Major systemic to pulmonary collateral artery supplying part of right lung (disorder)
Congenital Disease	SNOMED	450310006	Major systemic to pulmonary collateral artery supplying part of left lung (disorder)
Congenital Disease	SNOMED	450311005	Major systemic to pulmonary collateral artery supplying unknown zone of distribution (disorder)
Congenital Disease	SNOMED	450312003	Coarctation of aorta between subclavian artery and common carotid artery (disorder)
Congenital Disease	SNOMED	450313008	Coarctation of aorta between left common carotid artery and right common carotid artery (disorder)

Congenital Disease	SNOMED	450314002	Vascular ring with right aortic arch and left patent ductus arteriosus (disorder)
Congenital Disease	SNOMED	450315001	Vascular ring with right aortic arch and left ligamentum arteriosum (disorder)
Congenital Disease	SNOMED	450808003	Bathrocephaly (disorder)
Congenital Disease	SNOMED	451030007	Urachal sinus (disorder)
Congenital Disease	SNOMED	45142002	Congenital pulmonary lymphangiectasis (disorder)
Congenital Disease	SNOMED	45163000	Congenital pontocerebellar hypoplasia (disorder)
Congenital Disease	SNOMED	45237002	Congenital dilatation of aorta (disorder)
Congenital Disease	SNOMED	4530000	Madelung's deformity (disorder)
Congenital Disease	SNOMED	45482001	Partial aphalangia of upper limb (disorder)
Congenital Disease	SNOMED	45484000	Partial ablepharon (disorder)
Congenital Disease	SNOMED	45492009	Congenital stenosis of superior vena cava (disorder)
Congenital Disease	SNOMED	45503006	Common ventricle (disorder)
Congenital Disease	SNOMED	45582004	Rubinstein-Taybi syndrome (disorder)
Congenital Disease	SNOMED	45615004	Manus cava (disorder)
Congenital Disease	SNOMED	45623002	Glaucoma associated with anterior segment anomaly (disorder)
Congenital Disease	SNOMED	457707000	Thrombosis of truncal valve (disorder)
Congenital Disease	SNOMED	457712004	Calcification of truncal valve (disorder)
Congenital Disease	SNOMED	457717005	Perforation of truncal valve cusp (disorder)
Congenital Disease	SNOMED	457722005	Rupture of truncal valve cusp (disorder)
Congenital Disease	SNOMED	457727004	Thrombosis of common atrioventricular valve (disorder)
Congenital Disease	SNOMED	457732003	Calcification of common atrioventricular valve (disorder)
Congenital Disease	SNOMED	457737009	Thrombosis of right atrioventricular (not morphologically tricuspid) valve (disorder)
Congenital Disease	SNOMED	457742001	Calcification of right atrioventricular (not morphologically tricuspid) valve (disorder)
Congenital Disease	SNOMED	457747007	Rupture of right atrioventricular (not morphologically tricuspid) valve chordae tendinae (disorder)
Congenital Disease	SNOMED	457752002	Rupture of right atrioventricular (not morphologically tricuspid) valve papillary muscle (disorder)
Congenital Disease	SNOMED	457757008	Myxomatous degeneration of right atrioventricular (not morphologically tricuspid) valve leaflet (disorder)
Congenital Disease	SNOMED	457762009	Thrombosis of left atrioventricular (not morphologically mitral) valve (disorder)
Congenital Disease	SNOMED	457767003	Myxomatous degeneration of left atrioventricular (not morphologically mitral) valve leaflet (disorder)
Congenital Disease	SNOMED	457772007	Calcification of left atrioventricular (not morphologically mitral) valve (disorder)
Congenital Disease	SNOMED	457777001	Rupture of left atrioventricular (not morphologically mitral) valve chordae tendinae (disorder)
Congenital Disease	SNOMED	457782008	Rupture of left atrioventricular (not morphologically mitral) valve papillary muscle (disorder)
Congenital Disease	SNOMED	45795007	Melanosis oculi (disorder)
Congenital Disease	SNOMED	45798009	Congenital cardiospasm (disorder)
Congenital Disease	SNOMED	458039003	Anomalous origin of left circumflex coronary artery from pulmonary artery (disorder)
Congenital Disease	SNOMED	458040001	Thoracopagus with separate hearts and pericardial sacs (disorder)
Congenital Disease	SNOMED	458041002	Thoracopagus with separate hearts and common pericardial sac (disorder)
Congenital Disease	SNOMED	458042009	Thoracopagus with conjoined atria (disorder)
Congenital Disease	SNOMED	458043004	Thoracopagus with conjoined atria and ventricles (disorder)
Congenital Disease	SNOMED	458044005	Rachipagus (disorder)
Congenital Disease	SNOMED	45806008	Reduction deformity of upper limb (disorder)
Congenital Disease	SNOMED	458085000	Parapagus (disorder)
Congenital Disease	SNOMED	458086004	Dithoracic parapagus (disorder)
Congenital Disease	SNOMED	458087008	Dicephalic parapagus (disorder)
Congenital Disease	SNOMED	458088003	Major systemic to pulmonary collateral artery (disorder)
Congenital Disease	SNOMED	458422009	Malrotation of intestine with midgut volvulus (disorder)
Congenital Disease	SNOMED	458427003	Bridging bronchus (disorder)
Congenital Disease	SNOMED	458432002	Arterial tortuosity syndrome (disorder)
Congenital Disease	SNOMED	459054000	Malrotation of intestine with internal herniation (disorder)
Congenital Disease	SNOMED	459065005	Congenital atresia of left main stem coronary artery (disorder)
Congenital Disease	SNOMED	459066006	Anomalous origin of accessory coronary artery from pulmonary artery (disorder)
Congenital Disease	SNOMED	459158000	Infective endocarditis of left atrioventricular (not morphologically mitral) valve (disorder)
Congenital Disease	SNOMED	459159008	Infective endocarditis of right atrioventricular (not morphologically tricuspid) valve (disorder)
Congenital Disease	SNOMED	459160003	Abscess at site of aortic coarctation (disorder)
Congenital Disease	SNOMED	459161004	Abscess at site of ventricular septal defect (disorder)
Congenital Disease	SNOMED	459164007	Systemic to pulmonary collateral artery from descending thoracic aorta (disorder)
Congenital Disease	SNOMED	459165008	Systemic to pulmonary collateral artery from abdominal aorta (disorder)
Congenital Disease	SNOMED	459172009	Infective endocarditis at site of ventricular septal defect (disorder)
Congenital Disease	SNOMED	459174005	Infective endocarditis at site of interatrial communication (disorder)
Congenital Disease	SNOMED	459175006	Abscess of left atrioventricular (not morphologically mitral) valve (disorder)

Congenital Disease	SNOMED	459176007	Abscess of right atrioventricular (not morphologically tricuspid) valve (disorder)
Congenital Disease	SNOMED	459177003	Abscess at site of interatrial communication (disorder)
Congenital Disease	SNOMED	459178008	Infective endocarditis of common atrioventricular valve (disorder)
Congenital Disease	SNOMED	459180002	Aneurysm of patch of atrioventricular septal defect (disorder)
Congenital Disease	SNOMED	45920002	Pelvis plana (disorder)
Congenital Disease	SNOMED	45987002	Congenital shortening of tendon (disorder)
Congenital Disease	SNOMED	4602007	Robin sequence (disorder)
Congenital Disease	SNOMED	460307002	Systemic to pulmonary collateral artery from right carotid artery (disorder)
Congenital Disease	SNOMED	460312001	Systemic to pulmonary collateral artery from left carotid artery (disorder)
Congenital Disease	SNOMED	460358000	Infective endocarditis at site of patch of ventricular septal defect (disorder)
Congenital Disease	SNOMED	460365008	Systemic to pulmonary collateral artery from right renal artery (disorder)
Congenital Disease	SNOMED	460370001	Systemic to pulmonary collateral artery connecting with artery (disorder)
Congenital Disease	SNOMED	460375006	Systemic to pulmonary collateral artery from left renal artery (disorder)
Congenital Disease	SNOMED	460380002	Systemic to pulmonary collateral artery from right brachiocephalic artery (disorder)
Congenital Disease	SNOMED	460387004	Systemic to pulmonary collateral artery from left brachiocephalic artery (disorder)
Congenital Disease	SNOMED	46041001	Maffucci syndrome (disorder)
Congenital Disease	SNOMED	460437005	Anomalous origin of dual left anterior descending coronary arteries from right coronary artery and left coronary artery (disorder)
Congenital Disease	SNOMED	460438000	Anomalous origin of large conus artery from right coronary artery (disorder)
Congenital Disease	SNOMED	460471001	Anomalous origin of conus artery from separate aortic sinus orifice (disorder)
Congenital Disease	SNOMED	460490009	Infective endocarditis at site of patch of interatrial communication (disorder)
Congenital Disease	SNOMED	460510005	Anomalous origin of coronary arteries from anterior aortic sinus (disorder)
Congenital Disease	SNOMED	460517008	Anomalous origin of coronary arteries from both aortic sinuses of bicuspid valve (disorder)
Congenital Disease	SNOMED	460524009	Anomalous origin of right coronary artery from left anterior descending coronary artery (disorder)
Congenital Disease	SNOMED	460531008	Anomalous origin of right coronary artery from left circumflex coronary artery (disorder)
Congenital Disease	SNOMED	460538002	Anomalous origin of left coronary artery from right coronary artery (disorder)
Congenital Disease	SNOMED	460545002	Congenital atresia of right coronary artery orifice (disorder)
Congenital Disease	SNOMED	460581004	Anomalous origin of accessory coronary artery from aortic sinus (disorder)
Congenital Disease	SNOMED	460582006	Anomalous origin of left coronary artery and right coronary artery from pulmonary artery (disorder)
Congenital Disease	SNOMED	460583001	Anomalous origin of sinus node coronary artery from separate aortic sinus orifice (disorder)
Congenital Disease	SNOMED	460584007	Anomalous course of coronary artery posterior to pulmonary trunk (disorder)
Congenital Disease	SNOMED	460585008	Anomalous course of coronary artery posterior to aorta (disorder)
Congenital Disease	SNOMED	460586009	Anomalous origin of right coronary artery from left coronary artery (disorder)
Congenital Disease	SNOMED	460587000	Anomalous origin of single coronary artery from left coronary artery aortic sinus (disorder)
Congenital Disease	SNOMED	460588005	Anomalous origin of left circumflex coronary artery from right coronary aortic sinus (disorder)
Congenital Disease	SNOMED	460589002	Vascular ring with right aortic arch and left ligamentum arteriosum between left subclavian artery and left common carotid artery (disorder)
Congenital Disease	SNOMED	460590006	Vascular ring with right aortic arch and left ligamentum arteriosum with anomalous retroesophageal left subclavian artery (disorder)
Congenital Disease	SNOMED	460591005	Vascular ring with right aortic arch and left ductus arteriosus from anomalous retroesophageal left subclavian artery (disorder)
Congenital Disease	SNOMED	460592003	Vascular ring with right aortic arch and left ductus arteriosus from retroesophageal diverticulum of aorta and anomalous left subclavian artery (disorder)
Congenital Disease	SNOMED	460593008	Vascular ring with right aortic arch and left ductus arteriosus from anomalous retroesophageal brachiocephalic artery (disorder)
Congenital Disease	SNOMED	460594002	Vascular ring with right aortic arch and left ductus arteriosus from retroesophageal diverticulum of aorta (disorder)
Congenital Disease	SNOMED	460595001	Systemic to pulmonary collateral artery connecting with central pulmonary arteries (disorder)
Congenital Disease	SNOMED	460596000	Systemic to pulmonary collateral artery connecting with isolated intraparenchymal pulmonary arteries (disorder)
Congenital Disease	SNOMED	460597009	Systemic to pulmonary collateral artery connecting with tracheobronchial arteries (disorder)
Congenital Disease	SNOMED	460600005	Dysfunction of left atrioventricular (not morphologically mitral) valve papillary muscle (disorder)
Congenital Disease	SNOMED	460601009	Dysfunction of right atrioventricular (not morphologically tricuspid) valve papillary muscle (disorder)
Congenital Disease	SNOMED	460602002	Dilatation of right atrioventricular (not morphologically tricuspid) valve in double inlet ventricle (disorder)
Congenital Disease	SNOMED	460603007	Abnormality of ductus arteriosus (disorder)
Congenital Disease	SNOMED	460604001	Anomalous origin of ductus arteriosus from right carotid artery (disorder)
Congenital Disease	SNOMED	460605000	Anomalous origin of ductus arteriosus from left carotid artery (disorder)
Congenital Disease	SNOMED	460606004	Anomalous origin of ductus arteriosus from ascending aorta (disorder)

Congenital Disease	SNOMED	460607008	Anomalous origin of ductus arteriosus from aortic arch (disorder)
Congenital Disease	SNOMED	460608003	Anomalous origin of ductus arteriosus from left brachiocephalic artery (disorder)
Congenital Disease	SNOMED	460609006	Anomalous insertion of ductus arteriosus (disorder)
Congenital Disease	SNOMED	460610001	Anomalous insertion of ductus arteriosus into pulmonary trunk (disorder)
Congenital Disease	SNOMED	460611002	Anomalous insertion of ductus arteriosus into right pulmonary artery (disorder)
Congenital Disease	SNOMED	460612009	Ductus arteriosus dependent pulmonary circulation (disorder)
Congenital Disease	SNOMED	460613004	Ductus arteriosus dependent systemic circulation (disorder)
Congenital Disease	SNOMED	460614005	Dilatation of left atrioventricular (not morphologically mitral) valve in double inlet ventricle (disorder)
Congenital Disease	SNOMED	460619000	Abscess of wall of truncus arteriosus (disorder)
Congenital Disease	SNOMED	460880006	Arteriovenous fistula of great cerebral vein of Galen (disorder)
Congenital Disease	SNOMED	460890003	Anomalous common origin of brachiocephalic artery and left common carotid artery (disorder)
Congenital Disease	SNOMED	460899002	Anomalous origin of left common carotid artery from brachiocephalic artery (disorder)
Congenital Disease	SNOMED	460906001	Vascular ring with mirror image branching of right aortic arch and left ligamentum arteriosum (disorder)
Congenital Disease	SNOMED	460913001	Systemic to coronary collateral artery (disorder)
Congenital Disease	SNOMED	460918005	Abnormality within thorax due to coarctation of aorta (disorder)
Congenital Disease	SNOMED	460923005	Anomalous origin of right coronary artery from left coronary artery aortic sinus (disorder)
Congenital Disease	SNOMED	460930004	Anomalous origin of left coronary artery from right coronary aortic sinus (disorder)
Congenital Disease	SNOMED	460937001	Anomalous origin of single coronary artery from right coronary artery aortic sinus (disorder)
Congenital Disease	SNOMED	460944005	Anomalous origin of right coronary artery from left coronary artery aortic sinus and anomalous origin of left coronary artery from right coronary artery aortic sinus (disorder)
Congenital Disease	SNOMED	460952008	Abnormality of ligamentum arteriosum (disorder)
Congenital Disease	SNOMED	460953003	Anomalous insertion of ligamentum arteriosum (disorder)
Congenital Disease	SNOMED	460954009	Anomalous insertion of ligamentum arteriosum into distal left pulmonary artery (disorder)
Congenital Disease	SNOMED	460955005	Anomalous insertion of ligamentum arteriosum into pulmonary trunk (disorder)
Congenital Disease	SNOMED	460956006	Anomalous insertion of ligamentum arteriosum into right pulmonary artery (disorder)
Congenital Disease	SNOMED	460957002	Anomalous origin of ligamentum arteriosum (disorder)
Congenital Disease	SNOMED	460958007	Anomalous origin of ligamentum arteriosum from aortic arch (disorder)
Congenital Disease	SNOMED	460959004	Anomalous origin of ligamentum arteriosum from ascending aorta (disorder)
Congenital Disease	SNOMED	460960009	Anomalous origin of ligamentum arteriosum from distal descending aorta (disorder)
Congenital Disease	SNOMED	460961008	Anomalous origin of ligamentum arteriosum from right brachiocephalic artery (disorder)
Congenital Disease	SNOMED	460962001	Anomalous origin of ligamentum arteriosum from left brachiocephalic artery (disorder)
Congenital Disease	SNOMED	460963006	Anomalous origin of ligamentum arteriosum from right subclavian artery (disorder)
Congenital Disease	SNOMED	460964000	Anomalous origin of ligamentum arteriosum from left subclavian artery (disorder)
Congenital Disease	SNOMED	460965004	Anomalous origin of ligamentum arteriosum from right carotid artery (disorder)
Congenital Disease	SNOMED	460966003	Anomalous origin of ligamentum arteriosum from left carotid artery (disorder)
Congenital Disease	SNOMED	460967007	Right ligamentum arteriosum (disorder)
Congenital Disease	SNOMED	460968002	Anomalous origin of right ligamentum arteriosum from right aortic arch (disorder)
Congenital Disease	SNOMED	460969005	Anomalous origin of right ligamentum arteriosum from left aortic arch (disorder)
Congenital Disease	SNOMED	46100008	Parasitic twin of asymmetrical conjoined twins (disorder)
Congenital Disease	SNOMED	461088006	Cardiovascular abnormality due to anomalous origin of coronary artery from pulmonary artery (disorder)
Congenital Disease	SNOMED	461090007	Right ductus arteriosus (disorder)
Congenital Disease	SNOMED	461091006	Patent right ductus arteriosus (disorder)
Congenital Disease	SNOMED	461092004	Spontaneous closure of residual atrial septal defect within oval fossa (finding)
Congenital Disease	SNOMED	461093009	Bilateral ductus arteriosus (disorder)
Congenital Disease	SNOMED	461094003	Bilateral ductus arteriosus with patent left ductus arteriosus and closed right ductus arteriosus (disorder)
Congenital Disease	SNOMED	461095002	Bilateral ductus arteriosus with closed left ductus arteriosus and patent right ductus arteriosus (disorder)
Congenital Disease	SNOMED	461096001	Bilateral patent ductus arteriosus (disorder)
Congenital Disease	SNOMED	461097005	Bilateral closed ductus arteriosus (disorder)
Congenital Disease	SNOMED	461098000	Cardiovascular abnormality due to bilateral ductus arteriosus (disorder)

Congenital Disease	SNOMED	461099008	Cardiovascular abnormality due to patent ductus arteriosus (disorder)
Congenital Disease	SNOMED	461100000	Cardiovascular abnormality due to anomalous origin of ductus arteriosus (disorder)
Congenital Disease	SNOMED	461101001	Anomalous origin of ductus arteriosus from distal descending aorta (disorder)
Congenital Disease	SNOMED	461102008	Anomalous insertion of ductus arteriosus into distal left pulmonary artery (disorder)
Congenital Disease	SNOMED	461103003	Anomalous origin of ductus arteriosus from right subclavian artery (disorder)
Congenital Disease	SNOMED	461104009	Interruption of coronary artery (disorder)
Congenital Disease	SNOMED	461105005	Anomalous origin of coronary artery from aorta (disorder)
Congenital Disease	SNOMED	461107002	Single coronary artery dividing into right coronary artery and left coronary artery (disorder)
Congenital Disease	SNOMED	461109004	Anomalous course of coronary artery anterior to pulmonary trunk and aorta (disorder)
Congenital Disease	SNOMED	461110009	Anomalous course of coronary artery posterior to pulmonary trunk and aorta (disorder)
Congenital Disease	SNOMED	461111008	Anomalous course of coronary artery anterior to aorta (disorder)
Congenital Disease	SNOMED	461112001	Anomalous course of coronary artery anterior to pulmonary trunk (disorder)
Congenital Disease	SNOMED	461298006	Acquired abnormality of left atrioventricular (not morphologically mitral) valve (disorder)
Congenital Disease	SNOMED	461305001	Acquired abnormality of right atrioventricular (not morphologically tricuspid) valve (disorder)
Congenital Disease	SNOMED	461326001	Anomalous separate origins of internal carotid arteries and external carotid arteries from single aortic arch (disorder)
Congenital Disease	SNOMED	461331004	Malposition of coronary artery orifice (disorder)
Congenital Disease	SNOMED	461338005	Cardiovascular abnormality due to anomalous origin of coronary artery orifice (disorder)
Congenital Disease	SNOMED	461345005	Coronary artery orifice abnormally low (disorder)
Congenital Disease	SNOMED	461352007	Slit-like coronary artery orifice (disorder)
Congenital Disease	SNOMED	461359003	Common coronary artery orifice (disorder)
Congenital Disease	SNOMED	461366002	Congenital hypoplasia of descending aorta (disorder)
Congenital Disease	SNOMED	461371009	Congenital hypoplasia of abdominal aorta (disorder)
Congenital Disease	SNOMED	461376004	Congenital hypoplasia of thoracoabdominal aorta (disorder)
Congenital Disease	SNOMED	461381008	Anomalous intramural course of proximal portion of coronary artery within aortic sinus (disorder)
Congenital Disease	SNOMED	461382001	Anomalous intramural course of proximal portion of coronary artery across commissure of aortic valve (disorder)
Congenital Disease	SNOMED	461383006	Anomalous intramural course of proximal portion of coronary artery above aortic sinus (disorder)
Congenital Disease	SNOMED	461384000	Anomalous course of coronary artery across right ventricular outflow tract (disorder)
Congenital Disease	SNOMED	461385004	Anomalous course of coronary artery through infundibular septum (disorder)
Congenital Disease	SNOMED	461386003	Patent ductus arteriosus with normal origin and insertion (disorder)
Congenital Disease	SNOMED	461387007	Anomalous origin of left ductus arteriosus from right aortic arch (disorder)
Congenital Disease	SNOMED	461388002	Anomalous origin of right ductus arteriosus from right aortic arch (disorder)
Congenital Disease	SNOMED	461389005	Anomalous origin of ductus arteriosus from aortic diverticulum (disorder)
Congenital Disease	SNOMED	461390001	Anomalous insertion of ductus arteriosus into unknown site (disorder)
Congenital Disease	SNOMED	461397003	Patent ductus arteriosus with normal origin and insertion and within normal period for functional closure (finding)
Congenital Disease	SNOMED	461399000	Anomalous origin of ligamentum arteriosum from right aortic arch (disorder)
Congenital Disease	SNOMED	461400007	Anomalous origin of ligamentum arteriosum from aortic diverticulum (disorder)
Congenital Disease	SNOMED	461401006	Anomalous origin of ligamentum arteriosum from unknown site (disorder)
Congenital Disease	SNOMED	461415008	Hereditary dysplasia of blood vessel (disorder)
Congenital Disease	SNOMED	461420008	Anomalous coronary artery with acute angulation of less than 45 degrees relative to aorta (disorder)
Congenital Disease	SNOMED	461421007	Anomalous coronary artery without acute angulation of less than 45 degrees relative to aorta (disorder)
Congenital Disease	SNOMED	461428001	Restriction of coronary artery orifice due to valve cusp tissue (disorder)
Congenital Disease	SNOMED	461433002	Major systemic to pulmonary collateral artery with absent pulmonary arteries proximal to hilar bifurcation (disorder)
Congenital Disease	SNOMED	461434008	Major systemic to pulmonary collateral artery with pulmonary artery proximal to hilar bifurcation (disorder)
Congenital Disease	SNOMED	461435009	Anomalous origin of left anterior descending coronary artery from right coronary artery aortic sinus (disorder)
Congenital Disease	SNOMED	461436005	Anomalous origin of dual left anterior descending coronary arteries (disorder)
Congenital Disease	SNOMED	461438006	Double barrel dual coronary artery orifices within aortic sinus (disorder)
Congenital Disease	SNOMED	461439003	Widely spaced right coronary artery and left coronary artery orifices within single aortic sinus (disorder)
Congenital Disease	SNOMED	461440001	Congenital abnormality of systemic artery (disorder)
Congenital Disease	SNOMED	461557000	Congenital atresia of aortic arch (disorder)
Congenital Disease	SNOMED	461562004	Atresia of aortic arch with fibrous cord (disorder)
Congenital Disease	SNOMED	461567005	Atresia of aortic arch with fibrous cord distal to subclavian artery (disorder)

Congenital Disease	SNOMED	461572001	Atresia of aortic arch with fibrous cord between subclavian artery and common carotid artery (disorder)
Congenital Disease	SNOMED	461577007	Atresia of aortic arch with fibrous cord between left common carotid artery and right common carotid artery (disorder)
Congenital Disease	SNOMED	461587006	Congenital luminal atresia of aortic arch distal to subclavian artery (disorder)
Congenital Disease	SNOMED	461592008	Congenital luminal atresia of aortic arch between subclavian artery and common carotid artery (disorder)
Congenital Disease	SNOMED	461597002	Congenital luminal atresia of aortic arch between left common carotid artery and right common carotid artery (disorder)
Congenital Disease	SNOMED	461629004	Right aortic arch branching pattern (disorder)
Congenital Disease	SNOMED	461634000	Abnormality within thorax due to interruption of aortic arch (disorder)
Congenital Disease	SNOMED	46168003	Pigmentary glaucoma (disorder)
Congenital Disease	SNOMED	462165005	Fetal choroid plexus cyst (disorder)
Congenital Disease	SNOMED	46284005	Congenital obstruction of bladder neck (disorder)
Congenital Disease	SNOMED	46395002	Multiple renal arteries (disorder)
Congenital Disease	SNOMED	46434000	Assimilation pelvis (disorder)
Congenital Disease	SNOMED	46619002	Congenital heart block (disorder)
Congenital Disease	SNOMED	46659004	Von Hippel-Lindau syndrome (disorder)
Congenital Disease	SNOMED	46698009	Dicephalus dipus dibrachius (disorder)
Congenital Disease	SNOMED	46722007	Congenital anomaly of pleural folds (disorder)
Congenital Disease	SNOMED	46829007	Developmental displacement of brachial plexus (disorder)
Congenital Disease	SNOMED	46907007	Congenital duplication of gallbladder (disorder)
Congenital Disease	SNOMED	46965001	Agenesis of left lung (disorder)
Congenital Disease	SNOMED	47017007	Ring chromosome 1 syndrome (disorder)
Congenital Disease	SNOMED	47028006	Congenital hiatus hernia (disorder)
Congenital Disease	SNOMED	47032000	Congenital hydrocephalus (disorder)
Congenital Disease	SNOMED	47054003	Septate vagina (disorder)
Congenital Disease	SNOMED	47070001	Congenital web of larynx (disorder)
Congenital Disease	SNOMED	470750006	Failure of growth of fetal right cardiac ventricle (disorder)
Congenital Disease	SNOMED	470751005	Failure of growth of fetal left cardiac ventricle (disorder)
Congenital Disease	SNOMED	470752003	Progression of fetal right ventricular outflow tract obstruction (disorder)
Congenital Disease	SNOMED	470753008	Progression of fetal left ventricular outflow tract obstruction (disorder)
Congenital Disease	SNOMED	4711003	Congenital anomaly of bile ducts (disorder)
Congenital Disease	SNOMED	471274000	Systemic to pulmonary collateral artery contributing to dual lung supply (disorder)
Congenital Disease	SNOMED	471276003	Single left coronary artery supplying all of heart with usual distribution of right coronary artery derived from distal left coronary artery (disorder)
Congenital Disease	SNOMED	471277007	Single right coronary artery supplying all of heart with usual distribution of left coronary artery derived from distal right coronary artery (disorder)
Congenital Disease	SNOMED	471279005	Recurrent atrial component of atrioventricular septal defect after prior cardiovascular surgical procedure (disorder)
Congenital Disease	SNOMED	471280008	Recurrent ventricular component of atrioventricular septal defect after prior cardiovascular surgical procedure (disorder)
Congenital Disease	SNOMED	471282000	Chromosome microdeletion (finding)
Congenital Disease	SNOMED	471285003	Anomalous origin of coronary artery from aortic sinus to left of nonfacing aortic sinus (disorder)
Congenital Disease	SNOMED	471286002	Anomalous origin of coronary artery from aortic sinus to right of nonfacing aortic sinus (disorder)
Congenital Disease	SNOMED	471287006	Anomalous origin of left coronary artery and right coronary artery with dual orifices from aortic sinus to left of nonfacing aortic sinus (disorder)
Congenital Disease	SNOMED	471288001	Anomalous origin of left coronary artery and right coronary artery with dual orifices from aortic sinus to right of nonfacing aortic sinus (disorder)
Congenital Disease	SNOMED	471289009	Anomalous origin of circumflex artery from aortic sinus to right of nonfacing aortic sinus and anomalous origin of left anterior descending coronary artery and right coronary artery from aortic sinus to left of nonfacing aortic sinus (disorder)
Congenital Disease	SNOMED	471290000	Anomalous origin of left anterior descending artery from aortic sinus to right of nonfacing aortic sinus and anomalous origin of circumflex artery and right coronary artery from aortic sinus to left of nonfacing aortic sinus (disorder)
Congenital Disease	SNOMED	471291001	Anomalous origin of right coronary artery from aortic sinus to right of nonfacing aortic sinus and anomalous origin of left coronary artery from aortic sinus to left of nonfacing aortic sinus (disorder)
Congenital Disease	SNOMED	471292008	Anomalous origin of right coronary artery and circumflex artery from aortic sinus to right of nonfacing aortic sinus and anomalous origin of left anterior descending artery from aortic sinus to left on nonfacing aortic sinus (disorder)
Congenital Disease	SNOMED	471293003	Anomalous origin of left anterior descending artery and right coronary artery from aortic sinus to right of nonfacing aortic sinus and anomalous origin of circumflex artery from aortic sinus to left of nonfacing aortic sinus (disorder)
Congenital Disease	SNOMED	471294009	Congenital pericardial cyst (disorder)
Congenital Disease	SNOMED	471297002	Anomalous origin of single coronary artery from nonfacing aortic sinus (disorder)
Congenital Disease	SNOMED	471298007	Anomalous origin of single coronary artery from aortic sinus to left of nonfacing aortic sinus (disorder)

Congenital Disease	SNOMED	471299004	Anomalous origin of single coronary artery from aortic sinus to right of nonfacing aortic sinus (disorder)
Congenital Disease	SNOMED	47139007	Congenital leukonychia (disorder)
Congenital Disease	SNOMED	47147007	Congenital anomaly of lung (disorder)
Congenital Disease	SNOMED	471880001	Heart failure due to end stage congenital heart disease (disorder)
Congenital Disease	SNOMED	47206001	Long tubular intestinal duplication (disorder)
Congenital Disease	SNOMED	472101004	Interruption of aortic arch distal to subclavian artery (disorder)
Congenital Disease	SNOMED	472102006	Interruption of aortic arch between subclavian artery and common carotid artery (disorder)
Congenital Disease	SNOMED	472103001	Interruption of aortic arch between left common carotid artery and right common carotid artery (disorder)
Congenital Disease	SNOMED	472133008	Congenital tortuosity of branch of aortic arch (disorder)
Congenital Disease	SNOMED	472703008	Pseudoacardia (disorder)
Congenital Disease	SNOMED	472705001	Narrowing of fetal ductus arteriosus (disorder)
Congenital Disease	SNOMED	472706000	Closure of fetal ductus arteriosus (disorder)
Congenital Disease	SNOMED	47276000	Congenital absence of femur (disorder)
Congenital Disease	SNOMED	472772002	Fetal right atrial dilatation (disorder)
Congenital Disease	SNOMED	472775000	Fetal left ventricular dysfunction (disorder)
Congenital Disease	SNOMED	472776004	Fetal biventricular dysfunction (disorder)
Congenital Disease	SNOMED	472777008	Congenital intrapericardial cyst (disorder)
Congenital Disease	SNOMED	472778003	Congenital extrapericardial cyst (disorder)
Congenital Disease	SNOMED	472782001	Stenosis of subvalvular region of neopulmonary valve (disorder)
Congenital Disease	SNOMED	472785004	Right ventricular outflow tract obstruction due to neoplasm (disorder)
Congenital Disease	SNOMED	472787007	Left ventricular outflow tract obstruction due to neoplasm (disorder)
Congenital Disease	SNOMED	472792009	Congenital abnormality of supraaortic branch of thoracic aorta (disorder)
Congenital Disease	SNOMED	472794005	Acquired subaortic stenosis associated with functionally univentricular heart (disorder)
Congenital Disease	SNOMED	472795006	Acquired subaortic stenosis due to restrictive ventricular septal defect associated with functionally univentricular heart (disorder)
Congenital Disease	SNOMED	472796007	Congenital subaortic stenosis due to restrictive ventricular septal defect associated with functionally univentricular heart (disorder)
Congenital Disease	SNOMED	472797003	Subaortic stenosis associated with functionally univentricular heart as complication of procedure (disorder)
Congenital Disease	SNOMED	472799000	Acquired subpulmonary stenosis associated with functionally univentricular heart (disorder)
Congenital Disease	SNOMED	472800001	Acquired subpulmonary stenosis due to restrictive ventricular defect associated with functionally univentricular heart (disorder)
Congenital Disease	SNOMED	472801002	Congenital subpulmonary stenosis due to restrictive ventricular defect associated with functionally univentricular heart (disorder)
Congenital Disease	SNOMED	472802009	Subpulmonary stenosis associated with functionally univentricular heart as complication of procedure (disorder)
Congenital Disease	SNOMED	472820000	Abnormal ventriculoarterial connection with usual origin of left coronary artery from aortic sinus to right of nonfacing aortic sinus and usual origin of right coronary artery from aortic sinus to left of nonfacing aortic sinus (disorder)
Congenital Disease	SNOMED	472823003	Cecoureterocele (disorder)
Congenital Disease	SNOMED	472828007	Disorder of atrioventricular (not morphologically mitral or tricuspid) valve (disorder)
Congenital Disease	SNOMED	472840007	Regurgitation of fetal tricuspid valve (disorder)
Congenital Disease	SNOMED	472841006	Stenosis of fetal tricuspid valve (disorder)
Congenital Disease	SNOMED	472842004	Regurgitation of fetal mitral valve (disorder)
Congenital Disease	SNOMED	472843009	Stenosis of fetal mitral valve (disorder)
Congenital Disease	SNOMED	472844003	Regurgitation of fetal pulmonary valve (disorder)
Congenital Disease	SNOMED	472845002	Stenosis of fetal pulmonary valve (disorder)
Congenital Disease	SNOMED	472847005	Stenosis of fetal aortic valve (disorder)
Congenital Disease	SNOMED	472848000	Regurgitation of fetal right atrioventricular (not morphologically tricuspid) valve (disorder)
Congenital Disease	SNOMED	472849008	Stenosis of fetal right atrioventricular (not morphologically tricuspid) valve (disorder)
Congenital Disease	SNOMED	472850008	Regurgitation of fetal left atrioventricular (not morphologically mitral) valve (disorder)
Congenital Disease	SNOMED	472851007	Stenosis of fetal left atrioventricular (not morphologically mitral) valve (disorder)
Congenital Disease	SNOMED	472852000	Regurgitation of fetal common atrioventricular valve (disorder)
Congenital Disease	SNOMED	472853005	Stenosis of fetal common atrioventricular valve (disorder)
Congenital Disease	SNOMED	472855003	Stenosis of fetal truncal valve (disorder)
Congenital Disease	SNOMED	473005004	Anomalous origin of ligamentum arteriosum from retroesophageal aortic diverticulum (disorder)
Congenital Disease	SNOMED	473362006	Congenital stenosis of distal coronary artery (disorder)
Congenital Disease	SNOMED	473376007	Rupture of right atrioventricular (not morphologically tricuspid) valve leaflet (disorder)
Congenital Disease	SNOMED	473377003	Perforation of right atrioventricular (not morphologically tricuspid) valve leaflet (disorder)
Congenital Disease	SNOMED	473379000	Rupture of left atrioventricular (not morphologically mitral) valve leaflet (disorder)

Congenital Disease	SNOMED	473380002	Perforation of left atrioventricular (not morphologically mitral) valve leaflet (disorder)
Congenital Disease	SNOMED	473383000	Abnormality of fetal heart (disorder)
Congenital Disease	SNOMED	473393007	Congenital occlusion of coronary sinus (disorder)
Congenital Disease	SNOMED	473394001	Congenital occlusion of iliac vein (disorder)
Congenital Disease	SNOMED	473395000	Congenital occlusion of femoral vein (disorder)
Congenital Disease	SNOMED	473443007	Anomalous origin of right coronary artery (disorder)
Congenital Disease	SNOMED	473444001	Anomalous origin of left coronary artery (disorder)
Congenital Disease	SNOMED	473454002	Disorder of left atrioventricular (not morphologically mitral) valve (disorder)
Congenital Disease	SNOMED	473455001	Disorder of right atrioventricular (not morphologically tricuspid) valve (disorder)
Congenital Disease	SNOMED	473458004	Regurgitation of atrioventricular (not morphologically mitral or tricuspid) valve (disorder)
Congenital Disease	SNOMED	47434006	Waardenburg syndrome (disorder)
Congenital Disease	SNOMED	47507006	Rieger syndrome (disorder)
Congenital Disease	SNOMED	47535005	Coloboma, heart defects, choanal atresia, retardation of growth and development, genitourinary problems, ear abnormalities syndrome (disorder)
Congenital Disease	SNOMED	47583008	Congenital anomaly of the pelvis (disorder)
Congenital Disease	SNOMED	47686007	Incomplete bilateral cleft palate (disorder)
Congenital Disease	SNOMED	47713000	Congenital absence of humerus (disorder)
Congenital Disease	SNOMED	47880003	Congenital absence of muscle AND/OR tendon (disorder)
Congenital Disease	SNOMED	48008009	Manus vara (disorder)
Congenital Disease	SNOMED	48043005	Pelvis justo minor (disorder)
Congenital Disease	SNOMED	48061001	Congenital calculus of kidney (disorder)
Congenital Disease	SNOMED	48069004	Acrocephaly (disorder)
Congenital Disease	SNOMED	48082007	Anomaly of chromosome pair 8 (disorder)
Congenital Disease	SNOMED	48121000	Congenital cardiomegaly (disorder)
Congenital Disease	SNOMED	48180002	Otocephalic syndrome (disorder)
Congenital Disease	SNOMED	48241004	Meckel's diverticulitis (disorder)
Congenital Disease	SNOMED	48249002	Congenital anisocoria (disorder)
Congenital Disease	SNOMED	48251003	Spade-like hand (disorder)
Congenital Disease	SNOMED	48301005	Congenital absence of finger (disorder)
Congenital Disease	SNOMED	48334007	Congenital dislocation of hip (disorder)
Congenital Disease	SNOMED	48337000	Congenital stricture of urinary meatus (disorder)
Congenital Disease	SNOMED	48376004	Congenital pseudoporencephaly (disorder)
Congenital Disease	SNOMED	48449000	Congenital macrodactyly (disorder)
Congenital Disease	SNOMED	48520006	Congenital atresia of cardiac vein (disorder)
Congenital Disease	SNOMED	48528004	Recessive dystrophic epidermolysis bullosa (disorder)
Congenital Disease	SNOMED	48607003	Accessory parotid gland (disorder)
Congenital Disease	SNOMED	48611009	Darier disease (disorder)
Congenital Disease	SNOMED	48637007	Multiple malformation syndrome with early overgrowth (disorder)
Congenital Disease	SNOMED	48644003	Congenital hypertrophic pyloric stenosis (disorder)
Congenital Disease	SNOMED	48672005	Accessory fallopian tube (disorder)
Congenital Disease	SNOMED	48718006	Roberts-SC phocomelia syndrome (disorder)
Congenital Disease	SNOMED	4874006	11q partial trisomy syndrome (disorder)
Congenital Disease	SNOMED	48760005	10p partial trisomy syndrome (disorder)
Congenital Disease	SNOMED	48763007	Congenital hernia of foramen of Morgagni (disorder)
Congenital Disease	SNOMED	48777005	Cranioschisis (disorder)
Congenital Disease	SNOMED	48812004	17q partial trisomy syndrome (disorder)
Congenital Disease	SNOMED	48980001	Congenital obstruction of urethra (disorder)
Congenital Disease	SNOMED	49008000	Malrotation of kidney (disorder)
Congenital Disease	SNOMED	49024004	4p partial trisomy syndrome (disorder)
Congenital Disease	SNOMED	49096008	Duhamel's syndrome (disorder)
Congenital Disease	SNOMED	49224008	Supernumerary ear lobule (disorder)
Congenital Disease	SNOMED	49347007	Osteosclerosis (disorder)
Congenital Disease	SNOMED	49381001	Congenital anomaly of retina (disorder)
Congenital Disease	SNOMED	4945003	Microgyria (disorder)
Congenital Disease	SNOMED	49494003	Ectopic adrenal gland (disorder)
Congenital Disease	SNOMED	49496001	Double ureter (disorder)
Congenital Disease	SNOMED	49534003	Congenital atresia of ureter (disorder)
Congenital Disease	SNOMED	49714001	Congenital anomaly of gallbladder (disorder)
Congenital Disease	SNOMED	49813000	Bilateral congenital macrostomia (disorder)
Congenital Disease	SNOMED	49984004	FG syndrome (disorder)
Congenital Disease	SNOMED	50108000	Osteochondrodysplasia with osteopetrosis (disorder)
Congenital Disease	SNOMED	50123005	Beals auriculo-osteodysplasia syndrome (disorder)
Congenital Disease	SNOMED	50267003	Congenital enlargement of nasopharynx (disorder)
Congenital Disease	SNOMED	50307003	Funnel-shaped pelvis (disorder)
Congenital Disease	SNOMED	50429003	Congenital stenosis of aqueduct of Sylvius (disorder)
Congenital Disease	SNOMED	5051002	Anomaly of chromosome pair 9 (disorder)
Congenital Disease	SNOMED	50513008	Congenital atresia of bronchus (disorder)
Congenital Disease	SNOMED	50749006	Double Y syndrome (disorder)
Congenital Disease	SNOMED	50751005	Sinus pericranii (disorder)
Congenital Disease	SNOMED	50847000	Accessory skeletal muscle (disorder)
Congenital Disease	SNOMED	50869007	Ehlers-Danlos syndrome, type 8 (disorder)

Congenital Disease	SNOMED	5087009	Brachypellic pelvis (disorder)
Congenital Disease	SNOMED	50911000	Ectopic testis (disorder)
Congenital Disease	SNOMED	50913002	Grob's syndrome (disorder)
Congenital Disease	SNOMED	50943000	Congenital anomaly of bronchus (disorder)
Congenital Disease	SNOMED	50956007	Accessory nipple (disorder)
Congenital Disease	SNOMED	50986000	Congenital fistula of urachus (disorder)
Congenital Disease	SNOMED	50992006	22q partial trisomy (disorder)
Congenital Disease	SNOMED	51010007	Congenital organoaxial volvulus of stomach (disorder)
Congenital Disease	SNOMED	5102002	Agenesis of corpus callosum (disorder)
Congenital Disease	SNOMED	51038004	Congenital obstruction of bile duct (disorder)
Congenital Disease	SNOMED	51053007	Hemoglobin C disease (disorder)
Congenital Disease	SNOMED	51060001	Diprosopus tetrophthalmus (disorder)
Congenital Disease	SNOMED	51062009	Congenital constriction of pylorus (disorder)
Congenital Disease	SNOMED	51089004	Birthmark (disorder)
Congenital Disease	SNOMED	51118003	Congenital atresia of duodenum (disorder)
Congenital Disease	SNOMED	51174006	Congenital melanosis of sclera (disorder)
Congenital Disease	SNOMED	51219000	Bifid thumb (disorder)
Congenital Disease	SNOMED	5127009	Jaw-winking syndrome (disorder)
Congenital Disease	SNOMED	5132005	Keratosis pilaris (disorder)
Congenital Disease	SNOMED	51409009	Asymmetric crying face association (disorder)
Congenital Disease	SNOMED	51442005	Congenital atresia of aortic valve (disorder)
Congenital Disease	SNOMED	51485001	Congenital coloboma of iris (disorder)
Congenital Disease	SNOMED	51500006	Complete trisomy 18 syndrome (disorder)
Congenital Disease	SNOMED	51523009	Congenital laryngocele (disorder)
Congenital Disease	SNOMED	5153001	Ectopic anus (disorder)
Congenital Disease	SNOMED	51603000	Cutis verticis gyrata (disorder)
Congenital Disease	SNOMED	51635000	Congenital absence of all fingers (disorder)
Congenital Disease	SNOMED	51655004	Congenital anomaly of skull (disorder)
Congenital Disease	SNOMED	51665005	Facies bovina (finding)
Congenital Disease	SNOMED	51693009	Congenital absence of prostate (disorder)
Congenital Disease	SNOMED	51780007	Cerebro-costo-mandibular syndrome (disorder)
Congenital Disease	SNOMED	51789008	Congenital malposition of cardiac apex (disorder)
Congenital Disease	SNOMED	51794008	Congenital anomaly of ureter (disorder)
Congenital Disease	SNOMED	51819009	17p partial trisomy syndrome (disorder)
Congenital Disease	SNOMED	5187006	Prune belly syndrome (disorder)
Congenital Disease	SNOMED	51952004	Spondyloepiphyseal dysplasia tarda (disorder)
Congenital Disease	SNOMED	520004	Congenital bent nose (disorder)
Congenital Disease	SNOMED	52022007	Congenital absence of ulna (disorder)
Congenital Disease	SNOMED	52083000	Infantile lobar overinflation of lung (disorder)
Congenital Disease	SNOMED	52159006	Universal mesentery (disorder)
Congenital Disease	SNOMED	5230009	Congenital absence of coronary artery (disorder)
Congenital Disease	SNOMED	52330001	Meningoencephalocele (disorder)
Congenital Disease	SNOMED	52415006	Low assimilation pelvis (disorder)
Congenital Disease	SNOMED	52474000	Ectromelia of lower limb (disorder)
Congenital Disease	SNOMED	52579008	Accessory lung (disorder)
Congenital Disease	SNOMED	52616002	Freeman-Sheldon syndrome (disorder)
Congenital Disease	SNOMED	52713000	Infantile neuroaxonal dystrophy (disorder)
Congenital Disease	SNOMED	52757001	Congenital supravalvular pulmonary stenosis (disorder)
Congenital Disease	SNOMED	52781008	Congenital hip dysplasia (disorder)
Congenital Disease	SNOMED	5283001	Hound-dog facies (finding)
Congenital Disease	SNOMED	52837007	Longitudinal deficiency of femur (disorder)
Congenital Disease	SNOMED	5286009	Congenital absence of vas deferens (disorder)
Congenital Disease	SNOMED	52868006	Oral-facial-digital syndrome (disorder)
Congenital Disease	SNOMED	52879001	Congenital atresia of glottis (disorder)
Congenital Disease	SNOMED	52904006	Congenital anomaly of vagina (disorder)
Congenital Disease	SNOMED	53076002	Congenital gastric perforation (disorder)
Congenital Disease	SNOMED	53189005	Congenital atresia of trachea (disorder)
Congenital Disease	SNOMED	53190001	Vascular compression of esophagus by aberrant artery (disorder)
Congenital Disease	SNOMED	53318002	Spina bifida with hydrocephalus (disorder)
Congenital Disease	SNOMED	53329003	Platypellic pelvis (disorder)
Congenital Disease	SNOMED	53346000	Complete trisomy 20 syndrome (disorder)
Congenital Disease	SNOMED	53392002	Anomaly of chromosome pair 16 (disorder)
Congenital Disease	SNOMED	53599007	Testicular regression syndrome (disorder)
Congenital Disease	SNOMED	5361003	Embryonal nuclear cataract (disorder)
Congenital Disease	SNOMED	53633000	Peutz-Jeghers polyps of small bowel (disorder)
Congenital Disease	SNOMED	5364006	Uterus subseptus (disorder)
Congenital Disease	SNOMED	53697002	Accessory lacrimal canal (disorder)
Congenital Disease	SNOMED	53748002	Congenital junctional epidermolysis bullosa-pyloric atresia syndrome (disorder)
Congenital Disease	SNOMED	53776005	Encephalocystocele (disorder)
Congenital Disease	SNOMED	53790008	Beaked pelvis (disorder)
Congenital Disease	SNOMED	53842005	Congenital hallux valgus (disorder)
Congenital Disease	SNOMED	5397007	Congenital anomaly of renal pelvis (disorder)
Congenital Disease	SNOMED	53974002	Kniest dysplasia (disorder)
Congenital Disease	SNOMED	54008006	Sternum bifidum (disorder)

Congenital Disease	SNOMED	54036001	Oto-palato-digital syndrome, type I (disorder)
Congenital Disease	SNOMED	54073003	Monocuspid cardiac valve (disorder)
Congenital Disease	SNOMED	54160000	Congenital aneurysm of sinus of Valsalva (disorder)
Congenital Disease	SNOMED	54176009	Congenital cyst of canal of Nuck (disorder)
Congenital Disease	SNOMED	54209007	Hidrotic ectodermal dysplasia syndrome (disorder)
Congenital Disease	SNOMED	54265003	Congenital anomaly of cerebral artery (disorder)
Congenital Disease	SNOMED	5432003	Transposition of appendix (disorder)
Congenital Disease	SNOMED	54336006	Ichthyosis linearis circumflexa (disorder)
Congenital Disease	SNOMED	54359000	Congenital corneal opacity not interfering with vision (disorder)
Congenital Disease	SNOMED	54386000	Congenital fusion of ossicles of ear (disorder)
Congenital Disease	SNOMED	54411001	Peutz-Jeghers syndrome (disorder)
Congenital Disease	SNOMED	54554009	Pancreas divisum (disorder)
Congenital Disease	SNOMED	54616001	Congenital hypertrophy of pylorus (disorder)
Congenital Disease	SNOMED	54668008	Uterus biforis (disorder)
Congenital Disease	SNOMED	54682008	Congenital hypoplasia of pulmonary artery (disorder)
Congenital Disease	SNOMED	54694004	Mobile cecum (disorder)
Congenital Disease	SNOMED	54764003	Synchilia (disorder)
Congenital Disease	SNOMED	54794009	Ectopic gray matter in centrum ovale (disorder)
Congenital Disease	SNOMED	548004	13p partial trisomy syndrome (disorder)
Congenital Disease	SNOMED	54837006	Straight back syndrome (disorder)
Congenital Disease	SNOMED	54873004	Congenital anomaly of orbit proper (disorder)
Congenital Disease	SNOMED	54967001	Double kidney (disorder)
Congenital Disease	SNOMED	55016009	Congenital muscular hypertrophy-cerebral syndrome (disorder)
Congenital Disease	SNOMED	55193002	Congenital anomaly of small intestine (disorder)
Congenital Disease	SNOMED	55379003	Congenital pseudoarthrosis of tibia (disorder)
Congenital Disease	SNOMED	554003	2p partial trisomy syndrome (disorder)
Congenital Disease	SNOMED	55510008	Cor triatriatum (disorder)
Congenital Disease	SNOMED	55520003	Vascular compression of esophagus by aberrant right subclavian artery arising from descending aorta (disorder)
Congenital Disease	SNOMED	55536001	Congenital malposition of kidney (disorder)
Congenital Disease	SNOMED	55546004	Anomalous origin of left circumflex artery from right coronary artery (disorder)
Congenital Disease	SNOMED	55631001	Congenital anomaly of testis (disorder)
Congenital Disease	SNOMED	5565008	Congenital diverticulum of trachea (disorder)
Congenital Disease	SNOMED	55709000	Ethmocephalus (disorder)
Congenital Disease	SNOMED	55711009	Ehlers-Danlos syndrome, procollagen proteinase deficient (disorder)
Congenital Disease	SNOMED	55819001	Albinotic fundus (disorder)
Congenital Disease	SNOMED	55821006	Hay-Wells syndrome of ectodermal dysplasia (disorder)
Congenital Disease	SNOMED	55852007	Complete phocomelia of lower limb (disorder)
Congenital Disease	SNOMED	55856005	Congenital hyperplasia of kidney (disorder)
Congenital Disease	SNOMED	55999004	Encephalocele (disorder)
Congenital Disease	SNOMED	5601008	Klippel-Feil sequence (disorder)
Congenital Disease	SNOMED	56013008	Congenital duplication of biliary duct (disorder)
Congenital Disease	SNOMED	56048001	Xeroderma pigmentosum, group E (disorder)
Congenital Disease	SNOMED	56108007	Congenital pelvic kidney (disorder)
Congenital Disease	SNOMED	56155002	Hemispheric cerebral agenesis (disorder)
Congenital Disease	SNOMED	5619004	Bardet-Biedl syndrome (disorder)
Congenital Disease	SNOMED	56212008	Leydig cell agenesis (disorder)
Congenital Disease	SNOMED	56309007	Congenital subaortic stenosis of tunnel type (disorder)
Congenital Disease	SNOMED	56364004	Fibrous hamartoma of infancy (disorder)
Congenital Disease	SNOMED	5645008	Nasal glial heterotopia (disorder)
Congenital Disease	SNOMED	56525001	Nevus lipomatosus cutaneous superficialis (disorder)
Congenital Disease	SNOMED	56531003	Ulegyria (disorder)
Congenital Disease	SNOMED	56558005	Congenital hypotrichia (disorder)
Congenital Disease	SNOMED	56604005	Cohen syndrome (disorder)
Congenital Disease	SNOMED	56643009	Blunderbuss pelvis (disorder)
Congenital Disease	SNOMED	56653005	18p partial monosomy syndrome (disorder)
Congenital Disease	SNOMED	56677004	Pallister-Hall syndrome (disorder)
Congenital Disease	SNOMED	56692003	Rhizomelic chondrodysplasia punctata syndrome (disorder)
Congenital Disease	SNOMED	56759000	Congenital anomaly of subcutaneous tissue (disorder)
Congenital Disease	SNOMED	56797000	Congenital hypertrichosis (disorder)
Congenital Disease	SNOMED	56823000	Cervical thyroid remnant (disorder)
Congenital Disease	SNOMED	56975005	Strawberry nevus of skin (disorder)
Congenital Disease	SNOMED	57014008	Fistula colli congenita (disorder)
Congenital Disease	SNOMED	57058008	Uterus bicornis unicollis (disorder)
Congenital Disease	SNOMED	57088004	Microcystic renal disease (disorder)
Congenital Disease	SNOMED	57148006	Congenital anomaly of brain (disorder)
Congenital Disease	SNOMED	57201002	Marfanoid joint hypermobility syndrome (disorder)
Congenital Disease	SNOMED	57219006	Craniosynostosis syndrome (disorder)
Congenital Disease	SNOMED	57265009	Congenital obstruction of large intestine (disorder)
Congenital Disease	SNOMED	5731000119108	Congenital osteodystrophy (disorder)
Congenital Disease	SNOMED	57361003	Anomaly of chromosome pair 5 (disorder)
Congenital Disease	SNOMED	57436000	Congenital absence of external ear (disorder)
Congenital Disease	SNOMED	57451009	Congenital tracheobronchomegaly (disorder)
Congenital Disease	SNOMED	57497006	Congenital anomaly of spleen (disorder)

Congenital Disease	SNOMED	57514000	3-Oxo-5 alpha-steroid delta 4-dehydrogenase deficiency (disorder)
Congenital Disease	SNOMED	57544002	Multiple malformation syndrome due to non-infectious environmental agents (disorder)
Congenital Disease	SNOMED	57917004	Seckel syndrome (disorder)
Congenital Disease	SNOMED	57918009	Janiceps (disorder)
Congenital Disease	SNOMED	57921006	Congenital adhesions of omentum (disorder)
Congenital Disease	SNOMED	58010002	Congenital absence of tibia AND fibula (disorder)
Congenital Disease	SNOMED	58037000	Cowden syndrome (disorder)
Congenital Disease	SNOMED	58135005	Congenital duplication of cervix (disorder)
Congenital Disease	SNOMED	5842009	Spinal cord dysplasia (disorder)
Congenital Disease	SNOMED	58557008	Spina bifida aperta (disorder)
Congenital Disease	SNOMED	58561002	Diastrophic dysplasia (disorder)
Congenital Disease	SNOMED	58588007	Cutis laxa (disorder)
Congenital Disease	SNOMED	58670006	Accessory lacrimal gland disorder (disorder)
Congenital Disease	SNOMED	58694006	Teratogenesis (finding)
Congenital Disease	SNOMED	58724008	Rib syndrome (finding)
Congenital Disease	SNOMED	58882000	Congenital cystic eyeball (disorder)
Congenital Disease	SNOMED	590005	Congenital aneurysm of anterior communicating artery (disorder)
Congenital Disease	SNOMED	59033006	Anomaly of chromosome pair 18 (disorder)
Congenital Disease	SNOMED	59035004	Congenital anomaly of female genital system (disorder)
Congenital Disease	SNOMED	59068006	Congenital dislocation of knee (disorder)
Congenital Disease	SNOMED	59070002	Partial aphalangia of lower limb (disorder)
Congenital Disease	SNOMED	59128005	Congenital honeycomb lung (disorder)
Congenital Disease	SNOMED	59252009	Cutis laxa-corneal clouding-oligophrenia syndrome (disorder)
Congenital Disease	SNOMED	59352006	Longitudinal deficiency of radius AND ulna (disorder)
Congenital Disease	SNOMED	59399004	Cutis laxa, x-linked (disorder)
Congenital Disease	SNOMED	5941000119101	Congenital single renal cyst (disorder)
Congenital Disease	SNOMED	59423009	Congenital atresia of nasopharynx (disorder)
Congenital Disease	SNOMED	59451000	Cutis laxa, autosomal recessive (disorder)
Congenital Disease	SNOMED	59494005	Congenital septal defect of heart (disorder)
Congenital Disease	SNOMED	59514009	Congenital absence of large intestine (disorder)
Congenital Disease	SNOMED	59554006	Holoacardius acormus (disorder)
Congenital Disease	SNOMED	59600000	Persistent omphalomesenteric artery (disorder)
Congenital Disease	SNOMED	59631007	Anomalous pulmonary venous drainage (disorder)
Congenital Disease	SNOMED	5968001	Congenital fusion of sacroiliac joint (disorder)
Congenital Disease	SNOMED	59693007	Manus plana (disorder)
Congenital Disease	SNOMED	59708000	Multiple epiphyseal dysplasia (disorder)
Congenital Disease	SNOMED	59758007	Trifid pelvis of kidney (disorder)
Congenital Disease	SNOMED	59777009	Thoracopagus (disorder)
Congenital Disease	SNOMED	5982001	2q partial trisomy syndrome (disorder)
Congenital Disease	SNOMED	59857007	Branchial cleft cyst (disorder)
Congenital Disease	SNOMED	59877000	Congenital anomaly of aorta (disorder)
Congenital Disease	SNOMED	59981001	Congenital absence of penis (disorder)
Congenital Disease	SNOMED	6002006	10p partial monosomy syndrome (disorder)
Congenital Disease	SNOMED	60091004	Congenital stricture of artery (disorder)
Congenital Disease	SNOMED	60106004	Common arterial trunk and separate origin of pulmonary arteries (disorder)
Congenital Disease	SNOMED	60192008	Lethal multiple pterygium syndrome (disorder)
Congenital Disease	SNOMED	60220000	Partial congenital absence of limb (disorder)
Congenital Disease	SNOMED	60232001	Cleft leaflet of tricuspid valve (disorder)
Congenital Disease	SNOMED	60291005	Congenital absence of clitoris (disorder)
Congenital Disease	SNOMED	60399005	Dermatofibrosis lenticularis disseminata (disorder)
Congenital Disease	SNOMED	60447007	Congenital anomaly of tongue (disorder)
Congenital Disease	SNOMED	60475009	Congenital anomaly of limb (disorder)
Congenital Disease	SNOMED	60505005	Congenital anomaly of optic disc (disorder)
Congenital Disease	SNOMED	60628003	Mediterranean macrothrombocytopenia (disorder)
Congenital Disease	SNOMED	60637003	Congenital anomaly of adrenal gland (disorder)
Congenital Disease	SNOMED	60650002	Ring chromosome 9 syndrome (disorder)
Congenital Disease	SNOMED	60652005	Congenital cyst of mediastinum (disorder)
Congenital Disease	SNOMED	60680007	Duplication of duodenum (disorder)
Congenital Disease	SNOMED	60699003	Congenital anomaly of stomach (disorder)
Congenital Disease	SNOMED	60732002	Atrial septal defect with endocardial cushion defect, partial type (disorder)
Congenital Disease	SNOMED	60787001	Congenital hypoplasia of aortic arch (disorder)
Congenital Disease	SNOMED	60891003	Anomaly of chromosome pair 11 (disorder)
Congenital Disease	SNOMED	609209009	Infection of preauricular sinus (disorder)
Congenital Disease	SNOMED	609211000	Abscess of preauricular sinus (disorder)
Congenital Disease	SNOMED	60922004	Heterologous chimera (disorder)
Congenital Disease	SNOMED	609417004	Fetal anencephaly (disorder)
Congenital Disease	SNOMED	609437000	Fetal Alcohol Spectrum Disorder (disorder)
Congenital Disease	SNOMED	609528003	Posterior fossa arachnoid cyst (disorder)
Congenital Disease	SNOMED	609529006	Persistent Blake's pouch cyst (disorder)
Congenital Disease	SNOMED	60983006	Congenital esophagotracheal fistula (disorder)
Congenital Disease	SNOMED	61003004	Epidermolysis bullosa (disorder)
Congenital Disease	SNOMED	61108006	Ectopic intestinal mucosa (disorder)
Congenital Disease	SNOMED	61142002	Microphthalmos (disorder)
Congenital Disease	SNOMED	61293001	Congenital deformity of knee joint (disorder)

Congenital Disease	SNOMED	613003	Fragile X syndrome (disorder)
Congenital Disease	SNOMED	61367005	Jarcho-Levin syndrome (disorder)
Congenital Disease	SNOMED	61476004	Embryonic cyst of vagina (disorder)
Congenital Disease	SNOMED	61649007	Congenital oculocutaneous hypopigmentation (disorder)
Congenital Disease	SNOMED	61758007	Exstrophy of bladder sequence (disorder)
Congenital Disease	SNOMED	61819007	Rachischisis (disorder)
Congenital Disease	SNOMED	61852001	Ask-Upmark kidney (disorder)
Congenital Disease	SNOMED	61870003	Homologous chimera (disorder)
Congenital Disease	SNOMED	61900003	Longitudinal deficiency of radius (disorder)
Congenital Disease	SNOMED	61959006	Common truncus arteriosus (disorder)
Congenital Disease	SNOMED	62023000	Arterial anomaly of umbilical cord (disorder)
Congenital Disease	SNOMED	62042001	Splenogonadal fusion (disorder)
Congenital Disease	SNOMED	62058003	Congenital hourglass stomach (disorder)
Congenital Disease	SNOMED	62067003	Hypoplastic left heart syndrome (disorder)
Congenital Disease	SNOMED	62110005	Fetal methyl mercury syndrome (disorder)
Congenital Disease	SNOMED	62158001	Status marmoratus (disorder)
Congenital Disease	SNOMED	62192003	Diprosopus (disorder)
Congenital Disease	SNOMED	62216007	Familial arthrogryposis-cholestatic hepatorenal syndrome (disorder)
Congenital Disease	SNOMED	62218008	Polydactyly of toes (disorder)
Congenital Disease	SNOMED	62250003	Arachnodactyly (disorder)
Congenital Disease	SNOMED	62335009	Congenital stenosis of inferior vena cava (disorder)
Congenital Disease	SNOMED	62500006	Accessory bladder (disorder)
Congenital Disease	SNOMED	62501005	Chondroectodermal dysplasia (disorder)
Congenital Disease	SNOMED	62524003	Ectopic ovary (disorder)
Congenital Disease	SNOMED	62588002	Congenital complete absence of limb (disorder)
Congenital Disease	SNOMED	62628008	Multiple synostosis syndrome (disorder)
Congenital Disease	SNOMED	62631009	Congenital cyst of posterior segment of eye (disorder)
Congenital Disease	SNOMED	62654000	Failure of rotation of cecum (disorder)
Congenital Disease	SNOMED	62667002	Congenital deviation of nasal septum (disorder)
Congenital Disease	SNOMED	62727008	Angiokeratoma of Mibelli (disorder)
Congenital Disease	SNOMED	62803002	Frontometaphyseal dysplasia (disorder)
Congenital Disease	SNOMED	62815003	Incomplete bilateral cleft lip (disorder)
Congenital Disease	SNOMED	6282000	Congenital anomaly of vas deferens (disorder)
Congenital Disease	SNOMED	62821000119106	Anomaly of fetal ear (disorder)
Congenital Disease	SNOMED	6296006	Congenital dilatation of trachea (disorder)
Congenital Disease	SNOMED	62964007	Antley-Bixler syndrome (disorder)
Congenital Disease	SNOMED	62977003	Persistent tunica vasculosa lentis (disorder)
Congenital Disease	SNOMED	62991000119101	Fetal abdominal wall defect (disorder)
Congenital Disease	SNOMED	63051000119101	Fetal clubfoot (disorder)
Congenital Disease	SNOMED	63061000119104	Fetal cleft lip (disorder)
Congenital Disease	SNOMED	63119004	Weaver syndrome (disorder)
Congenital Disease	SNOMED	63168004	Peripheral congenital arteriovenous aneurysm (disorder)
Congenital Disease	SNOMED	63175003	Localized extracutaneous mastocytosis (disorder)
Congenital Disease	SNOMED	63216003	Congenital keratoconus (disorder)
Congenital Disease	SNOMED	63247009	Williams syndrome (disorder)
Congenital Disease	SNOMED	63253009	Congenital rectovestibular fistula (disorder)
Congenital Disease	SNOMED	63340009	Acephalocheiria (disorder)
Congenital Disease	SNOMED	63387002	Larsen syndrome (disorder)
Congenital Disease	SNOMED	63391007	Ulnar dimelia (disorder)
Congenital Disease	SNOMED	63413008	Accessory ossification center (disorder)
Congenital Disease	SNOMED	63567004	Cleft hard and soft palate (disorder)
Congenital Disease	SNOMED	63702009	Alstrom syndrome (disorder)
Congenital Disease	SNOMED	63711009	Brachydactyly syndrome type E (disorder)
Congenital Disease	SNOMED	6380005	Inverted pelvis (disorder)
Congenital Disease	SNOMED	63890001	Osteogenesis imperfecta with blue sclerae AND dentinogenesis imperfecta (disorder)
Congenital Disease	SNOMED	63934006	Overriding aorta (disorder)
Congenital Disease	SNOMED	63935007	Congenital absence of eyelash (disorder)
Congenital Disease	SNOMED	64162006	Multiple malformation syndrome with unusual brain and/or neuromuscular findings (disorder)
Congenital Disease	SNOMED	64233004	Embryonic cyst of fimbria of fallopian tube (disorder)
Congenital Disease	SNOMED	64283009	Congenital rectovesical fistula (disorder)
Congenital Disease	SNOMED	64320007	Congenital chordee (disorder)
Congenital Disease	SNOMED	64404003	Osteogenesis imperfecta with blue sclerae AND normal teeth (disorder)
Congenital Disease	SNOMED	64490002	Anakatadidymus (disorder)
Congenital Disease	SNOMED	64596006	Congenital clubnail (disorder)
Congenital Disease	SNOMED	64727007	Accessory ureter (disorder)
Congenital Disease	SNOMED	6477005	Malrotation of colon (disorder)
Congenital Disease	SNOMED	64981002	Congenital atresia of larynx (disorder)
Congenital Disease	SNOMED	65033000	Congenital anomaly of hair (disorder)
Congenital Disease	SNOMED	65048006	Accessory parathyroid gland (disorder)
Congenital Disease	SNOMED	65094009	Multiple malformation syndrome with facial defects as major feature (disorder)
Congenital Disease	SNOMED	65144005	Congenital spinal meningocele (disorder)
Congenital Disease	SNOMED	65146007	Ectopic spleen (disorder)

Congenital Disease	SNOMED	65178004	Deventer's pelvis (disorder)
Congenital Disease	SNOMED	65237009	Accessory thyroid gland (disorder)
Congenital Disease	SNOMED	65274008	Congenital anomaly of parathyroid glands (disorder)
Congenital Disease	SNOMED	6537000	Ectopic pituitary tissue (disorder)
Congenital Disease	SNOMED	65455002	Nasal encephalocele (disorder)
Congenital Disease	SNOMED	65573001	High assimilation pelvis (disorder)
Congenital Disease	SNOMED	65587001	Congenital anomaly of cerebrovascular system (disorder)
Congenital Disease	SNOMED	65704008	Congenital anomaly of vulva (disorder)
Congenital Disease	SNOMED	6573007	Reniform pelvis (disorder)
Congenital Disease	SNOMED	65937002	Imperforate hymen (disorder)
Congenital Disease	SNOMED	65950001	Bayonet hair (disorder)
Congenital Disease	SNOMED	65976001	Cleidocranial dysostosis (disorder)
Congenital Disease	SNOMED	65986000	Fetal aminopterin syndrome (disorder)
Congenital Disease	SNOMED	65997000	Congenital fold of posterior segment of eye (disorder)
Congenital Disease	SNOMED	66038001	Miller syndrome (disorder)
Congenital Disease	SNOMED	66042003	Congenital hyperplasia of muscle (disorder)
Congenital Disease	SNOMED	66078008	Longitudinal deficiency of lower limb (disorder)
Congenital Disease	SNOMED	66102006	Congenital fusion of ribs (disorder)
Congenital Disease	SNOMED	66345008	Congenital absence of toe (disorder)
Congenital Disease	SNOMED	66351003	Fetal trimethadione syndrome (disorder)
Congenital Disease	SNOMED	6636004	Congenital cardiovascular disorder in mother complicating pregnancy, childbirth AND/OR puerperium (disorder)
Congenital Disease	SNOMED	66403007	Vascular ring of aorta (disorder)
Congenital Disease	SNOMED	66426000	Craniopagus parietalis (disorder)
Congenital Disease	SNOMED	66489009	Congenital absence of lung (disorder)
Congenital Disease	SNOMED	66499004	Congenital cortical cataract (disorder)
Congenital Disease	SNOMED	66510004	Congenital anomaly of upper limb (disorder)
Congenital Disease	SNOMED	66597000	Congenital cervicoaural fistula (disorder)
Congenital Disease	SNOMED	66651005	Triploidy syndrome (disorder)
Congenital Disease	SNOMED	6667002	Anadidymus (disorder)
Congenital Disease	SNOMED	66729008	Hemoglobin D disease (disorder)
Congenital Disease	SNOMED	66758006	Acrodysostosis (disorder)
Congenital Disease	SNOMED	66783006	Popliteal pterygium syndrome (disorder)
Congenital Disease	SNOMED	66793004	Congenital absence of alimentary tract (disorder)
Congenital Disease	SNOMED	66858001	Anomalous pulmonary venous drainage to superior vena cava (disorder)
Congenital Disease	SNOMED	66865009	Congenital duplication of esophagus (disorder)
Congenital Disease	SNOMED	6688006	Congenital absence of patella (disorder)
Congenital Disease	SNOMED	66948001	Cleft palate with cleft lip (disorder)
Congenital Disease	SNOMED	66985009	18q partial trisomy syndrome (disorder)
Congenital Disease	SNOMED	67012005	Pyloric antral atresia (disorder)
Congenital Disease	SNOMED	67144006	Epidermolysis bullosa simplex (disorder)
Congenital Disease	SNOMED	67202007	Ehlers-Danlos syndrome, type 5 (disorder)
Congenital Disease	SNOMED	6724001	Ectopic pancreas in duodenum (disorder)
Congenital Disease	SNOMED	6729006	Cerebral-retinal arteriovenous aneurysm (disorder)
Congenital Disease	SNOMED	67341007	Longitudinal deficiency of limb (disorder)
Congenital Disease	SNOMED	673791000119108	Congenital deformity of soft tissue (disorder)
Congenital Disease	SNOMED	67510007	Ichthyosis hystrix (disorder)
Congenital Disease	SNOMED	67531005	Spina bifida (disorder)
Congenital Disease	SNOMED	67653003	Pretibial epidermolysis bullosa (disorder)
Congenital Disease	SNOMED	67787004	Tongue tie (disorder)
Congenital Disease	SNOMED	67817003	Pili torti-deafness syndrome (disorder)
Congenital Disease	SNOMED	67876003	Congenital obstruction of aqueduct of Sylvius (disorder)
Congenital Disease	SNOMED	67904000	Dwarf pelvis (disorder)
Congenital Disease	SNOMED	67944007	Lhermitte-Duclos disease (disorder)
Congenital Disease	SNOMED	67985002	Congenital anomaly of membranous labyrinth (disorder)
Congenital Disease	SNOMED	67988000	Congenital anomaly of cartilage (disorder)
Congenital Disease	SNOMED	679911000119104	Congenital cortical cataract of left eye (disorder)
Congenital Disease	SNOMED	679931000119109	Congenital cortical cataract of right eye (disorder)
Congenital Disease	SNOMED	68092007	Anomalous origin of pulmonary artery (disorder)
Congenital Disease	SNOMED	681041000119106	Congenital cortical cataract of bilateral eyes (disorder)
Congenital Disease	SNOMED	68237008	Partial anomalous pulmonary venous connection (disorder)
Congenital Disease	SNOMED	68284008	Talipes equinovalgus (disorder)
Congenital Disease	SNOMED	68352004	Accessory adrenal gland (disorder)
Congenital Disease	SNOMED	68359008	Congenital hemivertebra (disorder)
Congenital Disease	SNOMED	6839008	Vertebral abnormalities, anal atresia, cardiac abnormalities, tracheo-esophageal fistula, limb defects syndrome (disorder)
Congenital Disease	SNOMED	68454002	Complete trisomy 8 syndrome (disorder)
Congenital Disease	SNOMED	68539005	Congenital bronchopulmonary foregut malformation (disorder)
Congenital Disease	SNOMED	68551007	Limb reduction-ichthyosis syndrome (disorder)
Congenital Disease	SNOMED	68557006	Talipes planovalgus (disorder)
Congenital Disease	SNOMED	68591005	Congenital hypoplasia of pancreas (disorder)
Congenital Disease	SNOMED	68637004	Xeroderma pigmentosum, group D (disorder)
Congenital Disease	SNOMED	68716001	Situs inversus abdominalis (disorder)
Congenital Disease	SNOMED	6874009	Congenital keratoderma (disorder)
Congenital Disease	SNOMED	68914007	Congenital mesenteroaxial volvulus of stomach (disorder)

Congenital Disease	SNOMED	68926002	Idiopathic arterial calcification of infancy (disorder)
Congenital Disease	SNOMED	69015003	Double urethra (disorder)
Congenital Disease	SNOMED	69056000	Macrotia (disorder)
Congenital Disease	SNOMED	69093006	Rothmund-Thomson syndrome (disorder)
Congenital Disease	SNOMED	69248007	Congenital anomaly of fixation of intestine (disorder)
Congenital Disease	SNOMED	69278003	Congenital aniridia (disorder)
Congenital Disease	SNOMED	6936002	Cleft lip sequence (disorder)
Congenital Disease	SNOMED	69381005	Macrodactylia of fingers (disorder)
Congenital Disease	SNOMED	69390003	Vascular hamartoma of skin (disorder)
Congenital Disease	SNOMED	69408002	Gorlin syndrome (disorder)
Congenital Disease	SNOMED	69488000	Beaded hair (disorder)
Congenital Disease	SNOMED	69518005	Congenital anomaly of digestive system (disorder)
Congenital Disease	SNOMED	69664005	Ecchordosis physaliphora (disorder)
Congenital Disease	SNOMED	69729007	Unilobar lung (disorder)
Congenital Disease	SNOMED	69771008	Congenital anomaly of esophagus (disorder)
Congenital Disease	SNOMED	697905000	Pulmonary arterial hypertension associated with congenital heart disease (disorder)
Congenital Disease	SNOMED	697906004	Pulmonary arterial hypertension associated with congenital systemic-to-pulmonary shunt (disorder)
Congenital Disease	SNOMED	697916007	Pulmonary hypertension due to developmental abnormality of the lung (disorder)
Congenital Disease	SNOMED	697924002	Pulmonary hypertension in neurofibromatosis (disorder)
Congenital Disease	SNOMED	697928004	Pulmonary venous hypertension due to congenital stenosis of pulmonary vein (disorder)
Congenital Disease	SNOMED	69817007	Congenital occlusion of anus (disorder)
Congenital Disease	SNOMED	698597005	Single artery of fetal umbilical cord on obstetric ultrasound scan (finding)
Congenital Disease	SNOMED	698601005	Single congenital anomaly (disorder)
Congenital Disease	SNOMED	698765007	Posterior fossa brain malformation, hemangioma, arterial anomaly, cardiac defect and aortic coarctation, and eye abnormality syndrome (disorder)
Congenital Disease	SNOMED	698766008	Posterior fossa brain malformation, haemaniogma, arterial anomaly, cardiac defect and aortic coarctation, eye abnormality synodrome and sternal anomaly syndrome (disorder)
Congenital Disease	SNOMED	698849002	Tetrasomy 18p (disorder)
Congenital Disease	SNOMED	698851003	SOX2 anophthalmia syndrome (disorder)
Congenital Disease	SNOMED	698971006	Congenital subaortic diverticulum (disorder)
Congenital Disease	SNOMED	698999002	Congenital atresia of aqueduct of Sylvius (disorder)
Congenital Disease	SNOMED	699000000	Thyroglossal duct sinus (disorder)
Congenital Disease	SNOMED	699049007	Acrosyndactyly of toe (disorder)
Congenital Disease	SNOMED	699051006	Male subcoronal hypospadias (disorder)
Congenital Disease	SNOMED	699055002	Prominent renal pelvis (disorder)
Congenital Disease	SNOMED	699185005	Myostatin related hypertrophy of muscle (disorder)
Congenital Disease	SNOMED	699254009	Microdeletion of chromosome 15q13.3 (disorder)
Congenital Disease	SNOMED	699255005	Left to right flow of foramen ovale of fetal heart (disorder)
Congenital Disease	SNOMED	699256006	Timothy syndrome type 1 (disorder)
Congenital Disease	SNOMED	69927002	Persistent primary vitreous (disorder)
Congenital Disease	SNOMED	699275001	WNT4 Müllerian aplasia and ovarian dysfunction (disorder)
Congenital Disease	SNOMED	699297004	Blepharophimosis-intellectual disability syndrome Maat-Kievit-Brunner type (disorder)
Congenital Disease	SNOMED	699298009	Blepharophimosis, intellectual disability syndrome, Say-Barber-Biesecker-Young-Simpson type (disorder)
Congenital Disease	SNOMED	699300009	Oculofaciocardiodental syndrome (disorder)
Congenital Disease	SNOMED	699301008	Multiple venous malformation of skin and mucous membrane (disorder)
Congenital Disease	SNOMED	699305004	1q21.1 microdeletion syndrome (disorder)
Congenital Disease	SNOMED	699306003	Chromosome 1p36 deletion syndrome (disorder)
Congenital Disease	SNOMED	699307007	Chromosome 16p11.2 deletion syndrome (disorder)
Congenital Disease	SNOMED	699308002	Microdeletion of chromosome 15q24 (disorder)
Congenital Disease	SNOMED	699310000	22q13.3 deletion syndrome (disorder)
Congenital Disease	SNOMED	699311001	22q11.2 duplication syndrome (disorder)
Congenital Disease	SNOMED	699316006	Myhre syndrome (disorder)
Congenital Disease	SNOMED	699381006	Oculoskeletal dysplasia (disorder)
Congenital Disease	SNOMED	699420006	Hemifacial myohyperplasia (disorder)
Congenital Disease	SNOMED	699447001	Zimmermann-Laband syndrome (disorder)
Congenital Disease	SNOMED	6996004	Congenital absence of pulmonary valve (disorder)
Congenital Disease	SNOMED	699669001	Renpenning syndrome (disorder)
Congenital Disease	SNOMED	699700006	Median cleft lip and cleft of alveolar process of maxilla (disorder)
Congenital Disease	SNOMED	699762000	Congenital double lip (disorder)
Congenital Disease	SNOMED	699783006	Brachycephalic face (finding)
Congenital Disease	SNOMED	699812002	Subependymal nodular heterotopia (disorder)
Congenital Disease	SNOMED	699871003	Congenital penile adhesion (disorder)
Congenital Disease	SNOMED	699943005	Congenital anomaly of vertebral region of back (disorder)
Congenital Disease	SNOMED	700051000	Sepsis in asplenic subject (disorder)
Congenital Disease	SNOMED	700056005	Mosaic variegated aneuploidy syndrome (disorder)
Congenital Disease	SNOMED	700062000	Schöpf-Schulz-Passarge syndrome (disorder)
Congenital Disease	SNOMED	700063005	Megalencephaly capillary malformation (disorder)
Congenital Disease	SNOMED	70013003	Asymmetrical conjoined twins (disorder)

Congenital Disease	SNOMED	700211007	Ulnar mammary syndrome (disorder)
Congenital Disease	SNOMED	700257009	Congenital megaduodenum (disorder)
Congenital Disease	SNOMED	700278007	Congenital vascular anomaly of eyelid (disorder)
Congenital Disease	SNOMED	700279004	Congenital vascular anomaly of upper eyelid (disorder)
Congenital Disease	SNOMED	700283004	Congenital achalasia of esophagus (disorder)
Congenital Disease	SNOMED	700285006	Congenital velopharyngeal dysfunction (disorder)
Congenital Disease	SNOMED	700369004	Congenital vascular anomaly of lower eyelid (disorder)
Congenital Disease	SNOMED	70041004	Erythrokeratodermia variabilis (disorder)
Congenital Disease	SNOMED	700489002	Sensorineural deafness and male infertility (disorder)
Congenital Disease	SNOMED	70065001	Fetal hydantoin syndrome (disorder)
Congenital Disease	SNOMED	70123009	Congenital cubitus valgus (disorder)
Congenital Disease	SNOMED	70142008	Atrial septal defect (disorder)
Congenital Disease	SNOMED	70156005	Anomaly of chromosome pair 21 (disorder)
Congenital Disease	SNOMED	70173007	5p partial monosomy syndrome (disorder)
Congenital Disease	SNOMED	70195006	Congenital anomaly of superior vena cava (disorder)
Congenital Disease	SNOMED	702312009	Tarsal-carpal coalition syndrome (disorder)
Congenital Disease	SNOMED	702313004	Tetra-amelia syndrome (disorder)
Congenital Disease	SNOMED	702339001	Spondyloperipheral dysplasia (disorder)
Congenital Disease	SNOMED	702342007	Dolichospondylic dysplasia (disorder)
Congenital Disease	SNOMED	702344008	Pitt-Hopkins syndrome (disorder)
Congenital Disease	SNOMED	702345009	Ring chromosome 14 syndrome (disorder)
Congenital Disease	SNOMED	702346005	Chromosome 11p11.2 deletion syndrome (disorder)
Congenital Disease	SNOMED	702350003	Spondyloepimetaphyseal dysplasia, Strudwick type (disorder)
Congenital Disease	SNOMED	702351004	Spondylocarpotarsal synostosis syndrome (disorder)
Congenital Disease	SNOMED	702354007	X-linked intellectual developmental disorder Christianson type (disorder)
Congenital Disease	SNOMED	702357000	Chromosome 2q37 deletion syndrome (disorder)
Congenital Disease	SNOMED	702358005	Persistent Müllerian duct syndrome (disorder)
Congenital Disease	SNOMED	702360007	Congenital deafness with labyrinthine aplasia, microtia and microdontia (disorder)
Congenital Disease	SNOMED	702361006	Crouzon syndrome with acanthosis nigricans (disorder)
Congenital Disease	SNOMED	702362004	Craniofacial deafness hand syndrome (disorder)
Congenital Disease	SNOMED	702367005	Genitopatellar syndrome (disorder)
Congenital Disease	SNOMED	702381007	Horizontal gaze palsy with progressive scoliosis (disorder)
Congenital Disease	SNOMED	702384004	Intrauterine growth restriction, metaphyseal dysplasia, adrenal hypoplasia congenita, and genital anomaly syndrome (disorder)
Congenital Disease	SNOMED	702397002	Renal tubular dysgenesis (disorder)
Congenital Disease	SNOMED	702400006	Spondyloepiphyseal dysplasia with congenital joint dislocations (disorder)
Congenital Disease	SNOMED	702407009	Hydrometrocolpos, postaxial polydactyly, and congenital heart malformation (disorder)
Congenital Disease	SNOMED	702413000	RAPADILINO syndrome (disorder)
Congenital Disease	SNOMED	702417004	Supernumerary der(22)t(11;22) syndrome (disorder)
Congenital Disease	SNOMED	702418009	Diaphragmatic hernia-exomphalos-hypertelorism syndrome (disorder)
Congenital Disease	SNOMED	702425002	Hand-foot-genital syndrome (disorder)
Congenital Disease	SNOMED	702431004	Microcephaly-oculo-digito-esophageal-duodenal syndrome (disorder)
Congenital Disease	SNOMED	702432006	Diaphragmatic hernia, abnormal face and distal limb anomalies (disorder)
Congenital Disease	SNOMED	702433001	Congenital cataracts, facial dysmorphism and neuropathy (disorder)
Congenital Disease	SNOMED	702434007	Distal penile hypospadias (disorder)
Congenital Disease	SNOMED	702436009	Proximal penile hypospadias (disorder)
Congenital Disease	SNOMED	702437000	Amish lethal microcephaly (disorder)
Congenital Disease	SNOMED	702443003	Auriculo-condylar syndrome (disorder)
Congenital Disease	SNOMED	702447002	Arthrogryposis-like syndrome (disorder)
Congenital Disease	SNOMED	702450004	FOXG1 syndrome (disorder)
Congenital Disease	SNOMED	702462000	Scrotal hypospadias (disorder)
Congenital Disease	SNOMED	702610009	Congenital aplasia of vas deferens (disorder)
Congenital Disease	SNOMED	702611008	Congenital brain aplasia (disorder)
Congenital Disease	SNOMED	702612001	Congenital aplasia of lung (disorder)
Congenital Disease	SNOMED	702613006	Aplasia of diaphragm (disorder)
Congenital Disease	SNOMED	702614000	Aplasia of parathyroid gland (disorder)
Congenital Disease	SNOMED	702615004	Aplasia of adrenal gland (disorder)
Congenital Disease	SNOMED	702616003	Aplasia of spermatic cord (disorder)
Congenital Disease	SNOMED	702623002	Aplasia of thymus (disorder)
Congenital Disease	SNOMED	702624008	Aplasia of spleen (disorder)
Congenital Disease	SNOMED	702627001	Aplasia of testicle (disorder)
Congenital Disease	SNOMED	702628006	Congenital anomaly of cerebrum (disorder)
Congenital Disease	SNOMED	702644002	Congenital stenosis of nasal pyriform aperture (disorder)
Congenital Disease	SNOMED	702816000	Methyl-cytosine phosphate guanine binding protein-2 duplication syndrome (disorder)
Congenital Disease	SNOMED	702829000	Warsaw breakage syndrome (disorder)
Congenital Disease	SNOMED	702957008	Talipes equinocavovarus (disorder)
Congenital Disease	SNOMED	703000	Congenital adhesions of tongue (disorder)
Congenital Disease	SNOMED	70318002	Congenital anomaly of penis (disorder)
Congenital Disease	SNOMED	703193000	Congenital malformation of dural sinus (disorder)
Congenital Disease	SNOMED	703198009	Peripheral venous malformation (disorder)
Congenital Disease	SNOMED	703221003	Congenital intracranial vascular malformation (disorder)
Congenital Disease	SNOMED	703265006	Arteriovenous malformation of face (disorder)

Congenital Disease	SNOMED	703266007	Cerebrofacial arteriovenous metameric syndrome (disorder)
Congenital Disease	SNOMED	703267003	Cerebrofacial arteriovenous metameric syndrome type 1 (disorder)
Congenital Disease	SNOMED	703268008	Cerebrofacial arteriovenous metameric syndrome type 3 (disorder)
Congenital Disease	SNOMED	703284009	Phakomatosis cesioflammea (disorder)
Congenital Disease	SNOMED	703285005	Phakomatosis spilorosea (disorder)
Congenital Disease	SNOMED	703286006	Phakomatosis caesiomarmorata (disorder)
Congenital Disease	SNOMED	703298001	Diffuse lymphatic malformation (disorder)
Congenital Disease	SNOMED	703299009	Cutaneous capillary malformation (disorder)
Congenital Disease	SNOMED	703329004	Arteriovenous malformation of trunk (disorder)
Congenital Disease	SNOMED	703331008	Arteriovenous malformation of limb (disorder)
Congenital Disease	SNOMED	703334000	Arteriovenous malformation of mandible (disorder)
Congenital Disease	SNOMED	703335004	Arteriovenous malformation of maxilla (disorder)
Congenital Disease	SNOMED	703336003	Arteriovenous malformation of frontonasal process (disorder)
Congenital Disease	SNOMED	703369003	Microcephaly-capillary malformation syndrome (disorder)
Congenital Disease	SNOMED	703385008	Anomalous origin of pulmonary artery from ascending aorta (disorder)
Congenital Disease	SNOMED	703388005	Growth retardation, amino aciduria, cholestasis, iron overload, lactic acidosis, and early death (disorder)
Congenital Disease	SNOMED	703389002	Calcium/calmodulin-dependent serine protein kinase related intellectual disability (disorder)
Congenital Disease	SNOMED	703403003	Ophthalmo-acromelic syndrome (disorder)
Congenital Disease	SNOMED	703406006	Trichohepatoenteric syndrome (disorder)
Congenital Disease	SNOMED	703504006	Congenital reticular ichthyosiform erythroderma (disorder)
Congenital Disease	SNOMED	703508009	Ear, patella, short stature syndrome (disorder)
Congenital Disease	SNOMED	703523004	Spondyloenchondrodysplasia with immune dysregulation (disorder)
Congenital Disease	SNOMED	703525006	Anhidrotic ectodermal dysplasia with immune deficiency (disorder)
Congenital Disease	SNOMED	703528008	Cutis gyrata syndrome of Beare and Stevenson (disorder)
Congenital Disease	SNOMED	703533007	Capillary malformation-arteriovenous malformation syndrome (disorder)
Congenital Disease	SNOMED	703534001	Patent ductus arteriosus with facial dysmorphism and abnormal fifth digits (disorder)
Congenital Disease	SNOMED	703535000	Mowat-Wilson syndrome (disorder)
Congenital Disease	SNOMED	703539006	Manitoba oculotrichoanal syndrome (disorder)
Congenital Disease	SNOMED	703541007	Neurofibromatosis type 1-like syndrome (disorder)
Congenital Disease	SNOMED	703542000	Retinal detachment and occipital encephalocele (disorder)
Congenital Disease	SNOMED	7037003	Xeroderma, talipes and enamel defect syndrome (disorder)
Congenital Disease	SNOMED	703973009	First and second branchial arch syndrome (disorder)
Congenital Disease	SNOMED	70410008	Acrocephalosyndactyly type V (disorder)
Congenital Disease	SNOMED	70450006	Ectopic gastric tissue (disorder)
Congenital Disease	SNOMED	70452003	Anomaly of chromosome pair 22 (disorder)
Congenital Disease	SNOMED	705069006	Long axis of fetal heart deviated to left (disorder)
Congenital Disease	SNOMED	705070007	Long axis of fetal heart deviated to right (disorder)
Congenital Disease	SNOMED	70534000	Occult spinal dysraphism sequence (disorder)
Congenital Disease	SNOMED	70550008	Streak ovary (disorder)
Congenital Disease	SNOMED	70583001	Incomplete development of membranous labyrinth (disorder)
Congenital Disease	SNOMED	70602002	Pseudocoarctation of aorta (disorder)
Congenital Disease	SNOMED	70642007	Pygopagus (disorder)
Congenital Disease	SNOMED	706885006	Palmoplantar keratoderma (disorder)
Congenital Disease	SNOMED	70705008	Accessory salivary gland (disorder)
Congenital Disease	SNOMED	707147002	Asplenia (disorder)
Congenital Disease	SNOMED	707200002	Descending aorta anterior and same side as azygos vein with absent inferior vena cava (disorder)
Congenital Disease	SNOMED	707209001	Keratoderma (disorder)
Congenital Disease	SNOMED	707234001	Fourth branchial cleft cyst (disorder)
Congenital Disease	SNOMED	707254000	Amniotic adhesion (disorder)
Congenital Disease	SNOMED	707272006	Autosomal recessive dyskeratosis congenita (disorder)
Congenital Disease	SNOMED	707273001	Autosomal dominant dyskeratosis congenita (disorder)
Congenital Disease	SNOMED	707276009	Hoyeraal-Hreidarsson syndrome (disorder)
Congenital Disease	SNOMED	707371002	Congenital stenosis of left pulmonary artery (disorder)
Congenital Disease	SNOMED	707372009	Congenital stenosis of right pulmonary artery (disorder)
Congenital Disease	SNOMED	707433009	Lymphangioleiomyomatosis due to tuberous sclerosis syndrome (disorder)
Congenital Disease	SNOMED	707530009	Cystic hamartoma of lung and kidney (disorder)
Congenital Disease	SNOMED	70756004	Bronchial atresia with segmental pulmonary emphysema (disorder)
Congenital Disease	SNOMED	707770004	Congenital duplication of renal collecting system (disorder)
Congenital Disease	SNOMED	70794004	Congenital pseudoarthrosis of clavicle (disorder)
Congenital Disease	SNOMED	707994006	Absence of uvula (disorder)
Congenital Disease	SNOMED	708022000	Congenital pulmonary lymphatic dysplasia syndrome (disorder)
Congenital Disease	SNOMED	708028001	Congenital pulmonary alveolar capillary dysplasia (disorder)
Congenital Disease	SNOMED	708029009	Congenital pulmonary acinar dysplasia (disorder)
Congenital Disease	SNOMED	708059004	Atresia of penile urethra (disorder)
Congenital Disease	SNOMED	708536001	X-linked dyskeratosis congenita (disorder)
Congenital Disease	SNOMED	708541009	Complete ablepharon (disorder)
Congenital Disease	SNOMED	708669006	Bifid mandibular condyle (disorder)
Congenital Disease	SNOMED	709003000	Salivary gland heterotopia (disorder)
Congenital Disease	SNOMED	70902004	Longitudinal deficiency of upper limb (disorder)
Congenital Disease	SNOMED	709105005	Jackson-Weiss syndrome (disorder)
Congenital Disease	SNOMED	709416009	Acral peeling skin syndrome (disorder)

Congenital Disease	SNOMED	709418005	Autosomal translocation (disorder)
Congenital Disease	SNOMED	70943004	Allemann's syndrome (disorder)
Congenital Disease	SNOMED	709472003	Periodontitis due to Papillon-Lefèvre syndrome (disorder)
Congenital Disease	SNOMED	709490002	Desmosterolosis (disorder)
Congenital Disease	SNOMED	70974002	Congenital absence of rib (disorder)
Congenital Disease	SNOMED	70978004	Situs inversus thoracis (disorder)
Congenital Disease	SNOMED	710008008	Monosomy X (disorder)
Congenital Disease	SNOMED	710010005	Mosaic Turner syndrome (disorder)
Congenital Disease	SNOMED	710019006	Mosaicism 45, X or other cell line with abnormal sex chromosome (disorder)
Congenital Disease	SNOMED	71009001	Multiseptate gallbladder (disorder)
Congenital Disease	SNOMED	71016000	Dicheirus (disorder)
Congenital Disease	SNOMED	7102003	Cyst of omentum (disorder)
Congenital Disease	SNOMED	710232008	Congenital saddle nose (disorder)
Congenital Disease	SNOMED	710571007	Renal dysplasia due to fetal exposure to angiotensin converting enzyme inhibitor (disorder)
Congenital Disease	SNOMED	71084008	Congenital absence of upper arm AND forearm (disorder)
Congenital Disease	SNOMED	711039004	Acrobrachycephaly (disorder)
Congenital Disease	SNOMED	711100005	Caliber persistent labial artery (disorder)
Congenital Disease	SNOMED	71111008	Glaucoma of childhood (disorder)
Congenital Disease	SNOMED	711153001	Bowen-Conradi syndrome (disorder)
Congenital Disease	SNOMED	711154007	Guttate hypopigmentation and punctate palmoplantar keratoderma with or without ectopic calcification (disorder)
Congenital Disease	SNOMED	711156009	Short stature, facial and skeletal anomalies, intellectual disability, macrodontia syndrome (disorder)
Congenital Disease	SNOMED	711157000	Sudden infant death with dysgenesis of testes syndrome (disorder)
Congenital Disease	SNOMED	711543008	Mandibulofacial dysostosis with microcephaly (disorder)
Congenital Disease	SNOMED	711546000	Concordant ventriculoarterial connection with parallel great arteries (disorder)
Congenital Disease	SNOMED	71311003	Congenital kyphosis (disorder)
Congenital Disease	SNOMED	71322004	Ehlers-Danlos syndrome, familial joint laxity type (disorder)
Congenital Disease	SNOMED	7134007	Osteogenesis imperfecta, dominant perinatal lethal (disorder)
Congenital Disease	SNOMED	713406001	Balanced translocation of chromosome (disorder)
Congenital Disease	SNOMED	713509006	Congenital penile torsion (disorder)
Congenital Disease	SNOMED	714180007	Abnormal ostium of coronary artery (disorder)
Congenital Disease	SNOMED	714181006	Abnormal ostium of coronary sinus (disorder)
Congenital Disease	SNOMED	71464000	Dipygus (disorder)
Congenital Disease	SNOMED	715201005	Congenital short bowel syndrome (disorder)
Congenital Disease	SNOMED	715215007	Chromosome 11p13 deletion syndrome (disorder)
Congenital Disease	SNOMED	715216008	Distal arthrogryposis type 2B (disorder)
Congenital Disease	SNOMED	715217004	Distal arthrogryposis type 5 (disorder)
Congenital Disease	SNOMED	715240000	X-linked retinal dysplasia (disorder)
Congenital Disease	SNOMED	715242008	Ulna metaphyseal dysplasia syndrome (disorder)
Congenital Disease	SNOMED	715314008	Distal arthrogryposis type 1 (disorder)
Congenital Disease	SNOMED	715316005	Neurogenic arthrogryposis multiplex congenita (disorder)
Congenital Disease	SNOMED	715318006	Ehlers-Danlos syndrome classic type (disorder)
Congenital Disease	SNOMED	715342005	Alpha thalassemia X-linked intellectual disability syndrome (disorder)
Congenital Disease	SNOMED	715344006	Neurofibromatosis Noonan syndrome (disorder)
Congenital Disease	SNOMED	715371006	Cerebellar ataxia co-occurrent with ectodermal dysplasia (disorder)
Congenital Disease	SNOMED	715391004	Blepharophimosis epicanthus inversus ptosis syndrome (disorder)
Congenital Disease	SNOMED	715397000	Mesenchymal hamartoma of liver (disorder)
Congenital Disease	SNOMED	715404000	Ameloonychohypohidrotic syndrome (disorder)
Congenital Disease	SNOMED	715406003	Isolated lissencephaly type 1 without known genetic defect (disorder)
Congenital Disease	SNOMED	715409005	Trigonocephaly C syndrome (disorder)
Congenital Disease	SNOMED	715418007	Lethal congenital contracture syndrome type 1 (disorder)
Congenital Disease	SNOMED	715419004	Lethal congenital contracture syndrome type 2 (disorder)
Congenital Disease	SNOMED	715420005	Lethal congenital contracture syndrome type 3 (disorder)
Congenital Disease	SNOMED	715421009	Craniofrontonasal dysplasia (disorder)
Congenital Disease	SNOMED	715422002	Craniotelencephalic dysplasia (disorder)
Congenital Disease	SNOMED	715426004	X-linked corneal dermoid (disorder)
Congenital Disease	SNOMED	715427008	Acromelic frontonasal dysplasia (disorder)
Congenital Disease	SNOMED	715428003	Skeletal dysplasia with epilepsy and short stature syndrome (disorder)
Congenital Disease	SNOMED	715430001	Embryofetopathy caused by indomethacin (disorder)
Congenital Disease	SNOMED	715431002	Embryopathy caused by phenobarbital (disorder)
Congenital Disease	SNOMED	715434005	Holoprosencephaly craniosynostosis syndrome (disorder)
Congenital Disease	SNOMED	715436007	Congenital cerebellar hypoplasia co-occurrent with tapetoretinal degeneration (disorder)
Congenital Disease	SNOMED	715438008	Distal partial deletion of long arm of chromosome 11 (disorder)
Congenital Disease	SNOMED	715440003	Mirror hands and feet co-occurrent with nasal defect (disorder)
Congenital Disease	SNOMED	715441004	McDonough syndrome (disorder)
Congenital Disease	SNOMED	715442006	Syndactyly of fingers type 8 (disorder)
Congenital Disease	SNOMED	715462003	Microcephalus co-occurrent with cervical spine fusion anomaly (disorder)
Congenital Disease	SNOMED	715463008	Congenital pontocerebellar hypoplasia type 2 (disorder)
Congenital Disease	SNOMED	715464002	Microcephalus microcornea syndrome of Seemanova type (disorder)
Congenital Disease	SNOMED	715470008	Brachydactylous dwarfism Mseleni type (disorder)

Congenital Disease	SNOMED	715471007	Mesomelic dysplasia with cleft palate and camptodactyly syndrome (disorder)
Congenital Disease	SNOMED	715472000	Mesomelic dysplasia of hypoplastic ulna and fibula type (disorder)
Congenital Disease	SNOMED	715474004	Aplasia of fibula co-occurrent with complex brachydactyly (disorder)
Congenital Disease	SNOMED	715482004	Microcephalic primordial dwarfism of Toriello type (disorder)
Congenital Disease	SNOMED	715484003	Ophthalmomandibulomelic dysplasia (disorder)
Congenital Disease	SNOMED	715487005	Autosomal recessive distal osteolysis syndrome (disorder)
Congenital Disease	SNOMED	715505002	Rhizomelic dysplasia of Patterson Lowry type (disorder)
Congenital Disease	SNOMED	715506001	Phocomelia, ectrodactyly, deafness and sinus arrhythmia syndrome (disorder)
Congenital Disease	SNOMED	715522000	Phocomelia Schinzel type (disorder)
Congenital Disease	SNOMED	715523005	Mirror polydactyly, vertebral segmentation and limb defect syndrome (disorder)
Congenital Disease	SNOMED	715524004	Delayed membranous cranial ossification (disorder)
Congenital Disease	SNOMED	715530004	Tetrasomy of short arm of chromosome 9 (disorder)
Congenital Disease	SNOMED	715531000	Tibial aplasia and ectrodactyly syndrome (disorder)
Congenital Disease	SNOMED	715532007	Weismann Netter syndrome (disorder)
Congenital Disease	SNOMED	715533002	Microcephaly, microphthalmia, ectrodactyly of lower limbs and prognathism syndrome (disorder)
Congenital Disease	SNOMED	715535009	Keratosis palmoplantaris and arrhythmogenic cardiomyopathy syndrome (disorder)
Congenital Disease	SNOMED	715565004	Lethal arthrogryposis co-occurrent with anterior horn cell disease (disorder)
Congenital Disease	SNOMED	715568002	Gnathodiaphyseal dysplasia syndrome (disorder)
Congenital Disease	SNOMED	715575001	Distal arthrogryposis type 4 (disorder)
Congenital Disease	SNOMED	715576000	Ectodermal dysplasia with natal teeth Turnpenny type (disorder)
Congenital Disease	SNOMED	715628009	Intellectual disability, truncal obesity, retinal dystrophy and micropenis syndrome (disorder)
Congenital Disease	SNOMED	715631005	Chondrodysplasia punctata Toriello type (disorder)
Congenital Disease	SNOMED	715644000	Glomuvenous malformation (disorder)
Congenital Disease	SNOMED	715652002	Mesomelic dysplasia Savarirayan type (disorder)
Congenital Disease	SNOMED	715653007	Spondyloocular syndrome (disorder)
Congenital Disease	SNOMED	715654001	Ischio-vertebral syndrome (disorder)
Congenital Disease	SNOMED	715656004	Congenital aplasia of lacrimal gland co-occurrent with congenital aplasia of salivary gland (disorder)
Congenital Disease	SNOMED	715669000	Congenital epithelial dysplasia of intestine (disorder)
Congenital Disease	SNOMED	715670004	Mild spondyloepiphyseal dysplasia with early onset osteoarthritis due to collagen type II alpha 1 mutation (disorder)
Congenital Disease	SNOMED	715672007	Multiple epiphyseal dysplasia type 4 (disorder)
Congenital Disease	SNOMED	715673002	Multiple epiphyseal dysplasia type 1 (disorder)
Congenital Disease	SNOMED	715674008	Multiple epiphyseal dysplasia type 5 (disorder)
Congenital Disease	SNOMED	715704001	Postaxial polydactyly type A (disorder)
Congenital Disease	SNOMED	715707008	Postaxial polydactyly type B (disorder)
Congenital Disease	SNOMED	715710001	Polydactyly of triphalangeal thumb (disorder)
Congenital Disease	SNOMED	715720006	Brachydactyly type A1 (disorder)
Congenital Disease	SNOMED	715721005	Brachydactyly type A4 (disorder)
Congenital Disease	SNOMED	715722003	Brachydactyly type A6 (disorder)
Congenital Disease	SNOMED	715723008	Syndactyly type 1 (disorder)
Congenital Disease	SNOMED	715724002	Syndactyly type 2 (disorder)
Congenital Disease	SNOMED	715725001	Syndactyly type 3 (disorder)
Congenital Disease	SNOMED	715735007	Maternal uniparental disomy of chromosome 20 (disorder)
Congenital Disease	SNOMED	715736008	Paternal uniparental disomy of chromosome 20 (disorder)
Congenital Disease	SNOMED	715769008	Congenital abnormal retraction of eyelid (disorder)
Congenital Disease	SNOMED	715771008	Microphthalmos co-occurrent with congenital ocular coloboma (disorder)
Congenital Disease	SNOMED	715780008	Lissencephaly type 1 due to doublecortin gene mutation (disorder)
Congenital Disease	SNOMED	715817007	Lissencephaly co-occurrent with congenital cerebellar hypoplasia (disorder)
Congenital Disease	SNOMED	715819005	Lissencephaly co-occurrent with congenital cerebellar hypoplasia type B (disorder)
Congenital Disease	SNOMED	715820004	Lissencephaly co-occurrent with congenital cerebellar hypoplasia type C (disorder)
Congenital Disease	SNOMED	715821000	Lissencephaly co-occurrent with congenital cerebellar hypoplasia type D (disorder)
Congenital Disease	SNOMED	715822007	Lissencephaly co-occurrent with congenital cerebellar hypoplasia type F (disorder)
Congenital Disease	SNOMED	715828006	Proboscis lateralis (disorder)
Congenital Disease	SNOMED	715858000	Mucogingival deformity on edentulous ridge due to aberrant insertion of frenum of tongue (disorder)
Congenital Disease	SNOMED	715861004	Dysplasia of head of femur Meyer type (disorder)
Congenital Disease	SNOMED	715862006	Smith McCort dysplasia (disorder)
Congenital Disease	SNOMED	715867000	Pseudoaminopterin syndrome (disorder)
Congenital Disease	SNOMED	715905006	Unilateral polymicrogyria (disorder)
Congenital Disease	SNOMED	715951007	Acrocallosal syndrome (disorder)
Congenital Disease	SNOMED	715952000	Waardenburg Shah syndrome (disorder)
Congenital Disease	SNOMED	715981004	Autosomal recessive primary microcephaly (disorder)
Congenital Disease	SNOMED	715983001	Ring chromosome 8 syndrome (disorder)
Congenital Disease	SNOMED	715985008	Maxillonasal dysplasia syndrome (disorder)

Congenital Disease	SNOMED	715986009	Camptodactyly with joint contracture and facial skeletal defect syndrome (disorder)
Congenital Disease	SNOMED	715987000	Congenital heart defect with round face and developmental delay syndrome (disorder)
Congenital Disease	SNOMED	715988005	Cataract with aberrant oral frenula and growth delay syndrome (disorder)
Congenital Disease	SNOMED	715989002	Congenital cataract with intellectual disability and anal atresia and urinary defect syndrome (disorder)
Congenital Disease	SNOMED	715990006	Agenesis of cerebellum and hydrocephalus syndrome (disorder)
Congenital Disease	SNOMED	715991005	Crane Heise syndrome (disorder)
Congenital Disease	SNOMED	716005004	Fetal diethylstilbestrol syndrome (disorder)
Congenital Disease	SNOMED	716006003	Bifid femur co-occurrent with monodactylous ectrodactyly (disorder)
Congenital Disease	SNOMED	716007007	Cleft palate and cleft lip with deafness and sacral lipoma syndrome (disorder)
Congenital Disease	SNOMED	716022002	Frontofacionasal dysplasia syndrome (disorder)
Congenital Disease	SNOMED	716023007	Prominent glabella with microcephaly and hypogenitalism syndrome (disorder)
Congenital Disease	SNOMED	716024001	Goniodysgenesis with intellectual disability and short stature syndrome (disorder)
Congenital Disease	SNOMED	716088000	Follicular hamartoma with alopecia and cystic fibrosis syndrome (disorder)
Congenital Disease	SNOMED	716089008	Craniofacial digital and genital anomalies syndrome (disorder)
Congenital Disease	SNOMED	716090004	Short stature with craniofacial anomalies and genital hypoplasia syndrome (disorder)
Congenital Disease	SNOMED	716091000	Holoprosencephaly and postaxial polydactyly syndrome (disorder)
Congenital Disease	SNOMED	716092007	Radial hypoplasia and triphalangeal thumb with hypospadias and maxillary diastema syndrome (disorder)
Congenital Disease	SNOMED	716094008	Fibulo-ulnar hypoplasia and renal anomalies syndrome (disorder)
Congenital Disease	SNOMED	716096005	Hypospadias and intellectual disability syndrome Goldblatt type (disorder)
Congenital Disease	SNOMED	716097001	Ichthyosis cheek eyebrow syndrome (disorder)
Congenital Disease	SNOMED	716099003	Absence deformity of leg and congenital cataract syndrome (disorder)
Congenital Disease	SNOMED	716105001	Autosomal dominant diffuse palmoplantar keratoderma Norrbotten type (disorder)
Congenital Disease	SNOMED	716106000	Limb body wall complex (disorder)
Congenital Disease	SNOMED	716108004	Macrocephaly with spastic paraplegia and dysmorphism syndrome (disorder)
Congenital Disease	SNOMED	716110002	Upper limb defect with eye and ear abnormalities syndrome (disorder)
Congenital Disease	SNOMED	716111003	Mullerian duct and limb anomalies syndrome (disorder)
Congenital Disease	SNOMED	716112005	Microcephaly with deafness and intellectual disability syndrome (disorder)
Congenital Disease	SNOMED	716165003	Microcornea with corectopia and macular hypoplasia syndrome (disorder)
Congenital Disease	SNOMED	716166002	Microcornea with glaucoma and absent frontal sinus syndrome (disorder)
Congenital Disease	SNOMED	716169009	Holoprosencephaly sequence with hypokinesia and congenital joint contracture syndrome (disorder)
Congenital Disease	SNOMED	716170005	Deafness with cataract and skeletal anomaly syndrome (disorder)
Congenital Disease	SNOMED	716174001	Oculocerebral hypopigmentation syndrome of Preus type (disorder)
Congenital Disease	SNOMED	716180009	Odontoma dysphagia syndrome (disorder)
Congenital Disease	SNOMED	716189005	Osteoporosis and macrocephaly with blindness and joint hypermobility syndrome (disorder)
Congenital Disease	SNOMED	71619006	Congenital absence of ear (disorder)
Congenital Disease	SNOMED	716191002	Alopecia and intellectual disability syndrome (disorder)
Congenital Disease	SNOMED	716192009	Short stature and deafness with neutrophil dysfunction and facial dysmorphism syndrome (disorder)
Congenital Disease	SNOMED	716193004	Short stature with valvular heart disease and characteristic facies syndrome (disorder)
Congenital Disease	SNOMED	716194005	Pili torti with developmental delay and neurological abnormality syndrome (disorder)
Congenital Disease	SNOMED	716195006	Autosomal recessive brachyolmia and amelogenesis imperfecta syndrome (disorder)
Congenital Disease	SNOMED	716196007	Isolated polycystic liver disease (disorder)
Congenital Disease	SNOMED	716198008	Growth delay with hydrocephalus and lung hypoplasia syndrome (disorder)
Congenital Disease	SNOMED	716199000	Delayed speech and facial asymmetry with strabismus and ear lobe skin crease syndrome (disorder)
Congenital Disease	SNOMED	716230005	Shprintzen Goldberg omphalocele syndrome (disorder)
Congenital Disease	SNOMED	716231009	Spondylocamptodactyly syndrome (disorder)
Congenital Disease	SNOMED	716232002	Autosomal dominant spondylocostal dysostosis (disorder)
Congenital Disease	SNOMED	716233007	Steinfeld syndrome (disorder)
Congenital Disease	SNOMED	716238003	Deafness with epiphyseal dysplasia and short stature syndrome (disorder)
Congenital Disease	SNOMED	716243005	Deafness with malformation of ear and facial palsy syndrome (disorder)
Congenital Disease	SNOMED	716245003	Deafness craniofacial syndrome (disorder)
Congenital Disease	SNOMED	716248001	Cleft lip and cleft palate with ectodermal dysplasia syndrome (disorder)
Congenital Disease	SNOMED	716249009	Tetraamelia with multiple malformation syndrome (disorder)
Congenital Disease	SNOMED	716279002	Congenital duplication of nose (disorder)
Congenital Disease	SNOMED	716280004	Congenital tubular duplication of esophagus (disorder)
Congenital Disease	SNOMED	716334004	Intellectual disability and short stature with hand contracture and genital anomaly syndrome (disorder)
Congenital Disease	SNOMED	716337006	Seaver Cassidy syndrome (disorder)
Congenital Disease	SNOMED	716381003	8p23.1 microdeletion syndrome (disorder)
Congenital Disease	SNOMED	716387004	2q31.1 microdeletion syndrome (disorder)

Congenital Disease	SNOMED	716456000	3q29 microdeletion syndrome (disorder)
Congenital Disease	SNOMED	716515000	1q41q42 microdeletion syndrome (disorder)
Congenital Disease	SNOMED	716636008	Longitudinal deficiency of part of upper limb (disorder)
Congenital Disease	SNOMED	716638009	Longitudinal deficiency of part of limb (disorder)
Congenital Disease	SNOMED	716683005	17q21.31 microduplication syndrome (disorder)
Congenital Disease	SNOMED	716699004	Epidermolysis bullosa simplex due to plakophilin deficiency (disorder)
Congenital Disease	SNOMED	716700003	Epidermolysis bullosa simplex with circinate migratory erythema (disorder)
Congenital Disease	SNOMED	716701004	Epidermolysis bullosa simplex co-occurrent with pyloric atresia (disorder)
Congenital Disease	SNOMED	716708005	FRAXF syndrome (disorder)
Congenital Disease	SNOMED	716709002	FRAXE intellectual disability syndrome (disorder)
Congenital Disease	SNOMED	716740009	Potter sequence cleft lip and palate cardiopathy syndrome (disorder)
Congenital Disease	SNOMED	716741008	Bilateral hypoplasia of tibia and postaxial polydactyly syndrome (disorder)
Congenital Disease	SNOMED	716744000	Familial hypospadias of penis (disorder)
Congenital Disease	SNOMED	716768008	Dysplasia of trochlea of femur (disorder)
Congenital Disease	SNOMED	716773002	Familial idiopathic dilatation of right atrium (disorder)
Congenital Disease	SNOMED	716775009	Nanophthalmia (disorder)
Congenital Disease	SNOMED	716862002	Proteus like syndrome (disorder)
Congenital Disease	SNOMED	716868003	Multicentric osteolysis nodulosis arthropathy spectrum (disorder)
Congenital Disease	SNOMED	7169009	Congenital supravalvular aortic stenosis (disorder)
Congenital Disease	SNOMED	716996008	Hypoplasia of corpus callosum, intellectual disability, adducted thumbs, spasticity, hydrocephalus syndrome (disorder)
Congenital Disease	SNOMED	716997004	Joubert syndrome (disorder)
Congenital Disease	SNOMED	716998009	Joubert syndrome with ocular defect (disorder)
Congenital Disease	SNOMED	716999001	Joubert syndrome with renal defect (disorder)
Congenital Disease	SNOMED	717003001	Hereditary cavernous hemangioma of brain (disorder)
Congenital Disease	SNOMED	71703005	Complete trisomy 22 syndrome (disorder)
Congenital Disease	SNOMED	717041008	Syndromic recessive X-linked ichthyosis (disorder)
Congenital Disease	SNOMED	717049005	Trisomy 17p (disorder)
Congenital Disease	SNOMED	717156002	Biliary atresia with splenic malformation syndrome (disorder)
Congenital Disease	SNOMED	717157006	Trisomy 10p (disorder)
Congenital Disease	SNOMED	717158001	Partial chromosome Y deletion (disorder)
Congenital Disease	SNOMED	717183001	Keratoderma hereditarium mutilans with ichthyosis syndrome (disorder)
Congenital Disease	SNOMED	717184007	Punctate palmoplantar keratoderma type 1 (disorder)
Congenital Disease	SNOMED	717187000	Nephronophthisis hepatic fibrosis syndrome (disorder)
Congenital Disease	SNOMED	717192003	Familial digital arthropathy and brachydactyly syndrome (disorder)
Congenital Disease	SNOMED	717221005	Metaphyseal dysplasia Braun Tinschert type (disorder)
Congenital Disease	SNOMED	717222003	Microphthalmia with ankyloblepharon and intellectual disability syndrome (disorder)
Congenital Disease	SNOMED	717228004	Hereditary palmoplantar keratoderma Gamborg Nielsen type (disorder)
Congenital Disease	SNOMED	717232005	Caroli disease (disorder)
Congenital Disease	SNOMED	717262004	Isolated congenital alacrima (disorder)
Congenital Disease	SNOMED	717264003	Autosomal dominant brachyolmia (disorder)
Congenital Disease	SNOMED	71728002	Accessory liver (disorder)
Congenital Disease	SNOMED	717330004	Spondyloepimetaphyseal dysplasia Irapa type (disorder)
Congenital Disease	SNOMED	717331000	Familial thyroglossal duct cyst (disorder)
Congenital Disease	SNOMED	717335009	Mosaic trisomy 8 syndrome (disorder)
Congenital Disease	SNOMED	717337001	Syndromic hypoplasia of orbital border (disorder)
Congenital Disease	SNOMED	717338006	Koolen De Vries syndrome (disorder)
Congenital Disease	SNOMED	717459000	Congenital primary megaureter (disorder)
Congenital Disease	SNOMED	717632002	X-linked lissencephaly with agenesis of corpus callosum and genital anomaly syndrome (disorder)
Congenital Disease	SNOMED	717633007	Distal monosomy 1q syndrome (disorder)
Congenital Disease	SNOMED	717701008	Uterus bicornis bicollis with blind hemi-vagina (disorder)
Congenital Disease	SNOMED	717702001	Uterus bicornis bicollis with patent cervix and vagina (disorder)
Congenital Disease	SNOMED	717703006	Bilateral congenital absence of ovary (disorder)
Congenital Disease	SNOMED	717704000	Bilateral congenital absence of fallopian tube (disorder)
Congenital Disease	SNOMED	717705004	Mayer-Rokitansky-Küster-Hauser syndrome type 2 (disorder)
Congenital Disease	SNOMED	717742006	Primary renal dysplasia (disorder)
Congenital Disease	SNOMED	717744007	Secondary renal dysplasia (disorder)
Congenital Disease	SNOMED	717745008	Bilateral secondary renal dysplasia (disorder)
Congenital Disease	SNOMED	717746009	Congenital hemorrhagic renal cyst (disorder)
Congenital Disease	SNOMED	717748005	Bilateral medullary sponge kidney (disorder)
Congenital Disease	SNOMED	717749002	Bilateral multicystic renal dysplasia (disorder)
Congenital Disease	SNOMED	717750002	Bilateral congenital primary hydronephrosis (disorder)
Congenital Disease	SNOMED	717752005	Fetal lower urinary tract obstruction (disorder)
Congenital Disease	SNOMED	717753000	Fetal lower urinary tract obstruction due to atresia of urethra (disorder)
Congenital Disease	SNOMED	717754006	Fetal lower urinary tract obstruction due to posterior urethral valve (disorder)
Congenital Disease	SNOMED	717755007	Fetal lower urinary tract obstruction due to anterior urethral valve (disorder)
Congenital Disease	SNOMED	717756008	Complete duplication of urethra (disorder)
Congenital Disease	SNOMED	717757004	Partial duplication of urethra (disorder)
Congenital Disease	SNOMED	717758009	Congenital atresia and stenosis of ureter (disorder)
Congenital Disease	SNOMED	717759001	Congenital absence of bladder and urethra (disorder)
Congenital Disease	SNOMED	717765001	Capillary malformation of lower lip, lymphatic malformation of face and neck, asymmetry of face and limbs and overgrowth syndrome (disorder)

Congenital Disease	SNOMED	717766000	Alport syndrome autosomal dominant (disorder)
Congenital Disease	SNOMED	717767009	Alport syndrome autosomal recessive (disorder)
Congenital Disease	SNOMED	717768004	Alport syndrome X-linked (disorder)
Congenital Disease	SNOMED	717771007	Cloverleaf skull with multiple congenital anomalies syndrome (disorder)
Congenital Disease	SNOMED	717772000	Cerebro-oculo-dento-auriculo-skeletal syndrome (disorder)
Congenital Disease	SNOMED	717785002	Coloboma of macula with brachydactyly type B syndrome (disorder)
Congenital Disease	SNOMED	71779008	X-linked hydrocephalus syndrome (disorder)
Congenital Disease	SNOMED	717812000	Congenital cataract, hypertrophic cardiomyopathy, mitochondrial myopathy syndrome (disorder)
Congenital Disease	SNOMED	717813005	Global developmental delay, osteopenia, ectodermal defect syndrome (disorder)
Congenital Disease	SNOMED	717814004	Glossopalatine ankylosis (disorder)
Congenital Disease	SNOMED	717822006	Goldberg Shprintzen megacolon syndrome (disorder)
Congenital Disease	SNOMED	717823001	Chondrodysplasia, dentinogenesis imperfecta, joint laxity syndrome (disorder)
Congenital Disease	SNOMED	717824007	Progressive arterial occlusive disease, hypertension, heart defect, bone fragility, brachysyndactyly syndrome (disorder)
Congenital Disease	SNOMED	717835002	Atresia of the esophagus without tracheoesophageal fistula (disorder)
Congenital Disease	SNOMED	717850006	Brachymelia of lower limb (disorder)
Congenital Disease	SNOMED	717859007	Hydrocephalus, cardiac malformation, dense bone syndrome (disorder)
Congenital Disease	SNOMED	717866008	Arteriovenous malformation of stomach (disorder)
Congenital Disease	SNOMED	717880001	Arteriovenous malformation of duodenum (disorder)
Congenital Disease	SNOMED	717884005	Congenital diverticulum of duodenum (disorder)
Congenital Disease	SNOMED	717886007	Congenital malrotation of duodenum (disorder)
Congenital Disease	SNOMED	717887003	Biemond syndrome type 2 (disorder)
Congenital Disease	SNOMED	717897007	Hereditary isolated hypoparathyroidism due to agenesis of parathyroid gland (disorder)
Congenital Disease	SNOMED	717909004	Bilateral microtia with deafness and cleft palate syndrome (disorder)
Congenital Disease	SNOMED	717911008	Blepharocheilodontic syndrome (disorder)
Congenital Disease	SNOMED	717913006	Blepharonasofacial malformation syndrome (disorder)
Congenital Disease	SNOMED	717914000	Blepharophimosis, ptosis, esotropia, syndactyly, short stature syndrome (disorder)
Congenital Disease	SNOMED	717915004	Blepharoptosis, myopia, ectopia lentis syndrome (disorder)
Congenital Disease	SNOMED	717920004	Blindness, scoliosis, arachnodactyly syndrome (disorder)
Congenital Disease	SNOMED	717939009	Anencephaly without rachischisis (disorder)
Congenital Disease	SNOMED	717940006	Bifid nose, anorectal anomaly, renal anomaly syndrome (disorder)
Congenital Disease	SNOMED	717943008	Brain malformation, congenital heart disease, postaxial polydactyly syndrome (disorder)
Congenital Disease	SNOMED	717944002	Branchiogenic deafness syndrome (disorder)
Congenital Disease	SNOMED	717945001	Brain anomaly, severe intellectual disability, ectodermal dysplasia, skeletal deformity, ear anomaly, kidney dysplasia syndrome (disorder)
Congenital Disease	SNOMED	717963001	Isolated anterior cervical hypertrichosis (disorder)
Congenital Disease	SNOMED	717973004	Chromosome 3q29 duplication syndrome (disorder)
Congenital Disease	SNOMED	717977003	Lissencephaly syndrome Norman Roberts type (disorder)
Congenital Disease	SNOMED	718095000	Schisis association syndrome (disorder)
Congenital Disease	SNOMED	718099006	Enlarged parietal foramina (disorder)
Congenital Disease	SNOMED	718128009	Congenital dysplasia of cardiac valve (disorder)
Congenital Disease	SNOMED	718135001	Isolated right ventricular hypoplasia (disorder)
Congenital Disease	SNOMED	718181001	Congenital cardiac diverticulum (disorder)
Congenital Disease	SNOMED	718188007	8p inverted duplication deletion syndrome (disorder)
Congenital Disease	SNOMED	718189004	Recombinant chromosome 8 syndrome (disorder)
Congenital Disease	SNOMED	718194004	Hypothyroidism due to mutation in transcription factor of pituitary development (disorder)
Congenital Disease	SNOMED	718211004	Ehlers-Danlos syndrome kyphoscoliotic type (disorder)
Congenital Disease	SNOMED	718216009	Partial defect of atrioventricular canal (disorder)
Congenital Disease	SNOMED	718218005	Porokeratosis plantaris palmaris et disseminata (disorder)
Congenital Disease	SNOMED	718222000	Autosomal dominant popliteal pterygium syndrome (disorder)
Congenital Disease	SNOMED	718224004	Progressive hemifacial atrophy (disorder)
Congenital Disease	SNOMED	718226002	Wolf Hirschhorn syndrome (disorder)
Congenital Disease	SNOMED	718227006	Proximal 16p11.2 microdeletion syndrome (disorder)
Congenital Disease	SNOMED	718228001	Fetal iodine syndrome (disorder)
Congenital Disease	SNOMED	7183006	Anophthalmos (disorder)
Congenital Disease	SNOMED	718395009	Defects of tubular bones and spine (disorder)
Congenital Disease	SNOMED	718485003	Congenital dilatation of ureter (disorder)
Congenital Disease	SNOMED	718552009	Familial median cleft of upper and lower lip (disorder)
Congenital Disease	SNOMED	718556007	Cranio-cerebello-cardiac dysplasia syndrome (disorder)
Congenital Disease	SNOMED	718559000	Acromesomelic dysplasia Maroteaux type (disorder)
Congenital Disease	SNOMED	718573009	Achalasia microcephaly syndrome (disorder)
Congenital Disease	SNOMED	718574003	Cleft palate with coloboma of eye and deafness syndrome (disorder)
Congenital Disease	SNOMED	718575002	Ablepharon macrostomia syndrome (disorder)
Congenital Disease	SNOMED	718576001	Hydrocephalus with cleft palate and joint contracture syndrome (disorder)
Congenital Disease	SNOMED	718577005	X-linked intellectual disability Atkin type (disorder)
Congenital Disease	SNOMED	718605009	Congenital pontocerebellar hypoplasia type 7 (disorder)
Congenital Disease	SNOMED	718606005	Congenital pontocerebellar hypoplasia type 6 (disorder)
Congenital Disease	SNOMED	718607001	Congenital pontocerebellar hypoplasia type 5 (disorder)

Congenital Disease	SNOMED	718608006	Congenital pontocerebellar hypoplasia type 4 (disorder)
Congenital Disease	SNOMED	718609003	Congenital pontocerebellar hypoplasia type 3 (disorder)
Congenital Disease	SNOMED	718610008	Congenital pontocerebellar hypoplasia type 1 (disorder)
Congenital Disease	SNOMED	718611007	Congenital pontocerebellar hypoplasia type 8 (disorder)
Congenital Disease	SNOMED	718614004	Telangiectasia, erythrocytosis, monoclonal gammopathy, perinephric fluid collections and intrapulmonary shunting syndrome (disorder)
Congenital Disease	SNOMED	718615003	8q21.11 microdeletion syndrome (disorder)
Congenital Disease	SNOMED	718631006	Annular epidermolytic ichthyosis (disorder)
Congenital Disease	SNOMED	718632004	Self-healing collodion baby (disorder)
Congenital Disease	SNOMED	718633009	Acral self-healing collodion baby (disorder)
Congenital Disease	SNOMED	718679004	Mammary digital nail syndrome (disorder)
Congenital Disease	SNOMED	718680001	Oro-facial digital syndrome type 9 (disorder)
Congenital Disease	SNOMED	718681002	Oro-facial digital syndrome type 11 (disorder)
Congenital Disease	SNOMED	718687003	Distal monosomy 10q syndrome (disorder)
Congenital Disease	SNOMED	718688008	Distal monosomy 6p (disorder)
Congenital Disease	SNOMED	718689000	Distal trisomy 10q (disorder)
Congenital Disease	SNOMED	718691008	Isolated cryptophthalmos (disorder)
Congenital Disease	SNOMED	718715007	Acanthosis nigricans and insulin resistance with muscle cramp and acral enlargement syndrome (disorder)
Congenital Disease	SNOMED	718719001	Lissencephaly type 3 familial fetal akinesia sequence syndrome (disorder)
Congenital Disease	SNOMED	718720007	Lissencephaly type 3 metacarpal bone dysplasia syndrome (disorder)
Congenital Disease	SNOMED	718749004	Generalized peeling skin syndrome (disorder)
Congenital Disease	SNOMED	718759003	Lissencephaly due to tubulin alpha 1A mutation (disorder)
Congenital Disease	SNOMED	718761007	Syndromic microphthalmia due to orthodenticle homeobox 2 mutation (disorder)
Congenital Disease	SNOMED	718763005	Spondyloepiphyseal dysplasia with myopia and sensorineural deafness syndrome (disorder)
Congenital Disease	SNOMED	718764004	Spondyloepiphyseal dysplasia Reardon type (disorder)
Congenital Disease	SNOMED	718765003	Spondyloepiphyseal dysplasia and brachydactyly with speech disorder syndrome (disorder)
Congenital Disease	SNOMED	718766002	Spondyloepiphyseal dysplasia, craniosynostosis, cleft palate, cataract and intellectual disability syndrome (disorder)
Congenital Disease	SNOMED	718848000	Fried syndrome (disorder)
Congenital Disease	SNOMED	718851007	Cataract glaucoma syndrome (disorder)
Congenital Disease	SNOMED	718880003	Zellweger-like syndrome without peroxisomal anomaly (disorder)
Congenital Disease	SNOMED	718881004	Chromosome Xq27.3q28 duplication syndrome (disorder)
Congenital Disease	SNOMED	718896000	X-linked recessive intellectual disability and macrocephaly with ciliary dysfunction syndrome (disorder)
Congenital Disease	SNOMED	718897009	X-linked intellectual disability Seemanova type (disorder)
Congenital Disease	SNOMED	718900002	Syndromic X-linked intellectual disability type 11 (disorder)
Congenital Disease	SNOMED	718905007	X-linked intellectual disability Shrimpton type (disorder)
Congenital Disease	SNOMED	718908009	X-linked intellectual disability Siderius type (disorder)
Congenital Disease	SNOMED	718909001	X-linked intellectual disability Stevenson type (disorder)
Congenital Disease	SNOMED	718910006	X-linked intellectual disability Stocco Dos Santos type (disorder)
Congenital Disease	SNOMED	718911005	X-linked intellectual disability Stoll type (disorder)
Congenital Disease	SNOMED	718912003	X-linked intellectual disability Turner type (disorder)
Congenital Disease	SNOMED	718914002	X-linked intellectual disability Van Esch type (disorder)
Congenital Disease	SNOMED	719009006	X-linked intellectual disability Wilson type (disorder)
Congenital Disease	SNOMED	71901000	Congenital contracted pelvis (disorder)
Congenital Disease	SNOMED	719010001	X-linked intellectual disability Schimke type (disorder)
Congenital Disease	SNOMED	719011002	X-linked intellectual disability Pai type (disorder)
Congenital Disease	SNOMED	719012009	X-linked intellectual disability Miles Carpenter type (disorder)
Congenital Disease	SNOMED	719013004	X-linked intellectual disability Cilliers type (disorder)
Congenital Disease	SNOMED	719016007	X-linked intellectual disability Cantagrel type (disorder)
Congenital Disease	SNOMED	719017003	X-linked intellectual disability Armfield type (disorder)
Congenital Disease	SNOMED	719018008	X-linked intellectual disability Abidi type (disorder)
Congenital Disease	SNOMED	719020006	Pallister W syndrome (disorder)
Congenital Disease	SNOMED	719021005	DK phocomelia syndrome (disorder)
Congenital Disease	SNOMED	719041000	Hip dysplasia with enchondromata and ecchondroma syndrome (disorder)
Congenital Disease	SNOMED	719042007	Uveal coloboma with cleft lip and palate and intellectual disability syndrome (disorder)
Congenital Disease	SNOMED	719043002	Vertebral abnormality, anal atresia, cardiac abnormality, tracheo-esophageal fistula, renal anomaly, limb defect syndrome with hydrocephalus (disorder)
Congenital Disease	SNOMED	719044008	Partial agenesis of pancreas (disorder)
Congenital Disease	SNOMED	719046005	12q14 microdeletion syndrome (disorder)
Congenital Disease	SNOMED	719047001	14q11.2 microdeletion syndrome (disorder)
Congenital Disease	SNOMED	719069008	Shprintzen Goldberg craniosynostosis syndrome (disorder)
Congenital Disease	SNOMED	719096006	Brittle cornea syndrome (disorder)
Congenital Disease	SNOMED	719097002	Branchioskeletogenital syndrome (disorder)
Congenital Disease	SNOMED	719098007	Choreoathetosis with congenital hypothyroidism and neonatal respiratory distress syndrome (disorder)
Congenital Disease	SNOMED	719102004	Congenital cataract with ataxia and deafness syndrome (disorder)
Congenital Disease	SNOMED	719104003	Autosomal recessive palmoplantar keratoderma and congenital alopecia syndrome (disorder)

Congenital Disease	SNOMED	719136005	X-linked intellectual disability with cerebellar hypoplasia syndrome (disorder)
Congenital Disease	SNOMED	719138006	X-linked intellectual disability with cubitus valgus and dysmorphism syndrome (disorder)
Congenital Disease	SNOMED	719139003	X-linked Dandy-Walker malformation with intellectual disability, basal ganglia disease and seizure syndrome (disorder)
Congenital Disease	SNOMED	719140001	X-linked intellectual disability with dysmorphism and cerebral atrophy syndrome (disorder)
Congenital Disease	SNOMED	719155005	X-linked intellectual disability and epilepsy with progressive joint contracture and facial dysmorphism syndrome (disorder)
Congenital Disease	SNOMED	719156006	X-linked intellectual disability with hypogammaglobulinemia and progressive neurological deterioration syndrome (disorder)
Congenital Disease	SNOMED	719157002	X-linked intellectual disability and hypotonia with facial dysmorphism and aggressive behavior syndrome (disorder)
Congenital Disease	SNOMED	719158007	Syndactyly type 4 (disorder)
Congenital Disease	SNOMED	719159004	Syndactyly type 5 (disorder)
Congenital Disease	SNOMED	719160009	Syndromic X-linked intellectual disability type 7 (disorder)
Congenital Disease	SNOMED	719161008	Syndromic X-linked intellectual disability due to jumonji at-rich interactive domain 1c mutation (disorder)
Congenital Disease	SNOMED	719162001	Radioulnar synostosis with microcephaly and scoliosis syndrome (disorder)
Congenital Disease	SNOMED	719163006	Accessory anterior naris (disorder)
Congenital Disease	SNOMED	719165004	Spondyloepimetaphyseal dysplasia aggrecan type (disorder)
Congenital Disease	SNOMED	719166003	Spondyloepimetaphyseal dysplasia matrilin-3 type (disorder)
Congenital Disease	SNOMED	719171005	Spondyloepimetaphyseal dysplasia Missouri type (disorder)
Congenital Disease	SNOMED	719172003	Spondyloepimetaphyseal dysplasia 3-prime-phosphoadenosine 5-prime-phosphosulfate synthase 2 type (disorder)
Congenital Disease	SNOMED	719201004	Spondyloepimetaphyseal dysplasia Shohat type (disorder)
Congenital Disease	SNOMED	719202006	Spondyloepiphyseal dysplasia tarda Kohn type (disorder)
Congenital Disease	SNOMED	719203001	Spondyloepiphyseal dysplasia Kimberley type (disorder)
Congenital Disease	SNOMED	719204007	Spondyloepiphyseal dysplasia Maroteaux type (disorder)
Congenital Disease	SNOMED	719205008	Spondylometaphyseal dysplasia with cone-rod dystrophy syndrome (disorder)
Congenital Disease	SNOMED	719213009	Short stature Brussels type (disorder)
Congenital Disease	SNOMED	719256004	Pterygium colli with intellectual disability and digital anomaly syndrome (disorder)
Congenital Disease	SNOMED	719257008	Lathosterolosis (disorder)
Congenital Disease	SNOMED	719258003	Pyknoachondrogenesis (disorder)
Congenital Disease	SNOMED	719268008	Progressive non-infectious anterior vertebral fusion (disorder)
Congenital Disease	SNOMED	719282008	Primary ciliary dyskinesia and retinitis pigmentosa syndrome (disorder)
Congenital Disease	SNOMED	719298001	Pelvis shoulder dysplasia (disorder)
Congenital Disease	SNOMED	719299009	Pelviscapular dysplasia syndrome (disorder)
Congenital Disease	SNOMED	719304005	Spondylometaphyseal dysplasia Schmidt type (disorder)
Congenital Disease	SNOMED	719305006	Stapes ankylosis with broad thumb and toe syndrome (disorder)
Congenital Disease	SNOMED	719377004	Microcephalus with albinism and digital anomaly syndrome (disorder)
Congenital Disease	SNOMED	719378009	Microcephalus with brachydactyly and kyphoscoliosis syndrome (disorder)
Congenital Disease	SNOMED	719379001	Microcephalus with cardiac defect and lung malsegmentation syndrome (disorder)
Congenital Disease	SNOMED	719380003	Microcephalus cardiomyopathy syndrome (disorder)
Congenital Disease	SNOMED	719394002	Microcephalus cleft palate syndrome (disorder)
Congenital Disease	SNOMED	719395001	Microcephalus facio-cardio-skeletal syndrome Hadziselimovic type (disorder)
Congenital Disease	SNOMED	719397009	Mesomelic dysplasia Kantaputra type (disorder)
Congenital Disease	SNOMED	719398004	Malignant hyperthermia with arthrogryposis and torticollis syndrome (disorder)
Congenital Disease	SNOMED	719400000	Lethal faciocardiomelic dysplasia (disorder)
Congenital Disease	SNOMED	719404009	Lethal recessive chondrodysplasia (disorder)
Congenital Disease	SNOMED	719408007	Lethal omphalocele with cleft palate syndrome (disorder)
Congenital Disease	SNOMED	719409004	Lethal Larsen-like syndrome (disorder)
Congenital Disease	SNOMED	719427001	15q11q13 microduplication syndrome (disorder)
Congenital Disease	SNOMED	719429003	Ectodermal dysplasia with acanthosis nigricans syndrome (disorder)
Congenital Disease	SNOMED	719432000	Late-onset junctional epidermolysis bullosa (disorder)
Congenital Disease	SNOMED	719450007	Disorder of sex development with intellectual disability syndrome (disorder)
Congenital Disease	SNOMED	719452004	Congenital bronchobiliary fistula (disorder)
Congenital Disease	SNOMED	719456001	Cleft lip and cleft palate with intestinal malrotation and cardiopathy syndrome (disorder)
Congenital Disease	SNOMED	719466009	Cleft palate with short stature and vertebral anomaly syndrome (disorder)
Congenital Disease	SNOMED	719468005	Cleft palate with stapes fixation and oligodontia syndrome (disorder)
Congenital Disease	SNOMED	719471002	Cleidorhizomelic syndrome (disorder)
Congenital Disease	SNOMED	719475006	Congenital lipomatous overgrowth, vascular malformation, epidermal nevi, skeletal anomaly syndrome (disorder)
Congenital Disease	SNOMED	719518004	Autosomal dominant palmoplantar keratoderma and congenital alopecia (disorder)
Congenital Disease	SNOMED	71953008	Congenital anomaly of broad ligament (disorder)
Congenital Disease	SNOMED	719574007	14q12 microdeletion syndrome (disorder)
Congenital Disease	SNOMED	719575008	15q14 microdeletion syndrome (disorder)
Congenital Disease	SNOMED	719576009	16p11.2p12.2 microdeletion syndrome (disorder)

Congenital Disease	SNOMED	719577000	16p13.11 microdeletion syndrome (disorder)
Congenital Disease	SNOMED	719578005	16p13.11 microduplication syndrome (disorder)
Congenital Disease	SNOMED	719580004	16q24.3 microdeletion syndrome (disorder)
Congenital Disease	SNOMED	719582007	17p13.3 microduplication syndrome (disorder)
Congenital Disease	SNOMED	719583002	17q11.2 microduplication syndrome (disorder)
Congenital Disease	SNOMED	719584008	17q23.1q23.2 microdeletion syndrome (disorder)
Congenital Disease	SNOMED	719595002	Absence of fingerprints with congenital milia syndrome (disorder)
Congenital Disease	SNOMED	719597005	19p13.12 microdeletion syndrome (disorder)
Congenital Disease	SNOMED	719599008	19q13.11 microdeletion syndrome (disorder)
Congenital Disease	SNOMED	719600006	1p21.3 microdeletion syndrome (disorder)
Congenital Disease	SNOMED	719646006	8p11.2 deletion syndrome (disorder)
Congenital Disease	SNOMED	719649004	1q44 microdeletion syndrome (disorder)
Congenital Disease	SNOMED	719650004	20p12.3 microdeletion syndrome (disorder)
Congenital Disease	SNOMED	719651000	2p15p16.1 microdeletion syndrome (disorder)
Congenital Disease	SNOMED	719652007	2p21 microdeletion syndrome (disorder)
Congenital Disease	SNOMED	719657001	2q23.1 microdeletion syndrome (disorder)
Congenital Disease	SNOMED	719658006	2q24 microdeletion syndrome (disorder)
Congenital Disease	SNOMED	719659003	2q32q33 microdeletion syndrome (disorder)
Congenital Disease	SNOMED	719660008	4q21 microdeletion syndrome (disorder)
Congenital Disease	SNOMED	719661007	5q14.3 microdeletion syndrome (disorder)
Congenital Disease	SNOMED	719662000	6p22 microdeletion syndrome (disorder)
Congenital Disease	SNOMED	719663005	6q25 microdeletion syndrome (disorder)
Congenital Disease	SNOMED	719664004	8q22.1 microdeletion syndrome (disorder)
Congenital Disease	SNOMED	719665003	5q35 microduplication syndrome (disorder)
Congenital Disease	SNOMED	719666002	6q terminal deletion syndrome (disorder)
Congenital Disease	SNOMED	719684000	8q12 microduplication syndrome (disorder)
Congenital Disease	SNOMED	719685004	Absent thumb with short stature and immunodeficiency syndrome (disorder)
Congenital Disease	SNOMED	719686003	Distal monosomy 10p (disorder)
Congenital Disease	SNOMED	719687007	Gingival fibromatosis with facial dysmorphism syndrome (disorder)
Congenital Disease	SNOMED	719688002	Multiple epiphyseal dysplasia Al-Gazali type (disorder)
Congenital Disease	SNOMED	719689005	Multiple epiphyseal dysplasia Beighton type (disorder)
Congenital Disease	SNOMED	71973003	Congenital aplasia of inner ear (disorder)
Congenital Disease	SNOMED	719800009	Deafness, onychodystrophy, osteodystrophy, intellectual disability syndrome (disorder)
Congenital Disease	SNOMED	719808002	Chromosome Xp11.3 microdeletion syndrome (disorder)
Congenital Disease	SNOMED	719810000	X-linked intellectual disability with seizure and psoriasis syndrome (disorder)
Congenital Disease	SNOMED	719811001	X-linked intellectual disability Cabezas type (disorder)
Congenital Disease	SNOMED	719812008	X-linked intellectual disability with plagiocephaly syndrome (disorder)
Congenital Disease	SNOMED	719813003	X-linked mandibulofacial dysostosis (disorder)
Congenital Disease	SNOMED	719819004	Xeroderma pigmentosum and Cockayne syndrome complex (disorder)
Congenital Disease	SNOMED	719823007	Ventricular extrasystoles with syncope, perodactyly and Robin sequence syndrome (disorder)
Congenital Disease	SNOMED	719824001	Vici syndrome (disorder)
Congenital Disease	SNOMED	719825000	X-linked intellectual disability, macrocephaly, macroorchidism syndrome (disorder)
Congenital Disease	SNOMED	719826004	X-linked intellectual disability with acromegaly and hyperactivity syndrome (disorder)
Congenital Disease	SNOMED	719833004	Visceral neuropathy and brain anomaly with facial dysmorphism and developmental delay syndrome (disorder)
Congenital Disease	SNOMED	719834005	Wilson Turner syndrome (disorder)
Congenital Disease	SNOMED	719835006	Wooly hair and palmoplantar keratoderma with dilated cardiomyopathy syndrome (disorder)
Congenital Disease	SNOMED	719836007	X-linked distal arthrogryposis multiplex congenita (disorder)
Congenital Disease	SNOMED	719837003	X-linked dominant chondrodysplasia Chassaing Lacombe type (disorder)
Congenital Disease	SNOMED	719840003	Renal dysplasia with limb defect syndrome (disorder)
Congenital Disease	SNOMED	719842006	Congenital hypoplasia of ulna and intellectual disability syndrome (disorder)
Congenital Disease	SNOMED	719843001	Ulna fibula ray defect and brachydactyly syndrome (disorder)
Congenital Disease	SNOMED	719844007	Ulceration of umbilical cord and atresia of intestine syndrome (disorder)
Congenital Disease	SNOMED	719845008	Van den Ende-Gupta syndrome (disorder)
Congenital Disease	SNOMED	7199000	Tuberous sclerosis syndrome (disorder)
Congenital Disease	SNOMED	719909009	Chromosome Xq28 trisomy (disorder)
Congenital Disease	SNOMED	719910004	Uncombable hair, retinal pigmentary dystrophy, dental anomaly and brachydactyly syndrome (disorder)
Congenital Disease	SNOMED	719911000	Trichodysplasia with amelogenesis imperfecta syndrome (disorder)
Congenital Disease	SNOMED	719944006	Trichomegaly with retina pigmentary degeneration and dwarfism syndrome (disorder)
Congenital Disease	SNOMED	719945007	Taurodontia with absent teeth and sparse hair syndrome (disorder)
Congenital Disease	SNOMED	719946008	Tel Hashomer camptodactyly syndrome (disorder)
Congenital Disease	SNOMED	719947004	Craniofacial dysmorphism with coloboma of eye and corpus callosum agenesis syndrome (disorder)
Congenital Disease	SNOMED	719948009	Trigonocephaly with bifid nose and acral anomaly syndrome (disorder)
Congenital Disease	SNOMED	719949001	Trigonocephaly with broad thumb syndrome (disorder)
Congenital Disease	SNOMED	719950001	Triphalangeal thumb and polysyndactyly syndrome (disorder)
Congenital Disease	SNOMED	719951002	Triphalangeal thumb with brachyectrodactyly syndrome (disorder)
Congenital Disease	SNOMED	719955006	Atresia of pulmonary valve (disorder)

Congenital Disease	SNOMED	719973009	Haim Munk syndrome (disorder)
Congenital Disease	SNOMED	720009004	Intractable diarrhea with choanal atresia and eye anomaly syndrome (disorder)
Congenital Disease	SNOMED	720010009	Microphthalmia with brain atrophy syndrome (disorder)
Congenital Disease	SNOMED	72014004	Abnormal fetal duplication (disorder)
Congenital Disease	SNOMED	720345008	Severe T-cell immunodeficiency, congenital alopecia, nail dystrophy syndrome (disorder)
Congenital Disease	SNOMED	720394008	Congenital tracheobiliary fistula (disorder)
Congenital Disease	SNOMED	720408003	Acrofrontofacionasal dysostosis (disorder)
Congenital Disease	SNOMED	720410001	Ac[REDACTED]oocular syndrome (disorder)
Congenital Disease	SNOMED	720412009	Acropectoral syndrome (disorder)
Congenital Disease	SNOMED	720414005	Acrorenal mandibular syndrome (disorder)
Congenital Disease	SNOMED	720415006	Acrorenoocular syndrome (disorder)
Congenital Disease	SNOMED	720416007	Acrocapitofemoral dysplasia (disorder)
Congenital Disease	SNOMED	720417003	Acrocephalopolydactyly (disorder)
Congenital Disease	SNOMED	720418008	Acrocraniofacial dysostosis (disorder)
Congenital Disease	SNOMED	720419000	Acrofacial dysostosis Catania type (disorder)
Congenital Disease	SNOMED	720427009	Acrofacial dysostosis Kennedy Teebi type (disorder)
Congenital Disease	SNOMED	720429007	Acrofacial dysostosis Palagonia type (disorder)
Congenital Disease	SNOMED	720430002	Acrofacial dysostosis Rodriguez type (disorder)
Congenital Disease	SNOMED	720456009	Acromegaloid facial appearance syndrome (disorder)
Congenital Disease	SNOMED	720457000	Acropectorovertebral dysplasia (disorder)
Congenital Disease	SNOMED	720458005	Acrorenal syndrome (disorder)
Congenital Disease	SNOMED	720463009	Adducted thumbs and arthrogryposis syndrome Christian type (disorder)
Congenital Disease	SNOMED	720464003	Acro-dermato-ungual-lacrimal-tooth syndrome (disorder)
Congenital Disease	SNOMED	720467005	Aniridia and absent patella syndrome (disorder)
Congenital Disease	SNOMED	720468000	Aniridia and intellectual disability syndrome (disorder)
Congenital Disease	SNOMED	720492008	Ankylosing vertebral hyperostosis with tylosis syndrome (disorder)
Congenital Disease	SNOMED	720494009	Anonychia with microcephaly syndrome (disorder)
Congenital Disease	SNOMED	720495005	Anophthalmia and megalocornea with cardiopathy and skeletal anomalies syndrome (disorder)
Congenital Disease	SNOMED	720496006	Anophthalmia plus syndrome (disorder)
Congenital Disease	SNOMED	720498007	Aphalangy and syndactyly with microcephaly syndrome (disorder)
Congenital Disease	SNOMED	720499004	Aplasia cutis with myopia syndrome (disorder)
Congenital Disease	SNOMED	720500008	Aplasia cutis congenita with intestinal lymphangiectasia syndrome (disorder)
Congenital Disease	SNOMED	720501007	Arachnodactyly with abnormal ossification and intellectual disability syndrome (disorder)
Congenital Disease	SNOMED	720502000	Arachnodactyly and intellectual disability with facial dysmorphism syndrome (disorder)
Congenital Disease	SNOMED	720511000	Arrhinia with choanal atresia and microphthalmia syndrome (disorder)
Congenital Disease	SNOMED	720512007	Arterial dissection and lentiginosis syndrome (disorder)
Congenital Disease	SNOMED	720513002	Arthrogryposis with renal dysfunction and cholestasis syndrome (disorder)
Congenital Disease	SNOMED	720514008	Arthrogryposis multiplex congenita and whistling face syndrome (disorder)
Congenital Disease	SNOMED	720515009	Distal arthrogryposis type 6 (disorder)
Congenital Disease	SNOMED	720518006	Athabaskan brainstem dysgenesis syndrome (disorder)
Congenital Disease	SNOMED	720519003	Atherosclerosis, deafness, diabetes, epilepsy, nephropathy syndrome (disorder)
Congenital Disease	SNOMED	720565000	Bohring Opitz syndrome (disorder)
Congenital Disease	SNOMED	720567008	Bosley Salih Alorainy syndrome (disorder)
Congenital Disease	SNOMED	720568003	Brachydactyly and arterial hypertension syndrome (disorder)
Congenital Disease	SNOMED	720569006	Brachydactyly type A2 (disorder)
Congenital Disease	SNOMED	720570007	Brachydactyly type A5 (disorder)
Congenital Disease	SNOMED	720571006	Brachydactyly type A7 (disorder)
Congenital Disease	SNOMED	720572004	Brachydactyly with syndactyly Zhao type (disorder)
Congenital Disease	SNOMED	720573009	Brachymorphism with onychodysplasia and dysphalangism syndrome (disorder)
Congenital Disease	SNOMED	720574003	Brachytelephalangy, facial dysmorphism, Kallmann syndrome (disorder)
Congenital Disease	SNOMED	720575002	Braddock syndrome (disorder)
Congenital Disease	SNOMED	720598005	Doughnut lesion of calvaria and bone fragility syndrome (disorder)
Congenital Disease	SNOMED	720599002	Campomelia Cumming type (disorder)
Congenital Disease	SNOMED	720600004	Camptodactyly with fibrous tissue hyperplasia and skeletal dysplasia syndrome (disorder)
Congenital Disease	SNOMED	720601000	Camptodactyly and tall stature with scoliosis and hearing loss syndrome (disorder)
Congenital Disease	SNOMED	720602007	Camptodactyly syndrome Guadalajara type 1 (disorder)
Congenital Disease	SNOMED	720603002	Camptodactyly syndrome Guadalajara type 2 (disorder)
Congenital Disease	SNOMED	720605009	Cardiac anomaly and heterotaxy syndrome (disorder)
Congenital Disease	SNOMED	720606005	Cardiocranial syndrome Pfeiffer type (disorder)
Congenital Disease	SNOMED	720610008	Cardiomyopathy and renal anomaly syndrome (disorder)
Congenital Disease	SNOMED	720612000	Cardiospondylocarpofacial syndrome (disorder)
Congenital Disease	SNOMED	720632004	Central bilateral macrogyria (disorder)
Congenital Disease	SNOMED	720633009	Syndactyly type 7 (disorder)
Congenital Disease	SNOMED	720634003	Cerebellar ataxia, areflexia, pes cavus, optic atrophy, sensorineural hearing loss syndrome (disorder)
Congenital Disease	SNOMED	720635002	Cerebro-facio-thoracic dysplasia (disorder)

Congenital Disease	SNOMED	720636001	Cholestasis with pigmentary retinopathy and cleft palate syndrome (disorder)
Congenital Disease	SNOMED	720639008	Coloboma, congenital heart disease, ichthyosiform dermatosis, intellectual disability ear anomaly syndrome (disorder)
Congenital Disease	SNOMED	720640005	Choanal atresia, hearing loss, cardiac defect, craniofacial dysmorphism syndrome (disorder)
Congenital Disease	SNOMED	720746006	Contracture with ectodermal dysplasia and orofacial cleft syndrome (disorder)
Congenital Disease	SNOMED	720747002	Cooks syndrome (disorder)
Congenital Disease	SNOMED	720748007	Aural atresia with multiple congenital anomalies and intellectual disability syndrome (disorder)
Congenital Disease	SNOMED	720752007	Coxopodopatellar syndrome (disorder)
Congenital Disease	SNOMED	720754008	Craniofacial conodysplasia syndrome (disorder)
Congenital Disease	SNOMED	720755009	Craniofacial dyssynostosis syndrome (disorder)
Congenital Disease	SNOMED	720756005	Craniofacial ulnar renal syndrome (disorder)
Congenital Disease	SNOMED	720757001	Craniofrontonasal dysplasia with Poland anomaly syndrome (disorder)
Congenital Disease	SNOMED	720812002	Craniosynostosis, anal anomaly, porokeratosis syndrome (disorder)
Congenital Disease	SNOMED	720813007	Craniosynostosis with Dandy-Walker malformation and hydrocephalus syndrome (disorder)
Congenital Disease	SNOMED	720815000	Capra DeMarco syndrome (disorder)
Congenital Disease	SNOMED	720816004	Craniosynostosis and intracranial calcification syndrome (disorder)
Congenital Disease	SNOMED	720817008	Craniosynostosis Boston type (disorder)
Congenital Disease	SNOMED	720818003	Craniosynostosis Philadelphia type (disorder)
Congenital Disease	SNOMED	720819006	Curry Jones syndrome (disorder)
Congenital Disease	SNOMED	720826006	Czech dysplasia metatarsal type (disorder)
Congenital Disease	SNOMED	720850008	Choroidal atrophy and alopecia syndrome (disorder)
Congenital Disease	SNOMED	720851007	Chondrodysplasia with disorder of sex development syndrome (disorder)
Congenital Disease	SNOMED	720852000	Cervical hypertrichosis and peripheral neuropathy syndrome (disorder)
Congenital Disease	SNOMED	720855003	Cerebrooculonasal syndrome (disorder)
Congenital Disease	SNOMED	720856002	Ectodermal dysplasia with ectrodactyly and macular dystrophy syndrome (disorder)
Congenital Disease	SNOMED	720858001	Ehlers-Danlos syndrome cardiac valvular type (disorder)
Congenital Disease	SNOMED	720859009	Ehlers-Danlos syndrome kyphoscoliotic and deafness type (disorder)
Congenital Disease	SNOMED	720860004	Ehlers-Danlos syndrome musculocontractural type (disorder)
Congenital Disease	SNOMED	720861000	Ehlers-Danlos syndrome progeroid type (disorder)
Congenital Disease	SNOMED	720863002	Eiken syndrome (disorder)
Congenital Disease	SNOMED	72089000	Congenital deformity of nose (disorder)
Congenital Disease	SNOMED	720952001	Aplasia of fibula and ectrodactyly syndrome (disorder)
Congenital Disease	SNOMED	720953006	Fibular dimelia diplopodia syndrome (disorder)
Congenital Disease	SNOMED	720954000	Filippi syndrome (disorder)
Congenital Disease	SNOMED	720955004	Fine Lubinsky syndrome (disorder)
Congenital Disease	SNOMED	720957007	Deafness with skeletal dysplasia and lip granuloma syndrome (disorder)
Congenital Disease	SNOMED	720958002	Frank-Ter Haar syndrome (disorder)
Congenital Disease	SNOMED	720979002	Alopecia, contracture, dwarfism, intellectual disability syndrome (disorder)
Congenital Disease	SNOMED	720980004	Alopecia, psychomotor epilepsy, periodontal pyorrhea, intellectual disability syndrome (disorder)
Congenital Disease	SNOMED	720981000	Alopecia and intellectual disability with hypergonadotropic hypogonadism syndrome (disorder)
Congenital Disease	SNOMED	720982007	Alport syndrome, intellectual disability, midface hypoplasia, elliptocytosis syndrome (disorder)
Congenital Disease	SNOMED	720984008	Angel-shaped phalangoepiphyseal dysplasia (disorder)
Congenital Disease	SNOMED	720986005	Anhidrotic ectodermal dysplasia, immunodeficiency, osteopetrosis, lymphedema syndrome (disorder)
Congenital Disease	SNOMED	720987001	Aniridia, ptosis, intellectual disability, familial obesity syndrome (disorder)
Congenital Disease	SNOMED	721007005	Hair defect with photosensitivity and intellectual disability syndrome (disorder)
Congenital Disease	SNOMED	721008000	Hall Riggs syndrome (disorder)
Congenital Disease	SNOMED	721009008	Heart defect and limb shortening syndrome (disorder)
Congenital Disease	SNOMED	721010003	Heart-hand syndrome type 2 (disorder)
Congenital Disease	SNOMED	721013001	Heart-hand syndrome type 3 (disorder)
Congenital Disease	SNOMED	721014007	Heart-hand syndrome Slovenian type (disorder)
Congenital Disease	SNOMED	721015008	Hydrocephalus with endocardial fibroelastosis and cataract syndrome (disorder)
Congenital Disease	SNOMED	721017000	Postaxial polydactyly and intellectual disability syndrome (disorder)
Congenital Disease	SNOMED	721057002	Thoracic insufficiency syndrome (disorder)
Congenital Disease	SNOMED	721069005	Short fifth metacarpal insulin resistance syndrome (disorder)
Congenital Disease	SNOMED	721072003	Short stature, pituitary and cerebellar defect and small sella turcica syndrome (disorder)
Congenital Disease	SNOMED	721073008	Short stature with webbed neck and congenital heart disease syndrome (disorder)
Congenital Disease	SNOMED	721075001	Short tarsus with absence of lower eyelashes syndrome (disorder)
Congenital Disease	SNOMED	721076000	Siegler Brewer Carey syndrome (disorder)
Congenital Disease	SNOMED	721082002	Dacryocystitis and osteopoikilosis syndrome (disorder)
Congenital Disease	SNOMED	721083007	Lymphedema hypoparathyroidism syndrome (disorder)
Congenital Disease	SNOMED	721084001	Deaf blind hypopigmentation syndrome Yemenite type (disorder)

Congenital Disease	SNOMED	721085000	Deafness, enamel hypoplasia, nail defect syndrome (disorder)
Congenital Disease	SNOMED	721086004	Deafness, genital anomaly, metacarpal and metatarsal synostosis syndrome (disorder)
Congenital Disease	SNOMED	721087008	Deafness and intellectual disability Martin Probst type syndrome (disorder)
Congenital Disease	SNOMED	721089006	Dentinogenesis imperfecta, short stature, hearing loss, intellectual disability syndrome (disorder)
Congenital Disease	SNOMED	721090002	Dermatoosteolysis Kirghizian type (disorder)
Congenital Disease	SNOMED	721091003	Dermo-odonto dysplasia (disorder)
Congenital Disease	SNOMED	721092005	Developmental malformation, deafness, dystonia syndrome (disorder)
Congenital Disease	SNOMED	721094006	Diaphanospondylodysostosis (disorder)
Congenital Disease	SNOMED	721095007	Diaphragmatic defect, limb deficiency, skull defect syndrome (disorder)
Congenital Disease	SNOMED	721096008	Diffuse palmoplantar keratoderma and acrocyanosis syndrome (disorder)
Congenital Disease	SNOMED	721105004	Klippel Trenaunay syndrome (disorder)
Congenital Disease	SNOMED	721146009	Intellectual disability, epilepsy, bulbous nose syndrome (disorder)
Congenital Disease	SNOMED	721147000	Hidrotic ectodermal dysplasia Halal type (disorder)
Congenital Disease	SNOMED	721148005	Hip dysplasia Beukes type (disorder)
Congenital Disease	SNOMED	721152005	Congenital absence of gastric muscle (disorder)
Congenital Disease	SNOMED	721154006	Congenital chalasia of esophagus (disorder)
Congenital Disease	SNOMED	721155007	Congenital short esophagus (disorder)
Congenital Disease	SNOMED	721158009	Deletion 5q35 (disorder)
Congenital Disease	SNOMED	721161005	Duplication cyst of esophagus (disorder)
Congenital Disease	SNOMED	721168004	Congenital hydronephrosis due to ureteropelvic junction obstruction (disorder)
Congenital Disease	SNOMED	721182004	Long gap atresia of esophagus (disorder)
Congenital Disease	SNOMED	721208007	Ectodermal dysplasia with blindness syndrome (disorder)
Congenital Disease	SNOMED	721221000	Hirschsprung disease with deafness and polydactyly syndrome (disorder)
Congenital Disease	SNOMED	721222007	Hirschsprung disease with type D brachydactyly syndrome (disorder)
Congenital Disease	SNOMED	721223002	Hirschsprung disease with nail hypoplasia and dysmorphism (disorder)
Congenital Disease	SNOMED	721227001	Hunter McAlpine craniosynostosis syndrome (disorder)
Congenital Disease	SNOMED	721229003	Hydrocephalus, costovertebral dysplasia, Sprengel anomaly syndrome (disorder)
Congenital Disease	SNOMED	721231007	Hydrocephalus with obesity and hypogonadism syndrome (disorder)
Congenital Disease	SNOMED	721232000	Hydrolethalus syndrome (disorder)
Congenital Disease	SNOMED	721233005	Hypergonadotropic hypogonadism with cataract syndrome (disorder)
Congenital Disease	SNOMED	721296004	Fuhrmann syndrome (disorder)
Congenital Disease	SNOMED	721297008	Galloway Mowat syndrome (disorder)
Congenital Disease	SNOMED	721584005	Johnson neuroectodermal syndrome (disorder)
Congenital Disease	SNOMED	721608001	Hirschsprung disease of rectosigmoid region (disorder)
Congenital Disease	SNOMED	721609009	Extensive aganglionosis Hirschsprung disease (disorder)
Congenital Disease	SNOMED	721646008	Congenital diverticulitis of small intestine (disorder)
Congenital Disease	SNOMED	721647004	Congenital diverticulosis of small intestine (disorder)
Congenital Disease	SNOMED	721648009	Congenital diverticulum of small intestine (disorder)
Congenital Disease	SNOMED	721649001	Atresia of jejunum type IIIb (disorder)
Congenital Disease	SNOMED	721650001	Atresia of jejunum type IV (disorder)
Congenital Disease	SNOMED	721665000	Hamartoma of small intestine (disorder)
Congenital Disease	SNOMED	721688004	Arteriovenous malformation of large intestine (disorder)
Congenital Disease	SNOMED	721835008	Acrofrontofacionasal dysostosis type 2 (disorder)
Congenital Disease	SNOMED	721836009	Hypertelorism with microtia and facial clefting syndrome (disorder)
Congenital Disease	SNOMED	721841001	Hypogonadism with mitral valve prolapse and intellectual disability syndrome (disorder)
Congenital Disease	SNOMED	721843003	Growth retardation, alopecia, pseudoanodontia, optic atrophy syndrome (disorder)
Congenital Disease	SNOMED	721845005	Hypomandibular faciocranial dysostosis (disorder)
Congenital Disease	SNOMED	721847002	Joubert syndrome with congenital hepatic fibrosis (disorder)
Congenital Disease	SNOMED	721862000	Joubert syndrome with oculorenal defect (disorder)
Congenital Disease	SNOMED	721873007	Joubert syndrome with orofaciodigital defect (disorder)
Congenital Disease	SNOMED	721874001	Juberg Hayward syndrome (disorder)
Congenital Disease	SNOMED	721875000	Juberg Marsidi syndrome (disorder)
Congenital Disease	SNOMED	721878003	Microphthalmia with brain and digit anomaly (disorder)
Congenital Disease	SNOMED	721879006	Microphthalmia with linear skin defect syndrome (disorder)
Congenital Disease	SNOMED	721880009	Congenital microgastria with limb reduction defect syndrome (disorder)
Congenital Disease	SNOMED	721881008	Microduplication Xp11.22p11.23 syndrome (disorder)
Congenital Disease	SNOMED	721882001	Radioulnar synostosis with amegakaryocytic thrombocytopenia syndrome (disorder)
Congenital Disease	SNOMED	721883006	Radioulnar synostosis with developmental delay and hypotonia syndrome (disorder)
Congenital Disease	SNOMED	721887007	Puerto Rican infant hypotonia syndrome (disorder)
Congenital Disease	SNOMED	721888002	Scalp, ear, nipple syndrome (disorder)
Congenital Disease	SNOMED	721902002	Schilbach Rott syndrome (disorder)
Congenital Disease	SNOMED	721903007	Microcephaly, hypogammaglobulinemia, abnormal immunity syndrome (disorder)
Congenital Disease	SNOMED	721970009	Persistent Mullerian derivative with lymphangiectasia and polydactyly syndrome (disorder)
Congenital Disease	SNOMED	721972001	Limb mammary syndrome (disorder)
Congenital Disease	SNOMED	721973006	Lipodystrophy, intellectual disability, deafness syndrome (disorder)

Congenital Disease	SNOMED	721974000	Lowry MacLean syndrome (disorder)
Congenital Disease	SNOMED	721975004	Epiphyseal dysplasia, microcephalus, nystagmus syndrome (disorder)
Congenital Disease	SNOMED	721976003	Lung agenesis with heart defect and thumb anomaly syndrome (disorder)
Congenital Disease	SNOMED	721977007	Lung fibrosis, immunodeficiency, 46,XX gonadal dysgenesis syndrome (disorder)
Congenital Disease	SNOMED	721978002	Lymphedema, atrial septal defect, facial changes syndrome (disorder)
Congenital Disease	SNOMED	721979005	Lymphedema and cerebral arteriovenous anomaly syndrome (disorder)
Congenital Disease	SNOMED	722002002	Intellectual disability, balding, patella luxation, acromicria syndrome (disorder)
Congenital Disease	SNOMED	722003007	Intellectual disability with cataract and kyphosis syndrome (disorder)
Congenital Disease	SNOMED	722004001	Agenesis of internal carotid artery (disorder)
Congenital Disease	SNOMED	722006004	Isotretinoin embryopathy-like syndrome (disorder)
Congenital Disease	SNOMED	722019000	Oculootoradial syndrome (disorder)
Congenital Disease	SNOMED	722027009	Kallman syndrome with heart disease (disorder)
Congenital Disease	SNOMED	722031003	Kapur Toriello syndrome (disorder)
Congenital Disease	SNOMED	722032005	Karsch Neugebauer syndrome (disorder)
Congenital Disease	SNOMED	722033000	Macrocephaly, short stature, paraplegia syndrome (disorder)
Congenital Disease	SNOMED	722035007	Intellectual disability, enteropathy, deafness, peripheral neuropathy, ichthyosis, keratoderma syndrome (disorder)
Congenital Disease	SNOMED	722036008	Megalencephaly, polymicrogyria, postaxial polydactyly, hydrocephalus syndrome (disorder)
Congenital Disease	SNOMED	722037004	Intellectual disability, epileptic seizures, hypogonadism and hypogenitalism, microcephaly, obesity syndrome (disorder)
Congenital Disease	SNOMED	722051004	Obesity, colitis, hypothyroidism, cardiac hypertrophy, developmental delay syndrome (disorder)
Congenital Disease	SNOMED	722055008	Oculopalatocerebral syndrome (disorder)
Congenital Disease	SNOMED	722056009	Oculocerebrofacial syndrome Kaufman type (disorder)
Congenital Disease	SNOMED	722061006	Oculoosteocutaneous syndrome (disorder)
Congenital Disease	SNOMED	722062004	Oculotrichodysplasia (disorder)
Congenital Disease	SNOMED	722063009	Odonto-tricho-ungual-digito-palmar syndrome (disorder)
Congenital Disease	SNOMED	722065002	Okamoto syndrome (disorder)
Congenital Disease	SNOMED	722075004	Oro-facial digital syndrome type 10 (disorder)
Congenital Disease	SNOMED	722105002	Oro-facial digital syndrome type 5 (disorder)
Congenital Disease	SNOMED	722106001	Oro-facial digital syndrome type 8 (disorder)
Congenital Disease	SNOMED	722107005	Ossification anomaly with psychomotor developmental delay syndrome (disorder)
Congenital Disease	SNOMED	722109008	Osteocraniostenosis (disorder)
Congenital Disease	SNOMED	722110003	Osteogenesis imperfecta, retinopathy, seizures, intellectual disability syndrome (disorder)
Congenital Disease	SNOMED	722113001	Osteoporosis and oculocutaneous hypopigmentation syndrome (disorder)
Congenital Disease	SNOMED	722114007	Sclerosing dysplasia of bone, ichthyosis, premature ovarian failure syndrome (disorder)
Congenital Disease	SNOMED	722117000	Osteosclerosis, developmental delay, craniosynostosis syndrome (disorder)
Congenital Disease	SNOMED	722122000	Overgrowth, macrocephaly, facial dysmorphism syndrome (disorder)
Congenital Disease	SNOMED	722127006	Pacman dysplasia (disorder)
Congenital Disease	SNOMED	722132007	Pulmonary hypoplasia, agonadism, dextrocardia, diaphragmatic hernia syndrome (disorder)
Congenital Disease	SNOMED	722201004	Median cleft of upper lip, corpus callosum lipoma, cutaneous polyp syndrome (disorder)
Congenital Disease	SNOMED	722202006	Palmoplantar keratoderma, 46,XX sex reversal, predisposition to squamous cell carcinoma syndrome (disorder)
Congenital Disease	SNOMED	722203001	Palmoplantar keratoderma with deafness syndrome (disorder)
Congenital Disease	SNOMED	722205008	Palmoplantar keratoderma Nagashima type (disorder)
Congenital Disease	SNOMED	722206009	Pancreatic hypoplasia, diabetes mellitus, congenital heart disease syndrome (disorder)
Congenital Disease	SNOMED	722207000	Pancreatic insufficiency, dyserythropoietic anemia, calvarial hyperostosis syndrome (disorder)
Congenital Disease	SNOMED	722209002	Spastic paraplegia, intellectual disability, palmoplantar hyperkeratosis syndrome (disorder)
Congenital Disease	SNOMED	722210007	Parastremmatic dwarfism (disorder)
Congenital Disease	SNOMED	722211006	Patent ductus arteriosus, bicuspid aortic valve, hand anomaly syndrome (disorder)
Congenital Disease	SNOMED	722231005	Perlman syndrome (disorder)
Congenital Disease	SNOMED	722280000	Ackerman syndrome (disorder)
Congenital Disease	SNOMED	722281001	Agammaglobulinemia, microcephaly, craniosynostosis, severe dermatitis syndrome (disorder)
Congenital Disease	SNOMED	722282008	Agenesis of corpus callosum, intellectual disability, coloboma, micrognathia syndrome (disorder)
Congenital Disease	SNOMED	722283003	Agnathia, holoprosencephaly, situs inversus syndrome (disorder)
Congenital Disease	SNOMED	722284009	Hypoplasia and coloboma of alar cartilage with telecanthus syndrome (disorder)
Congenital Disease	SNOMED	722287002	Autism and facial port-wine stain syndrome (disorder)
Congenital Disease	SNOMED	722296002	Book syndrome (disorder)
Congenital Disease	SNOMED	722298001	Ballard syndrome (disorder)
Congenital Disease	SNOMED	722375007	Bamforth Lazarus syndrome (disorder)

Congenital Disease	SNOMED	722376008	Autosomal recessive popliteal pterygium syndrome (disorder)
Congenital Disease	SNOMED	722378009	Congenital cataract with deafness and hypogonadism syndrome (disorder)
Congenital Disease	SNOMED	722379001	Congenital cataract with hypertrichosis and intellectual disability syndrome (disorder)
Congenital Disease	SNOMED	722380003	Congenital cataract with intellectual disability and hypogonadotropic hypogonadism syndrome (disorder)
Congenital Disease	SNOMED	722381004	Congenital cataract, nephropathy, encephalopathy syndrome (disorder)
Congenital Disease	SNOMED	722382006	Cataract and microcornea syndrome (disorder)
Congenital Disease	SNOMED	722383001	Catel Manzke syndrome (disorder)
Congenital Disease	SNOMED	722385008	Cerebral dysgenesis, neuropathy, ichthyosis, palmoplantar keratoderma syndrome (disorder)
Congenital Disease	SNOMED	722389002	Congenital hereditary facial paralysis with variable hearing loss syndrome (disorder)
Congenital Disease	SNOMED	722390006	Congenital intrauterine infection-like syndrome (disorder)
Congenital Disease	SNOMED	72239002	Long narrow head (disorder)
Congenital Disease	SNOMED	722391005	Congenital lethal erythroderma (disorder)
Congenital Disease	SNOMED	72242008	Postductal coarctation of aorta (disorder)
Congenital Disease	SNOMED	722429003	Distal limb deficiency with micrognathia syndrome (disorder)
Congenital Disease	SNOMED	722430008	Distal trisomy 6p syndrome (disorder)
Congenital Disease	SNOMED	722431007	Double uterus, hemivagina, renal agenesis syndrome (disorder)
Congenital Disease	SNOMED	722432000	Duane anomaly, myopathy, scoliosis syndrome (disorder)
Congenital Disease	SNOMED	722433005	Dyschondrosteosis and nephritis syndrome (disorder)
Congenital Disease	SNOMED	722434004	Dysspondyloenchondromatosis (disorder)
Congenital Disease	SNOMED	722436002	Dystrophic epidermolysis bullosa nails only (disorder)
Congenital Disease	SNOMED	722437006	Ectopia lentis, chorioretinal dystrophy, myopia syndrome (disorder)
Congenital Disease	SNOMED	722439009	Endothelial dystrophy, iris hypoplasia, congenital cataract, stromal thinning syndrome (disorder)
Congenital Disease	SNOMED	722450007	Glaucoma, ectopia, microspherophakia, stiff joint, short stature syndrome (disorder)
Congenital Disease	SNOMED	722451006	Gomez Lopez Hernandez syndrome (disorder)
Congenital Disease	SNOMED	722452004	Preaxial deficiency, postaxial polydactyly, hypospadias syndrome (disorder)
Congenital Disease	SNOMED	722453009	Cutaneous mastocytosis, short stature, hearing loss syndrome (disorder)
Congenital Disease	SNOMED	722454003	Intellectual disability, craniofacial dysmorphism, hypogonadism, diabetes mellitus syndrome (disorder)
Congenital Disease	SNOMED	722455002	Intellectual disability, hypoplastic corpus callosum, preauricular tag syndrome (disorder)
Congenital Disease	SNOMED	722456001	Intellectual disability, developmental delay, contracture syndrome (disorder)
Congenital Disease	SNOMED	722457005	Juvenile cataract, microcornea, renal glucosuria syndrome (disorder)
Congenital Disease	SNOMED	722458000	Matthew Wood syndrome (disorder)
Congenital Disease	SNOMED	722459008	Male hypergonadotropic hypogonadism, intellectual disability, skeletal anomaly syndrome (disorder)
Congenital Disease	SNOMED	722461004	Meacham syndrome (disorder)
Congenital Disease	SNOMED	722463001	Macular coloboma, cleft palate, hallux valgus syndrome (disorder)
Congenital Disease	SNOMED	722477003	Toriello Carey syndrome (disorder)
Congenital Disease	SNOMED	722478008	Skeletal dysplasia with intellectual disability syndrome (disorder)
Congenital Disease	SNOMED	722493007	Familial caudal dysgenesis (disorder)
Congenital Disease	SNOMED	72252007	Congenital hypoplasia of cardiac vein (disorder)
Congenital Disease	SNOMED	722604002	Optic disc dysplasia (disorder)
Congenital Disease	SNOMED	722675000	Laryngo-onycho-cutaneous syndrome (disorder)
Congenital Disease	SNOMED	722853000	Immature ganglionosis of large intestine (disorder)
Congenital Disease	SNOMED	722854006	Congenital hypoganglionosis of large intestine (disorder)
Congenital Disease	SNOMED	722859001	PTEN hamartoma tumor syndrome (disorder)
Congenital Disease	SNOMED	722860006	Congenital perineal groove (disorder)
Congenital Disease	SNOMED	722868004	Congenital developmental anomaly of cystic duct (disorder)
Congenital Disease	SNOMED	722912007	Congenital pulmonary hypoplasia due to prolonged premature rupture of membranes (disorder)
Congenital Disease	SNOMED	722913002	Congenital pulmonary hypoplasia due to lung space occupying lesion (disorder)
Congenital Disease	SNOMED	722917001	Congenital cyst of aryepiglottic fold (disorder)
Congenital Disease	SNOMED	72292009	Congenital anomaly of larynx (disorder)
Congenital Disease	SNOMED	722980006	Dementia due to chromosomal anomaly (disorder)
Congenital Disease	SNOMED	722989007	Aplasia of optic nerve (disorder)
Congenital Disease	SNOMED	722990003	Congenital atrophy of optic nerve (disorder)
Congenital Disease	SNOMED	722992006	Communicating hydrocephalus co-occurrent and due to congenital agenesis of arachnoid villi (disorder)
Congenital Disease	SNOMED	722996009	Congenital malformation of autonomic nervous system (disorder)
Congenital Disease	SNOMED	723131006	Megalopapilla (disorder)
Congenital Disease	SNOMED	723163000	Basal epidermolysis bullosa simplex (disorder)
Congenital Disease	SNOMED	723183004	Aganglionosis of large intestine (disorder)
Congenital Disease	SNOMED	723304001	Microcephaly, seizure, intellectual disability, heart disease syndrome (disorder)
Congenital Disease	SNOMED	723308003	Epidermolysis bullosa simplex with muscular dystrophy (disorder)
Congenital Disease	SNOMED	723309006	Endocrine-cerebro-osteodysplasia syndrome (disorder)
Congenital Disease	SNOMED	723332005	Isodicentric chromosome 15 syndrome (disorder)
Congenital Disease	SNOMED	723333000	Faciocardiorenal syndrome (disorder)

Congenital Disease	SNOMED	723336008	Fallot complex with intellectual disability and growth delay syndrome (disorder)
Congenital Disease	SNOMED	723363009	Hypotrichosis, lymphedema, telangiectasia, renal defect syndrome (disorder)
Congenital Disease	SNOMED	723364003	Hypotrichosis with juvenile macular degeneration syndrome (disorder)
Congenital Disease	SNOMED	723365002	Hypotrichosis and intellectual disability syndrome Lopes type (disorder)
Congenital Disease	SNOMED	723366001	Macrostomia, preauricular tag, external ophthalmoplegia syndrome (disorder)
Congenital Disease	SNOMED	723367005	Macrocephaly, alopecia, cutis laxa, scoliosis syndrome (disorder)
Congenital Disease	SNOMED	723373006	Uromodulin related autosomal dominant tubulointerstitial kidney disease (disorder)
Congenital Disease	SNOMED	723383005	Midline cleft of lower lip (disorder)
Congenital Disease	SNOMED	723403008	Microbrachycephaly, ptosis, cleft lip syndrome (disorder)
Congenital Disease	SNOMED	723404002	Microcephalic osteodysplastic dysplasia Saul Wilson type (disorder)
Congenital Disease	SNOMED	723405001	Microlissencephaly micromelia syndrome (disorder)
Congenital Disease	SNOMED	723406000	Embryopathy caused by mycophenolate mofetil (disorder)
Congenital Disease	SNOMED	723409007	Multinodular goiter, cystic kidney, polydactyly syndrome (disorder)
Congenital Disease	SNOMED	723410002	N syndrome (disorder)
Congenital Disease	SNOMED	723411003	Nasopalpebral lipoma coloboma syndrome (disorder)
Congenital Disease	SNOMED	723442008	Non-eruption of teeth, maxillary hypoplasia, genu valgum syndrome (disorder)
Congenital Disease	SNOMED	723444009	Noonan syndrome-like disorder with loose anagen hair (disorder)
Congenital Disease	SNOMED	723446006	Polydactyly of index finger (disorder)
Congenital Disease	SNOMED	723448007	Polyvalvular heart disease syndrome (disorder)
Congenital Disease	SNOMED	723449004	Pierson syndrome (disorder)
Congenital Disease	SNOMED	723451000	Pili torti onychodysplasia syndrome (disorder)
Congenital Disease	SNOMED	723453002	Pterygia, heart anomaly, autosomal recessive inheritance, vertebral defect, ear anomaly, radial defect syndrome (disorder)
Congenital Disease	SNOMED	723455009	Phakomatosis pigmentokeratotica (disorder)
Congenital Disease	SNOMED	723461007	Pierre Robin sequence faciodigital anomaly syndrome (disorder)
Congenital Disease	SNOMED	723500009	Autosomal recessive aplasia cutis congenita of limb (disorder)
Congenital Disease	SNOMED	723503006	Retinal degeneration, nanophthalmos, glaucoma syndrome (disorder)
Congenital Disease	SNOMED	723504000	Ramos Arroyo syndrome (disorder)
Congenital Disease	SNOMED	723512008	Revesz syndrome (disorder)
Congenital Disease	SNOMED	723553000	Transient bullous dermolysis of newborn (disorder)
Congenital Disease	SNOMED	723554006	Aplasia cutis congenita with epibulbar dermoid syndrome (disorder)
Congenital Disease	SNOMED	723555007	Thymic, renal, anal, lung dysplasia syndrome (disorder)
Congenital Disease	SNOMED	723556008	Thoracolaryngopelvic dysplasia syndrome (disorder)
Congenital Disease	SNOMED	723578001	Terminal osseous dysplasia and pigmentary defect syndrome (disorder)
Congenital Disease	SNOMED	723581006	Syndactyly, telecanthus, anogenital and renal malformation syndrome (disorder)
Congenital Disease	SNOMED	723584003	Stern Lubinsky Durrie syndrome (disorder)
Congenital Disease	SNOMED	723610009	Spondylocostal dysostosis with anal atresia and genitourinary malformation syndrome (disorder)
Congenital Disease	SNOMED	723611008	Split hand, split foot malformation with sensorineural hearing loss syndrome (disorder)
Congenital Disease	SNOMED	723612001	Spinal muscular atrophy, Dandy-Walker malformation, cataract syndrome (disorder)
Congenital Disease	SNOMED	723676007	Severe intellectual disability, epilepsy, anal anomaly, distal phalangeal hypoplasia syndrome (disorder)
Congenital Disease	SNOMED	723716009	Severe generalized recessive dystrophic epidermolysis bullosa (disorder)
Congenital Disease	SNOMED	723720008	Sex reversion, kidney, adrenal and lung dysgenesis syndrome (disorder)
Congenital Disease	SNOMED	723827003	Grant syndrome (disorder)
Congenital Disease	SNOMED	723829000	Pulmonary fibrosis, hepatic hyperplasia, bone marrow hypoplasia syndrome (disorder)
Congenital Disease	SNOMED	723830005	Keratosis follicularis, dwarfism, cerebral atrophy syndrome (disorder)
Congenital Disease	SNOMED	723867002	Coarctation of aortic arch (disorder)
Congenital Disease	SNOMED	723888006	Clinodactyly of toe (disorder)
Congenital Disease	SNOMED	723973002	Sirenomelus (disorder)
Congenital Disease	SNOMED	723974008	Polysomia (disorder)
Congenital Disease	SNOMED	723991007	Angio-osteohypertrophic syndrome (disorder)
Congenital Disease	SNOMED	723995003	Schimke immuno-osseous dysplasia (disorder)
Congenital Disease	SNOMED	723998001	Short stature, Pierre Robin sequence, cleft mandible, hand anomalies, clubfoot syndrome (disorder)
Congenital Disease	SNOMED	723999009	Retinitis pigmentosa, hypopituitarism, nephronophthisis, skeletal dysplasia syndrome (disorder)
Congenital Disease	SNOMED	724000006	Retinohepatoendocrinologic syndrome (disorder)
Congenital Disease	SNOMED	724001005	Retinitis pigmentosa, intellectual disability, deafness, hypogenitalism syndrome (disorder)
Congenital Disease	SNOMED	724016008	Ptosis, upper ocular movement limitation, absence of lacrimal punctum syndrome (disorder)
Congenital Disease	SNOMED	724064004	Posterior fusion of lumbosacral vertebrae and blepharoptosis syndrome (disorder)
Congenital Disease	SNOMED	724066002	Polysyndactyly and cardiac malformation syndrome (disorder)
Congenital Disease	SNOMED	724067006	Permanent neonatal diabetes mellitus with cerebellar agenesis syndrome (disorder)

Congenital Disease	SNOMED	724068001	Pericardial and diaphragmatic defect syndrome (disorder)
Congenital Disease	SNOMED	724069009	Patterson Stevenson Fontaine syndrome (disorder)
Congenital Disease	SNOMED	724070005	Paternal 20q13.2q13.3 microdeletion syndrome (disorder)
Congenital Disease	SNOMED	724071009	Congenital hypoplasia of patella (disorder)
Congenital Disease	SNOMED	724092009	Nephrosis, deafness, urinary tract, digital malformation syndrome (disorder)
Congenital Disease	SNOMED	724093004	Nephropathy, deafness, hyperparathyroidism syndrome (disorder)
Congenital Disease	SNOMED	724094005	Neonatal diabetes, congenital hypothyroidism, congenital glaucoma, hepatic fibrosis, polycystic kidney syndrome (disorder)
Congenital Disease	SNOMED	724097003	Moyamoya angiopathy, short stature, facial dysmorphism, hypergonadotropic hypogonadism syndrome (disorder)
Congenital Disease	SNOMED	724098008	Monosomy 9q22.3 syndrome (disorder)
Congenital Disease	SNOMED	724137002	Macrocephaly, obesity, mental disability, ocular abnormality syndrome (disorder)
Congenital Disease	SNOMED	724139004	Microtia, eye coloboma, imperforation of nasolacrimal duct syndrome (disorder)
Congenital Disease	SNOMED	724140002	Microspherophakia with metaphyseal dysplasia syndrome (disorder)
Congenital Disease	SNOMED	724141003	Microcephalic primordial dwarfism due to zinc finger protein 335 deficiency (disorder)
Congenital Disease	SNOMED	724144006	Embryofetopathy caused by methimazole (disorder)
Congenital Disease	SNOMED	724145007	Metaphyseal dysplasia, maxillary hypoplasia, brachydactyly syndrome (disorder)
Congenital Disease	SNOMED	724146008	Metaphyseal chondromatosis co-occurrent with D-2 hydroxyglutaric aciduria (disorder)
Congenital Disease	SNOMED	724147004	8q13 microdeletion syndrome (disorder)
Congenital Disease	SNOMED	724170007	Mesoaxial synostotic syndactyly with phalangeal reduction syndrome (disorder)
Congenital Disease	SNOMED	724174003	Moebius syndrome, axonal neuropathy, hypogonadotropic hypogonadism syndrome (disorder)
Congenital Disease	SNOMED	724206005	Keratin 14 related epidermolysis bullosa simplex (disorder)
Congenital Disease	SNOMED	724207001	Kleefstra syndrome (disorder)
Congenital Disease	SNOMED	724208006	Keutel syndrome (disorder)
Congenital Disease	SNOMED	724212000	Congenital mallet toe (disorder)
Congenital Disease	SNOMED	724224007	Palmoplantar keratoderma with clinodactyly syndrome (disorder)
Congenital Disease	SNOMED	724225008	Junctional epidermolysis bullosa non-Herlitz type (disorder)
Congenital Disease	SNOMED	724226009	Infantile osteopetrosis with neuroaxonal dysplasia syndrome (disorder)
Congenital Disease	SNOMED	72424001	Congenital absence of diaphragm (disorder)
Congenital Disease	SNOMED	72425000	Bilateral left-sidedness sequence (disorder)
Congenital Disease	SNOMED	724277002	Congenital ichthyosis with hypotrichosis syndrome (disorder)
Congenital Disease	SNOMED	724278007	Neonatal sclerosing cholangitis, ichthyosis, hypotrichosis syndrome (disorder)
Congenital Disease	SNOMED	724281002	Hyposmia, nasal and ocular hypoplasia, hypogonadotropic hypogonadism syndrome (disorder)
Congenital Disease	SNOMED	724284005	Hypertelorism Teebi type (disorder)
Congenital Disease	SNOMED	724349009	Hereditary inclusion body myopathy, joint contracture, ophthalmoplegia syndrome (disorder)
Congenital Disease	SNOMED	724350009	Hereditary hypotrichosis with recurrent skin vesicles syndrome (disorder)
Congenital Disease	SNOMED	724435004	Congenital anomaly of descending thoracic aorta (disorder)
Congenital Disease	SNOMED	724436003	Congenital anomaly of abdominal aorta (disorder)
Congenital Disease	SNOMED	724437007	Compression of trachea and esophagus co-occurrent and due to congenital anomaly of aortic arch (disorder)
Congenital Disease	SNOMED	724469003	Congenital secondary hydronephrosis (disorder)
Congenital Disease	SNOMED	724513009	Congenital aplasia of lacrimal structure (disorder)
Congenital Disease	SNOMED	724615008	Congenital J shaped sella turcica (disorder)
Congenital Disease	SNOMED	724616009	Occipitalization of atlas (disorder)
Congenital Disease	SNOMED	724617000	Congenital wide symphysis pubis (disorder)
Congenital Disease	SNOMED	724618005	Congenital club finger (disorder)
Congenital Disease	SNOMED	724619002	Congenital radial deviation of finger (disorder)
Congenital Disease	SNOMED	724643004	Transient abnormal myelopoiesis co-occurrent with Down syndrome (disorder)
Congenital Disease	SNOMED	724644005	Myeloid leukemia co-occurrent with Down syndrome (disorder)
Congenital Disease	SNOMED	724837004	Keratinopathic ichthyosis (disorder)
Congenital Disease	SNOMED	724838009	Hereditary skin peeling syndrome (disorder)
Congenital Disease	SNOMED	724840004	Suprabasal epidermolysis bullosa simplex (disorder)
Congenital Disease	SNOMED	724842007	Congenital venous malformation of skin (disorder)
Congenital Disease	SNOMED	724999003	Isolated optic nerve hypoplasia (disorder)
Congenital Disease	SNOMED	725029001	Frontonasal dysplasia with alopecia and genital anomaly syndrome (disorder)
Congenital Disease	SNOMED	725030006	Familial scaphocephaly syndrome McGillivray type (disorder)
Congenital Disease	SNOMED	725045004	46,XY partial gonadal dysgenesis (disorder)
Congenital Disease	SNOMED	725050005	Autosomal dominant osteopetrosis type 2 (disorder)
Congenital Disease	SNOMED	725084009	Sex chromosome aneuploidy (disorder)
Congenital Disease	SNOMED	725096002	Cryptomicrotia brachydactyly syndrome (disorder)
Congenital Disease	SNOMED	725098001	Craniomicromelic syndrome (disorder)
Congenital Disease	SNOMED	725099009	Craniometadiaphyseal dysplasia wormian bone type (disorder)
Congenital Disease	SNOMED	725100001	Craniolenticulosutural dysplasia (disorder)

Congenital Disease	SNOMED	725101002	Congenital short costocoracoid ligament (disorder)
Congenital Disease	SNOMED	725104005	Cheirospondyloenchondromatosis (disorder)
Congenital Disease	SNOMED	725138002	Perineal hemangioma, external genitalia malformation, lipomyelomeningocele, vesicorenal abnormality, imperforate anus syndrome (disorder)
Congenital Disease	SNOMED	725140007	Temple Baraitser syndrome (disorder)
Congenital Disease	SNOMED	725141006	Atelosteogenesis type 1 (disorder)
Congenital Disease	SNOMED	725142004	Atelosteogenesis type 3 (disorder)
Congenital Disease	SNOMED	725145002	Atrial septal defect, atrioventricular conduction defect syndrome (disorder)
Congenital Disease	SNOMED	725149008	Auricular abnormality, cleft lip, ocular abnormality syndrome (disorder)
Congenital Disease	SNOMED	725164008	Omodysplasia (disorder)
Congenital Disease	SNOMED	725165009	Autosomal dominant omodysplasia (disorder)
Congenital Disease	SNOMED	725166005	Autosomal recessive omodysplasia (disorder)
Congenital Disease	SNOMED	72523005	X-linked ichthyosis with steryl-sulfatase deficiency (disorder)
Congenital Disease	SNOMED	725287006	Embryopathy caused by retinoid (disorder)
Congenital Disease	SNOMED	72535009	Cervical rib (disorder)
Congenital Disease	SNOMED	725407006	Recessive dystrophic epidermolysis bullosa non-Hallopeau Siemens type (disorder)
Congenital Disease	SNOMED	725409009	Polydactyly of biphalangeal thumb (disorder)
Congenital Disease	SNOMED	725417001	Carbohydrate sulfotransferase 3 related skeletal dysplasia (disorder)
Congenital Disease	SNOMED	725419003	Centripetalis recessive dystrophic epidermolysis bullosa (disorder)
Congenital Disease	SNOMED	725434009	Autosomal recessive faciodigitogenital syndrome (disorder)
Congenital Disease	SNOMED	725461009	Microcephalic osteodysplastic primordial dwarfism types I and III (disorder)
Congenital Disease	SNOMED	725588002	Bathing suit ichthyosis (disorder)
Congenital Disease	SNOMED	725589005	Bullous dystrophy macular type (disorder)
Congenital Disease	SNOMED	725904009	Genochondromatosis type 2 (disorder)
Congenital Disease	SNOMED	725905005	Infundibulopelvic stenosis multicystic kidney syndrome (disorder)
Congenital Disease	SNOMED	725906006	Intellectual disability Buenos Aires type (disorder)
Congenital Disease	SNOMED	725908007	Neurofaciodigitorenal syndrome (disorder)
Congenital Disease	SNOMED	725910009	Congenital duplication of rectum (disorder)
Congenital Disease	SNOMED	725911008	Pierre Robin sequence, congenital heart defect, talipes syndrome (disorder)
Congenital Disease	SNOMED	725912001	X-linked intellectual disability Brooks type (disorder)
Congenital Disease	SNOMED	726017001	Mucin 1 related autosomal dominant tubulointerstitial kidney disease (disorder)
Congenital Disease	SNOMED	726018006	Autosomal dominant tubulointerstitial kidney disease (disorder)
Congenital Disease	SNOMED	726029005	McCune Albright syndrome (disorder)
Congenital Disease	SNOMED	726031001	Cerebellar ataxia, intellectual disability, optic atrophy, skin abnormalities syndrome (disorder)
Congenital Disease	SNOMED	726032008	Short rib polydactyly syndrome type I (disorder)
Congenital Disease	SNOMED	726083008	Congenital sacral meningocele with conotruncal heart defect syndrome (disorder)
Congenital Disease	SNOMED	726106004	X-linked diffuse leiomyomatosis with Alport syndrome (disorder)
Congenital Disease	SNOMED	726116007	Congenital anomaly of bone of shoulder girdle (disorder)
Congenital Disease	SNOMED	726117003	Congenital deformity of toe (disorder)
Congenital Disease	SNOMED	726334003	Congenital dacryocele (disorder)
Congenital Disease	SNOMED	726335002	Common atrioventricular junction (disorder)
Congenital Disease	SNOMED	726338000	Partial trisomy of chromosome 1 (disorder)
Congenital Disease	SNOMED	726339008	Partial trisomy of short arm of chromosome 1 (disorder)
Congenital Disease	SNOMED	726340005	Partial trisomy of chromosome 2 (disorder)
Congenital Disease	SNOMED	726341009	Partial trisomy of chromosome 3 (disorder)
Congenital Disease	SNOMED	726342002	Partial trisomy of chromosome 4 (disorder)
Congenital Disease	SNOMED	726343007	Partial trisomy of chromosome 5 (disorder)
Congenital Disease	SNOMED	726344001	Partial trisomy of long arm of chromosome 5 (disorder)
Congenital Disease	SNOMED	726345000	Partial trisomy of chromosome 6 (disorder)
Congenital Disease	SNOMED	726346004	Partial trisomy of chromosome 7 (disorder)
Congenital Disease	SNOMED	726347008	Partial trisomy of chromosome 8 (disorder)
Congenital Disease	SNOMED	726348003	Partial trisomy of chromosome 9 (disorder)
Congenital Disease	SNOMED	726349006	Partial trisomy of chromosome 10 (disorder)
Congenital Disease	SNOMED	726350006	Partial trisomy of chromosome 11 (disorder)
Congenital Disease	SNOMED	726351005	Partial trisomy of chromosome 12 (disorder)
Congenital Disease	SNOMED	726352003	Partial trisomy of chromosome 13 (disorder)
Congenital Disease	SNOMED	726353008	Partial trisomy of chromosome 14 (disorder)
Congenital Disease	SNOMED	726354002	Partial trisomy of chromosome 15 (disorder)
Congenital Disease	SNOMED	726355001	Partial trisomy of chromosome 16 (disorder)
Congenital Disease	SNOMED	726356000	Partial trisomy of chromosome 17 (disorder)
Congenital Disease	SNOMED	726357009	Partial trisomy of chromosome 18 (disorder)
Congenital Disease	SNOMED	726358004	Partial trisomy of chromosome 19 (disorder)
Congenital Disease	SNOMED	726359007	Partial trisomy of short arm of chromosome 19 (disorder)
Congenital Disease	SNOMED	726360002	Partial trisomy of chromosome 20 (disorder)
Congenital Disease	SNOMED	726361003	Partial trisomy of chromosome 21 (disorder)
Congenital Disease	SNOMED	726362005	Partial trisomy of chromosome 22 (disorder)
Congenital Disease	SNOMED	726363000	Tetraploidy (disorder)
Congenital Disease	SNOMED	726364006	Complete monosomy of autosome (disorder)
Congenital Disease	SNOMED	726365007	Deletion of part of chromosome 1 (disorder)
Congenital Disease	SNOMED	726366008	Deletion of part of chromosome 2 (disorder)

Congenital Disease	SNOMED	726367004	Deletion of part of long arm of chromosome 2 (disorder)
Congenital Disease	SNOMED	726368009	Deletion of part of short arm of chromosome 2 (disorder)
Congenital Disease	SNOMED	726369001	Deletion of part of chromosome 3 (disorder)
Congenital Disease	SNOMED	726370000	Deletion of part of long arm of chromosome 3 (disorder)
Congenital Disease	SNOMED	726371001	Deletion of part of chromosome 4 (disorder)
Congenital Disease	SNOMED	726372008	Deletion of part of chromosome 5 (disorder)
Congenital Disease	SNOMED	726373003	Deletion of part of long arm of chromosome 5 (disorder)
Congenital Disease	SNOMED	726374009	Deletion of part of chromosome 6 (disorder)
Congenital Disease	SNOMED	726375005	Deletion of part of long arm of chromosome 6 (disorder)
Congenital Disease	SNOMED	726376006	Deletion of part of short arm of chromosome 6 (disorder)
Congenital Disease	SNOMED	726377002	Deletion of part of chromosome 7 (disorder)
Congenital Disease	SNOMED	726378007	Deletion of part of chromosome 8 (disorder)
Congenital Disease	SNOMED	726379004	Deletion of part of chromosome 9 (disorder)
Congenital Disease	SNOMED	726380001	Deletion of part of chromosome 10 (disorder)
Congenital Disease	SNOMED	726381002	Deletion of part of chromosome 11 (disorder)
Congenital Disease	SNOMED	726382009	Deletion of part of chromosome 12 (disorder)
Congenital Disease	SNOMED	726383004	Deletion of part of long arm of chromosome 12 (disorder)
Congenital Disease	SNOMED	726384005	Deletion of part of chromosome 13 (disorder)
Congenital Disease	SNOMED	726385006	Deletion of part of chromosome 14 (disorder)
Congenital Disease	SNOMED	726386007	Deletion of part of chromosome 15 (disorder)
Congenital Disease	SNOMED	726387003	Deletion of part of chromosome 16 (disorder)
Congenital Disease	SNOMED	726388008	Deletion of part of short arm of chromosome 16 (disorder)
Congenital Disease	SNOMED	726389000	Deletion of part of chromosome 17 (disorder)
Congenital Disease	SNOMED	726390009	Deletion of part of short arm of chromosome 17 (disorder)
Congenital Disease	SNOMED	726391008	Deletion of part of chromosome 18 (disorder)
Congenital Disease	SNOMED	726392001	Deletion of part of chromosome 19 (disorder)
Congenital Disease	SNOMED	726393006	Deletion of long arm of chromosome 19 (disorder)
Congenital Disease	SNOMED	726394000	Deletion of short arm of chromosome 19 (disorder)
Congenital Disease	SNOMED	726395004	Deletion of part of chromosome 20 (disorder)
Congenital Disease	SNOMED	726396003	Deletion of part of long arm of chromosome 20 (disorder)
Congenital Disease	SNOMED	726397007	Deletion of part of short arm of chromosome 20 (disorder)
Congenital Disease	SNOMED	726398002	Deletion of part of chromosome 21 (disorder)
Congenital Disease	SNOMED	726399005	Deletion of part of chromosome 22 (disorder)
Congenital Disease	SNOMED	726400003	Uniparental disomy (disorder)
Congenital Disease	SNOMED	726401004	Uniparental disomy of maternal origin (disorder)
Congenital Disease	SNOMED	726402006	Uniparental disomy of paternal origin (disorder)
Congenital Disease	SNOMED	726406009	Congenital malposition of eyelid (disorder)
Congenital Disease	SNOMED	726407000	Dystopia canthorum (disorder)
Congenital Disease	SNOMED	72645004	Uterus bicornis bicollis (disorder)
Congenital Disease	SNOMED	726619004	Ptosis, strabismus, ectopic pupil syndrome (disorder)
Congenital Disease	SNOMED	726620005	Arthrogryposis hyperkeratosis syndrome lethal form (disorder)
Congenital Disease	SNOMED	726621009	Caudal appendage deafness syndrome (disorder)
Congenital Disease	SNOMED	726629006	Scalp defect postaxial polydactyly syndrome (disorder)
Congenital Disease	SNOMED	726670008	Weaver Williams syndrome (disorder)
Congenital Disease	SNOMED	726672000	Short stature, unique facies, enamel hypoplasia, progressive joint stiffness, high-pitched voice syndrome (disorder)
Congenital Disease	SNOMED	726703000	Digestive duplication cyst of tongue (disorder)
Congenital Disease	SNOMED	726704006	Cataract, congenital heart disease, neural tube defect syndrome (disorder)
Congenital Disease	SNOMED	726705007	3q13 microdeletion syndrome (disorder)
Congenital Disease	SNOMED	726706008	4p16.3 microduplication syndrome (disorder)
Congenital Disease	SNOMED	726707004	7q11.23 microduplication syndrome (disorder)
Congenital Disease	SNOMED	726708009	Familial isolated congenital asplenia (disorder)
Congenital Disease	SNOMED	726709001	Intellectual disability, cataract, calcified pinna, myopathy syndrome (disorder)
Congenital Disease	SNOMED	726722009	Hemifacial microsomia with radial defect syndrome (disorder)
Congenital Disease	SNOMED	726723004	Ring chromosome 13 syndrome (disorder)
Congenital Disease	SNOMED	726724005	Splenogonadal fusion, limb defect, micrognathia syndrome (disorder)
Congenital Disease	SNOMED	726732002	X-linked intellectual disability Nascimento type (disorder)
Congenital Disease	SNOMED	726733007	Chromosome Xp22.3 microdeletion syndrome (disorder)
Congenital Disease	SNOMED	726734001	Short stature locking fingers syndrome (disorder)
Congenital Disease	SNOMED	726735000	Autosomal recessive amelia (disorder)
Congenital Disease	SNOMED	72744008	Gronblad-Strandberg syndrome (disorder)
Congenital Disease	SNOMED	72881003	Megaloappendix (disorder)
Congenital Disease	SNOMED	72913007	Moore-Federman syndrome (disorder)
Congenital Disease	SNOMED	72922008	Short rib-polydactyly syndrome, Majewski type (disorder)
Congenital Disease	SNOMED	72925005	Congenital cystic disease of liver (disorder)
Congenital Disease	SNOMED	72948000	Congenital anomaly of epiglottis (disorder)
Congenital Disease	SNOMED	72951007	Gastroschisis (disorder)
Congenital Disease	SNOMED	72991005	Polyploidy syndrome (disorder)
Congenital Disease	SNOMED	73035005	10q partial trisomy syndrome (disorder)
Congenital Disease	SNOMED	7305005	Coarctation of aorta (disorder)
Congenital Disease	SNOMED	7322007	Accessory adrenal cortex (disorder)
Congenital Disease	SNOMED	732247000	Cleft lip retinopathy syndrome (disorder)
Congenital Disease	SNOMED	732248005	Coxoauricular syndrome (disorder)
Congenital Disease	SNOMED	732249002	Bone dysplasia lethal Holmgren type (disorder)

Congenital Disease	SNOMED	732250002	Craniosynostosis fibular aplasia syndrome (disorder)
Congenital Disease	SNOMED	732251003	Cortical blindness, intellectual disability, polydactyly syndrome (disorder)
Congenital Disease	SNOMED	732259001	Distal monosomy 17q (disorder)
Congenital Disease	SNOMED	732261005	Cyprus facial neuromusculoskeletal syndrome (disorder)
Congenital Disease	SNOMED	732262003	Marfanoid syndrome De Silva type (disorder)
Congenital Disease	SNOMED	732263008	Melhem Fahl syndrome (disorder)
Congenital Disease	SNOMED	73284007	Marshall-Smith syndrome (disorder)
Congenital Disease	SNOMED	73291005	Congenital absence of parathyroid gland (disorder)
Congenital Disease	SNOMED	732926009	Hydrocephalus, tall stature, joint laxity syndrome (disorder)
Congenital Disease	SNOMED	732927000	Split hand, obstructive uropathy, spina bifida, diaphragmatic defect syndrome (disorder)
Congenital Disease	SNOMED	732928005	Aplasia of trochlea of humerus (disorder)
Congenital Disease	SNOMED	732950006	Ichthyosis, oral and digital anomalies syndrome (disorder)
Congenital Disease	SNOMED	732952003	Congenital cataract ichthyosis syndrome (disorder)
Congenital Disease	SNOMED	732953008	Ectodermal dysplasia and sensorineural deafness syndrome (disorder)
Congenital Disease	SNOMED	732954002	Osteopenia, intellectual disability, sparse hair syndrome (disorder)
Congenital Disease	SNOMED	732955001	Symphalangism with multiple anomalies of hands and feet syndrome (disorder)
Congenital Disease	SNOMED	732956000	Brachydactyly and distal symphalangism syndrome (disorder)
Congenital Disease	SNOMED	732957009	Brachydactyly and preaxial hallux varus syndrome (disorder)
Congenital Disease	SNOMED	732961003	Branchial dysplasia, intellectual disability, inguinal hernia syndrome (disorder)
Congenital Disease	SNOMED	733028000	Multiple sclerosis, ichthyosis, factor VIII deficiency syndrome (disorder)
Congenital Disease	SNOMED	733030003	Congenital hypoplasia of ulna and split foot syndrome (disorder)
Congenital Disease	SNOMED	733031004	Epilepsy, microcephaly, skeletal dysplasia syndrome (disorder)
Congenital Disease	SNOMED	733034007	Charlie M syndrome (disorder)
Congenital Disease	SNOMED	733037000	German syndrome (disorder)
Congenital Disease	SNOMED	733038005	Dysmorphism, pectus carinatum, joint laxity syndrome (disorder)
Congenital Disease	SNOMED	733045005	Camptobrachydactyly (disorder)
Congenital Disease	SNOMED	733046006	Hemifacial hyperplasia strabismus syndrome (disorder)
Congenital Disease	SNOMED	733049004	Encephalopathy, intracerebral calcification, retinal degeneration syndrome (disorder)
Congenital Disease	SNOMED	733050004	Dysmorphism, short stature, deafness, disorder of sex development syndrome (disorder)
Congenital Disease	SNOMED	733062000	Marfanoid habitus with autosomal recessive intellectual disability syndrome (disorder)
Congenital Disease	SNOMED	733064004	Osteosarcoma, limb anomalies, erythroid macrocytosis syndrome (disorder)
Congenital Disease	SNOMED	733066002	Trigonocephaly, short stature, developmental delay syndrome (disorder)
Congenital Disease	SNOMED	733067006	Telecanthus, hypertelorism, strabismus, pes cavus syndrome (disorder)
Congenital Disease	SNOMED	733068001	Absent tibia, polydactyly, arachnoid cyst syndrome (disorder)
Congenital Disease	SNOMED	733069009	Deafness, vitiligo, achalasia syndrome (disorder)
Congenital Disease	SNOMED	733070005	Duplication of eyebrow and syndactyly syndrome (disorder)
Congenital Disease	SNOMED	733072002	Alaninuria, microcephaly, dwarfism, enamel hypoplasia, diabetes mellitus syndrome (disorder)
Congenital Disease	SNOMED	733086003	Pseudoprogeria syndrome (disorder)
Congenital Disease	SNOMED	733087007	Polydactyly myopia syndrome (disorder)
Congenital Disease	SNOMED	733088002	Preaxial polydactyly, colobomata, intellectual disability syndrome (disorder)
Congenital Disease	SNOMED	733091002	Isolated hereditary congenital facial paralysis (disorder)
Congenital Disease	SNOMED	733092009	Microcephalus, hypergonadotropic hypogonadism, short stature syndrome (disorder)
Congenital Disease	SNOMED	733093004	Banki syndrome (disorder)
Congenital Disease	SNOMED	733094005	Dandy-Walker malformation with postaxial polydactyly syndrome (disorder)
Congenital Disease	SNOMED	733095006	Skeletal dysplasia brachydactyly syndrome (disorder)
Congenital Disease	SNOMED	733096007	Thyrocerebrorenal syndrome (disorder)
Congenital Disease	SNOMED	733097003	Ichthyosis, intellectual disability, dwarfism, renal impairment syndrome (disorder)
Congenital Disease	SNOMED	733110004	Van den Bosch syndrome (disorder)
Congenital Disease	SNOMED	733116005	Aniridia, renal agenesis, psychomotor retardation syndrome (disorder)
Congenital Disease	SNOMED	733117001	Thumb stiffness, brachydactyly, intellectual disability syndrome (disorder)
Congenital Disease	SNOMED	733118006	Aphalangy, hemivertebra, urogenital, intestinal dysgenesis syndrome (disorder)
Congenital Disease	SNOMED	733194007	Dementia co-occurrent and due to Down syndrome (disorder)
Congenital Disease	SNOMED	733300002	Deletion of part of long arm of chromosome 17 (disorder)
Congenital Disease	SNOMED	733302005	Congenital cyst of orbit (disorder)
Congenital Disease	SNOMED	73331006	Hemimyelia (disorder)
Congenital Disease	SNOMED	733416004	Exostosis, anetoderma, brachydactyly type E syndrome (disorder)
Congenital Disease	SNOMED	733417008	Facial dysmorphism, macrocephaly, myopia, Dandy-Walker malformation syndrome (disorder)
Congenital Disease	SNOMED	733418003	Joubert syndrome with Jeune asphyxiating thoracic dystrophy (disorder)
Congenital Disease	SNOMED	733419006	Metaphyseal dysostosis, intellectual disability, conductive deafness syndrome (disorder)
Congenital Disease	SNOMED	733425005	Acrocephalopolysyndactyly type IV (disorder)
Congenital Disease	SNOMED	733453005	Congenital nephrotic syndrome, interstitial lung disease, epidermolysis bullosa syndrome (disorder)
Congenital Disease	SNOMED	733454004	Long thumb brachydactyly syndrome (disorder)

Congenital Disease	SNOMED	733455003	Spastic paraplegia, glaucoma, intellectual disability syndrome (disorder)
Congenital Disease	SNOMED	733456002	Triphalangeal thumb and dislocation of patella syndrome (disorder)
Congenital Disease	SNOMED	733457006	Ehlers-Danlos and osteogenesis imperfecta syndrome (disorder)
Congenital Disease	SNOMED	733466005	Camptodactyly taurinuria syndrome (disorder)
Congenital Disease	SNOMED	733468006	Skeletal dysplasia with wormian bone, multiple fractures, dentinogenesis imperfecta syndrome (disorder)
Congenital Disease	SNOMED	733469003	Hereditary congenital hypomelanotic and hypermelanotic cutaneous macules, growth retardation, intellectual disability syndrome (disorder)
Congenital Disease	SNOMED	733472005	Microcephalus, glomerulonephritis, marfanoid habitus syndrome (disorder)
Congenital Disease	SNOMED	733473000	16p13.3 microduplication syndrome (disorder)
Congenital Disease	SNOMED	733518000	16p11.2p12.2 microduplication syndrome (disorder)
Congenital Disease	SNOMED	733519008	17q12 microdeletion syndrome (disorder)
Congenital Disease	SNOMED	733520002	20q13.33 microdeletion syndrome (disorder)
Congenital Disease	SNOMED	733521003	Distal 16p11.2 microdeletion syndrome (disorder)
Congenital Disease	SNOMED	733522005	Megalocornea with intellectual disability syndrome (disorder)
Congenital Disease	SNOMED	733598001	Acute myeloid leukemia with t(6;9)(p23;q34) translocation (disorder)
Congenital Disease	SNOMED	733604003	Microcephalus, lymphedema, chorioretinopathy syndrome (disorder)
Congenital Disease	SNOMED	733605002	XY type gonadal dysgenesis with associated anomalies syndrome (disorder)
Congenital Disease	SNOMED	733606001	Summitt syndrome (disorder)
Congenital Disease	SNOMED	733621007	46,XX disorder of sex development with skeletal anomalies syndrome (disorder)
Congenital Disease	SNOMED	733622000	46,XX disorder of sex development with anorectal anomalies syndrome (disorder)
Congenital Disease	SNOMED	733623005	Autism spectrum disorder, epilepsy, arthrogryposis syndrome (disorder)
Congenital Disease	SNOMED	733625003	48,XYYY syndrome (disorder)
Congenital Disease	SNOMED	733626002	Atypical Norrie disease due to monosomy Xp11.3 (disorder)
Congenital Disease	SNOMED	733628001	Thoraco-abdominal enteric duplication (disorder)
Congenital Disease	SNOMED	733638006	Acral dystrophic epidermolysis bullosa (disorder)
Congenital Disease	SNOMED	733650000	Adult familial nephronophthisis with spastic quadriparesia syndrome (disorder)
Congenital Disease	SNOMED	73371000119103	First branchial cleft cyst (disorder)
Congenital Disease	SNOMED	73381000119100	Second branchial cleft cyst (disorder)
Congenital Disease	SNOMED	73391000119102	Third branchial cleft cyst (disorder)
Congenital Disease	SNOMED	734001	Opocephalus (disorder)
Congenital Disease	SNOMED	734016004	17p11.2 microduplication syndrome (disorder)
Congenital Disease	SNOMED	734017008	Ectodermal dysplasia, intellectual disability, central nervous system malformation syndrome (disorder)
Congenital Disease	SNOMED	734018003	Ectodermal dysplasia trichoodontoonychial type (disorder)
Congenital Disease	SNOMED	734024009	Intermediate anorectal malformation (disorder)
Congenital Disease	SNOMED	734026006	Isolated congenital megalocornea (disorder)
Congenital Disease	SNOMED	734028007	49,XYYYY syndrome (disorder)
Congenital Disease	SNOMED	734029004	Distal 22q11.2 microdeletion syndrome (disorder)
Congenital Disease	SNOMED	734030009	12q15q21.1 microdeletion syndrome (disorder)
Congenital Disease	SNOMED	734173003	Skeletal abnormality, cutis laxa, craniostenosis, ambiguous genitalia, retardation, facial abnormality syndrome (disorder)
Congenital Disease	SNOMED	734477008	Congenital hydrothorax (disorder)
Congenital Disease	SNOMED	73465006	Congenital absence of germinal epithelium of testes (disorder)
Congenital Disease	SNOMED	735105001	Elongation of left ramus of mandible (disorder)
Congenital Disease	SNOMED	735107009	Elongation of right ramus of mandible (disorder)
Congenital Disease	SNOMED	735719008	Neonatal intestinal perforation co-occurrent and due to intestinal atresia (disorder)
Congenital Disease	SNOMED	735721003	Neonatal intestinal perforation due to in utero intestinal volvulus (disorder)
Congenital Disease	SNOMED	735722005	Neonatal intestinal perforation co-occurrent and due to in utero intraluminal obstruction (disorder)
Congenital Disease	SNOMED	73573004	Congenital anomaly of musculoskeletal system (disorder)
Congenital Disease	SNOMED	735749005	Myelomeningocele co-occurrent with hydrocephalus (disorder)
Congenital Disease	SNOMED	73660006	Congenital subaortic stenosis (disorder)
Congenital Disease	SNOMED	73663008	Neurologic xeroderma pigmentosum (disorder)
Congenital Disease	SNOMED	736781007	Congenital absence of right mandibular condyle (disorder)
Congenital Disease	SNOMED	736782000	Congenital absence of left mandibular condyle (disorder)
Congenital Disease	SNOMED	7368005	Double outlet left ventricle (disorder)
Congenital Disease	SNOMED	73699003	Common arterial trunk and common origin of pulmonary arteries (disorder)
Congenital Disease	SNOMED	737037004	Phosphatidylinositol-4,5-bisphosphate 3-kinase catalytic subunit alpha related overgrowth syndrome (disorder)
Congenital Disease	SNOMED	737155005	Congenital anomaly of atrioventricular valve (disorder)
Congenital Disease	SNOMED	737156006	Congenital anomaly of atrioventricular septum (disorder)
Congenital Disease	SNOMED	73716000	Congenital cutaneous angiomatosis (disorder)
Congenital Disease	SNOMED	737185000	Congenital hyperplasia of lung (disorder)
Congenital Disease	SNOMED	737197004	Congenital stenosis of large intestine (disorder)
Congenital Disease	SNOMED	737202006	Fibropolycystic disease of liver (disorder)
Congenital Disease	SNOMED	737215002	Congenital hypoplasia of odontoid process of axis (disorder)
Congenital Disease	SNOMED	737216001	Hypoplasia of sacrum (disorder)
Congenital Disease	SNOMED	737217005	Congenital absence of forearm and hand (disorder)
Congenital Disease	SNOMED	737219008	Aplasia of patella (disorder)
Congenital Disease	SNOMED	737266007	Longitudinal deficiency of upper and lower limbs (disorder)

Congenital Disease	SNOMED	737562008	Multicystic renal dysplasia (disorder)
Congenital Disease	SNOMED	737579002	Congenital coloboma of macula lutea (disorder)
Congenital Disease	SNOMED	737581000	Tibio-fibular synostosis (disorder)
Congenital Disease	SNOMED	738164003	Lop ear deformity (disorder)
Congenital Disease	SNOMED	73856006	Cutis laxa with osteodystrophy (disorder)
Congenital Disease	SNOMED	73943003	Congenital malposition of digestive organs (disorder)
Congenital Disease	SNOMED	74008005	Partial trisomy of short arm of chromosome 5 (disorder)
Congenital Disease	SNOMED	74012004	Congenital anomaly of pituitary gland (disorder)
Congenital Disease	SNOMED	74183001	Partial tetrasomy of chromosome 9 (disorder)
Congenital Disease	SNOMED	74218008	Coronary artery arising from main pulmonary artery (disorder)
Congenital Disease	SNOMED	74223008	Congenital scar (disorder)
Congenital Disease	SNOMED	74245009	Congenital absence of fibula (disorder)
Congenital Disease	SNOMED	742876007	Peroxisome biogenesis disorder (disorder)
Congenital Disease	SNOMED	74307005	Opodidymus (disorder)
Congenital Disease	SNOMED	74345006	Congenital disorder due to abnormality of chromosome number OR structure (disorder)
Congenital Disease	SNOMED	74350000	Complete trisomy 9 syndrome (disorder)
Congenital Disease	SNOMED	74370006	Micromelia (disorder)
Congenital Disease	SNOMED	7438000	Congenital atresia of aorta (disorder)
Congenital Disease	SNOMED	74398009	XX males (disorder)
Congenital Disease	SNOMED	74516008	Craniopagus (disorder)
Congenital Disease	SNOMED	74561007	Kommerell's diverticulum (disorder)
Congenital Disease	SNOMED	7458004	Diplopodia (disorder)
Congenital Disease	SNOMED	74594005	Crossed renal ectopia (disorder)
Congenital Disease	SNOMED	74622009	Congenital short hard palate (disorder)
Congenital Disease	SNOMED	74650009	Jugular lymphatic obstruction sequence (disorder)
Congenital Disease	SNOMED	74769007	Anomaly of chromosome pair 1 (disorder)
Congenital Disease	SNOMED	74788000	Tongue absent (disorder)
Congenital Disease	SNOMED	7481002	Congenital atresia of epiglottis (disorder)
Congenital Disease	SNOMED	74820003	Congenital coxa vara (disorder)
Congenital Disease	SNOMED	74829002	Persistent cloaca (disorder)
Congenital Disease	SNOMED	7484005	Double outlet right ventricle (disorder)
Congenital Disease	SNOMED	74877002	Congenital anomaly of spine (disorder)
Congenital Disease	SNOMED	74908007	Congenital absence of inferior vena cava (disorder)
Congenital Disease	SNOMED	74911008	Dyskeratosis congenita (disorder)
Congenital Disease	SNOMED	74919005	Congenital stricture of ureter (disorder)
Congenital Disease	SNOMED	74928006	Camptomelic dysplasia (disorder)
Congenital Disease	SNOMED	74969002	Congenital ectopic lens (disorder)
Congenital Disease	SNOMED	75049004	Jeune thoracic dystrophy (disorder)
Congenital Disease	SNOMED	75076004	Amyelencephalus (disorder)
Congenital Disease	SNOMED	75145007	Stricture of aorta (disorder)
Congenital Disease	SNOMED	75164001	Pseudohermaphroditism (disorder)
Congenital Disease	SNOMED	75231006	Congenital absence of eustachian tube (disorder)
Congenital Disease	SNOMED	75270000	Congenital diverticulum of left ventricle (disorder)
Congenital Disease	SNOMED	75311005	Congenital absence of ossicles of ear (disorder)
Congenital Disease	SNOMED	75340008	Congenital keratoconus posticus circumscriptus (disorder)
Congenital Disease	SNOMED	75355004	Congenital atresia of external auditory canal (disorder)
Congenital Disease	SNOMED	75372006	Congenital anomaly of mitral valve (disorder)
Congenital Disease	SNOMED	75398000	Anomalous origin of coronary artery (disorder)
Congenital Disease	SNOMED	75474006	Congenital absence of nipple (disorder)
Congenital Disease	SNOMED	75491005	Amyotrophia congenita (disorder)
Congenital Disease	SNOMED	75511006	Congenital deformity of chest wall (disorder)
Congenital Disease	SNOMED	75617000	Omphalopagus (disorder)
Congenital Disease	SNOMED	75633002	Congenital anoperineal fistula (disorder)
Congenital Disease	SNOMED	75654009	Benign autosomal dominant osteopetrosis (disorder)
Congenital Disease	SNOMED	75667007	Lingual goiter (disorder)
Congenital Disease	SNOMED	7586009	14q partial trisomy (disorder)
Congenital Disease	SNOMED	75875004	Dominant dystrophic epidermolysis bullosa, albopapular type (disorder)
Congenital Disease	SNOMED	7589002	Brachymegalodactyly (disorder)
Congenital Disease	SNOMED	75893003	19q partial trisomy syndrome (disorder)
Congenital Disease	SNOMED	75922002	Congenital anomaly of ossicles of ear (disorder)
Congenital Disease	SNOMED	75937003	Congenital absence of anus (disorder)
Congenital Disease	SNOMED	75950002	Brachymetacarpia (disorder)
Congenital Disease	SNOMED	75968004	Sotos' syndrome (disorder)
Congenital Disease	SNOMED	75979009	Johanson-Blizzard syndrome (disorder)
Congenital Disease	SNOMED	7601009	Double urinary meatus (disorder)
Congenital Disease	SNOMED	7603007	Platyspondylia (disorder)
Congenital Disease	SNOMED	76074006	Agenesis of right lung (disorder)
Congenital Disease	SNOMED	76089002	Symmetrical conjoined twins (disorder)
Congenital Disease	SNOMED	7611002	Septo-optic dysplasia sequence (disorder)
Congenital Disease	SNOMED	762195006	Congenital bronchogenic cyst (disorder)
Congenital Disease	SNOMED	762228008	Congenital cardiovascular disorder (disorder)
Congenital Disease	SNOMED	76223006	Bat ear (disorder)
Congenital Disease	SNOMED	762250009	Congenital anomaly of great vessel (disorder)

Congenital Disease	SNOMED	762251008	Double outlet right ventricle with subaortic or doubly committed ventricular septal defect without pulmonary stenosis - ventricular septal defect type (disorder)
Congenital Disease	SNOMED	762252001	Common arterial trunk with aortic dominance (disorder)
Congenital Disease	SNOMED	762253006	Common arterial trunk with pulmonary dominance co-occurrent with interrupted aortic arch (disorder)
Congenital Disease	SNOMED	762254000	Congenital dysplasia of tricuspid valve (disorder)
Congenital Disease	SNOMED	762273001	Fetal intrauterine intestinal perforation co-occurrent and due to congenital atresia of intestinal tract (disorder)
Congenital Disease	SNOMED	762295002	Congenital obstructive hydrocephalus (disorder)
Congenital Disease	SNOMED	762402007	Congenital anomaly of zonula (disorder)
Congenital Disease	SNOMED	762403002	Cleft hard palate with left cleft lip (disorder)
Congenital Disease	SNOMED	762404008	Cleft hard palate with right cleft lip (disorder)
Congenital Disease	SNOMED	762405009	Cleft soft palate with left cleft lip (disorder)
Congenital Disease	SNOMED	762406005	Cleft soft palate with right cleft lip (disorder)
Congenital Disease	SNOMED	762407001	Cleft hard and soft palate with left cleft lip (disorder)
Congenital Disease	SNOMED	762408006	Cleft hard and soft palate with right cleft lip (disorder)
Congenital Disease	SNOMED	762409003	Cleft palate with left cleft lip (disorder)
Congenital Disease	SNOMED	762410008	Cleft palate with right cleft lip (disorder)
Congenital Disease	SNOMED	762414004	Microphthalmic socket (disorder)
Congenital Disease	SNOMED	762433009	Tetralogy of Fallot with pulmonary atresia co-occurrent with systemic-to-pulmonary collateral artery (disorder)
Congenital Disease	SNOMED	762460002	Hemodynamically insignificant ventricular septal defect (disorder)
Congenital Disease	SNOMED	76257003	Bicuspid cardiac valve (disorder)
Congenital Disease	SNOMED	762580003	Cleft hard palate with bilateral cleft lip and bilateral cleft of alveolar process of maxilla (disorder)
Congenital Disease	SNOMED	762581004	Cleft hard palate with left cleft lip and cleft of left alveolar process of maxilla (disorder)
Congenital Disease	SNOMED	762582006	Cleft hard palate with right cleft lip and cleft of right alveolar process of maxilla (disorder)
Congenital Disease	SNOMED	762583001	Cleft soft palate with bilateral cleft lip and bilateral cleft of alveolar process of maxilla (disorder)
Congenital Disease	SNOMED	762584007	Cleft soft palate with left cleft lip and cleft of left alveolar process of maxilla (disorder)
Congenital Disease	SNOMED	762585008	Cleft soft palate with right cleft lip and cleft of right alveolar process of maxilla (disorder)
Congenital Disease	SNOMED	762586009	Cleft hard and soft palate with bilateral cleft lip and bilateral cleft of alveolar process of maxilla (disorder)
Congenital Disease	SNOMED	762587000	Cleft hard and soft palate with left cleft lip and left alveolar process of maxilla (disorder)
Congenital Disease	SNOMED	762588005	Cleft hard and soft palate with right cleft lip and cleft of right alveolar process of maxilla (disorder)
Congenital Disease	SNOMED	762658005	Aplasia of auditory canal (disorder)
Congenital Disease	SNOMED	762660007	Cleft palate with bilateral cleft lip and bilateral cleft of alveolar process of maxilla (disorder)
Congenital Disease	SNOMED	762662004	Hypoplasia of auditory canal (disorder)
Congenital Disease	SNOMED	76280006	Rudimentary tracheal bronchus (disorder)
Congenital Disease	SNOMED	762907005	Agenesis of left kidney (disorder)
Congenital Disease	SNOMED	762908000	Agenesis of right kidney (disorder)
Congenital Disease	SNOMED	762909008	Agenesis of left kidney co-occurrent with hypoplasia of right kidney (disorder)
Congenital Disease	SNOMED	762910003	Agenesis of right kidney co-occurrent with hypoplasia of left kidney (disorder)
Congenital Disease	SNOMED	762911004	Agenesis of right kidney co-occurrent with congenital dysplasia of left kidney (disorder)
Congenital Disease	SNOMED	762912006	Agenesis of left kidney co-occurrent with congenital dysplasia of right kidney (disorder)
Congenital Disease	SNOMED	762913001	Congenital hypoplasia of left kidney (disorder)
Congenital Disease	SNOMED	762914007	Congenital hypoplasia of right kidney (disorder)
Congenital Disease	SNOMED	76304001	Cronkhite-Canada syndrome (disorder)
Congenital Disease	SNOMED	763061004	20q11.2 microduplication syndrome (disorder)
Congenital Disease	SNOMED	763062006	2q33.1 microdeletion syndrome (disorder)
Congenital Disease	SNOMED	763066009	Atrioventricular septal defect, blepharophimosis, radial and anal defect syndrome (disorder)
Congenital Disease	SNOMED	763108005	Submucous cleft palate (disorder)
Congenital Disease	SNOMED	763109002	Central cleft of soft palate (disorder)
Congenital Disease	SNOMED	763128009	Bipartite talus (disorder)
Congenital Disease	SNOMED	763129001	Dermoid cyst of neck (disorder)
Congenital Disease	SNOMED	763130006	Cleft palate, large ears, small head syndrome (disorder)
Congenital Disease	SNOMED	763132003	Coloboma of superior eyelid (disorder)
Congenital Disease	SNOMED	763133008	Coloboma of inferior eyelid (disorder)
Congenital Disease	SNOMED	763134002	Chondroectodermal dysplasia with night blindness syndrome (disorder)
Congenital Disease	SNOMED	763186006	Grubben, De Cock, Borghgraef syndrome (disorder)
Congenital Disease	SNOMED	763213001	Conductive deafness, ptosis, skeletal anomalies syndrome (disorder)
Congenital Disease	SNOMED	763220008	Dermoid cyst of face (disorder)

Congenital Disease	SNOMED	763272003	Distal trisomy 2q (disorder)
Congenital Disease	SNOMED	763273008	Distal trisomy 4q (disorder)
Congenital Disease	SNOMED	763274002	Distal trisomy 5q syndrome (disorder)
Congenital Disease	SNOMED	763275001	Distal trisomy 6q (disorder)
Congenital Disease	SNOMED	763276000	Distal trisomy 7p syndrome (disorder)
Congenital Disease	SNOMED	763277009	Distal trisomy 8q (disorder)
Congenital Disease	SNOMED	763278004	Facial dysmorphism, cleft palate, loose skin syndrome (disorder)
Congenital Disease	SNOMED	763279007	Facial dysmorphism, conductive hearing loss, heart defect syndrome (disorder)
Congenital Disease	SNOMED	763316006	Congenital patent ductus arteriosus aneurysm (disorder)
Congenital Disease	SNOMED	763317002	Isolated congenital syngnathia (disorder)
Congenital Disease	SNOMED	763318007	Connective tissue disorder due to lysyl hydroxylase-3 deficiency (disorder)
Congenital Disease	SNOMED	763320005	Craniofaciofrontodigital syndrome (disorder)
Congenital Disease	SNOMED	763350002	Intellectual disability, obesity, brain malformation, facial dysmorphism syndrome (disorder)
Congenital Disease	SNOMED	763353000	Cerebrofacioarticular syndrome (disorder)
Congenital Disease	SNOMED	763366000	Leukoencephalopathy, thalamus and brainstem anomalies, high lactate syndrome (disorder)
Congenital Disease	SNOMED	763401009	Ichthyosis prematurity syndrome (disorder)
Congenital Disease	SNOMED	763404001	Ichthyosis, alopecia, eclabion, ectropion, intellectual disability syndrome (disorder)
Congenital Disease	SNOMED	763405000	Ring chromosome 15 syndrome (disorder)
Congenital Disease	SNOMED	763406004	Ring chromosome 16 syndrome (disorder)
Congenital Disease	SNOMED	763407008	Ring chromosome Y syndrome (disorder)
Congenital Disease	SNOMED	763462004	X-linked lethal multiple pterygium syndrome (disorder)
Congenital Disease	SNOMED	763498007	Incomplete left cleft lip and incomplete cleft of left alveolar process of maxilla (disorder)
Congenital Disease	SNOMED	763499004	Incomplete right cleft lip and incomplete cleft of right alveolar process of maxilla (disorder)
Congenital Disease	SNOMED	763527007	Distal monosomy 13q syndrome (disorder)
Congenital Disease	SNOMED	763528002	Distal monosomy 3p syndrome (disorder)
Congenital Disease	SNOMED	763529005	Distal monosomy 7q36 syndrome (disorder)
Congenital Disease	SNOMED	763530000	Distal monosomy 9p syndrome (disorder)
Congenital Disease	SNOMED	763532008	Familial nasal acilia (disorder)
Congenital Disease	SNOMED	763535005	Supernumerary phalanx (disorder)
Congenital Disease	SNOMED	763615003	Aortic arch anomaly, facial dysmorphism, intellectual disability syndrome (disorder)
Congenital Disease	SNOMED	763616002	Velofacioskeletal syndrome (disorder)
Congenital Disease	SNOMED	763618001	Wiedemann Steiner syndrome (disorder)
Congenital Disease	SNOMED	763619009	White forelock with malformations syndrome (disorder)
Congenital Disease	SNOMED	763620003	Trichodermodysplasia and dental alterations syndrome (disorder)
Congenital Disease	SNOMED	763624007	Syndactyly type 6 (disorder)
Congenital Disease	SNOMED	763630007	Satoyoshi syndrome (disorder)
Congenital Disease	SNOMED	763631006	Short stature, wormian bones, dextrocardia syndrome (disorder)
Congenital Disease	SNOMED	763658004	Hypotrichosis, osteolysis, periodontitis, palmoplantar keratoderma syndrome (disorder)
Congenital Disease	SNOMED	763665007	Craniodigital syndrome and intellectual disability syndrome (disorder)
Congenital Disease	SNOMED	763683004	46,XY ovotesticular disorder of sex development (disorder)
Congenital Disease	SNOMED	763684005	Craniosynostosis Herrmann Opitz type (disorder)
Congenital Disease	SNOMED	763686007	Curly hair, acral keratoderma, caries syndrome (disorder)
Congenital Disease	SNOMED	763691008	Familial isolated clinodactyly of finger (disorder)
Congenital Disease	SNOMED	763714006	Familial multiple nevi flammei (disorder)
Congenital Disease	SNOMED	763717004	Sporadic fetal brain disruption sequence (disorder)
Congenital Disease	SNOMED	763722004	Hypotonia, speech impairment, severe cognitive delay syndrome (disorder)
Congenital Disease	SNOMED	763727005	Complete left cleft lip and complete cleft of left alveolar process of maxilla (disorder)
Congenital Disease	SNOMED	763728000	Complete right cleft lip and complete cleft of right alveolar process of maxilla (disorder)
Congenital Disease	SNOMED	763733001	Complete left cleft lip (disorder)
Congenital Disease	SNOMED	763734007	Complete right cleft lip (disorder)
Congenital Disease	SNOMED	763735008	Bilateral complete cleft lip and bilateral complete cleft of alveolar process of maxilla (disorder)
Congenital Disease	SNOMED	763741001	Intellectual disability, alacrima, achalasia syndrome (disorder)
Congenital Disease	SNOMED	763742008	Intellectual disability, polydactyly, uncombable hair syndrome (disorder)
Congenital Disease	SNOMED	763743003	Intellectual disability, spasticity, ectrodactyly syndrome (disorder)
Congenital Disease	SNOMED	763744009	Intellectual disability, brachydactyly, Pierre Robin syndrome (disorder)
Congenital Disease	SNOMED	763745005	Intellectual disability Wolff type (disorder)
Congenital Disease	SNOMED	763747002	Congenital aneurysm of membranous portion of interventricular septum (disorder)
Congenital Disease	SNOMED	763748007	Isolated congenital adermatoglyphia (disorder)
Congenital Disease	SNOMED	763753002	Incomplete right cleft lip (disorder)
Congenital Disease	SNOMED	763754008	Incomplete left cleft lip (disorder)
Congenital Disease	SNOMED	763755009	Dislocation of hip and facial dysmorphism syndrome (disorder)
Congenital Disease	SNOMED	763767006	Erythema palmare hereditarium (disorder)
Congenital Disease	SNOMED	763768001	Autosomal recessive exfoliative ichthyosis (disorder)

Congenital Disease	SNOMED	763773007	Macrocephaly and developmental delay syndrome (disorder)
Congenital Disease	SNOMED	763774001	Keipert syndrome (disorder)
Congenital Disease	SNOMED	763775000	Keratosis linearis, ichthyosis congenita, sclerosing keratoderma syndrome (disorder)
Congenital Disease	SNOMED	763778003	Larsen-like syndrome beta-1,3-glucuronyltransferase 3 type (disorder)
Congenital Disease	SNOMED	763792009	Leukonychia totalis (disorder)
Congenital Disease	SNOMED	763795006	Malan overgrowth syndrome (disorder)
Congenital Disease	SNOMED	763797003	Agenesis of corpus callosum and abnormal genitalia syndrome (disorder)
Congenital Disease	SNOMED	763815000	Oculoauricular syndrome Schorderet type (disorder)
Congenital Disease	SNOMED	763821001	Porencephaly, cerebellar hypoplasia, internal malformations syndrome (disorder)
Congenital Disease	SNOMED	763828007	Odonto onycho dysplasia with alopecia syndrome (disorder)
Congenital Disease	SNOMED	763830009	Oculomaxillofacial dysostosis (disorder)
Congenital Disease	SNOMED	763833006	Oro-facial digital syndrome type 1 (disorder)
Congenital Disease	SNOMED	763834000	Oro-facial digital syndrome type 12 (disorder)
Congenital Disease	SNOMED	763835004	Oro-facial digital syndrome type 13 (disorder)
Congenital Disease	SNOMED	763837007	Oro-facial digital syndrome type 14 (disorder)
Congenital Disease	SNOMED	763839005	Neonatal Marfan syndrome (disorder)
Congenital Disease	SNOMED	763860004	Otofaciocervical syndrome (disorder)
Congenital Disease	SNOMED	763861000	Pachygyria, intellectual disability, epilepsy syndrome (disorder)
Congenital Disease	SNOMED	763863002	Pectus excavatum, macrocephaly, dysplastic nails syndrome (disorder)
Congenital Disease	SNOMED	763866005	Postaxial polydactyly, anterior pituitary anomalies, facial dysmorphism syndrome (disorder)
Congenital Disease	SNOMED	763867001	Segmental outgrowth, lipomatosis, arteriovenous malformation, epidermal nevus syndrome (disorder)
Congenital Disease	SNOMED	763868006	Short stature homeobox related short stature (disorder)
Congenital Disease	SNOMED	763885008	Spondyloepimetaphyseal dysplasia Handigodu type (disorder)
Congenital Disease	SNOMED	763886009	Spondyloperipheral dysplasia with short ulna syndrome (disorder)
Congenital Disease	SNOMED	763889002	Spina bifida and hypospadias syndrome (disorder)
Congenital Disease	SNOMED	763891005	Renal hepatic pancreatic dysplasia (disorder)
Congenital Disease	SNOMED	763893008	Multiple epiphyseal dysplasia with severe proximal femoral dysplasia (disorder)
Congenital Disease	SNOMED	76401000119105	Congenital anomaly of ureter and renal pelvis (disorder)
Congenital Disease	SNOMED	764091001	Right cleft lip (disorder)
Congenital Disease	SNOMED	764092008	Left cleft lip (disorder)
Congenital Disease	SNOMED	764105002	Pseudoxanthoma elasticum-like papillary dermal elastolysis (disorder)
Congenital Disease	SNOMED	764106001	Symbrachydactyly of digit of hand (disorder)
Congenital Disease	SNOMED	764108000	Wooly hair with palmoplantar keratoderma syndrome (disorder)
Congenital Disease	SNOMED	764435003	17q12 microduplication syndrome (disorder)
Congenital Disease	SNOMED	764437006	Brachydactyly elbow wrist dysplasia (disorder)
Congenital Disease	SNOMED	764440006	19p13.13 microdeletion syndrome (disorder)
Congenital Disease	SNOMED	764447009	Distal trisomy 11q (disorder)
Congenital Disease	SNOMED	764452004	Retinal arterial macroaneurysm with supravalvular pulmonic stenosis (disorder)
Congenital Disease	SNOMED	764454003	Distal trisomy 13q (disorder)
Congenital Disease	SNOMED	764455002	Cognitive impairment, coarse facies, heart defects, obesity, pulmonary involvement, short stature, skeletal dysplasia syndrome (disorder)
Congenital Disease	SNOMED	764459008	Distal trisomy 16q (disorder)
Congenital Disease	SNOMED	764460003	Spondyloepimetaphyseal dysplasia anauxetic type (disorder)
Congenital Disease	SNOMED	764461004	Mosaic trisomy 10 syndrome (disorder)
Congenital Disease	SNOMED	764463001	Mosaic trisomy 12 syndrome (disorder)
Congenital Disease	SNOMED	764466009	Mosaic trisomy 14 syndrome (disorder)
Congenital Disease	SNOMED	76447002	6q partial trisomy syndrome (disorder)
Congenital Disease	SNOMED	764500002	Distal trisomy 20q syndrome (disorder)
Congenital Disease	SNOMED	764512003	Distal trisomy 22q syndrome (disorder)
Congenital Disease	SNOMED	764517009	Craniofacial cleft (disorder)
Congenital Disease	SNOMED	764518004	Distal trisomy 2p (disorder)
Congenital Disease	SNOMED	764519007	Distal trisomy 3p (disorder)
Congenital Disease	SNOMED	764520001	Distal trisomy 9q (disorder)
Congenital Disease	SNOMED	764521002	Encircling double aortic arch (disorder)
Congenital Disease	SNOMED	764523004	Familial isolated trichomegaly (disorder)
Congenital Disease	SNOMED	764524005	Distal 22q11.2 microduplication syndrome (disorder)
Congenital Disease	SNOMED	764619001	Mosaic trisomy 15 syndrome (disorder)
Congenital Disease	SNOMED	764621006	Mosaic trisomy 16 syndrome (disorder)
Congenital Disease	SNOMED	764622004	Mosaic trisomy 17 syndrome (disorder)
Congenital Disease	SNOMED	764623009	Mosaic trisomy 2 syndrome (disorder)
Congenital Disease	SNOMED	764624003	Mosaic trisomy 20 syndrome (disorder)
Congenital Disease	SNOMED	764625002	Mosaic trisomy 22 syndrome (disorder)
Congenital Disease	SNOMED	764627005	Mosaic trisomy 3 syndrome (disorder)
Congenital Disease	SNOMED	764628000	Mosaic trisomy 4 syndrome (disorder)
Congenital Disease	SNOMED	764629008	Mosaic trisomy 5 syndrome (disorder)
Congenital Disease	SNOMED	764630003	Mosaic trisomy 7 syndrome (disorder)
Congenital Disease	SNOMED	764690001	Tetrasomy 21 (disorder)
Congenital Disease	SNOMED	764696007	Distal 17p13.3 microdeletion syndrome (disorder)
Congenital Disease	SNOMED	764697003	Verloove Vanhorick Brubakk syndrome (disorder)

Congenital Disease	SNOMED	764703002	7p22.1 microduplication syndrome (disorder)
Congenital Disease	SNOMED	764711007	Xq12-q13.3 duplication syndrome (disorder)
Congenital Disease	SNOMED	764725008	9p13 microdeletion syndrome (disorder)
Congenital Disease	SNOMED	764732004	Microcephalus, cerebellar hypoplasia, cardiac conduction defect syndrome (disorder)
Congenital Disease	SNOMED	764739008	Proximal chromosome 18q deletion syndrome (disorder)
Congenital Disease	SNOMED	764810000	Branchiootic syndrome (disorder)
Congenital Disease	SNOMED	76481000119102	Pressure injury due to spina bifida (disorder)
Congenital Disease	SNOMED	764812008	Autosomal recessive myogenic arthrogryposis multiplex congenita (disorder)
Congenital Disease	SNOMED	764857004	Tibial hemimelia, polysyndactyly, triphalangeal thumb syndrome (disorder)
Congenital Disease	SNOMED	764861005	Intellectual disability Birk-Barel type (disorder)
Congenital Disease	SNOMED	764942005	Colobomatous microphthalmia, rhizomelic dysplasia syndrome (disorder)
Congenital Disease	SNOMED	764946008	Constitutional mismatch repair deficiency syndrome (disorder)
Congenital Disease	SNOMED	764950001	Cryptorchidism, arachnodactyly, intellectual disability syndrome (disorder)
Congenital Disease	SNOMED	764955006	Laubry Pezzi syndrome (disorder)
Congenital Disease	SNOMED	764956007	Larsen-like osseous dysplasia, short stature syndrome (disorder)
Congenital Disease	SNOMED	764958008	Striate palmoplantar keratoderma (disorder)
Congenital Disease	SNOMED	764959000	Intellectual disability, myopathy, short stature, endocrine defect syndrome (disorder)
Congenital Disease	SNOMED	764963007	Focal palmoplantar and gingival keratoderma (disorder)
Congenital Disease	SNOMED	764965000	Familial thoracic aortic aneurysm and aortic dissection (disorder)
Congenital Disease	SNOMED	764989007	Mosaic trisomy 9 syndrome (disorder)
Congenital Disease	SNOMED	764995008	Nail and tooth abnormalities, marginal palmoplantar keratoderma, oral hyperpigmentation syndrome (disorder)
Congenital Disease	SNOMED	764996009	Non-distal trisomy 13q (disorder)
Congenital Disease	SNOMED	764997000	Non-distal trisomy 9q (disorder)
Congenital Disease	SNOMED	765089003	Focal epilepsy, intellectual disability, cerebro-cerebellar malformation syndrome (disorder)
Congenital Disease	SNOMED	765096001	Punctate palmoplantar keratoderma type 2 (disorder)
Congenital Disease	SNOMED	765135003	Primary essential cutis verticis gyrata (disorder)
Congenital Disease	SNOMED	765138001	Birth defect due to maternal hyperthermia (disorder)
Congenital Disease	SNOMED	765140006	8p23.1 duplication syndrome (disorder)
Congenital Disease	SNOMED	765142003	Proximal 16p11.2 microduplication syndrome (disorder)
Congenital Disease	SNOMED	765154003	Hypertrophy of right kidney co-occurrent and due to congenital hypoplasia of left kidney (disorder)
Congenital Disease	SNOMED	765155002	Hypertrophy of left kidney co-occurrent and due to congenital hypoplasia of right kidney (disorder)
Congenital Disease	SNOMED	765171002	Distal chromosome 18q deletion syndrome (disorder)
Congenital Disease	SNOMED	76520005	Robinow syndrome (disorder)
Congenital Disease	SNOMED	765204000	Dyssegmental dysplasia Silverman Handmaker type (disorder)
Congenital Disease	SNOMED	765206003	Constriction ring syndrome (disorder)
Congenital Disease	SNOMED	765330003	Autosomal dominant polycystic kidney disease (disorder)
Congenital Disease	SNOMED	765331004	Autosomal dominant polycystic kidney disease type 1 with tuberous sclerosis (disorder)
Congenital Disease	SNOMED	765434008	Human immunodeficiency virus type I enhancer binding protein 2 related intellectual disability (disorder)
Congenital Disease	SNOMED	765471005	X-linked intellectual disability, hypogonadism, ichthyosis, obesity, short stature syndrome (disorder)
Congenital Disease	SNOMED	765484001	Ring chromosome 19 syndrome (disorder)
Congenital Disease	SNOMED	765485000	Ring chromosome 2 syndrome (disorder)
Congenital Disease	SNOMED	765486004	Ring chromosome 3 syndrome (disorder)
Congenital Disease	SNOMED	765487008	Ring chromosome 5 syndrome (disorder)
Congenital Disease	SNOMED	765488003	Ring chromosome 6 syndrome (disorder)
Congenital Disease	SNOMED	765489006	Ring chromosome 7 syndrome (disorder)
Congenital Disease	SNOMED	76562003	Congenital subcapsular cataract (disorder)
Congenital Disease	SNOMED	765750001	Angio-osteohypotrophic syndrome (disorder)
Congenital Disease	SNOMED	765755006	Axial mesodermal dysplasia spectrum (disorder)
Congenital Disease	SNOMED	765757003	Bilateral polymicrogyria (disorder)
Congenital Disease	SNOMED	765758008	Microcephalic primordial dwarfism Montreal type (disorder)
Congenital Disease	SNOMED	765761009	Brachydactyly, mesomelia, intellectual disability, heart defect syndrome (disorder)
Congenital Disease	SNOMED	765763007	Congenital cyst of larynx (disorder)
Congenital Disease	SNOMED	765775002	Dysplasia of left kidney (disorder)
Congenital Disease	SNOMED	765776001	Dysplasia of right kidney (disorder)
Congenital Disease	SNOMED	765777005	Congenital left vesicoureterorenal reflux (disorder)
Congenital Disease	SNOMED	765778000	Congenital right vesicoureterorenal reflux (disorder)
Congenital Disease	SNOMED	765812004	Congenital absence of innominate vein (disorder)
Congenital Disease	SNOMED	766032007	Holoprosencephaly, ectrodactyly, cleft lip, cleft palate syndrome (disorder)
Congenital Disease	SNOMED	766050000	Distal monosomy 15q syndrome (disorder)
Congenital Disease	SNOMED	766051001	Distal trisomy 17q syndrome (disorder)
Congenital Disease	SNOMED	766052008	Distal trisomy 19q syndrome (disorder)
Congenital Disease	SNOMED	766053003	Distal trisomy 1p36 syndrome (disorder)
Congenital Disease	SNOMED	76621000	Urticaria pigmentosa, infantile form (disorder)
Congenital Disease	SNOMED	766237006	Maternal uniparental disomy of chromosome 2 (disorder)
Congenital Disease	SNOMED	766238001	Maternal uniparental disomy of chromosome 4 (disorder)

Congenital Disease	SNOMED	766239009	Maternal uniparental disomy of chromosome 6 (disorder)
Congenital Disease	SNOMED	766240006	Maternal uniparental disomy of chromosome 9 (disorder)
Congenital Disease	SNOMED	766243008	Talipes varus (disorder)
Congenital Disease	SNOMED	766245001	Talipes calcaneocavus (disorder)
Congenital Disease	SNOMED	766249007	Deafness, nephritis, anorectal malformation syndrome (disorder)
Congenital Disease	SNOMED	766303001	Talipes transversoplanus (disorder)
Congenital Disease	SNOMED	766708008	Isochromosomy Yp (disorder)
Congenital Disease	SNOMED	766709000	Isolated hypoplasia of cerebellar vermis (disorder)
Congenital Disease	SNOMED	766710005	Isolated focal cortical dysplasia (disorder)
Congenital Disease	SNOMED	766716004	Monosomy 13q34 syndrome (disorder)
Congenital Disease	SNOMED	766717008	Multiple epiphyseal dysplasia due to collagen 9 anomaly (disorder)
Congenital Disease	SNOMED	766719006	Paternal uniparental disomy of chromosome 1 (disorder)
Congenital Disease	SNOMED	766720000	Paternal uniparental disomy of chromosome 21 (disorder)
Congenital Disease	SNOMED	766721001	Paternal uniparental disomy of chromosome 7 (disorder)
Congenital Disease	SNOMED	766750008	Multiple epiphyseal dysplasia with miniepiphyses (disorder)
Congenital Disease	SNOMED	766751007	Neuhauser anomaly (disorder)
Congenital Disease	SNOMED	766753005	Nijmegen breakage syndrome-like disorder (disorder)
Congenital Disease	SNOMED	766755003	Tetrasomy 5p syndrome (disorder)
Congenital Disease	SNOMED	766756002	Subaortic course of innominate vein (disorder)
Congenital Disease	SNOMED	766760004	Small ring X chromosome (disorder)
Congenital Disease	SNOMED	766761000	X-linked cleft palate and ankyloglossia (disorder)
Congenital Disease	SNOMED	766765009	Radio-renal syndrome (disorder)
Congenital Disease	SNOMED	766766005	1p31p32 microdeletion syndrome (disorder)
Congenital Disease	SNOMED	766812005	Trichodysplasia xeroderma syndrome (disorder)
Congenital Disease	SNOMED	766813000	Trichoodontoonychial dysplasia (disorder)
Congenital Disease	SNOMED	766816008	2q23.1 microduplication syndrome (disorder)
Congenital Disease	SNOMED	766819001	Severe lateral tibial bowing with short stature (disorder)
Congenital Disease	SNOMED	766820007	Spondyloepimetaphyseal dysplasia with multiple dislocations (disorder)
Congenital Disease	SNOMED	766821006	Spondyloepimetaphyseal dysplasia, short limb, abnormal calcification syndrome (disorder)
Congenital Disease	SNOMED	766824003	Activity dependent neuroprotector homeobox related multiple congenital anomalies, intellectual disability, autism spectrum disorder (disorder)
Congenital Disease	SNOMED	766870005	Epiphyseal dysplasia, hearing loss, dysmorphism syndrome (disorder)
Congenital Disease	SNOMED	766871009	Diencephalic mesencephalic junction dysplasia (disorder)
Congenital Disease	SNOMED	766874001	Cono-spondylar dysplasia (disorder)
Congenital Disease	SNOMED	766881008	Carney complex, trismus, pseudocamptodactyly syndrome (disorder)
Congenital Disease	SNOMED	766927009	Familial supernumerary nipple (disorder)
Congenital Disease	SNOMED	766928004	Generalized basaloid follicular hamartoma syndrome (disorder)
Congenital Disease	SNOMED	766931003	Hypomyelination neuropathy arthrogryposis syndrome (disorder)
Congenital Disease	SNOMED	766932005	Hypothalamic hamartoma with gelastic seizure (disorder)
Congenital Disease	SNOMED	766934006	Isolated unilateral hemispheric cerebellar hypoplasia (disorder)
Congenital Disease	SNOMED	766976003	Pulmonary valve agenesis, tetralogy of Fallot, absence of ductus arteriosus syndrome (disorder)
Congenital Disease	SNOMED	766983005	Susceptibility to respiratory infection associated with CD8alpha chain mutation (disorder)
Congenital Disease	SNOMED	766987006	Moebius syndrome (disorder)
Congenital Disease	SNOMED	766992008	Multicentric carpotarsal osteolysis syndrome (disorder)
Congenital Disease	SNOMED	766999004	Congenital absence of left foot (disorder)
Congenital Disease	SNOMED	767000001	Congenital absence of right foot (disorder)
Congenital Disease	SNOMED	767001002	Congenital absence of lower leg and foot (disorder)
Congenital Disease	SNOMED	767002009	Congenital dislocation of left hip (disorder)
Congenital Disease	SNOMED	767003004	Congenital dislocation of right hip (disorder)
Congenital Disease	SNOMED	767004005	Congenital dislocation of left hip co-occurrent with congenital subluxation of right hip (disorder)
Congenital Disease	SNOMED	767005006	Congenital dislocation of right hip co-occurrent with congenital subluxation of left hip (disorder)
Congenital Disease	SNOMED	767029004	Congenital dysplasia of left lobe of lung co-occurrent with congenital vascular malformation (disorder)
Congenital Disease	SNOMED	767030009	Congenital dysplasia of right lobe of lung co-occurrent with congenital vascular malformation (disorder)
Congenital Disease	SNOMED	767031008	Congenital macrostomia of left side of mouth (disorder)
Congenital Disease	SNOMED	767032001	Congenital macrostomia of right side of mouth (disorder)
Congenital Disease	SNOMED	767213002	Talipes cavovalgus (disorder)
Congenital Disease	SNOMED	767214008	Talipes cavovarus (disorder)
Congenital Disease	SNOMED	767309006	Double aortic arch with dominant left arch and hypoplasia of right arch (disorder)
Congenital Disease	SNOMED	767311002	Double aortic arch with dominant right arch and hypoplasia of left arch (disorder)
Congenital Disease	SNOMED	767326000	Congenital absence of left hemidiaphragm (disorder)
Congenital Disease	SNOMED	767327009	Congenital absence of right hemidiaphragm (disorder)
Congenital Disease	SNOMED	767328004	Congenital absence of left testis (disorder)
Congenital Disease	SNOMED	767329007	Congenital absence of right testis (disorder)
Congenital Disease	SNOMED	76744005	Longitudinal deficiency of fibula (disorder)
Congenital Disease	SNOMED	767446006	Congenital instability of left hip joint (disorder)
Congenital Disease	SNOMED	767447002	Congenital instability of right hip joint (disorder)

Congenital Disease	SNOMED	767641005	Embryonic cyst of male genital structure (disorder)
Congenital Disease	SNOMED	767815001	Embryonic cyst of omentum (disorder)
Congenital Disease	SNOMED	76842000	Complete aphalangia of upper limb (disorder)
Congenital Disease	SNOMED	768471006	16p12.2 microdeletion syndrome (disorder)
Congenital Disease	SNOMED	768552007	Congenital ventricular septal defect (disorder)
Congenital Disease	SNOMED	768555009	5q31.3 microdeletion syndrome (disorder)
Congenital Disease	SNOMED	768713003	15q13.3 microduplication syndrome (disorder)
Congenital Disease	SNOMED	76880004	Angelman syndrome (disorder)
Congenital Disease	SNOMED	768843007	Tall stature, intellectual disability, facial dysmorphism syndrome (disorder)
Congenital Disease	SNOMED	768927001	Trisomy 1q syndrome (disorder)
Congenital Disease	SNOMED	768929003	Trisomy 8p syndrome (disorder)
Congenital Disease	SNOMED	768930008	Partial trisomy of short arm of chromosome 8 (disorder)
Congenital Disease	SNOMED	768931007	Partial trisomy of long arm of chromosome 1 (disorder)
Congenital Disease	SNOMED	768932000	Coloboma of choroid and retina (disorder)
Congenital Disease	SNOMED	768935003	Multiple epiphyseal dysplasia Lowry type (disorder)
Congenital Disease	SNOMED	768939009	Primary tethered cord syndrome (disorder)
Congenital Disease	SNOMED	76916001	Spina bifida occulta (disorder)
Congenital Disease	SNOMED	77016009	Amyoplasia congenita disruptive sequence (disorder)
Congenital Disease	SNOMED	770401007	10q22.3q23.3 microdeletion syndrome (disorder)
Congenital Disease	SNOMED	770404004	Autosomal recessive chorioretinopathy and microcephaly syndrome (disorder)
Congenital Disease	SNOMED	770406002	Brachydactyly type B2 (disorder)
Congenital Disease	SNOMED	770408001	Congenital stenosis of cervical spinal canal (disorder)
Congenital Disease	SNOMED	770409009	Crossed polysyndactyly (disorder)
Congenital Disease	SNOMED	770410004	Distal monosomy 14q syndrome (disorder)
Congenital Disease	SNOMED	770411000	Distal monosomy 19p13.3 (disorder)
Congenital Disease	SNOMED	770414008	Alport syndrome (disorder)
Congenital Disease	SNOMED	770432008	Ectasia of left atrial appendage (disorder)
Congenital Disease	SNOMED	770433003	Ectasia of right atrial appendage (disorder)
Congenital Disease	SNOMED	770435005	Familial bicuspid aortic valve (disorder)
Congenital Disease	SNOMED	770542008	Isochromosomy Yq (disorder)
Congenital Disease	SNOMED	770543003	Congenital hypoplasia of part of upper limb (disorder)
Congenital Disease	SNOMED	770544009	Congenital hypoplasia of entire upper limb (disorder)
Congenital Disease	SNOMED	770560008	Lissencephaly due to LIS1 mutation (disorder)
Congenital Disease	SNOMED	770561007	Lower limb malformation hypospadias syndrome (disorder)
Congenital Disease	SNOMED	770562000	Maternal uniparental disomy of chromosome 1 (disorder)
Congenital Disease	SNOMED	770563005	Maternal uniparental disomy of chromosome 13 (disorder)
Congenital Disease	SNOMED	770564004	Microcephalic primordial dwarfism Alazami type (disorder)
Congenital Disease	SNOMED	770565003	Microcephalic primordial dwarfism Dauber type (disorder)
Congenital Disease	SNOMED	770566002	Monosomy 13q14 syndrome (disorder)
Congenital Disease	SNOMED	770591002	Pseudounicornuate uterus (disorder)
Congenital Disease	SNOMED	770595006	Ring chromosome 12 syndrome (disorder)
Congenital Disease	SNOMED	770603000	X-linked spondyloepimetaphyseal dysplasia (disorder)
Congenital Disease	SNOMED	770604006	X-linked cerebral, cerebellar, coloboma syndrome (disorder)
Congenital Disease	SNOMED	770619007	Congenital upper esophageal web (disorder)
Congenital Disease	SNOMED	770625006	Combined immunodeficiency with faciooculoskeletal anomalies syndrome (disorder)
Congenital Disease	SNOMED	770629000	Distal 17p13.1 microdeletion syndrome (disorder)
Congenital Disease	SNOMED	770663003	Tetrasomy 11q24.1 (disorder)
Congenital Disease	SNOMED	770665005	Non-distal monosomy 10q (disorder)
Congenital Disease	SNOMED	770666006	Non-distal trisomy 10q (disorder)
Congenital Disease	SNOMED	770668007	Paternal uniparental disomy of chromosome 13 (disorder)
Congenital Disease	SNOMED	770669004	Paternal uniparental disomy of chromosome 5 (disorder)
Congenital Disease	SNOMED	770670003	Paternal uniparental disomy of chromosome 6 (disorder)
Congenital Disease	SNOMED	770679002	Polyneuropathy, intellectual disability, acromicria, premature menopause syndrome (disorder)
Congenital Disease	SNOMED	770680004	Prader-Willi-like syndrome (disorder)
Congenital Disease	SNOMED	770681000	Robin sequence and oligodactyly syndrome (disorder)
Congenital Disease	SNOMED	770719004	3q27.3 microdeletion syndrome (disorder)
Congenital Disease	SNOMED	770721009	Microcephaly, thin corpus callosum, intellectual disability syndrome (disorder)
Congenital Disease	SNOMED	770725000	Infantile cerebral and cerebellar atrophy with postnatal progressive microcephaly (disorder)
Congenital Disease	SNOMED	770728003	Facial dysmorphism, lens dislocation, anterior segment abnormalities, spontaneous filtering bleb syndrome (disorder)
Congenital Disease	SNOMED	770750002	Intellectual disability, seizures, macrocephaly, obesity syndrome (disorder)
Congenital Disease	SNOMED	770751003	Severe motor and intellectual disabilities, sensorineural deafness, dystonia syndrome (disorder)
Congenital Disease	SNOMED	770754006	2p21 microdeletion syndrome without cystinuria (disorder)
Congenital Disease	SNOMED	770755007	Intellectual disability, seizures, hypotonia, ophthalmologic, skeletal anomalies syndrome (disorder)
Congenital Disease	SNOMED	770756008	2p13.2 microdeletion syndrome (disorder)
Congenital Disease	SNOMED	770760006	16q24.1 microdeletion syndrome (disorder)
Congenital Disease	SNOMED	770788000	Tall stature, scoliosis, macrodactyly of great toe syndrome (disorder)
Congenital Disease	SNOMED	770793002	5p13 microduplication syndrome (disorder)

Congenital Disease	SNOMED	770794008	11p15.4 microduplication syndrome (disorder)
Congenital Disease	SNOMED	770900000	Familial omphalocele syndrome with facial dysmorphism (disorder)
Congenital Disease	SNOMED	770901001	Autosomal recessive intellectual disability, motor dysfunction, multiple joint contracture syndrome (disorder)
Congenital Disease	SNOMED	770902008	Distal monosomy 12p (disorder)
Congenital Disease	SNOMED	770905005	Distal 7q11.23 microdeletion syndrome (disorder)
Congenital Disease	SNOMED	770907002	Kagami Ogata syndrome (disorder)
Congenital Disease	SNOMED	770908007	49,XXXYY syndrome (disorder)
Congenital Disease	SNOMED	770909004	Metaphyseal chondrodysplasia Kaitila type (disorder)
Congenital Disease	SNOMED	770940006	Congenital panfollicular nevus (disorder)
Congenital Disease	SNOMED	770941005	Alopecia, progressive neurological defect, endocrinopathy syndrome (disorder)
Congenital Disease	SNOMED	770943008	Dentin dysplasia with sclerotic bone syndrome (disorder)
Congenital Disease	SNOMED	770944002	Oculootodental syndrome (disorder)
Congenital Disease	SNOMED	770945001	Tetramelic monodactyly (disorder)
Congenital Disease	SNOMED	770946000	Postaxial tetramelic oligodactyly (disorder)
Congenital Disease	SNOMED	770948004	Rhizomelic syndrome Urbach type (disorder)
Congenital Disease	SNOMED	771013004	Pilotto syndrome (disorder)
Congenital Disease	SNOMED	771072001	Monosomy 9p (disorder)
Congenital Disease	SNOMED	771074000	Microcephaly, short stature, intellectual disability, facial dysmorphism syndrome (disorder)
Congenital Disease	SNOMED	771076003	Leptomyelolipoma (disorder)
Congenital Disease	SNOMED	771077007	Intellectual disability, short stature, hypertelorism syndrome (disorder)
Congenital Disease	SNOMED	771142009	Cortical dysplasia with focal epilepsy syndrome (disorder)
Congenital Disease	SNOMED	771146007	Holoprosencephaly with caudal dysgenesis syndrome (disorder)
Congenital Disease	SNOMED	771147003	Isolated arhinencephaly (disorder)
Congenital Disease	SNOMED	771148008	X-linked colobomatous microphthalmia, microcephaly, intellectual disability, short stature syndrome (disorder)
Congenital Disease	SNOMED	771149000	Hepatic fibrosis, renal cyst, intellectual disability syndrome (disorder)
Congenital Disease	SNOMED	771177009	Ectrodactyly polydactyly syndrome (disorder)
Congenital Disease	SNOMED	771178004	Edinburgh malformation syndrome (disorder)
Congenital Disease	SNOMED	771179007	Extrasystoles, short stature, hyperpigmentation, microcephaly syndrome (disorder)
Congenital Disease	SNOMED	771180005	Hallux varus, preaxial polysyndactyly syndrome (disorder)
Congenital Disease	SNOMED	771181009	Hypertrichosis cubiti (disorder)
Congenital Disease	SNOMED	771182002	Thumb deformity, alopecia, pigmentation anomaly syndrome (disorder)
Congenital Disease	SNOMED	771184001	Leukoencephalopathy, palmoplantar keratoderma syndrome (disorder)
Congenital Disease	SNOMED	771185000	Imperforate oropharynx, costovertebral anomalies syndrome (disorder)
Congenital Disease	SNOMED	771186004	Poikiloderma, alopecia, retrognathism, cleft palate syndrome (disorder)
Congenital Disease	SNOMED	771187008	Camptodactyly, arthropathy, coxa-vara, pericarditis syndrome (disorder)
Congenital Disease	SNOMED	771234002	Isolated bilateral hemispheric cerebellar hypoplasia (disorder)
Congenital Disease	SNOMED	771239007	Hidrotic ectodermal dysplasia Christianson Fourie type (disorder)
Congenital Disease	SNOMED	771240009	Pilodental dysplasia, refractive errors syndrome (disorder)
Congenital Disease	SNOMED	771261002	Digital extensor muscle aplasia with polyneuropathy (disorder)
Congenital Disease	SNOMED	771263004	Ptosis and vocal cord paralysis syndrome (disorder)
Congenital Disease	SNOMED	771264005	Absent radius, anogenital anomalies syndrome (disorder)
Congenital Disease	SNOMED	771265006	Teebi Shaltout syndrome (disorder)
Congenital Disease	SNOMED	771266007	Torticollis, keloids, cryptorchidism, renal dysplasia syndrome (disorder)
Congenital Disease	SNOMED	771269000	Autosomal dominant multiple pterygium syndrome (disorder)
Congenital Disease	SNOMED	771301002	Axial spondylometaphyseal dysplasia (disorder)
Congenital Disease	SNOMED	771303004	Severe neonatal onset encephalopathy with microcephaly (disorder)
Congenital Disease	SNOMED	771306007	Hereditary fibrosing poikiloderma, tendon contractures, myopathy, pulmonary fibrosis syndrome (disorder)
Congenital Disease	SNOMED	771308008	Non-acquired combined pituitary hormone deficiency, sensorineural hearing loss, spine abnormalities syndrome (disorder)
Congenital Disease	SNOMED	771335004	Ectodermal dysplasia syndactyly syndrome (disorder)
Congenital Disease	SNOMED	771336003	Polymicrogyria with optic nerve hypoplasia (disorder)
Congenital Disease	SNOMED	771337007	1q21.1 microduplication syndrome (disorder)
Congenital Disease	SNOMED	771338002	Parietal foramina with clavicular hypoplasia (disorder)
Congenital Disease	SNOMED	771340007	15q11.2 microdeletion syndrome (disorder)
Congenital Disease	SNOMED	771341006	14q11.2 microduplication syndrome (disorder)
Congenital Disease	SNOMED	771342004	Microcornea, rod-cone dystrophy, cataract, posterior staphyloma syndrome (disorder)
Congenital Disease	SNOMED	771439009	14q22q23 microdeletion syndrome (disorder)
Congenital Disease	SNOMED	771440006	Hemihyperplasia with multiple lipomatosis syndrome (disorder)
Congenital Disease	SNOMED	771442003	Ogden syndrome (disorder)
Congenital Disease	SNOMED	771470001	Jawad syndrome (disorder)
Congenital Disease	SNOMED	771473004	Papular epidermal nevi with skyline basal cell layers syndrome (disorder)
Congenital Disease	SNOMED	771476007	Autosomal recessive leukoencephalopathy, ischemic stroke, retinitis pigmentosa syndrome (disorder)
Congenital Disease	SNOMED	771477003	15q overgrowth syndrome (disorder)
Congenital Disease	SNOMED	771511005	Thrombocythemia with distal limb defect (disorder)
Congenital Disease	SNOMED	771515001	Facial dysmorphism, immunodeficiency, livedo, short stature syndrome (disorder)
Congenital Disease	SNOMED	7720002	Metaphyseal chondrodysplasia, McKusick type (disorder)

Congenital Disease	SNOMED	7722005	Thoracopagus epigastricus (disorder)
Congenital Disease	SNOMED	772224009	Warburg micro syndrome (disorder)
Congenital Disease	SNOMED	772225005	RAB18, member RAS oncogene family deficiency (disorder)
Congenital Disease	SNOMED	77224008	Spina bifida of lumbar region (disorder)
Congenital Disease	SNOMED	77269002	Complete trisomy 16 syndrome (disorder)
Congenital Disease	SNOMED	7731005	Autosomal dominant hypohidrotic ectodermal dysplasia syndrome (disorder)
Congenital Disease	SNOMED	77311006	Omphaloangiopagus (disorder)
Congenital Disease	SNOMED	773139006	Congenital abnormality of cardiac ventricle (disorder)
Congenital Disease	SNOMED	773274001	X-linked intellectual disability, craniofacioskeletal syndrome (disorder)
Congenital Disease	SNOMED	773276004	Ehlers-Danlos syndrome spondylocheirodysplastic type (disorder)
Congenital Disease	SNOMED	773278003	Familial osteodysplasia Anderson type (disorder)
Congenital Disease	SNOMED	773279006	Postaxial polydactyly, dental, vertebral anomalies syndrome (disorder)
Congenital Disease	SNOMED	773280009	Hydrocephalus, blue sclera, nephropathy syndrome (disorder)
Congenital Disease	SNOMED	773281008	Thakker Donnai syndrome (disorder)
Congenital Disease	SNOMED	773282001	Macrosomia, microphthalmia, cleft palate syndrome (disorder)
Congenital Disease	SNOMED	773299000	Maternal uniparental disomy of chromosome 16 (disorder)
Congenital Disease	SNOMED	773300008	Spondyloepimetaphyseal dysplasia, hypotrichosis syndrome (disorder)
Congenital Disease	SNOMED	773302000	Spondyloepimetaphyseal dysplasia, abnormal dentition syndrome (disorder)
Congenital Disease	SNOMED	773303005	Spondyloepimetaphyseal dysplasia Genevieve type (disorder)
Congenital Disease	SNOMED	773304004	Spondylometaphyseal dysplasia Golden type (disorder)
Congenital Disease	SNOMED	773305003	Microcephaly, polymicrogyria, corpus callosum agenesis syndrome (disorder)
Congenital Disease	SNOMED	773307006	Zechi Ceide syndrome (disorder)
Congenital Disease	SNOMED	773325004	Distal 7q11.23 microduplication syndrome (disorder)
Congenital Disease	SNOMED	773326003	7q31 microdeletion syndrome (disorder)
Congenital Disease	SNOMED	773329005	CK syndrome (disorder)
Congenital Disease	SNOMED	773332008	Craniosynostosis and dental anomalies syndrome (disorder)
Congenital Disease	SNOMED	773346008	20p13 microdeletion syndrome (disorder)
Congenital Disease	SNOMED	773394007	Autosomal recessive frontotemporal pachygyria (disorder)
Congenital Disease	SNOMED	773396009	Distal arthrogryposis type 5D (disorder)
Congenital Disease	SNOMED	773400009	Severe feeding difficulties, failure to thrive, microcephaly due to ASXL transcriptional regulator 3 deficiency syndrome (disorder)
Congenital Disease	SNOMED	773404000	Roifman syndrome (disorder)
Congenital Disease	SNOMED	773415005	Contiguous ABCD1 DXS1357E deletion syndrome (disorder)
Congenital Disease	SNOMED	773416006	Intellectual disability, facial dysmorphism, hand anomalies syndrome (disorder)
Congenital Disease	SNOMED	773419004	Severe intellectual disability, short stature, behavioral abnormalities, facial dysmorphism syndrome (disorder)
Congenital Disease	SNOMED	773493002	9q31.1q31.3 microdeletion syndrome (disorder)
Congenital Disease	SNOMED	773494008	14q24.1q24.3 microdeletion syndrome (disorder)
Congenital Disease	SNOMED	773497001	Partial corpus callosum agenesis, cerebellar vermis hypoplasia with posterior fossa cysts syndrome (disorder)
Congenital Disease	SNOMED	773501006	Epidermolysis bullosa simplex due to BP230 deficiency (disorder)
Congenital Disease	SNOMED	773503009	Epidermolysis bullosa simplex due to exophilin 5 deficiency (disorder)
Congenital Disease	SNOMED	773547003	13q12.3 microdeletion syndrome (disorder)
Congenital Disease	SNOMED	773551001	Severe intellectual disability, poor language, strabismus, grimacing face, long fingers syndrome (disorder)
Congenital Disease	SNOMED	773552008	Intellectual disability, feeding difficulties, developmental delay, microcephaly syndrome (disorder)
Congenital Disease	SNOMED	773553003	Hypohidrosis, enamel hypoplasia, palmoplantar keratoderma, intellectual disability syndrome (disorder)
Congenital Disease	SNOMED	773554009	THO complex 6-related developmental delay, microcephaly, facial dysmorphism syndrome (disorder)
Congenital Disease	SNOMED	773556006	Short ulna, dysmorphism, hypotonia, intellectual disability syndrome (disorder)
Congenital Disease	SNOMED	773557002	Mandibulofacial dysostosis, macroblepharon, macrostomia syndrome (disorder)
Congenital Disease	SNOMED	773577009	Corneal intraepithelial dyskeratosis, palmoplantar hyperkeratosis, laryngeal dyskeratosis syndrome (disorder)
Congenital Disease	SNOMED	773581009	Intellectual disability, craniofacial dysmorphism, cryptorchidism syndrome (disorder)
Congenital Disease	SNOMED	773583007	Aphonia, deafness, retinal dystrophy, bifid halluces, intellectual disability syndrome (disorder)
Congenital Disease	SNOMED	773584001	Muscular hypertrophy, hepatomegaly, polyhydramnios syndrome (disorder)
Congenital Disease	SNOMED	773586004	Incomplete ossification of skull (disorder)
Congenital Disease	SNOMED	773587008	X-linked intellectual disability, cardiomegaly, congestive heart failure syndrome (disorder)
Congenital Disease	SNOMED	773588003	Incomplete ossification of vertebra (disorder)
Congenital Disease	SNOMED	773610007	Chudley McCullough syndrome (disorder)
Congenital Disease	SNOMED	773621003	Intellectual disability, hypotonia, brachycephaly, pyloric stenosis, cryptorchidism syndrome (disorder)
Congenital Disease	SNOMED	773622005	Craniofacial dysplasia osteopenia syndrome (disorder)
Congenital Disease	SNOMED	773623000	Spigelian hernia with cryptorchidism syndrome (disorder)
Congenital Disease	SNOMED	773625007	Short stature, onychodysplasia, facial dysmorphism, hypotrichosis syndrome (disorder)

Congenital Disease	SNOMED	773626008	Joint contracture, webbed neck, micrognathia, hypoplastic nipple syndrome (disorder)
Congenital Disease	SNOMED	773627004	Porencephaly, microcephaly, bilateral congenital cataract syndrome (disorder)
Congenital Disease	SNOMED	773628009	Frontonasal dysplasia, severe microphthalmia, severe facial clefting syndrome (disorder)
Congenital Disease	SNOMED	773643006	Multiple congenital anomalies, hypotonia, seizures syndrome type 2 (disorder)
Congenital Disease	SNOMED	773647007	Nephrotic syndrome, deafness, pretibial epidermolysis bullosa syndrome (disorder)
Congenital Disease	SNOMED	773648002	Congenital cataract, hearing loss, severe developmental delay syndrome (disorder)
Congenital Disease	SNOMED	773665006	Hypogonadotropic hypogonadism, severe microcephaly, sensorineural hearing loss, dysmorphism syndrome (disorder)
Congenital Disease	SNOMED	773667003	Hypertelorism, preauricular sinus, punctual pits, deafness syndrome (disorder)
Congenital Disease	SNOMED	773670004	Distal Xq28 microduplication syndrome (disorder)
Congenital Disease	SNOMED	773672007	Lethal occipital encephalocele, skeletal dysplasia syndrome (disorder)
Congenital Disease	SNOMED	773673002	Hypoplasia of pancreas, intestinal atresia, hypoplasia of gallbladder syndrome (disorder)
Congenital Disease	SNOMED	773690008	Microcornea, posterior megalolenticonus, persistent fetal vasculature, coloboma syndrome (disorder)
Congenital Disease	SNOMED	773691007	Congenital erosive and vesicular dermatosis (disorder)
Congenital Disease	SNOMED	773692000	Late-onset localized junctional epidermolysis bullosa, intellectual disability syndrome (disorder)
Congenital Disease	SNOMED	773693005	Spondylo-megaepiphyseal-metaphyseal dysplasia (disorder)
Congenital Disease	SNOMED	773700005	Leukonychia totalis, acanthosis-nigricans-like lesions, abnormal hair syndrome (disorder)
Congenital Disease	SNOMED	773730002	Osteopetrosis hypogammaglobulinemia syndrome (disorder)
Congenital Disease	SNOMED	773733000	Humeroradioulnar synostosis (disorder)
Congenital Disease	SNOMED	773735007	Deafness with onychodystrophy syndrome (disorder)
Congenital Disease	SNOMED	773737004	Nephrocystin 3-related Meckel-like syndrome (disorder)
Congenital Disease	SNOMED	773749003	Genitopalatocardiac syndrome (disorder)
Congenital Disease	SNOMED	773750003	Flat face, microstomia, ear anomaly syndrome (disorder)
Congenital Disease	SNOMED	773768000	Emery Nelson syndrome (disorder)
Congenital Disease	SNOMED	773769008	Ataxia, photosensitivity, short stature syndrome (disorder)
Congenital Disease	SNOMED	773770009	Ankyloblepharon filiforme adnatum with imperforate anus syndrome (disorder)
Congenital Disease	SNOMED	773773006	Acrodysplasia scoliosis (disorder)
Congenital Disease	SNOMED	773984007	Piebald trait with neurologic defects syndrome (disorder)
Congenital Disease	SNOMED	773985008	Peripheral dysostosis (disorder)
Congenital Disease	SNOMED	773986009	Nevus epidermicus verrucosus with angiodysplasia and aneurysm syndrome (disorder)
Congenital Disease	SNOMED	774068004	AT-hook DNA binding motif containing 1-related intellectual disability, obstructive sleep apnea, mild dysmorphism syndrome (disorder)
Congenital Disease	SNOMED	774070008	Fibulin 1-related developmental delay, central nervous system anomaly, syndactyly syndrome (disorder)
Congenital Disease	SNOMED	774102003	Intellectual disability, obesity, prognathism, eye and skin anomalies syndrome (disorder)
Congenital Disease	SNOMED	77414002	Cheilognathoschisis (disorder)
Congenital Disease	SNOMED	774149004	Severe intellectual disability, progressive postnatal microcephaly, midline stereotypic hand movements syndrome (disorder)
Congenital Disease	SNOMED	774150004	Sacral agenesis, abnormal ossification of vertebral bodies, persistent notochordal canal syndrome (disorder)
Congenital Disease	SNOMED	774155009	Short stature, auditory canal atresia, mandibular hypoplasia, skeletal anomalies syndrome (disorder)
Congenital Disease	SNOMED	774203000	Intellectual disability, severe speech delay, mild dysmorphism syndrome (disorder)
Congenital Disease	SNOMED	774208009	Sebaceous nevus, central nervous system malformations, aplasia cutis congenita, limbal dermoid, pigmented nevus syndrome (disorder)
Congenital Disease	SNOMED	774209001	Didymosis aplasticosebacea (disorder)
Congenital Disease	SNOMED	774210006	Angora hair nevus (disorder)
Congenital Disease	SNOMED	774211005	Severe dermatitis, multiple allergies, metabolic wasting syndrome (disorder)
Congenital Disease	SNOMED	774212003	Microcornea, myopic chorioretinal atrophy, telecanthus syndrome (disorder)
Congenital Disease	SNOMED	77471004	Synotus (disorder)
Congenital Disease	SNOMED	77497005	3p partial trisomy syndrome (disorder)
Congenital Disease	SNOMED	77527000	9p partial trisomy syndrome (disorder)
Congenital Disease	SNOMED	77542002	Grebe syndrome (disorder)
Congenital Disease	SNOMED	775907000	Congenital pontocerebellar hypoplasia type 9 (disorder)
Congenital Disease	SNOMED	77593006	Congenital bronchiectasis (disorder)
Congenital Disease	SNOMED	77595004	Reduction deformity of lower limb (disorder)
Congenital Disease	SNOMED	77608001	Baller-Gerold syndrome (disorder)
Congenital Disease	SNOMED	7762009	Paraspadias (disorder)
Congenital Disease	SNOMED	776204008	Colobomatous microphthalmia, obesity, hypogenitalism, intellectual disability syndrome (disorder)

Congenital Disease	SNOMED	7763004	Dicephalus dipus tribrachius (disorder)
Congenital Disease	SNOMED	77696009	Double aortic valve (disorder)
Congenital Disease	SNOMED	77701002	Multiple malformation syndrome, moderate short stature, facial (disorder)
Congenital Disease	SNOMED	77761000	Congenital absence of ureter (disorder)
Congenital Disease	SNOMED	777998000	Temtamy preaxial brachydactyly syndrome (disorder)
Congenital Disease	SNOMED	778000002	3q26q27 microdeletion syndrome (disorder)
Congenital Disease	SNOMED	778005007	Duplication of pituitary gland (disorder)
Congenital Disease	SNOMED	778007004	12p12.1 microdeletion syndrome (disorder)
Congenital Disease	SNOMED	778008009	Fibroblast growth factor receptor 2-related bent bone dysplasia (disorder)
Congenital Disease	SNOMED	778009001	Blepharophimosis, intellectual disability syndrome, Verloes type (disorder)
Congenital Disease	SNOMED	778010006	Skin fragility, wooly hair, palmoplantar keratoderma syndrome (disorder)
Congenital Disease	SNOMED	778012003	Temple syndrome (disorder)
Congenital Disease	SNOMED	778021002	Microphthalmia, retinitis pigmentosa, foveoschisis, optic disc drusen syndrome (disorder)
Congenital Disease	SNOMED	778022009	Ehlers-Danlos syndrome due to tenascin-X deficiency (disorder)
Congenital Disease	SNOMED	778023004	Syndromic multisystem autoimmune disease due to itchy E3 ubiquitin protein ligase deficiency (disorder)
Congenital Disease	SNOMED	778026007	Lethal polymalformative syndrome Boissel type (disorder)
Congenital Disease	SNOMED	778043005	Ring chromosome 17 syndrome (disorder)
Congenital Disease	SNOMED	778044004	Primary non-essential cutis verticis gyrata (disorder)
Congenital Disease	SNOMED	778051008	Focal palmoplantar keratoderma with joint keratoses (disorder)
Congenital Disease	SNOMED	778062008	Diffuse palmoplantar keratoderma with painful fissures (disorder)
Congenital Disease	SNOMED	778067002	Brachytelephalangic chondrodysplasia punctata (disorder)
Congenital Disease	SNOMED	778068007	Autosomal recessive cutis laxa type 2B (disorder)
Congenital Disease	SNOMED	778070003	Autosomal dominant primary microcephaly (disorder)
Congenital Disease	SNOMED	778073001	3q26 microduplication syndrome (disorder)
Congenital Disease	SNOMED	77817004	Neu-Laxova syndrome (disorder)
Congenital Disease	SNOMED	77945009	Simple renal cyst (disorder)
Congenital Disease	SNOMED	77976003	Pygomelus (disorder)
Congenital Disease	SNOMED	77978002	Persistent left superior vena cava (disorder)
Congenital Disease	SNOMED	78018008	Complete phocomelia of upper limb (disorder)
Congenital Disease	SNOMED	78044008	Atlanto-occipital malformation (disorder)
Congenital Disease	SNOMED	7806002	Non-neurologic xeroderma pigmentosum (disorder)
Congenital Disease	SNOMED	78071008	Hydromicrocephaly (disorder)
Congenital Disease	SNOMED	780818005	Congenital fistula of pinna of ear (disorder)
Congenital Disease	SNOMED	780819002	Congenital cyst of pinna of ear (disorder)
Congenital Disease	SNOMED	780842009	Aortopulmonary coronary arterial course (disorder)
Congenital Disease	SNOMED	781065009	Transposition of inferior vena cava (disorder)
Congenital Disease	SNOMED	781159007	Congenital levorotation of heart (disorder)
Congenital Disease	SNOMED	781641005	Schwannomatosis (disorder)
Congenital Disease	SNOMED	78196008	Double mitral valve (disorder)
Congenital Disease	SNOMED	782194002	Median raphe cyst (disorder)
Congenital Disease	SNOMED	782332007	Congenital negative ulnar variant of wrist (disorder)
Congenital Disease	SNOMED	78250005	Ectopia cordis (disorder)
Congenital Disease	SNOMED	782669004	10q22.3q23.3 microduplication syndrome (disorder)
Congenital Disease	SNOMED	782673001	Distal monosomy 4q (disorder)
Congenital Disease	SNOMED	782674007	Distal monosomy 7p syndrome (disorder)
Congenital Disease	SNOMED	782676009	Distal trisomy 18q (disorder)
Congenital Disease	SNOMED	782689003	Congenital pseudoarthrosis of limb (disorder)
Congenital Disease	SNOMED	782691006	Maternal uniparental disomy of chromosome 21 (disorder)
Congenital Disease	SNOMED	782692004	Maternal uniparental disomy of chromosome 22 (disorder)
Congenital Disease	SNOMED	782694003	Non-distal monosomy 12q (disorder)
Congenital Disease	SNOMED	782698000	Congenital atresia of ostium of coronary artery (disorder)
Congenital Disease	SNOMED	782699008	Congenital stenosis of ostium of coronary artery (disorder)
Congenital Disease	SNOMED	782720005	Congenital pontocerebellar hypoplasia type 10 (disorder)
Congenital Disease	SNOMED	782722002	Global developmental delay, lung cysts, overgrowth, Wilms tumor syndrome (disorder)
Congenital Disease	SNOMED	782736007	Intellectual disability, facial dysmorphism syndrome due to SET domain containing 5 haploinsufficiency (disorder)
Congenital Disease	SNOMED	782739000	Male emopamil-binding protein disorder with neurological defect (disorder)
Congenital Disease	SNOMED	782753000	Intellectual disability, coarse face, macrocephaly, cerebellar hypotrophy syndrome (disorder)
Congenital Disease	SNOMED	782754006	Foveal hypoplasia, optic nerve decussation defect, anterior segment dysgenesis syndrome (disorder)
Congenital Disease	SNOMED	782755007	Primary microcephaly, mild intellectual disability, young-onset diabetes syndrome (disorder)
Congenital Disease	SNOMED	782758009	Finger hyperphalangy, toe anomalies, severe pectus excavatum syndrome (disorder)
Congenital Disease	SNOMED	782773005	Lethal arteriopathy syndrome due to fibulin-4 deficiency (disorder)
Congenital Disease	SNOMED	782780007	Marfanoid habitus, inguinal hernia, advanced bone age syndrome (disorder)
Congenital Disease	SNOMED	782781006	High bone mass osteogenesis imperfecta (disorder)
Congenital Disease	SNOMED	782782004	Autosomal recessive spondylometaphyseal dysplasia Megarbane type (disorder)
Congenital Disease	SNOMED	782783009	Oculoauriculofrontonasal syndrome (disorder)
Congenital Disease	SNOMED	782786001	X-linked calvarial hyperostosis (disorder)

Congenital Disease	SNOMED	782820003	Spondylometaphyseal dysplasia Czarny Ratajczak type (disorder)
Congenital Disease	SNOMED	782821004	Spondyloepimetaphyseal dysplasia Isidor type (disorder)
Congenital Disease	SNOMED	782825008	Primary microcephaly, epilepsy, permanent neonatal diabetes syndrome (disorder)
Congenital Disease	SNOMED	782877002	Xp22.13p22.2 duplication syndrome (disorder)
Congenital Disease	SNOMED	782878007	Autosomal recessive nail dysplasia (disorder)
Congenital Disease	SNOMED	782879004	Occipital pachygyria and polymicrogyria (disorder)
Congenital Disease	SNOMED	782882009	Chondrodysplasia with joint dislocations gPAPP type (disorder)
Congenital Disease	SNOMED	782884005	Pontine tegmental cap dysplasia (disorder)
Congenital Disease	SNOMED	782912001	Spondylometaphyseal dysplasia A4 type (disorder)
Congenital Disease	SNOMED	782913006	Spondylometaphyseal dysplasia, bowed forearms, facial dysmorphism syndrome (disorder)
Congenital Disease	SNOMED	782914000	Brachydactyly, short stature, retinitis pigmentosa syndrome (disorder)
Congenital Disease	SNOMED	782916003	Dermotrichic syndrome (disorder)
Congenital Disease	SNOMED	782937006	Extensor tendons of finger anomalies (disorder)
Congenital Disease	SNOMED	782940006	Dobrow syndrome (disorder)
Congenital Disease	SNOMED	782941005	Richieri Costa-da Silva syndrome (disorder)
Congenital Disease	SNOMED	782942003	Renal caliceal diverticuli and deafness syndrome (disorder)
Congenital Disease	SNOMED	782945001	Ophthalmoplegia, intellectual disability, lingua scrotalis syndrome (disorder)
Congenital Disease	SNOMED	782949007	Facial dysmorphism, anorexia, cachexia, eye and skin anomalies syndrome (disorder)
Congenital Disease	SNOMED	782951006	Thoracic dysplasia and hydrocephalus syndrome (disorder)
Congenital Disease	SNOMED	782957005	Ichthyosis (disorder)
Congenital Disease	SNOMED	783003009	Thoracomelic dysplasia (disorder)
Congenital Disease	SNOMED	783004003	Thin ribs, tubular bones, dysmorphism syndrome (disorder)
Congenital Disease	SNOMED	783005002	Severe microbrachycephaly, intellectual disability, athetoid cerebral palsy syndrome (disorder)
Congenital Disease	SNOMED	783011004	Persistent Eustachian valve (disorder)
Congenital Disease	SNOMED	783061008	Facial dysmorphism, developmental delay, behavioral abnormalities syndrome due to 10p11.21p12.31 microdeletion (disorder)
Congenital Disease	SNOMED	783089006	Macrocephaly, intellectual disability, autism syndrome (disorder)
Congenital Disease	SNOMED	783091003	46,XY gonadal dysgenesis, motor and sensory neuropathy syndrome (disorder)
Congenital Disease	SNOMED	783092005	46,XY disorder of sex development, adrenal insufficiency due to cytochrome P450 family 11 subfamily A member 1 deficiency (disorder)
Congenital Disease	SNOMED	783095007	Aplasia of uterine cervix (disorder)
Congenital Disease	SNOMED	783096008	Subaortic stenosis and short stature syndrome (disorder)
Congenital Disease	SNOMED	783097004	Stickler syndrome type 3 (disorder)
Congenital Disease	SNOMED	783136007	Autosomal dominant focal non-epidermolytic palmoplantar keratoderma with plantar blistering (disorder)
Congenital Disease	SNOMED	783137003	Radial deficiency, tibial hypoplasia syndrome (disorder)
Congenital Disease	SNOMED	783140003	Pelvic dysplasia, arthrogryposis of lower limbs syndrome (disorder)
Congenital Disease	SNOMED	78314001	Osteogenesis imperfecta (disorder)
Congenital Disease	SNOMED	783143001	Noonan syndrome-like disorder with juvenile myelomonocytic leukemia (disorder)
Congenital Disease	SNOMED	783149002	Mosaic genome-wide paternal uniparental disomy (disorder)
Congenital Disease	SNOMED	783156008	Fibular aplasia, tibial campomelia, oligo-syndactyly syndrome (disorder)
Congenital Disease	SNOMED	783159001	Holzgreve syndrome (disorder)
Congenital Disease	SNOMED	78317008	XXXY syndrome (disorder)
Congenital Disease	SNOMED	783181006	Cloverleaf skull, asphyxiating thoracic dysplasia syndrome (disorder)
Congenital Disease	SNOMED	78320000	Quadricuspid cardiac valve (disorder)
Congenital Disease	SNOMED	783204009	Ankyloblepharon filiforme adnatum with cleft palate syndrome (disorder)
Congenital Disease	SNOMED	783230008	Agenesis of body of uterus (disorder)
Congenital Disease	SNOMED	783231007	Aplasia of body of uterus (disorder)
Congenital Disease	SNOMED	783246000	Megalocornea, spherophakia, secondary glaucoma syndrome (disorder)
Congenital Disease	SNOMED	783400002	Complete cleft of hard palate (disorder)
Congenital Disease	SNOMED	783407004	Incomplete cleft palate (disorder)
Congenital Disease	SNOMED	783409001	Ectopic artery (disorder)
Congenital Disease	SNOMED	783551005	Ichthyosis, short stature, brachydactyly, microspherophakia syndrome (disorder)
Congenital Disease	SNOMED	783553008	Syndactyly, camptodactyly and clinodactyly of fifth fingers, bifid toes syndrome (disorder)
Congenital Disease	SNOMED	783562005	Syndactyly, nystagmus syndrome due to 2q31.1 microduplication (disorder)
Congenital Disease	SNOMED	783619003	Dual specificity tyrosine phosphorylation regulated kinase 1A-related intellectual disability syndrome due to 21q22.13q22.2 microdeletion (disorder)
Congenital Disease	SNOMED	783629005	Congenital aneurysm of cerebral artery (disorder)
Congenital Disease	SNOMED	783630000	Congenital aneurysm of precerebral artery (disorder)
Congenital Disease	SNOMED	783700001	Syndactyly, polydactyly, ear lobe syndrome (disorder)
Congenital Disease	SNOMED	783701002	Port-wine nevi, mega cisterna magna, hydrocephalus syndrome (disorder)
Congenital Disease	SNOMED	783703004	White matter hypoplasia, corpus callosum agenesis, intellectual disability syndrome (disorder)
Congenital Disease	SNOMED	783718003	Paternal uniparental disomy of chromosome X (disorder)
Congenital Disease	SNOMED	78372005	Intrahepatic gallbladder (disorder)
Congenital Disease	SNOMED	783724009	Fetal spina bifida (disorder)

Congenital Disease	SNOMED	783726006	Fetal hydrocephalus (disorder)
Congenital Disease	SNOMED	783735004	Maternal uniparental disomy of chromosome X (disorder)
Congenital Disease	SNOMED	783737007	Hirschsprung disease, ganglioneuroblastoma syndrome (disorder)
Congenital Disease	SNOMED	783738002	Heart defect, tongue hamartoma, polysyndactyly syndrome (disorder)
Congenital Disease	SNOMED	783742004	Conductive hearing loss, malformation of external ear syndrome (disorder)
Congenital Disease	SNOMED	783773000	Congenital abnormal number of ostium of coronary artery (disorder)
Congenital Disease	SNOMED	783774006	External auditory canal atresia, vertical talus, hypertelorism syndrome (disorder)
Congenital Disease	SNOMED	783789002	Autosomal recessive brachyolmia (disorder)
Congenital Disease	SNOMED	78393008	Congenital hypoplasia of finger (disorder)
Congenital Disease	SNOMED	784006008	Spondylometaphyseal dysplasia (disorder)
Congenital Disease	SNOMED	784010006	Otopalatodigital syndrome spectrum disorder (disorder)
Congenital Disease	SNOMED	784344009	Cortical dysgenesis with pontocerebellar hypoplasia due to tubulin beta 3 class III mutation (disorder)
Congenital Disease	SNOMED	784349004	Cutis laxa with severe pulmonary, gastrointestinal and urinary anomalies (disorder)
Congenital Disease	SNOMED	784350004	Craniorhiny (disorder)
Congenital Disease	SNOMED	784351000	Antecubital pterygium syndrome (disorder)
Congenital Disease	SNOMED	784353002	Pulmonary valve agenesis, intact ventricular septum, persistent ductus arteriosus syndrome (disorder)
Congenital Disease	SNOMED	784381008	Autosomal recessive cutis laxa type 2A (disorder)
Congenital Disease	SNOMED	78485007	Acyanotic congenital heart disease (disorder)
Congenital Disease	SNOMED	78495000	Cleft leaflet of mitral valve (disorder)
Congenital Disease	SNOMED	785299009	Cobblestone lissencephaly without muscular or ocular involvement (disorder)
Congenital Disease	SNOMED	785303004	Multiple congenital anomalies, hypotonia, seizures syndrome (disorder)
Congenital Disease	SNOMED	785306007	Lissencephaly co-occurrent with congenital cerebellar hypoplasia type E (disorder)
Congenital Disease	SNOMED	785307003	Lissencephaly co-occurrent with congenital cerebellar hypoplasia type A (disorder)
Congenital Disease	SNOMED	78560000	Accessory hepatic duct (disorder)
Congenital Disease	SNOMED	78572006	Neurocutaneous syndrome (disorder)
Congenital Disease	SNOMED	785725008	Palmoplantar keratoderma, spastic paralysis syndrome (disorder)
Congenital Disease	SNOMED	785808002	Aneurysm osteoarthritis syndrome (disorder)
Congenital Disease	SNOMED	786039009	Arthrogryposis and ectodermal dysplasia syndrome (disorder)
Congenital Disease	SNOMED	786041005	Congenital systemic arteriovenous fistula (disorder)
Congenital Disease	SNOMED	786076007	Congenital pit of optic disc (disorder)
Congenital Disease	SNOMED	78675000	Stickler syndrome (disorder)
Congenital Disease	SNOMED	78693004	Congenital hypoplasia of part of brain (disorder)
Congenital Disease	SNOMED	787093004	Developmental delay, facial dysmorphism syndrome due to mediator complex subunit 13 like deficiency (disorder)
Congenital Disease	SNOMED	787094005	Segmental progressive overgrowth syndrome with fibroadipose hyperplasia (disorder)
Congenital Disease	SNOMED	787171006	21q22.11q22.12 microdeletion syndrome (disorder)
Congenital Disease	SNOMED	78728005	Gastrothoracopagus (disorder)
Congenital Disease	SNOMED	78740005	Complete monosomy 21 (disorder)
Congenital Disease	SNOMED	787407003	Muenke syndrome (disorder)
Congenital Disease	SNOMED	787408008	Osteopathia striata, pigmentary dermopathy, white forelock syndrome (disorder)
Congenital Disease	SNOMED	787411009	Monosomy 22 syndrome (disorder)
Congenital Disease	SNOMED	787413007	Bifid nose (disorder)
Congenital Disease	SNOMED	78745000	Urticaria pigmentosa (disorder)
Congenital Disease	SNOMED	787472005	Congenital rhabdomyomatous mesenchymal hamartoma (disorder)
Congenital Disease	SNOMED	78784005	Amyelia (disorder)
Congenital Disease	SNOMED	788533006	Congenital abnormality of atrium (disorder)
Congenital Disease	SNOMED	788584007	Blepharophimosis, intellectual disability syndrome (disorder)
Congenital Disease	SNOMED	788868003	Arteriovenous malformation of uterus (disorder)
Congenital Disease	SNOMED	788869006	Atresia of esophagus co-occurrent with esophagobronchial fistula (disorder)
Congenital Disease	SNOMED	788875002	Bilateral cleft lip and bilateral cleft of alveolar process of maxilla (disorder)
Congenital Disease	SNOMED	789097007	Agenesis of artery (disorder)
Congenital Disease	SNOMED	789120001	Neurenteric cyst (disorder)
Congenital Disease	SNOMED	789156003	Focal facial dermal dysplasia (disorder)
Congenital Disease	SNOMED	789157007	Focal facial dermal dysplasia type I (disorder)
Congenital Disease	SNOMED	789159005	Focal facial dermal dysplasia type II (disorder)
Congenital Disease	SNOMED	789160000	Focal facial dermal dysplasia type III (disorder)
Congenital Disease	SNOMED	789161001	Focal facial dermal dysplasia type IV (disorder)
Congenital Disease	SNOMED	789228000	Congenital anomaly of anterior portion of neck (disorder)
Congenital Disease	SNOMED	7903009	Hallermann-Streiff syndrome (disorder)
Congenital Disease	SNOMED	79037006	Accessory pancreas (disorder)
Congenital Disease	SNOMED	79120002	Congenital elevation of scapula (disorder)
Congenital Disease	SNOMED	79168008	Congenital genu varum (disorder)
Congenital Disease	SNOMED	79177001	Congenital absence of tibia (disorder)
Congenital Disease	SNOMED	79191007	Congenital anomaly of muscle AND/OR tendon (disorder)
Congenital Disease	SNOMED	7921007	Congenital deformity of clavicle (disorder)
Congenital Disease	SNOMED	79214007	Bifid patella (disorder)

Congenital Disease	SNOMED	79244005	Lumbosacral prespondylolisthesis (disorder)
Congenital Disease	SNOMED	79261008	Van der Woude syndrome (disorder)
Congenital Disease	SNOMED	79407008	Asymmetric talipes (disorder)
Congenital Disease	SNOMED	79410001	Congenital cataract (disorder)
Congenital Disease	SNOMED	79439001	Congenital anomaly of aortic arch (disorder)
Congenital Disease	SNOMED	79468000	Familial benign pemphigus (disorder)
Congenital Disease	SNOMED	79510004	Congenital displacement of esophagus (disorder)
Congenital Disease	SNOMED	79552009	Congenital duplication of liver (disorder)
Congenital Disease	SNOMED	79656000	Anomaly of chromosome pair 19 (disorder)
Congenital Disease	SNOMED	79665007	Wildervanck syndrome (disorder)
Congenital Disease	SNOMED	79702003	Monocephalus tetrapus dibrachius (disorder)
Congenital Disease	SNOMED	79788002	Atresia of urinary meatus (disorder)
Congenital Disease	SNOMED	79807003	Talipes valgus (disorder)
Congenital Disease	SNOMED	79855003	Congenital junctional epidermolysis bullosa (disorder)
Congenital Disease	SNOMED	7991000119102	Congenital dilatation of aortic [REDACTED] (disorder)
Congenital Disease	SNOMED	79969004	Congenital hallux varus (disorder)
Congenital Disease	SNOMED	79977000	Congenital atresia of broad ligament (disorder)
Congenital Disease	SNOMED	80281008	Cleft lip (disorder)
Congenital Disease	SNOMED	8032007	Congenital anomaly of thyroid cartilage (disorder)
Congenital Disease	SNOMED	80387009	Roger's disease (disorder)
Congenital Disease	SNOMED	80432009	Porokeratosis of Mibelli (disorder)
Congenital Disease	SNOMED	80446009	Complete bilateral cleft lip (disorder)
Congenital Disease	SNOMED	80533002	Uterus bicornuatus vetularum (disorder)
Congenital Disease	SNOMED	80651009	Aicardi's syndrome (disorder)
Congenital Disease	SNOMED	80667003	Juvenile pelvis (disorder)
Congenital Disease	SNOMED	80700005	Floating gallbladder (disorder)
Congenital Disease	SNOMED	80712009	Congenital spondylolysis of lumbosacral region (disorder)
Congenital Disease	SNOMED	80773006	Escobar syndrome (disorder)
Congenital Disease	SNOMED	80797002	Congenital stricture of vesicourethral orifice (disorder)
Congenital Disease	SNOMED	80825009	Congenital hypoplasia of lung (disorder)
Congenital Disease	SNOMED	80880002	Omphalomesenteric duct cyst (disorder)
Congenital Disease	SNOMED	81042008	Congenital anomaly of spinal cord (disorder)
Congenital Disease	SNOMED	81103005	Congenital atresia of vein (disorder)
Congenital Disease	SNOMED	81107006	Congenital diverticulum of bladder (disorder)
Congenital Disease	SNOMED	81208006	Ectrodactyly (disorder)
Congenital Disease	SNOMED	81304006	7q partial monosomy (disorder)
Congenital Disease	SNOMED	81307004	Ischiomelus (disorder)
Congenital Disease	SNOMED	81438002	Anomaly of chromosome Y (disorder)
Congenital Disease	SNOMED	81539007	Metatarsus primus varus (disorder)
Congenital Disease	SNOMED	81577001	Congenital anomaly of inferior vena cava (disorder)
Congenital Disease	SNOMED	81604003	Mulibrey nanism syndrome (disorder)
Congenital Disease	SNOMED	816067005	Diabetes, hypogonadism, deafness, intellectual disability syndrome (disorder)
Congenital Disease	SNOMED	816068000	Periventricular nodular heterotopia (disorder)
Congenital Disease	SNOMED	81678004	Ring chromosome 4 syndrome (disorder)
Congenital Disease	SNOMED	81771002	Opitz-Frias syndrome (disorder)
Congenital Disease	SNOMED	81780002	Beckwith-Wiedemann syndrome (disorder)
Congenital Disease	SNOMED	81793007	Polydactyly of fingers (disorder)
Congenital Disease	SNOMED	817968741000119105	Situs inversus of optic disc (disorder)
Congenital Disease	SNOMED	818949005	Congenital absence of body of uterus (disorder)
Congenital Disease	SNOMED	818951009	Congenital respiratory biliary fistula (disorder)
Congenital Disease	SNOMED	818959006	Trichorhinophalangeal syndrome type 1 and 3 (disorder)
Congenital Disease	SNOMED	818963004	Posterior hypospadias (disorder)
Congenital Disease	SNOMED	81990004	Cor biloculare (disorder)
Congenital Disease	SNOMED	82007007	Thoracoparacephalus (disorder)
Congenital Disease	SNOMED	82058009	Myelocele (disorder)
Congenital Disease	SNOMED	82203000	Treacher Collins syndrome (disorder)
Congenital Disease	SNOMED	822975003	Congenital arteriovenous malformation of duodenum (disorder)
Congenital Disease	SNOMED	8234004	XY females (disorder)
Congenital Disease	SNOMED	82354003	Multiple system malformation syndrome (disorder)
Congenital Disease	SNOMED	82393007	Giant esophagus (disorder)
Congenital Disease	SNOMED	82520000	Embryonic cyst of Gartner's duct (disorder)
Congenital Disease	SNOMED	82525005	Congenital cystic kidney disease (disorder)
Congenital Disease	SNOMED	82635007	Accessory urethra (disorder)
Congenital Disease	SNOMED	82642007	Segmental uterine aplasia (disorder)
Congenital Disease	SNOMED	82663009	Osteopathia striata (disorder)
Congenital Disease	SNOMED	82699004	Dyggve-Melchior-Clausen syndrome (disorder)
Congenital Disease	SNOMED	827078006	Eisenmenger ventricular septal defect (disorder)
Congenital Disease	SNOMED	82751009	7p partial trisomy (disorder)
Congenital Disease	SNOMED	82779003	Late fontanel closure (finding)
Congenital Disease	SNOMED	82785005	Astragaloscaphoid synostosis (disorder)
Congenital Disease	SNOMED	82820009	Congenital anomaly of prostate (disorder)
Congenital Disease	SNOMED	82837002	Kenny syndrome (disorder)
Congenital Disease	SNOMED	82949000	Congenital absence of pancreas (disorder)

Congenital Disease	SNOMED	829974003	Mosaic trisomy 1 syndrome (disorder)
Congenital Disease	SNOMED	830019003	Choristoma of right eye proper (disorder)
Congenital Disease	SNOMED	830020009	Choristoma of left eye proper (disorder)
Congenital Disease	SNOMED	830021008	Choristoma of eye proper (disorder)
Congenital Disease	SNOMED	8301004	Caudal regression syndrome (disorder)
Congenital Disease	SNOMED	83015004	Saethre-Chotzen syndrome (disorder)
Congenital Disease	SNOMED	83028006	Congenital partial absence of alimentary tract (disorder)
Congenital Disease	SNOMED	83031000119105	Congenital malformation of nasal septum (disorder)
Congenital Disease	SNOMED	83119008	Congenital insufficiency of tricuspid valve (disorder)
Congenital Disease	SNOMED	83145004	Abnormal dermatoglyphic pattern (disorder)
Congenital Disease	SNOMED	83190008	Congenital absence of adrenal gland (disorder)
Congenital Disease	SNOMED	83228001	Congenital anomaly of spermatic cord (disorder)
Congenital Disease	SNOMED	83330001	Patent ductus arteriosus (disorder)
Congenital Disease	SNOMED	83470009	Ehlers-Danlos syndrome, type 1 (disorder)
Congenital Disease	SNOMED	83546008	Congenital anomaly of breast (disorder)
Congenital Disease	SNOMED	83579008	Mixed gonadal dysgenesis (disorder)
Congenital Disease	SNOMED	83585001	14q partial proximal trisomy syndrome (disorder)
Congenital Disease	SNOMED	83586000	Ehlers-Danlos syndrome, dysfibronectinemic (disorder)
Congenital Disease	SNOMED	83714006	Congenital microgastria (disorder)
Congenital Disease	SNOMED	83721006	6p partial trisomy syndrome (disorder)
Congenital Disease	SNOMED	83799000	Corrected transposition of great vessels (disorder)
Congenital Disease	SNOMED	838296001	Buphthalmos of bilateral eyes (finding)
Congenital Disease	SNOMED	838300000	Blue sclera of bilateral eyes (disorder)
Congenital Disease	SNOMED	838310009	Camptodactyly of bilateral toes (disorder)
Congenital Disease	SNOMED	838313006	Camptodactyly of bilateral fingers (disorder)
Congenital Disease	SNOMED	838325009	46 XY disorder of sex development due to maternal ingestion of estrogen (disorder)
Congenital Disease	SNOMED	838326005	46,XX disorder of sex development due to maternal Krukenberg neoplasm (disorder)
Congenital Disease	SNOMED	838327001	46,XX disorder of sex development due to maternal arrhenoblastoma (disorder)
Congenital Disease	SNOMED	838328006	46,XX disorder of sex development due to maternal androluteoma (disorder)
Congenital Disease	SNOMED	838329003	46,XX disorder of sex development due to maternal adrenal neoplasm (disorder)
Congenital Disease	SNOMED	838333005	Atresia of mitral valve with absent atrioventricular connection (disorder)
Congenital Disease	SNOMED	838334004	Ablepharon of bilateral eyelids (disorder)
Congenital Disease	SNOMED	838336002	46,XY disorder of sex development due to maternal ingestion of progestogen (disorder)
Congenital Disease	SNOMED	838339009	Basal encephalocele (disorder)
Congenital Disease	SNOMED	838362006	Aplasia cutis congenita of limb (disorder)
Congenital Disease	SNOMED	838364007	Aortic aneurysm due to Loeys-Dietz syndrome (disorder)
Congenital Disease	SNOMED	838368005	Congenital generalized hypertrichosis (disorder)
Congenital Disease	SNOMED	838370001	Agenesis of bilateral lungs (disorder)
Congenital Disease	SNOMED	83923004	Familial interstitial nephritis (disorder)
Congenital Disease	SNOMED	840426005	Developmental anomaly of bilateral pleurae (disorder)
Congenital Disease	SNOMED	840451006	Clinodactyly of bilateral toes (disorder)
Congenital Disease	SNOMED	840456001	Coloboma of bilateral maculae (disorder)
Congenital Disease	SNOMED	840458000	Coloboma of bilateral lenses (disorder)
Congenital Disease	SNOMED	840469002	Deficient mural leaflet of left ventricular component of common atrioventricular valve (disorder)
Congenital Disease	SNOMED	840471002	Hydrocephalus due to Dandy-Walker malformation (disorder)
Congenital Disease	SNOMED	840475006	Congenital hypertrophy of lateral fold of hallux (disorder)
Congenital Disease	SNOMED	840476007	Congenital instability of spine (disorder)
Congenital Disease	SNOMED	840477003	Congenital hypertrophy of bilateral lower limbs (disorder)
Congenital Disease	SNOMED	840478008	Congenital distichiasis of bilateral eyelashes (disorder)
Congenital Disease	SNOMED	840479000	Congenital entropion of bilateral eyelids (disorder)
Congenital Disease	SNOMED	840480002	Congenital crumpled ear (disorder)
Congenital Disease	SNOMED	840481003	Congenital dislocation of bilateral knees (disorder)
Congenital Disease	SNOMED	840482005	Congenital anomaly of lobe of ear (disorder)
Congenital Disease	SNOMED	840483000	Congenital coronal cleft of vertebra (disorder)
Congenital Disease	SNOMED	840485007	Cornea plana of bilateral corneas (disorder)
Congenital Disease	SNOMED	840486008	Congenital abnormal retraction of bilateral eyelids (disorder)
Congenital Disease	SNOMED	840487004	Congenital stenosis of spinal canal (disorder)
Congenital Disease	SNOMED	840488009	Congenital regurgitation of truncal valve (disorder)
Congenital Disease	SNOMED	840489001	Congenital opacity of bilateral corneas (disorder)
Congenital Disease	SNOMED	840490005	Congenital refluxing megaureter (disorder)
Congenital Disease	SNOMED	840491009	Congenital malalignment of great toenail (disorder)
Congenital Disease	SNOMED	840492002	Microphthalmos co-occurrent with congenital ocular coloboma of bilateral eyes (disorder)
Congenital Disease	SNOMED	840493007	Congenital lobar emphysema of bilateral lungs (disorder)
Congenital Disease	SNOMED	840494001	Congenital macrophthalmos of bilateral eyes (disorder)
Congenital Disease	SNOMED	840495000	Common atrioventricular junction with spontaneous fibrous closure of atrioventricular septal defect (disorder)

Congenital Disease	SNOMED	840497008	Atrioventricular septal defect, atrial and ventricular components with common atrioventricular valve with unbalanced commitment of valve to left ventricle (disorder)
Congenital Disease	SNOMED	840505007	Down syndrome co-occurrent with leukemoid reaction associated transient neonatal pustulosis (disorder)
Congenital Disease	SNOMED	840507004	Double outlet right ventricle with subaortic ventricular septal defect without pulmonary stenosis (disorder)
Congenital Disease	SNOMED	840508009	Congenital absence of seminal vesicle (disorder)
Congenital Disease	SNOMED	840510006	Congenital absence of epiglottis (disorder)
Congenital Disease	SNOMED	840512003	Congenital absence of all bilateral toes (disorder)
Congenital Disease	SNOMED	840517009	Atrioventricular septal defect, atrial and ventricular components with common atrioventricular valve with unbalanced commitment of valve to right ventricle (disorder)
Congenital Disease	SNOMED	841002	Congenital absence of skull bone (disorder)
Congenital Disease	SNOMED	84150000	Arteriovenous malformation of liver (disorder)
Congenital Disease	SNOMED	84296002	Congenital atresia of small intestine (disorder)
Congenital Disease	SNOMED	8441000119103	Balanced autosomal translocation in normal individual (disorder)
Congenital Disease	SNOMED	84449007	Congenital accessory skin tag (disorder)
Congenital Disease	SNOMED	84461004	Exencephaly (disorder)
Congenital Disease	SNOMED	8447006	Congenital anomaly of skeletal bone (disorder)
Congenital Disease	SNOMED	84557007	Bifid tongue (disorder)
Congenital Disease	SNOMED	84581000	Cloacal disease (disorder)
Congenital Disease	SNOMED	84589003	Pygodidymus (disorder)
Congenital Disease	SNOMED	84598000	Polysyndactyly (disorder)
Congenital Disease	SNOMED	84648007	Acardius (disorder)
Congenital Disease	SNOMED	84711007	Anomaly of chromosome pair 4 (disorder)
Congenital Disease	SNOMED	84773003	Congenital anomaly of lower limb (disorder)
Congenital Disease	SNOMED	84873005	Dural arteriovenous malformation (disorder)
Congenital Disease	SNOMED	84918006	Congenital absence of radius (disorder)
Congenital Disease	SNOMED	84974003	Congenital volvulus of stomach (disorder)
Congenital Disease	SNOMED	85029000	Congenital anomaly of fallopian tubes (disorder)
Congenital Disease	SNOMED	85081000	Common arterial trunk and widely separate origin of pulmonary arteries (disorder)
Congenital Disease	SNOMED	85090007	Dextrocardia/situs inversus finding (finding)
Congenital Disease	SNOMED	85150007	Uterus bicameratus vetularum (disorder)
Congenital Disease	SNOMED	85280007	Congenital hammer toe (disorder)
Congenital Disease	SNOMED	85437001	Congenital hypoplasia of testis (disorder)
Congenital Disease	SNOMED	85445006	Dicephalus dipus tetrabrachius (disorder)
Congenital Disease	SNOMED	85589009	Radial aplasia-thrombocytopenia syndrome (disorder)
Congenital Disease	SNOMED	85630009	Craniopagus occipitalis (disorder)
Congenital Disease	SNOMED	85641006	Hemianencephaly (disorder)
Congenital Disease	SNOMED	85671000119102	Hallux valgus due to metatarsus primus varus (disorder)
Congenital Disease	SNOMED	8569001	Syncephalus (disorder)
Congenital Disease	SNOMED	85697009	Supernumerary eye muscle (disorder)
Congenital Disease	SNOMED	85700008	Microhepatia (disorder)
Congenital Disease	SNOMED	85836000	Congenital duodenal obstruction due to annular pancreas (disorder)
Congenital Disease	SNOMED	8587003	Congenital diverticulum of colon (disorder)
Congenital Disease	SNOMED	85901000	Megacalycosis (disorder)
Congenital Disease	SNOMED	85995004	Autosomal recessive hereditary disorder (disorder)
Congenital Disease	SNOMED	860820000	Hypoplasia of cranial sinus (disorder)
Congenital Disease	SNOMED	860951008	Congenital anomaly of cauda equina (disorder)
Congenital Disease	SNOMED	8611000119100	Complex craniosynostosis (disorder)
Congenital Disease	SNOMED	86204009	Immotile cilia syndrome (disorder)
Congenital Disease	SNOMED	86252004	Agenesis of pulmonary artery (disorder)
Congenital Disease	SNOMED	86268005	Achondroplasia (disorder)
Congenital Disease	SNOMED	86280002	Thoracomelus (disorder)
Congenital Disease	SNOMED	86299006	Tetralogy of Fallot (disorder)
Congenital Disease	SNOMED	8634009	Distichiasis-lymphedema syndrome (disorder)
Congenital Disease	SNOMED	86345004	Bertolotti's syndrome (disorder)
Congenital Disease	SNOMED	8641000119101	Polysplenia heterotaxy syndrome (disorder)
Congenital Disease	SNOMED	86426007	Dominant autosomal hereditary disorder, complete penetrance (disorder)
Congenital Disease	SNOMED	86463003	Solitary multilocular renal cyst (disorder)
Congenital Disease	SNOMED	86470003	Osteogenesis imperfecta, recessive perinatal lethal (disorder)
Congenital Disease	SNOMED	8649001	Congenital anomaly of thoracic cage (disorder)
Congenital Disease	SNOMED	86507001	Congenital absence of gallbladder (disorder)
Congenital Disease	SNOMED	86527000	Hypoplasia of eye (disorder)
Congenital Disease	SNOMED	8654005	Ectodermal dysplasia (disorder)
Congenital Disease	SNOMED	86587003	Platybasia (disorder)
Congenital Disease	SNOMED	86595004	Congenital mesocolic hernia (disorder)
Congenital Disease	SNOMED	866053004	Middle interhemispheric variant of holoprosencephaly (disorder)
Congenital Disease	SNOMED	866088004	Microcornea of bilateral eyes (disorder)
Congenital Disease	SNOMED	866089007	Megalocornea of bilateral eyes (disorder)
Congenital Disease	SNOMED	86610004	Frontonasal dysplasia sequence (disorder)
Congenital Disease	SNOMED	866118008	Left hand pattern ventricular topology (disorder)
Congenital Disease	SNOMED	86715000	Beta zero thalassemia (disorder)

Congenital Disease	SNOMED	86818009	Congenital ankylosis of elbow (disorder)
Congenital Disease	SNOMED	86993003	Congenital anomaly of rectum (disorder)
Congenital Disease	SNOMED	86996006	Macronychia (disorder)
Congenital Disease	SNOMED	86997002	Ring chromosome 10 syndrome (disorder)
Congenital Disease	SNOMED	87006007	Dominant autosomal hereditary disorder, incomplete penetrance (disorder)
Congenital Disease	SNOMED	870323006	Obstructed anomalous pulmonary venous pathway (disorder)
Congenital Disease	SNOMED	87047005	Congenital cyst of esophagus (disorder)
Congenital Disease	SNOMED	87093008	Congenital urethrorectal fistula (disorder)
Congenital Disease	SNOMED	87119009	Congenital cystic lung (disorder)
Congenital Disease	SNOMED	8712002	Congenital subaortic stenosis due to fibromuscular shelf (disorder)
Congenital Disease	SNOMED	87142002	Congenital absence of appendix (disorder)
Congenital Disease	SNOMED	871579003	Perimembranous inlet ventricular septal defect with atrioventricular septal malalignment (disorder)
Congenital Disease	SNOMED	871581001	Inferior muscular trabecular ventricular septal defect (disorder)
Congenital Disease	SNOMED	871583003	Congenital anomaly of left-sided atrioventricular valve in double inlet ventricle (disorder)
Congenital Disease	SNOMED	871585005	Congenital anomaly of right-sided atrioventricular valve in double inlet ventricle (disorder)
Congenital Disease	SNOMED	871588007	Innominate artery compression syndrome (disorder)
Congenital Disease	SNOMED	871594004	Congenital stenosis of truncal valve (disorder)
Congenital Disease	SNOMED	871597006	Congenital dysplasia of aortic valve (disorder)
Congenital Disease	SNOMED	871599009	Double outlet right ventricle with subaortic or doubly committed ventricular septal defect and pulmonary stenosis Fallot type (disorder)
Congenital Disease	SNOMED	871600007	Outlet ventricular septal defect with posteriorly malaligned outlet septum (disorder)
Congenital Disease	SNOMED	871601006	Double outlet right ventricle with subaortic ventricular septal defect and pulmonary stenosis Fallot type (disorder)
Congenital Disease	SNOMED	871602004	Perimembranous outlet ventricular septal defect with anteriorly malaligned outlet septum (disorder)
Congenital Disease	SNOMED	871603009	Transposition of great arteries with concordant atrioventricular connections and ventricular septal defect (disorder)
Congenital Disease	SNOMED	871604003	Doubly committed juxta-arterial outlet ventricular septal defect with perimembranous extension and posteriorly malaligned outlet septum (disorder)
Congenital Disease	SNOMED	871605002	Transposition of great arteries with concordant atrioventricular connections and ventricular septal defect and left ventricular outflow tract obstruction (disorder)
Congenital Disease	SNOMED	871606001	Outlet ventricular septal defect with anteriorly malaligned outlet septum (disorder)
Congenital Disease	SNOMED	871607005	Doubly committed juxta-arterial outlet ventricular septal defect with perimembranous extension and anteriorly malaligned outlet septum (disorder)
Congenital Disease	SNOMED	871609008	Doubly committed juxta-arterial outlet ventricular septal defect with anteriorly malaligned outlet septum (disorder)
Congenital Disease	SNOMED	871610003	Doubly committed juxta-arterial outlet ventricular septal defect with perimembranous extension (disorder)
Congenital Disease	SNOMED	871611004	Doubly committed juxta-arterial outlet ventricular septal defect with posteriorly malaligned outlet septum (disorder)
Congenital Disease	SNOMED	871612006	Anterior muscular trabecular ventricular septal defect (disorder)
Congenital Disease	SNOMED	871613001	Right hand pattern ventricular topology (disorder)
Congenital Disease	SNOMED	871614007	Abnormal intrapericardial course of great arteries (disorder)
Congenital Disease	SNOMED	871616009	Atrial situs inversus (disorder)
Congenital Disease	SNOMED	871618005	Congenital dysplasia of truncal valve (disorder)
Congenital Disease	SNOMED	871620008	Perimembranous inlet ventricular septal defect (disorder)
Congenital Disease	SNOMED	871622000	Common atrioventricular valve with unbalanced commitment of valve to ventricle (disorder)
Congenital Disease	SNOMED	871624004	Common atrioventricular valve with unbalanced commitment of valve to right ventricle (disorder)
Congenital Disease	SNOMED	871625003	Intermediate atrioventricular septal defect with atrial and ventricular components and separate atrioventricular valves (disorder)
Congenital Disease	SNOMED	871626002	Syndactyly of toes with fusion of bones of toes of bilateral feet (disorder)
Congenital Disease	SNOMED	871627006	Common atrium with common atrioventricular junction (disorder)
Congenital Disease	SNOMED	871630004	Atrioventricular septal defect with ventricular imbalance (disorder)
Congenital Disease	SNOMED	871636005	Doubly committed juxta-arterial ventricular septal defect with anteriorly malaligned fibrous outlet septum and muscular postero-inferior rim (disorder)
Congenital Disease	SNOMED	871643004	Atrioventricular septal defect with balanced ventricles (disorder)
Congenital Disease	SNOMED	871645006	Common atrium with separate arterioventricular junctions (disorder)
Congenital Disease	SNOMED	871647003	Common arterial trunk with pulmonary dominance (disorder)
Congenital Disease	SNOMED	871648008	Perimembranous outlet ventricular septal defect with posteriorly malaligned outlet septum (disorder)
Congenital Disease	SNOMED	871650000	Common arterial trunk with pulmonary dominance and aortic coarctation (disorder)
Congenital Disease	SNOMED	871652008	Mitral atresia with imperforate mitral valve (disorder)
Congenital Disease	SNOMED	871654009	Congenital hypoplasia of annulus fibrosus of aorta (disorder)

Congenital Disease	SNOMED	871655005	Tricuspid atresia with absent right atrioventricular connection (disorder)
Congenital Disease	SNOMED	871658007	Muscular ventricular septal defect opening to right ventricular inlet (disorder)
Congenital Disease	SNOMED	871659004	Double inlet to solitary ventricle of indeterminate morphology (disorder)
Congenital Disease	SNOMED	871660009	Congenital anomaly of left ventricle (disorder)
Congenital Disease	SNOMED	871666003	Atypical left ventricular component of common atrioventricular valve (disorder)
Congenital Disease	SNOMED	871667007	Disorder of ascending aorta due to conotruncal malformation (disorder)
Congenital Disease	SNOMED	871668002	Congenital right ventricular anomaly (disorder)
Congenital Disease	SNOMED	871669005	Acquired abnormality of aorta due to congenital heart anomaly (disorder)
Congenital Disease	SNOMED	871672003	Atypical right ventricular component of common atrioventricular valve (disorder)
Congenital Disease	SNOMED	87290003	Congenital anomaly of head (disorder)
Congenital Disease	SNOMED	87294007	Supernumerary vertebra (disorder)
Congenital Disease	SNOMED	87310001	Monorchism (disorder)
Congenital Disease	SNOMED	87380008	Congenital absence of vagina (disorder)
Congenital Disease	SNOMED	874782009	Acquired abnormality of atrioventricular (not morphologically mitral or tricuspid) following repair of atrioventricular septal defect (disorder)
Congenital Disease	SNOMED	874931001	Severe achondroplasia, developmental delay, acanthosis nigricans syndrome (disorder)
Congenital Disease	SNOMED	87541004	Ectopic gallbladder (disorder)
Congenital Disease	SNOMED	8757006	Hecht syndrome (disorder)
Congenital Disease	SNOMED	876846006	Congenital absence of chordae tendineae of tricuspid valve (disorder)
Congenital Disease	SNOMED	876847002	Congenital absence of carpal bone and metacarpal bone (disorder)
Congenital Disease	SNOMED	876853002	Agenesis of tarsal bone and metatarsal bone (disorder)
Congenital Disease	SNOMED	878878007	Congenital malformation caused by valproic acid (disorder)
Congenital Disease	SNOMED	8793008	Rokitansky sequence (disorder)
Congenital Disease	SNOMED	87979003	Cleft palate (disorder)
Congenital Disease	SNOMED	879919001	Bilateral megalencephaly (disorder)
Congenital Disease	SNOMED	879938005	46,XY disorder of sex development due to environmental chemical exposure (disorder)
Congenital Disease	SNOMED	879939002	14q32 deletion syndrome (disorder)
Congenital Disease	SNOMED	8800006	Female pseudohermaphroditism (disorder)
Congenital Disease	SNOMED	880078001	11p15 deletion syndrome (disorder)
Congenital Disease	SNOMED	880079009	11p15 duplication syndrome (disorder)
Congenital Disease	SNOMED	880081006	12q15 deletion syndrome (disorder)
Congenital Disease	SNOMED	880086001	12q24.31-q24.32 deletion syndrome (disorder)
Congenital Disease	SNOMED	880093002	17q11 deletion syndrome (disorder)
Congenital Disease	SNOMED	880094008	46,XY disorder of sex development (disorder)
Congenital Disease	SNOMED	880125002	46,XX androgen-induced disorder of sex development due to maternal adrenal hyperplasia (disorder)
Congenital Disease	SNOMED	880127005	46,XX androgen-induced disorder of sex development of iatrogenic maternal origin (disorder)
Congenital Disease	SNOMED	88035001	Congenital megalogastria (disorder)
Congenital Disease	SNOMED	88102009	Nodular renal blastema (disorder)
Congenital Disease	SNOMED	88103004	Congenital onychauxis (disorder)
Congenital Disease	SNOMED	88154004	Ring chromosome 18 syndrome (disorder)
Congenital Disease	SNOMED	88244008	Congenital stenosis of vena cava (disorder)
Congenital Disease	SNOMED	88386004	Congenital anomaly of diaphragm (disorder)
Congenital Disease	SNOMED	88425004	Congenital anomaly of nervous system (disorder)
Congenital Disease	SNOMED	88445007	Anomalous muscle bands of right ventricle (disorder)
Congenital Disease	SNOMED	88469006	Zellweger syndrome (disorder)
Congenital Disease	SNOMED	8851000	Nagele's pelvis (disorder)
Congenital Disease	SNOMED	88656003	Congenital absence of spermatic cord (disorder)
Congenital Disease	SNOMED	88659005	Cheilognathoprosoposchisis (disorder)
Congenital Disease	SNOMED	88721000119100	Congenital anomaly of craniovertebral junction (disorder)
Congenital Disease	SNOMED	88825008	Congenital anomaly of cervix (disorder)
Congenital Disease	SNOMED	88877002	Xeroderma pigmentosum, variant form (disorder)
Congenital Disease	SNOMED	88894000	4q partial trisomy syndrome (disorder)
Congenital Disease	SNOMED	890086003	46,XX disorder of sex development caused by synthetic oral progestogen (disorder)
Congenital Disease	SNOMED	890087007	46,XX disorder of sex development caused by synthetic oral diethylstilbestrol (disorder)
Congenital Disease	SNOMED	890088002	46,XX disorder of sex development caused by testosterone and/or testosterone derivative (disorder)
Congenital Disease	SNOMED	890089005	46,XX testicular disorder of sex development (disorder)
Congenital Disease	SNOMED	890099000	Atresia of ileum type I (disorder)
Congenital Disease	SNOMED	890101007	Atresia of ileum type II (disorder)
Congenital Disease	SNOMED	890102000	Atresia of ileum type IIIa (disorder)
Congenital Disease	SNOMED	890103005	Atresia of jejunum type I (disorder)
Congenital Disease	SNOMED	890104004	Atresia of jejunum type II (disorder)
Congenital Disease	SNOMED	890105003	Atresia of jejunum type IIIa (disorder)
Congenital Disease	SNOMED	890110004	17q23.1-q23.2 duplication syndrome (disorder)
Congenital Disease	SNOMED	890116005	17q24-qter duplication syndrome (disorder)
Congenital Disease	SNOMED	890117001	20p12.2 deletion syndrome (disorder)

Congenital Disease	SNOMED	890118006	Mowat-Wilson syndrome due to monosomy 2q22 (disorder)
Congenital Disease	SNOMED	890123006	3p25.3 deletion syndrome (disorder)
Congenital Disease	SNOMED	890124000	5q22.2 deletion syndrome (disorder)
Congenital Disease	SNOMED	890125004	7p12-p14 deletion syndrome (disorder)
Congenital Disease	SNOMED	890127007	7p21.1 deletion syndrome (disorder)
Congenital Disease	SNOMED	890128002	9p24.3 deletion syndrome (disorder)
Congenital Disease	SNOMED	890130000	9q34 deletion syndrome (disorder)
Congenital Disease	SNOMED	890180006	Blepharophimosis epicanthus inversus ptosis syndrome plus (disorder)
Congenital Disease	SNOMED	890190003	Brachymetatarsia of first metatarsal (disorder)
Congenital Disease	SNOMED	890191004	Brachymetatarsia of fourth metatarsal (disorder)
Congenital Disease	SNOMED	890201000	Congenital absence of cystic duct (disorder)
Congenital Disease	SNOMED	890202007	Embryopathy caused by acitretin (disorder)
Congenital Disease	SNOMED	890217006	Congenital anomalous arrangement of pancreatobiliary duct (disorder)
Congenital Disease	SNOMED	890219009	Congenital anomalous tracheobronchial branching (disorder)
Congenital Disease	SNOMED	890220003	Bicuspid aortic valve-associated aortopathy (disorder)
Congenital Disease	SNOMED	890221004	Acrocardiofacial syndrome (disorder)
Congenital Disease	SNOMED	890222006	Congenital complete absence of bilateral lower limbs (disorder)
Congenital Disease	SNOMED	890223001	Congenital complete absence of bilateral upper limbs (disorder)
Congenital Disease	SNOMED	890228005	Congenital atresia of bilateral anterior nares (disorder)
Congenital Disease	SNOMED	890229002	Autosomal dominant polycystic liver disease (disorder)
Congenital Disease	SNOMED	890233009	Autosomal dominant Robinow syndrome (disorder)
Congenital Disease	SNOMED	890235002	Autosomal recessive epidermolytic ichthyosis (disorder)
Congenital Disease	SNOMED	890237005	Autosomal recessive Robinow syndrome (disorder)
Congenital Disease	SNOMED	890285006	Bilateral frontal polymicrogyria (disorder)
Congenital Disease	SNOMED	890286007	Bilateral frontoparietal polymicrogyria (disorder)
Congenital Disease	SNOMED	890287003	Bilateral generalized polymicrogyria (disorder)
Congenital Disease	SNOMED	890288008	Bilateral parasagittal parieto-occipital polymicrogyria (disorder)
Congenital Disease	SNOMED	890346002	Holoprosencephaly co-occurrent with congenital nasal pyriform aperture stenosis (disorder)
Congenital Disease	SNOMED	890347006	Humeroradial synostosis of bilateral upper limbs (disorder)
Congenital Disease	SNOMED	890348001	Epiblepharon of bilateral eyelids (disorder)
Congenital Disease	SNOMED	890349009	Euryblepharon of bilateral eyelids (disorder)
Congenital Disease	SNOMED	890350009	Coralliform cataract (disorder)
Congenital Disease	SNOMED	890351008	Distal symphalangism of bilateral distal interphalangeal joints (disorder)
Congenital Disease	SNOMED	890352001	Congenital valgus deformity of bilateral great toes (disorder)
Congenital Disease	SNOMED	890353006	Congenital vertical talus deformity of bilateral ankles and feet (disorder)
Congenital Disease	SNOMED	890354000	Congenital subglottic stenosis Cotton-Myer grade 3 (disorder)
Congenital Disease	SNOMED	890355004	Congenital subglottic stenosis Cotton-Myer grade 4 (disorder)
Congenital Disease	SNOMED	890356003	Postaxial polydactyly type A of bilateral little fingers (disorder)
Congenital Disease	SNOMED	890357007	Postaxial polydactyly type B of bilateral little fingers (disorder)
Congenital Disease	SNOMED	890359005	Postaxial polydactyly of bilateral feet (disorder)
Congenital Disease	SNOMED	890360000	Polydactyly of bilateral thumbs (disorder)
Congenital Disease	SNOMED	890361001	Polydactyly of bilateral triphalangeal thumbs (disorder)
Congenital Disease	SNOMED	890362008	Partial cryptophthalmos of bilateral eyelids (disorder)
Congenital Disease	SNOMED	890363003	Persistent hyperplastic primary vitreous of bilateral eyes (disorder)
Congenital Disease	SNOMED	890364009	Macrodactyly of bilateral feet (disorder)
Congenital Disease	SNOMED	890365005	Incomplete congenital pyloric antral membrane (disorder)
Congenital Disease	SNOMED	890366006	Macrodactyly of bilateral fingers (disorder)
Congenital Disease	SNOMED	890367002	Congenital metatarsus valgus of bilateral feet (disorder)
Congenital Disease	SNOMED	890369004	Congenital malrotation of large intestine (disorder)
Congenital Disease	SNOMED	890370003	Congenital megacalycosis of bilateral kidneys (disorder)
Congenital Disease	SNOMED	890372006	Congenital malformation of lymphatic system of cervicofacial region (disorder)
Congenital Disease	SNOMED	890373001	Central polydactyly of bilateral fingers (disorder)
Congenital Disease	SNOMED	890376009	Congenital arteriovenous fistula of umbilical blood vessels (disorder)
Congenital Disease	SNOMED	890377000	Agenesis of umbilical cord (disorder)
Congenital Disease	SNOMED	890378005	Congenital epibulbar choristoma of bilateral eyes (disorder)
Congenital Disease	SNOMED	890379002	Congenital duplication of gallbladder type 2 (disorder)
Congenital Disease	SNOMED	890380004	Bilateral Madelung deformity (disorder)
Congenital Disease	SNOMED	890381000	Congenital ectropion of bilateral eyelids (disorder)
Congenital Disease	SNOMED	890382007	Choanal atresia of bilateral nasal passages (disorder)
Congenital Disease	SNOMED	890383002	Congenital diverticulum of large intestine (disorder)
Congenital Disease	SNOMED	890384008	Central polydactyly of bilateral toes (disorder)
Congenital Disease	SNOMED	890385009	Congenital duplication of gallbladder type 1 (disorder)
Congenital Disease	SNOMED	890386005	Congenital subglottic stenosis Cotton-Myer grade 1 (disorder)
Congenital Disease	SNOMED	890387001	Congenital subglottic stenosis Cotton-Myer grade 2 (disorder)
Congenital Disease	SNOMED	890388006	Congenital pulmonary airway malformation type 4 (disorder)
Congenital Disease	SNOMED	890389003	Congenital stenosis of the tracheobronchial tree (disorder)
Congenital Disease	SNOMED	890390007	Congenital pulmonary airway malformation type 2 (disorder)
Congenital Disease	SNOMED	890391006	Congenital pulmonary airway malformation type 3 (disorder)
Congenital Disease	SNOMED	890392004	Congenital pulmonary airway malformation type 1 (disorder)
Congenital Disease	SNOMED	890393009	Congenital prolapse of mitral valve (disorder)
Congenital Disease	SNOMED	890394003	Congenital pulmonary airway malformation type 0 (disorder)
Congenital Disease	SNOMED	890396001	Congenital pit of bilateral optic discs (disorder)
Congenital Disease	SNOMED	890397005	Congenital bowing of ulna (disorder)

Congenital Disease	SNOMED	890398000	Congenital bronchocele (disorder)
Congenital Disease	SNOMED	890399008	Congenital anomaly of patella (disorder)
Congenital Disease	SNOMED	890400001	Congenital absence of bilateral breasts (disorder)
Congenital Disease	SNOMED	890401002	Congenital absence of bilateral lower legs and feet (disorder)
Congenital Disease	SNOMED	890402009	Congenital abnormality of truncal valve (disorder)
Congenital Disease	SNOMED	890403004	Congenital absence of bilateral forearms and hands (disorder)
Congenital Disease	SNOMED	890404005	Complete phocomelia of bilateral lower limbs (disorder)
Congenital Disease	SNOMED	890405006	Complete phocomelia of bilateral upper limbs (disorder)
Congenital Disease	SNOMED	890406007	Complete cryptophthalmos (disorder)
Congenital Disease	SNOMED	890408008	Congenital diverticulum of gallbladder (disorder)
Congenital Disease	SNOMED	890409000	Congenital dislocation of bilateral shoulders (disorder)
Congenital Disease	SNOMED	890410005	Congenital dislocation of joint (disorder)
Congenital Disease	SNOMED	890411009	Congenital dislocation of bilateral patellas (disorder)
Congenital Disease	SNOMED	890412002	Congenital dacryocele of bilateral lacrimal sacs (disorder)
Congenital Disease	SNOMED	890413007	Congenital dislocation of bilateral elbows (disorder)
Congenital Disease	SNOMED	890414001	Congenital cryptophthalmos of bilateral eyelids (disorder)
Congenital Disease	SNOMED	890415000	Congenital cyst of vitreous body (disorder)
Congenital Disease	SNOMED	890416004	Congenital clinodactyly of bilateral fingers (disorder)
Congenital Disease	SNOMED	890417008	Congenital coloboma of bilateral eyelids (disorder)
Congenital Disease	SNOMED	890419006	Syndactyly with fusion of bones of bilateral fingers (disorder)
Congenital Disease	SNOMED	890422008	Cervicothoracic spina bifida aperta with hydrocephalus (disorder)
Congenital Disease	SNOMED	890424009	Secondary congenital hyperplasia of bilateral lungs (disorder)
Congenital Disease	SNOMED	890425005	Proximal symphalangism of bilateral fingers (disorder)
Congenital Disease	SNOMED	890427002	Primary congenital hyperplasia of bilateral lungs (disorder)
Congenital Disease	SNOMED	890428007	Primary congenital hypoplasia of bilateral lungs (disorder)
Congenital Disease	SNOMED	890429004	Preaxial polydactyly of bilateral great toes (disorder)
Congenital Disease	SNOMED	890430009	Complete agenesis of vermis (disorder)
Congenital Disease	SNOMED	890432001	Cockayne syndrome type 3 (disorder)
Congenital Disease	SNOMED	890433006	Cockayne syndrome type 1 (disorder)
Congenital Disease	SNOMED	890434000	Cockayne syndrome type 2 (disorder)
Congenital Disease	SNOMED	890437007	Chondrodysplasia punctata due to maternal autoimmune disease (disorder)
Congenital Disease	SNOMED	890438002	Brachydactyly type A3 (disorder)
Congenital Disease	SNOMED	890439005	Brachydactyly type D (disorder)
Congenital Disease	SNOMED	890460004	46,XX disorder of sex development (disorder)
Congenital Disease	SNOMED	890461000	Congenital symblepharon of bilateral conjunctivae (disorder)
Congenital Disease	SNOMED	8915006	Congenital hypoplasia of breast (disorder)
Congenital Disease	SNOMED	89166001	Congenital anomaly of liver (disorder)
Congenital Disease	SNOMED	89199004	Congenital web of esophagus (disorder)
Congenital Disease	SNOMED	89222008	Pericarditis co-occurrent with atrial septal defect (disorder)
Congenital Disease	SNOMED	89243009	Polymelia (disorder)
Congenital Disease	SNOMED	89318001	Congenital prolapsed uterus (disorder)
Congenital Disease	SNOMED	89321004	Köbner phenomenon (disorder)
Congenital Disease	SNOMED	89369001	Anencephalus (disorder)
Congenital Disease	SNOMED	89378007	Congenital displacement of stomach (disorder)
Congenital Disease	SNOMED	89392001	Prader-Willi syndrome (disorder)
Congenital Disease	SNOMED	89409002	Gastrothoracopagus dipygus (disorder)
Congenital Disease	SNOMED	894093007	Complete congenital web of pyloric antrum (disorder)
Congenital Disease	SNOMED	894125007	Cervicothoracic spina bifida aperta (disorder)
Congenital Disease	SNOMED	89434002	Complete aphalangia of lower limb (disorder)
Congenital Disease	SNOMED	89444000	Oromandibular-limb hypogenesis spectrum (disorder)
Congenital Disease	SNOMED	89497006	Persistent human tail (disorder)
Congenital Disease	SNOMED	8962004	Katadidymus (disorder)
Congenital Disease	SNOMED	89647000	Pyknodysostosis (disorder)
Congenital Disease	SNOMED	896774004	Cribriform hymen (disorder)
Congenital Disease	SNOMED	89689008	Congenital genu valgum (disorder)
Congenital Disease	SNOMED	896999006	Congenital agenesis of vagina (disorder)
Congenital Disease	SNOMED	897279002	Fetal epignathus with conjoined twins (disorder)
Congenital Disease	SNOMED	897507003	Distal trisomy 9p (disorder)
Congenital Disease	SNOMED	897509000	Distal trisomy 8p (disorder)
Congenital Disease	SNOMED	897510005	Distal trisomy 3q (disorder)
Congenital Disease	SNOMED	897511009	Distal trisomy 7q (disorder)
Congenital Disease	SNOMED	897523003	Triphalangeal deformity of bilateral thumbs (disorder)
Congenital Disease	SNOMED	897524009	Distal deletion of short arm of chromosome 1 (disorder)
Congenital Disease	SNOMED	897526006	Distal deletion of long arm of chromosome 8 (disorder)
Congenital Disease	SNOMED	897527002	Distal deletion of long arm of chromosome 9 (disorder)
Congenital Disease	SNOMED	897528007	Distal deletion of long arm of chromosome 6 (disorder)
Congenital Disease	SNOMED	897529004	Distal deletion of long arm of chromosome 7 (disorder)
Congenital Disease	SNOMED	897531008	Congenital absence of bilateral ulnas (disorder)
Congenital Disease	SNOMED	897532001	Congenital absence of bilateral tibias (disorder)
Congenital Disease	SNOMED	897533006	Congenital absence of bilateral radiuses (disorder)
Congenital Disease	SNOMED	897536003	Distal deletion of long arm of chromosome 3 (disorder)
Congenital Disease	SNOMED	897537007	Congenital anomaly of internal auditory canal (disorder)
Congenital Disease	SNOMED	897539005	Congenital anomaly of cochlea (disorder)
Congenital Disease	SNOMED	897541006	Congenital anomaly of biliary tract (disorder)
Congenital Disease	SNOMED	897542004	Distal deletion of long arm of chromosome 2 (disorder)

Congenital Disease	SNOMED	897543009	Distal trisomy 1p (disorder)
Congenital Disease	SNOMED	897544003	Distal trisomy 1q (disorder)
Congenital Disease	SNOMED	897545002	Distal duplication of chromosome 21 (disorder)
Congenital Disease	SNOMED	897546001	Distal trisomy 12q (disorder)
Congenital Disease	SNOMED	897547005	Distal duplication of chromosome 14 (disorder)
Congenital Disease	SNOMED	897548000	Distal duplication of chromosome 15 (disorder)
Congenital Disease	SNOMED	897549008	Distal duplication of chromosome 13 (disorder)
Congenital Disease	SNOMED	897554004	Dermal melanocytic hamartoma (disorder)
Congenital Disease	SNOMED	897557006	Congenital shortening of ear (disorder)
Congenital Disease	SNOMED	897559009	Congenital retinal dysplasia caused by teratogenic substance (disorder)
Congenital Disease	SNOMED	897560004	Congenital sagittal cleft of vertebra (disorder)
Congenital Disease	SNOMED	897561000	Distal deletion of long arm of chromosome 16 (disorder)
Congenital Disease	SNOMED	897564008	Distal deletion of long arm of chromosome 12 (disorder)
Congenital Disease	SNOMED	897566005	Distal deletion of chromosome 21 (disorder)
Congenital Disease	SNOMED	897568006	Distal deletion of chromosome 14 (disorder)
Congenital Disease	SNOMED	897569003	Distal deletion of chromosome 15 (disorder)
Congenital Disease	SNOMED	897570002	Distal arthrogryposis type 3 (disorder)
Congenital Disease	SNOMED	897572005	Congenital anomaly of semicircular canal (disorder)
Congenital Disease	SNOMED	897573000	Talipes calcaneovarus of bilateral ankles and feet (disorder)
Congenital Disease	SNOMED	897574006	Congenital anomaly of vestibule of inner ear (disorder)
Congenital Disease	SNOMED	897575007	Talipes equinovarus of bilateral ankles and feet (disorder)
Congenital Disease	SNOMED	897576008	Spherophakia of bilateral lenses (disorder)
Congenital Disease	SNOMED	897577004	Symbrachydactyly of bilateral hands and feet (disorder)
Congenital Disease	SNOMED	897579001	Simple syndactyly of toes of second web space of bilateral feet (disorder)
Congenital Disease	SNOMED	897585008	Congenital primary glaucoma of bilateral eyes (disorder)
Congenital Disease	SNOMED	897586009	Congenital radial deviation of bilateral fingers (disorder)
Congenital Disease	SNOMED	897587000	Congenital primary aphakia of bilateral lenses (disorder)
Congenital Disease	SNOMED	897588005	Congenital hypertrophy of bilateral upper limbs (disorder)
Congenital Disease	SNOMED	897589002	Congenital partial absence of gastric muscle (disorder)
Congenital Disease	SNOMED	897590006	Congenital dysplasia of bone caused by drug (disorder)
Congenital Disease	SNOMED	897591005	Congenital dysplasia of vestibule of ear (disorder)
Congenital Disease	SNOMED	897594002	Congenital anorectal fistula due to high anorectal malformation (disorder)
Congenital Disease	SNOMED	897595001	Congenital anorectal fistula due to low anorectal malformation (disorder)
Congenital Disease	SNOMED	8986002	Transposition of colon (disorder)
Congenital Disease	SNOMED	89886004	Congenital anomaly of skeletal muscle (disorder)
Congenital Disease	SNOMED	89954008	Ectopic pancreatic tissue in stomach (disorder)
Congenital Disease	SNOMED	90145001	Frontal bossing (disorder)
Congenital Disease	SNOMED	90313000	Autositic twin of asymmetrical conjoined twins (disorder)
Congenital Disease	SNOMED	90383006	Congenital absence of aorta (disorder)
Congenital Disease	SNOMED	90496008	Generalized epidermolysis bullosa simplex (disorder)
Congenital Disease	SNOMED	90575004	Persistent lanugo (disorder)
Congenital Disease	SNOMED	90669003	Congenital diverticulum of stomach (disorder)
Congenital Disease	SNOMED	90866007	3q partial trisomy syndrome (disorder)
Congenital Disease	SNOMED	90996003	Cordate pelvis (disorder)
Congenital Disease	SNOMED	91158006	Congenital anomaly of eyelid (disorder)
Congenital Disease	SNOMED	91210006	Congenital anourethral fistula (disorder)
Congenital Disease	SNOMED	91285009	Ectopic adrenal cortex (disorder)
Congenital Disease	SNOMED	91375006	Congenital atresia of rectum (disorder)
Congenital Disease	SNOMED	91443007	Accessory bronchus (disorder)
Congenital Disease	SNOMED	9147009	Osteopoikilosis (disorder)
Congenital Disease	SNOMED	91605000	Congenital absence of artery (disorder)
Congenital Disease	SNOMED	91841003	Abnormal communication between pericardial sac and peritoneal cavity (disorder)
Congenital Disease	SNOMED	91842005	Abnormal lung lobation (disorder)
Congenital Disease	SNOMED	91844006	Supernumerary gallbladder (disorder)
Congenital Disease	SNOMED	91846008	Supernumerary metacarpal bone (disorder)
Congenital Disease	SNOMED	91847004	Accessory sternebral ossification site (disorder)
Congenital Disease	SNOMED	91848009	Acephaly (disorder)
Congenital Disease	SNOMED	91849001	Acephalostomia (disorder)
Congenital Disease	SNOMED	91868003	Congenital absence of stomach (disorder)
Congenital Disease	SNOMED	91869006	Congenital absence of alisphenoid bone (disorder)
Congenital Disease	SNOMED	91870007	Congenital absence of basioccipital bone (disorder)
Congenital Disease	SNOMED	91871006	Congenital absence of basisphenoid bone (disorder)
Congenital Disease	SNOMED	91872004	Congenital absence of calcaneus (disorder)
Congenital Disease	SNOMED	91873009	Congenital absence of carpal bone (disorder)
Congenital Disease	SNOMED	91877005	Congenital absence of centrum of cervical vertebra (disorder)
Congenital Disease	SNOMED	91878000	Congenital absence of centrum of sacral vertebra (disorder)
Congenital Disease	SNOMED	91879008	Congenital absence of arch of cervical vertebra (disorder)
Congenital Disease	SNOMED	91880006	Congenital absence of cervical vertebra (disorder)
Congenital Disease	SNOMED	91882003	Congenital absence of epididymis (disorder)
Congenital Disease	SNOMED	91883008	Congenital absence of exoccipital bone (disorder)
Congenital Disease	SNOMED	91885001	Congenital absence of frontal bone (disorder)
Congenital Disease	SNOMED	91887009	Congenital absence of hyoid bone (disorder)
Congenital Disease	SNOMED	91888004	Congenital absence of ilium (disorder)
Congenital Disease	SNOMED	91889007	Congenital absence of interparietal bone (disorder)

Congenital Disease	SNOMED	91890003	Congenital absence of intestinal tract (disorder)
Congenital Disease	SNOMED	91891004	Congenital absence of ischium (disorder)
Congenital Disease	SNOMED	91892006	Congenital absence of lacrimal bone (disorder)
Congenital Disease	SNOMED	91893001	Congenital absence of arch of lumbar vertebra (disorder)
Congenital Disease	SNOMED	91894007	Congenital absence of centrum of lumbar vertebra (disorder)
Congenital Disease	SNOMED	91895008	Congenital absence of lumbar vertebra (disorder)
Congenital Disease	SNOMED	91896009	Congenital absence of mandible (disorder)
Congenital Disease	SNOMED	91897000	Congenital absence of maxilla (disorder)
Congenital Disease	SNOMED	91898005	Congenital absence of metacarpal bone (disorder)
Congenital Disease	SNOMED	91899002	Congenital absence of metatarsal bone (disorder)
Congenital Disease	SNOMED	91900007	Congenital absence of nasal bone (disorder)
Congenital Disease	SNOMED	91901006	Congenital absence of palatine bone (disorder)
Congenital Disease	SNOMED	91902004	Congenital absence of parietal bone (disorder)
Congenital Disease	SNOMED	91903009	Congenital absence of premaxilla (disorder)
Congenital Disease	SNOMED	91904003	Congenital absence of presphenoid bone (disorder)
Congenital Disease	SNOMED	91905002	Congenital absence of pubis (disorder)
Congenital Disease	SNOMED	91906001	Congenital absence of arch of sacral vertebra (disorder)
Congenital Disease	SNOMED	91907005	Congenital absence of sacral vertebra (disorder)
Congenital Disease	SNOMED	91908000	Congenital absence of scapula (disorder)
Congenital Disease	SNOMED	91909008	Congenital absence of squamosal bone (disorder)
Congenital Disease	SNOMED	91910003	Congenital absence of sternebra (disorder)
Congenital Disease	SNOMED	91911004	Congenital absence of supraoccipital bone (disorder)
Congenital Disease	SNOMED	91913001	Congenital absence of talus (disorder)
Congenital Disease	SNOMED	91914007	Congenital absence of tarsal bone (disorder)
Congenital Disease	SNOMED	91915008	Congenital absence of arch of thoracic vertebra (disorder)
Congenital Disease	SNOMED	91916009	Congenital absence of centrum of thoracic vertebra (disorder)
Congenital Disease	SNOMED	91917000	Congenital absence of thoracic vertebra (disorder)
Congenital Disease	SNOMED	91918005	Congenital absence of thymus (disorder)
Congenital Disease	SNOMED	91919002	Congenital absence of tympanic anulus (disorder)
Congenital Disease	SNOMED	91920008	Congenital absence of vomer (disorder)
Congenital Disease	SNOMED	91921007	Congenital absence of zygomatic bone (disorder)
Congenital Disease	SNOMED	91922000	Congenital absence of jaw (disorder)
Congenital Disease	SNOMED	91946007	Congenital absence of mouth (disorder)
Congenital Disease	SNOMED	92489003	Cleft cartilaginous centrum of cervical vertebra (disorder)
Congenital Disease	SNOMED	92490007	Cleft cartilaginous centrum of lumbar vertebra (disorder)
Congenital Disease	SNOMED	92491006	Cleft cartilaginous centrum of sacral vertebra (disorder)
Congenital Disease	SNOMED	92492004	Cleft cartilaginous centrum of thoracic vertebra (disorder)
Congenital Disease	SNOMED	92494003	Bipartite ossification of centrum of cervical vertebra (disorder)
Congenital Disease	SNOMED	92495002	Bipartite ossification of interparietal bone (disorder)
Congenital Disease	SNOMED	92496001	Bipartite ossification of centrum of lumbar vertebra (disorder)
Congenital Disease	SNOMED	92497005	Bipartite ossification of centrum of sacral vertebra (disorder)
Congenital Disease	SNOMED	92498000	Bipartite ossification of sternebra (disorder)
Congenital Disease	SNOMED	92499008	Bipartite ossification of supraoccipital bone (disorder)
Congenital Disease	SNOMED	92500004	Bipartite ossification of centrum of thoracic vertebra (disorder)
Congenital Disease	SNOMED	92501000	Congenital cleft of thymus (disorder)
Congenital Disease	SNOMED	92503002	Neurofibromatosis type 2 (disorder)
Congenital Disease	SNOMED	9252005	Congenital bowing of tibia and/or fibula (disorder)
Congenital Disease	SNOMED	92806000	Cervical hemivertebra (disorder)
Congenital Disease	SNOMED	92824003	Neurofibromatosis type 1 (disorder)
Congenital Disease	SNOMED	92830003	Combined valvular-subvalvular pulmonic stenosis (disorder)
Congenital Disease	SNOMED	92833001	Congenital abnormal fusion of adrenal glands (disorder)
Congenital Disease	SNOMED	92834007	Congenital abnormal fusion of alisphenoid bone (disorder)
Congenital Disease	SNOMED	92836009	Congenital abnormal fusion of arch of cervical vertebra (disorder)
Congenital Disease	SNOMED	92837000	Congenital abnormal fusion of arch of lumbar vertebra (disorder)
Congenital Disease	SNOMED	92838005	Congenital abnormal fusion of arch of sacral vertebra (disorder)
Congenital Disease	SNOMED	92839002	Congenital abnormal fusion of arch of thoracic vertebra (disorder)
Congenital Disease	SNOMED	92840000	Congenital abnormal fusion of basioccipital bone (disorder)
Congenital Disease	SNOMED	92841001	Congenital abnormal fusion of basisphenoid bone (disorder)
Congenital Disease	SNOMED	92842008	Congenital abnormal fusion of calcaneus (disorder)
Congenital Disease	SNOMED	92843003	Congenital abnormal fusion of carpal bone (disorder)
Congenital Disease	SNOMED	92844009	Congenital abnormal fusion of centrum cartilage of cervical vertebra (disorder)
Congenital Disease	SNOMED	92845005	Congenital abnormal fusion of centrum cartilage of lumbar vertebra (disorder)
Congenital Disease	SNOMED	92846006	Congenital abnormal fusion of centrum cartilage of sacral vertebra (disorder)
Congenital Disease	SNOMED	92847002	Congenital abnormal fusion of centrum cartilage of thoracic vertebra (disorder)
Congenital Disease	SNOMED	92849004	Congenital abnormal fusion of centrum of cervical vertebra (disorder)
Congenital Disease	SNOMED	92850004	Congenital abnormal fusion of centrum of lumbar vertebra (disorder)
Congenital Disease	SNOMED	92851000	Congenital abnormal fusion of centrum of sacral vertebra (disorder)
Congenital Disease	SNOMED	92852007	Congenital abnormal fusion of centrum of thoracic vertebra (disorder)
Congenital Disease	SNOMED	92853002	Congenital abnormal fusion of exoccipital bone (disorder)
Congenital Disease	SNOMED	92854008	Congenital abnormal fusion of femur (disorder)
Congenital Disease	SNOMED	92855009	Congenital abnormal fusion of fibula (disorder)
Congenital Disease	SNOMED	92857001	Congenital abnormal fusion of frontal bone (disorder)

Congenital Disease	SNOMED	92859003	Congenital abnormal fusion of humerus (disorder)
Congenital Disease	SNOMED	92860008	Congenital abnormal fusion of ilium (disorder)
Congenital Disease	SNOMED	92861007	Congenital abnormal fusion of interparietal bone (disorder)
Congenital Disease	SNOMED	92862000	Congenital abnormal fusion of ischium (disorder)
Congenital Disease	SNOMED	92863005	Congenital abnormal fusion of lacrimal bone (disorder)
Congenital Disease	SNOMED	92864004	Congenital abnormal fusion of liver lobes (disorder)
Congenital Disease	SNOMED	92865003	Congenital abnormal fusion of mandible (disorder)
Congenital Disease	SNOMED	92866002	Congenital abnormal fusion of maxilla (disorder)
Congenital Disease	SNOMED	92867006	Congenital abnormal fusion of metacarpal bone (disorder)
Congenital Disease	SNOMED	92868001	Congenital abnormal fusion of metatarsal bone (disorder)
Congenital Disease	SNOMED	92869009	Congenital abnormal fusion of nasal bone (disorder)
Congenital Disease	SNOMED	92870005	Congenital abnormal fusion of palatine bone (disorder)
Congenital Disease	SNOMED	92871009	Congenital abnormal fusion of parietal bone (disorder)
Congenital Disease	SNOMED	92872002	Congenital abnormal fusion of premaxilla (disorder)
Congenital Disease	SNOMED	92873007	Congenital abnormal fusion of presphenoid bone (disorder)
Congenital Disease	SNOMED	92874001	Congenital abnormal fusion of radius (disorder)
Congenital Disease	SNOMED	92875000	Congenital abnormal fusion of rib cartilage (disorder)
Congenital Disease	SNOMED	92876004	Congenital abnormal fusion of squamosal bone (disorder)
Congenital Disease	SNOMED	92877008	Congenital abnormal fusion of supraoccipital bone (disorder)
Congenital Disease	SNOMED	92878003	Congenital abnormal fusion of talus (disorder)
Congenital Disease	SNOMED	92879006	Congenital abnormal fusion of tarsal bone (disorder)
Congenital Disease	SNOMED	92880009	Congenital abnormal fusion of tibia (disorder)
Congenital Disease	SNOMED	92881008	Congenital abnormal fusion of tympanic anulus (disorder)
Congenital Disease	SNOMED	92882001	Congenital abnormal fusion of ulna (disorder)
Congenital Disease	SNOMED	92883006	Congenital abnormal fusion of vomer (disorder)
Congenital Disease	SNOMED	92884000	Congenital abnormal fusion of zygomatic bone (disorder)
Congenital Disease	SNOMED	92885004	Congenital abnormal long growth of bile duct (disorder)
Congenital Disease	SNOMED	92886003	Congenital abnormal shape of adrenal gland (disorder)
Congenital Disease	SNOMED	92887007	Congenital abnormal shape of alisphenoid bone (disorder)
Congenital Disease	SNOMED	92888002	Congenital abnormal shape of aortic valve (disorder)
Congenital Disease	SNOMED	92890001	Congenital abnormal shape of arch of cervical vertebra (disorder)
Congenital Disease	SNOMED	92891002	Congenital abnormal shape of arch of lumbar vertebra (disorder)
Congenital Disease	SNOMED	92892009	Congenital abnormal shape of arch of sacral vertebra (disorder)
Congenital Disease	SNOMED	92893004	Congenital abnormal shape of arch of thoracic vertebra (disorder)
Congenital Disease	SNOMED	92894005	Congenital abnormal shape of auditory ossicles (disorder)
Congenital Disease	SNOMED	92895006	Congenital abnormal shape of basioccipital bone (disorder)
Congenital Disease	SNOMED	92896007	Congenital abnormal shape of basisphenoid bone (disorder)
Congenital Disease	SNOMED	92897003	Congenital abnormal shape of calcaneus (disorder)
Congenital Disease	SNOMED	92898008	Congenital abnormal shape of carpal bone (disorder)
Congenital Disease	SNOMED	92900005	Congenital abnormal shape of centrum of cervical vertebra (disorder)
Congenital Disease	SNOMED	92901009	Congenital abnormal shape of centrum of lumbar vertebra (disorder)
Congenital Disease	SNOMED	92902002	Congenital abnormal shape of centrum of sacral vertebra (disorder)
Congenital Disease	SNOMED	92903007	Congenital abnormal shape of centrum of thoracic vertebra (disorder)
Congenital Disease	SNOMED	92904001	Congenital abnormal shape of cerebellum (disorder)
Congenital Disease	SNOMED	92905000	Congenital abnormal shape of cerebrum (disorder)
Congenital Disease	SNOMED	92906004	Congenital abnormal shape of clavicle (disorder)
Congenital Disease	SNOMED	92908003	Congenital abnormal shape of exoccipital bone (disorder)
Congenital Disease	SNOMED	92909006	Congenital abnormal shape of femur (disorder)
Congenital Disease	SNOMED	92910001	Congenital abnormal shape of fibula (disorder)
Congenital Disease	SNOMED	92912009	Congenital abnormal shape of frontal bone (disorder)
Congenital Disease	SNOMED	92913004	Congenital abnormal shape of gallbladder (disorder)
Congenital Disease	SNOMED	92915006	Congenital abnormal shape of humerus (disorder)
Congenital Disease	SNOMED	92916007	Congenital abnormal shape of hyoid bone (disorder)
Congenital Disease	SNOMED	92917003	Congenital abnormal shape of ilium (disorder)
Congenital Disease	SNOMED	92918008	Congenital abnormal shape of inner ear (disorder)
Congenital Disease	SNOMED	92919000	Congenital abnormal shape of interparietal bone (disorder)
Congenital Disease	SNOMED	92920006	Congenital abnormal shape of ischium (disorder)
Congenital Disease	SNOMED	92921005	Congenital abnormal shape of kidney (disorder)
Congenital Disease	SNOMED	92922003	Congenital abnormal shape of lacrimal bone (disorder)
Congenital Disease	SNOMED	92923008	Congenital abnormal shape of mitral valve (disorder)
Congenital Disease	SNOMED	92924002	Congenital abnormal shape of liver (disorder)
Congenital Disease	SNOMED	92925001	Congenital abnormal shape of lung (disorder)
Congenital Disease	SNOMED	92926000	Congenital abnormal shape of mandible (disorder)
Congenital Disease	SNOMED	92927009	Congenital abnormal shape of maxilla (disorder)
Congenital Disease	SNOMED	92928004	Congenital abnormal shape of metacarpal bone (disorder)
Congenital Disease	SNOMED	92929007	Congenital abnormal shape of metatarsal bone (disorder)
Congenital Disease	SNOMED	92930002	Congenital abnormal shape of nasal bone (disorder)
Congenital Disease	SNOMED	92932005	Congenital abnormal shape of ovary (disorder)
Congenital Disease	SNOMED	92933000	Congenital abnormal shape of fallopian tube (disorder)
Congenital Disease	SNOMED	92934006	Congenital abnormal shape of palate rugae (disorder)
Congenital Disease	SNOMED	92935007	Congenital abnormal shape of palatine bone (disorder)
Congenital Disease	SNOMED	92936008	Congenital abnormal shape of parietal bone (disorder)
Congenital Disease	SNOMED	92937004	Congenital abnormal shape of pinna (disorder)
Congenital Disease	SNOMED	92938009	Congenital abnormal shape of premaxilla (disorder)
Congenital Disease	SNOMED	92939001	Congenital abnormal shape of presphenoid bone (disorder)

Congenital Disease	SNOMED	92940004	Congenital abnormal shape of pubis (disorder)
Congenital Disease	SNOMED	92941000	Congenital abnormal shape of pulmonary valve (disorder)
Congenital Disease	SNOMED	92942007	Congenital abnormal shape of radius (disorder)
Congenital Disease	SNOMED	92943002	Congenital abnormal shape of rib (disorder)
Congenital Disease	SNOMED	92944008	Congenital abnormal shape of tricuspid valve (disorder)
Congenital Disease	SNOMED	92945009	Congenital abnormal shape of scapula (disorder)
Congenital Disease	SNOMED	92946005	Congenital abnormal shape of spleen (disorder)
Congenital Disease	SNOMED	92947001	Congenital abnormal shape of squamosal bone (disorder)
Congenital Disease	SNOMED	92948006	Congenital abnormal shape of sternebra (disorder)
Congenital Disease	SNOMED	92949003	Congenital abnormal shape of supraoccipital bone (disorder)
Congenital Disease	SNOMED	92950003	Congenital abnormal shape of talus (disorder)
Congenital Disease	SNOMED	92951004	Congenital abnormal shape of tarsal bone (disorder)
Congenital Disease	SNOMED	92952006	Congenital abnormal shape of testis (disorder)
Congenital Disease	SNOMED	92953001	Congenital abnormal shape of thymus (disorder)
Congenital Disease	SNOMED	92954007	Congenital abnormal shape of tibia (disorder)
Congenital Disease	SNOMED	92955008	Congenital abnormal shape of tympanic anulus (disorder)
Congenital Disease	SNOMED	92956009	Congenital abnormal shape of ulna (disorder)
Congenital Disease	SNOMED	92957000	Congenital abnormal shape of uterus (disorder)
Congenital Disease	SNOMED	92958005	Congenital abnormal shape of vomer (disorder)
Congenital Disease	SNOMED	92959002	Congenital abnormal shape of zygomatic bone (disorder)
Congenital Disease	SNOMED	92960007	Congenital absence of aortic valve (disorder)
Congenital Disease	SNOMED	92961006	Congenital absence of azygos vein (disorder)
Congenital Disease	SNOMED	92962004	Congenital absence of carotid artery (disorder)
Congenital Disease	SNOMED	92963009	Congenital absence of chordae tendineae (disorder)
Congenital Disease	SNOMED	92964003	Agenesis of clavicle (disorder)
Congenital Disease	SNOMED	92965002	Congenital absence of ductus arteriosus (disorder)
Congenital Disease	SNOMED	92966001	Congenital absence of eye bulge (disorder)
Congenital Disease	SNOMED	92969008	Congenital absence of mitral valve (disorder)
Congenital Disease	SNOMED	92970009	Congenital absence of nasal turbinate (disorder)
Congenital Disease	SNOMED	92971008	Congenital absence of nasal septum (disorder)
Congenital Disease	SNOMED	92972001	Monophthalmos (disorder)
Congenital Disease	SNOMED	92973006	Congenital absence of oviduct (disorder)
Congenital Disease	SNOMED	92974000	Congenital absence of papillary muscle (disorder)
Congenital Disease	SNOMED	92975004	Congenital absence of renal papilla (disorder)
Congenital Disease	SNOMED	92976003	Congenital absence of tricuspid valve (disorder)
Congenital Disease	SNOMED	92977007	Congenital absence of subclavian artery (disorder)
Congenital Disease	SNOMED	92980008	Congenital fenestration of alisphenoid bone (disorder)
Congenital Disease	SNOMED	92981007	Congenital bent clavicle (disorder)
Congenital Disease	SNOMED	92982000	Congenital bent humerus (disorder)
Congenital Disease	SNOMED	92983005	Congenital bent hyoid bone (disorder)
Congenital Disease	SNOMED	92984004	Congenital bent ilium (disorder)
Congenital Disease	SNOMED	92985003	Congenital bent ischium (disorder)
Congenital Disease	SNOMED	92986002	Congenital bent pubis (disorder)
Congenital Disease	SNOMED	92987006	Congenital bent radius (disorder)
Congenital Disease	SNOMED	92988001	Congenital bent rib (disorder)
Congenital Disease	SNOMED	92989009	Congenital bent scapula (disorder)
Congenital Disease	SNOMED	92991001	Congenital bent ulna (disorder)
Congenital Disease	SNOMED	92992008	Congenital anomaly of alisphenoid bone (disorder)
Congenital Disease	SNOMED	92994009	Congenital anomaly of azygos vein (disorder)
Congenital Disease	SNOMED	92995005	Congenital anomaly of basioccipital bone (disorder)
Congenital Disease	SNOMED	92996006	Congenital anomaly of basisphenoid bone (disorder)
Congenital Disease	SNOMED	92997002	Congenital anomaly of carotid artery (disorder)
Congenital Disease	SNOMED	92998007	Congenital anomaly of caudal vertebra (disorder)
Congenital Disease	SNOMED	92999004	Congenital anomaly of cervical vertebra (disorder)
Congenital Disease	SNOMED	93001004	Congenital anomaly of epididymis (disorder)
Congenital Disease	SNOMED	93002006	Congenital anomaly of exoccipital bone (disorder)
Congenital Disease	SNOMED	93003001	Congenital anomaly of fetal head bones (disorder)
Congenital Disease	SNOMED	93004007	Congenital anomaly of frontal bone (disorder)
Congenital Disease	SNOMED	93005008	Congenital anomaly of hyoid bone (disorder)
Congenital Disease	SNOMED	93006009	Congenital anomaly of ilium (disorder)
Congenital Disease	SNOMED	93007000	Congenital anomaly of interparietal bone (disorder)
Congenital Disease	SNOMED	93008005	Congenital anomaly of ischium (disorder)
Congenital Disease	SNOMED	93009002	Congenital anomaly of lacrimal bone (disorder)
Congenital Disease	SNOMED	93010007	Congenital anomaly of lumbar vertebra (disorder)
Congenital Disease	SNOMED	93011006	Congenital anomaly of nasal bone (disorder)
Congenital Disease	SNOMED	93012004	Congenital anomaly of nasal turbinate (disorder)
Congenital Disease	SNOMED	93014003	Congenital anomaly of palatine bone (disorder)
Congenital Disease	SNOMED	93015002	Congenital anomaly of parietal bone (disorder)
Congenital Disease	SNOMED	93017005	Congenital anomaly of pelvic bones (disorder)
Congenital Disease	SNOMED	93018000	Congenital anomaly of pericardium (disorder)
Congenital Disease	SNOMED	93019008	Congenital anomaly of premaxilla (disorder)
Congenital Disease	SNOMED	93020002	Congenital anomaly of presphenoid bone (disorder)
Congenital Disease	SNOMED	93021003	Congenital anomaly of pubis (disorder)
Congenital Disease	SNOMED	93022005	Congenital anomaly of rib cartilage (disorder)
Congenital Disease	SNOMED	93023000	Congenital anomaly of sacral vertebra (disorder)

Congenital Disease	SNOMED	93024006	Congenital anomaly of squamosal bone (disorder)
Congenital Disease	SNOMED	93025007	Congenital anomaly of sternebra (disorder)
Congenital Disease	SNOMED	93026008	Congenital anomaly of supraoccipital bone (disorder)
Congenital Disease	SNOMED	93027004	Congenital anomaly of thoracic vertebra (disorder)
Congenital Disease	SNOMED	93028009	Congenital anomaly of vomer (disorder)
Congenital Disease	SNOMED	93029001	Congenital anomaly of zygomatic bone (disorder)
Congenital Disease	SNOMED	93030006	Congenital absence of spleen (disorder)
Congenital Disease	SNOMED	93031005	Congenital atresia of inferior vena cava (disorder)
Congenital Disease	SNOMED	93032003	Congenital atresia of intestinal tract (disorder)
Congenital Disease	SNOMED	93033008	Congenital atresia of superior vena cava (disorder)
Congenital Disease	SNOMED	93034002	Congenital atresia of uterus (disorder)
Congenital Disease	SNOMED	93035001	Congenital fenestration of basioccipital bone (disorder)
Congenital Disease	SNOMED	93036000	Congenital fenestration of basisphenoid bone (disorder)
Congenital Disease	SNOMED	93037009	Congenital branched rib (disorder)
Congenital Disease	SNOMED	93038004	Congenital branched rib cartilage (disorder)
Congenital Disease	SNOMED	93039007	Congenital bilobed gallbladder (disorder)
Congenital Disease	SNOMED	93040009	Congenital blepharophimosis (disorder)
Congenital Disease	SNOMED	93044000	Congenital convoluted ureter (disorder)
Congenital Disease	SNOMED	93046003	Congenital cyst of bladder (disorder)
Congenital Disease	SNOMED	93050005	Congenital dilatation of aortic arch (disorder)
Congenital Disease	SNOMED	93051009	Congenital dilatation of atrium (disorder)
Congenital Disease	SNOMED	93052002	Congenital dilatation of bladder (disorder)
Congenital Disease	SNOMED	93053007	Congenital dilatation of cardiac ventricle (disorder)
Congenital Disease	SNOMED	93055000	Congenital dilatation of ductus arteriosus (disorder)
Congenital Disease	SNOMED	93056004	Congenital dilatation of inferior vena cava (disorder)
Congenital Disease	SNOMED	93057008	Congenital dilatation of innominate artery (disorder)
Congenital Disease	SNOMED	93058003	Congenital dilatation of intestinal tract (disorder)
Congenital Disease	SNOMED	93059006	Congenital dilatation of pulmonary artery (disorder)
Congenital Disease	SNOMED	93060001	Congenital dilatation of stomach (disorder)
Congenital Disease	SNOMED	93061002	Congenital dilatation of subclavian artery (disorder)
Congenital Disease	SNOMED	93062009	Congenital dilatation of superior vena cava (disorder)
Congenital Disease	SNOMED	93063004	Congenital diverticulum of intestinal tract (disorder)
Congenital Disease	SNOMED	93064005	Congenital duplication of aorta (disorder)
Congenital Disease	SNOMED	93066007	Congenital downward displacement of stomach (disorder)
Congenital Disease	SNOMED	93067003	Congenital elongation of innominate artery (disorder)
Congenital Disease	SNOMED	93068008	Congenital hypertrophy of adrenal gland (disorder)
Congenital Disease	SNOMED	93069000	Congenital hypertrophy of aortic valve (disorder)
Congenital Disease	SNOMED	93070004	Congenital enlargement of fontanel (disorder)
Congenital Disease	SNOMED	93071000	Congenital hypertrophy of lung (disorder)
Congenital Disease	SNOMED	93072007	Congenital hypertrophy of nasal cavity (disorder)
Congenital Disease	SNOMED	93073002	Congenital hypertrophy of ovary (disorder)
Congenital Disease	SNOMED	93074008	Congenital hypertrophy of fallopian tube (disorder)
Congenital Disease	SNOMED	93075009	Congenital hypertrophy of pulmonary valve (disorder)
Congenital Disease	SNOMED	93076005	Congenital hypertrophy of tricuspid valve (disorder)
Congenital Disease	SNOMED	93077001	Congenital hypertrophy of testis (disorder)
Congenital Disease	SNOMED	93078006	Congenital hypertrophy of cardiac ventricle (disorder)
Congenital Disease	SNOMED	93080000	Congenital fenestration of exoccipital bone (disorder)
Congenital Disease	SNOMED	93081001	Congenital exophthalmos (disorder)
Congenital Disease	SNOMED	93082008	Congenital fenestration of frontal bone (disorder)
Congenital Disease	SNOMED	93083003	Congenital fenestration of interparietal bone (disorder)
Congenital Disease	SNOMED	93084009	Congenital fenestration of nasal bone (disorder)
Congenital Disease	SNOMED	93085005	Congenital fenestration of parietal bone (disorder)
Congenital Disease	SNOMED	93086006	Congenital fenestration of premaxilla (disorder)
Congenital Disease	SNOMED	93087002	Congenital fenestration of presphenoid bone (disorder)
Congenital Disease	SNOMED	93088007	Congenital fenestration of squamosal bone (disorder)
Congenital Disease	SNOMED	93089004	Congenital fenestration of supraoccipital bone (disorder)
Congenital Disease	SNOMED	93091007	Congenital fistula of intestinal tract (disorder)
Congenital Disease	SNOMED	93093005	Congenital focal enlargement of rib (disorder)
Congenital Disease	SNOMED	93095003	Congenital hyperextension of limb (disorder)
Congenital Disease	SNOMED	93096002	Lack of ossification of fibula (disorder)
Congenital Disease	SNOMED	93098001	Lack of ossification of frontal bone (disorder)
Congenital Disease	SNOMED	93100001	Lack of ossification of humerus (disorder)
Congenital Disease	SNOMED	93101002	Lack of ossification of hyoid bone (disorder)
Congenital Disease	SNOMED	93102009	Lack of ossification of ilium (disorder)
Congenital Disease	SNOMED	93103004	Lack of ossification of interparietal bone (disorder)
Congenital Disease	SNOMED	93105006	Lack of ossification of lacrimal bone (disorder)
Congenital Disease	SNOMED	93106007	Lack of ossification of mandible (disorder)
Congenital Disease	SNOMED	93107003	Lack of ossification of maxilla (disorder)
Congenital Disease	SNOMED	93108008	Lack of ossification of metacarpal bone (disorder)
Congenital Disease	SNOMED	93109000	Lack of ossification of metatarsal bone (disorder)
Congenital Disease	SNOMED	93110005	Lack of ossification of nasal bone (disorder)
Congenital Disease	SNOMED	93111009	Lack of ossification of palatine bone (disorder)
Congenital Disease	SNOMED	93112002	Lack of ossification of parietal bone (disorder)
Congenital Disease	SNOMED	93113007	Lack of ossification of premaxilla (disorder)
Congenital Disease	SNOMED	93114001	Lack of ossification of presphenoid bone (disorder)

Congenital Disease	SNOMED	93116004	Lack of ossification of radius (disorder)
Congenital Disease	SNOMED	93117008	Lack of ossification of rib (disorder)
Congenital Disease	SNOMED	93118003	Lack of ossification of scapula (disorder)
Congenital Disease	SNOMED	93119006	Lack of ossification of squamosal bone (disorder)
Congenital Disease	SNOMED	93120000	Lack of ossification of sternebra (disorder)
Congenital Disease	SNOMED	93121001	Lack of ossification of supraoccipital bone (disorder)
Congenital Disease	SNOMED	93122008	Lack of ossification of talus (disorder)
Congenital Disease	SNOMED	93123003	Lack of ossification of tarsal bone (disorder)
Congenital Disease	SNOMED	93124009	Lack of ossification of tibia (disorder)
Congenital Disease	SNOMED	93125005	Lack of ossification of tympanic anulus (disorder)
Congenital Disease	SNOMED	93126006	Lack of ossification of ulna (disorder)
Congenital Disease	SNOMED	93127002	Lack of ossification of vomer (disorder)
Congenital Disease	SNOMED	93128007	Lack of ossification of zygomatic bone (disorder)
Congenital Disease	SNOMED	93131008	Leptocephaly (disorder)
Congenital Disease	SNOMED	93132001	Lethal Kniest-like syndrome (disorder)
Congenital Disease	SNOMED	93167001	Lumbar hemivertebra (disorder)
Congenital Disease	SNOMED	93180003	Macromelia (disorder)
Congenital Disease	SNOMED	93181004	Macrophthalmos (disorder)
Congenital Disease	SNOMED	93232005	Congenital hyperflexion of limb (disorder)
Congenital Disease	SNOMED	93234006	Congenital hypertrophy of mitral valve (disorder)
Congenital Disease	SNOMED	93235007	Congenital hypoplasia of adrenal gland (disorder)
Congenital Disease	SNOMED	93236008	Congenital hypoplasia of alisphenoid bone (disorder)
Congenital Disease	SNOMED	93237004	Congenital hypoplasia of aortic valve (disorder)
Congenital Disease	SNOMED	93239001	Congenital hypoplasia of arch of cervical vertebra (disorder)
Congenital Disease	SNOMED	93240004	Congenital hypoplasia of arch of lumbar vertebra (disorder)
Congenital Disease	SNOMED	93241000	Congenital hypoplasia of arch of sacral vertebra (disorder)
Congenital Disease	SNOMED	93242007	Congenital hypoplasia of arch of thoracic vertebra (disorder)
Congenital Disease	SNOMED	93243002	Congenital hypoplasia of basioccipital bone (disorder)
Congenital Disease	SNOMED	93244008	Congenital hypoplasia of basisphenoid bone (disorder)
Congenital Disease	SNOMED	93245009	Congenital hypoplasia of bladder (disorder)
Congenital Disease	SNOMED	93246005	Congenital hypoplasia of calcaneus (disorder)
Congenital Disease	SNOMED	93247001	Congenital hypoplasia of cardiac ventricle (disorder)
Congenital Disease	SNOMED	93248006	Congenital hypoplasia of carpal bone (disorder)
Congenital Disease	SNOMED	93249003	Congenital hypoplasia of cerebrum (disorder)
Congenital Disease	SNOMED	93250003	Congenital hypoplasia of clavicle (disorder)
Congenital Disease	SNOMED	93252006	Congenital hypoplasia of epididymis (disorder)
Congenital Disease	SNOMED	93253001	Congenital hypoplasia of exoccipital bone (disorder)
Congenital Disease	SNOMED	93254007	Congenital hypoplasia of eye bulge (disorder)
Congenital Disease	SNOMED	93255008	Congenital hypoplasia of femur (disorder)
Congenital Disease	SNOMED	93256009	Congenital hypoplasia of fibula (disorder)
Congenital Disease	SNOMED	93258005	Congenital hypoplasia of frontal bone (disorder)
Congenital Disease	SNOMED	93259002	Congenital hypoplasia of gallbladder (disorder)
Congenital Disease	SNOMED	93262004	Congenital hypoplasia of heart (disorder)
Congenital Disease	SNOMED	93264003	Congenital hypoplasia of humerus (disorder)
Congenital Disease	SNOMED	93265002	Congenital hypoplasia of hyoid bone (disorder)
Congenital Disease	SNOMED	93266001	Congenital hypoplasia of ilium (disorder)
Congenital Disease	SNOMED	93267005	Congenital hypoplasia of interparietal bone (disorder)
Congenital Disease	SNOMED	93268000	Congenital hypoplasia of intestinal tract (disorder)
Congenital Disease	SNOMED	93269008	Congenital hypoplasia of ischium (disorder)
Congenital Disease	SNOMED	93270009	Congenital hypoplasia of lacrimal bone (disorder)
Congenital Disease	SNOMED	93271008	Congenital hypoplasia of mitral valve (disorder)
Congenital Disease	SNOMED	93272001	Congenital hypoplasia of metacarpal bone (disorder)
Congenital Disease	SNOMED	93273006	Congenital hypoplasia of metatarsal bone (disorder)
Congenital Disease	SNOMED	93274000	Congenital hypoplasia of nasal bone (disorder)
Congenital Disease	SNOMED	93275004	Congenital hypoplasia of nasal cavity (disorder)
Congenital Disease	SNOMED	93276003	Congenital hypoplasia of nasal turbinate (disorder)
Congenital Disease	SNOMED	93277007	Congenital hypoplasia of nasal septum (disorder)
Congenital Disease	SNOMED	93278002	Congenital hypoplasia of nose (disorder)
Congenital Disease	SNOMED	93279005	Congenital hypoplasia of ovary (disorder)
Congenital Disease	SNOMED	93280008	Congenital hypoplasia of fallopian tube (disorder)
Congenital Disease	SNOMED	93281007	Congenital hypoplasia of palatine bone (disorder)
Congenital Disease	SNOMED	93282000	Congenital hypoplasia of parietal bone (disorder)
Congenital Disease	SNOMED	93284004	Congenital hypoplasia of premaxilla (disorder)
Congenital Disease	SNOMED	93285003	Congenital hypoplasia of presphenoid bone (disorder)
Congenital Disease	SNOMED	93286002	Congenital hypoplasia of pubis (disorder)
Congenital Disease	SNOMED	93287006	Congenital hypoplasia of pulmonary valve (disorder)
Congenital Disease	SNOMED	93288001	Congenital hypoplasia of radius (disorder)
Congenital Disease	SNOMED	93290000	Congenital hypoplasia of renal pelvis (disorder)
Congenital Disease	SNOMED	93291001	Congenital hypoplasia of rib (disorder)
Congenital Disease	SNOMED	93292008	Congenital hypoplasia of spleen (disorder)
Congenital Disease	SNOMED	93293003	Congenital hypoplasia of squamosal bone (disorder)
Congenital Disease	SNOMED	93294009	Congenital hypoplasia of supraoccipital bone (disorder)
Congenital Disease	SNOMED	93295005	Congenital hypoplasia of talus (disorder)
Congenital Disease	SNOMED	93296006	Congenital hypoplasia of tarsal bone (disorder)
Congenital Disease	SNOMED	93297002	Congenital hypoplasia of thymus (disorder)

Congenital Disease	SNOMED	93298007	Congenital hypoplasia of tibia (disorder)
Congenital Disease	SNOMED	93299004	Congenital hypoplasia of tympanic anulus (disorder)
Congenital Disease	SNOMED	93300007	Congenital hypoplasia of ulna (disorder)
Congenital Disease	SNOMED	93301006	Congenital hypoplasia of vas deferens (disorder)
Congenital Disease	SNOMED	93302004	Congenital hypoplasia of vomer (disorder)
Congenital Disease	SNOMED	93303009	Congenital hypoplasia of zygomatic bone (disorder)
Congenital Disease	SNOMED	93304003	Congenital malposition of adrenal gland (disorder)
Congenital Disease	SNOMED	93305002	Congenital malposition of aorta (disorder)
Congenital Disease	SNOMED	93307005	Congenital malposition of arch of cervical vertebra (disorder)
Congenital Disease	SNOMED	93308000	Congenital malposition of arch of lumbar vertebra (disorder)
Congenital Disease	SNOMED	93309008	Congenital malposition of arch of sacral vertebra (disorder)
Congenital Disease	SNOMED	93310003	Congenital malposition of arch of thoracic vertebra (disorder)
Congenital Disease	SNOMED	93311004	Congenital malposition of calcaneus (disorder)
Congenital Disease	SNOMED	93312006	Congenital malposition of carotid artery (disorder)
Congenital Disease	SNOMED	93313001	Congenital malposition of carpal bone (disorder)
Congenital Disease	SNOMED	93315008	Congenital malposition of cervical vertebra (disorder)
Congenital Disease	SNOMED	93317000	Congenital malposition of digit (disorder)
Congenital Disease	SNOMED	93318005	Congenital malposition of ductus arteriosus (disorder)
Congenital Disease	SNOMED	93319002	Congenital malposition of epididymis (disorder)
Congenital Disease	SNOMED	93320008	Congenital malposition of eye (disorder)
Congenital Disease	SNOMED	93321007	Congenital malposition of femur (disorder)
Congenital Disease	SNOMED	93322000	Congenital malposition of fibula (disorder)
Congenital Disease	SNOMED	93324004	Congenital malposition of gallbladder (disorder)
Congenital Disease	SNOMED	93326002	Congenital malposition of humerus (disorder)
Congenital Disease	SNOMED	93327006	Congenital malposition of ilium (disorder)
Congenital Disease	SNOMED	93328001	Congenital malposition of inferior vena cava (disorder)
Congenital Disease	SNOMED	93329009	Congenital malposition of innominate artery (disorder)
Congenital Disease	SNOMED	93330004	Congenital malposition of intestinal tract (disorder)
Congenital Disease	SNOMED	93331000	Congenital malposition of ischium (disorder)
Congenital Disease	SNOMED	93332007	Congenital malposition of liver (disorder)
Congenital Disease	SNOMED	93333002	Congenital malposition of lumbar vertebra (disorder)
Congenital Disease	SNOMED	93334008	Congenital malposition of lung (disorder)
Congenital Disease	SNOMED	93335009	Congenital malposition of metacarpal bone (disorder)
Congenital Disease	SNOMED	93336005	Congenital malposition of metatarsal bone (disorder)
Congenital Disease	SNOMED	93337001	Congenital malposition of nares (disorder)
Congenital Disease	SNOMED	93338006	Congenital malposition of nasal turbinate (disorder)
Congenital Disease	SNOMED	93339003	Congenital malposition of nasal septum (disorder)
Congenital Disease	SNOMED	93340001	Congenital malposition of nose (disorder)
Congenital Disease	SNOMED	93341002	Congenital malposition of ovary (disorder)
Congenital Disease	SNOMED	93342009	Congenital malposition of fallopian tube (disorder)
Congenital Disease	SNOMED	93343004	Congenital malposition of palate rugae (disorder)
Congenital Disease	SNOMED	93345006	Congenital malposition of pinna (disorder)
Congenital Disease	SNOMED	93346007	Congenital malposition of pubis (disorder)
Congenital Disease	SNOMED	93347003	Congenital malposition of pulmonary artery (disorder)
Congenital Disease	SNOMED	93348008	Congenital malposition of radius (disorder)
Congenital Disease	SNOMED	93349000	Congenital malposition of rib (disorder)
Congenital Disease	SNOMED	93350000	Congenital malposition of sacral vertebra (disorder)
Congenital Disease	SNOMED	93351001	Congenital malposition of spleen (disorder)
Congenital Disease	SNOMED	93352008	Congenital malposition of sternebra (disorder)
Congenital Disease	SNOMED	93353003	Congenital malposition of subclavian artery (disorder)
Congenital Disease	SNOMED	93354009	Congenital malposition of superior vena cava (disorder)
Congenital Disease	SNOMED	93356006	Congenital malposition of talus (disorder)
Congenital Disease	SNOMED	93357002	Congenital malposition of tarsal bone (disorder)
Congenital Disease	SNOMED	93358007	Congenital malposition of testis (disorder)
Congenital Disease	SNOMED	93359004	Congenital malposition of the thyroid gland (disorder)
Congenital Disease	SNOMED	93360009	Congenital malposition of thoracic vertebra (disorder)
Congenital Disease	SNOMED	93361008	Congenital malposition of thymus (disorder)
Congenital Disease	SNOMED	93362001	Congenital malposition of tibia (disorder)
Congenital Disease	SNOMED	93363006	Congenital malposition of trachea (disorder)
Congenital Disease	SNOMED	93364000	Congenital malposition of ulna (disorder)
Congenital Disease	SNOMED	93365004	Congenital malposition of vas deferens (disorder)
Congenital Disease	SNOMED	93367007	Congenital malrotation of limb (disorder)
Congenital Disease	SNOMED	93369005	Congenital microhepatia (disorder)
Congenital Disease	SNOMED	93372003	Congenital misalignment of arch of cervical vertebra (disorder)
Congenital Disease	SNOMED	93373008	Congenital misalignment of centrum of cervical vertebra (disorder)
Congenital Disease	SNOMED	93374002	Congenital misalignment of arch of lumbar vertebra (disorder)
Congenital Disease	SNOMED	93375001	Congenital misalignment of centrum of lumbar vertebra (disorder)
Congenital Disease	SNOMED	93376000	Congenital misalignment of palate rugae (disorder)
Congenital Disease	SNOMED	93377009	Congenital misalignment of pubis (disorder)
Congenital Disease	SNOMED	93378004	Congenital misalignment of rib (disorder)
Congenital Disease	SNOMED	93379007	Congenital misalignment of arch of sacral vertebra (disorder)
Congenital Disease	SNOMED	93380005	Congenital misalignment of centrum of sacral vertebra (disorder)
Congenital Disease	SNOMED	93381009	Congenital misalignment of sternebra (disorder)
Congenital Disease	SNOMED	93382002	Congenital misalignment of arch of thoracic vertebra (disorder)
Congenital Disease	SNOMED	93383007	Congenital misalignment of centrum of thoracic vertebra (disorder)

Congenital Disease	SNOMED	93384001	Congenital stenosis of aortic arch (disorder)
Congenital Disease	SNOMED	93385000	Congenital stenosis of innominate artery (disorder)
Congenital Disease	SNOMED	93386004	Congenital stenosis of intestinal tract (disorder)
Congenital Disease	SNOMED	93387008	Congenital stenosis of stomach (disorder)
Congenital Disease	SNOMED	93388003	Congenital stenosis of subclavian artery (disorder)
Congenital Disease	SNOMED	93390002	Congenital ocular coloboma (disorder)
Congenital Disease	SNOMED	93391003	Congenital protrusion of tongue (disorder)
Congenital Disease	SNOMED	93392005	Congenital short growth of innominate artery (disorder)
Congenital Disease	SNOMED	93394006	Congenital short trunk (disorder)
Congenital Disease	SNOMED	93395007	Congenital small anus (disorder)
Congenital Disease	SNOMED	93396008	Congenital stenosis of carotid artery (disorder)
Congenital Disease	SNOMED	93397004	Congenital thickening of clavicle (disorder)
Congenital Disease	SNOMED	93398009	Congenital thickening of femur (disorder)
Congenital Disease	SNOMED	93399001	Congenital thickening of fibula (disorder)
Congenital Disease	SNOMED	93402000	Congenital thickening of humerus (disorder)
Congenital Disease	SNOMED	93403005	Congenital thickening of ilium (disorder)
Congenital Disease	SNOMED	93404004	Congenital thickening of ischium (disorder)
Congenital Disease	SNOMED	93405003	Congenital thickening of pubis (disorder)
Congenital Disease	SNOMED	93406002	Congenital thickening of radius (disorder)
Congenital Disease	SNOMED	93407006	Congenital thickening of rib (disorder)
Congenital Disease	SNOMED	93408001	Congenital thickening of scapula (disorder)
Congenital Disease	SNOMED	93409009	Congenital thickening of talus (disorder)
Congenital Disease	SNOMED	93410004	Congenital thickening of tarsal bone (disorder)
Congenital Disease	SNOMED	93411000	Congenital thickening of tibia (disorder)
Congenital Disease	SNOMED	93412007	Congenital thickening of ulna (disorder)
Congenital Disease	SNOMED	93413002	Thoracoceloschisis (disorder)
Congenital Disease	SNOMED	93414008	Congenital thoracostenosis (disorder)
Congenital Disease	SNOMED	93415009	Congenital transposition of azygos vein (disorder)
Congenital Disease	SNOMED	93429005	Discontinuous rib (disorder)
Congenital Disease	SNOMED	93430000	Domed head (disorder)
Congenital Disease	SNOMED	93437002	Dumbbell ossification of centrum of cervical vertebra (disorder)
Congenital Disease	SNOMED	93438007	Dumbbell ossification of centrum of lumbar vertebra (disorder)
Congenital Disease	SNOMED	93439004	Dumbbell ossification of centrum of sacral vertebra (disorder)
Congenital Disease	SNOMED	93440002	Dumbbell ossification of centrum of thoracic vertebra (disorder)
Congenital Disease	SNOMED	93441003	Dumbbell-shaped cartilaginous centrum of cervical vertebra (disorder)
Congenital Disease	SNOMED	93442005	Dumbbell-shaped cartilaginous centrum of lumbar vertebra (disorder)
Congenital Disease	SNOMED	93443000	Dumbbell-shaped cartilaginous centrum of sacral vertebra (disorder)
Congenital Disease	SNOMED	93444006	Dumbbell-shaped cartilaginous centrum of thoracic vertebra (disorder)
Congenital Disease	SNOMED	93445007	Dynamic subaortic stenosis (disorder)
Congenital Disease	SNOMED	93460005	Full supernumerary rib (disorder)
Congenital Disease	SNOMED	93466004	Gonadal dysgenesis with auditory dysfunction, autosomal recessive inheritance (disorder)
Congenital Disease	SNOMED	93556005	Hologastroschisis (disorder)
Congenital Disease	SNOMED	93557001	Holorachischisis (disorder)
Congenital Disease	SNOMED	93562000	Incomplete ossification of alisphenoid bone (disorder)
Congenital Disease	SNOMED	93564004	Incomplete ossification of arch of cervical vertebra (disorder)
Congenital Disease	SNOMED	93565003	Incomplete ossification of arch of lumbar vertebra (disorder)
Congenital Disease	SNOMED	93566002	Incomplete ossification of arch of sacral vertebra (disorder)
Congenital Disease	SNOMED	93567006	Incomplete ossification of arch of thoracic vertebra (disorder)
Congenital Disease	SNOMED	93568001	Incomplete ossification of basioccipital bone (disorder)
Congenital Disease	SNOMED	93569009	Incomplete ossification of basisphenoid bone (disorder)
Congenital Disease	SNOMED	93570005	Incomplete ossification of calcaneus (disorder)
Congenital Disease	SNOMED	93571009	Incomplete ossification of carpal bone (disorder)
Congenital Disease	SNOMED	93573007	Incomplete ossification of centrum of cervical vertebra (disorder)
Congenital Disease	SNOMED	93574001	Incomplete ossification of centrum of lumbar vertebra (disorder)
Congenital Disease	SNOMED	93575000	Incomplete ossification of centrum of sacral vertebra (disorder)
Congenital Disease	SNOMED	93576004	Incomplete ossification of centrum of thoracic vertebra (disorder)
Congenital Disease	SNOMED	93577008	Incomplete ossification of clavicle (disorder)
Congenital Disease	SNOMED	93578003	Incomplete ossification of exoccipital bone (disorder)
Congenital Disease	SNOMED	93579006	Incomplete ossification of femur (disorder)
Congenital Disease	SNOMED	93580009	Incomplete ossification of fibula (disorder)
Congenital Disease	SNOMED	93582001	Incomplete ossification of frontal bone (disorder)
Congenital Disease	SNOMED	93584000	Incomplete ossification of humerus (disorder)
Congenital Disease	SNOMED	93585004	Incomplete ossification of hyoid bone (disorder)
Congenital Disease	SNOMED	93587007	Incomplete ossification of interparietal bone (disorder)
Congenital Disease	SNOMED	93588002	Incomplete ossification of ischium (disorder)
Congenital Disease	SNOMED	93589005	Incomplete ossification of lacrimal bone (disorder)
Congenital Disease	SNOMED	93590001	Incomplete ossification of mandible (disorder)
Congenital Disease	SNOMED	93591002	Incomplete ossification of maxilla (disorder)
Congenital Disease	SNOMED	93592009	Incomplete ossification of metacarpal bone (disorder)
Congenital Disease	SNOMED	93593004	Incomplete ossification of metatarsal bone (disorder)
Congenital Disease	SNOMED	93594005	Incomplete ossification of nasal bone (disorder)
Congenital Disease	SNOMED	93595006	Incomplete ossification of palatine bone (disorder)
Congenital Disease	SNOMED	93596007	Incomplete ossification of parietal bone (disorder)
Congenital Disease	SNOMED	93597003	Incomplete ossification of premaxilla (disorder)

Congenital Disease	SNOMED	93598008	Incomplete ossification of presphenoid bone (disorder)
Congenital Disease	SNOMED	93599000	Incomplete ossification of pubis (disorder)
Congenital Disease	SNOMED	93600002	Incomplete ossification of radius (disorder)
Congenital Disease	SNOMED	93601003	Incomplete ossification of rib (disorder)
Congenital Disease	SNOMED	93602005	Incomplete ossification of scapula (disorder)
Congenital Disease	SNOMED	93603000	Incomplete ossification of squamosal bone (disorder)
Congenital Disease	SNOMED	93604006	Incomplete ossification of sternebra (disorder)
Congenital Disease	SNOMED	93605007	Incomplete ossification of supraoccipital bone (disorder)
Congenital Disease	SNOMED	93606008	Incomplete ossification of talus (disorder)
Congenital Disease	SNOMED	93607004	Incomplete ossification of tarsal bone (disorder)
Congenital Disease	SNOMED	93608009	Incomplete ossification of tibia (disorder)
Congenital Disease	SNOMED	93609001	Incomplete ossification of tympanic anulus (disorder)
Congenital Disease	SNOMED	93610006	Incomplete ossification of ulna (disorder)
Congenital Disease	SNOMED	93611005	Incomplete ossification of vomer (disorder)
Congenital Disease	SNOMED	93612003	Incomplete ossification of zygomatic bone (disorder)
Congenital Disease	SNOMED	93615001	Intercostal rib (disorder)
Congenital Disease	SNOMED	93617009	Lack of ossification of alisphenoid bone (disorder)
Congenital Disease	SNOMED	93619007	Lack of ossification of arch of cervical vertebra (disorder)
Congenital Disease	SNOMED	93620001	Lack of ossification of arch of lumbar vertebra (disorder)
Congenital Disease	SNOMED	93621002	Lack of ossification of arch of sacral vertebra (disorder)
Congenital Disease	SNOMED	93622009	Lack of ossification of arch of thoracic vertebra (disorder)
Congenital Disease	SNOMED	93623004	Congenital lack of ossification of auditory ossicles (disorder)
Congenital Disease	SNOMED	93624005	Lack of ossification of basioccipital bone (disorder)
Congenital Disease	SNOMED	93625006	Lack of ossification of basisphenoid bone (disorder)
Congenital Disease	SNOMED	93626007	Lack of ossification of calcaneus (disorder)
Congenital Disease	SNOMED	93627003	Lack of ossification of carpal bone (disorder)
Congenital Disease	SNOMED	93629000	Lack of ossification of centrum of cervical vertebra (disorder)
Congenital Disease	SNOMED	93630005	Lack of ossification of centrum of lumbar vertebra (disorder)
Congenital Disease	SNOMED	93631009	Lack of ossification of centrum of sacral vertebra (disorder)
Congenital Disease	SNOMED	93632002	Lack of ossification of centrum of thoracic vertebra (disorder)
Congenital Disease	SNOMED	93633007	Lack of ossification of clavicle (disorder)
Congenital Disease	SNOMED	93634001	Lack of ossification of exoccipital bone (disorder)
Congenital Disease	SNOMED	93635000	Lack of ossification of femur (disorder)
Congenital Disease	SNOMED	94150003	Membranous ventricular septum defect (disorder)
Congenital Disease	SNOMED	94181000119108	Congenital duplication of renal collecting system without obstruction (disorder)
Congenital Disease	SNOMED	943003	Congenital retinal aneurysm (disorder)
Congenital Disease	SNOMED	94684003	Microblepharia (disorder)
Congenital Disease	SNOMED	94685002	Microdactyly (disorder)
Congenital Disease	SNOMED	94702005	Multiple congenital cardiac defects (disorder)
Congenital Disease	SNOMED	94706008	Muscular ventricular septum defect (disorder)
Congenital Disease	SNOMED	94720001	Myxoid transformation of tricuspid valve (disorder)
Congenital Disease	SNOMED	95198001	Pure gonadal dysgenesis 46,XX (disorder)
Congenital Disease	SNOMED	95202004	Coloboma of eyelid (disorder)
Congenital Disease	SNOMED	95218005	Pure gonadal dysgenesis 46,XY (disorder)
Congenital Disease	SNOMED	95219002	Pure gonadal dysgenesis (disorder)
Congenital Disease	SNOMED	95233002	Retrocaval ureter (disorder)
Congenital Disease	SNOMED	95234008	Retroesophageal aortic arch (disorder)
Congenital Disease	SNOMED	95235009	Retroesophageal carotid artery (disorder)
Congenital Disease	SNOMED	95236005	Retroesophageal pulmonary artery (disorder)
Congenital Disease	SNOMED	95237001	Retroesophageal subclavian artery (disorder)
Congenital Disease	SNOMED	95241002	Rhinocephaly (disorder)
Congenital Disease	SNOMED	95242009	Right-sided pulmonary arterial trunk (disorder)
Congenital Disease	SNOMED	95243004	Rolland-Debuqois syndrome (disorder)
Congenital Disease	SNOMED	95245006	Sacral hemivertebra (disorder)
Congenital Disease	SNOMED	95265004	Short supernumerary rib (disorder)
Congenital Disease	SNOMED	95266003	Single naris (disorder)
Congenital Disease	SNOMED	95268002	Sinus venosus atrial septal defect (disorder)
Congenital Disease	SNOMED	9527009	Tetrasomy 12p syndrome (disorder)
Congenital Disease	SNOMED	95282002	Supernumerary arch of cervical vertebra (disorder)
Congenital Disease	SNOMED	95283007	Supernumerary arch of lumbar vertebra (disorder)
Congenital Disease	SNOMED	95284001	Supernumerary arch of sacral vertebra (disorder)
Congenital Disease	SNOMED	95285000	Supernumerary arch of thoracic vertebra (disorder)
Congenital Disease	SNOMED	95286004	Supernumerary azygos vein (disorder)
Congenital Disease	SNOMED	95287008	Supernumerary calcaneus (disorder)
Congenital Disease	SNOMED	95289006	Supernumerary centrum of cervical vertebra (disorder)
Congenital Disease	SNOMED	95290002	Supernumerary centrum of lumbar vertebra (disorder)
Congenital Disease	SNOMED	95291003	Supernumerary centrum of sacral vertebra (disorder)
Congenital Disease	SNOMED	95292005	Supernumerary centrum of thoracic vertebra (disorder)
Congenital Disease	SNOMED	95293000	Supernumerary cervical vertebra (disorder)
Congenital Disease	SNOMED	95295007	Supernumerary fused sternebra (disorder)
Congenital Disease	SNOMED	95297004	Supernumerary liver lobe (disorder)
Congenital Disease	SNOMED	95298009	Supernumerary lumbar vertebra (disorder)
Congenital Disease	SNOMED	95299001	Supernumerary metatarsal bone (disorder)
Congenital Disease	SNOMED	95300009	Supernumerary sacral vertebra (disorder)

Congenital Disease	SNOMED	95301008	Supernumerary tarsal bone (disorder)
Congenital Disease	SNOMED	95302001	Supernumerary thoracic vertebra (disorder)
Congenital Disease	SNOMED	95304000	Thoracic hemivertebra (disorder)
Congenital Disease	SNOMED	95305004	Thoracoschisis (disorder)
Congenital Disease	SNOMED	95339000	Distichiasis (disorder)
Congenital Disease	SNOMED	95441000	Pulmonary artery stenosis (disorder)
Congenital Disease	SNOMED	95462004	Anomaly of sex chromosome (disorder)
Congenital Disease	SNOMED	95463009	Congenital anomaly of joint (disorder)
Congenital Disease	SNOMED	95464003	Congenital absence of cranial vault (disorder)
Congenital Disease	SNOMED	95465002	Congenital claw toe (disorder)
Congenital Disease	SNOMED	95466001	Congenital perforation of nasal septum (disorder)
Congenital Disease	SNOMED	95467005	Congenital tracheomalacia (disorder)
Congenital Disease	SNOMED	95468000	Congenital bronchomalacia (disorder)
Congenital Disease	SNOMED	95469008	Abnormal plantar creases (disorder)
Congenital Disease	SNOMED	95470009	Congenital anomaly of digestive tract (disorder)
Congenital Disease	SNOMED	95471008	Congenital arteriovenous malformation of gastrointestinal tract (disorder)
Congenital Disease	SNOMED	95472001	Multiple gastrointestinal atresias (disorder)
Congenital Disease	SNOMED	95474000	Arteriovenous malformation of kidney (disorder)
Congenital Disease	SNOMED	95477007	Congenital degeneration of nervous system (disorder)
Congenital Disease	SNOMED	95478002	Congenital sacral meningocele (disorder)
Congenital Disease	SNOMED	95479005	Congenital sclerocornea (disorder)
Congenital Disease	SNOMED	95480008	Anterior lenticonus (disorder)
Congenital Disease	SNOMED	95481007	Anterior lentiglobus (disorder)
Congenital Disease	SNOMED	95482000	Posterior lenticonus (disorder)
Congenital Disease	SNOMED	95483005	Posterior lentiglobus (disorder)
Congenital Disease	SNOMED	95484004	Congenital pigmentation of lens (disorder)
Congenital Disease	SNOMED	95485003	Congenital anterior capsular pigmentation (disorder)
Congenital Disease	SNOMED	95490000	Congenital anomaly of choroid (disorder)
Congenital Disease	SNOMED	95491001	Congenital hypoplasia of choroid (disorder)
Congenital Disease	SNOMED	95492008	Congenital hypopigmentation of choroid (disorder)
Congenital Disease	SNOMED	95493003	Congenital retinoschisis (disorder)
Congenital Disease	SNOMED	95494009	Retinal dysplasia (disorder)
Congenital Disease	SNOMED	95495005	Multifocal retinal dysplasia (disorder)
Congenital Disease	SNOMED	95496006	Geographic retinal dysplasia (disorder)
Congenital Disease	SNOMED	95497002	Diffuse retinal dysplasia (disorder)
Congenital Disease	SNOMED	95499004	Hypoplasia of the optic nerve (disorder)
Congenital Disease	SNOMED	95500008	Persistent pupillary membranes (disorder)
Congenital Disease	SNOMED	95501007	Retinal arteriovenous malformation (disorder)
Congenital Disease	SNOMED	95502000	Congenital anomaly of optic nerve (disorder)
Congenital Disease	SNOMED	95503005	Macropalpebral fissure (disorder)
Congenital Disease	SNOMED	95504004	Ectopic cilia of eyelid (disorder)
Congenital Disease	SNOMED	95505003	Congenitally small punctum lacrimale (disorder)
Congenital Disease	SNOMED	95506002	Congenital displacement of punctum lacrimale (disorder)
Congenital Disease	SNOMED	95507006	Congenital diverticulum of lacrimal canaliculus (disorder)
Congenital Disease	SNOMED	95508001	Congenital obstruction of lacrimal canaliculus (disorder)
Congenital Disease	SNOMED	95514008	Developmental dysplasia of inner ear (disorder)
Congenital Disease	SNOMED	95515009	Low set ears (disorder)
Congenital Disease	SNOMED	95610008	Congenital brain damage (disorder)
Congenital Disease	SNOMED	95659007	Hindbrain hernia headache (disorder)
Congenital Disease	SNOMED	95694000	Retinal depigmentation (disorder)
Congenital Disease	SNOMED	95714006	Hypoplasia of iris (disorder)
Congenital Disease	SNOMED	9634000	Congenital dislocation of radial head (disorder)
Congenital Disease	SNOMED	9660004	Congenital stenosis of trachea (disorder)
Congenital Disease	SNOMED	9721000119107	Malformation of central nervous system of fetus (disorder)
Congenital Disease	SNOMED	9723006	Hyperphosphatasemia with bone disease (disorder)
Congenital Disease	SNOMED	9740002	Macroencephaly (disorder)
Congenital Disease	SNOMED	975000	Anorectal agenesis (disorder)
Congenital Disease	SNOMED	9839007	Anomaly of chromosome pair 20 (disorder)
Congenital Disease	SNOMED	9904008	Congenital anomaly of cardiovascular system (disorder)
Congenital Disease	SNOMED	9989000	Congenital anomaly of toe (disorder)
Connective Tissue Disease	ICD10	J990	Rheumatoid lung disease
Connective Tissue Disease	ICD10	L405	Arthropathic psoriasis
Connective Tissue Disease	ICD10	L930	Lupus erythematosus
Connective Tissue Disease	ICD10	L931	Subacute cutaneous lupus erythematosus
Connective Tissue Disease	ICD10	L932	Other local lupus erythematosus
Connective Tissue Disease	ICD10	L940	Localized scleroderma [morphea]
Connective Tissue Disease	ICD10	L941	Linear scleroderma
Connective Tissue Disease	ICD10	M05	Seropositive rheumatoid arthritis
Connective Tissue Disease	ICD10	M06	Seronegative rheumatoid arthritis
Connective Tissue Disease	ICD10	M07	Distal interphalangeal psoriatic arthropathy
Connective Tissue Disease	ICD10	M09	Juvenile arthritis in psoriasis
Connective Tissue Disease	ICD10	M315	Giant cell arteritis with polymyalgia rheumatica
Connective Tissue Disease	ICD10	M32	Systemic lupus erythematosus (SLE)
Connective Tissue Disease	ICD10	M34	Systemic sclerosis
Connective Tissue Disease	ICD10	M350	Sicca syndrome [Sjögren]

Connective Tissue Disease	ICD10	M353	Polymyalgia rheumatica
Connective Tissue Disease	SNOMED	1048311000000106	Rheumatoid Arthritis Impact of Disease questionnaire
Connective Tissue Disease	SNOMED	10713006	Fibrosing alveolitis associated with rheumatoid arthritis
Connective Tissue Disease	SNOMED	1110961000000108	QOF (Quality and Outcomes Framework) rheumatoid arthritis quality indicator-related care invitation
Connective Tissue Disease	SNOMED	15084002	Lupus erythematosus profundus
Connective Tissue Disease	SNOMED	1573180000000000	Vibration white finger
Connective Tissue Disease	SNOMED	1573181000006104	Vibration white finger
Connective Tissue Disease	SNOMED	161567008	H/O: rheumatoid arthritis
Connective Tissue Disease	SNOMED	165927006	Lupus erythematosus (LE) cells present
Connective Tissue Disease	SNOMED	167901007	Synovial fluid: lupus erythematosus cells
Connective Tissue Disease	SNOMED	1728150000000000	Systemic lupus erythematosus encephalitis
Connective Tissue Disease	SNOMED	1728151000006102	Systemic lupus erythematosus encephalitis
Connective Tissue Disease	SNOMED	1875560000000000	Rheumatoid arthritis monitoring invitation
Connective Tissue Disease	SNOMED	1875561000006103	Rheumatoid arthritis monitoring invitation
Connective Tissue Disease	SNOMED	1875570000000000	Rheumatoid arthritis monitoring telephone invitation
Connective Tissue Disease	SNOMED	1875571000006105	Rheumatoid arthritis monitoring telephone invitation
Connective Tissue Disease	SNOMED	1875580000000000	Rheumatoid arthritis monitoring verbal invitation
Connective Tissue Disease	SNOMED	1875581000006108	Rheumatoid arthritis monitoring verbal invitation
Connective Tissue Disease	SNOMED	1875590000000000	Rheumatoid arthritis monitoring invitation first letter
Connective Tissue Disease	SNOMED	1875591000006106	Rheumatoid arthritis monitoring invitation first letter
Connective Tissue Disease	SNOMED	1875600000000000	Rheumatoid arthritis monitoring invitation second letter
Connective Tissue Disease	SNOMED	1875601000006103	Rheumatoid arthritis monitoring invitation second letter
Connective Tissue Disease	SNOMED	1875610000000000	Rheumatoid arthritis monitoring invitation third letter
Connective Tissue Disease	SNOMED	1875611000006100	Rheumatoid arthritis monitoring invitation third letter
Connective Tissue Disease	SNOMED	1932121000006100	3D study - problems with rheumatoid arthritis management
Connective Tissue Disease	SNOMED	193248005	Myopathy due to disseminated lupus erythematosus
Connective Tissue Disease	SNOMED	193250002	Myopathy due to rheumatoid arthritis
Connective Tissue Disease	SNOMED	193252005	Myopathy due to scleroderma
Connective Tissue Disease	SNOMED	193253000	Myopathy due to Sjogren's disease
Connective Tissue Disease	SNOMED	19514005	Arthritis mutilans
Connective Tissue Disease	SNOMED	196133001	Lung disease with systemic sclerosis
Connective Tissue Disease	SNOMED	196137000	Lung disease with SjÃ¶gren disease
Connective Tissue Disease	SNOMED	196138005	Lung disease with systemic lupus erythematosus
Connective Tissue Disease	SNOMED	200936003	Lupus erythematosus NOS
Connective Tissue Disease	SNOMED	200937007	Lupus erythematosus chronicus
Connective Tissue Disease	SNOMED	200939005	Lupus erythematosus migrans
Connective Tissue Disease	SNOMED	200940007	Lupus erythematosus nodularis
Connective Tissue Disease	SNOMED	200941006	Lupus erythematosus tumidus
Connective Tissue Disease	SNOMED	200942004	Lupus erythematosus unguium mutilans
Connective Tissue Disease	SNOMED	200956002	Psoriasis spondylitica
Connective Tissue Disease	SNOMED	201048007	Circumscribed scleroderma
Connective Tissue Disease	SNOMED	201436003	Drug-induced systemic lupus erythematosus
Connective Tissue Disease	SNOMED	201443009	Systemic sclerosis induced by drugs and chemicals
Connective Tissue Disease	SNOMED	201764007	Rheumatoid arthritis of cervical spine
Connective Tissue Disease	SNOMED	201766009	Rheumatoid arthritis of shoulder
Connective Tissue Disease	SNOMED	201767000	Rheumatoid arthritis of sternoclavicular joint
Connective Tissue Disease	SNOMED	201768005	Rheumatoid arthritis of acromioclavicular joint
Connective Tissue Disease	SNOMED	201769002	Rheumatoid arthritis of elbow
Connective Tissue Disease	SNOMED	201770001	Rheumatoid arthritis of distal radio-ulnar joint
Connective Tissue Disease	SNOMED	201771002	Rheumatoid arthritis of wrist
Connective Tissue Disease	SNOMED	201772009	Rheum. arth. - hand joint
Connective Tissue Disease	SNOMED	201773004	Rheumatoid arthritis of PIP joint of finger
Connective Tissue Disease	SNOMED	201774005	Rheumatoid arthritis of DIP joint of finger
Connective Tissue Disease	SNOMED	201775006	Rheum. arth. - hip joint
Connective Tissue Disease	SNOMED	201776007	Rheumatoid arthritis of sacro-iliac joint
Connective Tissue Disease	SNOMED	201777003	Rheum. arth. - knee joint
Connective Tissue Disease	SNOMED	201778008	Rheumatoid arthritis of tibiofibular joint
Connective Tissue Disease	SNOMED	201779000	Rheumatoid arthritis of ankle
Connective Tissue Disease	SNOMED	201780002	Rheumatoid arthritis of subtalar joint
Connective Tissue Disease	SNOMED	201781003	Rheumatoid arthritis of talonavicular joint
Connective Tissue Disease	SNOMED	201783000	Rheumatoid arthritis of 1st MTP joint
Connective Tissue Disease	SNOMED	201784006	Rheumatoid arthritis of lesser MTP joint
Connective Tissue Disease	SNOMED	201785007	Rheumatoid arthritis of IP joint of toe
Connective Tissue Disease	SNOMED	201791009	Flare of rheumatoid arthritis
Connective Tissue Disease	SNOMED	206541009	Scleroderma in newborn
Connective Tissue Disease	SNOMED	22784002	Linear scleroderma
Connective Tissue Disease	SNOMED	231041000000107	Delivery of rehabilitation for rheumatoid arthritis
Connective Tissue Disease	SNOMED	234034005	Vibration white finger
Connective Tissue Disease	SNOMED	236502006	Renal involvement in scleroderma
Connective Tissue Disease	SNOMED	236503001	Acute scleroderma renal crisis
Connective Tissue Disease	SNOMED	239791005	[X]Other seropositive rheumatoid arthritis
Connective Tissue Disease	SNOMED	239792003	Seronegative rheumatoid arthritis
Connective Tissue Disease	SNOMED	239793008	[X]Rheumatoid arthritis+involvement/other organs or systems
Connective Tissue Disease	SNOMED	239803008	Juvenile arthritis in psoriasis

Connective Tissue Disease	SNOMED	239812005	Distal interphalangeal psoriatic arthropathy
Connective Tissue Disease	SNOMED	239887007	Systemic lupus erythematosus with organ or sys involv
Connective Tissue Disease	SNOMED	239891002	SCLE - Subacute cutaneous lupus erythematosus
Connective Tissue Disease	SNOMED	239938009	Giant cell arteritis with polymyalgia rheumatica
Connective Tissue Disease	SNOMED	266261006	Raynaud's phenomenon
Connective Tissue Disease	SNOMED	287006005	Rheumatoid arthritis - multiple joint
Connective Tissue Disease	SNOMED	298285004	Systemic sclerosis with limited cutaneous involvement
Connective Tissue Disease	SNOMED	302896008	Keratoconjunctivitis sicca
Connective Tissue Disease	SNOMED	308143008	Seropositive errosive rheumatoid arthritis
Connective Tissue Disease	SNOMED	309762007	Systemic lupus erythematosus with pericarditis
Connective Tissue Disease	SNOMED	31848007	CREST syndrome
Connective Tissue Disease	SNOMED	33339001	Psoriatic arthropathy
Connective Tissue Disease	SNOMED	33719002	Rheumatoid nodule
Connective Tissue Disease	SNOMED	398640008	Caplans syndrome
Connective Tissue Disease	SNOMED	398726004	Rheumatoid lung disease
Connective Tissue Disease	SNOMED	400054000	Rheumatoid vasculitis
Connective Tissue Disease	SNOMED	429192004	Rheumatoid arthritis of other tarsal joint
Connective Tissue Disease	SNOMED	441870009	Disease activity score 28 joint in rheumatoid arthritis
Connective Tissue Disease	SNOMED	443720007	Family history of systemic sclerosis
Connective Tissue Disease	SNOMED	444133002	Progressive systemic sclerosis
Connective Tissue Disease	SNOMED	54072008	Libman-Sacks disease
Connective Tissue Disease	SNOMED	55464009	Disseminated lupus erythematosus
Connective Tissue Disease	SNOMED	57160007	Felty's syndrome
Connective Tissue Disease	SNOMED	65323003	Polymyalgia rheumatica
Connective Tissue Disease	SNOMED	68815009	Lupus nephritis
Connective Tissue Disease	SNOMED	7119001	[X]Other local lupus erythematosus
Connective Tissue Disease	SNOMED	763259004	Disease activity score in rheumatoid arthritis
Connective Tissue Disease	SNOMED	805941000000105	Exception reporting: rheumatoid arthritis quality indicators
Connective Tissue Disease	SNOMED	805961000000106	Except rheumatoid arthritis quality indicator: pt unsuitable
Connective Tissue Disease	SNOMED	805981000000102	Except rheumatoid arthritis qual indicator: informed dissent
Connective Tissue Disease	SNOMED	83901003	Sjogrens syndrome
Connective Tissue Disease	SNOMED	84017003	Rheumatoid bursitis
Connective Tissue Disease	SNOMED	847261000000104	Rheumatoid arthritis annual review
Connective Tissue Disease	SNOMED	882321000000000	Rheumatoid arthritis monitoring invitation
Connective Tissue Disease	SNOMED	882321000000105	Rheumatoid arthritis monitoring invitation
Connective Tissue Disease	SNOMED	882401000000000	Rheumatoid arthritis monitoring invitation first letter
Connective Tissue Disease	SNOMED	882401000000106	Rheumatoid arthritis monitoring invitation first letter
Connective Tissue Disease	SNOMED	882421000000000	Rheumatoid arthritis monitoring invitation second letter
Connective Tissue Disease	SNOMED	882421000000102	Rheumatoid arthritis monitoring invitation second letter
Connective Tissue Disease	SNOMED	882441000000000	Rheumatoid arthritis monitoring invitation third letter
Connective Tissue Disease	SNOMED	882441000000109	Rheumatoid arthritis monitoring invitation third letter
Connective Tissue Disease	SNOMED	882461000000000	Rheumatoid arthritis monitoring verbal invitation
Connective Tissue Disease	SNOMED	882461000000105	Rheumatoid arthritis monitoring verbal invitation
Connective Tissue Disease	SNOMED	882481000000000	Rheumatoid arthritis monitoring telephone invitation
Connective Tissue Disease	SNOMED	882481000000101	Rheumatoid arthritis monitoring telephone invitation
Connective Tissue Disease	SNOMED	89155008	[X]Other forms of systemic sclerosis
Connective Tissue Disease	SNOMED	905531000006108	[RFC] Raynaud's syndrome
Connective Tissue Disease	SNOMED	909191000006103	[RFC] Rheumatoid arthritis
Connective Tissue Disease	SNOMED	956161000006106	Vibration - white finger
Connective Tissue Disease	SNOMED	95644001	Systemic lupus erythematosus encephalitis
Coronary Heart Disease	ICD10	I22	Subsequent myocardial infarction
Coronary Heart Disease	ICD10	I23	Certain current complications following acute myocardial infarction
Coronary Heart Disease	ICD10	I20	Angina pectoris
Coronary Heart Disease	ICD10	I21	Acute myocardial infarction
Coronary Heart Disease	ICD10	I25	Chronic ischaemic heart disease
Coronary Heart Disease	ICD10	I24	Other acute ischaemic heart diseases
Coronary Heart Disease	SNOMED	103011000119106	Coronary arteriosclerosis in patient with history of previous myocardial infarction (situation)
Coronary Heart Disease	SNOMED	15629541000119106	Congestive heart failure stage C due to Ischemic cardiomyopathy (disorder)
Coronary Heart Disease	SNOMED	15629591000119103	Congestive heart failure stage B due to ischemic cardiomyopathy (disorder)
Coronary Heart Disease	SNOMED	15629641000119107	Systolic heart failure stage B due to ischemic cardiomyopathy (disorder)
Coronary Heart Disease	SNOMED	15629741000119102	Systolic heart failure stage C due to ischaemic cardiomyopathy
Coronary Heart Disease	SNOMED	15960061000119102	Unstable angina co-occurrent and due to coronary arteriosclerosis (disorder)
Coronary Heart Disease	SNOMED	15960141000119102	Angina co-occurrent and due to coronary arteriosclerosis (disorder)
Coronary Heart Disease	SNOMED	16754391000119100	Stable angina due to coronary arteriosclerosis (disorder)
Coronary Heart Disease	SNOMED	171223006	Ischemic heart disease screening
Coronary Heart Disease	SNOMED	194821006	Coronary thrombosis not resulting in myocardial infarction
Coronary Heart Disease	SNOMED	194849004	Generalized ischemic myocardial dysfunction
Coronary Heart Disease	SNOMED	233817007	Triple vessel disease of the heart
Coronary Heart Disease	SNOMED	233823002	Silent myocardial ischemia
Coronary Heart Disease	SNOMED	266882009	No FH: Ischaemic heart disease
Coronary Heart Disease	SNOMED	266895004	FH: Ischaemic heart dis. <60
Coronary Heart Disease	SNOMED	266896003	FH: Ischemic heart disease at greater than 60 years
Coronary Heart Disease	SNOMED	275931000	Aspirin prophylaxis - IHD
Coronary Heart Disease	SNOMED	297242006	Family history of ischemic heart disease

Coronary Heart Disease	SNOMED	308068007	H/O: Treatment for ischaemic heart disease
Coronary Heart Disease	SNOMED	315026000	Transient myocardial ischaemia
Coronary Heart Disease	SNOMED	315348000	Asymptomatic coronary heart disease
Coronary Heart Disease	SNOMED	315610002	Primary prevention of ischemic heart disease
Coronary Heart Disease	SNOMED	315614006	Coronary heart disease annual review
Coronary Heart Disease	SNOMED	390799004	Coronary heart disease review (procedure)
Coronary Heart Disease	SNOMED	394724007	Coronary heart disease medication review (procedure)
Coronary Heart Disease	SNOMED	413838009	Chronic ischemic heart disease (disorder)
Coronary Heart Disease	SNOMED	413844008	Chronic myocardial ischemia (disorder)
Coronary Heart Disease	SNOMED	414024009	Disorder of coronary artery (disorder)
Coronary Heart Disease	SNOMED	414545008	Ischemic heart disease (disorder)
Coronary Heart Disease	SNOMED	426579008	Coronary heart disease education (procedure)
Coronary Heart Disease	SNOMED	430091005	Family history of coronary arteriosclerosis (situation)
Coronary Heart Disease	SNOMED	443502000	Atherosclerosis of coronary artery (disorder)
Coronary Heart Disease	SNOMED	46109009	Subendocardial ischemia
Coronary Heart Disease	SNOMED	472100003	Ischemic dilated cardiomyopathy due to coronary artery disease (disorder)
Coronary Heart Disease	SNOMED	473146006	Coronary artery disease excluded
Coronary Heart Disease	SNOMED	53741008	Coronary arteriosclerosis
Coronary Heart Disease	SNOMED	67682002	Coronary artery atheroma
Coronary Heart Disease	SNOMED	699195003	Provision of coronary heart disease leaflet
Coronary Heart Disease	SNOMED	699196002	Coronary artery disease excluded
Coronary Heart Disease	SNOMED	699245006	Admit ischemic heart disease emergency (procedure)
Coronary Heart Disease	SNOMED	712866001	Resting ischaemia
Cystic Fibrosis	ICD10	E84	Cystic fibrosis
Cystic Fibrosis	ICD10	P75	Meconium ileus in cystic fibrosis
Cystic Fibrosis	SNOMED	1708960000000000	Cystic fibrosis monitoring
Cystic Fibrosis	SNOMED	1708961000006108	Cystic fibrosis monitoring
Cystic Fibrosis	SNOMED	1708970000000000	Cystic fibrosis annual review
Cystic Fibrosis	SNOMED	1708971000006101	Cystic fibrosis annual review
Cystic Fibrosis	SNOMED	1708981000006103	Annual cystic fibrosis blood test
Cystic Fibrosis	SNOMED	1709161000006100	Arthropathy in Cystic Fibrosis
Cystic Fibrosis	SNOMED	1709340000000000	Liver disease due to cystic fibrosis
Cystic Fibrosis	SNOMED	1709341000006101	Liver disease due to cystic fibrosis
Cystic Fibrosis	SNOMED	1763641000006109	Cirrhosis of liver due to cystic fibrosis
Cystic Fibrosis	SNOMED	1763651000006106	Pancreatic sufficient cystic fibrosis
Cystic Fibrosis	SNOMED	1834241000006100	Age at diagnosis of cystic fibrosis - months
Cystic Fibrosis	SNOMED	1834251000006103	Age at diagnosis of cystic fibrosis - years
Cystic Fibrosis	SNOMED	1834261000006101	Cystic fibrosis complications at birth
Cystic Fibrosis	SNOMED	1854661000006104	Date of cystic fibrosis diagnosis
Cystic Fibrosis	SNOMED	1854771000006101	Cystic fibrosis manifested by failure to thrive
Cystic Fibrosis	SNOMED	1854781000006103	Cystic fibrosis manifested by rectal prolapse
Cystic Fibrosis	SNOMED	1854791000006100	Cystic fibrosis with liver manifestations
Cystic Fibrosis	SNOMED	1854801000006104	Cystic fibrosis manifested by pancreatitis
Cystic Fibrosis	SNOMED	1854811000006101	Cystic fibrosis manifested by acute/persistent respiratory symp.
Cystic Fibrosis	SNOMED	1854831000006107	Cystic fibrosis manifested by bronchiectasis
Cystic Fibrosis	SNOMED	1854841000006102	Cystic fibrosis manifested by electrolyte imbalance
Cystic Fibrosis	SNOMED	1854851000006100	Cystic fibrosis manifested by male infertility
Cystic Fibrosis	SNOMED	1854861000006103	Cystic fibrosis manifested by malnutrition
Cystic Fibrosis	SNOMED	1854871000006105	Cystic fibrosis manifested by meconium ileus
Cystic Fibrosis	SNOMED	1854881000006108	Cystic fibrosis manifested by intestinal obstruction
Cystic Fibrosis	SNOMED	1854901000006105	Cystic fibrosis manifested by malabsorption
Cystic Fibrosis	SNOMED	1854911000006108	Cystic fibrosis manifested by nasal polyp/sinus disease
Cystic Fibrosis	SNOMED	190905008	Cystic fibrosis
Cystic Fibrosis	SNOMED	190909002	Cystic fibrosis with intestinal manifestations
Cystic Fibrosis	SNOMED	206471004	Perinatal jaundice due to mucoviscidosis
Cystic Fibrosis	SNOMED	426705001	Diabetes mellitus co-occurrent and due to cystic fibrosis
Cystic Fibrosis	SNOMED	515611000000000	Cystic fibrosis monitoring
Cystic Fibrosis	SNOMED	515611000000104	Cystic fibrosis monitoring
Cystic Fibrosis	SNOMED	515631000000000	Cystic fibrosis annual review
Cystic Fibrosis	SNOMED	515631000000107	Cystic fibrosis annual review
Cystic Fibrosis	SNOMED	526071000000104	Arthropathy in cystic fibrosis
Cystic Fibrosis	SNOMED	526091000000100	Cystic fibrosis with distal intestinal obstruction syndrome
Cystic Fibrosis	SNOMED	81423003	Cystic fibrosis without meconium ileus
Cystic Fibrosis	SNOMED	859041000000103	Exacerbation of cystic fibrosis
Cystic Fibrosis	SNOMED	86092005	Meconium ileus in cystic fibrosis
Cystic Fibrosis	SNOMED	86555001	Cystic fibrosis with pulmonary manifestations
Dementia	ICD10	F00	Dementia in Alzheimer's disease
Dementia	ICD10	F01	Vascular dementia
Dementia	ICD10	F03	Unspecified dementia
Dementia	ICD10	F05	Delirium superimposed on dementia
Dementia	ICD10	G30	Alzheimer's disease
Dementia	SNOMED	101421000119107	Dementia due to Parkinson's disease (disorder)
Dementia	SNOMED	10349009	Multi-infarct dementia with delirium (disorder)
Dementia	SNOMED	1089521000000106	Predominantly cortical dementia (disorder)
Dementia	SNOMED	1095101000000106	Offer of dementia advance care planning review declined (situation)

Dementia	SNOMED	1095111000000108	Review of dementia advance care plan (procedure)
Dementia	SNOMED	1095121000000102	Dementia advance care plan agreed (finding)
Dementia	SNOMED	1110901000000109	Quality and Outcomes Framework dementia quality indicator-related care invitation (procedure)
Dementia	SNOMED	12348006	Presenile dementia
Dementia	SNOMED	13092008	Pick's disease
Dementia	SNOMED	135811000119107	Lewy body dementia with behavioral disturbance (disorder)
Dementia	SNOMED	142001000119106	Depressed mood in Alzheimer's disease (disorder)
Dementia	SNOMED	15662003	Senile dementia
Dementia	SNOMED	16276361000119109	Vascular dementia without behavioral disturbance (disorder)
Dementia	SNOMED	191449005	Uncomplicated senile dementia
Dementia	SNOMED	191451009	Uncomplicated presenile dementia
Dementia	SNOMED	191452002	Presenile dementia with delirium
Dementia	SNOMED	191454001	Presenile dementia with paranoia
Dementia	SNOMED	191455000	Presenile dementia with depression
Dementia	SNOMED	191457008	Senile dementia with depressive or paranoid features
Dementia	SNOMED	191458003	Senile dementia with paranoia
Dementia	SNOMED	191459006	Senile dementia with depression
Dementia	SNOMED	191461002	Senile dementia with delirium
Dementia	SNOMED	191463004	Uncomplicated arteriosclerotic dementia
Dementia	SNOMED	191464005	Arteriosclerotic dementia with delirium
Dementia	SNOMED	191465006	Arteriosclerotic dementia with paranoia
Dementia	SNOMED	191466007	Arteriosclerotic dementia with depression
Dementia	SNOMED	191493005	Drug-induced dementia (disorder)
Dementia	SNOMED	191519005	Dementia in conditions EC
Dementia	SNOMED	20484008	Prion disease
Dementia	SNOMED	21921000119103	Dementia due to Pick's disease
Dementia	SNOMED	22381000119105	Primary degenerative dementia
Dementia	SNOMED	230265002	Familial Alzheimer's disease of early onset
Dementia	SNOMED	230266001	Non-familial Alzheimer's disease of early onset
Dementia	SNOMED	230267005	Familial Alzheimer's disease of late onset
Dementia	SNOMED	230268000	Non-familial Alzheimer's disease of late onset
Dementia	SNOMED	230269008	Focal Alzheimer's disease
Dementia	SNOMED	230270009	Frontotemporal dementia (disorder)
Dementia	SNOMED	230271008	Pick's disease with Pick bodies (disorder)
Dementia	SNOMED	230273006	Frontotemporal degeneration (disorder)
Dementia	SNOMED	230274000	Frontal lobe degeneration with motor neurone disease (disorder)
Dementia	SNOMED	230280008	Progressive aphasia in Alzheimer's disease
Dementia	SNOMED	230282000	Post-traumatic dementia (disorder)
Dementia	SNOMED	230283005	Punch drunk syndrome (disorder)
Dementia	SNOMED	230284004	Spongiform encephalopathy
Dementia	SNOMED	230285003	Vascular dementia of acute onset
Dementia	SNOMED	230286002	Subcortical vascular dementia
Dementia	SNOMED	230287006	Mixed cortical and subcortical vascular dementia
Dementia	SNOMED	230288001	Semantic dementia (disorder)
Dementia	SNOMED	230289009	Patchy dementia (disorder)
Dementia	SNOMED	26929004	Alzheimer's disease
Dementia	SNOMED	278855005	Frontal lobe degeneration (disorder)
Dementia	SNOMED	278857002	Dementia of frontal lobe type (disorder)
Dementia	SNOMED	279982005	Cerebral degeneration presenting primarily with dementia
Dementia	SNOMED	281004	Dementia associated with alcoholism
Dementia	SNOMED	288631000119104	Vascular dementia with behavioral disturbance (disorder)
Dementia	SNOMED	304603007	New variant of Creutzfeldt-Jakob disease
Dementia	SNOMED	312991009	Senile dementia of the Lewy body type
Dementia	SNOMED	416780008	Primary degenerative dementia of the Alzheimer type, presenile onset (disorder)
Dementia	SNOMED	416975007	Primary degenerative dementia of the Alzheimer type, senile onset (disorder)
Dementia	SNOMED	419261000000107	[X]Dementia in Alzheimer's dis, atypical or mixed type (disorder)
Dementia	SNOMED	429458009	Dementia due to Creutzfeldt Jakob disease (disorder)
Dementia	SNOMED	429998004	Vascular dementia (disorder)
Dementia	SNOMED	442344002	Dementia due to Huntington chorea
Dementia	SNOMED	45864009	Senile degeneration of brain (disorder)
Dementia	SNOMED	52448006	Dementia
Dementia	SNOMED	56267009	Multi-infarct dementia
Dementia	SNOMED	62239001	Parkinson-dementia complex of Guam (disorder)
Dementia	SNOMED	66108005	Primary degenerative dementia of the Alzheimer type, senile onset, uncomplicated
Dementia	SNOMED	67155006	Gerstmann-Straussler-Scheinker syndrome
Dementia	SNOMED	698626001	Dementia associated with multiple sclerosis (disorder)
Dementia	SNOMED	711441000000100	Dementia monitoring invitation (procedure)
Dementia	SNOMED	713060000	Sporadic Creutzfeldt-Jakob disease (disorder)
Dementia	SNOMED	715821000000107	Dementia monitoring first letter (procedure)
Dementia	SNOMED	716221000000104	Dementia monitoring verbal invitation (procedure)
Dementia	SNOMED	716671000000102	Dementia monitoring third letter (procedure)
Dementia	SNOMED	716991000000108	Dementia monitoring telephone invitation (procedure)

Dementia	SNOMED	717471000000101	Dementia monitoring second letter (procedure)
Dementia	SNOMED	723123001	Ischemic vascular dementia (disorder)
Dementia	SNOMED	723390000	Rapidly progressive dementia (disorder)
Dementia	SNOMED	792004	Jakob-Creutzfeldt disease
Dementia	SNOMED	79341000119107	Mixed dementia (disorder)
Dementia	SNOMED	80098002	Diffuse Lewy body disease
Dementia	SNOMED	83157008	Fatal familial insomnia
Dementia	SNOMED	9345005	Dialysis dementia (disorder)
Dementia	SNOMED	938551000000108	Dementia medication review (procedure)
Dementia	SNOMED	956841000000106	Dementia care plan agreed (finding)
Dementia	SNOMED	956861000000107	Dementia care plan reviewed (situation)
Dementia	SNOMED	956881000000103	Dementia care plan declined (situation)
Dementia	SNOMED	956901000000100	Dementia care plan review declined (situation)
Dementia	SNOMED	959461000000102	Dementia advance care plan review declined (situation)
Diabetes	ICD10	E10	Insulin-dependent diabetes mellitus
Diabetes	ICD10	E11	Non-insulin-dependent diabetes mellitus
Diabetes	ICD10	E12	Malnutrition-related diabetes mellitus
Diabetes	ICD10	E13	Other specified diabetes mellitus
Diabetes	ICD10	E14	Unspecified diabetes mellitus
Diabetes	ICD10	H280	Diabetic cataract
Diabetes	ICD10	H360	Diabetic retinopathy
Diabetes	ICD10	M142	Diabetic arthropathy
Diabetes	ICD10	N083	Glomerular disorders in diabetes mellitus
Diabetes	ICD10	O240	Diabetes mellitus in pregnancy: Pre-existing diabetes mellitus, insulin-dependent
Diabetes	ICD10	O241	Diabetes mellitus in pregnancy: Pre-existing diabetes mellitus, non-insulin-dependent
Diabetes	ICD10	O242	Diabetes mellitus in pregnancy: Pre-existing malnutrition-related diabetes mellitus
Diabetes	ICD10	O243	Diabetes mellitus in pregnancy: Pre-existing diabetes mellitus, unspecified
Diabetes	SNOMED	10660471000119109	Ulcer of left foot co-occurrent and due to diabetes mellitus type 2 (disorder)
Diabetes	SNOMED	1066911000000100	Diabetes monitoring short message service text message first invitation (procedure)
Diabetes	SNOMED	1066921000000106	Diabetes monitoring short message service text message second invitation (procedure)
Diabetes	SNOMED	1066931000000108	Diabetes monitoring short message service text message third invitation (procedure)
Diabetes	SNOMED	1083111000000108	Diabetes monitoring invitation email (procedure)
Diabetes	SNOMED	1110921000000100	Quality and Outcomes Framework diabetes mellitus quality indicator-related care invitation (procedure)
Diabetes	SNOMED	111552007	Diabetes mellitus without complication
Diabetes	SNOMED	111556005	Diabetic ketoacidosis without coma
Diabetes	SNOMED	11530004	Brittle diabetes
Diabetes	SNOMED	127013003	Diabetic renal disease
Diabetes	SNOMED	134395001	Diabetic retinopathy screening
Diabetes	SNOMED	140381000119104	Neuropathic toe ulcer due to type 2 diabetes mellitus (disorder)
Diabetes	SNOMED	140521000119107	Ischemic foot ulcer due to type 2 diabetes mellitus (disorder)
Diabetes	SNOMED	157141000119108	Proteinuria due to type 2 diabetes mellitus
Diabetes	SNOMED	170745003	Diabetic on diet only
Diabetes	SNOMED	170747006	Diabetic on insulin
Diabetes	SNOMED	170763003	Diabetic - good control
Diabetes	SNOMED	170766006	Loss of hypoglycemic warning
Diabetes	SNOMED	185756006	Diabetes monitoring first letter (procedure)
Diabetes	SNOMED	185757002	Diabetes monitoring second letter (procedure)
Diabetes	SNOMED	185758007	Diabetes monitoring third letter (procedure)
Diabetes	SNOMED	185759004	Diabetes monitoring verbal invite (procedure)
Diabetes	SNOMED	185760009	Diabetes monitoring telephone invite (procedure)
Diabetes	SNOMED	190331003	Diabetes mellitus, adult onset, with hyperosmolar coma
Diabetes	SNOMED	190388001	Type II diabetes mellitus with multiple complications
Diabetes	SNOMED	190389009	Type 2 diabetes mellitus with ulcer
Diabetes	SNOMED	193141005	Diabetic mononeuritis multiplex
Diabetes	SNOMED	193349004	Preproliferative diabetic retinopathy
Diabetes	SNOMED	193350004	Advanced diabetic maculopathy
Diabetes	SNOMED	193489006	Diabetic iritis
Diabetes	SNOMED	197605007	Nephrotic syndrome in diabetes mellitus
Diabetes	SNOMED	198121000000103	Hypoglycaemic warning impaired (disorder)
Diabetes	SNOMED	198131000000101	Hypoglycaemic warning good (disorder)
Diabetes	SNOMED	199230006	Pre-existing diabetes mellitus, non-insulin-dependent
Diabetes	SNOMED	200687002	Cellulitis in diabetic foot
Diabetes	SNOMED	201251005	Neuropathic diabetic ulcer - foot
Diabetes	SNOMED	201723002	Diabetic hand syndrome
Diabetes	SNOMED	201724008	Diabetic Charcot's arthropathy
Diabetes	SNOMED	232020009	Diabetic maculopathy
Diabetes	SNOMED	232021008	Proliferative diabetic retinopathy new vessels on disc
Diabetes	SNOMED	232023006	Diabetic traction retinal detachment
Diabetes	SNOMED	236499007	Microalbuminuric diabetic nephropathy
Diabetes	SNOMED	236500003	Clinical diabetic nephropathy

Diabetes	SNOMED	237599002	Insulin-treated non-insulin-dependent diabetes mellitus
Diabetes	SNOMED	237620003	Abnormal metabolic state in diabetes mellitus
Diabetes	SNOMED	237621004	Diabetic severe hyperglycemia
Diabetes	SNOMED	237622006	Poor glycemic control (disorder)
Diabetes	SNOMED	237627000	Pregnancy and type 2 diabetes mellitus (disorder)
Diabetes	SNOMED	237632004	Hypoglycemic event in diabetes (disorder)
Diabetes	SNOMED	237633009	Hypoglycemic state in diabetes
Diabetes	SNOMED	238982009	Diabetic dermopathy
Diabetes	SNOMED	238983004	Diabetic thick skin syndrome
Diabetes	SNOMED	24471000000103	Type 2 diabetic on insulin (finding)
Diabetes	SNOMED	24481000000101	Type 2 diabetic on diet only (finding)
Diabetes	SNOMED	25093002	Diabetic oculopathy
Diabetes	SNOMED	25412000	Diabetic retinal microaneurysm
Diabetes	SNOMED	26298008	Diabetic coma with ketoacidosis
Diabetes	SNOMED	267604001	Myasthenic syndrome due to diabetic amyotrophy
Diabetes	SNOMED	268519009	Diabetic - poor control
Diabetes	SNOMED	279321000000104	Diabetes type 2 review (regime/therapy)
Diabetes	SNOMED	280137006	Diabetic foot
Diabetes	SNOMED	308105005	O/E - Right diabetic foot at risk
Diabetes	SNOMED	308106006	O/E - Left diabetic foot at risk
Diabetes	SNOMED	309426007	Diabetic glomerulopathy
Diabetes	SNOMED	310425007	Diabetes monitoring invitation (procedure)
Diabetes	SNOMED	310505005	Diabetic hyperosmolar non-ketotic state
Diabetes	SNOMED	311782002	Advanced diabetic retinal disease
Diabetes	SNOMED	312903003	Mild non proliferative diabetic retinopathy
Diabetes	SNOMED	312904009	Moderate non proliferative diabetic retinopathy
Diabetes	SNOMED	312905005	Severe non proliferative diabetic retinopathy
Diabetes	SNOMED	312906006	Proliferative diabetic retinopathy - non high risk
Diabetes	SNOMED	312907002	Proliferative diabetic retinopathy - high risk
Diabetes	SNOMED	312908007	Proliferative diabetic retinopathy - quiescent
Diabetes	SNOMED	312910009	Diabetic vitreous hemorrhage
Diabetes	SNOMED	312912001	Diabetic macular edema
Diabetes	SNOMED	313436004	Non-insulin-dependent diabetes mellitus without complication
Diabetes	SNOMED	314011005	Focal diabetic maculopathy
Diabetes	SNOMED	314014002	Ischaemic diabetic maculopathy
Diabetes	SNOMED	314015001	Mixed diabetic maculopathy
Diabetes	SNOMED	314537004	Diabetic optic papillopathy
Diabetes	SNOMED	314903002	Non-insulin dependent diabetes mellitus with arthropathy
Diabetes	SNOMED	314904008	Type II diabetes mellitus with neuropathic arthropathy
Diabetes	SNOMED	371087003	Diabetic foot ulcer (disorder)
Diabetes	SNOMED	385041000000108	Diabetes mellitus with multiple complications (disorder)
Diabetes	SNOMED	385051000000106	Pre-existing diabetes mellitus (disorder)
Diabetes	SNOMED	390834004	Non proliferative diabetic retinopathy (disorder)
Diabetes	SNOMED	390850007	O/E - no right diabetic retinopathy (context-dependent category)
Diabetes	SNOMED	390853009	O/E - no left diabetic retinopathy (context-dependent category)
Diabetes	SNOMED	390854003	O/E - diabetic maculopathy present both eyes (context-dependent category)
Diabetes	SNOMED	390855002	O/E - diabetic maculopathy absent both eyes (context-dependent category)
Diabetes	SNOMED	395204000	Hyperosmolar non-ketotic state in type 2 diabetes mellitus (disorder)
Diabetes	SNOMED	399864000	Diabetic macular edema not clinically significant (disorder)
Diabetes	SNOMED	399865004	Very severe proliferative diabetic retinopathy (disorder)
Diabetes	SNOMED	399866003	Diabetic retinal venous beading (disorder)
Diabetes	SNOMED	399870006	Non-high-risk proliferative diabetic retinopathy with no macular edema (disorder)
Diabetes	SNOMED	401191002	Diabetic foot examination (regime/therapy)
Diabetes	SNOMED	408397002	Diabetic foot examination not indicated (context-dependent category)
Diabetes	SNOMED	408409007	O/E - right eye background diabetic retinopathy (context-dependent category)
Diabetes	SNOMED	408410002	O/E - left eye background diabetic retinopathy (context-dependent category)
Diabetes	SNOMED	408411003	O/E - right eye preproliferative diabetic retinopathy (context-dependent category)
Diabetes	SNOMED	408412005	O/E - left eye preproliferative diabetic retinopathy (context-dependent category)
Diabetes	SNOMED	408413000	O/E - right eye proliferative diabetic retinopathy (context-dependent category)
Diabetes	SNOMED	408414006	O/E - left eye proliferative diabetic retinopathy (context-dependent category)
Diabetes	SNOMED	412752009	Diabetic foot examination declined (context-dependent category)
Diabetes	SNOMED	414894003	O/E - left eye stable treated proliferative diabetic retinopathy (context-dependent category)
Diabetes	SNOMED	414910007	O/E - right eye stable treated proliferative diabetic retinopathy (context-dependent category)
Diabetes	SNOMED	417677008	O/E - sight threatening diabetic retinopathy (context-dependent category)
Diabetes	SNOMED	419100001	Infection of foot associated with diabetes (disorder)
Diabetes	SNOMED	420279001	Renal disorder associated with type II diabetes mellitus (disorder)
Diabetes	SNOMED	420422005	Ketoacidosis in diabetes mellitus (disorder)
Diabetes	SNOMED	420662003	Coma associated with diabetes mellitus (disorder)

Diabetes	SNOMED	420715001	Persistent microalbuminuria associated with type II diabetes mellitus (disorder)
Diabetes	SNOMED	420756003	Diabetic cataract associated with type II diabetes mellitus (disorder)
Diabetes	SNOMED	421326000	Neurologic disorder associated with type II diabetes mellitus (disorder)
Diabetes	SNOMED	421750000	Ketoacidosis in type II diabetes mellitus (disorder)
Diabetes	SNOMED	421779007	Exudative maculopathy associated with type II diabetes mellitus (disorder)
Diabetes	SNOMED	421847006	Ketoacidotic coma in type II diabetes mellitus (disorder)
Diabetes	SNOMED	421986006	Persistent proteinuria associated with type II diabetes mellitus (disorder)
Diabetes	SNOMED	422014003	Disorder associated with type II diabetes melliltus (disorder)
Diabetes	SNOMED	422034002	Diabetic retinopathy associated with type II diabetes mellitus (disorder)
Diabetes	SNOMED	422088007	Neurologic disorder associated with diabetes mellitus (disorder)
Diabetes	SNOMED	422099009	Diabetic oculopathy associated with type II diabetes mellitus (disorder)
Diabetes	SNOMED	422126006	Hyperosmolar coma associated with diabetes mellitus (disorder)
Diabetes	SNOMED	422183001	Skin ulcer associated with diabetes mellitus (disorder)
Diabetes	SNOMED	429729007	Diabetic education completed (situation)
Diabetes	SNOMED	43959009	Diabetic cataract
Diabetes	SNOMED	44054006	Diabetes mellitus type II
Diabetes	SNOMED	441656006	Hyperglycaemic crisis in diabetes mellitus
Diabetes	SNOMED	443694000	Type II diabetes mellitus uncontrolled (finding)
Diabetes	SNOMED	445353002	Brittle type II diabetes mellitus (finding)
Diabetes	SNOMED	472969004	History of diabetes mellitus type 2 (situation)
Diabetes	SNOMED	4855003	Diabetic retinopathy
Diabetes	SNOMED	48951005	Bullosis diabeticorum
Diabetes	SNOMED	532411000000102	Diabetes mellitus, adult onset, with no mention of complication (disorder)
Diabetes	SNOMED	59276001	Proliferative diabetic retinopathy
Diabetes	SNOMED	609561005	Maturity-onset diabetes of the young (disorder)
Diabetes	SNOMED	62260007	Pretibial pigmental patches in diabetes
Diabetes	SNOMED	658011000000104	Diabetes mellitus with other specified manifestation (disorder)
Diabetes	SNOMED	703136005	Diabetes mellitus in remission (disorder)
Diabetes	SNOMED	703138006	Type II diabetes mellitus in remission (disorder)
Diabetes	SNOMED	705072004	Diabetes monitoring invitation by short message service text messaging (procedure)
Diabetes	SNOMED	707221002	Diabetic glomerulosclerosis
Diabetes	SNOMED	713703005	Gastroparesis due to type 2 diabetes mellitus (disorder)
Diabetes	SNOMED	713704004	Gastroparesis due to diabetes mellitus (disorder)
Diabetes	SNOMED	719216001	Hypoglycemic coma co-occurrent and due to diabetes mellitus type II (disorder)
Diabetes	SNOMED	722161008	Diabetic retinal eye exam (procedure)
Diabetes	SNOMED	724136006	Diabetic mastopathy
Diabetes	SNOMED	724997001	Lumbosacral plexopathy co-occurrent and due to diabetes mellitus (disorder)
Diabetes	SNOMED	731000119105	Chronic kidney disease stage 3 associated with type 2 diabetes mellitus
Diabetes	SNOMED	73211009	Diabetes mellitus
Diabetes	SNOMED	735200002	Absence of lower limb due to diabetes mellitus (disorder)
Diabetes	SNOMED	74627003	Diabetic complication
Diabetes	SNOMED	754121000000107	Type II diabetic dietary review (regime/therapy)
Diabetes	SNOMED	762489000	Acute complication with diabetes mellitus
Diabetes	SNOMED	768792007	Cataract of right eye co-occurrent and due to diabetes mellitus
Diabetes	SNOMED	768794008	Bilateral diabetic cataracts
Diabetes	SNOMED	769181007	Preproliferative retinopathy of right eye co-occurrent and due to diabetes mellitus (disorder)
Diabetes	SNOMED	769182000	Preproliferative diabetic retinopathy of left eye
Diabetes	SNOMED	769183005	Mild nonproliferative diabetic retinopathy of right eye
Diabetes	SNOMED	769184004	Mild nonproliferative retinopathy of left eye
Diabetes	SNOMED	769185003	Moderate non-proliferative diabetic retinopathy of right eye
Diabetes	SNOMED	769186002	Moderate nonproliferative diabetic retinopathy of left eye
Diabetes	SNOMED	769217008	Diabetic macular edema of right eye
Diabetes	SNOMED	769218003	Macular oedema of left eye co-occurrent and due to diabetes mellitus
Diabetes	SNOMED	769221001	Clinically significant macular edema of right eye co-occurrent and due to diabetes mellitus (disorder)
Diabetes	SNOMED	769222008	Clinically significant macular oedema of left eye co-occurrent and due to diabetes mellitus
Diabetes	SNOMED	769244003	Diabetic maculopathy of right eye
Diabetes	SNOMED	769245002	Disorder of left macula co-occurrent and due to diabetes mellitus (disorder)
Diabetes	SNOMED	770097006	Clinically significant macular edema co-occurrent and due to diabetes mellitus (disorder)
Diabetes	SNOMED	775841000000109	Diabetic retinopathy detected by national screening programme (disorder)
Diabetes	SNOMED	791000119109	Angina associated with type II diabetes mellitus
Diabetes	SNOMED	894741000000107	Hypoglycaemic warning absent (disorder)
Drug Or Alcohol Misuse	ICD10	E244	Alcohol-induced pseudo-Cushing syndrome
Drug Or Alcohol Misuse	ICD10	F04	Organic amnesic syndrome, not induced by alcohol and other psychoactive
Drug Or Alcohol Misuse	ICD10	F05	Delirium, not induced by alcohol and other psychoactive substances
Drug Or Alcohol Misuse	ICD10	F10	Mental and behavioural disorders due to use of alcohol
Drug Or Alcohol Misuse	ICD10	F19	Mental and behavioural disorders due to multiple drug use and use of other
Drug Or Alcohol Misuse	ICD10	F55	Abuse of non-dependence-producing substances
Drug Or Alcohol Misuse	ICD10	G312	Degeneration of nervous system due to alcohol

Drug Or Alcohol Misuse	ICD10	G721	Alcoholic myopathy
Drug Or Alcohol Misuse	ICD10	I426	Alcoholic cardiomyopathy
Drug Or Alcohol Misuse	ICD10	K292	Alcoholic gastritis
Drug Or Alcohol Misuse	ICD10	K700	Alcoholic liver disease
Drug Or Alcohol Misuse	ICD10	K704	Alcoholic liver disease
Drug Or Alcohol Misuse	ICD10	K705	Alcoholic liver disease
Drug Or Alcohol Misuse	ICD10	K706	Alcoholic liver disease
Drug Or Alcohol Misuse	ICD10	K707	Alcoholic liver disease
Drug Or Alcohol Misuse	ICD10	K708	Alcoholic liver disease
Drug Or Alcohol Misuse	ICD10	K709	Alcoholic liver disease
Drug Or Alcohol Misuse	ICD10	K852	Alcohol-induced acute pancreatitis
Drug Or Alcohol Misuse	ICD10	K860	Alcohol-induced chronic pancreatitis
Drug Or Alcohol Misuse	ICD10	T436	Poisoning: Psychostimulants with abuse potential
Drug Or Alcohol Misuse	ICD10	Y497	Psychostimulants with abuse potential
Drug Or Alcohol Misuse	ICD10	Z722	Drug use
Drug Or Alcohol Misuse	ICD10	Z864	Personal history of psychoactive substance abuse
Drug Or Alcohol Misuse	SNOMED	1012911000006107	Adverse reaction to Drugs used in Substance Dependence
Drug Or Alcohol Misuse	SNOMED	1047881000000106	Opioid analgesic dependence
Drug Or Alcohol Misuse	SNOMED	105507009	[X]Other problems related to care provider dependency
Drug Or Alcohol Misuse	SNOMED	1084211000000102	Signposting to drug misuse service
Drug Or Alcohol Misuse	SNOMED	15167005	AA - Alcohol abuse
Drug Or Alcohol Misuse	SNOMED	1563941000006104	Drug Dependency
Drug Or Alcohol Misuse	SNOMED	160592001	Excessive alcohol use
Drug Or Alcohol Misuse	SNOMED	161466001	H/O: alcoholism
Drug Or Alcohol Misuse	SNOMED	161467005	H/O: drug dependency
Drug Or Alcohol Misuse	SNOMED	1662551000006109	Dental recall - excessive alcohol use
Drug Or Alcohol Misuse	SNOMED	1745961000006107	Extended intervention for excessive alcohol consumption declined
Drug Or Alcohol Misuse	SNOMED	176831000000102	Drug misuse - enhanced service completed
Drug Or Alcohol Misuse	SNOMED	1778191000006109	Group session: Substance misuse
Drug Or Alcohol Misuse	SNOMED	1808491000006109	Alcohol-related legal or disciplinary problem
Drug Or Alcohol Misuse	SNOMED	1823491000006107	Substance misuse management stopped - patient dropped out/left
Drug Or Alcohol Misuse	SNOMED	182969009	Detoxification dependence drug
Drug Or Alcohol Misuse	SNOMED	183388004	Aversion therapy - alcoholism
Drug Or Alcohol Misuse	SNOMED	1854730000000000	Seen in drug misuse clinic
Drug Or Alcohol Misuse	SNOMED	1854731000006104	Seen in drug misuse clinic
Drug Or Alcohol Misuse	SNOMED	1872991000006107	Safeguarding vulnerability - disclosure of abuse
Drug Or Alcohol Misuse	SNOMED	1876861000006106	Substance misuse annual review
Drug Or Alcohol Misuse	SNOMED	1876871000006104	Substance misuse 6 month review
Drug Or Alcohol Misuse	SNOMED	1877110000000000	In-house substance misuse treatment
Drug Or Alcohol Misuse	SNOMED	1877111000006101	In-house substance misuse treatment
Drug Or Alcohol Misuse	SNOMED	1877121000006109	Substance misuse treatment by other healthcare provider
Drug Or Alcohol Misuse	SNOMED	1880411000006100	Intravenous drug use in the last 12 months
Drug Or Alcohol Misuse	SNOMED	1894170000000000	Alcohol dependence resolved
Drug Or Alcohol Misuse	SNOMED	1894171000006107	Alcohol dependence resolved
Drug Or Alcohol Misuse	SNOMED	1897741000006108	Drug misuse - monthly
Drug Or Alcohol Misuse	SNOMED	1897751000006105	Drug misuse - weekly
Drug Or Alcohol Misuse	SNOMED	1897761000006107	Drug misuse - daily
Drug Or Alcohol Misuse	SNOMED	191471000	Korsakov's alcoholic psychosis with peripheral neuritis
Drug Or Alcohol Misuse	SNOMED	191475009	Chronic alcoholic brain syndrome
Drug Or Alcohol Misuse	SNOMED	191476005	Alcohol withdrawal hallucinosis
Drug Or Alcohol Misuse	SNOMED	191477001	Pathological alcohol intoxication
Drug Or Alcohol Misuse	SNOMED	191478006	Alcoholic paranoia
Drug Or Alcohol Misuse	SNOMED	191480000	[X]Mental and behav dis due to use alcohol: withdrawal state
Drug Or Alcohol Misuse	SNOMED	191483003	[X]Ment/behav dis mlti drug use/oth psyc sbs: psychotc dis
Drug Or Alcohol Misuse	SNOMED	191802004	Alcohol dependence with acute alcoholic intoxication
Drug Or Alcohol Misuse	SNOMED	191804003	Continuous acute alcoholic intoxication in alcoholism
Drug Or Alcohol Misuse	SNOMED	191805002	Episodic acute alcoholic intoxication in alcoholism
Drug Or Alcohol Misuse	SNOMED	191806001	Acute alcoholic intoxication in remission, in alcoholism
Drug Or Alcohol Misuse	SNOMED	191811004	Continuous chronic alcoholism
Drug Or Alcohol Misuse	SNOMED	191812006	Episodic chronic alcoholism
Drug Or Alcohol Misuse	SNOMED	191813001	Chronic alcoholism in remission
Drug Or Alcohol Misuse	SNOMED	191816009	Drug dependence
Drug Or Alcohol Misuse	SNOMED	191819002	Continuous opioid dependence
Drug Or Alcohol Misuse	SNOMED	191820008	Episodic opioid dependence
Drug Or Alcohol Misuse	SNOMED	191821007	Opioid dependence in remission
Drug Or Alcohol Misuse	SNOMED	191825003	Hypnotic or anxiolytic dependence, continuous
Drug Or Alcohol Misuse	SNOMED	191826002	Hypnotic or anxiolytic dependence, episodic
Drug Or Alcohol Misuse	SNOMED	191827006	Hypnotic or anxiolytic dependence in remission
Drug Or Alcohol Misuse	SNOMED	191829009	Cocaine type drug dependence
Drug Or Alcohol Misuse	SNOMED	191831000	Cocaine dependence, continuous
Drug Or Alcohol Misuse	SNOMED	191832007	Cocaine dependence, episodic
Drug Or Alcohol Misuse	SNOMED	191833002	Cocaine dependence in remission
Drug Or Alcohol Misuse	SNOMED	191837001	Cannabis dependence, continuous
Drug Or Alcohol Misuse	SNOMED	191838006	Cannabis dependence, episodic
Drug Or Alcohol Misuse	SNOMED	191839003	Cannabis dependence in remission

Drug Or Alcohol Misuse	SNOMED	191843004	Amphetamine or psychostimulant dependence, continuous
Drug Or Alcohol Misuse	SNOMED	191844005	Amphetamine or psychostimulant dependence, episodic
Drug Or Alcohol Misuse	SNOMED	191845006	Amfetamine or psychostimulant dependence in remission
Drug Or Alcohol Misuse	SNOMED	191849000	Hallucinogen dependence, continuous
Drug Or Alcohol Misuse	SNOMED	191850000	Hallucinogen dependence, episodic
Drug Or Alcohol Misuse	SNOMED	191851001	Hallucinogen dependence in remission
Drug Or Alcohol Misuse	SNOMED	191853003	Glue sniffing dependence
Drug Or Alcohol Misuse	SNOMED	191855005	Glue sniffing dependence, continuous
Drug Or Alcohol Misuse	SNOMED	191856006	Glue sniffing dependence, episodic
Drug Or Alcohol Misuse	SNOMED	191857002	Glue sniffing dependence in remission
Drug Or Alcohol Misuse	SNOMED	191865004	Combined opioid with other drug dependence
Drug Or Alcohol Misuse	SNOMED	191867007	Combined opioid with non-opioid drug dependence, continuous
Drug Or Alcohol Misuse	SNOMED	191868002	Combined opioid with other drug dependence, episodic
Drug Or Alcohol Misuse	SNOMED	191869005	Combined opioid with other drug dependence in remission
Drug Or Alcohol Misuse	SNOMED	191871005	Combined drug dependence, excluding opioids
Drug Or Alcohol Misuse	SNOMED	191873008	Combined drug dependence, excluding opioid, continuous
Drug Or Alcohol Misuse	SNOMED	191874002	Combined drug dependence, excluding opioid, episodic
Drug Or Alcohol Misuse	SNOMED	191875001	Combined drug dependence, excluding opioid, in remission
Drug Or Alcohol Misuse	SNOMED	191877009	Ecstasy type drug dependence
Drug Or Alcohol Misuse	SNOMED	191882002	Nondependent alcohol abuse, continuous
Drug Or Alcohol Misuse	SNOMED	191883007	Nondependent alcohol abuse, episodic
Drug Or Alcohol Misuse	SNOMED	191884001	Nondependent alcohol abuse in remission
Drug Or Alcohol Misuse	SNOMED	191891003	Nondependent cannabis abuse
Drug Or Alcohol Misuse	SNOMED	191893000	Nondependent cannabis abuse, continuous
Drug Or Alcohol Misuse	SNOMED	191894006	Nondependent cannabis abuse, episodic
Drug Or Alcohol Misuse	SNOMED	191895007	Nondependent cannabis abuse in remission
Drug Or Alcohol Misuse	SNOMED	191899001	Nondependent hallucinogen abuse, continuous
Drug Or Alcohol Misuse	SNOMED	191900006	Nondependent hallucinogen abuse, episodic
Drug Or Alcohol Misuse	SNOMED	191901005	Nondependent hallucinogen abuse in remission
Drug Or Alcohol Misuse	SNOMED	191905001	Nondependent hypnotic or anxiolytic abuse, continuous
Drug Or Alcohol Misuse	SNOMED	191906000	Nondependent hypnotic or anxiolytic abuse, episodic
Drug Or Alcohol Misuse	SNOMED	191907009	Nondependent hypnotic or anxiolytic abuse in remission
Drug Or Alcohol Misuse	SNOMED	191909007	Nondependent opioid abuse
Drug Or Alcohol Misuse	SNOMED	191912005	Nondependent opioid abuse, continuous
Drug Or Alcohol Misuse	SNOMED	191913000	Nondependent opioid abuse, episodic
Drug Or Alcohol Misuse	SNOMED	191914006	Nondependent opioid abuse in remission
Drug Or Alcohol Misuse	SNOMED	191916008	Nondependent cocaine abuse
Drug Or Alcohol Misuse	SNOMED	191918009	Nondependent cocaine abuse, continuous
Drug Or Alcohol Misuse	SNOMED	191919001	Nondependent cocaine abuse, episodic
Drug Or Alcohol Misuse	SNOMED	191920007	Nondependent cocaine abuse in remission
Drug Or Alcohol Misuse	SNOMED	191924003	Nondependent amfetamine or psychostimulant abuse, continuous
Drug Or Alcohol Misuse	SNOMED	191925002	Nondependent amfetamine or psychostimulant abuse, episodic
Drug Or Alcohol Misuse	SNOMED	191928000	Nondependent antidepressant type drug abuse
Drug Or Alcohol Misuse	SNOMED	191930003	Nondependent antidepressant type drug abuse, continuous
Drug Or Alcohol Misuse	SNOMED	191931004	Nondependent antidepressant type drug abuse, episodic
Drug Or Alcohol Misuse	SNOMED	191932006	Nondependent antidepressant type drug abuse in remission
Drug Or Alcohol Misuse	SNOMED	191934007	Nondependent mixed drug abuse, unspecified
Drug Or Alcohol Misuse	SNOMED	191936009	Nondependent mixed drug abuse, continuous
Drug Or Alcohol Misuse	SNOMED	191937000	Nondependent mixed drug abuse, episodic
Drug Or Alcohol Misuse	SNOMED	191938005	Nondependent mixed drug abuse in remission
Drug Or Alcohol Misuse	SNOMED	191939002	Misuse of prescription only drugs
Drug Or Alcohol Misuse	SNOMED	192811002	Alcohol induced encephalopathy
Drug Or Alcohol Misuse	SNOMED	19303008	Alcoholic myopathy
Drug Or Alcohol Misuse	SNOMED	1948781000006103	Substance misuse treatment programme commenced
Drug Or Alcohol Misuse	SNOMED	1971481000006103	Mental and behav dis due to hallucinogens: dependence syn, currently abstinent
Drug Or Alcohol Misuse	SNOMED	1971511000006106	Mental and behav dis due to hallucinogens: dependence syn, currently abstinent,
Drug Or Alcohol Misuse	SNOMED	1971551000006107	Mental and behav dis due to hallucinogens: dependence syn, currently on a
Drug Or Alcohol Misuse	SNOMED	1971581000006104	Mental and behav dis due to hallucinogens: dependence syn, currently abstinent,
Drug Or Alcohol Misuse	SNOMED	1971611000006107	Mental and behav dis due to hallucinogens: dependence syn, currently using the
Drug Or Alcohol Misuse	SNOMED	1971621000006104	Mental and behav dis due to hallucinogens: dependence syn, continuous use
Drug Or Alcohol Misuse	SNOMED	1971641000006106	Mental and behav dis due to hallucinogens: dependence syn, episodic use
Drug Or Alcohol Misuse	SNOMED	1971921000006109	Mental and behav dis due to cannabinoids: dependence synd, currently abstinent
Drug Or Alcohol Misuse	SNOMED	1971931000006107	Mental and behav dis due to cannabinoids: dependence synd, currently abstinent,
Drug Or Alcohol Misuse	SNOMED	1971941000006102	Mental and behav dis due to cannabinoids: dependence synd, currently on a
Drug Or Alcohol Misuse	SNOMED	1971951000006100	Mental and behav dis due to cannabinoids: dependence synd, currently abstinent,
Drug Or Alcohol Misuse	SNOMED	1971961000006103	Mental and behav dis due to cannabinoids: dependence synd, currently using the
Drug Or Alcohol Misuse	SNOMED	1971971000006105	Mental and behav dis due to cannabinoids: dependence synd, continuous use
Drug Or Alcohol Misuse	SNOMED	1971981000006108	Mental and behav dis due to cannabinoids: dependence synd, episodic use
Drug Or Alcohol Misuse	SNOMED	1972151000006106	Mental & behav dis due to use alcohol: acute intoxication, uncomplicated
Drug Or Alcohol Misuse	SNOMED	1972161000006108	Mental & behav dis due to use alcohol: acute intoxication, with trauma or other
Drug Or Alcohol Misuse	SNOMED	1972271000006108	Mental & behav dis due to use alcohol: acute intoxication, with other medical
Drug Or Alcohol Misuse	SNOMED	1972631000006107	Mental and behav dis due to seds/hypntcs: dependence synd, currently abstinent
Drug Or Alcohol Misuse	SNOMED	1972651000006100	Mental and behav dis due to seds/hypntcs: dependence synd, currently abstinent,
Drug Or Alcohol Misuse	SNOMED	1972671000006105	Mental and behav dis due to seds/hypntcs: dependence synd, currently on a
Drug Or Alcohol Misuse	SNOMED	1972691000006106	Mental and behav dis due to seds/hypntcs: dependence synd, currently abstinent,

Drug Or Alcohol Misuse	SNOMED	1972701000006106	Mental and behav dis due to seds/hypntcs: dependence synd, currently using the
Drug Or Alcohol Misuse	SNOMED	1972721000006101	Mental and behav dis due to seds/hypntcs: dependence synd, continuous use
Drug Or Alcohol Misuse	SNOMED	1972741000006108	Mental and behav dis due to seds/hypntcs: dependence synd, episodic use
Drug Or Alcohol Misuse	SNOMED	1972951000006108	Mental and behav dis due to use cocaine: dependence syndr, currently abstinent
Drug Or Alcohol Misuse	SNOMED	1972981000006100	Mental and behav dis due to use cocaine: dependence syndr, currently abstinent,
Drug Or Alcohol Misuse	SNOMED	1973201000006107	Mental and behav dis due to use cocaine: dependence syndr, currently on a
Drug Or Alcohol Misuse	SNOMED	1973211000006105	Mental and behav dis due to use cocaine: dependence syndr, currently abstinent,
Drug Or Alcohol Misuse	SNOMED	1973241000006109	Mental & behav dis due to use alcohol: acute intoxication, with delirium
Drug Or Alcohol Misuse	SNOMED	1973261000006108	Mental and behav dis due to use cocaine: dependence syndr, currently using the
Drug Or Alcohol Misuse	SNOMED	1973291000006100	Mental and behav dis due to use cocaine: dependence syndr, continuous use
Drug Or Alcohol Misuse	SNOMED	1973311000006101	Mental and behav dis due to use cocaine: dependence syndr, episodic use
Drug Or Alcohol Misuse	SNOMED	1973321000006109	Mental & behav dis due to use alcohol: acute intoxication, with perceptual
Drug Or Alcohol Misuse	SNOMED	1973411000006106	Mental & behav dis due to use alcohol: acute intoxication, with coma
Drug Or Alcohol Misuse	SNOMED	1973421000006103	Mental and behav dis mlti drg use/oth psych sbs: dependence syndr, currently
Drug Or Alcohol Misuse	SNOMED	1973441000006105	Mental & behav dis due to use alcohol: acute intoxication, with convulsions
Drug Or Alcohol Misuse	SNOMED	1973451000006107	Mental and behav dis mlti drg use/oth psych sbs: dependence syndr, currently on
Drug Or Alcohol Misuse	SNOMED	1973471000006102	Mental and behav dis mlti drg use/oth psych sbs: dependence syndr, currently
Drug Or Alcohol Misuse	SNOMED	1973481000006104	Mental & behav dis due to use alcohol: acute intoxication, pathological
Drug Or Alcohol Misuse	SNOMED	1973491000006101	Mental and behav dis mlti drg use/oth psych sbs: dependence syndr, currently
Drug Or Alcohol Misuse	SNOMED	1973521000006104	Mental and behav dis mlti drg use/oth psych sbs: dependence syndr, continuous
Drug Or Alcohol Misuse	SNOMED	1973541000006106	Mental and behav dis mlti drg use/oth psych sbs: dependence syndr, episodic use
Drug Or Alcohol Misuse	SNOMED	1973701000006103	Mental and behav dis due to use alcohol: dependence syndr, currently abstinent
Drug Or Alcohol Misuse	SNOMED	1973811000006109	Mental and behav dis due to use alcohol: dependence syndr, currently abstinent,
Drug Or Alcohol Misuse	SNOMED	1973831000006103	Ment/behav dis mlti drug use/oth psyc sbs: psychotc dis, schizophrenia-like
Drug Or Alcohol Misuse	SNOMED	1973861000006107	Ment/behav dis mlti drug use/oth psyc sbs: psychotc dis, predominantly
Drug Or Alcohol Misuse	SNOMED	1973871000006100	Mental and behav dis due to use alcohol: dependence syndr, currently on a
Drug Or Alcohol Misuse	SNOMED	1973891000006104	Ment/behav dis mlti drug use/oth psyc sbs: psychotc dis, predominantly
Drug Or Alcohol Misuse	SNOMED	1973931000006108	Ment/behav dis mlti drug use/oth psyc sbs: psychotc dis, predominantly
Drug Or Alcohol Misuse	SNOMED	1973961000006104	Ment/behav dis mlti drug use/oth psyc sbs: psychotc dis, predominantly
Drug Or Alcohol Misuse	SNOMED	1973991000006107	Ment/behav dis mlti drug use/oth psyc sbs: psychotc dis, predominantly manic
Drug Or Alcohol Misuse	SNOMED	1974011000006100	Ment/behav dis mlti drug use/oth psyc sbs: psychotc dis, mixed
Drug Or Alcohol Misuse	SNOMED	1974021000006108	Mental and behav dis due to use alcohol: dependence syndr, currently abstinent,
Drug Or Alcohol Misuse	SNOMED	1974071000006109	Mental and behav dis due to use alcohol: dependence syndr, currently using the
Drug Or Alcohol Misuse	SNOMED	1974111000006101	Mental and behav dis due to use alcohol: dependence syndr, continuous use
Drug Or Alcohol Misuse	SNOMED	1974141000006102	Mental and behav dis due to use alcohol: dependence syndr, episodic use
Drug Or Alcohol Misuse	SNOMED	1974191000006106	Mental and behav dis due to use alcohol: withdrawal state, uncomplicated
Drug Or Alcohol Misuse	SNOMED	1974231000006101	Mental and behav dis due to use alcohol: withdrawal state, with convulsions
Drug Or Alcohol Misuse	SNOMED	1974301000006101	Mental & behav dis due to use alcohol: withdrawl state with delirium, without
Drug Or Alcohol Misuse	SNOMED	1974381000006105	Mental and behav dis due to other stimulants inc caffeine: dependnce synd,
Drug Or Alcohol Misuse	SNOMED	1974441000006107	Mental and behav dis due to other stimulants inc caffeine: dependnce synd,
Drug Or Alcohol Misuse	SNOMED	1974521000006101	Mental & behav dis due to use alcohol: withdrawl state with delirium, with
Drug Or Alcohol Misuse	SNOMED	1975141000006101	Mental & behav dis due to use alcohol: psychotic disorder, schizophrenia-like
Drug Or Alcohol Misuse	SNOMED	1975181000006107	Mental & behav dis due to use alcohol: psychotic disorder, predominantly
Drug Or Alcohol Misuse	SNOMED	1975291000006101	Mental & behav dis due to use alcohol: psychotic disorder, predominantly
Drug Or Alcohol Misuse	SNOMED	1975311000006102	Mental and behav dis due to vol solvents: dependence synd, currently abstinent
Drug Or Alcohol Misuse	SNOMED	1975331000006108	Mental and behav dis due to vol solvents: dependence synd, currently abstinent,
Drug Or Alcohol Misuse	SNOMED	1975341000006103	Mental & behav dis due to use alcohol: psychotic disorder, predominantly
Drug Or Alcohol Misuse	SNOMED	1975361000006104	Mental and behav dis due to vol solvents: dependence synd, currently on a
Drug Or Alcohol Misuse	SNOMED	1975371000006106	Mental & behav dis due to use alcohol: psychotic disorder, predominantly
Drug Or Alcohol Misuse	SNOMED	1975391000006107	Mental and behav dis due to vol solvents: dependence synd, currently abstinent,
Drug Or Alcohol Misuse	SNOMED	197541000000101	Occasional drug user
Drug Or Alcohol Misuse	SNOMED	1975411000006107	Mental & behav dis due to use alcohol: psychotic disorder, predominantly manic
Drug Or Alcohol Misuse	SNOMED	1975421000006104	Mental and behav dis due to vol solvents: dependence synd, currently using the
Drug Or Alcohol Misuse	SNOMED	1975451000006108	Mental and behav dis due to vol solvents: dependence synd, continuous use
Drug Or Alcohol Misuse	SNOMED	1975461000006105	Mental & behav dis due to use alcohol: psychotic disorder, mixed
Drug Or Alcohol Misuse	SNOMED	1975511000006108	Mental & behav dis due to use alcohol: resid & late-onset psychot dis, flashbacks
Drug Or Alcohol Misuse	SNOMED	1975541000006107	Mental & behav dis due to use alcohol: resid & late-onset psychot dis, personality
Drug Or Alcohol Misuse	SNOMED	1975591000006103	Mental & behav dis due to use alcohol: resid & late-onset psychot dis, dementia
Drug Or Alcohol Misuse	SNOMED	1975661000006107	Mental & behav dis due to use alcohol: resid & late-onset psychot dis, residual
Drug Or Alcohol Misuse	SNOMED	1976071000006103	Mental & behav dis due to use alcohol: resid & late-onset psychot dis, other
Drug Or Alcohol Misuse	SNOMED	1976111000006106	Mental & behav dis due to use alcohol: resid & late-onset psychot dis, late-onset
Drug Or Alcohol Misuse	SNOMED	1976501000006101	Mental and behav dis due to use opioids: dependence syndr, currently abstinent
Drug Or Alcohol Misuse	SNOMED	1976531000006109	Mental and behav dis due to use opioids: dependence syndr, currently abstinent,
Drug Or Alcohol Misuse	SNOMED	1976551000006102	Mental and behav dis due to use opioids: dependence syndr, currently on a
Drug Or Alcohol Misuse	SNOMED	1976581000006105	Mental and behav dis due to use opioids: dependence syndr, currently abstinent,
Drug Or Alcohol Misuse	SNOMED	1976591000006108	Mental and behav dis due to use opioids: dependence syndr, currently using the
Drug Or Alcohol Misuse	SNOMED	1976611000006102	Mental and behav dis due to use opioids: dependence syndr, continuous use
Drug Or Alcohol Misuse	SNOMED	1976621000006105	Mental and behav dis due to use opioids: dependence syndr, episodic use
Drug Or Alcohol Misuse	SNOMED	1976811000006101	Abuse of non-dependence-producing substances, other substances
Drug Or Alcohol Misuse	SNOMED	1976821000006109	Abuse of non-dependence-producing substances, unspecified
Drug Or Alcohol Misuse	SNOMED	1976871000006105	Mental and behav dis mlti drg use/oth psych sbs: dependence syndr, currently
Drug Or Alcohol Misuse	SNOMED	1976981000006101	Mental and behav dis due to vol solvents: dependence synd, episodic use
Drug Or Alcohol Misuse	SNOMED	1977071000006100	Abuse of antidepressants
Drug Or Alcohol Misuse	SNOMED	198421000000108	Hazardous alcohol use
Drug Or Alcohol Misuse	SNOMED	198431000000105	Harmful alcohol use

Drug Or Alcohol Misuse	SNOMED	198861000000103	Substance misuse clinical management plan reviewed
Drug Or Alcohol Misuse	SNOMED	199252002	Drug dependence during pregnancy - baby delivered
Drug Or Alcohol Misuse	SNOMED	199253007	Postpartum drug dependence
Drug Or Alcohol Misuse	SNOMED	199254001	Drug dependence during pregnancy - baby not yet delivered
Drug Or Alcohol Misuse	SNOMED	199255000	Drug dependence in puerperium - baby previously delivered
Drug Or Alcohol Misuse	SNOMED	1994301000006106	Specialist substance misuse service signposted
Drug Or Alcohol Misuse	SNOMED	200861000000101	Substance misuse management stopped - self withdrawal
Drug Or Alcohol Misuse	SNOMED	201091000000109	Substance misuse treatment withdrawn
Drug Or Alcohol Misuse	SNOMED	201101000000101	Substance misuse treatment programme completed
Drug Or Alcohol Misuse	SNOMED	202561000000103	On substance misuse programme
Drug Or Alcohol Misuse	SNOMED	202961000000106	Ecstasy misuse
Drug Or Alcohol Misuse	SNOMED	202991000000100	Heroin misuse
Drug Or Alcohol Misuse	SNOMED	2043009	Alcoholic gastritis
Drug Or Alcohol Misuse	SNOMED	205231000000102	Opiate dependence detoxification
Drug Or Alcohol Misuse	SNOMED	205788004	FAS - Foetal alcohol syndrome
Drug Or Alcohol Misuse	SNOMED	206572008	Neonat withdrawal symptom from mat use of drug of addiction
Drug Or Alcohol Misuse	SNOMED	226034001	Injecting drug user
Drug Or Alcohol Misuse	SNOMED	228366006	Drug misuse behaviour
Drug Or Alcohol Misuse	SNOMED	228371004	Long-term drug misuser
Drug Or Alcohol Misuse	SNOMED	228372006	Multi-drug misuser
Drug Or Alcohol Misuse	SNOMED	228375008	Misuses drugs orally
Drug Or Alcohol Misuse	SNOMED	228381000	Intranasal drug user
Drug Or Alcohol Misuse	SNOMED	228382007	Misuses drugs vaginally
Drug Or Alcohol Misuse	SNOMED	228383002	Misuses drugs rectally
Drug Or Alcohol Misuse	SNOMED	228384008	Misuses drugs sublingually
Drug Or Alcohol Misuse	SNOMED	228386005	Subcutaneous drug user
Drug Or Alcohol Misuse	SNOMED	228387001	Intramuscular drug user
Drug Or Alcohol Misuse	SNOMED	228388006	IVU - Intravenous drug user
Drug Or Alcohol Misuse	SNOMED	228390007	Frequency of drug misuse
Drug Or Alcohol Misuse	SNOMED	228411009	Time since stopped drug misuse
Drug Or Alcohol Misuse	SNOMED	228412002	Total time drugs misused
Drug Or Alcohol Misuse	SNOMED	228413007	Age at starting drug misuse
Drug Or Alcohol Misuse	SNOMED	231161000000109	Delivery of rehabilitation for alcohol addiction
Drug Or Alcohol Misuse	SNOMED	231458000	Steroid abuse
Drug Or Alcohol Misuse	SNOMED	231461004	Hypnotic or anxiolytic abuse
Drug Or Alcohol Misuse	SNOMED	231462006	Barbiturate abuse
Drug Or Alcohol Misuse	SNOMED	231468005	Lysergic acid diethylamide dependence
Drug Or Alcohol Misuse	SNOMED	231469002	Mescaline dependence
Drug Or Alcohol Misuse	SNOMED	231470001	Anxiolytic dependence
Drug Or Alcohol Misuse	SNOMED	231472009	Barbiturate dependence
Drug Or Alcohol Misuse	SNOMED	231473004	Benzodiazepine dependence
Drug Or Alcohol Misuse	SNOMED	231474005	Valium dependence
Drug Or Alcohol Misuse	SNOMED	231475006	Librium dependence
Drug Or Alcohol Misuse	SNOMED	231477003	Heroin dependence
Drug Or Alcohol Misuse	SNOMED	231478008	Methadone dependence
Drug Or Alcohol Misuse	SNOMED	231479000	Morphine dependence
Drug Or Alcohol Misuse	SNOMED	231480002	Opium dependence
Drug Or Alcohol Misuse	SNOMED	235942001	Alcohol-induced acute pancreatitis
Drug Or Alcohol Misuse	SNOMED	235952002	Alcohol-induced chronic pancreatitis
Drug Or Alcohol Misuse	SNOMED	237228001	Pregnancy and drug dependence
Drug Or Alcohol Misuse	SNOMED	237738005	Alcohol-induced pseudo-Cushing's syndrome
Drug Or Alcohol Misuse	SNOMED	2403008	Dependence syndrome
Drug Or Alcohol Misuse	SNOMED	248021000000105	Current non recreational drug user
Drug Or Alcohol Misuse	SNOMED	25702006	[X]Acute alcoholic drunkenness
Drug Or Alcohol Misuse	SNOMED	26416006	Drug abuse
Drug Or Alcohol Misuse	SNOMED	266707007	Drug dependence therapy
Drug Or Alcohol Misuse	SNOMED	267206008	Drug dependence - unspec whether during pregnancy/puerperium
Drug Or Alcohol Misuse	SNOMED	268640002	Hypnotic or anxiolytic dependence NOS
Drug Or Alcohol Misuse	SNOMED	268641003	Cannabis type drug dependence
Drug Or Alcohol Misuse	SNOMED	268645007	Nondependent alcohol abuse
Drug Or Alcohol Misuse	SNOMED	268646008	Nondependent hallucinogen abuse, unspecified
Drug Or Alcohol Misuse	SNOMED	268647004	Nondependent hypnotic or anxiolytic abuse
Drug Or Alcohol Misuse	SNOMED	268648009	Nondependent amphetamine or other psychostimulant abuse
Drug Or Alcohol Misuse	SNOMED	268727002	[X]Abuse of steroids or hormones
Drug Or Alcohol Misuse	SNOMED	275471001	Psychostimulant dependence
Drug Or Alcohol Misuse	SNOMED	276981000000107	H/O opiate misuse
Drug Or Alcohol Misuse	SNOMED	276991000000109	History of anti-depressant misuse
Drug Or Alcohol Misuse	SNOMED	277001000000103	H/O major tranquilliser misuse
Drug Or Alcohol Misuse	SNOMED	277011000000101	H/O barbiturate misuse
Drug Or Alcohol Misuse	SNOMED	277021000000107	History of solvent misuse
Drug Or Alcohol Misuse	SNOMED	277031000000109	H/O cannabis misuse
Drug Or Alcohol Misuse	SNOMED	277041000000100	H/O hallucinogen misuse
Drug Or Alcohol Misuse	SNOMED	277051000000102	History of crack cocaine misuse
Drug Or Alcohol Misuse	SNOMED	277061000000104	H/O cocaine misuse
Drug Or Alcohol Misuse	SNOMED	277071000000106	History of amfetamine misuse

Drug Or Alcohol Misuse	SNOMED	277081000000108	H/O benzodiazepine misuse
Drug Or Alcohol Misuse	SNOMED	277091000000105	H/O methadone misuse
Drug Or Alcohol Misuse	SNOMED	277101000000102	H/O daily benzodiazepine misuse
Drug Or Alcohol Misuse	SNOMED	277131000000108	H/O weekly benzodiazepine misuse
Drug Or Alcohol Misuse	SNOMED	277161000000103	H/O infrequent benzodiazepine misuse
Drug Or Alcohol Misuse	SNOMED	277191000000109	Previous history of benzodiazepine misuse
Drug Or Alcohol Misuse	SNOMED	277221000000102	H/O daily heroin misuse
Drug Or Alcohol Misuse	SNOMED	277251000000107	H/O weekly heroin misuse
Drug Or Alcohol Misuse	SNOMED	277281000000101	Previous history of heroin misuse
Drug Or Alcohol Misuse	SNOMED	277311000000103	H/O infrequent heroin misuse
Drug Or Alcohol Misuse	SNOMED	277341000000102	H/O daily methadone misuse
Drug Or Alcohol Misuse	SNOMED	277371000000108	H/O weekly methadone misuse
Drug Or Alcohol Misuse	SNOMED	277401000000105	H/O infrequent methadone misuse
Drug Or Alcohol Misuse	SNOMED	277431000000104	Previous history of methadone misuse
Drug Or Alcohol Misuse	SNOMED	277461000000109	History of daily ecstasy misuse
Drug Or Alcohol Misuse	SNOMED	277491000000103	H/O weekly ecstasy misuse
Drug Or Alcohol Misuse	SNOMED	277521000000100	History of infrequent ecstasy misuse
Drug Or Alcohol Misuse	SNOMED	277551000000105	Previous history of ecstasy misuse
Drug Or Alcohol Misuse	SNOMED	277581000000104	H/O daily amphetamine misuse
Drug Or Alcohol Misuse	SNOMED	277611000000105	H/O weekly amphetamine misuse
Drug Or Alcohol Misuse	SNOMED	277641000000106	H/O infrequent amphetamine misuse
Drug Or Alcohol Misuse	SNOMED	277671000000100	Previous history of amphetamine misuse
Drug Or Alcohol Misuse	SNOMED	277701000000101	H/O daily major tranquilliser misuse
Drug Or Alcohol Misuse	SNOMED	277731000000107	H/O weekly major tranquilliser misuse
Drug Or Alcohol Misuse	SNOMED	277761000000102	H/O infrequent major tranquilliser misuse
Drug Or Alcohol Misuse	SNOMED	277791000000108	Previous history of major tranquilliser misuse
Drug Or Alcohol Misuse	SNOMED	277821000000103	History of daily anti-depressant misuse
Drug Or Alcohol Misuse	SNOMED	277851000000108	H/O weekly anti-depressant misuse
Drug Or Alcohol Misuse	SNOMED	277881000000102	H/O infrequent anti-depressant misuse
Drug Or Alcohol Misuse	SNOMED	277911000000102	Previous history of anti-depressant misuse
Drug Or Alcohol Misuse	SNOMED	277941000000101	H/O daily opiate misuse
Drug Or Alcohol Misuse	SNOMED	277971000000107	History of weekly opiate misuse
Drug Or Alcohol Misuse	SNOMED	278001000000102	History of infrequent opiate misuse
Drug Or Alcohol Misuse	SNOMED	278031000000108	Previous history of opiate misuse
Drug Or Alcohol Misuse	SNOMED	278061000000103	History of daily cocaine misuse
Drug Or Alcohol Misuse	SNOMED	278091000000109	H/O weekly cocaine misuse
Drug Or Alcohol Misuse	SNOMED	278121000000105	H/O infrequent cocaine misuse
Drug Or Alcohol Misuse	SNOMED	278151000000100	Previous history of cocaine misuse
Drug Or Alcohol Misuse	SNOMED	278181000000106	H/O daily crack cocaine misuse
Drug Or Alcohol Misuse	SNOMED	278211000000107	History of weekly crack cocaine misuse
Drug Or Alcohol Misuse	SNOMED	278241000000108	History of infrequent crack cocaine misuse
Drug Or Alcohol Misuse	SNOMED	278271000000102	Previous history of crack cocaine misuse
Drug Or Alcohol Misuse	SNOMED	278301000000104	History of daily hallucinogen misuse
Drug Or Alcohol Misuse	SNOMED	278331000000105	History of weekly hallucinogen misuse
Drug Or Alcohol Misuse	SNOMED	278361000000100	History of infrequent hallucinogen misuse
Drug Or Alcohol Misuse	SNOMED	278391000000106	Previous history of hallucinogen misuse
Drug Or Alcohol Misuse	SNOMED	278421000000100	H/O daily barbiturate misuse
Drug Or Alcohol Misuse	SNOMED	278451000000105	H/O weekly barbiturate misuse
Drug Or Alcohol Misuse	SNOMED	278481000000104	H/O infrequent barbiturate misuse
Drug Or Alcohol Misuse	SNOMED	278511000000105	Previous history of barbiturate misuse
Drug Or Alcohol Misuse	SNOMED	278541000000106	H/O daily cannabis misuse
Drug Or Alcohol Misuse	SNOMED	278571000000100	History of weekly cannabis misuse
Drug Or Alcohol Misuse	SNOMED	278601000000107	H/O infrequent cannabis misuse
Drug Or Alcohol Misuse	SNOMED	278631000000101	Previous history of cannabis misuse
Drug Or Alcohol Misuse	SNOMED	278661000000106	History of daily solvent misuse
Drug Or Alcohol Misuse	SNOMED	278691000000100	H/O weekly solvent misuse
Drug Or Alcohol Misuse	SNOMED	278721000000109	H/O infrequent solvent misuse
Drug Or Alcohol Misuse	SNOMED	278751000000104	Previous history of solvent misuse
Drug Or Alcohol Misuse	SNOMED	279441000000100	History of heroin misuse
Drug Or Alcohol Misuse	SNOMED	279451000000102	H/O ecstasy misuse
Drug Or Alcohol Misuse	SNOMED	279651000000104	Disqualified from driving due to excess alcohol
Drug Or Alcohol Misuse	SNOMED	280982009	Laxative abuse
Drug Or Alcohol Misuse	SNOMED	280983004	[X]Abuse of vitamins
Drug Or Alcohol Misuse	SNOMED	280984005	[X]Abuse of herbal or folk remedies
Drug Or Alcohol Misuse	SNOMED	280986007	Antacid abuse
Drug Or Alcohol Misuse	SNOMED	29212009	[X]Men & behav dis due to use alcohol: oth men & behav dis
Drug Or Alcohol Misuse	SNOMED	292410009	[X]Psychostim with abuse potentl caus advers eff therap use
Drug Or Alcohol Misuse	SNOMED	299881000000104	Substance misuse treatment declined
Drug Or Alcohol Misuse	SNOMED	299911000000104	Substance misuse treatment not available
Drug Or Alcohol Misuse	SNOMED	300471000000101	Abstinent from substance misuse
Drug Or Alcohol Misuse	SNOMED	307052004	Illicit drug use
Drug Or Alcohol Misuse	SNOMED	308742005	Alcohol withdrawal-induced convulsion
Drug Or Alcohol Misuse	SNOMED	313071005	Counseling for substance abuse
Drug Or Alcohol Misuse	SNOMED	31511000033102	Drugs used in Substance Dependence
Drug Or Alcohol Misuse	SNOMED	31956009	Cocaine dependence, unspecified

Drug Or Alcohol Misuse	SNOMED	361055000	Misuse of drugs NOS
Drug Or Alcohol Misuse	SNOMED	361272001	Cerebellar ataxia due to alcoholism
Drug Or Alcohol Misuse	SNOMED	366371000000105	Brief intervention for excessive alcohol consumptn completed
Drug Or Alcohol Misuse	SNOMED	366421000000103	Extended intervention for excessive alcohol consumptn complt
Drug Or Alcohol Misuse	SNOMED	371422002	[V]Personal history of alcoholism
Drug Or Alcohol Misuse	SNOMED	371435006	[V]Personal history of drug abuse by injection
Drug Or Alcohol Misuse	SNOMED	372511000000103	Shared care drug misuse treatment
Drug Or Alcohol Misuse	SNOMED	372541000000102	Drug misuse treatment primary care - enhanced services admin
Drug Or Alcohol Misuse	SNOMED	379411000000105	Brief intervention for excessive alcohol consumptn declined
Drug Or Alcohol Misuse	SNOMED	38247002	Hallucinogen dependence
Drug Or Alcohol Misuse	SNOMED	383871000000108	Health problem secondary to drug misuse
Drug Or Alcohol Misuse	SNOMED	407586004	H/O: recreational drug use
Drug Or Alcohol Misuse	SNOMED	408943006	Lifestyle advice regarding drug misuse
Drug Or Alcohol Misuse	SNOMED	408947007	Alcohol abuse prevention education
Drug Or Alcohol Misuse	SNOMED	409921000000107	[X]Men & behav disorder multiple drug use/psychoactive subst
Drug Or Alcohol Misuse	SNOMED	414054004	Drug dependence home detoxification
Drug Or Alcohol Misuse	SNOMED	414055003	Drug dependence home detoxification contraindicated
Drug Or Alcohol Misuse	SNOMED	414056002	Drug dependence self detoxification
Drug Or Alcohol Misuse	SNOMED	414874007	Nondependent amphetamine/psychostimulant abuse in remission
Drug Or Alcohol Misuse	SNOMED	415659002	Substance misuse clinical management plan agreed
Drug Or Alcohol Misuse	SNOMED	415660007	Substance misuse decreased
Drug Or Alcohol Misuse	SNOMED	415661006	Substance misuse increased
Drug Or Alcohol Misuse	SNOMED	416262000	Continuous use of drugs
Drug Or Alcohol Misuse	SNOMED	416437003	Preoccupied with substance misuse
Drug Or Alcohol Misuse	SNOMED	416479009	Previously injecting drug user
Drug Or Alcohol Misuse	SNOMED	416751004	Reduced drugs misuse
Drug Or Alcohol Misuse	SNOMED	417252002	Episodic use of drugs
Drug Or Alcohol Misuse	SNOMED	417284009	Current drug user
Drug Or Alcohol Misuse	SNOMED	417471002	Persistent substance misuse
Drug Or Alcohol Misuse	SNOMED	417633001	Alcohol induced hallucinations
Drug Or Alcohol Misuse	SNOMED	42344001	[X]Men & behav dis due alcoh: resid & late-onset psychot dis
Drug Or Alcohol Misuse	SNOMED	426590003	Nondependent other drug abuse, continuous
Drug Or Alcohol Misuse	SNOMED	427327003	[X]Mental and behav dis due to seds/hypntcs: dependence synd
Drug Or Alcohol Misuse	SNOMED	442406005	Amphetamine or other psychostimulant dependence
Drug Or Alcohol Misuse	SNOMED	44870007	Misused drugs in past
Drug Or Alcohol Misuse	SNOMED	468061000000108	[X]Mental and behavioural disorders due to multiple drug use and use of other
Drug Or Alcohol Misuse	SNOMED	468861000000101	[V]Drug use
Drug Or Alcohol Misuse	SNOMED	494041000000102	Seen in substance misuse clinic
Drug Or Alcohol Misuse	SNOMED	49540005	Nondependent other drug abuse
Drug Or Alcohol Misuse	SNOMED	505091000000102	Declined consent for notification of drug misuse
Drug Or Alcohol Misuse	SNOMED	507041000000101	Pharmacy attended for drug misuse
Drug Or Alcohol Misuse	SNOMED	589511000000000	Alcoholic liver damage unspecified
Drug Or Alcohol Misuse	SNOMED	589511000000102	Alcoholic liver damage unspecified
Drug Or Alcohol Misuse	SNOMED	623961000000108	Other alcoholic psychosis
Drug Or Alcohol Misuse	SNOMED	64297001	Detoxication therapy for alcoholism
Drug Or Alcohol Misuse	SNOMED	64386003	Tranquillizer abuse
Drug Or Alcohol Misuse	SNOMED	6525002	Dependent drug abuse
Drug Or Alcohol Misuse	SNOMED	66214007	Non-dependent abuse of drugs
Drug Or Alcohol Misuse	SNOMED	66590003	Alcoholism
Drug Or Alcohol Misuse	SNOMED	69482004	Wernicke-Korsakov syndrome
Drug Or Alcohol Misuse	SNOMED	699124006	Admission to substance misuse detoxification center
Drug Or Alcohol Misuse	SNOMED	700187009	Seen in drug misuse clinic
Drug Or Alcohol Misuse	SNOMED	7052005	[X]Alcoholic hallucinosis
Drug Or Alcohol Misuse	SNOMED	712542001	Abstinent from drug misuse
Drug Or Alcohol Misuse	SNOMED	712543006	Abstinent from drug misuse in normal environment
Drug Or Alcohol Misuse	SNOMED	712544000	Abstinent from drug misuse in protected environment
Drug Or Alcohol Misuse	SNOMED	712545004	Abstinent from drug misuse on maintenance replacement
Drug Or Alcohol Misuse	SNOMED	712546003	Abstinent from drug misuse when receiving blocking therapy
Drug Or Alcohol Misuse	SNOMED	713775002	Novel psychoactive substance misuse
Drug Or Alcohol Misuse	SNOMED	715200006	H/O novel psychoactive substance misuse
Drug Or Alcohol Misuse	SNOMED	7200002	Alcoholism
Drug Or Alcohol Misuse	SNOMED	73097000	[X]Mental and behav dis due to use alcohol: amnesic syndrome
Drug Or Alcohol Misuse	SNOMED	754831000000102	Extended interven for excessive alcohol consumption declined
Drug Or Alcohol Misuse	SNOMED	75544000	Unspecified opioid dependence
Drug Or Alcohol Misuse	SNOMED	783241000000102	Declined to give substance misuse history
Drug Or Alcohol Misuse	SNOMED	83521008	Alcoholic cardiomyopathy
Drug Or Alcohol Misuse	SNOMED	85005007	Cannabis dependence, unspecified
Drug Or Alcohol Misuse	SNOMED	851461000000000	Drug misuse behaviour
Drug Or Alcohol Misuse	SNOMED	851461000006104	Drug misuse behaviour
Drug Or Alcohol Misuse	SNOMED	852211000000000	Misused drugs in past
Drug Or Alcohol Misuse	SNOMED	852211000006108	Misused drugs in past
Drug Or Alcohol Misuse	SNOMED	85561006	Uncomplicated alcohol withdrawal
Drug Or Alcohol Misuse	SNOMED	8635005	Alcohol withdrawal delirium
Drug Or Alcohol Misuse	SNOMED	873931000000108	Benzodiazepine dependence detoxification
Drug Or Alcohol Misuse	SNOMED	894311000000000	In-house substance misuse treatment

Drug Or Alcohol Misuse	SNOMED	894311000000107	In-house substance misuse treatment
Drug Or Alcohol Misuse	SNOMED	907461000006107	[RFC] Prevention of substance misuse
Drug Or Alcohol Misuse	SNOMED	910581000006100	[RFC] Drug/ substance abuse
Drug Or Alcohol Misuse	SNOMED	910601000006105	[RFC] Substance abuse
Drug Or Alcohol Misuse	SNOMED	91388009	[X]Ment & behav dis due use alcohol: unsp ment & behav dis
Drug Or Alcohol Misuse	SNOMED	918321000000000	Alcohol dependence resolved
Drug Or Alcohol Misuse	SNOMED	918321000000104	Alcohol dependence resolved
Drug Or Alcohol Misuse	SNOMED	940781000006100	Evidence of alcohol withdrawal
Drug Or Alcohol Misuse	SNOMED	961561000006100	Abuses over-the-counter/illicit drugs
Drug Or Alcohol Misuse	SNOMED	961571000006107	Excessive use of alcohol
Drug Or Alcohol Misuse	SNOMED	968631000006100	Substance misuse of Amphetamines
Drug Or Alcohol Misuse	SNOMED	968651000006107	Substance misuse of Cannabis
Drug Or Alcohol Misuse	SNOMED	968681000006104	Misuse of Cannabis unspecified
Drug Or Alcohol Misuse	SNOMED	968691000006101	Misuse of Cannabis herbal
Drug Or Alcohol Misuse	SNOMED	968711000006103	Misuse of Cannabis resin
Drug Or Alcohol Misuse	SNOMED	968721000006106	Misuse of Cannabis Oil
Drug Or Alcohol Misuse	SNOMED	968731000006109	Misuse of Cannabis herbal (skunk)
Drug Or Alcohol Misuse	SNOMED	968741000006104	Substance misuse of Heroin
Drug Or Alcohol Misuse	SNOMED	968751000006102	Substance misuse of Solvents
Drug Or Alcohol Misuse	SNOMED	968761000006100	Misuse of Heroin illicit
Drug Or Alcohol Misuse	SNOMED	968771000006107	Misuse of Diamorphine
Drug Or Alcohol Misuse	SNOMED	968791000006108	Misuse of Diamorphine Hydrochl elixir
Drug Or Alcohol Misuse	SNOMED	968811000006107	Misuse of Diamorphine Hydrochl amps
Drug Or Alcohol Misuse	SNOMED	968821000006104	Misuse of Diamorphine Hydrochl reefers
Drug Or Alcohol Misuse	SNOMED	968841000006106	Substance misuse of Methadone
Drug Or Alcohol Misuse	SNOMED	968871000006103	Misuse of Methadone unspecified
Drug Or Alcohol Misuse	SNOMED	968901000006103	Misuse of Methadone mixture
Drug Or Alcohol Misuse	SNOMED	968931000006106	Misuse of Methadone linctus
Drug Or Alcohol Misuse	SNOMED	968951000006104	Misuse of Methadone tablets
Drug Or Alcohol Misuse	SNOMED	968961000006102	Misuse of Methadone amps
Drug Or Alcohol Misuse	SNOMED	968981000006107	Misuse of Methadone suppositories
Drug Or Alcohol Misuse	SNOMED	969001000006105	Substance misuse of other Opiates
Drug Or Alcohol Misuse	SNOMED	969011000006108	Misuse of Opiates unspecified
Drug Or Alcohol Misuse	SNOMED	969021000006100	Misuse of Morphine Sulphate
Drug Or Alcohol Misuse	SNOMED	969041000006107	Misuse of Opium
Drug Or Alcohol Misuse	SNOMED	969051000006109	Misuse of Dihydrocodeine
Drug Or Alcohol Misuse	SNOMED	969061000006106	Misuse of Dextromoramide
Drug Or Alcohol Misuse	SNOMED	969071000006104	Misuse of Dipianone
Drug Or Alcohol Misuse	SNOMED	969081000006101	Misuse of Pethidine
Drug Or Alcohol Misuse	SNOMED	969091000006103	Misuse of Morphine Sulphate amps
Drug Or Alcohol Misuse	SNOMED	969101000006109	Misuse of Hydromorphone
Drug Or Alcohol Misuse	SNOMED	969111000006107	Misuse of Oxymorphone
Drug Or Alcohol Misuse	SNOMED	969121000006104	Misuse of Anileridine
Drug Or Alcohol Misuse	SNOMED	969131000006101	Misuse of Hydrocodone
Drug Or Alcohol Misuse	SNOMED	969141000006106	Misuse of Oxycodone
Drug Or Alcohol Misuse	SNOMED	969151000006108	Misuse of Ethoheptazine
Drug Or Alcohol Misuse	SNOMED	969161000006105	Misuse of Fentanyl
Drug Or Alcohol Misuse	SNOMED	969171000006103	Misuse of Levorphanol
Drug Or Alcohol Misuse	SNOMED	969181000006100	Misuse of Phenoperidine
Drug Or Alcohol Misuse	SNOMED	969191000006102	Misuse of Phenazocine
Drug Or Alcohol Misuse	SNOMED	969201000006104	Misuse of Papaveretum
Drug Or Alcohol Misuse	SNOMED	969211000006101	Misuse of Meptazinol
Drug Or Alcohol Misuse	SNOMED	969221000006109	Misuse of Piritramide
Drug Or Alcohol Misuse	SNOMED	969231000006107	Misuse of Codeine tablets
Drug Or Alcohol Misuse	SNOMED	969241000006102	Misuse of Tramadol Hydrochloride
Drug Or Alcohol Misuse	SNOMED	969251000006100	Misuse of Dextropropoxyphene
Drug Or Alcohol Misuse	SNOMED	969261000006103	Misuse of Opiate containing mixture
Drug Or Alcohol Misuse	SNOMED	969271000006105	Misuse of Codeine linctus
Drug Or Alcohol Misuse	SNOMED	969281000006108	Misuse of Pentazocine
Drug Or Alcohol Misuse	SNOMED	969291000006106	Misuse of Buprenorphine
Drug Or Alcohol Misuse	SNOMED	969301000006107	Misuse of Gee's linctus
Drug Or Alcohol Misuse	SNOMED	969311000006105	Misuse of Codeine unspecified
Drug Or Alcohol Misuse	SNOMED	969321000006102	Misuse of Collis-brown
Drug Or Alcohol Misuse	SNOMED	969331000006104	Misuse of Opiate compound analgesics
Drug Or Alcohol Misuse	SNOMED	969341000006109	Misuse of Nalbuphine
Drug Or Alcohol Misuse	SNOMED	969351000006106	Misuse of Phensedyl
Drug Or Alcohol Misuse	SNOMED	969361000006108	Misuse of Alphaprodine
Drug Or Alcohol Misuse	SNOMED	969371000006101	Misuse of Actifed
Drug Or Alcohol Misuse	SNOMED	969381000006103	Misuse of Kaolin and Morphine
Drug Or Alcohol Misuse	SNOMED	969391000006100	Misuse of other Opiates
Drug Or Alcohol Misuse	SNOMED	969401000006103	Substance misuse of Benzodiazepines
Drug Or Alcohol Misuse	SNOMED	969411000006100	Misuse of Benzodiazepines unspecified
Drug Or Alcohol Misuse	SNOMED	969421000006108	Misuse of Diazepam
Drug Or Alcohol Misuse	SNOMED	969431000006106	Misuse of Chlordiazepoxide
Drug Or Alcohol Misuse	SNOMED	969441000006101	Misuse of Nitrazepam

Drug Or Alcohol Misuse	SNOMED	969451000006104	Misuse of Lorazepam
Drug Or Alcohol Misuse	SNOMED	969461000006102	Misuse of Clobazam
Drug Or Alcohol Misuse	SNOMED	969471000006109	Misuse of Clorazepate
Drug Or Alcohol Misuse	SNOMED	969481000006107	Misuse of Ketazolam
Drug Or Alcohol Misuse	SNOMED	969491000006105	Misuse of Medazepam
Drug Or Alcohol Misuse	SNOMED	969501000006102	Misuse of Oxazepam
Drug Or Alcohol Misuse	SNOMED	969511000006104	Misuse of Lurazepam
Drug Or Alcohol Misuse	SNOMED	969521000006107	Misuse of Temazepam
Drug Or Alcohol Misuse	SNOMED	969531000006105	Misuse of Bromazepam
Drug Or Alcohol Misuse	SNOMED	969541000006100	Misuse of Triazolam
Drug Or Alcohol Misuse	SNOMED	969551000006103	Misuse of Lormetazepam
Drug Or Alcohol Misuse	SNOMED	969561000006101	Misuse of Flunitrazepam
Drug Or Alcohol Misuse	SNOMED	969571000006108	Misuse of Prazepam
Drug Or Alcohol Misuse	SNOMED	969581000006106	Misuse of Chlormezanone
Drug Or Alcohol Misuse	SNOMED	969591000006109	Misuse of Loprazolam
Drug Or Alcohol Misuse	SNOMED	969601000006101	Misuse of Alprazolam
Drug Or Alcohol Misuse	SNOMED	969611000006103	Misuse of Amphetamine Sulphate
Drug Or Alcohol Misuse	SNOMED	969621000006106	Misuse of Amphetamine (pharmaceutical)
Drug Or Alcohol Misuse	SNOMED	969631000006109	Misuse of Methamphetamine
Drug Or Alcohol Misuse	SNOMED	969641000006104	Misuse of Dexamphetamine (Dexadrine)
Drug Or Alcohol Misuse	SNOMED	969651000006102	Misuse of Dexamphetamine linctus/syrup
Drug Or Alcohol Misuse	SNOMED	969661000006100	Misuse of Clonazepam
Drug Or Alcohol Misuse	SNOMED	969671000006107	Misuse of Midazolam
Drug Or Alcohol Misuse	SNOMED	969681000006105	Misuse of Dexamphetamine reefers
Drug Or Alcohol Misuse	SNOMED	969691000006108	Misuse of Methamphetamine amps
Drug Or Alcohol Misuse	SNOMED	969701000006108	Substance misuse of Ecstasy
Drug Or Alcohol Misuse	SNOMED	969711000006106	Substance misuse of Cocaine
Drug Or Alcohol Misuse	SNOMED	969721000006103	Misuse of MDMA
Drug Or Alcohol Misuse	SNOMED	969731000006100	Misuse of Methylenedioxyamphetamine
Drug Or Alcohol Misuse	SNOMED	969741000006105	Substance misuse of Crack
Drug Or Alcohol Misuse	SNOMED	969751000006107	Misuse of Solvents unspecified
Drug Or Alcohol Misuse	SNOMED	969761000006109	Misuse of Cocaine unspecified
Drug Or Alcohol Misuse	SNOMED	969771000006102	Misuse of Cocaine Hydrochloride
Drug Or Alcohol Misuse	SNOMED	969781000006104	Misuse of Cocaine freebase (crack)
Drug Or Alcohol Misuse	SNOMED	969791000006101	Substance misuse of Hallucinogens
Drug Or Alcohol Misuse	SNOMED	969801000006100	Misuse of Hallucinogens unspecified
Drug Or Alcohol Misuse	SNOMED	969811000006102	Misuse of Toluene (glue)
Drug Or Alcohol Misuse	SNOMED	969821000006105	Misuse of Mescaline
Drug Or Alcohol Misuse	SNOMED	969831000006108	Misuse of Butane
Drug Or Alcohol Misuse	SNOMED	969841000006103	Misuse of Psiloybin
Drug Or Alcohol Misuse	SNOMED	969851000006101	Misuse of Amyl Nitrate
Drug Or Alcohol Misuse	SNOMED	969861000006104	Misuse of Lysergide (LSD)
Drug Or Alcohol Misuse	SNOMED	969871000006106	Misuse of Acetone
Drug Or Alcohol Misuse	SNOMED	969881000006109	Misuse of Phencyclidine
Drug Or Alcohol Misuse	SNOMED	969891000006107	Misuse of Fluorocarbons
Drug Or Alcohol Misuse	SNOMED	969901000006106	Misuse of Trichloroethylene
Drug Or Alcohol Misuse	SNOMED	969911000006109	Substance misuse of Barbiturates
Drug Or Alcohol Misuse	SNOMED	969921000006101	Misuse of Barbiturates unspecified
Drug Or Alcohol Misuse	SNOMED	969931000006103	Misuse of Amylobarbitone
Drug Or Alcohol Misuse	SNOMED	969941000006108	Misuse of Pentabarbitone
Drug Or Alcohol Misuse	SNOMED	969951000006105	Misuse of Quinalbarbitone
Drug Or Alcohol Misuse	SNOMED	969961000006107	Misuse of Phenobarbitone
Drug Or Alcohol Misuse	SNOMED	969971000006100	Misuse of Ketamine
Drug Or Alcohol Misuse	SNOMED	969981000006102	Misuse of Amphetamines unspecified
Drug Or Alcohol Misuse	SNOMED	969991000006104	Substance misuse of other drugs
Drug Or Alcohol Misuse	SNOMED	970001000006107	Misuse of Sedatives unspecified
Drug Or Alcohol Misuse	SNOMED	970011000006105	Misuse of Anti-histamines unspecified
Drug Or Alcohol Misuse	SNOMED	970021000006102	Misuse of Hydroxyzine
Drug Or Alcohol Misuse	SNOMED	970031000006104	Misuse of Cyclizine
Drug Or Alcohol Misuse	SNOMED	970041000006109	Misuse of Promethazine
Drug Or Alcohol Misuse	SNOMED	970051000006106	Misuse of non-Barb/Benzo sedatives unsp
Drug Or Alcohol Misuse	SNOMED	970061000006108	Misuse of Methaqualone
Drug Or Alcohol Misuse	SNOMED	970071000006101	Misuse of Chlormethiazole
Drug Or Alcohol Misuse	SNOMED	970081000006103	Misuse of Meprobamate
Drug Or Alcohol Misuse	SNOMED	970091000006100	Misuse of Zopliclone
Drug Or Alcohol Misuse	SNOMED	970101000006106	Misuse of Butobarbitone
Drug Or Alcohol Misuse	SNOMED	970111000006109	Misuse of Zolpidem Tartrate
Drug Or Alcohol Misuse	SNOMED	970121000006101	Misuse of Heptabarbitone
Drug Or Alcohol Misuse	SNOMED	970131000006103	Misuse of Propranolol
Drug Or Alcohol Misuse	SNOMED	970141000006108	Misuse of Chloral derivatives
Drug Or Alcohol Misuse	SNOMED	970151000006105	Misuse of Glutethimide
Drug Or Alcohol Misuse	SNOMED	970161000006107	Misuse of Cyclobarbitone
Drug Or Alcohol Misuse	SNOMED	970171000006100	Misuse of Mephenesin
Drug Or Alcohol Misuse	SNOMED	970181000006102	Misuse of Hexobarbitone
Drug Or Alcohol Misuse	SNOMED	970191000006104	Misuse of Methylpentylynol

Drug Or Alcohol Misuse	SNOMED	970201000006101	Misuse of Barbitone unbranded
Drug Or Alcohol Misuse	SNOMED	970211000006103	Misuse of Methylprylone
Drug Or Alcohol Misuse	SNOMED	970221000006106	Misuse of Methylphenobarbitone
Drug Or Alcohol Misuse	SNOMED	970231000006109	Misuse of Oxyprenolol Hydrochloride
Drug Or Alcohol Misuse	SNOMED	970241000006104	Misuse of other sedatives
Drug Or Alcohol Misuse	SNOMED	970251000006102	Substance misuse of major tranquillisers
Drug Or Alcohol Misuse	SNOMED	970261000006100	Misuse of major tranquillisers unspecified
Drug Or Alcohol Misuse	SNOMED	970271000006107	Misuse of Chlorpromazine (Largactyl)
Drug Or Alcohol Misuse	SNOMED	970281000006105	Substance misuse of Anti-depressants
Drug Or Alcohol Misuse	SNOMED	970291000006108	Misuse of Anti-depressants unspecified
Drug Or Alcohol Misuse	SNOMED	970301000006109	Misuse of Fluoxetine
Drug Or Alcohol Misuse	SNOMED	970311000006107	Misuse of Amitriptyline
Drug Or Alcohol Misuse	SNOMED	970331000006101	Misuse of Dothepin Hydrochloride
Drug Or Alcohol Misuse	SNOMED	970341000006106	Misuse of Clonidine
Drug Or Alcohol Misuse	SNOMED	970351000006108	Misuse of Lofepramine
Drug Or Alcohol Misuse	SNOMED	970361000006105	Misuse of Paroxetine
Drug Or Alcohol Misuse	SNOMED	970371000006103	Misuse of Lofexidine
Drug Or Alcohol Misuse	SNOMED	970381000006100	Misuse of Steroids unspecified
Drug Or Alcohol Misuse	SNOMED	970421000006105	Misuse of Nandrolone
Drug Or Alcohol Misuse	SNOMED	970441000006103	Misuse of Stanozolol
Drug Or Alcohol Misuse	SNOMED	970471000006106	Misuse of Tetrecosactrin
Drug Or Alcohol Misuse	SNOMED	970481000006109	Misuse of Antipsychotic unspecified
Drug Or Alcohol Misuse	SNOMED	970501000006104	Misuse of Depixol
Drug Or Alcohol Misuse	SNOMED	970511000006101	Misuse of Thioridazine
Drug Or Alcohol Misuse	SNOMED	970521000006109	Misuse of Trifluoperazine
Drug Or Alcohol Misuse	SNOMED	970531000006107	Substance misuse of poly drug
Drug Or Alcohol Misuse	SNOMED	970551000006100	Misuse of Stimulants unspec
Drug Or Alcohol Misuse	SNOMED	970561000006103	Misuse of Drinamyl
Drug Or Alcohol Misuse	SNOMED	970571000006105	Misuse of appetite suppressant
Drug Or Alcohol Misuse	SNOMED	970581000006108	Misuse of Diethylproprion
Drug Or Alcohol Misuse	SNOMED	970591000006106	Misuse of Phenmetrazine
Drug Or Alcohol Misuse	SNOMED	970601000006103	Misuse of Fenfluoramine
Drug Or Alcohol Misuse	SNOMED	970611000006100	Misuse of Mazindol
Drug Or Alcohol Misuse	SNOMED	970621000006108	Misuse of Phenteramine
Drug Or Alcohol Misuse	SNOMED	970631000006106	Misuse of other stimulants
Drug Or Alcohol Misuse	SNOMED	970641000006101	Misuse of Methylphenidate
Drug Or Alcohol Misuse	SNOMED	970651000006104	Misuse of Pemoline
Drug Or Alcohol Misuse	SNOMED	970661000006102	Misuse of Prolintane
Drug Or Alcohol Misuse	SNOMED	970671000006109	Misuse of Fencamfamin
Drug Or Alcohol Misuse	SNOMED	970681000006107	Misuse of Caffeine
Drug Or Alcohol Misuse	SNOMED	970691000006105	Misuse of Khat
Drug Or Alcohol Misuse	SNOMED	970701000006105	Misuse of other Psychoactive drugs unsp
Drug Or Alcohol Misuse	SNOMED	970711000006108	Misuse of Procylidine Hydrochloride
Drug Or Alcohol Misuse	SNOMED	970721000006100	Misuse of GHB/GBH
Drug Or Alcohol Misuse	SNOMED	970731000006102	Misuse of minor Analgesics
Drug Or Alcohol Misuse	SNOMED	970741000006107	Misuse of anti-diarrhoea/anti-emetic
Drug Or Alcohol Misuse	SNOMED	970751000006109	Misuse of Naltrexone
Drug Or Alcohol Misuse	SNOMED	984921000000106	Injury following unknown drug use
Drug Or Alcohol Misuse	SNOMED	994121000006103	Abscess of antecubital fossa related to intravenous drug use
Eating Disorders	ICD10	F500	Anorexia nervosa
Eating Disorders	ICD10	F501	Atypical anorexia nervosa
Eating Disorders	ICD10	F502	Bulimia nervosa
Eating Disorders	ICD10	F503	Atypical bulimia nervosa
Eating Disorders	ICD10	F504	Overeating associated with other psychological disturbances
Eating Disorders	ICD10	F505	Vomiting associated with other psychological disturbances
Eating Disorders	ICD10	F506	Eating disorders
Eating Disorders	ICD10	F507	Eating disorders
Eating Disorders	ICD10	F508	Other eating disorders
Eating Disorders	ICD10	F509	Eating disorders
Eating Disorders	ICD10	F983	Pica of infancy and childhood
Eating Disorders	SNOMED	14077003	Pica
Eating Disorders	SNOMED	161471008	H/O: anorexia nervosa
Eating Disorders	SNOMED	1620911000006108	Eating disorders
Eating Disorders	SNOMED	1664151000006105	Seen in eating disorders clinic
Eating Disorders	SNOMED	1764491000006100	Referral to eating disorders outreach clinic
Eating Disorders	SNOMED	1776641000006108	Reason for referral: Eating Disorder
Eating Disorders	SNOMED	191970005	Cyclical vomiting - psychogenic
Eating Disorders	SNOMED	192017004	[X]Psychogenic loss of appetite
Eating Disorders	SNOMED	192450008	[X]Vomiting associated with other psychological disturbances
Eating Disorders	SNOMED	231522009	[X]Atypical anorexia nervosa
Eating Disorders	SNOMED	231523004	[X]Atypical bulimia nervosa
Eating Disorders	SNOMED	270902002	[X]Overeating associated with other psychological disturbncs
Eating Disorders	SNOMED	37224001	Psychogenic vomiting NOS
Eating Disorders	SNOMED	400973003	Referral to eating disorders clinic
Eating Disorders	SNOMED	407666007	Nocturnal sleep-related eating disorder

Eating Disorders	SNOMED	439437006	Seen in eating disorder clinic
Eating Disorders	SNOMED	56882008	[X]Anorexia nervosa
Eating Disorders	SNOMED	608241000000102	Eating disorder NOS - psychog.
Eating Disorders	SNOMED	608251000000000	Unspecified non-organic eating disorder
Eating Disorders	SNOMED	608251000000104	Unspecified non-organic eating disorder
Eating Disorders	SNOMED	72366004	Unspecified non-organic eating disorder
Eating Disorders	SNOMED	78004001	[X]Bulimia nervosa
Eating Disorders	SNOMED	897261000000108	Suspected binge eating disorder
Endometriosis Adenomyosis	ICD10	N80	endometriosis adenomyosis
Endometriosis Adenomyosis	SNOMED	10535001	Endometriosis of the broad ligament
Endometriosis Adenomyosis	SNOMED	128726006	[M]Stromal endometriosis
Endometriosis Adenomyosis	SNOMED	129103003	Endometriosis
Endometriosis Adenomyosis	SNOMED	17829005	Endometriosis of the umbilicus
Endometriosis Adenomyosis	SNOMED	198247003	Internal endometriosis
Endometriosis Adenomyosis	SNOMED	198251001	Endometriosis of the pelvic peritoneum NOS
Endometriosis Adenomyosis	SNOMED	22611009	Endometriosis of the fallopian tube
Endometriosis Adenomyosis	SNOMED	237115002	Endometriosis of myometrium
Endometriosis Adenomyosis	SNOMED	237116001	Chocolate cyst of ovary
Endometriosis Adenomyosis	SNOMED	266589005	Endometriosis of ovary
Endometriosis Adenomyosis	SNOMED	38780008	Endometriosis of the vulva
Endometriosis Adenomyosis	SNOMED	416155004	Laparoscopic laser destruction of endometriosis
Endometriosis Adenomyosis	SNOMED	44601009	Endometriosis of colon
Endometriosis Adenomyosis	SNOMED	50993001	Endometriosis of the lung
Endometriosis Adenomyosis	SNOMED	52533003	Endometriosis of the rectovaginal septum
Endometriosis Adenomyosis	SNOMED	5327000	Endometriosis of the round ligament
Endometriosis Adenomyosis	SNOMED	53913001	Endometriosis in scar of skin
Endometriosis Adenomyosis	SNOMED	5562006	Endometriosis of the intestine NOS
Endometriosis Adenomyosis	SNOMED	57493005	Endometriosis of the vagina
Endometriosis Adenomyosis	SNOMED	61640006	Endometriosis of cervix
Endometriosis Adenomyosis	SNOMED	64286001	Endometriosis of the cul-de-sac
Endometriosis Adenomyosis	SNOMED	65099004	Endometriosis of the bladder
Endometriosis Adenomyosis	SNOMED	76376003	Endometriosis of uterus
Endometriosis Adenomyosis	SNOMED	8421002	Endometriosis of appendix
Endometriosis Adenomyosis	SNOMED	84305006	Endometriosis of the parametrium
Endometriosis Adenomyosis	SNOMED	906431000006100	[RFC] Endometriosis
Endometriosis Adenomyosis	SNOMED	9563009	Endometriosis of the rectum
Epilepsy	ICD10	F803	Acquired aphasia with epilepsy [Landau-Kleffner]
Epilepsy	ICD10	G400	Localization-related (focal)(partial) idiopathic epilepsy and epileptic syndromes with seizures of localized onset
Epilepsy	ICD10	G401	Localization-related (focal)(partial) symptomatic epilepsy and epileptic syndromes with simple partial seizures
Epilepsy	ICD10	G402	Epilepsy
Epilepsy	ICD10	G403	Generalized idiopathic epilepsy and epileptic syndromes
Epilepsy	ICD10	G404	Other generalized epilepsy and epileptic syndromes
Epilepsy	ICD10	G405	Special epileptic syndromes
Epilepsy	ICD10	G406	Epilepsy
Epilepsy	ICD10	G407	Epilepsy
Epilepsy	ICD10	G408	Other epilepsy
Epilepsy	ICD10	G409	Epilepsy, unspecified
Epilepsy	ICD10	G410	Grand mal status epilepticus
Epilepsy	ICD10	G411	Petit mal status epilepticus
Epilepsy	ICD10	G412	Complex partial status epilepticus
Epilepsy	ICD10	G418	Other Status epilepticus
Epilepsy	ICD10	G419	Status epilepticus, unspecified
Epilepsy	SNOMED	108211000000104	Epilepsy impairs education
Epilepsy	SNOMED	111479008	[X]Epileptic psychosis NOS
Epilepsy	SNOMED	13973009	Grand mal status
Epilepsy	SNOMED	161480008	H/O: epilepsy
Epilepsy	SNOMED	1673671000006100	Epilepsy treatment continued
Epilepsy	SNOMED	170702005	Epilepsy monitoring
Epilepsy	SNOMED	170706008	Epilepsy associated problems
Epilepsy	SNOMED	170710006	Epilepsy treatment changed
Epilepsy	SNOMED	170711005	Epilepsy treatment started
Epilepsy	SNOMED	170712003	Epilepsy treatment stopped
Epilepsy	SNOMED	1855741000006105	Epilepsy monitoring in primary care
Epilepsy	SNOMED	1856761000006104	Epilepsy treatment compliance
Epilepsy	SNOMED	1856771000006106	Epilepsy treatment compliance good
Epilepsy	SNOMED	1856781000006109	Epilepsy treatment compliance poor
Epilepsy	SNOMED	1856791000006107	Epilepsy monitoring default
Epilepsy	SNOMED	192979009	Generalized nonconvulsive epilepsy
Epilepsy	SNOMED	192981006	Epileptic seizures - atonic
Epilepsy	SNOMED	192982004	Epileptic seizures - akinetic
Epilepsy	SNOMED	192990004	Neonatal myoclonic epilepsy
Epilepsy	SNOMED	192991000	Epileptic seizures - clonic
Epilepsy	SNOMED	192992007	Epileptic seizures - myoclonic

Epilepsy	SNOMED	192993002	Epileptic seizures - tonic
Epilepsy	SNOMED	192999003	Partial epilepsy with impairment of consciousness
Epilepsy	SNOMED	193000002	Temporal lobe epilepsy
Epilepsy	SNOMED	193002005	Psychosensory epilepsy
Epilepsy	SNOMED	193003000	Limbic system epilepsy
Epilepsy	SNOMED	193004006	Epileptic automatism
Epilepsy	SNOMED	193008009	Somatosensory epilepsy
Epilepsy	SNOMED	193009001	Partial epilepsy with autonomic symptoms
Epilepsy	SNOMED	193010006	Visual reflex epilepsy
Epilepsy	SNOMED	193011005	Unilateral epilepsy
Epilepsy	SNOMED	193021002	Cursive (running) epilepsy
Epilepsy	SNOMED	193022009	Locl-rlt(foc)(part)idiop epilep&epilptic syn seiz locl onset
Epilepsy	SNOMED	19598007	Grand mal (major) epilepsy
Epilepsy	SNOMED	199451000000106	Simple partial epileptic seizure
Epilepsy	SNOMED	230381009	Partial epilepsy without impairment of consciousness
Epilepsy	SNOMED	230387008	Panayiotopoulos syndrome
Epilepsy	SNOMED	230413002	Juvenile absence epilepsy
Epilepsy	SNOMED	230418006	Lennox-Gastaut syndrome
Epilepsy	SNOMED	230423006	Baltic myoclonus epilepsy
Epilepsy	SNOMED	230429005	Ohtahara syndrome
Epilepsy	SNOMED	230437002	Dravet syndrome
Epilepsy	SNOMED	230438007	[X]Acquired aphasia with epilepsy [Landau - Kleffner]
Epilepsy	SNOMED	230441003	Drug-induced epilepsy
Epilepsy	SNOMED	230444006	Menstrual epilepsy
Epilepsy	SNOMED	230445007	Sleep-related epilepsy
Epilepsy	SNOMED	230456007	Status epilepticus
Epilepsy	SNOMED	230460005	Complex partial status epilepticus
Epilepsy	SNOMED	241006	Kojevnikov's epilepsy
Epilepsy	SNOMED	267581004	Progressive myoclonic epilepsy
Epilepsy	SNOMED	28055006	Lightning spasms
Epilepsy	SNOMED	307356008	Motor epilepsy
Epilepsy	SNOMED	307357004	Jacksonian, focal or motor epilepsy
Epilepsy	SNOMED	313307000	Fit (in known epileptic) NOS
Epilepsy	SNOMED	314827004	Epilepsy control good
Epilepsy	SNOMED	314828009	Epilepsy control poor
Epilepsy	SNOMED	352818000	Tonic-clonic epilepsy
Epilepsy	SNOMED	361123003	Psychomotor epilepsy
Epilepsy	SNOMED	361268000	Alcohol-induced epilepsy
Epilepsy	SNOMED	36217008	[X]Limbic epilepsy personality
Epilepsy	SNOMED	395689002	Transient epileptic amnesia
Epilepsy	SNOMED	407623000	Emergency epilepsy treatment since last appointment
Epilepsy	SNOMED	407627004	Epilepsy confirmed
Epilepsy	SNOMED	407675009	Complex partial epileptic seizure
Epilepsy	SNOMED	413101007	Stress-induced epilepsy
Epilepsy	SNOMED	44145005	Temporal-central focal epilepsy
Epilepsy	SNOMED	50866000	Petit-mal epilepsy
Epilepsy	SNOMED	509341000000107	Epileptic absences
Epilepsy	SNOMED	5510009	[X]Schizophrenia-like psychosis in epilepsy
Epilepsy	SNOMED	6204001	Juvenile myoclonic epilepsy
Epilepsy	SNOMED	65120008	Generalised convulsive epilepsy NOS
Epilepsy	SNOMED	7033004	Epilepsia minoris continua
Epilepsy	SNOMED	719425009	SUDEP - sudden unexpected death in epilepsy
Epilepsy	SNOMED	721088003	Developmental delay, epilepsy, neonatal diabetes syndrome
Epilepsy	SNOMED	75023009	Traumatic epilepsy
Epilepsy	SNOMED	79631006	Absence seizure
Epilepsy	SNOMED	79745005	Sensory induced epilepsy
Epilepsy	SNOMED	810161000000101	Epilepsy monitoring in secondary care
Epilepsy	SNOMED	84757009	Epilepsy
Epilepsy	SNOMED	851121000006101	Partial epilepsy
Epilepsy	SNOMED	88491000000102	Epilepsy limits activities
Epilepsy	SNOMED	89525009	Gelastic epilepsy
Epilepsy	SNOMED	905881000006107	[RFC] Epilepsy/convulsion
Epilepsy	SNOMED	916571000000000	Epilepsy monitoring
Epilepsy	SNOMED	916571000006101	Epilepsy monitoring
Epilepsy	SNOMED	918561000000000	Epilepsy associated problems
Epilepsy	SNOMED	918561000006107	Epilepsy associated problems
Epilepsy	SNOMED	918601000006107	Nocturnal epilepsy
Epilepsy	SNOMED	93141000000105	Epilepsy restricts employment
Epilepsy	SNOMED	93151000000108	Epilepsy prevents employment
Epilepsy	SNOMED	93211000000101	Epilepsy does not limit activities
Epilepsy	SNOMED	95208000	Photosensitive epilepsy
Epilepsy	SNOMED	968661000000102	At risk of sudden unexpected death in epilepsy
Gout	ICD10	M10	Gout
Gout	ICD10	M140	Gouty arthropathy due to enzyme defects and other inherited disorders
Gout	SNOMED	1010591000006106	Adverse reaction to Drugs Used In Gout

Gout	SNOMED	1033961000000104	Gouty tophi of toe
Gout	SNOMED	14763005	Gouty tophi of ear
Gout	SNOMED	161451004	H/O: gout
Gout	SNOMED	164208001	On examination - auricle of ear - tophi
Gout	SNOMED	170730005	Gout monitoring
Gout	SNOMED	170731009	Initial gout assessment
Gout	SNOMED	170732002	Follow-up gout assessment
Gout	SNOMED	170733007	Joints gout affected
Gout	SNOMED	170735000	Date gout treatment started
Gout	SNOMED	170736004	Date of last gout attack
Gout	SNOMED	170737008	Gout associated problems
Gout	SNOMED	170738003	Gout drug side effects
Gout	SNOMED	170739006	Gout treatment changed
Gout	SNOMED	170740008	Date gout treatment stopped
Gout	SNOMED	1877501000006106	Keele CONTACT (treatment for acute gout) study
Gout	SNOMED	190828008	Gouty arthropathy
Gout	SNOMED	190829000	Gouty nephropathy
Gout	SNOMED	190842000	Gouty tophus of hand
Gout	SNOMED	201663006	Gouty arthritis of the shoulder region
Gout	SNOMED	201666003	Gouty arthritis of the hand
Gout	SNOMED	201667007	Gouty arthritis of the pelvic region and thigh
Gout	SNOMED	201669005	Gouty arthritis of the ankle and foot
Gout	SNOMED	201670006	Gouty arthritis of multiple sites
Gout	SNOMED	21811000033102	Drugs Used In Gout
Gout	SNOMED	239843003	[X]Other secondary gout
Gout	SNOMED	239844009	Gout secondary to renal impairment
Gout	SNOMED	239845005	Gout secondary to drug
Gout	SNOMED	239846006	Lead-induced gout
Gout	SNOMED	24595009	Idiopathic gout
Gout	SNOMED	27277001	Gouty tophi of heart
Gout	SNOMED	292671009	Drug for the treatment of gout adverse reaction
Gout	SNOMED	3875003	Urate tophus
Gout	SNOMED	402469004	Gouty tophi of other sites
Gout	SNOMED	428839004	Arthritis of toe due to gout
Gout	SNOMED	43193009	Gouty iritis
Gout	SNOMED	48440001	Gouty arthritis
Gout	SNOMED	646651000000108	Gout Uncomplicated
Gout	SNOMED	69896004	Rheumatic gout
Gout	SNOMED	850991000006106	Gouty tophi + gout NOS
Gout	SNOMED	90560007	Gout
Gout	SNOMED	924311000000106	Acute exacerbation of gout
Gout	SNOMED	9386003	Gouty neuritis
Hearing Loss	ICD10	H90	Conductive and sensorineural hearing loss
Hearing Loss	ICD10	H91	Other hearing loss
Hearing Loss	ICD10	Z453	Adjustment and management of implanted hearing device
Hearing Loss	ICD10	Z461	Fitting and adjustment of hearing aid
Hearing Loss	ICD10	Z974	Presence of external hearing-aid
Hearing Loss	SNOMED	134318003	Using lip-reading
Hearing Loss	SNOMED	134441005	Using British sign language, function
Hearing Loss	SNOMED	1706521000006107	Using American sign language
Hearing Loss	SNOMED	1706531000006105	Using Australian sign language
Hearing Loss	SNOMED	172732009	Implantation of intracochlear prosthesis
Hearing Loss	SNOMED	172733004	Implantation of cochlear implant, extracochlear
Hearing Loss	SNOMED	1778371000006105	Preferred communication Language: British Signing Language
Hearing Loss	SNOMED	1778641000006107	Preferred communication Language: Makaton (sign language)
Hearing Loss	SNOMED	183123004	Provide head worn hearing aid
Hearing Loss	SNOMED	183124005	Provide body worn hearing aid
Hearing Loss	SNOMED	183125006	Ear fitting hearing aid
Hearing Loss	SNOMED	183126007	Changing hearing aid battery
Hearing Loss	SNOMED	183281008	Lip reading training
Hearing Loss	SNOMED	1960331000006108	Hearing aid lost
Hearing Loss	SNOMED	1960341000006103	Hearing aid faulty
Hearing Loss	SNOMED	1960351000006101	Hearing aid left ear - serial number
Hearing Loss	SNOMED	1960361000006104	Hearing aid right ear - serial number
Hearing Loss	SNOMED	1962671000006104	Hearing aid mould requested
Hearing Loss	SNOMED	1964421000006109	Provision of transcutaneous air-conduction hearing aid tube
Hearing Loss	SNOMED	198061000000105	Uses hearing loop
Hearing Loss	SNOMED	204331000000107	Interpreter needed - British Sign Language
Hearing Loss	SNOMED	204341000000103	Interpreter needed - Makaton Sign Language
Hearing Loss	SNOMED	224625007	Interpreter for British sign language
Hearing Loss	SNOMED	225791000000104	Attention to fixtures for bone anchored hearing prosthesis
Hearing Loss	SNOMED	225861000000101	Insertion fixtures bone anchored hearing prosthesis Stage 1
Hearing Loss	SNOMED	225871000000108	Reduction soft tissue for bone anchored hearing prosthesis
Hearing Loss	SNOMED	225951000000109	Insertion fixtures bone anchored hearing prosthesis Stage 2
Hearing Loss	SNOMED	232183005	BAHA - Insertion of anchors for percutaneous bone anchored hearing aid

Hearing Loss	SNOMED	265316002	Attention to cochlear prosthesis
Hearing Loss	SNOMED	280651000000104	One stage insert fixtures bone anchored hearing prosthesis
Hearing Loss	SNOMED	281656009	Hearing aid provision
Hearing Loss	SNOMED	285055002	Does use hearing aid
Hearing Loss	SNOMED	290036002	Hearing aid care
Hearing Loss	SNOMED	312629000	Provision of support as Makaton user
Hearing Loss	SNOMED	312630005	Provision of support as signing user
Hearing Loss	SNOMED	3561000119106	Severe hearing loss
Hearing Loss	SNOMED	390967001	Hearing aid problem
Hearing Loss	SNOMED	394889008	Hearing aid requested
Hearing Loss	SNOMED	404903008	Uses sign language
Hearing Loss	SNOMED	408577006	Using Makaton sign language
Hearing Loss	SNOMED	411511000000107	[V]Fitting or adjustment of hearing aid
Hearing Loss	SNOMED	416121000000101	[V]Adjustment and management of implanted hearing device
Hearing Loss	SNOMED	426647008	Fitting external hearing prosthesis bone anchored fixtures
Hearing Loss	SNOMED	426706000	Removal of hearing implant from external ear
Hearing Loss	SNOMED	426733001	Attachment of bone anchored hearing prosthesis
Hearing Loss	SNOMED	427556004	Removal of hearing implant from middle ear
Hearing Loss	SNOMED	428004000	Placement of hearing implant in middle ear
Hearing Loss	SNOMED	428619000	Attention to hearing implant in external ear
Hearing Loss	SNOMED	429383008	Attention to hearing implant in middle ear
Hearing Loss	SNOMED	429585000	Placement of hearing implant in external ear
Hearing Loss	SNOMED	445488003	Uses telecommunications device for the deaf
Hearing Loss	SNOMED	446499008	Provision of replacement hearing aid
Hearing Loss	SNOMED	449840001	Cochlear prosthesis in situ
Hearing Loss	SNOMED	520421000000106	Preferred method of communication: Makaton
Hearing Loss	SNOMED	520471000000105	Preferred method of communication: British Sign Language
Hearing Loss	SNOMED	6012004	Hearing aid, device
Hearing Loss	SNOMED	61947007	Deaf mutism, NEC
Hearing Loss	SNOMED	694231000000100	Deaf mutism, NEC
Hearing Loss	SNOMED	700454004	Profound sensorineural hearing loss
Hearing Loss	SNOMED	704497002	Uses British sign language interpreter
Hearing Loss	SNOMED	710576002	Removal of cochlear prosthesis
Hearing Loss	SNOMED	737049003	Severe acquired hearing loss
Hearing Loss	SNOMED	737050003	Profound acquired hearing loss
Hearing Loss	SNOMED	761591000000105	[V]Bone anchored hearing aid in situ
Hearing Loss	SNOMED	855871000006106	Hearing adequate with aid
Hearing Loss	SNOMED	860811000000106	Severe sensorineural hearing loss
Hearing Loss	SNOMED	877191000000104	Bilateral profound sensorineural hearing loss
Hearing Loss	SNOMED	936641000000108	Requires information in Makaton
Hearing Loss	SNOMED	945711000000107	Visual frame sign language interpreter needed
Hearing Loss	SNOMED	945731000000104	Hands-on signing interpreter needed
Hearing Loss	SNOMED	987421000006102	Uses hearing loop
Hearing Loss	SNOMED	993811000006103	Sign interpreter needed
Hearing Loss	SNOMED	993821000006106	Sign interpreter needed - British sign language
Hearing Loss	SNOMED	993831000006109	Sign interpreter needed - Makaton sign language
Heart Failure	ICD10	I50	Heart failure
Heart Failure	SNOMED	10335000	Chronic right-sided heart failure (disorder)
Heart Failure	SNOMED	10633002	Acute congestive heart failure
Heart Failure	SNOMED	128404006	Right heart failure
Heart Failure	SNOMED	134401001	Left ventricular systolic dysfunction
Heart Failure	SNOMED	134440006	Referral to heart failure clinic
Heart Failure	SNOMED	195111005	Decompensated cardiac failure
Heart Failure	SNOMED	195112003	Compensated cardiac failure
Heart Failure	SNOMED	195114002	Acute left ventricular failure
Heart Failure	SNOMED	206586007	Congenital cardiac failure
Heart Failure	SNOMED	233924009	Heart failure as a complication of care (disorder)
Heart Failure	SNOMED	275514001	Impaired left ventricular function
Heart Failure	SNOMED	314206003	Refractory heart failure (disorder)
Heart Failure	SNOMED	367363000	Right ventricular failure
Heart Failure	SNOMED	407596008	Echocardiogram shows left ventricular systolic dysfunction (finding)
Heart Failure	SNOMED	420300004	New York Heart Association Classification - Class I (finding)
Heart Failure	SNOMED	420913000	New York Heart Association Classification - Class III (finding)
Heart Failure	SNOMED	421704003	New York Heart Association Classification - Class II (finding)
Heart Failure	SNOMED	422293003	New York Heart Association Classification - Class IV (finding)
Heart Failure	SNOMED	42343007	Congestive heart failure
Heart Failure	SNOMED	426263006	Congestive heart failure due to left ventricular systolic dysfunction (disorder)
Heart Failure	SNOMED	430396006	Chronic systolic dysfunction of left ventricle (disorder)
Heart Failure	SNOMED	43736008	Rheumatic left ventricular failure
Heart Failure	SNOMED	446221000	Heart failure with normal ejection fraction (disorder)
Heart Failure	SNOMED	48447003	Chronic heart failure (disorder)
Heart Failure	SNOMED	56675007	Acute heart failure
Heart Failure	SNOMED	698592004	Asymptomatic left ventricular systolic dysfunction (disorder)
Heart Failure	SNOMED	703272007	Heart failure with reduced ejection fraction (disorder)
Heart Failure	SNOMED	717491000000102	Excepted from heart failure quality indicators - informed dissent (finding)

Heart Failure	SNOMED	71892000	Cardiac asthma
Heart Failure	SNOMED	760361000000100	Fast track heart failure referral for transthoracic two dimensional echocardiogram
Heart Failure	SNOMED	79955004	Chronic cor pulmonale
Heart Failure	SNOMED	84114007	Heart failure
Heart Failure	SNOMED	85232009	Left heart failure
Heart Failure	SNOMED	87837008	Chronic pulmonary heart disease
Heart Failure	SNOMED	88805009	Chronic congestive heart failure
Heart Failure	SNOMED	92506005	Biventricular congestive heart failure
Hemi/Para/Quadriplegia	ICD10	G041	Tropical spastic paraplegia
Hemi/Para/Quadriplegia	ICD10	G800	Spastic quadriplegic cerebral palsy
Hemi/Para/Quadriplegia	ICD10	G802	Spastic hemiplegic cerebral palsy
Hemi/Para/Quadriplegia	ICD10	G81	Hemiplegia
Hemi/Para/Quadriplegia	ICD10	G82	Paraplegia and tetraplegia
Hemi/Para/Quadriplegia	SNOMED	11538006	Quadriplegia
Hemi/Para/Quadriplegia	SNOMED	1593000	Postnatal infantile hemiplegia
Hemi/Para/Quadriplegia	SNOMED	163601006	O/E - hemiplegia
Hemi/Para/Quadriplegia	SNOMED	163604003	On examination - paraplegia
Hemi/Para/Quadriplegia	SNOMED	163605002	On examination - quadriplegia
Hemi/Para/Quadriplegia	SNOMED	192949002	Congenital paraplegia
Hemi/Para/Quadriplegia	SNOMED	192964002	Flaccid tetraplegia
Hemi/Para/Quadriplegia	SNOMED	192965001	Spastic quadriplegia
Hemi/Para/Quadriplegia	SNOMED	192966000	Flaccid paraplegia
Hemi/Para/Quadriplegia	SNOMED	192967009	Spastic paraplegia
Hemi/Para/Quadriplegia	SNOMED	275468009	Congenital quadriplegia
Hemi/Para/Quadriplegia	SNOMED	278284007	Right hemiplegia
Hemi/Para/Quadriplegia	SNOMED	278285008	Left hemiplegia
Hemi/Para/Quadriplegia	SNOMED	39912006	Hereditary spastic paraplegia
Hemi/Para/Quadriplegia	SNOMED	48721008	Spastic quadriplegic cerebral palsy
Hemi/Para/Quadriplegia	SNOMED	50582007	Hemiplegia NOS
Hemi/Para/Quadriplegia	SNOMED	52072009	Thermoplegia
Hemi/Para/Quadriplegia	SNOMED	60389000	Paraplegia
Hemi/Para/Quadriplegia	SNOMED	714279000	Tropical spastic paraplegia
Hemi/Para/Quadriplegia	SNOMED	79633009	Spastic hemiplegia
Hemi/Para/Quadriplegia	SNOMED	80935004	Flaccid hemiplegia
Hemi/Para/Quadriplegia	SNOMED	813921000000104	Spastic hemiplegic cerebral palsy
Hemi/Para/Quadriplegia	SNOMED	82725007	Massive muscular calcification associated with paraplegia
Hemi/Para/Quadriplegia	SNOMED	856091000000000	Right hemiplegia
Hemi/Para/Quadriplegia	SNOMED	856091000006105	Right hemiplegia
Hemi/Para/Quadriplegia	SNOMED	857181000000000	Left hemiplegia
Hemi/Para/Quadriplegia	SNOMED	857181000006106	Left hemiplegia
Hemi/Para/Quadriplegia	SNOMED	905891000006105	[RFC] Paraplegias
HIV/AIDS	ICD10	B20	Human immunodeficiency virus [HIV] disease resulting in infectious and parasitic diseases
HIV/AIDS	ICD10	B21	Human immunodeficiency virus [HIV] disease resulting in malignant neoplasms
HIV/AIDS	ICD10	B22	Human immunodeficiency virus [HIV] disease resulting in other specified diseases
HIV/AIDS	ICD10	B23	Human immunodeficiency virus [HIV] disease resulting in other conditions
HIV/AIDS	ICD10	B24	Unspecified human immunodeficiency virus [HIV] disease
HIV/AIDS	ICD10	F024	Dementia in human immunodeficiency virus [HIV] disease
HIV/AIDS	ICD10	O987	Human immunodeficiency virus [HIV] disease complicating pregnancy, childbirth and the puerperium
HIV/AIDS	ICD10	R75	Laboratory evidence of human immunodeficiency virus [HIV]
HIV/AIDS	ICD10	Z21	Asymptomatic human immunodeficiency virus [HIV] infection status
HIV/AIDS	SNOMED	1007761000000101	Human immunodeficiency virus type 1 subtype identification
HIV/AIDS	SNOMED	1008351000000102	HIV 1 nucleic acid detection
HIV/AIDS	SNOMED	111880001	Acute human immunodeficiency virus infection
HIV/AIDS	SNOMED	1573641000006103	H/O: HIV
HIV/AIDS	SNOMED	165816005	HIV positive
HIV/AIDS	SNOMED	1761311000006102	[SHHAPT] HIV positive
HIV/AIDS	SNOMED	1761351000006101	[SHHAPT] New HIV diagnosis
HIV/AIDS	SNOMED	1761361000006104	[SHHAPT] New HIV diagnosis - acute
HIV/AIDS	SNOMED	1761381000006109	[SHHAPT] New HIV diagnosis - late
HIV/AIDS	SNOMED	1762131000006101	[SHHAPT] New HIV diagnosis - diagnosed previously elsewhere
HIV/AIDS	SNOMED	1762141000006106	[SHHAPT] New HIV diagnosis - acute - diagnosed prev. elsewhere
HIV/AIDS	SNOMED	1762151000006108	[SHHAPT] New HIV diagnosis - late - diagnosed prev. elsewhere
HIV/AIDS	SNOMED	186706006	Human immunodeficiency virus with constitutional disease
HIV/AIDS	SNOMED	186707002	Human immunodeficiency virus infection with neurological disease
HIV/AIDS	SNOMED	186708007	[X]HIV disease resulting in other viral infections
HIV/AIDS	SNOMED	198609003	HIV disease complicating pregnancy childbirth puerperium
HIV/AIDS	SNOMED	363346000	Human immunodeficiency virus with secondary cancers
HIV/AIDS	SNOMED	420403001	HIV disease resulting in Pneumocystis carinii pneumonia
HIV/AIDS	SNOMED	420524008	Kaposi sarcoma associated with AIDS
HIV/AIDS	SNOMED	420818005	HIV disease resulting in mycobacterial infection
HIV/AIDS	SNOMED	421529006	Dementia associated with AIDS
HIV/AIDS	SNOMED	421671002	Pneumonia associated with acquired immunodeficiency syndrome

HIV/AIDS	SNOMED	422003001	Human immunodeficiency virus infection wasting syndrome
HIV/AIDS	SNOMED	427921000000108	[V]Asymptomatic human immunodeficiency virus infection status
HIV/AIDS	SNOMED	442537007	[X]HIV disease resulting in other non-Hodgkin's lymphoma
HIV/AIDS	SNOMED	444356002	Exposure to Human immunodeficiency virus
HIV/AIDS	SNOMED	474391000006106	AIDS - First Presentation
HIV/AIDS	SNOMED	474401000006108	AIDS - Subsequent Presentation
HIV/AIDS	SNOMED	496781000006107	Asymptomatic HIV Infection - First Presentation
HIV/AIDS	SNOMED	496791000006105	Asymptomatic HIV Infection - Subsequent Presentation
HIV/AIDS	SNOMED	543791000000108	Aids +viral/chlamydial dis.NOS
HIV/AIDS	SNOMED	62479008	Acquired immune deficiency syndrome
HIV/AIDS	SNOMED	699433000	AIDS carrier
HIV/AIDS	SNOMED	713572001	[X]HIV disease resulting in other malignant neoplasms
HIV/AIDS	SNOMED	713897006	Burkitt's lymphoma co-occurrent with human immunodeficiency virus infection
HIV/AIDS	SNOMED	81000119104	Human immunodef virus resulting in other disease
HIV/AIDS	SNOMED	825881000006105	HIV Infection With Symptoms, Not AIDS - First Presentation
HIV/AIDS	SNOMED	825891000006108	HIV Infection With Symptoms, Not AIDS - Subsequent Presentation
HIV/AIDS	SNOMED	854381000006102	HIV infection
HIV/AIDS	SNOMED	86406008	Human immunodeficiency virus with other clinical findings
HIV/AIDS	SNOMED	88415009	HIV disease resulting in mycobacterial infection
HIV/AIDS	SNOMED	906841000006108	[RFC] HIV/AIDS
HIV/AIDS	SNOMED	908851000006109	[RFC] AIDS/HIV
HIV/AIDS	SNOMED	91947003	Asymptomatic human immunodeficiency virus infection
HIV/AIDS	SNOMED	925451000000000	AIDS carrier
HIV/AIDS	SNOMED	925451000006109	AIDS carrier
HIV/AIDS	SNOMED	943041000000105	[X]HIV disease result/haematological+immunologic abnorms,NEC
HIV/AIDS	SNOMED	95892003	Human immunodeficiency virus infection with persistent generalized lymphadenopathy
Hypertension	ICD10	I10	Essential (primary) hypertension
Hypertension	ICD10	I11	Hypertensive heart disease
Hypertension	ICD10	I12	Hypertensive renal disease
Hypertension	ICD10	I13	Hypertensive heart and renal disease
Hypertension	ICD10	I15	Secondary hypertension
Hypertension	SNOMED	10725009	Benign hypertension
Hypertension	SNOMED	1201005	Benign essential hypertension
Hypertension	SNOMED	123799005	Renovascular hypertension
Hypertension	SNOMED	129151000119102	Chronic kidney disease stage 4 due to hypertension
Hypertension	SNOMED	129171000119106	Chronic kidney disease stage 3 due to hypertension
Hypertension	SNOMED	132721000119104	Hypertensive emergency
Hypertension	SNOMED	169465000	Hypertension induced by oral contraceptive pill
Hypertension	SNOMED	194767001	Benign hypertensive heart disease with congestive cardiac failure
Hypertension	SNOMED	194779001	Hypertensive heart and renal disease with (congestive) heart failure
Hypertension	SNOMED	194780003	Hypertensive heart and renal disease with renal failure
Hypertension	SNOMED	194781004	Hypertensive heart and renal disease with both (congestive) heart failure and renal failure
Hypertension	SNOMED	194783001	Secondary malignant renovascular hypertension
Hypertension	SNOMED	194785008	Secondary benign hypertension
Hypertension	SNOMED	194788005	Hypertension secondary to endocrine disorders
Hypertension	SNOMED	194791005	Hypertension secondary to drug
Hypertension	SNOMED	198942000	Benign essential hypertension complicating pregnancy, childbirth and the puerperium
Hypertension	SNOMED	198944004	Benign essential hypertension complicating pregnancy, childbirth and the puerperium - delivered
Hypertension	SNOMED	198945003	Benign essential hypertension complicating pregnancy, childbirth and the puerperium - delivered with postnatal complication
Hypertension	SNOMED	198946002	Benign essential hypertension complicating pregnancy, childbirth and the puerperium - not delivered
Hypertension	SNOMED	198947006	Benign essential hypertension complicating pregnancy, childbirth and the puerperium with postnatal complication
Hypertension	SNOMED	198949009	Renal hypertension complicating pregnancy, childbirth and the puerperium
Hypertension	SNOMED	198951008	Renal hypertension complicating pregnancy, childbirth and the puerperium - delivered
Hypertension	SNOMED	198952001	Renal hypertension complicating pregnancy, childbirth and the puerperium - delivered with postnatal complication
Hypertension	SNOMED	198953006	Renal hypertension complicating pregnancy, childbirth and the puerperium - not delivered
Hypertension	SNOMED	198954000	Renal hypertension complicating pregnancy, childbirth and the puerperium with postnatal complication
Hypertension	SNOMED	198997005	Pre-eclampsia or eclampsia with pre-existing hypertension
Hypertension	SNOMED	198999008	Pre-eclampsia or eclampsia with pre-existing hypertension - delivered
Hypertension	SNOMED	199000005	Pre-eclampsia or eclampsia with pre-existing hypertension - delivered with postnatal complication
Hypertension	SNOMED	199002002	Pre-eclampsia or eclampsia with pre-existing hypertension - not delivered
Hypertension	SNOMED	199003007	Pre-eclampsia or eclampsia with pre-existing hypertension with postnatal complication

Hypertension	SNOMED	199005000	Pre-existing hypertension complicating pregnancy, childbirth and puerperium
Hypertension	SNOMED	199006004	Pre-existing hypertensive heart disease complicating pregnancy, childbirth and the puerperium
Hypertension	SNOMED	199007008	Pre-existing hypertensive heart and renal disease complicating pregnancy, childbirth and the puerperium
Hypertension	SNOMED	199008003	Pre-existing secondary hypertension complicating pregnancy, childbirth and puerperium
Hypertension	SNOMED	275516004	Cardiomegaly - hypertensive
Hypertension	SNOMED	31992008	Secondary hypertension
Hypertension	SNOMED	36221001	Benign hypertensive heart disease
Hypertension	SNOMED	36315003	Malignant hypertensive heart disease without congestive heart failure
Hypertension	SNOMED	371125006	Labile essential hypertension (disorder)
Hypertension	SNOMED	38341003	Hypertensive disease
Hypertension	SNOMED	401047000	Hypertension treatment refused (context-dependent category)
Hypertension	SNOMED	407567007	Patient on maximal tolerated antihypertensive therapy (finding)
Hypertension	SNOMED	428163005	Hypertensive left ventricular hypertrophy (disorder)
Hypertension	SNOMED	443402002	Lifestyle education regarding hypertension (procedure)
Hypertension	SNOMED	443482000	Hypertensive urgency (disorder)
Hypertension	SNOMED	471521000000108	[X]Hypertensive diseases (disorder)
Hypertension	SNOMED	48146000	Diastolic hypertension
Hypertension	SNOMED	5148006	Hypertensive heart disease with congestive heart failure
Hypertension	SNOMED	54225002	Malignant hypertensive heart disease
Hypertension	SNOMED	56218007	Systolic hypertension
Hypertension	SNOMED	59621000	Essential hypertension
Hypertension	SNOMED	64715009	Hypertensive heart disease
Hypertension	SNOMED	66052004	Benign hypertensive heart AND renal disease
Hypertension	SNOMED	66610008	Malignant hypertensive heart AND renal disease
Hypertension	SNOMED	67359005	Pre-eclampsia added to pre-existing hypertension
Hypertension	SNOMED	70272006	Malignant hypertension
Hypertension	SNOMED	706882009	Hypertensive crisis (disorder)
Hypertension	SNOMED	712832005	Supine hypertension
Hypertension	SNOMED	73410007	Benign secondary renovascular hypertension
Hypertension	SNOMED	762463000	Diastolic hypertension co-occurrent with systolic hypertension (disorder)
Hypertension	SNOMED	77970009	Benign hypertensive heart disease without congestive heart failure
Hypertension	SNOMED	78975002	Malignant essential hypertension
Hypertension	SNOMED	83105008	Malignant hypertensive heart disease with congestive heart failure
Hypertension	SNOMED	843821000000102	Stage 1 hypertension (National Institute for Health and Clinical Excellence 2011) (disorder)
Hypertension	SNOMED	843841000000109	Severe hypertension (National Institute for Health and Clinical Excellence 2011) (disorder)
Hypertension	SNOMED	845891000000103	Hypertension resistant to drug therapy (disorder)
Hypertension	SNOMED	846371000000103	Stage 2 hypertension (National Institute for Health and Clinical Excellence 2011) (disorder)
Hypertension	SNOMED	86041002	Pre-existing hypertension in obstetric context
Hypertension	SNOMED	86234004	Hypertensive heart AND renal disease
Hypertension	SNOMED	863191000000102	Nocturnal hypertension (disorder)
Hypertension	SNOMED	8762007	Chronic hypertension in obstetric context
Hypertension	SNOMED	89242004	Malignant secondary hypertension
Hypertension	SNOMED	908631000000108	Stage 1 hypertension (National Institute for Health and Clinical Excellence 2011) without evidence of end organ damage (disorder)
Hypertension	SNOMED	908651000000101	Stage 1 hypertension (National Institute for Health and Clinical Excellence 2011) with evidence of end organ damage (disorder)
Inflammatory Bowel Disease	ICD10	K50	Crohn disease [regional enteritis]
Inflammatory Bowel Disease	ICD10	K51	Ulcerative colitis
Inflammatory Bowel Disease	ICD10	M074	Arthropathy in Crohn disease [regional enteritis]
Inflammatory Bowel Disease	ICD10	M075	Arthropathy in ulcerative colitis
Inflammatory Bowel Disease	ICD10	M091	Juvenile arthritis in Crohn disease [regional enteritis]
Inflammatory Bowel Disease	ICD10	M092	Juvenile arthritis in ulcerative colitis
Inflammatory Bowel Disease	SNOMED	196578009	Orofacial Crohn's disease
Inflammatory Bowel Disease	SNOMED	196977009	Crohn's disease of the terminal ileum
Inflammatory Bowel Disease	SNOMED	201727001	Arthropathy in ulcerative colitis
Inflammatory Bowel Disease	SNOMED	201728006	Arthropathy in Crohn's disease
Inflammatory Bowel Disease	SNOMED	201805000	Juvenile arthritis in Crohn's disease
Inflammatory Bowel Disease	SNOMED	201807008	Juvenile arthritis in ulcerative colitis
Inflammatory Bowel Disease	SNOMED	235714007	Ulcerative (chronic) enterocolitis
Inflammatory Bowel Disease	SNOMED	275549008	H/O: ulcerative colitis
Inflammatory Bowel Disease	SNOMED	295046003	Ulcerative proctocolitis NOS
Inflammatory Bowel Disease	SNOMED	34000006	Crohn's disease
Inflammatory Bowel Disease	SNOMED	38106008	Crohn disease of ileum
Inflammatory Bowel Disease	SNOMED	3815005	Crohn's proctitis
Inflammatory Bowel Disease	SNOMED	414153008	Exacerbation of Crohn's disease of large intestine
Inflammatory Bowel Disease	SNOMED	414154002	Exacerbation of Crohn disease of small intestine
Inflammatory Bowel Disease	SNOMED	414156000	Exacerbation of ulcerative colitis
Inflammatory Bowel Disease	SNOMED	444548001	Ulcerative pancolitis
Inflammatory Bowel Disease	SNOMED	50440006	Crohn's colitis

Inflammatory Bowel Disease	SNOMED	52231000	Ulcerative proctitis
Inflammatory Bowel Disease	SNOMED	52506002	Chronic ulcerative rectosigmoiditis
Inflammatory Bowel Disease	SNOMED	56287005	Crohn disease of duodenum
Inflammatory Bowel Disease	SNOMED	56689002	Crohn's disease of the small bowel NOS
Inflammatory Bowel Disease	SNOMED	64766004	Ulcerative colitis
Inflammatory Bowel Disease	SNOMED	7620006	Crohn disease of large bowel
Inflammatory Bowel Disease	SNOMED	906051000006102	[RFC] Crohns disease
Inflammatory Bowel Disease	SNOMED	906191000006109	[RFC] Ulcerative colitis
Liver Disease	ICD10	K701	Alcoholic hepatitis
Liver Disease	ICD10	K702	Alcoholic fibrosis and sclerosis of liver
Liver Disease	ICD10	K703	Alcoholic cirrhosis of liver
Liver Disease	ICD10	K717	Toxic liver disease with fibrosis and cirrhosis of liver
Liver Disease	ICD10	K740	Hepatic fibrosis
Liver Disease	ICD10	K741	Hepatic sclerosis
Liver Disease	ICD10	K742	Hepatic fibrosis with hepatic sclerosis
Liver Disease	ICD10	K744	Secondary biliary cirrhosis
Liver Disease	ICD10	K745	Biliary cirrhosis, unspecified
Liver Disease	ICD10	K746	Other and unspecified cirrhosis of liver
Liver Disease	SNOMED	10295004	[X]Other chronic viral hepatitis
Liver Disease	SNOMED	123604002	Toxic cirrhosis
Liver Disease	SNOMED	12368000	Secondary biliary cirrhosis
Liver Disease	SNOMED	128302006	Chronic viral hepatitis C
Liver Disease	SNOMED	1287007	Congenital absence of bile duct
Liver Disease	SNOMED	14223005	Oesophageal varices without bleeding
Liver Disease	SNOMED	15999000	Mixed portal cirrhosis
Liver Disease	SNOMED	173617001	Open operation on oesophageal varices NOS
Liver Disease	SNOMED	173621008	Open injection sclerotherapy to oesophageal varices
Liver Disease	SNOMED	173639004	Fibreoptic oesophagoscopy & injection sclerotherapy varices
Liver Disease	SNOMED	173641003	Fibreoptic oesophagoscopy and banding of oesophageal varices
Liver Disease	SNOMED	173660005	Rigid oesophagoscopy and injection sclerotherapy of varices
Liver Disease	SNOMED	173661009	Rigid oesophagoscopy and banding of oesophageal varices
Liver Disease	SNOMED	173679008	Balloon tamponade of oesophagus
Liver Disease	SNOMED	174425003	Orthotopic liver transplant
Liver Disease	SNOMED	174426002	Heterotopic liver transplant
Liver Disease	SNOMED	174427006	Replacement of previous liver transplant
Liver Disease	SNOMED	1761006	Biliary cirrhosis NOS
Liver Disease	SNOMED	17709002	Bleeding oesophageal varices
Liver Disease	SNOMED	18027006	Transplantation of liver NOS
Liver Disease	SNOMED	186628001	Viral hepatitis C with coma
Liver Disease	SNOMED	186639003	Chronic viral hepatitis B without delta-agent
Liver Disease	SNOMED	195474004	Oesophageal varices in diseases EC
Liver Disease	SNOMED	195475003	Oesophageal varices with bleeding in diseases EC
Liver Disease	SNOMED	195476002	Oesophageal varices without bleeding in diseases EC
Liver Disease	SNOMED	197270009	Acute hepatic failure
Liver Disease	SNOMED	197275004	Subacute hepatic failure
Liver Disease	SNOMED	197279005	Cirrhosis and chronic liver disease
Liver Disease	SNOMED	197284004	Chronic hepatitis (& [NOS]/[active B]/[active]/[persistent])
Liver Disease	SNOMED	197286002	Recurrent hepatitis
Liver Disease	SNOMED	197291001	Unilobular portal cirrhosis
Liver Disease	SNOMED	197293003	Diffuse nodular cirrhosis
Liver Disease	SNOMED	197294009	Fatty portal cirrhosis
Liver Disease	SNOMED	197296006	Capsular portal cirrhosis
Liver Disease	SNOMED	197299004	Pigmentary portal cirrhosis
Liver Disease	SNOMED	197300007	Pipestem portal cirrhosis
Liver Disease	SNOMED	197301006	Toxic portal cirrhosis
Liver Disease	SNOMED	197303009	Bacterial portal cirrhosis
Liver Disease	SNOMED	197305002	Syphilitic portal cirrhosis
Liver Disease	SNOMED	197310003	Biliary cirrhosis of children
Liver Disease	SNOMED	197316009	Portal fibrosis without cirrhosis
Liver Disease	SNOMED	197324004	Liver abscess and sequelae of chronic liver disease
Liver Disease	SNOMED	197359004	Toxic liver disease with chronic persistent hepatitis
Liver Disease	SNOMED	197360009	Toxic liver disease with chronic lobular hepatitis
Liver Disease	SNOMED	197361008	Toxic liver disease with chronic active hepatitis
Liver Disease	SNOMED	197362001	Toxic liver disease with fibrosis and cirrhosis of liver
Liver Disease	SNOMED	197368002	Hepatic granulomas in sarcoidosis
Liver Disease	SNOMED	197441003	Primary sclerosing cholangitis
Liver Disease	SNOMED	19943007	Cirrhosis of liver NOS
Liver Disease	SNOMED	20415001	Progressive neuronal degeneration with liver cirrhosis
Liver Disease	SNOMED	204781002	Congenital absence of hepatic ducts
Liver Disease	SNOMED	204782009	Atresia of hepatic ducts
Liver Disease	SNOMED	213153001	Liver transplant failure and rejection
Liver Disease	SNOMED	213230009	Hepatic failure as a complication of care
Liver Disease	SNOMED	213231008	Hepatorenal syndrome as a complication of care
Liver Disease	SNOMED	21861000	Micronodular cirrhosis
Liver Disease	SNOMED	226941000000100	Transplantation of liver cells

Liver Disease	SNOMED	235212007	Balln tamponade oesophagus using Sengstaken-Blakemore tube
Liver Disease	SNOMED	235213002	Balloon tamponade of oesophagus using Minnesota tube
Liver Disease	SNOMED	235458006	Exploration of liver transplant
Liver Disease	SNOMED	235869004	Chronic viral hepatitis B with hepatitis D
Liver Disease	SNOMED	235870003	Chronic non-A non-B hepatitis
Liver Disease	SNOMED	235875008	Alcoholic hepatitis
Liver Disease	SNOMED	235880004	Alcoholic fibrosis and sclerosis of liver
Liver Disease	SNOMED	235881000	Alcoholic hepatic failure
Liver Disease	SNOMED	235884008	Fulminant hepatic failure
Liver Disease	SNOMED	235885009	Subfulminant hepatic failure
Liver Disease	SNOMED	235886005	Chronic hepatic failure
Liver Disease	SNOMED	235889003	Drug-induced chronic hepatitis
Liver Disease	SNOMED	235895002	Laennec's cirrhosis, non-alcoholic
Liver Disease	SNOMED	235896001	Infectious cirrhosis
Liver Disease	SNOMED	235897005	Hypoxia-associated cirrhosis
Liver Disease	SNOMED	235898000	Pericellular fibrosis of congenital syphilis
Liver Disease	SNOMED	235899008	Hepatic sclerosis
Liver Disease	SNOMED	235900003	Portal and splenic vein sclerosis
Liver Disease	SNOMED	235901004	Hepatic fibrosis with hepatic sclerosis
Liver Disease	SNOMED	235902006	Intrahepatic phlebosclerosis and fibrosis
Liver Disease	SNOMED	235910007	Liver transplant disorder
Liver Disease	SNOMED	235911006	Liver transplant rejection
Liver Disease	SNOMED	235912004	Liver transplant failure
Liver Disease	SNOMED	235916001	Ichthyosis congenita with biliary atresia
Liver Disease	SNOMED	253807009	Atresia of bile duct (& [intrahepatic] or [extrahepatic]
Liver Disease	SNOMED	265437009	Exploration liver (& transplanted)
Liver Disease	SNOMED	266468003	Cirrhosis - non-alcoholic
Liver Disease	SNOMED	266469006	Multilobular portal cirrhosis
Liver Disease	SNOMED	266470007	Cirrhosis: [cardiac portal] or [congestive]
Liver Disease	SNOMED	266471006	Cirrhosis: [juv portal] or [childh funct] or [Indian childh]
Liver Disease	SNOMED	271440004	Cirrhosis secondary to cholestasis
Liver Disease	SNOMED	28670008	Oesophageal varices NOS
Liver Disease	SNOMED	307757001	Chronic alcoholic hepatitis
Liver Disease	SNOMED	308129003	Oesophageal varices in cirrhosis of the liver
Liver Disease	SNOMED	309783001	Oesophageal varices in alcoholic cirrhosis of the liver
Liver Disease	SNOMED	31712002	Primary biliary cirrhosis
Liver Disease	SNOMED	31742004	Alagille syndrome
Liver Disease	SNOMED	328383001	Chronic liver disease NOS
Liver Disease	SNOMED	34736002	Chronic passive congestion of liver
Liver Disease	SNOMED	34742003	Portal hypertension
Liver Disease	SNOMED	362421000000102	Chronic hepatitis annual review
Liver Disease	SNOMED	3738000	[X]Other chronic viral hepatitis
Liver Disease	SNOMED	400381000000101	[V]Assessment for liver transplant
Liver Disease	SNOMED	408335007	Autoimmune hepatitis
Liver Disease	SNOMED	40946000	[X]Unspecified viral hepatitis with coma
Liver Disease	SNOMED	41309000	Alcoholic liver disease
Liver Disease	SNOMED	413438002	Acute hepatic failure due to drugs
Liver Disease	SNOMED	417371000000102	[X]Oesophageal varices in diseases classified elsewhere
Liver Disease	SNOMED	41889008	Chronic persistent hepatitis
Liver Disease	SNOMED	419531000000100	[X]Other chronic viral hepatitis
Liver Disease	SNOMED	419541000000109	[X]Chronic viral hepatitis, unspecified
Liver Disease	SNOMED	419728003	(Cirrhosis - non alcoholic) or (portal cirrhosis)
Liver Disease	SNOMED	420054005	Alcoholic cirrhosis of liver
Liver Disease	SNOMED	426356008	Orthotopic transplantation of whole liver
Liver Disease	SNOMED	426841006	Fibreopt endoscop rubber band ligation of upper GIT varices
Liver Disease	SNOMED	427022004	Liver disease due to cystic fibrosis
Liver Disease	SNOMED	428198008	Transplantation of liver cells
Liver Disease	SNOMED	43904005	Macronodular cirrhosis
Liver Disease	SNOMED	444071000000104	[X]Unspecified viral hepatitis with coma
Liver Disease	SNOMED	464381000000109	[V]Liver transplanted
Liver Disease	SNOMED	470971000000107	[X]Other and unspecified cirrhosis of liver
Liver Disease	SNOMED	50325005	Alcoholic fatty liver
Liver Disease	SNOMED	51292008	Hepatorenal syndrome
Liver Disease	SNOMED	57339008	Chronic lobular hepatitis
Liver Disease	SNOMED	59927004	Hepatic failure
Liver Disease	SNOMED	6183001	Cirrhosis: [juv portal] or [childh funct] or [Indian childh]
Liver Disease	SNOMED	61977001	Chronic viral hepatitis B
Liver Disease	SNOMED	62484002	(Liver cirrhos: [named vars] or [NOS]) or (hepat fibrosis)
Liver Disease	SNOMED	67364009	Local ligation of oesophageal varices
Liver Disease	SNOMED	68094008	Congenital hypoplasia of bile duct
Liver Disease	SNOMED	737297006	[V]Liver transplanted
Liver Disease	SNOMED	74669004	Cirrhosis: [cardiac portal] or [congestive]
Liver Disease	SNOMED	76301009	Florid cirrhosis
Liver Disease	SNOMED	76783007	Chronic hepatitis
Liver Disease	SNOMED	773411000000107	Compensation for liver failure NOS

Liver Disease	SNOMED	77480004	Atresia of bile ducts
Liver Disease	SNOMED	776981000000103	Cystic fibrosis related cirrhosis
Liver Disease	SNOMED	79607001	Congenital hepatic fibrosis
Liver Disease	SNOMED	79720007	Other non-alcoholic chronic liver disease
Liver Disease	SNOMED	81675001	Chronic yellow atrophy
Liver Disease	SNOMED	82821008	Extrahepatic biliary atresia
Liver Disease	SNOMED	89580002	Cryptogenic cirrhosis
Liver Disease	SNOMED	91109007	Gastric varices
Liver Disease	SNOMED	9953008	Acute alcoholic hepatitis
Long Covid	ICD10	U099	Post COVID-19 condition, unspecified
Long Covid	SNOMED	1119304009	Chronic post-COVID-19 syndrome
Long Covid	SNOMED	1325021000000106	Signposting to Your COVID Recovery
Long Covid	SNOMED	1325031000000108	Referral to post-COVID assessment clinic
Long Covid	SNOMED	1325041000000104	Referral to Your COVID Recovery rehabilitation platform
Long Covid	SNOMED	1325051000000101	Newcastle post-COVID syndrome Follow-up Screening Questionnaire
Long Covid	SNOMED	1325061000000103	Assessment using Newcastle post-COVID syndrome Follow-up Screening Questionnaire
Long Covid	SNOMED	1325071000000105	C19-YRS (COVID-19 Yorkshire Rehabilitation Screening) tool
Long Covid	SNOMED	1325081000000107	Assessment using C19-YRS (COVID-19 Yorkshire Rehabilitation Screening) tool
Long Covid	SNOMED	1325091000000109	PCFS (Post-COVID-19 Functional Status) Scale patient self-report
Long Covid	SNOMED	1325101000000101	Assessment using PCFS (Post-COVID-19 Functional Status) Scale patient self-report
Long Covid	SNOMED	1325121000000105	PCFS (Post-COVID-19 Functional Status) Scale patient self-report final scale grade
Long Covid	SNOMED	1325131000000107	PCFS (Post-COVID-19 Functional Status) Scale structured interview final scale grade
Long Covid	SNOMED	1325141000000103	Assessment using PCFS (Post-COVID-19 Functional Status) Scale structured interview
Long Covid	SNOMED	1325151000000100	PCFS (Post-COVID-19 Functional Status) Scale structured interview
Long Covid	SNOMED	1325161000000102	Post-COVID-19 syndrome
Long Covid	SNOMED	1325181000000106	Ongoing symptomatic COVID-19
Long Covid	SNOMED	1326351000000108	Post-COVID-19 syndrome resolved
Meniere's Disease	ICD10	H810	Ménière disease
Meniere's Disease	SNOMED	13445001	Unspecified Meniere's disease
Meniere's Disease	SNOMED	194348002	Active cochleovestibular Meniere's disease
Meniere's Disease	SNOMED	194349005	Active cochlear Meniere's disease
Meniere's Disease	SNOMED	194350005	Active vestibular Meniere's disease
Meniere's Disease	SNOMED	266994001	H/O: vertigo/Meniere's disease
Meniere's Disease	SNOMED	275542004	H/O: Meniere's disease
Meniere's Disease	SNOMED	29904009	Lermoyez's syndrome
Meniere's Disease	SNOMED	906701000006103	[RFC] Menieres disease
Meniere's Disease	SNOMED	9794007	Endolymphatic hydrops
Multiple Sclerosis	ICD10	G350	Multiple sclerosis
Multiple Sclerosis	SNOMED	166411000000100	Spec serv for pat with multiple sclerosis - enh serv admin
Multiple Sclerosis	SNOMED	1747820000000000	Multiple sclerosis monitoring administration
Multiple Sclerosis	SNOMED	1747821000006101	Multiple sclerosis monitoring administration
Multiple Sclerosis	SNOMED	1747830000000000	Multiple sclerosis monitoring first letter
Multiple Sclerosis	SNOMED	1747831000006103	Multiple sclerosis monitoring first letter
Multiple Sclerosis	SNOMED	1747840000000000	Multiple sclerosis monitoring second letter
Multiple Sclerosis	SNOMED	1747841000006108	Multiple sclerosis monitoring second letter
Multiple Sclerosis	SNOMED	1747850000000000	Multiple sclerosis monitoring third letter
Multiple Sclerosis	SNOMED	1747851000006105	Multiple sclerosis monitoring third letter
Multiple Sclerosis	SNOMED	1747861000006107	Multiple sclerosis monitoring telephone invite
Multiple Sclerosis	SNOMED	192926004	Multiple sclerosis of the brain stem
Multiple Sclerosis	SNOMED	192927008	Multiple sclerosis of the spinal cord
Multiple Sclerosis	SNOMED	192928003	Generalized multiple sclerosis
Multiple Sclerosis	SNOMED	192929006	Multiple sclerosis (MS) exacerbation
Multiple Sclerosis	SNOMED	198491000000106	Multiple sclerosis review
Multiple Sclerosis	SNOMED	205631000000104	Multiple sclerosis multidisciplinary review
Multiple Sclerosis	SNOMED	24700007	Multiple sclerosis
Multiple Sclerosis	SNOMED	367561000000107	Management of multiple sclerosis in onset phase
Multiple Sclerosis	SNOMED	367591000000101	Management of multiple sclerosis in early disease phase
Multiple Sclerosis	SNOMED	367621000000103	Management of multiple sclerosis in stable disability phase
Multiple Sclerosis	SNOMED	367681000000102	Management of multiple sclerosis in palliative phase
Multiple Sclerosis	SNOMED	425500002	Secondary progressive multiple sclerosis
Multiple Sclerosis	SNOMED	426373005	Relapsing and remitting multiple sclerosis
Multiple Sclerosis	SNOMED	428700003	Primary progressive multiple sclerosis
Multiple Sclerosis	SNOMED	438511000	Benign multiple sclerosis
Multiple Sclerosis	SNOMED	439567002	Malignant multiple sclerosis
Multiple Sclerosis	SNOMED	515931000000104	Multiple sclerosis review declined
Multiple Sclerosis	SNOMED	758981000000000	Multiple sclerosis monitoring administration
Multiple Sclerosis	SNOMED	758981000000100	Multiple sclerosis monitoring administration
Multiple Sclerosis	SNOMED	759051000000000	Multiple sclerosis monitoring first letter
Multiple Sclerosis	SNOMED	759051000000102	Multiple sclerosis monitoring first letter
Multiple Sclerosis	SNOMED	759091000000000	Multiple sclerosis monitoring second letter

Multiple Sclerosis	SNOMED	759091000000105	Multiple sclerosis monitoring second letter
Multiple Sclerosis	SNOMED	759131000000108	Multiple sclerosis monitoring telephone invitation
Multiple Sclerosis	SNOMED	759171000000000	Multiple sclerosis monitoring third letter
Multiple Sclerosis	SNOMED	759171000000105	Multiple sclerosis monitoring third letter
Multiple Sclerosis	SNOMED	811881000000109	Multiple sclerosis care plan agreed
Multiple Sclerosis	SNOMED	905781000000000	[RFC] Multiple sclerosis
Multiple Sclerosis	SNOMED	905781000006102	[RFC] Multiple sclerosis
Multiple Sclerosis	SNOMED	908811000000000	[RFC] Multiple sclerosis
Multiple Sclerosis	SNOMED	908811000006108	[RFC] Multiple sclerosis
Multiple Sclerosis	SNOMED	983261000006103	Multiple sclerosis - personal health plan
Multiple Sclerosis	SNOMED	983271000006105	Multiple sclerosis - multidisciplinary review
Multiple Sclerosis	SNOMED	983281000006108	Multiple sclerosis - review
Multiple Sclerosis	SNOMED	983291000006106	Multiple sclerosis - primary progressive
Multiple Sclerosis	SNOMED	983301000006107	Multiple sclerosis - secondary progressive
Multiple Sclerosis	SNOMED	983311000006105	Multiple sclerosis - relapsing remitting
Musculoskeletal Injury	ICD10	M484	Fatigue fracture of vertebra
Musculoskeletal Injury	ICD10	S120	Fracture of first cervical vertebra
Musculoskeletal Injury	ICD10	S121	Fracture of second cervical vertebra
Musculoskeletal Injury	ICD10	S122	Fracture of other specified cervical vertebra
Musculoskeletal Injury	ICD10	S127	Multiple fractures of cervical spine
Musculoskeletal Injury	ICD10	S22	Fracture of rib(s), sternum and thoracic spine
Musculoskeletal Injury	ICD10	S32	Fracture of lumbar spine and pelvis
Musculoskeletal Injury	ICD10	S422	Fracture of upper end of humerus
Musculoskeletal Injury	ICD10	S423	Fracture of shaft of humerus
Musculoskeletal Injury	ICD10	S424	Fracture of lower end of humerus
Musculoskeletal Injury	ICD10	S427	Multiple fractures of clavicle, scapula and humerus
Musculoskeletal Injury	ICD10	S52	Fracture of forearm
Musculoskeletal Injury	ICD10	S62	Fracture at wrist and hand level
Musculoskeletal Injury	ICD10	S720	Fracture of neck of femur
Musculoskeletal Injury	ICD10	S721	Pertrochanteric fracture
Musculoskeletal Injury	ICD10	S722	Subtrochanteric fracture
Musculoskeletal Injury	ICD10	T08	Fracture of spine, level unspecified
Musculoskeletal Injury	ICD10	T911	Sequelae of fracture of spine
Musculoskeletal Injury	ICD10	T922	Sequelae of fracture at wrist and hand level
Musculoskeletal Injury	SNOMED	111622009	C3 vertebra closed fracture without spinal cord lesion
Musculoskeletal Injury	SNOMED	111640008	Closed fracture of radius (alone), unspecified
Musculoskeletal Injury	SNOMED	111641007	Open fracture radial neck
Musculoskeletal Injury	SNOMED	11782000	Closed fracture of axis without spinal cord lesion
Musculoskeletal Injury	SNOMED	11964008	Primary int fxn(no red) prox fem #+screw/nail device alone
Musculoskeletal Injury	SNOMED	125606003	[X]Fracture of other specified cervical vertebra
Musculoskeletal Injury	SNOMED	125607007	Fracture of thoracic spine
Musculoskeletal Injury	SNOMED	125608002	Fracture of lumbar vertebra
Musculoskeletal Injury	SNOMED	125871005	Fracture of coccyx
Musculoskeletal Injury	SNOMED	125872003	Fracture of sacrum
Musculoskeletal Injury	SNOMED	12676007	Open fracture of radius (alone), unspecified
Musculoskeletal Injury	SNOMED	127286005	Fracture of upper end of humerus
Musculoskeletal Injury	SNOMED	12746007	Open fracture proximal femur, transcervical
Musculoskeletal Injury	SNOMED	1705000	Closed fracture of cervicotrochanteric section of femur
Musculoskeletal Injury	SNOMED	17222009	Closed fracture distal radius, intra-articular, other type
Musculoskeletal Injury	SNOMED	1734006	Fracture of vertebra with spinal cord lesion
Musculoskeletal Injury	SNOMED	178674000	Decompression of fracture of spine
Musculoskeletal Injury	SNOMED	178675004	Complex decompression of fracture of spine
Musculoskeletal Injury	SNOMED	178676003	Anterior decompression of fracture of spine
Musculoskeletal Injury	SNOMED	178677007	Posterior decompression of fracture of spine
Musculoskeletal Injury	SNOMED	178682000	Open reduction of fracture of spine & excis facet of spine
Musculoskeletal Injury	SNOMED	178684004	Manipulative reduction of fracture of spine
Musculoskeletal Injury	SNOMED	178685003	Spinal extension traction for fracture of spine
Musculoskeletal Injury	SNOMED	178686002	Halo skull traction for fracture of spine
Musculoskeletal Injury	SNOMED	178688001	Primary bedrest stabilization of spinal fracture
Musculoskeletal Injury	SNOMED	178689009	Primary collar stabilisation of spinal fracture
Musculoskeletal Injury	SNOMED	178690000	Primary skull traction stabilisation of spinal fracture
Musculoskeletal Injury	SNOMED	178691001	Primary cast stabilisation of spinal fracture
Musculoskeletal Injury	SNOMED	178692008	Primary external fixation stabilization of spinal fracture
Musculoskeletal Injury	SNOMED	178694009	Revision to bedrest stabilization of spinal fracture
Musculoskeletal Injury	SNOMED	178695005	Revision to collar stabilisation of spinal fracture
Musculoskeletal Injury	SNOMED	178696006	Revision to skull traction stabilisation of spinal fracture
Musculoskeletal Injury	SNOMED	178697002	Revision to cast stabilisation of spinal fracture
Musculoskeletal Injury	SNOMED	178698007	Revision to external fixation stabilisation spinal fracture
Musculoskeletal Injury	SNOMED	178701004	Primary cls reduction spinal fracture+collar stabilisation
Musculoskeletal Injury	SNOMED	178702006	Primary cls reduction spinal fracture+bedrest stabilisation
Musculoskeletal Injury	SNOMED	178703001	Primary closed reduction spinal fracture and skull traction stabilization
Musculoskeletal Injury	SNOMED	178704007	Primary cls reduction spinal fracture+cast stabilisation
Musculoskeletal Injury	SNOMED	178705008	Primary cls reduction spinal fracture+external fixation
Musculoskeletal Injury	SNOMED	178708005	Revision cls reduction spinal fracture+collar stabilisation
Musculoskeletal Injury	SNOMED	178709002	Revision cls reduc spinal fracture+bedrest stabilisation

Musculoskeletal Injury	SNOMED	178710007	Revision to closed reduction spinal fracture and skull traction stabilization
Musculoskeletal Injury	SNOMED	178711006	Revision cls reduc spinal fracture+cast stabilisation
Musculoskeletal Injury	SNOMED	178712004	Revision cls reduc spinal fracture+external fixation
Musculoskeletal Injury	SNOMED	178715002	Primary opn reduction spinal fracture+collar stabilisation
Musculoskeletal Injury	SNOMED	178716001	Primary opn reduction spinal fracture+bedrest stabilisation
Musculoskeletal Injury	SNOMED	178717005	Primary opn reduc spinal fracture+skull traction stabilisatn
Musculoskeletal Injury	SNOMED	178718000	Primary opn reduction spinal fracture+cast stabilisation
Musculoskeletal Injury	SNOMED	178719008	Primary open reduction spinal fracture and external fixation
Musculoskeletal Injury	SNOMED	178722005	Revision to open reduction spinal fracture and collar stabilisation
Musculoskeletal Injury	SNOMED	178723000	Rvsn open reduc spinal fracture+bedrest stabilisation
Musculoskeletal Injury	SNOMED	178724006	Rvsn open reduc spinal fracture+skull traction stabilisation
Musculoskeletal Injury	SNOMED	178725007	Rvsn open reduc spinal fracture+cast stabilisation
Musculoskeletal Injury	SNOMED	178726008	Rvsn open reduc spinal fracture+external fix
Musculoskeletal Injury	SNOMED	178733008	Primary open reduc spinal fracture+internal fix+plate
Musculoskeletal Injury	SNOMED	178734002	Fixation of fracture of spine using Harrington rod
Musculoskeletal Injury	SNOMED	178736000	Primary open reduc spinal fracture+internal fix+wire
Musculoskeletal Injury	SNOMED	178737009	Primary open reduc spinal fracture+internal fix+rod system
Musculoskeletal Injury	SNOMED	178738004	Primary open reduc spinal #+intern fix+segmental wire system
Musculoskeletal Injury	SNOMED	178739007	Primary open reduc spinal #+internal fix+internal fixator
Musculoskeletal Injury	SNOMED	178741008	Rvsn open reduc spinal fracture+internal fix+wire
Musculoskeletal Injury	SNOMED	178742001	Rvsn open reduc spinal fracture+internal fix+plate
Musculoskeletal Injury	SNOMED	178743006	Rvsn open reduc spinal fracture+internal fix+rod system
Musculoskeletal Injury	SNOMED	178744000	Rvsn open reduc spinal #+internal fix+segmental wire system
Musculoskeletal Injury	SNOMED	178745004	Rvsn open reduc spinal fracture+internal fix+internl fixator
Musculoskeletal Injury	SNOMED	179018001	Prmy open red+int fxn prox fem #+screw/nail+intramed device
Musculoskeletal Injury	SNOMED	179064006	Revision to open red+ext fxtn of proximal femoral #
Musculoskeletal Injury	SNOMED	179066008	Rvsn to opn red+int fxtn prox fem #+ scrw/nl+intramed device
Musculoskeletal Injury	SNOMED	179067004	Rvsn to opn red+int fxtn prox fem #+ scrw/nail+plate device
Musculoskeletal Injury	SNOMED	179097007	Primary int fxn(no red) prox fem #+scrw/nail+intramed device
Musculoskeletal Injury	SNOMED	179098002	Primary int fxn(no red) prox fem #+screw/nail+plate device
Musculoskeletal Injury	SNOMED	179100002	Rvsn to int fxn(no red) prox fem #+screw/nail+intramed dev
Musculoskeletal Injury	SNOMED	179102005	Revisn to int fxn(no red) prox fem #+screw/nail device alone
Musculoskeletal Injury	SNOMED	179104006	Prim cls rd+int fxn prox fem #+screw/nail+intramdulry device
Musculoskeletal Injury	SNOMED	179105007	Primary cls red+int fxn prox fem #+screw/nail+plate device
Musculoskeletal Injury	SNOMED	179106008	Rvsn to cls red+int fxn prox fem #+screw/nail device alone
Musculoskeletal Injury	SNOMED	179107004	Rvsn cls red+int fxn prox fem #+screw/nail+intramed device
Musculoskeletal Injury	SNOMED	179108009	Rvsn to cls red+int fxn prox fem #+screw/nail+plate device
Musculoskeletal Injury	SNOMED	179136004	Primary external fixation(without reduction) prox femoral #
Musculoskeletal Injury	SNOMED	179137008	Revision to ext fxn(without reduction) proximal femoral #
Musculoskeletal Injury	SNOMED	179138003	Primary cls reduction+external fixation proximal femoral #
Musculoskeletal Injury	SNOMED	179139006	Revision to closed reduction+ext fxn proximal femoral #
Musculoskeletal Injury	SNOMED	179159005	Closed reduction of fracture of hip
Musculoskeletal Injury	SNOMED	179172006	Closed reduction of fracture of radius and/or ulna
Musculoskeletal Injury	SNOMED	19688005	Open reduction of spinal fracture
Musculoskeletal Injury	SNOMED	198741000000106	Closed fracture of femur, upper epiphysis
Musculoskeletal Injury	SNOMED	198751000000109	Open fracture of femur, upper epiphysis
Musculoskeletal Injury	SNOMED	1992361000006101	VTE risk assessment - hip fracture
Musculoskeletal Injury	SNOMED	20100009	Closed fracture of intracapsular section of femur
Musculoskeletal Injury	SNOMED	202831001	Stress fracture of vertebra
Musculoskeletal Injury	SNOMED	203497002	Fracture malunion - upper arm
Musculoskeletal Injury	SNOMED	207895002	Closed fracture atlas, isolated arch or articular process
Musculoskeletal Injury	SNOMED	207896001	Closed fracture atlas, comminuted
Musculoskeletal Injury	SNOMED	207897005	Closed fracture axis, odontoid process
Musculoskeletal Injury	SNOMED	207898000	Closed fracture axis, spondylolysis
Musculoskeletal Injury	SNOMED	207899008	Closed fracture axis, spinous process
Musculoskeletal Injury	SNOMED	207900003	Closed fracture axis, transverse process
Musculoskeletal Injury	SNOMED	207901004	Closed fracture axis, posterior arch
Musculoskeletal Injury	SNOMED	207902006	Closed fracture axis, tricolumnar
Musculoskeletal Injury	SNOMED	207903001	Closed fracture cervical vertebra, burst
Musculoskeletal Injury	SNOMED	207904007	Closed fracture cervical vertebra, wedge
Musculoskeletal Injury	SNOMED	207905008	Closed fracture cervical vertebra, spondylolysis
Musculoskeletal Injury	SNOMED	207906009	Closed fracture cervical vertebra, spinous process
Musculoskeletal Injury	SNOMED	207907000	Closed fracture cervical vertebra, transverse process
Musculoskeletal Injury	SNOMED	207908005	Closed fracture cervical vertebra, posterior arch
Musculoskeletal Injury	SNOMED	207909002	Closed fracture cervical vertebra, tricolumnar
Musculoskeletal Injury	SNOMED	207910007	Multiple closed fractures of cervical vertebrae
Musculoskeletal Injury	SNOMED	207925007	Open fracture axis, spinous process
Musculoskeletal Injury	SNOMED	207929001	Open fracture cervical vertebra, burst
Musculoskeletal Injury	SNOMED	207930006	Open fracture cervical vertebra, wedge
Musculoskeletal Injury	SNOMED	207931005	Open fracture cervical vertebra, spondylolysis
Musculoskeletal Injury	SNOMED	207932003	Open fracture cervical vertebra, spinous process
Musculoskeletal Injury	SNOMED	207933008	Open fracture cervical vertebra, transverse process
Musculoskeletal Injury	SNOMED	207934002	Open fracture cervical vertebra, posterior arch
Musculoskeletal Injury	SNOMED	207935001	Open fracture cervical vertebra, tricolumnar
Musculoskeletal Injury	SNOMED	207936000	Multiple open fractures of cervical vertebrae

Musculoskeletal Injury	SNOMED	207938004	#Thoracic spine-no cord lesion
Musculoskeletal Injury	SNOMED	207939007	Closed fracture thoracic vertebra, burst
Musculoskeletal Injury	SNOMED	207940009	Closed fracture thoracic vertebra, wedge
Musculoskeletal Injury	SNOMED	207941008	Closed fracture thoracic vertebra, spondylolysis
Musculoskeletal Injury	SNOMED	207942001	Closed fracture thoracic vertebra, spinous process
Musculoskeletal Injury	SNOMED	207943006	Closed fracture thoracic vertebra, transverse process
Musculoskeletal Injury	SNOMED	207944000	Closed fracture thoracic vertebra, posterior arch
Musculoskeletal Injury	SNOMED	207945004	Closed fracture thoracic vertebra, tricolumnar
Musculoskeletal Injury	SNOMED	207949005	Open fracture thoracic vertebra
Musculoskeletal Injury	SNOMED	207950005	Open fracture thoracic vertebra, burst
Musculoskeletal Injury	SNOMED	207951009	Open fracture thoracic vertebra, wedge
Musculoskeletal Injury	SNOMED	207952002	Open fracture thoracic vertebra, spondylolysis
Musculoskeletal Injury	SNOMED	207953007	Open fracture thoracic vertebra, spinous process
Musculoskeletal Injury	SNOMED	207954001	Open fracture thoracic vertebra, transverse process
Musculoskeletal Injury	SNOMED	207955000	Open fracture thoracic vertebra, posterior arch
Musculoskeletal Injury	SNOMED	207956004	Open fracture thoracic vertebra, tricolumnar
Musculoskeletal Injury	SNOMED	207957008	#Lumbar spine - no cord lesion
Musculoskeletal Injury	SNOMED	207958003	Closed fracture lumbar vertebra, burst
Musculoskeletal Injury	SNOMED	207959006	Closed fracture lumbar vertebra, wedge
Musculoskeletal Injury	SNOMED	207960001	Closed fracture lumbar vertebra, spondylolysis
Musculoskeletal Injury	SNOMED	207961002	Closed fracture lumbar vertebra, spinous process
Musculoskeletal Injury	SNOMED	207962009	Closed fracture lumbar vertebra, transverse process
Musculoskeletal Injury	SNOMED	207963004	Closed fracture lumbar vertebra, posterior arch
Musculoskeletal Injury	SNOMED	207964005	Closed fracture lumbar vertebra, tricolumnar
Musculoskeletal Injury	SNOMED	207965006	Open fracture lumbar vertebra
Musculoskeletal Injury	SNOMED	207966007	Open fracture lumbar vertebra, burst
Musculoskeletal Injury	SNOMED	207967003	Open fracture lumbar vertebra, wedge
Musculoskeletal Injury	SNOMED	207968008	Open fracture lumbar vertebra, spondylolysis
Musculoskeletal Injury	SNOMED	207969000	Open fracture lumbar vertebra, spinous process
Musculoskeletal Injury	SNOMED	207970004	Open fracture lumbar vertebra, transverse process
Musculoskeletal Injury	SNOMED	207971000	Open fracture lumbar vertebra, posterior arch
Musculoskeletal Injury	SNOMED	207972007	Open fracture lumbar vertebra, tricolumnar
Musculoskeletal Injury	SNOMED	207974008	#Sacrum/coccyx-no cord lesion
Musculoskeletal Injury	SNOMED	207975009	Closed compression fracture sacrum
Musculoskeletal Injury	SNOMED	207976005	Closed vertical fracture of sacrum
Musculoskeletal Injury	SNOMED	207977001	Open fracture sacrum
Musculoskeletal Injury	SNOMED	207978006	Open compression fracture sacrum
Musculoskeletal Injury	SNOMED	207979003	Open vertical fracture of sacrum
Musculoskeletal Injury	SNOMED	207983003	Fracture of first cervical vertebra
Musculoskeletal Injury	SNOMED	207984009	Fracture of second cervical vertebra
Musculoskeletal Injury	SNOMED	207985005	Multiple fractures of cervical spine
Musculoskeletal Injury	SNOMED	207986006	Fracture of lumbar spine and pelvis
Musculoskeletal Injury	SNOMED	207993005	Multiple fractures of lumbar spine and/or pelvis
Musculoskeletal Injury	SNOMED	207998001	Closed fracture of cervical spine with cord lesion
Musculoskeletal Injury	SNOMED	208000003	Cls spinal fracture with complete cervcl cord lesion, C1-4
Musculoskeletal Injury	SNOMED	208001004	Cls spinal fracture with anterior cervcl cord lesion, C1-4
Musculoskeletal Injury	SNOMED	208002006	Closed spinal fracture with central cervical cord lesion, C1-4
Musculoskeletal Injury	SNOMED	208003001	Cls spinal fracture with posterior cervcl cord lesion, C1-4
Musculoskeletal Injury	SNOMED	208006009	Cls spinal fracture with complete cervcl cord lesion, C5-7
Musculoskeletal Injury	SNOMED	208007000	Cls spinal fracture with anterior cervcl cord lesion, C5-7
Musculoskeletal Injury	SNOMED	208008005	Cls spinal fracture with central cervical cord lesion, C5-7
Musculoskeletal Injury	SNOMED	208009002	Cls spinal fracture with posterior cervcl cord lesion, C5-7
Musculoskeletal Injury	SNOMED	208012004	Open fracture of cervical spine with spinal cord lesion
Musculoskeletal Injury	SNOMED	208014003	Opn spinal fracture with complete cervcl cord lesion, C1-4
Musculoskeletal Injury	SNOMED	208015002	Opn spinal fracture with anterior cervcl cord lesion, C1-4
Musculoskeletal Injury	SNOMED	208016001	Open spinal fracture with central cervical cord lesion, C1-4
Musculoskeletal Injury	SNOMED	208017005	Opn spinal fracture with posterior cervcl cord lesion, C1-4
Musculoskeletal Injury	SNOMED	208020002	Opn spinal fracture with complete cervcl cord lesion, C5-7
Musculoskeletal Injury	SNOMED	208021003	Opn spinal fracture with anterior cervcl cord lesion, C5-7
Musculoskeletal Injury	SNOMED	208022005	Open spinal fracture with central cervical cord lesion, C5-7
Musculoskeletal Injury	SNOMED	208023000	Opn spinal fracture with posterior cervcl cord lesion, C5-7
Musculoskeletal Injury	SNOMED	208026008	Closed fracture of thoracic spine with cord lesion NOS
Musculoskeletal Injury	SNOMED	208028009	Cls spinal fracture wth complete thoracic cord lesion,T1-6
Musculoskeletal Injury	SNOMED	208029001	Cls spinal fracture wth anterior thoracic cord lesion,T1-6
Musculoskeletal Injury	SNOMED	208030006	Cls spinal fracture with central thoracic cord lesion, T1-6
Musculoskeletal Injury	SNOMED	208031005	Cls spinal fracture with posterior thorac cord lesion, T1-6
Musculoskeletal Injury	SNOMED	208034002	Cls spinal fracture with complete thorac cord lesion, T7-12
Musculoskeletal Injury	SNOMED	208035001	Cls spinal fracture with anterior thorac cord lesion, T7-12
Musculoskeletal Injury	SNOMED	208036000	Cls spinal fracture with central thoracid cord lesion, T7-12
Musculoskeletal Injury	SNOMED	208037009	Cls spinal fracture with posterior thorac cord lesion, T7-12
Musculoskeletal Injury	SNOMED	208040009	Open fracture of thoracic spine with spinal cord lesion
Musculoskeletal Injury	SNOMED	208042001	Opn spinal fracture with complete thorac cord lesion, T1-6
Musculoskeletal Injury	SNOMED	208043006	Opn spinal fracture with anterior thorac cord lesion, T1-6
Musculoskeletal Injury	SNOMED	208044000	Open spinal fracture with central thoracic cord lesion, T1-6
Musculoskeletal Injury	SNOMED	208045004	Opn spinal fracture with posterior thorac cord lesion, T1-6

Musculoskeletal Injury	SNOMED	208048002	Opn spinal fracture with complete thorac cord lesion, T7-12
Musculoskeletal Injury	SNOMED	208049005	Opn spinal fracture with anterior thorac cord lesion, T7-12
Musculoskeletal Injury	SNOMED	208050005	Op spinal fracture with central thoracic cord lesion, T7-12
Musculoskeletal Injury	SNOMED	208051009	Opn spinal fracture with posterior thorac cord lesion, T7-12
Musculoskeletal Injury	SNOMED	208054001	Closed fracture of lumbar spine with spinal cord lesion
Musculoskeletal Injury	SNOMED	208056004	Closed spinal fracture with complete lumbar cord lesion
Musculoskeletal Injury	SNOMED	208057008	Closed spinal fracture with anterior lumbar cord lesion
Musculoskeletal Injury	SNOMED	208058003	Closed spinal fracture with central lumbar cord lesion
Musculoskeletal Injury	SNOMED	208059006	Closed spinal fracture with posterior lumbar cord lesion
Musculoskeletal Injury	SNOMED	208060001	Closed spinal fracture with cauda equina lesion
Musculoskeletal Injury	SNOMED	208061002	Open fracture of lumbar spine with spinal cord lesion
Musculoskeletal Injury	SNOMED	208063004	Open spinal fracture with complete lumbar cord lesion
Musculoskeletal Injury	SNOMED	208064005	Open spinal fracture with anterior lumbar cord lesion
Musculoskeletal Injury	SNOMED	208065006	Open spinal fracture with central lumbar cord lesion
Musculoskeletal Injury	SNOMED	208066007	Open spinal fracture with posterior lumbar cord lesion
Musculoskeletal Injury	SNOMED	208067003	Open spinal fracture with cauda equina lesion
Musculoskeletal Injury	SNOMED	208069000	#Sacrum/coccyx + cord lesion
Musculoskeletal Injury	SNOMED	208071000	Closed fracture of sacrum with complete cauda equina lesion
Musculoskeletal Injury	SNOMED	208076005	Open fracture of sacrum with unspecified spinal cord lesion
Musculoskeletal Injury	SNOMED	208078006	Open fracture of sacrum with complete cauda equina lesion
Musculoskeletal Injury	SNOMED	208082008	Closed fracture of coccyx with other cauda equina injury
Musculoskeletal Injury	SNOMED	208084009	Closed fracture of coccyx with complete cauda equina lesion
Musculoskeletal Injury	SNOMED	208088007	Open fracture of coccyx with spinal cord lesion
Musculoskeletal Injury	SNOMED	208090008	Open fracture of coccyx with complete cauda equina lesion
Musculoskeletal Injury	SNOMED	208146001	Closed fracture acetabulum, anterior lip alone
Musculoskeletal Injury	SNOMED	208147005	Closed fracture acetabulum, posterior lip alone
Musculoskeletal Injury	SNOMED	208148000	Closed fracture acetabulum, anterior column
Musculoskeletal Injury	SNOMED	208149008	Closed fracture acetabulum, posterior column
Musculoskeletal Injury	SNOMED	208150008	Closed fracture acetabulum, floor
Musculoskeletal Injury	SNOMED	208151007	Closed fracture acetabulum, double column transverse
Musculoskeletal Injury	SNOMED	208155003	Open fracture acetabulum, anterior lip alone
Musculoskeletal Injury	SNOMED	208156002	Open fracture acetabulum, posterior lip alone
Musculoskeletal Injury	SNOMED	208157006	Open fracture acetabulum, anterior column
Musculoskeletal Injury	SNOMED	208158001	Open fracture acetabulum, posterior column
Musculoskeletal Injury	SNOMED	208159009	Open fracture acetabulum, floor
Musculoskeletal Injury	SNOMED	208160004	Open fracture acetabulum, double column transverse
Musculoskeletal Injury	SNOMED	208186009	Closed fracture dislocation of sacro-iliac joint
Musculoskeletal Injury	SNOMED	208198004	Open fracture dislocation of sacro-iliac joint
Musculoskeletal Injury	SNOMED	208215006	Multiple fractures of thoracic spine
Musculoskeletal Injury	SNOMED	208228004	Closed fracture scapula, spine
Musculoskeletal Injury	SNOMED	208235007	Open fracture scapula, spine
Musculoskeletal Injury	SNOMED	208240004	Closed fracture proximal humerus, neck
Musculoskeletal Injury	SNOMED	208241000	Closed fracture of anatomical neck of humerus
Musculoskeletal Injury	SNOMED	208242007	Closed fracture proximal humerus, greater tuberosity
Musculoskeletal Injury	SNOMED	208244008	Closed fracture of humerus, upper epiphysis
Musculoskeletal Injury	SNOMED	208245009	Closed fracture proximal humerus, three part
Musculoskeletal Injury	SNOMED	208246005	Closed fracture proximal humerus, four part
Musculoskeletal Injury	SNOMED	208250003	Open fracture proximal humerus, neck
Musculoskeletal Injury	SNOMED	208251004	Open fracture of proximal humerus, anatomical neck
Musculoskeletal Injury	SNOMED	208253001	Open fracture proximal humerus, greater tuberosity
Musculoskeletal Injury	SNOMED	208254007	Open fracture proximal humerus, head
Musculoskeletal Injury	SNOMED	208255008	Open fracture of humerus, upper epiphysis
Musculoskeletal Injury	SNOMED	208256009	Open fracture proximal humerus, three part
Musculoskeletal Injury	SNOMED	208257000	Open fracture proximal humerus, four part
Musculoskeletal Injury	SNOMED	208267005	Closed fracture distal humerus, lateral condyle
Musculoskeletal Injury	SNOMED	208270009	Closed fracture of distal humerus, trochlea
Musculoskeletal Injury	SNOMED	208271008	Closed fracture distal humerus, lateral epicondyle
Musculoskeletal Injury	SNOMED	208272001	Closed fracture distal humerus, capitellum
Musculoskeletal Injury	SNOMED	208273006	Closed fracture distal humerus, bicondylar (T-Y fracture)
Musculoskeletal Injury	SNOMED	208274000	Closed fracture of distal humerus, multiple
Musculoskeletal Injury	SNOMED	208281007	Open fracture of distal humerus, trochlea
Musculoskeletal Injury	SNOMED	208283005	Open fracture distal humerus, lateral epicondyle
Musculoskeletal Injury	SNOMED	208284004	Open fracture distal humerus, capitellum
Musculoskeletal Injury	SNOMED	208285003	Open fracture distal humerus, bicondylar (T-Y fracture)
Musculoskeletal Injury	SNOMED	208286002	Open fracture of distal humerus, multiple
Musculoskeletal Injury	SNOMED	208309008	Closed fracture radius and ulna, middle
Musculoskeletal Injury	SNOMED	208312006	Open fracture radius and ulna, middle
Musculoskeletal Injury	SNOMED	208318005	Closed fracture of ulna, lower epiphysis
Musculoskeletal Injury	SNOMED	208321007	Closed Smith's fracture
Musculoskeletal Injury	SNOMED	208322000	Closed Galeazzi fracture
Musculoskeletal Injury	SNOMED	208323005	Closed volar Barton's fracture
Musculoskeletal Injury	SNOMED	208324004	Closed dorsal Barton's fracture-dislocation
Musculoskeletal Injury	SNOMED	208325003	Closed fracture radial styloid
Musculoskeletal Injury	SNOMED	208326002	Closed fracture distal radius, intra-articular, die-punch
Musculoskeletal Injury	SNOMED	208336005	Open fracture of ulna, styloid process

Musculoskeletal Injury	SNOMED	208337001	Open fracture of ulna, lower epiphysis
Musculoskeletal Injury	SNOMED	208340001	Smith's fracture - open
Musculoskeletal Injury	SNOMED	208341002	Open Galeazzi fracture
Musculoskeletal Injury	SNOMED	208342009	Open volar Barton fracture-dislocation
Musculoskeletal Injury	SNOMED	208343004	Open dorsal Barton's fracture
Musculoskeletal Injury	SNOMED	208344005	Open fracture radial styloid
Musculoskeletal Injury	SNOMED	208345006	Open fracture distal radius, intra-articular, die-punch
Musculoskeletal Injury	SNOMED	208388003	Fracture at wrist and hand level
Musculoskeletal Injury	SNOMED	208510002	Multiple fractures of clavicle, scapula and humerus
Musculoskeletal Injury	SNOMED	208513000	Multiple fractures of forearm
Musculoskeletal Injury	SNOMED	208521006	Closed fracture proximal femur, transcervical
Musculoskeletal Injury	SNOMED	208523009	Closed fracture proximal femur, transepiphyseal
Musculoskeletal Injury	SNOMED	208524003	Closed fracture proximal femur, midcervical section
Musculoskeletal Injury	SNOMED	208526001	Closed fracture of head of femur
Musculoskeletal Injury	SNOMED	208528000	Closed fracture proximal femur, subcapital, Garden grade I
Musculoskeletal Injury	SNOMED	208529008	Closed fracture proximal femur, subcapital, Garden grade II
Musculoskeletal Injury	SNOMED	208530003	Closed fracture proximal femur, subcapital, Garden grade III
Musculoskeletal Injury	SNOMED	208531004	Closed fracture proximal femur, subcapital, Garden grade IV
Musculoskeletal Injury	SNOMED	208536009	Open fracture proximal femur, transepiphyseal
Musculoskeletal Injury	SNOMED	208537000	Open fracture proximal femur, midcervical section
Musculoskeletal Injury	SNOMED	208540000	Open fracture proximal femur,subcapital, Garden grade unspec
Musculoskeletal Injury	SNOMED	208541001	Open fracture proximal femur,subcapital, Garden grade I
Musculoskeletal Injury	SNOMED	208542008	Open fracture proximal femur,subcapital, Garden grade II
Musculoskeletal Injury	SNOMED	208543003	Open fracture proximal femur,subcapital, Garden grade III
Musculoskeletal Injury	SNOMED	208544009	Open fracture proximal femur,subcapital, Garden grade IV
Musculoskeletal Injury	SNOMED	208548007	Closed fracture of proximal femur, pertrochanteric
Musculoskeletal Injury	SNOMED	208550004	Closed fracture proximal femur, intertrochanteric, two part
Musculoskeletal Injury	SNOMED	208551000	Closed fracture proximal femur, subtrochanteric
Musculoskeletal Injury	SNOMED	208552007	Cls # proximal femur, intertrochanteric, comminuted
Musculoskeletal Injury	SNOMED	208555009	Open fracture of proximal femur, pertrochanteric, NOS
Musculoskeletal Injury	SNOMED	208557001	Open fracture proximal femur, intertrochanteric, two part
Musculoskeletal Injury	SNOMED	208558006	Open fracture proximal femur, subtrochanteric
Musculoskeletal Injury	SNOMED	208559003	Open fracture proximal femur, intertrochanteric, comminuted
Musculoskeletal Injury	SNOMED	208562000	Pertrochanteric fracture
Musculoskeletal Injury	SNOMED	208613008	Closed fracture intercondylar spine of tibia
Musculoskeletal Injury	SNOMED	208622009	Open fracture tibial spine
Musculoskeletal Injury	SNOMED	209253005	Closed fracture-dislocation superior radio-ulnar joint
Musculoskeletal Injury	SNOMED	209256002	Open fracture-dislocation superior radio-ulnar joint
Musculoskeletal Injury	SNOMED	209259009	Closed fracture-subluxation superior radio-ulnar joint
Musculoskeletal Injury	SNOMED	209262007	Open fracture-subluxation superior radio-ulnar joint
Musculoskeletal Injury	SNOMED	209263002	Fracture-dislocation or subluxation of wrist
Musculoskeletal Injury	SNOMED	209264008	Closed fracture dislocation of wrist
Musculoskeletal Injury	SNOMED	209265009	Closed fracture-dislocation distal radio-ulnar joint
Musculoskeletal Injury	SNOMED	209266005	Closed fracture-dislocation radiocarpal joint
Musculoskeletal Injury	SNOMED	209273000	Open fracture dislocation wrist
Musculoskeletal Injury	SNOMED	209274006	Open fracture-dislocation, distal radio-ulnar joint
Musculoskeletal Injury	SNOMED	209276008	Open fracture-dislocation radiocarpal joint
Musculoskeletal Injury	SNOMED	209283001	Closed fracture-subluxation of the wrist
Musculoskeletal Injury	SNOMED	209284007	Closed fracture-subluxation, distal radio-ulnar jt
Musculoskeletal Injury	SNOMED	209285008	Closed fracture-subluxation radiocarpal joint
Musculoskeletal Injury	SNOMED	209292003	Open fracture-subluxation of the wrist
Musculoskeletal Injury	SNOMED	209293008	Open fracture-subluxation, distal radio-ulnar joint
Musculoskeletal Injury	SNOMED	209294002	Open fracture subluxation radiocarpal joint
Musculoskeletal Injury	SNOMED	209334003	Fracture-dislocation or subluxation hip
Musculoskeletal Injury	SNOMED	209335002	Closed fracture-dislocation, hip joint
Musculoskeletal Injury	SNOMED	209336001	Open fracture-dislocation, hip joint
Musculoskeletal Injury	SNOMED	209337005	Closed fracture-subluxation, hip joint
Musculoskeletal Injury	SNOMED	209338000	Open fracture-subluxation, hip joint
Musculoskeletal Injury	SNOMED	210958007	Sequelae of fracture at wrist and hand level
Musculoskeletal Injury	SNOMED	210967007	Late effect of fracture neck of femur
Musculoskeletal Injury	SNOMED	213350006	Fractures involving thorax with lower back and pelvis
Musculoskeletal Injury	SNOMED	21419000	Closed fracture distal humerus, medial condyle
Musculoskeletal Injury	SNOMED	21573009	C7 vertebra closed fracture without spinal cord lesion
Musculoskeletal Injury	SNOMED	230411000000106	Vertebroplasty of fracture of spine
Musculoskeletal Injury	SNOMED	230841000000109	Delivery of rehabilitation for hip fracture
Musculoskeletal Injury	SNOMED	23741005	Open fracture of humerus, shaft
Musculoskeletal Injury	SNOMED	239281003	Prim op red # nck femur & op fix - Deyerle multiple hip pin
Musculoskeletal Injury	SNOMED	239284006	Prim op red # nck femur & op fix- Charnley compression screw
Musculoskeletal Injury	SNOMED	239285007	DHS - Dynamic hip screw primary fixation of neck of femur
Musculoskeletal Injury	SNOMED	239286008	Prim open reduct # neck femur & op fix - Richards screw
Musculoskeletal Injury	SNOMED	239288009	Closed reduction and internal fixation of proximal femoral fracture with Richard cannulated hip screw
Musculoskeletal Injury	SNOMED	239291009	Prim open reduct # neck femur & op fix - Holt nail
Musculoskeletal Injury	SNOMED	239292002	Prim open reduct # neck femur & op fix - Ross Brown nail
Musculoskeletal Injury	SNOMED	239548003	Primary other external stabilisation of spinal fracture

Musculoskeletal Injury	SNOMED	239550006	Spinal traction for fracture of spine NEC
Musculoskeletal Injury	SNOMED	25415003	Closed fracture of unspecified proximal femur
Musculoskeletal Injury	SNOMED	25529004	Open fracture of the distal radius, unspecified
Musculoskeletal Injury	SNOMED	263178004	Fracture of transverse process spine - no spinal cord lesion
Musculoskeletal Injury	SNOMED	263179007	Fracture of transverse process of spine + spinal cord lesion
Musculoskeletal Injury	SNOMED	263188003	Open fracture of first cervical vertebra without spinal cord lesion
Musculoskeletal Injury	SNOMED	263189006	C2 vertebra open fracture without spinal cord lesion
Musculoskeletal Injury	SNOMED	263192005	Fracture of lower end of humerus
Musculoskeletal Injury	SNOMED	263198009	Closed fracture of radius, shaft, unspecified
Musculoskeletal Injury	SNOMED	263199001	Fracture of lower end of radius
Musculoskeletal Injury	SNOMED	263204007	Fracture of shaft of ulna
Musculoskeletal Injury	SNOMED	263207000	Fracture of shafts of both ulna and radius
Musculoskeletal Injury	SNOMED	263208005	Fracture of lower end of both ulna and radius
Musculoskeletal Injury	SNOMED	263225007	Hip fracture NOS
Musculoskeletal Injury	SNOMED	263229001	Subtrochanteric fracture
Musculoskeletal Injury	SNOMED	263231005	Open fracture base of neck of femur
Musculoskeletal Injury	SNOMED	265132005	Prim op red # nck femur & op fix- Zickel intramed nail plate
Musculoskeletal Injury	SNOMED	265145009	Hip pin for fixation of epiphysis
Musculoskeletal Injury	SNOMED	265721002	Other specified fixation of fracture of spine
Musculoskeletal Injury	SNOMED	26738009	Spinal cord lesion - no #
Musculoskeletal Injury	SNOMED	268824003	Fracture of radius and/or ulna due to birth trauma
Musculoskeletal Injury	SNOMED	269061001	Fracture of vertebra without spinal cord lesion
Musculoskeletal Injury	SNOMED	269062008	#Cervical spine-no cord lesion
Musculoskeletal Injury	SNOMED	269063003	Closed fracture atlas
Musculoskeletal Injury	SNOMED	269064009	Closed fracture of second cervical vertebra
Musculoskeletal Injury	SNOMED	269065005	Closed fracture of third cervical vertebra
Musculoskeletal Injury	SNOMED	269066006	Closed fracture of fourth cervical vertebra
Musculoskeletal Injury	SNOMED	269067002	Closed fracture of fifth cervical vertebra
Musculoskeletal Injury	SNOMED	269068007	Closed fracture of sixth cervical vertebra
Musculoskeletal Injury	SNOMED	269069004	Closed fracture of seventh cervical vertebra
Musculoskeletal Injury	SNOMED	269070003	Open fracture of cervical spine
Musculoskeletal Injury	SNOMED	269073001	Open fracture of third cervical vertebra
Musculoskeletal Injury	SNOMED	269074007	Open fracture of fourth cervical vertebra
Musculoskeletal Injury	SNOMED	269075008	Open fracture of fifth cervical vertebra
Musculoskeletal Injury	SNOMED	269076009	Open fracture of sixth cervical vertebra
Musculoskeletal Injury	SNOMED	269077000	Open fracture of seventh cervical vertebra
Musculoskeletal Injury	SNOMED	269080004	Closed fracture of the distal humerus
Musculoskeletal Injury	SNOMED	269081000	Open fracture of lower end of humerus
Musculoskeletal Injury	SNOMED	269083002	Closed Colles' fracture
Musculoskeletal Injury	SNOMED	26938002	Open fracture of femur, intertrochanteric
Musculoskeletal Injury	SNOMED	270507001	Pathological fracture of thoracic vertebra
Musculoskeletal Injury	SNOMED	270508006	Pathological fracture of lumbar vertebra
Musculoskeletal Injury	SNOMED	272300006	Primary cls red+int fxn prox fem #+screw/nail device alone
Musculoskeletal Injury	SNOMED	274156000	Opn spinal fracture with unspec cervical cord lesion, C1-4
Musculoskeletal Injury	SNOMED	274157009	Opn spinal fracture with unspec thoracic cord lesion, T7-12
Musculoskeletal Injury	SNOMED	274158004	Open spinal fracture with incomplete lumbar cord lesion NOS
Musculoskeletal Injury	SNOMED	275093002	Internal fixation of fracture of spine
Musculoskeletal Injury	SNOMED	275338001	Cls # prox femur, subcapital, Garden grade unspec.
Musculoskeletal Injury	SNOMED	275339009	Open fracture of femur, subcapital
Musculoskeletal Injury	SNOMED	275340006	Closed fracture of femur, greater trochanter
Musculoskeletal Injury	SNOMED	275341005	Closed fracture of femur, lesser trochanter
Musculoskeletal Injury	SNOMED	275342003	Open fracture of femur, lesser trochanter
Musculoskeletal Injury	SNOMED	275343008	Open fracture of greater trochanter of femur
Musculoskeletal Injury	SNOMED	275365008	Birth fracture of radius
Musculoskeletal Injury	SNOMED	275366009	Birth fracture of ulna
Musculoskeletal Injury	SNOMED	28078000	#Radius/ulna -shaft
Musculoskeletal Injury	SNOMED	28576007	Open fracture of unspecified proximal femur
Musculoskeletal Injury	SNOMED	287065007	Pathological fracture - upper arm
Musculoskeletal Injury	SNOMED	287066008	Pathological fracture - forearm
Musculoskeletal Injury	SNOMED	28753006	C1 vertebra closed fracture - no spinal cord lesion
Musculoskeletal Injury	SNOMED	307712005	Open Barton fracture
Musculoskeletal Injury	SNOMED	307713000	Closed Barton's fracture
Musculoskeletal Injury	SNOMED	308145001	Pathological fracture of cervical vertebra
Musculoskeletal Injury	SNOMED	311426001	Revsn to opn red+int fxtn prox fem #+screw/nail device alone
Musculoskeletal Injury	SNOMED	311488002	Primary closed reduction of spinal fracture
Musculoskeletal Injury	SNOMED	311489005	Revision cls reduc spinal fracture+other external stabilistn
Musculoskeletal Injury	SNOMED	311490001	Primary open reduction spinal fracture alone
Musculoskeletal Injury	SNOMED	311491002	Revision to open reduction spinal fracture alone
Musculoskeletal Injury	SNOMED	311814002	Closed multiple fractures of thoracic spine
Musculoskeletal Injury	SNOMED	311815001	Open multiple fracture of thoracic spine
Musculoskeletal Injury	SNOMED	311816000	Closed multiple fractures of clavicle, scapula and humerus
Musculoskeletal Injury	SNOMED	311817009	Open multiple fractures of clavicle and/or scapula and/or humerus
Musculoskeletal Injury	SNOMED	311862008	Closed fracture inv thorax wth low back and pelvis and limbs
Musculoskeletal Injury	SNOMED	311864009	Open fracture inv thorax wth low back and pelvis and limbs
Musculoskeletal Injury	SNOMED	312660000	Late effect of fracture of cervical vertebra

Musculoskeletal Injury	SNOMED	312661001	Late effect of fracture of thoracic vertebra
Musculoskeletal Injury	SNOMED	312662008	Late effect of fracture of lumbar vertebra
Musculoskeletal Injury	SNOMED	313330006	Closed reduction of intracapsular # NOF internal fixat DHS
Musculoskeletal Injury	SNOMED	3228009	Closed fracture of the radial shaft
Musculoskeletal Injury	SNOMED	33118001	Closed fracture acetabulum, double column unspecified
Musculoskeletal Injury	SNOMED	33192001	Closed fracture radius and ulna, distal
Musculoskeletal Injury	SNOMED	35442005	Closed fracture of forearm, lower end, NOS
Musculoskeletal Injury	SNOMED	359817006	Closed fracture of hip
Musculoskeletal Injury	SNOMED	359820003	Closed fracture of neck of femur NOS
Musculoskeletal Injury	SNOMED	361118003	Open fracture of hip
Musculoskeletal Injury	SNOMED	361119006	Open fracture of neck of femur NOS
Musculoskeletal Injury	SNOMED	37449000	Open fracture of ulna (alone), unspecified
Musculoskeletal Injury	SNOMED	391093004	H/O: hip fracture
Musculoskeletal Injury	SNOMED	391094005	H/O: vertebral fracture
Musculoskeletal Injury	SNOMED	39706009	Closed reduction of fracture of humerus
Musculoskeletal Injury	SNOMED	39993000	Open fracture of radius and ulna, shaft, NOS
Musculoskeletal Injury	SNOMED	41036008	Closed fracture of styloid process of ulna
Musculoskeletal Injury	SNOMED	41191003	Open fracture head, femur
Musculoskeletal Injury	SNOMED	413875004	Closed fracture proximal humerus, head
Musculoskeletal Injury	SNOMED	42157000	Closed fracture of spine, unspecified,
Musculoskeletal Injury	SNOMED	426243004	Balloon kyphoplasty of fracture of spine
Musculoskeletal Injury	SNOMED	42636007	Closed fracture of the proximal humerus
Musculoskeletal Injury	SNOMED	42760000	Open fracture of the ulnar shaft
Musculoskeletal Injury	SNOMED	42945005	Open fracture of radius
Musculoskeletal Injury	SNOMED	43295006	Closed fracture of humerus, shaft or unspecified part
Musculoskeletal Injury	SNOMED	448355005	Greenstick fracture of distal radius
Musculoskeletal Injury	SNOMED	448838000	Closed fracture distal radius, extra-articular, other type
Musculoskeletal Injury	SNOMED	449147001	Infracoccygeal uteropexy
Musculoskeletal Injury	SNOMED	46995009	Cls spinal fracture with unspec cervical cord lesion, C5-7
Musculoskeletal Injury	SNOMED	4750004	Open fracture distal humerus, medial condyle
Musculoskeletal Injury	SNOMED	50397009	Closed fracture of lower end of ulna
Musculoskeletal Injury	SNOMED	50448004	Fracture of vertebra
Musculoskeletal Injury	SNOMED	50890004	Fracture of shaft of humerus
Musculoskeletal Injury	SNOMED	52622006	C4 vertebra closed fracture without spinal cord lesion
Musculoskeletal Injury	SNOMED	53627009	Closed fracture of the radius and ulna
Musculoskeletal Injury	SNOMED	53792000	Closed fracture of the ulnar shaft
Musculoskeletal Injury	SNOMED	54556006	Fracture of ulna NOS
Musculoskeletal Injury	SNOMED	54819005	Closed fracture of radius and ulna, shaft
Musculoskeletal Injury	SNOMED	57470004	Prmy open red+int fxn prox femoral #+screw/nail device alone
Musculoskeletal Injury	SNOMED	576781000000100	Other specified decompression of fracture of spine
Musculoskeletal Injury	SNOMED	58580000	Closed fracture distal humerus, supracondylar
Musculoskeletal Injury	SNOMED	5913000	#Neck of femur
Musculoskeletal Injury	SNOMED	606041000000107	Fracture of humerus NOS
Musculoskeletal Injury	SNOMED	62356006	Open supracondylar fracture of humerus
Musculoskeletal Injury	SNOMED	623711000000000	Closed fracture of humerus, shaft or unspecified part
Musculoskeletal Injury	SNOMED	623711000000109	Closed fracture of humerus, shaft or unspecified part
Musculoskeletal Injury	SNOMED	64455005	Fracture of acetabulum
Musculoskeletal Injury	SNOMED	648581000000104	#Spine NOS - no cord lesion
Musculoskeletal Injury	SNOMED	649002	Open fracture distal ulna - other
Musculoskeletal Injury	SNOMED	65966004	Open fracture of forearm, lower end, unspecified
Musculoskeletal Injury	SNOMED	66246007	Open fracture of the radial shaft
Musculoskeletal Injury	SNOMED	66308002	Fracture of humerus
Musculoskeletal Injury	SNOMED	674461000000100	Closed fracture distal ulna, unspecified
Musculoskeletal Injury	SNOMED	674611000000104	Fracture of radius and ulna, NOS
Musculoskeletal Injury	SNOMED	687331000000100	Fracture of spine with spinal cord lesion NOS
Musculoskeletal Injury	SNOMED	68819003	Closed fracture of coronoid process of ulna
Musculoskeletal Injury	SNOMED	68854005	Closed fracture of head of radius
Musculoskeletal Injury	SNOMED	69427008	Open fracture radial head
Musculoskeletal Injury	SNOMED	708898008	Cl red intracaps frac neck femur fix-Garden cannulated screw
Musculoskeletal Injury	SNOMED	71555008	Closed fracture of ulna (alone), unspecified
Musculoskeletal Injury	SNOMED	72497001	Closed # radius neck
Musculoskeletal Injury	SNOMED	736957006	Hip fracture probability score using fracture risk assessment tool
Musculoskeletal Injury	SNOMED	75857000	Fracture of radius AND ulna
Musculoskeletal Injury	SNOMED	760471000000101	Removal of fracture fixation device from spine
Musculoskeletal Injury	SNOMED	766775007	Closed fracture pelvis, coccyx
Musculoskeletal Injury	SNOMED	767262002	Open fracture pelvis, coccyx
Musculoskeletal Injury	SNOMED	77598002	Open fracture acetabulum
Musculoskeletal Injury	SNOMED	78980006	Open fracture of forearm, lower end, NOS
Musculoskeletal Injury	SNOMED	80411001	Open Colles' fracture
Musculoskeletal Injury	SNOMED	81966000	Open fracture of the radius and ulna
Musculoskeletal Injury	SNOMED	82419009	Open fracture of spine, unspecified,
Musculoskeletal Injury	SNOMED	8303001	C6 vertebra closed fracture without spinal cord lesion
Musculoskeletal Injury	SNOMED	849671000000101	Open fracture distal radius, extra-articular other type
Musculoskeletal Injury	SNOMED	849681000000104	Open fracture distal radius, intra-articular other type
Musculoskeletal Injury	SNOMED	851421000006105	Torus fracture of radius

Musculoskeletal Injury	SNOMED	857661000006106	Wedge compression # lumbar spine
Musculoskeletal Injury	SNOMED	857671000006104	Wedge compression # of dorsal spine
Musculoskeletal Injury	SNOMED	86357008	Open fracture of coronoid process of ulna
Musculoskeletal Injury	SNOMED	86844009	Open fracture of proximal humerus not otherwise specified
Musculoskeletal Injury	SNOMED	87804006	C5 vertebra closed fracture without spinal cord lesion
Musculoskeletal Injury	SNOMED	88116004	Open fracture of radius and ulna, lower end
Musculoskeletal Injury	SNOMED	8840000	Cls spinal fracture with unspec cervical cord lesion, C1-4
Musculoskeletal Injury	SNOMED	89294002	Open fracture of humerus NOS
Musculoskeletal Injury	SNOMED	89820008	Closed fracture of femur, intertrochanteric
Musculoskeletal Injury	SNOMED	90235006	#Humerus - shaft
Musculoskeletal Injury	SNOMED	91296001	Open fracture of forearm, unspecified
Musculoskeletal Injury	SNOMED	91419009	Closed fracture of forearm, unspecified
Musculoskeletal Injury	SNOMED	9341001	Late effect of fracture of spine AND/OR trunk without spinal cord lesion
Musculoskeletal Injury	SNOMED	9468002	Wrist fracture - closed
Osteoarthritis	ICD10	G552	Nerve [REDACTED] and plexus compressions in spondylosis
Osteoarthritis	ICD10	M15	Polyarthrosis
Osteoarthritis	ICD10	M16	Coxarthrosis [arthrosis of hip]
Osteoarthritis	ICD10	M17	Gonarthrosis [arthrosis of knee]
Osteoarthritis	ICD10	M18	Arthrosis of first carpometacarpal joint
Osteoarthritis	ICD10	M19	Other arthrosis
Osteoarthritis	ICD10	M47	Spondylosis
Osteoarthritis	SNOMED	10948005	Thoracic spondylosis with myelopathy
Osteoarthritis	SNOMED	161568003	History of osteoarthritis
Osteoarthritis	SNOMED	192907007	Myelopathy due to spondylosis
Osteoarthritis	SNOMED	193120004	Nerve [REDACTED] and plexus compressions in spondylosis
Osteoarthritis	SNOMED	201819000	Generalised osteoarthritis of unspecified site
Osteoarthritis	SNOMED	201825001	Secondary multiple arthrosis
Osteoarthritis	SNOMED	201826000	Erosive osteoarthrosis
Osteoarthritis	SNOMED	201827009	Heberden's nodes with arthropathy
Osteoarthritis	SNOMED	201829007	Localised, primary osteoarthritis of other specified site
Osteoarthritis	SNOMED	201831003	Primary localised osteoarthrosis of shoulder region
Osteoarthritis	SNOMED	201834006	Localised, primary osteoarthritis of the hand
Osteoarthritis	SNOMED	201835007	Localized, primary osteoarthritis of the pelvic region and thigh
Osteoarthritis	SNOMED	201837004	Osteoarthritis NOS, of ankle and foot
Osteoarthritis	SNOMED	201839001	Primary coxarthrosis, bilateral
Osteoarthritis	SNOMED	201841000	Primary gonarthrosis, bilateral
Osteoarthritis	SNOMED	201842007	Primary arthrosis of first carpometacarpal joints, bilateral
Osteoarthritis	SNOMED	201847001	Localised, secondary osteoarthritis
Osteoarthritis	SNOMED	201849003	Secondary localized osteoarthrosis of shoulder region
Osteoarthritis	SNOMED	201852006	Localised, secondary osteoarthritis of the hand
Osteoarthritis	SNOMED	201855008	Localised, secondary osteoarthritis of the ankle and foot
Osteoarthritis	SNOMED	201857000	Post-traumatic coxarthrosis, bilateral
Osteoarthritis	SNOMED	201858005	Post-traumatic gonarthrosis, bilateral
Osteoarthritis	SNOMED	201859002	Post-traumatic arthrosis of first carpometacarpal jt bilat
Osteoarthritis	SNOMED	202657000	Single-level cervical spondylosis without myelopathy
Osteoarthritis	SNOMED	202658005	Two-level cervical spondylosis without myelopathy
Osteoarthritis	SNOMED	202659002	Multiple-level cervical spondylosis without myelopathy
Osteoarthritis	SNOMED	202661006	Single-level cervical spondylosis with myelopathy
Osteoarthritis	SNOMED	202662004	Two-level cervical spondylosis with myelopathy
Osteoarthritis	SNOMED	202663009	Multiple-level cervical spondylosis with myelopathy
Osteoarthritis	SNOMED	202666001	Single-level thoracic spondylosis without myelopathy
Osteoarthritis	SNOMED	202667005	Two-level thoracic spondylosis without myelopathy
Osteoarthritis	SNOMED	202668000	Multiple-level thoracic spondylosis without myelopathy
Osteoarthritis	SNOMED	202670009	Single-level thoracic spondylosis with myelopathy
Osteoarthritis	SNOMED	202671008	Two-level thoracic spondylosis with myelopathy
Osteoarthritis	SNOMED	202672001	Multiple-level thoracic spondylosis with myelopathy
Osteoarthritis	SNOMED	202674000	Single-level lumbosacral spondylosis without myelopathy
Osteoarthritis	SNOMED	202675004	Two-level lumbosacral spondylosis without myelopathy
Osteoarthritis	SNOMED	202676003	Multiple-level lumbosacral spondylosis without myelopathy
Osteoarthritis	SNOMED	202677007	Lumbosacral spondylosis with myelopathy
Osteoarthritis	SNOMED	202678002	Single-level lumbosacral spondylosis with myelopathy
Osteoarthritis	SNOMED	202679005	Two-level lumbosacral spondylosis with myelopathy
Osteoarthritis	SNOMED	202680008	Multiple-level lumbosacral spondylosis with myelopathy
Osteoarthritis	SNOMED	202683005	Cervical spondylosis with radiculopathy
Osteoarthritis	SNOMED	202684004	Single-level cervical spondylosis with radiculopathy
Osteoarthritis	SNOMED	202685003	Two-level cervical spondylosis with radiculopathy
Osteoarthritis	SNOMED	202686002	Multiple-level cervical spondylosis with radiculopathy
Osteoarthritis	SNOMED	202687006	Cervical spondylosis with vascular compression
Osteoarthritis	SNOMED	202688001	Thoracic spondylosis with [REDACTED] compression
Osteoarthritis	SNOMED	202689009	Single-level thoracic spondylosis with radiculopathy
Osteoarthritis	SNOMED	202690000	Two-level thoracic spondylosis with radiculopathy
Osteoarthritis	SNOMED	202692008	Multiple-level thoracic spondylosis with radiculopathy
Osteoarthritis	SNOMED	202693003	Lumbosacral spondylosis with radiculopathy
Osteoarthritis	SNOMED	202694009	Single-level lumbosacral spondylosis with radiculopathy
Osteoarthritis	SNOMED	202695005	Two-level lumbosacral spondylosis with radiculopathy

Osteoarthritis	SNOMED	202696006	Multiple-level lumbosacral spondylosis with radiculopathy
Osteoarthritis	SNOMED	22193007	Oligoarticular osteoarthritis, unspecified, of hand
Osteoarthritis	SNOMED	230771000000000	Delivery of rehabilitation for osteoarthritis
Osteoarthritis	SNOMED	239862000	Primary generalized osteoarthrosis
Osteoarthritis	SNOMED	239863005	Degenerative arthropathy of spinal facet joint
Osteoarthritis	SNOMED	239865003	Osteoarthritis NOS, of acromioclavicular joint
Osteoarthritis	SNOMED	239866002	Osteoarthritis NOS, of elbow
Osteoarthritis	SNOMED	239867006	OA - Osteoarthritis of wrist
Osteoarthritis	SNOMED	239868001	Degenerative joint disease of finger
Osteoarthritis	SNOMED	239869009	Degenerative joint disease of distal interphalangeal joint
Osteoarthritis	SNOMED	239870005	Osteoarthritis NOS, of PIP joint of finger
Osteoarthritis	SNOMED	239872002	Osteoarthritis NOS, of hip
Osteoarthritis	SNOMED	239873007	Gonarthrosis
Osteoarthritis	SNOMED	239874001	Osteoarthritis NOS, of ankle
Osteoarthritis	SNOMED	239876004	Degenerative joint disease of subtalar joint
Osteoarthritis	SNOMED	239877008	Osteoarthritis NOS, of 1st MTP joint
Osteoarthritis	SNOMED	239878003	Toe osteoarthritis NOS
Osteoarthritis	SNOMED	239880009	Lumbar spondylosis
Osteoarthritis	SNOMED	267889007	Generalised osteoarthritis of the hand
Osteoarthritis	SNOMED	267890003	Localised, secondary osteoarthritis of pelvic region/thigh
Osteoarthritis	SNOMED	267891004	Localised osteoarthritis, unspecified, of the pelvic region and thigh
Osteoarthritis	SNOMED	267969005	Spondylosis and allied disorders
Osteoarthritis	SNOMED	267970006	Cervical spondylosis without myelopathy
Osteoarthritis	SNOMED	267971005	Thoracic spondylosis without myelopathy
Osteoarthritis	SNOMED	268054009	Oligoarticular osteoarthritis, unspecified
Osteoarthritis	SNOMED	274135002	Arthritis/arthrosis
Osteoarthritis	SNOMED	275324008	Osteoarthritis NOS, of MCP joint
Osteoarthritis	SNOMED	309246000	Degenerative joint disease of foot
Osteoarthritis	SNOMED	309761000	Post-traumatic gonarthrosis, unilateral
Osteoarthritis	SNOMED	313257005	Localised, primary osteoarthritis of the wrist
Osteoarthritis	SNOMED	313258000	Localised, primary osteoarthritis of toe
Osteoarthritis	SNOMED	313259008	Localized, primary osteoarthritis of elbow
Osteoarthritis	SNOMED	33952002	Localized osteoarthrosis
Osteoarthritis	SNOMED	36186002	[X]Other polyarthrosis
Osteoarthritis	SNOMED	371598009	Heberdens' nodes
Osteoarthritis	SNOMED	37895003	Thumb osteoarthritis NOS
Osteoarthritis	SNOMED	387800004	Osteoarthritis cervical spine
Osteoarthritis	SNOMED	387802007	Thoracic spondylosis
Osteoarthritis	SNOMED	396275006	Osteoarthritis
Osteoarthritis	SNOMED	399269003	[X]Other specified arthrosis
Osteoarthritis	SNOMED	43270005	Localised osteoarthritis, unspecified, of the forearm
Osteoarthritis	SNOMED	445478004	Osteoarthritis NOS, pelvic region/thigh
Osteoarthritis	SNOMED	450521003	Osteoarthritis of patellofemoral joint
Osteoarthritis	SNOMED	45315006	Osteoarticular thermography
Osteoarthritis	SNOMED	48210000	Lumbosacral spondylosis without myelopathy
Osteoarthritis	SNOMED	55146009	Osteoarthritis NOS, of sacro-iliac joint
Osteoarthritis	SNOMED	55176002	Coxae malum senilis
Osteoarthritis	SNOMED	55705006	Spondylosyndesis
Osteoarthritis	SNOMED	576511000000000	Osteoarthritis NOS, of elbow
Osteoarthritis	SNOMED	587231000000000	Osteoarthritis NOS, of hip
Osteoarthritis	SNOMED	637091000000000	Osteoarthritis NOS, of the lower leg
Osteoarthritis	SNOMED	65260001	Cervical spondylosis with myelopathy
Osteoarthritis	SNOMED	653461000000000	Osteoarthritis - wrist joint
Osteoarthritis	SNOMED	666441000000000	Osteoarthritis NOS, of shoulder region
Osteoarthritis	SNOMED	666451000000000	Osteoarthritis NOS, other specified site
Osteoarthritis	SNOMED	666611000000000	Spondyloses
Osteoarthritis	SNOMED	667531000000000	Osteoarthritis NOS, of knee
Osteoarthritis	SNOMED	67315001	Osteoarthritis NOS, of shoulder region
Osteoarthritis	SNOMED	68859000	Spondylosis without myelopathy, NOS
Osteoarthritis	SNOMED	700324005	Osteoarthritis NOS, of talonavicular joint
Osteoarthritis	SNOMED	805881000000000	Exception reporting: osteoarthritis quality indicators
Osteoarthritis	SNOMED	805901000000000	Excepted fr osteoarthritis quality indicators: pt unsuitable
Osteoarthritis	SNOMED	805921000000000	Excepted osteoarthritis quality indicators: informed dissent
Osteoarthritis	SNOMED	82304009	Osteoarthrosis interspinalis
Osteoarthritis	SNOMED	850791000000000	Osteoarthritis NOS, of sternoclavicular joint
Osteoarthritis	SNOMED	8847002	Other spondyloses and allied disorders
Osteoarthritis	SNOMED	905311000000000	[RFC] Osteoarthritis
Osteoarthritis	SNOMED	909181000000000	[RFC] Osteoarthritis
Osteoarthritis	SNOMED	932071000000000	Exacerbation of osteoarthritis
Osteoarthritis	SNOMED	9631008	Spondylosis deformans
Osteoporosis	ICD10	M80	Osteoporosis with pathological fracture
Osteoporosis	ICD10	M81	Osteoporosis without pathological fracture
Osteoporosis	ICD10	M82	Osteoporosis in diseases classified elsewhere
Osteoporosis	SNOMED	102447009	Postmenopausal osteoporosis
Osteoporosis	SNOMED	14651005	Drug-induced osteoporosis

Osteoporosis	SNOMED	1573631000006108	H/O: osteoporosis
Osteoporosis	SNOMED	15743005	Posttraumatic osteoporosis
Osteoporosis	SNOMED	18040001	Senile osteoporosis
Osteoporosis	SNOMED	1823981000006104	Osteoporosis confirmed
Osteoporosis	SNOMED	1853181000006101	Osteoporosis treatment restart date
Osteoporosis	SNOMED	1932111000006108	3D study - problems with osteoporosis management
Osteoporosis	SNOMED	203429007	Idiopathic generalized osteoporosis
Osteoporosis	SNOMED	203433000	Postoophorectomy osteoporosis
Osteoporosis	SNOMED	203434006	Postsurgical malabsorption osteoporosis
Osteoporosis	SNOMED	203435007	Localized osteoporosis - Lequesne
Osteoporosis	SNOMED	203437004	Osteoporosis in endocrine disorders
Osteoporosis	SNOMED	203438009	Vertebral osteoporosis
Osteoporosis	SNOMED	203444008	Postoophorectomy osteoporosis with pathological fracture
Osteoporosis	SNOMED	203445009	Osteoporosis of disuse with pathological fracture
Osteoporosis	SNOMED	203446005	Postsurgical malabsorption osteoporosis with path fracture
Osteoporosis	SNOMED	203447001	Drug-induced osteoporosis with pathological fracture
Osteoporosis	SNOMED	203448006	Idiopathic osteoporosis with pathological fracture
Osteoporosis	SNOMED	203450003	Osteoporosis + pathological fracture lumbar vertebrae
Osteoporosis	SNOMED	203451004	Osteoporosis + pathological fracture thoracic vertebrae
Osteoporosis	SNOMED	203452006	Osteoporosis + pathological fracture cervical vertebrae
Osteoporosis	SNOMED	203453001	Postmenopausal osteoporosis with pathological fracture
Osteoporosis	SNOMED	203657009	Osteoporotic kyphosis
Osteoporosis	SNOMED	240156000	Juvenile idiopathic generalised osteoporosis
Osteoporosis	SNOMED	240157009	Secondary generalised osteoporosis
Osteoporosis	SNOMED	240159007	Transient osteoporosis of hip
Osteoporosis	SNOMED	240198002	Osteoporotic vertebral collapse
Osteoporosis	SNOMED	276661002	Age-related osteoporosis
Osteoporosis	SNOMED	306171000000106	Minimal trauma fracture
Osteoporosis	SNOMED	309745002	Osteoporosis localized to spine
Osteoporosis	SNOMED	311806008	Collapse of cervical vertebra due to osteoporosis
Osteoporosis	SNOMED	311890007	Collapse of lumbar vertebra due to osteoporosis
Osteoporosis	SNOMED	311891006	Collapse of thoracic vertebra due to osteoporosis
Osteoporosis	SNOMED	3345002	Idiopathic osteoporosis
Osteoporosis	SNOMED	390833005	Osteoporosis caused by corticosteroid
Osteoporosis	SNOMED	391020007	Osteoporosis treatment started
Osteoporosis	SNOMED	391021006	Osteoporosis treatment stopped
Osteoporosis	SNOMED	391022004	Osteoporosis treatment changed
Osteoporosis	SNOMED	391023009	Osteoporosis - no treatment
Osteoporosis	SNOMED	391028000	Osteoporosis - treatment response
Osteoporosis	SNOMED	391030003	Osteoporosis - no treatment response
Osteoporosis	SNOMED	391060008	Forearm DEXA scan result osteoporotic
Osteoporosis	SNOMED	391065003	Heel dual energy X-ray photon absorptiometry scan result osteoporotic
Osteoporosis	SNOMED	391070005	Hip DXA scan result osteoporotic
Osteoporosis	SNOMED	391075000	Lumbar dual energy X-ray photon absorptiometry scan result osteoporotic
Osteoporosis	SNOMED	391081008	Quantitative ultrasound scan of heel - result osteoporotic
Osteoporosis	SNOMED	391092009	H/O: fragility fracture
Osteoporosis	SNOMED	440100002	Femoral neck DEXA scan result osteoporotic
Osteoporosis	SNOMED	443165006	Fragility fracture due to unspecified osteoporosis
Osteoporosis	SNOMED	46675001	Osteoporotic fracture
Osteoporosis	SNOMED	473120007	History of osteoporosis
Osteoporosis	SNOMED	505141000000108	Osteoporosis - enhanced services administration
Osteoporosis	SNOMED	505171000000102	Bone sparing drug treatment offered for osteoporosis - ESA
Osteoporosis	SNOMED	53174001	Dissuse osteoporosis
Osteoporosis	SNOMED	64859006	Osteoporosis, unspecified
Osteoporosis	SNOMED	703264005	Secondary osteoporosis
Osteoporosis	SNOMED	739301006	Osteoporosis co-occurrent and due to multiple myeloma
Osteoporosis	SNOMED	965871000000101	Osteoporosis medication compliance review
Parkinson's Disease	ICD10	F023	Dementia in Parkinson disease
Parkinson's Disease	ICD10	G20	Parkinson disease
Parkinson's Disease	SNOMED	341551000000108	Cerebral degeneration in Parkinson's disease
Parkinson's Disease	SNOMED	425390006	[X]Dementia in Parkinson's disease
Parkinson's Disease	SNOMED	49049000	Parkinson's disease
Parkinson's Disease	SNOMED	515841000000104	History of Parkinson's disease
Parkinson's Disease	SNOMED	905821000000000	[RFC] Parkinson's disease
Parkinson's Disease	SNOMED	905821000006108	[RFC] Parkinson's disease
Parkinson's Disease	SNOMED	909021000000000	[RFC] Parkinson's disease
Parkinson's Disease	SNOMED	909021000006101	[RFC] Parkinson's disease
Peptic Ulcer Disease	ICD10	K221	Ulcer of oesophagus
Peptic Ulcer Disease	ICD10	K25	Gastric ulcer
Peptic Ulcer Disease	ICD10	K26	Duodenal ulcer
Peptic Ulcer Disease	ICD10	K27	Peptic ulcer, site unspecified
Peptic Ulcer Disease	ICD10	K280	Gastrojejunal ulcer
Peptic Ulcer Disease	SNOMED	111353003	Acute peptic ulcer with hemorrhage and perforation
Peptic Ulcer Disease	SNOMED	12274003	Acute peptic ulcer with haemorrhage
Peptic Ulcer Disease	SNOMED	128286008	Chronic duodenal ulcer

Peptic Ulcer Disease	SNOMED	128287004	Chronic peptic ulcer without mention of complication
Peptic Ulcer Disease	SNOMED	128288009	Chronic gastrojejunal ulcer
Peptic Ulcer Disease	SNOMED	12847006	Acute duodenal ulcer with hemorrhage
Peptic Ulcer Disease	SNOMED	13200003	Unspecified peptic ulcer without mention of complication
Peptic Ulcer Disease	SNOMED	15902003	Unspecified gastric ulcer with haemorrhage
Peptic Ulcer Disease	SNOMED	16121001	Unspecified gastrojejunal ulcer
Peptic Ulcer Disease	SNOMED	173841001	Other specified operation on gastric ulcer
Peptic Ulcer Disease	SNOMED	173842008	Closure of perforated gastric ulcer
Peptic Ulcer Disease	SNOMED	173887007	Operations on duodenal ulcer
Peptic Ulcer Disease	SNOMED	173889005	Closure of perforated duodenal ulcer
Peptic Ulcer Disease	SNOMED	196607008	Oesophageal ulcer due to aspirin
Peptic Ulcer Disease	SNOMED	196609006	Barrett's ulcer of esophagus
Peptic Ulcer Disease	SNOMED	196632005	Acute gastric ulcer with obstruction
Peptic Ulcer Disease	SNOMED	196639001	Chronic gastric ulcer with obstruction
Peptic Ulcer Disease	SNOMED	196652006	Acute duodenal ulcer
Peptic Ulcer Disease	SNOMED	196658005	Acute duodenal ulcer with obstruction
Peptic Ulcer Disease	SNOMED	196666001	Chronic duodenal ulcer with obstruction
Peptic Ulcer Disease	SNOMED	196671008	Recurrent duodenal ulcer
Peptic Ulcer Disease	SNOMED	196682000	Acute peptic ulcer
Peptic Ulcer Disease	SNOMED	196687006	Acute peptic ulcer with obstruction
Peptic Ulcer Disease	SNOMED	196694009	Chronic peptic ulcer with obstruction
Peptic Ulcer Disease	SNOMED	196707000	Acute gastrojejunal ulcer
Peptic Ulcer Disease	SNOMED	196712004	Acute gastrojejunal ulcer with obstruction
Peptic Ulcer Disease	SNOMED	196719008	Chronic gastrojejunal ulcer with obstruction
Peptic Ulcer Disease	SNOMED	196775009	Healed gastric ulcer leaving a scar
Peptic Ulcer Disease	SNOMED	19850005	Acute gastric ulcer with perforation
Peptic Ulcer Disease	SNOMED	22620000	Prepyloric ulcer
Peptic Ulcer Disease	SNOMED	226891000000104	Oversew of blood vessel of duodenal ulcer
Peptic Ulcer Disease	SNOMED	24060004	Gastrocolic ulcer
Peptic Ulcer Disease	SNOMED	248891000000103	Non steroidal anti inflammatory drug induced gastric ulcer
Peptic Ulcer Disease	SNOMED	266998003	H/O: peptic ulcer
Peptic Ulcer Disease	SNOMED	27281001	Unspecified duodenal ulcer with haemorrhage
Peptic Ulcer Disease	SNOMED	275547005	H/O: duodenal ulcer
Peptic Ulcer Disease	SNOMED	275548000	H/O: gastric ulcer
Peptic Ulcer Disease	SNOMED	2807004	Chronic gastrojejunal ulcer with perforation
Peptic Ulcer Disease	SNOMED	287828006	Excision of ulcer of duodenum
Peptic Ulcer Disease	SNOMED	287832000	Stomach ulcer excision
Peptic Ulcer Disease	SNOMED	307259004	Gastric ulcer suture
Peptic Ulcer Disease	SNOMED	307260009	Suture of duodenal ulcer
Peptic Ulcer Disease	SNOMED	307261008	Closure of perforated bowel ulcer NEC
Peptic Ulcer Disease	SNOMED	30811009	Ulcer of oesophagus
Peptic Ulcer Disease	SNOMED	31301004	Perforated chronic gastric ulcer
Peptic Ulcer Disease	SNOMED	313425006	Multiple gastric ulcers
Peptic Ulcer Disease	SNOMED	314627002	Endoscopic injection hemostasis of duodenal ulcer
Peptic Ulcer Disease	SNOMED	314629004	Endoscopic injection hemostasis of gastric ulcer
Peptic Ulcer Disease	SNOMED	3483000	Chronic peptic ulcer with perforation
Peptic Ulcer Disease	SNOMED	36975000	Chronic duodenal ulcer with haemorrhage AND perforation
Peptic Ulcer Disease	SNOMED	39204006	Pyloric ulcer
Peptic Ulcer Disease	SNOMED	397825006	Unspecified gastric ulcer
Peptic Ulcer Disease	SNOMED	423643000	Duodenal ulcer caused by anti-platelet agent
Peptic Ulcer Disease	SNOMED	424301005	Anti-platelet induced gastric ulcer
Peptic Ulcer Disease	SNOMED	439955006	Ulcerative oesophagitis
Peptic Ulcer Disease	SNOMED	45640006	Chronic gastrojejunal ulcer with haemorrhage AND perforation
Peptic Ulcer Disease	SNOMED	48974009	Acute gastric ulcer with haemorrhage AND perforation
Peptic Ulcer Disease	SNOMED	49232000	Chronic peptic ulcer with hemorrhage
Peptic Ulcer Disease	SNOMED	49916007	Chronic duodenal ulcer with perforation
Peptic Ulcer Disease	SNOMED	51868009	Duodenal ulcer disease
Peptic Ulcer Disease	SNOMED	541501000000105	Duodenal ulcer suture
Peptic Ulcer Disease	SNOMED	543881000000000	Stomach ulcer excision
Peptic Ulcer Disease	SNOMED	543881000000102	Stomach ulcer excision
Peptic Ulcer Disease	SNOMED	54822007	Jejunal ulceration
Peptic Ulcer Disease	SNOMED	557931000000102	Other specified operation on duodenal ulcer
Peptic Ulcer Disease	SNOMED	559771000000000	Closure of perforated bowel ulcer NEC
Peptic Ulcer Disease	SNOMED	559771000000108	Closure of perforated bowel ulcer NEC
Peptic Ulcer Disease	SNOMED	57246001	Chronic gastric ulcer with haemorrhage
Peptic Ulcer Disease	SNOMED	591741000000105	Acute peptic ulcer NOS
Peptic Ulcer Disease	SNOMED	606391000000100	Acute gastric ulcer NOS
Peptic Ulcer Disease	SNOMED	606411000000100	Chronic gastric ulcer NOS
Peptic Ulcer Disease	SNOMED	6129004	Peptic ulcer of oesophagus
Peptic Ulcer Disease	SNOMED	61300005	Chronic peptic ulcer with haemorrhage AND perforation
Peptic Ulcer Disease	SNOMED	61347001	Acute duodenal ulcer with perforation
Peptic Ulcer Disease	SNOMED	618291000000105	Acute duodenal ulcer NOS
Peptic Ulcer Disease	SNOMED	62366003	Unspecified gastric ulcer with haemorrhage and perforation
Peptic Ulcer Disease	SNOMED	62838000	Chronic gastrojejunal ulcer with haemorrhage
Peptic Ulcer Disease	SNOMED	633201000000104	Chronic duodenal ulcer NOS

Peptic Ulcer Disease	SNOMED	63954007	Acute gastrojejunal ulcer with haemorrhage
Peptic Ulcer Disease	SNOMED	64595005	Oesophageal ulcer due to medicines
Peptic Ulcer Disease	SNOMED	652941000000102	Chronic peptic ulcer NOS
Peptic Ulcer Disease	SNOMED	66636001	Acute gastrojejunal ulcer with perforation
Peptic Ulcer Disease	SNOMED	687731000000104	Closure of gastric ulcer NEC
Peptic Ulcer Disease	SNOMED	722200003	Non steroidal anti inflammatory drug induced duodenal ulcer
Peptic Ulcer Disease	SNOMED	76181002	Chronic gastric ulcer with haemorrhage and perforation
Peptic Ulcer Disease	SNOMED	79118000	Acute peptic ulcer with perforation
Peptic Ulcer Disease	SNOMED	81387001	Acute gastrojejunal ulcer with hemorrhage AND perforation
Peptic Ulcer Disease	SNOMED	849591000000103	Unspecified gastric ulcer with obstruction
Peptic Ulcer Disease	SNOMED	849601000000109	Unspecified duodenal ulcer with obstruction
Peptic Ulcer Disease	SNOMED	849611000000106	Unspecified peptic ulcer with obstruction
Peptic Ulcer Disease	SNOMED	849621000000100	Unspecified gastrojejunal ulcer with obstruction
Peptic Ulcer Disease	SNOMED	86895006	Acute duodenal ulcer with haemorrhage AND perforation
Peptic Ulcer Disease	SNOMED	88968005	DU - Perforated duodenal ulcer
Peptic Ulcer Disease	SNOMED	89469000	Chronic duodenal ulcer with haemorrhage
Peptic Ulcer Disease	SNOMED	89748001	Acute gastric ulcer with haemorrhage
Peptic Ulcer Disease	SNOMED	906031000006109	[RFC] Peptic and duodenal ulcers
Peptic Ulcer Disease	SNOMED	95529005	Acute gastric ulcer without mention of complication
Peptic Ulcer Disease	SNOMED	95530000	Chronic gastric ulcer without mention of complication
Peptic Ulcer Disease	SNOMED	9829001	Unspecified gastric ulcer with perforation
Peripheral Neuropathy	ICD10	G130	Paraneoplastic neuromyopathy and neuropathy
Peripheral Neuropathy	ICD10	G560	Carpal tunnel syndrome
Peripheral Neuropathy	ICD10	G569	Mononeuropathy of upper limb, unspecified
Peripheral Neuropathy	ICD10	G579	Mononeuropathy of lower limb, unspecified
Peripheral Neuropathy	ICD10	G589	Mononeuropathy, unspecified
Peripheral Neuropathy	ICD10	G59	Mononeuropathy in diseases classified elsewhere
Peripheral Neuropathy	ICD10	G60	Hereditary and idiopathic neuropathy
Peripheral Neuropathy	ICD10	G61	Inflammatory polyneuropathy
Peripheral Neuropathy	ICD10	G620	Drug-induced polyneuropathy
Peripheral Neuropathy	ICD10	G621	Alcoholic polyneuropathy
Peripheral Neuropathy	ICD10	G622	Polyneuropathy due to other toxic agents
Peripheral Neuropathy	ICD10	G629	Polyneuropathy, unspecified
Peripheral Neuropathy	ICD10	G63	Polyneuropathy in diseases classified elsewhere
Peripheral Neuropathy	ICD10	G900	Idiopathic peripheral autonomic neuropathy
Peripheral Neuropathy	ICD10	G990	Autonomic neuropathy in endocrine and metabolic diseases
Peripheral Neuropathy	SNOMED	1013751000006104	Adverse reaction to Treatment Of Diabetic Neuropathy
Peripheral Neuropathy	SNOMED	11060002	Peripheral autonomic neuropathy due to disease NOS
Peripheral Neuropathy	SNOMED	111499002	Hereditary motor and sensory neuropathy type III
Peripheral Neuropathy	SNOMED	11442006	Posterior sensory radicular neuropathy
Peripheral Neuropathy	SNOMED	128123007	Peripheral autonomic neuropathy disease EC
Peripheral Neuropathy	SNOMED	128194008	Superficial peroneal neuropathy
Peripheral Neuropathy	SNOMED	128205005	Hereditary sensory and autonomic neuropathy
Peripheral Neuropathy	SNOMED	128208007	Peripheral axonal neuropathy
Peripheral Neuropathy	SNOMED	128209004	Chronic inflammatory demyelinating polyradiculoneuropathy
Peripheral Neuropathy	SNOMED	129616004	Polyneuropathy in porphyria
Peripheral Neuropathy	SNOMED	13694005	Sensory polyneuropathy
Peripheral Neuropathy	SNOMED	15241006	[X]Autonomic neuropathy/endocrine+metabolic diseases CE
Peripheral Neuropathy	SNOMED	15346004	Alphalipoproteinaemia neuropathy
Peripheral Neuropathy	SNOMED	160265E+16	Right suprascapular neuropathy
Peripheral Neuropathy	SNOMED	1672020000000000	Axonal sensorimotor neuropathy
Peripheral Neuropathy	SNOMED	1672021000006104	Axonal sensorimotor neuropathy
Peripheral Neuropathy	SNOMED	1767005	Miller-Fisher variant of Guillain-Barre syndrome
Peripheral Neuropathy	SNOMED	186738001	Vestibular neuropathy
Peripheral Neuropathy	SNOMED	192918007	Autonomic neuropathy due to amyloid
Peripheral Neuropathy	SNOMED	193165008	Neuropathy in association with hereditary ataxia
Peripheral Neuropathy	SNOMED	193177003	Polyneuropathy in collagen vascular disease
Peripheral Neuropathy	SNOMED	193178008	Polyneuropathy in disseminated lupus erythematosus
Peripheral Neuropathy	SNOMED	193179000	Polyneuropathy in polyarteritis nodosa
Peripheral Neuropathy	SNOMED	193180002	Polyneuropathy in rheumatoid arthritis
Peripheral Neuropathy	SNOMED	193183000	Acute painful diabetic neuropathy
Peripheral Neuropathy	SNOMED	193184006	Chronic painful diabetic neuropathy
Peripheral Neuropathy	SNOMED	193185007	Asymptomatic diabetic neuropathy
Peripheral Neuropathy	SNOMED	193186008	Polyneuropathy associated with another disorder
Peripheral Neuropathy	SNOMED	193187004	Polyneuropathy in amyloidosis
Peripheral Neuropathy	SNOMED	193188009	Polyneuropathy due to vitamin B deficiency
Peripheral Neuropathy	SNOMED	193190005	Polyneuropathy in herpes zoster
Peripheral Neuropathy	SNOMED	193191009	Polyneuropathy in hypoglycaemia
Peripheral Neuropathy	SNOMED	193193007	Polyneuropathy in pellagra
Peripheral Neuropathy	SNOMED	193195000	Polyneuropathy in sarcoidosis
Peripheral Neuropathy	SNOMED	193196004	Uremic polyneuropathy
Peripheral Neuropathy	SNOMED	193203006	Serum neuropathy
Peripheral Neuropathy	SNOMED	22722001	Other idiopathic peripheral neuropathy
Peripheral Neuropathy	SNOMED	230540000	Idiopathic trigeminal neuropathy
Peripheral Neuropathy	SNOMED	230571009	[X]Polyneuropathy/other endocrine+metabolic diseases CE

Peripheral Neuropathy	SNOMED	230572002	Diabetic neuropathy
Peripheral Neuropathy	SNOMED	230575000	Diabetic chronic painful polyneuropathy
Peripheral Neuropathy	SNOMED	230577008	Diabetic mononeuropathy
Peripheral Neuropathy	SNOMED	230591002	Multifocal motor neuropathy
Peripheral Neuropathy	SNOMED	230594005	Critical illness-associated polyneuropathy
Peripheral Neuropathy	SNOMED	230595006	[X]Polyneuropathy in infectious+parasitic diseases CE
Peripheral Neuropathy	SNOMED	230596007	Polyneuropathy in diphtheria
Peripheral Neuropathy	SNOMED	230599000	Ischaemic neuropathy
Peripheral Neuropathy	SNOMED	230627003	Compression neuropathy of upper limb
Peripheral Neuropathy	SNOMED	230637008	Compression neuropathy of lower limb
Peripheral Neuropathy	SNOMED	230645003	Compression neuropathy of trunk
Peripheral Neuropathy	SNOMED	230655004	Idiopathic chronic neuropathy
Peripheral Neuropathy	SNOMED	230656003	Demyelinating sensorimotor neuropathy
Peripheral Neuropathy	SNOMED	230657007	Axonal sensorimotor neuropathy
Peripheral Neuropathy	SNOMED	23414001	Peripheral demyelinating neuropathy
Peripheral Neuropathy	SNOMED	25362006	Hereditary motor and sensory neuropathy type IV
Peripheral Neuropathy	SNOMED	25690000	Femoral neuropathy
Peripheral Neuropathy	SNOMED	267601009	Other toxic or inflammatory neuropathy
Peripheral Neuropathy	SNOMED	277187008	Proximal median neuropathy
Peripheral Neuropathy	SNOMED	277879009	Autonomic neuropathy
Peripheral Neuropathy	SNOMED	29159009	Hereditary sensory and autonomic neuropathy, type III
Peripheral Neuropathy	SNOMED	292061000119100	Right common peroneal neuropathy
Peripheral Neuropathy	SNOMED	302226006	Peripheral neuropathy
Peripheral Neuropathy	SNOMED	309520003	Symmetrical sensory neuropathy
Peripheral Neuropathy	SNOMED	315056009	Pudendal nerve neuropathy
Peripheral Neuropathy	SNOMED	31524007	Polyneuropathy in mumps
Peripheral Neuropathy	SNOMED	33209009	Idiopathic progressive polyneuropathy
Peripheral Neuropathy	SNOMED	3548001	BPN - Brachial plexus neuropathy
Peripheral Neuropathy	SNOMED	359837005	Ulnar neuropathy
Peripheral Neuropathy	SNOMED	38311000033103	Treatment Of Diabetic Neuropathy
Peripheral Neuropathy	SNOMED	386033004	[X]Polyneuropathy in other diseases classified elsewhere
Peripheral Neuropathy	SNOMED	3900008	Mixed sensory-motor polyneuropathy
Peripheral Neuropathy	SNOMED	39058009	Diabetic lumbosacral radiculoplexus neuropathy
Peripheral Neuropathy	SNOMED	397828008	Median neuropathy
Peripheral Neuropathy	SNOMED	398040009	Inherited dominant hypertrophic neuropathy
Peripheral Neuropathy	SNOMED	398100001	Hereditary motor and sensory neuropathy
Peripheral Neuropathy	SNOMED	398187000	Hereditary motor and sensory neuropathy type II
Peripheral Neuropathy	SNOMED	398229007	Amyloid polyneuropathy type I
Peripheral Neuropathy	SNOMED	399081005	Common peroneal neuropathy
Peripheral Neuropathy	SNOMED	40956001	Guillain-Barre syndrome
Peripheral Neuropathy	SNOMED	420436000	Mononeuropathy co-occurrent and due to type 2 diabetes mellitus
Peripheral Neuropathy	SNOMED	420918009	Type I diabetes mellitus with mononeuropathy
Peripheral Neuropathy	SNOMED	422426003	Facial neuropathy
Peripheral Neuropathy	SNOMED	42295001	Familial amyloid polyneuropathy
Peripheral Neuropathy	SNOMED	42345000	Polyneuropathy unspecified
Peripheral Neuropathy	SNOMED	424736006	Diabetic peripheral neuropathy
Peripheral Neuropathy	SNOMED	425659003	Vasculitic neuropathy
Peripheral Neuropathy	SNOMED	443805006	Auditory neuropathy spectrum disorder
Peripheral Neuropathy	SNOMED	444728005	Chronic inflammatory demyelinating polyneuropathy
Peripheral Neuropathy	SNOMED	4463009	Familial amyloid polyneuropathy type II
Peripheral Neuropathy	SNOMED	45600000	Toxic neuropathy
Peripheral Neuropathy	SNOMED	45781009	Entrapment neuropathy
Peripheral Neuropathy	SNOMED	473134007	Symptomatic diabetic peripheral neuropathy absent
Peripheral Neuropathy	SNOMED	49455004	Diabetic polyneuropathy
Peripheral Neuropathy	SNOMED	50620007	Autonomic neuropathy with diabetes mellitus
Peripheral Neuropathy	SNOMED	52585001	Sciatic neuropathy
Peripheral Neuropathy	SNOMED	54404000	Cervical root neuropathy
Peripheral Neuropathy	SNOMED	54509008	Neuropathy due to nutritional factors
Peripheral Neuropathy	SNOMED	57406009	Distal median nerve entrapment
Peripheral Neuropathy	SNOMED	60576007	Vitamin B>12< deficiency neuropathy
Peripheral Neuropathy	SNOMED	60703000	Axonal neuropathy
Peripheral Neuropathy	SNOMED	609614000	Entrapment neuropathy of lower limb
Peripheral Neuropathy	SNOMED	636121000000109	Hereditary or idiopathic peripheral neuropathy NOS
Peripheral Neuropathy	SNOMED	65017003	Hereditary peripheral neuropathy
Peripheral Neuropathy	SNOMED	65389002	Adrenomyeloneuropathy
Peripheral Neuropathy	SNOMED	66695004	Erythroedema polyneuropathy
Peripheral Neuropathy	SNOMED	709489006	Small fibre neuropathy
Peripheral Neuropathy	SNOMED	713705003	Type 1 diabetes mellitus with polyneuropathy
Peripheral Neuropathy	SNOMED	713706002	Type 2 diabetes mellitus with polyneuropathy
Peripheral Neuropathy	SNOMED	715770009	Acute motor axonal neuropathy
Peripheral Neuropathy	SNOMED	716722005	Acute motor sensory axonal neuropathy
Peripheral Neuropathy	SNOMED	724810001	Radiculoplexoneuropathy due to diabetes mellitus
Peripheral Neuropathy	SNOMED	73013002	Cranial neuropathy
Peripheral Neuropathy	SNOMED	7339009	Drug/toxic polyneuropathy

Peripheral Neuropathy	SNOMED	763258007	Site, Ischemia, Neuropathy, Bacterial Infection and Depth classification total score
Peripheral Neuropathy	SNOMED	76462000	Postherpetic polyneuropathy
Peripheral Neuropathy	SNOMED	76886005	[X]Inflammatory polyneuropathy, unspecified
Peripheral Neuropathy	SNOMED	773001000000103	Symptomatic diabetic peripheral neuropathy
Peripheral Neuropathy	SNOMED	77659000	Polyneuropathy in malignant disease
Peripheral Neuropathy	SNOMED	777601000000102	No diabetic autonomic neuropathy
Peripheral Neuropathy	SNOMED	7916009	Alcoholic polyneuropathy
Peripheral Neuropathy	SNOMED	79389008	Neuropathy due to vitamin B1 deficiency
Peripheral Neuropathy	SNOMED	851271000006107	Compression neuropathy
Peripheral Neuropathy	SNOMED	85423005	Motor polyneuropathy
Peripheral Neuropathy	SNOMED	86489003	Idiopathic peripheral autonomic neuropathy
Peripheral Neuropathy	SNOMED	9133005	Familial amyloid polyneuropathy, Iowa type
Peripheral Neuropathy	SNOMED	95662005	Sensory neuropathy
Peripheral Neuropathy	SNOMED	980351000000105	Young Townson FootSkin Scale for Diabetic Neuropathy
Peripheral Vascular Disease	ICD10	I70	Atherosclerosis
Peripheral Vascular Disease	ICD10	I73	Other peripheral vascular diseases
Peripheral Vascular Disease	ICD10	I742	Peripheral Artery Disease
Peripheral Vascular Disease	ICD10	I743	Peripheral Artery Disease
Peripheral Vascular Disease	ICD10	I744	Peripheral Artery Disease
Peripheral Vascular Disease	ICD10	I745	Peripheral Artery Disease
Peripheral Vascular Disease	ICD10	I748	Peripheral Artery Disease
Peripheral Vascular Disease	ICD10	I749	Peripheral Artery Disease
Peripheral Vascular Disease	ICD10	I792	Peripheral Artery Disease
Peripheral Vascular Disease	ICD10	I798	Peripheral Artery Disease
Peripheral Vascular Disease	SNOMED	112828007	Replace aneurysm fem art by fem/pop a anast c vein graft NEC
Peripheral Vascular Disease	SNOMED	116360008	Other specified other bypass of iliac artery
Peripheral Vascular Disease	SNOMED	118805000	Other open operations on superficial femoral artery
Peripheral Vascular Disease	SNOMED	127014009	Diabetic peripheral angiopathy
Peripheral Vascular Disease	SNOMED	16589005	Endarterectomy of femoral artery NEC
Peripheral Vascular Disease	SNOMED	168953002	Femoral arteriogram abnormal
Peripheral Vascular Disease	SNOMED	168957001	Lower limb arteriogram abnorm.
Peripheral Vascular Disease	SNOMED	175275009	Other specified extraanatomic bypass of aorta
Peripheral Vascular Disease	SNOMED	175276005	Emerg aortic bypass by anastomosis axillary to femoral art
Peripheral Vascular Disease	SNOMED	175279003	Axillo-unifemoral PTFE bypass graft
Peripheral Vascular Disease	SNOMED	175348000	Open embolectomy of bifurcation of aorta
Peripheral Vascular Disease	SNOMED	175562008	Emerg replace aneurysm iliac art by iliac/femoral art anast
Peripheral Vascular Disease	SNOMED	175564009	Emerg replace aneurysmal iliac artery by fem/fem art anast
Peripheral Vascular Disease	SNOMED	175566006	Emerg replace aneurys com iliac a by aorta/com iliac a anast
Peripheral Vascular Disease	SNOMED	175567002	Emerg replace aneurys iliac art by aorta/ext iliac art anast
Peripheral Vascular Disease	SNOMED	175568007	Emerg replace aneurysm leg artery by aorta/com fem art anast
Peripheral Vascular Disease	SNOMED	175569004	Emerg replace aneurysm leg artery by aorta/sup fem art anast
Peripheral Vascular Disease	SNOMED	175570003	Emerg replace aneurysm iliac artery by iliac/iliac art anast
Peripheral Vascular Disease	SNOMED	175594003	Ilio-femoral prosthetic cross over graft
Peripheral Vascular Disease	SNOMED	175612005	Percutaneous transluminal angioplasty of iliac artery
Peripheral Vascular Disease	SNOMED	175613000	Percutaneous transluminal embolectomy of iliac artery
Peripheral Vascular Disease	SNOMED	175615007	Insertion of iliac artery stent
Peripheral Vascular Disease	SNOMED	175620007	Emerg replace aneurysm fem art by fem/pop art anast c prosth
Peripheral Vascular Disease	SNOMED	175621006	Emerg replace aneurysm pop art by pop/pop art anast c prosth
Peripheral Vascular Disease	SNOMED	175622004	Emerg replace aneurysm fem art by fem/pop anast c vein graft
Peripheral Vascular Disease	SNOMED	175623009	Emerg replace aneurys pop art by pop/pop a anast c vein graf
Peripheral Vascular Disease	SNOMED	175624003	Emerg replace aneurysm femoral art by fem/tib a anast c pros
Peripheral Vascular Disease	SNOMED	175625002	Emerg replace aneurysm pop art by pop/tib art anast c prosth
Peripheral Vascular Disease	SNOMED	175626001	Emerg replace aneurys fem art by fem/tib a anast c vein graf
Peripheral Vascular Disease	SNOMED	175627005	Emerg replace aneurysm pop art by pop/tib anast c vein graft
Peripheral Vascular Disease	SNOMED	175628000	Emerg replace aneurysm fem art by fem/peron a anast c prosth
Peripheral Vascular Disease	SNOMED	175629008	Emerg replace aneurysm pop art by pop/peron a anast c prosth
Peripheral Vascular Disease	SNOMED	175630003	Emerg replace aneur fem art by fem/peron a anast c vein graf
Peripheral Vascular Disease	SNOMED	175631004	Emerg replace aneur pop art by pop/peron a anast c vein graf
Peripheral Vascular Disease	SNOMED	175632006	Emerg replace aneurysm fem artery by fem/fem art anastomosis
Peripheral Vascular Disease	SNOMED	175633001	Emerg replace aneurysm pop artery by pop/fem art anastomosis
Peripheral Vascular Disease	SNOMED	175685004	Femoro-femoral prosthetic cross over graft
Peripheral Vascular Disease	SNOMED	175689005	Endarterectomy and patch repair of femoral artery
Peripheral Vascular Disease	SNOMED	175690001	Endarterectomy and patch repair of popliteal artery
Peripheral Vascular Disease	SNOMED	175693004	Profundoplasty femoral artery & patch repair deep fem artery
Peripheral Vascular Disease	SNOMED	175694005	Profundoplasty and patch repair of popliteal artery
Peripheral Vascular Disease	SNOMED	175697003	Reconstruction of femoral artery with vein graft
Peripheral Vascular Disease	SNOMED	175698008	Reconstruction of popliteal artery with vein graft
Peripheral Vascular Disease	SNOMED	175704008	Open thrombectomy of femoral artery
Peripheral Vascular Disease	SNOMED	175705009	Open thrombectomy of popliteal artery
Peripheral Vascular Disease	SNOMED	175706005	Ligation of aneurysm of popliteal artery
Peripheral Vascular Disease	SNOMED	175710008	Repair of femoral artery with temporary silastic shunt
Peripheral Vascular Disease	SNOMED	175711007	Repair of popliteal artery with temporary silastic shunt
Peripheral Vascular Disease	SNOMED	175715003	Percutaneous transluminal angioplasty of femoral artery
Peripheral Vascular Disease	SNOMED	175716002	Percutaneous transluminal angioplasty of popliteal artery

Peripheral Vascular Disease	SNOMED	175717006	Percutaneous transluminal embolectomy of femoral artery
Peripheral Vascular Disease	SNOMED	175718001	Percutaneous transluminal embolectomy of popliteal artery
Peripheral Vascular Disease	SNOMED	175719009	Percutaneous transluminal embolisation of femoral artery
Peripheral Vascular Disease	SNOMED	175720003	Percutaneous transluminal embolisation of popliteal artery
Peripheral Vascular Disease	SNOMED	175722006	Arteriography of popliteal artery
Peripheral Vascular Disease	SNOMED	175729002	Revision of reconstruction of artery NOS
Peripheral Vascular Disease	SNOMED	175730007	Revision of reconstruction involving aorta
Peripheral Vascular Disease	SNOMED	175731006	Revision of reconstruction involving iliac artery
Peripheral Vascular Disease	SNOMED	175732004	Revision of reconstruction involving femoral artery
Peripheral Vascular Disease	SNOMED	175733009	Revision of reconstruction of popliteal artery
Peripheral Vascular Disease	SNOMED	195254008	Extremity artery atheroma NOS
Peripheral Vascular Disease	SNOMED	195295006	Raynaud's syndrome
Peripheral Vascular Disease	SNOMED	195302000	[D]Gangrene of toe in diabetic
Peripheral Vascular Disease	SNOMED	195303005	[D]Widespread diabetic foot gangrene
Peripheral Vascular Disease	SNOMED	195318006	Peripheral arterial embolism and thrombosis NOS
Peripheral Vascular Disease	SNOMED	201250006	Ischaemic ulcer diabetic foot
Peripheral Vascular Disease	SNOMED	233304004	Endarterectomy of common iliac artery NEC
Peripheral Vascular Disease	SNOMED	233310004	Endarterectomy of popliteal artery NEC
Peripheral Vascular Disease	SNOMED	233345007	Reconstruction of common iliac artery
Peripheral Vascular Disease	SNOMED	233365000	Emergency replacement aneurysmal superficial femoral artery
Peripheral Vascular Disease	SNOMED	233366004	Emergency replacement of aneurysmal popliteal artery
Peripheral Vascular Disease	SNOMED	233367008	Emergency replacement of aneurysmal deep femoral artery
Peripheral Vascular Disease	SNOMED	233368003	Emergency replacement of aneurysmal common femoral artery
Peripheral Vascular Disease	SNOMED	233379008	Bypass aorta by anastomosis axillary to femoral artery NEC
Peripheral Vascular Disease	SNOMED	233380006	Bypass iliac artery by femoral/femoral art anastomosis NEC
Peripheral Vascular Disease	SNOMED	233383008	Replace aneurys fem art by fem/peron a anast c vein graf NEC
Peripheral Vascular Disease	SNOMED	233384002	Other bypass of popliteal artery
Peripheral Vascular Disease	SNOMED	233388004	Replacement of aneurysmal iliac artery by anastomosis
Peripheral Vascular Disease	SNOMED	233958001	Peripheral ischaemia
Peripheral Vascular Disease	SNOMED	233961000	Lower limb ischemia
Peripheral Vascular Disease	SNOMED	234481000000105	Scanning laser ophthalmoscopy evaluation of retina
Peripheral Vascular Disease	SNOMED	238793001	Ischaemic leg ulcer
Peripheral Vascular Disease	SNOMED	265511008	Iliac and femoral artery operations NOS
Peripheral Vascular Disease	SNOMED	265512001	Replacement of aneurysmal iliac artery
Peripheral Vascular Disease	SNOMED	265516003	Reconstruction of iliac artery
Peripheral Vascular Disease	SNOMED	265517007	Endarterectomy and patch repair of iliac artery
Peripheral Vascular Disease	SNOMED	265521000	Open embolectomy of iliac artery
Peripheral Vascular Disease	SNOMED	265522007	Transluminal operations on iliac artery
Peripheral Vascular Disease	SNOMED	265523002	Arteriography of iliac artery
Peripheral Vascular Disease	SNOMED	265524008	Emergency replacement aneurysmal femoral/popliteal artery OS
Peripheral Vascular Disease	SNOMED	265528006	Reconstruction of femoral or popliteal artery NOS
Peripheral Vascular Disease	SNOMED	265530008	Transluminal operation on femoral or popliteal artery NOS
Peripheral Vascular Disease	SNOMED	265531007	Arteriography of femoral artery
Peripheral Vascular Disease	SNOMED	275040003	Emergency femoro-femoral prosthetic cross over graft
Peripheral Vascular Disease	SNOMED	275041004	Endarterectomy and patch repair of common iliac artery
Peripheral Vascular Disease	SNOMED	275045008	Transluminal operations on common iliac artery
Peripheral Vascular Disease	SNOMED	275051003	Reconstruction of popliteal artery
Peripheral Vascular Disease	SNOMED	275052005	Reconstruction of deep femoral artery
Peripheral Vascular Disease	SNOMED	275053000	Reconstruction of common femoral artery
Peripheral Vascular Disease	SNOMED	275054006	Reconstruction of femoral artery
Peripheral Vascular Disease	SNOMED	275059001	Transluminal procedure on superficial femoral artery
Peripheral Vascular Disease	SNOMED	275060006	Transluminal procedure on common femoral artery
Peripheral Vascular Disease	SNOMED	275061005	Transluminal procedure on deep femoral artery
Peripheral Vascular Disease	SNOMED	275062003	Transluminal procedure on popliteal artery
Peripheral Vascular Disease	SNOMED	275063008	Transluminal procedure on femoral artery
Peripheral Vascular Disease	SNOMED	275145005	Reconstruction of superficial femoral artery
Peripheral Vascular Disease	SNOMED	275254000	Open embolectomy of common iliac artery
Peripheral Vascular Disease	SNOMED	275255004	Emerg replacement aneurysmal femoral/popl art by anastomosis
Peripheral Vascular Disease	SNOMED	275520000	Claudication
Peripheral Vascular Disease	SNOMED	301755001	Ischaemic foot
Peripheral Vascular Disease	SNOMED	307408003	Ischaemic toe
Peripheral Vascular Disease	SNOMED	307800001	Endarterectomy of iliac artery NEC
Peripheral Vascular Disease	SNOMED	308071004	H/O: Peripheral vascular disease procedure
Peripheral Vascular Disease	SNOMED	309449009	Percut translum thrombolysis femoral graft streptokinase
Peripheral Vascular Disease	SNOMED	31211000119101	Type I diabetes mellitus with peripheral angiopathy
Peripheral Vascular Disease	SNOMED	312226000	Open insertion of iliac artery stent
Peripheral Vascular Disease	SNOMED	312227009	Percutaneous transluminal insertion of iliac artery stent
Peripheral Vascular Disease	SNOMED	312688006	Bypass femoral artery by fem/pop art anast c prosthesis NEC
Peripheral Vascular Disease	SNOMED	314902007	Type 2 diabetes mellitus with peripheral angiopathy
Peripheral Vascular Disease	SNOMED	388997005	Axillo-bifemoral bypass graft
Peripheral Vascular Disease	SNOMED	399957001	Peripheral angiopathy
Peripheral Vascular Disease	SNOMED	400047006	Peripheral vascular disease (disorder)
Peripheral Vascular Disease	SNOMED	405375003	Repair of popliteal artery NEC
Peripheral Vascular Disease	SNOMED	405376002	Profundoplasty of popliteal artery NEC
Peripheral Vascular Disease	SNOMED	405463009	Other bypass of superficial femoral artery

Peripheral Vascular Disease	SNOMED	405469008	Other bypass of femoral artery or popliteal artery NOS
Peripheral Vascular Disease	SNOMED	405477007	Emergency bypass of iliac artery by unspecified anastomosis
Peripheral Vascular Disease	SNOMED	405482000	Bypass leg artery by aorta/deep femoral art anastomosis NEC
Peripheral Vascular Disease	SNOMED	405516005	Other bypass of femoral or popliteal artery by anastomosis
Peripheral Vascular Disease	SNOMED	420825003	Type 1 diabetes mellitus with gangrene
Peripheral Vascular Disease	SNOMED	421365002	Peripheral circulatory disorder associated with type I diabetes mellitus (disorder)
Peripheral Vascular Disease	SNOMED	421631007	Diabetes mellitus, adult with gangrene
Peripheral Vascular Disease	SNOMED	421895002	Peripheral circulatory disorder associated with diabetes mellitus (disorder)
Peripheral Vascular Disease	SNOMED	422166005	NIDDM with peripheral circulatory disorder
Peripheral Vascular Disease	SNOMED	422275004	Diabetes mellitus with gangrene
Peripheral Vascular Disease	SNOMED	426093007	Percutaneous transluminal insertion of stent femoral artery
Peripheral Vascular Disease	SNOMED	44662004	Replace aneurysm fem artery by fem/fem art anastomosis NEC
Peripheral Vascular Disease	SNOMED	447920002	Emerg bypass iliac artery by iliac/iliac art anastomosis NEC
Peripheral Vascular Disease	SNOMED	448323005	Bypass femoral artery by fem/peron a anast c vein graft NEC
Peripheral Vascular Disease	SNOMED	448529008	Bypass femoral artery by fem/tib art anast c vein graft NEC
Peripheral Vascular Disease	SNOMED	450703003	Repair of iliac artery NEC
Peripheral Vascular Disease	SNOMED	450704009	Repair of common iliac artery NEC
Peripheral Vascular Disease	SNOMED	50807007	Bypass femoral artery by fem/pop art anast c vein graft NEC
Peripheral Vascular Disease	SNOMED	51018000	Bypass iliac artery by iliac/femoral artery anastomosis NEC
Peripheral Vascular Disease	SNOMED	583731000000000	Peripheral arterial embolism and thrombosis NOS
Peripheral Vascular Disease	SNOMED	583731000000103	Peripheral arterial embolism and thrombosis NOS
Peripheral Vascular Disease	SNOMED	63491006	Intermittent claudication
Peripheral Vascular Disease	SNOMED	713412006	Ischaemic foot pain at rest
Peripheral Vascular Disease	SNOMED	713674006	Ischaemic foot pain when walking
Peripheral Vascular Disease	SNOMED	77558004	Other replacement of aneurysmal popliteal artery
Peripheral Vascular Disease	SNOMED	836711000000108	Ischaemic lower limb pain at rest (finding)
Post Traumatic Stress Disorder	ICD10	F431	Post-traumatic stress disorder
Post Traumatic Stress Disorder	SNOMED	1820311000006101	PCL-C - Repeated, disturbing memories, thoughts or images of a stressful experience from the past: A little bit
Post Traumatic Stress Disorder	SNOMED	1820321000006109	PCL-C - Repeated, disturbing memories, thoughts or images of a stressful experience from the past: Moderately
Post Traumatic Stress Disorder	SNOMED	1820341000006102	PCL-C - Repeated, disturbing memories, thoughts or images of a stressful experience from the past: Quite a bit
Post Traumatic Stress Disorder	SNOMED	1820371000006105	PCL-C - Repeated, disturbing memories, thoughts or images of a stressful experience from the past: Extremely
Post Traumatic Stress Disorder	SNOMED	1820401000006108	PCL-C - Repeated disturbing dreams of a stressful experience from the past: A little bit
Post Traumatic Stress Disorder	SNOMED	1820411000006106	PCL-C - Repeated disturbing dreams of a stressful experience from the past: Moderately
Post Traumatic Stress Disorder	SNOMED	1820421000006103	PCL-C - Repeated disturbing dreams of a stressful experience from the past: Quite a bit
Post Traumatic Stress Disorder	SNOMED	1820431000006100	PCL-C - Repeated disturbing dreams of a stressful experience from the past: Extremely
Post Traumatic Stress Disorder	SNOMED	1820441000006105	PCL-C - Suddenly acting or feeling as if a stressful experience from the past were happening again (as if reliving it): A little bit
Post Traumatic Stress Disorder	SNOMED	1820461000006109	PCL-C - Suddenly acting or feeling as if a stressful experience from the past were happening again (as if reliving it): Moderately
Post Traumatic Stress Disorder	SNOMED	1820471000006102	PCL-C - Suddenly acting or feeling as if a stressful experience from the past were happening again (as if reliving it): Quite a bit
Post Traumatic Stress Disorder	SNOMED	1820481000006104	PCL-C - Suddenly acting or feeling as if a stressful experience from the past were happening again (as if reliving it): Extremely
Post Traumatic Stress Disorder	SNOMED	1820491000006101	PCL-C - Feeling very upset when something reminded you of a stressful experience from the past: Not at all
Post Traumatic Stress Disorder	SNOMED	1820501000006109	PCL-C - Feeling very upset when something reminded you of a stressful experience from the past: A little bit
Post Traumatic Stress Disorder	SNOMED	1820511000006107	PCL-C - Feeling very upset when something reminded you of a stressful experience from the past: Moderately
Post Traumatic Stress Disorder	SNOMED	1820521000006104	PCL-C - Feeling very upset when something reminded you of a stressful experience from the past: Quite a bit
Post Traumatic Stress Disorder	SNOMED	1820531000006101	PCL-C - Feeling very upset when something reminded you of a stressful experience from the past: Extremely
Post Traumatic Stress Disorder	SNOMED	1820551000006108	PCL-C - Having a physical reaction when something reminded you of a stressful experience from the past: A little bit
Post Traumatic Stress Disorder	SNOMED	1820561000006105	PCL-C - Having a physical reaction when something reminded you of a stressful experience from the past: Moderately
Post Traumatic Stress Disorder	SNOMED	1820571000006103	PCL-C - Having a physical reaction when something reminded you of a stressful experience from the past: Quite a bit
Post Traumatic Stress Disorder	SNOMED	1820581000006100	PCL-C - Having a physical reaction when something reminded you of a stressful experience from the past: Extremely
Post Traumatic Stress Disorder	SNOMED	1820601000006105	PCL-C - Avoiding thinking about or talking about a stressful experience from the past or avoiding having feelings related to it: A little bit
Post Traumatic Stress Disorder	SNOMED	1820611000006108	PCL-C - Avoiding thinking about or talking about a stressful experience from the past or avoiding having feelings related to it: Moderately

Post Traumatic Stress Disorder	SNOMED	1820631000006102	PCL-C - Avoiding thinking about or talking about a stressful experience from the past or avoiding having feelings related to it: Quite a bit
Post Traumatic Stress Disorder	SNOMED	1820641000006107	PCL-C - Avoiding thinking about or talking about a stressful experience from the past or avoiding having feelings related to it: Extremely
Post Traumatic Stress Disorder	SNOMED	1820671000006104	PCL-C - Avoiding activities or situations because they reminded you of a stressful experience from the past: A little bit
Post Traumatic Stress Disorder	SNOMED	1820681000006101	PCL-C - Avoiding activities or situations because they reminded you of a stressful experience from the past: Moderately
Post Traumatic Stress Disorder	SNOMED	1820691000006103	PCL-C - Avoiding activities or situations because they reminded you of a stressful experience from the past: Quite a bit
Post Traumatic Stress Disorder	SNOMED	1820701000006103	PCL-C - Avoiding activities or situations because they reminded you of a stressful experience from the past: Extremely
Post Traumatic Stress Disorder	SNOMED	1820721000006108	PCL-C - Trouble remembering important parts of a stressful experience from the past: A little bit
Post Traumatic Stress Disorder	SNOMED	1820731000006106	PCL-C - Trouble remembering important parts of a stressful experience from the past: Moderately
Post Traumatic Stress Disorder	SNOMED	1820741000006101	PCL-C - Trouble remembering important parts of a stressful experience from the past: Quite a bit
Post Traumatic Stress Disorder	SNOMED	1820751000006104	PCL-C - Trouble remembering important parts of a stressful experience from the past: Extremely
Post Traumatic Stress Disorder	SNOMED	192042008	Acute posttrauma stress state
Post Traumatic Stress Disorder	SNOMED	1949511000006109	Phased intervention programme for complex PTSD
Post Traumatic Stress Disorder	SNOMED	1949521000006101	Phased intervention programme for complex PTSD - phase 1
Post Traumatic Stress Disorder	SNOMED	1949531000006103	Phased intervention programme for complex PTSD - phase 2
Post Traumatic Stress Disorder	SNOMED	1949541000006108	Phased intervention programme for complex PTSD - phase 3
Post Traumatic Stress Disorder	SNOMED	247807001	Situation avoidance behaviour
Post Traumatic Stress Disorder	SNOMED	30871003	Flashbacks
Post Traumatic Stress Disorder	SNOMED	313182004	Chronic post-traumatic stress disorder
Post Traumatic Stress Disorder	SNOMED	416073003	Long duration flashbacks
Post Traumatic Stress Disorder	SNOMED	416714005	Episodic flashbacks
Post Traumatic Stress Disorder	SNOMED	417143004	Short duration flashbacks
Post Traumatic Stress Disorder	SNOMED	417360004	Duplicative flashbacks
Post Traumatic Stress Disorder	SNOMED	443919007	Complex posttraumatic stress disorder
Post Traumatic Stress Disorder	SNOMED	47505003	PTSD - Post-traumatic stress disorder
Post Traumatic Stress Disorder	SNOMED	620181000000106	Other post-traumatic stress disorder
Post Traumatic Stress Disorder	SNOMED	848121000000104	Post-traumatic stress disorder impact of events scale
Post Traumatic Stress Disorder	SNOMED	848141000000106	Post-traumatic stress disorder impact of events scale score
Post Traumatic Stress Disorder	SNOMED	909701000006109	[RFC] Post traumatic stress disorder
Schizophrenia or Bipolar Disorder	ICD10	F062	Organic delusional [schizophrenia-like] disorder
Schizophrenia or Bipolar Disorder	ICD10	F20	Schizophrenia
Schizophrenia or Bipolar Disorder	ICD10	F21	Schizotypal disorder
Schizophrenia or Bipolar Disorder	ICD10	F22	Persistent delusional disorders
Schizophrenia or Bipolar Disorder	ICD10	F23	Acute and transient psychotic disorders
Schizophrenia or Bipolar Disorder	ICD10	F24	Induced delusional disorder
Schizophrenia or Bipolar Disorder	ICD10	F25	Schizoaffective disorders
Schizophrenia or Bipolar Disorder	ICD10	F28	Other nonorganic psychotic disorders
Schizophrenia or Bipolar Disorder	ICD10	F29	Unspecified nonorganic psychosis
Schizophrenia or Bipolar Disorder	ICD10	F30	manic episode
Schizophrenia or Bipolar Disorder	ICD10	F31	Bipolar affective disorder
Schizophrenia or Bipolar Disorder	ICD10	F340	Cyclothymia
Schizophrenia or Bipolar Disorder	SNOMED	1086471000000103	Recurrent reactive depressive episodes, severe, with psychosis (disorder)
Schizophrenia or Bipolar Disorder	SNOMED	1089481000000106	Cataleptic schizophrenia (disorder)
Schizophrenia or Bipolar Disorder	SNOMED	1089511000000100	Recurrent depression with current severe episode and psychotic features (disorder)
Schizophrenia or Bipolar Disorder	SNOMED	1089661000000104	Mania with mood-congruent psychotic features (disorder)
Schizophrenia or Bipolar Disorder	SNOMED	1089671000000106	Mania with mood-incongruent psychotic features (disorder)
Schizophrenia or Bipolar Disorder	SNOMED	1089681000000108	Mania with psychotic features (disorder)
Schizophrenia or Bipolar Disorder	SNOMED	1089691000000105	Acute predominantly delusional psychotic disorder (disorder)
Schizophrenia or Bipolar Disorder	SNOMED	111482003	Subchronic schizophrenia with acute exacerbations (disorder)
Schizophrenia or Bipolar Disorder	SNOMED	111483008	Catatonic schizophrenia in remission (disorder)
Schizophrenia or Bipolar Disorder	SNOMED	111484002	Undifferentiated schizophrenia (disorder)
Schizophrenia or Bipolar Disorder	SNOMED	111485001	Mixed bipolar I disorder in full remission (disorder)
Schizophrenia or Bipolar Disorder	SNOMED	1127191000000108	First episode psychosis (finding)
Schizophrenia or Bipolar Disorder	SNOMED	1204417003	Early onset schizophrenia (disorder)
Schizophrenia or Bipolar Disorder	SNOMED	1231333004	Psychostimulant dependence episodic (disorder)
Schizophrenia or Bipolar Disorder	SNOMED	1231336007	Psychostimulant dependence continuous (disorder)
Schizophrenia or Bipolar Disorder	SNOMED	1231503005	Psychotic disorder with insight present (disorder)
Schizophrenia or Bipolar Disorder	SNOMED	12939007	Chronic disorganized schizophrenia (disorder)
Schizophrenia or Bipolar Disorder	SNOMED	13313007	Mild bipolar disorder (disorder)
Schizophrenia or Bipolar Disorder	SNOMED	13746004	Bipolar disorder (disorder)
Schizophrenia or Bipolar Disorder	SNOMED	14291003	Subchronic disorganized schizophrenia with acute exacerbations (disorder)
Schizophrenia or Bipolar Disorder	SNOMED	1499003	Bipolar I disorder, single manic episode with postpartum onset (disorder)
Schizophrenia or Bipolar Disorder	SNOMED	162004	Severe manic bipolar I disorder without psychotic features (disorder)
Schizophrenia or Bipolar Disorder	SNOMED	16238741000119105	Bipolar disorder caused by drug (disorder)
Schizophrenia or Bipolar Disorder	SNOMED	16506000	Mixed bipolar I disorder (disorder)

Schizophrenia or Bipolar Disorder	SNOMED	16990005	Subchronic schizophrenia (disorder)
Schizophrenia or Bipolar Disorder	SNOMED	17782008	Bipolar I disorder, most recent episode manic with catatonic features (disorder)
Schizophrenia or Bipolar Disorder	SNOMED	191525009	Non-organic psychosis (disorder)
Schizophrenia or Bipolar Disorder	SNOMED	191526005	Schizophrenic disorders (disorder)
Schizophrenia or Bipolar Disorder	SNOMED	191527001	Simple schizophrenia (disorder)
Schizophrenia or Bipolar Disorder	SNOMED	191530008	Acute exacerbation of subchronic schizophrenia (disorder)
Schizophrenia or Bipolar Disorder	SNOMED	191531007	Acute exacerbation of chronic schizophrenia (disorder)
Schizophrenia or Bipolar Disorder	SNOMED	191538001	Acute exacerbation of subchronic hebephrenic schizophrenia (disorder)
Schizophrenia or Bipolar Disorder	SNOMED	191539009	Acute exacerbation of chronic hebephrenic schizophrenia (disorder)
Schizophrenia or Bipolar Disorder	SNOMED	191542003	Catatonic schizophrenia (disorder)
Schizophrenia or Bipolar Disorder	SNOMED	191547009	Acute exacerbation of subchronic catatonic schizophrenia (disorder)
Schizophrenia or Bipolar Disorder	SNOMED	191548004	Acute exacerbation of chronic catatonic schizophrenia (disorder)
Schizophrenia or Bipolar Disorder	SNOMED	191554003	Acute exacerbation of subchronic paranoid schizophrenia (disorder)
Schizophrenia or Bipolar Disorder	SNOMED	191555002	Acute exacerbation of chronic paranoid schizophrenia (disorder)
Schizophrenia or Bipolar Disorder	SNOMED	191559008	Latent schizophrenia (disorder)
Schizophrenia or Bipolar Disorder	SNOMED	191561004	Subchronic latent schizophrenia (disorder)
Schizophrenia or Bipolar Disorder	SNOMED	191562006	Chronic latent schizophrenia (disorder)
Schizophrenia or Bipolar Disorder	SNOMED	191563001	Acute exacerbation of subchronic latent schizophrenia (disorder)
Schizophrenia or Bipolar Disorder	SNOMED	191564007	Acute exacerbation of chronic latent schizophrenia (disorder)
Schizophrenia or Bipolar Disorder	SNOMED	191565008	Latent schizophrenia in remission (disorder)
Schizophrenia or Bipolar Disorder	SNOMED	191567000	Schizoaffective schizophrenia (disorder)
Schizophrenia or Bipolar Disorder	SNOMED	191569002	Subchronic schizoaffective schizophrenia (disorder)
Schizophrenia or Bipolar Disorder	SNOMED	191570001	Chronic schizoaffective schizophrenia (disorder)
Schizophrenia or Bipolar Disorder	SNOMED	191571002	Acute exacerbation of subchronic schizoaffective schizophrenia (disorder)
Schizophrenia or Bipolar Disorder	SNOMED	191572009	Acute exacerbation of chronic schizoaffective schizophrenia (disorder)
Schizophrenia or Bipolar Disorder	SNOMED	191574005	Schizoaffective schizophrenia in remission (disorder)
Schizophrenia or Bipolar Disorder	SNOMED	191577003	Cenesthopathic schizophrenia (disorder)
Schizophrenia or Bipolar Disorder	SNOMED	191583000	Single manic episode, mild (disorder)
Schizophrenia or Bipolar Disorder	SNOMED	191584006	Single manic episode, moderate (disorder)
Schizophrenia or Bipolar Disorder	SNOMED	191586008	Single manic episode, severe, with psychosis (disorder)
Schizophrenia or Bipolar Disorder	SNOMED	191588009	Single manic episode in full remission (disorder)
Schizophrenia or Bipolar Disorder	SNOMED	191590005	Recurrent manic episodes (disorder)
Schizophrenia or Bipolar Disorder	SNOMED	191592002	Recurrent manic episodes, mild (disorder)
Schizophrenia or Bipolar Disorder	SNOMED	191593007	Recurrent manic episodes, moderate (disorder)
Schizophrenia or Bipolar Disorder	SNOMED	191595000	Recurrent manic episodes, severe, with psychosis (disorder)
Schizophrenia or Bipolar Disorder	SNOMED	191597008	Recurrent manic episodes, in full remission (disorder)
Schizophrenia or Bipolar Disorder	SNOMED	191604000	Single major depressive episode, severe, with psychosis (disorder)
Schizophrenia or Bipolar Disorder	SNOMED	191613003	Recurrent major depressive episodes, severe, with psychosis (disorder)
Schizophrenia or Bipolar Disorder	SNOMED	191618007	Bipolar affective disorder, current episode manic (disorder)
Schizophrenia or Bipolar Disorder	SNOMED	191620005	Bipolar affective disorder, currently manic, mild (disorder)
Schizophrenia or Bipolar Disorder	SNOMED	191621009	Bipolar affective disorder, currently manic, moderate (disorder)
Schizophrenia or Bipolar Disorder	SNOMED	191623007	Bipolar affective disorder, currently manic, severe, with psychosis (disorder)
Schizophrenia or Bipolar Disorder	SNOMED	191625000	Bipolar affective disorder, currently manic, in full remission (disorder)
Schizophrenia or Bipolar Disorder	SNOMED	191627008	Bipolar affective disorder, current episode depression (disorder)
Schizophrenia or Bipolar Disorder	SNOMED	191629006	Bipolar affective disorder, currently depressed, mild (disorder)
Schizophrenia or Bipolar Disorder	SNOMED	191630001	Bipolar affective disorder, currently depressed, moderate (disorder)
Schizophrenia or Bipolar Disorder	SNOMED	191632009	Bipolar affective disorder, currently depressed, severe, with psychosis (disorder)
Schizophrenia or Bipolar Disorder	SNOMED	191634005	Bipolar affective disorder, currently depressed, in full remission (disorder)
Schizophrenia or Bipolar Disorder	SNOMED	191636007	Mixed bipolar affective disorder (disorder)
Schizophrenia or Bipolar Disorder	SNOMED	191638008	Mixed bipolar affective disorder, mild (disorder)
Schizophrenia or Bipolar Disorder	SNOMED	191639000	Mixed bipolar affective disorder, moderate (disorder)
Schizophrenia or Bipolar Disorder	SNOMED	191641004	Mixed bipolar affective disorder, severe, with psychosis (disorder)
Schizophrenia or Bipolar Disorder	SNOMED	191643001	Mixed bipolar affective disorder, in full remission (disorder)
Schizophrenia or Bipolar Disorder	SNOMED	191658009	Atypical manic disorder (disorder)
Schizophrenia or Bipolar Disorder	SNOMED	191667009	Paranoid disorder (disorder)
Schizophrenia or Bipolar Disorder	SNOMED	191668004	Simple paranoid state (disorder)
Schizophrenia or Bipolar Disorder	SNOMED	191670008	Shared paranoid disorder (disorder)
Schizophrenia or Bipolar Disorder	SNOMED	191672000	Paranoia querulans (disorder)
Schizophrenia or Bipolar Disorder	SNOMED	191676002	Reactive depressive psychosis (disorder)
Schizophrenia or Bipolar Disorder	SNOMED	191677006	Acute hysterical psychosis (disorder)
Schizophrenia or Bipolar Disorder	SNOMED	191680007	Psychogenic paranoid psychosis (disorder)
Schizophrenia or Bipolar Disorder	SNOMED	191683009	Psychogenic stupor (disorder)
Schizophrenia or Bipolar Disorder	SNOMED	192362008	Bipolar affective disorder, current episode mixed (disorder)
Schizophrenia or Bipolar Disorder	SNOMED	231437006	Reactive psychoses (disorder)
Schizophrenia or Bipolar Disorder	SNOMED	231449007	Epileptic psychosis (disorder)
Schizophrenia or Bipolar Disorder	SNOMED	231485007	Post-schizophrenic depression (disorder)
Schizophrenia or Bipolar Disorder	SNOMED	231487004	Persistent delusional disorder (disorder)
Schizophrenia or Bipolar Disorder	SNOMED	231489001	Acute transient psychotic disorder (disorder)
Schizophrenia or Bipolar Disorder	SNOMED	231494001	Mania (disorder)
Schizophrenia or Bipolar Disorder	SNOMED	231495000	Manic stupor (disorder)
Schizophrenia or Bipolar Disorder	SNOMED	231496004	Hypomania (disorder)
Schizophrenia or Bipolar Disorder	SNOMED	238972008	Cutaneous monosymptomatic delusional psychosis (disorder)
Schizophrenia or Bipolar Disorder	SNOMED	238973003	Delusions of parasitosis (disorder)

Schizophrenia or Bipolar Disorder	SNOMED	238974009	Delusions of infestation (disorder)
Schizophrenia or Bipolar Disorder	SNOMED	238975005	Delusion of foul odor (disorder)
Schizophrenia or Bipolar Disorder	SNOMED	238977002	Delusional hyperhidrosis (disorder)
Schizophrenia or Bipolar Disorder	SNOMED	247804008	Schizophrenic prodrome (disorder)
Schizophrenia or Bipolar Disorder	SNOMED	26025008	Residual schizophrenia (disorder)
Schizophrenia or Bipolar Disorder	SNOMED	26472000	Paraphrenia (disorder)
Schizophrenia or Bipolar Disorder	SNOMED	268617001	Acute schizophrenic episode (disorder)
Schizophrenia or Bipolar Disorder	SNOMED	268619003	Manic disorder, single episode (disorder)
Schizophrenia or Bipolar Disorder	SNOMED	268622001	Chronic paranoid psychosis (disorder)
Schizophrenia or Bipolar Disorder	SNOMED	268624000	Acute paranoid reaction (disorder)
Schizophrenia or Bipolar Disorder	SNOMED	270901009	Schizoaffective disorder, mixed type (disorder)
Schizophrenia or Bipolar Disorder	SNOMED	271428004	Schizoaffective disorder, manic type (disorder)
Schizophrenia or Bipolar Disorder	SNOMED	27387000	Subchronic disorganized schizophrenia (disorder)
Schizophrenia or Bipolar Disorder	SNOMED	274952002	Borderline schizophrenia (disorder)
Schizophrenia or Bipolar Disorder	SNOMED	274953007	Acute polymorphic psychotic disorder (disorder)
Schizophrenia or Bipolar Disorder	SNOMED	278506006	Involutional paranoid state (disorder)
Schizophrenia or Bipolar Disorder	SNOMED	278508007	Delusional dysmorphophobia (disorder)
Schizophrenia or Bipolar Disorder	SNOMED	278853003	Acute schizophrenia-like psychotic disorder (disorder)
Schizophrenia or Bipolar Disorder	SNOMED	280949006	Erotomania (disorder)
Schizophrenia or Bipolar Disorder	SNOMED	28475009	Severe recurrent major depression with psychotic features (disorder)
Schizophrenia or Bipolar Disorder	SNOMED	28663008	Severe manic bipolar I disorder with psychotic features (disorder)
Schizophrenia or Bipolar Disorder	SNOMED	29599000	Chronic undifferentiated schizophrenia (disorder)
Schizophrenia or Bipolar Disorder	SNOMED	30336007	Chronic residual schizophrenia with acute exacerbations (disorder)
Schizophrenia or Bipolar Disorder	SNOMED	307417003	Cycloid psychosis (disorder)
Schizophrenia or Bipolar Disorder	SNOMED	307504004	Oneirophrenia (finding)
Schizophrenia or Bipolar Disorder	SNOMED	31027006	Schizotypal personality disorder (disorder)
Schizophrenia or Bipolar Disorder	SNOMED	31373002	Disorganized schizophrenia in remission (disorder)
Schizophrenia or Bipolar Disorder	SNOMED	31446002	Bipolar I disorder, most recent episode hypomanic (disorder)
Schizophrenia or Bipolar Disorder	SNOMED	31658008	Chronic paranoid schizophrenia (disorder)
Schizophrenia or Bipolar Disorder	SNOMED	33323008	Somatic delusion disorder (disorder)
Schizophrenia or Bipolar Disorder	SNOMED	35218008	Chronic disorganized schizophrenia with acute exacerbation (disorder)
Schizophrenia or Bipolar Disorder	SNOMED	35252006	Disorganized schizophrenia (disorder)
Schizophrenia or Bipolar Disorder	SNOMED	371596008	Bipolar I disorder (disorder)
Schizophrenia or Bipolar Disorder	SNOMED	38295006	Involutional paraphrenia (disorder)
Schizophrenia or Bipolar Disorder	SNOMED	38368003	Schizoaffective disorder, bipolar type (disorder)
Schizophrenia or Bipolar Disorder	SNOMED	39610001	Undifferentiated schizophrenia in remission (disorder)
Schizophrenia or Bipolar Disorder	SNOMED	397711000000100	[X]Severe depressive episode with psychotic symptoms (disorder)
Schizophrenia or Bipolar Disorder	SNOMED	416340002	Late onset schizophrenia (disorder)
Schizophrenia or Bipolar Disorder	SNOMED	417601000000102	[X]Schizophrenia, schizotypal and delusional disorders (disorder)
Schizophrenia or Bipolar Disorder	SNOMED	41836007	Bipolar disorder in full remission (disorder)
Schizophrenia or Bipolar Disorder	SNOMED	41932008	Amok (disorder)
Schizophrenia or Bipolar Disorder	SNOMED	42868002	Subchronic catatonic schizophrenia (disorder)
Schizophrenia or Bipolar Disorder	SNOMED	441704009	Affective psychosis (disorder)
Schizophrenia or Bipolar Disorder	SNOMED	4441000	Severe bipolar disorder with psychotic features (disorder)
Schizophrenia or Bipolar Disorder	SNOMED	48500005	Delusional disorder (disorder)
Schizophrenia or Bipolar Disorder	SNOMED	4926007	Schizophrenia in remission (disorder)
Schizophrenia or Bipolar Disorder	SNOMED	51133006	Residual schizophrenia in remission (disorder)
Schizophrenia or Bipolar Disorder	SNOMED	53049002	Severe bipolar disorder without psychotic features (disorder)
Schizophrenia or Bipolar Disorder	SNOMED	53607008	Depressed bipolar I disorder in remission (disorder)
Schizophrenia or Bipolar Disorder	SNOMED	5464005	Brief reactive psychosis (disorder)
Schizophrenia or Bipolar Disorder	SNOMED	55516002	Bipolar I disorder, most recent episode manic with postpartum onset (disorder)
Schizophrenia or Bipolar Disorder	SNOMED	58214004	Schizophrenia (disorder)
Schizophrenia or Bipolar Disorder	SNOMED	60401000119104	Postpartum psychosis in remission (disorder)
Schizophrenia or Bipolar Disorder	SNOMED	61403008	Severe depressed bipolar I disorder without psychotic features (disorder)
Schizophrenia or Bipolar Disorder	SNOMED	61831009	Induced psychotic disorder (disorder)
Schizophrenia or Bipolar Disorder	SNOMED	63181006	Paranoid schizophrenia in remission (disorder)
Schizophrenia or Bipolar Disorder	SNOMED	63204009	Bouff+®e d+®lirante (disorder)
Schizophrenia or Bipolar Disorder	SNOMED	63249007	Manic bipolar I disorder in partial remission (disorder)
Schizophrenia or Bipolar Disorder	SNOMED	64905009	Paranoid schizophrenia (disorder)
Schizophrenia or Bipolar Disorder	SNOMED	65971000052100	Acute psychosis (disorder)
Schizophrenia or Bipolar Disorder	SNOMED	68890003	Schizoaffective disorder (disorder)
Schizophrenia or Bipolar Disorder	SNOMED	68995007	Chronic catatonic schizophrenia (disorder)
Schizophrenia or Bipolar Disorder	SNOMED	69322001	Psychotic disorder (disorder)
Schizophrenia or Bipolar Disorder	SNOMED	7025000	Subchronic undifferentiated schizophrenia with acute exacerbations (disorder)
Schizophrenia or Bipolar Disorder	SNOMED	70814008	Subchronic residual schizophrenia with acute exacerbations (disorder)
Schizophrenia or Bipolar Disorder	SNOMED	71103003	Chronic residual schizophrenia (disorder)
Schizophrenia or Bipolar Disorder	SNOMED	712824002	Acute polymorphic psychotic disorder without symptoms of schizophrenia (disorder)
Schizophrenia or Bipolar Disorder	SNOMED	712850003	Acute polymorphic psychotic disorder co-occurrent with symptoms of schizophrenia (disorder)
Schizophrenia or Bipolar Disorder	SNOMED	73867007	Severe major depression with psychotic features (disorder)
Schizophrenia or Bipolar Disorder	SNOMED	755311000000100	Non-organic psychosis in remission (disorder)

Schizophrenia or Bipolar Disorder	SNOMED	755321000000106	Single major depressive episode, severe, with psychosis, psychosis in remission (disorder)
Schizophrenia or Bipolar Disorder	SNOMED	755331000000108	Recurrent major depressive episodes, severe, with psychosis, psychosis in remission (disorder)
Schizophrenia or Bipolar Disorder	SNOMED	760721000000109	Mixed bipolar affective disorder, in partial remission (disorder)
Schizophrenia or Bipolar Disorder	SNOMED	764591000000108	Mixed bipolar affective disorder, severe (disorder)
Schizophrenia or Bipolar Disorder	SNOMED	764621000000106	Recurrent manic episodes, severe (disorder)
Schizophrenia or Bipolar Disorder	SNOMED	764641000000104	Single manic episode, severe (disorder)
Schizophrenia or Bipolar Disorder	SNOMED	764671000000105	Recurrent manic episodes, in partial remission (disorder)
Schizophrenia or Bipolar Disorder	SNOMED	764681000000107	Recurrent manic episodes, in remission (disorder)
Schizophrenia or Bipolar Disorder	SNOMED	764731000000103	Single manic episode in partial remission (disorder)
Schizophrenia or Bipolar Disorder	SNOMED	764741000000107	Single manic episode in remission (disorder)
Schizophrenia or Bipolar Disorder	SNOMED	765176007	Psychosis and severe depression co-occurrent and due to bipolar affective disorder (disorder)
Schizophrenia or Bipolar Disorder	SNOMED	76566000	Subchronic residual schizophrenia (disorder)
Schizophrenia or Bipolar Disorder	SNOMED	767631007	Bipolar disorder, most recent episode depression (disorder)
Schizophrenia or Bipolar Disorder	SNOMED	767632000	Bipolar disorder, most recent episode manic (disorder)
Schizophrenia or Bipolar Disorder	SNOMED	767633005	Bipolar affective disorder, most recent episode mixed (disorder)
Schizophrenia or Bipolar Disorder	SNOMED	767635003	Bipolar I disorder, most recent episode manic (disorder)
Schizophrenia or Bipolar Disorder	SNOMED	767636002	Bipolar I disorder, most recent episode depression (disorder)
Schizophrenia or Bipolar Disorder	SNOMED	77475008	Jealous delusion disorder (disorder)
Schizophrenia or Bipolar Disorder	SNOMED	789061003	Rapid cycling bipolar II disorder (disorder)
Schizophrenia or Bipolar Disorder	SNOMED	79204003	Chronic undifferentiated schizophrenia with acute exacerbations (disorder)
Schizophrenia or Bipolar Disorder	SNOMED	79584002	Moderate bipolar disorder (disorder)
Schizophrenia or Bipolar Disorder	SNOMED	79866005	Subchronic paranoid schizophrenia (disorder)
Schizophrenia or Bipolar Disorder	SNOMED	83225003	Bipolar II disorder (disorder)
Schizophrenia or Bipolar Disorder	SNOMED	83746006	Chronic schizophrenia (disorder)
Schizophrenia or Bipolar Disorder	SNOMED	84760002	Schizoaffective disorder, depressive type (disorder)
Schizophrenia or Bipolar Disorder	SNOMED	85248005	Bipolar disorder in remission (disorder)
Schizophrenia or Bipolar Disorder	SNOMED	85861002	Subchronic undifferentiated schizophrenia (disorder)
Schizophrenia or Bipolar Disorder	SNOMED	87950005	Bipolar I disorder, single manic episode with catatonic features (disorder)
Schizophrenia or Bipolar Disorder	SNOMED	88975006	Schizophreniform disorder (disorder)
Schizophrenia or Bipolar Disorder	SNOMED	SNOMED_conceptId	SNOMED_conceptId_description
Sickle Cell Disease	SNOMED	127040003	Sickle-cell anaemia
Sickle Cell Disease	SNOMED	127041004	Sickle-cell thalassaemia
Sickle Cell Disease	SNOMED	13886001	Sickle cell nephropathy
Sickle Cell Disease	SNOMED	193370005	Proliferative retinopathy due to sickle cell disease
Sickle Cell Disease	SNOMED	2005411000006106	Sickle cell disease annual review
Sickle Cell Disease	SNOMED	25472008	Sickle-cell anaemia with haemoglobin D disease
Sickle Cell Disease	SNOMED	35434009	Sickle cell haemoglobin C
Sickle Cell Disease	SNOMED	416180004	Sickle-cell anaemia with no crisis
Sickle Cell Disease	SNOMED	417357006	[X]Other sickle-cell disorders
Sickle Cell Disease	SNOMED	417425009	Sickle-cell anaemia with crisis
Sickle Cell Disease	SNOMED	441482006	History of sickle cell anaemia
Sickle Cell Disease	SNOMED	47024008	Sickle-cell anaemia with haemoglobin E disease
Sickle Cell Disease	SNOMED	940531000006106	H/O: sickle cell disease
Sickle Cell Disease	SNOMED	955951000006104	Unfit for decompression - sickle cell anaemia
Stroke	ICD10	G951	Avascular myelopathies (arterial or venous)
Stroke	ICD10	I61	Nontraumatic intracerebral hemorrhage
Stroke	ICD10	I63	Cerebral infarction
Stroke	ICD10	I64	Stroke, not specified as haemorrhage or infarction
Stroke	SNOMED	1078001000000105	Haemorrhagic stroke (disorder)
Stroke	SNOMED	1089411000000104	Cerebral infarction due to occlusion of cerebral artery (disorder)
Stroke	SNOMED	1089421000000105	Cerebral infarction due to stenosis of cerebral artery (disorder)
Stroke	SNOMED	125081000119106	Cerebral infarction due to occlusion of precerebral artery
Stroke	SNOMED	140921000119102	Ischaemic stroke without coma
Stroke	SNOMED	16000511000119103	Cerebrovascular accident due to occlusion of left middle cerebral artery (disorder)
Stroke	SNOMED	16002031000119102	Cerebrovascular accident due to thrombus of right middle cerebral artery (disorder)
Stroke	SNOMED	16002111000119106	Cerebrovascular accident due to thrombus of left middle cerebral artery (disorder)
Stroke	SNOMED	16371781000119100	Cerebellar stroke (disorder)
Stroke	SNOMED	195165005	Basal ganglia hemorrhage
Stroke	SNOMED	195167002	External capsule hemorrhage
Stroke	SNOMED	195168007	Intracerebral haemorrhage, intraventricular
Stroke	SNOMED	195169004	Intracerebral hemorrhage, multiple localized
Stroke	SNOMED	195185009	Cerebral infarct due to thrombosis of precerebral arteries
Stroke	SNOMED	195186005	Cerebral infarction due to embolism of precerebral arteries
Stroke	SNOMED	195189003	Cerebral infarction due to thrombosis of cerebral arteries
Stroke	SNOMED	195190007	Cerebral infarction due to embolism of cerebral arteries
Stroke	SNOMED	195200006	Carotid artery syndrome hemispheric
Stroke	SNOMED	195201005	Multiple and bilateral precerebral artery syndromes
Stroke	SNOMED	195209007	Middle cerebral artery syndrome (disorder)
Stroke	SNOMED	195210002	Anterior cerebral artery syndrome (disorder)

Stroke	SNOMED	195211003	Posterior cerebral artery syndrome
Stroke	SNOMED	195212005	Brainstem stroke syndrome
Stroke	SNOMED	195213000	Cerebellar stroke syndrome
Stroke	SNOMED	195216008	Left sided cerebral hemisphere cerebrovascular accident
Stroke	SNOMED	195217004	Right sided CVA
Stroke	SNOMED	20059004	Occlusion of cerebral artery (disorder)
Stroke	SNOMED	20908003	Subcortical cerebral hemorrhage (disorder)
Stroke	SNOMED	230690007	CVA - Cerebrovascular accident
Stroke	SNOMED	230691006	CVA - cerebrovascular accident due to cerebral artery occlusion
Stroke	SNOMED	230692004	Infarction - precerebral
Stroke	SNOMED	230693009	Anterior cerebral circulation infarction (disorder)
Stroke	SNOMED	230694003	Total anterior cerebral circulation infarction (disorder)
Stroke	SNOMED	230695002	Partial anterior cerebral circulation infarction (disorder)
Stroke	SNOMED	230696001	Posterior cerebral circulation infarction (disorder)
Stroke	SNOMED	230698000	Lacunar infarction
Stroke	SNOMED	230699008	Pure motor lacunar syndrome
Stroke	SNOMED	230700009	Pure sensory lacunar infarction
Stroke	SNOMED	230701008	Pure sensorimotor lacunar infarction (disorder)
Stroke	SNOMED	230702001	Lacunar ataxic hemiparesis (disorder)
Stroke	SNOMED	230703006	Dysarthria-clumsy hand syndrome (disorder)
Stroke	SNOMED	230704000	Multi-infarct state (disorder)
Stroke	SNOMED	230706003	Hemorrhagic cerebral infarction (disorder)
Stroke	SNOMED	230707007	Anterior cerebral circulation hemorrhagic infarction (disorder)
Stroke	SNOMED	230708002	Posterior cerebral circulation hemorrhagic infarction (disorder)
Stroke	SNOMED	230709005	Massive supratentorial cerebral hemorrhage (disorder)
Stroke	SNOMED	230710000	Lobar cerebral hemorrhage
Stroke	SNOMED	230711001	Thalamic hemorrhage (disorder)
Stroke	SNOMED	230712008	Lacunar hemorrhage (disorder)
Stroke	SNOMED	230713003	Stroke of uncertain pathology (disorder)
Stroke	SNOMED	230714009	Anterior circulation stroke of uncertain pathology (disorder)
Stroke	SNOMED	230715005	Posterior circulation stroke of uncertain pathology (disorder)
Stroke	SNOMED	24654003	Weber-Gubler syndrome (disorder)
Stroke	SNOMED	25133001	Completed stroke (disorder)
Stroke	SNOMED	274100004	Cerebral hemorrhage
Stroke	SNOMED	275434003	Stroke in the puerperium
Stroke	SNOMED	276219001	Occipital cerebral infarction (disorder)
Stroke	SNOMED	276220007	Foville syndrome (disorder)
Stroke	SNOMED	276221006	Millard-Gubler syndrome (disorder)
Stroke	SNOMED	276222004	Top of basilar syndrome (disorder)
Stroke	SNOMED	276722003	Intracerebellar and posterior fossa hemorrhage (disorder)
Stroke	SNOMED	281240008	Extension of cerebrovascular accident (disorder)
Stroke	SNOMED	297138001	Embolus of circle of Willis (disorder)
Stroke	SNOMED	307363008	Multiple lacunar infarcts (disorder)
Stroke	SNOMED	307766002	Left sided cerebral infarction
Stroke	SNOMED	307767006	Right sided cerebral infarction
Stroke	SNOMED	308128006	Right sided intracerebral hemorrhage, unspecified
Stroke	SNOMED	329641000119104	Cerebrovascular accident due to thrombus of basilar artery (disorder)
Stroke	SNOMED	371041009	Embolic stroke (disorder)
Stroke	SNOMED	373606000	Occlusive stroke (disorder)
Stroke	SNOMED	413102000	Infarction of basal ganglia (disorder)
Stroke	SNOMED	413758000	Cardioembolic stroke (disorder)
Stroke	SNOMED	426107000	Acute lacunar infarction (disorder)
Stroke	SNOMED	432504007	Cerebral infarction (disorder)
Stroke	SNOMED	444172003	Recurrent transient cerebral ischemic attack (disorder)
Stroke	SNOMED	49422009	Cortical hemorrhage
Stroke	SNOMED	52201006	Internal capsule hemorrhage
Stroke	SNOMED	71444005	Cerebral thrombosis
Stroke	SNOMED	724424009	Cerebral ischemic stroke due to small artery occlusion (disorder)
Stroke	SNOMED	732923001	Hemorrhage of medulla oblongata (disorder)
Stroke	SNOMED	734383005	Thrombosis of left middle cerebral artery (disorder)
Stroke	SNOMED	734384004	Thrombosis of right middle cerebral artery (disorder)
Stroke	SNOMED	75038005	Cerebellar hemorrhage
Stroke	SNOMED	75543006	Cerebral embolism
Stroke	SNOMED	7713009	Intrapontine hemorrhage
Stroke	SNOMED	78569004	Posterior inferior cerebellar artery syndrome
Stroke	SNOMED	87555007	Claude's syndrome (disorder)
Stroke	SNOMED	90099008	Subcortical leukoencephalopathy (disorder)
Stroke	SNOMED	95454007	Brain stem hemorrhage (disorder)
Stroke	SNOMED	95457000	Brain stem infarction
Stroke	SNOMED	95460007	Cerebellar infarction
Thyroid Disease	ICD10	E001	Congenital iodine-deficiency syndrome, myxoedematous type
Thyroid Disease	ICD10	E030	Other hypothyroidism
Thyroid Disease	ICD10	E050	Thyrotoxicosis [hyperthyroidism]
Thyroid Disease	ICD10	E890	Postprocedural hypothyroidism
Thyroid Disease	ICD10	G132	Systemic atrophy primarily affecting central nervous system in myxoedema

Thyroid Disease	SNOMED	111566002	Hypothyroidism-congen.+ acqui.
Thyroid Disease	SNOMED	12330002	Complete parathyroidectomy
Thyroid Disease	SNOMED	161442007	History of hyperthyroidism
Thyroid Disease	SNOMED	161443002	H/O: hypothyroidism
Thyroid Disease	SNOMED	161512007	H/O: pulmonary embolus
Thyroid Disease	SNOMED	175262009	Trendelenburg pulmonary embolectomy
Thyroid Disease	SNOMED	175268008	Transluminal pulmonary embolectomy
Thyroid Disease	SNOMED	1823931000006100	Hypothyroidism confirmed
Thyroid Disease	SNOMED	1874471000006105	Newborn blood spot outcome status (congenital hypothyroidism)
Thyroid Disease	SNOMED	1877411000006108	Hypothyroidism clinical management plan no longer in place
Thyroid Disease	SNOMED	190241003	Toxic diffuse goitre with no crisis
Thyroid Disease	SNOMED	190242005	Toxic diffuse goitre with crisis
Thyroid Disease	SNOMED	190244006	Toxic uninodular goitre with no crisis
Thyroid Disease	SNOMED	190247004	Toxic multinodular goitre with no crisis
Thyroid Disease	SNOMED	190255006	Thyrotoxicosis from ectopic thyroid nodule with no crisis
Thyroid Disease	SNOMED	190256007	Thyrotoxicosis from ectopic thyroid nodule with crisis
Thyroid Disease	SNOMED	190268003	Other specified congenital hypothyroidism
Thyroid Disease	SNOMED	190277005	Irradiation hypothyroidism
Thyroid Disease	SNOMED	190279008	Iodine hypothyroidism
Thyroid Disease	SNOMED	190282003	Hypothyroidism resulting from para-aminosalicylic acid
Thyroid Disease	SNOMED	190283008	Hypothyroidism resulting from phenylbutazone
Thyroid Disease	SNOMED	190284002	Hypothyroidism resulting from resorcinol
Thyroid Disease	SNOMED	190291004	Premature puberty due to hypothyroidism
Thyroid Disease	SNOMED	190309006	Thyroid atrophy
Thyroid Disease	SNOMED	192816007	Cerebral degeneration due to myxoedema
Thyroid Disease	SNOMED	192876003	Cerebellar ataxia due to myxoedema
Thyroid Disease	SNOMED	193212008	Myasthenic syndrome due to hypothyroidism
Thyroid Disease	SNOMED	194883006	Post operative pulmonary embolus
Thyroid Disease	SNOMED	19885005	Thyrotoxic exophthalmos
Thyroid Disease	SNOMED	200284000	Obstetric pulmonary embolism
Thyroid Disease	SNOMED	200299000	Obstetric blood-clot pulmonary embolism NOS
Thyroid Disease	SNOMED	200301007	Obstetric blood-clot pulmonary embolism - delivered
Thyroid Disease	SNOMED	200303005	Obstetric blood-clot pulmonary embolism + a/n complication
Thyroid Disease	SNOMED	200304004	Obstetric blood-clot pulmonary embolism + p/n complication
Thyroid Disease	SNOMED	200308001	Obstetric pyaemic and septic pulmonary embolism - delivered
Thyroid Disease	SNOMED	200309009	Obstetric pyemic and septic pulmonary embolism - delivered with postnatal complication
Thyroid Disease	SNOMED	200310004	Obstetric pyemic and septic pulmonary embolism with antenatal complication
Thyroid Disease	SNOMED	200311000	Obstetric pyemic and septic pulmonary embolism with postnatal complication
Thyroid Disease	SNOMED	206457007	Neonatal jaundice with congenital hypothyroidism
Thyroid Disease	SNOMED	21263006	Myxoedema coma
Thyroid Disease	SNOMED	217710005	Cretinism
Thyroid Disease	SNOMED	237498007	Toxic goitre
Thyroid Disease	SNOMED	237515009	Congenital hypothyroidism without goitre
Thyroid Disease	SNOMED	237519003	Autoimmune myxoedema
Thyroid Disease	SNOMED	237527007	Other postablative hypothyroidism
Thyroid Disease	SNOMED	237528002	Postinfectious hypothyroidism
Thyroid Disease	SNOMED	237558008	Acquired atrophy of thyroid
Thyroid Disease	SNOMED	237565000	Endemic cretinism - mixed type
Thyroid Disease	SNOMED	237566004	Endemic cretinism - neurological type
Thyroid Disease	SNOMED	237825005	Graves' dermopathy
Thyroid Disease	SNOMED	23853001	[X]Systemic atrophy affecting the CNS in myxoedema
Thyroid Disease	SNOMED	24443003	Complete thyroidectomy
Thyroid Disease	SNOMED	248341000000100	Hypothyroidism monitoring telephone invitation
Thyroid Disease	SNOMED	26389007	Toxic multinodular goitre
Thyroid Disease	SNOMED	267284008	Obstetric pyaemic and septic pulmonary embolism unspecified
Thyroid Disease	SNOMED	267374005	Toxic diffuse goitre
Thyroid Disease	SNOMED	27059002	Postsurgical hypothyroidism
Thyroid Disease	SNOMED	276177000	Graves' ophthalmopathy
Thyroid Disease	SNOMED	278503003	Congenital hypothyroidism with diffuse goitre
Thyroid Disease	SNOMED	286909009	Thyrotoxicosis +/- goitre
Thyroid Disease	SNOMED	29028009	Thyrotoxic crisis
Thyroid Disease	SNOMED	341861000000109	Hypothyroidism annual review
Thyroid Disease	SNOMED	34486009	Hyperthyroidism
Thyroid Disease	SNOMED	353295004	Graves' disease
Thyroid Disease	SNOMED	40930008	Hypothyroidism
Thyroid Disease	SNOMED	414439002	Hypothyroidism monitoring first letter
Thyroid Disease	SNOMED	414441001	Hypothyroidism monitoring second letter
Thyroid Disease	SNOMED	414442008	Hypothyroidism monitoring third letter
Thyroid Disease	SNOMED	416326003	Hypothyroidism monitoring verbal invite
Thyroid Disease	SNOMED	416477006	Hyperthyroidism resolved
Thyroid Disease	SNOMED	426900002	Pulmonary thromboendarterectomy
Thyroid Disease	SNOMED	43153006	Myxoedema

Thyroid Disease	SNOMED	438773007	Recurrent pulmonary embolism
Thyroid Disease	SNOMED	52826006	Total substernal thyroidectomy
Thyroid Disease	SNOMED	57777000	Toxic nodular goitre unspecified
Thyroid Disease	SNOMED	59282003	Pulmonary embolus
Thyroid Disease	SNOMED	60446003	Thyrotoxic heart disease
Thyroid Disease	SNOMED	609480009	Induced termination of pregnancy complicated by pulmonary embolism
Thyroid Disease	SNOMED	62278002	Toxic multinodular goiter with thyrotoxic storm
Thyroid Disease	SNOMED	64662007	Infarction - pulmonary
Thyroid Disease	SNOMED	654491000000108	Other acquired hypothyroidism
Thyroid Disease	SNOMED	681051000000104	Hypothyroidism NOS
Thyroid Disease	SNOMED	68437005	Thyrotoxic myopathy
Thyroid Disease	SNOMED	69329005	Toxic uninodular goitre with thyrotoxic storm
Thyroid Disease	SNOMED	70348004	Hypothyroidism with sensorineural deafness
Thyroid Disease	SNOMED	713741000000000	Hypothyroidism monitoring administration
Thyroid Disease	SNOMED	713741000000106	Hypothyroidism monitoring administration
Thyroid Disease	SNOMED	736287007	Hypothyroidism clinical management plan
Thyroid Disease	SNOMED	73869005	Toxic uninodular goitre
Thyroid Disease	SNOMED	75065003	Endemic cretinism - hypothyroid
Thyroid Disease	SNOMED	82598004	Hypothyrotropic hypothyroidism
Thyroid Disease	SNOMED	83728000	Primary hypothyroidism AND adrenocortical insufficiency
Thyroid Disease	SNOMED	87232008	Thyrotoxicosis from ectopic thyroid tissue
Thyroid Disease	SNOMED	87844004	Myopathy due to myxoedema
Thyroid Disease	SNOMED	88273006	Iatrogenic hypothyroidism NOS
Thyroid Disease	SNOMED	905451000006102	[RFC] Pulmonary embolism/pulmonary hypertension
Thyroid Disease	SNOMED	905741000006108	[RFC] Hyperthyroidism
Thyroid Disease	SNOMED	90739004	Thyrotoxicosis
Thyroid Disease	SNOMED	92978002	Aplasia of thyroid with myxoedema
Thyroid Disease	SNOMED	939981000006105	Hypothyroidism medication review
Thyroid Disease	SNOMED	940091000000000	Hypothyroidism monitoring administration
Thyroid Disease	SNOMED	940091000006104	Hypothyroidism monitoring administration
Thyroid Disease	SNOMED	940101000006105	Hypothyroidism monitoring invite 1
Thyroid Disease	SNOMED	940111000006108	Hypothyroidism monitoring invite 2
Thyroid Disease	SNOMED	940121000006100	Hypothyroidism monitoring invite 3
Transient Ischemic Attack	ICD10	G450	Transient cerebral ischaemic attacks and related syndromes
Transient Ischemic Attack	SNOMED	195206000	Intermittent cerebral ischaemia
Transient Ischemic Attack	SNOMED	230716006	Anterior circulation transient ischaemic attack
Transient Ischemic Attack	SNOMED	230717002	Vertebrobasilar territory transient ischemic attack (disorder)
Transient Ischemic Attack	SNOMED	266257000	TIA
Tuberculosis	ICD10	A150	Tuberculosis of lung, confirmed by sputum microscopy with or without culture
Tuberculosis	ICD10	A151	Tuberculosis of lung, confirmed by culture only
Tuberculosis	ICD10	A152	Respiratory tuberculosis, bacteriologically and histologically confirmed
Tuberculosis	ICD10	A153	Tuberculosis of lung, confirmed by unspecified means
Tuberculosis	ICD10	A154	Respiratory tuberculosis, bacteriologically and histologically confirmed
Tuberculosis	ICD10	A155	Tuberculosis of larynx, trachea and bronchus, confirmed bacteriologically and histologically
Tuberculosis	ICD10	A156	Respiratory tuberculosis, bacteriologically and histologically confirmed
Tuberculosis	ICD10	A157	Primary respiratory tuberculosis, confirmed bacteriologically and histologically
Tuberculosis	ICD10	A158	Other respiratory tuberculosis, confirmed bacteriologically and histologically
Tuberculosis	ICD10	A159	Respiratory tuberculosis, bacteriologically and histologically confirmed
Tuberculosis	ICD10	A16	Respiratory tuberculosis, not confirmed bacteriologically or histologically
Tuberculosis	ICD10	A17	Tuberculosis of nervous system
Tuberculosis	ICD10	A18	Tuberculosis of other organs
Tuberculosis	ICD10	A19	Miliary tuberculosis
Tuberculosis	ICD10	J65	Pneumoconiosis associated with tuberculosis
Tuberculosis	ICD10	M490	Tuberculosis of spine
Tuberculosis	ICD10	M900	Tuberculosis of bone
Tuberculosis	ICD10	O980	Tuberculosis complicating pregnancy, childbirth and the puerperium
Tuberculosis	ICD10	U843	Resistance to tuberculostatic drug(s)
Tuberculosis	SNOMED	1034541000000109	Infection resistant to tuberculostatic drug
Tuberculosis	SNOMED	10528009	Lupus vulgaris
Tuberculosis	SNOMED	10706006	Tuberculosis - meninges/CNS
Tuberculosis	SNOMED	10893003	Tuberculous adenitis
Tuberculosis	SNOMED	111830007	Tuberculosis of urinary tract
Tuberculosis	SNOMED	111832004	Other primary progressive tuberculosis
Tuberculosis	SNOMED	113858008	Mycobacterium tuberculosis complex
Tuberculosis	SNOMED	113861009	Mycobacterium tuberculosis
Tuberculosis	SNOMED	12137005	Tuberculosis of small intestine
Tuberculosis	SNOMED	12985005	Tuberculosis seminal vesicle
Tuberculosis	SNOMED	14188007	Tuberculosis of hip
Tuberculosis	SNOMED	15284007	Tuberculosis oesophagus
Tuberculosis	SNOMED	154283005	Pulmonary tuberculosis
Tuberculosis	SNOMED	16409009	Tuberculosis of retroperitoneal lymph nodes
Tuberculosis	SNOMED	170525009	Notification of tuberculosis

Tuberculosis	SNOMED	17136009	Tuberculosis of knee
Tuberculosis	SNOMED	1763591000006104	Sputum culture mycobacterium tuberculosis negative
Tuberculosis	SNOMED	17653001	Tuberculosis of bones and/or joints
Tuberculosis	SNOMED	182159002	Disseminated tuberculosis
Tuberculosis	SNOMED	182975000	Tuberculosis chemotherapy
Tuberculosis	SNOMED	1834101000006107	Sputum culture mycobacterium tuberculosis positive
Tuberculosis	SNOMED	186172004	Primary progressive tuberculosis with tuberculous pleurisy
Tuberculosis	SNOMED	186175002	Infiltrative lung tuberculosis
Tuberculosis	SNOMED	186177005	Tuberculosis of lung with cavitation
Tuberculosis	SNOMED	186178000	Tuberculosis of bronchus
Tuberculosis	SNOMED	186182003	Tuberculosis of pleura
Tuberculosis	SNOMED	186192006	[X]Resp tuberculos unspcfd,confirmd bacteriolog+histologicly
Tuberculosis	SNOMED	186193001	TB lung confirm sputum microscopy with or without culture
Tuberculosis	SNOMED	186194007	Tuberculosis of lung, confirmed by culture only
Tuberculosis	SNOMED	186195008	Tuberculosis of lung, confirmed histologically
Tuberculosis	SNOMED	186199002	Tuberculosis of larynx, trachea & bronchus conf bact/hist'y
Tuberculosis	SNOMED	186201000	Primary respiratory TB confirm bact and histologically
Tuberculosis	SNOMED	186202007	Prim respiratory TB without mention of bact or hist confirm
Tuberculosis	SNOMED	186203002	Tuberculosis of lung, bacteriologically & histolog'y neg
Tuberculosis	SNOMED	186225008	Tuberculosis of intestines, peritoneum and mesenteric glands
Tuberculosis	SNOMED	186231006	Tuberculosis limb bones - Tuberculous dactylitis
Tuberculosis	SNOMED	186259007	Scrofulous tuberculous abscess
Tuberculosis	SNOMED	186263000	Tuberculous chorioretinitis
Tuberculosis	SNOMED	186266008	Tuberculous chronic iridocyclitis
Tuberculosis	SNOMED	186267004	Tuberculous keratoconjunctivitis
Tuberculosis	SNOMED	186269001	Tuberculosis of ear
Tuberculosis	SNOMED	186270000	Tuberculosis of adrenal glands - Addison's disease
Tuberculosis	SNOMED	186272008	Tuberculosis of stomach
Tuberculosis	SNOMED	186273003	Tuberculosis of liver
Tuberculosis	SNOMED	186276006	Acute miliary tuberculosis
Tuberculosis	SNOMED	186278007	Acute miliary tuberculosis of multiple sites
Tuberculosis	SNOMED	190710003	Tuberculosis of other specified joint
Tuberculosis	SNOMED	1934651000006109	TB chemotherapy declined
Tuberculosis	SNOMED	1934681000006101	TB chemotherapy started
Tuberculosis	SNOMED	1934751000006104	TB chemotherapy discontinued - side effect
Tuberculosis	SNOMED	193787009	Keratitis caused by tuberculosis
Tuberculosis	SNOMED	194908003	TB - acute pericarditis
Tuberculosis	SNOMED	196017002	Pneumoconiosis associated with tuberculosis
Tuberculosis	SNOMED	199175001	Maternal tuberculosis in pregnancy/childbirth/puerperium
Tuberculosis	SNOMED	199178004	Maternal tuberculosis during pregnancy - baby delivered
Tuberculosis	SNOMED	199179007	Maternal tuberculosis in puerperium - baby delivered
Tuberculosis	SNOMED	199180005	Maternal tuberculosis in pregnancy - baby not yet delivered
Tuberculosis	SNOMED	199181009	Maternal tuberculosis in puerperium - baby previously deliv.
Tuberculosis	SNOMED	2028007	Bazin's disease - erythema induratum - TB hypersensitivity
Tuberculosis	SNOMED	203263006	Tuberculosis of cervical spine
Tuberculosis	SNOMED	203264000	Tuberculosis of thoracic spine
Tuberculosis	SNOMED	203265004	Tuberculosis of lumbar spine
Tuberculosis	SNOMED	203266003	Tuberculosis of sacrum/coccyx
Tuberculosis	SNOMED	203267007	Tuberculosis of limb bones
Tuberculosis	SNOMED	203269005	Tuberculosis of humerus
Tuberculosis	SNOMED	203270006	Tuberculosis of the forearm bone
Tuberculosis	SNOMED	203271005	Tuberculosis of the pelvic and thigh bones
Tuberculosis	SNOMED	203272003	Tuberculosis of the lower leg bone
Tuberculosis	SNOMED	203274002	Tuberculosis of multiple limb bones
Tuberculosis	SNOMED	203278004	Tuberculosis of the bones of the shoulder region
Tuberculosis	SNOMED	203279007	Tuberculosis of the bones of the hand
Tuberculosis	SNOMED	203280005	Tuberculosis of the bones of the ankle and foot
Tuberculosis	SNOMED	203282002	Tuberculosis of the bones of multiple sites
Tuberculosis	SNOMED	20372007	Prostatitis in tuberculosis
Tuberculosis	SNOMED	234090004	Tuberculosis of mesenteric lymph glands
Tuberculosis	SNOMED	235471000000107	Tuberculosis support
Tuberculosis	SNOMED	240376003	Tuberculosis of gastrointestinal tract NOS
Tuberculosis	SNOMED	240379005	Tuberculosis of other male genital organs NOS
Tuberculosis	SNOMED	25738004	Tuberculous synovitis
Tuberculosis	SNOMED	25831001	Tuberculosis of nasal septum
Tuberculosis	SNOMED	259837006	Mycobacterium tuberculosis antibody
Tuberculosis	SNOMED	26935004	Tuberculosis of thyroid gland
Tuberculosis	SNOMED	269912000	Sputum: tubercle on Z-N stain
Tuberculosis	SNOMED	271423008	Tuberculosis of skin and subcutaneous tissue
Tuberculosis	SNOMED	28399005	Tuberculosis spleen
Tuberculosis	SNOMED	32268008	Tuberculosis of bladder
Tuberculosis	SNOMED	33893009	Tuberculosis verrucosa cutis
Tuberculosis	SNOMED	35585004	Tuberculous endometritis
Tuberculosis	SNOMED	35786001	Tuberculoma of meninges
Tuberculosis	SNOMED	35984006	Pott's disease

Tuberculosis	SNOMED	36354002	Mycobacterium tuberculosis hominis
Tuberculosis	SNOMED	37133005	Tuberculous pyelitis
Tuberculosis	SNOMED	38115001	Tuberculosis of spinal meninges
Tuberculosis	SNOMED	38279006	Tuberculosis of other specified bones
Tuberculosis	SNOMED	38420005	Tuberculosis of large intestine
Tuberculosis	SNOMED	39306006	Tuberculosis of anus
Tuberculosis	SNOMED	40258005	Tuberculosis of testis
Tuberculosis	SNOMED	41156006	Tuberculosis papulonecrotica
Tuberculosis	SNOMED	413852006	Ciprofloxacin resistant tuberculosis
Tuberculosis	SNOMED	414146004	Ethambutol resistant tuberculosis
Tuberculosis	SNOMED	414149006	Ethionamide resistant tuberculosis
Tuberculosis	SNOMED	414546009	Isoniazid resistant tuberculosis
Tuberculosis	SNOMED	415222009	Pyrazinamide resistant tuberculosis
Tuberculosis	SNOMED	415345001	Rifampicin resistant tuberculosis
Tuberculosis	SNOMED	415622003	Streptomycin resistant tuberculosis
Tuberculosis	SNOMED	415759006	Tuberculosis index case
Tuberculosis	SNOMED	416265003	Tuberculoma of brain
Tuberculosis	SNOMED	416903004	Tuberculoma of spinal cord
Tuberculosis	SNOMED	423092005	Multidrug resistant tuberculosis
Tuberculosis	SNOMED	44323002	Tuberculosis of kidney
Tuberculosis	SNOMED	4445009	Tuberculosis of genitourinary system
Tuberculosis	SNOMED	44572005	Tuberculosis (TB) peritonitis
Tuberculosis	SNOMED	47292005	Tuberculosis myocardium
Tuberculosis	SNOMED	47604008	Miliary tuberculosis
Tuberculosis	SNOMED	49107007	Tuberculosis of eye
Tuberculosis	SNOMED	49558004	Tuberculous salpingitis
Tuberculosis	SNOMED	52721006	Tuberculosis of nasal sinus
Tuberculosis	SNOMED	52987001	Tuberculosis of endocardium
Tuberculosis	SNOMED	54084005	Scrofula
Tuberculosis	SNOMED	56498009	Tuberculosis of nasopharynx
Tuberculosis	SNOMED	56717001	Tuberculosis
Tuberculosis	SNOMED	58437007	TB - Tuberculous meningitis
Tuberculosis	SNOMED	58764007	Tuberculous osteomylelytis
Tuberculosis	SNOMED	597781000000105	Tuberculosis - other organs
Tuberculosis	SNOMED	62819009	Tuberculous cervicitis
Tuberculosis	SNOMED	63309002	Primary tuberculosis
Tuberculosis	SNOMED	66986005	Tuberculosis of skin
Tuberculosis	SNOMED	67256000	Tuberculosis pericardium
Tuberculosis	SNOMED	700273003	Isolated tracheal or bronchial tuberculosis
Tuberculosis	SNOMED	70341005	Tuberculosis (TB) with laryngitis
Tuberculosis	SNOMED	710106005	Extensively drug resistant tuberculosis
Tuberculosis	SNOMED	71375000	Tuberculous mastoiditis
Tuberculosis	SNOMED	74181004	Tuberculosis of other female genital organs
Tuberculosis	SNOMED	74387008	Tuberculosis of hilar lymph nodes
Tuberculosis	SNOMED	7444001	Tuberculosis of rectum
Tuberculosis	SNOMED	74610006	Tuberculous erythema nodosum
Tuberculosis	SNOMED	77038006	Tuberculosis of peripheral lymph nodes
Tuberculosis	SNOMED	77668003	Isolated tracheal tuberculosis
Tuberculosis	SNOMED	78436002	Tuberculosis of intrathoracic lymph nodes NOS
Tuberculosis	SNOMED	79158003	Tuberculous interstitial keratitis
Tuberculosis	SNOMED	80010008	Isolated bronchial tuberculosis
Tuberculosis	SNOMED	80602006	Nodular lung tuberculosis
Tuberculosis	SNOMED	81359005	Tuberculosis of ureter
Tuberculosis	SNOMED	82495004	Tuberculosis of mediastinal lymph nodes
Tuberculosis	SNOMED	82796002	Scrofuloderma
Tuberculosis	SNOMED	83652003	Tuberculosis of epididymis
Tuberculosis	SNOMED	83784000	Tuberculosis of mediastinum
Tuberculosis	SNOMED	84194006	Tuberculous oophoritis
Tuberculosis	SNOMED	84288007	Tuberculous episcleritis
Tuberculosis	SNOMED	85692003	Tuberculosis of tracheobronchial lymph nodes
Tuberculosis	SNOMED	90302003	Tuberculosis of cerebral meninges
Tuberculosis	SNOMED	907551000006106	[RFC] Tuberculosis
Tuberculosis	SNOMED	91483004	Encephalitis due to tuberculosis
Tuberculosis	SNOMED	925721000000000	Notification of tuberculosis
Tuberculosis	SNOMED	925721000006105	Notification of tuberculosis
Tuberculosis	SNOMED	970941000000000	Tuberculosis of urinary tract
Tuberculosis	SNOMED	970941000006105	Tuberculosis of urinary tract
Tuberculosis	SNOMED	9768003	Tuberculosis lichenoides
Valvular Diseases	ICD10	G110	Congenital nonprogressive ataxia
Valvular Diseases	ICD10	G111	Early-onset cerebellar ataxia
Valvular Diseases	ICD10	G112	Late-onset cerebellar ataxia
Valvular Diseases	ICD10	G113	Cerebellar ataxia with defective DNA repair
Valvular Diseases	ICD10	G114	Hereditary spastic paraplegia
Valvular Diseases	ICD10	G118	Other hereditary ataxias
Valvular Diseases	ICD10	G119	Hereditary ataxia, unspecified

Valvular Diseases	ICD10	I05	Rheumatic mitral valve diseases
Valvular Diseases	ICD10	I06	Rheumatic aortic valve diseases
Valvular Diseases	ICD10	I07	Rheumatic tricuspid valve diseases
Valvular Diseases	ICD10	I08	Multiple valve diseases
Valvular Diseases	ICD10	I091	Rheumatic diseases of endocardium, valve unspecified
Valvular Diseases	ICD10	I34	Nonrheumatic mitral valve disorders
Valvular Diseases	ICD10	I35	Nonrheumatic aortic valve disorders
Valvular Diseases	ICD10	I36	Nonrheumatic tricuspid valve disorders
Valvular Diseases	ICD10	I37	Pulmonary valve disorders
Valvular Diseases	ICD10	I38	Endocarditis, valve unspecified
Valvular Diseases	ICD10	I39	Endocarditis and heart valve disorders in diseases classified elsewhere
Valvular Diseases	ICD10	Q22	Congenital malformations of pulmonary and tricuspid valves
Valvular Diseases	ICD10	Q23	Congenital malformations of aortic and mitral valves
Valvular Diseases	ICD10	Q243	Pulmonary infundibular stenosis
Valvular Diseases	ICD10	Q244	Congenital subaortic stenosis
Valvular Diseases	ICD10	Q642	Congenital posterior urethral valves
Valvular Diseases	ICD10	T820	Mechanical complication of heart valve prosthesis
Valvular Diseases	ICD10	T822	Mechanical complication of coronary artery bypass and valve grafts
Valvular Diseases	ICD10	T826	Infection and inflammatory reaction due to cardiac valve prosthesis
Valvular Diseases	ICD10	Z952	Presence of prosthetic heart valve
Valvular Diseases	ICD10	Z953	Presence of xenogenic heart valve
Valvular Diseases	ICD10	Z954	Presence of other heart-valve replacement
Valvular Diseases	SNOMED	111287006	Tricuspid regurgitation, cause unspecified
Valvular Diseases	SNOMED	112814001	Closed heart valvotomy of aortic valve
Valvular Diseases	SNOMED	112816004	Aortic valvuloplasty
Valvular Diseases	SNOMED	1137091000006104	Adverse reaction to Catheter valve with integral bag
Valvular Diseases	SNOMED	1140231000006105	Adverse reaction to Catheter Valve
Valvular Diseases	SNOMED	11851006	Chronic rheumatic mitral valve
Valvular Diseases	SNOMED	12023003	Rheumatic aortic valve disease
Valvular Diseases	SNOMED	129243001	Closed tricuspid valvotomy
Valvular Diseases	SNOMED	13213009	Fusion of tricuspid valve cusps NEC
Valvular Diseases	SNOMED	13721004	Operation on chordae tendineae
Valvular Diseases	SNOMED	1411001	Operation on papillary muscle
Valvular Diseases	SNOMED	15096009	Congenital insufficiency of pulmonary valve
Valvular Diseases	SNOMED	160219005	Valveman
Valvular Diseases	SNOMED	161667004	[V]Heart valve transplanted
Valvular Diseases	SNOMED	161677002	H/O: artificial heart valve
Valvular Diseases	SNOMED	171522002	Renewal of valve of cerebroventricular shunt
Valvular Diseases	SNOMED	173133004	Changing of tracheo-oesophageal valve
Valvular Diseases	SNOMED	17401000	[SO]Specified valve of heart NEC
Valvular Diseases	SNOMED	174815002	Rep tetralog Fallot valved right ventricular outflow conduit
Valvular Diseases	SNOMED	174880000	Creation of valved cardiac conduit NOS
Valvular Diseases	SNOMED	174881001	Creation valved conduit between heart atrium and ventricle
Valvular Diseases	SNOMED	174882008	Creation of valved conduit between right atrium+pulm artery
Valvular Diseases	SNOMED	174883003	Creation of valved conduit between right ventr +pulm artery
Valvular Diseases	SNOMED	174884009	Creation of valved conduit between left ventricle and aorta
Valvular Diseases	SNOMED	174885005	Revision of valved cardiac conduit
Valvular Diseases	SNOMED	174916002	Valves of heart and adjacent structures operations
Valvular Diseases	SNOMED	174918001	Allograft replacement of mitral valve
Valvular Diseases	SNOMED	174919009	Xenograft replacement of mitral valve
Valvular Diseases	SNOMED	174927000	Allograft replacement of aortic valve
Valvular Diseases	SNOMED	174928005	Pericardial aortic valve replacement
Valvular Diseases	SNOMED	174929002	Prosthetic replacement of aortic valve
Valvular Diseases	SNOMED	174935002	Allograft replacement of tricuspid valve
Valvular Diseases	SNOMED	174936001	Xenograft replacement of tricuspid valve
Valvular Diseases	SNOMED	174937005	Prosthetic replacement of tricuspid valve
Valvular Diseases	SNOMED	174939008	Repositioning of tricuspid valve
Valvular Diseases	SNOMED	174944001	Allograft replacement of pulmonary valve
Valvular Diseases	SNOMED	174945000	Xenograft replacement of pulmonary valve
Valvular Diseases	SNOMED	174946004	Replacement of pulmonary valve with prosthesis
Valvular Diseases	SNOMED	174959007	Other specified revision of plastic repair of valve of heart
Valvular Diseases	SNOMED	174960002	Revision of plastic repair of mitral valve
Valvular Diseases	SNOMED	174961003	Revision of plastic repair of aortic valve
Valvular Diseases	SNOMED	174962005	Revision of plastic repair of tricuspid valve
Valvular Diseases	SNOMED	174963000	Revision of plastic repair of pulmonary valve
Valvular Diseases	SNOMED	174966008	Open incision of valve of heart NOS
Valvular Diseases	SNOMED	174967004	Open mitral valvotomy
Valvular Diseases	SNOMED	174968009	Open aortic valvotomy
Valvular Diseases	SNOMED	174969001	Open tricuspid valvotomy
Valvular Diseases	SNOMED	174970000	Open pulmonary valvotomy
Valvular Diseases	SNOMED	174973003	Other specified closed incision of valve of heart
Valvular Diseases	SNOMED	174980001	MVA - Mitral valve annuloplasty
Valvular Diseases	SNOMED	174983004	Excision of vegetations of valve of heart
Valvular Diseases	SNOMED	174987003	Percutaneous transluminal mitral valvotomy
Valvular Diseases	SNOMED	174988008	Percutaneous transluminal aortic valvotomy

Valvular Diseases	SNOMED	174989000	Percutaneous transluminal tricuspid valvotomy
Valvular Diseases	SNOMED	174990009	Percutaneous transluminal pulmonary valvotomy
Valvular Diseases	SNOMED	174994000	Remove obstruction from structure adjacent to valve of heart
Valvular Diseases	SNOMED	174995004	Infundibulectomy of heart using patch
Valvular Diseases	SNOMED	174997007	Repair of subaortic stenosis
Valvular Diseases	SNOMED	174998002	Repair of supraaortic stenosis
Valvular Diseases	SNOMED	175103009	Inspection of valve of heart
Valvular Diseases	SNOMED	175807007	Other specified repair of valve of vein
Valvular Diseases	SNOMED	175808002	Transposition of valve of vein
Valvular Diseases	SNOMED	175809005	Interposition of valve of vein
Valvular Diseases	SNOMED	176327008	Congenit. urethral valve excis
Valvular Diseases	SNOMED	176358009	Endoscopic destruction of posterior urethral valve
Valvular Diseases	SNOMED	176374004	Hook ablation of posterior urethral valve
Valvular Diseases	SNOMED	17759006	Rheumatic aortic stenosis with regurgitation
Valvular Diseases	SNOMED	18546004	Congenital aortic stenosis
Valvular Diseases	SNOMED	18687009	RH. pulmonary valve disease
Valvular Diseases	SNOMED	186875004	Syphilitic endocarditis of mitral valve
Valvular Diseases	SNOMED	186877007	Syphilitic endocarditis of tricuspid valve
Valvular Diseases	SNOMED	186878002	Syphilitic endocarditis of pulmonary valve
Valvular Diseases	SNOMED	1916830000000000	Insertion of endobronchial valve
Valvular Diseases	SNOMED	1916831000006101	Insertion of endobronchial valve
Valvular Diseases	SNOMED	194726006	RH. mitral stenosis + insuffic
Valvular Diseases	SNOMED	194727002	Nonrheumatic mitral valve stenosis
Valvular Diseases	SNOMED	194732001	Diseases of mitral and aortic valves
Valvular Diseases	SNOMED	194733006	Mitral and aortic stenosis
Valvular Diseases	SNOMED	194734000	Mitral stenosis and aortic insufficiency
Valvular Diseases	SNOMED	194735004	Mitral regurgitation and aortic stenosis
Valvular Diseases	SNOMED	194736003	Mitral and aortic incompetence
Valvular Diseases	SNOMED	194737007	Multiple mitral and aortic valve involvement
Valvular Diseases	SNOMED	194741006	Rheumatic tricuspid stenosis and incompetence
Valvular Diseases	SNOMED	194978002	Mitral incompetence, non-rheumatic
Valvular Diseases	SNOMED	194983005	Aortic insufficiency, non-rheumatic
Valvular Diseases	SNOMED	194984004	Aortic stenosis, non-rheumatic
Valvular Diseases	SNOMED	194987006	Aortic valve stenosis with insufficiency
Valvular Diseases	SNOMED	194989009	Tricuspid valve disorders, non-rheumatic
Valvular Diseases	SNOMED	194990000	Tricuspid insufficiency, non-rheumatic
Valvular Diseases	SNOMED	194991001	Tricuspid stenosis, non-rheumatic
Valvular Diseases	SNOMED	194992008	Nonrheumatic tricuspid valve stenosis with insufficiency
Valvular Diseases	SNOMED	194995005	Pulmonary incompetence, non-rheumatic
Valvular Diseases	SNOMED	194997002	Pulmonary stenosis, non-rheumatic
Valvular Diseases	SNOMED	195000004	Pulmonary valve stenosis with insufficiency
Valvular Diseases	SNOMED	195002007	[X]Multiple valve disorders/diseases CE
Valvular Diseases	SNOMED	195003002	Disorders of both aortic and tricuspid valves
Valvular Diseases	SNOMED	195004008	Disorders of both mitral and tricuspid valves
Valvular Diseases	SNOMED	195005009	Combined disorders of mitral, aortic and tricuspid valves
Valvular Diseases	SNOMED	204339005	Pulmonary valve anomaly, unspecified
Valvular Diseases	SNOMED	204342004	Congenital atresia of the pulmonary valve
Valvular Diseases	SNOMED	204343009	Hypoplasia of pulmonary valve
Valvular Diseases	SNOMED	204346001	Congenital fusion of pulmonary valve segment
Valvular Diseases	SNOMED	204352000	Supernumerary pulmonary valve cusps
Valvular Diseases	SNOMED	204357006	Ebstein's anomaly
Valvular Diseases	SNOMED	204362007	Parachute malformation of mitral valve
Valvular Diseases	SNOMED	204363002	Supernumerary cusps of the mitral valve
Valvular Diseases	SNOMED	204368006	Subaortic stenosis
Valvular Diseases	SNOMED	204370002	Subvalvular pulmonic stenosis
Valvular Diseases	SNOMED	204402008	Fusion of mitral valve cusps
Valvular Diseases	SNOMED	20721001	Tricuspid valve disease NEC
Valvular Diseases	SNOMED	213036006	Valve prosthesis mech.complic.
Valvular Diseases	SNOMED	213038007	Infect and inflammatory reaction due to cardiac valve pros
Valvular Diseases	SNOMED	232765005	Excision of supramitral ring
Valvular Diseases	SNOMED	232773001	Bjork-Shiley prosthetic replacement of mitral valve
Valvular Diseases	SNOMED	232783002	De Vega tricuspid annuloplasty
Valvular Diseases	SNOMED	232808000	Closure of tricuspid valve
Valvular Diseases	SNOMED	232811004	Resection of tricuspid valve
Valvular Diseases	SNOMED	232816009	AV - Aortic valve operation
Valvular Diseases	SNOMED	232850000	Aortoventriculoplasty
Valvular Diseases	SNOMED	232867007	Oversewing of pulmonary valve
Valvular Diseases	SNOMED	232872003	Pulmonary valvectomy
Valvular Diseases	SNOMED	232931004	Truncal valve repair
Valvular Diseases	SNOMED	232945005	Open truncal valvotomy
Valvular Diseases	SNOMED	232948007	Replacement of truncal valve
Valvular Diseases	SNOMED	233065007	Infundibulectomy of heart NEC
Valvular Diseases	SNOMED	233601000000100	Ao ro repl us pulm val auto ri vent pul art val cond aortov
Valvular Diseases	SNOMED	233848004	Disease of endocardium and heart valve
Valvular Diseases	SNOMED	233849007	Syphilitic valve disease

Valvular Diseases	SNOMED	250978003	Aortic valve calcification
Valvular Diseases	SNOMED	250989005	Mitral valve leaf prolapse
Valvular Diseases	SNOMED	251003004	Rupture of mitral valve leaflet
Valvular Diseases	SNOMED	251088005	Aortic valve ventriculoarterial gradient
Valvular Diseases	SNOMED	25236004	TVR - Tricuspid valve replacement
Valvular Diseases	SNOMED	253601007	Hypoplastic aortic orifice or valve
Valvular Diseases	SNOMED	253672004	Preductal aortic stenosis
Valvular Diseases	SNOMED	253676001	Postductal aortic stenosis
Valvular Diseases	SNOMED	253887007	Congenital ureteric valves
Valvular Diseases	SNOMED	253900005	Congenital posterior urethral valve
Valvular Diseases	SNOMED	253901009	Congenital urethral posterior valvular stricture
Valvular Diseases	SNOMED	255172003	Benign neoplasm of ileocaecal valve
Valvular Diseases	SNOMED	2583009	Remnants of valves of sinus venosus
Valvular Diseases	SNOMED	26212005	Replacement of aortic valve
Valvular Diseases	SNOMED	265472007	Plastic repair of mitral valve
Valvular Diseases	SNOMED	265473002	Other specified plastic repair of aortic valve
Valvular Diseases	SNOMED	265474008	Other specified plastic repair of tricuspid valve
Valvular Diseases	SNOMED	265475009	Pulmonary valvuloplasty NEC
Valvular Diseases	SNOMED	265480000	Annuloplasty of tricuspid valve
Valvular Diseases	SNOMED	268185002	Supra-valvular aortic stenosis
Valvular Diseases	SNOMED	268237006	Congenital urethral valvular stricture
Valvular Diseases	SNOMED	274097009	Heart valve disorders - non-rheumatic
Valvular Diseases	SNOMED	275162000	Hofmeister valved gastrectomy
Valvular Diseases	SNOMED	275212003	Valvuloplasty of heart NEC
Valvular Diseases	SNOMED	27613000	Ventriculopleural anastomosis with valve
Valvular Diseases	SNOMED	278480000	Syphilitic endocarditis of aortic valve
Valvular Diseases	SNOMED	279317000	[SO]Truncal valve
Valvular Diseases	SNOMED	285701000119108	[X]Presence of other heart valve replacement
Valvular Diseases	SNOMED	28656008	Congenital aortic valve insufficiency, unspecified
Valvular Diseases	SNOMED	29928006	Congenital mitral insufficiency
Valvular Diseases	SNOMED	303647007	Drainage of ventricle of brain using Holter valve
Valvular Diseases	SNOMED	307279007	Starr prosthetic replacement of valve of heart
Valvular Diseases	SNOMED	307799000	Incision of heart valve
Valvular Diseases	SNOMED	31085000	Rheumatic mitral insufficiency
Valvular Diseases	SNOMED	34068001	Replacement of valve of heart NEC
Valvular Diseases	SNOMED	34126003	Rheumatic pulmonary valve incompetence
Valvular Diseases	SNOMED	34202007	[SO]Aortic valve
Valvular Diseases	SNOMED	35688006	[X] Embolism from prosthetic heart valve
Valvular Diseases	SNOMED	36233006	Congenital tricuspid stenosis
Valvular Diseases	SNOMED	368009	Non-rheumatic valvular disease
Valvular Diseases	SNOMED	384441000000101	Percutaneous transluminal pulmonary valve perfor dilation
Valvular Diseases	SNOMED	384641003	Mitral valve repair NEC
Valvular Diseases	SNOMED	384642005	Mitral valvuloplasty
Valvular Diseases	SNOMED	384643000	Tricuspid valvuloplasty NEC
Valvular Diseases	SNOMED	386727004	Therapeutic transluminal operations on valve of heart
Valvular Diseases	SNOMED	386749005	Pulmonary valve repair NEC
Valvular Diseases	SNOMED	386815009	Repair of aortic sinus of Valsalva aneurysm
Valvular Diseases	SNOMED	39057004	[SO]Pulmonary valve
Valvular Diseases	SNOMED	40445007	Insufficiency of unspecified heart valve
Valvular Diseases	SNOMED	40964007	Chronic cardiac valvulitis NOS
Valvular Diseases	SNOMED	409712001	Mitral valve prolapse
Valvular Diseases	SNOMED	425447009	Bag valve mask ventilation
Valvular Diseases	SNOMED	425785006	Repair of tetralogy of Fallot with absent pulmonary valve
Valvular Diseases	SNOMED	426506002	Creation valved conduit between left vent heart pulm artery
Valvular Diseases	SNOMED	426611007	Congestive heart failure due to valvular disease
Valvular Diseases	SNOMED	427265007	Closure of aorto-left ventricular tunnel
Valvular Diseases	SNOMED	427536000	Endoscopic destruction of urethral valves
Valvular Diseases	SNOMED	428501002	Aortic [REDACTED] replac us pul val auto ri vent pulm art val cond
Valvular Diseases	SNOMED	428893001	Replacement of valved cardiac conduit
Valvular Diseases	SNOMED	429248007	Percutaneous transluminal pulmonary valve replacement
Valvular Diseases	SNOMED	429269009	Aortoventriculoplasty with pulmonary valve autograft
Valvular Diseases	SNOMED	429359000	Revision of plastic repair of truncal valve
Valvular Diseases	SNOMED	429616001	Radical aortopulmonary recon rig vent pulmon art valvel cond
Valvular Diseases	SNOMED	431339008	Edwards prosthetic replacement of mitral valve
Valvular Diseases	SNOMED	432071000000105	[V]Has cerebrospinal fluid valve in situ
Valvular Diseases	SNOMED	4374004	[X]Other congenital malformations of tricuspid valve
Valvular Diseases	SNOMED	44241007	Stenosis of unspecified heart valve
Valvular Diseases	SNOMED	443887000	Transapical aortic valve implantation
Valvular Diseases	SNOMED	443916000	Transluminal aortic valve implantation
Valvular Diseases	SNOMED	46030003	Right atrioventricular valve
Valvular Diseases	SNOMED	46165000	Other chronic rheumatic endocardial disease
Valvular Diseases	SNOMED	48724000	Mitral valve regurgitation
Valvular Diseases	SNOMED	488551000006106	Aortic valve opening restricted
Valvular Diseases	SNOMED	48872007	Rheumatic endocarditis NOS
Valvular Diseases	SNOMED	49699002	Rheumatic tricuspid valve disease

Valvular Diseases	SNOMED	49915006	Tricuspid stenosis and incompetence, cause unspecified
Valvular Diseases	SNOMED	53059001	Replacement of mitral valve
Valvular Diseases	SNOMED	553061000000101	Allograft replacement of valve of heart NEC
Valvular Diseases	SNOMED	56786000	Pulmonary stenosis, cause unspecified
Valvular Diseases	SNOMED	56819008	[X]Endocarditis,valve unspecified,in diseases CE
Valvular Diseases	SNOMED	583021000000103	Endocarditis, valve unspecified
Valvular Diseases	SNOMED	585591000000109	Repair of valve of heart NEC
Valvular Diseases	SNOMED	60234000	Aortic incompetence alone, cause unspecified
Valvular Diseases	SNOMED	60573004	Aortic stenosis alone, cause unspecified
Valvular Diseases	SNOMED	612101000000109	RH. tricuspid valve disease
Valvular Diseases	SNOMED	63042009	Congenital atresia of tricuspid valve
Valvular Diseases	SNOMED	635721000006108	Echo - mitral valve excursion
Valvular Diseases	SNOMED	639451000000102	Rheumatic mitral disease NOS
Valvular Diseases	SNOMED	639461000000104	Rheumatic aortic valve disease NOS
Valvular Diseases	SNOMED	639491000000000	Tricuspid valve disease NEC
Valvular Diseases	SNOMED	639491000000105	Tricuspid valve disease NEC
Valvular Diseases	SNOMED	639601000000100	Endocarditis, valve unspecified, NOS
Valvular Diseases	SNOMED	6433003	Closed mitral valvotomy
Valvular Diseases	SNOMED	64915003	Operation on structure adjacent to valve of heart NOS
Valvular Diseases	SNOMED	653341000000107	Rheumatic endocardium disease
Valvular Diseases	SNOMED	654271000000108	Other chronic rheumatic heart
Valvular Diseases	SNOMED	67278007	Congenital pulmonary stenosis NOS
Valvular Diseases	SNOMED	67391006	Syphilitic endocarditis of unspecified valve
Valvular Diseases	SNOMED	67696008	Tricuspid incompetence - rheumatic
Valvular Diseases	SNOMED	67754003	Aortic valve sclerosis
Valvular Diseases	SNOMED	682551000000000	Non-rheumatic valvular disease
Valvular Diseases	SNOMED	682551000000104	Non-rheumatic valvular disease
Valvular Diseases	SNOMED	68860005	Percutaneous transluminal valvuloplasty
Valvular Diseases	SNOMED	700331000000109	Non-rheumatic aortic sclerosis
Valvular Diseases	SNOMED	70320004	Congenital heart valve abnormality
Valvular Diseases	SNOMED	715563006	Insertion of endobronchial valve
Valvular Diseases	SNOMED	72011007	Rheumatic aortic stenosis
Valvular Diseases	SNOMED	72352009	Bicuspid aortic valve - congen
Valvular Diseases	SNOMED	73544002	Heart valve operation NOS
Valvular Diseases	SNOMED	754521000033107	Catheter valve with integral bag
Valvular Diseases	SNOMED	76267008	[X]Other pulmonary valve disorders
Valvular Diseases	SNOMED	78031003	Aortic incompetence - rheumatic
Valvular Diseases	SNOMED	792691000000103	Operations on mitral subvalvar apparatus
Valvular Diseases	SNOMED	79619009	Mitral stenosis
Valvular Diseases	SNOMED	82458004	Congenital mitral stenosis
Valvular Diseases	SNOMED	82762009	Insertion of Halber valve for spina bifida
Valvular Diseases	SNOMED	83187002	Closed pulmonary valvotomy
Valvular Diseases	SNOMED	83898004	Rheumatic mitral valve disease
Valvular Diseases	SNOMED	85971001	Rheumatic pulmonary valve stenosis with regurgitation
Valvular Diseases	SNOMED	86466006	Rheumatic mitral stenosis
Valvular Diseases	SNOMED	87123001	Excision of valve of heart NOS
Valvular Diseases	SNOMED	8722008	Aortic valve disorders NOS
Valvular Diseases	SNOMED	87648004	Rheumatic valvulitis, chronic NOS
Valvular Diseases	SNOMED	88045004	Replacement of pulmonary valve
Valvular Diseases	SNOMED	88318005	Rheumatic tricuspid valve obstruction
Valvular Diseases	SNOMED	88797001	Implant of heart valve prosthesis + complication, no blame
Valvular Diseases	SNOMED	89736004	Endocarditis, valve unspecified, OS
Valvular Diseases	SNOMED	91134007	[SO]Mitral valve
Valvular Diseases	SNOMED	91434003	Pulmonary regurgitation, cause unspecified
Valvular Diseases	SNOMED	91442002	Rheumatic pulmonary valve obstruction
Valvular Diseases	SNOMED	91634006	Fused commissures of mitral valve
Venous Thromboembolism	ICD10	I801	Phlebitis and thrombophlebitis of femoral vein
Venous Thromboembolism	ICD10	I802	Deep vein thrombosis NOS
Venous Thromboembolism	ICD10	I81	Portal vein thrombosis
Venous Thromboembolism	ICD10	I82	Other venous embolism and thrombosis (vena cava, renal vein etc)
Venous Thromboembolism	SNOMED	111293003	Thrombosis of vein NOS
Venous Thromboembolism	SNOMED	128053003	DVT - Deep vein thrombosis
Venous Thromboembolism	SNOMED	134399007	Deep vein thrombosis of leg related to air travel
Venous Thromboembolism	SNOMED	14246007	Embolism of central nervous system venous sinus
Venous Thromboembolism	SNOMED	161508001	[V] Personal history deep vein thrombosis
Venous Thromboembolism	SNOMED	1729330000000000	Cerebral venous thrombosis
Venous Thromboembolism	SNOMED	1729331000006100	Cerebral venous thrombosis
Venous Thromboembolism	SNOMED	1786920000000000	Recurrent deep vein thrombosis
Venous Thromboembolism	SNOMED	1786921000006100	Recurrent deep vein thrombosis
Venous Thromboembolism	SNOMED	17920008	PVT - Portal vein thrombosis
Venous Thromboembolism	SNOMED	192754003	Embolism cavernous sinus
Venous Thromboembolism	SNOMED	192755002	Embolism superior longitudinal sinus
Venous Thromboembolism	SNOMED	192756001	Embolism lateral sinus
Venous Thromboembolism	SNOMED	192757005	Embolism transverse sinus
Venous Thromboembolism	SNOMED	192759008	Thrombosis of central nervous system venous sinus NOS

Venous Thromboembolism	SNOMED	192760003	Thrombosis of superior longitudinal sinus
Venous Thromboembolism	SNOMED	192761004	Thrombosis transverse sinus
Venous Thromboembolism	SNOMED	195229008	Nonpyogenic venous sinus thrombosis
Venous Thromboembolism	SNOMED	195230003	Cereb infarct due cerebral venous thrombosis, nonpyogenic
Venous Thromboembolism	SNOMED	195437003	Embolism and thrombosis of the vena cava
Venous Thromboembolism	SNOMED	195438008	Embolism and thrombosis of the renal vein
Venous Thromboembolism	SNOMED	197001004	Deep venous thrombosis of the superior mesenteric vein
Venous Thromboembolism	SNOMED	200232006	Antenatal deep vein thrombosis - delivered
Venous Thromboembolism	SNOMED	200233001	Antenatal deep vein thrombosis with antenatal complication
Venous Thromboembolism	SNOMED	200237000	Postnatal deep vein thrombosis - delivered with p/n comp
Venous Thromboembolism	SNOMED	200238005	Postnatal deep vein thrombosis with postnatal complication
Venous Thromboembolism	SNOMED	200258006	Obstetric cerebral venous thrombosis
Venous Thromboembolism	SNOMED	200259003	Cerebral venous thrombosis in pregnancy
Venous Thromboembolism	SNOMED	200260008	Cerebral venous thrombosis in the puerperium
Venous Thromboembolism	SNOMED	21258007	Thrombosis lateral sinus
Venous Thromboembolism	SNOMED	213220000	Post operative deep vein thrombosis
Venous Thromboembolism	SNOMED	213310008	Superfic.venous thrombosis leg
Venous Thromboembolism	SNOMED	234049002	Embolism of vein NOS
Venous Thromboembolism	SNOMED	266267005	Deep vein thrombosis, leg
Venous Thromboembolism	SNOMED	281595001	Thrombosis of inferior vena cava
Venous Thromboembolism	SNOMED	297156001	Axillary vein thrombosis
Venous Thromboembolism	SNOMED	309735004	Thrombosis of vein of leg
Venous Thromboembolism	SNOMED	404223003	Deep venous thrombosis of leg
Venous Thromboembolism	SNOMED	413956008	Deep vein thrombosis of leg related to intravenous drug use
Venous Thromboembolism	SNOMED	429098002	Other venous embolism and thrombosis
Venous Thromboembolism	SNOMED	438647008	Thrombosis of subclavian vein
Venous Thromboembolism	SNOMED	443210003	Deep venous thrombosis of peroneal vein
Venous Thromboembolism	SNOMED	49956009	Antepartum DVT
Venous Thromboembolism	SNOMED	56272000	DVT - deep venous thrombosis, postnatal
Venous Thromboembolism	SNOMED	587851000000108	Embolus of vein NOS
Venous Thromboembolism	SNOMED	690131000000000	Thrombosis of vein NOS
Venous Thromboembolism	SNOMED	690131000000103	Thrombosis of vein NOS
Venous Thromboembolism	SNOMED	710167004	Recurrent deep vein thrombosis
Venous Thromboembolism	SNOMED	82385007	Hepatic vein thrombosis
Venous Thromboembolism	SNOMED	851241000000000	Axillary vein thrombosis
Venous Thromboembolism	SNOMED	851241000006104	Axillary vein thrombosis
Venous Thromboembolism	SNOMED	864191000000104	Thrombosis of internal jugular vein
Venous Thromboembolism	SNOMED	864211000000100	Thrombosis of external jugular vein
Venous Thromboembolism	SNOMED	864311000000105	Thrombosis of vein of transplanted kidney
Venous Thromboembolism	SNOMED	89980009	Thrombosis cavernous sinus
Venous Thromboembolism	SNOMED	905491000006108	[RFC] Venous thrombosis
Venous Thromboembolism	SNOMED	909471000006105	[RFC] Deep vein thrombosis
Venous Thromboembolism	SNOMED	95455008	Cerebral venous thrombosis
Venous Thromboembolism	SNOMED	978421000000101	Unprovoked DVT (deep vein thrombosis)
Venous Thromboembolism	SNOMED	978441000000108	Provoked deep vein thrombosis
Visual Impairment	ICD10	H540	Blindness, binocular
Visual Impairment	ICD10	H541	Severe visual impairment, binocular
Visual Impairment	SNOMED	105597003	Blindness
Visual Impairment	SNOMED	13734003	Better eye: profound VI, Lesser eye: total VI
Visual Impairment	SNOMED	1583901000006104	Ineligibility for diab. eye screen: Blind (screening contraind)
Visual Impairment	SNOMED	170727003	Registered blind
Visual Impairment	SNOMED	183361009	Blind rehabilitation
Visual Impairment	SNOMED	1909000	Better eye: severe VI, Lesser eye: near total VI
Visual Impairment	SNOMED	193413001	Congenital retinal blindness
Visual Impairment	SNOMED	193699007	Blind - both eyes
Visual Impairment	SNOMED	193703005	Better eye: near total VI, Lesser eye: total VI
Visual Impairment	SNOMED	193704004	Better eye: near total VI, Lesser eye: near total VI
Visual Impairment	SNOMED	193707006	Better eye: profound VI, Lesser eye: near total VI
Visual Impairment	SNOMED	193708001	Better eye: profound VI, Lesser eye: profound VI
Visual Impairment	SNOMED	193709009	Acquired blindness, both eyes
Visual Impairment	SNOMED	193711000	Better eye: moderate VI, Lesser eye: blind, unspecified
Visual Impairment	SNOMED	193714008	Better eye: severe VI, Lesser eye: total VI
Visual Impairment	SNOMED	193716005	Better eye: severe VI, Lesser eye: profound VI
Visual Impairment	SNOMED	193718006	Better eye: moderate VI, Lesser eye: total VI
Visual Impairment	SNOMED	193719003	Better eye: moderate VI, Lesser eye: near total VI
Visual Impairment	SNOMED	193720009	Better eye: moderate VI, Lesser eye: profound VI
Visual Impairment	SNOMED	193725004	Better eye: severe VI, Lesser eye: severe VI
Visual Impairment	SNOMED	193727007	Better eye: moderate VI, Lesser eye: severe VI
Visual Impairment	SNOMED	193731001	Legal blindness USA
Visual Impairment	SNOMED	212141007	Blindness - traumatic - NOS
Visual Impairment	SNOMED	2531000000100	Registered partially blind
Visual Impairment	SNOMED	267727004	Blind/low vision - both eyes
Visual Impairment	SNOMED	29960003	Blind lead dog rehabilitation
Visual Impairment	SNOMED	300994001	Registered partially sighted
Visual Impairment	SNOMED	374691000000100	Excluded from diabetic retinopathy screening as blind

Visual Impairment	SNOMED	397540003	Blindness and low vision
Visual Impairment	SNOMED	433147009	Deafblind
Visual Impairment	SNOMED	566991000000106	Blindness/low vision NOS
Visual Impairment	SNOMED	713031000000100	Registered sight impaired
Visual Impairment	SNOMED	727391000000102	Certificate of vision impairment
Visual Impairment	SNOMED	796201000000109	Requires deafblind communicator guide
Visual Impairment	SNOMED	7973008	Sight impaired
Visual Impairment	SNOMED	813871000000108	Severe binocular visual impairment
Visual Impairment	SNOMED	857631000006102	Vision - blind despite any aid
Visual Impairment	SNOMED	862731000000105	Uses guide dog for the blind
Visual Impairment	SNOMED	907831000006101	[RFC] Advice/care for the blind
Visual Impairment	SNOMED	957031000000107	Uses deafblind intervener
Visual Impairment	SNOMED	957051000000100	Requires deafblind manual alphabet interpreter
Visual Impairment	SNOMED	957071000000109	Requires deafblind block alphabet interpreter
Visual Impairment	SNOMED	957111000000103	Requires deafblind haptic communication interpreter
Visual Impairment	SNOMED	981781000000107	Uses Deafblind Manual Alphabet
Visual Impairment	SNOMED	982291000006102	Sight impairment

Supplementary Figure S1: Illustration of combinations of multiple long-term conditions and associated number of outcomes (hospitalisation or death).

MLTC combinations	Number
A+B+C	1000
A+B+C+D	250
A+B+C+E	190
A+B+C+D+E	60

*Note: This table is for illustrative purposes only; other combinations involving A+B+C, such as A+B+C+F or A+B+C+G, are also possible.

As additional conditions are combined with A+B+C (e.g., D, E and so on), the number of observed outcome events for each combination decreases. This reduction in frequency may lead to the erroneous conclusion that A+B+C is not a significant MLTC combination, even though it may play an important role in winter pressure.

Supplementary Figure S2: Flowchart of individuals within the cohort analysed to identify the combinations of long-term conditions associated with the highest risk of hospitalization and deaths during winter, 2021-2022.

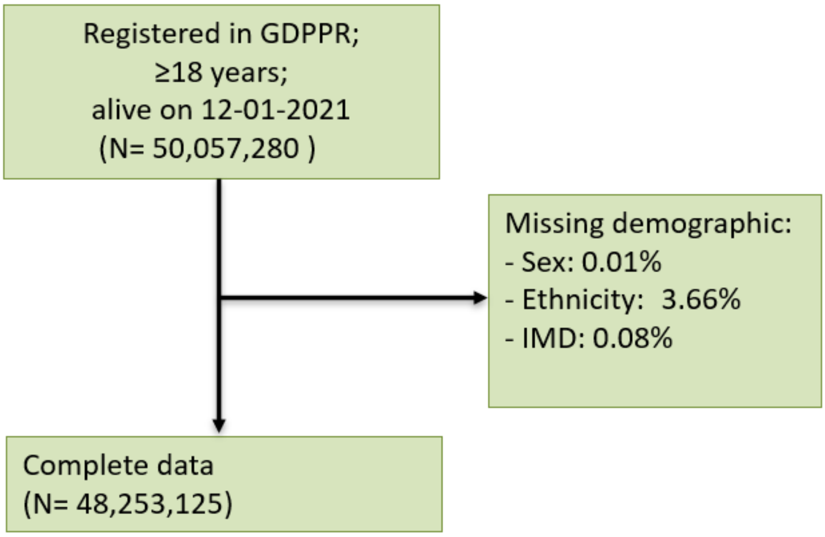

Note: Numbers are rounded to the nearest five as per NHS Data Access Environment (DAE) safe output guidelines.

GDPPR: General Practice Extraction Service (GPES) Data for Pandemic Planning and Research; IMD = index of multiple deprivation.
