# Supplementary material for: Combinations of multiple long term conditions and risk of hospital admission or death during winter 2021-22 in England: population based cohort study
Source: BMJ Med. 2024 Nov 12;3(1):e001016. doi: 10.1136/bmjmed-2024-001016 (PMC11580288; doi:10.1136/bmjmed-2024-001016)
Supplement: Supplementary file 2 [file bmjmed-2024-001016.reviewer_comments.pdf]

PEER REVIEW HISTORY

BMJ Medicine publishes all reviews undertaken for accepted manuscripts. Reviewers are asked to complete a checklist review form and are provided with free text boxes to elaborate on their assessment. These free text comments are reproduced below.

ARTICLE DETAILS

**TITLE (PROVISIONAL)**	Combinations of multiple long-term conditions and risk of hospitalisation or death during winter 2021-22: population-based cohort study of 48 million people in England
**AUTHORS**	Islam, Nazrul; Shabnam, Sharmin; Khan, Nusrat; Gillies, Clare; Zaccardi, Francesco; Banerjee, Amitava; Nafilyan, Vahé; Khunti, Kamlesh; Dambha-Miller, Hajira

VERSION 1 - REVIEW

**REVIEWER NAME**	Harron, Katie
**REVIEWER AFFILIATION**	
**REVIEWER CONFLICT OF INTEREST**	None
**DATE REVIEW RETURNED**	24-Jul-2024

**GENERAL COMMENTS**	This study aims to determine which combination of multiple long-term conditions are associated with the greatest risk of winter hospitalisations and deaths.<br><br>The population coverage of this study, including both primary and secondary care data, is a real strength. The findings should be highly generalisable to the English population. Overall the study is well described and I only have a few points of clarification.<br><br>Abstract results: Please state absolute rates of hospitalisations to give context to the relative effect sizes. The adjusted rate of death is given as 14.6 for CVD+dementia. I think this is the risk ratio rather than the rate so this should be reworded.<br><br>It would be helpful to give more clarity on the coverage of the datasets, i.e. who is and is not included in HES (those admitted to hospitals outside of England, or private hospitals etc would be missed).<br><br>How did you assess model fit? Were all the covariates significant?<br><br>The discussion is rather long and could be made more concise. However, the limitations of how the MLTC combinations should be more thoroughly discussed. These were subjective decisions, and different decisions about how to combine and categorise conditions could have led to different results. There might be different combinations of MLTCs which affect smaller numbers of patients, but which contribute disproportionately to the overall number of hospitalisations and deaths. It would be helpful to explore whether other data-driven approaches could be used to derive the combinations of interest.

**REVIEWER NAME**	GULLIFORD, Martin
**REVIEWER AFFILIATION**	Kings College London, School of Population Health and Environmental Sciences
**REVIEWER CONFLICT OF INTEREST**	None
**DATE REVIEW RETURNED**	25-Jul-2024

**GENERAL COMMENTS**	This is a large UK-wide study that evaluates combinations of long term conditions and risks of hospital admission or all cause mortality. The study finds that pre-selected combinations of conditions are associated with very high absolute and relative risks of these outcomes. The study concludes that these estimates identify groups that could be targeted for policy interventions.<br><br>The study addresses a topical subject and while the results have most relevance in the UK, the findings could be of interest to an international readership. As the paper points out, many previous studies have addressed related issues but this study considers specific condition combinations, rather than levels of multiplicity. However, it might be questioned whether the results are surprising, given that conditions were selected for study based on their known associations with hospital admission or death.<br><br>1. The title and text refer to hospitalisation AND death (i.e persons admitted to hospital who died), however, the study seems to include hospitalisation OR death (i.e. including people admitted who did not die, as well as people who died but were not admitted). As a minimum, all the ANDs should be changed to ORs. But there could be a case for treating the two outcomes separately.<br><br>2. The abstract should mention the study period is from Dec 21 to March 2022. The text should mention that the third wave of the COVID-19 pandemic was ongoing at that time. Where the title refers to 'winter', 'winter 2021/2' might be more accurate.<br><br>3. In common with most multimorbidity studies, the paper included a large number of conditions, with a high potential number of condition combinations. As this number was intractable, the study relied on professional opinion to select conditions that were association with most deaths/admissions (page 6, lines 49-52). The approach might seem problematic as it is not surprising that persons with several of the most common causes of death/admission have very high risks, as subsequently demonstrated.<br><br>4. It seems surprising that respiratory conditions have not been considered as a relevant contributor to combinations in view of the ongoing pandemic.<br><br>5. It may not be clear what is a condition: does a person with diabetes, CKD and CVD have three conditions, or a single condition associated with advanced complications (greater severity of illness)? Severity of illness and patient frailty have not been included in the analysis.<br><br>6. For comparison, ONS has listed the most frequent causes of death as<br>Malignant neoplasm of trachea, bronchus and lung, Ischaemic heart diseases, Influenza and pneumonia, Dementia and Alzheimer disease, Chronic lower respiratory diseases, and Cerebrovascular

	diseases.<br>https://www.ons.gov.uk/peoplepopulationandcommunity/healthandsocialcare/causesofdeath/articles/leadingcausesofdeathuk/2001to2018<br><br>7. The ethics section could mention that the research only had access to anonymised/pseudonymised data.<br><br>8. The approach to data analysis and results presentation is appropriate. the results section is quite brief based on the scale of the study.<br><br>9. In the Discussion (page 9, line 16-) where it says 'we found that the<br>highest risk of winter hospitalisation was observed among individuals with<br>cancer+CKD+CVD+diabetes mellitus and<br>cancer+CKD+CVD+osteoarthritis, while the<br>highest rate of deaths was among those with<br>cancer+CKD+CVD+dementia and<br>CKD+CVD+dementia+OA.' This implies that this is an empirical finding. However, this is not the case, 10 condition combinations were shortlisted based on professional opinion and, of these, the ones mentioned were at greatest risk. If other condition combinations had been shortlisted for analysis, other conclusions might hold.<br><br>10. The abstract should mention that condition combinations were selected for analysis based on professional opinion.

**REVIEWER NAME**	Batty, Jonathan
**REVIEWER AFFILIATION**	University of Leeds, Leeds Institute for Data Analytics
**REVIEWER CONFLICT OF INTEREST**	None
**DATE REVIEW RETURNED**	01-Aug-2024

**GENERAL COMMENTS**	Many thanks for your submission to BMJ Medicine, entitled: "Combinations of multiple long-term conditions and risk of hospitalisation and death during winter: population-based cohort study of 48 million people in England" (manuscript ID: bmjmed-2024-001016), which I read with great interest.<br><br>Briefly, this manuscript described the findings of a retrospective cohort study using linked, UK primary and secondary care data, made available through the PDPPR and HES datasets, respectively. Mortality data were provided by the Office for National Statistics. The study entry date was 1st December 2001: outcomes included hospitalisation events and deaths from 1st December 2021 to 31st March 2022 (which were used to generate IRRs using "overdispersed Poisson regression models"). The exposure of interest was the presence of one or more of 10 multiple long-term conditions (MLTCs) vs. the comparator of no LTCs. Adjustment was made for age, sex, ethnicity and socioeconomic deprivation status. The authors report the association of specific MLTC combinations with hospitalisation and mortality. A particularly high risk was observed in those patients with the combination of cardiovascular disease and dementia. Although the authors initially had strong ambitions to study combinations of 59 LTCs, this was revised as a

result of the inherent combinatorial complexity down to 10 LTCs.

Overall, this is a topical and well-written piece of work that addresses a reasonably novel and important research question. There is a particular need for research in the context of the UK NHS that focuses on the determinants of (and identifies strategies to reduce) winter pressures, especially within the context of the ageing (and increasingly multimorbid) UK population. I believe that it would be of interest to the broad readership of BMJ Medicine. I must also acknowledge that studies of this scale are never straightforward and come with their own analytical and computational challenges: the ambition of this whole-population analysis must be recognised.

This study employs a suitably sophisticated design (with a very large sample size) and generally appropriate statistical methods to reach some robust - if not entirely surprising - conclusions. I believe that the concept of the study (and the present gaps in the literature that it fills) justifies ongoing consideration of publication. However, there are some issues that I believe preclude the publication of this study in its current form, which I have itemised below.

Major issues:
Methods
- Further information must be supplied about the primary data source -- the General Practice Extraction Service (GPES) Data for Pandemic Planning and Research (GDPPR). If necessary, this could be supplied in a supplementary appendix.
- My understanding is that the GDPPR only contains a subset of the observation (diagnosis) codes available in EMIS/SystmOne/Vision, etc. (specifically, those 'code clusters' specified for the COVID 19 planning and research extract). Having looked at the NHS BSA rules that define these clusters, I don't believe that these cover the entire breadth of codes that exist in the 'entire' GP record, and therefore may lead to issues in the ascertainment of LTCs. They also make it impossible to study a LTC that was not included in the extracted subset of codes.
- What proportion of GP practices have opted in to GDPPR? Is there evidence that the patients registered at those GP practices that have opted in to GDPPR are broadly representative of the population of England?
- There were a number of factors that may confound the MLTC-outcome associations reported in this study during the 2021-2022 winter period, including (i) the ongoing COVID-19 pandemic, (ii) significant NHS waiting lists, and (iii) a crisis in social care provision. The inability of the methods to account for all of these (and therefore to generalise beyond 2021-2 in the UK) should be stated clearly in the discussion.
- What proportion of patients in the GDPPR were successfully linked to an individual in the HES/ONS datasets? This should be reported. Failure of linkage could lead to immortal time bias (i.e. where an individual in GDPPR is 'unable' to be hospitalised or die in the dataset as these data were not successfully linked).
- More information is required on how the LTCs included were selected. A reference is made to a paper that seems to be a protocol for a study, but does not provide sufficient justification (reference 20: Dambha-Miller et al, JMIR Res Protoc. 2022). To my knowledge, one of the best studies published to answer this question used a Delphi concensus methodology to identify conditions that should be included in a study such as this (Ho ISS,

	Azcoaga-Lorenzo A, Akbari A, et al. Measuring multimorbidity in research: Delphi consensus study. BMJ Medicine 2022;1:e000247. doi: 10.1136/bmjmed-2022-000247). Justification should be made as to why a different selection of LTCs was chosen.<br>- How were the codelists for each condition developed? Were these mapped from a pre-existing codelist (in e.g. the HDR-UK phenotype library, or CALIBER, etc?). If these are bespoke, what testing has been done to validate these lists (e.g. clinical involvement, local testing using medical records, etc)?<br>- How was ethnicity ascertained? I believe that this is provided both in GDPPR and HES-APC. How were discordant ethnicity records handled?<br>- How was IMD status linked to an individual record? More detail is required: was this linked using their address, postcode (or the address/postcode of their GP surgery)?<br>- The manuscript mentions different numbers of LTCs ascertained: 59, 23, 19 and 10. This is somewhat confusing, especially if only 10 long-term conditions were examined (both marginally, and in combination) during the analysis. For clarity, I believe that the messaging should be made more consistent throughout the paper with the single final number of LTCs examined: 10.<br>- It is stated that "overdispersed Poisson regression" models were used to calculate multivariable adjusted IRRs and 95% CIs for the association between LTCs and hospitalisation and death. What exactly is meant by "overdispersed Poisson regression"? Does this refer to the introduction of a scale term, Poisson inverse gaussian model, the use of a negative binomial model, or something else? What command/package in R was used? How was overdispersion tested for? Presumably this was decided a priori.<br>- Was consideration given to the use of a more contemporary analytic strategy, such as the use of an individual level survival model (e.g. Royston-Parmar flexible parametric survival model)? This would allow modelling of cumulative incidence functions (CIFs) rather than IRRs, which have a more absolute interpretation.<br>- How were competing risks handled in this study (if at all)? If a patient died, they were censored and therefore could no longer be hospitalised. This should ideally be addressed by the analytic methodology (again, this could be done using flexible parametric survival models).<br><br>Results<br>- Does the results section use the presence/absence of 10 conditions in the calculation of the proportion of patients with multimorbidity, or is one of the other definitions (19, 23, 59) used? As per MacRae et al (reference 15 in this study), the larger the number of LTCs studied, the higher the prevalence of MLTCs will be. Therefore, clarity with regard to the number of conditions included in the definition is needed when reporting prevalence estimates.<br>- Table 2 and 3 shows 12 different patterns of MLTCs (grouping all those patients with 1 LTC into a single category, which I believe is appropriate). There are a lot more potential combinations, some of which must have been observed in the data. Is this the top 10 most associated with hospitalisation (or mortality), plus the two 'reference categories' of one and no LTCs? If so, the bottom two rows of the table should be divided off in some way, to show that they have been selectively included despite not being in the 'top 10'.<br>- Table 2 and 3 should specify that the incidence rate is the 'crude' incidence rate, per 1000 person years.<br>- The authors correctly recognise that respiratory infections are the

	major determinant of the increase in hospital admissions observed in winter, and also discuss other determinants (falls, AF, HF, PE, stroke, etc). It would be helpful to know if the cause of hospitalisation (and cause of death) for those with greater burdens of LTCs was different from those with lower burdens of LTC (although this may be a future avenue of research, rather than something to be included in the present paper).<br><br>Discussion:<br>- Why do a greater burden of MLTCs increase hospitalisation (largely due to respiratory infections in winter)? Is it due to an increased vulnerability to viral infections (e.g. dysregulated host-immune responses), an increased susceptibility to become more unwell, or due to other factors (i.e. decreased physiological reserves due to co-existing frailty and disability, leading to unexpected care needs that cannot be met at home)? Potential mechanisms should be considered in greater detail.<br>- The discussion section needs to be revised to include more succinct, actionable outcomes of the present study, with a particular re-focussing on the clinical implications of the findings. Ideally, concrete 'next steps' should be described.<br>- Additional limitations must be included, as per the points raised above.<br>- This study did not evaluate the cause of hospitalisation or vaccination patterns; nebulous mentions of vaccination in the introduction and conclusions should be removed.<br><br>Minor issues:<br>- The proposed short title, "Multiple Long-term Combinations and NHS Winter Pressure" should be revised. Specifically, the grammar of this short title is not ideal, and I believe that the replacement of "NHS Winter Pressure" with "hospitalisation and death during winter" (or similar) would improve this.<br>- The 'Study Design' section of the methods refers to "ICD-19" - I believe this is meant to be "ICD-10".<br>- It is noted that the authors have used a complete case analysis, based on the low observed proportion of missing data (<4% of demographic variables). It should be noted that this would be expected to introduce some bias into the analysis (those with missing data are unlikely to be similar to those with complete data in a number of ways). However, I think that this is acceptable given very high expected computational complexity of multiple imputation methods for a dataset of this scale, and the expectation that MI probably wouldn't drastically alter the final results. This should be briefly noted as a study limitation.<br>- Consider removing 'elderly' as a descriptor of an older population (Results section; "Individuals with MLTC were elderly [...] compared to those..."). This could be substituted with "older than", especially when the "elderly" group have a mean age of only 61.<br>- The GitHub repository linked in the 'Data and code sharing' section returns a 404 error (https://github.com/BHFDSC/CCU059_01). This may be because it is still set to private. This should be visible for review during peer review - I would generally have a look through sections of the code used to generate the key findings so as to also base my feedback on this.

VERSION 1 – AUTHOR RESPONSE

Reviewer(s)' Comments to Author (if any):<br><br>Reviewer: 1<br>Prof. Katie Harron<br>Comments to the Author<br>This study aims to determine which combination of multiple long-term conditions are associated with the greatest risk of winter hospitalisations and deaths.<br><br>The population coverage of this study, including both primary and secondary care data, is a real strength. The findings should be highly generalisable to the English population. Overall the study is well described and I only have a few points of clarification.	**Authors' response:** Thank you for your appreciation of our study.
Abstract results: Please state absolute rates of hospitalisations to give context to the relative effect sizes. The adjusted rate of death is given as 14.6 for CVD+dementia. I think this is the risk ratio rather than the rate so this should be reworded.	**Authors' response:** Thank you for the suggestion. We added the absolute rates of hospitalisation and deaths in the reference group in the abstract:<br><br>"The rates of hospitalisation and deaths amongst individuals with no LTC were 96.3 and 0.8 per 1000 person-years, respectively."<br><br>We also clarified that these were adjusted rate ratios:<br><br>"Compared to those with no LTCs, the adjusted incidence rate ratio of hospitalisation were 11.0 (95% CI: 9.4, 12.7), 9.8 (8.3, 11.4), and 9.6 (8.6, 10.7) for those with cancer+chronic kidney disease (CKD)+cardiovascular disease (CVD)+type-2 diabetes mellitus (T2DM), cancer+CKD+CVD+osteoarthritis (OA), and cancer+CKD+CVD, respectively. Compared to those with no LTC, the adjusted rate ratio of death was 14.6 (12.0, 17.8), 21.4 (17.5, 26.0), 23.2 (17.5, 30.3), and 24.3 (19.1, 30.4) among those with CVD+dementia, CKD+CVD+dementia, cancer+CKD+CVD+dementia, and CKD+CVD+dementia+OA."
It would be helpful to give more clarity on the coverage of the datasets, i.e. who is and is not included in HES (those admitted to hospitals outside of England, or private hospitals etc would be missed).	**Authors' response:** Thank you for the suggestion. We included this in the limitation section: "Our analyses could not capture those admitted to hospitals outside of England, or private hospitals."

How did you assess model fit? Were all the covariates significant?	**Authors' response:** The model fit was assessed by checking the proportionality of the mean and variance as is assumed in a quasipoisson model. Following the methodology described here (https://sscc.wisc.edu/sscc/pubs/glm-r/), we checked the proportionality of the variance of the residuals to the mean (which was met).<br><br>Yes, all the covariates were statistically significant at 0.05. Since the covariates adjusted in the final model were selected a priori to estimate the coefficients of the MLTC combinations over and beyond the covariates in the model, we only reported the coefficients associated with MLTC combinations.
The discussion is rather long and could be made more concise. However, the limitations of how the MLTC combinations should be more thoroughly discussed. These were subjective decisions, and different decisions about how to combine and categorise conditions could have led to different results. There might be different combinations of MLTCs which affect smaller numbers of patients, but which contribute disproportionately to the overall number of hospitalisations and deaths. It would be helpful to explore whether other data-driven approaches could be used to derive the combinations of interest.	**Authors' response:** Thank you. We curtailed the Discussion section substantially. We also mentioned this in the Limitation section: "A different set of LTCs, and different decisions about how to combine and categorise conditions could have led to different results."<br><br>We also agree that some rare diseases excluded from our study could have had a higher rate/risk of these outcome. We, have therefore, included in the Limitation section: "Since we focused on the top ten combinations of LTCs with the highest number of hospitalisation or deaths to inform healthcare policy, rare conditions affecting smaller number of people were excluded although they could have had disproportionately higher risk of these outcomes."<br><br>We initially considered other techniques such as Market-Basket analysis. However, we did not explore those considering the computational resource needed and the complexity in summarising the MLTC combinations. We are currently benchmarking some of the alternative options that could potentially be used in big data context.
Reviewer: 2<br>Dr. Martin GULLIFORD, Kings College London<br>Comments to the Author<br>This is a large UK-wide study that evaluates combinations of long term conditions and risks of hospital admission or all cause mortality. The study finds that pre-selected combinations of conditions are associated with very high absolute and relative risks of these outcomes. The study concludes that these estimates identify groups	**Authors' response:** Thank you for your kind appreciation of our study.

that could be targeted for policy interventions.<br><br>The study addresses a topical subject and while the results have most relevance in the UK, the findings could be of interest to an international readership. As the paper points out, many previous studies have addressed related issues but this study considers specific condition combinations, rather than levels of multiplicity. However, it might be questioned whether the results are surprising, given that conditions were selected for study based on their known associations with hospital admission or death.	
1. The title and text refer to hospitalisation AND death (i.e persons admitted to hospital who died), however, the study seems to include hospitalisation OR death (i.e. including people admitted who did not die, as well as people who died but were not admitted). As a minimum, all the ANDs should be changed to ORs. But there could be a case for treating the two outcomes separately.	**Authors' response:** Thank you. We agree and have revised the manuscript accordingly.
2. The abstract should mention the study period is from Dec 21 to March 2022. The text should mention that the third wave of the COVID-19 pandemic was ongoing at that time. Where the title refers to 'winter', 'winter 2021/2' might be more accurate.	**Authors' response:** Thank you. We agree and have revised the manuscript accordingly.
3. In common with most multimorbidity studies, the paper included a large number of conditions, with a high potential number of condition combinations. As this number was intractable, the study relied on professional opinion to select conditions that were association with most deaths/admissions (page 6, lines 49-52). The approach might seem problematic as it is not surprising that persons with several of the most common causes of death/admission have very high risks, as subsequently demonstrated.	**Authors' response:** Thank you for your insightful comment. We did not select the combinations a priori; we only reported the top ten combinations (not ten individual conditions) that had the highest number (volume) of hospitalisations or deaths to inform healthcare policy.<br><br>Even though we reported the MLTC combinations with top ten absolute numbers, the incidence rates were not monotonic with relation to the absolute numbers. For example, the number of total hospitalisations was almost three times higher among people with CKD+CVD+T2DM (n=39440) than those with AXDP+CKD+CVD+T2DM (n=13605). However, the incidence rate in the former group was 300 per 1000 person-years lower than the latter group.
4. It seems surprising that respiratory conditions have not been considered as a relevant	**Authors' response:** Apologies for any confusion. We considered all the 19 long-term condition groups that included respiratory conditions

contributor to combinations in view of the ongoing pandemic.	asthma and COPD. COPD also appears in the top ten MLTC combinations associated with winter mortality.
5. It may not be clear what is a condition: does a person with diabetes, CKD and CVD have three conditions, or a single condition associated with advanced complications (greater severity of illness)? Severity of illness and patient frailty have not been included in the analysis.	**Authors' response:** We defined each condition (e.g., diabetes) on its own. In the given example, the person had diagnostic codes for each of the three conditions which could be due to advanced complications of one condition. We agree, the severity and patient frailty have not been included in the analysis as these data are not available in the databases used. We mentioned that as a limitation of the study: "...our study did not consider the duration or severity of illness or frailty, nor did it address the sequence of LTC."
6. For comparison, ONS has listed the most frequent causes of death as<br>Malignant neoplasm of trachea, bronchus and lung, Ischaemic heart diseases, Influenza and pneumonia, Dementia and Alzheimer disease, Chronic lower respiratory diseases, and Cerebrovascular diseases.<br>https://www.ons.gov.uk/peoplepopulationandcommunity/healthandsocialcare/causesofdeath/articles/leadingcausesofdeathuk/2001to2018	**Authors' response:** Thank you for referring to the ONS list which focuses on the *underlying cause of death* according to death certificate (which ignores other contributing causes of death). Our analysis focused on all-cause deaths among those with respective MLTC combinations.
7. The ethics section could mention that the research only had access to anonymised/pseudonymised data.	**Authors' response:** Thank you for the suggestion. We included this in the Data and code sharing section:<br><br>"This project used anonymised electronic health records and administrative data which were collected and curated by NHS Digital in a Trusted Research Environment."
8. The approach to data analysis and results presentation is appropriate. the results section is quite brief based on the scale of the study.	**Authors' response:** Thank you for the feedback. We spent a fair amount of time to decide on the best way to present the findings given high complexity of the results. We wanted to provide a concise overview of the results while focusing on the most significant findings.
9. In the Discussion (page 9, line 16-) where it says 'we found that the<br>highest risk of winter hospitalisation was observed among individuals with<br>cancer+CKD+CVD+diabetes mellitus and cancer+CKD+CVD+osteoarthritis, while the highest rate of deaths was among those with cancer+CKD+CVD+dementia and CKD+CVD+dementia+OA.' This implies that this is an empirical finding. However, this is not the	**Authors' response:** Apologies for the confusion. We consulted clinicians (including primary and secondary care practitioners), policy makers, and PPI members for the first two steps— (i) finalising the 59 long-term conditions (LTC), and (ii) shortlisting and reclassifying 23 conditions to 19 groups.

case, 10 condition combinations were shortlisted based on professional opinion and, of these, the ones mentioned were at greatest risk. If other condition combinations had been shortlisted for analysis, other conclusions might hold.	We found that >52,000 unique combinations existed from the 19 LTC groups. Therefore, the team concluded to focus on the top 10 combinations (without specifying what those conditions or combinations would be) that had the highest absolute burden on hospitalisation and death. We reported those ten combinations, which are empirical findings.
10. The abstract should mention that condition combinations were selected for analysis based on professional opinion.	**Authors' response:** As outlined above, the combinations were not selected based on professional opinions, rather based on the absolute volume of the outcomes (without any prior knowledge as to what those combinations would be). Hope it clarifies.
Reviewer: 3<br>Dr. Jonathan Batty, University of Leeds<br>Comments to the Author<br>Many thanks for your submission to BMJ Medicine, entitled: "Combinations of multiple long-term conditions and risk of hospitalisation and death during winter: population-based cohort study of 48 million people in England" (manuscript ID: bmjmed-2024-001016), which I read with great interest.<br><br>Briefly, this manuscript described the findings of a retrospective cohort study using linked, UK primary and secondary care data, made available through the PDPPR and HES datasets, respectively. Mortality data were provided by the Office for National Statistics. The study entry date was 1st December 2001: outcomes included hospitalisation events and deaths from 1st December 2021 to 31st March 2022 (which were used to generate IRRs using "overdispersed Poisson regression models"). The exposure of interest was the presence of one or more of 10 multiple long-term conditions (MLTCs) vs. the comparator of no LTCs. Adjustment was made for age, sex, ethnicity and socioeconomic deprivation status. The authors report the association of specific MLTC combinations with hospitalisation and mortality. A particularly high risk was observed in those patients with the combination of cardiovascular disease and dementia. Although the authors initially had strong ambitions to study combinations of 59 LTCs, this was revised as a result of the inherent combinatorial complexity down to 10 LTCs.<br><br>Overall, this is a topical and well-written piece of	**Authors' response:** Thank you for your insightful appreciation of our work highlighting the importance of the work along with the computational challenges.

work that addresses a reasonably novel and important research question. There is a particular need for research in the context of the UK NHS that focuses on the determinants of (and identifies strategies to reduce) winter pressures, especially within the context of the ageing (and increasingly multimorbid) UK population. I believe that it would be of interest to the broad readership of BMJ Medicine. I must also acknowledge that studies of this scale are never straightforward and come with their own analytical and computational challenges: the ambition of this whole-population analysis must be recognised.<br><br>This study employs a suitably sophisticated design (with a very large sample size) and generally appropriate statistical methods to reach some robust - if not entirely surprising - conclusions. I believe that the concept of the study (and the present gaps in the literature that it fills) justifies ongoing consideration of publication. However, there are some issues that I believe preclude the publication of this study in its current form, which I have itemised below.	
Major issues:<br>Methods<br>- Further information must be supplied about the primary data source -- the General Practice Extraction Service (GPES) Data for Pandemic Planning and Research (GDPPR). If necessary, this could be supplied in a supplementary appendix	**Authors' Response:** Thank you for the suggestion. We have added a new section in the Supplementary Information titled "Supplementary Methods" which lists all the data sources and their relevant information.
My understanding is that the GDPPR only contains a subset of the observation (diagnosis) codes available in EMIS/SystmOne/Vision, etc. (specifically, those 'code clusters' specified for the COVID 19 planning and research extract). Having looked at the NHS BSA rules that define these clusters, I don't believe that these cover the entire breadth of codes that exist in the 'entire' GP record, and therefore may lead to issues in the ascertainment of LTCs. They also make it impossible to study a LTC that was not included in the extracted subset of codes.	**Authors' Response:** Thank you for this comment and we acknowledge this limitation of using the GDPPR dataset alone. GDPPR dataset includes patients' records coded with approximately 34,000 distinct SNOMED-CT concepts, representing over 90% of those currently extracted by GPES.[1]<br><br>Despite such a high coverage at GDPPR, we used both primary and secondary care dataset for the maximum coverage of LTC diagnoses.<br><br>We still noted this as a possible limitation: "Despite using a combination of primary (GDPPR which represents over 90% of the SNOMED codes currently extracted by GPES) and secondary care, underestimation of the MLTC burden cannot be ruled out."

	Reference:<br><br>[1] Wood A, Denholm R, Hollings S, Cooper J, Ip S, Walker V, et al. Linked electronic health records for research on a nationwide cohort of more than 54 million people in England: data resource. BMJ. 2021;373:n826.
- What proportion of GP practices have opted in to GDPPR? Is there evidence that the patients registered at those GP practices that have opted in to GDPPR are broadly representative of the population of England?	**Authors' Response:** GDPPR includes data from 98% of all English general practices across all relevant general practice computer system suppliers (TPP, EMIS, In Practice Systems, and Microtest) and contains records from approximately 96% of the population in England. We have included this information in the supplementary material. However, if the Editors prefer, we will be happy to move this information to the main manuscript.
There were a number of factors that may confound the MLTC-outcome associations reported in this study during the 2021-2022 winter period, including (i) the ongoing COVID-19 pandemic, (ii) significant NHS waiting lists, and (iii) a crisis in social care provision. The inability of the methods to account for all of these (and therefore to generalise beyond 2021-2 in the UK) should be stated clearly in the discussion.	**Authors' Response:** We agree. We added the following in the limitations section: "This is an observational study that overlapped with the covid -19 pandemic during which there was a substantial disruption in health and social care provisions (e.g., backlog, waitlists). Therefore, the findings should be interpreted with caution without direct causal association."
- What proportion of patients in the GDPPR were successfully linked to an individual in the HES/ONS datasets? This should be reported. Failure of linkage could lead to immortal time bias (i.e. where an individual in GDPPR is 'unable' to be hospitalised or die in the dataset as these data were not successfully linked).	**Authors' Response:** HES contains all hospital admissions in England and therefore if a patient is registered in GDPPR and hospitalised ever, their data would appear in HES APC. Linkage between GDPPR and HES APC is carried out by NHS Digital Master Person Service using NHS numbers and associated personal details (e.g., age, sex, and postcode) that are included in primary and secondary records. Since, almost 97-100% of records submitted to and processed by NHS Digital include the NHS number and other key personal details, the accuracy of the linking process can be deemed as highly accurate.<br><br>Reference:

	[1] Wood A, Denholm R, Hollings S, Cooper J, Ip S, Walker V, et al. Linked electronic health records for research on a nationwide cohort of more than 54 million people in England: data resource. BMJ. 2021;373:n826.
- More information is required on how the LTCs included were selected. A reference is made to a paper that seems to be a protocol for a study, but does not provide sufficient justification (reference 20: Dambha-Miller et al, JMIR Res Protoc. 2022). To my knowledge, one of the best studies published to answer this question used a Delphi concensus methodology to identify conditions that should be included in a study such as this (Ho ISS, Azcoaga-Lorenzo A, Akbari A, et al. Measuring multimorbidity in research: Delphi consensus study. BMJ Medicine 2022;1:e000247. doi: 10.1136/bmjmed-2022-000247). Justification should be made as to why a different selection of LTCs was chosen.	**Authors' Response:** Thank you for highlighting this. Our grants proposal, clinical consultations, PPI engagement, statistical analysis plan were finalised before the Ho et al paper was published. At the time, the Dambha-Miller study was the one we had as a reference (please see the updated citation submitted to NIHR Open Research).[1] This list of conditions was agreed through national consensus work across research in England working on MLTC. A full reference is listed below. Nevertheless, our study included most of the conditions listed in Ho et al reference provided by the reviewer.<br><br>[1] Dambha-Miller H, Farmer A, Nirantharakumar K *et al.* Artificial Intelligence for Multiple Long-term conditions (AIM): A consensus statement from the NIHR AIM consortia. *NIHR Open Res* 2023, **3**:21 doi: 10.3310/nihropenres.1115210.1
- How were the codelists for each condition developed? Were these mapped from a pre-existing codelist (in e.g. the HDR-UK phenotype library, or CALIBER, etc?). If these are bespoke, what testing has been done to validate these lists (e.g. clinical involvement, local testing using medical records, etc)?	**Authors' Response:** Thank you. We used the codelists from BHF (BHF Data Science Centre (github.com) primary source), OpenSAFELY OpenCodelists, Health Data Research UK Phenotype library, and University of Cambridge Developing Multimorbidity Codelists. The final codelists were reviewed and verified by the clinicians through an iterative process (HDM, NI, NK). For transparency and reproducibility, we uploaded the clinical codes used to define the conditions in the Supplement and on the GitHub link.
- How was ethnicity ascertained? I believe that this is provided both in GDPPR and HES-APC. How were discordant ethnicity records handled?	**Authors' Response:** Ethnicity was determined using the most recent available non-missing value from either primary care (GPDPR) or secondary care (HES-APC) records. Primary care record was prioritised in cases where both sources had matching most recent dates but discordant records.
How was IMD status linked to an individual record? More detail is required: was this linked using their address, postcode (or the	**Authors' Response:** We clarified in the Methods section that IMD was based on individual's residential address mapped with LSOAs: "Individual's residential address was mapped with Lower-layer Super Output Areas (LSOAs), which

address/postcode of their GP surgery)?	was then linked with 2019 English IMD. The IMD ranking was further categorised into quintiles whereby quintiles 1 and 5 represent the most and least deprived areas, respectively.”
- The manuscript mentions different numbers of LTCs ascertained: 59, 23, 19 and 10. This is somewhat confusing, especially if only 10 long-term conditions were examined (both marginally, and in combination) during the analysis. For clarity, I believe that the messaging should be made more consistent throughout the paper with the single final number of LTCs examined: 10.	**Authors’ response:** Apologies for the confusion. We used all 59 conditions for the descriptive statistics (Table 1). For the association between MLTC combinations and outcomes (Tables 2-3, Figures 2-3), we focused on 19 MLTC groups.<br><br>One point to particularly clarify is that we did not focus on ten MLTCs, rather top ten combinations of MLTC that had the highest absolute number of outcome events. We clarified this in the revised version.
- It is stated that "overdispersed Poisson regression" models were used to calculate multivariable adjusted IRRs and 95% CIs for the association between LTCs and hospitalisation and death. What exactly is meant by "overdispersed Poisson regression"? Does this refer to the introduction of a scale term, Poisson inverse gaussian model, the use of a negative binomial model, or something else? What command/package in R was used? How was overdispersion tested for? Presumably this was decided a priori.	**Authors’ response:** Yes, the model was decided a-priori. We looked at the dispersion parameter (which was more than 10 in all regression models). We clarified in the analysis section that we used GLM model with family= “quasipoisson” in R. We also tested the proportionality of the mean and variance (discussed above).
- Was consideration given to the use of a more contemporary analytic strategy, such as the use of an individual level survival model (e.g. Royston-Parmar flexible parametric survival model)? This would allow modelling of cumulative incidence functions (CIFs) rather than IRRs, which have a more absolute interpretation	**Authors’ response:** We acknowledge the possibility of alternative models. However, we opted for Poisson models for several reasons. First, our primary objective was to estimate the rate of hospitalisation or deaths (as opposed to time-to-event) to inform healthcare policy on the burden of hospitalisation or deaths (not the risk of individual time-to-death or survival). The follow-up time was short and largely uniform (median [IQR]: 120 [120, 120] days), hence the differential follow-up time would not make a substantial difference. Moreover, running individual-level regression models on more than 48 million participants with additional covariates is computationally extremely expensive. Therefore, we aggregated the follow-up time and the number of events by the strata of the covariates in the model to reduce the computational time and resources without loss of information. [1]

	[1] Errington A, Einbeck J, Cumming J, Rössler U, Endesfelder David. The effect of data aggregation on dispersion estimates in count data models. International J Biostat. 2022;18(1):183- 202. Doi:10.1515/ijb-2020-0079
- How were competing risks handled in this study (if at all)? If a patient died, they were censored and therefore could no longer be hospitalised. This should ideally be addressed by the analytic methodology (again, this could be done using flexible parametric survival models).	**Authors' response:** We appreciate the point raised. Our objective was to inform health services policy regarding the burden of hospitalisation or deaths rather than examining the aetiology of conditions. Therefore, we did not consider competing risk.
Results<br>- Does the results section use the presence/absence of 10 conditions in the calculation of the proportion of patients with multimorbidity, or is one of the other definitions (19, 23, 59) used? As per MacRae et al (reference 15 in this study), the larger the number of LTCs studied, the higher the prevalence of MLTCs will be. Therefore, clarity with regard to the number of conditions included in the definition is needed when reporting prevalence estimates.	**Authors' response:** Apologies for the confusion. We have clarified this in the revised version by indicating the number of MLTCs considered under each of the tables and figures.
- Table 2 and 3 shows 12 different patterns of MLTCs (grouping all those patients with 1 LTC into a single category, which I believe is appropriate). There are a lot more potential combinations, some of which must have been observed in the data. Is this the top 10 most associated with hospitalisation (or mortality), plus the two 'reference categories' of one and no LTCs? If so, the bottom two rows of the table should be divided off in some way, to show that they have been selectively included despite not being in the 'top 10'.	**Authors' response:** Thank you for the suggestion. We separated the 'none' and 'one' to indicate these were selected for comparison, as you suggested.
- Table 2 and 3 should specify that the incidence rate is the 'crude' incidence rate, per 1000 person years.	**Authors' response:** Thank you, we have updated the table titles in the manuscript as suggested.
- The authors correctly recognise that respiratory infections are the major determinant of the increase in hospital admissions observed in winter, and also discuss other determinants (falls, AF, HF, PE, stroke, etc). It would be helpful to know if the cause of hospitalisation (and cause of death) for those with greater burdens of LTCs was different from those with lower burdens of LTC	**Authors' response:** We agree that these would be important outcomes. However, we also agree that this is beyond the scope of the current analyses. We mentioned this in the limitation section: " Our analysis did not consider the cause of hospitalisation or deaths which could be explored in future research."

(although this may be a future avenue of research, rather than something to be included in the present paper).	
Discussion:<br>- Why do a greater burden of MLTCs increase hospitalisation (largely due to respiratory infections in winter)? Is it due to an increased vulnerability to viral infections (e.g. dysregulated host-immune responses), an increased susceptibility to become more unwell, or due to other factors (i.e. decreased physiological reserves due to co-existing frailty and disability, leading to unexpected care needs that cannot be met at home)? Potential mechanisms should be considered in greater detail.	**Authors' response:** Thank you for your insightful question regarding the link between MLTCs and increased hospitalisation. We included in the Discussion section: "Potential mechanisms include, but not limited to, a higher burden of respiratory infections in winter, an increased vulnerability to viral infections due to dysregulated immune responses, co-existing frailty and disability, unmet social care needs etc. Additionally, patients with MLTCs may have a compromised ability to cope with infection-related stressors, such as fever or hypoxia."
- The discussion section needs to be revised to include more succinct, actionable outcomes of the present study, with a particular re-focussing on the clinical implications of the findings. Ideally, concrete 'next steps' should be described.	**Authors' response:** Thank you. We clearly identified the limitations, and suggested some next steps with regards to clinical implications:<br><br>"Future research should focus on longitudinal studies to elucidate the temporal patterns and long-term impact of MLTCs on health outcomes. Furthermore, efforts should be directed towards developing integrated care models that address the complex needs of individuals with MLTC during winter, particularly those with high-risk combinations of chronic conditions.<br><br>Future research should investigate whether we can integrate an improved stratification incorporating more granular data, such as stages of disease severity, or other indicators of patient frailty and functionality, history, duration, and effectiveness of the medication(s) used to treat the underlying condition(s), any social support they receive along with the clinical care etc. This could pave the way for more nuanced, patient-centred new models of care in the future."
- Additional limitations must be included, as per the points raised above.	**Authors' response:** Thank you, we have included all the suggested points in the limitation section.
- This study did not evaluate the cause of hospitalisation or vaccination patterns; nebulous mentions of vaccination in the introduction and conclusions should be removed.	**Authors' response:** Revised as suggested. Thank you.

Minor issues:<br>- The proposed short title, "Multiple Long-term Combinations and NHS Winter Pressure" should be revised. Specifically, the grammar of this short title is not ideal, and I believe that the replacement of "NHS Winter Pressure" with "hospitalisation and death during winter" (or similar) would improve this.	**Authors' response:** We revised as suggested: "Multiple long-term conditions and hospitalisation and death during winter"
- The 'Study Design' section of the methods refers to "ICD-19" - I believe this is meant to be "ICD-10".	**Authors' response:** Apologies; this has been corrected in the revised manuscript.
- It is noted that the authors have used a complete case analysis, based on the low observed proportion of missing data (<4% of demographic variables). It should be noted that this would be expected to introduce some bias into the analysis (those with missing data are unlikely to be similar to those with complete data in a number of ways). However, I think that this is acceptable given very high expected computational complexity of multiple imputation methods for a dataset of this scale, and the expectation that MI probably wouldn't drastically alter the final results. This should be briefly noted as a study limitation.	**Authors' response:** Thank you. We added: "Missing data in our study was small but could still introduce some biases. However, we believe it would not have any major impact on the study findings."
- Consider removing 'elderly' as a descriptor of an older population (Results section; "Individuals with MLTC were elderly [...] compared to those..."). This could be substituted with "older than", especially when the "elderly" group have a mean age of only 61.	**Authors' response:** Thank you. Revised as suggested.
- The GitHub repository linked in the 'Data and code sharing' section returns a 404 error (https://github.com/BHFDSC/CCU059_01). This may be because it is still set to private. This should be visible for review during peer review - I would generally have a look through sections of the code used to generate the key findings so as to also base my feedback on this	**Authors' response:** Apologies. The GitHub repository is visible now.
Reviewer: 4<br>Dr. Chris McParland, University of Glasgow<br>Comments to the Author<br>Thank you for the opportunity to review this manuscript. It describes a well-designed analysis of multimorbidity clusters using routinely collected data, for a very large cohort in England. It addresses how these conditions are associated	**Authors' response:** Thank you for your appreciation of our study.

with healthcare usage during winter. The study is original and addresses an extremely important area for clinical and academic colleagues. I am sure it will be of great interest to BMJ Medicine's readers.	
I only have one significant comment about the reporting of methods.<br>P8 ~line 36, "Patient and public contributors helped us with developing the study" doesn't provide enough detail. I don't think the section needs to be very long, but at a minimum it would be useful to understand (a) what type of people contributed to this study, e.g. people with multimorbidity, general public, and (b) what the impact of this involvement was, e.g. changes to methods, approach to dissemination, securing funding. One or two lines would suffice.<br><br>Other than this, I have no issues and would like to congratulate the authors on a very interesting study.	**Authors' response:** Thank you for your comment. We have added the following statement in the Acknowledgement to explain further:<br><br>"Patient and public contributors played a crucial role in shaping our study through two rounds of meetings. We engaged ten members of the general public, all aged 18 or older with multiple long-term conditions, in discussions with the research team. The meetings focused on key issues such as the risks associated with living in cold homes during winter, access to adequate healthcare for existing conditions, and the financial impact of a potential increased need for health services. Their valuable insights provided a clearer perspective on these challenges and helped us refine the study's methodology to better address the real-world concerns of those affected."
Editor(s)' Comments to Author (if any):<br>Thank you for submitting this interesting and timely study. We hope you find the reviewer comments and our editorial comments below useful - and we look forward to reading a revised manuscript.	**Authors' response:** Thank you for your feedback. We highly appreciate the insightful and constructive feedback and suggestions from you and the reviewers. These have substantially improved the quality of the revised manuscript.
Editor comments (Tom Nolan, clinical editor):<br><br>1. Rationale for combinations of LTC selected for analysis. Although the rationale for selecting the 10 combinations is given ("after extensive consultation with the practicing clinicians, to balance the computational burden with clinical utility, and considering the primary aim of this project we shortlisted ten combinations of MLTC associated with the highest number of hospital admissions or deaths (separately) during the winter season") when looking more closely at the combinations, I find the many of the choices surprising, which leaves me asking more questions about how they were selected. For instance:	**Authors' response:** Apologies for the confusion. We used all 59 conditions for the descriptive statistics (Table 1). For the association between MLTC combinations and outcomes (Tables 2-3, Figures 2-3), we focused on 19 MLTC after consultation with the practising GPs and members of the patient and public.<br><br>One point to particularly clarify is that we did not focus on ten individual LTC, rather top ten combinations of MLTC that had the highest absolute volume of hospitalisations or deaths during the study period (without knowing or specifying what those conditions would be). We described this in the revised version. Hope it clarifies the issue.

-CKD or CVD are included in all combinations<br>-asthma is missing altogether;<br>-COPD only once<br>-COPD for death but not hospitalizations - why have separate lists for admissions and deaths?<br>-dementia is included in 5 combinations for death but none for hospitalisation? (without an explanation readers are left to draw their own conclusions eg is a bias against people with dementia receiving hospital care when they are acutely unwell)	We did not select a priori two sets of diseases for hospitalisation and deaths. We selected the top ten combinations that had the highest absolute volume of these outcomes without knowing what the individual conditions would be.
Although the authors argue that restricting to 10 combinations reduces the risk of type 1 error and makes the research feasible, the above omissions of common, major causes of hospitalisation/death (eg COPD) would suggest there are too few combinations and the methods used to come up with them may not be robust (in contrast to a delphi process, for instance, as suggested by a reviewer). The risk of misinterpretation seems high, as readers could easily conclude that these combinations of comorbidity are the highest risk factors for hospitalisation/death clinically, when really they're the highest risk factors of the 10 combinations studied.	Hospitalisation was chosen as the primary outcome to inform the healthcare policy on the case-mix of patients with possible combinations of MLTC during the Wintertime to aid their planning with the healthcare personnel and other resources. Mortality was the secondary outcome to help inform healthcare organisations stratify the patients that are at the highest risk of mortality. We clarified this in the Discussion section: "... our objective was to estimate the healthcare burden of hospitalisation and mortality rather than individual risk of these outcomes. Therefore, the findings should not be interpreted as the guide for individual risk predictions."
A more detailed description of the rationale and process that was followed to come up with these combinations seems necessary (eg if the experts were considering groups that may be easier to target for vaccination or proactive care, for instance). Note that the methods say that details of the patient, clinician and policy experts are included in the acknowledgements, but only four policy colleagues are mentioned in this box (and no patients or clinicians, it appears). I would also suggest stronger caveats in the abstract and discussion about the limitations of this approach. Finally, the authors may also wish to explain further 1) why different lists were prepared for hospitalisation and death, 2) why some conditions that readers will think may have a lower risk of admissions feature heavily (eg osteoarthritis) and other conditions that one might expect to have a higher risk of hospitalisation are not (eg asthma, COPD), and 3) why existing data on deaths/hospitalisations - eg from ONS, as a reviewer suggests, or wider literature - were not used, at least in combination with expert opinion.	We elaborated the process of selecting the MLTC combinations in the revised version, clarified that it was the absolute volume of hospitalisation or deaths that was the deciding factor for this report. We also added an illustrative example how increasing number of conditions may mask important disease combinations.<br><br>For example, in the following table, 1000 event would be reported if we considered MLTC combination A+B+C. However, if we keep considering rarer disease (e.g., D, E, F etc), each combination will have lower numbers than A+B+C, leading to an erroneous conclusion that A+B+C is a less important disease combination. Therefore, there needs to be a compromise between the number of long-term conditions we consider in the combination, computational complexity, clinical importance, and practical applications.<br><br>In theory, the long-term conditions are not restricted to 59 or 35 (Ho et al. BMJ Med); there are hundreds of conditions which we often group them to 3- or 4-digit ICD codes, for example. A 3-

	digit ICD code also hides many important clinical differences, but we use it from a pragmatic point of view.<br><br>We discussed above why the ONS list of underlying cause of death was not particularly relevant for our research question. We will be happy to provide more information/clarification if needed.<br><br><table><tr><th>MLTC combinations</th><th>Number</th></tr><tr><td>A+B+C</td><td>1000</td></tr><tr><td>A+B+C+D</td><td>250</td></tr><tr><td>A+B+C+E</td><td>190</td></tr><tr><td>A+B+C+D+E</td><td>60</td></tr></table>*For illustration only; it is possible to have other combinations with A+B+C, such as F, G, H etc.
2. "Winter 2021/22" would be more accurate than just "winter" in the title, as it was a winter during the covid19 pandemic, so findings may not reflect a 'typical' winter.	**Authors' Response:** Revised as suggested. Thank you.
3. The inability to grade conditions is acknowledged as a limitation, but from my clinical perspective is likely to have a profound effect on the risk of admission and death (asymptomatic osteoporosis versus severe disability from multiple wedge fractures), and would surely affect the incident rates well beyond the 95% CIs quoted. We saw a lot of harm during the pandemic from attributing risk to individuals based on a diagnostic code (without considering severity). Could this limitation be an opportunity to call for improved risk stratification within disease categories?	**Authors' Response:** We appreciate your clinical perspective and insightful feedback. We agree, in principle, that more granular data on the severity would be ideal along with other parameters such as the duration of illnesses, history, duration, and effectiveness of the medication(s) used to treat the underlying condition(s), any social support they receive along with the clinical care etc. However, these data are not always readily available in the clinical care settings, and it would make the risk stratification more challenging given the time and resources. We added this as an avenue for future research: "Future research should investigate whether we can integrate an improved stratification incorporating more granular data, such as stages of disease severity, or other indicators of patient frailty and functionality, history, duration, and effectiveness of the medication(s) used to treat the underlying condition(s), any social support they receive along

	with the clinical care etc. This could pave the way for more nuanced, patient-centred risk models in the future.”

VERSION 2 – REVIEW

**REVIEWER NAME**	Harron, Katie
**REVIEWER AFFILIATION**	
**REVIEWER CONFLICT OF INTEREST**	None
**DATE REVIEW RETURNED**	24-Sep-2024

**GENERAL COMMENTS**	The authors have addressed all of my previous comments and I have no further queries.

**REVIEWER NAME**	Batty, Jonathan
**REVIEWER AFFILIATION**	University of Leeds, Leeds Institute for Data Analytics
**REVIEWER CONFLICT OF INTEREST**	None
**DATE REVIEW RETURNED**	25-Sep-2024

**GENERAL COMMENTS**	Many thanks for the revisions to your manuscript submitted to BMJ Medicine, re-titled: "Combinations of multiple long-term conditions and risk of hospitalisation or death during winter 2021-22: population -based cohort study of 48 million people in England" (manuscript ID: bmjmed-2024-001016.R1). I have re-read your manuscript and the responses made to the first round of reviewer comments. I believe these have generally been addressed to a high standard.<br><br>With regard to my comments, I note that there is now a supplementary appendix which contains comprehensive details of the data sources and codelists used to define the long-term conditions studied. This satisfactorily addresses many of my comments (those regarding the lack of detail given about these in the first draft) -- many thanks. I also note that the GitHub for this project (https://github.com/BHFDSC/CCU059_01) is now live. This contains a wealth of useful information to support this publication. The authors should be commended for sharing these additional code and data and their promotion of an open and reproducible scientific approach.<br><br>I only have one very minor final point, which (from my perspective) could probably be addressed during the proofing process. On Page 25, Line 4. "Increasing number of [MLTC] is" should read "Increasing numbers of [MLTC] are".<br><br>Overall, I believe that this is an important, timely and well-conducted study that is reported very well, and believe that it should now be accepted for publication.
